# Frequent Loss and Alteration of the *MOXD2* Gene in Catarrhines and Whales: A Possible Connection with the Evolution of Olfaction

**DOI:** 10.1371/journal.pone.0104085

**Published:** 2014-08-07

**Authors:** Dong Seon Kim, Yao Wang, Hye Ji Oh, Kangseok Lee, Yoonsoo Hahn

**Affiliations:** Department of Life Science, Research Center for Biomolecules and Biosystems, Chung-Ang University, Seoul, Korea; Chang Gung University, Taiwan

## Abstract

The *MOXD2* gene encodes a membrane-bound monooxygenase similar to dopamine-β-hydroxylase, and has been proposed to be associated with olfaction. In this study, we analyzed *MOXD2* genes from 64 mammalian species, and identified loss-of-function mutations in apes (humans, Sumatran and Bornean orangutans, and five gibbon species from the four major gibbon genera), toothed whales (killer whales, bottlenose dolphins, finless porpoises, baijis, and sperm whales), and baleen whales (minke whales and fin whales). We also identified a shared 13-nt deletion in the last exon of Old World cercopithecine monkeys that results in conversion of a membrane-bound protein to a soluble form. We hypothesize that the frequent inactivation and alteration of *MOXD2* genes in catarrhines and whales may be associated with the evolution of olfaction in these clades.

## Introduction

Olfaction, the sense of smell, is an essential perception that animals use to explore their environment. Olfaction plays an important role in detecting predators, locating food, and identifying mating partners. Specifically, airborne odorant molecules are recognized and discriminated by olfactory receptors (ORs) in the olfactory epithelium [1]. Catarrhines, Old World primates of the infraorder Catarrhini, have a smaller olfactory bulb and olfactory epithelium compared with other mammals, which is likely due to the increased dependence on vision rather than olfaction in these species [2], [3]. It was previously reported that OR genes exhibit relaxed selective constraints in apes (humans, chimpanzees, and orangutans), implying that the dependency on olfaction is reduced in apes [4]. However, there are no significant differences in the number of functional OR genes among New World monkeys, Old World monkeys, and apes, indicating that there is no direct link between the acquisition of full trichromatic vision and the degeneration of OR genes [5], [6].

The olfactory apparatus is completely absent in toothed whales, which also have a high number of degenerated OR genes [5], [7]–[10]. In contrast to toothed whales, baleen whales still maintain an olfactory apparatus, and have a high number of intact OR genes [7], [8], [11]. For example, the bowhead whale (*Balaena mysticetus*), a baleen whale species, has a histologically complex olfactory bulb, which comprises approximately 0.13% of the total brain weight [12]. These whales also possess a high proportion (51%) of intact olfactory receptor genes, and it proposed that bowhead whales can use olfaction to locate krill, which comprise a major part of the diet of certain bowhead whales [12]. Examination of the anatomical structure of the olfactory apparatus of minke whales (*Balaenoptera acutorostrata*) also suggested that these whales might maintain the olfactory system, and use it to detect airborne odorants from clouds of plankton on which they feed [13]. Nevertheless, the absence or reduction of an olfactory apparatus in some whales, especially in toothed whales, is likely due to their fully aquatic lifestyle and reliance on sophisticated vocal communication and/or echolocation.

The monooxygenase, DBH-like 2 (*MOXD2*) gene was proposed to be associated with olfaction because mouse *Moxd2* is strongly expressed in the medial olfactory epithelium [14], [15]. The *MOXD2* gene comprises 13 coding exons, and encodes a protein with an endoplasmic reticulum (ER) signal peptide at its N-terminus, and a glycosylphosphatidylinositol (GPI) anchor signal at its C-terminus. The MOXD2 protein thus enters the secretory pathway and eventually becomes bound to the cell membrane by a GPI-anchor. The membrane-bound MOXD2 protein contains “DOMON” (Pfam accession number PF03351), “Cu2_monooxygen” (PF01082), and “Cu2_monoox_C” (PF03712) domains. Two other genes, the dopamine-β-hydroxylase (*DBH*) gene and the monooxygenase, DBH-like 1 (*MOXD1*) gene, encode proteins with the same domain organization [16], [17]. These three genes were generated by duplication during bilaterian evolution [14]. Specifically, the *DBH* gene encodes soluble and membrane-bound monooxygenases present in the synaptic vesicles of postganglionic sympathetic neurons [18]. Through this, DBH converts dopamine to norepinephrine, both of which are neurotransmitters that transmit signals between nerve cells. It is probable that the function of MOXD2 is similar to that of DBH with respect to neurotransmitter metabolism. Moreover, the mouse *Moxd2* gene is strongly expressed in the medial olfactory epithelium [15], raising the possibility of its involvement in olfactory function. To date, however, there is no direct evidence of this functionality.

We previously reported that the *MOXD2* gene is mutated in humans (*Homo sapiens*), Sumatran orangutans (*Pongo abelii*), Bornean orangutans (*Pongo pygmaeus*), and rhesus macaques (*Macaca mulatta*) [14]. The human *MOXD2* is inactivated due to the deletion of a genomic region containing exons 12 and 13, which occurred after human-chimpanzee divergence. The orangutan *MOXD2* has two nonsense mutations and a splice site mutation, rendering it non-functional. Similarly, the *MOXD2* of rhesus macaques has a frameshift mutation due to a 13-nt deletion in the last exon, which results in a C-terminal truncation. Finally, the western lowland gorilla (*Gorilla gorilla gorilla*) *MOXD2* is intact but has an elevated dN/dS ratio, implying relaxed selection.

In this study, we examined *MOXD2* genes from 64 mammalian species and found additional gene inactivation in gibbons and whales: the *MOXD2* gene is absent in five gibbon species representing all four extant genera (*Nomascus*, *Hoolock*, *Hylobates*, and *Symphalangus*) due to a complete gene deletion; the *MOXD2* genes of six whale species, including four toothed and two baleen whale species, have many open reading frame (ORF)-disrupting mutations; and the sperm whale, a toothed whale species, completely lost the *MOXD2* gene due to a genomic deletion. We hypothesize that the loss of functional *MOXD2* genes in apes and whales may be associated with the evolution of olfaction in these clades.

## Materials and Methods

### Identification of *MOXD2* gene sequences

Mammalian *MOXD2* gene sequences were identified by BLASTN searches (http://blast.ncbi.nlm.nih.gov/Blast.cgi) of the whole genome shotgun (WGS) contigs database in the National Center for Biotechnology Information (NCBI) (http://www.ncbi.nlm.nih.gov), and by BLAT searches (http://genome.ucsc.edu/cgi-bin/hgBlat) of genome assemblies using the University of California Santa Cruz (UCSC) Genome Browser web server (http://genome.ucsc.edu). The accession numbers of sequence data and genome assemblies used in this analysis are listed in Table 1. Genomic sequences were downloaded and aligned with the chimpanzee (*Pan troglodytes*) *MOXD2* cDNA or that of closely related species using SIM4 to predict exon sequences [19]. Successfully matched exon sequences were extracted and concatenated to generate virtual cDNA sequences, which were translated to obtain protein sequences. cDNA and protein sequences as well as exon coordinates are provided in Data S1.

**Table 1:** Summary of species presented in this study.

No	Scientific name	Common name	NCBI accession or UCSC genome assembly	Length (aa)	SP^a^	GPI^b^	Mutations	Reference
1	*Homo sapiens*	Human	NR_024346, hg19	NA^c^	NA	NA	exon 6: nonsense codon, polymorphic; exons 12 and 13: exon deletion	[14], [40]
2	*Pan troglodytes*	Chimpanzee	panTro4	618	Yes	Yes		[41]
3	*Pan paniscus*	Bonobo	AJFE01039607	618	Yes	Yes		[42]
4	*Gorilla gorilla gorilla*	Western lowland gorilla	gorGor3	618	Yes	Yes		[43]
5	*Pongo abelii*	Sumatran orangutan	ponAbe2, SRP001577	NA	NA	NA	exon 3: splice donor deletion, polymorphic; exon 8: nonsense codon; exon 13: nonsense codon, polymorphic	[44]
6	*Pongo pygmaeus*	Bornean orangutan	SRP001577	NA	NA	NA	exon 3: splice donor deletion, polymorphic; exon 8: nonsense codon; exon 13: nonsense codon, polymorphic	[44]
7	*Nomascus leucogenys*	Northern white-cheeked gibbon	nomLeu3	NA	NA	NA	gene deletion	unpublished^d^
8	*Hoolock leuconedys*	Eastern hoolock gibbon	SRP010913	NA	NA	NA	gene deletion	unpublished^e^
9	*Hylobates moloch*	Silvery gibbon	SRP010914	NA	NA	NA	gene deletion	unpublished^e^
10	*Hylobates pileatus*	Pileated gibbon	SRP010915	NA	NA	NA	gene deletion	unpublished^e^
11	*Symphalangus syndactylus*	Siamang	SRP010917	NA	NA	NA	gene deletion	unpublished^e^
12	*Macaca mulatta*	Rhesus macaque	rheMac3	587	Yes	No	exon 13: 13-nt deletion, C-terminal truncation	[45], [46]
13	*Macaca fascicularis*	Crab-eating macaque	AQIA01048523	587	Yes	No	exon 13: 13-nt deletion, C-terminal truncation	unpublished^f^
14	*Papio anubis*	Olive baboon	AHZZ01107346	587	Yes	No	exon 13: 13-nt deletion, C-terminal truncation	unpublished^g^
15	*Chlorocebus sabaeus*	Green monkey	AQIB01162307	587	Yes	No	exon 13: 13-nt deletion, C-terminal truncation	unpublished^h^
16	*Saimiri boliviensis*	Black-capped squirrel monkey	saiBol1	618	Yes	Yes		unpublished^i^
17	*Callithrix jacchus*	Common marmoset	calJac3	618	Yes	Yes		unpublished^j^
18	*Tarsius syrichta*	Philippine tarsier	ABRT02374379	618	Yes	Yes		[47]
19	*Microcebus murinus*	Gray mouse lemur	ABDC01141541, SRP021223	618	Yes	Yes		[47], [48]
20	*Daubentonia madagascariensis*	Aye-aye	AGTM000000000, SRP018575	618	Yes	Yes		[49], [50]
21	*Otolemur garnettii*	Northern greater galago (bushbaby)	otoGar3	618	Yes	Yes		[47]
22	*Tupaia chinensis*	Chinese treeshrew	ALAR01033741	618	Yes	Yes		[51]
23	*Oryctolagus cuniculus*	European rabbit	oryCun2	618	Yes	Yes		unpublished^k^
24	*Mus musculus*	House mouse	NM_139296, mm10	619	Yes	Yes		[52]
25	*Rattus norvegicus*	Brown Norway rat	NM_001109229, rn4	619	Yes	Yes		[53]
26	*Cricetulus griseus*	Chinese hamster	NW_003614308	619	Yes	Yes		[54]
27	*Dipodomys ordii*	Ord's kangaroo rat	dipOrd1	615	Yes	Yes		[47]
28	*Heterocephalus glaber*	Naked mole rat	NW_004624765, NW_004636903	619	Yes	Yes		unpublished^l^
29	*Ictidomys tridecemlineatus*	Thirteen-lined ground squirrel	AGTP01119753	618	Yes	Yes		[47]
30	*Pteropus alecto*	Black flying fox	KB030581	618	Yes	Yes		[55]
31	*Eptesicus fuscus*	Big brown bat	ALEH01088560	618	Yes	Yes		unpublished^m^
32	*Myotis lucifugus*	Little brown bat	myoLuc2	618	Yes	Yes		unpublished^n^
33	*Myotis brandtii*	Brandt's bat	ANKR01258667	618	Yes	Yes		[56]
34	*Myotis davidii*	David's bat	ALWT01228485	618	Yes	Yes		[55]
35	*Felis catus*	Cat	felCat5	618	Yes	Yes		[57]
36	*Canis lupus familiaris*	Dog	canFam3	618	Yes	Yes		[58]
37	*Ailuropoda melanoleuca*	Giant panda	AilMel1	618	Yes	Yes		[59]
38	*Leptonychotes weddellii*	Weddell seal	APMU01110865	618	Yes	Yes		unpublished^o^
39	*Odobenus rosmarus divergens*	Pacific walrus	NW_004451520	618	Yes	Yes		unpublished^p^
40	*Mustela putorius furo*	Ferret	AEYP01008749	618	Yes	Yes		unpublished^q^
41	*Equus caballus*	Horse	equCab2	618	Yes	Yes		[60]
42	*Ceratotherium simum simum*	Southern white rhinoceros	AKZM01025525	618	Yes	Yes		unpublished^r^
43	*Camelus ferus*	Wild Bactrian camel	AGVR01031487	618	Yes	Yes		[61]
44	*Sus scrofa*	Pig	susScr3	618	Yes	Yes		[62]
45	*Capra hircus*	Goat	AJPT01248665	617	Yes	Yes		[63]
46	*Ovis aries*	Sheep	AMGL01089435	617	Yes	Yes		[64]
47	*Pantholops hodgsonii*	Chiru (Tibetan antelope)	AGTT01184576	617	Yes	Yes		[65]
48	*Bos taurus*	Cow	bosTau7	618	Yes	Yes		[66]
49	*Orcinus orca*	Killer whale	ANOL02032434	NA	NA	NA	exon 2: 4-nt deletion; exon 3: 1-nt deletion; splice donor mutation (GT to GA); exon 5: splice acceptor mutation (AG to TG); 1-nt insertion; exon 9: two nonsense codons; exon 11: nonsense codon	unpublished^s^
50	*Tursiops truncatus*	Common bottlenose dolphin	turTru2	NA	NA	NA	exon 2: 4-nt deletion; exon 3: 1-nt deletion; 7-nt deletion; exon 5: 1-nt insertion; exon 9: two nonsense codons; exon 12: splice donor mutation (GT to AT); exon 13: 2-nt deletion	[47]
51	*Neophocaena phocaenoides*	Finless porpoise	SRX326372				exon 2: 4-nt deletion; exon 3: 1-nt deletion; exon 4: splice acceptor mutation (AG to GG); exon 5: 1-nt insertion; exon 6: splice acceptor deletion; exon 9: nonsense codon	[10]
52	*Lipotes vexillifer*	Baiji	KE559720	NA	NA	NA	exon 1: start codon mutation (ATG to GTG); exon 2: 4-nt deletion; exon 3: splice acceptor mutation (AG to CG); 1-nt deletion; exon 5: 1-nt insertion; exon 6: nonsense codon; exon 9: nonsense codon; exon 11: 1-nt insertion; nonsense codon; exon 13: nonsense codon	[67]
53	*Physeter macrocephalus*	Sperm whale	SRP015690	NA	NA	NA	gene deletion	unpublished^t^
54	*Balaenoptera acutorostrata*	Minke whale	KI537599	NA	NA	NA	exon 2: nonsense codon; exon 6: splice acceptor deletion	[10]
55	*Balaenoptera physalus*	Fin whale	SRX323050	NA	NA	NA	exon 1: nonsense codon; exon 2: nonsense codon, polymorphic; exon 6: nonsense codon; exon 7: nonsense codon; exon 10: splice acceptor mutation (AG to AA); exon 11: 1-nt insertion; exon 13: 2-nt insertion; 1-nt insertion	[10]
56	*Erinaceus europaeus*	Western European hedgehog	AMDU01081674, AMDU01081675	617	Yes	Yes		unpublished^u^
57	*Loxodonta africana*	African bush elephant	AAGU03080315	618	Yes	Yes		unpublished^v^
58	*Trichechus manatus latirostris*	Florida manatee	AHIN01087863	618	Yes	Yes		unpublished^w^
59	*Elephantulus edwardii*	Cape sengi (elephant shrew)	AMGZ01099393, AMGZ01099394, AMGZ01099395	614	Yes	Yes		unpublished^x^
60	*Chrysochloris asiatica*	Cape golden mole	AMDV01101065, AMDV01101067	616	Yes	Yes		unpublished^y^
61	*Echinops telfairi*	Lesser hedgehog tenrec	AAIY02229631	620	Yes	Yes		unpublished^z^
62	*Dasypus novemcinctus*	Nine-banded armadillo	AAGV03115163	618	Yes	Yes		[47]
63	*Monodelphis domestica*	Gray short-tailed opossum	AAFR03013986	614	Yes	Yes		[68]
64	*Sarcophilus harrisii*	Tasmanian devil	AFEY01427030	614	Yes	Yes		[69]

a Endoplasmic reticulum signal peptide predicted by SignalP.

b Glycosylphosphatidylinositol anchor signal predicted by PredGPI.

c Not applicable.

d Gibbon Genome Sequencing Consortium (2012) (http://genome.ucsc.edu/goldenPath/credits.html#gibbon_credits).

e Rogers and Fawcett (2012) (https://www.hgsc.bcm.edu/non-human-primates/gibbon-genome-project).

f International *Macaca fascicularis* Genome Sequencing Consortium (2013) (http://genome.wustl.edu/genomes/detail/macaca-fascicularis).

g Liu et al. (2012) (http://genome.ucsc.edu/goldenPath/credits.html#baboon_credits).

h International *Chlorocebus aethiops sabeus* Genome Analysis Consortium (2013) (http://genome.wustl.edu/genomes/detail/chlorocebus-aethiops).

i The Broad Institute Genome Assembly & Analysis Group, Computational R&D Group, and Sequencing Platform (2011) (http://genome.ucsc.edu/goldenPath/credits.html#squirrel_monkey_credits).

j Warren et al. (2009) (https://www.hgsc.bcm.edu/marmoset-genome-project).

k Di Palma et al. (2009) http://genome.ucsc.edu/goldenPath/credits.html#rabbit_credits).

l Di Palma et al. (2012) (http://genome.ucsc.edu/goldenPath/credits.html#naked_mole-rat_credits).

m Di Palma et al. (2012) (http://www.broadinstitute.org).

n Lindblad-Toh et al. (2010) (http://genome.ucsc.edu/goldenPath/credits.html#microbat_credits).

o Di Palma et al. (2013) (http://www.broadinstitute.org/software/allpaths-lg/blog/?p=647).

p Liu et al. (2012) (https://sites.google.com/site/marinemammalgenomics/project-definition#TOC-Walrus-Odobenus-rosmarus).

q Di Palma et al. (2011) (http://genome.ucsc.edu/goldenPath/credits.html#ferret_credits).

r Di Palma et al. (2012) (http://genome.ucsc.edu/goldenPath/credits.html#white_rhinoceros_credits).

s Foote et al. (2012) (https://sites.google.com/site/marinemammalgenomics/project-definition#TOC-Killer-whale-Orcinus-orca).

t Walter et al. (2013) (http://genome.wustl.edu/genomes/detail/physeter-macrocephalus).

u Di Palma et al. (2012) (http://genome.ucsc.edu/goldenPath/credits.html#hedgehog_credits).

v Di Palma et al. (2009) (http://genome.ucsc.edu/goldenPath/credits.html#elephant_credits).

w Di Palma et al. (2011) (http://genome.ucsc.edu/goldenPath/credits.html#manatee_credits).

x Di Palma et al. (2012) (http://www.broadinstitute.org/software/allpaths-lg/blog/?p=572).

y Di Palma et al. (2012) (http://www.broadinstitute.org/software/allpaths-lg/blog/?p=549).

z Di Palma et al. (2012) (http://genome.ucsc.edu/goldenPath/credits.html#tenrec_credits).

To identify the *MOXD2* gene cDNA sequences from species for which genome sequences were not available, we analyzed high-throughput sequencing data available in the NCBI Sequence Read Archive (SRA) (http://www.ncbi.nlm.nih.gov/sra). Genome sequence data for the Bornean orangutan (*Pongo pygmaeus*), the aye-aye (*Daubentonia madagascariensis*), the finless porpoise (*Neophocaena phocaenoides*), and the fin whale (*Balaenoptera physalus*), and transcriptome sequence data for the gray mouse lemur (*Microcebus murinus*) were obtained from the SRA. Sequences for *MOXD2* gene exons were identified using a locally-installed BLASTN program (version 2.2.24+). Chimpanzee and bottlenose dolphin *MOXD2* exons were used as queries for primates and whales, respectively. The BLASTN command line parameter was “-num_descriptions 10000 -num_alignments 10000”. Matched reads with an expectation (E) value of 1 or less were extracted. Paired-end reads were split to separate reads. CAP3 (version date 12/21/07) was used to align and assemble sequences into contigs [20]. For the assembly of Bornean orangutan sequences, which were short and showed comparatively high heterogeneity, we used parameters “-k 0 -m 5 -n -1 -o 16 -p 66” to relax the stringency. Otherwise, we used the default parameters. CAP3 alignments for exons are presented in Data S1.

To determine whether the *MOXD2* gene loci were deleted in gibbon species, we examined WGS reads of the eastern hoolock gibbon (*Hoolock leuconedys*), the silvery gibbon (*Hylobates moloch*), the pileated gibbon (*Hylobates pileatus*), and the siamang (*Symphalangus syndactylus*). The sequence for the chimpanzee *MOXD2-PRSS58* genomic locus (panTro4 chr7:143686156-143725295) was downloaded from the UCSC Genome Browser database. Gibbon sequences matching the repeat-masked chimpanzee *MOXD2-PRSS58* genomic locus were identified by BLASTN (version 2.2.24+) with the command line parameter “-num_descriptions 10000 -num_alignments 10000”. Gibbon genome reads that aligned the chimpanzee genomic sequence with 90% sequence identity or greater, and with query coverage of 90% or longer, were selected as matches. Paired-end reads were split to separate reads. The genomic regions covered by gibbon sequences were plotted using gnuplot software (version 4.4 patchlevel 3) (http://www.gnuplot.info). Additionally, the Bornean orangutan WGS reads were analyzed as a control.

### Sequence analyses

SignalP 4.1 (http://www.cbs.dtu.dk/services/SignalP) was used to predict ER signal peptides [21], PredGPI (http://gpcr.biocomp.unibo.it/predgpi) was used to predict GPI anchor signals [22], and the Pfam database (http://pfam.sanger.ac.uk) was used to predict domain organization [23]. RepeatMasker (http://www.repeatmasker.org) was used to search for repetitive elements in genomic sequences. Advanced PipMaker and MultiPipMaker (http://pipmaker.bx.psu.edu/pipmaker) were used to produce dot plots [24]. All analyses were performed using each respective web server with the default settings. Multiple sequence alignments of cDNA, exon, or protein sequences were performed using locally-installed MUSCLE software (v3.8.31) (http://www.drive5.com/muscle) with the default parameters [25].

### Statistical analyses

The ratio of nonsynonymous to synonymous substitution rates (dN/dS, ω) was estimated by a likelihood method implemented in the codeml program of the PAML package (version 4.7a) [26]. We prepared two datasets: one for the catarrhine primate *MOXD2* gene, and the other for the whale *MOXD2* gene.

For the catarrhine primate dataset, we collected coding sequences of *MOXD2* genes from 13 primate species: humans, chimpanzees, lowland gorillas, Sumatran orangutans, rhesus macaques, olive baboons, green monkeys, black-capped squirrel monkeys, common marmosets, Philippine tarsiers, aye-ayes, gray mouse lemurs, and Northern greater galagos. Sequences from the bonobo, Bornean orangutan, and crab-eating macaque were not included because they were almost identical to chimpanzee, Sumatran orangutan, and rhesus macaque sequences, respectively. Sequences of exons 12 and 13 were removed from the alignment because they are absent in humans. The nonsense codon position in orangutan exon 8 was also removed. The final sequence dataset comprised 1494 nucleotides. Sequence data files, tree files, control files, and major results files for the codeml analyses are provided in Data S2.

For the whale dataset, we collected *MOXD2* coding sequences from six whales (killer whales, bottlenose dolphins, finless porpoises, baijis, minke whales, and fin whales), cows, and pigs. Disrupted codon positions in the whale sequences caused by nonsense mutation, insertion, and deletion were excluded from the aligned sequence data. The final sequence dataset comprised 1791 nucleotides. Sequence data files, tree files, control files, and major results files for the codeml analyses are provided in Data S3.

Twice the log likelihood difference [2Δ(ln L)] and the degree of freedom (df) were calculated for likelihood ratio tests between selected models. The *P* values for likelihood ratio tests were calculated using pchisq function (with the option “lower.tail = FALSE”) implemented in R software (version 2.14.1) (http://www.r-project.org).

## Results

### Identification of *MOXD2* genes in 64 mammalian species

We previously identified *MOXD2* genes in 12 mammalian species [14]. In our prior study, we showed that the human, Sumatran orangutan, and Bornean orangutan *MOXD2* genes are non-functional due to multiple deleterious mutations. Similarly, the rhesus macaque *MOXD2* gene had a 13-nt deletion in the last exon, resulting in a C-terminal truncation and loss of the GPI anchor signal. The *MOXD2* gene was previously proposed to be associated with mammalian olfactory function, and we concluded that these mutations may be implicated in the alteration of olfaction.

**Figure 1:**
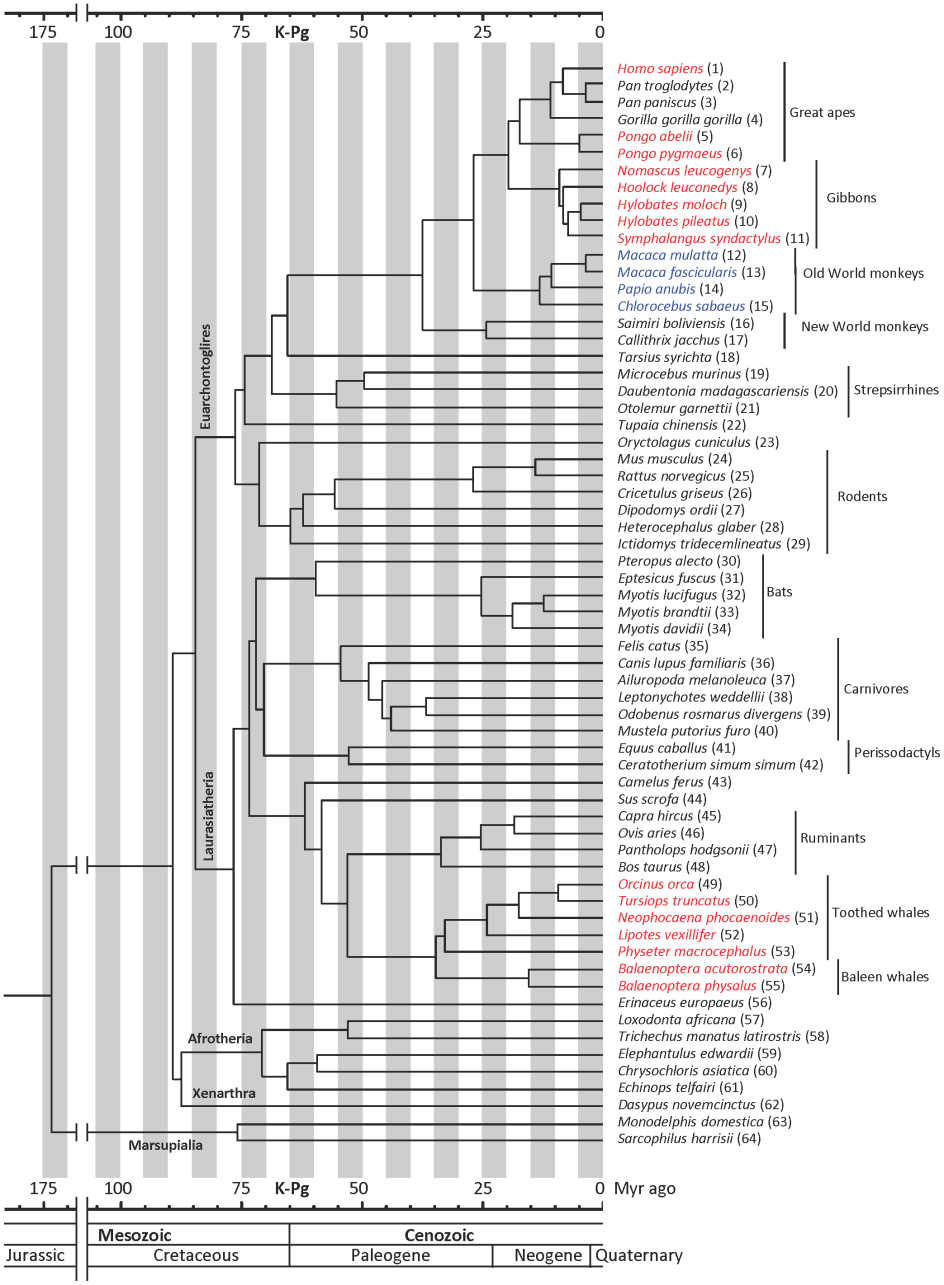
Phylogenetic tree of the species investigated in this study. The major groups are indicated either on the branch or to the right. Species with a mutated *MOXD2* gene are indicated by color: red, gene loss by gene deletion, exon deletion, or ORF-disrupting mutations; blue, C-terminal truncation due to a 13-nt deletion in the last exon. Numbers in parentheses correspond to those in Table 1. K-Pg, Cretaceous-Paleogene boundary.

These results prompted us to perform the extensive analysis described herein on genome sequences of diverse mammalian species to investigate the molecular evolution of the *MOXD2* gene, and its possible connection with olfaction. In this study, we analyzed genome assemblies, WGS contigs, and WGS and transcriptome reads to identify *MOXD2* genes from 64 mammalian species, including members of the Euarchontoglires, Laurasiatheria, Afrotheria, Xenarthra, and Marsupialia. A complete list and phylogenetic tree of taxa analyzed are shown in Table 1 and Figure 1, respectively. The geologic timescale of mammalian phylogeny is primarily based on the report of dos Reis et al. [27] and supplemented by other data [28]–[31].

The complete 13 coding exons of *MOXD2* genes were identified in 53 species out of 64 reported in this study, 49 of which produced intact proteins (Data S1). The remaining 15 species, for which the *MOXD2* gene exhibited deleterious mutations, consisted of humans, Sumatran orangutans, Bornean orangutans, northern white-cheeked gibbons, eastern hoolock gibbons, silvery gibbons, pileated gibbons, siamangs, killer whales, bottlenose dolphins, finless porpoises, baijis, sperm whales, minke whales, and fin whales (see Table 1 for the detailed list of mutations). As previously reported, the human and orangutan *MOXD2* genes exhibit a deletion of the last 2 exons and multiple inframe nonsense codons, respectively [14]. Furthermore, the *MOXD2* gene is absent in the five gibbon species due to a genomic deletion (see below), while *MOXD2* genes of the six whale species have multiple open reading frame (ORF)-disrupting mutations in their coding sequences. Lastly, the *MOXD2* gene appeared to be absent in sperm whales (see below).

Figure S1 shows the multiple sequence alignment of 49 full-length MOXD2 proteins together with the partial human sequence, and the Sumatran orangutan sequence with inframe stop codons. Both marsupial and placental sequences were strongly conserved; specifically, the chimpanzee and the gray short-tailed opossum (*Monodelphis domestica*) MOXD2 proteins exhibited an 82.4% amino acid sequence identity.

The MOXD2 protein was previously reported to contain an ER signal peptide and a GPI anchor signal at its N- and C-termini, respectively [14], with the exception of the rhesus macaque, in which the MOXD2 lacks a C-terminal GPI anchor signal due to a frameshift deletion mutation in the last exon. We analyzed the 49 full-length protein sequences to determine if they had the predicted ER signal peptide and GPI anchor signal. All proteins were predicted to have an ER signal peptide at their N-termini, indicating that they enter the secretory pathway. However, all but four of the sequences were predicted to have a GPI anchor signal at their C-termini, namely, the rhesus macaque, the crab-eating macaque (*Macaca fascicularis*), the olive baboon (*Papio anubis*), and the green monkey (*Chlorocebus sabaeus*). These Old World cercopithecine monkeys shared the same 13-nt deletion in the last exon, resulting in C-terminal truncation and loss of the GPI-anchor (see below).

### Complete deletion of the *MOXD2* gene in gibbons

We searched the northern white-cheeked gibbon (*Nomascus leucogenys*) genome assembly “nomLeu3” available through the UCSC Genome Browser for a gibbon *MOXD2* genomic sequence. There were no matching sequences in the current northern white-cheeked gibbon genome assembly. We then searched the NCBI sequence databases, including WGS contigs, expressed sequence tags, and the Trace Archive, but failed to find any matches.

**Figure 2:**
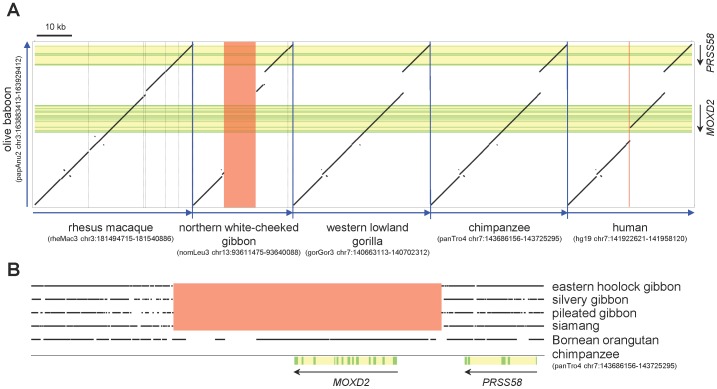
Complete deletion of the *MOXD2* gene in gibbons. (A) Dot plots between the olive baboon *MOXD2*-*PRSS58* genomic locus and orthologous regions from five other primate species (the rhesus macaque, the northern white-cheeked gibbon, the western lowland gorilla, the chimpanzee, and the human) are depicted. The northern white-cheeked gibbon *MOXD2* gene is replaced by a translocated DNA fragment (red box). The locations of the *MOXD2* and *PRSS58* genes are indicated at the right (downward arrows), with coding regions and noncoding regions (introns and untranslated regions) in green and yellow, respectively. The human genomic segment containing *MOXD2* exons 12 and 13 was deleted after the human-chimpanzee divergence (red vertical line). Gray vertical lines in the rhesus macaque and the northern white-cheeked gibbon genomes mark short assembly gaps. Blue arrows indicate chromosome direction (from p- to q-telomere). (B) Deletion of *MOXD2* gene in four other gibbon species. WGS reads from the hoolock gibbon, the silvery gibbon, the pileated gibbon, the siamang, and the Bornean orangutan were mapped to the repeat-masked chimpanzee *MOXD2-PRSS58* genomic sequence. Where matched WGS read positions are marked with dots, the *MOXD2* gene region (red box) lacked matching gibbon WGS reads but not orangutan WGS reads, indicating that the region is missing in these four gibbon species. The locations of the *MOXD2* and *PRSS58* genes are indicated at the bottom with coding and noncoding regions in green and yellow, respectively. Parentheses indicate the genome assembly version and position for each species available from the UCSC Genome Browser database.

The absence of sequence data raised the possibility of complete deletion of the *MOXD2* gene in the northern white-cheeked gibbon, or incomplete sequencing of the *MOXD2* genomic locus. To further assess these explanations, we searched the northern white-cheeked gibbon genome sequence for the *PRSS58* gene, which is located close to the *MOXD2* gene in humans and other mammalian species. We successfully located the northern white-cheeked gibbon *PRSS58* genomic locus, which was orthologous to the *MOXD2-PRSS58* genomic loci of humans and other mammalian species. We then compared the *MOXD2-PRSS58* genomic locus of the olive baboon and those of the rhesus macaque, northern white-cheeked gibbon, Sumatran orangutan, western lowland gorilla, chimpanzee, and human (Figure 2A), which revealed that the *MOXD2* gene was indeed absent from the northern white-cheeked gibbon genome. Therefore, this deletion was not due to an incomplete northern white-cheeked gibbon genome, because there was no sequence assembly gap in its genomic region orthologous to the *MOXD2* gene locus of other primates. Interestingly, the northern white-cheeked gibbon *MOXD2* gene locus was not simply deleted, but was replaced with a DNA fragment rich in repetitive elements.

Comparison of the *MOXD2-PRSS58* genomic loci of the northern white-cheeked gibbon and the chimpanzee genome assemblies revealed that the 22.5 kb-long genomic fragment containing the entire chimpanzee *MOXD2* gene was replaced with an 11.8 kb-long fragment in the gibbon genome (Figure S2A). There were multiple gibbon WGS trace data that spanned each of the two replacement boundaries, indicating that our evaluation of the northern white-cheeked gibbon genome sequence was not due to erroneous assembly (Figure S2B,C).

There are four extant gibbon genera: *Nomascus*, *Hoolock*, *Hylobates*, and *Symphalangus* [32]. To determine whether the *MOXD2* gene deletion in the *Nosmacus* species is shared with the other three gibbon genera, we examined WGS reads derived from four gibbon species: the eastern hoolock gibbon (a *Hoolock* species), the silvery gibbon and the pileated gibbon (*Hylobates* species), and the siamang (a *Symphalangus* species). When WGS reads were mapped to the chimpanzee *MOXD2-PRSS58* genomic locus, no WGS read mapped to the chimpanzee *MOXD2* gene segment (Figure 2B), indicating that the *MOXD2* genomic locus was also deleted in these three gibbon genera. Because the *MOXD2* gene is absent in all the four extant gibbon genera, the deletion event must have preceded the divergence of these genera, which occurred more than 8 million years ago [27], [32].

### Effect of the GPI anchor signal loss on MOXD2 protein localization in the Old World cercopithecine monkeys

**Figure 3:**
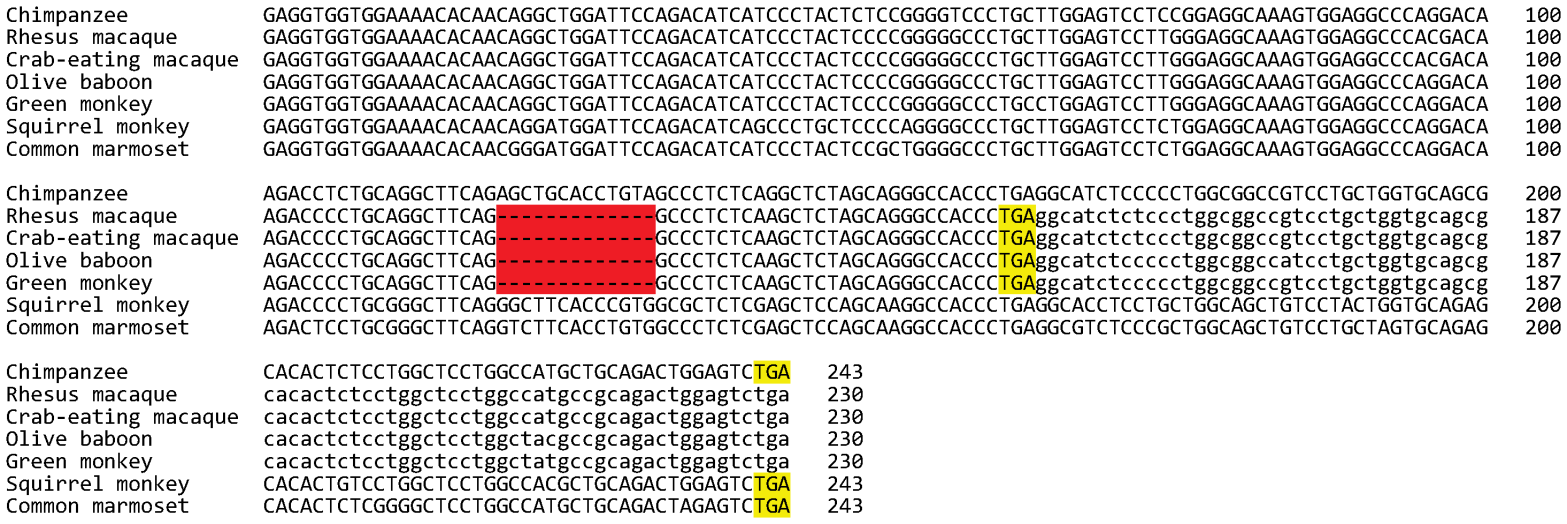
A 13-nt deletion in Old World cercopithecine monkey *MOXD2* exon 13. The *MOXD2* exon 13 sequences from the chimpanzee (*Pan troglodytes*), the rhesus macaque (*Macaca mulatta*), the crab-eating macaque (*Macaca fascicularis*), the olive baboon (*Papio anubis*), the green monkey (*Chlorocebus sabaeus*), the squirrel monkey (*Saimiri boliviensis*), and the common marmoset (*Callithrix jacchus*) were subjected to multiple alignment. The 13-nt deletion, which was common to the four cercopithecine monkeys, is highlighted in red. Stop codons are highlighted in yellow. Note the premature stop codons present in the cercopithecine monkey *MOXD2* genes caused by a frameshift deletion. Uppercase and lowercase letters indicate coding and noncoding sequences, respectively.

We previously showed that the rhesus macaque, an Old World cercopithecine monkey species, had a 13-nt deletion in the last exon that resulted in C-terminal truncation and loss of the GPI anchor signal [14]. In this study, we identified the *MOXD2* genes from three additional cercopithecine monkey species, namely, the crab-eating macaque, the olive baboon, and the green monkey. All of these species exhibited a 13-nt deletion in the last exon, indicating that the deletion was present in a common ancestor of these four species (Figure 3). However, following the 13-nt deletion, the coding sequence integrity was maintained up to about 13 million years after a common ancestor of these species might have lived [27]. Because the deletion occurred in the last exon and there are no other deleterious mutations, the cercopithecine monkey *MOXD2* genes still produce functional proteins.

The 13-nt deletion described above results in a loss of the GPI anchor signal of the MOXD2 protein, which is required for membrane attachment to either the inner aspect of the vesicular membrane or outer surface of the plasma membrane [14]. Therefore, the cercopithecine monkey MOXD2 proteins do not localize on the membrane. Instead, mutation results in a soluble protein that is localized within vesicles and/or secreted from cells. The *DBH* gene, a paralog of *MOXD2*, encodes both soluble and membrane-bound isoforms of dopamine-β-hydroxylase within vesicles [17]. If the MOXD2 protein is contained in the vesicles as DBH, the soluble cercopithecine monkey MOXD2 may maintain the original molecular function and phenotype.

### Inactive *MOXD2* genes in toothed whales and baleen whales

We examined genome assemblies and WGS reads of seven whale species. Five were toothed whale species: the killer whale (*Orcinus orca*), the bottlenose dolphin (*Tursiops truncatus*), the finless porpoise (*Neophocaena phocaenoides*), the baiji (*Lipotes vexillifer*), and the sperm whale (*Physeter macrocephalus*). The other two were baleen whale species: the minke whale (*Balaenoptera acutorostrata*) and the fin whale (*Balaenoptera physalus*).

**Figure 4:**
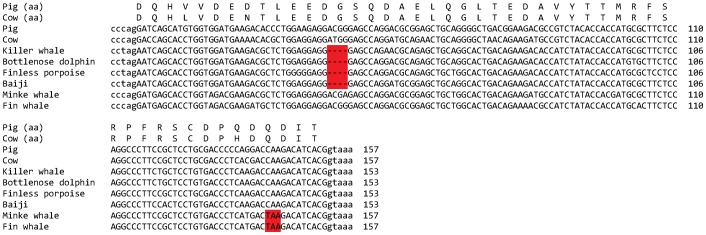
Disruptive mutations of the *MOXD2* gene in whales. Exon 2 sequences of the pig (*Sus scrofa*), the cow (*Bos Taurus*), the killer whale (*Orcinus orca*), the bottlenose dolphin (*Tursiops truncatus*), the finless porpoise (*Neophocaena phocaenoides*), the baiji (*Lipotes vexillifer*), the minke whale (*Balaenoptera acutorostrata*), and the fin whale (*Balaenoptera physalus*) *MOXD2* genes are shown. Disruptive mutations, a 4-nt deletion in toothed whales, and an inframe nonsense codon in baleen whales, are highlighted in red. Exon and intron sequences are in uppercase and lowercase letters, respectively. Additional mutations in other exons are presented in Figure S3.

The complete exon sequences of the *MOXD2* gene were identified in six of these whale species; the sperm whale *MOXD2* gene was deleted (see below). Upon analyzing the exon sequences of *MOXD2* genes of the six whale species, we found many ORF-disrupting mutations, including frameshift insertions and deletions, nonsense mutations, and splice site mutations (see Table 1 for details). For example, there was a 4-nt deletion in exon 2 that was shared among the four toothed whale species, and an inframe nonsense codon (“TAA”) that was common to the two baleen whale species (Figure 4), indicating that these mutations appeared in a common ancestor of each lineage. There were additional mutations in many exons, indicating that *MOXD2* genes of these whale species are non-functional pseudogenes (Figure S3).

**Figure 5:**
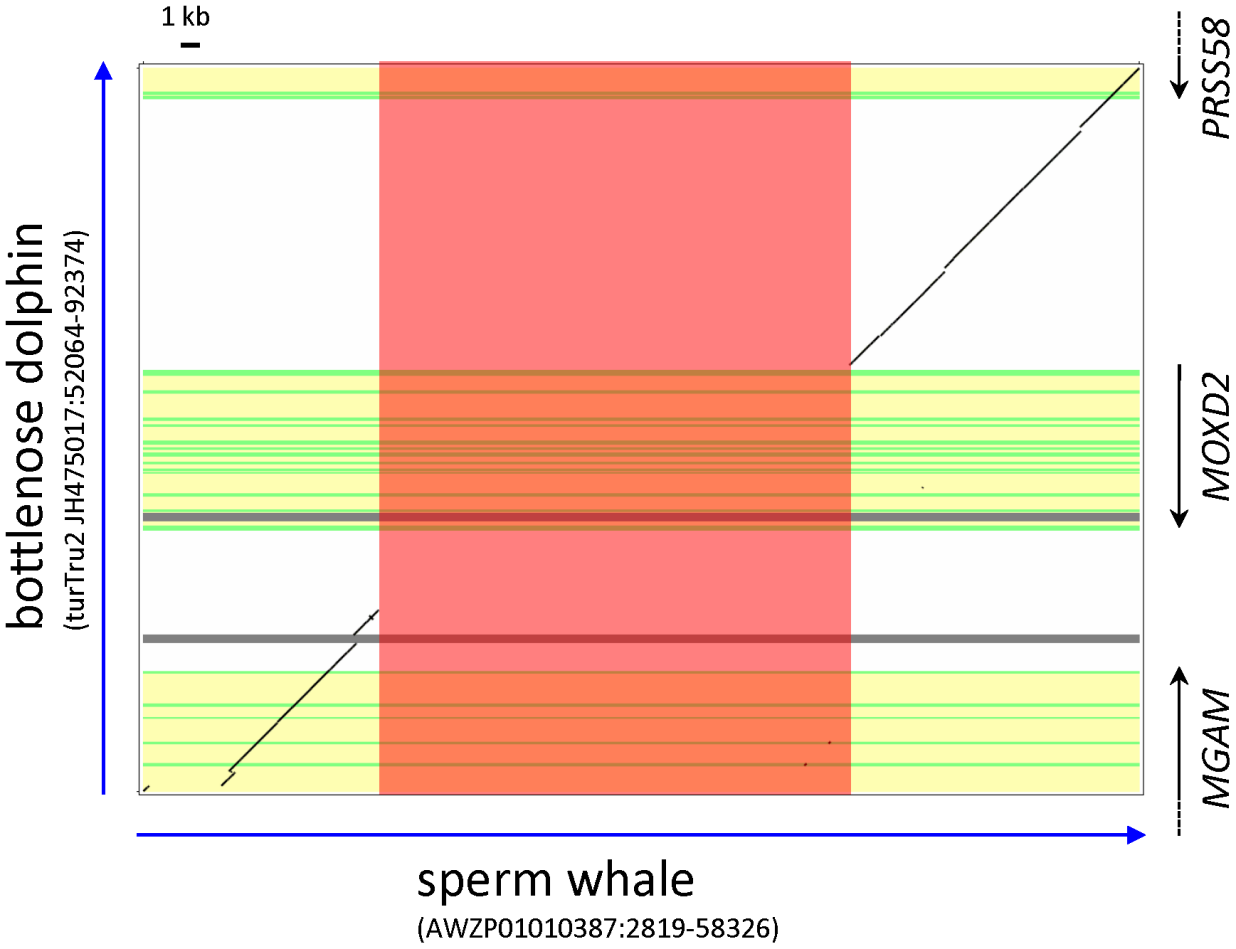
Complete deletion of the *MOXD2* gene in the sperm whale. Sequence comparison of the sperm whale (*Physeter macrocephalus*) (horizontal) and the bottlenose dolphin (*Tursiops truncatus*) (vertical) *MOXD2* gene loci is presented. In the sperm whale, the *MOXD2* genomic region was replaced with a repeat-rich segment (red box). The bottlenose dolphin *MGAM*, *MOXD2*, and *PRSS58* genes are marked at the right, with coding regions and introns in green and yellow, respectively. Gray horizontal lines indicate gaps in the bottlenose dolphin genome assembly “turTru2”.

When we searched the sperm whale WGS contigs for the *MOXD2* genomic sequence, there were no matching sequence data. To determine if the *MOXD2* gene was deleted in the sperm whale genome as in gibbons, the sperm whale WGS contigs were searched for the *PRSS58* gene, which is located close to the *MOXD2* gene in other mammalian species. We were able to locate the sperm whale *PRSS58* genomic locus, which was orthologous to the *MGAM-MOXD2-PRSS58* genomic loci of the cow (*Bos taurus*) and the bottlenose dolphin. Upon comparing the *MGAM-MOXD2-PRSS58* genomic loci of the sperm whale and the bottlenose dolphin, we found that the *MOXD2* gene was absent from the sperm whale genome (Figure 5). As in gibbons, the sperm whale *MOXD2* gene locus was replaced with a repeat-rich DNA fragment.

The large number of deleterious mutations and gene deletion in both toothed whales and baleen whales clearly indicated that the *MOXD2* gene lost its functionality during the evolution of whales. The habitat and ecology of whales may have been associated with relaxation of selection pressure on *MOXD2* gene, which has subsequently allowed it to degenerate or be deleted.

### Evolutionary analysis of catarrhine *MOXD2*

Codeml analyses and likelihood ratio tests were performed in the same manner as in Jiang et al.'s study [33], which reported frequent and independent inactivation of the type 1 taste receptor Tas1r2/Tas1r3 in carnivorans. To evaluate whether selective pressure was relaxed on humans and orangutans with an inactivated *MOXD2*, and possibly on gorillas and chimpanzees with an intact *MOXD2*, we performed likelihood ratio tests (Tables 2 and 3). First, the average ω across the 13 primate species was estimated to be 0.18967 (one-ratio model A in Table 2), which was significantly different from 1 (one-ratio model B) (*P* = 2.34×10^−74^; B vs A in Table 3), indicating an overall purifying selection in species of the data set.

**Table 2:** Models and parameter estimates for likelihood ratio tests of selective pressure on catarrhine *MOXD2* gene^a^.

Model	Description	ω (dN/dS)	ln L^b^	np^c^
A	All branches have the same ω0	ω0 = 0.18967	−4803.2004	25
B	All branches have the same ω0 = 1	ω0 = 1	−4969.6092	24
C	The human and orangutan branches have ω1; other branches have ω0	ω0 = 0.16649, ω1 = 0.70206	−4793.1791	26
D	The human and orangutan branches have ω1 = 1; other branches have ω0	ω0 = 0.16660, ω1 = 1	−4793.7635	25
E	The human, gorilla, and orangutan branches have ω1; other branches have ω0	ω0 = 0.15984, ω1 = 0.77453	−4789.1776	26
F	The human, gorilla, and orangutan branches have ω1 = 1; other branches have ω0	ω0 = 0.15994, ω1 = 1	−4789.5358	25
G	The great ape branches and the ancestral branch leading to great apes have ω1; other branches have ω0	ω0 = 0.16431, ω1 = 0.39834	−4796.8743	26
H	The great ape branches and the ancestral branch leading to great apes have ω1 = 1; other branches have ω0	ω0 = 0.16451, ω1 = 1	−4804.5961	25
I	The human, gorilla, orangutan, and cercopithecine monkey branches, and the ancestral branch leading to cercopithecine monkeys have the ω1; other branches have ω0	ω0 = 0.15182, ω1 = 0.43710	−4792.2753	26
J	The human, gorilla, orangutan, and cercopithecine monkey branches, and the ancestral branch leading to cercopithecine monkeys have ω1 = 1; other branches have ω0	ω0 = 0.15199, ω1 = 1	−4800.1977	25
K	The human, gorilla, and orangutan branches have the ω1; the cercopithecine monkey branches and the ancestral branch leading to cercopithecine monkeys have the ω2; other branches have ω0	ω0 = 0.15194, ω1 = 0.77665, ω2 = 0.24051	−4788.1184	27
L	Each branch has its own ω	variable ω^d^	−4780.1982	47

a The sequence data file, tree files, control files, and main result files for the codeml analyses were provided in Data S2.

b The natural logarithm of the likelihood value.

c Number of parameters.

d See Figure 6A.

**Table 3:** Likelihood ratio tests of selective pressure on catarrhine *MOXD2* gene^a^.

Models compared	2Δ(ln L)^b^	df^c^	*P* value	Significance level^d^
B vs A	332.818	1	2.34×10^−74^	***
A vs C	20.0427	1	7.57×10^−6^	***
D vs C	1.1689	1	0.2796	ns
C vs L	25.9617	21	0.2079	ns
A vs E	28.0456	1	1.18×10^−7^	***
F vs E	0.7165	1	0.3973	ns
E vs L	17.9588	21	0.6516	ns
A vs G	12.6522	1	3.75×10^−4^	***
H vs G	15.4436	1	8.50×10^−5^	***
G vs L	33.3522	21	0.04245	*
A vs I	21.8502	1	2.95×10^−6^	***
J vs I	15.8447	1	6.88×10^−5^	***
I vs L	24.1542	21	0.2856	ns
J vs L	39.9989	22	0.01081	*
A vs K	30.1641	2	2.82×10^-7^	***
E vs K	2.1185	1	0.1455	ns
K vs L	15.8403	20	0.7265	ns

a Models in Table 3 were compared using likelihood ratio test.

b Twice the difference in log likelihood values between the two models compared.

c Degree of freedom.

d ***, *P*<0.001; **, *P*<0.01; *, *P*<0.05; ns, not significant.

**Figure 6:**
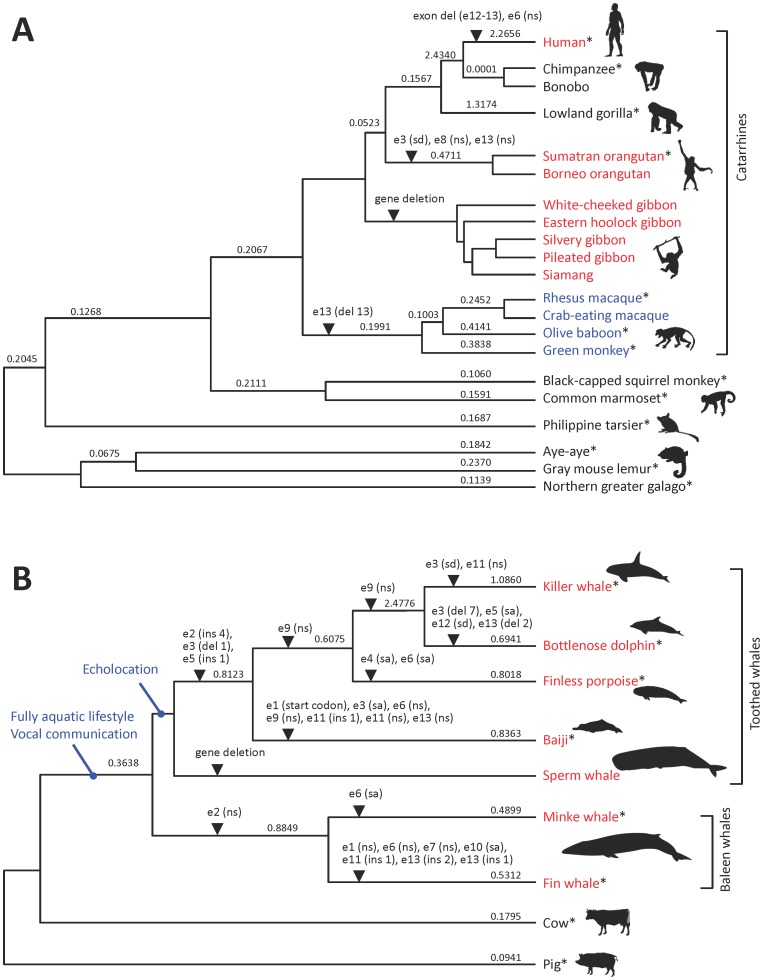
Molecular evolution of the *MOXD2* gene in catarrhines and whales. Molecular evolutionary histories of the *MOXD2* gene in catarrhines (A) and whales (B) are presented. Gene-disrupting mutations are indicated with arrowheads. dN/dS ratios (ω) estimated by the “free-ratio” model are presented on each branch. Asterisks indicate species of which coding sequences were used for the dN/dS analysis. e#, exon number; ins, insertion (followed by number of inserted residues); del, deletion (followed by number of deleted residues); ns, nonsense mutation; sa, splice acceptor mutation; sd, splice donor mutation.

To test whether the selection pressure was relaxed on humans and orangutans, a two-ratio model was tested (model C in Table 2), with the assumption of a uniform ω for human and orangutan branches (ω1), and for other branches (ω0), respectively. In this model, ω0 was estimated to be 0.16649, similar to that estimated in model A. However, ω1 was estimated to be 0.70206, indicating that selection pressure on human and orangutan *MOXD2* was relaxed. This two-ratio model C fitted significantly better than one-ratio model A (*P* = 7.57×10^−6^; A vs C in Table 3), also implying that the selection pressure was relaxed in the human and orangutan branches. Furthermore, when we tested another two-ratio model (model D in Table 2) that allowed ω1 (humans and orangutans) to be fixed to 1, and assumed a uniform ω0 for other branches, this model was not significantly different from model C (*P* = 0.2796; D vs C in Table 3), consistent with the scenario of relaxed selection pressure on human and orangutan *MOXD2* (ω1 = 1). An alternative model (model L in Table 2), in which ω was allowed to vary among branches, was not significantly different from the two-ratio model C (*P* = 0.2079; C vs L in Table 3). Figure 6A shows values of ω on each branch, as estimated by model L.

To examine whether selection pressure was also relaxed on gorillas, which had an intact *MOXD2* coding sequence, a two-ratio model (model E in Table 2) was tested, with the assumption of a uniform ω for human, gorilla, and orangutan branches (ω1), and for other branches (ω0), respectively. In this model, ω0 was estimated to be 0.15984, slightly smaller than that estimated in model C; ω1 was estimated to be 0.77453, slightly larger than that in model C. This two-ratio model E also fitted significantly better than the one-ratio model A (*P* = 1.18×10^−7^; A vs E in Table 3), suggesting that selection pressure on gorilla *MOXD2* was also relaxed. Another two-ratio model (model F in Table 2) that allowed ω1 (humans, gorillas, and orangutans) to be fixed to 1, and assumed a uniform ω0 for other branches, showed no difference from model E (*P* = 0.3973; F vs E in Table 3), consistent with the scenario of relaxed selection pressure on gorilla *MOXD2* (ω1 = 1).

We further tested whether selection pressure was relaxed before the divergence of great apes, including chimpanzees and bonobos, which had intact *MOXD2*. We tested a two-ratio model (model G in Table 2) with the assumption of a uniform ω for the great ape branches and the ancestral branch leading to great apes (ω1), and for other branches (ω0), respectively. In this model, ω0 was estimated to be 0.16431; ω1 was estimated to be 0.39834, smaller than those estimated by models C and E. This two-ratio model G was also significantly different from the one-ratio model A (*P* = 3.75×10^−4^; A vs G in Table 3), but the difference between these models was less than that observed between models F and A. Another two-ratio model (model H in Table 2) that allowed ω1 (great ape branches and the ancestral branch leading to great apes) to be fixed to 1, and a uniform ω0 for other branches, was significantly different from model G (*P* = 8.50×10^−5^; H vs G in Table 3), implying that chimpanzee *MOXD2* was under selection. Therefore, it is highly likely that the *MOXD2* gene is still functioning in chimpanzees and bonobos but not in humans, gorillas, orangutans, and gibbons.

To test whether the selection pressure was also relaxed in Old World cercopithecine monkeys as in humans, orangutans, and gorillas, a two-ratio model was tested (model I in Table 2), with the assumption of a uniform ω for human, gorilla, orangutan, and cercopithecine monkey branches, and the ancestral branch leading to cercopithecine monkeys (ω1), and for other branches (ω0), respectively. In this model, ω0 was estimated to be 0.15182, similar to that estimated in model E. However, ω1 was estimated to be 0.43710, largely smaller than that estimated in model E, implying that the selection pressure on cercopithecine *MOXD2* was not relaxed. Furthermore, when we tested another two-ratio model (model J in Table 2) that allowed ω1 to be fixed to 1, and assumed a uniform ω0 for other branches, this model was significantly different from model I (*P* = 6.88×10^−5^; J vs I in Table 3), further indicating that cercopithecine *MOXD2* was under selective pressure. Finally, we tested a three-ratio model (model K in Table 2), with the assumption of a uniform ω for the human, gorilla, and orangutan branches (ω1), for the cercopithecine monkeys and the ancestral branch leading to cercopithecine monkeys (ω2), and for other branches (ω0), respectively. In this model, ω0 and ω1 were estimated to be 0.15194 and 0.77665, similar to those estimated in model E; ω2 was estimated to be 0.24051, indicating that cercopithecine monkey *MOXD2* genes are under selective pressure. This model (K) showed no difference from model E (*P* = 0.1455; E vs K in Table 3), consistent with the scenario that cercopithecine *MOXD2* genes are under selection.

Therefore, it is likely that the *MOXD2* genes are still functioning in chimpanzees, bonobos, and Old World cercopithecine monkeys, and that it was independently inactivated in humans, gorillas, orangutans, and gibbons.

### Evolutionary analysis of whale *MOXD2*

Whale *MOXD2* genes have many disruptive mutations, some of which are shared by two or more species. Three mutations occurred before the divergence of toothed whales examined in this study, and one mutation occurred before the divergence of the two baleen whales. However, there were no ORF-disrupting mutations shared by both the toothed whales and the baleen whales, implying that the gene might be intact in the common ancestor of the extant whales.

To verify whether the selective pressure on whale *MOXD2* was relaxed, we performed likelihood ratio tests (Tables 4 and 5). The average ω across the six whales, cows, and pigs was estimated to be 0.31510 (one-ratio model A in Table 4), which was significantly different from 1 (one-ratio model B) (*P* = 1.90×10^−34^; B vs A in Table 5), and indicated an overall purifying selection in species of this dataset, probably in cows and pigs.

**Table 4:** Models and parameter estimates for likelihood ratio tests of selective pressure on whale *MOXD2* gene^a^.

Model	Description	ω (dN/dS)	ln L^b^	np^c^
A	All branches have the same ω0	ω0 = 0.31510	−5047.2740	15
B	All branches have the same ω0 = 1	ω0 = 1	−5122.1820	14
C	The toothed whale branches, the branch leading to toothed whales, the baleen whale branches, and the ancestral branch leading to baleen whales have ω1; other branches have ω0	ω0 = 0.15926, ω1 = 0.74234	−5013.9409	16
D	The toothed whale branches, the branch leading to toothed whales, the baleen whale branches, and the ancestral branch leading to baleen whales have ω1 = 1; other branches have ω0	ω0 = 0.15938 ω1 = 1	−5015.8933	15
E	The whale branches and the ancestral branch leading to whales have the ω1; other branches have ω0	ω0 = 0.12471 ω1 = 0.69721	−5011.6733	16
F	The whale branches and the ancestral branch leading to whales have ω1 = 1; other branches have ω0	ω0 = 0.12110 ω1 = 1	−5014.6813	15
G	Each branch has its own ω	variable ω^d^	−5006.3099	27

a The sequence data file, tree files, control files, and main result files for the codeml analyses were provided in Data S3.

b The natural logarithm of the likelihood value.

c Number of parameters.

d See Figure 6B.

**Table 5:** Likelihood ratio tests of selective pressure on whale *MOXD2* gene^a^.

Models compared	2Δ(ln L)^b^	df^c^	*P* value	Significance level^d^
B vs A	149.8160	1	1.90×10^−34^	***
A vs C	66.6663	1	3.22×10^−16^	***
D vs C	3.9049	1	0.04815	*
C vs G	15.2620	11	0.1708	ns
A vs E	71.2014	1	3.22×10^−17^	***
F vs E	6.0161	1	0.01418	*
E vs G	10.7269	11	0.4664	ns

a Models in Table 4 were compared using likelihood ratio test.

b Twice the difference in log likelihood values between the two models compared.

c Degree of freedom.

d ***, *P*<0.001; **, *P*<0.01; *, *P*<0.05; ns, not significant.

Because there were no shared mutations between the toothed whales and the baleen whales, we tested two hypotheses using separate two-ratio models (models C and E in Table 4). In model C, a uniform ω for the toothed whale branches, the ancestral branch leading to toothed whales, the baleen whale branches, and the ancestral branch leading to baleen whales (ω1) and for cow and pig branches and the ancestral branch leading to whales (ω0), respectively, was assumed. To examine whether the selection pressure was relaxed in the ancestral branch of both whale clades, we tested a two-ratio model (model E in Table 4) with the assumption of a uniform ω for all the whale branches and the ancestral branch leading to whales (ω1), and for other branches (pigs and cows) (ω0), respectively.

In model C, ω0 was estimated to be 0.15926, while ω1 was estimated to be 0.74234, indicating that the selection pressure on *MOXD2* of both whale clades was relaxed. This two-ratio model C fitted significantly better than the one-ratio model A (*P* = 3.22×10^−16^; A vs C in Table 5). When we tested another two-ratio model (model D in Table 4) that allowed ω1 (both whale clades, the ancestral branch leading to toothed whales, and the ancestral branch leading to baleen whales) to be fixed to 1, and used a uniform ω0 for other branches, we observed that this model was marginally significantly different from model C (*P* = 0.04815; D vs C in Table 5). An alternative model (model G in Table 4) in which ω was allowed to vary among branches was not significantly different from the two-ratio model C (*P* = 0.1708; C vs G in Table 5). Figure 6B shows ω estimated by the model G on each branch.

In model E, ω0 and ω1 were estimated to be 0.12471 and 0.69721, respectively. This two-ratio model E was also significantly different from the one-ratio model A (*P* = 3.23×10^−17^; A vs E in Table 5), suggesting that selection pressure on *MOXD2* in the ancestral branch leading to whales was also relaxed. Another two-ratio model (model F in Table 4) that allowed ω1 (all the whale branches and the ancestral branch leading to whales) to be fixed to 1, and used a uniform ω0 for other branches, was marginally significantly different from model E (*P* = 0.01418; F vs E in Table 5), implying that the selective pressure on ancestral whale *MOXD2* was partially relaxed.

Therefore, it was not clear whether the selection pressure on whale *MOXD2* had become relaxed in the common whale ancestor, or independently in the toothed whale ancestor and the baleen whale ancestor. The relaxation of selection pressure may have occurred close to when the two whale clades diverged.

## Discussion

When organisms adapt to a particular environment where some of their traits are no longer of use, selection pressure on those phenotypes become relaxed, and the associated genes are free to accumulate deleterious mutations [34], [35]. One example of this phenomenon is the diminishment of olfactory function in catarrhine primates and toothed whales [3], [7], [8]. In this study, we found that the *MOXD2* gene was frequently inactivated and altered in catarrhines and whales, which may be associated with the evolution of olfaction in both clades.

The *MOXD2* gene, which is strongly expressed in the olfactory epithelium, has been proposed to be associated with olfactory function, although no direct evidence supporting this hypothesis has been established [14]. The loss of the *MOXD2* gene in gibbons is concordant with the trend of pseudogenization of this gene in humans and orangutans. Human *MOXD2* is inactive due to an exon deletion, and orangutan *MOXD2* has multiple nonsense mutations. Likelihood ratio tests have suggested that selection pressure on western lowland gorilla *MOXD2* might be relaxed. However, statistical analyses have implied that chimpanzee, bonobo, and Old World cercopithecine monkey *MOXD2* genes are under selective constraint. Thus, it is highly likely that the *MOXD2* gene became of no use in some-but not all-catarrhines, and as a result was inactivated during evolution. We hypothesize that loss of the *MOXD2* gene, together with relaxed selection pressure on OR genes [4], may be associated with the altered olfaction in some catarrhines.

Many mammals have secondarily returned to aquatic environments, and acquired adaptive molecular changes in many proteins during evolution [36]. Whales are adapted to a fully aquatic lifestyle, where the detection of airborne odorants is improbable. Indeed, the olfactory apparatus is greatly reduced in some baleen whales, and completely absent in toothed whales; consequently, many OR genes have become pseudogenized [7], [8]. In this study, we found that whale *MOXD2* genes are not functional due to multiple deleterious mutations and gene deletion. We hypothesize that loss of *MOXD2* genes in whales may be associated with altered olfaction concomitant with the evolution of a fully aquatic lifestyle.

It was previously shown that functional OR gene composition in mammals is related to ecological adaptation [11]. Thus, functional OR gene repertoires are reduced in aquatic mammals. In this study, we identified *MOXD2* in three aquatic mammalian species in addition to whale species, namely, the Weddell seal (*Leptonychotes weddellii*), the Pacific walrus (*Odobenus rosmarus divergens*), and the Florida manatee (*Trichechus manatus latrirostris*). The former two are partially aquatic and the latter is fully aquatic. Interestingly, the *MOXD2* genes of these three species do not have any deleterious mutations, indicating that they might be functional.

Inactivation of the transient receptor potential cation channel, subfamily C, member 2 (*TRPC2*) gene in mammalian species exhibits a similar pattern to that of the *MOXD2* gene. The *TRPC2* gene is expressed in the vomeronasal organ, and is a crucial component of pheromone transduction [37]. *TRPC2* is a pseudogene in Old World monkeys and apes [38], as well as in whales, including both baleen and toothed whales [39]. The *TRPC2* gene is inactive in two partially aquatic mammals, the harbor seal (*Phoca vitulina*) and the river otter (*Lutra lutra*), but is intact in the California sea lion (*Zalophus californianus*), which are also partially aquatic, indicating that the gene was randomly lost in aquatic mammals. Frequent loss of genes expressed in olfactory epithelium (*MOXD2*) or vomeronasal organs (*TRPC2*) in catarrhines and whales further demonstrates the diminished olfactory function in these clades, as well as relaxed selection pressure on the associated genes.

### Conclusions

In summary, we analyzed the *MOXD2* genes from 64 mammalian species and identified loss-of-function mutations in humans, orangutans, gibbons, and whales (Figure 6). We hypothesize that the inactivation and alteration of the *MOXD2* gene may be associated with the evolution of olfaction in catarrhine primates and whales. Molecular functional studies of the *MOXD2* gene in model animals with highly sensitive olfaction, such as mice and dogs, may provide direct evidence of its role in olfaction.

## Supporting Information

Figure S1 **Multiple sequence alignment of MOXD2 protein sequences.** Multiple sequence alignment was prepared using MUSCLE. Amino acid sequences that are the same as chimpanzees (*Pan troglodytes*) are marked by dots (.). Stop codons and alignment gaps are denoted by red asterisks (*) and hyphens (−), respectively. The human MOXD2 lacks the C-terminal region due to deletion of exons 12 and 13. The whale sequences were not included due to multiple disruptive mutations. ClustalW consensus labels are shown below sequences: asterisks (*), identical; colons (:), highly conserved; dots (.), moderately conserved.
(PDF)

Figure S2 **Sequence comparison of the chimpanzee (** ***Pan troglodytes*** **) and the northern white-cheeked gibbon (** ***Nomascus leucogenys*** **)** ***MOXD2*** **gene loci.** (A) Dot plot comparison of the chimpanzee (horizontal) and the northern white-cheeked gibbon (vertical) *MOXD2* loci. In the northern white-cheeked gibbon, the *MOXD2* genomic region was replaced with a translocated DNA segment (red box). The chimpanzee *MOXD2* and *PRSS58* genes are marked at the top with coding and non-coding regions (introns and untranslated regions) in green and yellow, respectively. A dotted horizontal line indicates a gap in the northern white-cheeked gibbon genome assembly “nomLeu3”. The left and the right alignment boundaries are marked by dotted blue boxes. (B, C) Alignments of chimpanzee and gibbon sequences for the left (B) and right boundaries (C). Identical residues between the two species are marked by vertical lines. The northern white-cheeked gibbon WGS trace data are in blue. The unaligned region is highlighted in red. Note that the boundary regions are supported by multiple trace data, indicating that *MOXD2* deletion in the northern white-cheeked gibbon was not a result of erroneous assembly.
(PDF)

Figure S3 **Disruptive mutations in the** ***MOXD2*** **genes of whales.** Exon sequences of the pig (*Sus scrofa*), the cow (*Bos taurus*), the killer whale (*Orcinus orca*), the bottlenose dolphin (*Tursiops truncatus*), the finless porpoise (*Neophocaena phocaenoides*), the baiji (*Lipotes vexillifer*), the sperm whale (*Physeter macrocephalus*), the minke whale (*Balaenoptera acutorostrata*), and the fin whale (*Balaenoptera physalus*) *MOXD2* genes are shown. Disruptive mutations are highlighted in red. Ancestral stop codons are highlighted in yellow. Coding and noncoding sequences are in uppercase and lowercase letters, respectively.
(PDF)

Data S1 **Coding and protein sequences of** ***MOXD2*** **genes.**
(PDF)

Data S2 **The sequence data file, tree files, control files, and main result files for the codeml analyses of catarrhine** ***MOXD2*** **gene.**
(PDF)

Data S3 **The sequence data file, tree files, control files, and main result files for the codeml analyses of whale** ***MOXD2*** **gene.**
(PDF)
