# Supplementary material for: Frequent Loss and Alteration of the MOXD2 Gene in Catarrhines and Whales: A Possible Connection with the Evolution of Olfaction
Source: PLoS One. 2014 Aug 7;9(8):e104085. doi: 10.1371/journal.pone.0104085 (PMC4125168; doi:10.1371/journal.pone.0104085)
Supplement: Figure S1 — Multiple sequence alignment of MOXD2 protein sequences. Multiple sequence alignment was prepared using MUSCLE. Amino acid sequences that are the same as chimpanzees (Pan troglodytes) are marked by dots (.). Stop codons and alignment gaps are denoted by red asterisks (*) and hyphens (−), respectively. The human MOXD2 lacks the C-terminal region due to deletion of exons 12 and 13. The whale sequences were not included due to multiple disruptive mutations. ClustalW consensus labels are shown below sequences: asterisks (*), identical; colons (:), highly conserved; dots (.), moderately conserved. (PDF) [file pone.0104085.s001.pdf]

Figure S1. Multiple sequence alignment of MOXD2 protein sequences. Multiple sequence alignment was prepared using MUSCLE. Amino acid sequences that are the same as chimpanzees (*Pan troglodytes*) are marked by dots (.). Stop codons and alignment gaps are denoted by red asterisks (*) and hyphens (-), respectively. The human MOXD2 lacks the C-terminal region due to deletion of exons 12 and 13. The whale sequences were not included due to multiple disruptive mutations. ClustalW consensus labels are shown below sequences: asterisks (*), identical; colons (:), highly conserved; dots (.), moderately conserved.

```
Pan troglodytes                  MAHDLLFRLFPLL-ALG--APLQSNRLGPTSRLRYSRFLDPSNVIFLRWDFDLEAEIISFELQVRTAGWVGFGVTNRYTNVGSDLVVGGVLPNGNVYFSDQHLVDEDTLKEDGSQDAELLGLTED  122
Homo sapiens                     .............-...--V....................................................................................................R....  122
Pan paniscus                     .............-...--..........................................................................................................  122
Gorilla gorilla gorilla          .............-...--V...........................S.............................................................................  122
Pongo abelii                     .............-...--..............C...............................R.....L....................K.......................T........  122
Macaca mulatta                   .............-..A--.......P............................................L................................NG...................  122
Macaca fascicularis              .............-..A--.......P............................................L................................NG...................  122
Papio anubis                     .............-..A--.......P............................................L................................NG..............E....  122
Chlorocebus sabaeus              ..........L..-..A--.......P............................................L................................NG...................  122
Saimiri boliviensis              ..C.......L..-..A--.........................M..L....F...........Q......L.....................................Q...............  122
Callithrix jacchus               ..C.......L..-..A--.A.....................S.M..L.......................L.....................................Q..A............  122
Tarsius syrichta                 .SCA.F....MF.-..I--..SLG....L.....F.........V.....I..K....T............L.IK.................D............D...E..R......Q.....  122
Microcebus murinus               ..CT...G..L..-G.E--..SPG.C.A.........................K..V.T............L...S...RA...........D..........................H...Q.  122
Daubentonia madagascariensis     ..CA.....LL..-..E--..SPG.C.SH...........................V.T............L.......R............D....................N.....H....G  122
Otolemur garnettii               ..CA...C..L..-..E--V.FLG.CF..M..............V........K..F.T............L.......RA..........SD................R.........H....G  122
Tupaia chinensis                 .TCV......L..-V.A--S.S.GK..................A..............T.......T....L..............I.....D................R...............  122
Oryctolagus cuniculus            ..CA.....SL..-..T--..SPGKH...K.........G.....L............T.....Q......L...M...F............D................E..R......Q.....  122
Mus musculus                     ..CV......L..-V.A--.FS.GK......P..........RAV.......Y.....T.....Q.T....L.I.D...F.......................L.....EQ.........R....  122
Rattus norvegicus                .GYF.F....L.W-V.A--.FS.GK..S...P..........HAV.......Y.T...T.....Q.T....L.I.D...F.......................L.....EQ.........K....  122
Cricetulus griseus               ..CV..C..IL.S-V.A--VFS.GK......L..........HAV.......Y.....T.....Q.T....L.I.....F............D..........L...S.EP.........K....  122
Dipodomys ordii                  .SCV......L..-G.V--I.S.GS......P........S..I.........QT...T.....Q.S....L.I........G.........D.K..............E...............  122
Heterocephalus glaber            ..CIH.LKF.L..-..T--.TS.GKH.....H......I....A.........K..A.V.....Q......V.IA....V..G.........D................E...............  122
Ictidomys tridecemlineatus       .TCV...K..L..-V.S--DTCPGSH.....................L....F.....T............L.ISH.H.V..............H...........N..E...............  122
Pteropus alecto                  ..RV..L..LL.T-..A--..S.G....S.P.........................V.T............L...H......G.....A....................E...R...........  122
Eptesicus fuscus                 ..C......LL.M-V.A--..S.G................................M.T............L....G..F..G.........E................EA......K..K....  122
Myotis lucifugus                 ..C......LL.M-V.A--.TS.G.H..............................M.T............L....G..F..A.........D................E.......K.......  122
Myotis brandtii                  ..C......LL.M-V.A--..SRG.H..............................M.T............L....G..F..A.........D................E.......K.......  122
Myotis davidii                   ..C......LL.M-V.A--..SRG.H..............................M.T............L....G..F..A.........D................EM......K.......  122
Felis catus                      .TCA.....LL.M-T.A--.LF.GK.F...............................T............L.......R............D................E.........Q.....  122
Canis lupus familiaris           .TCA.....LL.T-..V--V.SPGKG................................T............L.....NART...........D................E.........Q.....  122
Ailuropoda melanoleuca           .TCA......L.M-..A--V.S.GKC............................T...T.......T....L...S...RA...........D.I..............E.........Q.....  122
Leptonychotes weddellii          .TCA.....LL.T-..A--..S.GKH.....................C..........T.......T....L.......R............D.I..............E.........Q.....  122
Odobenus rosmarus divergens      .TYA.....LL.T-..A--..S.GKH................................T.....Q.T....L.......R............D................E.........Q.....  122
Mustela putorius furo            .TCA.....LL.M-..V--.SS.GK.................................T.......T....L.......R............D................E.........Q..M..  122
Equus caballus                   ..CA.....LL.M-..A--..S.G.GP...............................T............L....................D.I..............A.........Q.....  122
Ceratotherium simum simum        ..CA.....LL.M-...--..S.G.G.S...H...........I..............T............L....................D.......R........E.........R.....  122
Camelus ferus                    ..RA...Q.LL.T-..A--..S.G.H..........................YK....T............L.......R............D................EK........QR....  122
Sus scrofa                       ...A..LQ.LL.S-..A--..S.G.......S.....................D....A...R........L.I.....RA.........S.D.H.......V......E.........Q.....  122
Capra hircus                     ..CP..SW.-LFT-..A--T.S.GD.....PH........A..AV.......F.....T............L......SRA.G.......S.D................E.........Q.....  121
Ovis aries                       ..CP...W.-LFT-V.A--T.S.GDH...AP.........A..AV.......F.....T............L......SRA.G.......S.D................E.........Q.....  121
Pantholops hodgsonii             ..CP...W.-LFT-..A--T.S.GD.....PH........A..AV.......F.....T.....Q......L......SRA.........S.D................E.........Q...Q.  121
Bos taurus                       ..CA..SW.LLFT-..A--T.S.C.....MP............AV.......F.....T............L...D..SRA.........S.D.............N..E.........Q.....  122
Erinaceus europaeus              ..CA.FIGVLL.MVV.A--..T.GRL...S.P...........AM........DS...T...K........L.......RA...........D.......W..L....ME..S......Q..M..  123
Loxodonta africana               .TCT.FSW.IL..-..T--T.S.GHHF..K..............M..C........V.T............L..........G.........D..........................Q..M..  122
Trichechus manatus latirostris   .TCA..S..LL..-..A--..S.GHH...K...........A..M...........V.M.....Q......L..........G.........D................TA........Q.....  122
Elephantulus edwardii            .TGV...Q.VLF.-P.A--..S.GHHH..K..........S...L...........V.T...K........L..........A..........................Q.........Q.....  122
Chrysochloris asiatica           .TRV...W.LLI---.T--P.S.GH....K..........S..DV...........V.T.....Q......L..........G.........G..........K.....E...............  120
Echinops telfairi                ...VF.I..IL..-..TLVTHTKDS....R..............M.......H...V.T...K........L..........G.......................Y..E.........Q.....  124
Dasypus novemcinctus             .TCA.....LL..-V.S--.SS.GHHHSS..H...............S....FD....T...K........L..........G.........D................E..R......Q..M..  122
Monodelphis domestica            .TCV..P..LL..-P.A--..ATGIHH....P............MY........T...T.D.....T....L.I........A.........D.T..........DE..H.........QL.K..  122
Sarcophilus harrisii             .TCA..PK.LL..-T.A--..ATGIPH....P.......G....MY......IQT.V.T.D.....T....L..........A.........D.T..........D.............QL.K..  122
                                 *    :  .  :   *           .  . * :***:..:  : * **:* .:* * *:*.*. :****.*:    : .*.***:*.* . * :****.*: :  ::  *  **::*  * :.
```

```
Pan troglodytes                 AVYTTMRFSRPFRSCDPHDLDITSDTVRVLAAYGLDDTLKLDRERTFVKSIFLLQVVHPDDLDVPEDTIIHDLEITDFLIPEDDTTYACTFLPLPIVSEKHHIYKFEPKLVYHNETTVHHILVYA  247
Homo sapiens                    ......H.................N......T.........Y..................................N................................................  247
Pan paniscus                    .............................................................................................................................  247
Gorilla gorilla gorilla         ...................................N.........................................................................................  247
Pongo abelii                    ......................M...............P...Q.................N.....................................Q.................M........  247
Macaca mulatta                  ................L.........................Q......................K..................................................M........  247
Macaca fascicularis             ..........................................Q......................K..................................................M........  247
Papio anubis                    ..........................................Q......................K..................................................M........  247
Chlorocebus sabaeus             ..........................................Q......................K..................................................M........  247
Saimiri boliviensis             ...................................................................A................................................M........  247
Callithrix jacchus              .I..........................................H......................A................................................M........  247
Tarsius syrichta                .G..............................F..............................Y............E....................NK...........I.....M........  247
Microcebus murinus              ...................Q...A..M................D................................N.....................K...........MAR...M........  247
Daubentonia madagascariensis    ................R..Q...G...................N......V...............................................K.........T..D....M........  247
Otolemur garnettii              ...................Q...G.....M.............N.I................E...................................K............N....M........  247
Tupaia chinensis                ...................Q..A.......T.......V................I......E...................................K...........I.....M........  247
Oryctolagus cuniculus           GI.................Q..MG...........N..VE...............I......E...................................K...........AP....M.....L..  247
Mus musculus                    ..S..........T.....R......M.......P..IP.MS..H..........MLQY..Q.A.........K.SN.I...................K.........I..ER...M...V....  247
Rattus norvegicus               S.S................H......M..........IP.MN.............ILQY..E.A....V.Y..K.SN.....E...............K.........I..ER...M...V....  247
Cricetulus griseus              S.S................R......M..........IP.MNP............MLQY..Q.A.........A.SN.....................K.........L..ER...M........  247
Dipodomys ordii                 GL...............Q.Q......M...T.......P..H.K...........IFYH..T.A...A..Y...........A...............K...........LA....M........  247
Heterocephalus glaber           .A.................Q......I...T......M...R.............IF..E...F....MV............V....................F......TD....M........  247
Ictidomys tridecemlineatus      ...................Q......I...............P............MF.....E...EI..............................K............E....M........  247
Pteropus alecto                 .......................G................M.QG.A.................A...A..............................K...........MD....I........  247
Eptesicus fuscus                G.....L............K......................P............IL....H....................................K............S....M........  247
Myotis lucifugus                D.....L............K.................M....P............I..........................................K.................M........  247
Myotis brandtii                 G.....L............K.................M....P............I..........................................K.................M........  247
Myotis davidii                  G.....L............K......................P............I..........................................K.................M........  247
Felis catus                     ................A..Q......I................D...........I..................L.......................K....F......LN....M........  247
Canis lupus familiaris          ...................Q......M....T...........D...........I.......I..................................K....F.....M.E.D..M........  247
Ailuropoda melanoleuca          ...................Q......I....T..Q.......QD......................................................R....F......LE....M........  247
Leptonychotes weddellii         .I.................K......IK...T...........D...I.......I.......................V..................K....F......PE.H.SM........  247
Odobenus rosmarus divergens     .I.................Q......IK...T...........D...I.......I..........................................K....F......LE....M........  247
Mustela putorius furo           ...................Q......I....T...........D...I.......I..................L.......................K....F......LV....M........  247
Equus caballus                  ...................Q......M....T.......................IF.........................................K............H....M........  247
Ceratotherium simum simum       ...................Q...................................I..........................................K..........M.Q....M........  247
Camelus ferus                   .......S.........Y.Q..............P......H.............MIQ....H...........L.......................K............DRS..M........  247
Sus scrofa                      .................Q.Q..............P..........M.........II......A...A..............................K...........EP....M........  247
Capra hircus                    ...................Q..................P.I..............I.......A..................................K............Q....I........  246
Ovis aries                      ...................Q..................P.I..............I.......A..................................K............Q....I........  246
Pantholops hodgsonii            ...................Q..................P.I.Q............I.......A..................................K............Q.R..V........  246
Bos taurus                      ...................Q..................P.M..............I.......A..................................K............Q.K..I........  247
Erinaceus europaeus             .I.........L.......M..AG..............P................I....E.E...........L.N.....................Q............E....M........  248
Loxodonta africana              .F.................Q..............Q.........HI.........I..........................................K...........AHR...M........  247
Trichechus manatus latirostris  ................H..Q..............Q.........HI.........II.....................................................AH....M........  247
Elephantulus edwardii           ...................Q..............Q...P.....HI.........II.....Q...........L.N.....................K....H......IS....M........  247
Chrysochloris asiatica          ...................H..............Q........DHI.........II.....E...................................K....H......ID...SM........  245
Echinops telfairi               .......Y...........Q..SV..........Q....T.SP.HI.........M......E.....L.............................K....H......I.....M........  249
Dasypus novemcinctus            .K.................Q........................HI.........I......E...................................K....H...Q...D....M........  247
Monodelphis domestica           ................S..G......I...G.......V....K...........I.......A.KISY............................K............I.....M........  247
Sarcophilus harrisii            .............T..S..G......I...........V..E.............II......A.AISY............................K............I.....M........  247
                                   ***  ***:*:**  * **  :*:.*: ::* :*   :    . *:**:****:.  ::   *    ::** :::*::** *************. ****.***  :  . *: ***:*:**
```

```
Pan troglodytes                  CGNASVLPTGISDCYGADPAFSLCSQVIVGWAVGGTSYQFPDDVGVSIGTPLDPQWIRLEIHYSNFNNLPGVYDSSGIRVVYYTSQLRKYDMGVLQLGFFTFPIHFIPPGAESFMSYGLCRTEKFE  372
Homo sapiens                     .....................................................L........................C...........TD..................................  372
Pan paniscus                     ..............................................................................................................................  372
Gorilla gorilla gorilla          .............................................I................................................................................  372
Pongo abelii                     .................................R............................................H...................L.................C.........  372
Macaca mulatta                   .....I.................................................................................................V..........L...........  372
Macaca fascicularis              ..................................................................................................................L...........  372
Papio anubis                     ..................................................................................................................L...........  372
Chlorocebus sabaeus              ..................................................................................................................L...........  372
Saimiri boliviensis              .............................................I.....................................A..........................................  372
Callithrix jacchus               ...S.........................................I.....................................A..........................................  372
Tarsius syrichta                 ........K....................................I....................H...............S.....................................K.D...  372
Microcebus murinus               .....A....................I..................I..............V.....H.R..........M..S.H.........................S.........K.....  372
Daubentonia madagascariensis     .....A.......................................I..............V.....H...............S.....................................K.....  372
Otolemur garnettii               .....A.......................................I..............V.....H.................H...................................K.....  372
Tupaia chinensis                 .....A.......................................I..............V.....H.......T.......SP....................................K.....  372
Oryctolagus cuniculus            ............................A................I....................H....L................................................K.....  372
Mus musculus                     ...S.......GE...S........H..A......L.........I.....F..............Q....IR.T..M.LF...H............ISV............A.L.....K.D...  372
Rattus norvegicus                ...S.......GE............H..A......L.........I.....F..............Q....IR.T..M.L....H............ISV............A.L.....K.D...  372
Cricetulus griseus               ...........GE...S........H..A......L.........I..............V.....Q....IH.T..M.L....H............ISV............A.L.....K.....  372
Dipodomys ordii                  .....T.....DE...F.A.........M......L...L..E..I....................H....I.......L....H.....L.I.E..VL...............K.....K.D...  372
Heterocephalus glaber            .....T..K...E...F.........I........L......E..I..............V.....H............L...TR............VL.......L.............K.....  372
Ictidomys tridecemlineatus       ........K..N.......D......................N..I.L..................H.......T....L....H..E.........V......................K.....  372
Pteropus alecto                  ..................................................................H.R..L..T....M...AR..AH........V...........R....L.....K.....  372
Eptesicus fuscus                 ............................................................V.....H.....H.T....M...AH.............L.....................K.....  372
Myotis lucifugus                 .....A............................................................H.....H.T....M...AH.............L.....................K.....  372
Myotis brandtii                  .....A............................................................H.....H.T....M...AH.............L.....................K.....  372
Myotis davidii                   .....A..M.........................................................H.....H.T....M...AH.............L.....................K.....  372
Felis catus                      ..................................................................H.V..........L...AK.............................L.....K.....  372
Canis lupus familiaris           ...............................................L..................H............L...A..............................L.....K.....  372
Ailuropoda melanoleuca           ...............................................L..................H............L...AR.............................L.....K.....  372
Leptonychotes weddellii          ..H..........................................I....................H............L...A......T.......................L.....K.....  372
Odobenus rosmarus divergens      ..................................................................H............L...A..............................L.....K.....  372
Mustela putorius furo            ..................................................................H....I.......L...A..............................L.....K.....  372
Equus caballus                   .....T......................M................I.L..................H............M...AT...................................K.....  372
Ceratotherium simum simum        .....A......................M................I.L..................H................AE...................................K.....  372
Camelus ferus                    .....T.......................................I....................H............M........H....................H..........K.....  372
Sus scrofa                       .....A.......................................I....................H................AK...................................K.D...  372
Capra hircus                     .....A.............................A.........I....................H....L...........AH...F........V...........S....R.....K.....  371
Ovis aries                       .....T.............................A.........I....................H....L...........AH...F...I....V...........S....R.....K.....  371
Pantholops hodgsonii             .....A.............................A.........I....................H....L...........AH...F........V...........S....R.....K.....  371
Bos taurus                       .....A..................T....................I....................H....L...........AH...F........V......................K.....  372
Erinaceus europaeus              ..........VD.....N.S.........................I...M................R..A.I...........AR.....................L......Y......K.....  373
Loxodonta africana               .....T.....................V.................I.....M..............H....I.......M..SPR..R......................TQ........K.....  372
Trichechus manatus latirostris   .....A.....................VL...L............I....SM..............Y....I.......M...PR..RH........I.............Q..I.....K.....  372
Elephantulus edwardii            .....I....T........................L.........I.L..................H....I.......L..SP...R.......................Q........K.....  372
Chrysochloris asiatica           .....A.......................................I.....R..............H....I..........SPR..R.......................Q..L.....K.....  370
Echinops telfairi                .....A.............................M.........I....................H..Q.I..........SPR............................YL.....KS....  374
Dasypus novemcinctus             .....A.......................................I....................H.R..........I..SPK...................................K.D...  372
Monodelphis domestica            .....S.......................................I..............V.....H..............F.PI...................................E.....  372
Sarcophilus harrisii             .....A......................M................I....................H....I.........F.PI.............................T.....M..Q.D  372
                                 **:.* ** * .:*** :. ****:::: ***: * ***:**:**:*:* . * ******:***** *  *: *:**: ::::. ** .* .:*:**. .**:**:** :::: * *** :::*:
```

```
Pan troglodytes                  EMNGAPMPDIQVYGYLLHTHLAGRALQAVQYRNGTQLRKICKDDSYDFNLQETRDLPSRVEIKPGDELLVECHYQTLDRDSMTFGGPSTINEMCLIFLFYYPRNNISSCMGYPDIIYVAHELGEE  497
Homo sapiens                     ......................................................................................................Q......................  497
Pan paniscus                     .............................................................................................................................  497
Gorilla gorilla gorilla          .........M..................................................Q....................V...........................................  497
Pongo abelii                     ....................F........*........T........................L..G...........C.................................A............  497
Macaca mulatta                   ......V................Q..............T.........................................F.........................................K..  497
Macaca fascicularis              ......V................Q..............T.........................................F............................................  497
Papio anubis                     ......V................Q..............T........................L................F............................................  497
Chlorocebus sabaeus              ....G.V..........................R...QT.........................................F........M...................................  497
Saimiri boliviensis              .I....V...............................T.........................................A............................................  497
Callithrix jacchus               ......V...............................T.........................................A............................................  497
Tarsius syrichta                 ....V.F...............................T........................................................V.............................  497
Microcebus murinus               ....G.V..............................QT..................A..V....................L.........................................Q.  497
Daubentonia madagascariensis     ....G.V..............................QT.....................V................................................................  497
Otolemur garnettii               ......V..............................QT..................A..A..................EAL.....................................Q.....  497
Tupaia chinensis                 ......V.............................IQT.....................A...................T............................................  497
Oryctolagus cuniculus            ......V...............................T..................A...............................V...................H......F........  497
Mus musculus                     .L....VS..YISAC.........S...L........QVV...F.......S....HP.V........I..........F.....A..........F.....I..............TN.....  497
Rattus norvegicus                ......VA..Y.SAC.........S...L........QIV...F.......S....HP.V........I..........F.....A..........F.....I..............TN.....  497
Cricetulus griseus               ......V...Y..S..I.......SQ..........VQI....Y.............HPAV........I..N.............A........V.F.....I.....L........TN.....  497
Dipodomys ordii                  ......VN.MI.AS............R.......K...T.........S........HL.D...........N........T....I......................L.F.......N.....  497
Heterocephalus glaber            ......VA.MK.I.................F...R...T.....A............HH..........I..N.............T..L.....V.............L.....GH.VQ....A  497
Ictidomys tridecemlineatus       ....T.VA....F..........Q..............QT..................H..V........I..R........VI......L.....V.............Q......S..R.....  497
Pteropus alecto                  ......V..............T................T....A.............G.......................L...........................................  497
Eptesicus fuscus                 ......V...............................T..................H...........I....E.....FL..................F..................Q.....  497
Myotis lucifugus                 ......V...............................T.....T............H...........I..........FL.............V.............................  497
Myotis brandtii                  ......V...............................T.....T............H...........I...........L.............V.............................  497
Myotis davidii                   ......V...............................T.....T............H...........I...........L.............V.F...........................  497
Felis catus                      ....V.................................I.....F............Y.M...Q.................L..................................H........  497
Canis lupus familiaris           ......V..............................QT..................Y.......................L..................................H........  497
Ailuropoda melanoleuca           ......V...........................A...T..................Y.A.....................L..................................H..Q.....  497
Leptonychotes weddellii          ......V...........................A...T.........S........Y.A...L.................L...........................................  497
Odobenus rosmarus divergens      ......V...........................A...T..................Y.A...L.................L...........................................  497
Mustela putorius furo            ..................................A...T..................Y.A.....................L..................................H........  497
Equus caballus                   ......V.....F.......................F.T..................HL......................L.......V.....V.........................M...  497
Ceratotherium simum simum        ...R..V...............................T..................H.......................L.............................F.......Q.....  497
Camelus ferus                    ......V...............................T..................Y......................FL..................................F....M...  497
Sus scrofa                       ......VL.............................QT..................Y.MV....................FL...........................................  497
Capra hircus                     ...R..V.....F.........................V.....A............Y..V...........R........L.............V.........V...Q...............  496
Ovis aries                       ...R..V.....F.........................V.....A............Y..V...........R........L.............V.........V...Q...............  496
Pantholops hodgsonii             ...R..V.....F.........................V..Q..A............Y..V...........R........L.............V.........V...H...............  496
Bos taurus                       ......V.....F............I............V.....A............Y..V...........R........L...............F.......V...Q...............  497
Erinaceus europaeus              .I....V..............T....K...........T.....F............H...............................T..........................S.......D  498
Loxodonta africana               ....T.V...........................S...T..R.G....S........A..............Q........L...........................................  497
Trichechus manatus latirostris   ......L..............T............S...T..................A......................FL..A....V.........................VS..R..E..  497
Elephantulus edwardii            .I....V...................K.......SE..T..................A.......................L..................................VS........  497
Chrysochloris asiatica           ......V..............T...........S...T.....A............E.L...T........Q.......T..............V.............................  495
Echinops telfairi                .I....V.....F.....................K.IKT..................K..V..A..D.............TL...................................S....M...  499
Dasypus novemcinctus             .I.R..V.................S.............T.....A............Y.......................L...........................................  497
Monodelphis domestica            ....T.V.................S.........K.IKT.....A...........DK..T..M...............TTL..................................H........  497
Sarcophilus harrisii             .I..V.V.................S.......K.K.VGI.....A...........KE.IT..M...............TTL..................................H........  497
                                 *:*  *. *: : . *:***::*.: .:: :*:  :.  :*.*  ***.***:***     ** ** **:**.*:***     *.* ** *****:*:**:*. *:*** *:.**  *..*: :
```

```
Pan troglodytes                  ASD-SMEGMMAMNNVEWTPESIKKAEKVCKEAQQTVIIKTIDEVVENTTGWIPDIIPTLRGPCLESSGGKVEAQDKTSAGFRAA-PVALSGSSRATLRHLPLAAVLLVQRTLSWLLAMLQTGV*  618
Homo sapiens                     ..                                                                                                                           499
Pan paniscus                     ...-.......................A.............................P........................-.................................*  618
Gorilla gorilla gorilla          ...-Y......................A...............L.............P........................-.............L...............P.......*  618
Pongo abelii                     ...-P.....D..D.K..L........A............................AP.................PT.....-.....S...........V.I..L.H..F.......N..*  618
Macaca mulatta                   ...-....I..................A.............................P.......L......H...P....PS-Q-..A.PP*                              587
Macaca fascicularis              ...-....I..................A.............................P.......L......H...P....PS-Q-..A.PP*                              587
Papio anubis                     ...-....I..................A.............................P.......L.........P....PS-Q-..A.PP*                              587
Chlorocebus sabaeus              ...-....I..................A.............................P.......L.........P....PS-Q-..A.PP*                              587
Saimiri boliviensis              ...-....I..................A..........................S.APQ.................P.....S-.....S..K......L........S.......T.....*  618
Callithrix jacchus               ...-.......................A.............................PL..................P....SS-.....S..K....R..........S...G.......R.*  618
Tarsius syrichta                 ...-....I..I...............A......M...................T.PP.............P....P......-.M......I...KR....TF....G..........P.N*  618
Microcebus murinus               V..-................N......A.R..R...L..................T..P.............P....P......-.....S.GP.A..R.....H....GA.....GR.H..G*  618
Daubentonia madagascariensis     ...-.........S..............A....R.......................P.............P....P......-.........T.S.KR.....H....G.......T....G*  618
Otolemur garnettii               V..-.......................RA....R.......................P.............P....P...K..-.........TVS..R...V.H....GI.........R..G*  618
Tupaia chinensis                 ...-................N......A...............M.............P.............P....P......-....R..GT.P.QC..V.VL.F..GA...I..T....A*  618
Oryctolagus cuniculus            V..-P...............N......A........L..............N.T..P.............P...KP......-.M..PS..AT...C...T.L.F..GA..R..GT...RG*  618
Mus musculus                     ..ENP..NL.VLD.......N......A...S....L......E.............P.......T.....P..N.P.....V-.L.....NT....P..MI...FL.GS..C.........*  619
Rattus norvegicus                ..ENP..NL.VL........N..T...A...S....L......E.............P..A..........P....P.....V-.M.....NT.......MIT..FL.GC............*  619
Cricetulus griseus               ..ENP..NL.VL........N......A...S....L......E.............P.............PG...P......-.MV....NT.....V..VTI.FL.G.V....P......*  619
Dipodomys ordii                  ..ENP..NLIVLE.ID....N.R....A....R...M....N.LI..........T.AP.............P...NP......-....PH.NM..PAAF....L.WLHGA.L...VI.*     615
Heterocephalus glaber            .AESP..NL.V.SS......N..A...A....R...ML...N.LI.............P.............P..Q.P......-..V....NT....SI....L.FL.G.IP.........I*  619
Ictidomys tridecemlineatus       V..-P...T.V.SSI...........RA...S..I.V......L..............P.............P..H.P......-.........T.A.N......L.FM.G..........PE.*  618
Pteropus alecto                  V..-.......I...............A....E.M...............L..E.T..P....V........P..ESP...K.S-.M...DAGT....C.....L.FG.GA.......T...RA*  618
Eptesicus fuscus                 ...-..............L.N......A...S.............G.......E....P.............P..E.P......-.M..LA.GN..P....M..L.FG.GAFF....T..A.I*  618
Myotis lucifugus                 ...-..............L.N......A........L................E....P.............P..E.P......-.M..LA.GN.......M..L.FG.GAFF....T..A.I*  618
Myotis brandtii                  ...-..............L.N......A........L................E....P.............P..E.P......-.M..LA.GN.......M..L.FG.GAFF....T..A.I*  618
Myotis davidii                   ...-..............L.N......A........L........G.......E....P.............P..E.P......-.M..LA.GN.......M..L.FG.GAFF....T..A.I*  618
Felis catus                      V..-.....I..........N......A.........................E..RAP.............P....P......-.I......T....C.....L.FG.GA.......T..V..*  618
Canis lupus familiaris           V..-..........I.....N......A..G...M..................E....T.............P....P......-..G..S..T....C.....L.FG.GAF.....T.KVV.*  618
Ailuropoda melanoleuca           V..-................N......A......M.......L..........E...AT.............P..Q.P......-.........T....C.....L.FG.GV......T.RV..*  618
Leptonychotes weddellii          V..-P...............N......A......M.......L..........E....A.............P....P...K.E-.........T.S.......L.FG.GA......N..V..*  618
Odobenus rosmarus divergens      V..-P......I........N......A......M.......L..........E....A.............P....P......-.........T.S......V.L.FG.GA......T..V..*  618
Mustela putorius furo            V..-P...............N......A......M.......L...............T.............P....P......-.........T..Q.R.S...L.FG.GA......T.KV..*  618
Equus caballus                   ...-P...............N......A......M.......I........SE....P............AP...IP......-.M.....ET..P.......LFFG.GA......T..V..*  618
Ceratotherium simum simum        ...-...A...................A..............I........SE..RAP.............P...IPE.....-.M.....NT..W.R.....L.FG.GV.FL..TT..A..*  618
Camelus ferus                    ...-................N......A..............I.......V.E.T..P....S.........P...AP....T.-.........T..P.R.....L..G.GAV.....T..SR.*  618
Sus scrofa                       ...-.......................A..............L.........E.V..P.............PE.Q.P......-.M...V..A..PSR.....F..G.G.......T..A..*  618
Capra hircus                     V..-.........S............NA..............L........Q..N..P.............P....P......-.RV.....S....C....TL.FG.GA......T..A.I*  617
Ovis aries                       V..-.........S............NA...G..........L........R..N..P.............S....P......-.GV.....S....C....TL.FG.GA......T..A.I*  617
Pantholops hodgsonii             ...-.........S............NA..............L........R..S..P.............P....P......-.RV.....S....C....TL.FG.GA......T..A.I*  617
Bos taurus                       V..-.........S..........V..A..............L........Q..N..P.............P....P......-.IV..RA.S....C.....L.FG.GA......T..S.I*  619
Erinaceus europaeus              .M.-P.DAV..L..ID....N..M...A..............F........Q..T..P..V....I.....PL..IP....S.-.A..R..GMIPPQ.FS..SL.FA.GA.Y....T..A.I*  619
Loxodonta africana               ...-................N......A..............................P.......T.....PH...P.....K-.LS...A.T.SPGS....TL.FT.G...LF..T..ARD*  618
Trichechus manatus latirostris   V..-...A............N......A....E............G........V.IP.......T.....PH..NPV....I-.L....A.M.AP.I...VTL.FA.G..L....S..SRD*  618
Elephantulus edwardii            ...-................N......A..........................A..S.......T.....P....P......T..T...T.TIAP.T....TL.IT.GA......L*     614
Chrysochloris asiatica           ...-.......................A.........................R..L..P.......T.....P....P.....S-.LV..S..TTAPKG..PVTL.FT.WA.....IT..ARD*  616
Echinops telfairi                ...-P.........I.....N......A......I.V............Q....V..P.......T..........P......-.LT..SA.T.SSGGFS..TL.FA.GA....F.S..ARD*  620
Dasypus novemcinctus             V..-................N......A..............................P.............P...NP......-.L..LD..T....C.....L.FG.GA......S..A..*  618
Monodelphis domestica            .T.-..........I..N..I......A....D.V.V......M.M.Q..H.......P.......I.....P....P......-.PIQ..C.S.----FS.TIL..L.G.F....IS..SAI*  614
Sarcophilus harrisii             ...-...........D....N......A..M.D.V.........L.K.Q..H.......P.......T.....PG...P..Y...-.LVQ....PT----V..TIL..L.GVF....DS..GA.*  614
                                 .
```
