# Supplementary material for: Frequent Loss and Alteration of the MOXD2 Gene in Catarrhines and Whales: A Possible Connection with the Evolution of Olfaction
Source: PLoS One. 2014 Aug 7;9(8):e104085. doi: 10.1371/journal.pone.0104085 (PMC4125168; doi:10.1371/journal.pone.0104085)
Supplement: Figure S2 — Sequence comparison of the chimpanzee ( Pan troglodytes ) and the northern white-cheeked gibbon ( Nomascus leucogenys ) MOXD2 gene loci. (A) Dot plot comparison of the chimpanzee (horizontal) and the northern white-cheeked gibbon (vertical) MOXD2 loci. In the northern white-cheeked gibbon, the MOXD2 genomic region was replaced with a translocated DNA segment (red box). The chimpanzee MOXD2 and PRSS58 genes are marked at the top with coding and non-coding regions (introns and untranslated regions) in green and yellow, respectively. A dotted horizontal line indicates a gap in the northern white-cheeked gibbon genome assembly “nomLeu3”. The left and the right alignment boundaries are marked by dotted blue boxes. (B, C) Alignments of chimpanzee and gibbon sequences for the left (B) and right boundaries (C). Identical residues between the two species are marked by vertical lines. The northern white-cheeked gibbon WGS trace data are in blue. The unaligned region is highlighted in red. Note that the boundary regions are supported by multiple trace data, indicating that MOXD2 deletion in the northern white-cheeked gibbon was not a result of erroneous assembly. (PDF) [file pone.0104085.s002.pdf]

Figure S2. Sequence comparison of the chimpanzee (*Pan troglodytes*) and the northern white-cheeked gibbon (*Nomascus leucogenys*) *MOXD2* gene loci. (A) Dot plot comparison of the chimpanzee (horizontal) and the northern white-cheeked gibbon (vertical) *MOXD2* loci. In the northern white-cheeked gibbon, the *MOXD2* genomic region was replaced with a translocated DNA segment (red box). The chimpanzee *MOXD2* and *PRSS58* genes are marked at the top with coding regions in green and non-coding regions (introns and untranslated regions) in yellow. A dotted horizontal line indicates a gap in the northern white-cheeked gibbon genome assembly "nomLeu3." The left and the right alignment boundaries are marked by dotted blue boxes. (B, C) Alignments of chimpanzee and gibbon sequences for the left (B) and right boundaries (C). Identical residues between the two species are marked by vertical lines. The northern white-cheeked gibbon WGS trace data are in blue. The unaligned region is on a red background. Note that the boundary regions are supported by multiple trace data, indicating that *MOXD2* deletion in the northern white-cheeked gibbon was not a result of erroneous assembly.

A. Northern white-cheeked gibbon versus chimpanzee

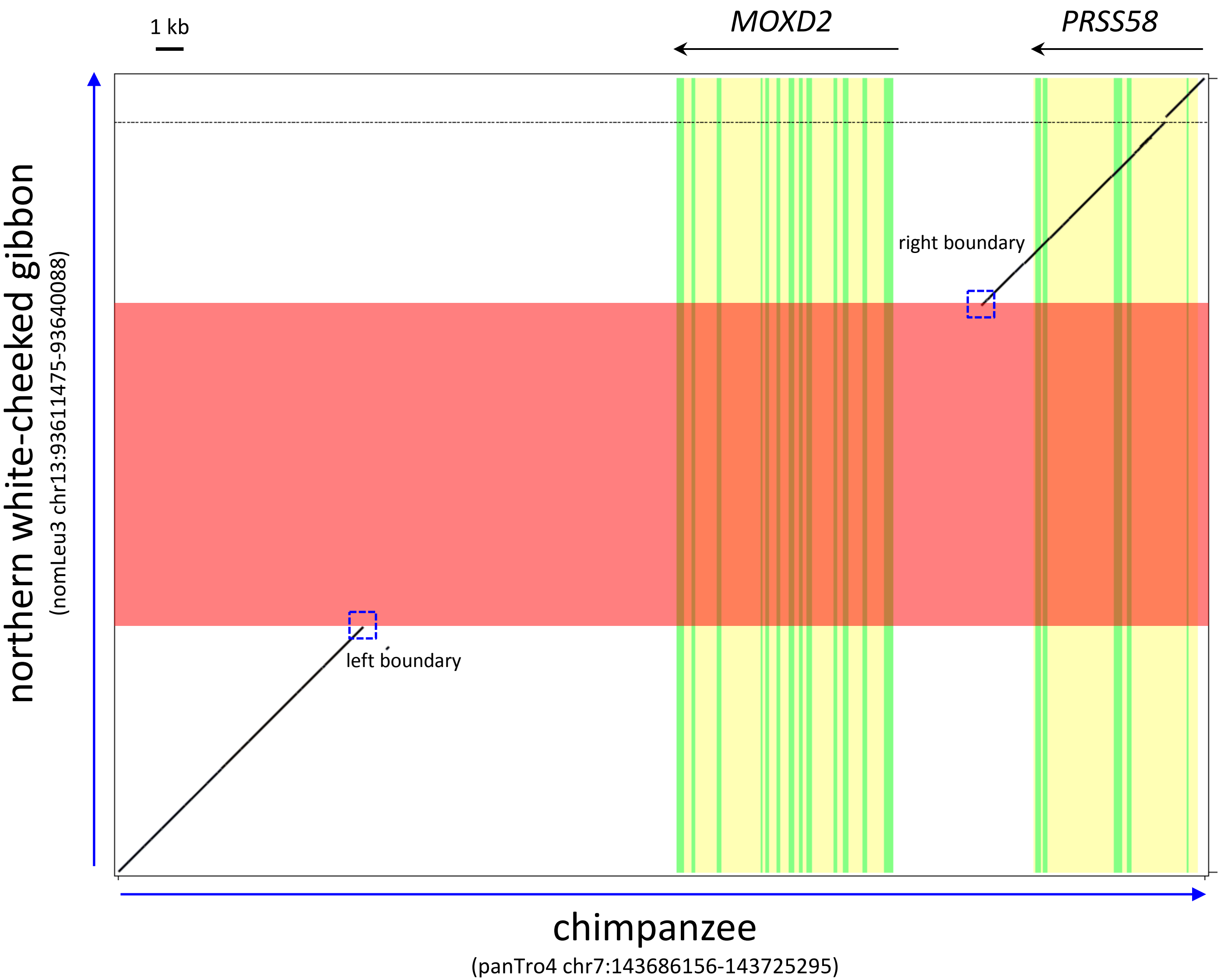

B. Left boundary

```
Pan troglodytes      TTTTCAGTCTGCAAAATGCTGCACAAAGGTTTACTATTATTTATTACTGTTATTCTATAATTCCCTGAGTGTTCTGATGTCTCTTGTGGGTTGTTTTTTAAGTTTTGTTTTTGTTTTTGT
                     ||||||||||| ||||||||||||||||||||||||||||||||||||||||||||||||            |    | |  ||     |        |  |       |      |
Nomascus leucogenys  TTTTCAGTCTGTAAAATGCTGCACAAAGGTTTACTATTATTTATTACTGTTATTCTATAACATATAATTCTATAATCTCTGCCTGACTAGCACAGCAATTTGACCATCCTGGTGGGTGCA
2116797184+          TTTTCAGTCTGTAAAATGCTGCACAAAGGTTTACTATTATTTATTACTGTTATTCTATAACATATAATTCTATAATCTCTGCCTGACTAGCACAGCAATTTGACCATCCTGGTGGGTGCA
2132212808+          TTTTCAGTCTGTAAAATGCTGCACAAAGGTTTACTATTATTTATTACTGTTATTCTATAACATATAATTCTATAATCTCTGCCTGACTAGCACAGCAATTTGACCATCCTGGTGGGTGCA
1856024305-          TTTTCAGTCTGTAAAATGCTGCACAAAGGTTTACTATTATTTATTACTGTTATTCTATAACATATAATTCTATAATCTCTGCCTGACTAGCACAGCAATTTGACCATCCTGGTGGGTGCA
1880182019-          TTTTCAGTCTGTAAAATGTTGCACAAAGGTTTACTATTATTTATTACTGTTATTCTATAACATATAATTCTATAATCTCTGCGTGACTAGCACAGCAATTTGACCATCCTGGTGGGTGCA
1891455408+                                  AAAGGTTTACTATTATTTATTACTGTTATTCTATAACATATAATTCTATAATCTCTGCCTGACTAGCACAGCAATTTGACCATCCTGGTGGGTGCA
```

C. Right boundary

```
Pan troglodytes      GGGTTTTACCATATTGATCAGGCTGTTCTCAAACTCCTTACCTTAGGTGATCCACTCACATCGGCCTCCCAAAGTGCTGGGATTACAGGCGTGAGCCACTGCACCTGGCCTCATTTTTTT
                        |  |||      | |    | ||  ||    ||          ||||||  |   | ||||||||||||||||||||||||||||||||||||||| ||||||||||||||||||||
Nomascus leucogenys  TTTTAGTACAGACGGGGTTTCACCGTGGTCTTGATCTCCTGACCTCGTGATCTGCCTGCCTCGGCCTCCCAAAGTGCTGGGATTACAGGCGTGAGCCACCGCACCTGGCCTCATTTTTTT
2111935942-          TTTTAGTACAGACGGGGTTTCACCGTGGTCTTGATCTCCTGACCTCGTGATCTGCCTGCCTCGGCCTCCCAAAGTGCTGGGATTACAGGCGTGAGCCACCGCACCTGGCCTCATTTTTTT
1910817237-          TTTTAGTACAGACGGGGTTTCACCGTGGTCTTGATCTCCTGACCTCGTGATCTGCCTGCCTCGGCCTCCCAAAGTGCTGGGATTACAGGCGTGAGCCACCGCACCTGGCCTCATTTTTTT
1855600873+          TTTTAGTACAGACGGGGTTTCACCGTGGTCTTGATCTCCTGACCTCGTGATCTGCCTGCCTCGGCCTCCCAAAGTGCTGGGATTACAGGCGTGAGCCACCGCACCTGGCCTCATTTTTTT
1880930328+          TTTTAATACAGACGGGGTTTCACCGTGGTCTTGATCTCCTGACCTCGTGATCTGCCTGCCTCGGCCTCCCAAAGTGCTGGGATTACAGGCGTGAGCCACCGCACCAGGCCTCATTTTTTT
1887967223-          TTTTAGTACAGACGGGGTTTCACCGTGGTCTTGATCTCCTGACCTCGTGATCTGCCTGCCTCGGCCTCCCAAAGTGCTGGGATTACAGGCGTGAGCCACCGCACCTGGCCTCATTTTTTT
1902026262-          TTTTAGTACAGACGGGGTTTCACCGTGGTCTTGATCTCCTGACCTCGTGATCTGCCTGCCTCGGCCTCCCAAAGTGCTGGGATTACAGGCGTGAGCCACCGCACCAGGCCTCATTTTTTT
1903759274+          TTTTAGTACAGACGGGGTTTCACCGTGGTCTTGATCTCCTGACCTCGTGATCTGCCTGCCTCGGCCTCCCAAAGTGCTGGGATTACAGGCGTGAGCCACCGCACCTGGCCTCATTTTTTT
2038866918-          TTTTAGTACAGACGGGGTTTCACCGTGGTCTTGATCTCCTGACCTCGTGATCTGCCTGCCTCGGCCTCCCAAAGTGCTGGGATTACAGGCGTGAGCCACCGCACCAGGCCTCATTTTTTT
2129080945-          TTTTAGTACAGACGGGGTTTCACCGTGGTCTTGATCTCCTGACCTCGTGATCTGCCTTCCTCGGCCTCCCAAAGTGCTGGGATTACAGGCGTGAGCCACCGCACCAGGCCTCATTTTTTT
```
