# Supplementary material for: Frequent Loss and Alteration of the MOXD2 Gene in Catarrhines and Whales: A Possible Connection with the Evolution of Olfaction
Source: PLoS One. 2014 Aug 7;9(8):e104085. doi: 10.1371/journal.pone.0104085 (PMC4125168; doi:10.1371/journal.pone.0104085)
Supplement: Figure S3 — Disruptive mutations in the MOXD2 genes of whales. Exon sequences of the pig (Sus scrofa), the cow (Bos taurus), the killer whale (Orcinus orca), the bottlenose dolphin (Tursiops truncatus), the finless porpoise (Neophocaena phocaenoides), the baiji (Lipotes vexillifer), the sperm whale (Physeter macrocephalus), the minke whale (Balaenoptera acutorostrata), and the fin whale (Balaenoptera physalus) MOXD2 genes are shown. Disruptive mutations are highlighted in red. Ancestral stop codons are highlighted in yellow. Coding and noncoding sequences are in uppercase and lowercase letters, respectively. (PDF) [file pone.0104085.s003.pdf]

Figure S3. Disruptive mutations in the *MOXD2* genes of whales. Exon sequences of the pig (*Sus scrofa*), the cow (*Bos taurus*), the killer whale (*Orcinus orca*), the bottlenose dolphin (*Tursiops truncatus*), the finless porpoise (*Neophocaena phocaenoides*), the baiji (*Lipotes vexillifer*), the sperm whale (*Physeter macrocephalus*), the minke whale (*Balaenoptera acutorostrata*), and the fin whale (*Balaenoptera physalus*) *MOXD2* genes are shown. Disruptive mutations are highlighted on a red background. Ancestral stop codons are highlighted on a yellow background. Coding and noncoding sequences are in uppercase and lowercase letters, respectively.

A. exon 1

```
Sus scrofa                  gagccATGGCCCACGCCCTTCTCCTCCAGCTTCTTCTGCTTTCAGCCCTGGCAGCCCCCTCCCAAGGCAACCGCCTCGGCCCCACCTCGAGTCTGCGTTATTCTAGGTTC  110
Bos taurus                  aagccATGGCCTGTGCCCTTCTCTCCTGGCTTCTCCTATTTACGGCCCTTGCAACTCCCTCCCAATGCAATCGCCTTGGCCCCATGCCGCGTCTGCGTTATTCCAGGTTT  110
Orcinus orca                gagccATGGCCTGTGCCCTTCTCTTTCGGCTTCTCCTACTTATGGCCCTGCTGACCTCCTCTCAAGGCAACCACCTGGGCCTCACATTATGTCTGCATTATTCCATGTTC  110
Tursiops truncatus          gagccATGGCCTGTGCCCTTCTCTTTCGGCTTCTCCTACTTACGGCCCTGCCGACCTCCTCTCAAGGCAACCACCTGGGCCTCACATCACGTCTGCGTTATTCCATGTTC  110
Neophocaena phocaenoides    gagccATGGCCTGTGCCCTTCTCTTTCGGCTTCTCCTACTTATGGCCCTGCTGACCTCCTCTCAAGGCAACCACCTGGGCCTCACATCACGTCTGCGTTATTCCATGTTC  110
Lipotes vexillifer          gagccGTGGCCTGTGCCCTTATCTTTCGGCTTCTCCTACTTACGGCCCTGCCGACCCCCTCTCAAGGCAACCACCTGGGCCTCACATCATGTCTGCGTTATTCCACATTC  110
Balaenoptera acutorostrata  gagccATGGCCTGTGCCCTTCTCTTTTGGCTTCTCCTACTTACGGCCCTGCCGACCTCCTGTCGAGGCAACCACCTGGGCCCCACATCGCGTCTGCGTTATTCCACGTTC  110
Balaenoptera physalus       gagccATGGCCTGTGCCCTTCTCTTTCGGCTTCTTCTACTTACGGCCCTGCCGACCTCCTCTCAAGGCAACCACTTGGGCCCCACATCGCGTCTGCGTTATTCCACATTC  110

Sus scrofa                  CTAGATCCTTCTAATGTCATTTTCCTGCGCTGGGACTTTGACCTGGACGCTGAGATCATCGCTTTTGAGCTCCGGGTCCGGACAGCTGGCTGGGTGGGCTTGGGCATCAC  220
Bos taurus                  CTAGACCCTTCTAACGCTGTTTTCCTGCGCTGGGACTTTGACTTTGAGGCTGAGATCATCACTTTCGAGCTCCAGGTCCGGACAGCTGGCTGGGTGGGTTTGGGTGTCAC  220
Orcinus orca                CTAGATCCTTCTAATGTTACTTTCCTGCACTGGGACTTTGACCTTGAGGCTGAGATCGTCACTTTTGATCTCCAGGTCCAGACAGCTGGCTGGGTGGGCTTGGGTATCAC  220
Tursiops truncatus          CTAGATCCTTCTAATGTTACTTTCCTGCACTGGGACTTTGACCTTGAGGCTGAGGTCGTCACTTTTGAGCTCCAGGTCCAGACAGCTGGCTGGGTGGGCTTGGGTATCAC  220
Neophocaena phocaenoides    CTAGATCCTTCTAATGTTATTTTCCTGCACTGGGACTTTGACCTTGAGGCTGAGATCATCACTTTTGAGCTCCAGGTCCAGACAGCTGGCTGGGTGGGCTTGGGTATCAC  220
Lipotes vexillifer          CTAGATCCTTCTAATGTCATTTTCCTGCACTGGGACTTTGACCTTGAGGCTGAGATCATCACTTTTGAGCTCCAGGTCCAGACAGCTGGCTGGGTGGGCTTGGATATCAC  220
Balaenoptera acutorostrata  CTAGATCCTTCTAATGTCATTTTCCTGCACTGGGACTTTGACCTTGAGGCTGAGATCATCACTTTTGAGCTCCAGGTCCGGACAGCTGGCTGGGTGGGCTTGGGTATCAC  220
Balaenoptera physalus       CTAGATCCTTCTAATGTCATTTTCCTGCACTGAGACTTTGACCTTGAGGCTGAGATCATCACTTTTGAGCTCCAGGTCCGGACAGCTGGCTGGGTGGGCTTGGGTATCAC  220

Sus scrofa                  CAATCGCTACACCAGAGCAGGCAGCGATCTGGTTGTCGGAGGGGTCTCGCCTGACGGTCATGTCTATTTCTCGgtgag  298
Bos taurus                  AGATCGCTACAGCAGAGCGGGAAGCGACCTGGTTGTCGGAGGCGTCTCGCCTGACGGCAATGTCTATTTCTCGgtgag  298
Orcinus orca                AAATCGCTACACCATGGTGGGAAGCAATCTGGTTGTTGGAGGAGTCTCGCCGGATGGCAATGTCTATTTCTCGgtgag  298
Tursiops truncatus          AAATAGCTACACCATGGTGGGAAGCAATCTGGTTGTTGGAGGAGTCTCGCCGGACGGCAATGTCTATTTCTCGgtgag  298
Neophocaena phocaenoides    AAATCGCTACACCATGGTGGGAAGCAATCTGGTTGTTGGAGGAGTCTCGCCAGACGGCAATGTCTATTTCTCGgtgag  298
Lipotes vexillifer          AAATCGCTACACCAGAGTGGGAAGCAATCTGGTTATTGGAGGAGTCTCGCCGGACGGCAATGTCTATTTCCCGgtgag  298
Balaenoptera acutorostrata  AAATCGCTACACCAGAGTGGGAAGCGATCTGGTTGTTGGAGGAGTCTCGCCGGACGGCAATGTCTATTTCTCGgtgag  298
Balaenoptera physalus       AAATCGCTACACCAGAGTGGGAAGCGATCTGGTTGTTGGAGGAGTCTCGCCGGACGGCAATGTCTATTTCTCGgtgac  298
```

B. exon 2

```
Sus scrofa                 cccagGATCAGCATGTGGTGGATGAAGACACCCTGGAAGAGGACGGGAGCCAGGACGCGGAGCTGCAGGGGCTGACGGAAGACGCCGTCTACACCACCATGCGCTTCTCC  110
Bos taurus                 cccagGACCAGCACCTGGTGGATGAAAACACGCTGGAGGAGGATGGGAGCCAGGATGCAGAACTGCAGGGGCTAACAGAAGATGCCGTCTACACCACCATGCGCTTCTCC  110
Orcinus orca               cctagAATCAGCACCTGGTGGATGAAGACGCTCTGGAGGAGG----GAGCCAGAACGCAGAGCTGCAGGCACTGACAGAAGACACCATCTATACCACCATGCGCTTCTCC  106
Tursiops truncatus         cctagAATCAGCACCTGGTGGATGAAGACGCTCTGGAGGAGG----GAGCCAGGACGCAGAGCTGCAGGCACTGACAGAAGACACCATCTATACCACCATGTGCTTCTCC  106
Neophocaena phocaenoides   cctagAATCAGCACCTGGTGGATGAAGACGCTCTGGGGGAGG----GAGCCAGGACGCAGAGCTGCTGGCACTGACAGAAGACACCATCTATACCACCATGCGCTCCTCC  106
Lipotes vexillifer         cctagAATCAGCACCTGGTGGATGAAGACGCTCTGGAGGAGG----GAGCCAGGATGCGGAGCTGCAGGCACTGACAGAAGACACCATCTATACCACCATGCGCTTCTCC  106
Balaenoptera acutorostrata cccagGATGAGCACCTGGTAGACGAAGACGCTCTGGAGGAGGACGAGAGCCAGGACGCGGAGCTGCTGGCACTGACAGAAGATGCCATCTATACCACGATGCGCTTCTCC  110
Balaenoptera physalus      cccagGATGAGCACCTGGTAGACGAAGATGCTCTGGAGGAGGACGGGAGCCAGGACGCGGAGCTGCTGGCACTGACAGAAAACGCCATCTATACCACCATGCACTTCTCC  110

Sus scrofa                 AGGCCCTTCCGCTCCTGCGACCCCCAGGACCAAGACATCACGgtaaa  157
Bos taurus                 AGGCCCTTCCGCTCCTGCGACCCTCACGACCAAGACATCACGgtaaa  157
Orcinus orca               AGGCCCTTCTGCTCCTGTGACCCTCAAGACCAAGACATCACGgtaaa  153
Tursiops truncatus         AGGCCCTTCTGCTCCTGTGACCCTCAAGACCAAGACATCACGgtaaa  153
Neophocaena phocaenoides   AGGCCCTTCCGCTCCTGCGACCCTCAAGACCAAGACATCACGgtaaa  153
Lipotes vexillifer         AGGCCCTTCCACTCCTGTGACCCTCAAGACCAAGACATCACGgtaaa  153
Balaenoptera acutorostrata AGGCCCTTCCGCTCCTGTGACCCTCATGACTAAGACATCACGgtaaa  157
Balaenoptera physalus      AGGCCCTTCCGCTCCTGTGACCCTCATGACTAAGACATCACGgtaaa  157
```

C. exon 3

```
Sus scrofa                 cccagAGTGACACAGTGAGGGTGCTGGCTGCCTATGGCCCTGATGACACCCTGAAGCTGGATCGGGAGCGGATGTTCGTGAAGTCCATCTTCCTGCTCCAGATCATCCAC  110
Bos taurus                 tacagAGCGACACTGTGAGGGTACTGGCTGCCTACGGCCTGGATGACACCCCAAAGATGGATCGGGAACGTACTTTTGTGAAGTCCATCTTCCTGCTCCAAATCGTCCAC  110
Orcinus orca               cccagAGTGACACTGTGAGGGTGCTTGCCACCTACGGCCCAGATGACACTCTGAAGCTGGATCGGGAGCGTACTTTTGTG-AGTCCATCTTCCTGCTCCAAATTGTCCAC  109
Tursiops truncatus         cccagAGTGACACTGTGAGGGTGCTTGCCACCTACGGCCCAGATGACACTCTGAAGCTGGATCGGGAGCGTACTTTTGTG-AGTCCAT-------CTACAAATTGTCCAC  102
Neophocaena phocaenoides   cccagAGTGACACTGTGAGGGTGCTTGCCACCTACGGCCCAGATGACACTCTGAAGCTGGATCGGGAGCGTACTTTTGTG-AGTCCATCTTCCCGCTCCACATTGTCCAC  109
Lipotes vexillifer         ccccgAGTGACACTGTGAGGGTGCTTGCCACTTACGGCCCAGATGACACTCTGAAGCTGGATTGGGAGCGTACTTTTGTG-AGTCCATCTTCCTGCTCCAAACTGTCCAC  109
Balaenoptera acutorostrata ctcagAGTGACACTGTGAGGGTGCTTGCCGCCTACGGCCCAGATGCCACTCTGAAGTTGGATCAGGAGCGTACTTTTGTGAAGTCCATCTTCCTGCTCCAAATTGTCCAC  110
Balaenoptera physalus      cccagAGTGACACTGTGAGGGTGCTTGCTGCCTATGGCCCAGATGCCACTCTGAAGCTGGATTGGGAGTGTACTTTTGTGAAGTCCATCTTCCTGCTCCAAATTGTCCAC  110

Sus scrofa                 CCCGACGATCTCGACGCCCCCGAGGACGCCATCATCCACGACCTGGAGATCACGGATgtaag  172
Bos taurus                 CCTGATGATCTCGATGCCCCTGAGGACACCATCATCCATGACTTGGAGATCACTGATgtaag  172
Orcinus orca               CCCGATGATCTCGATGTCCCCGAGGACACCATCATCCATGACTTGGAGATCACTGATgaagg  171
Tursiops truncatus         CCCGATGATCTTGATGTCCCCGAGGACACCATCATCCATGACTTGGAGATCACTGATgtaag  164
Neophocaena phocaenoides   CCCGATGATCTCGATGTCCCCGAGGACACCATCGTCCATGACTTGGAGATCACTGATgtgag  171
Lipotes vexillifer         CCTGATGATCTCGATGTCCCCGAGGACACCATCATCCATGACTTGGAGATCACTGATgtaag  171
Balaenoptera acutorostrata CCCAATGATCTCGATGTCCCCGAGGACACCATCATCCATGACTTGGAGATCACTGATgtaag  172
Balaenoptera physalus      CCCGATGATCTCGATGTCCCCGAGGACACCATCATCCATGACTTGGAGATCACTGATgtaag  172
```

D. exon 4

```
Sus scrofa                  tccagTTCCTCATTCCAGAGGATGACACCACCTATGCCTGCACCTTCCTCCCTCTCCCCATCGTTAGCAAGAAGCATCACATCTACAAGgtacc  94
Bos taurus                  tctagTTCCTCATCCCAGAGGATGACACCACGTACGCCTGCACCTTCCTCCCTCTCCCCATCGTTAGCAAGAAGCACCACATCTACAAGgtacc  94
Orcinus orca                tccagTTCTTCATCCCAGAGGATGACACCACGTACGCCTGCACCTTCCTCCCTCTCTCCATCGTTAGCAAGAAGCACCGTATCTACAAGgtacc  94
Tursiops truncatus          tccagTTCTTCATCCCAGAGGATGACACCACGTACGCCTGCACCTTCCTCCCTCTCTCCATCGTTAGCAAGAAGCACCGTATCTACAAGgtacc  94
Neophocaena phocaenoides    tccggTTCTTCATCCCAGAGGATGACACCACGTACGCCTGCCCCTTCCTCCCTCTCTCCATCGTTGGCAAGAAGCACCGTATCTCCAAGgtacc  94
Lipotes vexillifer          tccagTTCCTCTTTCCAGAGGATGACACCACGTACGCCTGCACCTTCCTCCCTCTCTCCATCGTTAGCAAGAAGCACCATATCTACAAGgtacc  94
Balaenoptera acutorostrata  tccagTTCCTCATCCCAGAGGATGACACCACGTATGCCTGCACCTTCCTCCCTCTCCCCATCGTTAGCAAGAAGCACCATATCTACAAGgtacc  94
Balaenoptera physalus       tccagTTCCTCATCCCAGAGGATGACACCACGTATGCCTGCACCTTCCTCCCTCTCCCCATCATTAGCAAGAAGCACCATATCTACAAGgtacc  94
```

E. exon 5

```
Sus scrofa                  cccagTTTGAGCCCAAACTGGAACCTCACAACGAGACCATGGTGCACCACATCCTGGTATA-CGCCTGCGGCAACGCCAGCGCCCTCCCCACGGGCATAAGCGACTGCTA  109
Bos taurus                  cccagTTCGAGCCCAAGTTGGTCCAACACAAGGAGACGATTGTGCACCACATCCTGGTGTA-CGCCTGTGGCAATGCCAGCGCTCTCCCCACGGGCATCAGCGACTGCTA  109
Orcinus orca                ccctgTTTGAGCCCAAGTTGGTCCATCACAAGGAGACGATGGTGCACCACATCCTGGTGTACCGCCTGCAGCAAAACCAGTGCTCTGCCCAAGGGCATCAGCGACTGCTA  110
Tursiops truncatus          cccagTTTGAGCCCAAGTTGGTCCATCACAAGGAGACGACGGTGCACCACATCCTGGTGTACCGCCTGCGGCAACACCAGTGCTCTGCCCAAGGGCATCAGCGACTGCTA  110
Neophocaena phocaenoides    cccagTTTGAGCCCAAGTTGGTCCATCACAAGGGGACGACGGTGCACCACATCCTGGTGTACCGCCTGGGGCAACACCAGTGCTCTGCCCAAGGGCATCAGCGACTGCTA  110
Lipotes vexillifer          cccagTTTGAGCCCAAGTTGGTCCATCACAAGGAGACGATGGTGCACCACATCCTGGTGTACCGCCTGTGGCAACGCCAGTGCTCTGCCCAAGGGCATCAGAGACTGCTA  110
Balaenoptera acutorostrata  cccagTTTGAGCCCAAGTTGGTCCATCACAAGGAGACGATGGTGCACCACATCGTGGGGTA-CGCCTGCGGCAAGGCCAGTGCTCTCCCCAAGGGCATCAGCGACTGCTA  109
Balaenoptera physalus       cccagTTTGAGCCCAAGTTGGTCCATCACAAGGAGACGATGGTGCACCACATCGTGGGGTA-CGCCTGCAACAAGGCCAGTGCTCTCCCCAAGGGCATCAGCGACTGCTA  109

Sus scrofa                  CGGGGCCGACCCTGCCTTCTCCCTCTGCTCCCAGGTCATCGTGGGCTGGGCTGTCGGGGGCACAgtgag  178
Bos taurus                  TGGGGCCGACCCCGCCTTCTCCCTCTGCACACAGGTCATCGTGGGCTGGGCTGTCGGGGGCACAgtgag  178
Orcinus orca                CGGGGCTGACCCCGCCTCCTCCCTCTGCTCACAGGTCATCGTGGGCTGGGCTATCGGGGACACAgtgag  179
Tursiops truncatus          CGGGGCCGACCCCGCCTCCTCCCTCTGCTCACAGGTCATCGTGGGCTGGGCTGTCGGGGACACAgtgag  179
Neophocaena phocaenoides    CGGGGCTGACCCCGCCTCCTCCCTCTGCTCACAGGTCATCGTGGGCTGGGCTGTCGGGGACACAgtgag  179
Lipotes vexillifer          CGGGGCCAACCCCGCCTCCTCCCTCTGCTCGCAGGTCATTGTGGGCTGGCCTGTCAGGGGCACAgtgag  179
Balaenoptera acutorostrata  TGGGGCCCACCCCGCCTTCTCCCTCTGCTCGCAGGTCATCGTGGGCTGGGCTGTCGGGGGCACAgtgag  178
Balaenoptera physalus       CGGGGCCCACCCCGCCTTCTCCCTCTGCTCGCAGGTCATCGTGTGCTGGGCTGTCGGGGGCACAgtgag  178
```

F. exon 6

```
Sus scrofa                  accagAGTTACCAGTTTCCGGATGACGTGGGCATCTCTATTGGGACGCCCCTGGACCCCCAGTGGATCCGACTGGAGATTCATTATAGCAATTTTCACAATCTTCCCGgt  110
Bos taurus                  accagAGTTACCAGTTTCCAGATGACGTGGGCATCTCTATTGGGACGCCCTTGGACCCCCAGTGGATCCGCCTGGAGATTCATTACAGCAATTTTCACAATCTGCCTGgt  110
Orcinus orca                aacagAGTTACCAGTTTCCAGGTGACGTGGGTATCTCTACTGGGATGGCTTTGGACCCCCAGTGGGTCCCACTGGAGATTCACTACAGCAATTTGCACAGTCTTCCTGgt  110
Tursiops truncatus          aacagAGTTACCAGTTTCCAGGTGACGTGGGTATCTCTACTGGGATGGCTTTGGACCCCCAGTGGGTCCGACTGGAGATTCACTACAGCAGTTTGCACAGTCTTCCTGgt  110
Neophocaena phocaenoides    aac--AGTTACCAGTTTCCAGGTGACGTGGGTATCTCTACTGGGATGGCTTTGGACCCCCAGTGGGTCCGACTGGAGATTCACTACAGCAATTTGCACAGTCTTCCTGgt  110
Lipotes vexillifer          aacagAGTTACCAGTTTCCAGGTGACGTGGGTATCTCTACTGGGATGGCTTTGGACCCCCAGTGGGTCTGACTGGAGGTTCACTACAGCAATTTGCACAGTCTTCCTGgt  110
Balaenoptera acutorostrata  aac--AGTTACCAGTTTCCAGGTGAGGTGGGTATCTCTATTGGGACGCCTTTGGACCCCCAGTGGGTCCGACTGGAGATTCACTACAGCAATTTCCACAGCCTTCCTGgt  110
Balaenoptera physalus       aatagAGTTACCAGTTTCCAGGTGATGTGGGTATCTCTATTGGGACGCCTTTGGACACCCAGTGGGTCTGACTGGAGATTCACTACAGCAATCTCCACAGCCTTCCTGgt  110

Sus scrofa                  gag  113
Bos taurus                  gag  113
Orcinus orca                gag  113
Tursiops truncatus          gag  113
Neophocaena phocaenoides    gag  113
Lipotes vexillifer          gag  113
Balaenoptera acutorostrata  gag  113
Balaenoptera physalus       gag  113
```

G. exon 7

```
Sus scrofa                  tccagGGGTGTACGACTCCTCGGGAATTCGAGTCTACTACACGGCAAAGCTGCGCAAATACGACATGGGTGTCCTGCAGCTGGGCTTCTTCACGTTCCCCATCCACTTCA  110
Bos taurus                  cccagGTCTGTACGACTCCTCAGGGATCCGAGTATACTACACCGCACACCTGCGCAAGTTTGACATGGGCGTCCTGCAGCTGGGCGTCTTCACTTTCCCCATCCACTTCA  110
Orcinus orca                accagGTGTGTACGGCTCCTCGGGGATTCGAGTGTACTACACGGCGCAGCTACGCAAATATGACATGGGTGTCCTGCAGCTGGGCTTCTGCACTTTGCCCATCCACTTCG  110
Tursiops truncatus          accagGTGTATACGGCTCCTCGGGGATTCGAGTGTACTACACGGCGCAGCTACGCAAATATGACATGGGTGTCCTGCAGCTGGGCTTCTGCACTTTGCCCATCCACTTCA  110
Neophocaena phocaenoides    accagGTGTGTACGGCTCCTCGGGGATTCGAGTGTACTACACGGCGCAGCTACGCAAATATGACATGGGTGTCCTGCAGCTGGGCTTCTGCACTTTGCCCATCCACTTCA  110
Lipotes vexillifer          accagGTGTGTACGGCTCCTCGGGGATTCGAGTGTACTACACGGCGCAGCTGTGCAAACATGACATGGGTGTCCTGCAGCTGGGCTTCTGCACTTTCTCCATCCACTTCA  110
Balaenoptera acutorostrata  accagGTGTGTACGACTCCTCGGGGATTCGAGTGTACCTCACGGCGCAGCTGCGCAAATATGACATGGGTGTCCTGCAGCTGGGCTTCTTCACTTTCCCCATCCACTTCA  110
Balaenoptera physalus       accagATGTGTACGACTCCTCGGGGATTTGAGTGTACCTCACGGCGCAGCTGCGCAAATATGACATGGGTGTCCTGCAGCTGGGCTTCTTCACTTTCCCCATCCACTTCA  110

Sus scrofa                  TCCCCCCGGGCGCCGAGTCCTTCATGTCCTACGGGCTGTGTAAGACGGACAAGTTTGAGGAGgtgag  177
Bos taurus                  TCCCCCCCGGCGCTGAGTCCTTCATGTCCTACGGACTGTGTAAGACGGAGAAGTTTGAAGAGgtgaa  177
Orcinus orca                TACCCCCGGGTGCCGAGTCCTTCGCGTCCTATGGGCTGTGTAAGGCCGAGAAGTTTGATGAGgtgaa  177
Tursiops truncatus          TACCCCCGAGTGCCGAGTCCTTCGCGTCCTATGGGCTGTGTAAGGCGGAGAAGTTTGACGAGgtgaa  177
Neophocaena phocaenoides    TACCCCCGGGTGCCGAGTCCTTCACGTCCTATGGGCTGTGTAAGGCAGAGAAGTTTGACGAGgtgaa  177
Lipotes vexillifer          TACCCCCGGGCGCCGAGTCCTTCGCATCCTATGGGCTGTGTAAGACGGAGAAGTTTGAAGAGgtgaa  177
Balaenoptera acutorostrata  TACCCCCGGGCGCCGAGTCCTTCGCGTCCTACGGGCTGTGTAAGACGGAGAAGTTTGAAGAGgtgaa  177
Balaenoptera physalus       TACCCCCGGGCGCCGAGTCCTTCGCGTCCTACGGGCTGTGTAAGAAGGAGCAGTTTGAAGAGgtgaa  177
```

H. exon 8

```
Sus scrofa                  tacagATGAATGGGGCCCCGGTGCTTGACATCCAGGTGTATGGCTACCTGCTCCACACCCACTTGGCTGGCCGGGCTCTGCAAGCCGTGCAATACAGgtaag  102
Bos taurus                  cacagATGAACGGGGCTCCAGTGCCCGATATCCAGGTCTTTGGCTACCTGCTCCACACCCACTTGGCTGGCCGTGCTATACAGGCAGTGCAGTACAGgtaag  102
Orcinus orca                cacagATGAACGGGGCCCCGGTGCCTGACATCCAGGTCTGCGGCTACCTGCTCCACACCCACTTGGCTGGCCGCACTCTGCAGGCCGTGCAATACAGgtaag  102
Tursiops truncatus          cacagATGAACGGGGCCCCGGTGCCTGACATCCAGGTCTGCGGCTACCTGTTCCACACCCACTTGGCTGGCCGCACTCTGCAGGCCGTGCAATACAGgtaag  102
Neophocaena phocaenoides    cacagATGAACGGGGCCCCGGTGCCTGACATCCAGGTCTGCAGCTACCTGCTCCACACCCACTTGGCTGGCCGCACTCTGGAGGCCGTGCAATACAGgtaag  102
Lipotes vexillifer          cacagATGTACGGGGCCCTGGTGCCTGGCATCCAGGTCTGCGGCTACCTGCTCCACACCCACTTGGCTGGCCGCACTCTGCAGGCCGTGCAAAACAGgtaag  102
Balaenoptera acutorostrata  cacagATGAACGGGGCCCCGATGCCTGACATCCAGGTCTGCGGCTACCTGCTCCACACCCACTTGGCTGGCCGCACTCTGCAGGCCGTGCAATACAGgtaag  102
Balaenoptera physalus       cacagATGAACGGGGCCCCGGTGCCTGACATCCAGGTCTGCGGCTACCTGCTCCACACCCACTTGGCTGGCCTCACTCTGCAGGCCGTGCAATACAGgtaag  102
```

I. exon 9

```
Sus scrofa                  tctagAAATGGAACACAACTCCAAACAATCTGTAAAGATGACTCCTACGATTTCAATCTGCAGGAGACTCGAGATTTACCTTATCGCATGGTCATCAAGCCGgtgag  107
Bos taurus                  tccagAAACGGAACTCAACTCCGCGTAATCTGTAAAGATGATGCCTACGACTTCAACCTACAGGAGACTCGAGATTTACCTTATCGAGTGGTGATCAAGCCGgtgcg  107
Orcinus orca                tccagAAATGGAACACAACGCCAAACAATCTGGAAAGATGATACCTATGACTTGAATCTGCAGGAGACTTGAGATTTACCTTCTTGAGTGGCCATCAAGCCAgtggg  107
Tursiops truncatus          ttcagAAATGGAACACAACGCCAAACAATCTGGAAAGATGATACCTATGACTTGAATCTGCAGGAGACTTGAGATTTACCTTCTTGAGTGGCCATCAAGCCAgtggg  107
Neophocaena phocaenoides    tccagAAATGGAACACAATGCCGAACAATCTGGAAAGATGATTCCTATGACTTGAATCTGCAGGAGACTCGAGATTTACCTTCTTGAGTGGCCATCAAGCCAgtggt  107
Lipotes vexillifer          tccagAAATGGAACACAACGCCGAACAATCTGTAAAGATGATTCCTAAGACTTCAATCTGCAGGAGACTCGAGATTTACCTTCTCGAGTGGCCATCAAGCCGgtggg  107
Balaenoptera acutorostrata  tccagAAATGGAACACAACTCCGAACAATCTGTAAACATGATTCCTACGACTTCAATCTGCAGGAGACTCGAGATTTACCTTCTCGAGTGGCCATCAAGCCGgtggg  107
Balaenoptera physalus       tccagAAATGGAACACAACTCCGAACAATCTGTAAAGATGATTCCTACGACTTCAATCTGCAGGAGACTCGAGATTTACCTTCTCGAGTGGCCATCAAGCTGgtggg  107
```

J. exon 10

```
Sus scrofa                  cacagGGAGATGAACTGCTGGTAGAGTGTCACTACCAGACGCTGGACCGAGACTTCTTGACCTTTgtaag  70
Bos taurus                  cacagGGGGATGAATTGCTGGTAGAGTGTCGCTACCAGACGCTGGACCGCGACTCCTTGACATTTgtaag  70
Orcinus orca                cacagGGAGATGAATTGTTGGTAGGGTGTCACCATCAGACGCTGGACCACGACTCCTTGACTTTTgtaag  70
Tursiops truncatus          cacagGGAGATGAATTGTTGGTAGGGTGTCACTATCAGACGCTGGACCACGACTCCTTGACTTTTgtaag  70
Neophocaena phocaenoides    cacagGGAGATGAATTGTTGGTAGGGTGTCACTACCAGACGCTGGACCGTGACTCCTTGACTTTTgtaag  70
Lipotes vexillifer          cacagGGAGATGAATTGCTGGTAGGGTGTCACTACCAGACGCTGGACCGCGATTCCTTGACCTTTgttaa  70
Balaenoptera acutorostrata  cacagGGAGATGAATTGCTGGTAGAGTGTCACTACCAGACGCTGGACCGCGACTCCTTGACTTTTgtaag  70
Balaenoptera physalus       cacaaGGAGATGAATTGCTGGTAGAGTGTCACTACCAGACGCTGGACCGCGACTCCTTGACTTTTgtaag  70
```

K. exon 11

```
Sus scrofa                tccag-GGGGGGCCCAGCACCATTAATGAGATGTGCCTCATCTTCCTCTTCTACTATCCCCGAAACAACATCTCCAGCTGCATGGGCTACCCCGACATCATCTATGTGGC  109
Bos taurus                tccag-GGGGGTCCCAGCACCATCAATGAGATGTGCCTCATCTTTTTCTTCTACTATCCCCGAAACAATGTCTCCAGCTGCCAGGGGTACCCGGACATCATCTACGTGGC  109
Orcinus orca              tccag-GGGGGTCCCAGCACCGTTAATGAGATGTGCCTCATCTTTCTCTTCTACTATATCTGAAACAACATCTCCAGCTGCATGGGGTACGCTGACATCATCTACGTGGC  109
Tursiops truncatus        tccag-GGGGGTCCCAGCACCGTTAATGAGATGTGCCTCATCTTTCTCTTCTACTATATCCGAAACAACATCTCCAGCTGCATGGGGTACGCTGACATCATCTACGTGGC  109
Neophocaena phocaenoides  tccag-GGGGGTCCCAGCACCGTTAATGAGATGTGCCTCATCTATCTCTTCTACTATATCCGAAACAACATCTCCAGCTGCATGGGGTACGCTGACATCATCTACGTGGC  109
Lipotes vexillifer        tccagGGGGGGTCCCAGCACCGTTAATGAGATGTGCCTCATCTTTCTCTTCTACTGTCCCCGAAACAACATCTCCAGCTGCATGGGGTAGCCTGACATCATCTACGTGGC  110
Balaenoptera acutorostrata tccag-GGGGGTCCCAGCACCGTTAATGAGATGTGCTTCATCTTTCTCTTCTACTATCCCCGAAACAACATCTCCAGCTGCATGGGGTACGCTGACATCATCCACGTGGC  109
Balaenoptera physalus     tcaagGGGGGGTCCCAGCACCATTAATGAGATGTGCCTCATCTTTCTCTTCTACTATCCCCGAAACAACATCTCCAGCTGCATGGGGTACGCTGACATCATCTACATGGC  110

Sus scrofa                CCATGAGCTGGGGGAGGAGGCTTCAGAgtgag  141
Bos taurus                CCACGAGCTGGGGGAGGAGGTATCAGAgtgag  141
Orcinus orca              CCATGTGCTGGGGGAGGAGGCATCAGAgtgag  141
Tursiops truncatus        CCATGTGCTGGGGGAGGAGGCATCAGAgtgag  141
Neophocaena phocaenoides  CCATGTCCTGGGGGAGGAGGCATCAGAgtgag  141
Lipotes vexillifer        CCATGTGCTGGGGGAGGAGGCATCAGAgtgag  142
Balaenoptera acutorostrata CCATGAGCTGGGGGAGGAGGCATCAGAgtgag  141
Balaenoptera physalus     CCATGAGCTGGGGGAGGAGGCATCAGAgtgag  142
```

L. exon 12

```
Sus scrofa                aacagTTCCATGGAGGGCATGATGGCCATGAACAATGTGGAGTGGACCCCGGAAAGCATTAAGAAGGCAGAGAAAGCCTGCAAGGAGGCCCAGCAGACGGTGATAATAAA  110
Bos taurus                ggcagCTCCATGGAGGGCATGATGGCCATGAGCAATGTGGAGTGGACCCCGGAGAGCATTAAGAAGGTTGAGAAGGCCTGCAAGGAGGCCCAGCAGACAGTGATAATAAA  110
Orcinus orca              gacagTTCTATGGAGGGCATGATGGCCATGAACAATGTGGAGTGGACTCCGGAGAACATTAAAAAGGCTGAGAAGGCCTGCGAGGAGGCCCAGCAGATGGTGCTAATAAA  110
Tursiops truncatus        gacagTTCCATGGAGGGCATGATGGCCATGAACAATGTGGAATGGACTCCGGAGAACATTAAAAAGGCTGAGAAGGCCTGCGAGGAGGCCCAGCAGATGGTGATAATAAA  110
Neophocaena phocaenoides  gacagTTCCATGGAGGGCATGATGGCCATGAACAATGTAGAGTGGACTCTGGAGAACATTAAAAAGGCTGAGAAGGCCTGCGAGGAGGCCCAGCAGATGGTGATAATAAA  110
Lipotes vexillifer        gacagTTCCATGCAGGGCATGATGGCCATGAACAATGTGGAGTGGACTCCGGAGAACATTAAAAAGGCTGAGAAGGGCTGTGAGGAGGCCCAGCAGACGGTGATAATAAA  110
Balaenoptera acutorostrata gacagTTCCATGGAAGGCATGATGGCCATGAACAATGTGGAGTGGACCCCGGAGAACATTAAAAAGGCTGAGAAGGCCTGCAAGGAGGCCCAGCAGACGGTGATAATAAA  110
Balaenoptera physalus     gacagCTCCATGGAGGGTATGATGGCCACGAACAAGGTGGAGTGGACCCCGGAGAACATCAAAAAGGCTGAGAAGGCGTGCAAGGAGGCCCAGCAGACGGTGATAATAAA  110

Sus scrofa                GACCATTGACgtaag  125
Bos taurus                GACCATTGATgtaag  125
Orcinus orca              GACCATTGACgtaag  125
Tursiops truncatus        GACCATTGACataag  125
Neophocaena phocaenoides  GACCATTGATgtaag  125
Lipotes vexillifer        GACCATTGACgtaag  125
Balaenoptera acutorostrata GACCATTGATgtaag  125
Balaenoptera physalus     GACCATTGACgtaag  125
```

M. exon 13

```
Sus scrofa                  tgcagGAGCTAGTGGAAAACACCACAGGCTGGATTCCGGAAATTGTCCCTACTCCCCGAGGCCCCTGCTTGGAGTCCTCCGGAGG------CAAAGTGG--AGCCCGAGG  102
Bos taurus                  tgcagGAGCTAGTGGAAAACACAACAGGCTGGATTCAGGACATCAACCCTACTCCCCGGGGTCCTTGTTTGGAGTCCTCTGGAGG------CAAAGTGG--AACCCCAGG  102
Orcinus orca                tgcagGAGCTAGTGGAAAACAGAACAGGCTGGATTCTGGAAATCATCCCTGCTCCCCGGGGTCCTTGCTTAGAGTCCTCCAGAGG------CAAAGTGG--AGCCCCAGG  102
Tursiops truncatus          tgcagGAACTAGTGGAAAACAGAACAGGCTGGATTCCGGGAATCATCCCTGCTCCCCGGGGTCCTTGCTTAGAGTCCTCCAGAGG------CAAAGTGG--AGCCCCAGG  102
Neophocaena phocaenoides    tgcagGAGCTAGTGGAAAACACAACAGGCTGGATTCCGGAAATCATCCCTGCTCCCCGGGGTCCTTGCTTAGAGTCCTCCAGAGG------CAAAGCGG--AGCCCCAGG  102
Lipotes vexillifer          tgcagGAGCTAGTGGAAAACACAACAGGCTGGATTCTGGAAATCATCCCTGCTCCCCGGGGTCCTTGCTTAGAGTCCTCCAGAGG------CAAAGTGG--AGCCCCAGG  102
Balaenoptera acutorostrata  tgcagGAGCTAGTGGAAAACACAACAGGCTGGATTCCGGAAATCATCCCTGCTCCCCGGGGTCCTTGCTTGGAGTCCTCCAGAGG------CAAAGTGG--AGCCCCAGG  102
Balaenoptera physalus       tgcagGAGCTAGTGGAAAACACAACAGGCTGGATTCTGGAAATCATCCCTGCTCCCCGGGGTCCTTGCTTGGAGTCCTCCAGAGGCCTCCTCCTGGTGGACAGAACCAGG  110

Sus scrofa                  ACCAAACCCCCGCTGGCTTCAGGGCTGCACCAATGGCCCTCTCCGTCTCCAGCGCTGCTACTCCAAGTCGCCTCCCCCTGGCTGCCTTCTTACTTGGGCAGGGGACCC-T  211
Bos taurus                  ACAAAACCCCTGCAGGCTTCAGGGCGGCCCCCATTGTCCTCTCAAGGGCCAGCAGTGCCACCCTGAGGTGCCTCCCCCTGGCCGCCCTCTTGTTTGGGCAGGGGGCCC-T  211
Orcinus orca                ACAGAACCCCTGTAGGCTTCAGGGCTGCCCCCATGGCCCTCTCGGGCTCCAGCACTGCCACACCAAGGCGCCTCCCCCTGACTGCCCTCTTGTTTGGGCAGGGGGCCG-T  211
Tursiops truncatus          ACAGAACCCCTGTAGGCTTCAGGGCTGCCCCCATGGCCCTCTCGGGCTCCAGCGCTGCCACACCAAGGCGC--CCCCCCGACTGCCCTCTTGTTTGGGCAGGGGGCCG-T  209
Neophocaena phocaenoides    ACAGAACCCCTGTAGGCTTCAGGGCTGCCCCCATGGCCCTCTCGGGCTCCAGCACTGCCACACCAAGGCGCCTCCCCCTGACTGCCCTCTTGTTTGGGCAGGGGGCCG-T  211
Lipotes vexillifer          ACAGAACCCCTGTAGGCTTCAGGGCTGCCCCCATGGCCCTCTCGGGCTCCGGCACTGCCACACCAAGGCGCCGCCCCCTGACTGCCCTCTTGTTTGGGCAGAGGGCCG-T  211
Balaenoptera acutorostrata  ACAGAACCCCTGCAGGCTTCAGGGCGGCCCCCATGGCCCTCTCAGGCTCCAGCACTGCCACACCAAGGCGCCTCCCCCTGACTGCCCTCTTGTTTGGGCAGGGGGCCG-T  211
Balaenoptera physalus       ACAGAACCCCTGCAGGCTTCAGGGCGGCCCCCATGGCCCTCTCGGGCTCCAGCACTGCCACACCAAGGCGGCTCCCCCTGACTGCCCTCTTGTTTGGTCAGGGGGCCCGT  220

Sus scrofa                  CTCTTGGCTCCTTGCCACCCTGCAGGCTGGAGTCTGAcactg  253
Bos taurus                  GTCTTGGCTCCTTGCCACCCTGCAGTCTGGAATCTGAtaccg  253
Orcinus orca                GTCTTGGCTCCTAGCCACCCTGCGGGCTGGAGCCTGAtaccg  253
Tursiops truncatus          GTCTTGGCTCCTAGCCACCCTGCGGGCTGGAGCCTGAtaccg  251
Neophocaena phocaenoides    GTCTTGGCTCCTAGCCACCCTGCGGGCTGGAGCCTGAtaccg  253
Lipotes vexillifer          GTCTTGGTTCCTAGCCACGCTGTAGGCTGGAGCCTGAtaccg  253
Balaenoptera acutorostrata  GTCTTGGCTCCTAGCCACCCTGCGGGCTGGAGCCTGAtaccg  253
Balaenoptera physalus       GTCTTGGCTCCTAGCCACCCTGCGGGCTGGAGCCTGAtaccg  262
```
