# Supplementary material for: Frequent Loss and Alteration of the MOXD2 Gene in Catarrhines and Whales: A Possible Connection with the Evolution of Olfaction
Source: PLoS One. 2014 Aug 7;9(8):e104085. doi: 10.1371/journal.pone.0104085 (PMC4125168; doi:10.1371/journal.pone.0104085)
Supplement: Data S1 — Coding and protein sequences of MOXD2 genes. (PDF) [file pone.0104085.s004.pdf]

Data S1. Coding and protein sequences of *MOXD2* genes

1. Human (*Homo sapiens*)

```
>Homo_sapiens No=1 length=1499 name="Human"
ATGGCCCATGACCTTCTCTTCAGGCTTTTTCCACTTTTGGCCCTGGGAGTCCCCTTACAAAGCAACCGCCTTGGCCCCACATCTCGCCTGCGCTATTCCA
GGTTCCTAGATCCTTCTAATGTCATTTTCCTGCGTTGGGACTTTGACCTTGAGGCTGAAATCATCAGTTTTGAGCTCCAGGTCCGTACAGCTGGCTGGGT
GGGCTTCGGTGTCACAAATCGCTACACCAACGTGGGAAGTGATCTGGTTGTTGGAGGAGTCTTGCCTAATGGCAATGTCTATTTCTCGGATCAGCACTTG
GTGGACGAAGACACTCTGAAGGAGGATGGGAGCCAGGATGCTGAGCTGCTGCGGCTGACGGAAGATGCTGTCTACACCACCATGCACTTTTCCAGGCCCT
TCCGCTCCTGCGACCCTCATGACCTGGATATTACGAGCAACACCGTGAGGGTGCTGGCCACCTATGGCCTGGATGACACTCTGAAGCTGTATCGGGAGCG
TACTTTTGTCAAGTCCATCTTTCTGCTACAAGTCGTCCACCCTGACGATCTGGATGTCCCTGAGGACACCATCATCCATGACTTGGAGATCACTAATTTC
CTCATTCCAGAGGATGACACCACGTATGCCTGCACCTTTCTTCCTCTCCCTATTGTGAGCGAGAAGCATCACATTTACAAGTTTGAGCCTAAGTTGGTCT
ACCACAATGAGACAACGGTGCATCACATCCTGGTGTACGCCTGCGGCAATGCTAGCGTTCTCCCCACAGGCATCAGCGACTGCTATGGGGCCGACCCTGC
CTTCTCCCTTTGCTCACAGGTCATCGTGGGCTGGGCTGTCGGGGGCACAAGCTACCAGTTTCCAGATGACGTAGGCGTCTCTATTGGGACCCCCTTGGAC
CTTCAGTGGATCCGACTGGAGATTCATTACAGCAATTTTAACAACCTTCCTGGTGTGTATGATTCCTCGGGGATTTGCGTGTACTACACTTCTCAGCTGC
GCAAATACGACACGGATGTCCTCCAGCTGGGCTTCTTCACGTTTCCCATCCACTTCATCCCCCCGGGCGCTGAGTCCTTCATGTCCTATGGGCTGTGTAG
GACGGAGAAGTTTGAGGAGATGAATGGAGCTCCTATGCCTGACATACAGGTGTATGGCTACCTGCTGCACACCCACTTGGCTGGACGGGCTCTGCAAGCA
GTGCAATACAGGAATGGAACACAACTTCGAAAAATCTGTAAAGACGATTCCTATGACTTCAACCTGCAGGAGACTCGAGATTTGCCCTCTAGAGTGGAGA
TCAAGCCGGGAGATGAATTGCTGGTAGAATGTCACTACCAGACACTGGACCGTGACTCCATGACATTTGGAGGCCCCAGCACCATTAATGAGATGTGCCT
CATCTTCCTCTTCTACTATCCCCAAAATAACATCTCCAGCTGCATGGGGTACCCTGACATTATCTACGTGGCCCACGAGCTGGGGGAGGAGGCATCAGA

>Homo_sapiens No=1 length=499 name="Human"
MAHDLLFRLFPLLALGVPLQSNRLGPTSRLRYSRFLDPSNVIFLRWDFDLEAEIISFELQVRTAGWVGFGVTNRYTNVGSDLVVGGVLPNGNVYFSDQHL
VDEDTLKEDGSQDAELLRLTEDAVYTTMHFSRPFRSCDPHDLDITSNTVRVLATYGLDDTLKLYRERTFVKSIFLLQVVHPDDLDVPEDTIIHDLEITNF
LIPEDDTTYACTFLPLPIVSEKHHIYKFEPKLVYHNETTVHHILVYACGNASVLPTGISDCYGADPAFSLCSQVIVGWAVGGTSYQFPDDVGVSIGTPLD
LQWIRLEIHYSNFNNLPGVYDSSGICVYYTSQLRKYDTDVLQLGFFTFPIHFIPPGAESFMSYGLCRTEKFEEMNGAPMPDIQVYGYLLHTHLAGRALQA
VQYRNGTQLRKICKDDSYDFNLQETRDLPSRVEIKPGDELLVECHYQTLDRDSMTFGGPSTINEMCLIFLFYYPQNNISSCMGYPDIIYVAHELGEEAS
```

(1) Exon coordinates

Exon	UCSC Chromosome	Strand	Start	End
1	hg19 chr7	-	141946599	141946886
2	hg19 chr7	-	141945806	141945952
3	hg19 chr7	-	141945109	141945270
4	hg19 chr7	-	141944780	141944863
5	hg19 chr7	-	141943796	141943963
6	hg19 chr7	-	141943502	141943604
7	hg19 chr7	-	141943143	141943309
8	hg19 chr7	-	141942701	141942792
9	hg19 chr7	-	141942317	141942413
10	hg19 chr7	-	141942123	141942182
11	hg19 chr7	-	141940560	141940690
12	not present			
13	not present			

2. Chimpanzee (*Pan troglodytes*)

```
>Pan_troglodytes No=2 length=1857 name="Chimpanzee"
ATGGCCCATGACCTTCTCTTCAGGCTTTTTCCACTTTTGGCCCTGGGAGCCCCCTTACAAAGCAACCGCCTTGGCCCCACATCTCGCCTGCGCTATTCCA
GGTTCCTAGATCCTTCTAATGTCATTTTCCTGCGTTGGGACTTTGACCTTGAGGCTGAAATCATCAGTTTTGAGCTCCAGGTCCGTACAGCTGGCTGGGT
GGGCTTCGGTGTCACAAATCGCTACACCAACGTGGGAAGTGATCTGGTTGTTGGAGGAGTCTTGCCTAATGGCAATGTCTATTTCTCGGATCAGCACTTG
GTGGACGAAGACACTCTGAAGGAGGATGGGAGCCAGGATGCTGAGCTGCTGGGGCTGACGGAAGATGCTGTCTACACCACCATGCGCTTTTCCAGGCCCT
TCCGCTCCTGCGACCCTCATGACCTGGATATTACGAGCGACACCGTGAGGGTGCTGGCCGCCTATGGCCTGGATGACACTCTGAAGCTGGATCGGGAGCG
TACTTTTGTCAAGTCCATCTTTCTGCTACAAGTCGTCCACCCTGACGATCTGGATGTCCCTGAGGACACCATCATCCATGACTTGGAGATCACTGATTTC
CTCATTCCAGAGGATGACACCACGTATGCCTGCACCTTTCTTCCTCTCCCTATTGTGAGCGAGAAGCATCACATTTACAAGTTTGAGCCTAAGTTGGTCT
ACCACAATGAGACAACGGTGCATCACATCCTGGTGTACGCCTGTGGCAATGCTAGCGTTCTCCCCACAGGCATCAGCGACTGCTATGGGGCCGACCCTGC
CTTCTCCCTTTGCTCACAGGTCATCGTGGGCTGGGCTGTCGGGGGCACAAGCTACCAGTTTCCAGATGACGTAGGCGTCTCTATTGGGACCCCCTTGGAC
CCTCAGTGGATCCGACTGGAGATTCATTACAGCAATTTTAACAACCTTCCTGGTGTATATGATTCCTCGGGGATTCGCGTGTACTACACTTCTCAGCTGC
GCAAATACGACATGGGTGTCCTCCAGCTGGGCTTCTTCACGTTTCCCATCCACTTCATCCCCCCGGGCGCTGAGTCCTTCATGTCCTATGGGCTGTGTAG
GACGGAGAAGTTTGAGGAGATGAATGGAGCTCCTATGCCTGACATACAGGTGTATGGCTACCTGCTGCACACCCACTTGGCTGGACGGGCTCTGCAAGCA
GTGCAGTACAGGAATGGAACACAACTTCGAAAAATCTGTAAAGACGATTCCTATGACTTCAACCTGCAGGAGACTCGAGATTTGCCCTCTCGAGTGGAGA
TCAAGCCGGGAGATGAATTGCTGGTAGAATGTCACTACCAGACACTGGACCGTGACTCCATGACATTTGGAGGCCCCAGCACCATTAATGAGATGTGCCT
CATCTTCCTCTTCTACTATCCCCGAAATAACATCTCCAGCTGCATGGGGTACCCTGACATTATCTACGTGGCCCACGAGCTGGGGGAGGAGGCATCAGAC
TCCATGGAGGGAATGATGGCCATGAACAATGTCGAGTGGACCCCGGAGAGCATTAAGAAAGCTGAGAAAGTCTGCAAGGAGGCCCAGCAGACAGTGATAA
TAAAGACCATTGATGAGGTGGTGGAAAACACAACAGGCTGGATTCCAGACATCATCCCTACTCTCCGGGGTCCCTGCTTGGAGTCCTCCGGAGGCAAAGT
GGAGGCCCAGGACAAGACCTCTGCAGGCTTCAGAGCTGCACCTGTAGCCCTCTCAGGCTCTAGCAGGGCCACCCTGAGGCATCTCCCCCTGGCGGCCGTC
CTGCTGGTGCAGCGCACACTCTCCTGGCTCCTGGCCATGCTGCAGACTGGAGTCTGA

>Pan_troglodytes No=2 length=618 name="Chimpanzee"
MAHDLLFRLFPLLALGAPLQSNRLGPTSRLRYSRFLDPSNVIFLRWDFDLEAEIISFELQVRTAGWVGFGVTNRYTNVGSDLVVGGVLPNGNVYFSDQHL
VDEDTLKEDGSQDAELLGLTEDAVYTTMRFSRPFRSCDPHDLDITSDTVRVLAAYGLDDTLKLDRERTFVKSIFLLQVVHPDDLDVPEDTIIHDLEITDF
LIPEDDTTYACTFLPLPIVSEKHHIYKFEPKLVYHNETTVHHILVYACGNASVLPTGISDCYGADPAFSLCSQVIVGWAVGGTSYQFPDDVGVSIGTPLD
PQWIRLEIHYSNFNNLPGVYDSSGIRVYYTSQLRKYDMGVLQLGFFTFPIHFIPPGAESFMSYGLCRTEKFEEMNGAPMPDIQVYGYLLHTHLAGRALQA
VQYRNGTQLRKICKDDSYDFNLQETRDLPSRVEIKPGDELLVECHYQTLDRDSMTFGGPSTINEMCLIFLFYYPRNNISSCMGYPDIIYVAHELGEEASD
SMEGMMAMNNVEWTPESIKKAEKVCKEAQQTVIIKTIDEVVENTTGWIPDIIPTLRGPCLESSGGKVEAQDKTSAGFRAAPVALSGSSRATLRHLPLAAV
LLVQRTLSWLLAMLQTGV
```

(1) Exon coordinates

Exon	UCSC Chromosome	Strand	Start	End
1	panTro4 chr7	-	143713775	143714062
2	panTro4 chr7	-	143712983	143713129
3	panTro4 chr7	-	143712286	143712447
4	panTro4 chr7	-	143711957	143712040
5	panTro4 chr7	-	143710976	143711143
6	panTro4 chr7	-	143710682	143710784
7	panTro4 chr7	-	143710323	143710489
8	panTro4 chr7	-	143709881	143709972
9	panTro4 chr7	-	143709497	143709593
10	panTro4 chr7	-	143709303	143709362
11	panTro4 chr7	-	143707730	143707860
12	panTro4 chr7	-	143706823	143706937
13	panTro4 chr7	-	143706289	143706531

3. Bonobo (*Pan paniscus*)

```
>Pan_paniscus No=3 length=1857 name="Bonobo"
ATGGCCCATGACCTTCTCTTCAGGCTTTTTCCACTTTTGGCCCTGGGAGCCCCCTTACAAAGCAACCGCCTTGGCCCCACATCTCGCCTGCGCTATTCCA
GGTTCCTAGATCCTTCTAATGTCATTTTCCTGCGTTGGGACTTTGACCTTGAGGCTGAAATCATCAGTTTTGAGCTCCAGGTCCGTACAGCTGGCTGGGT
GGGCTTCGGTGTCACAAATCGCTACACCAACGTGGGAAGTGATCTGGTTGTTGGAGGAGTCTTGCCTAATGGCAATGTCTATTTCTCGGATCAGCACTTG
GTGGACGAAGACACTCTGAAGGAGGATGGGAGCCAGGATGCTGAGCTGCTGGGGCTGACGGAAGATGCTGTCTACACCACCATGCGCTTTTCCAGGCCCT
TCCGCTCCTGCGACCCTCATGACCTGGATATTACGAGCGACACCGTGAGGGTGCTGGCCGCCTATGGCCTGGATGACACTCTGAAGCTGGATCGGGAGCG
TACTTTTGTCAAGTCCATCTTTCTGCTACAAGTCGTCCACCCTGACGATCTGGATGTCCCTGAGGACACCATCATCCATGACTTGGAGATCACTGATTTC
CTCATTCCAGAGGATGACACCACGTATGCCTGCACCTTTCTTCCTCTCCCTATTGTGAGCGAGAAGCATCACATTTACAAGTTTGAGCCTAAGTTGGTCT
ACCACAATGAGACAACGGTGCATCACATCCTGGTGTACGCCTGTGGCAATGCTAGCGTTCTCCCCACAGGCATCAGCGACTGCTATGGGGCCGACCCTGC
CTTCTCCCTTTGCTCACAGGTCATCGTGGGCTGGGCTGTCGGGGGCACAAGCTACCAGTTTCCAGATGACGTAGGCGTCTCTATTGGGACCCCCTTGGAC
CCTCAGTGGATCCGACTGGAGATTCATTACAGCAATTTTAACAACCTTCCTGGTGTATATGATTCCTCGGGGATTCGCGTGTACTACACTTCTCAGCTGC
GCAAATACGACATGGGTGTCCTCCAGCTGGGCTTCTTCACGTTTCCCATCCACTTCATCCCCCCGGGCGCTGAGTCCTTCATGTCCTATGGGCTGTGTAG
GACGGAGAAGTTTGAGGAGATGAATGGAGCTCCTATGCCTGACATACAGGTGTATGGCTACCTGCTGCACACCCACTTGGCTGGACGGGCTCTGCAAGCA
GTGCAGTACAGGAATGGAACACAACTTCGAAAAATCTGTAAAGACGATTCCTATGACTTCAACCTGCAGGAGACTCGAGATTTGCCCTCTCGAGTGGAGA
TCAAGCCGGGAGATGAATTGCTGGTAGAATGTCACTACCAGACACTGGACCGTGACTCCATGACATTTGGAGGCCCCAGCACCATTAATGAGATGTGCCT
CATCTTCCTCTTCTACTATCCCCGAAATAACATCTCCAGCTGCATGGGGTACCCTGACATTATCTACGTGGCCCACGAGCTGGGGGAGGAGGCATCAGAC
TCCATGGAGGGAATGATGGCCATGAACAATGTCGAGTGGACCCCGGAGAGCATTAAGAAAGCTGAGAAAGCCTGCAAGGAGGCCCAGCAGACAGTGATAA
TAAAGACCATTGATGAGGTGGTGGAAAACACAACAGGCTGGATTCCAGACATCATCCCTACTCCCCGGGGTCCCTGCTTGGAGTCCTCCGGAGGCAAAGT
GGAGGCCCAGGACAAGACCTCTGCAGGCTTCAGAGCTGCACCTGTAGCCCTCTCAGGCTCTAGCAGGGCCACCCTGAGGCATCTCCCCCTGGCGGCCGTC
CTGCTGGTGCAGCGCACACTCTCCTGGCTCCTGGCCATGCTGCAGACTGGAGTCTGA

>Pan_paniscus No=3 length=618 name="Bonobo"
MAHDLLFRLFPLLALGAPLQSNRLGPTSRLRYSRFLDPSNVIFLRWDFDLEAEIISFELQVRTAGWVGFGVTNRYTNVGSDLVVGGVLPNGNVYFSDQHL
VDEDTLKEDGSQDAELLGLTEDAVYTTMRFSRPFRSCDPHDLDITSDTVRVLAAYGLDDTLKLDRERTFVKSIFLLQVVHPDDLDVPEDTIIHDLEITDF
LIPEDDTTYACTFLPLPIVSEKHHIYKFEPKLVYHNETTVHHILVYACGNASVLPTGISDCYGADPAFSLCSQVIVGWAVGGTSYQFPDDVGVSIGTPLD
PQWIRLEIHYSNFNNLPGVYDSSGIRVYYTSQLRKYDMGVLQLGFFTFPIHFIPPGAESFMSYGLCRTEKFEEMNGAPMPDIQVYGYLLHTHLAGRALQA
VQYRNGTQLRKICKDDSYDFNLQETRDLPSRVEIKPGDELLVECHYQTLDRDSMTFGGPSTINEMCLIFLFYYPRNNISSCMGYPDIIYVAHELGEEASD
SMEGMMAMNNVEWTPESIKKAEKACKEAQQTVIIKTIDEVVENTTGWIPDIIPTPRGPCLESSGGKVEAQDKTSAGFRAAPVALSGSSRATLRHLPLAAV
LLVQRTLSWLLAMLQTGV
```

(1) Exon coordinates

Exon	NCBI Accession	Strand	Start	End
1	AJFE01039607	-	55003	55290
2	AJFE01039607	-	54211	54357
3	AJFE01039607	-	53514	53675
4	AJFE01039607	-	53185	53268
5	AJFE01039607	-	52203	52370
6	AJFE01039607	-	51909	52011
7	AJFE01039607	-	51550	51716
8	AJFE01039607	-	51112	51203
9	AJFE01039607	-	50728	50824
10	AJFE01039607	-	50534	50593
11	AJFE01039607	-	48961	49091
12	AJFE01039607	-	48054	48168
13	AJFE01039607	-	47514	47756

4. Western lowland gorilla (*Gorilla gorilla gorilla*)

>Gorilla_gorilla_gorilla No=4 length=1857 name="Western lowland gorilla"
ATGGCCCATGACCTTCTCTTCAGGCTTTTTCCACTTTTGGCCCTGGGAGTCCCCTTACAAAGCAACCGCCTTGGCCCCACATCTCGCCTGCGCTATTCCA
GGTTCCTAGATCCTTCTAATGTCATTTTCCTGAGTTGGGACTTTGACCTTGAGGCTGAAATCATCAGTTTTGAGCTCCAGGTCCGTACAGCTGGCTGGGT
GGGCTTCGGTGTCACAAATCGCTACACCAACGTGGGAAGTGATCTGGTTGTTGGAGGAGTCTTGCCTAATGGCAATGTCTATTTCTCGGATCAGCACTTG
GTGGACGAAGACACTCTGAAGGAGGATGGGAGCCAGGATGCTGAGCTGCTGGGGCTGACGGAAGATGCTGTCTACACCACCATGCGCTTTTCCAGGCCCT
TCCGCTCCTGCGACCCTCATGACCTGGATATTACGAGCGACACCGTGAGGGTGCTGGCCGCCTATGGCCTGAATGACACTCTGAAGCTGGATCGGGAGCG
TACTTTTGTCAAGTCCATCTTTCTGCTACAAGTCGTCCACCCTGACGATCTGGATGTCCCTGAGGACACCATCATCCATGACTTGGAGATCACTGATTTC
CTCATTCCAGAGGATGACACCACGTATGCCTGCACCTTTCTTCCTCTCCCTATTGTGAGCGAGAAGCATCACATTTACAAGTTTGAGCCTAAGTTGGTCT
ACCACAATGAGACAACGGTGCATCACATCCTGGTGTACGCCTGTGGCAATGCTAGCGTTCTCCCCACAGGCATCAGCGACTGCTATGGGGCCGACCCTGC
CTTCTCCCTTTGCTCACAGGTCATCGTGGGCTGGGCTGTCGGGGGCACAAGCTATCAGTTTCCAGATGACGTAGGCATCTCTATTGGGACCCCTTTGGAC
CCTCAGTGGATCCGACTGGAGATTCATTACAGCAATTTTAACAACCTTCCTGGTGTGTATGATTCCTCGGGGATTCGCGTGTACTACACTTCTCAGCTGC
GCAAATACGACATGGGTGTCCTCCAGCTGGGCTTCTTCACGTTTCCCATCCACTTCATCCCCCCGGGCGCTGAGTCCTTCATGTCCTATGGGCTGTGTAG
GACGGAGAAGTTTGAGGAGATGAATGGAGCTCCTATGCCTGACATGCAGGTGTATGGCTACCTGCTGCACACCCACTTGGCTGGACGGGCTCTGCAAGCA
GTGCAGTACAGGAATGGAACACAACTTCGAAAAATCTGTAAAGACGATTCCTATGACTTCAACCTGCAGGAGACTCGAGATTTGCCCTCTCGAGTGCAGA
TCAAGCCGGGAGATGAATTGCTGGTAGAATGTCACTACCAGACACTGGACCGTGACTCCGTGACATTTGGAGGCCCCAGCACCATTAATGAGATGTGCCT
CATCTTCCTCTTCTACTATCCCCGAAATAACATCTCCAGCTGCATGGGGTACCCTGACATTATCTACGTGGCCCACGAGCTGGGGGAGGAGGCATCAGAC
TACATGGAGGGAATGATGGCCATGAACAATGTCGAGTGGACCCCAGAGAGCATTAAGAAAGCTGAGAAAGCCTGCAAGGAGGCCCAGCAGACAGTGATAA
TAAAGACCATTGATGAGGTGTTGGAAAACACAACAGGCTGGATTCCAGACATCATCCCTACTCCCCGGGGGCCCTGCTTGGAGTCCTCCGGAGGCAAAGT
GGAGGCCCAGGACAAGACCTCTGCAGGCTTCAGAGCTGCACCTGTAGCCCTCTCAGGCTCTAGCAGGGCCACCCTGAGGCTCCTCCCCCTGGCGGCCGTC
CTGCTGGTGCAGCGCACACTCTCCTGGCTCCCCGCCATGCTGCAGACTGGAGTCTGA

>Gorilla_gorilla_gorilla No=4 length=618 name="Western lowland gorilla"
MAHDLLFRLFPLLALGVPLQSNRLGPTSRLRYSRFLDPSNVIFLSWDFDLEAEIISFELQVRTAGWVGFGVTNRYTNVGSDLVVGGVLPNGNVYFSDQHL
VDEDTLKEDGSQDAELLGLTEDAVYTTMRFSRPFRSCDPHDLDITSDTVRVLAAYGLNDTLKLDRERTFVKSIFLLQVVHPDDLDVPEDTIIHDLEITDF
LIPEDDTTYACTFLPLPIVSEKHHIYKFEPKLVYHNETTVHHILVYACGNASVLPTGISDCYGADPAFSLCSQVIVGWAVGGTSYQFPDDVGISIGTPLD
PQWIRLEIHYSNFNNLPGVYDSSGIRVYYTSQLRKYDMGVLQLGFFTFPIHFIPPGAESFMSYGLCRTEKFEEMNGAPMPDMQVYGYLLHTHLAGRALQA
VQYRNGTQLRKICKDDSYDFNLQETRDLPSRVQIKPGDELLVECHYQTLDRDSVTFGGPSTINEMCLIFLFYYPRNNISSCMGYPDIIYVAHELGEEASD
YMEGMMAMNNVEWTPESIKKAEKACKEAQQTVIIKTIDEVLENTTGWIPDIIPTPRGPCLESSGGKVEAQDKTSAGFRAAPVALSGSSRATLRLLPLAAV
LLVQRTLSWLPAMLQTGV

(1) Exon coordinates

Exon	UCSC Chromosome	Strand	Start	End
1	gorGor3 chr7	-	140690779	140691066
2	gorGor3 chr7	-	140689987	140690133
3	gorGor3 chr7	-	140689290	140689451
4	gorGor3 chr7	-	140688961	140689044
5	gorGor3 chr7	-	140687971	140688138
6	gorGor3 chr7	-	140687677	140687779
7	gorGor3 chr7	-	140687318	140687484
8	gorGor3 chr7	-	140686876	140686967
9	gorGor3 chr7	-	140686492	140686588
10	gorGor3 chr7	-	140686298	140686357
11	gorGor3 chr7	-	140684715	140684845
12	gorGor3 chr7	-	140683808	140683922
13	gorGor3 chr7	-	140683268	140683510

5. Sumatran orangutan (*Pongo abelii*)

>Pongo_abelii No=5 length=1857 name="Sumatran orangutan"
ATGGCCCATGACCTTCTCTTCAGGCTTTTTCCACTTTTGGCCCTGGGAGCCCCCTTACAAAGCAACCGCCTTGGCCCCACATCTCGCCTGTGCTATTCCA
GGTTCCTAGATCCTTCTAATGTCATTTTCCTGCGTTGGGACTTTGACCTTGAGGCTGAAATCATCAGTTTTGAGCTCCAGGTCCGGAGAGCTGGCTGGGT
GGGCTTGGGTGTCACAAATCGCTACACCAACGTGGGAAGTGACCTGGTCGTTGGAGGAGTCTTGCCTAAAGGCAATGTCTATTTCTCGGATCAGCACTTG
GTGGACGAAGACACTCTGAAGGAGGATGGGAGCCAGGATACTGAGCTGCTGGGGCTGACGGAAGATGCTGTTTACACCACCATGCGTTTTTCCAGGCCCT
TCCGCTCCTGCGATCCTCATGACCTGGATATTATGAGCGACACTGTGAGGGTGCTGGCTGCCTATGGCCTGGATGACACTCCGAAGCTGGATCAGGAGCG
TACTTTTGTCAAGTCTATCTTTCTGCTACAAGTCGTCCACCCTGACAATCTGGATGTCCCTGAGGACACCATCATCCATGACTTGGAGATCACTGATTTC
CTCATTCCAGAGGATGACACCACGTATGCCTGCACCTTTCTTCCTCTCCCTATTGTGAGCCAGAAGCATCACATTTACAAGTTTGAGCCTAAGTTGGTCT
ACCACAATGAGACAATGGTGCATCACATCCTGGTGTATGCCTGTGGCAATGCTAGCGTTCTCCCCACAGGCATCAGCGACTGCTATGGGGCCGACCCCGC
CTTCTCCCTCTGCTCACAGGTCATCGTGGGCTGGGCTGTCAGGGGCACTAGCTACCAGTTTCCAGATGACGTAGGCGTCTCTATTGGGACCCCCTTAGAC
CCTCAGTGGATCCGACTGGAGATTCATTACAGCAATTTTAACAACCTTCCTGGTGTGTATGATTCCTCAGGGATTCACGTGTACTACACTTCTCAGCTGC
GCAAATATGACATGGGCGTCCTCCAGCTGGGCTTCCTCACGTTTCCCATCCACTTCATCCCCCCGGGCGCTGAGTCCTTCATGTCCTGTGGGCTGTGTAG
GACGGAGAAGTTTGAGGAGATGAATGGAGCTCCTATGCCTGACATACAGGTATATGGCTACCTGCTGCACACCCACTTTGCTGGACGGGCTCTGCAAGCA
GTGTAATACAGGAATGGAACACAACTTCGAACAATCTGTAAAGACGATTCCTACGACTTCAACCTGCAGGAGACTCGAGATTTGCCCTCTCGAGTGGAGA
TCAAGCTGGGAGATGGATTGCTGGTAGAATGTCACTACCAGACACTGGACTGTGACTCCATGACATTTGGAGGCCCCAGCACCATTAATGAGATGTGCCT
CATCTTCCTCTTCTACTATCCCCGAAATAACATCTCTAGCTGCATGGGGTACGCTGACATCATCTACGTGGCCCACGAGCTGGGGGAGGAGGCATCAGAC
CCCATGGAGGGAATGATGGACATGAATGATGTCAAGTGGACCCTGGAGAGCATTAAGAAAGCTGAGAAAGCCTGCAAGGAGGCCCAGCAGACAGTGATAA
TAAAGACCATTGATGAGGTGGTGGAAAACACAACAGGCTGGATTCCAGACATCATCCCTGCTCCCCGGGGGCCCTGCTTGGAGTCATCTGGAGGCAAAGT
GGAGGCCCAGGACAAGACCCCTACAGGCTTCAGAGCTGCACCTGTGGCCCTCTCGAGCTCTAGCAGGGCCACCCTGAGGCATCTCCCCCTGGTGGCCATC
CTGCTGCTGCAGCACACACTCTTCTGGCTCCTGGCCATGCTGCAGAATGGAGTCTGA

>Pongo_abelii No=5 length=618 name="Sumatran orangutan"
MAHDLLFRLFPLLALGAPLQSNRLGPTSRLCYSRFLDPSNVIFLRWDFDLEAEIISFELQVRRAGWVGLGVTNRYTNVGSDLVVGGVLPKGNVYFSDQHL
VDEDTLKEDGSQDTELLGLTEDAVYTTMRFSRPFRSCDPHDLDIMSDTVRVLAAYGLDDTPKLDQERTFVKSIFLLQVVHPDNLDVPEDTIIHDLEITDF
LIPEDDTTYACTFLPLPIVSQKHHIYKFEPKLVYHNETMVHHILVYACGNASVLPTGISDCYGADPAFSLCSQVIVGWAVRGTSYQFPDDVGVSIGTPLD
PQWIRLEIHYSNFNNLPGVYDSSGIHVYYTSQLRKYDMGVLQLGFLTFPIHFIPPGAESFMSCGLCRTEKFEEMNGAPMPDIQVYGYLLHTHFAGRALQA
V*YRNGTQLRTICKDDSYDFNLQETRDLPSRVEIKLGDGLLVECHYQTLDCDSMTFGGPSTINEMCLIFLFYYPRNNISSCMGYADIIYVAHELGEEASD
PMEGMMDMNDVKWTLESIKKAEKACKEAQQTVIIKTIDEVVENTTGWIPDIIPAPRGPCLESSGGKVEAQDKTPTGFRAAPVALSSSSRATLRHLPLVAI
LLLQHTLFWLLAMLQNGV

(1) Exon coordinates

Exon	UCSC Chromosome	Strand	Start	End
1	ponAbe2 chr7	-	139565936	139566223
2	ponAbe2 chr7	-	139565145	139565291
3	ponAbe2 chr7	-	139564448	139564609
4	ponAbe2 chr7	-	139558382	139558465
5	ponAbe2 chr7	-	139563115	139563282
6	ponAbe2 chr7	-	139557084	139557186
7	ponAbe2 chr7	-	139556725	139556891
8	ponAbe2 chr7	-	139562020	139562111
9	ponAbe2 chr7	-	139561639	139561735
10	ponAbe2 chr7	-	139561445	139561504
11	ponAbe2 chr7	-	139555099	139555229
12	ponAbe2 chr7	-	139554192	139554306
13	ponAbe2 chr7	-	139553658	139553900

6. Bornean orangutan (*Pongo pygmaeus*)

```
>Pongo_pygmaeus No=6 length=1857 name="Bornean orangutan"
ATGGCCCATGACCTTCTCTTCAGGCTTTTTCCACTTTTGGCCCTGGGAGCCCCCTTACAAAGCAACCGCCTTGGCCCCACATCTCGCCTGTGCTATTCCA
GGTTCCTAGATCCTTCTAATGTCATTTTCCTGCGTTGGGACTTTGACCTTGAGGCTGAAATCATCAGTTTTGAGCTCCAGGTCCGGAGAGCTGGCTGGGT
GGGCTTGGGTGTCACAAATCGCTACACCAACGTGGGAAGTGACCTGGTCGTTGGAGGAGTCTTGCCTAAAGGCAATGTCTATTTCTCGGATCAGCACTTG
GTGGACGAAGACACTCTGAAGGAGGATGGGAGCCAGGATGCTGAGCTGCTGGGGCTGACGGAAGATGCTGTTTACACCACCATGCGTTTTTCCAGGCCCT
TCCGCTCCTGCGATCCTCATGACCTGGATATTATGAGCGACACTGTGAGGGTGCTGGCTGCCTATGGCCTGGATGACACTCCGAAGCTGGATCAGGAGCG
TACTTTTGTCAAGTCTATCTTTCTGCTACAAGTCGTCCACCCTGACAATCTGGATGTCCCTGAGGACACCATCATCCATGACTTGGAGATCACTGATTTC
CTCATTCCAGAGGATGACACCACGTATGCCTGCACCTTTCTTCCTCTCCCTATTGTGAGCCAGAAGCATCACATTTACAAGTTTGAGCCTAAGTTGGTCT
ACCACAATGAGACAATGGTGCATCACATCCTGGTGTACGCCTGTGGCAATGCTAGCGTTCTCCCCACAGGCATCAGCGACTGCTATGGGGCCGACCCCGC
CTTCTCCCTCTGCTCACAGGTCATCGTGGGCTGGGCTGTCGGGGGCACTAGCTACCAGTTTCCAGATGACGTAGGCGTCTCTATTGGGACCCCCTTAGAC
CCTCAGTGGATCCGACTGGAGATTCATTACAGCAATTTTAACAACCTTCCTGGTGTGTATGATTCCTCAGGGATTCACGTGTACTACACTTCTCAGCTGC
GCAAATATGACATGGGCGTCCTCCAGCTGGGCTTCCTCACGTTTCCCATCCACTTCATCCCCCCGGGCGCTGAGTCCTTCATGTCCTGTGGGCTGTGTAG
GACGGAGAAGTTTGAGGAGATGAATGGAGCTCCTATGCCTGACATACAGGTATATGGCTACCTGCTGCACACCCACTTTGCTGGACGGGCTCTGCAAGCA
GTGTAATACAGGAATGGAACACAACTTCGAACAATCTGTAAAGACGATTCCTACGACTTCAACCTGCAGGAGACTCGAGATTTGCCCTCTCGAGTGGAGA
TCAAGCTGGGAGATGGATTGCTGGTAGAATGTCACTACCAGACACTGGACTGTGACTCCATGACATTTGGAGGCCCCAGCACCATTAATGAGATGTGCCT
CATCTTCCTCTTCTACTATCCCCGAAATAACATCTCTAGCTGCATGGGGTACGCTGACATCATCTACGTGGCCCACGAGCTGGGGGAGGAGGCATCAGAC
CCCATGGAGGGAATGATGGACATGAATGATGTCAAGTGGACCCTGGAGAGCATTAAGAAAGCTGAGAAAGCCTGCAAGGAGGCCCAGCAGACAGTGATAA
TAAAGACCATTGATGAGGTGGTGGAAAACACAACAGGCTGGATTCCAGACATCATCCCTGCTCCCCGGGGGCCCTGCTTGGAGTCATCTGGAGGCAAAGT
GGAGGCCCAGGACAAGACCCCTACAGGCTTCAGAGCTGCACCTGTGGCCCTCTCGAGCTCTAGCAGGGCCACCCTGAGGCATCTCCCCCTGGCGGCCATC
CTGCTGCTGCAGCACACACTCTTCTGGCTCCTGGCCATGCTGCAGAATGGAGTCTGA

>Pongo_pygmaeus No=6 length=618 name="Bornean orangutan"
MAHDLLFRLFPLLALGAPLQSNRLGPTSRLCYSRFLDPSNVIFLRWDFDLEAEIISFELQVRRAGWVGLGVTNRYTNVGSDLVVGGVLPKGNVYFSDQHL
VDEDTLKEDGSQDAELLGLTEDAVYTTMRFSRPFRSCDPHDLDIMSDTVRVLAAYGLDDTPKLDQERTFVKSIFLLQVVHPDNLDVPEDTIIHDLEITDF
LIPEDDTTYACTFLPLPIVSQKHHIYKFEPKLVYHNETMVHHILVYACGNASVLPTGISDCYGADPAFSLCSQVIVGWAVGGTSYQFPDDVGVSIGTPLD
PQWIRLEIHYSNFNNLPGVYDSSGIHVYYTSQLRKYDMGVLQLGFLTFPIHFIPPGAESFMSCGLCRTEKFEEMNGAPMPDIQVYGYLLHTHFAGRALQA
V*YRNGTQLRTICKDDSYDFNLQETRDLPSRVEIKLGDGLLVECHYQTLDCDSMTFGGPSTINEMCLIFLFYYPRNNISSCMGYADIIYVAHELGEEASD
PMEGMMDMNDVKWTLESIKKAEKACKEAQQTVIIKTIDEVVENTTGWIPDIIPAPRGPCLESSGGKVEAQDKTPTGFRAAPVALSSSSRATLRHLPLAAI
LLLQHTLFWLLAMLQNGV
```

(1) The exon 1 coding sequence was predicted by assembling WGS data using CAP3. The exon 1 is marked in red.

```
                          .    :    .    :    .    :    .    :    .    :    .    :
SRR033341.4097076.1+  GGAGATGCTCCAGGTCCCAGGAGCCATGGCCCATGACCTTCTCTTCAGGC
SRR033514.6327413.1+  GGAGATGCTCCAGGTCCCAGGAGCCATGGCCCATGACCTTCTCTTCAGGC
SRR033487.1248230-          GCTCCAGGTCCCAGGAGCCATGGCCCATGACCTTCTCTTCAGGCTTTTTC
SRR033338.1911109.1+          TCCACGTCCCAGGAGCCATGGCCCATGACCTTCTCTTCAGGCTTTTTCCA
SRR033156.5124902.1+            CAGGTCCCAGGAGCCATGGCCCATGACCTTCTCTTCAGGCTTTTTCCACT
SRR033110.5871114.2+                TCCCAGGAGCCATGGCCCATGACCTTCTCTTCAGGCTTTTTCCACT
SRR033352.2977969.1+                TCCCAGGAGCCATGGCCCATGACCTTCTCTTCAGGCTTTTTCCACT
SRR033115.1052769.1-                         CTATCGCCCATGACCTTCTCTTCAGGCTTTTTCCACT
SRR033351.4119436.2-                          CATGGCCCATGACCTTCTCTTCAGGCTTTTTCCACT
SRR033115.8996436.2+                                               CAGGCTTTTTCCACA
SRR033340.1043939.2-                               CCCATGACCTTCTCTTCAGGCTTTTTCCACT
SRR033341.9960516.2-                               CGCATGACCTTCTCTTCAGGCTTTTTCCACT
SRR033517.7525688.2+                           ATGGCCCATGACCTTCTCCTCAGGCTTTTTCCACT
SRR033109.2053350.1+                                CCATGACCTTCTCTTCAGGCTTTTTCCACT
SRR033115.1092679.1-                               CCCATGACCTTCTCTTCAGGCTTTTTCCACT
SRR033359.4172970.1+                               CCCATGACCTTCTCTTCAGGCTTTTTCCACT
SRR033510.556820.2+                                                     TTTTTCCACT
                      ____________________________________________________________
consensus             GGAGATGCTCCAGGTCCCAGGAGCCATGGCCCATGACCTTCTCTTCAGGCTTTTTCCACT

                          .    :    .    :    .    :    .    :    .    :    .    :
SRR033110.5871114.2+  TTTG
SRR033352.2977969.1+  TTTG
SRR033115.1052769.1-  TTTGGCCCTGGGA
SRR033351.4119436.2-  TTTGGCCCTGGGAG
SRR033112.7300222.2-                          GCCAGCCCGCCTTGGCCCCACATCTCGCCTGTGCTA
SRR033137.381746.2+                                                   TCGCCTGTGCTA
SRR033155.966300.2-                                                   GCGCCGGGGCTA
SRR033114.6493945.1+                                                TCTCGCCTGTGCTA
SRR033115.8996436.2+  TTTGGCCCTGGGAGCACCCTTACAAAGCAACCGCC
SRR033340.9038819.1+                            GCAACCGCCTTGGCCCCACATCTCGCCTGTGCTC
SRR033350.9726310.1+                                                  TCGCCTGTGCTA
SRR033134.278946.1+                                                            CNA
SRR033340.1043939.2-  TTTGGCCCTGGGAGCCCCA
SRR033341.9960516.2-  TTTGGCCCTGGGAGCCCCA
SRR033517.7525688.2+  TTTGGCCCTGGGAGC
SRR033517.7525688.1-                  CCCTTACAAAGCAACCGCCTTGGCCCCACATCTCGCCTGTGCTA
SRR033528.7201572-      TGGCCCTGGGACCCCCCTTACAAAGCAACCGCCTTGGCCCCACATCTCGC
SRR033532.8930224+       GGCCCTGGGAGCCCCCTTACAAAGCAACCGCCTTGGCCCCACATCTCGCA
SRR033538.7857308.1-       CCCTGGGAGCCCCCTTACAAAGCACCCGCCTTGGCCCCACATCTCGCCTG
```

```
SRR033538.10125900.1-                     TAAAAAGCAACCGCCTTGGCCCCACATCTCGCCTGTGCTA
SRR033109.2053350.1+  TTTGGCCCTGGGAGCCCCCT
SRR033155.2018688.2+                                                             A
SRR033349.4195183.1+                                                            TA
SRR033350.2834888.1-                                                          GCTA
SRR033115.1092679.1-  TTTGGCCCTGGGAGCCCCC
SRR033359.4172970.1+  TTTGGCCCTGGGAGCCCCC
SRR033510.556820.2+   TTTGGCCCTGGGAGCCCCCTTACAAAGCAACCGCCTTGGC
SRR033338.1911109.2-                                                     CCTGTGCTA
SRR033511.5366420.1+                                                     CCTGTGCTA
SRR033527.2854855-                                               ACATCTCGCCTGTGCTA
SRR033526.1669461+                    CCCTTACAAAGCAACCGCCTTGGCCCCACATCTCGCCTGTGCTA
SRR033528.6304531+                    CCCTTACAAAGCAACCGCCTTGGCCCCACATCTCGCCTGTGCTA
SRR033533.2063590.1+                                               CCACATCTCGCCTGTGCTA
SRR033538.7803773.1-                                                  CATCTCGCCTGTGCTA
                      ____________________________________________________________
consensus             TTTGGCCCTGGGAGCCCCCTTACAAAGCAACCGCCTTGGCCCCACATCTCGCCTGTGCTA

                          .    :    .    :    .    :    .    :    .    :    .    :
SRR033112.7300222.2-  TTCCAGGTTCCTAG
SRR033137.381746.2+   TTCCAGGTTCCTAGATCCTCCTAATGTCATTTTCCCTC
SRR033155.966300.2-   TTCCAGGTTCCTAGATCCTTCTAATGTCATTTTCCTGC
SRR033114.6493945.1+  TTCCAGGTTCCTAGATCCTTCTAATGTCATTCTACT
SRR033340.9038819.1+  TTCCCGGTTCCTAGAT
SRR033350.9726310.1+  TTACAGGTTCCTAGATACTTCTAATGTCATTTTCCTGC
SRR033484.7395923+                AGATCCTTCTAATGTCATTTTCCTGCGTTGGGAATTTGACCCTGAGGC
SRR033358.5621785.2+                                                 GACTTTGACATTGAGGC
SRR033109.2053350.2-                     TCTAATGTCATTTTCGTGCGTTGGGACTTTGACCTTGAGGC
SRR033134.278946.1+   TTCCAGGTTCCTAGATCCTTCTAATGTCATTTTCCTGCGTTGGGACT
SRR033155.1331093.1+        GTTCCTAGATCCTTCTAATGTCATTTTCCTGCGTTGGGAATTTGACCTTG
SRR033347.7824912.2+                    TTCTAATGTCATTTTCCTGCGTTGGGACTTTGACCTTGAGGA
SRR033512.7569778.1+                           GTCATTTTCCTGCGTTGGGACTCTGACCTTGAGGC
SRR033517.7525688.1-  TACCAG
SRR033538.10125900.1- TTCCAGGTTC
SRR033110.5871114.1-                     TCTAATGTCATTTTCCTGCGTTGGGACTTTGACCTTGAGGC
SRR033113.5122780.1+    CCAGGTTCCTAGATCCTTCTAATGTCATTTTCCTGCGTTGGGACTTTGAC
SRR033115.8996436.1-                                   CCTGCGTTGGGACTTTGACCTTGAGGC
SRR033135.5653904.1-                                                     CCTTGAGGC
SRR033155.2018688.2+  TTCCAGGTTCCTAGATCCTTCTAATGTCATTTTCCTGCGTTGGGACTTT
SRR033338.8970329.1+                                                           GGC
SRR033339.3052085.2+                                                    ACCTTGAGGC
SRR033341.4097076.2-   TCCAGGTTCCTAGATCCTTCTAATGTCATTTTCCTGCGTTGGGACTTTGA
SRR033344.1125701.1+               GATCCTTCTAATGTCATTTTCCTGCGTTGGGACTTTGACCTTGAGGC
SRR033349.3220930.2+                    TTCTAATGTCATTTTCCTGCGTTGGGACTTTGACCTTGAGGC
SRR033349.4195183.1+  TTCCAGGTTCCTAGATCCTTCTAATGTCATTTTCCTGCGTTGGGACTT
SRR033349.6970579.2+                                                TTTGACCTTGAGGC
SRR033350.2834888.1-  TTCCAGGTTCCTAGATCCTTCTAATGTCATTTTCCTGCGTTGGGAC
SRR033350.4128249.1+                                                         GAGGC
SRR033358.1926116.1+                              ATTTTCCTGCGTTGGGACTTTGACCTTGAGGC
SRR033113.2574268.1+                                          TGGGACTTTGACCTTGAGGC
SRR033510.556820.1-                                           TGGGACTTTGACCTTGAGGC
SRR033338.1911109.2-  TTCCAGGTTCCTAGATCCTTCTAATGTCATTTTCCTGCGTT
SRR033511.5366420.1+  TTCCAGGTTCCTAGATCCTTCTAATGTCATTTTCCTGCGTT
SRR033359.4172970.2-                                    CTGCGTTGGGACTTTGACCTTGAGGC
SRR033512.1022588.1+                                    CTGCGTTGGGACTTTGACCTTGAGGC
SRR033116.7379009.2+           CCTAGATCCTTCTAATGTCATTTTCCTGCGTTGGGACTTTGACCTTGAGG
SRR033514.6327413.2-           CCTAGATCCTTCTAATGTCATTTTCCTGCGTTGGGACTTTGACCTTGAGG
SRR033515.5116070.1-     CAGGTTCCTAGATCCTTCTAATGTCATTTTCCTGCGTTGGGACTTTGACC
SRR033527.2854855-    TTCCAGGTTCCTAGATCCTTCTAATGTCATTTT
SRR033527.12259118+                                              GACTTTGACCTTGAGGC
SRR033526.1669461+    TTCCAG
SRR033528.6304531+    TTCCAG
SRR033533.2063590.1+  TTCCAGGTTCCTAGATCCTTCTAATGTCATT
SRR033491.7661816-                                                   TTGACCTTGAGGC
SRR033156.5124902.2-                                                 TTGACCTTGAGGC
SRR033533.6811769.1-                                                 TTGACCTTGAGGC
SRR033517.7574857.1-  TTCCAGGTTCCTAGATCCTTCTAATGTCATTTTCCTGCGTTGGGACTTTG
SRR033535.1261803.1+  TTCCAGGTTCCTAGATCCTTCTAATGTCATTTTCCTGCGTTGGGACTTTG
SRR033535.7440993.1+                                        GTTGGGACTTTGACCTTGAGGC
SRR033536.6555872.1+      AGGTTCCTAGATCCTTCTAATGTCATTTTCCTGCGTTGGGACTTTGACCT
SRR033538.7803773.1-  TTCCAGGTTCCTAGATCCTTCTAATGTCATTTTC
                      ____________________________________________________________
consensus             TTCCAGGTTCCTAGATCCTTCTAATGTCATTTTCCTGCGTTGGGACTTTGACCTTGAGGC

                          .    :    .    :    .    :    .    :    .    :    .    :
SRR033484.7395923+    TG
SRR033515.1561757.1+                                                          TCAC
```

```
SRR033358.5621785.2+  TGAAATCATCAGTTTTGAGCTCCAGGTCCGGAC
SRR033109.2053350.2-  TGAAATCAT
SRR033137.4606557.1+    AAATCATCAGTTTTGAGCTCCAGGTCCGGAGAGCTGGCTGGGGGGGCTTG
SRR033155.1331093.2-                                                      GGTGTCAC
SRR033347.7824912.2+  TGAAATCA
SRR033512.7569778.1+  TGAAATCATCAGTTT
SRR033536.4505778.1+                                       GGCTGGGTGGGCTTGGGTGTCAC
SRR032969.6603817-                                                 GGGCTTGGGTGTCAC
SRR033110.5871114.1-  TGAAATCAT
SRR033115.8996436.1-  TGAAATCATCAGTTTTGAGCTCC
SRR033135.5653904.1-  TGAAATCATCAGTTTTGAGCTCCAGGTCCGGAGAGCTGGCT
SRR033156.3775127.2+                               GGAGAGCTGGCTGGGTGGGCTTGGGTGTCAC
SRR033338.8970329.1+  TGAAATCATCAGTTTTGAGCTCCAGGTCCGGAGAGCTGGCTGGGTGG
SRR033339.3052085.2+  TGAAATCATCAGTTTTGAGCTCCAGGTCCGGAGAGCTGGC
SRR033116.7379009.1-                                                 GCTTGGGTGTCAC
SRR032967.3164840+                                               GCTTGGGTGTCAC
SRR033339.8253321.1+                                                 GCTTGGGTGTCAC
SRR033113.5122780.2-                         AGGTCCGGAGAGCTGGCTGGGTGGGCTTGGGTGTCAC
SRR033340.9038819.2-                         AGGTCCGGAGAGCTGGCTGGGTGGGCTTGGGTGTCAC
SRR033344.1125701.1+  TGA
SRR033349.3220930.2+  TGAAATCA
SRR033349.6426565.2+                                          TGGGTGGGCTTGGGTGTCAC
SRR033349.6970579.2+  TGAAATCATCAGTTTTGAGCTCCAGGTCCGGAGAGC
SRR033350.4128249.1+  TGAAATCATCAGTTTTGAGCTCCAGGTCCGGAGAGCTGGCTGGGT
SRR033350.10066521.1+                             CGGAGAGCTGGCTGGGTGGGCTTGGGTGTCAC
SRR033358.1926116.1+  TGAAATCATCAGTTTTGA
SRR033359.8561460.2+                  GAGCTCCAGGTCCGGAGAGCTGGCTGGGTGGGCTTGGGTGTCAC
SRR033485.10308562-                 TTGAGCTCCAGGTCCGGAGAGCTGGCTGGGTGGGCTTGGGTGTCAC
SRR033113.2574268.1+  TGAAATCATCAGTTTTGAGCTCCAGGTCCG
SRR033510.556820.1-   TGAAATCATCAGTTTTGAGCTCCAGGTCCG
SRR033359.4172970.2-  TGAAATCATCAGTTTTGAGCTCCA
SRR033512.1022588.1+  TGAAATCATCAGTTTTGAGCTCCA
SRR033345.9005432.2+                                                        TGTCAC
SRR033512.7569778.2-                                                        TGTCAC
SRR033490.4215374-         TCATCAGTTTTGAGCTCCAGGTCCGGAGAGCTGGCTGGGTGGGCTTGGGT
SRR032966.7922748+         TCATCAGTTTTGAGCTCCAGGTCCGGAGAGCTGGCTGGGTGGGCTTGGGT
SRR032966.1353443-         TCATCAGTTTTGAGCTCCAGGTCCGGAGAGCTGGCTGGGTGGGCTTGGGT
SRR033526.12793032-        TCATCAGTTTTGAGCTCCAGGTCCGGAGAGCTGGCTGGGTGGGCTTGGGT
SRR033527.12259118+   TGAAATCATCAGTTTTGAGCTCCAGGTCCGGAG
SRR033533.2063590.2-                                                GGCTTGGGTGTCAC
SRR033533.2434591.1+                                     CTGGCTGGGTGGGCTTGGGTGTCAC
SRR033491.7661816-    TGAAATCATCAGTTTTGAGCTCCAGGTCCGGAGAGCT
SRR033156.5124902.2-  TGAAATCATCAGTTTTGAGCTCCAGGTCCGGAGAGCT
SRR033533.6811769.1-  TGAAATCATCAGTTTTGAGCTCCAGGTCCGGAGAGCT
SRR033533.8706241.2+                                             GTGGGCTTGGGTGTCAC
SRR033535.7440993.1+  TGAAATCATCAGTTTTGAGCTCCAGGTC
SRR033536.6555872.2-                                                       GTGTCAC
                      ____________________________________________________________
consensus             TGAAATCATCAGTTTTGAGCTCCAGGTCCGGAGAGCTGGCTGGGTGGGCTTGGGTGTCAC

                          .    :    .    :    .    :    .    :    .    :    .    :
SRR033515.1561757.1+  AAATCGCTACACCAACGTGGGAAGGGACATGGTCGTTGGAGGAGTC
SRR032968.85846-              ACACCAACGTGGGAAGTGCCCTGGTCGTTGGAGGAGTCTTGCCTAAAGGC
SRR033155.1331093.2-  ACATCGCTACACCAACGTGGGAAGTGACCTGGTCGTTGGAGG
SRR033155.3733224.1+   AATCGATACACCAACGTGGGAAGTGACCTGGTCGTTGGAGGAGTCTTGCC
SRR033484.2372546-               CCAGCGTGGGAAGTGACCTGGTCGTTGGAGGAGTCTTGCCTAAAGGCAA
SRR033485.5595938+           TACACCAACGTGGGAAGTGACCTGGTCGCTGGAGGAGTCTTGCCTAAAGG
SRR033536.4505778.1+  AAATCGCTACACCAACGTGGGAAGGGA
SRR032969.6603817-    AAATCGCTACACCAACGTGGGAAGTGACCTGGTCG
SRR033111.7886340.1-    ATCGCTACACCAACGTGGGAAGTGACCTGGTCGTTGGAGGAGTCTTGCCT
SRR033155.2018688.1-               AACGTGGGAAGTGACCTGGTCGTTGGAGGAGTCTTGCCTAAAGGCAA
SRR033156.3775127.2+  AAATCGCTACACCAACGTG
SRR033137.9675043.1+         TACACCAACGTGGGAAGTGACCTGGTCGTTGGAGGAGTCTTGCCTAAAGG
SRR033134.278946.2-          TACACCAACGTGGGAAGTGACCTGGTCGTTGGAGGAGTCTTGCCTAAAGG
SRR032968.2001146-           TACACCAACGTGGGAAGTGACCTGGTCGTTGGAGGAGTCTTGCCTAAAGG
SRR033156.9449366.1+         TACACCAACGTGGGAAGTGACCTGGTCGTTGGAGGAGTCTTGCCTAAAGG
SRR033339.3491798.1+              CAACGTGGGAAGTGACCTGGTCGTTGGAGGAGTCTTGCCTAAAGGCAA
SRR033116.7379009.1-  AAATCGCTACACCAACGTGGGAAGTGACCTGGTCGTT
SRR032967.3164840+    AAATCGCTACACCAACGTGGGAAGTGACCTGGTCGTT
SRR033339.8253321.1+  AAATCGCTACACCAACGTGGGAAGTGACCTGGTCGTT
SRR033113.5122780.2-  AAATCGCTACACC
SRR033340.9038819.2-  AAATCGCTACACC
SRR033344.1125701.2-                  GTGGGAAGTGACCTGGTCGTTGGAGGAGTCTTGCCTAAAGGCAA
SRR033349.3220930.1-                  GTGGGAAGTGACCTGGTCGTTGGAGGAGTCTTGCCTAAAGGCAA
SRR033347.7824912.1-                         GTGACCTGGTCGTTGGAGGAGTCTTGCCTAAAGGCAA
SRR033349.4195183.2-                         GTGACCTGGTCGTTGGAGGAGTCTTGCCTAAAGGCAA
SRR033349.6426565.2+  AAATCGCTACACCAACGTGGGAAGTGACCT
```

```
SRR033350.7482197.2+             CCAACGTGGGAAGTGACCTGGTCGTTGGAGGAGTCTTGCCTAAAGGCAA
SRR033350.9726310.2-                       AAGTGACCTGGTCGTTGGAGGAGTCTTGCCTAAAGGCAA
SRR033350.10066521.1+ AAATCGCTACACCAACGT
SRR033359.8561460.2+  AAATCG
SRR033485.10308562-   AAAT
SRR033511.5366420.2-                    GGGAAGTGACCTGGTCGTTGGAGGAGTCTTGCCTAAAGGCAA
SRR033345.9005432.2+  AAATCGCTACACCAACGTGGGAAGTGACCTGGTCGTTGGAGGAG
SRR033512.7569778.2-  AAATCGCTACACCAACGTGGGAAGTGACCTGGTCGTTGGAGGAG
SRR033533.2063590.2-  AAATCGCTACACCAACGTGGGAAGTGACCTGGTCGT
SRR033533.2434591.1+  AAATCGCTACACCAACGTGGGAAGT
SRR033533.8706241.2+  AAATCGCTACACCAACGTGGGAAGTGACCTGGT
SRR033536.6555872.2-  AAATCGCTACACCAACGTGGGAAGTGACCTGGTCGTTGGAGGA
SRR033489.7569165-                              TGACCTGGTCGTTGGAGGAGTCTTGCCTAAAGGCAA
SRR033338.8970329.2-                                 TGGTCGTTGGAGGAGTCTTGCCTAAAGGCAA
SRR033535.7470118.1+                                  GGTCGTTGGAGGAGTCTTGCCTAAAGGCAA
SRR033349.6970579.1-                                   GTCGTTGGAGGAGTCTTGCCTAAAGGCAA
SRR033535.1261803.2-                                   GTCGTTGGAGGAGTCTTGCCTAAAGGCGA
SRR033484.380397-                                      GTCGTTGGAGGAGTCTTGCCTAAAGGCAA
SRR033112.7006347.2+                                    TCGTTGGAGGAGTCTTGCCTAAAGGCAA
SRR033358.1926116.2-                                    TCGTTGGAGGAGTCTTGCCTAAAGGCAA
SRR033134.2599811.2+                                     CGTTGGAGGAGTCTTGCCTAAAGGCAA
SRR033345.6402372.1+                                      GTTGGAGGAGTCTTGCCTAAAGGCAA
SRR033529.8040415-                                        GTTGGAGGAGTCTTGCCTAAAGGCAA
SRR033491.5884736+                                          TGGAGGAGTCTTGCCTAAAGGCAA
SRR033531.3550702-                                            GAGGAGTCTTGTCTAAAGGCAA
SRR033113.2574268.2-                                             GAGTCTTGCCTAAAGGCAA
SRR033512.1022588.2-                                                 CTTGCCTAAAGGCAA
SRR033350.4128249.2-                                                   TGCCTAAAGGCAA
SRR033516.4141767.1+                                                   TGCCTAAAGGCAA
SRR033535.7440993.2-                                                   TGCCTAAAGGCAA
SRR032967.6659772+                                                       CCTAAAGGCAA
SRR033534.10524384.1+                                                        AAGGCAA
                      ____________________________________________________________
consensus             AAATCGCTACACCAACGTGGGAAGTGACCTGGTCGTTGGAGGAGTCTTGCCTAAAGGCAA

                          .    :    .    :    .    :    .    :    .    :    .    :
SRR033484.2372546-    T
SRR033155.2018688.1-  TGT
SRR033339.3491798.1+  TG
SRR033344.1125701.2-  TGTCTA
SRR033349.3220930.1-  TGTCTA
SRR033347.7824912.1-  TGTCTATTTCTCG
SRR033349.4195183.2-  TGTCTATTTCTCG
SRR033350.7482197.2+  T
SRR033350.9726310.2-  TGTCTATTTCT
SRR033511.5366420.2-  TGTCTATT
SRR033489.7569165-    TGTCTATTTCTCGG
SRR033338.8970329.2-  TGTCTATTTCTCGGTGTGA
SRR033535.7470118.1+  TGTCTATTTCTCGGTGTGAG
SRR033349.6970579.1-  TGTCTATTTCTCGGTGTGAGG
SRR033535.1261803.2-  TGTCTATTTCTCGGTGTGAGG
SRR033484.380397-     TGTCTATTTCTCGGTGTGAGG
SRR033112.7006347.2+  TGTCTATTTCTCGGTCTCGGGG
SRR033358.1926116.2-  TGTCTATTTCTCGGTGTGAGGG
SRR033134.2599811.2+  TGTCTATTTCTCGGTGTGACGGG
SRR033345.6402372.1+  TGTCTATTTCTCGGTGTGAGGGTG
SRR033529.8040415-    TGTCTATTTCTCGGTGTGAGGGTG
SRR033491.5884736+    TGTCTATTTCTCGGTGTGAGGGTGAC
SRR033531.3550702-    TGTCTATTTCTCGGTGTGAGGGTGGCTT
SRR033113.2574268.2-  TGTCTATTTCTCGGTGTGAGGGTGACTTGCT
SRR033512.1022588.2-  TGTCTATTTCTCGGTGTGAGGGTGACTTGCTTCCA
SRR033350.4128249.2-  TGTCTATTTCTCGGTGTGAGGGTGACTTGCTTCCAAG
SRR033516.4141767.1+  TGTCTATTTCTCGGTGTGAGGGTGACTTGCTTCCAAG
SRR033535.7440993.2-  TGTCTATTTCTCGGTGTGAGGGTGACTTGCTTCCAAG
SRR032967.6659772+    TGTCTATTTCTCGGTGTGAGGGTGACTTGCTTCCAAGGA
SRR033534.10524384.1+ TGTCTATTTCTCGGTGTGAGGGTGACTTGCTTCCAAGGATGAG
                      ____________________________________________________________
consensus             TGTCTATTTCTCGGTGTGAGGGTGACTTGCTTCCAAGGATGAG
```

(2) The exon 2 sequence was predicted by assembling WGS data using CAP3. The exon 2 is on the reverse strand and marked in red.

```
                          .    :    .    :    .    :    .    :    .    :    .    :
SRR033115.3599691.1-  ATGCTTTTCTCTTGTCGCTATTTTACCATAATATCCAGGTCATGAGGATC
SRR033540.7765971.1-  ATGCTTTTCTCTGCTCGCTATTTTACCATAATATCCAGGTCATGAGGATC
SRR033358.13784.2+      GCTTTTCTCTGCTCGCTATTTTACCATAATATCCAGGTAATGAGGATCGC
```

```
SRR033531.9224986+          TTCTCTGCTCGCTATTTTACCATAATATCCAGGTCATGAGGATCGCAAGA
SRR033530.5544236-                 CTCGCTATTTTACCATAATATCCAGGTCATGAGGATCGCAAGAGCGG
SRR033353.5328003.2-                CTGCTATTTTACCATAATATCCAGGTCATGAGGATCGCAGGAGCGG
SRR033110.954882.1+                    CTATTTTACCATAATATCCAGGTCATGAGGATCGCAGGAGCGG
SRR033341.3674044.2+                    TATTTTACCATAATATCCAGGTCATGAGGATCGCAGGAGCGG
SRR032965.6618589-                       ATTTTACCATAATATCCAGGTCATGAGGATCGCAAGAGCGG
SRR033529.7480472+                       ATTTTACCATAATATCCAGGTCATGAGGATCGCAAGAGCGG
SRR033515.10025144.1-                      TTTACCATAATATCCAGGTCATGAGGATCGCAGGAGCGG
SRR033540.5126956.2+                                   TCCAGGTCATGAGGATCGGAAGAGCGG
SRR033537.3594976.2+                                 TATCCAGGTCATGAGGATCGCAAGAGCGG
SRR032968.569530+                                         AGGTCATGAGGATCGCAGGAGCGG
SRR033338.338424.2-                                                            CGG
SRR033360.8633158.1-                        CATAATATCCAGGTCATGAGGATCGCAGGAGCGG
SRR033531.5562422+                                                GATCGCAGGAGCGG
                      ____________________________________________________________
consensus             ATGCTTTTCTCTGCTCGCTATTTTACCATAATATCCAGGTCATGAGGATCGCAGGAGCGG

                          .    :    .    :    .    :    .    :    .    :    .    :
SRR033530.5544236-    AAG
SRR033353.5328003.2-  AAGG
SRR033110.954882.1+   AAGGGCC
SRR033341.3674044.2+  AAGGGACT
SRR032965.6618589-    AAGGGCCTG
SRR033529.7480472+    AAGGGCCTG
SRR033515.10025144.1- AAGGGCCTGGA
SRR033514.9952179.1-                                                GCCTCAACAGCTCA
SRR033540.5126956.2+  AAGGGCCTGGAAAAACGCAGGGT
SRR033114.7406358.2-          GTAACAACGCATGGTGGTGTAAACAGCATCTTCCGTCAGCCCCAGCACCT
SRR033341.9245912.2-                                                         CCTCA
SRR033155.1003897.1+                                        TTCCGTCAGCCCCAGCACCTCA
SRR033360.7030864.2+                                                       CAGCTCA
SRR033488.1612518+                        GTGTAAACAGCATCTTCCGTCAGCCCCAGCACCTCA
SRR033109.5859771.2+                      GTGTAAACAGCATCTTCCGTCAGCCCCAGCAGCTCA
SRR033136.6249566.2+                             CAGCATCTTCCGTCAGCCCCAGCACCTCA
SRR033155.1295242.1-                     GTGGTGTAAACAGCATCTTCCGTCAGCCCCAGCAGCTCA
SRR033340.8605939.2+                                        TTCCGTCAGCCCCAGCAGCTCA
SRR033341.6984613.2-                ACGCATGGTGGTGTAAACAGCATCTTCCGTCAGCCCCAGCAGCTCA
SRR033341.9245912.1+                                             TCAGCCCCAGCACCTCA
SRR033342.8369009.2+                            ACAGCATCTTCCGTCAGCCCCAGCAGCTCA
SRR033349.7682200.1-                  GCATGGTGGTGTAAACAGCATCTTCCGTCAGCCCCAGCACCTCA
SRR033514.2899932.1+                                                             A
SRR033535.1898759.1+                                                          CTCA
SRR033537.3594976.2+  AAGGGCCTGGAAAAACGCATG
SRR032968.569530+     AAGGGCCTGGAAAAACGCATGGTGGT
SRR033135.690493.1+                                                              A
SRR033135.9605374.2-   AGGGCCTGGAAAAACGCATGGTGGTGTAAACAGCATCTTCCGTCAGCCCC
SRR033156.9374248.2-  AAGGGCCTGGAAAAACGCATGGTGGTGTAAACAGCATCTTCCGTCAGCCC
SRR033338.338424.2-   AAGGGCCTGGAAAAACGCATGGTGGTGTAAACAGCATCTTCCGTCAG
SRR033338.1131672.2+          GGAAAAACGCATGGTGGTGTAAACAGCATCTTCCGTCAGCCCCAGCAGCT
SRR033360.8633158.1-  AAGGGCCTGGAAAAAC
SRR033531.5562422+    AAGGGCCTGGAAAAACGCATGGTGGTGTAAACAGCA
                      ____________________________________________________________
consensus             AAGGGCCTGGAAAAACGCATGGTGGTGTAAACAGCATCTTCCGTCAGCCCCAGCAGCTCA

                          .    :    .    :    .    :    .    :    .    :    .    :
SRR033514.9952179.1-  GTATCCTGGCTCCCATCCTCCTTCAGAGTGTCTTCG
SRR033341.9245912.2-  CCATCCTGGCTCCCATCCTCCTCCAGAGTGTCTTCGTCCACCAAG
SRR033155.1003897.1+  GCATCCTGGCTCCCATCCTCCTTCCGAG
SRR033360.7030864.2+  GTATCCTGGCTCCCATCCTCCTTCAGAGTGTCTTCGTCCATCA
SRR033488.1612518+    GCATCCTGGATCCC
SRR033109.5859771.2+  GTATCCTGGCTCCC
SRR033136.6249566.2+  GCATCCTGGCTCCCATCCTCC
SRR033155.1295242.1-  GTATCCTGGCT
SRR033340.8605939.2+  GTATCCTGGCTCCCATCCTCCTTCAGAG
SRR033341.6984613.2-  GTAT
SRR033341.9245912.1+  GCATCCTGGCTCCCATCCTCCTTCAGAGTGTCT
SRR033342.8369009.2+  GTATCCTGGCTCCCATCCTC
SRR033349.7682200.1-  GCATCC
SRR033514.2899932.1+  GTATCCTGGCTCCCATCCTCCTTCAGAGTGTCTTCGTCCACCAAGTGCT
SRR033535.1898759.1+  GCATCCTGGCTCCCATCCTCCTTCAGAGTGTCTTCGTCCACCCAGT
SRR033135.690493.1+   GCATCCTGGCTCCCATCCTCCTTCAGAGTGTCTTCGTCCACCAAGTGCT
SRR033349.4457911.1-       CTGGCTCCCATCCTCCTTCAGAGTGTCTTCGTCCACCAAGTGCTGATCCT
SRR033540.5126956.1-             CCCATCCTCCTTCAGAGTGTCTTCGTCCACCAAGTGCTGATCCTTGGGT
SRR033136.8058788.2+              CCATCCTCCTTCAGAGTGTCTGCGTCCACCAAGTGCTGATCCTTGGGT
SRR033110.954882.2-                  GCCTGCGTCAGAGTGTCTTCGTCCACCAAGTGCTGATCCTTGGGT
SRR033109.7983721.1-                  CCTCCTTCAGAGTGTCTTCGTCCACCAAGTGCTGATCCTTGGGT
SRR033358.13784.1-                       CCTTAAGAGTGTCTTCGTCCACCAAGTGCTGATCCTTGGGT
```

```
SRR033338.2799738.2-                      CTTCAGAGTGTCTTCGTCCACCAAGTGCTGATCCTTGGGT
SRR033531.7397620-                         TTCAGAGTGTCTTCGTCCACCAAGTGCTGATCCTTGGGT
SRR032967.50917+                              AGAGTGTCTTCGTCCACCAAGTGCTGATCCTTGGGT
SRR033516.5279905.2+                          AGAGTGTCTTCGTCCACCAAGTGCTGATCCTTGGGT
                      ____________________________________________________________
consensus             GCATCCTGGCTCCCATCCTCCTTCAGAGTGTCTTCGTCCACCAAGTGCTGATCCTTGGGT

                          .    :    .    :    .    :    .    :    .    :    .    :
SRR033540.5126956.1-  C
SRR033136.8058788.2+  CC
SRR033110.954882.2-   CCAGC
SRR033109.7983721.1-  CCAGTG
SRR033358.13784.1-    CCAGTGAAG
SRR033338.2799738.2-  CCAGTGAAGG
SRR033531.7397620-    CCAGTGAAGGT
SRR032967.50917+      CCAGTGAAGGTCAA
SRR033516.5279905.2+  CCAGTGCAGGTCCA
                      ____________________________________________________________
consensus             CCAGTGAAGGTCAA
```

(3) The exon 3 sequence was predicted by assembling WGS data using CAP3. The exon 3 is on the reverse strand and marked in red.

```
                          .    :    .    :    .    :    .    :    .    :    .    :
SRR033338.10268151.1- GGGTGTGACGAAGGTGTCTTATCAGTGATCTCCAAGTCATGGATGATGGT
SRR033343.378264.1+   GGGTGTGACGAAGGTGTCTTCTCAGTGATCTCCAAGTCATGGATGCTGGT
SRR033514.1410406.1+      GTGACGAAGGTGTCTTATCAGTGATCTCCAAGTCATGGATGATGGTGTCC
SRR032969.613307+          TGACGAAGGTGTCTTATCAGTGATCTCCAAGTCATGGATGATGGTGTCCT
SRR033342.2171269.2-         ACGAAGGTGTCTTATCAGTGGTCTCCACGTCATGGATGATGGTGTCCTCA
SRR033136.3968739.1+              GGTGTCTTATCAGTGATCTCCAAGTCATGGATGATGGTGTCCTCAGGG
SRR033347.4543389.2-              TGTCTTACATCAGTGATCTCCACGTCATGGATGATGGTGTCCTCAGGG
SRR033359.769400.1+                GTCTTACATCAGTGATCTCCAAGTCATGGATGATGGTGTCCTCAGGG
SRR033156.9422811.2-                 GTCTTATCAGTGATCTCCAAGTCATGGATGATGGTGTCCTCAGGG
SRR033510.2920004.2+                 CTTACATCAGTGATCTCCAAGTCATGGATGATGGTGTCCTCAGGG
SRR033510.3010884.1-                    TTATCAGTGATCTCCAAGTCATGGATGATGGTGTCCTCAGGG
SRR033347.8278657.2+                         AGTGATCCCCAAGTCATGGATGATGGTGTCCTCAGGG
SRR033340.10730744.1-                         GTGATCTCCAAGTCCTGGATGATGGTGTCCTCAGGG
SRR033360.8618374.1+                               CTCCAAGTCATGGATGATGGTGTCCTCAGGG
SRR033527.2125202+                                  TCCAAGTCATGGATGATGGTGTCCTCAGGG
SRR033539.3484660.1-                                     GTCATGGATGATGGTGTCCTCAGGG
SRR033344.7918302.2+                        CAGTGATCTCCAAGTCATGGATGATGGTGTCCTCAGGG
SRR033346.4442042.1-                                     GTCATGGATGATGGTGTCCTCAGGG
SRR033346.4442042.2+                                   AAGTCATGGATGATGGTGTCCTCAGGG
SRR033349.9555912.1+                                                       CTCAGGG
SRR033354.4521346.1+                                    AGTCATGGATGATGGTGTCCTCAGGG
SRR033359.4145594.2-                                             TGATGGTGTCCTCAGGG
SRR033361.5320487.2-                                          GGATGATGGTGTCCTCAGGG
SRR033529.8432170-                                                   TGTCCTCAGGG
                      ____________________________________________________________
consensus             GGGTGTGACGAAGGTGTCTTATCAGTGATCTCCAAGTCATGGATGATGGTGTCCTCAGGG

                          .    :    .    :    .    :    .    :    .    :    .    :
SRR033136.3968739.1+  AC
SRR033347.4543389.2-  AC
SRR033359.769400.1+   ACA
SRR033156.9422811.2-  ACATC
SRR033510.2920004.2+  ACATC
SRR033510.3010884.1-  ACATCCAG
SRR033136.4578603.1-                                                    AAACGTACGC
SRR033347.8278657.2+  CCATCCAGATTGT
SRR032968.7774472+                                                       AAAGTACGC
SRR033113.8225773.1+   CATCCAGATTGTCAGGGTGGACGACTTGTAGCAGAAAGATAGACCTGACA
SRR033338.3066963.2+                     GGACGACTTGTAGCAGAAATATAGACTTGACAAAAGTACGC
SRR033340.10730744.1- ACATCCAGATTGTC
SRR033343.378264.2-                                                       AAGTACGC
SRR033346.6437666.2+                                  CAGAAAGATAGACTTGACAAAAGTACGC
SRR033360.8618374.1+  ACATCCAGATTGTCGGGGT
SRR033342.8679123.2+                               TAGCAGAAAGATAGACTTGACAAAAGGACGC
SRR033527.2125202+    ACATCCAGATTGTCAGGGGG
SRR033538.3070815.1-                                            GACTTGACAAAAGTGCGC
SRR033539.3484660.1-  ACATCCAGATTGTCCGGGTGGACGA
SRR033110.1426432.1-                                         ATAGACTTGACAAAAGTACGC
SRR033156.6681769.2+                AGGGTGGACGACTTGTAGCAGAAAGATAGACTTGACAAAAGTACGC
SRR033110.5801150.1+        AGATTGTCAGGGTGGACGACTTGTAGCAGAAAGATAGACTTGACAAAAGT
SRR033344.7918302.1-        AGATTGTCAGGGTGGACGACTTGTAGCAGAAAGATAGACTTGACAAAAGT
SRR033344.7918302.2+  ACATCCAGATTG
```

```
SRR033346.4442042.1-  ACATCCAGATTGTCAGGGTGGACGA
SRR033346.4442042.2+  ACATCCAGATTGTCAGGGTGGAC
SRR033349.9555912.1+  ACATCCAGATTGTCAGGGTGGACGACTTGTAGCAGAAAGATAG
SRR033354.4521346.1+  ACATCCAGATTGTCAGGGTGGACG
SRR033359.4145594.2-  ACATCCAGATTGTCAGGGTGGACGACTTGTAGC
SRR033359.4757346.1+                      GACGACTTGTAGCAGAAAGATAGACTTGACAAAAGTACGC
SRR033360.1847302.2-                                                         TACGC
SRR033360.7437972.2-                                                         TACGC
SRR033361.5320487.2-  ACATCCAGATTGTCAGGGTGGACGACTTGT
SRR033490.6187174+                                                             CGC
SRR033512.2038924.1+                               TAGCAGAAAGATAGACTTGACAAAAGTACGC
SRR033489.10318049+              GTCAGGGTGGACGACTTGTAGCAGAAAGATAGACTTGACAAAAGTACGC
SRR033513.4879018.1-             GTCAGGGTGGACGACTTGTAGCAGAAAGATAGACTTGACAAAAGTACGC
SRR033513.5005059.1+                                   AGAAAGATAGACTTGACAAAAGTACGC
SRR033487.9916300+                     ACGACTTGTAGCAGAAAGATAGACTTGACAAAAGTACGC
SRR033514.949092.1+                    ACGACTTGTAGCAGAAAGATAGACTTGACAAAAGTACGC
SRR033529.8432170-    ACATCCAGATTGTCAGGGTGGACGACTTGTAGCAGAAAG
SRR033536.3527307.1-                     GACTTGTAGCAGAAAGATAGACTTGACAAAAGTACGC
SRR033538.1534595.1+                                                TGACAAAAGTACGC
                      ____________________________________________________________
consensus             ACATCCAGATTGTCAGGGTGGACGACTTGTAGCAGAAAGATAGACTTGACAAAAGTACGC

                          .    :    .    :    .    :    .    :    .    :    .    :
SRR033136.4578603.1-  GCCTGATCCAGCTTCGGAGTGTCATCCAGGCCATAGGCAG
SRR033514.4147285.1+  TCCTGATCCAGCTTCGGAGTGTCATCCAGGCCATAGGAAGCCAGCACCCC
SRR032968.7774472+    TCCTGATCCAGCTTCGGAGTGTCATCCAGGCCAGAGGCAGC
SRR033338.3066963.2+  TCCTGATCC
SRR033343.378264.2-   TCCTGATCCAGCTTGGGAGTGTCATCCAGGCCATAGGCAGCC
SRR033346.6437666.2+  TCCTGATCCAGCTTCGGAGGGT
SRR033341.8860934.2-        TCCGGCTTCGGAGTGTCATCCAGGCCATAGGCAGCCAGCACCCTCACAGT
SRR033342.8679123.2+  TCCTGATCCAGCTTCGGAG
SRR033538.3070815.1-  TCCTGATCCAGCTTCGGAGTGTCATCCAGGCC
SRR033110.1426432.1-  TCCTGATCCAGCTTCGGAGTGTCATCCAG
SRR033156.6681769.2+  TCCT
SRR033359.4757346.1+  TCCTGATCCA
SRR033360.1847302.2-  TCCTGATCCAGCTTCGGAGTGTCATCCAGGCCATAGGCAGCCAGC
SRR033360.7437972.2-  TCCTGATCCAGCTTCGGAGTGTCATCCAGGCCATAGGCAGCCAGC
SRR033490.6187174+    TCCTGATCCAGCTTCGGAGTGTCATCCAGGCCATAGGCAGCCAGCAC
SRR033511.356684.2+      TGATCCAGCTTCGGAGTGTCATCCAGGCCATAGGCAGCCAGCACCCTCAC
SRR033136.7291734.2-        TCCAGCTTCGGAGTGTCATCCAGGCCATAGGCAGCCAGCACCCTCACAGT
SRR033511.816557.1+         TCCAGCTTCGGAGTGTCATCCAGGCCATAGGCAGCCAGCACCCTCACAGT
SRR033512.2038924.1+  TCCTGATCCAGCTTCGGAG
SRR033489.10318049+   T
SRR033513.4879018.1-  T
SRR033513.5005059.1+  TCCTGATCCAGCTTCGGAGTGTC
SRR033487.9916300+    TCCTGATCCAG
SRR033514.949092.1+   TCCTGATCCAG
SRR033534.8597285.1-   CCTGATCCAGCTTCGGAGTGTCATCCAGGCCATAGGCAGCCAGCACCCTC
SRR033536.3527307.1-  TCCTGATCCAGCT
SRR033538.1534595.1+  TCCTGATCCAGCTTCGGAGTGTCATCCAGGCCATAG
SRR033538.10042109.1-              TCGGAGTGTCATCCAGGCCATAGGCAGCCAGCACCCTCACAGTGTCG
SRR033135.3914045.1-                   AGTGTCATCCAGGCCATAGGCAGCCAGCACCCTCACAGTGTCG
SRR033343.7851325.2-                   GGTGTCATCCAGGTCCTAGGCAGCCAGCACCCTCACAGTGTCG
SRR033341.3666715.2-                     TGTCATCCAGGCCATAGGCAGCCAGCACCCTCACAGTGTCG
SRR033514.1410406.2-                         ATCCAGGCCATAGGCAGCCAGCACCCTCACAGTGTCG
                      ____________________________________________________________
consensus             TCCTGATCCAGCTTCGGAGTGTCATCCAGGCCATAGGCAGCCAGCACCCTCACAGTGTCG

                          .    :    .    :    .    :    .    :    .    :    .    :
SRR033538.10042109.1- CTC
SRR033135.3914045.1-  CTCTGCT
SRR033343.7851325.2-  CTCTGCT
SRR033341.3666715.2-  CTCTGCGGG
SRR033514.1410406.2-  CTCTGCGGGGTGC
                      ____________________________________________________________
consensus             CTCTGCGGGGTGC
```

(4) The exon 4 sequence was predicted by assembling WGS data using CAP3. The exon 4 is marked in red.

```
                          .    :    .    :    .    :    .    :    .    :    .    :
SRR033150.8900463.2+  TCACTGTCATCTGGCTCAGCTTCTGTGCACCCTTCCACAGTTCCTCATTC
SRR033535.6669938.1-    ACTGTCATCTGGCTCAGCTTCTGTGCACCCTTCCACAGTTCCTCATTCCA
SRR033339.5776891.1+      TGTCATCTGGCTCAGCTTCTGTGCACCCTTCCACAGTTCCTCATTCCAGC
SRR033137.7789615.1-        TCATCTGGCTCAGCTTCTGTGCACCCTTCCACAGTTCCTCATTCCAGAGG
SRR033136.6695200.1-          ATCTGGCTCAGCTTCTGTGCACCCTTCCACAGTTCCTCATTCCAGAGGAT
SRR033517.2215208.2+          ATCTGGCTCAGCTTCTGTGCACCCTTCCACAGTTCCTCATTCCAGAGGAT
```

```
SRR033360.110651.1-            TCTGGCTCAGCTTCTGTGCACCCTTCCACAGTTCCTCATTCCAGAGGATG
SRR033535.2050565.2-            CTGGCTCAGCTTCTGTGCACCCTTCCACAGTTCCTCATTCCAGAGGATGC
SRR033511.2851973.1-              GGCTCAGCTTCTGTGCACCCTTCCACAGTTCCTCATTCCAGAGGATGA
SRR033339.3376377.1+                CTCAGCTTCTGTGCACCCTTCCACAGTTCCTCATTCCAGAGGATGA
SRR033349.6851093.2+                 TCAGCTTCTGTGCACCCTTCCACAGTTCCTCATTCCAGAGGATGA
SRR033155.572392.1+                     GCTTCTGTGCACCCTTCCACAGTTCCTCATTCCAGAGGATGA
SRR033517.6443008.1-                      TTCTGTGCACCCTTCCACAGTTCCTCATTCCAGAGGATGA
SRR033535.2372533.2-                        CTGTGCCCCCTTCCACAGTTCCTCATTCCAGAGGATGA
SRR033533.9643592.1+                            GCACCCTTCCACAGTTCCTCATTCCAGAGGATGA
SRR033516.6238262.1+                               CCCTTCCACAGTTCCTCATTCCAGAGGATGA
SRR033345.6787299.2+                                CCTTCCACAGTTCCTCATTCCAGAGGATGA
SRR033517.2215208.1-                                CCTTCCACAGTTCCTCATTCCAGAGGATGA
SRR033358.3998974.2+                                 CTTCCACAGTTCCTCATTCCAGAGGATGA
SRR033343.288119.1-                                    TCCACAGTTCCTCATTCCAGAGGATGA
SRR033536.5507264.2+                                     CACAGTTCCTCATTCCAGAGGATGA
SRR033510.4575165.2+                                                ATTCCAGAGGATGA
SRR033156.27501.1+                                                        GAGGATGA
SRR033350.6393621.1+                                                           TGA
SRR033510.6937995.2-                                                         GATGA
SRR033512.7472760.1-                                            ATTCCAGAGGATGA
SRR033513.2643024.1+                                       TCCTCATTCCAGAGGATGA
SRR033361.6152160.1-                                                       AGGATGA
SRR033513.7523906.1-                                                       AGGATGA
SRR033515.4891375.1-                                                   CCAGAGGATGA
SRR033515.9834465.1-                                              TCATTCCAGAGGATGA
                      ____________________________________________________________
consensus             TCACTGTCATCTGGCTCAGCTTCTGTGCACCCTTCCACAGTTCCTCATTCCAGAGGATGA

                          .    :    .    :    .    :    .    :    .    :    .    :
SRR033511.2851973.1-  CA
SRR033339.3376377.1+  CACC
SRR033349.6851093.2+  CACCA
SRR033155.572392.1+   CACCACGT
SRR033517.6443008.1-  CACCACGTAT
SRR033535.2372533.2-  CACCACGTATGC
SRR033533.9643592.1+  CACCACGTATGCCTGC
SRR033516.6238262.1+  CACCACGTATGCCTGCACC
SRR033345.6787299.2+  CACCACGTATGCCTGCACCT
SRR033517.2215208.1-  CACCACGTATGCCTGCACCT
SRR033358.3998974.2+  CACCATGTATGCCTGCACCTT
SRR033343.288119.1-   CACCACGTATGCCTGCACCTTTC
SRR033536.5507264.2+  CACCACGTATGCCTGCACCTGTCTT
SRR033539.2069673.2+        GTAAGACTGCACCTTTCTTCCTCTCCCTATTGGGAGCCAGAAGCATCACA
SRR033134.2376430.1+      ACGTATGCCTGCACCTTTCTTCCTCTCCCTATTGTGAGCCAGCAGCATCC
SRR033346.1485658.1+    CCACGTATGCCTGCTCCTTTCTTCCTCTCCCTATTGTGAGCCAGAAGCAT
SRR033360.5293225.2+             CCTGCACCTTTCTTCCTATCCCTATTGTGAGCCAGAAGCATCACATTTA
SRR033510.4575165.2+  CACCACGTATGCCTGCACCTTTCTTCATCTCCCTAT
SRR033485.5664640-                 TGCACCTTTCTTCCTCTCCCTATTGTGAGTCAGAAGCATCACATTTA
SRR033358.5832455.1-              CTGCACCTTTCTTCCTCTCCCTATTGTGAGCCAGAAGCATCACACTTA
SRR033156.27501.1+    CACCACGTATGCCTGCACCTTTCTTCCTCTCCCTATTGTGAG
SRR033350.6393621.1+  CACCACGTATGCCTGCACCTTTCTTCCTCTCCCTATTGTGAGCCAGA
SRR033485.8864684+               CCTGCACCTTTCTTCCTCTCCCTATTGTGAGCCAGAAGCATCACATTTA
SRR033510.6937995.2-  CACCACGTATGCCTGCACCTTTCTTCCTCTCCCTATTGTGAGCCA
SRR033512.7472760.1-  CACCACGTATGCCTGCACCTTTCTTCCTCTCCCTAT
SRR033513.2643024.1+  CACCACGTATGCCTGCACCTTTCTTCCTCTC
SRR033513.6498968.1-  CACCACGTATGCCTGCACCTTTCTTCCTCTCCCTATTGTGAGCCAGAAGC
SRR033361.6152160.1-  CACCACGTATGCCTGCACCTTTCTTCCTCTCCCTATTGTGAGC
SRR033513.7523906.1-  CACCACGTATGCCTGCACCTTTCTTCCTCTCCCTATTGTGAGC
SRR033513.7987093.2+               TGCACCTTTCTTCCTCTCCCTATTGTGAGCCAGAAGCATCACATTTA
SRR033515.4891375.1-  CACCACGTATGCCTGCACCTTTCTTCCTCTCCCTATTGT
SRR033515.9834465.1-  CACCACGTATGCCTGCACCTTTCTTCCTCTCCCT
SRR033532.5551810-                CTGCACCTTTCTTCCTCTCCCTATTGTGAGCCAGAAGCATCACATTTA
SRR033512.7089593.1+                 CACCTTTCTTCCTCTCCCTATTGTGAGCCAGAAGCATCACATTTA
SRR033511.8640195.2+                    CTTTCTTCCTCTCCCTATTGTGAGCCAGAAGCATCACATTTA
SRR033341.4941619.1+                        CTTCCTCTCCCTATTGTGAGCCAGAAGCATCACATTTA
SRR033338.9609169.2-                         TTCCTCTCCCTATTGTGAGCCAGAAGCATCACATTTA
SRR033512.8156694.1+                         TTCCTCTCCCTATTGTGAGCCAGAAGCATCACATTTA
SRR033510.4575165.1-                           CCTCTCCCTATTGTGAGCCAGAAGCATCACATTTA
SRR033347.223341.1+                             CTCTCCCTATTGTGAGCCAGAAGCATCACATTTA
SRR033536.3576861.1-                            CTCTCCCTATTGTGAGCCAGAAGCATCACATTTA
SRR033137.5579528.2+                                  CTATTGTGAGCCAGAAGCATCACATTTA
SRR033510.1232413.2+                                  CTATTGTGAGCCAGAAGCATCACATTTA
SRR033533.7672877.1+                                    ATTGTGAGCCAGAAGCATCACATTTA
SRR033137.6598278.1-                                     TTGTGAGCCAGAAGCATCACATTTA
SRR033358.1191401.1-                                     TTGTGAGCCAGAAGCATCACATTTA
SRR033484.2964814-                                       TTGTGAGCCAGAAGCATCACATTTA
SRR033136.9440099.1-                                         GAGCCAGAAGCATCACATTTA
```

```
SRR033512.7846369.2-                                         GAGCCAGAAGCATCACATTGA
                      ____________________________________________________________
consensus             CACCACGTATGCCTGCACCTTTCTTCCTCTCCCTATTGTGAGCCAGAAGCATCACATTTA

                          .    :    .    :    .    :    .    :    .    :    .    :
SRR033360.5293225.2+  C
SRR033485.5664640-    CAA
SRR033358.5832455.1-  CA
SRR033485.8864684+    C
SRR033513.7987093.2+  CAA
SRR033532.5551810-    CA
SRR033512.7089593.1+  CAAGG
SRR033511.8640195.2+  CACGGCAT
SRR033341.4941619.1+  CAAGGTATGGTG
SRR033338.9609169.2-  CAAGGTATGGTGG
SRR033512.8156694.1+  CAAGGTATGGTGG
SRR033510.4575165.1-  CAAGGTATGGTGGAC
SRR033347.223341.1+   CAAGGTATGGTGGACC
SRR033536.3576861.1-  CAAGGTATGGTGGACC
SRR033137.5579528.2+  CAAGGTATGGTGGACCAGATGG
SRR033510.1232413.2+  CAAGGTATGGTGGACCAGATGG
SRR033533.7672877.1+  CAAGGTATGGTGGACCAGATGGGG
SRR033137.6598278.1-  CAAGGTATGGTGGACCAGATGGGGG
SRR033358.1191401.1-  CAAGGTATGGTGGACCAGATGGGGG
SRR033484.2964814-    CAAGGTATGGTGGACCAGATGGGGG
SRR033136.9440099.1-  CAAGGTATGGTGGACCAGATGGGGGACAT
SRR033512.7846369.2-  CAAGGTATGGTGGACCAGATGGGGGACAT
                      ____________________________________________________________
consensus             CAAGGTATGGTGGACCAGATGGGGGACAT
```

(5) The exon 5 sequence was predicted by assembling WGS data using CAP3. The exon 5 is on the reverse strand and marked in red.

```
                          .    :    .    :    .    :    .    :    .    :    .    :
SRR033351.4777212.2+  CCCGCTTAGAAATGTGTTTACCCCATCATGGGACTCACAGTGCCCCCGAC
SRR033347.684775.2-     CGCTTCGAAATGTGTTTACCCCATCATGGGACTCACAGTGCCCCCGACAG
SRR033537.3644035.2+     GCTTAGAAATGTGTTTACCCCATCATGGGACTCACAGTGCCCCTGACAGC
SRR033538.2794141.1+     GCTTAGAAATGTGTTTACCCCATCATGGGACTCACAGTGCCCCTGACAGC
SRR033343.8028082.1-         AGAAATGTGTTTACCCCATCATGGGACTCACAGTGCCCCCGACAGCCCAG
SRR033346.363782.1-            AAATGTGTTTACCCCATCATGGGACTCACAGTGCCCCCGACAGCCCAGCC
SRR033510.8449358.2-               GTGTTTACCCCATCATGGGACTCACAGTGCCCCCGACAGCCCAGCCC
SRR033360.2867109.2-                 GTTTACCCCATCATGGGACTCACAGTGCCCCTGACAGCCCAGCCC
SRR033531.8867192+                         CCCATCATGGGACTCACAGTGCCCCTGACAGCCCAGCCC
SRR033531.971349+                           CCATCATGGGACTCACAGTGCCCCCGACAGCCCAGCCC
SRR033338.8222237.1+                         CATCATGGGACTCACAGTGCCCCTGACAGCCCAGCCC
SRR033155.7077050.2+                                GGACTCACAGTGCCCCCGACAGCCCAGCCC
SRR033511.7688566.1+                                GGACTCACAGTGCCCCCGACAGCCCAGCCC
SRR033155.7642565.1+                                GGACTCACAGTGCCCCCGACAGCCCAGCCC
SRR033539.714161.1+                                     TCACAGTGCCCCCGACAGCCCAGCCC
SRR033484.7701277+                                        ACAGTGCCCCTGACAGCCCAGCCC
SRR033538.8944889.1-                                       CAGTGCCCCTGACAGCCCAGCCC
SRR033530.11114387+                                         AGTGCCCCTCACAGCCCAGCCC
SRR033111.4457101.1+                                                       CCAGCCC
SRR033343.7657464.1+                                                   CAGCCCAGCCC
SRR033349.6801419.1-                                               GCCCCTGACAGCCCAGCCC
SRR033539.4581972.1-                                                            CC
SRR033137.8493289.2-                                                    CGACAGCCCAGCCC
SRR033150.3208496.2-                                                          GCCC
SRR033344.7099896.2+                                                      CCCAGCCC
SRR033359.4743555.1-                                             GTGCCCCCGACAGCCCAGCCC
SRR033510.2875829.1+                                                        CAGCCC
                      ____________________________________________________________
consensus             CCCGCTTAGAAATGTGTTTACCCCATCATGGGACTCACAGTGCCCCCGACAGCCCAGCCC

                          .    :    .    :    .    :    .    :    .    :    .    :
SRR033510.8449358.2-  ACG
SRR033360.2867109.2-  ACGAT
SRR033531.8867192+    ACGATGACCTG
SRR033531.971349+     ACGATGACCTGT
SRR033338.8222237.1+  ACGATGACCTGTG
SRR033155.7077050.2+  ACGATGACCTGTGAGCAGAG
SRR033511.7688566.1+  ACGATGACCTGTGAGAAGAG
SRR033155.7642565.1+  ACGATGACCTGTGAGCAGAG
SRR033539.714161.1+   ACGATGACCTGTGAGCAGAGGGAG
SRR033484.7701277+    ACGAGGACCTGTGAGCAGAGGGAGAA
SRR033538.8944889.1-  ACGATGACCTGTGAGCAGAGGGAGAAG
```

```
SRR033354.9429644.1+                GCAGAGGGAGAAGGCGCGGTCGGCACCATAGCAGTCGCTGATGCCT
SRR033538.3478988.1-        ACCTGTGAGCAGAGGGAGACGGCGGGGTCGTCCCCATAGCAGTCGCTGAT
SRR033530.11114387+   ACGATGACCTGTGAGCAGAGGGAGAAGG
SRR033111.4457101.1+  ACGATGACCTGTGAGCAGAGGGAGAAGGCGGGGGCGGCCCCAT
SRR033137.4803828.1+                                    CGGCCCCATAGCAGTCGCTGATGCCT
SRR033116.2424298.2+                                                AGTCGCTGATGCCT
SRR033343.7657464.1+  ACGATGACCTGTGAGCAGAGGGAGAAGGCGGGGTCGGAC
SRR033349.6801419.1-  ACGATGACCTGTGAGCAGAGGGAGAAGGCGG
SRR033341.7915166.1+                                            TAGCAGTCGCTGATGCCT
SRR033354.7985015.2+                                                          GCCT
SRR033510.5001387.2-                                                        ATGCCT
SRR033514.7535354.2-  ACGATGACATGTGAGCAGAGGGAGAAGGCGGGGTCGGCCCCATAGCAGTC
SRR033527.10419970+          CCTGTGAGCAGAGGGAGAAGGCGGGGTCGGCCCCATAGCAGACGCTGATG
SRR033528.2474477+                                     TCGGCCCCATAGCAGTCGCTGATGCCT
SRR033539.4581972.1-  ACGATGGCCTGTGAGCAGAGGGAGAAGGCGGGGTCGGCCCCATAGCAG
SRR032967.2773773+             TGTGAGCAGAGGGAGAAGGCGGGGTCGGCCCCATAGCAGTCGCTGATGCC
SRR032971.914122-                                    GGCCCCATAGCAGTCGCTGATGCCT
SRR033113.3066573.1+                                    CCCATAGCAGTCGCTGATGCCT
SRR033135.4589336.1+                          AAGGCGGGGTCGGCCCCATAGCAGTCGCTGATGCCT
SRR033136.2789267.2+                                     CCATAGCAGTCGCTGATGCCT
SRR033137.8493289.2-  ACGATGACCTGTGAGCAGAGGGAGAAGGCGGGGTCG
SRR033150.3208496.2-  ACGATGACCTGTGAGCAGAGGGAGAAGGCGGGGTCGGCCCCATAGC
SRR033155.434658.1-                    GAGGGAGAAGGCGGGGTCGGCCCCATAGCAGTCGCTGATGCCT
SRR033155.5231966.1+                                              TCGCTGATGCCT
SRR033340.8767403.1+                                                         T
SRR033342.356480.2+                                             AGTCGCTGATGCCT
SRR033344.7099896.2+  ACGATGACCTGTGAGCAGAGGGAGAAGGCGGGGTCGGCCCCA
SRR033349.6546750.1+                                                        CT
SRR033349.7629870.1+                                        TAGCAGTCGCTGATGCCT
SRR033350.8966124.1-              GAGCAGAGGGAGAAGGCGGGGTCGGCCCCATAGCAGTCGCTGATGCCT
SRR033350.10307601.1+                    GGGAGAAGGCGGGGTCGGCCCCATAGCAGTCGCTGATGCCT
SRR033359.4743555.1-  ACGATGACCTGTGAGCAGAGGGAGAAGGC
SRR033510.2875829.1+  ACGATGACCTGTGAGCAGAGGGAGAAGGCGGGGTCGGCCCCATA
SRR033514.72022.1-                               GCGGGGTCGGCCCCATAGCAGTCGCTGATGCCT
SRR032968.6391771+                               GCGGGGTCGGCCCCATAGCAGTCGCTGATGCCT
SRR033514.176295.1-                              GCGGGGTCGGCCCCATAGCAGTCGCTGATGCCT
SRR033516.8734636.2+                                                     CTGATGCCT
SRR033537.4328360.2+                                   TCGGCCCCATAGCAGTCGCTGATGCCT
                      ____________________________________________________________
consensus             ACGATGACCTGTGAGCAGAGGGAGAAGGCGGGGTCGGCCCCATAGCAGTCGCTGATGCCT

                          .    :    .    :    .    :    .    :    .    :    .    :
SRR033150.958272.2-                             GGCAGACACCAGGATGTGATGCACCATTGTCTCA
SRR033354.9429644.1+  GTGG
SRR033115.6785853.2+                             AGGCGTACACCAGGATGTGATGCACCATTGTCTCA
SRR033137.4803828.1+  GTGGGGAGAACGCTAGCATCGCCA
SRR033137.6998063.2+      GGAGAACGCTAGCATTGCCACAGGCATACACCAGGATGTGATGCACCATT
SRR033150.8133851.2+      GGAGAACGCTAGCATTGCCACAGGCATACACCAGGATGTGATGCACCATT
SRR033116.2424298.2+  GTGGGGAGAACGCTAGCATTGCCACAGGCATACACC
SRR033341.7915166.1+  GTGGGGAGAACGCTAGCATTGCCACCGGCGTA
SRR033354.7985015.2+  GTGGGGAGAACGCTAGCATTGCCACAGGCATACACCAGGATGTGAT
SRR033486.5199731-                         NCACAGGCGTACACCAGGATGTGATGCACCATTGTCTCA
SRR033510.5001387.2-  GTGGGGAGAACGCTAGCATTGCCACAGGCATACACCAGGATGTG
SRR033515.766782.1-           AACCCTAGCATTGCCACAGGCGTACACCAGGATGTGATGCACCATTGTCT
SRR033531.8436755-                         CCACAGGCATACACCAGGATGTGATGCACCATTGTCTCA
SRR033537.3644035.1-                                  TCCACCAGGATGTGATGCACCATTGTCTCA
SRR033528.2474477+    GTGGGGAGAACGCTAGCATTGAC
SRR032971.914122-     GTGGGGAGAACGCTAGCATTGCCAC
SRR033113.3066573.1+  GTGGGGAGAACGCTAGCATTGCCACAGG
SRR033135.4589336.1+  GTGGGGAGAACGCT
SRR033136.2789267.2+  GTGGGGAGAACGCTAGCATTGCCACAGGC
SRR033155.434658.1-   GTGGGGA
SRR033155.5231966.1+  GTGGGGAGAACGCTAGCATTGCCACAGGCGTACACCAG
SRR033150.2862260.1+                                   ACACCAGGATGTGATGCACCATTGTCTCA
SRR033338.8222237.2-                                   ACACCAGGATGTGATGCACCATTGTCTCA
SRR033340.8767403.1+  GTGGGGAGAACGCTAGCATTGCCACAGGCGTACACCAGGATGTGATGCA
SRR033342.356480.2+   GTGGGGAGAACGCTAGCATTGCCACAGGCGTACACC
SRR033349.6546750.1+  GTGGGGAGAACGCTAGCATTGCCACAGGCGTACACCAGGATGTGATGC
SRR033349.7629870.1+  GTGGGGAGAACGCTAGCATTGCCACAGGCGTA
SRR033350.8966124.1-  GT
SRR033350.10307601.1+ GTGGGGAGA
SRR033351.878786.1+                           CAGGCGTACACCAGGATGTGATGCACCATTGTCTCA
SRR033351.2700644.1-            CGCTAGCATTGCCACAGGCGTACACCAGGATGTGATGCACCATTGTCTCA
SRR033351.4777212.1-                                    TACACCAGGATGTGATGCACCATTGTCTCA
SRR033491.3988418-                                          CCAGGATGTGATGCACCATTGTCTCA
SRR033510.8135041.1+                              CGTACACCAGGATGTGATGCACCATTGTCTCA
SRR033514.72022.1-    GTGGGGAGAACGCTAGC
```

```
SRR032968.6391771+    GTGGGGAGAACGCTAGC
SRR033514.176295.1-   GTGGGGAGAACGCTAGC
SRR033516.8734636.2+  GTGGGGAGAACGCTAGCATTGCCACAGGCGTACACCAGGAT
SRR033517.1477519.1+                                  CACCAGGATGTGATGCACCATTGTCTCA
SRR033537.4328360.2+  GTGGGGAGAACGCTAGCATTGCC
SRR033343.7657464.2-                                              ATGCACCATTGTCTCA
SRR033341.3323921.1+                                                GCACCATTGTCTCA
SRR033517.2030839.2+                                                  ACCATTGTCTCA
SRR033531.1398163-                                                          TGTCTCA
SRR033540.6365478.1+                                                        TGTCTCA
SRR033110.5594214.1+                                                          CTCA
SRR033528.7590723-                                                            CTCA
                      ____________________________________________________________
consensus             GTGGGGAGAACGCTAGCATTGCCACAGGCGTACACCAGGATGTGATGCACCATTGTCTCA
```

```
                          .    :    .    :    .    :    .    :    .    :    .    :
SRR033150.958272.2-   TTGTGGTAGACCAACT
SRR033115.6785853.2+  TTGTGGTAGACCACC
SRR033486.5199731-    TTGTGGTAGAC
SRR033531.8436755-    TTGTGGTAGAC
SRR033537.3644035.1-  TTGTGGTAGACCAACTTAGG
SRR033150.2862260.1+  TTGTGGTAGACCAACTTAGGC
SRR033338.8222237.2-  TTGTGGTAGACCAACTTAGGC
SRR033351.878786.1+   TTGTGGTAGACCAA
SRR033351.4777212.1-  TTGTGGTAGACCAACTTAGG
SRR033491.3988418-    TTGTGGTAGACCAACTTAGGCTCA
SRR033510.8135041.1+  TTGTGGTAGACCAACTTA
SRR033517.1477519.1+  TTGTGGTAGACCAACTTAGGCT
SRR033343.7657464.2-  TTGTGGTAGACCAACTTAGGCTCAAACTGGGCAG
SRR033341.3323921.1+  TTGTGGTAGACCAACTTAGGCTCAAACTGGGCAGAC
SRR033517.2030839.2+  TTGTGGTAGACCAACTTAGGCTCAAACCGGGCAGAAAA
SRR033531.1398163-    TTGTGGTAGACCAACTTAGGCTCAAACTGGGCAGAAAAAGGAG
SRR033540.6365478.1+  TTGTGGTAGACCAACTTAGGCTCAAACTGGGCAGAAAAGGGAG
SRR033110.5594214.1+  TTGTGGTAGACCAACTTAGGCTCAAACTGGGCAGAAAAGGGAGAGC
SRR033528.7590723-    TTGTGGTAGACCAACTTAGGCTCAAACTGGGCAGAAAAAGGAGAGC
SRR033134.8617518.2+   TGTGGTAGACCAACTTAGGCTCAAACTGGGCAGAAAAGGGAGAGCAGAAC
SRR033136.2789267.1-   TGTGGTAGACCAACTTAGGCTCAAACTGGGCAGAAAAAGGAGAGCAGAAC
SRR033135.4589336.2-     TGGTAGACCAACTTAGGCTCAAACTGGGCAGAAAAAGGAGAGCAGAACAT
SRR033155.7077050.1-      GGTAGACCAACTTAGGCTCAAACTGGGCAGAAAAGGGAGAGCAGAACATG
SRR033155.7642565.2-      GGTAGACCAACTTAGGCTCAAACTGGGCAGAAAAGGGAGAGCAGAACATG
SRR033155.624107.1-        GTAGACCAACTTAGGCTCAAACTGGGCAGAAAAAGGAGAGCAGAACATGA
SRR032967.3847470-            GACCAACTTAGGCTCAAACTGGGCAGAAAAAGGAGAGCAGAACATGATCA
SRR033136.1368125.2+            CCAACTTAGGCTCAAACTGGGCAGAAAAGGGAGAGCAGAACATGATCAAG
SRR033530.1368267-              CCAACTTAGGCTCAAACTGGGCAGAAAAAGGAGAGCAGAACATGATCAAG
                      ____________________________________________________________
consensus             TTGTGGTAGACCAACTTAGGCTCAAACTGGGCAGAAAAAGGAGAGCAGAACATGATCAAG
```

(6) The exon 6 sequence was predicted by assembling WGS data using CAP3. The exon 6 is marked in red.

```
                          .    :    .    :    .    :    .    :    .    :    .    :
SRR033515.6459139.1-  GCCCTTCGGGCTGCCAAATCTACTTCCTTCCAACAGAGCTACCAGTTTCC
SRR033512.9761057.1-            CTGCCAAATCTACTTCCTTCCAACAGAGCTACCAGTTTCCAGATGACGTA
SRR033116.6842553.1-               CCAAATCTACTTCCTTCCAACAGAGCTACCAGTTTCCAGATGACGTA
SRR033361.2645576.2-               CCAAATCTACCTCCTCCCAACAGAGCTACCAGTTTCCAGATGACGTA
SRR033530.7418434-                 CCAAATCTACCTCCTTCCAACAGAGCTACCAGTTTCCAGATGACGTA
SRR033135.8169787.2+                   ATCTACTTCCTTCCAACAGAGCTACCAGTTTCCAGATGACGTA
SRR033511.8097657.1-                       ACTTCCTTCCAACAGAGCTACCAGTTTCCAGATGACGTA
SRR033515.5228956.2+                                CAACAGAGCTACCAGTTTCCAGATGACGTA
SRR033539.3439540.2+                                             AGTTTCCAGATGACGTA
SRR032965.3309545+                                           TACCAGTTTCCAGATGACGTA
SRR033491.8628593-                                                      AGATGACGTC
SRR033116.5102245.1-                                      AGCTACCAGTTTCCAGATGACGTA
SRR033351.5807973.2-                                               TTTCCAGATGACGTA
SRR033484.8742627+                                              CAGTTTCCAGATGACGTA
SRR033510.8484510.2+                                              GTTTCCAGATGACGTA
SRR033352.6107784.2-                                          ACCAGTTTCCAGATGACGTA
SRR033512.6324585.1+                                          ACCAGTTTCCAGATGACGTA
SRR033514.9685370.2-                                                 TCCAGATGACGTA
SRR033529.4735815-                                                  TTCCAGATGACGTA
SRR033539.6908051.1-                                                             A
                      ____________________________________________________________
consensus             GCCCTTCGGGCTGCCAAATCTACTTCCTTCCAACAGAGCTACCAGTTTCCAGATGACGTA
```

```
                          .    :    .    :    .    :    .    :    .    :    .    :
SRR033116.6842553.1-  GGC
SRR033361.2645576.2-  GGC
```

```
SRR033530.7418434-    GGC
SRR033135.8169787.2+  GGCGCCT
SRR033511.8097657.1-  GGCGTCTCTAT
SRR033515.5228956.2+  GGCGTCTCTATTGGGACCCC
SRR033340.3695113.1-                 ACCCCCTTAGACCCTCCGTGGATCCGACTGGAGCTTCATTACAGC
SRR033539.3439540.2+  GGCGTCTCGATTGGGACCCCCTTAGACCCCCAG
SRR032965.3309545+    GGCGTCTCTCTTGGGACCCCCTTAGACCC
SRR033114.9296278.1+             TGGGACCCCCTTAGACCCTCAGTGGATCCGACTGGAGATTCAGTACAGC
SRR033491.8628593-    GGCGTCTCTATTGGGACCCCCTTAGACCCTCAGTGGATCC
SRR033514.910690.1+                         GACCCTCAGTGGATCCGACTGGAGATTCATTACAGC
SRR033115.7343719.2+                CCCCCTTAGACCCTCAGTGGATCCGACTGGAGATTCATTACAGC
SRR033116.3907782.2+                          CCCTCAGTGGATCCGACTGGAGATTCATTACAGC
SRR033116.5102245.1-  GGCGTCTCTATTGGGACCCCCTTAGA
SRR033351.5807973.2-  GGCGTCTCTATTGGGACCCCCTTAGACCCTCAGTG
SRR033361.6281736.2-                      TAGACCCTCAGTGGATCCGACTGGAGATTCATTACAGC
SRR033484.8742627+    GGCGTCTCTATTGGGACCCCCTTAGACCCTCA
SRR033510.8484510.2+  GGCGTCTCTATTGGGACCCCCTTAGACCCTCAGT
SRR033352.6107784.2-  GGCGTCTCTATTGGGACCCCCTTAGACCCT
SRR033512.6324585.1+  GGCGTCTCTATTGGGACCCCCTTAGACCCT
SRR033514.9685370.2-  GGCGTCTCTATTGGGACCCCCTTAGACCCTCAGTGGA
SRR033515.1456794.2-                  CCCTTAGACCCTCAGTGGATCCGACTGGAGATTCATTACAGC
SRR033516.3960828.1-    CGTCTCTATTGGGACCCCCTTAGACCCTCAGTGGATCCGACTGGAGATTC
SRR033516.4704963.1+   GCGTCTCTATTGGGACCCCCTTAGACCCTCAGTGGATCCGACTGGAGATT
SRR033529.4735815-    GGCGTCTCTATTGGGACCCCCTTAGACCCTCAGTGG
SRR033530.1169829+                GGGACCCCCTTAGACCCTCAGTGGATCCGACTGGAGATTCATTACAGC
SRR033360.7654617.1+                            CTCAGTGGATCCGACTGGAGATTCATTACAGC
SRR033530.1621804+                              CTCAGTGGATCCGACTGGAGATTCATTACAGC
SRR033539.6908051.1-  GGCGTCTCTATTGGGACCCCCTTAGACCCTCAGTGGATCCGACTGGAGA
SRR033540.6590236.2+          TATTGGGACCCCCTTAGACCCTCAGTGGATCCGACTGGAGATTCATTACA
SRR033354.4447565.2+                               AGTGGATCCGACTGGAGATTCATTACAGC
SRR033340.677069.2-                                    GATCCGACTGGAGATTCCTTACAGC
SRR033490.1791332+                                       TCCGACTGGAGACTCATTACAGC
SRR032965.6113814+                                       TACGACTGGAGATTCATTACAGC
SRR033135.6933003.2+                                        GACTGGAGATTCATTACAGC
SRR033347.19644.2+                                           ACTGGAGATTCATTACAGC
SRR033510.357268.1+                                            TGGAGATTCATTACAGC
SRR033342.2940450.1+                                              AGATTCATTACAGC
SRR033345.1226627.2-                                                  TCATTACAGC
SRR033346.3579174.1+                                                         AGC
                      ____________________________________________________________
consensus             GGCGTCTCTATTGGGACCCCCTTAGACCCTCAGTGGATCCGACTGGAGATTCATTACAGC

                          .    :    .    :    .    :    .    :    .    :    .    :
SRR033340.3695113.1-  AATTT
SRR033114.9296278.1+  A
SRR033514.910690.1+   AATTTTAACAACCN
SRR033115.7343719.2+  AATTTT
SRR033116.3907782.2+  AATTTTAACAACCTTC
SRR033361.6281736.2-  AATTTTAACAAC
SRR033515.1456794.2-  AATTTTAA
SRR033530.1169829+    AA
SRR033360.7654617.1+  AATTTTAACAACCTTCCT
SRR033530.1621804+    AATTTTAACAACCTTCCT
SRR033354.4447565.2+  AATTTTAACAACCTTCCTGGT
SRR033340.677069.2-   AATTTTAACAACCTTCCTGGTGAGC
SRR033490.1791332+    AATTTTAACAACCTTCCTGGTGAGCAT
SRR032965.6113814+    AATTTTAACAACCTTCCTGGTGAGAAT
SRR033135.6933003.2+  AATTTTAACAACCTTCCTGGTGAGCATCCA
SRR033347.19644.2+    AATTTTAACAACCTTCCTGGTGAGCATCCAG
SRR033510.357268.1+   AATTTTAACAACCTTCCTGGTGAGCATCCAGAG
SRR033342.2940450.1+  AATTTTAACAACCTTCCTGGTGAGCATCCAGAGGAT
SRR033345.1226627.2-  AATTTTAACAACCTTCCTGGTGAGCATCCAGAGGATTTAA
SRR033346.3579174.1+  AATTTTAACAACCTTCCTGGTGAGCATCCAGAGGATTTAAGGAAGGG
SRR033350.8612470.1-         ACAACCTTCCTGGTGAGCATCCAGAGGATTTAAGGAAGGGAAGCTGGTGG
SRR033513.5500733.1+          CAACCTTCCTGGTGAGCATCCAGAGGATTTAAGGAAGGGAAGCTGGTGGA
SRR033347.4424783.1+            ACCTTCCTGGTGAGCATCCAGAGGATTTAAGGAAGGGAAGCTGGTGGGGC
SRR033487.6590998-              ACCTTCCTGGTGAGCATCCAGAGGATTTAAGGAAGGGAAGCTGGTGGAGC
SRR033535.5968839.2+            ACCTTCCTGGTGAGCATCCAGAGGATTTAAGGAAGGGAAGCTGGTGGAGC
                      ____________________________________________________________
consensus             AATTTTAACAACCTTCCTGGTGAGCATCCAGAGGATTTAAGGAAGGGAAGCTGGTGGAGC
```

(7) The exon 7 sequence was predicted by assembling WGS data using CAP3. The exon 7 is on the reverse strand and marked in red.

```
                          .    :    .    :    .    :    .    :    .    :    .    :
SRR033347.1866475.2+  GTGTTTCCTCCTCAGGAAGGGTGTGCCTCCCAGCTCCACCTCCTCAAACT
```

```
SRR033517.7931944.2+    GTTTCCTCCTCAGGAAGGGTGTGCCTCCCAGCTCCACCTCCTCAAACTTC
SRR033341.3215316.2+             TCAGGAAGGGTGTGCCTCCCAGCTCCACCTCCTCAAACTTCTCCGTCCT
SRR033341.549036.2+               CAGGAAGGGCGTGCCTCCCAGCTCCACCTCCTCAAACTTCTCCGTCCT
SRR033113.6841465.1+                  AAGGGTGTGCCTCCCAGCTCCACCTCCTCAAACTTCTCCGTCCT
SRR033338.618545.1-                      GGTGTTCCTCCCAGCTCCGCCTCCTCAAACTTCTCCGTCCT
SRR033511.9601286.1-                       TGTGCCTCCCAGCTCCACCTCCTCAAACTTCTCCTTCCT
SRR033511.9350737.2+                          GCCTCCCAGCTCCACCTCCTCAAACTTCTCCGTCCT
SRR033135.947959.2+                                CCAGCTCCACCTCCTCAAACTTCTCCGTCCT
SRR033489.2256813+                                  CAGCTCCACCTCCTCAAACTTCTCCGTCCT
SRR033346.9819675.2+                                   CTCCACCTCCTCAAACTTCTCCGTCCT
SRR032968.5896321+                                         ACCTCCTCAAACTTCTCCGTCCT
SRR033512.8266273.1-                                        CCTCCTCAACCTTCTCCGTCCT
SRR033116.7811484.2-                                               AAGCGTCTCCGTCCT
SRR033513.9406451.2-                                                AACTTCCCCGTCCT
SRR033350.4678138.1-                                                   TTCTCCGTCCT
SRR033155.5534285.1-                                                  CTTCTCCGTCCT
SRR033341.6774623.1+                                                       CCGTCCT
SRR033340.9044080.1+                                                       CCGTCCT
SRR033489.7932777-                                                         CCGTCCT
SRR033516.9009731.1-                                                      TCCGTCCT

consensus             GTGTTTCCTCCTCAGGAAGGGTGTGCCTCCCAGCTCCACCTCCTCAAACTTCTCCGTCCT

                          .    :    .    :    .    :    .    :    .    :    .    :
SRR033341.3215316.2+  A
SRR033341.549036.2+   AC
SRR033113.6841465.1+  ACACAG
SRR033338.618545.1-   ACACAGCCC
SRR033511.9601286.1-  ACACAGCCCAC
SRR033511.9350737.2+  ACACAGCCCACAGG
SRR033135.947959.2+   ACACAGCCCACAGGACATG
SRR033489.2256813+    ACACAGCCCACAGGACATGA
SRR033346.9819675.2+  ACACAGCCCACAGGACATGAAGG
SRR032968.5896321+    ACACAGCCCACAGGACATGAAGGACTC
SRR033512.8266273.1-  ACACAGCCCACAGGACATGAAGGACTCA
SRR033113.6841465.2-                                                         AGAGT
SRR033116.7811484.2-  CCACAGCCCACAGGACATGAAGGACTCAGCGCCCG
SRR033511.2485644.1-                                                          ACGT
SRR033513.9406451.2-  ACACAGCCCACAGGACATGGAGGACTCAGCGCCCGG
SRR033514.7636159.2-                                                           CGT
SRR033112.7564284.1+                ACATGAAGGACTCAGCGCCCGGGGGGATGAAGTGGATGGGGAACGT
SRR033350.4678138.1-  ACACAGCGCACAGGACATGAAGGACTCAGCGCCCGGGGG
SRR033360.130972.2+                                           ATGAAGTGGATGGGAAACGT
SRR033360.6354067.2+                                          ATGAAGTGGATGGGAAACGT
SRR033360.4773415.1+                                                       GAAACGT
SRR033526.5486165-                                               GTGGATGGGAAACGT
SRR033535.8922109.2+                       GGACTCAGCGCCCGGGGGGATGAAGTGGATGGGAAACGT
SRR033536.2577043.2+                ACATGAAGGACTCAGCGCCCGGGGGGAGGAAGTGGATGGGAAACGT
SRR033115.5194796.1-                          CTCAGCGCCCGGGGGGATGAAGTGGATGGGAAACGT
SRR033155.5534285.1-  ACACAGCCCACAGGACATGAAGGACTCAGCGCCCGGGG
SRR033339.1764888.1+     CAGCCCACAGGACATGAAGGACTCAGCGCCCGGGGGGATGAAGTGGATGG
SRR033339.7360014.2+                                                            GT
SRR033341.549036.1-                                                  GGGAAACGT
SRR033341.2825448.1-                     AAGGACTCAGCGCCCGGGGGGATGAAGTGGATGGGAAACGT
SRR033341.10314045.1-                   GAAGGACTCAGCGCCCGGGGGGATGAAGTGGATGGGAAACGT
SRR033347.8238150.1-                                             GGATGGGAAACGT
SRR033349.3943740.2+                   TGAAGGACTCAGCGCCCGGGGGGATGAAGTGGATGGGAAACGT
SRR033349.4239862.1+                                    GGGGGGATGAAGTGGATGGGAAACGT
SRR033349.4985624.1-       GCCCACAGGACATGAAGGACTCAGCGCCCGGGGGGATGAAGTGGATGGGA
SRR033353.7680569.2+   CACAGCCCACAGGACATGAAGGACTCAGCGCCCGGGGGGATGAAGTGGAT
SRR033342.2110210.2-                                   GGGATGAAGTGGATGGGAAACGT
SRR033354.5378375.2-                                   GGGATGAAGTGGATGGGAAACGT
SRR033487.10517734-         CCCACAGGACATGAAGGACTCAGCGCCCGGGGGGATGAAGTGGATGGGAA
SRR033341.6774623.1+  ACACAGCCCACAGGACATGAAGGACTCAGCGCCCGGGGGGATG
SRR033340.9044080.1+  ACACAGCCCACAGGACATGAAGGACTCAGCGCCCGGGGGGATG
SRR033489.7932777-    ACACAGCCCACAGGACATGAAGGACTCAGCGCCCGGGGGGATG
SRR033511.1666319.1-                                                          ACGT
SRR033486.6127117-                                                               T
SRR033512.5930707.1+                                                             T
SRR033341.11073972.2+                               GGGGGGATGAAGTGGATGGGAAACGT
SRR033515.5222637.1+                                GGGGGGATGAAGTGGATGGGAAACGT
SRR033516.9009731.1-  ACACAGCCCACAGGACATGAAGGACTCAGCGCCCGGGGGGAT
SRR033530.763115-                    CATGAAGGACTCAGCGCCCGGGGGGATGAAGTGGATGGGAAACGT
SRR033537.8247101.2+           ACAGGACATGAAGGACTCAGCGCCCGGGGGGATGAAGTGGATGGGAAACG

consensus             ACACAGCCCACAGGACATGAAGGACTCAGCGCCCGGGGGGATGAAGTGGATGGGAAACGT
```

```
                          .    :    .    :    .    :    .    :    .    :    .    :
SRR033113.6841465.2-  GAGCAAGCCCAGCTGGAGGACGCCCATGTCATATTTGCGCAGCTG
SRR033116.1556461.1+          CCAGCTGGAGGACGCCCATGTCATATTTGCGCAGCACAGAAGTGTAGTAC
SRR033341.9528145.2-                       CCCCATGTCATATTTGCGCAGCTGAGAAGTGTAGTACCC
SRR033511.2485644.1-  GAGTAAGCCCAGCTGGATGACGCCCATGTCATATTTGCGCAGCTGA
SRR033514.7636159.2-  GAGGAAGCCCAGCTGTAGGACGCCCATGTCAGATTTGCGCAGCTGAG
SRR033112.7564284.1+  GAGG
SRR033360.130972.2+   GAGGAAGCCCAGCTGGAGGACACCCATGTC
SRR033360.6354067.2+  GAGGAAGCCCAGCTGAAGGACACCCATGTC
SRR033360.4773415.1+  GAGGAAGCCCAGCTGGAGGACACCCATGTCATATTTGCGCAGC
SRR033526.5486165-    GAGGAAGCCCAGCTGGAGGACACCCATGTCATATT
SRR033528.4696898-              AGCTGGGGGACGCCCATGTCATATTTGCGCAGCTGAGAAGTGTAGTACAC
SRR033535.8922109.2+  GAGCAAGCCCA
SRR033536.2577043.2+  GAGG
SRR033109.1906362.1-                                ATATTTGCGCAGCTGAGAAGTGTAGTACAC
SRR033115.5194796.1-  GAGGAAGCCCAGCT
SRR033136.9451740.1+      AAGCCCAGCTGGAGGACGCCCATGTCATATTTGCGCAGCTGAGAAGTGTA
SRR033155.2132541.1+                       GCCCATGTCATATTTGCGCAGCTGAGAAGTGTAGTACAC
SRR033339.1764888.2-              CTGGAGGACGCCCATGTCATATTTGCGCAGCTGAGAAGTGTAGTACAC
SRR033339.7360014.2+  GAGGAAGCCCAGCTGGAGGACGCCCATGTCATATTTGCGCAGCTGAGA
SRR033341.549036.1-   GAGGAAGCCCAGCTGGAGGACGCCCATGTCATATTTGCGCA
SRR033341.2825448.1-  GAGGAAGCC
SRR033341.10314045.1- GAGGAAGC
SRR033347.1866475.1-         CCCAGCTGGAGGACGCCCATGTCATATTTGCGCAGCTGAGAAGTGTAGTA
SRR033347.8238150.1-  GAGGAAGCCCAGCTGGAGGACGCCCATGTCATATTTG
SRR033349.3943740.2+  GAGGAAG
SRR033349.4239862.1+  GAGGAAGCCCAGCTGGAGGACGCCC
SRR033350.7084478.1+                           ATGTCATATTTGCGCAGCTGAGAAGTGTAGTACAC
SRR033342.2110210.2-  GAGGAAGCCCAGCTGGAGGACGCCCAT
SRR033354.5378375.2-  GAGGAAGCCCAGCTGGAGGACGCCCAT
SRR033490.4225324+                   GAGGACGCCCATGTCATATTTGCGCAGCTGAGAAGTGTAGTACAC
SRR033511.1666319.1-  GAGGAAGCCCAGCTGGAGGACGCCCATGTCATATTTGCGCAGCTGA
SRR033512.3368015.2+                         CCATGTCATATTTGCGCAGCTGAGAAGTGTAGTACAC
SRR033486.6127117-    GAGGAAGCCCAGCTGGAGGACGCCCATGTCATATTTGCGCAGCTGAGAA
SRR033512.5930707.1+  GAGGAAGCCCAGCTGGAGGACGCCCATGTCATATTTGCGCAGCTGAGAA
SRR033513.5500733.2+                    GACGCCCATGTCATATTTGCGCAGCTGAGAAGTGTAGTACAC
SRR033341.11073972.2+ GAGGAAGCCCAGCTGGAGGACGCC
SRR033515.5222637.1+  GAGGAAGCCCAGCTGGAGGACGCC
SRR033517.7931944.1-                  AGGACGCCCATGTCATATTTGCGCAGCTGAGAAGTGTAGTACAC
SRR033530.763115-     GAGGA
SRR033531.3280465+          GCCCAGCTGGAGGACGCCCATGTCATATTTGCGCAGCTGAGAAGTGTAGT
SRR033532.12573662-                  GGACGCCCATGTCATATTTGCGCAGCTGAGAAGTGTAGTACAC
SRR033533.1381777.1-               TGGAGGACGCCCATGTCATATTTGCGCAGCTGAGAAGTGTAGTACAC
SRR033533.7719003.1+                                       CGCAGCTGAGAAGTGTAGTACAC
SRR033485.4275796+                                         CAGCTGAGAAGTGTAGTACAC
SRR033538.4748888.1+                                       GCTGAGAAGTGTAGTACAC
SRR033340.9044080.2-                                        CTGAGAAGTGTAGTACAC
SRR033341.6774623.2-                                        CTGAGAAGTGTAGTACAC
SRR033537.8247101.1-                                         TGAGAAGTGTAGTACAC
SRR033512.5569641.2+                                          GAGAAGTGTAGTACAC
SRR033516.4952387.2+                                          GAGAAGTGTAGTACAC
SRR033346.556722.2+                                             GAAGTGTAGTACAC
SRR033346.9819675.1-                                            GAAGTGTAGTACAC
SRR033511.9350737.1-                                             AAGTGTAGTACAC
SRR033532.807168+                                                    GTAGTACAC
SRR033110.6759320.2+                                                  TAGTACAC
SRR033341.3215316.1-                                                    GTACAC
SRR033113.904506.1+                                                      TACAC
SRR033347.509543.1+                                                        CAC
SRR033345.9216404.2+                                                         C
                      ____________________________________________________________
consensus             GAGGAAGCCCAGCTGGAGGACGCCCATGTCATATTTGCGCAGCTGAGAAGTGTAGTACAC

                          .    :    .    :    .    :    .    :    .    :    .    :
SRR033341.9528145.2-  GTGAATCCCTG
SRR033109.1906362.1-  GTGAATCCCTGAGGAATCAT
SRR033155.2132541.1+  GTGAATCCCTG
SRR033339.1764888.2-  GT
SRR033350.7084478.1+  GTGAATCCCTGAGGA
SRR033490.4225324+    GTGAA
SRR033512.3368015.2+  GTGAATCCCTGAG
SRR033513.5500733.2+  GTGAATCC
SRR033517.7931944.1-  GTGAAT
SRR033532.12573662-   GTGAATC
SRR033533.1381777.1-  GTG
SRR033533.7719003.1+  GTGAATCCCTGAGGAATCATACACACC
SRR033485.4275796+    GTGAATCCCTGAGGAATCATACACACCTG
```

```
SRR033538.4748888.1+  GTGAATCCCTGAGGAATCATACACACCTGGT
SRR033340.9044080.2-  GTGAATCCCTGAGGAATCATACACACCTGGTG
SRR033341.6774623.2-  GTGAATCCCTGAGGAATCATACACACCTGGTG
SRR033537.8247101.1-  GTGAATCCCTGAGGAATCATACACACCTGGTGC
SRR033512.5569641.2+  GTGAATCCCTGAGGAATCATACACACCTGGTGCA
SRR033516.4952387.2+  GTGAATCCCTGAGGAATCATACACACCTGGTGCA
SRR033346.556722.2+   GTGAATCCCTGAGGAATCATACACACCTGGTGCAGA
SRR033346.9819675.1-  GTGAATCCCTGAGGAATCATACACACCTGGTGCAGA
SRR033511.9350737.1-  GTGAATCCCTGAGGAATCATACACACCTGGTGCAGAG
SRR033532.807168+     GTGAATCCCTGAGGAATCATACACACCTGGTGCAGAGAGGA
SRR033110.6759320.2+  GTGAATCCCTGAGGAATCATACACACATGGTGCAGAGAGGAA
SRR033341.3215316.1-  GTGAATCCCTGGGGAATCATACACACCTGGTGCAGAGAGGAACA
SRR033113.904506.1+   GTGAATCCCTGAGGAATCATACACACCTGGTGCAGAGAGGAACAG
SRR033347.509543.1+   GTGAATCCCTGAGGAATCATACACACCTGGTGCAGAGAGGAACAGTC
SRR033345.9216404.2+  GTGAATCCCTGAGGAATCATACACACCTGGTGCAGAGAGGAACAGTCAA
SRR033339.8682384.2+   TGAATCCCTGAGGAATCATACACACCTGGTGCAGAGAGGAACAGTCAATC
SRR033535.8922109.1-     AATCCCTGAGGAATCATACACACCTGGTGCAGAGAGGAACAGTCAATCAC
SRR033351.3310832.1+      ATCCCTGAGGAATCATACACACCTGGTGCAGAGAGGAACAGTCAATCACA
SRR033517.350671.1+        TCCCTGAGGAATCATACACACCTGGTGCAGAGAGGAACTGTCAATCACAG
SRR033347.8610220.2+         CCTGAGGAATCATACACACCTGGTGCAGAGAGGAACAGTCAATCACAGGA
SRR033527.6432057-            CTGAGGAATCATACACACCTGGTGCAGAGAGGAACAGTCAATCACAGGAG
SRR033349.3943740.1-            GAGGAATCATACACACCTGGTGCAGAGAGGAACAGTCAATCACAGGAGAC
SRR033540.613262.1+             GAGGAATCATACACACCTGGTGCAGAGAGGAACAGTCAATCACAGGAGAC
SRR033353.7680569.1-             AGGAATCATACACACCTGGTGCAGAGAGGAACAGTCAATCACAGGAGAC
SRR033515.8476118.1+             AGGAATCATACACACCTGGTGCAGAGAGGAACAGTCAATCACAGGAGAC
SRR033135.947959.1-                 AATCATACACACCTGGTGCAGAGAGGAACAGTCAATCACAGGAGAC
                      ____________________________________________________________
consensus             GTGAATCCCTGAGGAATCATACACACCTGGTGCAGAGAGGAACAGTCAATCACAGGAGAC

                          .    :    .    :    .    :    .    :    .    :    .    :
SRR033353.7680569.1-  A
SRR033515.8476118.1+  A
SRR033135.947959.1-   AGGA
                      ____________________________________________________________
consensus             AGGA
```

(8) The exon 8 sequence was predicted by assembling WGS data using CAP3. The exon 8 is on the reverse strand and marked in red.

```
                          .    :    .    :    .    :    .    :    .    :    .    :
SRR033528.10842930+   CTTCCCTCACCTGTATTACACTGCTTGCAGAGCCCGTCCAGCAAAGTGGG
SRR033360.6079434.1+         CACCTGTATTACACTGCTTGCAGAGCCCGTCCAGCAAAGTGGGTGTGCAG
SRR033112.6675018.1+                          TTGCAGAGCCCGTCCAGCAAAGGGGGTGTGCAGCAG
SRR033515.3336928.1+                                                 GGGTGTGCAGCAG
SRR032966.9131407+                            TTGCAGAGCCCGTCCAGCAAAGTGGGTGTGCAGCAG
SRR033112.5263786.2-                                      TCCAGCAAAGTGGGTGTGCAGCAG
SRR033338.4560937.2-                                            AAAGTGGGTGTGCAGCAG
SRR033343.5239868.1+              GTATTACACTGCTTGCAGAGCCCGTCCAGCAAAGTGGGTGTGCAGCAG
SRR033349.7428556.1+                                                      TGCAGCAG
SRR033350.4433789.2-                                                   GTGTGCAGCAG
SRR033351.4548935.1-                        GCTTGCAGAGCCCGTCCAGCAAAGTGGGTGTGCAGCAG
SRR033351.4898264.1+                   ACACTGCTTGCAGAGCCCGTCCAGCAAAGTGGGTGTGCAGCAG
SRR033513.1875759.2-                                        CAGCAAAGTGGGTGTGCAGCAG
SRR033352.7384308.2-                                           CAAAGTGGGTGTGCAGCAG
SRR033536.4484763.1-                                           CAAAGTGGGTGTGCAGCAG
SRR033538.2160466.1+            CTGTATTACACTGCTTGCAGAGCCCGTCCAGCAAAGTGGGTGTGCAGCAG
SRR033540.7561999.1+                                     CCAGCAAAGTGGGTGTGCAGCAG
SRR033351.4898264.2-                                                         CAG
SRR033539.1858703.1+                                                          AG
                      ____________________________________________________________
consensus             CTTCCCTCACCTGTATTACACTGCTTGCAGAGCCCGTCCAGCAAAGTGGGTGTGCAGCAG

                          .    :    .    :    .    :    .    :    .    :    .    :
SRR033112.6675018.1+  GTAGCCATATCCCT
SRR033515.3336928.1+  GTAGCCATATACCTGTATGTCAGGCATAGGAGCTCAT
SRR032966.9131407+    GTAGCCATATACCT
SRR033112.5263786.2-  GTAGCCATATACCTGTATGTCAGGCA
SRR033338.4560937.2-  GTAGCCATATACCTGTATGTCAGGCATAGGAG
SRR033343.5239868.1+  GT
SRR033349.7428556.1+  GTAGCCATATACCTGTATGTCAGGCATAGGAGCTCCATTCAT
SRR033350.4433789.2-  GTAGCCATATACCTGTATGTCAGGCATAGGAGCTCCATT
SRR033351.4548935.1-  GTAGCCATATAC
SRR033351.4898264.1+  GTAGCCA
SRR033513.1875759.2-  GTAGCCATATACCTGTATGTCAGGCATA
SRR033352.7384308.2-  GTAGCCATATACCTGTATGTCAGGCATAGGA
SRR033536.4484763.1-  GTAGCCATATACCTGTATGTCAGGCATAGGA
```

```
SRR033540.7561999.1+  GTAGCCATATACCTGTATGTCAGGCAT
SRR033351.4898264.2-  GTAGCCATATACCTGTATGTCAGGCATAGGAGCTCCATTCATCTGTA
SRR033539.1858703.1+  GTAGCCATATACCTGTATGTCAGGCATAGGAGCTCCATTCATCTGTAA
SRR032967.988615+     GTAGCCATATACCTGTATGTCAGGCATAGGAGCTCCATTCATCTGTAACA
SRR033114.1853918.1+       CATATACCTGTATGTCAGGCATAGGAGCTCCATTCATCTGTAACAGGGAA
SRR033361.6418162.2-        ATATACCTGTATGTCAGGCATAGGAGCTCCATTCATCTGTAACAGGGAAG
SRR033349.647973.1+          TATACCTGTATGTCAGGCATAGGAGCTCCATTCATCTGTAACAGGGAAGA
SRR033109.788795.2-            TACCTGTATGTCAGGCATAGGAGCTCCATTCATCTGTAACAGGGAAGAGG
SRR033156.5789929.1+            ACCTGTATGTCAGGCATAGGAGCTCCATTCATCTGTAAAAGGGAAGAGGT
SRR032965.1438571-               CCTGTATGTCAGGCATAGGAGCTCCATTCATCTGTAACAGGGAAGAGGT
SRR033531.12664577-               CTGTATGTCAGGCATAGGAGCTCCATTCATCTGTAACAGGGAAGAGGT
SRR033339.8348388.1-               TGTATGTCAGGCATAGGAGCTCCATTCATCTGTAACAGGGAAGAGGT
SRR033360.2053008.2-                  ATGTCAGGCATAGGAGCTCCATTCATCTGTAACAGGGAAGAGGT
SRR033156.389889.1+                        AGGCATAGGAGCTCCATTCATCTGTAACAGGGAAGAGGT
SRR033539.1858703.2-                       AGGCATAGGAGCTCCATTCATCTGTAACAGGGAAGAGGT
SRR032965.3850347+                           GCATAGGAGCTCCATTCATCTGTAACAGGGAAGAGGT
SRR032969.5970060+                           GCATAGGAGCTCCATTCATCTGTAACAGGGAAGAGGT
SRR033489.1781267+                           GCATAGGAGCTCCATTCATCTGTAACAGGGAAGAGGT
SRR033156.5789929.2-                          CATAGGAGCTCCATTCATCTGTAACAGGGAAGAGGT
SRR033342.429466.1+                             TAGGAGCTCCATTCATCTGTAACAGGGAAGAGGT
SRR033359.6936389.1-                              GGAGCTCCATTCATCTGTAACAGGGAAGAGGT
SRR033353.9757492.1-                                AGCTCCATTCATCTGTAACAGGGAAGAGGT
SRR033532.7630860+                                  AGCTCCATTCATCTGTAACAGGGAAGAGGT
SRR033110.4769249.2-                                 GCTCCATTCATCTGTAGCAGGGAAGAGGT

consensus             GTAGCCATATACCTGTATGTCAGGCATAGGAGCTCCATTCATCTGTAACAGGGAAGAGGT

                          .    :    .    :    .    :    .    :    .    :    .    :
SRR032965.1438571-    G
SRR033531.12664577-   GC
SRR033339.8348388.1-  GCA
SRR033360.2053008.2-  GCACCT
SRR033156.389889.1+   GCACCTGATTT
SRR033539.1858703.2-  GCACCTGATTT
SRR032965.3850347+    GCACCTGATTTGT
SRR032969.5970060+    GCACCTGATTTGT
SRR033489.1781267+    GCACCTGATTTGT
SRR033156.5789929.2-  GCACCTGATTTGTT
SRR033342.429466.1+   GCACCTGATTTGTTAA
SRR033359.6936389.1-  GCACCTGATTTGTTAAGG
SRR033353.9757492.1-  GCACCTGATTTGTTAAGGAC
SRR033532.7630860+    GCACCTGATTTGTTAAGGAC
SRR033110.4769249.2-  GCACCTGATTTGTTAAGGACT

consensus             GCACCTGATTTGTTAAGGACT
```

(9) The exon 9 sequence was predicted by assembling WGS data using CAP3. The exon 9 is on the reverse strand and marked in red.

```
                          .    :    .    :    .    :    .    :    .    :    .    :
SRR033510.4632884.2+  TCAGACTCTCACCAGCTTGATCTCCACTCGAGAGGGCAAATCTCGAGTCT
SRR033134.4323132.1+    AGACTCTCACCAGCTTGATCTCCACTCGAGAGGGCAAATCTAGAGTCTCC
SRR032967.9474485+           CTCACCAGCTTGATCTCCACTCGAGAGGGCAAATCTCGAGTCTCCTGCAG
SRR033533.5009631.2-           CACCAGCTTGATCTCCACTCGAGAGGGCAAATCTCGAGTCTCCTGCAGGT
SRR033110.5642974.2+                                                    CCTGCAGGTT
SRR033352.7906386.2+                            CTCGAGAGGGCAAATCTCGAGTCTCCTGCAGGGT
SRR033526.12427092+                                     GGCAAATCTCGAGTCTCCTGCAGGTT
SRR033342.8500528.1-                           ACTCGAGAGGGCAAATCTCGAGTCTCCTGCAGGTT
SRR033112.7368824.1+                                        AATCTCGAGTCTCCTGCAGGTT
SRR033343.10580514.2-                                       AATCTCGAGTCTCCTGCAGGTT
SRR033345.278026.1+                       TCTCCACTCGAGAGGGCAAATCTCGAGTCTCCTGCAGGTT
SRR033352.1896229.1+                                               AGTCTCCTGCAGGTT
SRR033512.5212707.2-                                                  CTCCTGCAGGTT
SRR033512.7039747.1-                                     GCAAATCTCGAGTCTCCTGCAGGTT
SRR033517.7611347.1+                                                             T
SRR033528.1581554-                                                           AGGTT
SRR033533.5014075.1-                                                      TGCAGGTT

consensus             TCAGACTCTCACCAGCTTGATCTCCACTCGAGAGGGCAAATCTCGAGTCTCCTGCAGGTT

                          .    :    .    :    .    :    .    :    .    :    .    :
SRR033110.5642974.2+  GAAGTCGTAGGAATCGTCTTTACAGATTGTTCGAAGTCGC
SRR033352.7906386.2+  GAAGTCGTAGGAATCG
SRR033526.12427092+   GAAGTCGTAGGAATCATCTTTACA
SRR033342.8500528.1-  GAAGTCGTAGGAATC
SRR033112.7368824.1+  GAAGTCGTAGGAATCGTCTTTACAGATT
```

```
SRR033343.10580514.2- GAAGTCGTAGGAATCGTCTTTACAGATT
SRR033345.278026.1+   GAAGTCGTAG
SRR033352.1896229.1+  GAAGTCGTAGGAATCGTCTTTACAGATTGTTCGAA
SRR033512.5212707.2-  GAAGTCGTAGGAATCGTCTTTACAGATTGTTCGAAGTT
SRR033512.7039747.1-  GAAGTCGTAGGAATCGTCTTTACAG
SRR033517.7611347.1+  GAAGTCGTAGGAATCGTCTTTACAGATTGTTCGAAGTTGTGTTCCATTC
SRR033528.1581554-    GAAGTCGTAGGAATCGTCTTTACAGATTGTTCGAAGTTGTGTTCC
SRR033533.5014075.1-  GAAGTCGTAGGAATCGTCTTTACAGATTGTTCGAAGTTGTGT
SRR033527.13018437-   GAAGTCGTAGGAATCATCTTTACAGATTGTTCGAAGTTGTGTTCCATTCC
SRR033339.6466281.2-       CGTAGGAATCGTCTTTACAGATTGTTCGAAGTTGTGTTCCATTCCTAGAG
SRR033338.1588021.1-         TAGGAATCGTCTTTACAGATTGTTCGAAGTTGTGTTCCATTCCTAGAGGA
SRR033489.4297420-                 TCGTCTTTACAGATTGTTCGAAGTTGTGTTCCATTCCTAGAGGAAGA
SRR033342.7352865.1+                  TCTTTACAGATTGTTCGAAGTTGTGTTCCATTCCTAGAGGAAGA
SRR033487.2718764+                     CTTTACAGATTGTTCGAAGTTGTGTTCCATTCCTAGAGGAAGA
SRR033526.2954960+                           AGATTGTTCGAAGTTGTGTTCCATTCCTAGAGGAAGA
SRR033514.9812890.1-                             TGTTCGAAGTTGTGTTCCATTCCTAGAGGAAGA
SRR033347.2630881.2-                                TCGAAGTTGTGTTCCACTCCTAGAGGAAGA
SRR033349.4943152.1+                                 AGAAGTTGTGTTCCATTCCTAGAGGAAGA
                      ____________________________________________________________
consensus             GAAGTCGTAGGAATCGTCTTTACAGATTGTTCGAAGTTGTGTTCCATTCCTAGAGGAAGA

                          .    :    .    :    .    :    .    :    .    :    .    :
SRR033489.4297420-    GAA
SRR033342.7352865.1+  GAAGGC
SRR033487.2718764+    GAAGGCG
SRR033526.2954960+    GAAGGCGGGAATT
SRR033514.9812890.1-  GAAGGCGGGAATTCAGA
SRR033347.2630881.2-  GAAGGCGGGAATTCAGAAAT
SRR033349.4943152.1+  GAAGGCGGGAATTAAGAAATA
                      ____________________________________________________________
consensus             GAAGGCGGGAATTCAGAAATA
```

(10) The exon 10 sequence was predicted by assembling WGS data using CAP3. The exon 10 is marked in red.

```
                          .    :    .    :    .    :    .    :    .    :    .    :
SRR033341.3646303.2+  CACTCTGAACAGGGAGATGGATTGCTGGTAGAATGTCACTACCAGACACT
SRR033491.9824307-         TGAACAGGGAGATGGATTGCTGGTAGAATGTCACTACCAGACACTGGACT
SRR033511.1469520.1-       TGAACAGGGAGATGGATTGCTGGTAGAATGTCACTACCAGACACTGGACT
SRR033351.8849855.1-                     GATTGCTGGTACAATGTCACTACCAGACACTGGACTGTGAC
SRR033486.9537316+                     TGGATTGCTGGTAGAATGTCACTACCAGACACTGGACTGTGAC
SRR033512.7039747.2-                 GATGGATTGCTGGTAGAATGTCACTACCAGACACTGGACTGTGAC
SRR033360.2373126.1+                     GATTGCTGGTAGAATGTCACTACCAGACACTGGACTGTGAC
SRR033515.1461018.1-                     GATTGCTGGTAGAATGTCACTACCAGACACTGGACTGTGAC
SRR033116.4147181.1-                           TGGTAGAATGTCACTACCAGACACTGGACTGTGAC
SRR033511.9764889.1+                           TGGTAGAATGTCACTACCAGACACTCGAATGTGAC
SRR033512.3469522.1+                            GGTAGAATGTCACTACCAGACACTGGACTGTGAC
SRR033514.4749261.1+                                 AATGTCACTACCAGACACTGGACTGTGAC
SRR033516.2331834.1-                                  ATGTCACTACCAGACACTGGACTGTGAC
SRR033531.11824448+                                       CACTACCAGACACTGGACTGTGAC
SRR033536.2355517.1+                                         TACCAGACACTGGACTGTGAC
SRR033358.7647056.2+                                               ATACTGGACTGTGAC
SRR033533.729355.1+                                                ACACTGGACTGTGAC
SRR032967.1656588-                                                  CACTGGCCTGTGAC
SRR033352.8637956.1-                                                 ACTGGACTGTGAC
SRR033537.1702200.1+                                                 ACTGGACTGTGAC
SRR033342.9005150.1-                                                        TGTGAC
                      ____________________________________________________________
consensus             CACTCTGAACAGGGAGATGGATTGCTGGTAGAATGTCACTACCAGACACTGGACTGTGAC

                          .    :    .    :    .    :    .    :    .    :    .    :
SRR033351.8849855.1-  TCCATGACA
SRR033486.9537316+    TCCATGA
SRR033512.7039747.2-  TCCAT
SRR033360.2373126.1+  TCCATGACA
SRR033515.1461018.1-  TCCATGACA
SRR033116.4147181.1-  TCCATGACATTTGTA
SRR033511.9764889.1+  TCCATGACATTTGTA
SRR033512.3469522.1+  TCCATGACATTTGTAA
SRR033514.4749261.1+  TCCATGACATTTGTAAGTGAC
SRR033516.2331834.1-  TCCATGACATTTGTAAGTGACC
SRR033531.11824448+   TCCATGACATTTGTAAGTGACCTCTC
SRR033536.2355517.1+  TCCATGACATTTGTAAGTGACCTCTCTCC
SRR033358.7647056.2+  TCCATGACATTTGTAAGTGACCTCTCTCCCCGCCA
SRR033533.729355.1+   TCCATGACATTTGTAAGTGACCTCTCTCCCCGCCA
SRR032967.1656588-    TCCATGACATTTGTAAGTGACCTCTCTCCCCGCCAT
```

```
SRR033352.8637956.1-  TCCATGACATTTGTAAGTGACCTCTCTCCCCGCCATG
SRR033537.1702200.1+  TCCATGACATTTGTAAGTGACCTCTCTCCCCGCCATG
SRR033342.9005150.1-  TCCATGACATTTGTAAGTGACCTCTCTCCCCGCCATGGTCACCA
SRR033345.2687204.2-  TCCATGACATTTGTAAGTGACCTCTCTCCCCGCCATGGTCACCATGACCA
SRR033536.5930786.1+   CCATGACATTTGTAAGGGACCTCTCTCCCCGCCATGGTCACCATGACCAT
                      ____________________________________________________________
consensus             TCCATGACATTTGTAAGTGACCTCTCTCCCCGCCATGGTCACCATGACCAT
```

(11) The exon 11 sequence was predicted by assembling WGS data using CAP3. The exon 11 is marked in red.

```
                          .    :    .    :    .    :    .    :    .    :    .    :
SRR033156.7945884.1-  CAGGGAGGCCCCAGCACCATTAATGAGATGTGCCTCATCTTCCTCTTCTA
SRR033342.7883724.1+    GGGAGGCCCCAGCACCATTAATGAGATGTGCCTCATCTTCCTCTTCTACT
SRR033485.8383087-      GGGAGGCCCCAGCACCATTAATGAGATGTGCCTCATCTTCCTCTTCTACT
SRR033135.2096287.2+                            GATGTGCCTCATCTTCCTCTTCTACTATCCCCGA
SRR033135.2096287.1-                       GATCTGATGTGCCTCATCTTCCTCTTCTACTATCCCCGA
SRR033534.8924756.2-                                                       CCCGC
SRR033134.820203.2+                                         CTTCCTCTTCTACTATCCCCGC
SRR033339.9798213.2+        GGCCCCAGCACCATTAATGAGATGTGCCTCATCCTCCTCTTCTACTATCA
SRR033115.2602825.1+           CCCAGCACCATTAATGAGATGTGCCTCATCTTCCTCTTCTACTATCACCG
SRR033150.4779953.2-                        ATGAGATGTGCCTCATCTTCCTCTTCTACTATCACCGA
SRR033349.4503993.2+                   CATTAATGAGATGTGCCTCATCTTCCTCTTCTACTATCACCGA
SRR033351.895468.1-                              ATGTGCCTCATCTTCCTCTTCTACTATCACCGA
SRR033351.4104967.2-                          GAGATGTGCCTCATCTTCCTCTTCTACTATCACCGA
SRR033360.8370444.1-                                          TCCTCTTCTACTATCACCGA
SRR033517.8343213.1+                                                       ACCGA
SRR033360.3771535.2+                                                      CACCGA
SRR033538.2019996.2-             CAGCACCATTAATGAGATGTGCCTCATCTTCCTCTTCTACTATCACCGA
SRR033109.8543666.1-              AGCACCATTAATGAGATGTGCCTCATCTTCCTCTTCTACTATCACCGA
SRR033515.5064678.2+               GCACCATTAATGAGATGTGCCTCATCTTCCTCTTCTACTATCACCGA
SRR033540.8422886.2-                              CCTCATCTTCCTCTTCTACTATCCCCGA
SRR033113.9545731.1+               GCACCATTAATGAGATGTGCCTCATCTTCCTCTTCTACTATCCCCGA
SRR033134.820203.1-                                                   CTATCCCCGA
SRR033490.1572658-                                         TCTTCCTCTTCTACTATCCCCGA
SRR033511.4206612.2+                                                   TATCCCCGA
SRR033513.9931940.1+                           AGATGTGCCTCATCTTCCTCTTCTACTATCCCCGA
SRR032971.5799315+                                          CTTCCTCTTCTACTATCCCCGA
SRR033515.7008699.1+                                        CTTCCTCTTCTACTATCCCCGA
SRR033534.3039593.2-        GGCCCCAGCACCATTAATGAGATGTGCCTCATCTTCCTCTTCTACTATCC
                      ____________________________________________________________
consensus             CAGGGAGGCCCCAGCACCATTAATGAGATGTGCCTCATCTTCCTCTTCTACTATCCCCGA

                          .    :    .    :    .    :    .    :    .    :    .    :
SRR033135.2096287.2+  AATAACATCTCAGATC
SRR033135.2096287.1-  AATAACATCTC
SRR033534.8924756.2-  CATAACATCTCTAGCTGCACGGGGTACGCTGACATCATCTACGTG
SRR033487.1419987+               TAGCTGCATGGGGTACGCTGACATCATCTACGTGGCCCCCGCGCTGGGG
SRR033134.820203.2+   AATAACATCTCTAGCTGCCTGGGGTACG
SRR033150.4779953.2-  AATAACATCTCT
SRR033349.4503993.2+  AATAACA
SRR033351.895468.1-   AATAACATCTCTAGCTG
SRR033351.4104967.2-  AATAACATCTCTAG
SRR033360.8370444.1-  AATAACATCTCTAGCTGCATGGGGTACGCT
SRR033113.8372327.1+                    ATGGGGTACGCTGACATCATCTACGTGGCCCACGCGCTGGGG
SRR033517.8343213.1+  AATAACATCTCTAGCTGCATGGGGTACGCTGACATCATCTACGTG
SRR033360.3771535.2+  AATAACATCTCTAGCTGCATGGGGTACGCTGACATCATCTACGT
SRR033538.2019996.2-  A
SRR033109.8543666.1-  AA
SRR033515.5064678.2+  AAT
SRR033540.8422886.2-  GATAACATCTCTAGCTGCATGG
SRR032967.6892465-           TCTCTAGCTGCATGGGGTACGCTGACATCATCTACGTGGCCCACGAGCTG
SRR033113.9545731.1+  AAT
SRR033134.820203.1-   AATAACATCTCTAGCTGCATGGGGTACGCTGACATCATCT
SRR033135.9050672.1+        ATCTCTAGCTGCATGGGGTACGCTGACATCATCTACGTGGCCCACGAGCT
SRR033344.2504421.2+                     TGGGGTACGCTGACATCATCTACGTGGCCCACGAGCTGGGG
SRR033487.9375900-                          TACGCTGACATCATCTACGTGGCCCACGAGCTGGGG
SRR033488.43587+                        ATGGGGTACGCTGACATCATCTACGTGGCCCACGAGCTGGGG
SRR033490.1572658-    AATAACATCTCTAGCTGCATGGGGTAC
SRR033511.4206612.2+  AATAACATCTCTAGCTGCATGGGGTACGCTGACATCATCTA
SRR033513.9931940.1+  AATAACATCTCTAGC
SRR033514.1731751.1-                 TGCATGGGGTACGCTGACATCATCTACGTGGCCCACGAGCTGGGG
SRR032971.5799315+    AATAACATCTCTAGCTGCATGGGGTACG
SRR033515.7008699.1+  AATAACATCTCTAGCTGCATGGGGTACG
SRR033350.7232635.1+                            CGCTGACATCATCTACGTGGCCCACGAGCTGGGG
SRR033339.11257074.2+                             CTGACATCATCTACGTGGCCCACGAGCTGGGG
```

```
SRR033339.7256737.1+                                 ACATCATCTACGTGGCCCACGAGCTGGGG
SRR033135.5868117.2+                                     CATCTACGTGGCCCACGAGCTGGGG
SRR033491.6437106+                                           TACGTGGCCCACGAGCTGGGG
SRR033533.4456728.1-                                         TACGTGGCCCACGAGCTGGGG
SRR032969.7726455-                                             CGTGGCCCACGAGCTGGGG
SRR033112.3273814.1+                                             TGGCCCACGAGCTGGGG
SRR033134.7511794.1+                                             TGGCCCACGAGCTGGGG
SRR033516.2799449.2+                                                  CACGAGCTGGGG
SRR033515.753246.2-                                                     CGAGCTGGGG
                      ____________________________________________________________
consensus             AATAACATCTCTAGCTGCATGGGGTACGCTGACATCATCTACGTGGCCCACGAGCTGGGG

                          .    :    .    :    .    :    .    :    .    :    .    :
SRR033487.1419987+    G
SRR033113.8372327.1+  GAGGAGGC
SRR033344.2504421.2+  GAGGAGGCA
SRR033487.9375900-    GAGGAGGCATCAGA
SRR033488.43587+      GAGGAGGC
SRR033514.1731751.1-  GAGGA
SRR033350.7232635.1+  GAGGAGGCATCAGAGT
SRR033339.11257074.2+ GAGGAGGCATCAGAGTGA
SRR033339.7256737.1+  GAGGAGGCATCAGAGTGCGTC
SRR033135.5868117.2+  GAGGAGGCATCAGAGTGAGTCATCC
SRR033491.6437106+    GAGGAGGCATCAGAGTGAGTCATCCTGAA
SRR033533.4456728.1-  GAGGAGGCATCAGAGTGAGTCATCCTGAA
SRR032969.7726455-    GAGGAGGCATCAGAGTGAGTCATCCTGAAAT
SRR033112.3273814.1+  GAGGAGGCATCAGAGTGAGTCATCCTGAAATAT
SRR033134.7511794.1+  GAGGAGGCATCAGAGTGAGTCATCCTGAAATAT
SRR033516.2799449.2+  GAGGAGGCATCAGAGTGAGTCATCCTGAAATATAGGAC
SRR033515.753246.2-   GAGGAGGCATCAGAGTGAGTCATCCTGAAATATAGGACCT
SRR033339.9798213.1-  GAGGAGGCATCAGAGTGAGTCATCCTGAAATATAGGACCTCGTGTGTGTG
SRR033535.4632509.2+   AGGAGGCATCAGAGTGAGTCATCCTGAAATATAGGACCTCGTGTGTGTGG
SRR033115.2602825.2-    GGAGGCATCAGAGTGAGTCATCCTGAAATATAGGACCTCGTGTGTGTGGA
                      ____________________________________________________________
consensus             GAGGAGGCATCAGAGTGAGTCATCCTGAAATATAGGACCTCGTGTGTGTGGA
```

(12) The exon 12 sequence was predicted by assembling WGS data using CAP3. The exon 12 is marked in red.

```
                          .    :    .    :    .    :    .    :    .    :    .    :
SRR033352.3175980.2+  GTTCCCCCTGCCCTGATCCTACCACCCACAGCAGCCCCATGGAGGGAATG
SRR033112.7792983.1+    TCCCCCTGCCCTGATCCTACCACCCACAGCAGCCCCATGGAGGGAATGCT
SRR033345.7364210.2-    TCCCCCTGCCCTGATCCTACCACCCACAGCAGCCCCATGGAGGGAATGAT
SRR033537.9272045.1+    TCCCCCTGCCCTGATCCTACCACCCACAGCAGCCCCATGGAGGGAATGAT
SRR033349.6881009.1-     CCCCCTGCCCTGATCCTACCACCCACAGCAGCCCCATGGAGGGAATGATG
SRR033115.3372076.1+       CCCTGCCCTGATCCTACCACCCACAGCAGCCCCATGGAGGGAATGATGGA
SRR033486.3754255+          CCTGCCCTGATCCTACCACCCACAGCAGCCCCATGGAGGGAATGATGGAC
SRR033516.6611957.1-         CTGCCCTGATCCTACCACCCACAGCAGCCCCATGGAGGGAATGATGGACG
SRR033341.5172761.1-           GCCCTGATCCTACCACCCACAGCAGCCCCATGGAGGGAATGATGGACATG
SRR033351.6589536.1+           GCCCTGATCCTACCACCCACAGCAGCCCCATGGAGGGAATGATGGACATG
SRR033343.2510467.1+              CTGATCCTACCACCCACAGCAGCCCCATGGAGGGAATGATGGACATGA
SRR033517.669923.2+               CTGATCCTACCACCCACAGCAGCCCCATGGAGGGAATGATGGACATGA
SRR033137.6716725.2+                GATCCTACCACCCACAGCAGCCCCATGGAGGGAGTGATGGACATGA
SRR033352.2590007.2+                  TCCTACCACCCACAGCAGCCCCATGGAGGGAATGATGGACATGA
SRR033155.3768414.2+                    CTACCACCCACAGCAGCCCCATGGAGGGAATGATGGACATGA
SRR033540.8099086.2-                      CACACCCACAGCAGCCCAATGGAGGGAATGATGGACATGA
SRR033532.3085339+                           ACCCACAGCAGCCCCATGGAGGGAATGATGGACATGA
SRR033534.9289699.2-                           CCACAGCAGCCCCATGGAGGGAATGATGGACATGA
SRR033137.10104806.1+                             CAGCAGCCCCATGGAGGGAATGATGGACATGA
SRR033342.6440281.1-                                                          ATGA
SRR033338.133770.2-                                                        GACATGA
SRR033342.10005674.2-                                                      GACATGA
SRR033361.797582.2-                                                GAATGATGGACATGA
SRR033489.6710795-                                           TGGAGGGAATGATGGACATGA
SRR033353.10425268.1+                                                  GATGGACATGA
SRR033536.1742730.1-                                                 ATGATGGACATGA
SRR033540.8287141.1-                                                 ATGATGGACATGA
SRR032965.3103460+                                           TGGAGGGAATGATGGACATGA
SRR033112.8352547.1+                                                     TGGACATGA
SRR033134.8500176.1+                                                  TGATGGACATGA
SRR033341.9044934.1+                                                      GGACATGA
SRR033343.7303421.1+                                     CCCATGGAGGGAATGATGGACATGA
SRR033353.9835349.1+                                                        ACATGA
SRR033513.942179.2-                                                 AATGATGGACATGA
SRR033535.5477025.2+                                                   GATGGACATGA
                      ____________________________________________________________
```

```
consensus             GTTCCCCCTGCCCTGATCCTACCACCCACAGCAGCCCCATGGAGGGAATGATGGACATGA

                          .    :    .    :    .    :    .    :    .    :    .    :
SRR033343.2510467.1+  AT
SRR033517.669923.2+   AT
SRR033137.6716725.2+  ATGA
SRR033352.2590007.2+  ATGATG
SRR033155.3768414.2+  ATGATGTC
SRR033540.8099086.2-  ATGATGTCAA
SRR033532.3085339+    ATGATGTCGAGTG
SRR033534.9289699.2-  ATGATGTCGAGTGGA
SRR033137.10104806.1+ ATGATGTCAAGTGGACCC
SRR033155.1937416.1+            GTGGACCCTGGAGAGCATTAAGAAAGCTGAGAAAGCCTGCTAGGAGGCCC
SRR033342.6440281.1-  ATGATGTCGAGTGGACCCTGGAGAGCATTAAGAAAGCTGAGAAAGC
SRR033338.133770.2-   ATGATGTCGAGTGGACCCTGGAGAGCATTAAGAAAGCTGAGAA
SRR033342.10005674.2- ATGATGTCGAGTGGACCCTGGAGAGCATTAAGAAAGCTGAGAA
SRR033341.7088616.1+          GAGTGGACCCTGGAGAGCATTAAGAAAGCTGAGAAAGCCTGCAAGGAGGC
SRR033351.4075284.1+         CGAGTGGACCCTGGAGAGCATTAAGAAAGCTGAGAAAGCCTGCAAGGAGG
SRR033361.797582.2-   ATGATGTCGAGTGGACCCTGGAGAGCATTAAGAAA
SRR033361.7225047.2+      TGTCGAGTGGACCCTGGAGAGCATTAAGAAAGCTGAGAAAGCCTGCAAGG
SRR033489.6710795-    ATGATGTCGAGTGGACCCTGGAGAGCATT
SRR033346.2350413.2+                                   AAGCTGAGAAAGCATGCAAGGAGGCCC
SRR033526.10214497+            AGTGGACCCTGGAGCGCATTAAGAAAGCTGAGAAAGCCTGCAAGGAGGCC
SRR033353.10425268.1+ ATGATGTCGAGTGGACCCTGGAGAGCATTAAGAAAGCTG
SRR033536.1742730.1-  ATGATGTCGAGTGGACCCTGGAGAGCATTAAGAAAGC
SRR033540.8287141.1-  ATGATGTCGAGTGGACCCTGGAGAGCATTAAGAAAGC
SRR032965.3103460+    ATGATGTCAAGTGGACCCTGGAGAGCATT
SRR033112.8352547.1+  ATGATGTCAAGTGGACCCTGGAGAGCATTAAGAAAGCTGAG
SRR033134.8500176.1+  ATGATGTCAAGTGGACCCTGGAGAGCATTAAGAAAGCT
SRR033155.7424518.2-            GTGGACCCTGGAGAGCATTAAGAAAGCTGAGAAAGCCTGCAAGGAGGCCC
SRR033341.9044934.1+  ATGATGTCAAGTGGACCCTGGAGAGCATTAAGAAAGCTGAGA
SRR033343.7303421.1+  ATGATGTCAAGTGGACCCTGGAGAG
SRR033352.3175980.1-                                       TGAGAAAGCCTGCAAGGAGGCCC
SRR033352.4083717.1+                        GAGCATTAAGAAAGCTGAGAAAGCCTGCAAGGAGGCCC
SRR033353.8480190.2+   TGATGTCAAGTGGACCCTGGAGAGCATTAAGAAAGCTGAGAAAGCCTGCA
SRR033353.9835349.1+  ATGATGTCAAGTGGACCCTGGAGAGCATTAAGAAAGCTGAGAAA
SRR033354.4932652.2-    GATGTCAAGTGGACCCTGGAGAGCATTAAGAAAGCTGAGAAAGCCTGCAA
SRR033487.622766+                                TTAAGAAAGCTGAGAAAGCCTGCAAGGAGGCCC
SRR033487.1551828-               TGGACCCTGGAGAGCATTAAGAAAGCTGAGAAAGCCTGCAAGGAGGCCC
SRR033488.1574118+                                 AAGAAAGCTGAGAAAGCCTGCAAGGAGGCCC
SRR033512.1013528.2-                                AGCTGAGAAAGCCTGCAAGGAGGCCC
SRR033513.942179.2-   ATGATGTCAAGTGGACCCTGGAGAGCATTAAGAAAG
SRR033155.6782524.1-               GACCCTGGAGAGCATTAAGAAAGCTGAGAAAGCCTGCAAGGAGGCCC
SRR033515.1165311.1+               GACCCTGGAGAGCATTAAGAAAGCTGAGAAAGCCTGCAAGGAGGCCC
SRR033516.1971384.2-                               AAGCTGAGAAAGCCTGCAAGGAGGCCC
SRR033535.5477025.2+  ATGATGTCAAGTGGACCCTGGAGAGCATTAAGAAAGCTG
SRR033351.6589536.2-                                          GCCTGCAAGGAGGCCC
SRR033510.8455479.1-                                            CTGCAAGGAGGCCC
SRR033513.1575086.1-                                            CTGCAAGGAGGCCC
SRR033114.6778809.1-                                              GCAAGGAGGCCC
SRR033517.669923.1-                                                     AGGCCC
SRR033137.10104806.2-                                                     GCCC

consensus             ATGATGTCAAGTGGACCCTGGAGAGCATTAAGAAAGCTGAGAAAGCCTGCAAGGAGGCCC

                          .    :    .    :    .    :    .    :    .    :    .    :
SRR033346.2350413.2+  AGCAGACAGTGATAATAAAGACC
SRR033352.3175980.1-  AGCAGACAGTGATAATAAAGACCATTG
SRR033352.4083717.1+  AGCAGACAGTGA
SRR033487.622766+     AGCAGACAGTGATAATA
SRR033487.1551828-    A
SRR033488.1574118+    AGCAGACAGTGATAATAAA
SRR033512.1013528.2-  AGCAGACAGTGATAATAAAGACCA
SRR033155.6782524.1-  AGC
SRR033515.1165311.1+  AGC
SRR033516.1971384.2-  AGCAGACAGTGATAATAAAGACC
SRR033351.6589536.2-  AGCAGACAGTGATAATAAAGACCATTGATGTGAG
SRR033510.8455479.1-  AGCAGACAGTGATAATAAAGACCATTGATGTGAGTG
SRR033513.1575086.1-  AGCAGACAGTGATAATAAAGACCATTGATGTGAGTG
SRR033114.6778809.1-  AGCAGACAGTGATAATAAAGACCATTGATGTGAGTGTC
SRR033517.669923.1-   AGCAGACAGTGATAATAAAGACCATTGATGTGAGTGTCCCAGGA
SRR033137.10104806.2- AGCAGACAGTGATAATAAAGACCATTGATGTGAGTGTCCCAGGAGA
SRR033517.1544843.1-  AGCAGACAGTGATAATAAAGACCATTGATGTGAGTGTCCCAGGAGAGACC
SRR033343.2510467.2-          GTGATAATAAAGACCATTGATGTGAGTGTCCCAGGAGAGACCACCCTTTA
SRR033489.1298733-             TGATAATAAAGACCATTGATGTGAGTGTCCCAGGAGAGACCACCCTTTAG
SRR033114.6424715.1-             ATAATAAAGACCATTGATGTGAGTGTCCCAGGAGAGACCACCCTTTAGA
SRR033361.7225047.1-                 TAAAGACCATTGATGTGAGTGTCCCAGGAGAGACCACCCTTTAGA
```

```
SRR033352.2590007.1-                    AGACCATTGATGTGAGTGTCCCAGGAGAGACCACCCTTTAGA
SRR033513.6790503.2+                    AGACCATTGATGTGAGTGTCCCAGGAGAGACCACCCTTTAGA
                      ____________________________________________________________
consensus             AGCAGACAGTGATAATAAAGACCATTGATGTGAGTGTCCCAGGAGAGACCACCCTTTAGA

                          .    :    .    :    .    :    .    :    .    :    .    :
SRR033114.6424715.1-  G
SRR033361.7225047.1-  GTTAG
SRR033352.2590007.1-  GTTAGAGA
SRR033513.6790503.2+  GTTAGAGA
                      ____________________________________________________________
consensus             GTTAGAGA
```

(13) The exon 13 coding sequence was predicted by assembling WGS data using CAP3. The exon 13 is on the reverse strand and marked in red.

```
                          .    :    .    :    .    :    .    :    .    :    .    :
SRR033515.6030636.1+  TACGCGAAGAGACAGCATCAGACTCCATTCTGCAGCATGGCCAGGAGCCA
SRR032969.8464317+      TTTGAAGAGACAGCATCAGACTCCATTCTGCAGCATGGCCAGGAGCCAGA
SRR033534.9663715.2-      TGAAGAGACAGCATCAGACTCCATTCTGCAGCATGGCCAGGAGCCAGAAG
SRR033134.939995.2-         AAGAGACAGCATCAGACGCCATTCTGCAGCATGGCCAGGAGCCAGAAGAG
SRR033512.6180055.2-        AAGAGACAGCATCAGACTCCATTCTGCAGCATGGCCAGGAGCCAGAAGAG
SRR033536.186275.1+           GAGACAGCATCAGACTCCATTCTGCAGCATGGCCAGGAGCCAGAAGAGTC
SRR033338.7855431.2-             ACAGCATCAGACTCCATTCTGCAGCATGGCCAGGAGCCAGAAGAGTGTG
SRR033346.2680064.2+             ACAGCATCAGACTCCATTCTGCAGCATGGCCAGGAGCCAGAAGAGTGTG
SRR032968.4004876+                 AGCATCAGACTCCATTCTGCAGCATGGCCAGGAGCCAGAAGAGTGTG
SRR032965.5781525+                   CATCAGACTCCATTCTGCAGCATGGCCAGGAGCCAGAAGAGTGTG
SRR033515.1878925.2+                 CATCAGACTCCATTCTGCAGCATGGCCAGGAGCCAGAAGAGTGTG
SRR033137.2399432.1-                         CCCATTCTGGAGGATGGCCAGGAGCCAGAAGAGTGTG
SRR033347.4815792.2-                                                          AAGAGTGTG
SRR033515.7255283.2-                   GCAGACTCCATTCTGCAGCAGGGCCAGGAGCCAGAAGAGTGTG
SRR033150.8222913.1-                                   CATGGCCAGGAGCTAGAAGAGTGTG
SRR033359.7035014.1-                                        CCAGGAGCCAGAAGAGTGTG
SRR033513.4782663.2-                                                              TGTG
SRR033533.7967930.2-                                          AGGAGCCAGAAGAGTGTG
SRR032968.2344279-                                                  AAGAAGAGTGTG
SRR033136.6503895.2-                   TCAGACTCCATTCTGCAGCATGGCCAGGAGCCAGAAGAGTGTG
SRR033137.6691102.1-                         TCCATTCTGCAGCATGGCCAGGAGCCAGAAGAGTGTG
SRR033340.7205032.2-                           CATTCTGCAGCATGGCCAGGAGCCAGAAGAGTGTG
SRR033340.9055136.2-                                    GCATGGCCAGGAGCCAGAAGAGTGTG
SRR033338.8618963.1+                     AGACTCCATTCTGCAGCATGGCCAGGAGCCAGAAGAGTGTG
SRR033347.7111524.2+                     AGACTCCATTCTGCAGCATGGCCAGGAGCCAGAAGAGTGTG
SRR033352.5975561.1-                                      TGGCCAGGAGCCAGAAGAGTGTG
SRR033358.4812096.2-                                                     AGAAGAGTGTG
                      ____________________________________________________________
consensus             TACGTGAAGAGACAGCATCAGACTCCATTCTGCAGCATGGCCAGGAGCCAGAAGAGTGTG

                          .    :    .    :    .    :    .    :    .    :    .    :
SRR033338.7855431.2-  T
SRR033346.2680064.2+  T
SRR032968.4004876+    TGC
SRR032965.5781525+    TGCTG
SRR033515.1878925.2+  TGCTG
SRR033137.2399432.1-  TGCTGCAGCAGCA
SRR033347.4815792.2-  TGCTGCATCAGCAGGATGGCCTCCAGGGGGAGATGCCTCAG
SRR033351.3455089.2+             CAGGATGGCAGCCAGGGGGAGATGCCTCAGGGGGGCCCTGCTAGAGCTC
SRR033515.7255283.2-  TGCTGCA
SRR033515.9596823.2+           AGCAGGATGGCCGCCAGGGGGAGATGCCTCAGGGTGGCGCTGCTTGAGCT
SRR033150.8222913.1-  TGCTGCAGCAGCAGGATGGCCGCCA
SRR033339.6346061.2-                                                             AGCTC
SRR033345.8082593.2-                                                       TGCTAGAGCTC
SRR033359.7035014.1-  TGCTGCACCAGCAGGATGGCCGCCAGGGGG
SRR033513.4782663.2-  GGCTGCAGCAGCAGGATGGCCGCCAGGGGGAGATGCCTCAGGGTGG
SRR033517.6112947.1+                                                            GAGCTC
SRR033514.6260123.1+                                                         CTAGAGCTC
SRR033352.2211734.1-                                                         CTAGAGCTC
SRR033531.9415444+                                                                 CTC
SRR033533.7967930.2-  TGCTGCAGCAGCATGATGGCCGCCAGGGGGAG
SRR033535.5096307.1+                                                  GGCCCTGCTAGAGCTC
SRR032968.2344279-    TGCTGCAGCAGCAGGATGGCCGCCAGGGGGAGATGCCT
SRR032967.4649717-                                                      CCCTGCTAGAGCTC
SRR033136.6503895.2-  TGCTGCA
SRR033137.6691102.1-  TGCTGCAGCAGCA
SRR033340.1681051.2-                    GCCGCCAGGGGGAGATGCCTCAGGGTGGCCCTGCTAGAGCTC
SRR033340.7205032.2-  TGCTGCAGCAGCAGG
SRR033340.9055136.2-  TGCTGCAGCAGCAGGATGGCCGCC
```

```
SRR033347.4819621.2+                                           GGTGGCCCTGCTAGAGCTC
SRR033338.8618963.1+  TGCTGCAGC
SRR033347.7111524.2+  TGCTGCAGC
SRR033349.4437629.1-                   GGCCGCCAGGGGGAGATGCCTCAGGGTGGCCCTGCTAGAGCTC
SRR033352.5975561.1-  TGCTGCAGCAGCAGGATGGCCGCCAGG
SRR033358.4812096.2-  TGCTGCAGCAGCAGGATGGCCGCCAGGGGGAGATGCCTC
SRR033489.10288329-            AGCAGGATGGCCGCCAGGGGGAGATGCCTCAGGGTGGCCCTGCTAGAGCT
SRR033510.2289637.2-                           GGGGGAGATGCCTCAGGGTGGCCCTGCTAGAGCTC
SRR033512.7918451.1+                 ATGGCCGCCAGGGGGAGATGCCTCAGGGTGGCCCTGCTAGAGCTC
SRR033528.5025060+                                                     CTAGAGCTC
SRR033156.5671040.1-                              GGAGATGCCTCAGGGTGGCCCTGCTAGAGCTC
SRR033533.549391.2-                               GGAGATGCCTCAGGGTGGCCCTGCTAGAGCTC
SRR033536.9421044.2+  TGCTGCAGCAGCAGGATGGCCGCCAGGGGGAGATGCCTCAGGGTGGCCCT
SRR033540.8446931.2-   GCTGCAGCAGCAGGATGGCCGCCAGGGGGAGATGCCTCAGGGTGGCCCTG
                      ____________________________________________________________
consensus             TGCTGCAGCAGCAGGATGGCCGCCAGGGGGAGATGCCTCAGGGTGGCCCTGCTAGAGCTC

                          .    :    .    :    .    :    .    :    .    :    .    :
SRR033110.4742642.1-                                                          NNNN
SRR033346.2680064.1-             AGGTGCAGCTCTGAAGCCTGCAGGGGTCTTGTCCTAGGCCTCCACTTTG
SRR033351.3455089.2+  G
SRR033359.8216573.2+                                              CTGGGCCTCCACTTGG
SRR033486.8927123+                             AGCCTGCAGGGGTCTTGTCCTAGGCCTCCACTTTG
SRR033516.592516.1+                       TCTGAAGCCTGCAGGGGTCTTGTCCTAGGCCTCCACTTTG
SRR033537.7094893.1+                                GCAGGGGTCTTGTCCTAGGCCTCCACTTTG
SRR033351.3455089.1-                              CTGCAGGGGTCTTGTCCTAGGCCTCCACTTTG
SRR033513.7924860.2+                              CTGCAGGGGTCTTGTCCTAGGCCTCCACTTTG
SRR033339.6346061.2-  GAGATGGCCACAGGTGCAGCTCTGAAGCCTGTAGGGGTCTTGTCC
SRR033345.8082593.2-  GAGAGGGCCACAGGTGCAGCTCTGAAGCCTGCAGGGGTC
SRR033359.7634689.2+                        TGAAGCCTGTAGGGGTCTTGTCCTGGGCTTCCACTTTG
SRR033485.10451894-            ACGGGTGCAGCTCTGAAGCCTGTAGGGGTCTTGTCCTGGGCCTCCACTTT
SRR033515.8211060.1+        GCCACAGGTGCAGCTCTGAAGCCTGTAGGGGTCTTGTCCCGGGCCTCCAC
SRR033517.6112947.1+  GAGAGGGCCACAGGTGCAGCTCTGAAGCCTGTAGGGGTCTTGTA
SRR033514.6260123.1+  GAGAGGGCCACAGGTGCAGCTCTGAAGCCTGTAGGGGCCTT
SRR033352.2211734.1-  GAGAGGGCCACAGGTGCAGCTCTGAAGCCTGCAGGGGTCTT
SRR033531.9415444+    GAGAGGGCCACAGGTGCAGCTCTGAAGCCTGCAGGGGTCTTGTCCTG
SRR033338.8618963.2-                                             CCTAGGCCTCCACTTTG
SRR033535.5096307.1+  GAGAGGGCCACAGGTGCAGCTCTGAAGCCTGCAG
SRR033535.8454409.1+                                     GGTCTTGTCCTAGGCCTCCACTTTG
SRR033539.10186139.1-                                        TTGTCCTGGGCCTCCACTCTG
SRR032967.4649717-    GAGAGGGCCACAGGTGCAGCTCTGAAGCCTGTAGGG
SRR033109.358708.2+                       TCTGAAGCCTGTAGGGGTCTTGTCCTGGGCCTCCACTTTG
SRR033340.1681051.2-  GAGAGGGC
SRR033347.4819621.2+  GAGAGGGCCACAGGTGCAGCTCTGAAGCCTG
SRR033347.7111524.1-                                                      CCACTTTG
SRR033349.4437629.1-  GAGAGGG
SRR033352.9612189.2+  GAGAGGGCCACAGGTGCAGCTCTGAAGCCTGTAGGGGTCTTGTCCTGGGC
SRR033510.2289637.2-  GAGAGGGCCACAGGT
SRR033511.443442.2+                            AGCCTGTAGGGGTCTTGTCCTGGGCCTCCACTTTG
SRR033512.7918451.1+  GAGAG
SRR033517.6045102.2+                                                        ACTTTG
SRR033527.991326+                 GGTGCAGCTCTGAAGCCTGTAGGGGTCTTGTCCTGGGCCTCCACTTTG
SRR033528.5025060+    GAGAGGGCCACAGGTGCAGCTCTGAAGCCTGTAGGGGTCTT
SRR033156.5671040.1-  GAGAGGGCCACAGGTGCA
SRR033533.549391.2-   GAGAGGGCCACAGGTGCA
SRR033515.1878925.1-                                             CCTGGGCCTCCACTTTG
SRR033533.680839.2+                                              CCTGGGCCTCCACTTTG
SRR033533.956434.2+                                                     CTCCACTTTG
                      ____________________________________________________________
consensus             GAGAGGGCCACAGGTGCAGCTCTGAAGCCTGTAGGGGTCTTGTCCTGGGCCTCCACTTTG

                          .    :    .    :    .    :    .    :    .    :    .    :
SRR033110.4742642.1-  CCTCCAGATGACTCCAAGCAGGGCCCCCGGGGAGCAGGGATGATGT
SRR033346.2680064.1-  C
SRR033359.8216573.2+  CCTCCAGAGGACTCCAAGCAGGGCCCCCGGGGAG
SRR033486.8927123+    CCTCCAGATGACTCC
SRR033516.592516.1+   CCTCCAGATG
SRR033537.7094893.1+  CCTCCAGATGACTCCAAGCA
SRR033351.3455089.1-  CCTCCAGATGACTCCAAG
SRR033513.7924860.2+  CCTCCAGATGACTCCAAG
SRR033116.6217760.1+            ACTCCAAGCAGGGCCCCCGGGGAGCAGGGATGATGTCTGCAATCCAGCCT
SRR033359.7634689.2+  CCTCCAGATGAC
SRR033515.6030636.2-        GATGACTCCAAGCAGGGGCCCCGGGGAGCAGGGATGATGTCTGGAATCCA
SRR033515.9596823.1-                              GGTGAGCAGGGATGATGTCTGGAATCCAGCCT
SRR033338.8618963.2-  CCTCCAGATGACTCCAAGCAGGGCCCCCGGGGA
SRR033535.8454409.1+  CCTCCAGATGACTCCAAGCAGGGCC
SRR033539.10186139.1- CCTCCAGATGACTCCAAGCAGGGCCCCCG
```

```
SRR033109.358708.2+   CCTCCAGATG
SRR033110.7224598.1+                 AAGCAGGGCCCCCGGGGAGCAGGGATGATGTCTGGAATCCAGCCT
SRR033347.7111524.1-  CCTCCAGATGACTCCAAGCAGGGCCCCCGGGGAGCAGGGATG
SRR033347.8129156.1+             CTCCAAGCAGGGCCCCCGGGGAGCAGGGATGATGTCTGGAATCCAGCCT
SRR033350.614685.1+     TCCAGATGACTCCAAGCAGGGCCCCCGGGGAGCAGGGATGATGTCTGGAA
SRR033354.4356745.1+               CCAAGCAGGGCCCCCGGGGAGCAGGGATGATGTCTGGAATCCAGCCT
SRR033510.9683448.1+                         CCCCCGGGGAGCAGGGATGATGTCTGGAATCCAGCCT
SRR033347.4819621.1-                         CCCCCGGGGAGCAGGGATGATGTCTGGAATCCAGCCT
SRR033510.9780827.1+                         CCCCCGGGGAGCAGGGATGATGTCTGGAATCCAGCCT
SRR033511.443442.2+   CCTCCAGATGACTCC
SRR033512.7914934.1+                CAAGCAGGGCCCCCGGGGAGCAGGGATGATGTCTGGAATCCAGCCT
SRR033517.6045102.2+  CCTCCAGATGACTCCAAGCAGGGCCCCCGGGGAGCAGGGATGAT
SRR033527.991326+     CC
SRR033515.1878925.1-  CCTCCAGATGACTCCAAGCAGGGCCCCCGGGGA
SRR033533.680839.2+   CCTCCAGATGACTCCAAGCAGGGCCCCCGGGGA
SRR033533.956434.2+   CCTCCAGATGACTCCAAGCAGGGCCCCCGGGGAGCAGGGA
SRR033511.6868648.1-                                  AGCAGGGATGATGTCTGGAATCCAGCCT
SRR033517.6112947.2-                                  AGCAGGGATGATGTCTGGAATCCAGCCT
SRR033516.9302786.2+                                      GGGATGATGTCTGGAATCCAGCCT
SRR033536.9421044.1-                                            ATGTGTGGAATCCAGCCT
SRR033533.2878291.2+                                                    AATCCAGCCT
SRR033352.9612189.1-                                                     ATCCAGCCT
SRR033353.7351971.1+                                                     ATCCAGCCT
SRR033341.8398226.1+                                                      TCCAGCCT
SRR033484.2417233+                                                                 CCT
SRR033339.9169436.1+                                                            CT

consensus             CCTCCAGATGACTCCAAGCAGGGCCCCCGGGGAGCAGGGATGATGTCTGGAATCCAGCCT

                          .    :    .    :    .    :    .    :    .    :    .    :
SRR033515.9596823.1-  GTTGTGTTTTCCACCACC
SRR033110.7224598.1+  GTTGT
SRR033347.8129156.1+  G
SRR033354.4356745.1+  GTT
SRR033510.9683448.1+  GTTGTGTTTTCCA
SRR033347.4819621.1-  GTTGTGTTTTCCA
SRR033510.9780827.1+  GTTGTGTTTTCCA
SRR033512.7914934.1+  GTTG
SRR033511.6868648.1-  GTTGTGTTTTCCACCACCTCCT
SRR033517.6112947.2-  GTTGTGTTTTCCACCACCTCCT
SRR033516.9302786.2+  GTTGTGTTTTCCACCACCTCCTGCAA
SRR033536.9421044.1-  GTTGTGTTTTCCACCACCTCCTGCAAGGGAGA
SRR033533.2878291.2+  GTTGTGTTTTCCACCACCTCCTGCAAGGGAGAGCAAAAAA
SRR033352.9612189.1-  GTTGTGTTTTCCACCACCTCCTGCAAGGGAGAGCAAAAAAC
SRR033353.7351971.1+  GTTGTGTTTTCCACCACCTCCTGCAAGGGAGAGCAAAAAAC
SRR033341.8398226.1+  GTTGTGTTTTCCACCACCTCCTGCAAGGGAGAGCAAAAAACA
SRR033484.2417233+    GTTGTGTTTTCCACCACCTCCTGCAAGGGAGAGCAAAAAACAGGGTA
SRR033339.9169436.1+  GTTGTGTTTTCCACCACCTCCTGCAAGGGAGAGCAAAAAACAGGGTAA
SRR033514.10081575.2+ GTTGTGTTTTCCACCACCTCCTGCAAGGGAGAGCAAAAAACAGGGTAAAG
SRR033528.10968528+   CTTGTGTTTTCCACCACCTCCTGCAAGGGAGGGCAAAAAACAGGGTAAAG
SRR033516.592516.2-        GTTTTCCACCACCTCCTGCAAGGGAGAGCAAAAAACAGGGTAAAGGTAAA
SRR033527.7853422+         GTTTTCCACCACCTCCTGCAAGGGAGAGCAAAAAACAGGGTAAAGGTAAA
SRR033531.11306155-         TTTTCCACCACCTCCTGCAAGGGAGAGCAAAAAACAGGGTAAAGGTAAAG
SRR033111.6960337.1+         TTTCCACCACCTCCTGCAAGGGAGAGCAAAAAACAGGGTAAAGGTAAAGG
SRR033526.4523823-           TTTCCACCACCTCCTGCAAGGGAGAGCAAAAAACAGGGTAAAGGTAAAGG
SRR033529.749110-            TTTCCACCACCTCCTGCAAGGGAGAGCAAAAAACAGGGTAAAGGTAAAGG

consensus             GTTGTGTTTTCCACCACCTCCTGCAAGGGAGAGCAAAAAACAGGGTAAAGGTAAAGG
```

7. Northern white-cheeked gibbon (*Nomascus leucogenys*)

```
The gene is not present.
```

8. Eastern hoolock gibbon (*Hoolock leuconedys*)

```
The gene is not present.
```

9. Silvery gibbon (*Hylobates moloch*)

```
The gene is not present.
```

10. Pileated gibbon (*Hylobates pileatus*)

```
The gene is not present.
```

11. Siamang (*Symphalangus syndactylus*)

```
The gene is not present.
```

12. Rhesus macaque (*Macaca mulatta*)

>Macaca_mulatta No=12 length=1764 name="Rhesus macaque"
ATGGCCCATGACCTTCTCTTCAGGCTTTTTCCGCTTTTGGCCCTGGCAGCCCCCTTACAAAGCAACCGCCCTGGCCCCACATCTCGCCTGCGCTATTCCA
GGTTCCTAGATCCTTCCAATGTCATTTTCCTGCGTTGGGACTTTGATCTTGAGGCTGAAATCATCAGTTTTGAGCTCCAGGTCCGGACAGCTGGCTGGGT
GGGCTTAGGTGTCACAAATCGCTACACCAATGTGGGAAGTGATCTGGTTGTTGGAGGAGTCTTGCCCAATGGCAATGTCTATTTCTCGGATCAGCACTTG
GTAAATGGAGACACTCTGAAGGAGGATGGGAGCCAGGATGCTGAGCTGCTGGGGCTGACAGAAGATGCTGTCTACACCACCATGCGTTTTTCCAGGCCCT
TCCGCTCCTGTGACCTGCATGACCTGGATATTACGAGTGACACTGTGAGGGTGCTGGCCGCCTATGGTCTGGATGACACTCTGAAGCTGGATCAGGAGCG
TACTTTTGTCAAGTCCATCTTTCTGCTACAAGTTGTCCACCCTGATGATCTGGATGTCCCCAAGGACACCATCATCCATGACTTGGAGATCACTGATTTC
CTCATTCCAGAGGATGACACCACGTACGCCTGCACCTTTCTTCCTCTCCCCATTGTTAGCGAGAAACATCACATTTACAAGTTTGAGCCTAAGTTGGTCT
ACCACAATGAGACAATGGTGCATCACATCCTGGTGTATGCCTGTGGCAATGCTAGCATTCTCCCCACAGGCATCAGCGACTGCTATGGGGCCGACCCTGC
CTTCTCCCTCTGCTCACAGGTCATCGTGGGCTGGGCTGTCGGGGGCACTAGCTACCAGTTTCCAGATGATGTGGGTGTCTCTATTGGGACTCCCTTGGAC
CCTCAGTGGATCCGACTGGAGATTCATTACAGCAATTTTAACAACCTGCCTGGTGTGTATGATTCCTCGGGGATTCGCGTGTACTACACTTCTCAGCTGC
GCAAATATGACATGGGCGTCCTCCAGCTGGGCTTCTTCACGTTTCCCGTCCACTTCATCCCCCCGGGCGCTGAGTCCTTCCTGTCCTACGGGCTGTGTAG
GACAGAGAAGTTTGAGGAGATGAATGGAGCCCCCGTGCCTGACATACAGGTGTATGGCTACCTGCTGCACACCCACTTGGCTGGACAGGCTCTGCAAGCC
GTGCAATACAGGAATGGAACACAACTTCGAACAATCTGTAAAGACGATTCCTACGACTTCAATCTGCAGGAGACTCGAGATTTGCCCTCTCGAGTGGAGA
TCAAGCCGGGAGATGAATTGCTGGTAGAATGTCACTACCAGACACTGGACCGTGACTTCATGACATTTGGAGGCCCCAGCACCATTAATGAGATGTGCCT
CATCTTCCTCTTCTACTATCCGCGAAATAACATCTCCAGCTGCATGGGGTACCCTGACATTATCTACGTGGCCCACGAACTGAAGGAGGAGGCATCAGAC
TCCATGGAGGGAATCATGGCCATGAACAATGTCGAGTGGACCCCGGAGAGCATTAAGAAAGCTGAGAAAGCCTGCAAGGAGGCCCAGCAGACAGTGATAA
TAAAGACCATTGATGAGGTGGTGGAAAACACAACAGGCTGGATTCCAGACATCATCCCTACTCCCCGGGGGCCCTGCTTGGAGTCCTTGGGAGGCAAAGT
GGAGGCCCACGACAAGACCCCTGCAGGCTTCAGGCCCTCTCAAGCTCTAGCAGGGCCACCCTGA

>Macaca_mulatta No=12 length=587 name="Rhesus macaque"
MAHDLLFRLFPLLALAAPLQSNRPGPTSRLRYSRFLDPSNVIFLRWDFDLEAEIISFELQVRTAGWVGLGVTNRYTNVGSDLVVGGVLPNGNVYFSDQHL
VNGDTLKEDGSQDAELLGLTEDAVYTTMRFSRPFRSCDLHDLDITSDTVRVLAAYGLDDTLKLDQERTFVKSIFLLQVVHPDDLDVPKDTIIHDLEITDF
LIPEDDTTYACTFLPLPIVSEKHHIYKFEPKLVYHNETMVHHILVYACGNASILPTGISDCYGADPAFSLCSQVIVGWAVGGTSYQFPDDVGVSIGTPLD
PQWIRLEIHYSNFNNLPGVYDSSGIRVYYTSQLRKYDMGVLQLGFFTFPVHFIPPGAESFLSYGLCRTEKFEEMNGAPVPDIQVYGYLLHTHLAGQALQA
VQYRNGTQLRTICKDDSYDFNLQETRDLPSRVEIKPGDELLVECHYQTLDRDFMTFGGPSTINEMCLIFLFYYPRNNISSCMGYPDIIYVAHELKEEASD
SMEGIMAMNNVEWTPESIKKAEKACKEAQQTVIIKTIDEVVENTTGWIPDIIPTPRGPCLESLGGKVEAHDKTPAGFRPSQALAGPP

(1) Exon coordinates

Exon	UCSC Chromosome	Strand	Start	End
1	rheMac3 chr3	-	181523051	181523338
2	rheMac3 chr3	-	181522262	181522408
3	rheMac3 chr3	-	181521565	181521726
4	rheMac3 chr3	-	181521236	181521319
5	rheMac3 chr3	-	181520229	181520396
6	rheMac3 chr3	-	181519935	181520037
7	rheMac3 chr3	-	181519576	181519742
8	rheMac3 chr3	-	181519134	181519225
9	rheMac3 chr3	-	181518758	181518854
10	rheMac3 chr3	-	181518565	181518624
11	rheMac3 chr3	-	181516983	181517113
12	rheMac3 chr3	-	181516067	181516181
13	rheMac3 chr3	-	181515547	181515776

13. Crab-eating macaque (*Macaca fascicularis*)

```
>Macaca_fascicularis No=13 length=1764 name="Crab-eating macaque"
ATGGCCCATGACCTTCTCTTCAGGCTTTTTCCGCTTTTGGCCCTGGCAGCCCCCTTACAAAGCAACCGCCCTGGCCCCACATCTCGCCTGCGCTATTCCA
GGTTCCTAGATCCTTCCAATGTCATTTTCCTGCGTTGGGACTTTGACCTTGAGGCTGAAATCATCAGTTTTGAGCTCCAGGTCCGGACAGCTGGCTGGGT
GGGCTTAGGTGTCACAAATCGCTACACCAATGTGGGAAGTGATCTGGTTGTTGGAGGAGTCTTGCCCAATGGCAATGTCTATTTCTCGGATCAGCACTTG
GTAAATGGAGACACTCTGAAGGAGGATGGGAGCCAGGATGCTGAGCTGCTGGGGCTGACAGAAGATGCTGTCTACACCACCATGCGCTTTTCCAGGCCCT
TCCGCTCCTGTGACCCGCATGACCTGGATATTACGAGTGACACTGTGAGGGTGCTGGCCGCCTATGGTCTGGATGACACTCTGAAGCTGGATCAGGAGCG
TACTTTTGTCAAGTCCATCTTTCTGCTACAAGTTGTCCACCCTGATGATCTGGATGTCCCCAAGGACACCATCATCCATGACTTGGAGATCACTGATTTC
CTCATTCCAGAGGATGACACCACGTACGCCTGCACCTTTCTTCCTCTCCCCATTGTTAGCGAGAAACATCACATTTACAAGTTTGAGCCTAAGTTGGTCT
ACCACAATGAGACAATGGTGCATCACATCCTGGTGTATGCCTGTGGCAATGCTAGCGTTCTCCCCACAGGCATCAGCGACTGCTATGGGGCCGACCCTGC
CTTCTCCCTCTGCTCACAGGTCATCGTGGGCTGGGCTGTCGGGGGCACTAGCTACCAGTTTCCAGATGATGTGGGTGTCTCTATTGGGACTCCCTTGGAC
CCTCAGTGGATCCGACTGGAGATTCATTACAGCAATTTTAACAACCTGCCTGGTGTGTATGATTCCTCGGGGATTCGCGTGTACTACACTTCTCAGCTGC
GCAAATATGACATGGGCGTCCTCCAGCTGGGCTTCTTCACGTTTCCCATCCACTTCATCCCCCCGGGCGCTGAGTCCTTCCTGTCCTACGGGCTGTGTAG
GACAGAGAAGTTTGAGGAGATGAATGGAGCCCCCGTGCCTGACATACAGGTGTATGGCTACCTGCTGCACACCCACTTGGCTGGACAGGCTCTGCAAGCC
GTGCAATACAGGAATGGAACACAACTTCGAACAATCTGTAAAGACGATTCCTACGACTTCAATCTGCAGGAGACTCGAGATTTGCCCTCTCGAGTGGAGA
TCAAGCCGGGAGATGAATTGCTGGTAGAATGTCACTACCAGACACTGGACCGTGACTTCATGACATTTGGAGGCCCCAGCACCATTAATGAGATGTGCCT
CATCTTCCTCTTCTACTATCCGCGAAATAACATCTCCAGCTGCATGGGGTACCCTGACATTATCTACGTGGCCCACGAACTGGGGGAGGAGGCATCAGAC
TCCATGGAGGGAATCATGGCCATGAACAATGTCGAGTGGACCCCGGAGAGCATTAAGAAAGCTGAGAAAGCCTGCAAGGAGGCCCAGCAGACAGTGATAA
TAAAGACCATTGATGAGGTGGTGGAAAACACAACAGGCTGGATTCCAGACATCATCCCTACTCCCCGGGGGCCCTGCTTGGAGTCCTTGGGAGGCAAAGT
GGAGGCCCACGACAAGACCCCTGCAGGCTTCAGGCCCTCTCAAGCTCTAGCAGGGCCACCCTGA

>Macaca_fascicularis No=13 length=587 name="Crab-eating macaque"
MAHDLLFRLFPLLALAAPLQSNRPGPTSRLRYSRFLDPSNVIFLRWDFDLEAEIISFELQVRTAGWVGLGVTNRYTNVGSDLVVGGVLPNGNVYFSDQHL
VNGDTLKEDGSQDAELLGLTEDAVYTTMRFSRPFRSCDPHDLDITSDTVRVLAAYGLDDTLKLDQERTFVKSIFLLQVVHPDDLDVPKDTIIHDLEITDF
LIPEDDTTYACTFLPLPIVSEKHHIYKFEPKLVYHNETMVHHILVYACGNASVLPTGISDCYGADPAFSLCSQVIVGWAVGGTSYQFPDDVGVSIGTPLD
PQWIRLEIHYSNFNNLPGVYDSSGIRVYYTSQLRKYDMGVLQLGFFTFPIHFIPPGAESFLSYGLCRTEKFEEMNGAPVPDIQVYGYLLHTHLAGQALQA
VQYRNGTQLRTICKDDSYDFNLQETRDLPSRVEIKPGDELLVECHYQTLDRDFMTFGGPSTINEMCLIFLFYYPRNNISSCMGYPDIIYVAHELGEEASD
SMEGIMAMNNVEWTPESIKKAEKACKEAQQTVIIKTIDEVVENTTGWIPDIIPTPRGPCLESLGGKVEAHDKTPAGFRPSQALAGPP
```

(1) Exon coordinates

Exon	NCBI Accession	Strand	Start	End
1	AQIA01048523	-	30902	31189
2	AQIA01048523	-	30113	30259
3	AQIA01048523	-	29416	29577
4	AQIA01048523	-	29087	29170
5	AQIA01048523	-	28080	28247
6	AQIA01048523	-	27786	27888
7	AQIA01048523	-	27427	27593
8	AQIA01048523	-	26985	27076
9	AQIA01048523	-	26609	26705
10	AQIA01048523	-	26416	26475
11	AQIA01048523	-	24834	24964
12	AQIA01048523	-	23918	24032
13	AQIA01048523	-	23478	23627

14. Olive baboon (*Papio anubis*)

```
>Papio_anubis No=14 length=1764 name="Olive baboon"
ATGGCCCATGACCTTCTCTTCAGGCTTTTTCCGCTTTTGGCCCTGGCAGCCCCCTTACAAAGCAACCGCCCTGGCCCCACATCTCGCCTGCGCTATTCCA
GGTTCCTAGATCCTTCCAATGTCATTTTCCTGCGTTGGGACTTTGACCTTGAGGCTGAAATCATCAGTTTTGAGCTCCAGGTCCGGACAGCTGGCTGGGT
GGGCTTAGGTGTCACAAATCGCTACACCAACGTGGGAAGTGATCTGGTTGTTGGAGGAGTCTTGCCCAATGGCAATGTCTATTTCTCGGATCAGCACTTG
GTAAACGGAGACACTCTGAAGGAGGATGGGAGCCAGGATGCTGAGCTGCTGGAGCTGACAGAAGATGCTGTCTACACCACCATGCGCTTTTCCAGGCCCT
TCCGCTCCTGTGACCCGCATGACCTGGATATTACGAGTGACACTGTGAGGGTGCTGGCCGCCTACGGTCTGGATGACACTCTGAAGCTGGATCAGGAGCG
TACTTTTGTCAAGTCCATCTTTCTGCTACAAGTTGTCCACCCTGATGATCTGGATGTCCCCAAGGACACCATCATCCATGACTTGGAGATCACTGATTTC
CTCATTCCAGAGGATGACACCACGTACGCCTGcacctttcttcctctccccattGTTAGCGAGAAACATCACATTTACAAGTTTGAGCCTAAGTTGGTCT
ACCACAATGAGACAATGGTGCATCACATCCTGGTGTATGCCTGTGGCAATGCTAGCGTTCTCCCCACAGGCATCAGCGACTGCTATGGGGCCGATCCTGC
CTTCTCCCTCTGCTCACAGGTCATCGTGGGCTGGGCTGTCGGGGGCACTAGCTACCAGTTTCCAGATGATGTGGGTGTCTCTATTGGGACTCCCTTGGAC
CCTCAGTGGATCCGACTGGAGATTCATTACAGCAATTTTAACAACCTGCCTGGTGTGTATGATTCCTCGGGGATTCGCGTGTACTACACTTCTCAGCTGC
GCAAATATGACATGGGCGTCCTCCAGCTGGGCTTCTTCACGTTTCCCATCCACTTCATCCCCCCGGGCGCTGAGTCCTTCCTGTCCTACGGGCTGTGTAG
GACCGAGAAGTTTGAGGAGATGAATGGAGCCCCCGTGCCTGACATACAGGTGTATGGCTACCTGCTGCACACCCACTTGGCTGGACAGGCTCTGCAAGCC
GTGCAATACAGGAATGGAACACAACTTCGAACAATCTGTAAAGACGATTCCTACGACTTCAATCTGCAGGAGACTCGAGATTTGCCCTCTCGAGTGGAGA
TCAAGCTGGGAGATGAATTGCTGGTAGAATGTCACTACCAGACACTGGACCGTGACTTCATGACATTTGGAGGCCCCAGCACCATTAATGAGATGTGCCT
CATCTTCCTCTTCTACTATCCCCGAAATAACATCTCCAGCTGCATGGGGTACCCTGACATTATCTACGTGGCCCACGAGCTGGGGGAGGAGGCATCAGAC
TCCATGGAGGGAATCATGGCCATGAACAATGTCGAGTGGACCCCGGAGAGCATTAAGAAAGCTGAGAAAGCCTGCAAGGAGGCCCAGCAGACAGTGATAA
TAAAGACCATTGATGAGGTGGTGGAAAACACAACAGGCTGGATTCCAGACATCATCCCTACTCCCCGGGGGCCCTGCTTGGAGTCCTTGGGAGGCAAAGT
GGAGGCCCAGGACAAGACCCCTGCAGGCTTCAGGCCCTCTCAAGCTCTAGCAGGGCCACCCTGA

>Papio_anubis No=14 length=587 name="Olive baboon"
MAHDLLFRLFPLLALAAPLQSNRPGPTSRLRYSRFLDPSNVIFLRWDFDLEAEIISFELQVRTAGWVGLGVTNRYTNVGSDLVVGGVLPNGNVYFSDQHL
VNGDTLKEDGSQDAELLELTEDAVYTTMRFSRPFRSCDPHDLDITSDTVRVLAAYGLDDTLKLDQERTFVKSIFLLQVVHPDDLDVPKDTIIHDLEITDF
LIPEDDTTYACTFLPLPIVSEKHHIYKFEPKLVYHNETMVHHILVYACGNASVLPTGISDCYGADPAFSLCSQVIVGWAVGGTSYQFPDDVGVSIGTPLD
PQWIRLEIHYSNFNNLPGVYDSSGIRVYYTSQLRKYDMGVLQLGFFTFPIHFIPPGAESFLSYGLCRTEKFEEMNGAPVPDIQVYGYLLHTHLAGQALQA
VQYRNGTQLRTICKDDSYDFNLQETRDLPSRVEIKLGDELLVECHYQTLDRDFMTFGGPSTINEMCLIFLFYYPRNNISSCMGYPDIIYVAHELGEEASD
SMEGIMAMNNVEWTPESIKKAEKACKEAQQTVIIKTIDEVVENTTGWIPDIIPTPRGPCLESLGGKVEAQDKTPAGFRPSQALAGPP
```

(1) Exon coordinates

Exon	NCBI Accession	Strand	Start	End
1	AHZZ01107346	+	84448	84735
2	AHZZ01107346	+	85378	85524
3	AHZZ01107346	+	86060	86221
4	AHZZ01107346	+	86467	86550
5	AHZZ01107346	+	87390	87557
6	AHZZ01107346	+	87749	87851
7	AHZZ01107346	+	88044	88210
8	AHZZ01107346	+	88561	88652
9	AHZZ01107346	+	88932	89028
10	AHZZ01107346	+	89162	89221
11	AHZZ01107346	+	90673	90803
12	AHZZ01107346	+	91605	91719
13	AHZZ01107346	+	92010	92159

15. Green monkey (*Chlorocebus sabaeus*)

```
>Chlorocebus_sabaeus No=15 length=1764 name="Green monkey"
ATGGCCCATGACCTTCTCTTCAGGCTTTTTCTGCTTTTGGCCCTGGCAGCCCCCTTACAAAGCAACCGCCCTGGCCCCACCTCTCGCCTGCGCTATTCCA
GGTTCCTAGATCCTTCCAATGTCATTTTCCTGCGTTGGGACTTTGACCTTGAGGCTGAAATCATCAGTTTTGAGCTCCAGGTCCGGACAGCTGGCTGGGT
GGGCTTAGGTGTCACAAATCGCTACACCAACGTGGGAAGTGATCTGGTTGTTGGAGGAGTCTTGCCCAATGGCAATGTCTATTTCTCGGATCAGCACTTG
GTAAACGGAGACACTCTGAAGGAGGATGGGAGCCAGGATGCTGAGCTGCTGGGGCTGACAGAAGATGCTGTCTACACCACCATGCGCTTTTCCAGGCCCT
TCCGCTCCTGCGACCCGCATGACCTGGATATTACGAGTGACACTGTGAGGGTGCTGGCCGCCTACGGTCTGGATGACACTCTGAAGCTGGATCAGGAGCG
TACTTTTGTCAAGTCCATCTTTCTGCTACAAGTCGTCCACCCTGACGATCTGGATGTCCCCAAGGACACCATCATCCATGACTTGGAGATCACTGATTTC
CTCATTCCAGAGGATGACACCACGTACGCCTGCACCTTTCTTCCTCTCCCCATTGTCAGCGAGAAACATCACATTTACAAGTTTGAGCCTAAGTTGGTCT
ACCACAATGAGACAATGGTGCATCACATCCTGGTGTATGCCTGTGGCAATGCTAGCGTTCTCCCCACAGGCATCAGCGACTGCTATGGGGCCGACCCTGC
CTTCTCCCTCTGCTCACAGGTCATCGTGGGCTGGGCTGTCGGGGGCACTAGCTACCAGTTTCCAGATGATGTGGGTGTCTCTATTGGGACTCCCTTGGAC
CCTCAGTGGATCCGACTGGAGATTCATTACAGCAATTTTAACAACCTGCCTGGTGTGTATGATTCCTCGGGGATTCGCGTGTACTACACTTCTCAGCTGC
GCAAATATGATATGGGCGTCCTCCAGCTGGGCTTCTTCACGTTTCCCATCCACTTCATCCCCCCGGGCGCTGAGTCCTTCCTGTCCTATGGGCTATGTAG
GACGGAGAAGTTTGAGGAGATGAATGGAGGCCCCGTGCCTGACATACAGGTGTATGGCTACCTGCTGCACACCCACTTGGCTGGACGGGCTCTGCAAGCC
GTGCAATACAGGAATAGAACACAACTTCAAACAATCTGTAAAGACGATTCCTACGACTTCAATCTGCAGGAGACTCGAGATTTGCCCTCTCGAGTGGAGA
TCAAGCCGGGAGATGAATTGCTGGTAGAATGTCACTACCAGACACTGGACCGTGACTTCATGACATTTGGAGGCCCCAGCACCATGAATGAGATGTGCCT
CATCTTCCTCTTCTACTATCCCCGAAATAACATCTCCAGCTGCATGGGGTACCCTGACATTATCTACGTGGCCCACGAGCTGGGGGAGGAGGCATCAGAC
TCCATGGAGGGAATCATGGCCATGAACAATGTCGAGTGGACCCCGGAGAGCATTAAGAAAGCTGAGAAAGCCTGCAAGGAGGCCCAGCAGACAGTGATAA
TAAAGACCATTGATGAGGTGGTGGAAAACACAACAGGCTGGATTCCAGACATCATCCCTACTCCCCGGGGGCCCTGCCTGGAGTCCTTGGGAGGCAAAGT
GGAGGCCCAGGACAAGACCCCTGCAGGCTTCAGGCCCTCTCAAGCTCTAGCAGGGCCACCCTGA
```

```
>Chlorocebus_sabaeus No=15 length=587 name="Green monkey"
MAHDLLFRLFLLLALAAPLQSNRPGPTSRLRYSRFLDPSNVIFLRWDFDLEAEIISFELQVRTAGWVGLGVTNRYTNVGSDLVVGGVLPNGNVYFSDQHL
VNGDTLKEDGSQDAELLGLTEDAVYTTMRFSRPFRSCDPHDLDITSDTVRVLAAYGLDDTLKLDQERTFVKSIFLLQVVHPDDLDVPKDTIIHDLEITDF
LIPEDDTTYACTFLPLPIVSEKHHIYKFEPKLVYHNETMVHHILVYACGNASVLPTGISDCYGADPAFSLCSQVIVGWAVGGTSYQFPDDVGVSIGTPLD
PQWIRLEIHYSNFNNLPGVYDSSGIRVYYTSQLRKYDMGVLQLGFFTFPIHFIPPGAESFLSYGLCRTEKFEEMNGGPVPDIQVYGYLLHTHLAGRALQA
VQYRNRTQLQTICKDDSYDFNLQETRDLPSRVEIKPGDELLVECHYQTLDRDFMTFGGPSTMNEMCLIFLFYYPRNNISSCMGYPDIIYVAHELGEEASD
SMEGIMAMNNVEWTPESIKKAEKACKEAQQTVIIKTIDEVVENTTGWIPDIIPTPRGPCLESLGGKVEAQDKTPAGFRPSQALAGPP
```

(1) Exon coordinates

Exon	NCBI Accession	Strand	Start	End
1	AQIB01162307	-	30942	31229
2	AQIB01162307	-	30145	30291
3	AQIB01162307	-	29448	29609
4	AQIB01162307	-	29118	29201
5	AQIB01162307	-	28111	28278
6	AQIB01162307	-	27817	27919
7	AQIB01162307	-	27458	27624
8	AQIB01162307	-	27015	27106
9	AQIB01162307	-	26639	26735
10	AQIB01162307	-	26446	26505
11	AQIB01162307	-	24864	24994
12	AQIB01162307	-	23949	24063
13	AQIB01162307	-	23509	23658

16. Black-capped squirrel monkey (*Saimiri boliviensis*)

>Saimiri_boliviensis No=16 length=1857 name="Black-capped squirrel monkey"
ATGGCCTGTGACCTTCTCTTCAGGCTTTTTCTGCTTTTGGCCCTGGCAGCCCCCTTACAGAGCAACCGCCTTGGCCCCACATCTCGCCTGCGCTATTCCA
GGTTCCTAGATCCTTCCAATGTCATGTTCCTGCTTTGGGACTTTGACTTTGAGGCTGAAATCATCAGTTTTGAGCTCCAGGTCCAGACAGCTGGCTGGGT
GGGCTTGGGTGTCACAAATCGCTACACCAACGTGGGAAGTGATCTGGTTGTTGGAGGAGTCTTGCCTAATGGCAATGTCTATTTCTCGGATCAGCACTTG
GTAGACGAAGACACTCTGCAGGAGGATGGGAGCCAGGATGCTGAGCTCCTGGGGCTGACGGAAGATGCTGTCTACACCACCATGCGCTTTTCCAGGCCCT
TCCGCTCCTGCGACCCTCATGACCTGGATATTACGAGTGACACTGTGAGGGTGCTGGCTGCCTATGGTCTGGATGACACTCTGAAGCTGGATCGGGAGCG
TACTTTTGTCAAGTCCATCTTTCTGCTACAAGTTGTCCACCCTGATGATCTGGATGTCCCCGAGGATGCCATCATTCATGACTTGGAGATCACTGATTTC
CTTATTCCAGAGGATGACACCACGTATGCCTGCACCTTTCTTCCTCTCCCCATTGTTAGTGAGAAGCATCACATTTACAAGTTTGAGCCCAAGTTGGTCT
ACCACAATGAGACAATGGTGCATCACATCCTGGTGTATGCCTGCGGCAATGCTAGCGTTCTTCCCACAGGCATCAGTGACTGCTATGGGGCCGACCCTGC
CTTCTCCCTCTGCTCACAGGTCATCGTGGGCTGGGCTGTTGGGGGCACTAGCTACCAGTTTCCAGATGATGTGGGCATCTCTATTGGGACCCCCTTGGAC
CCTCAGTGGATTCGACTGGAGATTCATTACAGCAATTTTAACAACCTTCCTGGTGTGTATGACTCCTCGGGGATTCGTGTGTACTACACTGCTCAGCTCC
GCAAATATGACATGGGTGTCCTCCAGCTGGGCTTCTTCACGTTCCCCATCCACTTCATCCCCCCGGGCGCTGAGTCCTTCATGTCCTATGGGCTGTGTAG
GACGGAGAAGTTTGAGGAGATCAATGGAGCCCCCGTGCCTGACATACAGGTTTATGGCTACCTGCTGCACACTCACTTGGCTGGAAGGGCTTTGCAAGCT
GTGCAGTACAGGAATGGAACACAACTTCGAACAATCTGTAAAGACGATTCCTATGACTTCAATCTGCAGGAGACTCGAGATTTGCCTTCTCGAGTGGAGA
TCAAGCCGGGAGATGAATTGCTGGTAGAATGTCACTACCAGACACTGGACCGTGACGCCATGACATTTGGAGGCCCCAGCACCATTAATGAGATGTGCCT
CATCTTCCTCTTCTACTATCCCCGAAATAACATCTCCAGCTGCATGGGGTACCCTGACATTATCTACGTGGCCCATGAGCTGGGGGAGGAGGCATCAGAC
TCCATGGAGGGAATTATGGCCATGAACAATGTTGAGTGGACCCCAGAGAGCATTAAGAAGGCTGAGAAAGCCTGCAAGGAGGCCCAGCAGACAGTGATAA
TAAAGACCATTGACGAGGTGGTGGAAAACACAACAGGATGGATTCCAGACATCAGCCCTGCTCCCCAGGGGCCCTGCTTGGAGTCCTCTGGAGGCAAAGT
GGAGGCCCAGGACAAGACCCCTGCGGGCTTCAGGGCTTCACCCGTGGCGCTCTCGAGCTCCAGCAAGGCCACCCTGAGGCACCTCCTGCTGGCAGCTGTC
CTACTGGTGCAGAGCACACTGTCCTGGCTCCTGGCCACGCTGCAGACTGGAGTCTGA

>Saimiri_boliviensis No=16 length=618 name="Black-capped squirrel monkey"
MACDLLFRLFLLLALAAPLQSNRLGPTSRLRYSRFLDPSNVMFLLWDFDFEAEIISFELQVQTAGWVGLGVTNRYTNVGSDLVVGGVLPNGNVYFSDQHL
VDEDTLQEDGSQDAELLGLTEDAVYTTMRFSRPFRSCDPHDLDITSDTVRVLAAYGLDDTLKLDRERTFVKSIFLLQVVHPDDLDVPEDAIIHDLEITDF
LIPEDDTTYACTFLPLPIVSEKHHIYKFEPKLVYHNETMVHHILVYACGNASVLPTGISDCYGADPAFSLCSQVIVGWAVGGTSYQFPDDVGISIGTPLD
PQWIRLEIHYSNFNNLPGVYDSSGIRVYYTAQLRKYDMGVLQLGFFTFPIHFIPPGAESFMSYGLCRTEKFEEINGAPVPDIQVYGYLLHTHLAGRALQA
VQYRNGTQLRTICKDDSYDFNLQETRDLPSRVEIKPGDELLVECHYQTLDRDAMTFGGPSTINEMCLIFLFYYPRNNISSCMGYPDIIYVAHELGEEASD
SMEGIMAMNNVEWTPESIKKAEKACKEAQQTVIIKTIDEVVENTTGWIPDISPAPQGPCLESSGGKVEAQDKTPAGFRASPVALSSSSKATLRHLLLAAV
LLVQSTLSWLLATLQTGV

(1) Exon coordinates

Exon	UCSC Chromosome	Strand	Start	End
1	saiBol1 JH378140	+	8475596	8475883
2	saiBol1 JH378140	+	8476600	8476746
3	saiBol1 JH378140	+	8477288	8477449
4	saiBol1 JH378140	+	8477695	8477778
5	saiBol1 JH378140	+	8478606	8478773
6	saiBol1 JH378140	+	8478965	8479067
7	saiBol1 JH378140	+	8479261	8479427
8	saiBol1 JH378140	+	8479771	8479862
9	saiBol1 JH378140	+	8480147	8480243
10	saiBol1 JH378140	+	8480378	8480437
11	saiBol1 JH378140	+	8481914	8482044
12	saiBol1 JH378140	+	8482833	8482947
13	saiBol1 JH378140	+	8483250	8483492

17. Common marmoset (*Callithrix jacchus*)

```
>Callithrix_jacchus No=17 length=1857 name="Common marmoset"
ATGGCCTGTGACCTTCTCTTCAGGCTTTTTCTGCTTTTGGCCCTGGCAGCTGCCTTACAAAGCAACCGCCTTGGCCCCACATCTCGCCTGCGCTATTCCA
GGTTCCTGGATCCTTCCAGTGTCATGTTCCTGCTTTGGGACTTTGACCTTGAAGCTGAAATCATCAGTTTTGAGCTCCAGGTCCGGACAGCTGGCTGGGT
GGGCTTGGGTGTCACAAATCGCTACACCAACGTGGGAAGTGATCTGGTTGTTGGAGGAGTCTTGCCTAATGGCAATGTCTATTTCTCGGATCAGCACTTG
GTAGACGAAGACACTCTGCAGGAGGATGCGAGCCAGGATGCTGAGCTGCTGGGGCTGACGGAAGATGCTATCTACACCACCATGCGCTTTTCCAGGCCCT
TCCGCTCCTGCGACCCTCATGACCTAGATATTACGAGTGACACTGTGAGGGTGCTGGCCGCCTATGGCCTGGATGACACTCTGAAGCTGGATCGGGAGCA
TACTTTTGTTAAGTCCATCTTTCTGCTACAAGTTGTTCACCCTGACGATCTGGATGTCCCCGAGGATGCCATCATTCATGACTTGGAGATCACTGATTTC
CTTATTCCAGAGGATGACACCACGTATGCCTGTACCTTTCTTCCTCTCCCCATTGTTAGTGAGAAGCATCACATTTACAAGTTTGAGCCCAAGTTGGTCT
ACCACAATGAGACAATGGTGCATCACATCCTGGTGTATGCCTGTGGCAATTCTAGCGTTCTTCCCACAGGCATCAGCGACTGCTACGGGGCTGACCCTGC
CTTCTCCCTCTGCTCACAGGTCATCGTGGGCTGGGCTGTTGGGGGCACTAGCTACCAGTTTCCAGATGATGTGGGCATCTCTATTGGGACCCCCTTGGAC
CCTCAGTGGATCCGACTGGAGATTCATTACAGCAATTTTAACAACCTTCCTGGTGTGTATGACTCCTCGGGGATTCGTGTGTACTACACTGCTCAGCTCC
GCAAATATGACATGGGAGTCCTCCAGCTGGGCTTCTTCACATTCCCCATCCACTTCATCCCCCCGGGCGCTGAGTCCTTCATGTCCTATGGGCTGTGTAG
GACGGAGAAGTTTGAGGAGATGAATGGAGCCCCCGTGCCTGACATACAGGTGTATGGTTACCTGCTGCACACTCACTTGGCTGGAAGGGCTCTGCAAGCT
GTGCAGTACAGGAATGGAACACAACTTCGAACAATCTGTAAAGATGATTCCTATGACTTCAATCTGCAGGAGACTCGAGATTTGCCTTCTCGAGTGGAGA
TCAAGCCGGGAGATGAATTGCTGGTAGAATGTCACTACCAGACACTGGACCGTGACGCCATGACATTTGGAGGCCCCAGCACCATTAATGAGATGTGCCT
CATCTTCCTCTTCTACTATCCCCGAAATAACATCTCCAGCTGCATGGGGTACCCTGACATTATCTATGTGGCCCATGAGCTGGGGGAGGAGGCATCAGAC
TCCATGGAGGGAATGATGGCCATGAACAATGTCGAGTGGACCCCAGAGAGCATTAAGAAGGCTGAGAAAGCCTGCAAGGAGGCCCAGCAGACAGTGATAA
TAAAGACCATTGATGAGGTGGTGGAAAACACAACGGGATGGATTCCAGACATCATCCCTACTCCGCTGGGGCCCTGCTTGGAGTCCTCTGGAGGCAAAGT
GGAGGCCCAGGACAAGACTCCTGCGGGCTTCAGGTCTTCACCTGTGGCCCTCTCGAGCTCCAGCAAGGCCACCCTGAGGCGTCTCCCGCTGGCAGCTGTC
CTGCTAGTGCAGAGCACACTCTCGGGGCTCCTGGCCATGCTGCAGACTAGAGTCTGA
```

```
>Callithrix_jacchus No=17 length=618 name="Common marmoset"
MACDLLFRLFLLLALAAALQSNRLGPTSRLRYSRFLDPSSVMFLLWDFDLEAEIISFELQVRTAGWVGLGVTNRYTNVGSDLVVGGVLPNGNVYFSDQHL
VDEDTLQEDASQDAELLGLTEDAIYTTMRFSRPFRSCDPHDLDITSDTVRVLAAYGLDDTLKLDREHTFVKSIFLLQVVHPDDLDVPEDAIIHDLEITDF
LIPEDDTTYACTFLPLPIVSEKHHIYKFEPKLVYHNETMVHHILVYACGNSSVLPTGISDCYGADPAFSLCSQVIVGWAVGGTSYQFPDDVGISIGTPLD
PQWIRLEIHYSNFNNLPGVYDSSGIRVYYTAQLRKYDMGVLQLGFFTFPIHFIPPGAESFMSYGLCRTEKFEEMNGAPVPDIQVYGYLLHTHLAGRALQA
VQYRNGTQLRTICKDDSYDFNLQETRDLPSRVEIKPGDELLVECHYQTLDRDAMTFGGPSTINEMCLIFLFYYPRNNISSCMGYPDIIYVAHELGEEASD
SMEGMMAMNNVEWTPESIKKAEKACKEAQQTVIIKTIDEVVENTTGWIPDIIPTPLGPCLESSGGKVEAQDKTPAGFRSSPVALSSSSKATLRRLPLAAV
LLVQSTLSGLLAMLQTRV
```

(1) Exon coordinates

Exon	UCSC Chromosome	Strand	Start	End
1	calJac3 chr8	+	103864921	103865208
2	calJac3 chr8	+	103865927	103866073
3	calJac3 chr8	+	103866603	103866764
4	calJac3 chr8	+	103867010	103867093
5	calJac3 chr8	+	103867903	103868070
6	calJac3 chr8	+	103868261	103868363
7	calJac3 chr8	+	103868557	103868723
8	calJac3 chr8	+	103869072	103869163
9	calJac3 chr8	+	103869448	103869544
10	calJac3 chr8	+	103869677	103869736
11	calJac3 chr8	+	103871366	103871496
12	calJac3 chr8	+	103872292	103872406
13	calJac3 chr8	+	103872707	103872949

18. Philippine tarsier (*Tarsius syrichta*)

```
>Tarsius_syrichta No=18 length=1857 name="Philippine tarsier"
ATGTCCTGTGCCCTTTTCTTCAGGCTTTTCATGTTTCTGGCCCTGATAGCACCCTCCCTAGGCAACCGTCTAGGTCTCACATCTCGCCTGCGTTTTTCCA
GGTTCCTAGATCCTTCCAATGTCGTTTTCCTGCGCTGGGACATTGACCTTAAGGCTGAGATAATCACTTTCGAGCTCCAGGTCCGGACGGCTGGCTGGGT
GGGCTTGGGTATCAAAAATCGCTACACCAACGTGGGAAGTGATCTGGTCGTTGGAGGAGTCCTGCCTGATGGCAATGTTTATTTTTCGGACCAGCACCTC
GTGGACGATGACACCCTGGAAGAGGACAGGAGCCAGGATGCTGAGTTGCAGGGGCTGACGGAAGATGCTGGCTACACCACCATGCGCTTTTCAAGACCCT
TCCGCTCCTGTGACCCTCATGACCTCGATATTACGAGTGACACTGTGAGGGTGCTGGCTGCCTTTGGCCTGGATGACACTCTGAAGCTGGATCGGGAACG
TACTTTTGTCAAGTCCATCTTTCTGCTACAAGTAGTCCACCCTGATGATCTGGATTACCCCGAGGACACCATCATTCATGACTTGGAGATCACTGAATTC
CTCATTCCAGAAGATGACACCACGTATGCCTGCACCTTTCTCCCTCTCCCTATCGTTAACAAGAAGCATCATATCTACAAGTTTGAACCCAAGTTGATCT
ACCACAATGAGACTATGGTGCATCACATCCTGGTGTATGCCTGTGGCAATGCCAGTGTTCTCCCCAAGGGCATCAGCGACTGCTATGGGGCCGACCCTGC
CTTTTCCCTCTGCTCGCAGGTCATCGTGGGCTGGGCCGTTGGGGGCACTAGTTACCAGTTTCCAGATGACGTAGGTATCTCTATTGGGACCCCCTTGGAC
CCTCAGTGGATCCGACTGGAGATTCATTATAGCAATTTTCACAACCTTCCTGGTGTGTATGACTCCTCGGGTATTCGAGTGTACTACAGTTCTCAGCTGC
GCAAATACGACATGGGCGTCCTCCAATTGGGCTTCTTCACATTCCCCATCCACTTCATACCCCCAGGCGCTGAGTCCTTCATGTCCTACGGACTGTGTAA
AACGGATAAGTTTGAGGAGATGAATGGGGTCCCATTTCCTGACATACAGGTATATGGCTACCTGCTGCATACCCACTTGGCTGGGCGGGCTCTGCAGGCT
GTGCAATACAGAAATGGAACACAACTTCGAACAATCTGTAAAGATGATTCCTATGACTTCAATCTGCAGGAAACTCGAGATTTGCCTTCTCGAGTGGAGA
TCAAGCCGGGAGATGAATTGCTGGTAGAATGTCACTATCAGACACTGGACCGTGACTCCATGACTTTTGGAGGTCCCAGCACCATTAATGAGATGTGTCT
CGTCTTTCTCTTCTACTATCCCCGAAATAACATCTCCAGCTGCATGGGGTACCCTGACATCATCTATGTGGCTCATGAACTGGGGGAAGAGGCATCAGAC
TCCATGGAGGGTATCATGGCCATAAACAATGTTGAGTGGACTCCAGAGAGTATTAAGAAGGCTGAGAAAGCCTGCAAGGAGGCCCAGCAGATGGTGATAA
TAAAGACCATTGATGAGGTGGTGGAAAACACAACAGGCTGGATTCCAGACATCACCCCTCCTCCTCGGGGGCCCTGTTTAGAGTCCTCTGGAGGCAAAGT
GGAGCCCCAGGACAAGACCCCTGCAGGCTTCAGGGCTGCACCAATGGCCCTCTCAGGCTCCAGCATTGCTACCCTGAAGCGCCTCCCGCTAGCGACCTTC
CTGTTGGTTCAGGGAACCCTCTCCTGGCTCCTTGCCATGCTGCAGCCTGGAAACTGA

>Tarsius_syrichta No=18 length=618 name="Philippine tarsier"
MSCALFFRLFMFLALIAPSLGNRLGLTSRLRFSRFLDPSNVVFLRWDIDLKAEIITFELQVRTAGWVGLGIKNRYTNVGSDLVVGGVLPDGNVYFSDQHL
VDDDTLEEDRSQDAELQGLTEDAGYTTMRFSRPFRSCDPHDLDITSDTVRVLAAFGLDDTLKLDRERTFVKSIFLLQVVHPDDLDYPEDTIIHDLEITEF
LIPEDDTTYACTFLPLPIVNKKHHIYKFEPKLIYHNETMVHHILVYACGNASVLPKGISDCYGADPAFSLCSQVIVGWAVGGTSYQFPDDVGISIGTPLD
PQWIRLEIHYSNFHNLPGVYDSSGIRVYYSSQLRKYDMGVLQLGFFTFPIHFIPPGAESFMSYGLCKTDKFEEMNGVPFPDIQVYGYLLHTHLAGRALQA
VQYRNGTQLRTICKDDSYDFNLQETRDLPSRVEIKPGDELLVECHYQTLDRDSMTFGGPSTINEMCLVFLFYYPRNNISSCMGYPDIIYVAHELGEEASD
SMEGIMAINNVEWTPESIKKAEKACKEAQQMVIIKTIDEVVENTTGWIPDITPPPRGPCLESSGGKVEPQDKTPAGFRAAPMALSGSSIATLKRLPLATF
LLVQGTLSWLLAMLQPGN
```

(1) Exon coordinates

Exon	NCBI Accession	Strand	Start	End
1	ABRT02374379	-	62892	63179
2	ABRT02374379	-	61841	61987
3	ABRT02374379	-	61174	61335
4	ABRT02374379	-	60843	60926
5	ABRT02374379	-	59904	60071
6	ABRT02374379	-	59611	59713
7	ABRT02374379	-	59281	59447
8	ABRT02374379	-	58859	58950
9	ABRT02374379	-	58482	58578
10	ABRT02374379	-	58288	58347
11	ABRT02374379	-	56621	56751
12	ABRT02374379	-	55731	55845
13	ABRT02374379	-	55193	55435

19. Gray mouse lemur (*Microcebus murinus*)

```
>Microcebus_murinus No=19 length=1857 name="Gray mouse lemur"
ATGGCCTGCACCCTTCTCTTCGGGCTTTTCCTGCTTCTGGGCCTGGAAGCTCCCTCCCCTGGCAACTGCCTCGCTCCCACATCTCGCCTGCGTTATTCCA
GGTTCCTAGATCCTTCCAACGTCATTTTCCTGCGCTGGGACTTTGACCTTAAGGCTGAAGTCATCACTTTTGAGCTCCAAGTCCGGACAGCTGGCTGGGT
GGGCTTGGGTGTCACAAGTCGCTACACCAGAGCGGGAAGTGATCTGGTTGTTGGAGGAGTCTTGCCCGATGGCAATGTCTATTTTTCGGACCAGCACCTG
GTAGACGAAGACACCCTGAAAGAGGACGGGAGCCAGGATGCTGAGCTGCACGGGCTGACGCAAGACGCTGTCTACACCACCATGCGCTTTTCCAGGCCCT
TCCGCTCCTGCGACCCCCATGACCAGGACATCACGGCTGACACCATGAGGGTGCTGGCCGCCTACGGCCTGGATGACACTCTGAAGCTGGATCGGGACCG
TACCTTTGTCAAGTCCATCTTCCTGTTACAAGTAGTCCACCCTGACGATCTGGACGTCCCTGAGGACACCATCATCCATGACTTGGAGATCACTAATTTC
CTCATCCCAGAGGACGACACCACGTACGCCTGCACGTTTCTCCCTCTCCCCATCGTTAGCAAGAAGCATCACATCTACAAGTTTGAGCCCAAGTTAATGG
CCCGCAACGAGACGATGGTGCATCACATCCTGGTGTACGCCTGCGGCAACGCCAGCGCCCTCCCGACCGGCATCAGCGACTGCTACGGGGCCGACCCTGC
CTTCTCCCTCTGCTCGCAGATCATCGTGGGCTGGGCTGTCGGCGGCACAAGTTACCAGTTTCCAGATGACGTGGGCATCTCTATTGGGACCCCCTTGGAC
CCTCAGTGGATCCGACTGGAGGTTCATTACAGCAACTTTCACAACCGTCCTGGTGTGTACGACTCCTCAGGCATTCGCATGTACTACAGCTCTCACCTGC
GCAAATACGACATGGGCGTCCTCCAGCTGGGCTTCTTCACTTTCCCCATCCACTTCATACCCCCGGGCTCTGAGTCCTTCATGTCCTACGGGCTGTGTAA
GACGGAGAAGTTTGAAGAGATGAACGGGGGCCCCGTGCCCGACATACAGGTGTATGGCTACCTGCTGCACACCCACCTGGCGGGGCGGGCTCTGCAGGCC
GTGCAATACAGAAATGGAACACAACTCCAAACCATCTGCAAAGACGATTCCTATGACTTCAACCTGCAGGAGACTCGAGATCTACCTGCCCGCGTGGTGA
TCAAGCCGGGAGATGAGTTGCTGGTGGAATGTCACTACCAGACACTGGACCGTGACTCCCTGACTTTCGGAGGTCCCAGCACCATTAATGAGATGTGCCT
CATCTTCCTCTTCTACTATCCCCGAAATAACATCTCCAGCTGCATGGGGTACCCCGACATCATCTACGTGGCCCACGAGCTGGGGCAGGAGGTATCCGAC
TCCATGGAGGGCATGATGGCCATGAACAACGTCGAGTGGACCCCAGAGAACATCAAGAAGGCGGAGAAAGCCTGCAGGGAGGCCAGGCAGACGGTGCTAA
TAAAGACCATTGACGAGGTGGTGGAAAACACAACAGGCTGGATTCCGGACATCACCCCTACTCCTCGGGGGCCCTGCTTGGAGTCCTCTGGAGGCAAAGT
GGAGCCCCAGGACAAGACCCCTGCAGGCTTCAGGGCTGCACCGGTGGCCCTCTCCAGCTCCGGCCCGGCCGCCCTGCGGCGCCTGCCCCTGGCGGCCCAC
TTGCTGGTGCAGGGCGCCCTCTCCTGGCTGCTGGGCAGGCTGCACACGGGAGGCTGA

>Microcebus_murinus No=19 length=618 name="Gray mouse lemur"
MACTLLFGLFLLLGLEAPSPGNCLAPTSRLRYSRFLDPSNVIFLRWDFDLKAEVITFELQVRTAGWVGLGVTSRYTRAGSDLVVGGVLPDGNVYFSDQHL
VDEDTLKEDGSQDAELHGLTQDAVYTTMRFSRPFRSCDPHDQDITADTMRVLAAYGLDDTLKLDRDRTFVKSIFLLQVVHPDDLDVPEDTIIHDLEITNF
LIPEDDTTYACTFLPLPIVSKKHHIYKFEPKLMARNETMVHHILVYACGNASALPTGISDCYGADPAFSLCSQIIVGWAVGGTSYQFPDDVGISIGTPLD
PQWIRLEVHYSNFHNRPGVYDSSGIRMYYSSHLRKYDMGVLQLGFFTFPIHFIPPGSESFMSYGLCKTEKFEEMNGGPVPDIQVYGYLLHTHLAGRALQA
VQYRNGTQLQTICKDDSYDFNLQETRDLPARVVIKPGDELLVECHYQTLDRDSLTFGGPSTINEMCLIFLFYYPRNNISSCMGYPDIIYVAHELGQEVSD
SMEGMMAMNNVEWTPENIKKAEKACREARQTVLIKTIDEVVENTTGWIPDITPTPRGPCLESSGGKVEPQDKTPAGFRAAPVALSSSGPAALRRLPLAAH
LLVQGALSWLLGRLHTGG
```

(1) Exon coordinates

Exon	NCBI Accession	Strand	Start	End
1	ABDC01141541	+	1314	1601
2	ABDC01141541	+	2556	2702
3	ABDC01141541	+	3202	3363
4	ABDC01141541	+	3610	3693
5	ABDC01141541	+	4464	4631
6	ABDC01141541	+	4821	4923
7	ABDC01141541	+	5105	5271
8	ABDC01141541	+	5598	5689
9	ABDC01141541	+	5968	6064
10	ABDC01141541	+	6194	6253
11	ABDC01141541	+	7664	7794
12	ABDC01141541	+	8566	8680
13	SRP021223	predicted by assembling transcriptome data		

(2) The exon 13 coding sequence was predicted by assembling transcriptome data using CAP3. The exon 13 is marked in red.

```
                          .    :    .    :    .    :    .    :    .    :    .    :
SRR832933.149494043.+ AAAGCCTGCAGGGAGGCCAGGCAGACGGTGCTAATAAAGACCATTGACGAGGTGGTGGAA
SRR832933.552683387.+        GCAGGGAGGCCAGGCAGACGGTGCTAATAAAGACCATTGACGAGGTGGTGGAA
SRR832933.213258248.+        GCAGGGAGGCCAGGCAGACGGTGCTAATAAAGACCATTGACGAGGTGGTGGAA
SRR832933.221827620.+        GCAGGGAGGCCAGGCAGACGGTGCTAATAAAGACCATTGACGAGGTGGTGGAA
SRR832933.523425517.+                               CTAATAAAGACCATTGACGAGGTGGTGGAA
SRR832933.213258248.-                                           ATTGACGAGGTGGTGGAA
SRR832933.684016774.+                                                 GAGGTGGTGGAA
SRR832933.221827620.-                                           ATTGACGAGGTGGTGGAA

consensus             AAAGCCTGCAGGGAGGCCAGGCAGACGGTGCTAATAAAGACCATTGACGAGGTGGTGGAA

                          .    :    .    :    .    :    .    :    .    :    .    :
SRR832933.149494043.+ AACACAACAGGCTGGATTCCGGACATCACCCCTACTCCTCG
SRR832933.552683387.+ AACACAACAGGCTGGATTCCGGACATCACCCCTACTCC
SRR832933.213258248.+ AACACAACAGGCTGGATTCCGGACATCACCCCTACTCCTCGGGG
SRR832933.221827620.+ AACACAACAGGCTGGATTCCGGACATCACCCCTACTCCTCGGGG
SRR832933.523425517.+ AACACAACAGGCTGGATTCCGGACATCACCCCTACTCCTCGGGGGCCCTGCTTGGAGTCC
SRR832933.213258248.- AACACAACAGGCTGGATTCCGGACATCACCCCTACTCCTCGGGGGCCCTGCTTGGAGTCC
```

```
SRR832933.684016774.+ AACACAACAGGCTGGATTCCGGACATCACCCCTACTCCTCGGGGGCCCTGCTTGGA
SRR832933.221827620.- AACACAACAGGCTGGATTCCGGACATCACCCCTACTCCTCGGGGGCCCTGCTTGGAGTCC
SRR832933.322746528.-                     GGACATCACCCCTACTCCTCGGGGGCCCTGCTTGGAGTCC
SRR832933.574719240.-                     GGACATCACCCCTACTCCTCGGGGGCCCTGCTTGGAGTCC
SRR832933.684016774.-                                    TCCTCGGGGGCCCTGCTTGGAGTCC
SRR832933.346976807.+                                                    TTGGAGTCC
SRR832933.493113118.+                                                    TTGGAGTCC
                      ____________________________________________________________
consensus             AACACAACAGGCTGGATTCCGGACATCACCCCTACTCCTCGGGGGCCCTGCTTGGAGTCC

                          .    :    .    :    .    :    .    :    .    :    .    :
SRR832933.523425517.+ TCTGGAGGCAA
SRR832933.213258248.- TCTGGAGGCAAAGTGGAGCCCCA
SRR832933.221827620.- TCTGGAGGCAAAGTGGAGCCCCA
SRR832933.322746528.- TCTGGAGGCAAAGTGGAGCCCCAGGACAAGACCCCTGCAGGCTTCAGGGCTGCACCGGTG
SRR832933.574719240.- TCTGGAGGCAAAGTGGAGCCCCAGGACAAGACCCCTGCAGGCTTCAGGGCTGCACCGGTG
SRR832933.684016774.- TCTGGAGGCAAAGTGGAGCCCCAGGACAAGACCCCTGCAGGCTTCAGGGCTGCACCGGTG
SRR832933.346976807.+ TCTGGAGGCAAAGTGGAGCCCCAGGACAAGACCCCTGCAGGCTTCAGGGCTGCACCGGTG
SRR832933.493113118.+ TCTGGAGGCAAAGTGGAGCCCCAGGACAAGACCCCTGCAGGCTTCAGGGCTGCACCGGTG
SRR832933.552683387.- TCTGGAGGCAAAGTGGAGCCCCAGGACAAGACCCCCGCAGGCTTCAGGGCTGCACCGGTG
SRR832933.523425517.-       GGCAAAGTGGAGCCCCAGGACAAGACCCCCGCAGGCTTCAGGGCTGCACCGGTG
SRR832933.275417789.-                               ACCCCCGCAGGCTTCAGGGCTGCACCGGTN
                      ____________________________________________________________
consensus             TCTGGAGGCAAAGTGGAGCCCCAGGACAAGACCCCTGCAGGCTTCAGGGCTGCACCGGTG

                          .    :    .    :    .    :    .    :    .    :    .    :
SRR832933.322746528.- G
SRR832933.574719240.- G
SRR832933.684016774.- GCCCTCTCCAGCTCCG
SRR832933.346976807.+ GCCCTCTCCAGCTCCGGCCCGGCCGCCCTGCG
SRR832933.493113118.+ GCCCTCTCCAGCTCCGGCCCGGCCGCCCTGCG
SRR832933.552683387.- GCCCTCTCCAGCTCCGGCCCGGCCGCCCTGCGGCGCCTGCC
SRR832933.523425517.- GCCCTCTCCAGCTCCGGCCCGGCCGCCCTGCGGCGCCTGCC
SRR832933.275417789.- GCGCTCTCCAGCTCCGGCCCGGCCGCCCTGCGGCGCCTGCCCCTGGCGGCCCACTTGCTG
SRR832933.346976807.-                 GCCCGGCCGCCCTGCGGCGCCTGCCCCTGGCGGCGCGCTTGCTG
SRR832933.493113118.-                 GCCCGGCCGCCCTGCGGCGCCTGCCCCTGGCGGCCCACTTGCTG
                      ____________________________________________________________
consensus             GCCCTCTCCAGCTCCGGCCCGGCCGCCCTGCGGCGCCTGCCCCTGGCGGCCCACTTGCTG

                          .    :    .    :    .    :    .    :    .    :    .    :
SRR832933.275417789.- GTGCAGGGCGC
SRR832933.346976807.- GTGCAGGGCGCCCTCTCCTGGCTGCTGGGCAGGCTGCACACGGGAGGCTGATTCTGC
SRR832933.493113118.- GTGCAGGGCGCCCTCTCCTGGCTGCTGGGCAGGCTGCACACGGGAGGCTGATTCTGC
                      ____________________________________________________________
consensus             GTGCAGGGCGCCCTCTCCTGGCTGCTGGGCAGGCTGCACACGGGAGGCTGATTCTGC
```

20. Aye-aye (*Daubentonia madagascariensis*)

```
>Daubentonia_madagascariensis No=20 length=1857 name="Aye-aye"
ATGGCCTGTGCCCTTCTCTTCAGGCTTCTCCTGCTTCTGGCCCTGGAAGCCCCCTCCCCAGGCAACTGCCTCAGTCATACATCTCGCCTGCGTTATTCCA
GGTTCCTAGATCCTTCCAATGTCATTTTCCTGCGCTGGGACTTTGACCTTGAGGCTGAAGTCATCACTTTTGAGCTTCAGGTCCGGACAGCTGGCTGGGT
GGGCTTGGGTGTCACAAATCGCTACACCAGAGTGGGAAGTGATCTGGTTGTTGGAGGAGTCTTGCCCGATGGCAATGTCTATTTTTCGGATCAGCACCTG
GTGGATGAAGACACCCTGAAGGAGGATGGGAACCAGGATGCAGAGCTGCACGGGCTGACGGAAGGTGCTGTCTACACCACCATGCGCTTTTCCAGGCCCT
TCCGCTCCTGCGACCGCCATGACCAGGACATCACGGGTGACACTGTGAGGGTGCTGGCCGCCTACGGCCTGGATGACACTCTGAAGCTGGATCGGAACCG
CACCTTTGTCAAGTCCGTCTTTCTGCTGCAAGTAGTCCACCCTGACGATCTGGATGTCCCCGAGGACACCATCATCCACGACTTGGAGATCACTGATTTC
CTCATTCCAGAGGATGACACCACGTACGCCTGCACGTTTCTCCCTCTCCCCATCGTTAGCAAGAAGCATCACATCTACAAGTTTGAGCCCACGTTGGTGG
ACCACAATGAGACAATGGTGCATCACATCCTGGTGTACGCCTGCGGCAACGCCAGCGCTCTCCCGACTGGCATCAGCGACTGCTACGGGGCCGACCCTGC
CTTCTCCCTCTGCTCGCAGGTCATCGTGGGCTGGGCTGTCGGGGGCACCAGTTACCAGTTTCCAGATGACGTGGGCATCTCTATTGGGACCCCCTTGGAC
CCTCAGTGGATCCGACTGGAGGTTCATTATAGCAACTTTCACAACCTTCCTGGTGTGTACGACTCCTCAGGAATTCGAGTGTACTACAGCTCTCAGCTGC
GCAAATACGACATGGGCGTCCTGCAGCTGGGCTTCTTCACTTTCCCCATCCACTTCATACCCCCGGGCGCTGAGTCCTTCATGTCCTACGGGCTGTGTAA
GACGGAGAAGTTTGAAGAGATGAATGGGGGCCCTGTGCCTGACATACAGGTGTATGGCTACCTGCTGCACACCCACCTGGCAGGGCGGGCTCTGCAGGCC
GTGCAATACAGAAATGGAACACAACTCCAAACCATCTGTAAAGACGATTCCTATGACTTCAATCTGCAGGAGACTCGAGATCTACCTTCCCGAGTGGTGA
TCAAGCCGGGAGATGAGTTGCTTGTAGAATGTCACTACCAGACACTGGACCGTGACTCCATGACTTTTGGAGGTCCCAGCACCATTAATGAGATGTGCCT
CATCTTCCTCTTCTACTATCCCCGAAATAACATCTCCAGCTGCATGGGGTACCCCGACATCATCTACGTGGCCCATGAGCTGGGGGAGGAGGCATCTGAC
TCCATGGAGGGCATGATGGCCATGAGCAACGTTGAGTGGACCCCAGAGAGCATTAAGAAGGCTGAGAAAGCCTGCAAGGAGGCCAGGCAGACGGTGATAA
TAAAGACCATTGATGAGGTGGTGGAAAACACAACAGGCTGGATTCCAGACATCATCCCTACTCCTCGGGGGCCCTGCCTGGAGTCCTCTGGAGGCAAAGT
GGAGCCCCAGGACAAGACTCCCGCAGGCTTCAGGGCCGCACCGGTGGCCCTCTCGGGCTCCAGCACTGCTTCCCTGAAGCGCCTCCCCCTGGCGGCCCAC
TTGCTGGTGCAGGGCACCCTGTCCTGGCTCCTTGCCACGCTGCAGACGGGAGGCTGA
```

```
>Daubentonia_madagascariensis No=20 length=618 name="Aye-aye"
MACALLFRLLLLLALEAPSPGNCLSHTSRLRYSRFLDPSNVIFLRWDFDLEAEVITFELQVRTAGWVGLGVTNRYTRVGSDLVVGGVLPDGNVYFSDQHL
VDEDTLKEDGNQDAELHGLTEGAVYTTMRFSRPFRSCDRHDQDITGDTVRVLAAYGLDDTLKLDRNRTFVKSVFLLQVVHPDDLDVPEDTIIHDLEITDF
LIPEDDTTYACTFLPLPIVSKKHHIYKFEPTLVDHNETMVHHILVYACGNASALPTGISDCYGADPAFSLCSQVIVGWAVGGTSYQFPDDVGISIGTPLD
PQWIRLEVHYSNFHNLPGVYDSSGIRVYYSSQLRKYDMGVLQLGFFTFPIHFIPPGAESFMSYGLCKTEKFEEMNGGPVPDIQVYGYLLHTHLAGRALQA
VQYRNGTQLQTICKDDSYDFNLQETRDLPSRVVIKPGDELLVECHYQTLDRDSMTFGGPSTINEMCLIFLFYYPRNNISSCMGYPDIIYVAHELGEEASD
SMEGMMAMSNVEWTPESIKKAEKACKEARQTVIIKTIDEVVENTTGWIPDIIPTPRGPCLESSGGKVEPQDKTPAGFRAAPVALSGSSTASLKRLPLAAH
LLVQGTLSWLLATLQTGG
```

(1) Exon coordinates

Exon	NCBI Accession	Strand	Start	End
1	AGTM010366419	+	123	410
2	SRP018575	predicted by assembling WGS data		
3	AGTM011664969	+	20	181
4	AGTM011664969	+	428	511
5	AGTM011664969	+	1339	1506
6	AGTM011664969	+	1696	1798
7	AGTM011903334	+	82	248
8	AGTM011903334	+	575	666
9	AGTM011903334	+	936	1032
10	AGTM011903334	+	1166	1225
11	AGTM011903334	+	2657	2787
12	AGTM010735679	+	120	234
13	SRP018575	predicted by assembling WGS data		

(2) The exon 2 sequence was predicted by assembling WGS data using CAP3. The exon 2 is marked in red.

```
                              .    :    .    :    .    :    .    :    .    :    .    :
SRR709449.85843889.2- TGATCTTCACTGGACCCCAGGATCAGCACCTGGTGGATGAAGACACCCTGAAGGAGGATG
SRR709445.61659285.2+     CTTCACTGGACCCCAGGATCAGCACCTGGTGGATGAAGACACCCTGAAGGAGGATG
SRR709439.46592067.1-     CTTCACTGGACCCCAGGATCAGCACCTGGTGGATGAAGACACCCTGAAGGAGGATG
SRR709440.57295999.2-      TTCACTGCACCCCAGGATCAGCACCTGGTGGATGAAGACACCCTGAAGGAGGATG
SRR709444.41214292.1+     CTTCACTGGACCCCAGGATCAGCACCTGGTGGATGAAGACACCCTGAAGGAGGATG
SRR709450.61517042.1-                CCCAGGATCAGCACCTGGTGGATGAAGACACCCTGAAGGAGGATG
SRR709445.70314432.2-                 CCAGGATCACGACCTGGTGGATGAACACACCCTGAAGGAGGATG
SRR709450.81453004.1-                CCCAGGATCAGCACCNGGTGGATGAAGACACCCTGAAGGAGGATG
SRR709440.75887335.2+                      ATCAGCACCTGGTGGATGAAGACACCCTGAAGGAGGATG
SRR709441.75325365.1+                      ATCAGCACCTGGTGGATGAAGACACCCTGAAGGAGGATG
SRR709445.32686869.1-                          GCACCTGGTGGATGAAGACACCCTGAAGGAGGATG
SRR709449.29172404.1+                               TGGTGGATGAAGACACCCTGAAGGAGGATG
SRR709442.64668641.1+                                        AAGACACCCTGAAGGAGGATG
SRR709449.37686249.1+                                         AGACACCCTGAAGGAGGATG
SRR709438.27268167.1+                                           ACACCCTGAAGGAGGATG
SRR709445.92277326.2-                                              CCCTGAAGGAGGATG
SRR709442.44544843.1+                                                  GAAGGAGGATG
SRR709444.71375770.1-                                                            G
                      ____________________________________________________________
```

```
consensus             TGATCTTCACTGGACCCCAGGATCAGCACCTGGTGGATGAAGACACCCTGAAGGAGGATG

                          .    :    .    :    .    :    .    :    .    :    .    :
SRR709449.85843889.2- GGAACCAGGATGCAGAGCTGCACGGGCTGACGGAAGGTGCT
SRR709445.61659285.2+ GGAACCAGGATGCAGAGCTGCACGGGCTGACGGAAGGTGCTGT
SRR709439.46592067.1- GGAACCAGGATGCAGAGCTGCACGGGCTGACGGAAGGTGCTGTCT
SRR709440.57295999.2- GGAACCAGGATGCAGAGCTGCACGGGCTGACGGAAGGTGCTGTCT
SRR709444.41214292.1+ GGAACCAGGATGCAGAGCTGCACGGGCTGACGGAAGGTGCTGTCT
SRR709450.61517042.1- GGAACCAGGATGCAGAGCTGCACGGGCTGACGGAAGGTGCTGTCTACACCACCATG
SRR709445.70314432.2- GGAACCAGGATGCAGAGCTGCACGGGCTGACGGAAGGTGCTGTCTACACCACCAT
SRR709450.81453004.1- GGAACCAGGATGCAGAGCTGCACGGGCTGACGGAAGGTGCTGTCNACACCACCATG
SRR709440.75887335.2+ GGAACCAGGATGCAGAGCTGCACGGGCTGACGGAAGGTGCTGTCTACACCCCCATGCGCT
SRR709441.75325365.1+ GGAACCAGGATGCAGAGCTGCACGGGCTGACGGAAGGTGCTGTCTACACCACCCTGCGC
SRR709445.32686869.1- GGAACCAGGATGCAGAGCTGCACGGGCTGACGGAAGGTGCTGTCTACACCACCATGCGCT
SRR709449.29172404.1+ GGAACCAGGATGCAGAGCTGCACGGGCTGACGGAAGGTGCTGTCTACACCACCATGCGCT
SRR709442.64668641.1+ GGAACCAGGATGCAGAGCTGCACGGGCTGACGGAAGGTGCTGTCTACACCACCATGCGCT
SRR709449.37686249.1+ GGAACCAGGATGCAGAGCTGCACGGGCTGACGGAAGGGGCCGTCTACACCACCATGCGCT
SRR709438.27268167.1+ GGAACCAGGATGCAGAGCTGCACGGGCTGACGGAAGGTGCTGTCTACACCACCATGCGCT
SRR709445.92277326.2- GGAACCAGGATGCAGAGCTGCACGGGCTGACGGAAGGTGCTGTCTACACCACCATGCGCT
SRR709442.44544843.1+ GGAACCAGGATGCAGAGCTGCACGGGCTGACGGAAGGTGCTGTCTACACCACCATGCGCT
SRR709444.71375770.1- GGAACCAGGATGCAGAGCTGCACGGGCTGACGGAAGGTGCTGTCTACACCACCATGCGCT
SRR709439.96449359.2+  GAACCAGGATGCAGAGCTGCACGGGCTGACGGAAGGTGCTGTCTACACCACCATGCGCT
SRR709440.108259534.+                                   AGGTGCTGTCTACACCACCATGCGCT
SRR709450.73117383.1+                                   AGGTGCTGTCTACACCACCATGCGCT
SRR709441.8908888.1+                                                     CCATGCGCT
                      ____________________________________________________________
consensus             GGAACCAGGATGCAGAGCTGCACGGGCTGACGGAAGGTGCTGTCTACACCACCATGCGCT

                          .    :    .    :    .    :    .    :    .    :    .    :
SRR709440.75887335.2+ TT
SRR709445.32686869.1- TTTCCA
SRR709449.29172404.1+ TTTCCAGGCCC
SRR709442.64668641.1+ TTTCCAGGCCCTTCCGCTCC
SRR709449.37686249.1+ TTTCCAGGCCCTTCCGCTCCT
SRR709438.27268167.1+ TTTCCAGGCCCTTCCGCTCCTGC
SRR709445.92277326.2- TTTCCAGGCCCTTCCGCTCCTGCG
SRR709442.44544843.1+ TTTCCAGGCCCCTCCGCTCCTGCCACCGCC
SRR709444.71375770.1- TTTCCAGGCCCTTCCGCTCCTGCGACCGC
SRR709439.96449359.2+ TTTCCAGGCCCTTCCGCTCCTGCGACCGCCATGACCCGGACA
SRR709440.108259534.+ TTTCCAGGCCCTTCCGCTCCTGCGACCGCCATGACCAGGACATCACGGTAAAATAGAGAG
SRR709450.73117383.1+ TTTCCAGGCCCTTCCGCTCCTGCGACCGCCATGACCAGGACATCACGGTAAAATAGAGAG
SRR709441.8908888.1+  TTTCCAGGCCCTTCCGCTCCTGCGACCGCCATGACCAGGACATCACGGTAAAATAGAGAG
                      ____________________________________________________________
consensus             TTTCCAGGCCCTTCCGCTCCTGCGACCGCCATGACCAGGACATCACGGTAAAATAGAGAG

                          .    :    .    :    .    :    .    :    .    :    .    :
SRR709440.108259534.+ GGGTGAAGAACATTT
SRR709450.73117383.1+ GGGTGAAGAACATTT
SRR709441.8908888.1+  GGGCGAAGAACATTTAATCACAAGCAGCTCCT
                      ____________________________________________________________
consensus             GGGTGAAGAACATTTAATCACAAGCAGCTCCT
```

(3) The exon 13 coding sequence was predicted by assembling WGS data using CAP3. The exon 13 is on the reverse strand and marked in red.

```
                          .    :    .    :    .    :    .    :    .    :    .    :
SRR709441.96328630.1+ GGTGTCTGTATGTAAGGAGACAGAATCAGCCTCCCGTCTGCAGCGTGGCAAGGAGCCAGG
SRR709444.87124778.1+        GTATGTAAGGAGACAGAATCAGCCTCCCGTCTGCAGCGTGGCAAGGAGCCAGG
SRR709444.104969167.+        GTATGTAAGGAGACAGAATCAGCCTCCCGTCTGCAGCGTGGCAAGGAGCCAGG
SRR709448.30060074.1+                                  CCTCCCGTCTGCAGCGTGGCAAGGAGCCAGG
SRR709450.15597395.1+                                        GTCTGCAGCGTGGCAAGGAGCCAGG
SRR709447.17798046.2+                                                      AAGGAGCCAGG
SRR709442.8182440.2+                                                               AGG
                      ____________________________________________________________
consensus             GGTGTCTGTATGTAAGGAGACAGAATCAGCCTCCCGTCTGCAGCGTGGCAAGGAGCCAGG

                          .    :    .    :    .    :    .    :    .    :    .    :
SRR709441.96328630.1+ ACAGGGTGCCCTGCACCAGCAAGTGGGCCGCCAGGGGGAGG
SRR709444.87124778.1+ ACAGGGTGCCCTGCACCAGCAAGTGGGCCGCCAGGGGGAGG
SRR709444.104969167.+ ACAGGGTGCCCTGCACCAGCAAGTGGGGCGCCAGGGGGGGGCGCT
SRR709448.30060074.1+ ACAGGGTGCCCTGCACCAGCAAGTGGGCCGCCAGGGGGAGGCGCTTCAGGGAAGCAGTGC
SRR709450.15597395.1+ ACAGGGTGCCCTGCACCAGCAAGTGGGCCGCCAGGGGGAGGCGCTTCAGGGAAGCAGTGC
SRR709447.17798046.2+ ACAGGGTGCCCTGCACCAGCAAGTGGGCCGCCAGGGGGAGGCGCTTCAGGGAAGCAGTGC
SRR709442.8182440.2+  ACAGGGTGCCCTGCACCAGCAAGTGGGCCGCCAGGGGGAGGCGCTTCAGGGAAGCAGTGC
SRR709442.92200498.1+                                           GCTTCAGGGAAGCAGTGC
SRR709449.29381523.1-                                           GCTTCAGGGAAGCAGTGC
```

```
SRR709441.11034400.2+                                           GCTTCAGGGAAGCAGTGC
SRR709442.2476172.1+                                              TTCAGGGAAGCAGTGC
SRR709450.72103658.1+                                               CAGGGAAGCAGTGC
SRR709438.111077230.+                                                 GGGAAGCAGTGC
SRR709445.12813273.1+                                                 GGGAAGCAGTGC
SRR709439.23204140.2+                                                    AAGCAGTGC
SRR709438.22398249.1+                                                     AGCAGTGC
SRR709440.86387899.1-                                                     AGCAGTGC
SRR709450.40977591.1+                                                         GTGC

consensus             ACAGGGTGCCCTGCACCAGCAAGTGGGCCGCCAGGGGGAGGCGCTTCAGGGAAGCAGTGC

                          .    :    .    :    .    :    .    :    .    :    .    :
SRR709448.30060074.1+ TGG
SRR709450.15597395.1+ TGG
SRR709447.17798046.2+ TGGAGCCCGAGAGGGCCAGCCGTGCGGCCC
SRR709442.8182440.2+  TGGAGCCCGAGAGGGCCACCGGTGCGGCCC
SRR709442.92200498.1+ TGGAGCCCGAGAGGGCCACCGGTGCGGCCCTGAAGCCTGCGGGAGTCTTGTCCTGGGGCT
SRR709449.29381523.1- TGGAGCCCGAGAGGGCCACCGGTGCGGCCCTGAAGCCTGCGGGAGTCTTGTCCTGGGGCT
SRR709441.11034400.2+ TGGAGCCCGAGAGGGCCACCGGTGCGGCCCTGAAGCCTGCGGGAGTCTTGTCCTGGGGCT
SRR709442.2476172.1+  TGGAGCCCGAGAGGGCCACCGGTGCGGCCCTGAAGCCTGCGGGAGTCTTGTCCTGGGGCT
SRR709450.72103658.1+ TGGAGCCCGAGAGGGCCACCGGTGCGGCCCTGAAGCCTGCGGGAGTCTTGTCCTGGGGCT
SRR709438.111077230.+ TGGAGCCCGAGAGGGCCACCGGTGCGGCCCTGAAGCCTGCGGGAGTCTTGTCCTGGGGCT
SRR709445.12813273.1+ TGGAGCCCGAGAGGGCCACCGGTGCGGCCCTGAAGCCTGCGGGAGTCTTGTCCTGGGGCT
SRR709439.23204140.2+ TGGAGCCCGAGAGGGCCACCGGTGCGGCCCTGAAGCCTGCGGGAGTCTTGTCCTGGGGCT
SRR709438.22398249.1+ TGGAGCCCGAGAGGGCCACCGGTGCGGCCCTGAAGCCTGCGGGAGTCTTGTCCTGGGGCT
SRR709440.86387899.1- TGGAGCCCGAGAGGGCCACCGGTGCGGCCCTGAAGCCTGCGGGAGTCTTGTCCTGGGGCT
SRR709450.40977591.1+ TGGAGCCCGAGAGGGCCACCGGTGCGGCCCTGAAGCCTGCGGGAGTCTTGTCCTGGGGCT
SRR709442.8182440.1-  TGGAGCCGGAGGGGGCCACCGGTGCGGCCCTGAAGCCTGCGGGAGTCTTGTCCTGGGGCT
SRR709442.97536395.1-             GGGCCACCGGTGCGGCCCTGAAGCCTGCGGGAGTCTTGTCCTGGGGCT
SRR709446.80301225.1-     GCCCGAGAGGGCCACCGGTGCGGCCCTGAAGCCTGCGGGAGTCTTGTCCTGGGGCT
SRR709444.104969167.-     GCCCGAGAGGCCCCCGGTGCGGCCCTGAAGCCTGCGGGAGTCTTGTCCTGGGGCT
SRR709449.42012551.2-           GAGGGCCACGTGTGCGGCCCGGAAGCCTGCGGGAGTCTTGTCCTGGGGCT
SRR709441.126246534.-               GCCACCGGTGCGGCCCTGAAGCCTGCGGGAGTCTTGTCCTGGGGCT
SRR709449.59627364.2-                  ACCGGTGCGGCCCTGAAGCCTGCGGGAGTCTTGTCCTGGGGCT
SRR709439.80347029.2-                   CCGGGGCGGCCTTGAAGCCTGCGGGAGTCTTGTCCTGGGGCT
SRR709439.44174118.1+                    CGGTGCGGCCCTGAAGCCTGCGGGAGTCTTGTCCTGGGGCT
SRR709450.15597395.2-                       GGCGGCCCTGAAGCCTGCGGGAGTCTTGTCCTGGGGCT
SRR709439.70552255.1-                              CTGAAGCCTGAGGGAGTCTTGTCCTGGGGCT
SRR709438.5290677.1+                               CTGAAGCCTGCGGGAGTCTTGTCCTGGGGCT
SRR709444.52784982.2-                                      TGCGGGAGTCTTGTCCTTGGGCT
SRR709439.19290261.2+                              CTGAAGCCTGCGGGAGTCTTGTCCTGGGGCT
SRR709442.82966569.1-                                     CTGCGGGAGTCTTGTCCTGGGGCT
SRR709446.10938056.1-                                       GCGGGAGTCTTGTCCTGGGGCT
SRR709439.13154492.1+                                            AGTCTTGTCCTGGGGCT
SRR709444.24808237.2-                                               CTTGTCCTGGGGCT
SRR709450.39567117.1-                                               CTTGTCCTGGGGCT
SRR709450.79787656.2+                                                    CCTGGGGCT
SRR709440.84050727.1+                                                        GGGCT
SRR709443.74846418.2-                                                            T

consensus             TGGAGCCCGAGAGGGCCACCGGTGCGGCCCTGAAGCCTGCGGGAGTCTTGTCCTGGGGCT

                          .    :    .    :    .    :    .    :    .    :    .    :
SRR709442.92200498.1+ CCACTTTGCCTCCAGAGGACT
SRR709449.29381523.1- CCACTTTGCCTCCAGAGGACT
SRR709441.11034400.2+ CCACTTTGCCTCCAGAGGAC
SRR709442.2476172.1+  CCACTTTGCCTCCAGAGGACTCCAG
SRR709450.72103658.1+ CCACTTTGCCTCCAGAGGACTCCAGGC
SRR709438.111077230.+ CCACTTTGCCTCCAGCGGACTCCCGGCAG
SRR709445.12813273.1+ CCACTTTGCCTCCAGAGGACTCCAGGCAG
SRR709439.23204140.2+ CCACTTTGCCTCCAGAGGACTCCAGGCCGGGC
SRR709438.22398249.1+ CCACTTTGCCTCCAGAGGACTCCCGGCAGGGCC
SRR709440.86387899.1- CCACTTTGCCTCCAGAGGACTCCAGGCAGGGCC
SRR709450.40977591.1+ CCACTTTGCCTCCAGAGGACTCCAGGCAGGGCCCCCG
SRR709442.8182440.1-  CCACTTTGCCTCCAGAGGACTCCAGGCAGGGCCCCCGAGG
SRR709442.97536395.1- CCACTTTGCCTCCAGAGGACTCCAGGCAGGGCCCCC
SRR709446.80301225.1- CCACTTTGCCTCCAGAGGACTCCAGGCAGGGCCCCCGAGGAGTA
SRR709444.104969167.- CCACTTTGCCTCCAGAGGACTCCAGGCAGGGCCCCCGAGGAGTAG
SRR709449.42012551.2- CCACTTTGCCTCCAGAGGACTCCAGGCAGGGCCCCCGAGGAGTAG
SRR709441.126246534.- CCACTTTGCCTCCAGAGGACTCCAGGCAGGGCCCCCGAGGAGTAGGGATGATGTC
SRR709449.59627364.2- CCACTTTGCCTCCAGAGGACTCCAGGCAGGGCCC
SRR709439.80347029.2- CCACTTTGCCTCCAGAGGACTCCAGGCAGGGCCCCCGAGGAGTAGGGATGATG
SRR709439.44174118.1+ CCACTTTGCCTCCAGAGGACTCCAGGCAGGGCCCCCGAGGAGTAGGGATGATGTCTGGAA
SRR709450.15597395.2- CCACTTTGCCTCCAGAGGACTCCAGGCAGGGCCCCCGAGGAGTAGGGATGATG
SRR709439.70552255.1- CCACTTTGCCTCCAGAGGACTCCAGGCAGGGCCCCCGAGGAGTAGGGATGATGTCTGGAA
SRR709438.5290677.1+  CCACTTTGCCTCCAGAGGACTCCAGGCAGGGCCCCCGAGGAGCAGGGATGATGTCTGGAA
```

```
SRR709444.52784982.2- CCACTTTGCCTCCAGAGGACTCCAGGCAGG
SRR709439.19290261.2+ CCACTTTGCCTCCAGAGGACTCCAGGCAGGGCCCCCGAGGAGTAGGGATGATGTCTGGAA
SRR709442.82966569.1- CCACTTTGCCTCCAGAGGACTCCAGGCAGGGCCCCCGAGGAGTAGGGATGATGTCTGGAA
SRR709446.10938056.1- CCACTTTGCCTCCAGAGGACTCCAGGCAGGGCCCCCGAGGAGTAGGGATGATGTCTGGAA
SRR709439.13154492.1+ CCACTTTGCCTCCAGAGGACTCCAGGCAGGGCCCCCGAGGAGTAGGGATGATGTCTGGAA
SRR709444.24808237.2- CCACTTTGCCTCCAGAGGACTCCAGGCAGGGCCCCCGAGGAGTAGGGATGATGTCTGGAA
SRR709450.39567117.1- CCACTTTGCCTCCAGAGGACTCCAGGCAGGGCCCCCGAGGAGTAGGGATGATGTCTGGAA
SRR709450.79787656.2+ CCACTTTGCCTCCAGAGGACTCCAGGCAGGGCCCCCGAGGAGTAGGGATGATGTCTGGAA
SRR709440.84050727.1+ CCACTTTGCCTCCAGAGGACTCCAGGCAGGGCCCCCGAGGAGTAGGGATGATGTCTGGAA
SRR709443.74846418.2- CCACTTTGCCTCCAGAGGACTCCAGGCAGGGCCCCCGAGGAGTAGGGATGATGTCTGGAA
SRR709444.43037549.2+          CTCCAGAGGACTCCAGGCAGGGCCCCCGAGGAGTAGGGATGATGTCTGGAA
SRR709445.2533889.1-            TCCAGAGGACTCCAGGCAGGGCCCCCGAGGAGTAGGGATGATGTCTGGAA
SRR709447.17798046.1-           TCCAGGGGACTCCGGGCAGGGCCCCCGAGGAGTAGGGATGATGTCTGGAA
SRR709438.94074157.2+            CCAGAGGACTCCAGGCAGGGCCCCCGAGGAGTAGGGATGATGTCTGGAA
SRR709439.19290261.1-                     TCCAGGCAGGGCCCCCGAGGAGTAGGGATGATGTCTGGAA
SRR709448.69303831.2+                      CCAGGCAGGGCCCCCGAGGAGTAGGGATGATGTCTGGAA
SRR709439.23204140.1-                         GGCAGGGCCCCCGAGGAGTAGGGATGATGTCTGGAA
SRR709442.78683411.2+                         GGCAGGGCCCCCGAGGAGTAGGGATGATGTCTGGAA
SRR709443.5205894.1+                                GCCCCCGAGGAGTAGGGATGATGTCTGGAA
SRR709441.96328630.2-                                  CCCGAGGAGTAGGGATGATTTCTGGAA
                      ____________________________________________________________
consensus             CCACTTTGCCTCCAGAGGACTCCAGGCAGGGCCCCCGAGGAGTAGGGATGATGTCTGGAA

                          .    :    .    :    .    :    .    :    .    :    .    :
SRR709439.70552255.1- TCCAGC
SRR709438.5290677.1+  TCCAGCCTGT
SRR709439.19290261.2+ CCCAGCCTGT
SRR709442.82966569.1- TCCAGCCTGTTGTGTTT
SRR709446.10938056.1- TCCAGCCTGTTGTGTT
SRR709439.13154492.1+ TCCCGCCTGTTCTGTTTTCCACC
SRR709444.24808237.2- TCCAGCCTGTTGTGTT
SRR709450.39567117.1- TCCAGCCTGTTGTGTTTTCCACCACCT
SRR709450.79787656.2+ TCCAGCCTGTTGTGTTTTCCACCACC
SRR709440.84050727.1+ TCCAGCCTGTTGTGTTTTCCACCACCTCCTGCAGGA
SRR709443.74846418.2- TCCAGCC
SRR709444.43037549.2+ TCCAGCCTGTTGTGTTTTCCACCACCTCCTGCAGCAGCGGGAAGACAA
SRR709445.2533889.1-  TCCAGCCTGTTGTGTTTTCCACCACCTCCTGCAGGAGCGGGAA
SRR709447.17798046.1- TCCAGCCTGTTGTGTTTTCCACCACCTCCTGCAGGAGCGGGAAGACAAAAG
SRR709438.94074157.2+ TCCAGCCTGTTGTGTTTTCCACCACCTACTGCAGGAGCGGGAAGACAAAAGG
SRR709439.19290261.1- TCCAGCCTGTTGTGTTTTCCACCACCTCCTGCAGGAGCGGGAAGACAAAAGGCAAAGGAT
SRR709448.69303831.2+ TCCAGCCTGTTGTGTTTTCCACCACCTCCTGCAGGAGCGGGAAGACAAAAGGCAAAGG
SRR709439.23204140.1- TCCAGCCTGTTGTGTTTTCCACCACCTCCTGCAGGAGCGGGAAGACAAAAGGCAAAGGAT
SRR709442.78683411.2+ TCCAGCCTGTTGTGTTTTCCACCACCTCCTGCAGGAGCGGGAAGACAAAAGGCAACGGAT
SRR709443.5205894.1+  TCCAGCCTGTTGTGTTTTCCACCACCTCCTGCAGGAGCGGGAAGCCAAAAGGCAAAGGAT
SRR709441.96328630.2- TCCAGCCTGTTGTGTTTTCCACCACCTCCTGCAGGAGCGGGAAGACAAAAGGCAAAGGAT
                      ____________________________________________________________
consensus             TCCAGCCTGTTGTGTTTTCCACCACCTCCTGCAGGAGCGGGAAGACAAAAGGCAAAGGAT

                          .    :    .    :    .    :    .    :    .    :    .    :
SRR709439.19290261.1- G
SRR709439.23204140.1- G
SRR709442.78683411.2+ G
SRR709443.5205894.1+  G
SRR709441.96328630.2- GAAGCACCCCCCTT
                      ____________________________________________________________
consensus             GAAGCACCCCCCTT
```

21. Northern greater galago (bushbaby) (*Otolemur garnettii*)

>Otolemur_garnettii No=21 length=1857 name="Northern greater galago (bushbaby)"
ATGGCCTGTGCCCTTCTCTTCTGTCTTTTCCTGCTTCTAGCCCTGGAAGTCCCTTTCCTGGGCAACTGCTTCGGCCCCATGTCTCGCTTACGTTATTCTA
GGTTCCTAGATCCTTCCAATGTCGTGTTCCTGCGTTGGGACTTTGACCTTAAGGCTGAATTCATCACTTTTGAGCTCCAAGTCCGCACAGCTGGCTGGGT
GGGCTTGGGTGTCACAAATCGCTACACCAGAGCAGGCAGTGACCTGGTTGTTGGAGGAGTCTTATCCGACGGCAATGTCTATTTTTCGGATCAGCACCTG
GTGGACGAAGACACTCTGAGAGAAGACGGCAGCCAGGACGCCGAGCTGCACGGGCTGACGGAAGGTGCTGTCTACACCACCATGCGCTTCTCCAGGCCCT
TCCGCTCCTGCGACCCTCATGACCAGGACATCACGGGTGACACTGTGAGGGTGATGGCTGCCTACGGCCTGGATGACACCCTGAAACTGGATCGGAACCG
GATTTTTGTCAAGTCCATCTTTCTGCTACAAGTAGTCCATCCTGACGATCTGGAAGTCCCTGAGGACACCATCATCCATGACTTGGAGATCACTGATTTC
CTTATTCCAGAGGATGACACCACATACGCCTGCACATTCCTCCCTCTGCCCATTGTTAGCAAGAAGCATCACATCTACAAGTTTGAACCCAAGTTGGTCA
ACCACAACGAGACGATGGTGCATCACATCCTGGTGTACGCCTGTGGCAACGCCAGCGCCCTCCCGACCGGCATCAGCGACTGCTACGGGGCCGACCCTGC
CTTCTCCCTCTGCTCCCAGGTCATCGTGGGCTGGGCTGTTGGGGGCACTAGTTACCAGTTTCCCGATGACGTGGGCATCTCTATTGGGACCCCCTTGGAC
CCTCAGTGGATCCGCCTGGAGGTTCATTACAGCAACTTTCACAACCTTCCTGGTGTGTACGACTCCTCAGGCATCCGTGTGTACTACACCAGTCACCTGC
GCAAATACGACATGGGCGTCCTCCAGCTCGGCTTCTTCACATTCCCCATCCACTTCATACCCCCAGGCGCCGAGTCCTTCATGTCCTACGGGCTGTGTAA
GACGGAGAAGTTTGAAGAGATGAACGGGGCGCCCGTCCCTGACATACAGGTATACGGCTACCTGCTGCACACCCACCTGGCTGGCCGGGCTCTGCAGGCC
GTGCAGTACAGAAATGGAACACAACTCCAAACCATCTGTAAAGATGATTCCTACGACTTCAACCTGCAGGAGACTCGAGATCTACCTGCCCGAGTGGCAA
TCAAGCCGGGAGACGAATTGCTGGTAGAATGCCACTACCAAACCCTGGACCGTGAAGCCTTGACTTTTGGAGGTCCCAGCACCATCAATGAGATGTGCCT
CATTTTCCTCTTCTACTACCCTCGAAATAACATCTCCAGCTGCATGGGGTATCCTGACATCATCTACGTGGCACAGGAGCTTGGGGAGGAGGTATCAGAC
TCCATGGAGGGCATGATGGCCATGAACAATGTAGAATGGACCCCAGAGAGCATTAAGAAGGCTGAGCGAGCCTGCAAAGAGGCCAGACAGACGGTGATAA
TAAAGACTATTGATGAGGTGGTGGAAAACACTACAGGCTGGATTCCAGACATCATCCCTACTCCTCGGGGCCCCTGCTTGGAGTCCTCTGGAGGCAAAGT
GGAGCCCCAGGACAAGACCCCTGCAGGCTTCAAGGCTGCCCCAGTGGCCCTCTCTGGCTCCAGCACTGTATCCCTGAGGCGCCTCCCACTGGTAGCCCAC
CTGCTGGTGCAGGGCATCCTCTCCTGGCTCCTTGCCATGCTGCGGACTGGAGGCTGA

>Otolemur_garnettii No=21 length=618 name="Northern greater galago (bushbaby)"
MACALLFCLFLLLALEVPFLGNCFGPMSRLRYSRFLDPSNVVFLRWDFDLKAEFITFELQVRTAGWVGLGVTNRYTRAGSDLVVGGVLSDGNVYFSDQHL
VDEDTLREDGSQDAELHGLTEGAVYTTMRFSRPFRSCDPHDQDITGDTVRVMAAYGLDDTLKLDRNRIFVKSIFLLQVVHPDDLEVPEDTIIHDLEITDF
LIPEDDTTYACTFLPLPIVSKKHHIYKFEPKLVNHNETMVHHILVYACGNASALPTGISDCYGADPAFSLCSQVIVGWAVGGTSYQFPDDVGISIGTPLD
PQWIRLEVHYSNFHNLPGVYDSSGIRVYYTSHLRKYDMGVLQLGFFTFPIHFIPPGAESFMSYGLCKTEKFEEMNGAPVPDIQVYGYLLHTHLAGRALQA
VQYRNGTQLQTICKDDSYDFNLQETRDLPARVAIKPGDELLVECHYQTLDREALTFGGPSTINEMCLIFLFYYPRNNISSCMGYPDIIYVAQELGEEVSD
SMEGMMAMNNVEWTPESIKKAERACKEARQTVIIKTIDEVVENTTGWIPDIIPTPRGPCLESSGGKVEPQDKTPAGFKAAPVALSGSSTVSLRRLPLVAH
LLVQGILSWLLAMLRTGG

(1) Exon coordinates

Exon	UCSC Chromosome	Strand	Start	End
1	otoGar3 GL873568	+	2853615	2853902
2	otoGar3 GL873568	+	2854822	2854968
3	otoGar3 GL873568	+	2855500	2855661
4	otoGar3 GL873568	+	2855914	2855997
5	otoGar3 GL873568	+	2856782	2856949
6	otoGar3 GL873568	+	2857135	2857237
7	otoGar3 GL873568	+	2857427	2857593
8	otoGar3 GL873568	+	2857912	2858003
9	otoGar3 GL873568	+	2858285	2858381
10	otoGar3 GL873568	+	2858497	2858556
11	otoGar3 GL873568	+	2860285	2860415
12	otoGar3 GL873568	+	2861156	2861270
13	otoGar3 GL873568	+	2861562	2861804

22. Chinese treeshrew (*Tupaia chinensis*)

>Tupaia_chinensis No=22 length=1857 name="Chinese treeshrew"
ATGACCTGTGTCCTTCTCTTCAGGCTTTTCCTACTTCTGGTCCTAGCATCCCCTTCCCAAGGCAAGCGTCTTGGCCCCACATCTCGCCTGCGTTATTCTA
GGTTCCTAGATCCTTCCAATGCCATTTTTCTGCGTTGGGACTTTGACCTTGAAGCTGAGATCATCACTTTTGAGCTCCAGGTCCGAACAACTGGATGGGT
GGGCTTGGGTGTCACAAACCGCTACACCAACGTGGGAAGTGACCTGGTCATTGGAGGAGTCTTGCCCGATGGCAATGTCTATTTCTCGGACCAGCACCTG
GTGGATGAAGACACCCTTAGGGAGGACGGAAGCCAAGATGCTGAGCTGCTAGGGCTCACAGAAGATGCTGTCTACACCACCATGCGCTTTTCCAGGCCCT
TCCGCTCCTGCGACCCTCATGACCAAGATATTGCGAGTGACACAGTGAGGGTGCTCACTGCCTACGGCCTGGATGACACTGTGAAGTTGGATCGGGAGCG
TACTTTTGTCAAGTCCATCTTCCTGCTACAAATAGTCCACCCTGATGATCTGGAAGTCCCCGAGGACACCATCATCCATGACTTGGAGATCACTGATTTT
CTCATTCCAGAGGATGACACCACGTATGCCTGCACCTTTCTCCCTCTCCCCATCGTAAGCAAGAAGCATCACATCTACAAGTTTGAGCCCAAGCTTATCT
ACCACAACGAGACCATGGTGCATCACATCCTGGTGTATGCCTGTGGCAATGCCAGTGCTCTCCCCACGGGCATCAGCGACTGCTATGGAGCTGACCCCGC
CTTCTCCCTCTGCTCACAGGTCATCGTGGGCTGGGCTGTCGGGGGCACGAGTTACCAGTTTCCCGATGATGTAGGCATTTCTATTGGGACCCCCTTGGAC
CCTCAGTGGATCCGACTGGAGGTTCACTACAGCAATTTTCACAACCTTCCTGGTGTGTATGACACTTCAGGGATTCGAGTGTACTACAGTCCTCAGCTGC
GCAAATACGACATGGGTGTCCTCCAGCTGGGCTTCTTCACTTTCCCCATTCACTTCATACCTCCGGGTGCTGAGTCCTTCATGTCCTATGGGCTGTGTAA
GACTGAGAAGTTTGAAGAGATGAATGGGGCCCCCGTGCCTGACATACAGGTATACGGCTACCTGCTCCATACCCACTTGGCAGGGCGGGCTCTGCAGGCT
GTGCAGTACAGAAATGGAACACAAATTCAAACGATCTGTAAAGATGATTCCTATGACTTCAATCTGCAGGAGACTCGAGATTTGCCTTCTCGAGTGGCAA
TCAAGCCGGGAGATGAGCTGCTGGTAGAATGTCACTACCAGACACTGGACCGTGACACAATGACCTTTGGAGGTCCCAGCACCATCAATGAGATGTGCCT
CATCTTCCTCTTCTACTATCCCCGAAATAACATCTCCAGCTGCATGGGGTACCCTGACATCATCTACGTGGCCCATGAGCTGGGAGAAGAGGCATCAGAC
TCCATGGAGGGCATGATGGCCATGAACAACGTGGAGTGGACCCCAGAGAACATTAAGAAAGCTGAGAAAGCCTGCAAGGAGGCCCAACAGACTGTGATAA
TAAAGACCATTGACGAGATGGTGGAAAACACCACAGGATGGATTCCAGACATCATCCCTACTCCTCGGGGACCCTGTCTGGAGTCCTCTGGAGGCAAAGT
TGAGCCCCAGGACAAGACTCCAGCAGGCTTCAGGGCAGCACCAGTGGCCCTCCGGGGTTCTGGCACTGCTCCCCTGCAGTGCCTCCCTGTGGCTGTCCTC
TTGTTTGTGCAAGGTGCCCTTTCTTGGATCCTTGCCACACTGCAGACTGGAGCATGA

>Tupaia_chinensis No=22 length=618 name="Chinese treeshrew"
MTCVLLFRLFLLLVLASPSQGKRLGPTSRLRYSRFLDPSNAIFLRWDFDLEAEIITFELQVRTTGWVGLGVTNRYTNVGSDLVIGGVLPDGNVYFSDQHL
VDEDTLREDGSQDAELLGLTEDAVYTTMRFSRPFRSCDPHDQDIASDTVRVLTAYGLDDTVKLDRERTFVKSIFLLQIVHPDDLEVPEDTIIHDLEITDF
LIPEDDTTYACTFLPLPIVSKKHHIYKFEPKLIYHNETMVHHILVYACGNASALPTGISDCYGADPAFSLCSQVIVGWAVGGTSYQFPDDVGISIGTPLD
PQWIRLEVHYSNFHNLPGVYDTSGIRVYYSPQLRKYDMGVLQLGFFTFPIHFIPPGAESFMSYGLCKTEKFEEMNGAPVPDIQVYGYLLHTHLAGRALQA
VQYRNGTQIQTICKDDSYDFNLQETRDLPSRVAIKPGDELLVECHYQTLDRDTMTFGGPSTINEMCLIFLFYYPRNNISSCMGYPDIIYVAHELGEEASD
SMEGMMAMNNVEWTPENIKKAEKACKEAQQTVIIKTIDEMVENTTGWIPDIIPTPRGPCLESSGGKVEPQDKTPAGFRAAPVALRGSGTAPLQCLPVAVL
LFVQGALSWILATLQTGA

(1) Exon coordinates

Exon	NCBI Accession	Strand	Start	End
1	ALAR01033741	-	20852	21139
2	ALAR01033741	-	19484	19630
3	ALAR01033741	-	18852	19013
4	ALAR01033741	-	18531	18614
5	ALAR01033741	-	16722	16889
6	ALAR01033741	-	16421	16523
7	ALAR01033741	-	16041	16207
8	ALAR01033741	-	15618	15709
9	ALAR01033741	-	15248	15344
10	ALAR01033741	-	15045	15104
11	ALAR01033741	-	13477	13607
12	ALAR01033741	-	11334	11448
13	ALAR01033741	-	10802	11044

23. European rabbit (*Oryctolagus cuniculus*)

>Oryctolagus_cuniculus No=23 length=1857 name="European rabbit"
ATGGCCTGTGCTTTGCTGTTCAGGCTTTCCCTGCTTCTGGCCCTGACTGCCCCCTCCCCCGGCAAACACCTTGGCCCCAAGTCCCGCTTGCGTTATTCCA
GGTTCCTGGGTCCGTCCAATGTCATTCTCCTGCGCTGGGACTTTGACCTTGAGGCCGAAATCATCACTTTTGAGCTCCAGGTCCAGACAGCTGGCTGGGT
GGGCTTAGGCGTCATGAATCGCTACACCTTCGTGGGCAGTGACCTTGTGGTTGGAGGAGTCTTGCCTGATGGCAACGTCTATTTCTCGGATCAGCACCTG
GTGGATGAAGACACCCTGGAGGAAGACAGGAGCCAGGATGCTGAGCTGCAGGGACTAACAGAAGATGGAATCTATACCACCATGCGCTTCTCCAGGCCCT
TCCGCTCCTGTGACCCTCATGACCAAGACATTATGGGTGACACCGTAAGGGTACTGGCTGCCTATGGTCTGAACGACACTGTGGAACTGGATCGGGAGCG
CACTTTTGTCAAGTCCATCTTCCTGCTACAAATAGTCCACCCTGATGACCTGGAAGTCCCCGAGGACACCATCATTCACGACTTGGAGATCACTGATTTC
CTCATTCCAGAAGATGACACCACATATGCCTGCACCTTTCTTCCTCTCCCAATTGTCAGCAAGAAGCATCACATCTACAAGTTTGAGCCCAAGCTGGCAC
CCCACAATGAGACAATGGTGCATCACATCCTGCTGTATGCCTGTGGCAATGCCAGTGTGCTCCCCACCGGCATCAGCGACTGCTACGGGGCAGATCCTGC
CTTCTCCCTCTGCTCACAAGTCATCGCCGGCTGGGCTGTTGGAGGCACCAGTTACCAGTTTCCAGATGATGTGGGCATCTCTATTGGGACCCCCTTGGAC
CCTCAGTGGATCCGGCTGGAGATTCACTACAGCAATTTTCACAACCTTCCTGGTTTGTACGACTCCTCGGGGATCCGCGTGTACTACACTTCTCAGTTGC
GCAAATACGACATGGGGGTCCTCCAGCTGGGCTTCTTCACCTTCCCCATCCACTTCATACCCCCAGGAGCCGAGTCATTCATGTCCTATGGGCTGTGCAA
AACAGAGAAGTTTGAAGAGATGAATGGGGCCCCGGTGCCTGACATACAGGTGTATGGCTACCTGCTCCACACCCACTTGGCTGGGCGGGCTCTGCAGGCT
GTGCAATACAGAAATGGAACACAACTCCGAACAATCTGTAAAGATGATTCCTACGACTTCAATCTGCAGGAGACCCGAGATTTACCTGCTCGAGTGGAGA
TCAAGCCGGGAGATGAACTGCTGGTAGAATGTCACTACCAGACCCTGGACCGTGACTCCATGACTTTCGGAGGTCCCAGCACTGTCAATGAGATGTGCCT
CATCTTCCTCTTCTACTATCCCCGGAATAACATCTCCAGCTGCCACGGGTACCCTGACATCATCTTTGTGGCCCATGAGCTGGGGGAAGAGGTGTCAGAT
CCCATGGAGGGTATGATGGCCATGAACAACGTTGAGTGGACCCCAGAGAATATTAAGAAGGCTGAGAAGGCCTGCAAGGAGGCCCAGCAGACAGTGTTAA
TCAAGACCATTGATGAGGTTGTGGAAAACACAACAGGCTGGATTCCAAACATCACTCCCACTCCTCGGGGGCCCTGCTTGGAGTCCTCTGGAGGCAAAGT
GGAGCCCCAGGACAAGAAGCCTGCGGGCTTCAGGGCAGCACCAATGGCCTTGCCCAGCTCCAGTGCTACCACCCTGAGGTGCCTTCCCCTGACCGCCCTC
TTGTTTGTGCAGGGTGCCCTCTCCCGGCTCCTTGGCACACTGCAGACCAGGGGCTGA

>Oryctolagus_cuniculus No=23 length=618 name="European rabbit"
MACALLFRLSLLLALTAPSPGKHLGPKSRLRYSRFLGPSNVILLRWDFDLEAEIITFELQVQTAGWVGLGVMNRYTFVGSDLVVGGVLPDGNVYFSDQHL
VDEDTLEEDRSQDAELQGLTEDGIYTTMRFSRPFRSCDPHDQDIMGDTVRVLAAYGLNDTVELDRERTFVKSIFLLQIVHPDDLEVPEDTIIHDLEITDF
LIPEDDTTYACTFLPLPIVSKKHHIYKFEPKLAPHNETMVHHILLYACGNASVLPTGISDCYGADPAFSLCSQVIAGWAVGGTSYQFPDDVGISIGTPLD
PQWIRLEIHYSNFHNLPGLYDSSGIRVYYTSQLRKYDMGVLQLGFFTFPIHFIPPGAESFMSYGLCKTEKFEEMNGAPVPDIQVYGYLLHTHLAGRALQA
VQYRNGTQLRTICKDDSYDFNLQETRDLPARVEIKPGDELLVECHYQTLDRDSMTFGGPSTVNEMCLIFLFYYPRNNISSCHGYPDIIFVAHELGEEVSD
PMEGMMAMNNVEWTPENIKKAEKACKEAQQTVLIKTIDEVVENTTGWIPNITPTPRGPCLESSGGKVEPQDKKPAGFRAAPMALPSSSATTLRCLPLTAL
LFVQGALSRLLGTLQTRG

(1) Exon coordinates

Exon	UCSC Chromosome	Strand	Start	End
1	oryCun2 Un0060	-	1140127	1140414
2	oryCun2 Un0060	-	1138617	1138763
3	oryCun2 Un0060	-	1138130	1138291
4	oryCun2 Un0060	-	1137806	1137889
5	oryCun2 Un0060	-	1136150	1136317
6	oryCun2 Un0060	-	1135886	1135988
7	oryCun2 Un0060	-	1135537	1135703
8	oryCun2 Un0060	-	1134755	1134846
9	oryCun2 Un0060	-	1134383	1134479
10	oryCun2 Un0060	-	1134185	1134244
11	oryCun2 Un0060	-	1133249	1133379
12	oryCun2 Un0060	-	1132389	1132503
13	oryCun2 Un0060	-	1131851	1132093

24. House mouse (*Mus musculus*)

```
>Mus_musculus No=24 length=1860 name="House mouse"
ATGGCCTGTGTTCTACTCTTCAGACTTTTCTTACTTTTGGTCCTGGCAGCCTTTTCTCAAGGCAAGCGCCTGGGTCCTACATCTCCCCTTCGTTATTCCA
GGTTCCTAGATCCTTCCCGTGCTGTTTTCCTGCGCTGGGACTTTGACTATGAGGCTGAGATAATCACATTTGAGCTCCAAGTCCAAACAACTGGCTGGGT
TGGCCTGGGCATCACAGACCGGTACACCTTTGTGGGAAGTGATCTGGTAGTTGGAGGAGTCCTACCCAATGGCAATGTCTACTTTTCGGATCAGCACCTT
CTGGATGAAGACACTCTAGAACAGGATGGGAGCCAGGATGCTGAACTCCTAAGGCTCACAGAAGATGCTGTCTCGACCACCATGCGCTTTTCCAGGCCTT
TCAGAACCTGTGATCCACATGATCGTGACATTACGAGTGATACCATGAGGGTCCTGGCTGCCTATGGCCCGGATGACATACCAAAGATGAGTCGGGAGCA
TACTTTTGTCAAGTCCATCTTCTTGCTTCAAATGCTACAATATGATGATCAAGATGCCCCTGAAGACACCATCATCCATGACTTGAAGATCAGTAATTTC
ATCATTCCAGAGGATGATACCACATATGCCTGTACCTTTCTCCCACTCCCTATTGTCAGCAAAAAGCATCACATCTACAAGTTTGAACCTATATTGGTGG
AACGCAATGAGACGATGGTGCATCACGTTCTCGTGTATGCATGTGGCAATTCTAGTGTGCTCCCCACAGGCATCGGCGAATGCTATGGATCAGACCCTGC
CTTCTCTCTCTGCTCCCACGTCATCGCGGGCTGGGCTGTCGGGGGCCTTAGTTACCAGTTTCCAGATGATGTGGGCATCTCTATTGGAACCCCCTTTGAC
CCTCAGTGGATCAGACTGGAAATTCACTACAGCAATTTTCAGAACCTTCCTGGTATCCGTGATACCTCAGGGATGCGGCTGTTCTACACCTCGCACCTTC
GCAAATATGACATGGGAGTCCTCCAGCTGGGCATCTCAGTTTTCCCTATTCACTTTATACCCCCGGGTGCAGAGGCTTTCTTGTCCTATGGACTATGCAA
AACAGACAAGTTTGAAGAGCTGAATGGGGCTCCAGTATCTGACATATATATATCAGCCTGCCTGCTCCACACCCACTTAGCTGGGCGGTCACTGCAAGCT
CTGCAATACAGAAATGGAACCCAACTCCAAGTAGTATGTAAAGATTTTTCCTATGATTTCAATCTGCAAGAGTCTCGGGACTTACCTCATCCTGTGGTGA
TTAAGCCGGGGGATGAACTGCTGATAGAATGTCATTACCAGACACTGGATCGTGACTTCATGACTTTTGGAGGTGCCAGCACCATTAATGAGATGTGCCT
CATCTTCTTCTTCTACTATCCCAGAATTAACATCTCCAGTTGCATGGGATACCCTGATATCATCTATGTGACCAATGAGCTGGGGGAAGAAGCATCAGAG
AATCCCATGGAGAACCTGATGGTCCTGGATAATGTGGAGTGGACTCCAGAGAACATTAAGAAAGCTGAGAAAGCCTGCAAGGAGTCCCAGCAGACAGTAT
TGATCAAGACCATTGATGAAGAAGTAGAAAATACAACAGGTTGGATTCCTGATATTATACCAACTCCTCGAGGACCATGCTTAGAGTCTACTGGAGGCAA
GGTGGAACCTCAGGACAATACCCCGGCAGGCTTCAGGGCTGTACCACTGGCCCTCTCTGGCTCCAATACTGCTACTCTGAGGCCCCTGCCCATGATTGCT
GTACTGTTCCTGCAGGGTAGCCTCTCGTGTCTCCTTGCAATGCTGCAGACTGGAGTATGA
```

```
>Mus_musculus No=24 length=619 name="House mouse"
MACVLLFRLFLLLVLAAFSQGKRLGPTSPLRYSRFLDPSRAVFLRWDFDYEAEIITFELQVQTTGWVGLGITDRYTFVGSDLVVGGVLPNGNVYFSDQHL
LDEDTLEQDGSQDAELLRLTEDAVSTTMRFSRPFRTCDPHDRDITSDTMRVLAAYGPDDIPKMSREHTFVKSIFLLQMLQYDDQDAPEDTIIHDLKISNF
IIPEDDTTYACTFLPLPIVSKKHHIYKFEPILVERNETMVHHVLVYACGNSSVLPTGIGECYGSDPAFSLCSHVIAGWAVGGLSYQFPDDVGISIGTPFD
PQWIRLEIHYSNFQNLPGIRDTSGMRLFYTSHLRKYDMGVLQLGISVFPIHFIPPGAEAFLSYGLCKTDKFEELNGAPVSDIYISACLLHTHLAGRSLQA
LQYRNGTQLQVVCKDFSYDFNLQESRDLPHPVVIKPGDELLIECHYQTLDRDFMTFGGASTINEMCLIFFFYYPRINISSCMGYPDIIYVTNELGEEASE
NPMENLMVLDNVEWTPENIKKAEKACKESQQTVLIKTIDEEVENTTGWIPDIIPTPRGPCLESTGGKVEPQDNTPAGFRAVPLALSGSNTATLRPLPMIA
VLFLQGSLSCLLAMLQTGV
```

(1) Exon coordinates

Exon	UCSC Chromosome	Strand	Start	End
1	mm10 chr6	-	40887207	40887494
2	mm10 chr6	-	40885936	40886082
3	mm10 chr6	-	40885287	40885448
4	mm10 chr6	-	40884956	40885039
5	mm10 chr6	-	40884007	40884174
6	mm10 chr6	-	40883730	40883832
7	mm10 chr6	-	40883390	40883556
8	mm10 chr6	-	40882455	40882546
9	mm10 chr6	-	40882119	40882215
10	mm10 chr6	-	40881920	40881979
11	mm10 chr6	-	40880326	40880456
12	mm10 chr6	-	40879320	40879437
13	mm10 chr6	-	40878794	40879036

25. Brown Norway rat (*Rattus norvegicus*)

```
>Rattus_norvegicus No=25 length=1860 name="Brown Norway rat"
ATGGGCTATTTTCTCTTCTTCAGGCTTTTCTTACTTTGGGTCCTGGCAGCCTTTTCCCAAGGCAAGCGCCTTAGCCCTACATCTCCCCTTCGTTATTCCA
GATTCCTAGATCCTTCCCATGCTGTTTTCCTGCGCTGGGACTTTGATTATGAGACTGAGATAATCACATTTGAACTGCAAGTCCAAACAACTGGCTGGGT
TGGCCTGGGCATCACAGATCGCTACACGTTTGTGGGAAGTGATCTGGTAGTTGGAGGTGTCCTACCCAATGGCAATGTCTACTTTTCGGATCAGCACCTT
CTGGATGAAGACACTCTAGAACAGGATGGGAGTCAAGATGCTGAACTCCTAAAGCTCACAGAAGATTCTGTCTCGACCACTATGCGCTTTTCCCGGCCCT
TCAGATCCTGTGATCCACATGATCATGACATTACGAGTGATACCATGAGGGTCCTGGCTGCCTATGGCCTGGATGACATACCAAAGATGAATCGGGAGCG
TACTTTTGTCAAGTCCATCTTCTTGCTTCAAATACTACAATATGATGATGAAGATGCCCCTGAAGACACTGTCATCTATGACTTGAAGATCAGTAATTTC
CTCATTCCAGAGGAGGATACCACATATGCCTGCACCTTTCTCCCGCTCCCTATTGTCAGCAAAAAGCATCACATCTACAAGTTTGAACCTATATTGGTGG
AACGCAATGAGACAATGGTGCATCATGTTCTCGTGTATGCATGCGGCAATTCTAGTGTACTCCCCACAGGCATCGGTGAATGTTACGGAGCAGACCCTGC
CTTCTCCCTCTGCTCCCATGTCATCGCAGGCTGGGCTGTTGGGGGCCTTAGCTACCAGTTTCCAGATGATGTAGGCATCTCTATTGGAACCCCCTTTGAC
CCTCAATGGATCCGACTGGAAATTCACTACAGCAATTTTCAGAACCTTCCTGGTATCCGTGATACCTCAGGGATGCGGCTGTACTACACCTCGCACCTTC
GCAAATATGACATGGGAGTCCTCCAGCTGGGCATCTCAGTTTTCCCTATTCACTTTATACCCCCGGGTGCTGAGGCTTTCTTGTCCTATGGGCTATGCAA
GACAGACAAGTTTGAAGAGATGAATGGGGCTCCAGTAGCTGACATATATGTATCAGCCTGCCTGCTCCATACCCACTTAGCTGGGCGGTCACTGCAAGCT
CTGCAATACAGAAATGGAACCCAACTCCAAATAGTATGTAAAGATTTCTCCTATGATTTCAACCTGCAAGAATCTCGGGACCTACCTCATCCTGTAGTGA
TTAAGCCGGGGGATGAGCTGCTGATAGAATGCCATTATCAGACGCTGGATCGTGACTTCATGACTTTTGGAGGTGCCAGCACCATTAATGAGATGTGCCT
CATCTTCTTCTTCTACTATCCCAGAATTAACATCTCCAGTTGCATGGGATACCCTGATATCATCTATGTGACCAATGAGCTGGGGGAAGAAGCATCAGAG
AATCCCATGGAAAACCTCATGGTCCTGAATAATGTTGAGTGGACTCCAGAGAACATTAAGACAGCTGAGAAAGCCTGCAAGGAGTCCCAGCAGACAGTAT
TGATCAAGACGATTGATGAAGAAGTAGAAAATACAACAGGTTGGATTCCCGATATTATACCAACTCCTCGAGGAGCATGCTTAGAGTCTTCTGGAGGCAA
AGTGGAACCTCAGGACAAGACCCCTGCAGGCTTCAGGGCTGTACCAATGGCCCTCTCTGGCTCCAATACTGCTACTCTGAGGCACCTGCCCATGATTACT
GTCTTGTTCCTGCAGGGTTGCCTCTCATGGCTCCTTGCAATGCTACAGACTGGAGTATGA

>Rattus_norvegicus No=25 length=619 name="Brown Norway rat"
MGYFLFFRLFLLWVLAAFSQGKRLSPTSPLRYSRFLDPSHAVFLRWDFDYETEIITFELQVQTTGWVGLGITDRYTFVGSDLVVGGVLPNGNVYFSDQHL
LDEDTLEQDGSQDAELLKLTEDSVSTTMRFSRPFRSCDPHDHDITSDTMRVLAAYGLDDIPKMNRERTFVKSIFLLQILQYDDEDAPEDTVIYDLKISNF
LIPEEDTTYACTFLPLPIVSKKHHIYKFEPILVERNETMVHHVLVYACGNSSVLPTGIGECYGADPAFSLCSHVIAGWAVGGLSYQFPDDVGISIGTPFD
PQWIRLEIHYSNFQNLPGIRDTSGMRLYYTSHLRKYDMGVLQLGISVFPIHFIPPGAEAFLSYGLCKTDKFEEMNGAPVADIYVSACLLHTHLAGRSLQA
LQYRNGTQLQIVCKDFSYDFNLQESRDLPHPVVIKPGDELLIECHYQTLDRDFMTFGGASTINEMCLIFFFYYPRINISSCMGYPDIIYVTNELGEEASE
NPMENLMVLNNVEWTPENIKTAEKACKESQQTVLIKTIDEEVENTTGWIPDIIPTPRGACLESSGGKVEPQDKTPAGFRAVPMALSGSNTATLRHLPMIT
VLFLQGCLSWLLAMLQTGV
```

(1) Exon coordinates

Exon	UCSC Chromosome	Strand	Start	End
1	rn4 chr4	-	68513950	68514237
2	rn4 chr4	-	68512684	68512830
3	rn4 chr4	-	68512044	68512205
4	rn4 chr4	-	68511718	68511801
5	rn4 chr4	-	68510704	68510871
6	rn4 chr4	-	68510412	68510514
7	rn4 chr4	-	68510073	68510239
8	rn4 chr4	-	68509161	68509252
9	rn4 chr4	-	68508819	68508915
10	rn4 chr4	-	68508621	68508680
11	rn4 chr4	-	68507145	68507275
12	rn4 chr4	-	68506259	68506376
13	rn4 chr4	-	68505742	68505984

26. Chinese hamster (*Cricetulus griseus*)

```
>Cricetulus_griseus No=26 length=1860 name="Chinese hamster"
ATGGCCTGTGTTCTGCTCTGCAGACTGATTTTACTTTCAGTCCTGGCAGTCTTTTCCCAAGGCAAGCGCCTGGGCCCTACATCTCTCCTCCGTTATTCCA
GGTTCCTAGATCCTTCCCATGCTGTTTTTCTGCGCTGGGATTTTGATTATGAGGCTGAAATCATCACATTTGAGCTCCAAGTCCAAACAACTGGCTGGGT
TGGCCTGGGCATTACAAATCGCTACACCTTTGTGGGAAGTGACCTAGTAGTTGGAGGAGTCCTTCCTGATGGCAATGTCTACTTTTCGGATCAGCACCTT
CTGGATGAAGACTCTCTAGAACCGGATGGGAGCCAGGATGCTGAACTACTAAAGCTCACAGAAGATTCTGTCTCTACCACCATGCGCTTTTCCAGGCCCT
TCCGATCATGTGACCCTCATGACCGAGACATTACGAGTGACACCATGAGGGTCCTGGCTGCCTATGGTCTGGATGACATACCCAAGATGAATCCGGAGCG
TACTTTTGTCAAGTCAATCTTCTTGCTACAAATGCTACAATATGATGATCAAGATGCCCCTGAAGACACCATCATCCATGACTTGGCAATCAGTAATTTC
CTCATTCCAGAGGATGACACCACATACGCCTGCACCTTCCTCCCGCTCCCTATCGTCAGCAAGAAGCATCACATCTACAAGTTTGAACCCTTGCTAGTGG
AACGCAATGAGACAATGGTGCATCACATCCTCGTGTACGCATGCGGCAATGCCAGCGTGCTTCCCACAGGCATTGGTGAATGCTATGGGTCGGACCCTGC
CTTCTCCCTCTGCTCCCACGTCATCGCAGGCTGGGCTGTTGGGGGCCTTAGTTACCAGTTTCCAGATGATGTAGGCATCTCCATTGGAACCCCCTTGGAC
CCTCAGTGGATTCGACTGGAGGTTCACTACAGCAATTTTCAAAACCTTCCTGGTATACATGATACCTCAGGGATGCGGCTGTACTACACCTCACACCTTC
GCAAATATGACATGGGAGTCCTCCAGCTGGGCATCTCAGTTTTCCCTATTCACTTTATACCCCCGGGTGCTGAGGCTTTCCTGTCCTATGGGCTATGCAA
GACAGAGAAGTTTGAAGAGATGAATGGGGCCCCAGTACCTGACATATATGTGTATAGCTACTTGATCCACACCCACTTAGCTGGCCGCTCCCAGCAAGCT
GTGCAATACAGAAATGGAACCCAAGTCCAAATAATATGTAAAGATTATTCTTATGACTTCAATCTGCAAGAGACACGAGACTTACCTCATCCTGCAGTGA
TCAAACCGGGGGATGAGTTGCTGATAGAATGTAACTACCAGACGCTGGATCGTGACTCCATGACTTTTGGAGGTGCCAGCACCATTAATGAGATGTGTCT
CGTCTTCTTCTTCTACTATCCCAGAATTAACATCTCCAGTTGCCTGGGATACCCTGACATTATCTATGTGACTAATGAGCTGGGAGAAGAAGCATCAGAG
AATCCCATGGAGAACCTGATGGTCCTAAATAATGTTGAATGGACTCCAGAGAACATTAAGAAAGCTGAGAAAGCCTGCAAGGAGTCCCAGCAGACAGTGT
TGATCAAGACCATTGATGAAGAAGTAGAAAATACAACAGGTTGGATTCCTGATATTATCCCAACTCCTCGAGGGCCGTGCTTAGAGTCCTCTGGAGGCAA
AGTGGAACCTGGGGACAAGACCCCTGCAGGCTTCAGGGCTGCGCCAATGGTCCTCTCTGGTTCCAATACTGCTACCCTGAGGCACGTGCCCCTGGTTACT
ATCTTGTTCCTGCAGGGGACAGTCTCATGGCTCCTTCCCATGCTGCAGACTGGAGTATGA
```

```
>Cricetulus_griseus No=26 length=619 name="Chinese hamster"
MACVLLCRLILLSVLAVFSQGKRLGPTSLLRYSRFLDPSHAVFLRWDFDYEAEIITFELQVQTTGWVGLGITNRYTFVGSDLVVGGVLPDGNVYFSDQHL
LDEDSLEPDGSQDAELLKLTEDSVSTTMRFSRPFRSCDPHDRDITSDTMRVLAAYGLDDIPKMNPERTFVKSIFLLQMLQYDDQDAPEDTIIHDLAISNF
LIPEDDTTYACTFLPLPIVSKKHHIYKFEPLLVERNETMVHHILVYACGNASVLPTGIGECYGSDPAFSLCSHVIAGWAVGGLSYQFPDDVGISIGTPLD
PQWIRLEVHYSNFQNLPGIHDTSGMRLYYTSHLRKYDMGVLQLGISVFPIHFIPPGAEAFLSYGLCKTEKFEEMNGAPVPDIYVYSYLIHTHLAGRSQQA
VQYRNGTQVQIICKDYSYDFNLQETRDLPHPAVIKPGDELLIECNYQTLDRDSMTFGGASTINEMCLVFFFYYPRINISSCLGYPDIIYVTNELGEEASE
NPMENLMVLNNVEWTPENIKKAEKACKESQQTVLIKTIDEEVENTTGWIPDIIPTPRGPCLESSGGKVEPGDKTPAGFRAAPMVLSGSNTATLRHVPLVT
ILFLQGTVSWLLPMLQTGV
```

(1) Exon coordinates

Exon	NCBI Accession	Strand	Start	End
1	NW_003614308	-	501789	502076
2	NW_003614308	-	500493	500639
3	NW_003614308	-	499832	499993
4	NW_003614308	-	499505	499588
5	NW_003614308	-	498534	498701
6	NW_003614308	-	498257	498359
7	NW_003614308	-	497918	498084
8	NW_003614308	-	496985	497076
9	NW_003614308	-	496637	496733
10	NW_003614308	-	496441	496500
11	NW_003614308	-	494669	494799
12	NW_003614308	-	492162	492279
13	NW_003614308	-	491660	491902

27. Ord's kangaroo rat (*Dipodomys ordii*)

>Dipodomys_ordii No=27 length=1860 name="Ord's kangaroo rat"
ATGAGCTGTGTCCTTCTCTTCAGGCTTTTCCTACTTCTGGGCCTGGTAATACCTTCACAGGGCAGCCGCCTGGGGCCTACATCTCCCCTGCGCTATTCCA
GGTTCCTAGATTCTTCCAATATAATTTTCCTGCGCTGGGACTTTGATCTTCAGACTGAGATCATCACTTTTGAGCTCCAAGTCCAGACATCTGGTTGGGT
GGGCCTGGGCATTACCAATCGTTACACCAATGTGGGAGGTGATCTGGTGGTCGGAGGAGTCCTGCCCGATGGCAAAGTCTACTTCTCGGATCAGCATCTT
GTAGATGAAGACACCCTAGAGGAGGATGGGAGCCAGGATGCTGAGCTCCTGGGGCTGACAGAAGATGGCCTGTACACCACCATGCGCTTCTCCAGACCCT
TCCGCTCCTGCGACCCTCAGGATCAAGATATTACGAGCGACACCATGAGAGTCCTGACCGCCTATGGCCTGGATGACACACCCAAGCTTCACCGGAAACG
AACCTTCGTCAAGTCCATTTTCCTGCTGCAAATATTCTATCATGATGACACAGATGCTCCCGAAGATGCCATCATCTACGACCTGGAAATCACAGATTTC
CTCATTCCAGAGGCAGACACCACATATGCCTGCACCTTTCTTCCTCTCCCCATCGTCAGCAAGAAGCATCACATCTACAAGTTCGAGCCCAAGTTGCTGG
CCCACAATGAGACAATGGTGCACCACATCTTGGTGTATGCCTGTGGCAACGCCAGCACACTCCCCACGGGCATCGACGAATGCTATGGGTTTGATGCCGC
CTTCTCCCTCTGCTCCCAAGTCATCATGGGCTGGGCTGTCGGGGGCCTTAGCTACCAGCTTCCTGATGAGGTGGGCATCTCTATTGGGACCCCCTTGGAC
CCTCAGTGGATCCGACTGGAGATTCACTACAGCAACTTTCACAACCTTCCTGGTATCTATGACTCTTCAGGGATACGTCTATACTACACCTCTCACCTGC
GTAAATACGACCTAGGAATCCTGGAGCTGGGCGTCTTAACTTTTCCCATCCACTTCATTCCGCCGGGTGCTGAATCCTTCAAGTCCTATGGGCTGTGTAA
GACAGACAAGTTTGAAGAGATGAACGGAGCTCCAGTGAATGACATGATCGTAGCAAGCTACCTCCTCCACACTCACTTGGCTGGCCGGGCTCTGCGGGCT
GTGCAGTACAGAAATGGAAAACAACTCCGGACAATCTGTAAAGATGATTCCTACGACTTCAGTCTGCAGGAGACACGAGATTTACCTCATCTTGTGGACA
TCAAGCCGGGAGATGAGCTGCTGGTAGAGTGTAATTACCAGACACTGGATCGGGACTCCACAACGTTTGGAGGCATCAGCACCATTAATGAGATGTGCCT
CATCTTTCTCTTCTACTATCCGAGAAATAACATCTCCAGCTGCCTAGGCTTCCCTGACATCATCTATGTGGCCAACGAGCTGGGGGAGGAGGCGTCAGAG
AATCCCATGGAGAACCTGATCGTCTTGGAAAATATCGATTGGACTCCAGAGAACATAAGGAAGGCCGAGAAAGCCTGCAAGGAGGCCCGGCAGACGGTGA
TGATCAAAACCATCAATGAGCTGATTGAAAACACCACAGGATGGATTCCTGACATCACTCCTGCTCCTCGAGGGCCCTGCTTGGAGTCTTCTGGAGGCAA
AGTCGAGCCTCAGGACAAGAACCCTGCAGGATTCAGGGCAGCACCCGTGGCCCTCCCACATTCCAACATGGCCACCCCTGCAGCCTTTCCTCTGGCTGCC
CTCTTGTGGCTCCATGGTGCCCTCTTGTGGCTCTTGGTCATCCTCTAG

>Dipodomys_ordii No=27 length=615 name="Ord's kangaroo rat"
MSCVLLFRLFLLLGLVIPSQGSRLGPTSPLRYSRFLDSSNIIFLRWDFDLQTEIITFELQVQTSGWVGLGITNRYTNVGGDLVVGGVLPDGKVYFSDQHL
VDEDTLEEDGSQDAELLGLTEDGLYTTMRFSRPFRSCDPQDQDITSDTMRVLTAYGLDDTPKLHRKRTFVKSIFLLQIFYHDDTDAPEDAIIYDLEITDF
LIPEADTTYACTFLPLPIVSKKHHIYKFEPKLLAHNETMVHHILVYACGNASTLPTGIDECYGFDAAFSLCSQVIMGWAVGGLSYQLPDEVGISIGTPLD
PQWIRLEIHYSNFHNLPGIYDSSGIRLYYTSHLRKYDLGILELGVLTFPIHFIPPGAESFKSYGLCKTDKFEEMNGAPVNDMIVASYLLHTHLAGRALRA
VQYRNGKQLRTICKDDSYDFSLQETRDLPHLVDIKPGDELLVECNYQTLDRDSTTFGGISTINEMCLIFLFYYPRNNISSCLGFPDIIYVANELGEEASE
NPMENLIVLENIDWTPENIRKAEKACKEARQTVMIKTINELIENTTGWIPDITPAPRGPCLESSGGKVEPQDKNPAGFRAAPVALPHSNMATPAAFPLAA
LLWLHGALLWLLVIL

(1) Exon coordinates

Exon	UCSC Chromosome	Strand	Start	End
1	dipOrd1 1919	+	42496	42783
2	dipOrd1 1919	+	43682	43828
3	dipOrd1 1919	+	44561	44722
4	dipOrd1 1919	+	44969	45052
5	dipOrd1 1919	+	45824	45991
6	dipOrd1 1919	+	46397	46499
7	dipOrd1 1919	+	46627	46793
8	dipOrd1 1919	+	47109	47200
9	dipOrd1 1919	+	47467	47563
10	dipOrd1 1919	+	47696	47755
11	dipOrd1 1919	+	49333	49463
12	dipOrd1 1919	+	50206	50323
13	dipOrd1 1919	+	50637	50867

28. Naked mole rat (*Heterocephalus glaber*)

```
>Heterocephalus_glaber No=28 length=1860 name="Naked mole rat"
ATGGCCTGTATCCATCTCTTAAAGTTCTTCCTGCTTCTGGCCCTCACAGCCACCTCCCAAGGCAAACACCTTGGCCCCACATCTCACCTGCGTTACTCCA
GATTCATAGATCCTTCCAATGCCATTTTCCTGCGCTGGGACTTTGACCTTAAGGCTGAGGCCATCGTTTTTGAGCTCCAGGTTCAAACTGCTGGCTGGGT
AGGTGTGGGCATTGCAAATCGCTACACCGTTGTGGGAGGTGACCTGGTCGTTGGAGGAGTCCTGCCTGATGGCAATGTCTATTTCTCGGATCAGCACCTT
GTGGATGAAGACACTCTAGAGGAGGATGGGAGCCAGGATGCTGAGCTGCTGGGGCTAACAGAAGATGCTGCCTATACTACCATGCGCTTTTCTAGGCCTT
TCCGCTCCTGCGATCCTCATGACCAGGACATTACAAGTGACACGATTCGAGTCCTGACTGCCTATGGCCTGGATGACATGCTAAAGCTGAGGCGGGAGCG
CACTTTTGTCAAGTCCATCTTCCTGCTACAAATATTCCACCCTGAGGATCTGGATTTCCCCGAGGACACCATGGTCCATGACTTGGAGATCACTGATTTC
CTCATTCCAGAGGTCGATACCACCTACGCCTGCACCTTTCTGCCTCTCCCCATCGTCAGCGAGAAGCATCACATCTTCAAGTTTGAGCCCAAGTTGACAG
ACCACAACGAGACCATGGTGCACCACATCCTGGTGTATGCCTGCGGCAATGCCAGCACCCTCCCCAAGGGGATCAGCGAATGCTACGGATTCGACCCTGC
CTTCTCCCTCTGTTCACAGATTATCGTGGGCTGGGCTGTCGGGGGCCTTAGTTACCAGTTTCCAGATGAAGTGGGCATCTCTATAGGGACCCCCTTGGAC
CCTCAATGGATCCGCCTGGAGGTTCACTACAGTAATTTCCACAACCTACCTGGTGTGTATGACTCCTCAGGGATTCGATTGTACTACACCACTCGTCTGC
GGAAATACGATATGGGTGTCCTCCAGTTGGGCGTACTGACTTTCCCCATCCATTTCCTACCCCCGGGTGCTGAGTCCTTCATGTCTTATGGGCTGTGTAA
GACAGAGAAGTTTGAAGAGATGAATGGGGCCCCGGTGGCTGACATGAAGGTCATAGGCTACTTGCTCCACACCCACCTGGCTGGGCGGGCTCTCCAGGCT
GTGCAATTCAGAAATGGAAGACAGCTCCGAACAATCTGTAAAGATGATGCTTATGACTTCAATCTGCAAGAGACTCGAGATTTACCTCATCATGTGGAGA
TCAAGCCGGGAGATGAATTGCTGATAGAATGTAACTACCAGACGCTGGATCGGGACTCCATGACTTTCGGAGGTACCAGCACCCTTAATGAGATGTGCCT
TGTCTTCCTCTTCTACTATCCCCGAAATAACATCTCTAGCTGCCTGGGGTATCCTGACATCGGTCACGTGGTCCAGGAGCTGGGGGAAGCAGCTGCAGAA
AGTCCCATGGAGAACCTGATGGTCATGAGCAGTGTTGAATGGACTCCAGAGAACATTAAGGCAGCCGAGAAAGCCTGCAAGGAGGCCCGGCAGACAGTGA
TGCTCAAGACTATCAATGAGTTGATAGAAAACACCACAGGCTGGATTCCTGACATCATCCCTACACCTCGGGGTCCTTGTTTGGAGTCCTCTGGAGGCAA
AGTGGAGCCCCAGGACCAGACCCCTGCAGGCTTCCGGGCTGCACCAGTGGTCCTTTCTGGCTCCAACACTGCTACCCTGAGGAGCATCCCCCTGGCTGCC
CTCTTGTTCCTGCAGGGCACCATTCCATGGCTCCTTGCCATGCTGCAGACTGGAATCTAA

>Heterocephalus_glaber No=28 length=619 name="Naked mole rat"
MACIHLLKFFLLLALTATSQGKHLGPTSHLRYSRFIDPSNAIFLRWDFDLKAEAIVFELQVQTAGWVGVGIANRYTVVGGDLVVGGVLPDGNVYFSDQHL
VDEDTLEEDGSQDAELLGLTEDAAYTTMRFSRPFRSCDPHDQDITSDTIRVLTAYGLDDMLKLRRERTFVKSIFLLQIFHPEDLDFPEDTMVHDLEITDF
LIPEVDTTYACTFLPLPIVSEKHHIFKFEPKLTDHNETMVHHILVYACGNASTLPKGISECYGFDPAFSLCSQIIVGWAVGGLSYQFPDEVGISIGTPLD
PQWIRLEVHYSNFHNLPGVYDSSGIRLYYTTRLRKYDMGVLQLGVLTFPIHFLPPGAESFMSYGLCKTEKFEEMNGAPVADMKVIGYLLHTHLAGRALQA
VQFRNGRQLRTICKDDAYDFNLQETRDLPHHVEIKPGDELLIECNYQTLDRDSMTFGGTSTLNEMCLVFLFYYPRNNISSCLGYPDIGHVVQELGEAAAE
SPMENLMVMSSVEWTPENIKAAEKACKEARQTVMLKTINELIENTTGWIPDIIPTPRGPCLESSGGKVEPQDQTPAGFRAAPVVLSGSNTATLRSIPLAA
LLFLQGTIPWLLAMLQTGI
```

(1) Exon coordinates

Exon	NCBI Accession	Strand	Start	End
1	NW_004624765	-	22264428	22264715
2	NW_004624765	-	22263379	22263525
3	NW_004624765	-	22262698	22262859
4	NW_004624765	-	22262456	22262539
5	NW_004624765	-	22261025	22261192
6	NW_004624765	-	22260731	22260833
7	NW_004624765	-	22260322	22260488
8	NW_004636903	+	35001	35092
9	NW_004636903	+	35377	35473
10	NW_004636903	+	35615	35674
11	NW_004636903	+	36531	36661
12	NW_004636903	+	37408	37525
13	NW_004636903	+	38224	38466

29. Thirteen-lined ground squirrel (*Ictidomys tridecemlineatus*)

```
>Ictidomys_tridecemlineatus No=29 length=1857 name="Thirteen-lined ground squirrel"
ATGACCTGTGTACTTCTCTTTAAACTTTTTCTACTTCTGGTCCTGTCAGACACCTGCCCAGGCAGCCACCTCGGCCCCACATCCCGCCTGCGCTATTCCA
GGTTCCTTGACCCTTCCAATGTCATTTTCCTGCTCTGGGACTTTGACTTTGAGGCTGAGATCATCACTTTTGAGCTCCAGGTCCGGACAGCTGGCTGGGT
GGGCTTGGGCATCAGCCACCGCCACACTGTGGTGGGAAGTGACCTGGTTGTTGGAGGAGTCCTGCCTAATGGCCATGTCTATTTCTCGGATCAGCACCTT
GTGGATGAAAACACCCTGGAGGAGGACGGGAGCCAGGATGCCGAGCTGCTGGGGCTCACAGAAGACGCTGTCTACACAACCATGCGCTTTTCCAGGCCCT
TCCGCTCCTGTGATCCTCACGATCAAGACATCACGAGTGACACCATCAGGGTGCTGGCTGCCTACGGCCTGGATGACACACTGAAGCTGGATCCCGAGCG
TACTTTCGTTAAGTCCATCTTCCTGCTACAAATGTTCCACCCTGACGATCTGGAGGTCCCCGAGGAGATCATCATCCATGACCTGGAGATCACTGACTTC
CTCATTCCAGAAGATGATACCACATATGCCTGTACCTTTCTTCCTCTACCCATCGTCAGCAAGAAGCATCACATTTACAAGTTCGAGCCCAAGCTGGTGG
AGCACAATGAGACTATGGTGCATCACATCCTGGTGTATGCCTGTGGCAATGCCAGCGTGCTCCCCAAGGGCATCAACGACTGCTACGGGGCCGACCCTGA
CTTCTCCCTCTGCTCCCAGGTCATCGTGGGCTGGGCTGTCGGGGGCACTAGTTACCAGTTTCCAGACAACGTGGGCATCTCTCTTGGGACCCCCTTGGAC
CCTCAGTGGATTCGTCTAGAGATTCACTACAGCAATTTCCACAACCTTCCCGGTGTATATGACACATCAGGGATCCGACTCTACTACACCTCTCACCTGC
GTGAATACGACATGGGAGTCCTCCAGCTGGGTGTCTTCACATTCCCTATACACTTCATTCCCCCGGGTGCTGAGTCCTTTATGTCCTACGGGCTGTGTAA
GACGGAGAAGTTTGAAGAGATGAACGGCACCCCGGTGGCTGACATCCAGGTGTTTGGCTACCTGCTCCACACCCACTTGGCTGGGCAGGCGCTGCAGGCT
GTGCAATACAGAAATGGAACACAGCTCCAAACAATCTGTAAAGATGACTCCTATGACTTCAATCTTCAGGAGACACGAGACTTACCACACCGGGTGGTGA
TCAAGCCGGGAGATGAACTGCTCATAGAGTGTCGCTACCAGACGCTGGACCGTGACTCTGTGATTTTTGGAGGCCCCAGCACTCTCAACGAGATGTGTCT
TGTGTTTCTCTTCTACTACCCACGAAATAACATCTCCAGCTGCCAGGGCTACCCTGACATCATCTCTGTGGCCCGTGAGCTGGGGGAGGAGGTATCAGAT
CCCATGGAGGGTACCATGGTCATGAGCAGCATTGAGTGGACTCCAGAGAGCATCAAGAAGGCCGAGAGAGCCTGCAAGGAGTCCCAGCAGATTGTGGTCA
TCAAGACTATCGATGAGCTGGTAGAAAACACAACAGGCTGGATTCCTGACATCATCCCCACTCCTCGGGGGCCCTGCTTGGAATCCTCCGGAGGCAAAGT
GGAGCCCCAGGACCACACCCCTGCGGGCTTCAGGGCTGCACCGGTGGCCCTCTCGGGGTCCTCCACTGCTGCCCTGAACCACCTGCCGCTGGCTGCCCTC
TTGTTCATGCAGGGCACCCTCTCGTGGCTCCTTGCCATGCTACAGCCTGAAGTCTGA

>Ictidomys_tridecemlineatus No=29 length=618 name="Thirteen-lined ground squirrel"
MTCVLLFKLFLLLVLSDTCPGSHLGPTSRLRYSRFLDPSNVIFLLWDFDFEAEIITFELQVRTAGWVGLGISHRHTVVGSDLVVGGVLPNGHVYFSDQHL
VDENTLEEDGSQDAELLGLTEDAVYTTMRFSRPFRSCDPHDQDITSDTIRVLAAYGLDDTLKLDPERTFVKSIFLLQMFHPDDLEVPEEIIIHDLEITDF
LIPEDDTTYACTFLPLPIVSKKHHIYKFEPKLVEHNETMVHHILVYACGNASVLPKGINDCYGADPDFSLCSQVIVGWAVGGTSYQFPDNVGISLGTPLD
PQWIRLEIHYSNFHNLPGVYDTSGIRLYYTSHLREYDMGVLQLGVFTFPIHFIPPGAESFMSYGLCKTEKFEEMNGTPVADIQVFGYLLHTHLAGQALQA
VQYRNGTQLQTICKDDSYDFNLQETRDLPHRVVIKPGDELLIECRYQTLDRDSVIFGGPSTLNEMCLVFLFYYPRNNISSCQGYPDIISVARELGEEVSD
PMEGTMVMSSIEWTPESIKKAERACKESQQIVVIKTIDELVENTTGWIPDIIPTPRGPCLESSGGKVEPQDHTPAGFRAAPVALSGSSTAALNHLPLAAL
LFMQGTLSWLLAMLQPEV
```

(1) Exon coordinates

Exon	NCBI Accession	Strand	Start	End
1	AGTP01119753	-	21850	22137
2	AGTP01119753	-	21219	21365
3	AGTP01119753	-	20560	20721
4	AGTP01119753	-	20250	20333
5	AGTP01119753	-	18878	19045
6	AGTP01119753	-	18591	18693
7	AGTP01119753	-	18261	18427
8	AGTP01119753	-	17888	17979
9	AGTP01119753	-	17536	17632
10	AGTP01119753	-	17337	17396
11	AGTP01119753	-	15855	15985
12	AGTP01119753	-	15008	15122
13	AGTP01119753	-	14478	14720

30. Black flying fox (*Pteropus alecto*)

```
>Pteropus_alecto No=30 length=1857 name="Black flying fox"
ATGGCCCGTGTCCTTCTCCTCAGGCTTCTCCTGCTTACAGCGCTGGCAGCCCCCTCCCAAGGCAACCGCCTCGGATCCACACCTCGCCTGCGTTATTCCA
GGTTCCTAGATCCTTCCAATGTCATTTTCCTGCGCTGGGACTTTGACCTGGAGGCTGAGGTCATCACTTTTGAGCTCCAGGTCCGGACGGCTGGCTGGGT
GGGCCTGGGTGTCACGCATCGCTACACCAACGTGGGAGGTGACCTTGTTGTGGGAGCAGTCTTGCCCAATGGCAATGTCTATTTCTCGGACCAGCACCTA
GTGGATGAAGACACCCTGGAGGAGGACGGGCGTCAGGACGCCGAGCTGCTGGGGCTAACGGAAGACGCTGTCTATACCACCATGCGCTTCTCCAGGCCCT
TCCGCTCCTGCGACCCTCACGACCTAGACATCACGGGTGACACCGTGAGGGTGCTGGCGGCCTACGGCCTGGATGACACACTCAAGATGGACCAGGGGCG
TGCTTTCGTCAAGTCCATCTTCCTGCTACAAGTGGTCCACCCAGATGACCTGGATGCCCCTGAGGACGCCATCATCCATGACTTGGAAATCACTGATTTC
CTCATCCCAGAGGATGACACCACGTATGCCTGTACCTTCCTCCCCCTCCCCATCGTCAGCAAGAAACATCACATCTACAAGTTTGAGCCCAAGCTGATGG
ACCACAACGAGACGATAGTGCACCACATCCTCGTGTACGCCTGTGGCAATGCCAGCGTCCTCCCCACGGGCATCAGCGACTGCTACGGGGCTGACCCCGC
CTTCTCCCTCTGCTCGCAGGTCATCGTGGGCTGGGCTGTCGGGGGCACCAGTTACCAGTTCCCAGACGACGTGGGCGTCTCCATTGGGACGCCCTTGGAC
CCCCAGTGGATCCGGCTGGAGATTCATTACAGCAATTTCCACAACCGTCCCGGCCTGTACGACACCTCGGGGATCCGCATGTACTACACGGCGCGGCTGC
GCGCGCACGACATGGGCGTCCTGCAGCTGGGCGTCTTCACCTTCCCCATCCACTTCATCCCGCCGCGCGCCGAGTCCTTCCTGTCCTACGGGCTGTGCAA
GACCGAGAAGTTTGAAGAGATGAACGGGGCCCCTGTGCCCGACATACAGGTGTATGGCTACCTGCTCCACACCCACCTGACTGGGCGGGCGCTGCAGGCC
GTGCAGTACAGAAATGGAACGCAACTCCGAACGATCTGTAAAGATGCTTCCTACGACTTCAACCTGCAGGAGACTCGAGATTTACCTGGTCGAGTGGAGA
TCAAGCCGGGAGATGAGTTGCTGGTCGAGTGTCACTACCAGACGCTGGACCGCGACTCCCTGACTTTTGGGGGTCCCAGCACCATTAATGAGATGTGCCT
CATCTTCCTCTTCTACTACCCCCGAAACAACATCTCCAGCTGCATGGGGTACCCTGACATCATCTACGTGGCCCATGAGCTGGGGGAGGAGGTTTCAGAT
TCCATGGAGGGCATGATGGCCATAAACAACGTCGAGTGGACCCCGGAGAGCATTAAGAAGGCCGAGAAGGCCTGCAAGGAGGCTGAGCAGATGGTGATAA
TAAAGACCATTGATGAGGTGGTGGAAAACACGACAGGCTTGATTCCGGAAATCACGCCTACTCCCCGGGGGCCCTGCGTGGAGTCCTCCGGAGGCAAAGT
GGAGCCCCAGGACGAGAGCCCTGCAGGCTTCAAGGCTTCGCCGATGGCCCTCTCGGACGCGGGCACTGCCACCCTGAGGTGCCTCCCCCTGGCTGCCCTC
TTGTTTGGGCAGGGGGCCCTCTCTTGGCTCCTTGCCACCCTGCAGACTAGAGCCTGA
```

```
>Pteropus_alecto No=30 length=618 name="Black flying fox"
MARVLLLRLLLLTALAAPSQGNRLGSTPRLRYSRFLDPSNVIFLRWDFDLEAEVITFELQVRTAGWVGLGVTHRYTNVGGDLVVGAVLPNGNVYFSDQHL
VDEDTLEEDGRQDAELLGLTEDAVYTTMRFSRPFRSCDPHDLDITGDTVRVLAAYGLDDTLKMDQGRAFVKSIFLLQVVHPDDLDAPEDAIIHDLEITDF
LIPEDDTTYACTFLPLPIVSKKHHIYKFEPKLMDHNETIVHHILVYACGNASVLPTGISDCYGADPAFSLCSQVIVGWAVGGTSYQFPDDVGVSIGTPLD
PQWIRLEIHYSNFHNRPGLYDTSGIRMYYTARLRAHDMGVLQLGVFTFPIHFIPPRAESFLSYGLCKTEKFEEMNGAPVPDIQVYGYLLHTHLTGRALQA
VQYRNGTQLRTICKDASYDFNLQETRDLPGRVEIKPGDELLVECHYQTLDRDSLTFGGPSTINEMCLIFLFYYPRNNISSCMGYPDIIYVAHELGEEVSD
SMEGMMAINNVEWTPESIKKAEKACKEAEQMVIIKTIDEVVENTTGLIPEITPTPRGPCVESSGGKVEPQDESPAGFKASPMALSDAGTATLRCLPLAAL
LFGQGALSWLLATLQTRA
```

(1) Exon coordinates

Exon	NCBI Accession	Strand	Start	End
1	KB030581	+	830069	830356
2	KB030581	+	831202	831348
3	KB030581	+	831836	831997
4	KB030581	+	832236	832319
5	KB030581	+	833039	833206
6	KB030581	+	833330	833432
7	KB030581	+	833552	833718
8	KB030581	+	834066	834157
9	KB030581	+	834454	834550
10	KB030581	+	834693	834752
11	KB030581	+	835891	836021
12	KB030581	+	836580	836694
13	KB030581	+	837021	837263

31. Big brown bat (*Eptesicus fuscus*)

>Eptesicus_fuscus No=31 length=1857 name="Big brown bat"
ATGGCCCGTGTCCTTCTCCTCAGGCTTCTCCTGCTTACAGCGCTGGCAGCCCCCTCCCAAGGCAACCGCCTCGGATCCACACCTCGCCTGCGTTATTCCA
GGTTCCTAGATCCTTCCAATGTCATTTTCCTGCGCTGGGACTTTGACCTGGAGGCTGAGGTCATCACTTTTGAGCTCCAGGTCCGGACGGCTGGCTGGGT
GGGCCTGGGTGTCACGCATCGCTACACCAACGTGGGAGGTGACCTTGTTGTGGGAGCAGTCTTGCCCAATGGCAATGTCTATTTCTCGGACCAGCACCTA
GTGGATGAAGACACCCTGGAGGAGGACGGGCGTCAGGACGCCGAGCTGCTGGGGCTAACGGAAGACGCTGTCTATACCACCATGCGCTTCTCCAGGCCCT
TCCGCTCCTGCGACCCTCACGACCTAGACATCACGGGTGACACCGTGAGGGTGCTGGCGGCCTACGGCCTGGATGACACACTCAAGATGGACCAGGGGCG
TGCTTTCGTCAAGTCCATCTTCCTGCTACAAGTGGTCCACCCAGATGACCTGGATGCCCCTGAGGACGCCATCATCCATGACTTGGAAATCACTGATTTC
CTCATCCCAGAGGATGACACCACGTATGCCTGTACCTTCCTCCCCCTCCCCATCGTCAGCAAGAAACATCACATCTACAAGTTTGAGCCCAAGCTGATGG
ACCACAACGAGACGATAGTGCACCACATCCTCGTGTACGCCTGTGGCAATGCCAGCGTCCTCCCCACGGGCATCAGCGACTGCTACGGGGCTGACCCCGC
CTTCTCCCTCTGCTCGCAGGTCATCGTGGGCTGGGCTGTCGGGGGCACCAGTTACCAGTTCCCAGACGACGTGGGCGTCTCCATTGGGACGCCCTTGGAC
CCCCAGTGGATCCGGCTGGAGATTCATTACAGCAATTTCCACAACCGTCCCGGCCTGTACGACACCTCGGGGATCCGCATGTACTACACGGCGCGGCTGC
GCGCGCACGACATGGGCGTCCTGCAGCTGGGCGTCTTCACCTTCCCCATCCACTTCATCCCGCCGCGCGCCGAGTCCTTCCTGTCCTACGGGCTGTGCAA
GACCGAGAAGTTTGAAGAGATGAACGGGGCCCCTGTGCCCGACATACAGGTGTATGGCTACCTGCTCCACACCCACCTGACTGGGCGGGCGCTGCAGGCC
GTGCAGTACAGAAATGGAACGCAACTCCGAACGATCTGTAAAGATGCTTCCTACGACTTCAACCTGCAGGAGACTCGAGATTTACCTGGTCGAGTGGAGA
TCAAGCCGGGAGATGAGTTGCTGGTCGAGTGTCACTACCAGACGCTGGACCGCGACTCCCTGACTTTTGGGGGTCCCAGCACCATTAATGAGATGTGCCT
CATCTTCCTCTTCTACTACCCCCGAAACAACATCTCCAGCTGCATGGGGTACCCTGACATCATCTACGTGGCCCATGAGCTGGGGGAGGAGGTTTCAGAT
TCCATGGAGGGCATGATGGCCATAAACAACGTCGAGTGGACCCCGGAGAGCATTAAGAAGGCCGAGAAGGCCTGCAAGGAGGCTGAGCAGATGGTGATAA
TAAAGACCATTGATGAGGTGGTGGAAAACACGACAGGCTTGATTCCGGAAATCACGCCTACTCCCCGGGGGCCCTGCGTGGAGTCCTCCGGAGGCAAAGT
GGAGCCCCAGGACGAGAGCCCTGCAGGCTTCAAGGCTTCGCCGATGGCCCTCTCGGACGCGGGCACTGCCACCCTGAGGTGCCTCCCCCTGGCTGCCCTC
TTGTTTGGGCAGGGGGCCCTCTCTTGGCTCCTTGCCACCCTGCAGACTAGAGCCTGA

>Eptesicus_fuscus No=31 length=618 name="Big brown bat"
MACDLLFRLLLLMVLAAPSQGNRLGPTSRLRYSRFLDPSNVIFLRWDFDLEAEMITFELQVRTAGWVGLGVTNGYTFVGGDLVVGGVLPEGNVYFSDQHL
VDEDTLEADGSQDAKLLKLTEDGVYTTMLFSRPFRSCDPHDKDITSDTVRVLAAYGLDDTLKLDPERTFVKSIFLLQILHPDDHDVPEDTIIHDLEITDF
LIPEDDTTYACTFLPLPIVSKKHHIYKFEPKLVSHNETMVHHILVYACGNASVLPTGISDCYGADPAFSLCSQVIVGWAVGGTSYQFPDDVGVSIGTPLD
PQWIRLEVHYSNFHNLPGVHDTSGIRMYYTAHLRKYDMGVLQLGFLTFPIHFIPPGAESFMSYGLCKTEKFEEMNGAPVPDIQVYGYLLHTHLAGRALQA
VQYRNGTQLRTICKDDSYDFNLQETRDLPHRVEIKPGDELLIECHYETLDRDFLTFGGPSTINEMCLIFLFYFPRNNISSCMGYPDIIYVAQELGEEASD
SMEGMMAMNNVEWTLENIKKAEKACKESQQTVIIKTIDEVVGNTTGWIPEIIPTPRGPCLESSGGKVEPQDETPAGFRAAPMALLASGNATPRHLPMAAL
LFGQGAFFWLLATLQAGI

(1) Exon coordinates

Exon	NCBI Accession	Strand	Start	End
1	ALEH01088560	+	10451	10738
2	ALEH01088560	+	11582	11728
3	ALEH01088560	+	12241	12402
4	ALEH01088560	+	12641	12724
5	ALEH01088560	+	13516	13683
6	ALEH01088560	+	13858	13960
7	ALEH01088560	+	14137	14303
8	ALEH01088560	+	14616	14707
9	ALEH01088560	+	14991	15087
10	ALEH01088560	+	15224	15283
11	ALEH01088560	+	16155	16285
12	ALEH01088560	+	17009	17123
13	ALEH01088560	+	17412	17654

32. Little brown bat (*Myotis lucifugus*)

>Myotis_lucifugus No=32 length=1857 name="Little brown bat"
ATGGCCTGTGACCTTCTCTTCAGGCTCCTTCTACTTATGGTTCTGGCAGCCACCTCCCAAGGCAACCACCTTGGTCCCACCTCTCGATTGCGTTATTCCA
GGTTCCTAGATCCTTCCAATGTCATTTTCCTGCGCTGGGACTTTGACCTTGAGGCTGAGATGATCACGTTTGAGCTCCAGGTCCGAACAGCTGGCTGGGT
GGGCTTGGGAGTCACAAATGGCTACACCTTCGTGGGAGCCGATCTGGTTGTTGGAGGAGTCTTGCCTGATGGCAATGTCTATTTCTCGGATCAGCACCTG
GTAGATGAAGACACCCTGGAGGAGGACGGGAGTCAGGATGCTAAGCTGCTGGGTCTAACAGAAGATGATGTCTATACCACCATGCTTTTCTCCAGGCCCT
TCCGCTCCTGTGACCCTCATGACAAAGACATTACGAGTGACACGGTGAGAGTGCTGGCTGCCTACGGCCTGGATGACATGCTGAAGCTGGATCCGGAGCG
TACTTTTGTCAAGTCCATCTTCCTGCTACAAATAGTCCACCCAGATGATCTTGATGTACCTGAGGACACAATCATCCATGACTTGGAGATCACTGATTTC
CTTATCCCAGAGGATGACACCACATATGCCTGCACCTTCCTCCCTCTCCCCATCGTCAGTAAGAAGCATCACATCTACAAGTTTGAGCCCAAGCTGGTCT
ACCACAATGAGACGATGGTGCATCACATCCTTGTGTACGCCTGTGGCAATGCCAGTGCTCTGCCCACGGGCATCAGCGACTGCTATGGGGCTGACCCCGC
CTTCTCCCTCTGCTCCCAGGTCATCGTGGGCTGGGCTGTCGGGGGCACAAGTTACCAGTTTCCAGATGATGTGGGCGTCTCTATTGGGACCCCCTTGGAC
CCCCAGTGGATCCGACTGGAGATTCATTACAGCAACTTTCACAACCTTCCTGGTGTGCACGACACCTCGGGGATTCGAATGTACTACACGGCACACCTGC
GCAAATACGACATGGGAGTCCTGCAGCTGGGCTTCCTCACGTTCCCCATCCACTTCATCCCCCCGGGTGCTGAGTCCTTCATGTCCTACGGACTGTGTAA
GACAGAGAAGTTTGAAGAGATGAATGGGGCCCCGGTGCCTGACATACAGGTGTATGGCTACCTGCTCCATACCCATCTGGCTGGGCGGGCTCTGCAGGCT
GTGCAGTACAGAAATGGAACACAACTCCGAACAATCTGTAAAGATGACACCTATGACTTCAATCTGCAGGAGACTCGAGATTTACCTCATCGAGTGGAGA
TCAAGCCGGGAGACGAATTGCTAATAGAGTGTCACTACCAGACACTGGACCGAGACTTCTTGACTTTTGGGGGTCCCAGCACCATTAACGAGATGTGCCT
CGTCTTCCTCTTCTACTATCCCCGAAACAATATCTCCAGCTGCATGGGGTACCCTGACATCATCTATGTGGCCCATGAGCTGGGGGAGGAGGCATCAGAT
TCCATGGAGGGCATGATGGCCATGAACAATGTTGAGTGGACCCTGGAGAACATTAAGAAGGCTGAGAAAGCCTGCAAGGAGGCCCAGCAAACCGTGTTAA
TAAAGACCATTGACGAAGTAGTGGAAAACACAACAGGCTGGATTCCAGAAATCATCCCTACACCTCGGGGGCCCTGTTTGGAGTCATCGGGAGGCAAAGT
GGAGCCCCAGGATGAGACCCCTGCAGGCTTCAGGGCTGCACCAATGGCCCTCTTGGCCTCTGGCAATGCTACCCTGAGGCACCTCCCTATGGCTGCCCTC
TTGTTTGGGCAGGGGGCCTTCTTTTGGCTCCTTGCTACCCTGCAGGCTGGAATCTGA

>Myotis_lucifugus No=32 length=618 name="Little brown bat"
MACDLLFRLLLLMVLAATSQGNHLGPTSRLRYSRFLDPSNVIFLRWDFDLEAEMITFELQVRTAGWVGLGVTNGYTFVGADLVVGGVLPDGNVYFSDQHL
VDEDTLEEDGSQDAKLLGLTEDDVYTTMLFSRPFRSCDPHDKDITSDTVRVLAAYGLDDMLKLDPERTFVKSIFLLQIVHPDDLDVPEDTIIHDLEITDF
LIPEDDTTYACTFLPLPIVSKKHHIYKFEPKLVYHNETMVHHILVYACGNASALPTGISDCYGADPAFSLCSQVIVGWAVGGTSYQFPDDVGVSIGTPLD
PQWIRLEIHYSNFHNLPGVHDTSGIRMYYTAHLRKYDMGVLQLGFLTFPIHFIPPGAESFMSYGLCKTEKFEEMNGAPVPDIQVYGYLLHTHLAGRALQA
VQYRNGTQLRTICKDDTYDFNLQETRDLPHRVEIKPGDELLIECHYQTLDRDFLTFGGPSTINEMCLVFLFYYPRNNISSCMGYPDIIYVAHELGEEASD
SMEGMMAMNNVEWTLENIKKAEKACKEAQQTVLIKTIDEVVENTTGWIPEIIPTPRGPCLESSGGKVEPQDETPAGFRAAPMALLASGNATLRHLPMAAL
LFGQGAFFWLLATLQAGI

(1) Exon coordinates

Exon	UCSC Chromosome	Strand	Start	End
1	myoLuc2 GL429908	-	1487015	1487302
2	myoLuc2 GL429908	-	1486009	1486155
3	myoLuc2 GL429908	-	1485345	1485506
4	myoLuc2 GL429908	-	1485023	1485106
5	myoLuc2 GL429908	-	1484077	1484244
6	myoLuc2 GL429908	-	1483801	1483903
7	myoLuc2 GL429908	-	1483477	1483643
8	myoLuc2 GL429908	-	1483081	1483172
9	myoLuc2 GL429908	-	1482703	1482799
10	myoLuc2 GL429908	-	1482507	1482566
11	myoLuc2 GL429908	-	1481504	1481634
12	myoLuc2 GL429908	-	1480677	1480791
13	myoLuc2 GL429908	-	1480146	1480388

33. Brandt's bat (*Myotis brandtii*)

>Myotis_brandtii No=33 length=1857 name="Brandt's bat"
ATGGCCTGTGACCTTCTCTTCAGGCTCCTTCTACTTATGGTCCTGGCAGCCCCCTCCCGAGGCAACCACCTTGGTCCCACCTCTCGATTGCGTTATTCCA
GGTTCCTAGATCCTTCCAATGTCATTTTCCTGCGCTGGGACTTTGACCTTGAGGCTGAGATGATCACTTTTGAGCTCCAGGTCCGAACAGCTGGCTGGGT
GGGCTTGGGAGTCACAAATGGCTACACCTTCGTGGGAGCCGATCTGGTTGTTGGAGGAGTCTTGCCTGATGGCAATGTCTATTTCTCGGATCAGCACCTG
GTGGATGAAGACACCCTGGAGGAGGACGGGAGTCAGGATGCTAAGCTGCTGGGTCTAACAGAAGATGGTGTCTATACCACCATGCTTTTCTCCAGGCCCT
TCCGCTCTTGTGACCCTCATGACAAAGACATTACGAGTGACACGGTGAGAGTGCTGGCTGCCTATGGCCTGGATGACATGCTGAAGCTGGACCCGGAGCG
TACTTTTGTCAAGTCCATCTTCCTGCTACAAATAGTCCACCCAGATGATCTTGATGTACCTGAGGACACAATCATCCATGACTTGGAGATCACTGATTTC
CTTATCCCAGAGGACGACACCACATATGCCTGCACCTTCCTCCCTCTCCCCATCGTCAGTAAGAAGCATCACATCTACAAGTTTGAGCCCAAGCTGGTCT
ACCACAATGAGACGATGGTGCATCACATCCTTGTGTACGCCTGTGGCAATGCCAGTGCTCTGCCCACGGGCATCAGCGACTGCTATGGGGCTGACCCTGC
CTTCTCCCTCTGCTCCCAGGTCATCGTGGGCTGGGCTGTCGGGGGCACAAGTTACCAGTTTCCAGATGATGTGGGCGTCTCTATTGGGACCCCCTTGGAC
CCCCAGTGGATCCGACTGGAGATTCATTACAGCAATTTTCACAACCTTCCTGGTGTGCACGACACCTCGGGGATTCGAATGTACTACACGGCACACCTGC
GCAAATACGACATGGGAGTCCTGCAGCTGGGCTTCCTCACGTTCCCCATCCACTTCATCCCCCCGGGTGCTGAGTCCTTCATGTCCTACGGACTGTGTAA
GACAGAGAAGTTTGAAGAGATGAATGGGGCCCCGGTGCCTGACATACAGGTATATGGCTACCTGCTCCATACCCATCTGGCTGGGCGGGCTCTGCAGGCT
GTGCAGTACAGAAATGGAACACAACTTCGAACAATCTGTAAAGATGACACCTATGACTTCAATCTGCAGGAGACTCGAGATTTACCTCATCGAGTGGAGA
TCAAGCCGGGAGACGAATTGCTAATAGAGTGTCACTACCAGACACTGGACCGAGACTCCTTGACTTTTGGGGGTCCCAGCACCATTAATGAGATGTGCCT
CGTCTTCCTCTTCTACTATCCCCGAAACAATATCTCCAGCTGCATGGGGTACCCTGACATCATCTATGTGGCCCATGAGCTGGGGGAGGAGGCATCAGAT
TCCATGGAGGGCATGATGGCCATGAACAACGTTGAGTGGACCCTGGAGAACATTAAGAAGGCTGAGAAAGCCTGCAAGGAGGCCCAGCAAACCGTGTTAA
TAAAGACAATTGACGAAGTAGTGGAAAACACAACAGGCTGGATTCCAGAAATCATCCCTACGCCTCGGGGGCCCTGTTTGGAGTCATCGGGAGGCAAAGT
GGAGCCCCAGGATGAGACCCCTGCAGGCTTCAGGGCTGCACCAATGGCCCTCTTGGCCTCTGGCAATGCTACCCTGAGGCACCTCCCTATGGCTGCCCTC
TTGTTTGGGCAGGGGGCCTTCTTTTGGCTCCTTGCTACCCTGCAGGCTGGAATCTGA

>Myotis_brandtii No=33 length=618 name="Brandt's bat"
MACDLLFRLLLLMVLAAPSRGNHLGPTSRLRYSRFLDPSNVIFLRWDFDLEAEMITFELQVRTAGWVGLGVTNGYTFVGADLVVGGVLPDGNVYFSDQHL
VDEDTLEEDGSQDAKLLGLTEDGVYTTMLFSRPFRSCDPHDKDITSDTVRVLAAYGLDDMLKLDPERTFVKSIFLLQIVHPDDLDVPEDTIIHDLEITDF
LIPEDDTTYACTFLPLPIVSKKHHIYKFEPKLVYHNETMVHHILVYACGNASALPTGISDCYGADPAFSLCSQVIVGWAVGGTSYQFPDDVGVSIGTPLD
PQWIRLEIHYSNFHNLPGVHDTSGIRMYYTAHLRKYDMGVLQLGFLTFPIHFIPPGAESFMSYGLCKTEKFEEMNGAPVPDIQVYGYLLHTHLAGRALQA
VQYRNGTQLRTICKDDTYDFNLQETRDLPHRVEIKPGDELLIECHYQTLDRDSLTFGGPSTINEMCLVFLFYYPRNNISSCMGYPDIIYVAHELGEEASD
SMEGMMAMNNVEWTLENIKKAEKACKEAQQTVLIKTIDEVVENTTGWIPEIIPTPRGPCLESSGGKVEPQDETPAGFRAAPMALLASGNATLRHLPMAAL
LFGQGAFFWLLATLQAGI

(1) Exon coordinates

Exon	NCBI Accession	Strand	Start	End
1	ANKR01258667	-	23587	23874
2	ANKR01258667	-	22583	22729
3	ANKR01258667	-	21919	22080
4	ANKR01258667	-	21597	21680
5	ANKR01258667	-	20646	20813
6	ANKR01258667	-	20370	20472
7	ANKR01258667	-	20027	20193
8	ANKR01258667	-	19631	19722
9	ANKR01258667	-	19253	19349
10	ANKR01258667	-	19057	19116
11	ANKR01258667	-	18055	18185
12	ANKR01258667	-	17225	17339
13	ANKR01258667	-	16694	16936

34. David's bat (*Myotis davidii*)

>Myotis_davidii No=34 length=1857 name="David's bat"
ATGGCCTGTGACCTTCTCTTCAGGCTCCTTCTACTTATGGTTCTGGCAGCCCCCTCCCGAGGCAACCACCTTGGTCCCACCTCTCGATTGCGTTATTCCA
GGTTCCTAGATCCTTCCAATGTCATTTTCCTGCGCTGGGACTTTGACCTTGAGGCTGAGATGATCACTTTTGAGCTTCAGGTCCGAACAGCTGGCTGGGT
GGGCTTGGGAGTCACAAATGGCTACACCTTCGTGGGAGCCGATCTGGTTGTTGGAGGAGTCTTGCCTGATGGCAATGTCTATTTCTCGGATCAGCACCTG
GTGGATGAAGACACCCTGGAGATGGACGGAAGTCAGGATGCTAAGCTGCTCGGTCTAACAGAAGATGGTGTCTATACCACCATGCTTTTCTCCAGGCCCT
TCCGCTCCTGTGACCCTCATGACAAAGACATTACGAGTGACACGGTAAGGGTGCTGGCTGCCTATGGCCTGGATGACACGCTGAAGCTGGACCCGGAGCG
CACTTTTGTCAAGTCCATCTTCCTGCTACAAATAGTCCACCCAGATGATCTTGATGTACCTGAGGACACAATCATCCATGACTTGGAGATCACTGATTTC
CTTATCCCAGAGGATGACACCACATATGCCTGCACCTTCCTCCCTCTCCCCATCGTCAGTAAGAAGCATCACATCTACAAGTTTGAGCCCAAGCTGGTCT
ACCACAACGAGACGATGGTGCATCACATCCTTGTGTATGCCTGTGGCAATGCCAGTGCTCTGCCCATGGGCATCAGCGACTGCTATGGGGCTGACCCTGC
CTTCTCCCTCTGCTCCCAGGTCATCGTGGGCTGGGCTGTCGGGGGCACAAGTTACCAGTTTCCAGATGATGTGGGCGTCTCTATTGGGACCCCCTTGGAC
CCCCAGTGGATCAGACTGGAGATTCATTACAGCAATTTTCACAACCTTCCTGGCGTGCATGACACCTCAGGGATTCGAATGTACTACACGGCACACCTGC
GCAAATACGACATGGGAGTCCTGCAGCTGGGCTTCCTCACGTTCCCCATCCACTTCATCCCCCCGGGGGCTGAGTCCTTCATGTCCTACGGACTGTGTAA
GACAGAGAAGTTTGAAGAGATGAATGGGGCCCCGGTGCCTGACATACAGGTATATGGCTACCTGCTCCATACCCATCTGGCTGGGCGGGCTCTGCAGGCT
GTGCAGTACAGAAATGGAACACAACTCCGAACAATCTGTAAAGATGACACCTATGACTTCAATCTGCAGGAGACTCGAGATTTACCTCATCGAGTGGAGA
TCAAGCCGGGAGACGAATTGCTAATAGAGTGTCACTACCAGACACTGGACCGAGACTCCTTGACTTTTGGGGGTCCTAGCACCATTAATGAGATGTGCCT
CGTCTTCTTCTTCTACTATCCTCGAAACAACATCTCCAGCTGCATGGGGTATCCTGACATCATCTATGTGGCCCATGAGCTGGGGGAGGAGGCATCAGAT
TCCATGGAGGGCATGATGGCCATGAACAATGTTGAGTGGACCCTGGAGAACATTAAGAAGGCTGAGAAAGCCTGCAAGGAGGCCCAGCAAACCGTGTTAA
TAAAGACCATTGATGAAGTAGTGGGAAACACAACAGGCTGGATTCCAGAAATCATCCCTACGCCTCGGGGGCCCTGTTTGGAGTCCTCAGGAGGCAAAGT
GGAGCCCCAGGATGAGACCCCTGCAGGCTTCAGGGCTGCACCAATGGCCCTCTTGGCCTCTGGCAATGCTACCCTGAGGCACCTCCCTATGGCTGCCCTC
TTATTTGGGCAGGGGGCCTTCTTTTGGCTCCTCGCTACCCTGCAGGCTGGAATCTGA

>Myotis_davidii No=34 length=618 name="David's bat"
MACDLLFRLLLLMVLAAPSRGNHLGPTSRLRYSRFLDPSNVIFLRWDFDLEAEMITFELQVRTAGWVGLGVTNGYTFVGADLVVGGVLPDGNVYFSDQHL
VDEDTLEMDGSQDAKLLGLTEDGVYTTMLFSRPFRSCDPHDKDITSDTVRVLAAYGLDDTLKLDPERTFVKSIFLLQIVHPDDLDVPEDTIIHDLEITDF
LIPEDDTTYACTFLPLPIVSKKHHIYKFEPKLVYHNETMVHHILVYACGNASALPMGISDCYGADPAFSLCSQVIVGWAVGGTSYQFPDDVGVSIGTPLD
PQWIRLEIHYSNFHNLPGVHDTSGIRMYYTAHLRKYDMGVLQLGFLTFPIHFIPPGAESFMSYGLCKTEKFEEMNGAPVPDIQVYGYLLHTHLAGRALQA
VQYRNGTQLRTICKDDTYDFNLQETRDLPHRVEIKPGDELLIECHYQTLDRDSLTFGGPSTINEMCLVFFFYYPRNNISSCMGYPDIIYVAHELGEEASD
SMEGMMAMNNVEWTLENIKKAEKACKEAQQTVLIKTIDEVVGNTTGWIPEIIPTPRGPCLESSGGKVEPQDETPAGFRAAPMALLASGNATLRHLPMAAL
LFGQGAFFWLLATLQAGI

(1) Exon coordinates

Exon	NCBI Accession	Strand	Start	End
1	ALWT01228485	+	23645	23932
2	ALWT01228485	+	24791	24937
3	ALWT01228485	+	25454	25615
4	ALWT01228485	+	25852	25935
5	ALWT01228485	+	26715	26882
6	ALWT01228485	+	27056	27158
7	ALWT01228485	+	27335	27501
8	ALWT01228485	+	27806	27897
9	ALWT01228485	+	28187	28283
10	ALWT01228485	+	28420	28479
11	ALWT01228485	+	29351	29481
12	ALWT01228485	+	30193	30307
13	ALWT01228485	+	30597	30839

35. Cat (*Felis catus*)

```
>Felis_catus No=35 length=1857 name="Cat"
ATGACCTGTGCCCTTCTCTTCAGGCTTCTCCTACTTATGACTCTGGCAGCCCTCTTCCAAGGCAAACGCTTTGGCCCCACATCTCGCCTGCGTTACTCCA
GGTTCCTAGATCCTTCCAATGTCATTTTCCTGCGCTGGGACTTTGACCTTGAGGCTGAGATCATCACGTTTGAGCTCCAGGTCCGGACAGCCGGCTGGGT
GGGCTTGGGTGTCACAAATCGCTACACCAGAGTGGGGAGCGATCTGGTGGTTGGAGGAGTCCTGCCCGATGGCAATGTCTATTTCTCGGATCAGCACCTG
GTGGATGAAGACACCCTGGAGGAGGACGGGAGCCAGGATGCTGAGCTGCAGGGGCTGACAGAAGACGCTGTCTACACCACCATGCGCTTCTCCAGGCCCT
TCCGCTCCTGTGATGCCCACGACCAAGACATTACGAGTGACACCATAAGGGTTCTGGCTGCCTATGGCCTGGATGACACTCTGAAGCTGGATCGGGATCG
TACTTTTGTCAAGTCCATCTTCCTGCTACAAATAGTCCACCCTGACGACCTTGACGTCCCTGAGGACACCATCATCCATGACTTGGAACTCACTGATTTC
CTCATCCCAGAAGATGACACCACATATGCCTGCACCTTCCTCCCTCTCCCCATCGTCAGCAAGAAGCATCACATCTTCAAGTTTGAGCCCAAGCTGCTCA
ATCACAACGAGACGATGGTGCATCACATCCTGGTTTATGCCTGTGGCAATGCCAGTGTTCTCCCCACGGGCATCAGTGACTGCTACGGGGCTGACCCTGC
CTTCTCCCTCTGCTCGCAGGTCATCGTGGGCTGGGCTGTCGGGGGCACAAGTTACCAGTTTCCAGATGATGTGGGTGTCTCTATTGGGACTCCCTTAGAC
CCCCAGTGGATCCGATTGGAGATTCATTACAGCAATTTTCACAACGTTCCGGGTGTGTACGACTCCTCAGGCATCCGATTATACTACACGGCAAAGCTGC
GCAAATACGACATGGGAGTCCTCCAGCTGGGTTTCTTCACTTTCCCCATCCACTTCATACCCCCAGGCGCGGAGTCCTTCCTGTCCTATGGGCTGTGTAA
GACGGAGAAGTTTGAGGAGATGAATGGGGTCCCAATGCCTGACATACAGGTATATGGCTACCTGCTCCACACCCACCTGGCTGGCCGTGCTCTGCAGGCC
GTGCAATACAGAAATGGAACACAACTCCGAATAATCTGCAAAGATGATTTCTACGACTTCAATCTGCAGGAGACTCGAGATTTACCTTATCGAATGGAGA
TTAAACAGGGAGATGAGTTGCTGGTGGAGTGTCATTACCAGACGCTGGACCGGGACTCCTTGACTTTTGGGGGTCCCAGCACCATTAATGAGATGTGCCT
CATCTTCCTCTTCTACTATCCCCGAAACAACATCTCCAGCTGCATGGGGTACCCTGACATCATCCACGTGGCCCATGAGCTGGGGGAGGAGGTATCAGAT
TCCATGGAGGGCATGATAGCCATGAACAATGTTGAATGGACCCCAGAGAACATTAAGAAGGCTGAGAAAGCCTGCAAGGAGGCCCAGCAGACAGTGATAA
TAAAGACCATTGATGAGGTAGTGGAAAACACAACAGGCTGGATTCCAGAAATTATCCGTGCGCCTCGGGGACCCTGCTTGGAGTCCTCCGGAGGCAAAGT
GGAGCCCCAGGACAAAACCCCTGCAGGCTTCAGGGCTGCACCAATTGCCCTCTCAGGCTCCAGCACTGCTACCCTGAGGTGCCTCCCTCTGGCTGCCCTC
TTGTTTGGGCAGGGGGCTCTTTCTTGGCTTCTTGCCACCCTGCAGGTTGGAGTCTGA
```

```
>Felis_catus No=35 length=618 name="Cat"
MTCALLFRLLLLMTLAALFQGKRFGPTSRLRYSRFLDPSNVIFLRWDFDLEAEIITFELQVRTAGWVGLGVTNRYTRVGSDLVVGGVLPDGNVYFSDQHL
VDEDTLEEDGSQDAELQGLTEDAVYTTMRFSRPFRSCDAHDQDITSDTIRVLAAYGLDDTLKLDRDRTFVKSIFLLQIVHPDDLDVPEDTIIHDLELTDF
LIPEDDTTYACTFLPLPIVSKKHHIFKFEPKLLNHNETMVHHILVYACGNASVLPTGISDCYGADPAFSLCSQVIVGWAVGGTSYQFPDDVGVSIGTPLD
PQWIRLEIHYSNFHNVPGVYDSSGIRLYYTAKLRKYDMGVLQLGFFTFPIHFIPPGAESFLSYGLCKTEKFEEMNGVPMPDIQVYGYLLHTHLAGRALQA
VQYRNGTQLRIICKDDFYDFNLQETRDLPYRMEIKQGDELLVECHYQTLDRDSLTFGGPSTINEMCLIFLFYYPRNNISSCMGYPDIIHVAHELGEEVSD
SMEGMIAMNNVEWTPENIKKAEKACKEAQQTVIIKTIDEVVENTTGWIPEIIRAPRGPCLESSGGKVEPQDKTPAGFRAAPIALSGSSTATLRCLPLAAL
LFGQGALSWLLATLQVGV
```

(1) Exon coordinates

Exon	UCSC Chromosome	Strand	Start	End
1	felCat5 A2	-	156600042	156600329
2	felCat5 A2	-	156598786	156598932
3	felCat5 A2	-	156598108	156598269
4	felCat5 A2	-	156597811	156597894
5	felCat5 A2	-	156596867	156597034
6	felCat5 A2	-	156596575	156596677
7	felCat5 A2	-	156596223	156596389
8	felCat5 A2	-	156595796	156595887
9	felCat5 A2	-	156595470	156595566
10	felCat5 A2	-	156595250	156595309
11	felCat5 A2	-	156593590	156593720
12	felCat5 A2	-	156592730	156592844
13	felCat5 A2	-	156592175	156592417

36. Dog (*Canis lupus familiaris*)

>Canis_lupus_familiaris No=36 length=1857 name="Dog"
ATGACTTGTGCCCTTCTCTTCAGGCTTCTCCTACTTACGGCCCTGGTAGTCCCCTCCCCAGGCAAAGGCCTTGGTCCCACATCTCGCCTGCGTTATTCCA
GGTTCCTAGATCCGTCCAACGTCATTTTCCTGCGCTGGGACTTTGACCTTGAGGCTGAGATCATCACTTTTGAGCTGCAGGTCCGGACAGCTGGCTGGGT
GGGCTTGGGTGTCACAAATCGTAACGCCAGAACGGGGAGCGATCTGGTTGTTGGAGGAGTCCTGCCCGACGGCAACGTCTATTTCTCGGATCAGCACCTG
GTAGATGAAGACACCCTGGAGGAGGACGGGAGCCAGGATGCTGAGCTGCAGGGGCTGACGGAGGATGCTGTCTACACCACCATGCGCTTCTCCAGGCCCT
TCCGCTCTTGTGACCCTCACGACCAAGACATTACGAGTGACACCATGAGGGTGCTGGCCACCTATGGCCTGGATGACACTCTGAAGCTGGACCGGGACCG
TACTTTTGTCAAGTCTATCTTCCTGCTACAAATAGTCCACCCTGATGACCTTGACATTCCTGAGGATACCATCATCCATGACCTGGAGATCACTGATTTC
CTCATTCCAGAGGATGACACCACGTATGCCTGCACCTTCCTCCCTCTCCCCATCGTTAGCAAGAAGCATCACATCTTCAAGTTTGAGCCCAAGATGGTGG
AGCATGATGAGACAATGGTGCATCACATCCTGGTGTATGCCTGTGGCAATGCCAGCGTTCTACCCACGGGCATCAGTGACTGCTATGGAGCTGACCCTGC
CTTCTCCCTCTGCTCACAGGTCATCGTAGGCTGGGCTGTCGGGGGCACAAGTTACCAGTTTCCAGATGATGTGGGTGTCTCTCTTGGGACCCCCTTGGAC
CCCCAGTGGATCCGACTGGAGATTCACTACAGCAATTTTCACAACCTTCCTGGTGTGTATGACTCCTCGGGGATCCGATTATACTACACTGCACAGCTGC
GCAAATATGACATGGGAGTCCTCCAGCTGGGCTTCTTCACTTTCCCCATCCACTTCATACCCCCGGGCGCAGAGTCCTTCCTGTCCTATGGGCTGTGCAA
GACGGAGAAGTTTGAGGAGATGAATGGGGCCCCAGTGCCTGACATACAGGTGTACGGCTACCTGCTCCACACCCACCTGGCTGGCCGTGCTCTGCAGGCT
GTGCAATACAGAAATGGGACACAACTCCAAACAATCTGCAAAGATGATTCCTATGACTTCAATCTGCAGGAGACTCGGGATTTACCGTATCGAGTGGAGA
TCAAACCGGGAGACGAGTTGCTGGTAGAGTGTCACTACCAGACGCTGGACCGGGACTCCTTGACTTTTGGGGGTCCCAGCACAATTAATGAGATGTGCCT
CATCTTCCTCTTCTACTATCCCCGAAACAACATCTCCAGCTGCATGGGGTACCCTGACATCATCCATGTGGCCCATGAGCTGGGGGAGGAGGTATCAGAC
TCCATGGAGGGCATGATGGCCATGAACAATATTGAGTGGACTCCAGAGAACATTAAGAAGGCTGAGAAAGCCTGCAAGGGCGCCCAGCAGATGGTGATAA
TAAAGACCATTGATGAGGTGGTGGAAAATACGACAGGCTGGATTCCAGAAATCATCCCTACTACTCGGGGACCCTGCTTGGAGTCCTCTGGAGGCAAAGT
GGAACCCCAGGACAAAACCCCTGCAGGTTTCAGGGCTGCACCAGTGGGCCTCTCAAGCTCCAGCACTGCTACCCTGAGGTGCCTCCCTCTGGCTGCCCTC
TTGTTTGGGCAGGGGGCTTTTTCTTGGCTTCTTGCCACCCTGAAGGTTGTAGTCTGA

>Canis_lupus_familiaris No=36 length=618 name="Dog"
MTCALLFRLLLLTALVVPSPGKGLGPTSRLRYSRFLDPSNVIFLRWDFDLEAEIITFELQVRTAGWVGLGVTNRNARTGSDLVVGGVLPDGNVYFSDQHL
VDEDTLEEDGSQDAELQGLTEDAVYTTMRFSRPFRSCDPHDQDITSDTMRVLATYGLDDTLKLDRDRTFVKSIFLLQIVHPDDLDIPEDTIIHDLEITDF
LIPEDDTTYACTFLPLPIVSKKHHIFKFEPKMVEHDETMVHHILVYACGNASVLPTGISDCYGADPAFSLCSQVIVGWAVGGTSYQFPDDVGVSLGTPLD
PQWIRLEIHYSNFHNLPGVYDSSGIRLYYTAQLRKYDMGVLQLGFFTFPIHFIPPGAESFLSYGLCKTEKFEEMNGAPVPDIQVYGYLLHTHLAGRALQA
VQYRNGTQLQTICKDDSYDFNLQETRDLPYRVEIKPGDELLVECHYQTLDRDSLTFGGPSTINEMCLIFLFYYPRNNISSCMGYPDIIHVAHELGEEVSD
SMEGMMAMNNIEWTPENIKKAEKACKGAQQMVIIKTIDEVVENTTGWIPEIIPTTRGPCLESSGGKVEPQDKTPAGFRAAPVGLSSSSTATLRCLPLAAL
LFGQGAFSWLLATLKVVV

(1) Exon coordinates

Exon	UCSC Chromosome	Strand	Start	End
1	canFam3 chr16	+	7020163	7020450
2	canFam3 chr16	+	7020958	7021104
3	canFam3 chr16	+	7021582	7021743
4	canFam3 chr16	+	7021964	7022047
5	canFam3 chr16	+	7022821	7022988
6	canFam3 chr16	+	7023178	7023280
7	canFam3 chr16	+	7023469	7023635
8	canFam3 chr16	+	7023985	7024076
9	canFam3 chr16	+	7024305	7024401
10	canFam3 chr16	+	7024543	7024602
11	canFam3 chr16	+	7025894	7026024
12	canFam3 chr16	+	7026774	7026888
13	canFam3 chr16	+	7027197	7027439

37. Giant panda (*Ailuropoda melanoleuca*)

```
>Ailuropoda_melanoleuca No=37 length=1857 name="Giant panda"
ATGACCTGTGCCCTTCTCTTCAGGCTTTTCCTACTTATGGCCCTGGCGGTCCCCTCCCAAGGCAAATGCCTTGGCCCCACATCTCGCCTGCGTTATTCCA
GGTTCCTAGATCCTTCCAATGTCATTTTCCTGCGCTGGGACTTTGACCTTGAGACTGAGATCATCACTTTTGAGCTGCAGGTCCGGACAACTGGCTGGGT
GGGCTTGGGTGTCACAAGTCGCTACACCAGAGCAGGGAGTGATCTGGTTGTTGGAGGAGTCCTGCCTGACGGCAACATCTATTTCTCGGACCAACACCTG
GTGGATGAAGACACCCTGGAGGAGGACGGGAGCCAGGATGCTGAGCTGCAGGGGCTGACGGAAGATGCTGTCTACACCACCATGCGCTTCTCCAGGCCCT
TCCGCTCCTGTGACCCTCACGACCAAGACATTACGAGCGACACCATAAGGGTGCTGGCCACTTACGGCCAGGACGACACTCTGAAGCTGGATCAGGACCG
TACATTTGTCAAGTCCATCTTCCTGCTACAAGTAGTCCATCCTGACGACCTTGATGTCCCTGAGGATACCATCATCCATGACTTGGAGATTACCGATTTC
CTCATTCCAGAGGATGACACCACGTATGCCTGCACCTTCCTCCCTCTCCCCATCGTTAGCAGGAAGCATCACATCTTCAAGTTTGAGCCAAAGCTGCTTG
AGCACAACGAGACGATGGTGCATCACATCCTGGTGTATGCCTGTGGCAATGCCAGTGTTCTCCCCACGGGCATCAGTGACTGCTATGGGGCTGACCCTGC
CTTCTCCCTCTGCTCGCAGGTCATCGTGGGCTGGGCTGTCGGGGGCACAAGTTACCAGTTTCCAGATGATGTGGGTGTCTCTCTTGGGACCCCCTTAGAC
CCGCAGTGGATCCGACTGGAGATTCATTACAGCAATTTTCACAACCTTCCCGGCGTGTATGACTCCTCAGGCATCAGATTATACTACACCGCACGGCTGC
GCAAATACGACATGGGGGTCCTCCAGCTGGGCTTCTTCACTTTCCCCATCCACTTCATACCCCCAGGCGCGGAGTCCTTCCTGTCCTATGGGCTGTGTAA
GACAGAGAAGTTTGAGGAGATGAATGGGGCCCCAGTGCCTGACATACAGGTATATGGCTACCTGCTCCACACCCACCTGGCTGGCCGTGCTCTGCAGGCC
GTGCAATACAGAAACGGAGCACAACTCCGAACAATCTGCAAAGATGATTCCTATGACTTCAATCTGCAGGAGACTCGAGATTTACCTTATCGAGCAGAGA
TCAAACCGGGAGATGAGTTGCTGGTGGAATGTCACTACCAGACACTGGACCGGGACTCCTTGACTTTTGGCGGTCCCAGCACCATTAATGAGATGTGCCT
CATCTTCCTCTTCTACTATCCCCGAAACAACATCTCCAGCTGCATGGGGTACCCTGACATCATCCACGTGGCCCAGGAGCTTGGGGAGGAGGTATCAGAC
TCCATGGAGGGCATGATGGCCATGAACAATGTTGAGTGGACCCCAGAGAACATTAAGAAGGCTGAGAAAGCCTGCAAGGAGGCCCAGCAGATGGTGATAA
TAAAAACCATTGATGAATTGGTGGAAAACACGACAGGCTGGATTCCGGAAATCATCCCAGCTACACGGGGACCCTGCTTGGAGTCCTCTGGAGGCAAAGT
GGAGCCCCAGGACCAAACCCCTGCAGGCTTCAGGGCTGCACCAGTAGCCCTCTCAGGCTCTAGCACTGCTACCCTGAGGTGCCTCCCTTTGGCTGCCCTC
TTGTTTGGGCAGGGGGTTCTTTCTTGGCTCCTTGCTACCCTGCGGGTTGGAGTCTGA

>Ailuropoda_melanoleuca No=37 length=618 name="Giant panda"
MTCALLFRLFLLMALAVPSQGKCLGPTSRLRYSRFLDPSNVIFLRWDFDLETEIITFELQVRTTGWVGLGVTSRYTRAGSDLVVGGVLPDGNIYFSDQHL
VDEDTLEEDGSQDAELQGLTEDAVYTTMRFSRPFRSCDPHDQDITSDTIRVLATYGQDDTLKLDQDRTFVKSIFLLQVVHPDDLDVPEDTIIHDLEITDF
LIPEDDTTYACTFLPLPIVSRKHHIFKFEPKLLEHNETMVHHILVYACGNASVLPTGISDCYGADPAFSLCSQVIVGWAVGGTSYQFPDDVGVSLGTPLD
PQWIRLEIHYSNFHNLPGVYDSSGIRLYYTARLRKYDMGVLQLGFFTFPIHFIPPGAESFLSYGLCKTEKFEEMNGAPVPDIQVYGYLLHTHLAGRALQA
VQYRNGAQLRTICKDDSYDFNLQETRDLPYRAEIKPGDELLVECHYQTLDRDSLTFGGPSTINEMCLIFLFYYPRNNISSCMGYPDIIHVAQELGEEVSD
SMEGMMAMNNVEWTPENIKKAEKACKEAQQMVIIKTIDELVENTTGWIPEIIPATRGPCLESSGGKVEPQDQTPAGFRAAPVALSGSSTATLRCLPLAAL
LFGQGVLSWLLATLRVGV
```

(1) Exon coordinates

Exon	UCSC Chromosome	Strand	Start	End
1	ailMel1 GL192847.1	+	36264	36551
2	ailMel1 GL192847.1	+	37405	37551
3	ailMel1 GL192847.1	+	38062	38223
4	ailMel1 GL192847.1	+	38446	38529
5	ailMel1 GL192847.1	+	39326	39493
6	ailMel1 GL192847.1	+	39695	39797
7	ailMel1 GL192847.1	+	39986	40152
8	ailMel1 GL192847.1	+	40487	40578
9	ailMel1 GL192847.1	+	40805	40901
10	ailMel1 GL192847.1	+	41042	41101
11	ailMel1 GL192847.1	+	42228	42358
12	ailMel1 GL192847.1	+	43100	43214
13	ailMel1 GL192847.1	+	43525	43767

38. Weddel seal (*Leptonychotes weddellii*)

>Leptonychotes_weddellii No=38 length=1857 name="Weddel seal"
ATGACCTGTGCCCTTCTCTTCAGACTTCTCCTACTTACGGCCCTGGCAGCCCCCTCCCAAGGCAAACACCTTGGCCCCACATCTCGCCTGCGTTATTCCA
GGTTCCTAGATCCTTCCAATGTCATTTTCCTGTGCTGGGACTTTGACCTTGAGGCTGAGATCATCACTTTTGAGCTGCAGGTCCGGACAACTGGCTGGGT
GGGCTTGGGTGTCACAAATCGCTACACCAGAGTGGGGAGTGATCTGGTTGTTGGAGGAGTCCTGCCTGATGGCAACATCTATTTCTCGGATCAGCACCTG
GTGGATGAAGACACCCTGGAGGAGGACGGGAGCCAGGATGCTGAGCTGCAAGGGCTGACCGAAGATGCTATCTACACCACCATGCGCTTCTCCAGGCCCT
TCCGCTCCTGTGACCCTCACGACAAAGACATTACGAGTGACACCATAAAGGTGCTGGCCACCTATGGCCTGGATGACACTCTGAAGCTGGATCGGGACCG
TACTTTTATCAAGTCCATCTTCCTGCTACAAATAGTCCATCCTGATGACCTTGATGTCCCTGAGGATACCATCATCCATGACTTGGAGATCACTGATTTC
CTCGTTCCAGAGGATGATACCACATATGCCTGCACCTTCCTCCCTCTCCCCATCGTTAGCAAGAAGCATCACATCTTCAAGTTTGAGCCCAAGCTGCCGG
AGCACCACGAGTCAATGGTGCATCACATCCTGGTGTATGCATGTGGCCATGCCAGTGTTCTCCCCACGGGCATCAGTGACTGCTATGGGGCTGACCCTGC
CTTCTCCCTCTGCTCACAGGTCATCGTGGGCTGGGCTGTCGGGGGCACAAGTTACCAGTTTCCAGATGATGTGGGTATCTCTATTGGGACCCCCTTAGAC
CCCCAGTGGATACGACTGGAGATTCATTACAGCAATTTTCACAACCTTCCTGGTGTGTATGACTCCTCAGGCATCCGATTATACTACACTGCACAGCTGC
GCAAATACGACACGGGGGTCCTCCAGCTGGGCTTCTTCACTTTCCCCATCCACTTCATACCCCCAGGCGCAGAGTCCTTCCTGTCCTATGGGCTGTGTAA
GACAGAGAAGTTTGAAGAGATGAATGGGGCCCCAGTGCCTGACATACAGGTCTATGGCTACCTGCTCCACACCCACCTGGCTGGCCGTGCTCTGCAGGCC
GTGCAATACAGAAATGGAGCACAACTCCGAACAATCTGCAAAGATGATTCCTATGACTTCAGTCTGCAGGAGACTCGAGATTTACCTTATCGAGCAGAGA
TCAAACTGGGAGATGAGTTGCTGGTGGAATGTCACTACCAGACGCTGGACCGGGACTCCTTGACTTTTGGGGGTCCCAGCACCATTAATGAGATGTGCCT
CATCTTCCTCTTCTACTATCCCCGAAACAACATCTCCAGCTGCATGGGGTACCCTGACATCATCTATGTGGCCCATGAGCTTGGGGAGGAGGTATCAGAC
CCCATGGAGGGCATGATGGCCATGAACAATGTTGAGTGGACCCCAGAGAACATTAAGAAGGCTGAGAAAGCCTGCAAGGAGGCCCAGCAGATGGTGATAA
TAAAGACCATTGATGAATTGGTGGAAAACACGACAGGCTGGATTCCGGAAATCATCCCAACTGCTCGGGGACCCTGCTTGGAGTCCTCTGGAGGCAAAGT
GGAGCCCCAGGACAAAACCCCTGCAGGCTTCAAGGCTGAACCAGTGGCCCTCTCAGGCTCCAGCACTGCTAGCCTGAGGCACCTCCCTCTGGCTGCCCTC
TTGTTTGGGCAGGGGGCTCTTTCTTGGCTTCTTGCCAACCTGCAGGTTGGAGTCTGA

>Leptonychotes_weddellii No=38 length=618 name="Weddel seal"
MTCALLFRLLLLTALAAPSQGKHLGPTSRLRYSRFLDPSNVIFLCWDFDLEAEIITFELQVRTTGWVGLGVTNRYTRVGSDLVVGGVLPDGNIYFSDQHL
VDEDTLEEDGSQDAELQGLTEDAIYTTMRFSRPFRSCDPHDKDITSDTIKVLATYGLDDTLKLDRDRTFIKSIFLLQIVHPDDLDVPEDTIIHDLEITDF
LVPEDDTTYACTFLPLPIVSKKHHIFKFEPKLPEHHESMVHHILVYACGHASVLPTGISDCYGADPAFSLCSQVIVGWAVGGTSYQFPDDVGISIGTPLD
PQWIRLEIHYSNFHNLPGVYDSSGIRLYYTAQLRKYDTGVLQLGFFTFPIHFIPPGAESFLSYGLCKTEKFEEMNGAPVPDIQVYGYLLHTHLAGRALQA
VQYRNGAQLRTICKDDSYDFSLQETRDLPYRAEIKLGDELLVECHYQTLDRDSLTFGGPSTINEMCLIFLFYYPRNNISSCMGYPDIIYVAHELGEEVSD
PMEGMMAMNNVEWTPENIKKAEKACKEAQQMVIIKTIDELVENTTGWIPEIIPTARGPCLESSGGKVEPQDKTPAGFKAEPVALSGSSTASLRHLPLAAL
LFGQGALSWLLANLQVGV

(1) Exon coordinates

Exon	NCBI Accession	Strand	Start	End
1	APMU01110865	+	6659	6946
2	APMU01110865	+	7831	7977
3	APMU01110865	+	8475	8636
4	APMU01110865	+	8859	8942
5	APMU01110865	+	9722	9889
6	APMU01110865	+	10088	10190
7	APMU01110865	+	10379	10545
8	APMU01110865	+	10882	10973
9	APMU01110865	+	11200	11296
10	APMU01110865	+	11437	11496
11	APMU01110865	+	12601	12731
12	APMU01110865	+	13461	13575
13	APMU01110865	+	13886	14128

39. Pacific walrus (*Odobenus rosmarus divergens*)

>Odobenus_rosmarus_divergens No=39 length=1857 name="Pacific walrus"
ATGACCTATGCCCTTCTCTTCAGACTTCTCCTACTTACGGCCCTGGCAGCCCCCTCCCAAGGCAAACACCTTGGCCCCACATCTCGCCTGCGTTATTCCA
GGTTCCTAGATCCTTCCAATGTCATTTTCCTGCGCTGGGACTTTGACCTTGAGGCTGAGATCATCACTTTTGAGCTGCAGGTCCAGACAACTGGCTGGGT
GGGCTTGGGTGTCACAAATCGCTACACCAGAGTGGGGAGTGATCTGGTTGTTGGAGGAGTCCTGCCTGATGGCAACGTCTATTTCTCGGATCAGCACCTG
GTGGATGAAGACACCCTGGAGGAGGACGGGAGCCAGGATGCTGAGCTGCAGGGGCTGACCGAAGATGCTATCTACACCACCATGCGCTTCTCCAGGCCCT
TCCGCTCCTGTGACCCTCACGACCAAGACATTACGAGTGACACCATAAAGGTGCTGGCCACCTACGGTCTGGATGACACTCTGAAGCTGGATCGGGACCG
TACTTTTATCAAGTCCATCTTCCTGCTACAAATAGTCCATCCTGACGACCTTGATGTCCCTGAGGATACCATCATCCATGACTTGGAGATCACTGATTTC
CTCATTCCAGAGGATGATACCACATATGCCTGCACCTTCCTTCCTCTCCCCATCGTTAGCAAGAAGCATCACATCTTCAAGTTTGAGCCCAAGCTGCTCG
AGCACAATGAGACAATGGTGCATCACATCCTGGTGTATGCCTGTGGCAATGCCAGTGTTCTCCCCACGGGCATCAGTGACTGCTATGGGGCTGACCCTGC
CTTCTCCCTCTGCTCACAGGTCATCGTGGGCTGGGCTGTCGGGGGCACAAGTTACCAGTTTCCAGATGATGTGGGTGTCTCTATTGGGACCCCCTTAGAC
CCCCAGTGGATACGGCTGGAGATTCATTACAGCAATTTTCACAACCTTCCTGGTGTGTATGACTCCTCAGGCATCCGATTATACTACACTGCACAGCTGC
GCAAATATGACATGGGGGTCCTCCAGCTGGGCTTCTTCACTTTCCCCATCCACTTCATACCCCCAGGCGCAGAGTCCTTCCTGTCCTATGGGCTGTGTAA
GACAGAGAAGTTTGAAGAGATGAATGGGGCCCCAGTGCCTGACATACAGGTCTATGGCTACCTGCTCCACACCCACCTGGCTGGCCGTGCTCTGCAGGCC
GTGCAATACAGAAATGGAGCACAACTCCGAACAATCTGCAAAGACGATTCCTATGACTTCAATCTGCAGGAGACTCGAGATTTACCTTATCGAGCAGAGA
TCAAACTGGGAGATGAGTTGCTGGTGGAATGTCACTACCAGACGCTGGACCGGGACTCCTTGACTTTTGGGGGTCCCAGCACCATTAATGAGATGTGCCT
CATCTTCCTCTTCTACTATCCCCGAAACAACATCTCCAGCTGCATGGGGTACCCTGACATCATCTACGTGGCCCATGAGCTTGGGGAGGAGGTATCAGAC
CCCATGGAGGGCATGATGGCCATAAACAATGTTGAGTGGACCCCAGAGAACATTAAGAAGGCTGAGAAAGCCTGCAAGGAGGCCCAGCAGATGGTGATAA
TAAAGACCATTGATGAATTGGTGGAAAATACGACAGGCTGGATTCCGGAAATCATCCCAACTGCTCGGGGACCCTGCTTGGAGTCCTCTGGAGGCAAAGT
GGAGCCCCAGGACAAAACCCCTGCAGGATTCAGGGCTGCACCAGTGGCCCTCTCAGGCTCCAGCACTGCTAGCCTGAGGCACCTCCCTCTGGTTGCCCTC
TTGTTTGGGCAGGGGGCTCTTTCTTGGCTTCTTGCCACCCTGCAGGTTGGAGTCTGA

>Odobenus_rosmarus_divergens No=39 length=618 name="Pacific walrus"
MTYALLFRLLLLTALAAPSQGKHLGPTSRLRYSRFLDPSNVIFLRWDFDLEAEIITFELQVQTTGWVGLGVTNRYTRVGSDLVVGGVLPDGNVYFSDQHL
VDEDTLEEDGSQDAELQGLTEDAIYTTMRFSRPFRSCDPHDQDITSDTIKVLATYGLDDTLKLDRDRTFIKSIFLLQIVHPDDLDVPEDTIIHDLEITDF
LIPEDDTTYACTFLPLPIVSKKHHIFKFEPKLLEHNETMVHHILVYACGNASVLPTGISDCYGADPAFSLCSQVIVGWAVGGTSYQFPDDVGVSIGTPLD
PQWIRLEIHYSNFHNLPGVYDSSGIRLYYTAQLRKYDMGVLQLGFFTFPIHFIPPGAESFLSYGLCKTEKFEEMNGAPVPDIQVYGYLLHTHLAGRALQA
VQYRNGAQLRTICKDDSYDFNLQETRDLPYRAEIKLGDELLVECHYQTLDRDSLTFGGPSTINEMCLIFLFYYPRNNISSCMGYPDIIYVAHELGEEVSD
PMEGMMAINNVEWTPENIKKAEKACKEAQQMVIIKTIDELVENTTGWIPEIIPTARGPCLESSGGKVEPQDKTPAGFRAAPVALSGSSTASLRHLPLVAL
LFGQGALSWLLATLQVGV

(1) Exon coordinates

Exon	NCBI Accession	Strand	Start	End
1	NW_004451520	+	65104	65391
2	NW_004451520	+	66265	66411
3	NW_004451520	+	66907	67068
4	NW_004451520	+	67291	67374
5	NW_004451520	+	68151	68318
6	NW_004451520	+	68517	68619
7	NW_004451520	+	68811	68977
8	NW_004451520	+	69313	69404
9	NW_004451520	+	69632	69728
10	NW_004451520	+	69869	69928
11	NW_004451520	+	71031	71161
12	NW_004451520	+	71900	72014
13	NW_004451520	+	72331	72573

40. Ferret (*Mustela putorius furo*)

>Mustela_putorius_furo No=40 length=1857 name="Ferret"
ATGACCTGTGCCCTGCTCTTCAGGCTTCTCCTACTTATGGCCCTGGTGGCCTCCTCCCAAGGCAAACGCCTTGGCCCCACATCTCGCCTGCGTTATTCCA
GGTTCCTAGACCCTTCCAATGTCATTTTCCTGCGCTGGGACTTTGACCTTGAGGCAGAGATCATCACTTTTGAGCTGCAGGTCCGGACAACTGGCTGGGT
GGGCCTGGGTGTCACAAATCGCTACACCAGAGTGGGGAGTGATCTGGTTGTTGGAGGAGTCTTGCCTGATGGCAACGTCTATTTCTCGGATCAGCACCTG
GTGGATGAAGACACCCTAGAGGAGGACGGGAGCCAGGATGCTGAGTTGCAGGGGCTGATGGAAGACGCCGTCTACACCACCATGCGCTTCTCTAGGCCCT
TCCGCTCCTGTGATCCTCATGACCAAGACATTACGAGTGACACCATAAGGGTGTTGGCCACCTATGGCCTGGATGACACTCTGAAGCTGGATCGGGACCG
TACTTTTATCAAGTCCATCTTCCTGCTACAAATAGTCCATCCTGACGACCTTGATGTCCCTGAGGATACCATCATCCATGACTTGGAGCTCACTGATTTC
CTCATCCCAGAGGATGACACCACATATGCCTGCACCTTCCTCCCTCTCCCCATCGTTAGCAAGAAGCATCACATCTTCAAGTTTGAGCCCAAGCTGCTCG
TGCACAATGAGACGATGGTGCATCACATCCTGGTGTATGCTTGTGGCAATGCCAGTGTTCTCCCCACGGGTATCAGTGACTGCTATGGAGCTGACCCTGC
CTTCTCCCTCTGCTCACAGGTCATCGTGGGCTGGGCTGTCGGGGGCACAAGTTACCAGTTTCCAGACGATGTGGGTGTCTCTATTGGCACCCCCTTAGAC
CCACAGTGGATCCGACTGGAGATTCATTATAGCAATTTTCACAACCTTCCTGGCATTTATGACTCCTCGGGCATCCGATTATACTACACCGCACAGCTGC
GCAAATATGACATGGGGGTCCTCCAGCTGGGCTTCTTCACCTTCCCCATCCACTTCATACCCCCGGGCGCGGAGTCCTTCCTGTCCTACGGGCTGTGTAA
GACAGAGAAGTTTGAAGAGATGAATGGGGCCCCAATGCCCGACATACAGGTATATGGCTACCTGCTTCACACACATCTGGCTGGCCGTGCTCTGCAGGCT
GTGCAATACAGAAACGGAGCACAACTCCGAACAATCTGCAAAGATGACTCTTATGACTTCAATCTGCAGGAGACTCGAGATTTACCTTATCGAGCAGAGA
TCAAACCGGGAGATGAGTTGCTGGTGGAATGTCACTACCAGACGCTGGACCGGGACTCCTTGACTTTTGGGGGCCCCAGCACCATTAATGAGATGTGCCT
CATCTTCCTCTTCTACTATCCCCGAAATAATATCTCCAGCTGCATGGGGTACCCTGACATCATCCACGTGGCCCATGAGCTTGGGGAGGAGGTATCAGAC
CCCATGGAGGGCATGATGGCCATGAACAATGTTGAGTGGACCCCAGAGAACATTAAGAAGGCTGAGAAAGCTTGCAAGGAGGCCCAGCAGATGGTGATAA
TAAAGACCATTGATGAATTGGTGGAAAACACGACAGGCTGGATTCCAGACATCATCCCAACGACTCGGGGACCCTGCTTGGAGTCCTCTGGAGGCAAAGT
GGAGCCTCAAGACAAAACCCCTGCAGGCTTCAGGGCTGCACCAGTGGCCCTCTCGGGTTCCAGCACTGCTACCCAGAGGCGCCTCTCTCTGGCTGCCCTC
TTGTTTGGGCAGGGGGCTCTTTCTTGGCTTCTTGCCACCCTGAAAGTCGGAGTCTGA

>Mustela_putorius_furo No=40 length=618 name="Ferret"
MTCALLFRLLLLMALVASSQGKRLGPTSRLRYSRFLDPSNVIFLRWDFDLEAEIITFELQVRTTGWVGLGVTNRYTRVGSDLVVGGVLPDGNVYFSDQHL
VDEDTLEEDGSQDAELQGLMEDAVYTTMRFSRPFRSCDPHDQDITSDTIRVLATYGLDDTLKLDRDRTFIKSIFLLQIVHPDDLDVPEDTIIHDLELTDF
LIPEDDTTYACTFLPLPIVSKKHHIFKFEPKLLVHNETMVHHILVYACGNASVLPTGISDCYGADPAFSLCSQVIVGWAVGGTSYQFPDDVGVSIGTPLD
PQWIRLEIHYSNFHNLPGIYDSSGIRLYYTAQLRKYDMGVLQLGFFTFPIHFIPPGAESFLSYGLCKTEKFEEMNGAPMPDIQVYGYLLHTHLAGRALQA
VQYRNGAQLRTICKDDSYDFNLQETRDLPYRAEIKPGDELLVECHYQTLDRDSLTFGGPSTINEMCLIFLFYYPRNNISSCMGYPDIIHVAHELGEEVSD
PMEGMMAMNNVEWTPENIKKAEKACKEAQQMVIIKTIDELVENTTGWIPDIIPTTRGPCLESSGGKVEPQDKTPAGFRAAPVALSGSSTATQRRLSLAAL
LFGQGALSWLLATLKVGV

(1) Exon coordinates

Exon	NCBI Accession	Strand	Start	End
1	AEYP01008749	+	1301	1588
2	AEYP01008749	+	2480	2626
3	AEYP01008749	+	3137	3298
4	AEYP01008749	+	3517	3600
5	AEYP01008749	+	4346	4513
6	AEYP01008749	+	4708	4810
7	AEYP01008749	+	4993	5159
8	AEYP01008749	+	5481	5572
9	AEYP01008749	+	5800	5896
10	AEYP01008749	+	6038	6097
11	AEYP01008749	+	7200	7330
12	AEYP01008749	+	7981	8095
13	AEYP01008749	+	8386	8628

41. Horse (*Equus caballus*)

```
>Equus_caballus No=41 length=1857 name="Horse"
ATGGCCTGTGCCCTTCTCTTCAGGCTTCTCCTACTTATGGCCCTGGCAGCCCCCTCCCAAGGCAATGGCCCTGGCCCCACATCTCGCCTGCGTTATTCCA
GGTTTCTGGATCCTTCCAATGTCATTTTCCTGCGCTGGGACTTTGACCTTGAGGCTGAGATCATCACTTTTGAGCTCCAGGTCCGGACAGCTGGCTGGGT
AGGCTTGGGTGTCACAAATCGCTATACCAACGTGGGAAGTGATCTGGTTGTTGGAGGAGTCTTGCCTGATGGCAACATCTATTTTTCGGATCAGCACCTG
GTAGATGAAGACACCCTGGCGGAAGATGGGAGCCAGGATGCTGAGCTACAGGGGCTAACGGAAGATGCTGTCTATACCACCATGCGCTTCTCCAGGCCTT
TCCGCTCTTGCGACCCTCATGACCAAGACATTACGAGTGACACTATGAGGGTGCTGGCCACCTACGGCCTGGATGACACTCTGAAGCTGGATCGGGAGCG
TACTTTTGTCAAGTCCATCTTCCTGCTACAAATATTCCACCCTGATGATCTTGATGTCCCTGAAGACACCATCATCCATGACTTGGAGATCACTGATTTC
CTCATTCCAGAGGATGACACCACCTATGCCTGCACCTTCCTCCCTCTCCCCATCGTTAGCAAGAAACATCATATCTACAAGTTTGAGCCCAAGTTGGTCC
ACCACAATGAGACGATGGTGCATCACATCCTGGTGTACGCCTGTGGCAATGCCAGCACGCTCCCCACGGGCATCAGCGACTGCTATGGGGCTGACCCTGC
CTTCTCCCTCTGCTCCCAGGTCATCATGGGCTGGGCTGTCGGGGGCACAAGTTACCAGTTTCCAGATGATGTCGGCATCTCTCTTGGGACACCTTTGGAC
CCCCAATGGATCCGACTGGAGATACATTACAGCAATTTTCACAACCTTCCTGGTGTGTACGATTCCTCGGGGATTCGAATGTACTATACTGCCACGCTGC
GCAAATACGACATGGGAGTCCTCCAGCTGGGCTTCTTCACCTTCCCCATCCACTTCATACCCCCCGGTGCTGAGTCTTTCATGTCCTATGGGCTGTGTAA
GACAGAGAAGTTTGAAGAGATGAACGGGGCCCCCGTGCCTGACATACAGGTGTTTGGCTACCTGCTCCACACCCACTTGGCTGGCCGGGCTCTGCAGGCT
GTGCAATATAGAAATGGAACACAATTCCGAACAATCTGTAAAGATGACTCCTATGACTTCAATCTACAGGAGACTCGAGATTTACCTCATCTAGTGGAGA
TTAAGCCGGGAGATGAGTTGTTGGTTGAGTGTCACTACCAGACGCTGGACCGCGACTCCTTGACTTTTGGGGGTCCCAGCACCGTTAATGAGATGTGTCT
CGTCTTCCTCTTCTACTATCCCCGAAACAACATCTCCAGTTGCATGGGGTACCCTGACATCATCTACGTGGCCCATGAAATGGGAGAGGAGGCATCAGAT
CCCATGGAGGGCATGATGGCCATGAACAATGTTGAGTGGACCCCAGAGAACATTAAGAAGGCTGAGAAAGCCTGCAAGGAGGCCCAGCAGATGGTGATAA
TAAAGACCATTGATGAGATAGTGGAAAACACAACAGGCTGGATTTCAGAAATCATCCCTACTCCACGGGGGCCCTGCCTGGAGTCCTCGGGAGGCAAAGT
GGCGCCCCAGGACAAAATCCCTGCAGGCTTCAGGGCTGCACCAATGGCCCTCTCAGGTTCTGAGACTGCTACCCCAAGGCACCTCCCCCTGGCTGCCCTA
TTCTTTGGGCAGGGGGCCCTGTCTTGGCTCCTTGCCACCCTGCAGGTTGGAGTCTGA

>Equus_caballus No=41 length=618 name="Horse"
MACALLFRLLLLMALAAPSQGNGPGPTSRLRYSRFLDPSNVIFLRWDFDLEAEIITFELQVRTAGWVGLGVTNRYTNVGSDLVVGGVLPDGNIYFSDQHL
VDEDTLAEDGSQDAELQGLTEDAVYTTMRFSRPFRSCDPHDQDITSDTMRVLATYGLDDTLKLDRERTFVKSIFLLQIFHPDDLDVPEDTIIHDLEITDF
LIPEDDTTYACTFLPLPIVSKKHHIYKFEPKLVHHNETMVHHILVYACGNASTLPTGISDCYGADPAFSLCSQVIMGWAVGGTSYQFPDDVGISLGTPLD
PQWIRLEIHYSNFHNLPGVYDSSGIRMYYTATLRKYDMGVLQLGFFTFPIHFIPPGAESFMSYGLCKTEKFEEMNGAPVPDIQVFGYLLHTHLAGRALQA
VQYRNGTQFRTICKDDSYDFNLQETRDLPHLVEIKPGDELLVECHYQTLDRDSLTFGGPSTVNEMCLVFLFYYPRNNISSCMGYPDIIYVAHEMGEEASD
PMEGMMAMNNVEWTPENIKKAEKACKEAQQMVIIKTIDEIVENTTGWISEIIPTPRGPCLESSGGKVAPQDKIPAGFRAAPMALSGSETATPRHLPLAAL
FFGQGALSWLLATLQVGV
```

(1) Exon coordinates

Exon	UCSC Chromosome	Strand	Start	End
1	equCab2 chr4	-	94975602	94975889
2	equCab2 chr4	-	94974540	94974686
3	equCab2 chr4	-	94973890	94974051
4	equCab2 chr4	-	94973560	94973643
5	equCab2 chr4	-	94972568	94972735
6	equCab2 chr4	-	94972276	94972378
7	equCab2 chr4	-	94971935	94972101
8	equCab2 chr4	-	94971516	94971607
9	equCab2 chr4	-	94971133	94971229
10	equCab2 chr4	-	94970936	94970995
11	equCab2 chr4	-	94969750	94969880
12	equCab2 chr4	-	94969084	94969198
13	equCab2 chr4	-	94968528	94968770

42. Southern white rhinoceros (*Ceratotherium simum simum*)

```
>Ceratotherium_simum_simum No=42 length=1857 name="Southern white rhinoceros"
ATGGCCTGTGCCCTTCTCTTCAGGCTTCTCCTACTTATGGCCTTGGGAGCACCCTCCCAAGGCAACGGCCTCAGCCCCACATCTCACCTTCGTTATTCCA
GGTTCCTGGATCCTTCCAATATCATTTTCCTGCGCTGGGACTTTGACCTTGAGGCTGAGATCATCACTTTTGAGCTCCAGGTCCGGACGGCTGGCTGGGT
AGGCTTGGGTGTCACAAATCGCTACACCAACGTGGGGAGCGATCTGGTTGTTGGAGGAGTCCTGCCTGACGGCAATGTCTATTTCTCGGACCGGCACCTG
GTAGATGAAGACACACTGGAGGAGGACGGGAGCCAGGATGCTGAGCTGCGGGGGCTAACGGAAGACGCTGTCTACACAACAATGCGCTTCTCCAGGCCCT
TCCGCTCCTGCGACCCTCATGACCAAGACATTACGAGTGACACCGTGAGAGTGCTGGCTGCCTACGGCCTAGATGACACTCTGAAGCTGGATCGGGAGCG
TACTTTTGTCAAGTCCATCTTCCTGCTACAAATAGTCCACCCTGATGATCTTGATGTCCCTGAAGACACCATCATCCATGATTTGGAAATCACTGATTTC
CTCATTCCAGAGGATGATACCACATATGCCTGCACCTTCCTCCCTCTCCCCATCGTTAGCAAGAAACATCACATCTACAAGTTTGAGCCCAAGATGGTCC
AGCACAATGAGACGATGGTGCATCACATCCTGGTGTACGCCTGTGGCAATGCCAGTGCACTCCCCACCGGCATCAGCGACTGCTATGGGGCTGACCCTGC
CTTCTCCCTCTGCTCGCAGGTCATCATGGGCTGGGCTGTCGGGGGCACAAGTTACCAGTTTCCAGATGACGTGGGCATCTCTCTTGGGACGCCCTTGGAC
CCCCAGTGGATCCGACTAGAGATTCATTACAGCAATTTTCATAACCTTCCCGGTGTGTATGACTCCTCAGGGATTCGAGTGTACTACACTGCAGAGCTGC
GCAAATACGACATGGGAGTCCTCCAGCTGGGCTTCTTCACTTTCCCCATCCACTTCATACCCCCGGGTGCTGAATCCTTCATGTCCTATGGGCTGTGTAA
GACTGAGAAGTTTGAAGAGATGAATAGGGCCCCAGTGCCTGACATACAGGTGTATGGCTACCTGCTCCACACGCACTTGGCTGGCCGGGCTCTGCAGGCC
GTGCAATACAGAAATGGAACACAACTCCGAACAATCTGTAAAGATGACTCCTACGACTTCAATCTACAGGAGACTCGAGATTTACCTCATCGAGTGGAGA
TTAAGCCAGGTGATGAGTTGCTGGTTGAGTGTCACTACCAGACGCTGGACCGCGACTCCTTGACTTTTGGGGGTCCCAGCACCATTAATGAGATGTGCCT
CATCTTCCTCTTCTACTATCCCCGAAACAACATCTCCAGCTGCATGGGGTTCCCTGACATCATCTACGTGGCCCAGGAGCTGGGGGAGGAGGCATCAGAT
TCCATGGAGGCCATGATGGCCATGAACAATGTTGAGTGGACCCCAGAGAGCATTAAGAAGGCTGAGAAAGCCTGCAAGGAGGCCCAGCAGACGGTGATAA
TAAAGACCATTGACGAGATAGTGGAAAACACAACAGGCTGGATTTCGGAAATCATCCGTGCTCCTCGGGGGCCCTGCTTGGAGTCCTCAGGAGGCAAAGT
GGAGCCCCAGGACAAAATCCCTGAAGGCTTCAGGGCTGCACCAATGGCCCTCTCAGGTTCTAACACTGCTACCTGGAGGCGCCTCCCCCTGGCTGCCCTC
CTGTTTGGGCAGGGAGTCCTGTTTTTGCTCCTTACCACTCTGCAGGCTGGAGTCTGA
```

```
>Ceratotherium_simum_simum No=42 length=618 name="Southern white rhinoceros"
MACALLFRLLLLMALGAPSQGNGLSPTSHLRYSRFLDPSNIIFLRWDFDLEAEIITFELQVRTAGWVGLGVTNRYTNVGSDLVVGGVLPDGNVYFSDRHL
VDEDTLEEDGSQDAELRGLTEDAVYTTMRFSRPFRSCDPHDQDITSDTVRVLAAYGLDDTLKLDRERTFVKSIFLLQIVHPDDLDVPEDTIIHDLEITDF
LIPEDDTTYACTFLPLPIVSKKHHIYKFEPKMVQHNETMVHHILVYACGNASALPTGISDCYGADPAFSLCSQVIMGWAVGGTSYQFPDDVGISLGTPLD
PQWIRLEIHYSNFHNLPGVYDSSGIRVYYTAELRKYDMGVLQLGFFTFPIHFIPPGAESFMSYGLCKTEKFEEMNRAPVPDIQVYGYLLHTHLAGRALQA
VQYRNGTQLRTICKDDSYDFNLQETRDLPHRVEIKPGDELLVECHYQTLDRDSLTFGGPSTINEMCLIFLFYYPRNNISSCMGFPDIIYVAQELGEEASD
SMEAMMAMNNVEWTPESIKKAEKACKEAQQTVIIKTIDEIVENTTGWISEIIRAPRGPCLESSGGKVEPQDKIPEGFRAAPMALSGSNTATWRRLPLAAL
LFGQGVLFLLLTTLQAGV
```

(1) Exon coordinates

Exon	NCBI Accession	Strand	Start	End
1	AKZM01025525	+	71489	71776
2	AKZM01025525	+	72703	72849
3	AKZM01025525	+	73375	73536
4	AKZM01025525	+	73783	73866
5	AKZM01025525	+	74684	74851
6	AKZM01025525	+	75041	75143
7	AKZM01025525	+	75319	75485
8	AKZM01025525	+	75820	75911
9	AKZM01025525	+	76197	76293
10	AKZM01025525	+	76431	76490
11	AKZM01025525	+	77585	77715
12	AKZM01025525	+	78293	78407
13	AKZM01025525	+	78716	78958

43. Wild Bactrian camel (*Camelus ferus*)

>Camelus_ferus No=43 length=1857 name="Wild Bactrian camel"
ATGGCCCGTGCCCTTCTCTTCCAGCTTCTCCTGCTTACAGCCCTGGCAGCCCCCTCCCAAGGCAACCACCTTGGCCCCACCTCTCGGCTGCGTTATTCCA
GGTTCCTAGATCCTTCTAATGTCATTTTCCTGCGCTGGGACTTTGACTATAAGGCTGAGATCATTACTTTTGAGCTCCAGGTCCGGACAGCTGGCTGGGT
GGGCCTGGGTGTCACAAATCGCTACACCAGAGTGGGAAGCGATCTGGTTGTTGGCGGAGTCCTGCCCGACGGCAATGTCTATTTCTCGGACCAGCACCTG
GTGGATGAAGACACCCTGGAGAAGGACGGGAGCCAGGATGCTGAGCTGCAGCGGCTCACGGAAGATGCTGTCTACACCACCATGCGCTCCTCCAGGCCCT
TCCGCTCTTGTGACCCTTATGACCAAGACATCACGAGCGACACCGTGAGGGTGCTGGCCGCCTACGGCCCGGATGACACTCTGAAGCTGCATCGGGAGCG
TACTTTTGTGAAGTCTATCTTCCTGCTGCAAATGATCCAGCCTGACGACCTCCATGTCCCCGAGGACACCATCATCCATGACTTGGAGCTCACTGATTTC
CTCATTCCAGAGGATGACACCACGTACGCCTGCACCTTCCTCCCTCTCCCCATCGTCAGCAAGAAGCATCACATCTACAAGTTTGAGCCCAAGCTGGTGG
ACCGCAGCGAGACCATGGTGCATCACATCCTGGTGTATGCCTGCGGCAACGCCAGCACTCTCCCCACGGGCATCAGCGACTGCTACGGCGCTGACCCCGC
CTTCTCCCTCTGCTCACAGGTCATCGTGGGCTGGGCTGTCGGGGGCACAAGTTACCAGTTCCCAGACGATGTGGGCATCTCCATTGGGACGCCTTTGGAC
CCCCAGTGGATCCGACTGGAGATTCACTACAGCAATTTTCACAACCTGCCTGGTGTGTACGACTCCTCGGGGATTCGAATGTACTACACGTCGCAGCTGC
GCAAACACGACATGGGCGTCCTGCAGCTGGGCTTCTTCACCTTCCCCATCCACTTCATCCCCCCGCACGCCGAGTCCTTCATGTCCTACGGGCTGTGTAA
GACGGAGAAGTTTGAAGAGATGAATGGGGCCCCGGTGCCTGACATACAGGTGTACGGTTACCTGCTCCACACCCACCTGGCGGGCCGCGCTCTGCAGGCC
GTGCAATACAGAAATGGAACCCAACTTCGAACAATCTGTAAAGATGATTCCTATGACTTCAATCTGCAGGAGACTCGAGATCTACCTTATCGAGTAGAGA
TCAAGCCGGGAGATGAATTGCTGGTAGAGTGTCACTACCAGACGCTGGACCGCGACTTCTTGACGTTTGGGGGTCCCAGCACCATTAATGAGATGTGCCT
CATCTTCCTCTTCTACTATCCGAGAAACAACATCTCCAGCTGCATGGGGTACCCTGACATCATCTTCGTGGCCCATGAGATGGGGGAAGAGGCATCAGAT
TCCATGGAAGGCATGATGGCCATGAACAATGTTGAATGGACCCCAGAGAACATTAAGAAGGCTGAGAAGGCCTGCAAGGAGGCCCAGCAGACAGTGATAA
TAAAGACCATTGATGAGATAGTGGAAAACACAACAGGCTGGGTTCCGGAAATCACCCCTACTCCCCGGGGCCCTTGCTCAGAGTCCTCTGGAGGCAAAGT
GGAGCCCCAGGACAAAGCCCCTGCAGGCTTCAGGACCGCACCAGTGGCCCTCTCAGGCTCCAGCACTGCTACCCCAAGGCGCCTCCCCCTGGCTGCTCTC
TTGCTTGGGCAGGGGGCTGTCTCTTGGCTCCTTGCCACCCTGCAGTCTAGAGTCTGA

>Camelus_ferus No=43 length=618 name="Wild Bactrian camel"
MARALLFQLLLLTALAAPSQGNHLGPTSRLRYSRFLDPSNVIFLRWDFDYKAEIITFELQVRTAGWVGLGVTNRYTRVGSDLVVGGVLPDGNVYFSDQHL
VDEDTLEKDGSQDAELQRLTEDAVYTTMRSSRPFRSCDPYDQDITSDTVRVLAAYGPDDTLKLHRERTFVKSIFLLQMIQPDDLHVPEDTIIHDLELTDF
LIPEDDTTYACTFLPLPIVSKKHHIYKFEPKLVDRSETMVHHILVYACGNASTLPTGISDCYGADPAFSLCSQVIVGWAVGGTSYQFPDDVGISIGTPLD
PQWIRLEIHYSNFHNLPGVYDSSGIRMYYTSQLRKHDMGVLQLGFFTFPIHFIPPHAESFMSYGLCKTEKFEEMNGAPVPDIQVYGYLLHTHLAGRALQA
VQYRNGTQLRTICKDDSYDFNLQETRDLPYRVEIKPGDELLVECHYQTLDRDFLTFGGPSTINEMCLIFLFYYPRNNISSCMGYPDIIFVAHEMGEEASD
SMEGMMAMNNVEWTPENIKKAEKACKEAQQTVIIKTIDEIVENTTGWVPEITPTPRGPCSESSGGKVEPQDKAPAGFRTAPVALSGSSTATPRRLPLAAL
LLGQGAVSWLLATLQSRV

(1) Exon coordinates

Exon	NCBI Accession	Strand	Start	End
1	AGVR01031487	-	70241	70528
2	AGVR01031487	-	69261	69407
3	AGVR01031487	-	68591	68752
4	AGVR01031487	-	68269	68352
5	AGVR01031487	-	67278	67445
6	AGVR01031487	-	66993	67095
7	AGVR01031487	-	66637	66803
8	AGVR01031487	-	66228	66319
9	AGVR01031487	-	65844	65940
10	AGVR01031487	-	65646	65705
11	AGVR01031487	-	65267	65397
12	AGVR01031487	-	64603	64717
13	AGVR01031487	-	64070	64312

44. Pig (*Sus scrofa*)

```
>Sus_scrofa No=44 length=1857 name="Pig"
ATGGCCCACGCCCTTCTCCTCCAGCTTCTTCTGCTTTCAGCCCTGGCAGCCCCCTCCCAAGGCAACCGCCTCGGCCCCACCTCGAGTCTGCGTTATTCTA
GGTTCCTAGATCCTTCTAATGTCATTTTCCTGCGCTGGGACTTTGACCTGGACGCTGAGATCATCGCTTTTGAGCTCCGGGTCCGGACAGCTGGCTGGGT
GGGCTTGGGCATCACCAATCGCTACACCAGAGCAGGCAGCGATCTGGTTGTCGGAGGGGTCTCGCCTGACGGTCATGTCTATTTCTCGGATCAGCATGTG
GTGGATGAAGACACCCTGGAAGAGGACGGGAGCCAGGACGCGGAGCTGCAGGGGCTGACGGAAGACGCCGTCTACACCACCATGCGCTTCTCCAGGCCCT
TCCGCTCCTGCGACCCCCAGGACCAAGACATCACGAGTGACACAGTGAGGGTGCTGGCTGCCTATGGCCCTGATGACACCCTGAAGCTGGATCGGGAGCG
GATGTTCGTGAAGTCCATCTTCCTGCTCCAGATCATCCACCCCGACGATCTCGACGCCCCCGAGGACGCCATCATCCACGACCTGGAGATCACGGATTTC
CTCATTCCAGAGGATGACACCACCTATGCCTGCACCTTCCTCCCTCTCCCCATCGTTAGCAAGAAGCATCACATCTACAAGTTTGAGCCCAAACTGGAAC
CTCACAACGAGACCATGGTGCACCACATCCTGGTATACGCCTGCGGCAACGCCAGCGCCCTCCCCACGGGCATAAGCGACTGCTACGGGGCCGACCCTGC
CTTCTCCCTCTGCTCCCAGGTCATCGTGGGCTGGGCTGTCGGGGGCACAAGTTACCAGTTTCCGGATGACGTGGGCATCTCTATTGGGACGCCCCTGGAC
CCCCAGTGGATCCGACTGGAGATTCATTATAGCAATTTTCACAATCTTCCCGGGGTGTACGACTCCTCGGGAATTCGAGTCTACTACACGGCAAAGCTGC
GCAAATACGACATGGGTGTCCTGCAGCTGGGCTTCTTCACGTTCCCCATCCACTTCATCCCCCCGGGCGCCGAGTCCTTCATGTCCTACGGGCTGTGTAA
GACGGACAAGTTTGAGGAGATGAATGGGGCCCCGGTGCTTGACATCCAGGTGTATGGCTACCTGCTCCACACCCACTTGGCTGGCCGGGCTCTGCAAGCC
GTGCAATACAGAAATGGAACACAACTCCAAACAATCTGTAAAGATGACTCCTACGATTTCAATCTGCAGGAGACTCGAGATTTACCTTATCGCATGGTCA
TCAAGCCGGGAGATGAACTGCTGGTAGAGTGTCACTACCAGACGCTGGACCGAGACTTCTTGACCTTTGGGGGGCCCAGCACCATTAATGAGATGTGCCT
CATCTTCCTCTTCTACTATCCCCGAAACAACATCTCCAGCTGCATGGGCTACCCCGACATCATCTATGTGGCCCATGAGCTGGGGGAGGAGGCTTCAGAT
TCCATGGAGGGCATGATGGCCATGAACAATGTGGAGTGGACCCCGGAAAGCATTAAGAAGGCAGAGAAAGCCTGCAAGGAGGCCCAGCAGACGGTGATAA
TAAAGACCATTGACGAGCTAGTGGAAAACACCACAGGCTGGATTCCGGAAATTGTCCCTACTCCCCGAGGCCCCTGCTTGGAGTCCTCCGGAGGCAAAGT
GGAGCCCGAGGACCAAACCCCCGCTGGCTTCAGGGCTGCACCAATGGCCCTCTCCGTCTCCAGCGCTGCTACTCCAAGTCGCCTCCCCCTGGCTGCCTTC
TTACTTGGGCAGGGGACCCTCTCTTGGCTCCTTGCCACCCTGCAGGCTGGAGTCTGA

>Sus_scrofa No=44 length=618 name="Pig"
MAHALLLQLLLLSALAAPSQGNRLGPTSSLRYSRFLDPSNVIFLRWDFDLDAEIIAFELRVRTAGWVGLGITNRYTRAGSDLVVGGVSPDGHVYFSDQHV
VDEDTLEEDGSQDAELQGLTEDAVYTTMRFSRPFRSCDPQDQDITSDTVRVLAAYGPDDTLKLDRERMFVKSIFLLQIIHPDDLDAPEDAIIHDLEITDF
LIPEDDTTYACTFLPLPIVSKKHHIYKFEPKLEPHNETMVHHILVYACGNASALPTGISDCYGADPAFSLCSQVIVGWAVGGTSYQFPDDVGISIGTPLD
PQWIRLEIHYSNFHNLPGVYDSSGIRVYYTAKLRKYDMGVLQLGFFTFPIHFIPPGAESFMSYGLCKTDKFEEMNGAPVLDIQVYGYLLHTHLAGRALQA
VQYRNGTQLQTICKDDSYDFNLQETRDLPYRMVIKPGDELLVECHYQTLDRDFLTFGGPSTINEMCLIFLFYYPRNNISSCMGYPDIIYVAHELGEEASD
SMEGMMAMNNVEWTPESIKKAEKACKEAQQTVIIKTIDELVENTTGWIPEIVPTPRGPCLESSGGKVEPEDQTPAGFRAAPMALSVSSAATPSRLPLAAF
LLGQGTLSWLLATLQAGV
```

(1) Exon coordinates

Exon	UCSC Chromosome	Strand	Start	End
1	susScr3 chr18	+	8009872	8010159
2	susScr3 chr18	+	8011154	8011300
3	susScr3 chr18	+	8011800	8011961
4	susScr3 chr18	+	8012196	8012279
5	susScr3 chr18	+	8013525	8013692
6	susScr3 chr18	+	8013883	8013985
7	susScr3 chr18	+	8014176	8014342
8	susScr3 chr18	+	8014928	8015019
9	susScr3 chr18	+	8015301	8015397
10	susScr3 chr18	+	8015537	8015596
11	susScr3 chr18	+	8016912	8017042
12	susScr3 chr18	+	8018257	8018371
13	susScr3 chr18	+	8018641	8018883

45. Goat (*Capra hircus*)

>Capra_hircus No=45 length=1854 name="Goat"
ATGGCCTGTCCCCTTCTCTCCTGGCTTCTATTTACGGCACTTGCAACTCCCTCCCAAGGCGATCGCCTTGGCCCCACGCCGCATCTGCGTTATTCCAGGT
TCCTAGACGCTTCTAACGCTGTTTTCCTGCGCTGGGACTTTGACTTTGAGGCTGAAATCATCACTTTCGAGCTCCAGGTCCGGACAGCTGGCTGGGTGGG
CTTGGGTGTCACAAATCGCTACAGCAGAGCAGGAGGTGACCTGGTTGTCGGAGGCGTCTCGCCTGACGGCAATGTCTATTTCTCGGACCAGCACCTGGTG
GATGAAGACACGCTGGAGGAGGATGGGAGCCAGGATGCAGAGCTGCAGGGGCTGACGGAAGATGCCGTCTACACCACCATGCGCTTCTCCAGGCCCTTCC
GCTCCTGCGACCCTCACGACCAAGACATCACGAGCGACACTGTGAGGGTGCTGGCCGCCTACGGCCTGGATGACACCCCAAAGATAGATCGGGAGCGTAC
TTTTGTGAAGTCCATCTTCCTGCTCCAAATCGTCCACCCTGATGATCTCGATGCCCCTGAGGACACCATCATCCATGACTTGGAGATCACTGATTTCCTC
ATCCCAGAGGATGACACCACGTACGCCTGCACCTTCCTTCCTCTCCCCATCGTTAGCAAGAAGCACCACATCTACAAGTTCGAGCCCAAGCTGGTCCAGC
ACAACGAGACAATTGTGCACCACATCCTGGTGTACGCCTGCGGCAATGCCAGCGCTCTCCCCACCGGCATCAGCGACTGCTACGGGGCCGACCCCGCCTT
CTCCCTCTGCTCGCAGGTCATCGTGGGCTGGGCTGTCGGGGGCGCAAGTTACCAGTTTCCAGACGACGTGGGCATTTCTATTGGGACACCCTTGGACCCC
CAGTGGATCCGCCTGGAGATTCATTACAGCAATTTTCACAATCTGCCTGGTCTGTATGACTCATCGGGGATCCGAGTATACTACACCGCGCACCTGCGCA
AGTTTGACATGGGCGTCCTGCAGCTGGGTGTCTTCACTTTCCCCATCCACTTCATCCCCCCCAGCGCCGAGTCCTTCAGGTCCTATGGACTGTGTAAGAC
AGAGAAGTTTGAAGAGATGAACCGGGCTCCAGTGCCCGACATCCAAGTCTTCGGCTACCTGCTCCACACCCACTTGGCCGGCCGCGCTCTGCAGGCGGTG
CAGTACAGAAATGGAACTCAACTCCGCGTAATCTGTAAAGATGATGCCTACGACTTCAATCTGCAGGAGACTCGAGATTTACCTTATCGAGTGGTGATCA
AGCCGGGGGATGAATTGCTGGTAGAGTGTCGCTACCAGACGCTGGACCGCGACTCCTTGACATTCGGGGGTCCCAGCACCATTAACGAGATGTGCCTCGT
CTTTCTCTTCTACTATCCCCGAAACAACGTCTCCAGCTGCCAGGGGTACCCTGACATCATCTATGTGGCCCACGAGCTGGGGGAGGAGGTATCAGACTCC
ATGGAGGGCATGATGGCCATGAGCAATGTGGAGTGGACCCCAGAGAGCATTAAGAAGGCTGAGAATGCCTGCAAGGAGGCCCAGCAGACAGTGATAATAA
AGACCATTGATGAGCTAGTGGAAAACACAACAGGCTGGATTCAGGACATCAACCCTACTCCCCGGGGCCCTTGTTTGGAGTCCTCTGGAGGCAAAGTGGA
GCCCCAGGACAAAACCCCTGCAGGCTTCAGGGCAGCCCCCAGGGTCCTCTCGGGGTCCAGCAGTGCCACCCTGAGGTGCCTCCCCCTGGCCACCCTCTTG
TTTGGGCAGGGGGCCCTGTCTTGGCTCCTTGCCACCCTGCAGGCTGGAATCTGA

>Capra_hircus No=45 length=617 name="Goat"
MACPLLSWLLFTALATPSQGDRLGPTPHLRYSRFLDASNAVFLRWDFDFEAEIITFELQVRTAGWVGLGVTNRYSRAGGDLVVGGVSPDGNVYFSDQHLV
DEDTLEEDGSQDAELQGLTEDAVYTTMRFSRPFRSCDPHDQDITSDTVRVLAAYGLDDTPKIDRERTFVKSIFLLQIVHPDDLDAPEDTIIHDLEITDFL
IPEDDTTYACTFLPLPIVSKKHHIYKFEPKLVQHNETIVHHILVYACGNASALPTGISDCYGADPAFSLCSQVIVGWAVGGASYQFPDDVGISIGTPLDP
QWIRLEIHYSNFHNLPGLYDSSGIRVYYTAHLRKFDMGVLQLGVFTFPIHFIPPSAESFRSYGLCKTEKFEEMNRAPVPDIQVFGYLLHTHLAGRALQAV
QYRNGTQLRVICKDDAYDFNLQETRDLPYRVVIKPGDELLVECRYQTLDRDSLTFGGPSTINEMCLVFLFYYPRNNVSSCQGYPDIIYVAHELGEEVSDS
MEGMMAMSNVEWTPESIKKAENACKEAQQTVIIKTIDELVENTTGWIQDINPTPRGPCLESSGGKVEPQDKTPAGFRAAPRVLSGSSSATLRCLPLATLL
FGQGALSWLLATLQAGI

(1) Exon coordinates

Exon	NCBI Accession	Strand	Start	End
1	AJPT01248665	-	16287	16571
2	AJPT01248665	-	15261	15407
3	AJPT01248665	-	13458	13619
4	AJPT01248665	-	13130	13213
5	AJPT01248665	-	12130	12297
6	AJPT01248665	-	11827	11929
7	AJPT01248665	-	11481	11647
8	AJPT01248665	-	11080	11171
9	AJPT01248665	-	10728	10824
10	AJPT01248665	-	10532	10591
11	AJPT01248665	-	10159	10289
12	AJPT01248665	-	9152	9266
13	AJPT01248665	-	8618	8860

46. Sheep (*Ovis aries*)

```
>Ovis_aries No=46 length=1854 name="Sheep"
ATGGCCTGTCCCCTTCTCTTCTGGCTTCTATTTACGGTGCTTGCAACTCCCTCCCAAGGTGATCACCTTGGCCCCGCGCCGCGTCTGCGTTATTCCAGGT
TCCTAGACGCTTCTAACGCTGTTTTCCTGCGCTGGGACTTTGACTTTGAGGCTGAAATCATCACTTTCGAGCTCCAGGTCCGGACAGCTGGCTGGGTGGG
CTTGGGTGTCACCAATCGCTACAGCAGAGCAGGAGGTGACCTGGTTGTCGGAGGCGTCTCACCTGACGGCAATGTTTATTTCTCGGACCAGCACCTGGTG
GATGAAGACACGCTGGAGGAGGATGGGAGCCAGGATGCAGAGCTGCAGGGGCTGACGGAAGATGCCGTCTACACCACCATGCGCTTCTCCAGGCCCTTCC
GCTCCTGCGACCCTCACGACCAAGACATCACGAGCGACACTGTGAGGGTGCTGGCCGCCTACGGCCTGGATGACACCCCAAAGATAGATCGGGAGCGTAC
TTTTGTGAAGTCCATCTTCCTGCTCCAAATTGTCCACCCTGATGATCTCGATGCCCCCGAGGACACCATCATCCATGACTTGGAGATCACTGATTTCCTC
ATCCCAGAGGATGACACCACGTACGCCTGCACCTTCCTCCCTCTCCCCATCGTTAGCAAGAAGCACCACATCTACAAGTTCGAGCCCAAGCTGGTCCAGC
ACAACGAGACAATTGTGCACCACATCCTGGTGTACGCCTGTGGCAACGCCAGCACTCTCCCTACCGGCATCAGCGACTGCTACGGGGCCGACCCCGCCTT
CTCCCTCTGCTCGCAGGTCATCGTGGGCTGGGCTGTCGGGGGCGCAAGTTACCAGTTTCCAGACGACGTGGGCATTTCTATTGGGACGCCGTTGGACCCC
CAGTGGATCCGCCTGGAGATTCATTACAGCAATTTTCACAATCTGCCTGGTCTGTACGACTCATCGGGGATCCGAGTATACTACACCGCGCACCTGCGCA
AGTTTGACATGGGCATCCTGCAGCTGGGCGTCTTCACTTTCCCCATCCACTTCATCCCCCCCAGCGCCGAGTCCTTCAGGTCCTATGGACTGTGTAAGAC
GGAGAAGTTTGAAGAGATGAACCGGGCTCCAGTGCCCGACATCCAAGTCTTCGGCTACCTGCTCCACACCCACTTGGCCGGCCGTGCTCTGCAGGCGGTG
CAGTACAGAAATGGAACTCAACTCCGCGTAATCTGTAAAGATGATGCCTACGACTTCAATCTGCAGGAGACTCGAGATTTACCTTATCGAGTGGTGATCA
AGCCGGGGGATGAATTGCTGGTAGAGTGTCGCTACCAGACACTGGACCGCGACTCCTTGACATTCGGGGGTCCCAGCACCATTAACGAGATGTGCCTCGT
CTTTCTCTTCTACTATCCCCGAAACAACGTCTCCAGCTGCCAGGGGTACCCTGACATCATCTATGTGGCCCACGAGCTGGGGGAGGAGGTATCAGACTCC
ATGGAGGGCATGATGGCCATGAGCAATGTGGAGTGGACCCCAGAGAGCATTAAGAAGGCTGAGAATGCCTGCAAGGAGGGCCAGCAGACAGTGATAATAA
AGACCATTGATGAGCTAGTGGAAAACACAACAGGCTGGATTCGGGACATCAACCCTACTCCCCGGGGCCCTTGTTTGGAGTCCTCTGGAGGCAAAGTGGA
GTCCCAGGACAAAACCCCTGCAGGCTTCAGGGCAGCCCCCGGGGTCCTCTCGGGGTCCAGCAGTGCCACCCTGAGGTGCCTCCCCCTGGCCACCCTCTTG
TTTGGGCAGGGGGCCCTGTCTTGGCTCCTTGCCACCCTGCAGGCTGGAATCTGA

>Ovis_aries No=46 length=617 name="Sheep"
MACPLLFWLLFTVLATPSQGDHLGPAPRLRYSRFLDASNAVFLRWDFDFEAEIITFELQVRTAGWVGLGVTNRYSRAGGDLVVGGVSPDGNVYFSDQHLV
DEDTLEEDGSQDAELQGLTEDAVYTTMRFSRPFRSCDPHDQDITSDTVRVLAAYGLDDTPKIDRERTFVKSIFLLQIVHPDDLDAPEDTIIHDLEITDFL
IPEDDTTYACTFLPLPIVSKKHHIYKFEPKLVQHNETIVHHILVYACGNASTLPTGISDCYGADPAFSLCSQVIVGWAVGGASYQFPDDVGISIGTPLDP
QWIRLEIHYSNFHNLPGLYDSSGIRVYYTAHLRKFDMGILQLGVFTFPIHFIPPSAESFRSYGLCKTEKFEEMNRAPVPDIQVFGYLLHTHLAGRALQAV
QYRNGTQLRVICKDDAYDFNLQETRDLPYRVVIKPGDELLVECRYQTLDRDSLTFGGPSTINEMCLVFLFYYPRNNVSSCQGYPDIIYVAHELGEEVSDS
MEGMMAMSNVEWTPESIKKAENACKEGQQTVIIKTIDELVENTTGWIRDINPTPRGPCLESSGGKVESQDKTPAGFRAAPGVLSGSSSATLRCLPLATLL
FGQGALSWLLATLQAGI
```

(1) Exon coordinates

Exon	NCBI Accession	Strand	Start	End
1	AMGL01089435	-	47385	47669
2	AMGL01089435	-	46359	46505
3	AMGL01089435	-	44555	44716
4	AMGL01089435	-	44227	44310
5	AMGL01089435	-	43227	43394
6	AMGL01089435	-	42925	43027
7	AMGL01089435	-	42579	42745
8	AMGL01089435	-	42174	42265
9	AMGL01089435	-	41815	41911
10	AMGL01089435	-	41618	41677
11	AMGL01089435	-	41245	41375
12	AMGL01089435	-	40238	40352
13	AMGL01089435	-	39741	39983

47. Chiru (Tibetan antelope) (*Pantholops hodgsonii*)

```
>Pantholops_hodgsonii No=47 length=1854 name="Chiru (Tibetan antelope)"
ATGGCCTGTCCCCTTCTCTTCTGGCTTCTATTTACGGCGCTTGCAACTCCCTCCCAAGGCGATCGCCTTGGCCCCACACCACATCTGCGTTACTCCAGGT
TCCTAGACGCTTCTAACGCTGTTTTCCTGCGCTGGGACTTTGACTTTGAGGCTGAAATCATCACTTTCGAGCTCCAGGTCCAGACAGCTGGCTGGGTGGG
CTTGGGTGTCACAAATCGCTACAGCAGAGCAGGAAGTGACCTGGTTGTCGGAGGCGTCTCACCTGACGGCAATGTCTATTTCTCTGACCAGCACCTGGTG
GATGAAGACACGCTGGAGGAGGATGGGAGCCAGGATGCAGAGCTGCAGGGGCTGACACAAGATGCCGTCTACACCACCATGCGCTTCTCCAGGCCCTTCC
GCTCCTGCGACCCTCACGACCAAGACATCACGAGCGACACTGTGAGGGTGCTGGCCGCCTATGGCCTGGATGACACCCCAAAGATAGATCAGGAGCGTAC
TTTTGTGAAGTCCATCTTCCTGCTCCAAATCGTCCACCCTGATGATCTCGATGCCCCTGAGGACACCATCATCCATGACTTGGAGATCACTGATTTCCTC
ATCCCAGAGGATGACACCACGTATGCCTGCACCTTCCTCCCTCTCCCCATCGTTAGCAAGAAGCACCACATCTACAAGTTCGAGCCCAAGTTGGTCCAGC
ACCGCGAGACAGTTGTGCACCACATCCTGGTGTACGCCTGCGGCAATGCCAGCGCTCTCCCCACCGGCATCAGCGACTGCTACGGGGCCGACCCCGCCTT
CTCCCTCTGCTCGCAGGTCATCGTGGGCTGGGCTGTCGGGGGCGCAAGTTACCAGTTTCCAGACGACGTGGGCATTTCTATTGGGACGCCCTTGGACCCC
CAGTGGATCCGCCTGGAGATTCATTACAGCAATTTTCACAATCTGCCTGGTCTGTACGACTCATCAGGGATCCGAGTATACTACACCGCGCACCTGCGCA
AGTTTGACATGGGCGTCCTGCAGCTGGGTGTCTTCACTTTCCCCATCCACTTCATCCCCCCCAGCGCCGAGTCCTTCAGGTCCTATGGACTGTGTAAGAC
GGAGAAGTTTGAAGAGATGAACCGGGCTCCAGTGCCCGACATCCAAGTCTTTGGCTACCTGCTCCACACCCACTTGGCCGGCCGCGCTCTGCAGGCGGTG
CAGTACAGAAATGGAACTCAACTCCGTGTAATCTGTCAAGATGATGCCTACGACTTCAATCTGCAGGAGACTCGAGATTTACCTTATCGAGTGGTGATCA
AGCCGGGGGATGAATTGCTGGTAGAGTGTCGCTACCAGACGCTGGACCGTGACTCCTTGACATTCGGGGGTCCCAGCACCATTAACGAGATGTGCCTCGT
CTTTCTCTTCTACTATCCCCGAAACAACGTCTCCAGCTGCCACGGCTACCCCGACATCATCTATGTGGCCCATGAGCTGGGGGAGGAGGCATCAGACTCC
ATGGAGGGCATGATGGCCATGAGCAATGTGGAGTGGACCCCAGAGAGCATTAAGAAGGCTGAGAATGCCTGCAAGGAGGCCCAGCAGACAGTGATAATAA
AGACCATTGATGAGCTAGTGGAAAACACGACAGGCTGGATTCGGGACATCAGCCCTACTCCCCGGGGCCCTTGTTTGGAGTCCTCTGGAGGCAAAGTGGA
GCCCCAGGACAAAACCCCTGCAGGCTTCAGGGCGGCCCCCAGGGTCCTCTCGGGGTCCAGCAGTGCCACCCTGAGGTGCCTCCCCCTGGCCACCCTCTTG
TTTGGGCAGGGGGCCCTGTCTTGGCTCCTTGCCACCCTGCAGGCTGGAATCTGA
```

```
>Pantholops_hodgsonii No=47 length=617 name="Chiru (Tibetan antelope)"
MACPLLFWLLFTALATPSQGDRLGPTPHLRYSRFLDASNAVFLRWDFDFEAEIITFELQVQTAGWVGLGVTNRYSRAGSDLVVGGVSPDGNVYFSDQHLV
DEDTLEEDGSQDAELQGLTQDAVYTTMRFSRPFRSCDPHDQDITSDTVRVLAAYGLDDTPKIDQERTFVKSIFLLQIVHPDDLDAPEDTIIHDLEITDFL
IPEDDTTYACTFLPLPIVSKKHHIYKFEPKLVQHRETVVHHILVYACGNASALPTGISDCYGADPAFSLCSQVIVGWAVGGASYQFPDDVGISIGTPLDP
QWIRLEIHYSNFHNLPGLYDSSGIRVYYTAHLRKFDMGVLQLGVFTFPIHFIPPSAESFRSYGLCKTEKFEEMNRAPVPDIQVFGYLLHTHLAGRALQAV
QYRNGTQLRVICQDDAYDFNLQETRDLPYRVVIKPGDELLVECRYQTLDRDSLTFGGPSTINEMCLVFLFYYPRNNVSSCHGYPDIIYVAHELGEEASDS
MEGMMAMSNVEWTPESIKKAENACKEAQQTVIIKTIDELVENTTGWIRDISPTPRGPCLESSGGKVEPQDKTPAGFRAAPRVLSGSSSATLRCLPLATLL
FGQGALSWLLATLQAGI
```

(1) Exon coordinates

Exon	NCBI Accession	Strand	Start	End
1	AGTT01184576	-	17100	17384
2	AGTT01184576	-	16074	16220
3	AGTT01184576	-	14284	14445
4	AGTT01184576	-	13956	14039
5	AGTT01184576	-	12956	13123
6	AGTT01184576	-	12650	12752
7	AGTT01184576	-	12304	12470
8	AGTT01184576	-	11900	11991
9	AGTT01184576	-	11539	11635
10	AGTT01184576	-	11343	11402
11	AGTT01184576	-	10974	11104
12	AGTT01184576	-	9964	10078
13	AGTT01184576	-	9429	9671

48. Cow (*Bos taurus*)

>Bos_taurus No=48 length=1857 name="Cow"
ATGGCCTGTGCCCTTCTCTCCTGGCTTCTCCTATTTACGGCCCTTGCAACTCCCTCCCAATGCAATCGCCTTGGCCCCATGCCGCGTCTGCGTTATTCCA
GGTTTCTAGACCCTTCTAACGCTGTTTTCCTGCGCTGGGACTTTGACTTTGAGGCTGAGATCATCACTTTCGAGCTCCAGGTCCGGACAGCTGGCTGGGT
GGGTTTGGGTGTCACAGATCGCTACAGCAGAGCGGGAAGCGACCTGGTTGTCGGAGGCGTCTCGCCTGACGGCAATGTCTATTTCTCGGACCAGCACCTG
GTGGATGAAAACACGCTGGAGGAGGATGGGAGCCAGGATGCAGAACTGCAGGGGCTAACAGAAGATGCCGTCTACACCACCATGCGCTTCTCCAGGCCCT
TCCGCTCCTGCGACCCTCACGACCAAGACATCACGAGCGACACTGTGAGGGTACTGGCTGCCTACGGCCTGGATGACACCCCAAAGATGGATCGGGAACG
TACTTTTGTGAAGTCCATCTTCCTGCTCCAAATCGTCCACCCTGATGATCTCGATGCCCCTGAGGACACCATCATCCATGACTTGGAGATCACTGATTTC
CTCATCCCAGAGGATGACACCACGTACGCCTGCACCTTCCTCCCTCTCCCCATCGTTAGCAAGAAGCACCACATCTACAAGTTCGAGCCCAAGTTGGTCC
AACACAAGGAGACGATTGTGCACCACATCCTGGTGTACGCCTGTGGCAATGCCAGCGCTCTCCCCACGGGCATCAGCGACTGCTATGGGGCCGACCCCGC
CTTCTCCCTCTGCACACAGGTCATCGTGGGCTGGGCTGTCGGGGGCACAAGTTACCAGTTTCCAGATGACGTGGGCATCTCTATTGGGACGCCCTTGGAC
CCCCAGTGGATCCGCCTGGAGATTCATTACAGCAATTTTCACAATCTGCCTGGTCTGTACGACTCCTCAGGGATCCGAGTATACTACACCGCACACCTGC
GCAAGTTTGACATGGGCGTCCTGCAGCTGGGCGTCTTCACTTTCCCCATCCACTTCATCCCCCCCGGCGCTGAGTCCTTCATGTCCTACGGACTGTGTAA
GACGGAGAAGTTTGAAGAGATGAACGGGGCTCCAGTGCCCGATATCCAGGTCTTTGGCTACCTGCTCCACACCCACTTGGCTGGCCGTGCTATACAGGCA
GTGCAGTACAGAAACGGAACTCAACTCCGCGTAATCTGTAAAGATGATGCCTACGACTTCAACCTACAGGAGACTCGAGATTTACCTTATCGAGTGGTGA
TCAAGCCGGGGGATGAATTGCTGGTAGAGTGTCGCTACCAGACGCTGGACCGCGACTCCTTGACATTTGGGGGTCCCAGCACCATCAATGAGATGTGCCT
CATCTTTTTCTTCTACTATCCCCGAAACAATGTCTCCAGCTGCCAGGGGTACCCGGACATCATCTACGTGGCCCACGAGCTGGGGGAGGAGGTATCAGAC
TCCATGGAGGGCATGATGGCCATGAGCAATGTGGAGTGGACCCCGGAGAGCATTAAGAAGGTTGAGAAGGCCTGCAAGGAGGCCCAGCAGACAGTGATAA
TAAAGACCATTGATGAGCTAGTGGAAAACACAACAGGCTGGATTCAGGACATCAACCCTACTCCCCGGGGTCCTTGTTTGGAGTCCTCTGGAGGCAAAGT
GGAACCCCAGGACAAAACCCCTGCAGGCTTCAGGGCGGCCCCCATTGTCCTCTCAAGGGCCAGCAGTGCCACCCTGAGGTGCCTCCCCCTGGCCGCCCTC
TTGTTTGGGCAGGGGGCCCTGTCTTGGCTCCTTGCCACCCTGCAGTCTGGAATCTGA

>Bos_taurus No=48 length=618 name="Cow"
MACALLSWLLLFTALATPSQCNRLGPMPRLRYSRFLDPSNAVFLRWDFDFEAEIITFELQVRTAGWVGLGVTDRYSRAGSDLVVGGVSPDGNVYFSDQHL
VDENTLEEDGSQDAELQGLTEDAVYTTMRFSRPFRSCDPHDQDITSDTVRVLAAYGLDDTPKMDRERTFVKSIFLLQIVHPDDLDAPEDTIIHDLEITDF
LIPEDDTTYACTFLPLPIVSKKHHIYKFEPKLVQHKETIVHHILVYACGNASALPTGISDCYGADPAFSLCTQVIVGWAVGGTSYQFPDDVGISIGTPLD
PQWIRLEIHYSNFHNLPGLYDSSGIRVYYTAHLRKFDMGVLQLGVFTFPIHFIPPGAESFMSYGLCKTEKFEEMNGAPVPDIQVFGYLLHTHLAGRAIQA
VQYRNGTQLRVICKDDAYDFNLQETRDLPYRVVIKPGDELLVECRYQTLDRDSLTFGGPSTINEMCLIFFFYYPRNNVSSCQGYPDIIYVAHELGEEVSD
SMEGMMAMSNVEWTPESIKKVEKACKEAQQTVIIKTIDELVENTTGWIQDINPTPRGPCLESSGGKVEPQDKTPAGFRAAPIVLSRASSATLRCLPLAAL
LFGQGALSWLLATLQSGI

(1) Exon coordinates

Exon	UCSC Chromosome	Strand	Start	End
1	bosTau7 chr4	-	108609843	108610130
2	bosTau7 chr4	-	108608828	108608974
3	bosTau7 chr4	-	108607174	108607335
4	bosTau7 chr4	-	108606847	108606930
5	bosTau7 chr4	-	108605841	108606008
6	bosTau7 chr4	-	108605545	108605647
7	bosTau7 chr4	-	108605200	108605366
8	bosTau7 chr4	-	108604796	108604887
9	bosTau7 chr4	-	108604438	108604534
10	bosTau7 chr4	-	108604242	108604301
11	bosTau7 chr4	-	108603886	108604016
12	bosTau7 chr4	-	108602874	108602988
13	bosTau7 chr4	-	108602342	108602584

49. Killer whale (*Orcinus orca*)

```
>Orcinus_orca No=49 length=1853 name="Killer whale"
ATGGCCTGTGCCCTTCTCTTTCGGCTTCTCCTACTTATGGCCCTGCTGACCTCCTCTCAAGGCAACCACCTGGGCCTCACATTATGTCTGCATTATTCCA
TGTTCCTAGATCCTTCTAATGTTACTTTCCTGCACTGGGACTTTGACCTTGAGGCTGAGATCGTCACTTTTGATCTCCAGGTCCAGACAGCTGGCTGGGT
GGGCTTGGGTATCACAAATCGCTACACCATGGTGGGAAGCAATCTGGTTGTTGGAGGAGTCTCGCCGGATGGCAATGTCTATTTCTCGAATCAGCACCTG
GTGGATGAAGACGCTCTGGAGGAGGGAGCCAGAACGCAGAGCTGCAGGCACTGACAGAAGACACCATCTATACCACCATGCGCTTCTCCAGGCCCTTCTG
CTCCTGTGACCCTCAAGACCAAGACATCACGAGTGACACTGTGAGGGTGCTTGCCACCTACGGCCCAGATGACACTCTGAAGCTGGATCGGGAGCGTACT
TTTGTGAGTCCATCTTCCTGCTCCAAATTGTCCACCCCGATGATCTCGATGTCCCCGAGGACACCATCATCCATGACTTGGAGATCACTGATTTCTTCAT
CCCAGAGGATGACACCACGTACGCCTGCACCTTCCTCCCTCTCTCCATCGTTAGCAAGAAGCACCGTATCTACAAGTTTGAGCCCAAGTTGGTCCATCAC
AAGGAGACGATGGTGCACCACATCCTGGTGTACCGCCTGCAGCAAAACCAGTGCTCTGCCCAAGGGCATCAGCGACTGCTACGGGGCTGACCCCGCCTCC
TCCCTCTGCTCACAGGTCATCGTGGGCTGGGCTATCGGGGACACAAGTTACCAGTTTCCAGGTGACGTGGGTATCTCTACTGGGATGGCTTTGGACCCCC
AGTGGGTCCCACTGGAGATTCACTACAGCAATTTGCACAGTCTTCCTGGTGTGTACGGCTCCTCGGGGATTCGAGTGTACTACACGGCGCAGCTACGCAA
ATATGACATGGGTGTCCTGCAGCTGGGCTTCTGCACTTTGCCCATCCACTTCGTACCCCCGGGTGCCGAGTCCTTCGCGTCCTATGGGCTGTGTAAGGCC
GAGAAGTTTGATGAGATGAACGGGGCCCCGGTGCCTGACATCCAGGTCTGCGGCTACCTGCTCCACACCCACTTGGCTGGCCGCACTCTGCAGGCCGTGC
AATACAGAAATGGAACACAACGCCAAACAATCTGGAAAGATGATACCTATGACTTGAATCTGCAGGAGACTTGAGATTTACCTTCTTGAGTGGCCATCAA
GCCAGGAGATGAATTGTTGGTAGGGTGTCACCATCAGACGCTGGACCACGACTCCTTGACTTTTGGGGGTCCCAGCACCGTTAATGAGATGTGCCTCATC
TTTCTCTTCTACTATATCTGAAACAACATCTCCAGCTGCATGGGGTACGCTGACATCATCTACGTGGCCCATGTGCTGGGGGAGGAGGCATCAGATTCTA
TGGAGGGCATGATGGCCATGAACAATGTGGAGTGGACTCCGGAGAACATTAAAAAGGCTGAGAAGGCCTGCGAGGAGGCCCAGCAGATGGTGCTAATAAA
GACCATTGACGAGCTAGTGGAAAACAGAACAGGCTGGATTCTGGAAATCATCCCTGCTCCCCGGGGTCCTTGCTTAGAGTCCTCCAGAGGCAAAGTGGAG
CCCCAGGACAGAACCCCTGTAGGCTTCAGGGCTGCCCCCATGGCCCTCTCGGGCTCCAGCACTGCCACACCAAGGCGCCTCCCCCTGACTGCCCTCTTGT
TTGGGCAGGGGGCCGTGTCTTGGCTCCTAGCCACCCTGCGGGCTGGAGCCTGA
```

(1) Exon coordinates

Exon	NCBI Accession	Strand	Start	End
1	ANOL02032434	+	78576	78863
2	ANOL02032434	+	79751	79893
3	ANOL02032434	+	81104	81264
4	ANOL02032434	+	81506	81589
5	ANOL02032434	+	82418	82586
6	ANOL02032434	+	82774	82876
7	ANOL02032434	+	83075	83241
8	ANOL02032434	+	83561	83652
9	ANOL02032434	+	83935	84031
10	ANOL02032434	+	84159	84218
11	ANOL02032434	+	85326	85456
12	ANOL02032434	+	86218	86332
13	ANOL02032434	+	86631	86873

50. Common bottlenose dolphin (*Tursiops truncatus*)

```
>Tursiops_truncatus No=50 length=1844 name="Common bottlenose dolphin"
ATGGCCTGTGCCCTTCTCTTTCGGCTTCTCCTACTTACGGCCCTGCCGACCTCCTCTCAAGGCAACCACCTGGGCCTCACATCACGTCTGCGTTATTCCA
TGTTCCTAGATCCTTCTAATGTTACTTTCCTGCACTGGGACTTTGACCTTGAGGCTGAGGTCGTCACTTTTGAGCTCCAGGTCCAGACAGCTGGCTGGGT
GGGCTTGGGTATCACAAATAGCTACACCATGGTGGGAAGCAATCTGGTTGTTGGAGGAGTCTCGCCGGACGGCAATGTCTATTTCTCGAATCAGCACCTG
GTGGATGAAGACGCTCTGGAGGAGGGAGCCAGGACGCAGAGCTGCAGGCACTGACAGAAGACACCATCTATACCACCATGTGCTTCTCCAGGCCCTTCTG
CTCCTGTGACCCTCAAGACCAAGACATCACGAGTGACACTGTGAGGGTGCTTGCCACCTACGGCCCAGATGACACTCTGAAGCTGGATCGGGAGCGTACT
TTTGTGAGTCCATCTACAAATTGTCCACCCCGATGATCTTGATGTCCCCGAGGACACCATCATCCATGACTTGGAGATCACTGATTTCTTCATCCCAGAG
GATGACACCACGTACGCCTGCACCTTCCTCCCTCTCTCCATCGTTAGCAAGAAGCACCGTATCTACAAGTTTGAGCCCAAGTTGGTCCATCACAAGGAGA
CGACGGTGCACCACATCCTGGTGTACCGCCTGCGGCAACACCAGTGCTCTGCCCAAGGGCATCAGCGACTGCTACGGGGCCGACCCCGCCTCCTCCCTCT
GCTCACAGGTCATCGTGGGCTGGGCTGTCGGGGACACAAGTTACCAGTTTCCAGGTGACGTGGGTATCTCTACTGGGATGGCTTTGGACCCCCAGTGGGT
CCGACTGGAGATTCACTACAGCAGTTTGCACAGTCTTCCTGGTGTATACGGCTCCTCGGGGATTCGAGTGTACTACACGGCGCAGCTACGCAAATATGAC
ATGGGTGTCCTGCAGCTGGGCTTCTGCACTTTGCCCATCCACTTCATACCCCCGAGTGCCGAGTCCTTCGCGTCCTATGGGCTGTGTAAGGCGGAGAAGT
TTGACGAGATGAACGGGGCCCCGGTGCCTGACATCCAGGTCTGCGGCTACCTGTTCCACACCCACTTGGCTGGCCGCACTCTGCAGGCCGTGCAATACAG
AAATGGAACACAACGCCAAACAATCTGGAAAGATGATACCTATGACTTGAATCTGCAGGAGACTTGAGATTTACCTTCTTGAGTGGCCATCAAGCCAGGA
GATGAATTGTTGGTAGGGTGTCACTATCAGACGCTGGACCACGACTCCTTGACTTTTGGGGGTCCCAGCACCGTTAATGAGATGTGCCTCATCTTTCTCT
TCTACTATATCCGAAACAACATCTCCAGCTGCATGGGGTACGCTGACATCATCTACGTGGCCCATGTGCTGGGGGAGGAGGCATCAGATTCCATGGAGGG
CATGATGGCCATGAACAATGTGGAATGGACTCCGGAGAACATTAAAAAGGCTGAGAAGGCCTGCGAGGAGGCCCAGCAGATGGTGATAATAAAGACCATT
GACGAACTAGTGGAAAACAGAACAGGCTGGATTCCGGGAATCATCCCTGCTCCCCGGGGTCCTTGCTTAGAGTCCTCCAGAGGCAAAGTGGAGCCCCAGG
ACAGAACCCCTGTAGGCTTCAGGGCTGCCCCCATGGCCCTCTCGGGCTCCAGCGCTGCCACACCAAGGCGCCCCCCCGACTGCCCTCTTGTTTGGGCAGG
GGGCCGTGTCTTGGCTCCTAGCCACCCTGCGGGCTGGAGCCTGA
```

(1) Exon coordinates

Exon	UCSC Chromosome	Strand	Start	End
1	turTru2 JH475017	-	75254	75541
2	turTru2 JH475017	-	74230	74372
3	turTru2 JH475017	-	72729	72882
4	turTru2 JH475017	-	72403	72486
5	turTru2 JH475017	-	71406	71574
6	turTru2 JH475017	-	71116	71218
7	turTru2 JH475017	-	70748	70914
8	turTru2 JH475017	-	70337	70428
9	turTru2 JH475017	-	69958	70054
10	turTru2 JH475017	-	69771	69830
11	turTru2 JH475017	-	68532	68662
12	turTru2 JH475017	-	67651	67765
13	turTru2 JH475017	-	66593	66833

51. Finless porpoise (*Neophocaena phocaenoides*)

```
>Neophocaena_phocaenoides No=51 length=1853 name="Finless porpoise"
ATGGCCTGTGCCCTTCTCTTTCGGCTTCTCCTACTTATGGCCCTGCTGACCTCCTCTCAAGGCAACCACCTGGGCCTCACATCACGTCTGCGTTATTCCA
TGTTCCTAGATCCTTCTAATGTTATTTTCCTGCACTGGGACTTTGACCTTGAGGCTGAGATCATCACTTTTGAGCTCCAGGTCCAGACAGCTGGCTGGGT
GGGCTTGGGTATCACAAATCGCTACACCATGGTGGGAAGCAATCTGGTTGTTGGAGGAGTCTCGCCAGACGGCAATGTCTATTTCTCGAATCAGCACCTG
GTGGATGAAGACGCTCTGGGGGAGGGAGCCAGGACGCAGAGCTGCTGGCACTGACAGAAGACACCATCTATACCACCATGCGCTCCTCCAGGCCCTTCCG
CTCCTGCGACCCTCAAGACCAAGACATCACGAGTGACACTGTGAGGGTGCTTGCCACCTACGGCCCAGATGACACTCTGAAGCTGGATCGGGAGCGTACT
TTTGTGAGTCCATCTTCCCGCTCCACATTGTCCACCCCGATGATCTCGATGTCCCCGAGGACACCATCGTCCATGACTTGGAGATCACTGATTTCTTCAT
CCCAGAGGATGACACCACGTACGCCTGCCCCTTCCTCCCTCTCTCCATCGTTGGCAAGAAGCACCGTATCTCCAAGTTTGAGCCCAAGTTGGTCCATCAC
AAGGGGACGACGGTGCACCACATCCTGGTGTACCGCCTGGGGCAACACCAGTGCTCTGCCCAAGGGCATCAGCGACTGCTACGGGGCTGACCCCGCCTCC
TCCCTCTGCTCACAGGTCATCGTGGGCTGGGCTGTCGGGGACACAAGTTACCAGTTTCCAGGTGACGTGGGTATCTCTACTGGGATGGCTTTGGACCCCC
AGTGGGTCCGACTGGAGATTCACTACAGCAATTTGCACAGTCTTCCTGGTGTGTACGGCTCCTCGGGGATTCGAGTGTACTACACGGCGCAGCTACGCAA
ATATGACATGGGTGTCCTGCAGCTGGGCTTCTGCACTTTGCCCATCCACTTCATACCCCCGGGTGCCGAGTCCTTCACGTCCTATGGGCTGTGTAAGGCA
GAGAAGTTTGACGAGATGAACGGGGCCCCGGTGCCTGACATCCAGGTCTGCAGCTACCTGCTCCACACCCACTTGGCTGGCCGCACTCTGGAGGCCGTGC
AATACAGAAATGGAACACAATGCCGAACAATCTGGAAAGATGATTCCTATGACTTGAATCTGCAGGAGACTCGAGATTTACCTTCTTGAGTGGCCATCAA
GCCAGGAGATGAATTGTTGGTAGGGTGTCACTACCAGACGCTGGACCGTGACTCCTTGACTTTTGGGGGTCCCAGCACCGTTAATGAGATGTGCCTCATC
TATCTCTTCTACTATATCCGAAACAACATCTCCAGCTGCATGGGGTACGCTGACATCATCTACGTGGCCCATGTCCTGGGGGAGGAGGCATCAGATTCCA
TGGAGGGCATGATGGCCATGAACAATGTAGAGTGGACTCTGGAGAACATTAAAAAGGCTGAGAAGGCCTGCGAGGAGGCCCAGCAGATGGTGATAATAAA
GACCATTGATGAGCTAGTGGAAAACACAACAGGCTGGATTCCGGAAATCATCCCTGCTCCCCGGGGTCCTTGCTTAGAGTCCTCCAGAGGCAAAGCGGAG
CCCCAGGACAGAACCCCTGTAGGCTTCAGGGCTGCCCCCATGGCCCTCTCGGGCTCCAGCACTGCCACACCAAGGCGCCTCCCCCTGACTGCCCTCTTGT
TTGGGCAGGGGGCCGTGTCTTGGCTCCTAGCCACCCTGCGGGCTGGAGCCTGA
```

(1) The exon 1 coding sequence was predicted by assembling WGS data using CAP3. The exon 1 is marked in red.

```
                          .    :    .    :    .    :    .    :    .    :    .    :
SRR940959.400686013.+ GCCTAGCACAGGCAGAGGTACATATAAGTCCATACTTCTTGAGTGTCCATCTAAGGAGAG
SRR940959.170141498.-    TAGCACAGGCAGAGGTACATATAAGTCCATACTTCTTGAGTGTCCATCTAAGGAGAG
SRR940959.286897225.+        ACAGGCAGAGGTACATATAAGTCCATACTTCTTGAGTGTCCATCTAAGGAGAG
SRR940959.236186330.+          AGGCAGAGGTACATATAAGTCCATACTTCTTGAGTGTCCATCTAAGGAGAG
SRR940959.75028513.1-             CAGAGGTACATATAAGTCCATACTTCTTGAGTGTCCATCTAAGGAGAG
SRR940959.282873597.+             CAGAGGTACATATAAGTCCATACTTCTTGAGTGTCCATCTAAGGAGAG
SRR940959.90677119.2-                    ACATATAAGTCCATACTTCTTGAGTGTCCATCTAAGCAGAG
SRR940959.157206613.-                    ACATATAAGTCCATACTTCTTGAGTGTCCATCTAAGGAGAG
SRR940959.429743884.-                      ATATAAGTCCATACTTCTTGAGTGTCCATCTAAGGAGAG
SRR940959.440705240.-                      ATATAAGTCCATACTTCTTGAGTGTCCATCTAAGGAGAG
SRR940959.13629182.1-                              CCATACTTCTTGAGTGTCCATCTAAGGAGAG
SRR940959.64016330.1+                                ATACTTCTTGAGTGTCCATCTAAGGAGAG
SRR940959.118974291.+                                         GAGTGTCCATCTAAGGAGAG
SRR940959.205784032.+                                         GAGTGTCCATCTAAGGAGAG
SRR940959.279165074.+                                         GAGTGTCCATCTAAGGAGAG
SRR940959.305653689.+                                                 ATCTAAGGAGAG
SRR940959.50517177.2+                                                      AGGAGAG
SRR940959.358909436.+                                                      AGGAGAG
SRR940959.261495061.+                                                        GAGAG
                      ____________________________________________________________
consensus             GCCTAGCACAGGCAGAGGTACATATAAGTCCATACTTCTTGAGTGTCCATCTAAGGAGAG
```

```
                          .    :    .    :    .    :    .    :    .    :    .    :
SRR940959.400686013.+ ATGCTTCAGACCCCAGGAGCCATGGCCTGTGCCCTTCTCT
SRR940959.170141498.- ATGCTTCAGACCCCAGGAGCCATGGCCTGTGCCCTTCTCTGTC
SRR940959.286897225.+ ATGCTTCAGAGCCCAGGAGCCATGGCCTGTGCCCTTCTCTTTCGGCT
SRR940959.236186330.+ ATGCTTCAGACCGCAGGAGCCATGGCCTGTGCCCTTCTCTTTCGGCTTC
SRR940959.75028513.1- ATGCTTCAGACCCCAGGAGCCATGGCCTGTGCCCTTCTCTTTCGGCTTCTCC
SRR940959.282873597.+ ATGCTTCAGACCCCAGGAGCCATGGCCTGTGCCCTTCTCTTTCGGCTTCTCC
SRR940959.90677119.2- ATGCTTCACACCCCAGGAGCCATGGCCTGTGCCCTTCTCTTTCGGCTTCTCCTACTTAT
SRR940959.157206613.- ATGCTTCAGACCCCAGGAGCCATGGCCTGTGCCCTTCTCTTTCGGCTTCTCCTACTTAT
SRR940959.429743884.- ATGCTTCAGACCCCAGGAGCCATGGCCTGTGCCCTTCTCTTTCGGCTTCTCCTACTTATG
SRR940959.440705240.- ATGCTTCAGACCCCAGGAGCCATGGCCTGTGCCCTTCTCTTTCGGCTTCTCCTACTTATG
SRR940959.13629182.1- ATGCTTCAGACCCCAGGAGCCATGGCCTGTGCCCTTCTCTTTCGGCTTCTCCTACTTATG
SRR940959.64016330.1+ ATGCTTCAGACCCCAGGAGCCATGGCCTGTGCCCTTCTCTTTCGGCTTCTCCTACTTATG
SRR940959.118974291.+ ATGCTTCAGACCCCAGGAGCCATGGCCTGTGCCCTTCTCTTTCGGCTTCTCCTACTTATG
SRR940959.205784032.+ ATGCTTCAGACCCCAGGAGCCATGGCCTGTGCCCTTCTCTTTCGGCTTCTCCTACTTATG
SRR940959.279165074.+ ATGCTTCAGACCCCAGGAGCCATGGCCTGTGCCCTTCTCTTTCGGCTTCTCCTACTTATG
SRR940959.305653689.+ ATGCTTCAGACCCCAGGAGCCATGGCCTGTGCCCTTCTCTTTCGGCTTCTCCTACTTATG
SRR940959.50517177.2+ ATGCTTCAGACCCCAGGAGCCATGGCCTGTGCCCTTCTCTTTCGGCTTCTCCTACTTATG
SRR940959.358909436.+ ATGCTTCAGACCCCAGGAGCCATGGCCTGTGCCCTTCTCTTTCGGCTTCTCCTACTTATG
SRR940959.261495061.+ ANGCTTCAGACCCCAGGAGCCATGGTCTGTGCCCTTCTCTTTCGGCTGCTCCTACGTATG
SRR940959.165070591.-    CTTCAGACCCCAGGAGCCATGGCCTGTGCCCTTCTCTTTCGGCTTCTCCTACTTATG
SRR940959.170260804.-    CTTCAGTCCCCAGGAGCCATGGCCTGTGCCCTTCTCTTTCGGCTTCTCCTACTTATG
SRR940959.505142265.-    NTTCAGACCCCAGGAGCCATGGCCTGTGCCCTTCTCTTTCGGCTTCTCCTACTTATG
SRR940959.281541208.-       CAGCCCCAGGAGCCATGGCCTGTGCCCTTCTCTTTCGGCTTCTCCTACTTATG
SRR940959.454596752.-       CAGACCCCAGGAGCCATGGCCTGTGCCCTTCTCTTTCGGCTTCTCCTACTTATG
SRR940959.391430906.+               AGGAGCCATGGCCTGTGCCCTTCTCTTTCGGCTTCTCCTACTTATG
SRR940959.444515711.+               AGGAGCCATGGCCTGTGCCCTTCTCTTTCGGCTTCTCCTACTTATG
```

```
SRR940959.353374098.-                     CATGGCCTGTGCCCTTCTCTTTCGGCTTCTCCTACTTATG
SRR940959.263431075.-                         GCCTGTGCCCTTCTCTTTCGGCTTCTCCTACTTATG
SRR940959.304744550.-                                                TCTCCTACTTATG
SRR940959.186610766.+                                                          ATG
SRR940959.295941335.-                                            GGCTTCTCCTACTTATG
SRR940959.48863463.1-                             GTGCCCTTCTCTTTCGGCTTCTCCTACTTATG
SRR940959.312942858.-                             GTGCCCTTCTCTTTCGGCTTCTCCTACTTATG
SRR940959.422306854.-                                                   CCTACTTATG
SRR940959.291328423.-                                       TCTTTCGGCTTCTCCTACTTATG
SRR940959.486713378.-                                       TCTTTCGGCTTCTCCTACTTATG
SRR940959.499867599.+                                            CGGCTTCTCCTACTTATG
SRR940959.522698290.+                                   CTTCTCTTTCGGCTTCTCCTACTTATG
                      ____________________________________________________________
consensus             ATGCTTCAGACCCCAGGAGCCATGGCCTGTGCCCTTCTCTTTCGGCTTCTCCTACTTATG

                          .    :    .    :    .    :    .    :    .    :    .    :
SRR940959.429743884.- G
SRR940959.440705240.- G
SRR940959.13629182.1- GCCCTGCTG
SRR940959.64016330.1+ GCCCTGCTGAC
SRR940959.118974291.+ GCCCTGCTGACCTCCTCTCA
SRR940959.205784032.+ GCCCTGCTGACCTCCTCTCA
SRR940959.279165074.+ GCCCTGCTGACCTCCTCTCA
SRR940959.305653689.+ GCCCTGCTGACCTCCTCTCAAGGCAACC
SRR940959.50517177.2+ GCCCTGCTGACCTCCTCTCAAAGCAACCACCTG
SRR940959.358909436.+ GCCCTGCTGACCTCCTCTCAAGGCAACCACCTG
SRR940959.261495061.+ GCCCTGCTGACCTCCTCGCAAGGCAACCACCTGGG
SRR940959.165070591.- GCCCTGCTGACCTCCTCTCAAGGCAACCACCTGGGCCTCACAT
SRR940959.170260804.- GCCCTGCTGACCTCCTCTCAAGGCAACCTCCTGGGCCTCACAT
SRR940959.505142265.- GCCCTGCTGACCTCCTCTCAAGGCAACCACCTGGGCCTCACAT
SRR940959.281541208.- GCCCTGCTGACCTCCTCTCAAGGCAACCACCTGGGCCTCACATCAC
SRR940959.454596752.- GCCCTGCTGACCTCCTCTCAAGGCAACCACCTGGGCCTCACATCAC
SRR940959.391430906.+ GCCCTGCTGACCTCCTCTCAAGGCAACCACCTGGGCCTCACATCACGTCTGCGT
SRR940959.444515711.+ GCCCTGCTGACCTCCTCTCAAGGCAACCACCTGGGCCTCACATCACGTCTGCGT
SRR940959.353374098.- GCCCTGCTGACCTCCTCGCAAGGCAACCACCTGGGCCTCACATCACGTCTGCGTTATTCC
SRR940959.267063405.+                      GGCAACNACCTGGGCCTCACATCACGTCTGTGTTATTCC
SRR940959.263431075.- GCCATGCTGACCTCCTCTCAAGGCAACCACCTGTGCCTCACATCACGTCTGCGTTATNCC
SRR940959.263027516.+                   CAAGGCNACCACCTGGGCCTCACATCACGTTTGCGTTATTCC
SRR940959.113434796.+                                                 CTGCGTTATTCC
SRR940959.175038186.+              CCTCTCAAGGCAACCACCGGGGCCTCACATCACGTCTGCGTTATTCC
SRR940959.304744550.- GCCCTGCTTACCTCCTCTCAAGGCAACCCCCTGGGCCTCACATCACGTCTGCGTTATTCC
SRR940959.263424944.-                      GGCAACCACCTGGGCCTCACATCACGTCTGCGTTATTCC
SRR940959.324454424.+                                       ACATCACGTCTGCGTTATTCC
SRR940959.55885928.1+                                    CTCACATCACGTCTGCGTTATTCC
SRR940959.126351496.-                               TGGGCCTCACATCACGTCTGCGTTATTCC
SRR940959.71054726.1+                                                   GCGTTATTCC
SRR940959.170610408.+                                                   GCGTTATTCC
SRR940959.175639215.+                                                          TCC
SRR940959.186610766.+ GCCCTGCTGACCTCCTCTCAAGGCAACCACCTGGGCCTCACATCACGTCTGCGTTATTCC
SRR940959.194971739.-                                      CACATCACGTCTGCGTTATTCC
SRR940959.207465442.+  CCCTGCTGACCTCCTCTCAAGGCAACCACCTGGGCCTCACATCACGTCTGCGTTATTCC
SRR940959.215290207.-                                             ACGTCTGCGTTATTCC
SRR940959.295941335.- GCCCTGCTGACCTCCTCTCAAGGCAACCACCTGGGCCTCACATCACGTCTGCGTTATTCC
SRR940959.48863463.1- GCCCTGCTGACCTCCTCTCAAGGCAACCACCTGGGCCTCACATCACGTCTGCGTTATTCC
SRR940959.312942858.- GCCCTGCTGACCTCCTCTCAAGGCAACCACCTGGGCCTCACATCACGTCTGCGTTATTCC
SRR940959.325940630.-                                  GGCCTCACATCACGTCTGCGTTATTCC
SRR940959.326434896.-                 CTCAAGGCAACCACCTGGGCCTCACATCACGTCTGCGTTATTCC
SRR940959.55013119.2-                      GGCAACCACCTGGGCCTCACATCACGTCTGCGTTATTCC
SRR940959.392191146.+                      GGCAACCACCTGGGCCTCACATCACGTCTGCGTTATTCC
SRR940959.421103346.+                   CAAGGCAACCACCTGGGCCTCACATCACGTCTGCGTTATTCC
SRR940959.422306854.- GCCCTGCTGACCTCCTCTCAAGGCAACCACCTGGGCCTCACATCACGTCTGCGTTATTCC
SRR940959.467774847.+      GCTGACCTCCTCTCAAGGCAACCACCTGGGCCTCACATCACGTCTGCGTTATTCC
SRR940959.471259936.+                         ACCACCTGGGCCTCACATCACGTCTGCGTTATTCC
SRR940959.291328423.- GCCCTGCTGACCTCCTCTCAAGGCAACCACCTGGGCCTCACATCACGTCTGCGTTATTCC
SRR940959.486713378.- GCCCTGCTGACCTCCTCTCAAGGCAACCACCTGGGCCTCACATCACGTCTGCGTTATTCC
SRR940959.308009405.-            CTCCTCTCAAGGCAACCACCTGGGCCTCACATCACGTCTGCGTTATTCC
SRR940959.270914562.-            CTCCTCTCAAGGCAACCACCTGGGCCTCACATCACGTCTGCGTTATTCC
SRR940959.488362309.-            CTCCTCTCAAGGCAACCACCTGGGCCTCACATCACGTCTGCGTTATTCC
SRR940959.499867599.+ GCCCTGCTGACCTCCTCTCAAGGCAACCACCTGGGCCTCACATCACGTCTGCGTTATTCC
SRR940959.522698290.+ GCCCTGCTGACCTCCTCTCAAGGCAACCACCTGGGCCTCACATCACGTCTGCGTTATTCC
                      ____________________________________________________________
consensus             GCCCTGCTGACCTCCTCTCAAGGCAACCACCTGGGCCTCACATCACGTCTGCGTTATTCC

                          .    :    .    :    .    :    .    :    .    :    .    :
SRR940959.113434796.-                                                      GGCTGAG
SRR940959.267063405.+ ATGTTCCTAGATCCTTCTAATATTATTTACCTGCACTGGGACTTTGACCTTGAGGCTGAG
SRR940959.263431075.- ATGT
```

```
SRR940959.263027516.+ GTGTTCCTAGATCCTTCTAATGTTATTTTCCTGCACTGGGACTTTGACCTTGAGGCTG
SRR940959.113434796.+ CTGTTCCTAGATCCTTCTAATGTTATTTTCCTGCACTGGGACTTTGACCTTGAGNCTGAG
SRR940959.175038186.+ ATGTTCCTAGATCCTTCTAATGTTATTTTCCTGCACTGGGGCTTTGACCTTGA
SRR940959.304744550.- ATGTTCCTAGATCCTTCTAATGTTATT
SRR940959.263424944.- TTGTTCCTAGATCCTTCTAATGTTATTTTCCTGCACTGGGACTTTGACCTTGAGNCTGAG
SRR940959.90883575.1-                                                        CTGAG
SRR940959.102275422.-                                    CTGGGACTTTGACCTTGAGGCTGAG
SRR940959.31698779.2-                                                     AGTCTGAG
SRR940959.324454424.+ ATGTTCCTAGATCCTTCTAATGTTATTTTCCTGCACTTGGACTTTGACCTTGAGGCTGAG
SRR940959.113877286.-                                              GACCTTGAGGCTGAG
SRR940959.50537691.1-                                                      GGCTGAG
SRR940959.10516563.1+                TCTAATGTTATTTTCCTGCACTGGGACTTTGACCTTGAGGCTGAG
SRR940959.55885928.1+ ATGTTCCTAGATCCTTCTAATGTTATTTTCCTGCACTGGGACTTTGACCTTGAGGCTGAG
SRR940959.64659881.2-              CTTCTAATGTTATTTTCCTGCACTGGGACTTTGACCTTGAGGCTGAG
SRR940959.126351496.- ATGTTCCTAGATCCTTCTAATGTTATTTTCCTGCACTGGGACTTTGACCTTGAGGCTGAG
SRR940959.140480633.+                    ATGTTATTTTCCTGCACTGGGACTTTGACCTTGAGGCTGAG
SRR940959.71054726.1+ ATGTTCCTAGATCCTTCTAATGTTATTTTCCTGCACTGGGACTTTGACCTTGAGGCTGAG
SRR940959.170610408.+ ATGTTCCTAGATCCTTCTAATGTTATTTTCCTGCACTGGGACTTTGACCTTGAGGCTGAG
SRR940959.175639215.+ ATGTTCCTAGATCCTTCTAATGTTATTTTCCTGCACTGGGACTTTGACCTTGAGGCTGAG
SRR940959.186610766.+ ATGTTCCTAGATCCTTCTAATGTTATTTTCCTGCACT
SRR940959.194971739.- ATGTTCCTAGATCCTTCTAATGTTATTTTCCTGCACTGGGACTTTGACCTTGAGGCTGAG
SRR940959.207465442.+ ATGTTCCTAGATCCTTCTAATGTTATTTTCCTGCACTGGGA
SRR940959.210586738.-                                                     AGGCTGAG
SRR940959.215108946.-         AGATCCTTCTAATGTTATTTTCCTGCACTGGGACTTTGACCTTGAGGCTGAG
SRR940959.215290207.- ATGTTCCTAGATCCTTCTAATGTTATTTTCCTGCACTGGGACTTTGACCTTGAGGCTGAG
SRR940959.218346462.+                                                CCTTGAGGCTGAG
SRR940959.279165074.-      CCTAGATCCTTCTAATGTTATTTTCCTGCACTGGGACTTTGACCTTGAGGCTGAG
SRR940959.295442191.-  TGTTCCTAGATCCTTCTAATGTTATTTTCCTGCACTGGGACTTTGACCTTGAGGCTGAG
SRR940959.295941335.- ATGTTCCTAGATCCTTCTAATGT
SRR940959.306237691.+          GATCCTTCTAATGTTATTTTCCTGCACTGGGACTTTGACCTTGAGGCTGAG
SRR940959.48863463.1- ATGTTCCT
SRR940959.312942858.- ATGTTCCT
SRR940959.325940630.- ATGTTCCTAGATCCTTCTAATGTTATTTTCCTGCACTGGGACTTTGACCTTGAGGCTGAG
SRR940959.326434896.- ATGTTCCTAGATCCTTCTAATGTTATTTTCCTGCACTGGGACTTTGACCTTGAGGC
SRR940959.103693456.-                              CCTGCACTGGGACTTTGACCTTGAGGCTGAG
SRR940959.379107729.-                              CCTGCACTGGGACTTTGACCTTGAGGCTGAG
SRR940959.55013119.2- ATGTTCCTAGATCCTTCTAATGTTATTTTCCTGCACTGGGACTTTGACCTTGAGGCTGAG
SRR940959.392191146.+ ATGTTCCTAGATCCTTCTAATGTTATTTTCCTGCACTGGGACTTTGACCTTGAGGCTGAG
SRR940959.421103346.+ ATGTTCCTAGATCCTTCTAATGTTATTTTCCTGCACTGGGACTTTGACCTTGAGGCTG
SRR940959.422306854.- ATGTTCCTAGATCCTTCTAATGTTATTTTC
SRR940959.424879066.-                                              GACCTTGAGGCTGAG
SRR940959.467774847.+ ATGTTCCTAGATCCTTCTAATGTTATTTTCCTGCACTGGGACTTT
SRR940959.471259936.+ ATGTTCCTAGATCCTTCTAATGTTATTTTCCTGCACTGGGACTTTGACCTTGAGGCTGAG
SRR940959.479210167.-                                                      GGCTGAG
SRR940959.291328423.- ATGTTCCTAGATCCTTC
SRR940959.486713378.- ATGTTCCTAGATCCTTC
SRR940959.308009405.- ATGTTCCTAGATCCTTCTAATGTTATTTTCCTGCACTGGGACTTTGACCTT
SRR940959.270914562.- ATGTTCCTAGATCCTTCTAATGTTATTTTCCTGCACTGGGACTTTGACCTT
SRR940959.488362309.- ATGTTCCTAGATCCTTCTAATGTTATTTTCCTGCACTGGGACTTTGACCTT
SRR940959.499867599.+ ATGTTCCTAGATCCTTCTAATG
SRR940959.522698290.+ ATGTTCCTAGATC
                      ____________________________________________________________
consensus             ATGTTCCTAGATCCTTCTAATGTTATTTTCCTGCACTGGGACTTTGACCTTGAGGCTGAG

                          .    :    .    :    .    :    .    :    .    :    .    :
SRR940959.113434796.- ATCATCACTTCTGAGCTCCAGGTNCAGACAGCTGGCTGGGTGNGCTTGGNTATCACAAAT
SRR940959.267063405.+ A
SRR940959.113434796.+ ATCATCACTTTTGAGCTCCAGGTCCAGA
SRR940959.263424944.- A
SRR940959.522237424.+                          AGACAGCTGGCTGGGGGGGCTTGGGTATCACAAAT
SRR940959.90883575.1- ATCATCACTTTTGAGCTCCAGGTCCAGACAGCTGGCTGGGTGGGNTTGGGTATCACAAAT
SRR940959.102275422.- ATCATCACTTTTGAGCTCCAGGTCNAGACAGCTGGCTGGGTGGGCTTGGGTATCACAAAT
SRR940959.31698779.2- ATCATCACTTTTGAGCTCCAGGTCCAGACAGCTGGCTGGGTGGGCTTGGGTATCACAAAT
SRR940959.281507910.-        CTTTTGAGCTCCAGGTCCAGACAGCTGGCTGGGTGGGCTTGGGTACCACAAAT
SRR940959.324454424.+ ATCATCACTTTTGAGCTCC
SRR940959.113877286.- ATCATCACTTTTGAGCTCNAGGTCCAGACAGCTGGCTGGGTGGGCTTGGGTATCACAAAT
SRR940959.50537691.1- ATCATCACTTTTGAGCTCCAGGGCCAGACAGCTGGCTGGGTGGGCTTGGGTATCACAAAT
SRR940959.10516563.1+ ATCATCACTTTTGAGCTCCAGGTCCAGACAGCTGGCTGGGTGGGCTTGGGTATCA
SRR940959.55516267.2+                   CAGGTCCAGACAGCTGGCTGGGTGGGCTTGGGTATCACAAAT
SRR940959.55885928.1+ ATCATCACTTTTGAGC
SRR940959.64659881.2- ATCATCACTTTTGAGCTCCAGGTCCAGACAGCTGGCTGGGTGGGCTTGGGTAT
SRR940959.69877817.2+                CTCCAGGTCCAGACAGCTGGCTGGGTGGGCTTGGGTATCACAAAT
SRR940959.126351496.- ATCATCACTTT
SRR940959.140480633.+ ATCATCACTTTTGAGCTCCAGGTCCAGACAGCTGGCTGGGTGGGCTTGGGTATCACAA
SRR940959.71054726.1+ ATCATCACTTTTGAGCTCCAGGTCCAGACA
SRR940959.170610408.+ ATCATCACTTTTGAGCTCCAGGTCCAGACA
SRR940959.175639215.+ ATCATCACTTTTGAGCTCCAGGTCCAGACAGCTGGCT
```

```
SRR940959.194971739.- ATCATCACTTTTGAGCTC
SRR940959.210586738.- ATCATCACTTTTGAGCTCCAGGTCCAGACAGCTGGCTGGGTGGGCTTGGGTATCACAAAT
SRR940959.215108946.- ATCATCACTTTTGAGCTCCAGGTCCAGACAGCTGGCTGGGTGGGCTTG
SRR940959.215290207.- ATCATCACTTTTGAGCTCCAGGTC
SRR940959.218346462.+ ATCATCACTTTTGAGCTCCAGGTCCAGACAGCTGGCTGGGTGGGCTTGGGTATCACAAAT
SRR940959.279165074.- ATCATCACTTTTGAGCTCCAGGTCCAGACAGCTGGCTGGGTGGGC
SRR940959.295442191.- ATCATCACTTTTGAGCTCCAGGTCCAGACAGCTGGCTGGGT
SRR940959.306237691.+ ATCATCACTTTTGAGCTCCAGGTCCAGACAGCTGGCTGGGTGGGCTTGG
SRR940959.325940630.- ATCATCACTTTTG
SRR940959.139341223.+                          AGACAGCTGGCTGGGTGGGCTTGGGTATCACAAAT
SRR940959.373034935.+                          AGACAGCTGGCTGGGTGGGCTTGGGTATCACAAAT
SRR940959.103693456.- ATCATCACTTTTGAGCTCCAGGTCCAGACAGCTGGCTGGGTGGGCTTGGGTATCACAAAT
SRR940959.379107729.- ATCATCACTTTTGAGCTCCAGGTCCAGACAGCTGGCTGGGTGGGCTTGGGTATCACAAAT
SRR940959.55013119.2- A
SRR940959.392191146.+ A
SRR940959.424879066.- ATCATCACTTTTGAGCTCCAGGTCCAGACAGCTGGCTGGGTGGGCTTGGGTATCACAAAT
SRR940959.471259936.+ ATCAT
SRR940959.479210167.- ATCATCACTTTTGAGCTCCAGGTCCAGACAGCTGGCTGGGTGGGCTTGGGTATCACAAAT
SRR940959.519497181.+       ACTTTTGAGCTCCAGGTCCAGACAGCTGGCTGGGTGGGCTTGGGTATCACAAAT
SRR940959.516081584.-                                      GGGTGGGCTTGGGTATCACAAAT
SRR940959.322824329.-                                          GGGCTTGGGTATCACAAAT
SRR940959.5845345.1+                                                TGGGTATCACAAAT
SRR940959.311658866.-                                                047GGGTATCACAAAT
SRR940959.502808763.-                                                NGGTATCACAAAT
SRR940959.282873597.-                                                  GTATCACAAAT
SRR940959.286897225.-                                                  GTATCACAAAT
SRR940959.127293354.-                                                      CACAAAT
SRR940959.160428733.+                                                      CACAAAT
SRR940959.172811999.+                                                         AAAT
SRR940959.284407326.+                                                           AT
SRR940959.467188273.+                                                           AT
                      ____________________________________________________________
consensus             ATCATCACTTTTGAGCTCCAGGTCCAGACAGCTGGCTGGGTGGGCTTGGGTATCACAAAT

                          .    :    .    :    .    :    .    :    .    :    .    :
SRR940959.113434796.- CGCTACCCCATGGTGGGAAGCAATCTGGTTGTT
SRR940959.522237424.+ CGCTACACCATGGGGGGAAGCAATCTGGTTGTTGGAGGAGTCTCGCCAGACGGCAATGTC
SRR940959.90883575.1- CGCTACACCATGGTGGGAAGCAATCTGGTTGTTGG
SRR940959.102275422.- CGCTACACCATGGTG
SRR940959.31698779.2- CGCTACACCATGGTGGGAAGCAATCTGGTTGT
SRR940959.281507910.- CGCTACACCATGGTGGGAAGCAATCTGGTTGTTGGAGGAGTCTCGCC
SRR940959.113877286.- CGCTACACCATGGTGGGAAGCAATC
SRR940959.50537691.1- CGCTACACCATGGTGGGAAGCAATCTGGTTGTT
SRR940959.55516267.2+ CGCTACACCATGGTGGGAAGCAATCTGGTTGTTGGAGGAGTCTCGCCAGACGGCAATG
SRR940959.69877817.2+ CGCTACACCATGGTGGGAAGCAATCTGGTTGTTGGAGGAGTCTCGCCAGACGGCA
SRR940959.210586738.- CGCTACACCATGGTGGGAAGCAATCTGGTTGT
SRR940959.218346462.+ CGCTACACCATGGTGGGAAGCAATCTG
SRR940959.139341223.+ CGCTACACCATGGTGGGAAGCAATCTGGTTGTTGGAGGAGTCTCGCCAGACGGCAATGTC
SRR940959.373034935.+ CGCTACACCATGGTGGGAAGCAATCTGGTTGTTGGAGGAGTCTCGCCAGACGGCAATGTC
SRR940959.103693456.- CGCTACACC
SRR940959.379107729.- CGCTACACC
SRR940959.424879066.- CGCTACACCATGGTGGGAAGCAATC
SRR940959.479210167.- CGCTACACCATGGTGGGAAGCAATCTGGTTGTT
SRR940959.519497181.+ CGCTACACCATGGTGGGAAGCAATCTGGTTGTTGGAGGAGTCTCGC
SRR940959.516081584.- CGCTACACCATGGTGGGAAGCAATCTGGTTGTTGGAGGAGTCTCGCCAGACGGCAATGTC
SRR940959.322824329.- CGCTACACCATGGTGGGAAGCAATCTGGTTGTTGGAGGAGTCTCGCCAGACGGCAATGTC
SRR940959.5845345.1+  CGCTACACCATGGTGGGAAGCAATCTGGTTGTTGGAGGAGTCTCGCCAGACGGCAATGTC
SRR940959.311658866.- CGCTACACCATGGTGGGAAGCAATCTGGTTGTTGGAGGAGTCTCGCCAGACGGCAATGTC
SRR940959.502808763.- CGCTACACCATGGTGGGAAGCAATCTGGTTGTTGGAGGAGTCTCGCCAGACGGCAATGTC
SRR940959.282873597.- CGCTACACCATGGTGGGAAGCAATCTGGTTGTTGGAGGAGTCTCGCCAGACGGCAATGTC
SRR940959.286897225.- CGCTACACCATGGTGGGAAGCAATCTGGTTGTTGGAGGAGTCTCGCCAGACGGCAATGTC
SRR940959.127293354.- CGCTACACCATGGTGGGAAGCAATCTGGTTGTTGGAGGAGTCTCGCCAGACGGCAATGTC
SRR940959.160428733.+ CGCTACACCATGGTGGGAAGCAATCTGGTTGTTGGAGGAGTCTCGCCAGACGGCAATGTC
SRR940959.172811999.+ CGCTACACCATGGGGGGAAGCAATCTGGTTGTTGGAGGAGTCTCGCCAGACGGCAATGTC
SRR940959.284407326.+ CGCTACACCATGGTGGGAAGCAATCTGGTTGTTGGAGGAGTCTCGCCAGACGGCAATGTC
SRR940959.467188273.+ CGCTACACCATGGAGGGAATGAGGCTGGTTGTTGGAGGAGTCTCGCCAGACGGCAATGTC
SRR940959.154383445.-     ACACCATGGTGGGAAGCAATCTGGTTGTTGGAGGAGTCTCGCCAGACGGCAATGTC
SRR940959.522424728.-     ACACCATGGTGGGAAGCAATCTGGTTGTTGGAGGAGTCTCGCCAGACGGCAATGTC
SRR940959.488444643.+      CACCATGGTGGGAAGCAATCTGGTTGTTGGAGGAGTCTCGCCAGACGGCAATGTC
SRR940959.32696534.1+          ATGGTGGGAAGCAATCTGGATGTTGGAGGAGTCTCGCCAGACGGCAATGTC
SRR940959.519277934.+          ATGGTGGGAAGCAATCTGGTTGTTGGAGGAGTCTCGCCAGACGGCAATGTC
SRR940959.73742648.1+                GGAAGCAATCTGGTTGTTGGAGGAGTCTCGCCAGACGGCAATGTC
SRR940959.520100745.+                GGAAGCAATCTGGTTGTTGGAGGAGTCTCGCCAGACGGCAATGTC
SRR940959.196771138.+                 GAAGCAATCTGGTTGTTGGAGGAGTCTCGCAAGACGGCAATGTC
SRR940959.276007480.+                  AAGCAATCTGGTTGTTGGAGGAGTCTCGCCAGACGGCAATGTC
SRR940959.428440826.+                  AAGCAATCTGGTTGTTGGAGAAGTCTCGCCAGACGGCAATGTC
SRR940959.434558517.+                       ATCTGGTTGTTGGAGGAGTCTCGCCAGACGGCAATGTC
```

```
SRR940959.428135557.+                        TCTGGTTGTTGGAGGAGTCTCGCCAGACGGCAATGTC
SRR940959.10158707.1+                              GTTGGAGGAGTCTCGCCAGACGGCAATGTC
SRR940959.364685701.+                              GTTGGAGGAGTCTCGCCAGACGGCAATGTC
SRR940959.73891225.2+                                 GGAGGAGTCTCGCCAGACGGCAATGTC
SRR940959.274263848.+                                  GAGGAGTCTCGCCAGACGGCAATGTC
SRR940959.434097628.+                                            GCCAGACGGCAATGTC
SRR940959.503522536.+                                            GCCAGACGGCAATGTC
SRR940959.400686013.-                                                 ACGGCAATGTC
                      ____________________________________________________________
consensus             CGCTACACCATGGTGGGAAGCAATCTGGTTGTTGGAGGAGTCTCGCCAGACGGCAATGTC

                          .    :    .    :    .    :    .    :    .    :    .    :
SRR940959.522237424.+ TATTT
SRR940959.139341223.+ TATTT
SRR940959.373034935.+ TATTT
SRR940959.516081584.- TATTTCTCGGTGAGTAT
SRR940959.322824329.- TATTTCTCGGTGAGTATGAGG
SRR940959.5845345.1+  TATTTCTCGGTGAGTATGAGGGTGAC
SRR940959.311658866.- TATTTCTCGGTGAGTATGAGGGTGACT
SRR940959.502808763.- TATTTCTCGGTGAGTATGAGGGTGACT
SRR940959.282873597.- TATTTCTCGGTGAGTATGAGGGTGACTCC
SRR940959.286897225.- TATTTCTCGGTGAGTATGAGGGTGACTCC
SRR940959.127293354.- TATTTCTCGGTGAGTATGAGGGTGACTCCCTCC
SRR940959.160428733.+ TATTTCTCGGTGAGTATGAGGGTGACTCCCTCC
SRR940959.172811999.+ TATTTCTCGGTGAGTATGAGGGTGACTCCCTCCCCA
SRR940959.284407326.+ TATTTCTCGGTGAGTATGAGGGTGACTCCCTCCCAAGC
SRR940959.467188273.+ TATTTCTCGGTGAGTATGAGGGGGACTCCCTCCCAAGC
SRR940959.154383445.- TATTTCTCGGTGAGTATGAGGGTGACTCCCTCCCAAGCGTGAGG
SRR940959.522424728.- TATTTCTCGGTGAGTATGAGGGTGACTCCCTCCCAAGCGTGAGG
SRR940959.488444643.+ TATTTCTCGGTGAGTATGAGGGTGACTCCCTCCCAAGCGTGAGGG
SRR940959.32696534.1+ TATTTCTCGGTGAGTATGAGGGAGACTCCCTCCCAAGCGTGAGGGGCTC
SRR940959.519277934.+ TATTTCTCGGTGAGTATGAGGGTGACTCCCTCCCAAGCGTGAGGGTCTC
SRR940959.73742648.1+ TATTTCTCGGTGAGTATGAGGGTGACTCCCTCCCAAGCGTGAGGGTCTCTTGCTG
SRR940959.520100745.+ TATTTCTCGGTGAGTATGAGGGTGACTCCCTCCCAAGCGTGAGGGTCTCTTGCTG
SRR940959.196771138.+ TATTTCTCGGTGAGTATGAGGGTGACTCCCTCCCAAGCGTGAGGGTCTCTTGCTGG
SRR940959.276007480.+ TATTTCTCGGTGAGTATGAGGGTGACTCCCTCCCAAGCGTGAGGGTCTCTTGCTGGG
SRR940959.428440826.+ TATTTCTCGGTGAGTATGAGGGTGACTCCCTCCCAAGCGTGAGGGTCTCTTGCTGGG
SRR940959.434558517.+ TATTTCTCGGTGAGTATGAGGGTGACTCCCTCCCAAGCGTGAGGGTCTCTTGCTGGGTGG
SRR940959.428135557.+ TATTTCTCGGTGAGTATGAGGGTGACTCCCTCCCAAGCGTGAGGGTCTCTTGCTGGGTGG
SRR940959.10158707.1+ TATTTCTCGGTGAGTATGAGGGTGACTCCCTCCCAAGCGTGAGGGTCTCTTGCTGGGTGG
SRR940959.364685701.+ TATTTCTCGGTGAGTATGAGGGTGACTCCCTCCCAAGCGTGAGGGTCTCTTGCTGGGTGG
SRR940959.73891225.2+ TATTTCTCGGTGAGTATGAGGGTGACTCCCTCCCAAGCGTGAGGGTCTCTTGCTGGGTGG
SRR940959.274263848.+ TATTTCTCGGTGAGTATGAGGGTGACTCCCTCCCAAGCGTGAGGGTCTCTTGCTGGGTGG
SRR940959.434097628.+ TATTTCTCGGTGAGTATGAGGGTGACTCCCTCCCAAGCGTGAGGGTCTCTTGCTGGGTGG
SRR940959.503522536.+ TATTTCTCGGTGAGTATGAGGGTGACTCCCTCCCAAGCGTGAGGGTCTCTTGCTGGGTGG
SRR940959.400686013.- TATTTCTCGGTGAGTATGAGGGTGACTCCCTCCCAAGCGTGAGGGTCTCTTGCTGGGTGG
                      ____________________________________________________________
consensus             TATTTCTCGGTGAGTATGAGGGTGACTCCCTCCCAAGCGTGAGGGTCTCTTGCTGGGTGG

                          .    :    .    :    .    :    .    :    .    :    .    :
SRR940959.434558517.+ AG
SRR940959.428135557.+ AGG
SRR940959.10158707.1+ AGGGTGGCTC
SRR940959.364685701.+ AGGGTGGCTC
SRR940959.73891225.2+ AGGGTGGCTCTGA
SRR940959.274263848.+ AGGGTGGCTCTGAG
SRR940959.434097628.+ AGGGTGGCTCTGAGTCCAGGAAGG
SRR940959.503522536.+ AGGGTGGCTCTGAGTCCAGGAAGN
SRR940959.400686013.- AGGGTGGCTCTGAGTCCAGGAAGGATCCG
                      ____________________________________________________________
consensus             AGGGTGGCTCTGAGTCCAGGAAGGATCCG
```

(2) The exon 2 sequence was predicted by assembling WGS data using CAP3. The exon 2 is on the reverse strand and marked in red.

```
                          .    :    .    :    .    :    .    :    .    :    .    :
SRR940959.251581775.- TCCAGGTTGGGGGTGACTTCACCTCCTAGAAGGAAGGCTGCTTGTGAGCAAATGCTCCTC
SRR940959.511588247.+    AGGTTGGGGGTGACTTCACCTCCTAGAAGGAAGGCTGCTTGTGAGCAAATGCTCCTC
SRR940959.481748416.-       TTGGGGGTGACTTCACCTCCTAGAAGGAAGGCTGCTTGTGAGCAAATGCTCCTC
SRR940959.465559123.+        TGGGGGTGACTTCACCTCCTAGAAGGAAGGCTGCTTGTGAGCAAATGCTCCTC
SRR940959.121243455.+                ACTTCACCTCCTAGAAGGAAGGCTGCTTGTGAGCAAATGCTCCTC
SRR940959.377364060.+                ACTTCACCTCCTAGAAGGAAGGCTGCTTGTGAGCAAATGCTCCTC
SRR940959.427680724.+                ACTTCACCTCCTAGAAGGAAGGCTGCTTGTGAGCAAATGCTCCTC
SRR940959.508166988.-                   NCACCTCCTAGAAGGAAGGCTGCTTGTGAGCAAATGCTCCTC
SRR940959.458044493.-                      CCTCCTAGAAGGAAGGCTGCTTGTGAGCAAATGCTCCTC
SRR940959.46098962.1-                        TCCTAGAAGGAAGGCTGCTTGTGAGCAAATGCTCCTC
```

```
SRR940959.456726301.+                         CCTAGAAGGAAGGCTGCTTGTGAGCAAATGCTCCTC
SRR940959.371005678.+                              AAGGAAGGCTGCTTGTGAGCAAATGCTCCTC
SRR940959.293262198.-                                 GAAGGCTGCTTGTGAGCAAATGCTCCTC
SRR940959.476882791.-                                 GAAGGCTGCTTGTGAGCAAATGCTCCTC
SRR940959.136881322.-                                  AAGGCTGCTTGTGAGCAAATGCTCCTC
SRR940959.478208654.-                                   AGGCTGCTTGTGAGCAAATGCTCCTC
SRR940959.71805665.1+                                    GGCTGCTTGTGAGCAAATGCTCCTC
SRR940959.201735635.-                                       TGCTTGTGAGCAAATGCTCCTC
SRR940959.81653987.2-                                         CTTGTGAGCAAATTCTCCTC
SRR940959.173459484.+                                            GTGAGCAAATGCTCCTC
SRR940959.259049245.+                                                      GCTCCTC
SRR940959.322590032.-                                                            C
                      ____________________________________________________________
consensus             TCCAGGTTGGGGGTGACTTCACCTCCTAGAAGGAAGGCTGCTTGTGAGCAAATGCTCCTC

                          .    :    .    :    .    :    .    :    .    :    .    :
SRR940959.251581775.- TCTCCTCCCTCTTTTACCGTGATGTCTTGGTCTTGAGGGT
SRR940959.511588247.+ TCTCCTCCCTCTTTTACCGTGATGTCTTGGTCTTGAGGGTCGN
SRR940959.481748416.- TCTCCTCCCTCTTTTACCGTGATGTCTTGGTCTTGAGGGTCGCAGG
SRR940959.465559123.+ TCTCCTCCCTCTTTTACCGTGATGTCTTGGTCTTGAGGGTCGCAGGA
SRR940959.121243455.+ TCTCCTCCCTCTTTTACCGTGATGTCTTGGTCTTGAGGGTCGCAGGAGCGGAAGG
SRR940959.377364060.+ TCTCCTCCCTCTTTTACCGTGATGTCTTGGTCTTGAGGGTCGCAGGAGCGGAAGG
SRR940959.427680724.+ TCTCCTCCCTCTTTTACCGTGATGTCTTGGTCTTGAGGGTCGCAGGAGCGGAAGG
SRR940959.508166988.- TCTCCTCCCTCTTTTACCGTGATGTCTTGGTCTTGAGGGTCGCAGGAGCGGAAGGGCC
SRR940959.458044493.- TCTCCTCCCTCTTTTACCGTGATGTCTTGGTCTTGAGGGTCGCAGGAGCGGAAGGGCCTG
SRR940959.46098962.1- TCTCCTCCCTCTTTTACCGTGATGTCTTGGTCTTGAGGGTCGCAGGAGCGGAAGGGCCTG
SRR940959.456726301.+ TCTCCTCCCTCTTTTACCGTGATGTCTTGGTCTTGAGGGTCGCAGGAGCGGAAGGGCCTG
SRR940959.371005678.+ TCTCCTCCCTCTTTTACCGTGATGTCTTGGTCTTGAGGGTCGCAGGAGCGGAAGGGCCTG
SRR940959.293262198.- TCTCCTCCCTCTTTTACCGTGATGTCTTGGTCTTGAGGGTCGCAGGAGCGGAAGGGCCTG
SRR940959.476882791.- TCTCCTCCCTCTTTTACCGTGATGTCTTGGTCTTGAGGGTCGCAGGAGCGGAAGGGCCTG
SRR940959.136881322.- TCTCCTCCCTCTTTTACCTTGATGTCTTGGTCTTGAGGGTCGCAGGAGCGGAAGGGCCTG
SRR940959.478208654.- TCTCCTCCCTCTTTTACCGTGATGTCTTGGTCTTGAGGGTCGCAGGAGCGGAAGGGCCTG
SRR940959.71805665.1+ TCTCCTCCCTCTTTTACCGTGATGTCTTGGTCTTGAGGGTCGCAGGAGCGGAAGGGCCTG
SRR940959.201735635.- TCTCCTCCCTCTTTTACCGTGATGTCTTGGTCTTGAGGGTCGCAGGAGCGGAAGGGCCTG
SRR940959.81653987.2- GCTCCTCCCTCTTTTACCGTGATGTCTTGGTCTTGAGGTTCGCAGGAGCGGAAGGGCCTG
SRR940959.173459484.+ TCTCCTCCCTCTTTTACCGTGATGTCTTGGTCTTGAGGGTCGCAGGAGCGGAAGGGCCTG
SRR940959.259049245.+ TCTCCTCCCTCTTTTACCGTGATGTCTTGGTCTTGAGGGTCGCAGGAGCGGAAGGGCCTG
SRR940959.322590032.- TCTCCTCCCTCTTTTACCGTGATGTCTTGGTCTTGAGGGTCGCAGGAGCGGAAGGGCCTG
SRR940959.200200646.+   TCCTCCCTCTTTTACCGTGATGTCTTGGTCTTGAGGGTCGCAGGAGCGGAAGGGCCTG
SRR940959.427880313.-         CTCTTTTACCGTGATGTCTTGGTCTTGAGGGTCGCAGGAGCGGAAGGGCCTG
SRR940959.54247504.1+           CTTTTACCGTGATGTCTTGGTCTTGAGGGTCGCAGGAGCGGAAGGGCCTG
SRR940959.473464341.+              TTACCGTGATGTCTTGGTCTTGAGGGTCGCAGGAGCGGAAGGGCCTG
SRR940959.111152098.-                          CTTGGTCTTGAGGGTCGCAGGAGCGGAAGGNCCTG
SRR940959.254124452.-                             GGTCTTGAGGGTCGCAGGAGCGGAAGGGCCTG
SRR940959.384606631.-                                              GAGCGGAAGGGCCTG
SRR940959.82379381.2+                                                    AAGGGCCTG
SRR940959.506564819.-                         NCTTGGTCTTGAGGGTCGCAGGAGCGGAAGGGCCTG
SRR940959.230848247.+                         TCTTGGTCTTGAGGGTCGCAGGAGCGGAAGGGCCTG
SRR940959.282022586.+                                  TGAGGGTCGCAGGAGCGGAAGGGCCTG
SRR940959.306461692.-                  CGTGATGTCTTGGTCTTGAGGGTCGCAGGAGCGGAAGGGCCTG
SRR940959.319442866.-                  CGTGATGTCTTGGTCTTGAGGGTCGCAGGAGCGGAAGGGCCTG
SRR940959.322549078.+                     GATGTCTTGGTCTTGAGGGTCGCAGGAGCGGAAGGGCCTG
                      ____________________________________________________________
consensus             TCTCCTCCCTCTTTTACCGTGATGTCTTGGTCTTGAGGGTCGCAGGAGCGGAAGGGCCTG

                          .    :    .    :    .    :    .    :    .    :    .    :
SRR940959.458044493.- G
SRR940959.46098962.1- GAG
SRR940959.456726301.+ GAGG
SRR940959.371005678.+ GAGGAGCGC
SRR940959.293262198.- GAGGAGCGCATG
SRR940959.476882791.- GAGGAGCGCATG
SRR940959.136881322.- GAGGAGCGCATGG
SRR940959.478208654.- GAGGAGCGCATGGT
SRR940959.71805665.1+ GAGGAGCGCATGGGG
SRR940959.201735635.- GAGGAGCGCATGGTGGTA
SRR940959.81653987.2- GAGGAGCGCATGGTGGTATA
SRR940959.173459484.+ GAGGAGCGCATGGTGGTATAGAT
SRR940959.259049245.+ GAGGAGCGCATGGTGGTATAGATGGTGTCTTCT
SRR940959.322590032.- GAGGAGCGCATGGTGGTATAGATGGTGTCTTCTGTCAGT
SRR940959.200200646.+ GAGGAGCGCATGGTGGTATAGATGGTGTCTTCTGTCAGTGCC
SRR940959.427880313.- GAGGAGCGCATGGTGGTATAGATGGTGTCTTCTGTCAGTGCCAGCAGC
SRR940959.54247504.1+ GAGGAGCGCATGGTGGTATAGATGGTGTCTTCTGTCAGTGCCAGCAGCTC
SRR940959.473464341.+ GAGGAGCGCATGGTGGTATAGATGGAGTCTTCTGTCAGTGCCAGCAGCTCTGC
SRR940959.111152098.- GAGGAGCGCATGGTNGTATAGNTGGTGTCTTCTGTCAGNGCCAGCAGCTCTGCGTCCTGG
SRR940959.254124452.- GAGGAGCACATGGTGGTATAGATGGTGTCTTCTGTCATTGCCAGCAGCTCTGCGTCCTGG
SRR940959.384606631.- GAGGAGCGCATGGTGGTATAGATGGTGTCTTCTATCAGTGCCAGCAGCTCTCCGTCCTGG
```

```
SRR940959.82379381.2+ GAGGAGCGCATGGTGGTATAGATGGTGTCTTCTGTCAGTGCCAGCAGCTCTGCGTCCTGG
SRR940959.506564819.- GAGGAGCGCATGGTGGTATAGATGGTGTCTTCTGTCAGTGCCAGCAGCTCTGCGTCCTGG
SRR940959.230848247.+ GAGGAGCGCATGGTGGTATAGATGGTGTCTTCTGTCAGTGCCAGCAGCTCTGCGTCCTGG
SRR940959.282022586.+ GAGGAGCGCATGGTGGTATAGATGGTGTCTTCTGTCAGTGCCAGCAGCTCTGCGTCCTGG
SRR940959.306461692.- GAGGAGCGCATGGTGGTATAGATGGTGTCTTCTGTCAGTGCCAGCAGCTCTGCGTCC
SRR940959.319442866.- GAGGAGCGCATGGTGGTATAGATGGTGTCTTCTGTCAGTGCCAGCAGCTCTGCGTCC
SRR940959.322549078.+ GAGGAGCGCATGGTGGTATAGATGGTGTCTTCTGTCAGTGCCAGCAGCTCTGCGTCCTGG
SRR940959.92447514.1+  AGGAGCGCATGGTGGTATAGATGGTGTCTTCTGTCAGTGCCAGCAGCTCTNCGTCCTGG
SRR940959.217685635.+  AGGAGCGCATGGTGGTATAGATGGTGTCTTCTGTCAGTGCCAGCAGCTCTGCGTCCTGG
SRR940959.377364060.-  AGGAGCGCATGGTGGTATAGATGGTGTCTTCTGTCAGTGCCAGCAGCTCTGCGTCCTGG
SRR940959.13744980.2+    GAGCGCATGGTGGTATAGATGGTGTCTTCTGTCAGTGCCAGCAGCTCTGCGTCCTGG
SRR940959.329302939.+        GCATGGTGGTATAGATGGTGTCTTCTGTCAGTGCCAGCAGCTCTGCGTCCTGG
SRR940959.297603424.+         CATGGTGGTATAGATGGTGTCTTCTGTCAGTGCCAGCAGCTCTGCGTCCTGG
SRR940959.342885464.+         CATGGTGGTATAGATGGTGTCTTCTGTCAGTGCCAGCAGCTCTGCGTCCTGG
SRR940959.143875321.-            GGTGGTATAGATGGTGTCTTCTGTCAGTGCCAGCAGCTCTGCGTCCTGG
SRR940959.98418824.1+             GTGGTATAGATGGTTTCTTCTGTCAGTGCCAGCAGCTCTGCGTCCTGG
SRR940959.262307116.+             GTGGTANAGATGGTGTCTTCTGTCAGTGCCTGCAGCTCTGCGTCCTGG
SRR940959.313770949.+                    AGATGGTGTCTTCTGTCAGTGCCAGCAGCTCTGCGTCCTGG
SRR940959.406064376.-                       TGGTGTCTTCTGTCAGTTCCAGCAGCTCTGCGTCCTGG
SRR940959.411867337.-                       TGGTGTCTTCTGTCAGTGCCAGCAGCTCTGCGTCCTGG
SRR940959.414051138.-                       TGGTGTCTTCTGTCAGTGCCAGCAGCTCTGCGTCCTGG
SRR940959.93816380.2+                            TCTTCTGTCAGTGCCAGCAACTCTGCGTCCTGG
SRR940959.388370099.+                                CTGTCAGTGCCAGCAGCTCTGCGTCCTGG
SRR940959.26060530.1-                                  GTCAGTGCCAGCAGCTCTGCGTCCTGG
SRR940959.53804324.2+                                  GTCAGTGCCAGCAGCTCTGCGTCCTGG
SRR940959.347591718.+                                                 ATATTTTAATGG
SRR940959.1742506.1-                                                            GG
                      ____________________________________________________________
consensus             GAGGAGCGCATGGTGGTATAGATGGTGTCTTCTGTCAGTGCCAGCAGCTCTGCGTCCTGG

                          .    :    .    :    .    :    .    :    .    :    .    :
SRR940959.111152098.- CTCCC
SRR940959.254124452.- CNCCCTCC
SRR940959.384606631.- CTCCCTCCCCCAGAGCGTCTTCATC
SRR940959.82379381.2+ CTCCCTCCCCAAGAGCGTCTTCATCCACCAG
SRR940959.506564819.- CTCC
SRR940959.230848247.+ CTCC
SRR940959.282022586.+ CTCCCTCCCCCAG
SRR940959.92447514.1+ CTCCCTCCCCCAGAGCGTCTTCATCCACCAGGTGCTGATTC
SRR940959.217685635.+ CTCCCTCCCCCAGAGCGTCTTCATCCACCAGGTGCTGATTC
SRR940959.377364060.- CTCCCTCCCCCAGAGCGTCTTCATCCACCAGGTGCTGATTC
SRR940959.13744980.2+ CTCCCTCCCCCAGAGCGTCTTCATCCACCAGGTGCTGATTCTA
SRR940959.329302939.+ CTCCCTCCCCCAGAGCGTCTTCATCCACCAGGTGCTGATTCTAGGGT
SRR940959.297603424.+ CTCCCTCCCCCAGAGCGTCTTCATCCACCAGGTGCTGATTCTAGGGTC
SRR940959.342885464.+ CTCCCTCCCCCAGAGCGTCTTCATCCACCAGGTGCTGATTCTAGGGTC
SRR940959.143875321.- CTCCCTCCCCCAGAGCGTCTTCATCCACCAGGTGCTGATTCTAGGGTCCAG
SRR940959.98418824.1+ CTNCCTACCCCAGAGCGTCTTCATCCACCAGGTGCTGATTCTAGGGTCCCGG
SRR940959.262307116.+ CTCCCTCCCCCAGAGCGTCTTCATCCACCAGGTGCTGATTCTAGGGTCCAGG
SRR940959.313770949.+ CTCCCTCCCCCAGAGCGTCTTCATCCACCAGGTGCTGATTCTAGGGCCCAGGAAAGGTG
SRR940959.406064376.- CTCCATCCCCCAGCGCGTCTTCATCCACCAGGTGCTGATTCTAGGGTCCAGGAAAGGTGA
SRR940959.411867337.- CTCCCTCCCCCAGAGCGTCTCCATCCACCAGGTGCTGATTCTAGGGTCCAGGAAAGGTGA
SRR940959.414051138.- CTCCCTCCCCCAGAGCGTCTACATCCACCAGGTGCTGATTCTAGGGTCCAGGAAAGGTGA
SRR940959.93816380.2+ CTCCCTCCCCCAGAGCGTCTTCATCCACCAGGTGCTGATTCTAGGGTCCATGAAAGGTGA
SRR940959.388370099.+ CTCCCTCCCCCAGAGCGTCTTCATCCACCAGGTGCTGATTCTAGGGTCCAGGAAAGGTGA
SRR940959.26060530.1- CTCCCTCCCCCAGAGCGTCTTCATCCACCAGGTGCTGATTCTAGGGTCCAGGAAAGGTGA
SRR940959.53804324.2+ CTCCCTCCCCCAGAGCGTCTTCATCCACCAGGTGCTGATTCTAGGGTCCAGGAAAGGTGA
SRR940959.347591718.+ CTCCCTCCCCCAGAGCGTCTTCATCCACCCGGTGCTGATTCTAGGGTCCAGGAAAGGTGA
SRR940959.1742506.1-  CTCCCTCCCCCAGAGCGTCTTCATCCACCAGGTGCTGATTCTAGGGTCCAGGAAAGGTGA
SRR940959.19476267.1-        CCCCAGAGCGTCTTCATCCACCAGGTGCTGATTCTAGGGTCCAGGAAAGGTGA
SRR940959.456124176.-        CCCCAGAGCGTCTTCATCCACCAGGTGCTGATTCTAGGGTCCAGGAAAGGTGA
SRR940959.137321930.+           CAGAGCGTCTTCATCCACCAGGTGCTGATTCTAGGGTCCAGGAAAGGTGA
SRR940959.519998680.+           CAGAGCGTCTTCATCCACCAGGTGCTGATTCTAGGGTCCAGGAAAGGTGA
SRR940959.63223076.2-             GAGCGTCTTCATCCACCAGGTGCTGATTCTAGGGTCCAGGAAAGGTGA
SRR940959.322549078.-                  TCTTCATCCACCAGGTGCTGATTCTAGGGTCCAGGAAAGGTGA
SRR940959.202613668.-                   CTTCATCCACCAGGTGCTGATTCTAGGGTCCAGGAAAGGTGA
SRR940959.291093113.+                   CTTCATCCACCAGGTGCTGATTCTAGGGTCCAGGAAAGGTGA
                      ____________________________________________________________
consensus             CTCCCTCCCCCAGAGCGTCTTCATCCACCAGGTGCTGATTCTAGGGTCCAGGAAAGGTGA

                          .    :    .    :    .    :    .    :    .    :    .    :
SRR940959.406064376.- AG
SRR940959.411867337.- AG
SRR940959.414051138.- AG
SRR940959.93816380.2+ AGGGGTA
SRR940959.388370099.+ AGGGGTAAGAA
SRR940959.26060530.1- AGGGGTAAGAACC
SRR940959.53804324.2+ AGGGGGAAGAACC
```

```
SRR940959.347591718.+ AGGGGGAAGAACCAGGAGCCCTACGTGG
SRR940959.1742506.1-  AGGGGTAAGAACCAGGAGCCCTACGTGGTCACGGATTC
SRR940959.19476267.1- AGGGGTAAGAACCAGGAGCCCTACGTGGTCACGGATTCAGAGTAGTC
SRR940959.456124176.- AGGGGTAAGAACCAGGAGCCCTACGTGGTCACGGATTCAGAGTAGTC
SRR940959.137321930.+ AGGGTTAAGAACCAGGAGCCCTACGTGGTCACGGATTCAGAGTAGTCTGG
SRR940959.519998680.+ AGGGGTAAGAACCAGGAGCCCTACGTGGTCACGGATTCAGAGTAGTCTGG
SRR940959.63223076.2- AGGGGTAAGAACCAGGAGCCCTACGTGGTCACGGATTCAGAGTAGTCTGGGT
SRR940959.322549078.- AGGGGTAAGAACCAGGAGCCCTACGTGGTCACGGATTCAGAGTAGTCTGGGTCCATT
SRR940959.202613668.- AGGGGTAAGAACCAGGAGCCCTACGTGGTCACGGATTCAGAGTAGTCTGGGTCCATTT
SRR940959.291093113.+ AGGGGTAAGAACCAGGAGCCCTACGTGGTCACGGATTCAGAGTAGTCTGGGTCCATTT

consensus             AGGGGTAAGAACCAGGAGCCCTACGTGGTCACGGATTCAGAGTAGTCTGGGTCCATTT
```

(3) The exon 3 sequence was predicted by assembling WGS data using CAP3. The exon 3 is on the reverse strand and marked in red.

```
                          .    :    .    :    .    :    .    :    .    :    .    :
SRR940959.314702271.+ ATGTTGGGTGCAAGAGGAAGGTCTGGATTGAGAGGAAGGTGGGCAGTGGCTGGAGGCGGC
SRR940959.133527941.-                 GAAGGTCTGGATTGAGAGGAAGGTGGGCAGTGGCTGGAGGCGGC
SRR940959.157814344.-                 GAAGGTCTGGATTGAGAGGAAGGTGGGCAGTGGCTGGAGGCGGC
SRR940959.155309265.+                 GAAGGTCTGGATTGAGAGGAAGGTGGGCAGTGGCTGGAGGCGGC
SRR940959.354439360.-                 GAAGGTCTGGATTGAGAGGAAGGTGGGCAGTGGCTGGAGGCGGC
SRR940959.240073267.-                  AAGGTCTGGATTGAGAGGAAGGTGGGCAGTGGCTGGAGGCGGC
SRR940959.378097191.-                    GGTCTGGATTGAGAGGAAGGTGGGCAGTGGCTGGAGGCGGC
SRR940959.319342500.+                      TCTGGATTGAGAGGAAGGTGGGCAGTGGCTGGAGGCGGC
SRR940959.355657346.+                      TCTGGATTGAGAGGAAGGTGGGCAGTGGCTGGAGGCGGC
SRR940959.220672047.-                       CTGGATTGAGAGGAAGGTGGGCAGTGGCTGGAGGCGGC
SRR940959.483580262.+                           ATTGAGAGGAAGGTGGGCAGTGGCTGGAGGCGGC
SRR940959.52352767.2-                             TGAGAGGAAGGTGGGCAGTGGCTGGAGGCGGC
SRR940959.392121618.+                               AGAGGAAGGTGGGCAGTGGCTGGAGGCGGC
SRR940959.486098496.+                               AGAGGAAGGTGGGCAGTGGCTGGAGGCGGC
SRR940959.517846464.+                                GAGGAAGGTGGGCAGTGGCTGGAGGCGGC
SRR940959.463902652.-                                  GGAAGGTGGGCAGTGGCTGGAGGCGGC

consensus             ATGTTGGGTGCAAGAGGAAGGTCTGGATTGAGAGGAAGGTGGGCAGTGGCTGGAGGCGGC

                          .    :    .    :    .    :    .    :    .    :    .    :
SRR940959.314702271.+ TGGGTGGGGGGGAGGGACCTCACATCAGTGATCTCCAAGG
SRR940959.133527941.- TGGGTGGGGAGGAGGGACCTCACATCAGTGATCTCCAAGTCATGGACGATGGTGTC
SRR940959.157814344.- TGGGTGGGGAGGAGGGACCTCACATCAGTGATCTCCAAGTCATGGACGATGGTGTC
SRR940959.155309265.+ TGGGTGGGGAGGAGGGACCTCACATCAGTGATCTCCAAGTCATGGACGATGGTGTC
SRR940959.354439360.- TGGGTGGGGAGGAGGGACCTCACATCAGTGATCTCCAAGTCATGGACGATGGTGTC
SRR940959.240073267.- TGGGTGGGGAGGAGGGACCTCACATCAGTGATCTCCAAGTCATGGACGATGGTGTCC
SRR940959.378097191.- TGGGTGGGGAGGAGGGACCTCACATCAGTGATCTCCAAGTCATGGACGATGGTGTCCTC
SRR940959.319342500.+ TGGGTGGGGAGGAGGGACCTCACATCAGTGATCTCCAAGTCATGGACGATGGTGTCCTCG
SRR940959.355657346.+ TGGGGGGGGAGGAGGGACCTCACATCAGTGATCTCCAAGTCATGGACGATGGTGTCCTCG
SRR940959.220672047.- TGGGTGGGGAGGAGGGACCTCACATCAGTGATCTCCAAGTCATGGACGATGGTGTCCTCG
SRR940959.483580262.+ TGGGTGGGGAGGAGGGACCTCACATCAGTGATCTCCAAGTCATGGACGATGGTGGCCTCG
SRR940959.52352767.2- TGGGTGGGGAGGAGGGACCTCACATCAGTGATCTCCAAGTCATGGACGATGGTGTCCTCG
SRR940959.392121618.+ TGGGTGGGGAGGAGGGACCTCACATCAGTGATCTCCAAGTCATGGACGATGGTGTCCTCG
SRR940959.486098496.+ TGGGTGGGGAGGAGGGACCTCACATCAGTGATCTCCAAGTCATGGACGATGGTGTCCTCG
SRR940959.517846464.+ TGGGTGGGGAGGAGGGACCTCACATCAGTGATCTCCAAGTCATGGACGATGGTGTCCTCG
SRR940959.463902652.- TGGGTGGGGAGGAGGGACCTCACATCAGTGATCTCCAAGTCATGGACGATGGTGTCCTCG
SRR940959.319342500.-           GGAGGGACCTCACATCAGTGATCTCCAAGTCATGGACGATGGTGTCCTCG
SRR940959.355657346.-           GGAGGGACCTCACATCAGTGATCTCCAAGTCATGGACGATGGTGTCCTCG
SRR940959.191547556.-                                                            G

consensus             TGGGTGGGGAGGAGGGACCTCACATCAGTGATCTCCAAGTCATGGACGATGGTGTCCTCG

                          .    :    .    :    .    :    .    :    .    :    .    :
SRR940959.319342500.+ G
SRR940959.355657346.+ G
SRR940959.220672047.- GG
SRR940959.483580262.+ GGGACA
SRR940959.52352767.2- GGGACATC
SRR940959.392121618.+ GGGACATCGA
SRR940959.486098496.+ GGGACATCGA
SRR940959.517846464.+ GGGACATCGAG
SRR940959.463902652.- GGGACATCGAGAT
SRR940959.319342500.- GGGACATCGAGATCATCGGGGTGGACAATGTGGAGCGGGAAGATGGACTC
SRR940959.355657346.- GGGACATCGAGATCATCGGGGTGGACAATGTGGAGCGGGAAGATGGACTC
SRR940959.503064837.+                    GGTGGACAATGTGGAGNGGGAAGATGGACTCACNAAAGTAC
SRR940959.174571703.+                   GGGTGGACAATGTGGAGCGGGAAGATGGACTCACAAAAGTAC
SRR940959.212347108.+                      TGGACAATGTGGAGCGGGAAGATGGACTCACAAAAGTAC
SRR940959.492005375.-                         ACAATGTGGAGCGGGAAGATGGACTCACAAAAGTAC
SRR940959.14118139.2+                       GGACAATGTGGAGCGGGAAGATGGACTCACAAAAGTAC
```

```
SRR940959.154816229.+ GGGACATCGAGATCATCGGGGTGGACAATGTGGAGCGGGAAGATGGACTCACAAAAGTAC
SRR940959.173018166.+                    GGTGGACAATGTGGAGCGGGAAGATGGACTCACAAAAGTAC
SRR940959.191547556.- GGGACATCGAGATCATCGGGGTGGACAATGTGGAGCGGGAAGATGGACTCACAAAAGTAC
SRR940959.227114799.+                  GGGGTGGACAATGTGGAGCGGGAAGATGGACTCACAAAAGTAC
SRR940959.209329369.+     CATCGAGATCATCGGGGTGGACAATGTGGAGCGGGAAGATGGACTCACAAAAGTAC
SRR940959.396573641.+     CATCGAGATCATCGGGGTGGACAATGTGGAGCGGGAAGATGGACTCACAAAAGTAC
SRR940959.453934536.+        CGAGATCATCGGGGTGGACAATGTGGAGCGGGAAGATGGACTCACAAAAGTAC
SRR940959.369825616.+                      TGGACAATGTGGAGCGGGAAGATGGACTCACAAAAGTAC
SRR940959.463156140.+                      TGGACAATGTGGAGCGGGAAGATGGACTCACAAAAGTAC
SRR940959.519717962.+   GACATCGAGATCATCGGGGTGGACAATGTGGAGCGGGAAGATGGACTCACAAAAGTAC
SRR940959.388193322.+                                      GGAAGATGGACTCACAAAAGTAC
SRR940959.479070874.-                                                  CACAAAAGTAC
SRR940959.194460169.+                                                     AAAAGTAC
SRR940959.283687311.+                                                     AAAAGTAC
                      ____________________________________________________________
consensus             GGGACATCGAGATCATCGGGGTGGACAATGTGGAGCGGGAAGATGGACTCACAAAAGTAC

                          .    :    .    :    .    :    .    :    .    :    .    :
SRR940959.503064837.+ GCTCCCGATCCAGCTTCAGAGTGTCATCTGGGCCGTAGGTGGCAAGCACCCTCACAGTG
SRR940959.174571703.+ GCTCCCGATCCAGCTTCAGAGTGTCATCTGGGCCGTAGGGGGGCAAGCACCCTCACAGT
SRR940959.212347108.+ GCTCCCGATCCAGCTTCAGAGGGTCATCTGGGCCGTAGGTGGCAAGCACCCTCACAGTGT
SRR940959.492005375.- GCTCCCGATCCAGCTTCAGAGTGTCATCTGGGCCGTAGGTGGCAAGCACCCTCACATTGT
SRR940959.14118139.2+ GCTCCCGATCCAGCTTCAGAGTGTCATCTGGGCCGTAGGTGGCAAGCACCCTCACAGTGT
SRR940959.154816229.+ GCTCCCGATCCAGCTTCAGAGTGTCATCTGGGCCGTAGGT
SRR940959.173018166.+ GCTCCCGATCCAGCTTCAGAGTGTCATCTGGGCCGTAGGTGGCAAGCACCCTCACAGTG
SRR940959.191547556.- GCTCCCGATCCAGCTTCAGAGTGTCATCTGGGCCGTAGG
SRR940959.227114799.+ GCTCCCGATCCAGCTTCAGAGTGTCATCTGGGCCGTAGGTGGCAAGCACCCTCACAG
SRR940959.209329369.+ GCTCCCGATCCAGCTTCAGAGTGTCATCTGGGCCGTAGGTGGCA
SRR940959.396573641.+ GCTCCCGATCCAGCTTCAGAGTGTCATCTGGGCCGTAGGTGGCA
SRR940959.453934536.+ GCTCCCGATCCAGCTTCAGAGTGTCATCTGGGCCGTAGGTGGCAAGC
SRR940959.369825616.+ GCTCCCGATCCAGCTTCAGAGTGTCATCTGGGCCGTAGGTGGCAAGCACCCTCACAGTGT
SRR940959.463156140.+ GCTCCCGATCCAGCTTCAGAGTGTCATCTGGGCCGTAGGTGGCAAGCACCCTCACAGTGT
SRR940959.519717962.+ GCTCCCGATCCAGCTTCAGAGTGTCATCTGGGCCGTAGGTGG
SRR940959.388193322.+ GCTCCCGATCCAGCTTCAGAGTGTCATCTGGGCCGTAGGTGGCAAGCACCCTCACAGTGT
SRR940959.479070874.- GCTCCCGATCCAGCTTCAGAGTGTCATCTGGGCCGTAGGTGGCAAGCACCCTCACAGTGT
SRR940959.194460169.+ GCTCCCGATCCAGCTTCAGAGTGTCATCTGGGCCGTAGGTGGCAAGCACCCTCACAGTGT
SRR940959.283687311.+ GCTCCCGATCCAGCTTCAGAGTGTCATCTGGGCCGTAGGTGGCAAGCACCCTCACAGTGT
SRR940959.3242465.2+                   AGAGTGTCATCTGGGCCGTAGGTGGCAAGCACCCTCACAGTGT
SRR940959.194437320.+                  AGAGTGTCATCTGGGCCGTAGGTGGCAAGCACCCTCACAGTGT
SRR940959.299229654.-                   GAGTGTCATCTGGGCCGTAGGTGGCAAGCACCCTCACAGTGT
SRR940959.38280252.2+                    AGTGTCATCTGGGCCGTAGGTGGCAAGCACCCTCACAGTGT
SRR940959.511833097.-                        TCATCTGGGCCGTAGGTGGCAAGCACCCTCACAGTGT
SRR940959.63988482.2+                            CTGGGCCGTAGGTGGCAAGCACCCTCACAGTGT
SRR940959.194460169.-                            CTGGGCCGTAGGTGGCAAGCACCCTCACAGTGT
SRR940959.283687311.-                            CTGGGCCGTAGGTGGCAAGCACCCTCACAGTGT
SRR940959.405913974.+                                     AGGTGGCAAGCACCCTCACAGTGT
SRR940959.154407486.+                                              GCACCCTCACAGTGT
SRR940959.84718457.2+                                                ACCCTCACAGTGT
                      ____________________________________________________________
consensus             GCTCCCGATCCAGCTTCAGAGTGTCATCTGGGCCGTAGGTGGCAAGCACCCTCACAGTGT

                          .    :    .    :    .    :    .    :    .    :    .    :
SRR940959.212347108.+ C
SRR940959.492005375.- CACT
SRR940959.14118139.2+ CA
SRR940959.369825616.+ C
SRR940959.463156140.+ C
SRR940959.388193322.+ CACTCTGGGGCATCGAG
SRR940959.479070874.- CACTCTGGGGCATCGAGGTGCTGCAGCCC
SRR940959.194460169.+ CACTCTGGGGCATCGAGGTGCTGCAGCCCACG
SRR940959.283687311.+ CACTCTGGGGCATCGAGGTGCTGCAGCCCACG
SRR940959.3242465.2+  CACTCTGGGGCATCGAGGTGCTGCAGCCCACGAGCAGCACTTTGCACGCACCCACAC
SRR940959.194437320.+ CACTCTGGGGCATCGAGGTGCTGCAGCCCACGAGCAGCACTTTGCACGCACCCACAC
SRR940959.299229654.- CACTCTGGGGCATCGAGGTGCTGCAGCCCACGAGCAGCACTTTGCACGCACCCACACC
SRR940959.38280252.2+ CACTCTGGGGCATCGAGGTGCTGCAGCCCACGAGCAGCACTTTGCACGCACCCACACCC
SRR940959.511833097.- CACTCTGGGGCATCGAGGTGCTGCAGCCCACGAGCAGCACTTTGCACGCACCCACACCCA
SRR940959.63988482.2+ CACTCTGGGGCATCGAGGAGCTGCAGCCCACGAGCAGCACTTTGCACGCACCCACACCCA
SRR940959.194460169.- CACTCTGGGGCATCGAGGTGCTGCAGCCCACGAGCAGCACTTTGCACGCACCCACACCCA
SRR940959.283687311.- CACTCTGGGGCATCGAGGTGCTGCAGCCCACGAGCAGCACTTTGCACGCACCCACACCCA
SRR940959.405913974.+ CACTCTGGGGCATCGAGGTGCTGCAGCCCACGAGTAGAACTTTGCACGCACCCACACCGA
SRR940959.154407486.+ CACTCTGGGGCATCGAGGTGCTGCAGCCCACGAGCAGCACTTTGCACGCACCCACACCCA
SRR940959.84718457.2+ CACTCTGGGGCGTCGAGGTGCTGCAGCCCACGAGCAGCACTTTGCACGCACCCACACCCA
                      ____________________________________________________________
consensus             CACTCTGGGGCATCGAGGTGCTGCAGCCCACGAGCAGCACTTTGCACGCACCCACACCCA

                          .    :    .    :    .    :    .    :    .    :    .    :
SRR940959.511833097.- ACC
```

```
SRR940959.63988482.2+ ACCACCG
SRR940959.194460169.- AACCACA
SRR940959.283687311.- AACCACA
SRR940959.405913974.+ ACCACCGACACGAGCC
SRR940959.154407486.+ ACCACCGACACGAGCCACTTGCACA
SRR940959.84718457.2+ ACCACCGACACGATCCACTTGCACACA
                      ____________________________________________________________
consensus             ACCACCGACACGAGCCACTTGCACACA
```

(4) The exon 4 sequence was predicted by assembling WGS data using CAP3. The exon 4 is marked in red.

```
                          .    :    .    :    .    :    .    :    .    :    .    :
SRR940959.362808710.+ GGCCACCCTTCCCCGACCTGTCCCTCCCCCTGCTTCTCCGGGAGCCTCCCTACCACCTGC
SRR940959.450737162.+ GGCCACCCTTCCCCGACCTGTCCCTCCCCCTGCTTCTCCGGGAGCCTCCCTACCACCTGC
SRR940959.191547556.-                          CCCCCTGCTTCCCCGGGAGCCTCCCTCCCACCTGC
SRR940959.84865214.1+                                GCTTCTCCGGGAGCCTCCCTACCACCTGC
SRR940959.135676596.+                                                       ACCTGC
SRR940959.513163294.+                                                       ACCTGC
SRR940959.513165091.+                                                       ACCTGC
SRR940959.223105701.-                                                            C
                      ____________________________________________________________
consensus             GGCCACCCTTCCCCGACCTGTCCCTCCCCCTGCTTCTCCGGGAGCCTCCCTACCACCTGC

                          .    :    .    :    .    :    .    :    .    :    .    :
SRR940959.362808710.+ TCAGCCTCTGTGTGTCCTTGTCCGGTTCTTCATCCCAGAG
SRR940959.450737162.+ TCAGCCTCTGTGTGTCCTTGTCCGGTTCTTCATCCCAGAG
SRR940959.191547556.- TCAGCCTCTGTGTGTCCTTGTCCGGTTCTTCATCCCAGAGGATGACACCACGTACGCCTG
SRR940959.84865214.1+ TCAGCCTCTGTGTGTCCTTGTNCGGTTCTTCATCCCAGAGGATGACACCACGTACGCCTG
SRR940959.135676596.+ TCAGCCTCTGTGTGTCCTTGTCCGGTTCTTCATCCCAGAGGATGACACCTCGTACGCCTG
SRR940959.513163294.+ TCAGCCTCTGTGTGTCCTTGTCCGGTTCTTCATCCCAGAGGATGACACCACGTACGCCTG
SRR940959.513165091.+ TCAGCCTCTGTGTGTCCTTGTCCGGTTCTTCATCCCAGAGGATGACACCACGTACGCCTG
SRR940959.223105701.- CCGGCCTCTGTGTGTCCTTGTCCGGTTCTTCATCCCAGAGGATGACACCACGTACTCCTG
SRR940959.309361544.-  CACCCTCTGTGTGTCCTTCTCCGGTTCTTCATCCCAGAGGATGCCCCCACGTACGCCTG
SRR940959.193915983.+           TGTGTCCTTGTCCGGTTCTTCATCCCAGAGGATGACACCACGTACGCCTG
SRR940959.265751744.+           TGTGTCNTTGTCCGGTTCTTCATCCCAGAGAATGACACCACGTACGCCTG
SRR940959.360476767.+           TGTGTCCTTGTCCGGTTCTTCATCCCAGAGGATGACACCACGTACGCCTG
SRR940959.203831643.-           TGTGTCCTTGTCCGGTTCTTCATCCCAGAGGATGACACCACGTACGCCTG
SRR940959.431997617.+           TGTGTCCTTGTCCGGTTCTTCATCCCAGAGGATGACACCACGTACGCCTG
SRR940959.361470486.+                    GTCCGGTTCTTCATCCCAGAGGATGACACCACGTACGCCTG
SRR940959.423347026.+                      CCGGTTCTTCATCCCAGAGGATGACACCACGTACGCCTG
SRR940959.484300361.-                           TCTTCATCCCAGAGGATGACACCACGTACGCCTG
SRR940959.142708922.-                            CTTCATCCCAGAGGATGACACCACGTACGCCTG
SRR940959.380295259.-                            CTTCATCCCAGAGGATGACACCACGTACGCCTG
SRR940959.365141535.-                               CATCCCAGAGGATGACACCACGTACGCCTG
SRR940959.133527941.-                                  CCCAGAGGATGACACCACGTACGCCTG
SRR940959.354439360.-                                  CCCAGAGGATGACACCCCGTACGCCTG
SRR940959.157814344.-                                  CCCAGAGGATGACACCACGTACGCCTG
SRR940959.69986416.2+                                         GATGACACCACGTACGCCTG
SRR940959.52352767.1-                                          ATGACACCACGTACGCCTG
SRR940959.463902652.-                                            GACACCACGTACGCCTG
                      ____________________________________________________________
consensus             TCAGCCTCTGTGTGTCCTTGTCCGGTTCTTCATCCCAGAGGATGACACCACGTACGCCTG

                          .    :    .    :    .    :    .    :    .    :    .    :
SRR940959.191547556.- CCCCT
SRR940959.84865214.1+ CCCCTTCCTCC
SRR940959.135676596.+ CCCCTTCATCCCTCTCTCCATCGTTGGCAAGAAA
SRR940959.513163294.+ CCCCTTCCTCCCTCTCTCCATCGTTGGCAAGAAG
SRR940959.513165091.+ CCCCTTCCTCCCTCTCTCCATCGTTGGCAAGAAG
SRR940959.223105701.- CCCCTTCCTCCCTCTCTCCATCGTTGGCAAGAAGCACCG
SRR940959.309361544.- CCCCTTCCTCCCTCTCTCCATCGTTGGCAAGAAGCACCGTA
SRR940959.193915983.+ CCCCTTCCTCCCTCTCTCCATCGTTGGCAAGAAGCACCGTATCTCCAAGG
SRR940959.265751744.+ GCCCTTCCTCACTCTCTCCATCGTTGGCAAGAAGCACCGTATCTCCAAGG
SRR940959.360476767.+ CCCCTTCCTCCCTCTCTCCATCGTTGGCAAGAAGCACCGTATCTCCAAGG
SRR940959.203831643.- CCCCTTCCTCCCTCTCTCCATCGTTGGCAAGAAGCACCGTATCTCCAAGG
SRR940959.431997617.+ CCCCTTCCTCCCTCTCTCCATCGTTGGCAAGAAGCACCGTATCTCCAAGG
SRR940959.361470486.+ CCCCTTCCTCCCTCTCTCCATCGTTGGCAAGAAGCACCGTATCTCCAAGGTACCATGAA
SRR940959.423347026.+ CCCCTTCCTCCCTCTCTCCATCGTTGGCAAGAAGCACCGTATCTCCAAGGTACCATGAAA
SRR940959.484300361.- CCCCTTCCTCCCTCTCTCCATCGTTGGCAAGAAGCACCGTATCTCCAAGGTACCATGAAA
SRR940959.142708922.- CCCCTTCCTCCCTCTCTCCATCGTTGGCAAGAAGCACCGTATCTCCAAGGTACCATGAAA
SRR940959.380295259.- CCCCTACCTCCCTCTCTCCATCGTTGGCAAGAAGCACCGTATCTCCAAGGTACCATGAAA
SRR940959.365141535.- CCCCTTCCTCCCTCTCTCCATCGTTGGCAAGAAGCACCGTATCTCCAAGGTACCATGAAA
SRR940959.133527941.- CCCCTTCCTCCCTCTCTCCATCGTTGGCAAGAAGCACCGTATCTCCAAGGTACCATGAAA
SRR940959.354439360.- CCCCTTCCTCCCTCTCTCCATCGTTGGCAAGAAGCACCGTATCTCCAAGGTACCATGAAA
SRR940959.157814344.- CCCCTTCCTCCCTCTCTCCATCGTTGGCAAGAAGCACCGTATCTCCAAGGTACCATGAAA
SRR940959.69986416.2+ CCCCTTCCTCCCTCTCTCCATCGTTGGCAAGAAGCACCGTATCTCCAAGGTACCATGAAA
```

```
SRR940959.52352767.1- CCCCTTCCTCCCTCTCTCCATCGTTGGCAAGAAGCACCGTATCTCCAAGGTACCATGAAA
SRR940959.463902652.- CCCCTTCCTCCCTCTCTCCATCGTTGGCAAGAAGCACCGTATCTCCAAGGTACCATGAAA
SRR940959.220672047.- CCCCTTCCTCCCTCTCTCCATCGTTGGCAAGAAGCACCGTATCTCCAAGGTACCATGAAA
SRR940959.242187489.-       CCTCCCTCTCTCCATCGTTGGCAAGAAGCACCGTATCTCCAAGGTACCATGAAA
SRR940959.378097191.-          CCCTCTCTCCATCGTTGGCAAGAAGCACCGTATCTCCAAGGTACCATGAAA
SRR940959.257481623.+           CCTCTCTCCATCGTTGGCAAGAAGCACCGTATCTCCAAGGTACCATGAAA
SRR940959.145535357.+              CTCTCCATCGTTGGCAAGAAGCACCGTATCTCCAAGGTACCATGAAA
SRR940959.240073267.-               TCTCCATCGTTGGCAAGAAGCACCGTATCTCCAAGGTACCATGAAA
SRR940959.111466552.+                    ATCGTTGGCAAGAAGCACCGTATCTCNAAGGTACCATGAAA
SRR940959.65233986.1+                       GTTGGCAAGAAGCACCGTATCTCCAAGGTACCATGAAA
SRR940959.464705134.+                             AAGAAGCACCGTATCTCCAAGGTACCATGAAA
SRR940959.69986416.1-                                AAGCACCGTATCTCCAAGGTACCATGAAA
                      ____________________________________________________________
consensus             CCCCTTCCTCCCTCTCTCCATCGTTGGCAAGAAGCACCGTATCTCCAAGGTACCATGAAA

                          .    :    .    :    .    :    .    :    .    :    .    :
SRR940959.423347026.+ C
SRR940959.484300361.- CAGACT
SRR940959.142708922.- CAGACTT
SRR940959.380295259.- CAGACTT
SRR940959.365141535.- CAGACTTCAG
SRR940959.133527941.- CAGACTTCAGGAC
SRR940959.354439360.- CAGACTTCAGGAC
SRR940959.157814344.- CAGACTTCAGGAC
SRR940959.69986416.2+ CAGACTTCAGGACATTGTCA
SRR940959.52352767.1- CAGACTTCAGGACATTGTCAA
SRR940959.463902652.- CAGACTTCAGGACATTGTCAAGG
SRR940959.220672047.- CAGACTTCAGGACATTGTCAAGGTGGTCGAGACAGGCAGT
SRR940959.242187489.- CAGACTTCAGGACATTGTCAAGGTGGTCGAGACAGGCAGTGGGCAT
SRR940959.378097191.- CAGACTTCAGGACATTGTCAAGGTGGTCGAGACAGGCAGTGGGCATGGC
SRR940959.257481623.+ CAGACTTCAGAACATTGTCAAGGTGGTCGAGACAGGCAGTGGACATGGCT
SRR940959.145535357.+ CAGACTTCAGGACATTGTCAAGGTGGTCGAGACAGGCAGTGGGCATGGCTGAC
SRR940959.240073267.- CAGACTTCAGGACATTGTCAAGGTGGTCGAGACAGGCAGTGGGCATGGCTGACT
SRR940959.111466552.+ CANACTTCANGACATTGTCAAGGTGGTCNAGACAGGCAGTGGGCATGGCTGACTCTGAA
SRR940959.65233986.1+ CAGACTTCAGGACATTGTCAAGGTGGTCGAGACAGGCAGTGGGCATGGCTGACTCTGAAG
SRR940959.464705134.+ CAGACTTCAGGACATTGTCAAGGAGGTCGAGACAGGCAGTGGGCATGGCTGACTCTGAAG
SRR940959.69986416.1- CAGACTTCAGGACATTGTCAAGGTGGTCGAGACAGGCAGTGGGCATGGCTGACTCTGAAG
                      ____________________________________________________________
consensus             CAGACTTCAGGACATTGTCAAGGTGGTCGAGACAGGCAGTGGGCATGGCTGACTCTGAAG

                          .    :    .    :    .    :    .    :    .    :    .    :
SRR940959.65233986.1+ AG
SRR940959.464705134.+ AGATGGAG
SRR940959.69986416.1- AGATGGTGTTC
                      ____________________________________________________________
consensus             AGATGGAGTTC
```

(5) The exon 5 sequence was predicted by assembling WGS data using CAP3. The exon 5 is on the reverse strand and marked in red.

```
                          .    :    .    :    .    :    .    :    .    :    .    :
SRR940959.272197980.+ TATCAATCCAACAATCCCAGTTGCCCAACCACACGTGTCCCCTTAGCAGCTGATTTCTCT
SRR940959.182498493.-   TCAATCCAACAATCCCAGTTGCCCAACCACACGTGTCCCCTTAGCAGCTGATTTCTCT
SRR940959.165953777.+    CAATCCAACAATCCCAGTTGCCCAACCACACGTGTCCCCTTAGCAGCTGATTTCTCT
SRR940959.302744533.-              ATCCCAGTTGCCCAACCACACGTGTCCCCTTAGCAGCTGATTTCTCT
SRR940959.190317949.-                  CAGTTGCCCAACCACACGTGTCCCCTTAGCAGCTGATTTCTCT
SRR940959.235711204.+                  CAGTTGCCCAACCACACGTGTCCCCTTAGCAGCTGATTTCTCT
SRR940959.293240563.+                  CAGTTGCCCAACCACACGTGTCCCCTTAGCAGCTGATTTCTCT
SRR940959.52946295.1-                   AGTTGCCCAACCACACGTGTCCCCTTAGCAGCTGATTTCTCT
SRR940959.386725613.+                       GCCCAACCACACGTGTCCCCTTAGCAGCTGATTTCTCT
SRR940959.155826843.+                                        CCCTTAGCAGCTGATTTCTCT
SRR940959.55142381.1-                                         CCTTAGCAGCTGATTTCTCT
SRR940959.284825308.+                                          CTTAGCAGCTGATTTCTCT
SRR940959.419022641.-                                              GCAGCTGATTTCTCT
SRR940959.463825868.-                                              GCAGCTGATTTCTCT
SRR940959.130878416.+                                                 GCTGATTTCTCT
SRR940959.212285374.+                                                 GCTGATTTCTCT
SRR940959.288902258.-                                                    GATTTCTCT
SRR940959.376548274.-                                                    GATTTCTCT
                      ____________________________________________________________
consensus             TATCAATCCAACAATCCCAGTTGCCCAACCACACGTGTCCCCTTAGCAGCTGATTTCTCT

                          .    :    .    :    .    :    .    :    .    :    .    :
SRR940959.272197980.+ CATCACACGACTCACTGTGTCCCCGACAGCCCAGCCCACG
SRR940959.182498493.- CATCACACGACTCACTGTGTCCCCGACAGCCCAGCCCACGAT
SRR940959.165953777.+ CATCACACGACTCACTGTGTCCCCGACAGCCCAGCCCACGATG
```

```
SRR940959.302744533.- CATCACACGACTCACTGTGTCCCCGACAGCCCAGCCCACGATGACCTGTGAGC
SRR940959.190317949.- CATCACACGACTCACTGTGTCCCCGACAGCCCAGCCCACGATGACCTGTGAGCAGAG
SRR940959.235711204.+ CATCACACGGCTCACTGTGTCCCCGACAGCCCAGCCCACGATGACCTGTGAGCAGAG
SRR940959.293240563.+ CATCACAGGACTCACTGTGTCCCCGACAGCCCAGCCCACGATGACCTGTGAGCAGAG
SRR940959.52946295.1- CATCACACGACGCACTGTGTCCCCGACAGCCCAGCCCACGATGACCTGTGAGCAGAGG
SRR940959.386725613.+ CATCACACGACTCACTGTGTCCCCGACAGCCCAGCCCACGATGACCTGTGAGCAGAGGGA
SRR940959.155826843.+ CATCACACGACTCACTGTGTCCCCGACAGCCCAGCCCACGATGACCTGTGAGCAGAGGGA
SRR940959.55142381.1- CATCACACGACTCACTGTGTCCCCGCCAGCCCAGCCCACGATGACCTGTGAGCAGAGGGA
SRR940959.284825308.+ CATCACACGACTCACTGTGTCCCCGACAGCCCAGCCCACGATGACCTGTGAGCAGAGGGA
SRR940959.419022641.- CATCACACGACTCACTGTGTCCCCGACAGCCCAGCCCACGATGACCTGTGAGCAGAGGGA
SRR940959.463825868.- CATCACACGACTCACTGTGTCCCCGACAGCCCAGCCCACGATGACCTGTGAGCAGAGGGA
SRR940959.130878416.+ CATCACACGACTCACTGTGTCCCCGACAGCCCAGCCCACGATGACCTGTGAGCAGAGGGA
SRR940959.212285374.+ CATCACACGACTCACTGTGTCCCCGACAGCCCAGCCCACGATGACCTGTGAGCAGAGGGA
SRR940959.288902258.- CATCACACGACTCACTGTGTCCCCGACAGCCCAGCCCACGATGACCTGTGAGCAGAGGGA
SRR940959.376548274.- CATCACACGACTCACTGTGTCCCCGACAGCCCAGCCCACGATGACCTGTGAGCAGAGGGA
SRR940959.24972658.2-   TCACACGACTCACTGTGTCCCCGACAGCCCAGCCCACGATGACCTGTGAGCAGAGGGA
SRR940959.89700685.1+         GACTCACTGTGTCCCCGACAGCCCAGCCCACGATGACCTGTGAGCAGAGGNA
SRR940959.123613695.-         GACTCACTGTGTCCCCGACAGCCCAGCCCACGATGACCTGTGAGCAGAGGGA
SRR940959.173034016.+             CACTGTGTCCCCGACAGCCCAGCCCACGATGACCTGTGAGCAGAGGGA
SRR940959.356062931.+             CACTGTGTCCCCGACAGCCCAGCCCACGATGACCTGTGAGCAGAGGGA
SRR940959.87171683.1-                              CCCAGCCCACGATGACCTTTGAGCAGAGGGA
SRR940959.386725613.-                                   CCCACGGTGACCTGTGAGCAGAGGGA
SRR940959.100808631.+                                            ACCTGTGAGCAGAGGGA
SRR940959.434405535.+                                                           GA
SRR940959.463494001.-                                                   AGCAGAGGGA
SRR940959.14376046.2-                       CCGACAGCCCAGCCCACGATGACCTGTGAGCAGAGGGA
SRR940959.154734533.+                                  GCCCACGATGACCTGTGAGCAGAGGGA
SRR940959.204926400.-                           CAGCCCAGCCCACGATGACCTGTGAGCAGAGGGA
SRR940959.221390388.+                             GCCCAGCCCACGATGACCTGTGAGCAGAGGGA
SRR940959.385015909.+                             GCCCAGCCCACGATGACCTGTGAGCAGAGGGA
SRR940959.351074174.-                 GTGTCCCCGACAGCCCAGCCCACGATGACCTGTGAGCAGAGGGA
SRR940959.335030759.+                 GTGTCCCCGACAGCCCAGCCCACGATGACCTGTGAGCAGAGGGA
SRR940959.494733450.-                 GTGTCCCCGACAGCCCAGCCCACGATGACCTGTGAGCAGAGGGA

consensus             CATCACACGACTCACTGTGTCCCCGACAGCCCAGCCCACGATGACCTGTGAGCAGAGGGA

                          .    :    .    :    .    :    .    :    .    :    .    :
SRR940959.386725613.+ GG
SRR940959.155826843.+ GGAGGCGGGGTCAGCCCCG
SRR940959.55142381.1- GGAGGCGGGGTCAGCCCCGT
SRR940959.284825308.+ GGAGGCGGGGTCAGCCCCGTA
SRR940959.419022641.- GGAGGCGGGGTCAGCCCCGTAGCAG
SRR940959.463825868.- GGAGGCGGGGTCAGCCCCGTAGCAG
SRR940959.130878416.+ GGAGGCGGGGTCACCCCCGTAGCAGTCG
SRR940959.212285374.+ GGAGGCGGGGTCAGCCCCGTAGCAGTCG
SRR940959.288902258.- GGAGGCGGGGTCAGCCCCGTAGCAGTCGCTG
SRR940959.376548274.- GGAGGCGGGGTCAGCCCCGTAGCAGTCGCTG
SRR940959.24972658.2- GGAGGCGGGGTCAGCCCCGTAGCAGTCGCTGATGCCCTTGGG
SRR940959.89700685.1+ GGAGGCGGCGTCAGCCCCGTAGCAGTCGCTGATGCCCTTGGGCAGAGC
SRR940959.123613695.- GGAGGCGGGGTCAGCCCCGTAGCAGTCGCTGATGCCCTTGGGCAGAGC
SRR940959.173034016.+ GGAGGCGGGGTCAGCCCCGTAGCAGTCGCTGATGCCCTTGGGCAGAGCACTG
SRR940959.356062931.+ GGAGGCGGGGTCAGCCCCGTAGCAGTCGCTGATGCCCTTGGGCAGAGCACTG
SRR940959.260594741.-        GGGTCAGCCCCGTAGCAGTCGCTGATGCCCTTGGGCAGACCACTGGTGTTGCC
SRR940959.87171683.1- GGAGGCGGGGTCAGCCCCNTAGCAGTCGCTGATGCCCTTGGGCAGAGCACTGGTGTTGCC
SRR940959.386725613.- GGAGGCGGGGTCAGCCCCGTAGCAGTCGCTGAGGCCCTTGGGCAGAGCACTGGTGTTGCC
SRR940959.100808631.+ GGAGGCGGGGTCAGCCCCGTAGCAGTCGCTGATNCCCTTGGGCAGAGCACTGGTGTTGCC
SRR940959.158840495.+ GGAGGCGGGGTCAGCCCCGTAGCAGTCGCTGATGCCCTTGGGCAGAGCACTGGGGTTGCC
SRR940959.434405535.+ GGAGGCGGGGTCAGCCCCGTAGCAGTCGCTGATGCCCTTGGGCAGAGCACTGGTGTTGCC
SRR940959.463494001.- GGAGGCGGGGTCAGCCCCGTAGCATTCGCTGATGCCCTTGGGCAGAGCACTGGTGTTGCC
SRR940959.14376046.2- GGAGGCGGGGTCAGCCCCGTAGCAGTCGCTGATGCCCTTGGGCAGAGCACTGGTGTTGCC
SRR940959.154734533.+ GGAGGCGGGGTCAGCCCCGTAGCAGTCGCTGATGCCCTTGGGCAGAGCACTGGTGTTGCC
SRR940959.204926400.- GGAGGCGGGGTCAGCCCCGTAGCAGTCGCTGATGCCCTTGGGCAGAGCACTGGTGTTGCC
SRR940959.222179102.+            CAGCCCCGTAGCAGTCGCTGATGCCCTTGGGCAGAGCACTGGTGTTGCC
SRR940959.221390388.+ GGAGGCGGGGTCAGCCCCGTAGCAGTCGCTGATGCCCTTGGGCAGAGCACTGGTGTTGCC
SRR940959.385015909.+ GGAGGCGGGGTCAGCCCCGTAGCAGTCGCTGATGCCCTTGGGCAGAGCACTGGTGTTGCC
SRR940959.101966339.-       GGGGTCAGCCCCGTAGCAGTCGCTGATGCCCTTGGGCAGAGCACTGGTGTTGCC
SRR940959.428006657.-       GGGGTCAGCCCCGTAGCAGTCGCTGATGCCCTTGGGCAGAGCACTGGTGTTGCC
SRR940959.351074174.- GGAGGCGGGGTCAGCCCCGTAGCAGTCGCTGATGCCCTTGGGCAGAGCACTGGTGT
SRR940959.335030759.+ GGAGGCGGGGTCAGCCCCGTAGCAGTCGCTGATGCCCTTGGGCAGAGCACTGGTGT
SRR940959.494733450.- GGAGGCGGGGTCAGCCCCGTAGCAGTCGCTGATGCCCTTGGGCAGAGCACTGGTGT
SRR940959.353802257.+                                     ATTGGGCAGAGCACTGGTGTTGCC
SRR940959.47731150.1+                                             GAGCACTGGTGTTGCC
SRR940959.501595762.-                                               NCACTGGTTTTGCC
SRR940959.353978071.+                                                 ACTGGTGTTGCC
SRR940959.396446915.+                                                 ACTGGTGTTGCC
SRR940959.514548033.+                                                    GGTGTTGCC
SRR940959.380861679.+                                                           CC
```

```
SRR940959.428896204.+                                                           CC
                      ____________________________________________________________
consensus             GGAGGCGGGGTCAGCCCCGTAGCAGTCGCTGATGCCCTTGGGCAGAGCACTGGTGTTGCC

                          .    :    .    :    .    :    .    :    .    :    .    :
SRR940959.260594741.- CCAGGCGGTACACCAGCATGTGGTGCACCGTCGTCCCCTTNTGATGG
SRR940959.87171683.1- CCAGGCGGT
SRR940959.386725613.- CCAGGCGGTACACC
SRR940959.100808631.+ CCAGGCGGTACACCAGGATGTGG
SRR940959.158840495.+ CCAGGCGGTACACCAGGATGTGGTGCACCGTCGTCCCCTT
SRR940959.434405535.+ CCAGGCAGTACACCAGGATGTGGTGCACCGTCGTCCCC
SRR940959.463494001.- CCAGGCGGTACACCAGGATGTGGTGCACCG
SRR940959.14376046.2- CC
SRR940959.154734533.+ CCAGGCGGTACAC
SRR940959.204926400.- CCAGGC
SRR940959.222179102.+ CCAGGCGGTACACCAGGATGTGGTGCACCGTCGTCCCCTTGTGATGGACCA
SRR940959.221390388.+ CCAGGCGG
SRR940959.385015909.+ CCAGGCGG
SRR940959.101966339.- CCAGGCGGTACACCAGGATGTGGTGCACCGTCGTCCCCTTGTGATG
SRR940959.428006657.- CCAGGCGGTACACCAGGATGTGGTGCACCGTCGTCCCCTTGTGATG
SRR940959.353802257.+ CCAGGCGGTACACCAGGATGTGGTGCACCGTCGTCCCCTTGTGATGGACCAACTTGGGCT
SRR940959.47731150.1+ CCAGGCGGTACACCAGGATGTGGTGCACCGTCGTCCCCTTGTGATGGACCAACTTGGGCT
SRR940959.501595762.- CCAGGCGGTACTCCAGGATGTGGTGCACCGTCGTCCCCTTGTGATGGACCAACTTGGGCT
SRR940959.353978071.+ CCAGGCGGTACACCAGGATGTGGTGCACCGTCGTCCCCTTGTGATGGACCAACTTGGGCT
SRR940959.396446915.+ CCAGGCGGTACACCAGGATGTGGTGCACCGTCGTCCCCTTGTGATGGACCAACTTGGGCT
SRR940959.514548033.+ CCAGGCGGTACACCAGGATGTGGTGCACCGTCGTCCCCTTGTGATGGACCAACTTGGGCT
SRR940959.380861679.+ CCAGGCGGTACACCAGGATGTGGTGCACCGTCGTCCCCTTGTGATGGACCAACTTGGGCT
SRR940959.428896204.+ CCAGGCGGTACACCAGGAGGTGGTGCACCGTCGTCCCCTTGTGATGGACCAACTTGGGCT
SRR940959.15523980.1+       GGTACACCAGGATGTGGTGCACCGTCGTCCCCTTGTGATGGACCAACTTGGGCT
SRR940959.86634604.2+       GGTACACCAGGATGTGGTGCACCGTCGTCCCCTTGTGATGGACCAACTGGGGCT
SRR940959.173337384.+            ACCAGGATGTGGTGCACCGTCGTCCCCTTGTGATGGACCAACTTGGGCT
SRR940959.424404552.+                GGATGTGGTGCACCGTCGTCCCCTTGTGATGGACCAACTTGGGCT
SRR940959.222956517.+                 GATGTGGTGCACCGTCGTCCCCTTGTGATGGACCAACTTGGGCT
SRR940959.205781993.-                  ATGTGGTGCACCGTCGTCCCCTTGTGATGGACCAACTTGGGCT
SRR940959.391650191.+                      GGTGCACCGTCGTCCCCTTGTGATGGACCAACTTGGGCT
SRR940959.468867748.+                      GGTGCACCGTCGTCCCCTTGTGATGGACCAACTTGGGCT
SRR940959.216599781.-                          CACCGTCGTCCCCTTGTGATGGACCAACTTGGGCT
SRR940959.63681579.1-                           CCCGTCGTCCCCTTGTGATGGACCAACTTGGGCT
SRR940959.60882870.1+                                         GTGATGGACCAACTTGGGCT
SRR940959.475831135.-                                         GTGATGGACCAACTTGGGCT
SRR940959.477797101.-                                             TGGACCAACTTGGGCT
SRR940959.45088177.1+                                              GGACCAACTTGGGCT
SRR940959.69979238.2+                                              GGACCAACTTGGGCT
SRR940959.180444889.+                                                 CCAACTTGGGCT
SRR940959.193650067.-                                                 CCAACTTGGGCT
SRR940959.304733890.-                                                  CAACTTGGGCT
                      ____________________________________________________________
consensus             CCAGGCGGTACACCAGGATGTGGTGCACCGTCGTCCCCTTGTGATGGACCAACTTGGGCT

                          .    :    .    :    .    :    .    :    .    :    .    :
SRR940959.353802257.+ CAAACTGGGCGGGGGA
SRR940959.47731150.1+ CAAACTGGGCGGGGGACGCAGGAC
SRR940959.501595762.- CACACTGGGCGGGGGACGCAGGACAG
SRR940959.353978071.+ CAAACTGGGCGGGGGACGCAGGACAGAG
SRR940959.396446915.+ CAAACTGGGCGGGGGACGCAGGACAGAG
SRR940959.514548033.+ CAAACTGGGCGGGGGACGCAGGACAGAGTGT
SRR940959.380861679.+ CAAACTGGGCGGGGGACGCAGGACAGAGTGTGGTCAGG
SRR940959.428896204.+ CAAACTGGGCGGGGGACGCAGGACAGAGTGTGGTCAAG
SRR940959.15523980.1+ CAAACTGGGCGGGGGACGCAGGACAGAGTGTGGTCAAGATCTTCTG
SRR940959.86634604.2+ CAAACTGGGCGGGGGACGCAGGACAGAGTGTGGTCAAGATCTTGTG
SRR940959.173337384.+ CAAACTGGGCGGGGGACGCAGGACAGAGTGTGGTCATGATCTTCTGGGCCA
SRR940959.424404552.+ CAAACTGGGCGGGGGACGCAGGACAGAGTGTGGTCAAGATCTTCTGGGCCATGTT
SRR940959.222956517.+ CAAACTGGGCGGGGGACGCAGGACAGAGTGTGGTCAAGATCTTCTGGGCCATGTTC
SRR940959.205781993.- CAAACTGGGCGGGGGACGCCGGACAGAGTGTGCTCAAGATCTTCTGGGCCCTGTTCT
SRR940959.391650191.+ CAAACTGGGCGGGGGACGCAGGACAGAGTGTGGTCAAGATCTTCTGGGCCATGTTCTTAT
SRR940959.468867748.+ CAAACTGGGCGGGGGACGCAGGACAGAGTGTGGTCAAGATCTTCTGGGCCATGTTCTTAT
SRR940959.216599781.- CAAACTGGGCGGGGGACGCAGGACAGAGTGTGGTCAAGATCTTCTGGGCCATGTTCTTAT
SRR940959.63681579.1- CAAACTGGGCGGGGGACGCAGGACAGAGTGTGGTCAAGATCTTCTGGGCCATGTTCTTAT
SRR940959.60882870.1+ CAAACTGGGCGGGGGACGCAGGACAGAGTGTGGTCAAGATCTTCTGGGCCATGTTCTTAT
SRR940959.475831135.- CAAACTGGGCGGGGGACGCAGGACAGAGTGTGGTCAAGATCTTCTGGGCCATGTTCTTAT
SRR940959.477797101.- CAAACTGGGCGGGGGACGCAGGACAGAGTGTGGTCAAGATCTTCTGGGCCATGTTCTTAT
SRR940959.45088177.1+ CAAACTGGGCGGGGGACGCAGGACAGAGTGTGGTCAAGATCTTCTGGGCCATGTTCTTAT
SRR940959.69979238.2+ CAAACTGGGCGGGGGACGCAGGACAGAGTGTGGTCAAGATCTTCTGGGCCATGTTCTTAT
SRR940959.180444889.+ CAAACTGGGCGGGGGACGCAGGACAGAGTGGGGGCAAGATCTTCTGGGCCATGTTCTTAT
SRR940959.193650067.- CAAACTGGGCGGGGGACGCAGGACAGAGTGTGGTCAAGATCTTCTGGGCCATGTTCTTAT
SRR940959.304733890.- CAAACTGGGCGGGGGACGCAGGACAGAGTGTGGTCAAGATCTTCTGGGCCATGTTCTTAT
                      ____________________________________________________________
```

```
consensus             CAAACTGGGCGGGGGACGCAGGACAGAGTGTGGTCAAGATCTTCTGGGCCATGTTCTTAT

                          .    :    .    :    .    :    .    :    .    :    .    :
SRR940959.391650191.+ C
SRR940959.468867748.+ C
SRR940959.216599781.- CCCCT
SRR940959.63681579.1- CCCCTC
SRR940959.60882870.1+ CCCCTCTGTTCCTTTTCCCC
SRR940959.475831135.- CCCCTCTGTTCCTTTTCCCC
SRR940959.477797101.- CCCCTCTGTTCCTTTTCCCCAAAT
SRR940959.45088177.1+ CCCCTCTGTTCCTTTTCCCCAAATA
SRR940959.69979238.2+ CCCCTCTGTTCCTTTTCCCCAAATA
SRR940959.180444889.+ CCCCTCTGTTCCTTTTCCCCAAATAAAA
SRR940959.193650067.- CCCCTCTGTTCCTTTTCCCCAAATAAAA
SRR940959.304733890.- CCCCTCTGTTCCTTTTCCCCAAGTAAAAC
                      ____________________________________________________________
consensus             CCCCTCTGTTCCTTTTCCCCAAATAAAAC
```

(6) The exon 6 sequence was predicted by assembling WGS data using CAP3. The exon 6 is on the reverse strand and marked in red.

```
                          .    :    .    :    .    :    .    :    .    :    .    :
SRR940959.302744533.+ AAGTAGGCGTGCATTCTGCATCCCTTCTCGACACCTAGAGGGCTCAGCCAGCCTCACCGC
SRR940959.277610206.+     AGGCGTGCATTCTGCATCCCTTCTCGACACCTAGAGGGCTCAGCCAGCCTCACCGC
SRR940959.24972658.1+               TCTGCATCCCTTCTCGACACCTAGAGGGCTCAGCCAGCCTCACCGC
SRR940959.14376046.1+                              GACACCTAGAGGGCTCAGCCAGCCTCACCGC
SRR940959.414779458.+                                                CCAGCCTCACCGC
SRR940959.351074174.+                                                          CGC
SRR940959.494733450.+                                                          CGC
                      ____________________________________________________________
consensus             AAGTAGGCGTGCATTCTGCATCCCTTCTCGACACCTAGAGGGCTCAGCCAGCCTCACCGC

                          .    :    .    :    .    :    .    :    .    :    .    :
SRR940959.302744533.+ CGCTCTCCCGTAAATGCTCTAGGCGCTCACCAGGAAGACT
SRR940959.277610206.+ CGCTCTCCCGTAAATGCTCTAGGCGCTCACCAGGAAGACTGTGC
SRR940959.24972658.1+ CGCTCTCCCGTAAATGCTCTAGGCGCTCACCAGGAAGACTGTGCAAATTGCTGT
SRR940959.14376046.1+ CGCTCTCCCGTAAATGCTCTAGGCGCTCACCAGGAAGACTGTGCAAATTGCTGTAGTGAA
SRR940959.414779458.+ CGCTCTCCCGTAAATGCTCTAGGCGCTCACCAGGAAGACTGTGCAAATTGCTGTAGTGAA
SRR940959.351074174.+ CGCTCTCCCGTAAATGCTCTAGGCGCTCACCAGGAAGACTGTGCAAATTGCTGTAGTGAA
SRR940959.494733450.+ CGCTCTCCCGTAAATGCTCTAGGCGCTCACCAGGAAGACTGTGCAAATTGCTGTAGTGAA
SRR940959.463494001.+              ATGCTCTAGGCGCTCACCAGGAAGACTGTGCAAATTGCTGTAGTGAA
SRR940959.55142381.2+                 CTCTAGGCGCTCACCAGGAAGACTGTGCAAATTGCTGTAGTGAA
SRR940959.203117712.+                              CCAGGAAGACTGTGCAAATTGCTGTAGTGAA
SRR940959.123613695.+                                 GGAAGACTGTGCAAATTGCTGTAGTGAA
SRR940959.87171683.2+                                   AAGACTGTGCAAATTGCTGTAGTGAA
SRR940959.69895381.1-                                                           AA
                      ____________________________________________________________
consensus             CGCTCTCCCGTAAATGCTCTAGGCGCTCACCAGGAAGACTGTGCAAATTGCTGTAGTGAA

                          .    :    .    :    .    :    .    :    .    :    .    :
SRR940959.14376046.1+ TCTCCAGTC
SRR940959.414779458.+ TCTCCAGTCGGACCCACTGGGGGTCCA
SRR940959.351074174.+ TCTCCAGTCGGACCCACTGGGGGTCCAAAGCCATCCC
SRR940959.494733450.+ TCTCCAGTCGGACCCACTGGGGGTCCAAAGCCATCCC
SRR940959.463494001.+ TCTCCAGTCGGACCCACTGGGGGTCCAAAGCCATCCCAGTAGAGATACCCACG
SRR940959.55142381.2+ TCTCCAGTCGGACCCACTGGGGGTCCAAAGCCATCCCAGTAGAGATACCCACGTCA
SRR940959.203117712.+ TCTCCAGTCGGACCCACTGGGGGTCCAAAGCCATCCCAGTAGAGATACCCACGTCACCTG
SRR940959.123613695.+ TCTCCAGTCGGACCCACTGGGGGTCCAAAGCCATCCCAGTAGAGATACCCACGTCACCTG
SRR940959.87171683.2+ TCGCCAGTCGGACCCACTGGGGATCCAAAGCCATCCCAGTAGAGATACCCACGTCACCTG
SRR940959.69895381.1- TCTCCAGTCGGACCCACTGGGGGTCCAAAGCCATCCCAGTAGAGATACCCACGTCACCTG
SRR940959.347673020.-     CAGTCGGACCCACTGGGGGTCCAAAGCCATCCCAGTAGAGATACCCACGTCACCTG
SRR940959.501595762.+               CACTGGGGGTCCAAAGNCATCCCAGTAGAGATANCCACGTCACCTG
SRR940959.95092905.1-                ACTGGGGGTCCAAAGCCATCCCAGTAGAGATACCCACGTCACCTG
SRR940959.101966339.+                                     AGTAGAGATACCCACGTCACCTG
SRR940959.428006657.+                                     AGTAGAGATACCCACGTCACCTG
SRR940959.512234557.-                                                      CACCTG
SRR940959.475831135.+                                                        CCTG
                      ____________________________________________________________
consensus             TCTCCAGTCGGACCCACTGGGGGTCCAAAGCCATCCCAGTAGAGATACCCACGTCACCTG

                          .    :    .    :    .    :    .    :    .    :    .    :
SRR940959.203117712.+ GAAACTGGT
SRR940959.123613695.+ GAAACTGGTAAC
SRR940959.87171683.2+ GAAACTGGTAACTG
SRR940959.69895381.1- GAAACTGGTAACTGTTGGATGGAGGGTGATTTGGCGGC
SRR940959.347673020.- GAAACTGGTAACTGTTGGATGGAGGGTGATTTGGCGGCAAGAAT
```

```
SRR940959.501595762.+ GAAACTGGTAACTGTTGGATGGAGGGTGATTTGGCGGCAAGAATGGCTTGGAAG
SRR940959.95092905.1- GAAANTGGTAACTGTTGGATGGAGGGTGATTTGGCGGCAAGAATGGCTGGGAAGT
SRR940959.101966339.+ GAAACTGGTAACTGTTGGATGGAGGGTNATTTGGCGGCAAGAATGGCTGGGAAGTAGGTG
SRR940959.428006657.+ GAAACTGGTAACTGTTGGAAGGAGGGTGATTTGGCGGCAAGAATGGCTGGGAAGTAGGTG
SRR940959.512234557.- GAAACTGGTAACTGTTGGATGGAGGGTGATTTGGCGGCAAGAATGGCTGGGAAGTAGGTG
SRR940959.475831135.+ GAAACTGGTAACTGTTGGATGGAGGGTGATTTGGCGGCAAGAATGGCTGGGAAGTAGGTG
                      ____________________________________________________________
consensus             GAAACTGGTAACTGTTGGATGGAGGGTGATTTGGCGGCAAGAATGGCTGGGAAGTAGGTG

                          .    :    .    :    .    :    .    :    .    :    .    :
SRR940959.101966339.+ CAGGGACCCGGGCCTGC
SRR940959.428006657.+ CAGGGACCCGGGCCTGC
SRR940959.512234557.- CAGGGACCCGGGCCTGCCCTTGTCTTTTGGTCCT
SRR940959.475831135.+ CAGGGACCCGGGCCTGCCCTTGTCTTTTGGTCCTGG
                      ____________________________________________________________
consensus             CAGGGACCCGGGCCTGCCCTTGTCTTTTGGTCCTGG
```

(7) The exon 7 sequence was predicted by assembling WGS data using CAP3. The exon 7 is marked in red.

```
                          .    :    .    :    .    :    .    :    .    :    .    :
SRR940959.347340487.- CCCCCCCTCCACTTTCCCAGGGCCTCTTCCTCCCCGCCGTGCGACACGGTGTCCTGCATC
SRR940959.380337020.+         CCACTTTCCCAGGGCCTCTTCCTCCCCACAGTGCGACACGGTGTCCTGCATC
SRR940959.72741554.1+                                           GACACGGTGTCCTGCATC
SRR940959.344535215.+                                                     CCTGCATC
                      ____________________________________________________________
consensus             CCCCCCCTCCACTTTCCCAGGGCCTCTTCCTCCCCACAGTGCGACACGGTGTCCTGCATC

                          .    :    .    :    .    :    .    :    .    :    .    :
SRR940959.347340487.- CTGTGATTGGTCCTTCCTGCACCAGGTGTGTACGGCTCCT
SRR940959.380337020.+ CTGTGAATGGTCCTTACTGCACCAGGTGTGTACGGCTCCTCGGGGATT
SRR940959.72741554.1+ CTGTGATTGGTCCTTCCTGCACCAGGTGTGTACGGCTCCTCGGGGATTCGAGCGTACTAC
SRR940959.344535215.+ CTGTGATTGGTCCTTCCTGCACCAGGTGTGTACGGCTCCTCGGGGATTCGAGTGTACTAC
SRR940959.247684362.+                 CTGCACCAGGTGTGTACGGCTCCTCGGGGATTCGAGTGTACTAC
SRR940959.5799872.1+                       CCAGGTGTGTACGGCTCCTCGGGGATTCGAGTGTACTAC
SRR940959.474454994.+                       CAGGTGTGTACGGCTCCTCGGGGATTCGAGTGTACTAC
SRR940959.344535215.-                                                      GTACTAC
SRR940959.398850632.+                                   GCTCCTCGGGGATTCGAGTGTACTAC
                      ____________________________________________________________
consensus             CTGTGATTGGTCCTTCCTGCACCAGGTGTGTACGGCTCCTCGGGGATTCGAGTGTACTAC

                          .    :    .    :    .    :    .    :    .    :    .    :
SRR940959.72741554.1+ ACGGCGCAGCTACGCAAATATG
SRR940959.344535215.+ ACGGCGCAGCTACGCAAATATGACATGGGGGT
SRR940959.247684362.+ ACGGCGCAGCTACGCAAATATGACATGGGTGTCCTGCAGCTGGGCTTCTGCACTTT
SRR940959.5799872.1+  ACGGCGCAGCTACGCAAATATGACATGGGTGTCCTGCAGCTGGGCTTCTGCACTTTGCCC
SRR940959.474454994.+ ACGGCGCAGCTACGCAAATATGACATGGGTGTCCTGCAGCTGGGCTTCTGCACTTTGCCC
SRR940959.47482045.2+                            GGTGTCCTGCAGCTGGGCTTCTGCACTTTGCCC
SRR940959.344535215.- ACGGCGCAGCTACGCAAATATGACATGGGTGTCCTGCAGCTGGGCTTCTGCACTTTGCCC
SRR940959.398850632.+ ACGGCGCAGCTACGCAAATATGACATGGGTGTCCTGCAGCTGGGCTTCTGCACTTTGCCC
SRR940959.430449639.+                  ATATGACATGGGTGTCCTGCAGCTGGGCTTCTGCACTTTGCCC
SRR940959.424717539.+                                     AGCTGGGCTTCTGCACTTTGCCC
                      ____________________________________________________________
consensus             ACGGCGCAGCTACGCAAATATGACATGGGTGTCCTGCAGCTGGGCTTCTGCACTTTGCCC

                          .    :    .    :    .    :    .    :    .    :    .    :
SRR940959.5799872.1+  A
SRR940959.474454994.+ AT
SRR940959.47482045.2+ ATCCACTTCATACCCCCGGGTGCCGAGTCCTTCACGTCCTATGGGCTGTGTAAGGCAGAG
SRR940959.344535215.- ATCCACTTCATACCCCCGGGTGCCGAGTCCTTC
SRR940959.398850632.+ ATCCACTTCATACC
SRR940959.430449639.+ ATCCACTTCATACCCCCGGGTGCCGAGTCCTTCACGTCCTATGGGCTGTGTAAGGCA
SRR940959.424717539.+ ATCCACTTCATACCCCCGGGTGCCGAGTCCTTCACGTCCTATGGGCTGTGTAAGGCAGAG
SRR940959.52296932.1+   CCACTTCATACCCCCGGGTGCCGAGTCCTTCACGTCCTATGGGCTGTGTAAGGCAGAG
SRR940959.240556384.+                              CTTCACGTCCTATGGGCTGTGTAAGGCAGAG
SRR940959.288050296.+                               TTCACGTCCTATGGGCTGTGTAAGGCAGAG
SRR940959.437781.1+                                                            GAG
SRR940959.43667062.1+                                                          GAG
                      ____________________________________________________________
consensus             ATCCACTTCATACCCCCGGGTGCCGAGTCCTTCACGTCCTATGGGCTGTGTAAGGCAGAG

                          .    :    .    :    .    :    .    :    .    :    .    :
SRR940959.47482045.2+ AAGTTTG
SRR940959.424717539.+ AAGTTTGACGAGGTGAA
SRR940959.52296932.1+ AAGTTTGACGAGGTGAAGCTGGGAGGCATCCCTCCTCCCCTG
SRR940959.240556384.+ AAGTTTGACGAGGTGAAGCTGGGAGGCATCCCTCCTCCCCTGAGCTTCCCGGTTCTCCAT
SRR940959.288050296.+ AAGTTTGACGAGGTGAAGCTGGGAGGAATCCCTCCTCCCCTGAGCTTCCCGGTTCTCCAT
```

```
SRR940959.437781.1+   AAGTTTGACGAGGTGAAGCTGGGAGGCATCCCTCCTCCCCTGAGCTTCCCGGTTCTCCAT
SRR940959.43667062.1+ AAGTTTGACGAGGTGAAGCTGGGAGGCATCCCTCCTCCCCTGAGCTTCCCGGTTCTCCAT
                      ____________________________________________________________
consensus             AAGTTTGACGAGGTGAAGCTGGGAGGCATCCCTCCTCCCCTGAGCTTCCCGGTTCTCCAT

                          .    :    .    :    .    :    .    :    .    :    .    :
SRR940959.240556384.+ CGCAAATCT
SRR940959.288050296.+ CGCAAATCTG
SRR940959.437781.1+   CGCAAATCTGCTGTGACCCACAAGACACAACTTGAGC
SRR940959.43667062.1+ CGCAAATCTGCTGTGACCCACAAGACACAACTTGAGC
                      ____________________________________________________________
consensus             CGCAAATCTGCTGTGACCCACAAGACACAACTTGAGC
```

(8) The exon 8 sequence was predicted by assembling WGS data using CAP3. The exon 8 is marked in red.

```
                          .    :    .    :    .    :    .    :    .    :    .    :
SRR940959.157709363.+ ACGAAGGTGACAGATGTGAAAGGAGGAAACCGAGGAGCGGGGCCAGACGAGAGTTCCTAA
SRR940959.189973846.+ ACGAAGGTGTCAGATGTGAAAGGAGGAAACCGAGGAGCGGGGCCAGACGAGAGTTCCTAA
SRR940959.92476664.2+            AGATGTGAAAGGAGGAAACCGAGGAGCGGGGCCAGACGAGAGTTCCTAA
SRR940959.331963127.+            AGATGTGAAAGGAGGAAACCGAGGAGCGGGGCCAGACGAGAGTTCCTAA
SRR940959.113669618.+                     AGGAGGAAACCGAGGAGCGGGGCCAGACGAGAGTTCCTAA
SRR940959.158063890.+                     AGGAGGAAACCGAGGAGCGGGGCCAGACGAGAGTTCCTAA
SRR940959.240556384.-                      GGAGGAACCCGAGGAGCGGGGCCAGCCGAGAGTTCCTAA
SRR940959.158694893.+                            AACCGAGGAGCGGGGCCAGACGAGAGTTCCTAA
SRR940959.412058904.+                                GAGGAGCGGGGCCAGACGAGAGTTCCTAA
SRR940959.63681231.2+                                                    AGTTCCTAA
SRR940959.34938406.1+                                                           AA
SRR940959.141022903.+                                                           AA
SRR940959.241939881.+                                                            A
SRR940959.331596001.+                                                            A
SRR940959.387467835.+                                                            A
                      ____________________________________________________________
consensus             ACGAAGGTGACAGATGTGAAAGGAGGAAACCGAGGAGCGGGGCCAGACGAGAGTTCCTAA

                          .    :    .    :    .    :    .    :    .    :    .    :
SRR940959.157709363.+ AAAGCCAGGTGGTCTCCTCCCTGCCACAGATGAACGGGGC
SRR940959.189973846.+ CAAGCCAGGTGGTCTCCTCCCTGCCACAGATGAACGGGGC
SRR940959.92476664.2+ CAAGCCAGGTGGTCTCCTGCCTGCCACAGATGAACGGGGCCCCGGTGCCTG
SRR940959.331963127.+ CAAGCCAGGTGGTCTCCTCCCTGCCACAGATGAACGGGGCCCCGGTGCCTG
SRR940959.113669618.+ CAANCCAGGTNGTCTCCTCCCTGCCACAGNTGAACGGGGCCCCGGTGCCTGACATCCAGG
SRR940959.158063890.+ CAAGCCAGGTGGTCTCCTCCCTGCCACAGATGAACGGGGCCCCGGTGCCTGACATCCAGG
SRR940959.240556384.- CAAGCCAGGTGGCCTCCTCCCTGCCACAGATGAACGGGGCCCCGGTGCCTGACATCCAGG
SRR940959.158694893.+ CAAGCCAGGTGGTCTCCTCCCTGCCACAGATGAACGGGGCCCCGGTGCCTGACATCCAGG
SRR940959.412058904.+ CAAGCCAGGTGGCCTCCTCCCTGCCACAGATGAACGGGGCCCCGGTGCCTGACATCCAGG
SRR940959.63681231.2+ CAAGCCAGGTGGTCTCCTCCCTGCCACAGATGAACGGGGCCCCGGTGCCTGACATCCAGG
SRR940959.34938406.1+ CAAGCCAGGTGGTCTCCTCCCTGCCACAGATGAACGGGGCCCCGGTGCCTGACATCCAGG
SRR940959.141022903.+ CAAGCCAGGTGGTCTCCTCCCTGCCACAGATGAACGGGGCCCCGGTGCCTGACATCCAGG
SRR940959.241939881.+ CAAGCCAGGTGGTCTCCTCCCTGCCACAGATGAACGGGGCCCCGGTGCCTGACATCCAGG
SRR940959.331596001.+ CAAGCCAGGTGGTCTCCTCCCTGCCACAGATGAACGGGGCCCCGGTGCCTGACATCCAGG
SRR940959.387467835.+ CAAGCCAGGTGGTCTCCTCCCTGCCACAGATGAACGGGGCCCCGGTGCCTGACATCCAGG
SRR940959.239980244.-  AAGCCAGGTGGTCTCCTCCCTGCCACAGATGAACGGGGCCCCGGTGCCTGACATCCAGG
SRR940959.522711930.+   AGCCAGGTGGTCTCCTCCCTGCCACAGATGAACGGGGCCCCGGTGCCTGACATCCAGG
SRR940959.6060068.2+               CTCCTCCCTGCCACAGATGAACGGGGCCCCGGTGCCTGACATCCAGG
SRR940959.52226140.2-                        GCCACAGATGAACGGGGCCCCGGTGCCTGACATCCAGG
SRR940959.158694893.-                        GCCACAGATGAACGGGGCCCCGGTGCCTGACATCCAGG
SRR940959.338270676.-                        GCCACAGATGAACGGGGCCCCGGTGCCTGACATCCAGG
SRR940959.52296932.2-                          CACATATGAACGGGGCCCCGGTTCCTGACATCCAGG
SRR940959.311225037.-                             AGATGAACGGGGCCCCGGTGCCTGACATCCAGG
SRR940959.396741421.-                             AGATGAACGGGGCCCCGGTGCCTGACATCCAGG
SRR940959.253103377.+                                                 GTGCCTNACATCCAGG
SRR940959.358940049.+                                                    CCTGACATCCAGG
SRR940959.365460759.+                                                      TGACATCCAGG
SRR940959.450806280.+                                                       GACATCCAGG
SRR940959.9713801.1+                                                         ACATCCAGG
                      ____________________________________________________________
consensus             CAAGCCAGGTGGTCTCCTCCCTGCCACAGATGAACGGGGCCCCGGTGCCTGACATCCAGG

                          .    :    .    :    .    :    .    :    .    :    .    :
SRR940959.240556384.- T
SRR940959.158694893.+ TCTGCAG
SRR940959.412058904.+ TCTGACGCTAC
SRR940959.63681231.2+ TCTGCAGCTACCTGCTCCACACCCACTTGGC
SRR940959.34938406.1+ TCTGCAGCTACCTGCTCCACACCCACTTGGCTGGCCGC
SRR940959.141022903.+ TCTGCAGCTACCTGCTCCACACCCACTTGGCTGGCCGC
SRR940959.241939881.+ TCTGCAGCTACCTGCTCCACACCCACTTGGCTGGCCGCA
SRR940959.331596001.+ TCTGCAGCTACCTGCTCCACACCCACTTGGCTGGCCGCA
```

```
SRR940959.387467835.+ TCTGCAGCTACCTGCTCCACACCCACTTGGCTGGCCGCA
SRR940959.239980244.- TCTGCAGCTACCTGCTCCACACCCACTTGGCTGGCCGCACT
SRR940959.522711930.+ TCTGCAGCTACCTGCTCCACACCCACTTGGCTGGCCGCACTC
SRR940959.6060068.2+  TCTGCAGCTACCTGCTCCACACCCACTTGGCTGGCCGCACTCTGGAGGCCGTG
SRR940959.52226140.2- TCTGCAGCTACCTGCTCCACACCCACTTGGCTGGCCGCACTCTGGAGGCCGTGCAATACA
SRR940959.158694893.- TCTGCAGCTACCTGCTCCACACCCACTTGGCTGGCCGCACTCTGGAGGCCGTGCAATACA
SRR940959.338270676.- TCTGCAGCTACCTGCTCCACACCCACTTGGCTGGCCGCACTCTGGAGGCCGTGCAATACA
SRR940959.52296932.2- TCTGCAGCTACCTGCTCCACACCCACTTGGCTGGCCGCACTCTGGAGGCCGTGCAATACA
SRR940959.311225037.- TCTGCAGCTACCTGCTCCACACCCACTTGGCTGGCCGCACTCTGGAGGCCGTGCAATACA
SRR940959.396741421.- TCTGCAGCTACCTGCTCCACACCCACTTGGCTGGCCGCACTCTGGAGGCCGTGCAATACA
SRR940959.253103377.+ TCTGCAGCTACCTGTTCCACACCCACTTGGCTGGCCGCACTCTGAAGGCCGTGCAATACA
SRR940959.358940049.+ TCTGCAGCTACCTGCTCCACACCCACTTGGCTGGCCGCACTCTGGAGGCCGTGCAATACA
SRR940959.365460759.+ TCTGCAGCTACCTGCTCCACACCCACTTGGCTGGCCGCACTCTGGAGGCCGTGCAATACA
SRR940959.450806280.+ TCTGCAGCTACCTGCTCCACACCCACTTGGCTGGCCGCACTCTGGAGGCCGTGCAATACA
SRR940959.9713801.1+  TCTGCAGCTACCTGCTCCACACCCACTTGGCTGGCCGCACTCTGGAGGCCGTGCAATACA
SRR940959.92476664.1-      AGCTACCTGCTCCACACCCACTTGGCTGGCCGCACTCTGGAGGCCGTGCNATACA
SRR940959.101468502.-              GCTCCACACCCACTTGGCTGGCCGCACTCTGGAGGCCGTGCAATACA
SRR940959.514409941.-               CTCCACCCCCACTTGGCTGGCCGCACTCTGGAGGCCGTGCAATACA
SRR940959.239635736.-                TCCACACCCACTTGGCTGGCCGCACTCTGGAGGCCGTGCAATACA
SRR940959.6170942.1-                   CACACCCACTTGGCTGGCCGCACTCTGGAGGCCGTGCAATACA
SRR940959.67600525.1-                  CACACCCACTTGGCTGGCCGCACTCTGGAGGCCGTGCAATACA
SRR940959.68351720.1-                     ACCCACTTGGCTGGCCGCACTCTGGAGGCCGTGCAATACA
SRR940959.371211889.-                     ACCCACTTGGCTGGCCGCACTCTGGAGGCCGTGCAATACA
SRR940959.470628636.-                            TGGCTGGCCGCACTCTGGAGGCCGTGCAATACA
SRR940959.516537765.+                            TGGCTGGCCGCACTCTGGAGGCCGTGCAATACA
SRR940959.306456467.+                              GCTGGCCGCACTCTGGAGGCCGTGCAATACA
SRR940959.374170115.-                                TGGCCGCACTCTGGAGGCCGTGCAATACA
SRR940959.506180826.-                                NGGCCGCACTCTGGAGGCCGTGCAATACA
SRR940959.273158372.-                                    CGCACTCTGGAGGCCGTGCAATACA
SRR940959.194918784.-                                               GGCCGTGCAATACA
                      ____________________________________________________________
consensus             TCTGCAGCTACCTGCTCCACACCCACTTGGCTGGCCGCACTCTGGAGGCCGTGCAATACA

                          .    :    .    :    .    :    .    :    .    :    .    :
SRR940959.52226140.2- GG
SRR940959.158694893.- GG
SRR940959.338270676.- GG
SRR940959.52296932.2- GGTA
SRR940959.311225037.- GGTAAGG
SRR940959.396741421.- GGTAAGG
SRR940959.253103377.+ GGTAAGGAAGACACGGATTGGAAG
SRR940959.358940049.+ GGTAAGGAAGACACGGATTGGAAGCTC
SRR940959.365460759.+ GGTAAGGAAGACACGGATTGGAAGCTCTC
SRR940959.450806280.+ GGTAAGGAAGACACGGATTGGAAGCTCTCC
SRR940959.9713801.1+  GGTAAGGAAGACACGGATTGGAAGCTCTCCC
SRR940959.92476664.1- GGTAAGGAAGACACGGATTGGAAGCTCTCCCTTCTGTATTTGGAA
SRR940959.101468502.- GGNAAGGAAGACATGGATTGGAAGCTCTCCCTTCTGTATTTGGAAATAAATGT
SRR940959.514409941.- GGTAAGGAAGACACGGATTGGAAGCTCTCCCTTCTGTATTTGGAAATAAATGTT
SRR940959.239635736.- GGTAAGGAAGACACGGATTGGAAGCTCTCCCTTCTGTATTTGGAAATAAATGTTA
SRR940959.6170942.1-  GGTAAGGAAGACACGGATTGGAAGCTCTCCCTTCTGTATTTGGAAATAAATGTTAGC
SRR940959.67600525.1- GGTAAGGAAGACACGGATTGGAAGCTCTCCCTTCTGTATTTGGAAATAAATGTTAGC
SRR940959.68351720.1- GGTAAGGAAGACACGGATTGGAAGCTCTCCCTTCTGTATTTGGAAATAAATGTTAGCAAT
SRR940959.371211889.- GGTAAGGAAGACACGGATTGGAAGCTCTCCCTTCTGTATTTGGAAATAAATGTTAGCAAT
SRR940959.470628636.- GGTAAGGAAGACACGGATTGGAAGCTCTCCCTTCTGTATTTGGAAATAAATGTTAGCAAT
SRR940959.516537765.+ GGTAAGGAAGACACGGATTGGAAGCTCTCCCTTCTGTATTTGGAAATAAATGTTAGCAAT
SRR940959.306456467.+ GGTAAGGAAGACACGGATTGGAAGCTCTCCCTTCTGTATTTGGAAATAAATGTTAGCAAT
SRR940959.374170115.- GGTAAGGAAGACACGGATTGGAAGCTCTCCCTTCTGTATTTGGAAATAAATGTTAGCAAT
SRR940959.506180826.- GGTAAGGAAGACACGGATTGGAAGCTCTCCCTTCTGTATTTGGAAATAAATGTTAGCAAT
SRR940959.273158372.- GGTAAGGAAGACACGGATTGGAAGCTCTCCCTTCTGTATTTGGAAATAAATGTTAGCAAT
SRR940959.194918784.- GGTAAGGAAGACACGGATTGGAAGCTCTCCCTTCTGTATTTGGAAATAAATGTTAGCAAT
                      ____________________________________________________________
consensus             GGTAAGGAAGACACGGATTGGAAGCTCTCCCTTCTGTATTTGGAAATAAATGTTAGCAAT

                          .    :    .    :    .    :    .    :    .    :    .    :
SRR940959.470628636.- AATTATT
SRR940959.516537765.+ AATTATT
SRR940959.306456467.+ AATTATTTA
SRR940959.374170115.- AATTATTTATT
SRR940959.506180826.- AATTATTTATT
SRR940959.273158372.- AATTATTTATTCTGC
SRR940959.194918784.- AATTATTTATTCTGCGGCATAACGTT
                      ____________________________________________________________
consensus             AATTATTTATTCTGCGGCATAACGTT
```

(9) The exon 9 sequence was predicted by assembling WGS data using CAP3. The exon 9 is marked in red.

```
                          .    :    .    :    .    :    .    :    .    :    .    :
SRR940959.34938406.2- CCGCACACGCTGCTTCTTTGTTGATAGACCCCCTCCCTGCTTCATCCTCTCCTTATTCCT
SRR940959.141022903.- CCGCACACGCTGCTTCTTTGTTGATAGACCCCCTCCCTGCTTCATCCTCTCCTTATTCCT
SRR940959.6060068.1-     CACACGCTGCTTCTTTGTTGATAGACCCCCTCCCTGCTTCATCCTCTCCTTATTCCT
SRR940959.91293546.1-           TGCATCTTTGTTGATAGACCCCCTCCCTGCTTCATCCTCTCCTTATTCCN
SRR940959.147531906.-           TGCTTCTTTGTTGATAGACCCCCTCCCTGCTTCATCCTCTCCTTATTCCT
SRR940959.365814246.-           TGCTTCTTTGTTGATAGCCCCCCTCCCTGCTTCATCCTCTCCTTATTCCT
SRR940959.441539159.-           TGCTTCTTTGTTGATAGACCCCCTCCCTGCTTCATCCTCTCCTTATTCCT
SRR940959.446898736.-           TGCTTCTTTGTTGATAGACCCCCTCCCTGCTTCATCCTCTCCTTATTCCT
SRR940959.249696441.+             CTTCTTTGTTGATAGACCCCCTCCCTGCTTCATCCTCTCCTTATTCCT
SRR940959.429902438.+                   TGTTGATAGACCCCCTCCCTGCTTCATCCTCTCCTTATTCCT
SRR940959.177483123.-                     TTGATAGACCCCCTCCCTGCTTCATCCTCTCCTTATTCCT
SRR940959.473959259.-                     TTGATAGACCCCCTCCCTGCTTCATCCTCTCCTTATTCCT
SRR940959.300370376.-                         TAGACCCCCTCCCTGCTTCATCCTCTCCTTATTCCT
SRR940959.450806280.-                                                   CCTTATTCCT
SRR940959.505974593.+                                                    CTTATTCCT
SRR940959.517825499.+                                                      TATTCCT
SRR940959.161010540.+                                                       ATTCCT
SRR940959.365187655.+                                                       ATTCCT
SRR940959.173637992.+                                                          CCT
SRR940959.242570193.+                                                          CCT
SRR940959.275457231.+                                                          CCT

consensus             CCGCACACGCTGCTTCTTTGTTGATAGACCCCCTCCCTGCTTCATCCTCTCCTTATTCCT

                          .    :    .    :    .    :    .    :    .    :    .    :
SRR940959.34938406.2- GTCCCCTCCCCTTGTCCCCCTCCAGAAATGGAACACAATG
SRR940959.141022903.- GTCCCCTCCCCTTGTCCCCCTCCAGAAATGGAACACAATG
SRR940959.6060068.1-  GTCCCCTCCCCTTGTCCCCCTCCAGAAATGGAACACAATGCCG
SRR940959.91293546.1- GTCCCCTCCCCTTGTCCCCCTCCAGAAATGGAACACAATGCCGAACAATC
SRR940959.147531906.- GTCCCCTCCCCTTGTCCCCCTCCAGAAATGGAACACAATGCCGAACAATC
SRR940959.365814246.- GTCCCCTCCCCTTGTCCCCCTCCAGAAATGGAACACAATGCCGAACAATC
SRR940959.441539159.- GTCCCCTCCCCTTGTCCCCCTCCAGAAATGGAACACAATGCCGAACAATC
SRR940959.446898736.- GTCCCCTCCCCTTGTCCCCCTCCAGAAATGGAACACAATGCCGAACAATC
SRR940959.249696441.+ GTCCCCTCCCCTTGTCCCCCTCCAGAAATGGAACACAATGCCGAACAATCTG
SRR940959.429902438.+ GTCCCCTCCCCTTGTCCCCCTCCAGAAATGGAACACAATGCCGAACAATCTGGAAAGA
SRR940959.177483123.- GTCCCCTCCCCTTGTCCCCCTCCAGAAATGGAACACAATGCCGAACAATCTGGAAAGATG
SRR940959.473959259.- GTCCCCTCCCCTTGTCCCCCTCCAGAAATGGAACACAATGCCGAACAATCTGGAAAGATG
SRR940959.300370376.- GTCCCCTCCCCTTGTCCCCCTCCAGAAATGGAACACAATGCCGAACAATCTGGAAAGATG
SRR940959.450806280.- GTCCCCTCCCCTTGTCCCCCTCCAGAAATGGAACACAATGCCGAACAATCTGGAAAGATG
SRR940959.505974593.+ GTCCCCTNCCCTTGTCCCCCTCCAGAAATGGAACACAATGCCGAACAATCTGGAAAGATG
SRR940959.517825499.+ GTCCCCTCCCCTTGTCCCCCTCCAGAAATGGAACACAATGCCGAACAATCTGGAAAGATG
SRR940959.161010540.+ GTCCCCTCCCCTTGTCCCCCTCCAGAAATGGAACACAATGCCGAACAATCTGGAAAGATG
SRR940959.365187655.+ GTCCCCTCCCCTTGTCCCCCTCCAGAAATGGAACACAATGCCGAACAATCTGGAAAGATG
SRR940959.173637992.+ GTCCCCTCCCCTTGTCCCCCTCCAGAAATGGAACACAATGCCGAACAATCTGGAAAGATG
SRR940959.242570193.+ GTCCCCTCCCCTTGTCCCCCACCAGAGATGGAACACAATGCCGAACAATCTGGAAAGATG
SRR940959.275457231.+ GTCCCCTCCCCTTGTCCCCCTCCAGAAATGGAACACAATGCCGAACAATCTGGAAAGATG
SRR940959.9713801.2-  GTCCCCTCCCCTTGTCCCCCTCCAGAAATGGAACACAATGCCGAACAATCTGGAAAGATG
SRR940959.133294881.+   CCCCTCCCCTTGTCCCCCTCCAGAAATGGAACACAATGCCGAACAATCTGGAAAGATG
SRR940959.365460759.-   CCCCTCCCCTTGTCCCCCTCCAGAAATGGAACACAATGCCGAACAATCTGGAAAGATG
SRR940959.306456467.-      CTCCCCTTGTCCCCCTCCAGAAATGGAACACAATGCCGAACAATCTGGAAAGATG
SRR940959.456908967.-      CTCCCCTTGTCCCCCTCCAGAAATGGAACACAATGCCGAACAATCTGGAAAGATG
SRR940959.348164396.+        CCCCTTGTCCCCCTCCAGAAATGGAACACAATGCCGAACAATCTGGAAAGATG
SRR940959.45460800.1+           CTTGTCCCCCTCCAGAAATGGAACACAATGCCGAACAATCTGGAAAGATG
SRR940959.179518450.+           CTTGTCCCCCTCCAGAAATGGAACACAATGCCGAACAATCTGGAAAGATG
SRR940959.178062693.+               TCCCCCTCCAGAAATGGAACACAATGCCGAACAATCTGGAAAGATG
SRR940959.477533356.+               TCCCCCTCCAGAAATGGAACACAATGCCGAACAATCTGGAAAGATG
SRR940959.127184377.+                          AAATGGAACACAATGCCGAACAATCTGGAAAGATG
SRR940959.254721899.+                          AAATGGNACACAATGCCGAACAATCTGGAATGATG
SRR940959.143983109.+                               GAACACAATGCCGAACAATCTGGAAAGATG
SRR940959.241588087.+                               GAACACAATGCCGAACAATCTGGAAAGATG
SRR940959.447749060.-                               GAACACAATGCCGAACAATCTGGAAAGATG
SRR940959.435677203.+                                     AATGCCGAACAATCTGGAAAGATG
SRR940959.13054656.2+                                      ATGCCGAACAATCTGGAAAGATG
SRR940959.189077901.+                                           GAACAATCTGGAAAGATG
SRR940959.154638866.+                                                ATCTGGAAAGATG
SRR940959.262416565.-                                                ATCTGGAAAGATG
SRR940959.474281692.-                                                  CTGGAAAGATG
SRR940959.501557890.-                                                  CTGGATAGATG
SRR940959.387514720.+                                                      AAAGATG
SRR940959.328501364.+                                                        AGATG
SRR940959.516537765.-                                                            G

consensus             GTCCCCTCCCCTTGTCCCCCTCCAGAAATGGAACACAATGCCGAACAATCTGGAAAGATG

                          .    :    .    :    .    :    .    :    .    :    .    :
SRR940959.300370376.- ATTC
```

```
SRR940959.450806280.- ATTCCTATGACTTGAATCTGCAGGAGACTC
SRR940959.505974593.+ ATTCCTATGACTTGAATCTGCAGGAGACTCG
SRR940959.517825499.+ ATTCCTATGACTTGAATCTGCAGGAGACTCGAG
SRR940959.161010540.+ ATTCCTATGACTTGAATCTGCAGGAGACTCGAGA
SRR940959.365187655.+ ATTCCTATGACTTGAATCTGCAGGAGACTCGAGA
SRR940959.173637992.+ ATTCCTATGACTTGAATCTGCAGGAGACTCGAGATTT
SRR940959.242570193.+ ATTCCTATGACTTGAATCTGCAGGAGACTCGAGATTT
SRR940959.275457231.+ ATTCCTATGACTTGAATCTGCAGGAGACTCGAGATTT
SRR940959.9713801.2-  ATTCCTATGACTTGAATCTGCAGGAGACTCGAGATTTACC
SRR940959.133294881.+ ATTCCTATGACTTGAATCTGCAGGAGACTCGAGATTTACCTT
SRR940959.365460759.- ATTCCTATGACTTGAATCTGCAGGAGACTCGAGATTTACCTT
SRR940959.306456467.- ATTCCTATGACTTGAATCTGCAGGAGACTCGAGATTTACCTTCTT
SRR940959.456908967.- ATTCCTATGACTTGAATCTGCAGGAGACTCGAGATTTACCTTCTT
SRR940959.348164396.+ ATTCCTATGACTTGAATCTGCAGGAGACTCGAGATTTACCTTCTTGA
SRR940959.45460800.1+ ATTCCTATGACTTGAATCTGCAGGAGACTCGAGATTTACCTTCTTGAGTG
SRR940959.179518450.+ ATTCCTATGACTTGAATCTGCAGGAGACTCGAGATTTACCTTCTTGAGTG
SRR940959.178062693.+ ATTCCTATGACTTGAATCTGCAGGAGACTCGAGATTTACCTTCTTGAGTGGCCA
SRR940959.477533356.+ ATTCCTATGACTTGAATCTGCAGGAGACTCGAGATTTACCTTCTTGAGTGGCCA
SRR940959.127184377.+ ATTCCTATGACTTGAATCTGCAGGAGACTCGAGATTTACCTTCTTGAGTGGCCATCAAGC
SRR940959.254721899.+ ATTCCTATGACTTGAATCTGCAGGATACTCGAGATTTACCTTCTTGAGTGGCCATCAAGC
SRR940959.143983109.+ ATTCCTATGACTTGAATCTGCAGGAGACTCGAGATTTACCTTCTTGAGTGGCCATCAAGC
SRR940959.241588087.+ ATTCCTATGACTTGAATCTGTAGGAGACTCGAGATTTGCCTTCTTGAGTGGCCATCAAGC
SRR940959.447749060.- ATTCCTATGACTTGAATCTGCAGGAGACTCGAGATTTACCTTCTTGAGTGGCCATCAAGC
SRR940959.435677203.+ ATTCCTATGACTTGAATCTGCAGGAGACTCGAGATTTACCTTCTTGAGTGGCCATCAAGC
SRR940959.13054656.2+ ATTCCTATGACTTGAATCTGCAGGAGACTCGAGATTTACCTTCTTGAGTGGCCATCAAGC
SRR940959.189077901.+ ATTCCTATGACTTGAATCTGCAGGAGACTCGAGATTTACCTTCTTGAGTGGCCATCAAGC
SRR940959.154638866.+ ATTCCTATGACTTGAATCTGCAGGAGACTCGAGATTTACCTTCTTGAGTGGCCATCAAGC
SRR940959.262416565.- ATTCCTATGACTTGAATCTGCAGGAGACTCGAGATTTACCTTCTTGAGTGGCCATCAAGC
SRR940959.474281692.- ATTCCTATGACTTGAATCTGCAGGAGACTCGAGATTTACCTTCTTGAGTGGCCATCAAGC
SRR940959.501557890.- ATTCCTATGACTTGAATCTGCAGGAGACTCGAGATTTACCTTCTTGAGTGGCCATNAAGC
SRR940959.387514720.+ ATTCCTATGACTTGAATCTGCAGGAGACTCGAGATTTACCTTCTTGAGTGGCCATCAAGC
SRR940959.328501364.+ ATTCCTATGACTTGAATCTGCAGGAGACTCGAGATTTACCTTCTTGAGTGGCCATCAAGC
SRR940959.516537765.- ATTCCTATGACTTGAATCTGCAGGAGACTCGAGATTTACCTTCTTGAGTGGCCATCAAGC
SRR940959.188304483.+         GACTTGAATCTGCAGGAGACTCGAGATTTACCTTCTTGAGTGGCCATCAAGC
SRR940959.304837636.+         GACTTGAATCTGCAGGAGACTCGAGATTTACCTTCTTGAGTGGCCATCAAGC
SRR940959.376600794.+         GACTTGAATCTGCAGGAGACTCGAGATTTACCTTCTTGAGTGGCCATCAAGC
SRR940959.80465343.1+          ACTTGAATCTGCAGGAGACTCGAGATTTACCTTCTTGAGTGGCCATCAAGN
SRR940959.130853508.+               AATCTGCAGGAGACTCGAGATTTACCTTCTTGAGTGGCCATCAAGC
SRR940959.177098779.-                   TGCAGGAGCCTCTAGATTTACCTTCTTGAGTGGCCATCAAGC
SRR940959.78501673.1+                      AGGAGACTCGAGATTTACCTTCTTGAGTGGCCATCAAGC
SRR940959.474055874.-                      AGGAGACTCGAGATTTACCTTCTTGAGTGGCCATCAAGC
SRR940959.263181599.+                       GGAGACTCGAGATTTACCTTCTTGAGTGGCCATCAAGC
SRR940959.330135010.+                       GGAGACTCGAGATTTACCTTCTTGAGTGGCCATCAAGC
SRR940959.70882250.2+                           ACTCGAGATTTACCTTCTTGAGTGGCCATCAAGC
SRR940959.209589420.+                                AGATTTACCTTCTTGAGTGGCCATCAAGC
SRR940959.495402927.+                                AGATTTACCTTCTTGAGTGGCCATCAAGC
SRR940959.422487225.+                                   TTTACCTTCTTGAGTGGCCATCAAGC
SRR940959.148495904.+                                     TACCTTCTTGAGTGGCCATCAAGC
SRR940959.180366586.+                                      ACCTTCTTGAGTGGCCATCAAGC
SRR940959.518528208.+                                      ACCTTCTTGAGTGGCCATCAAGC
SRR940959.43947546.1+                                       CCTTCTTGAGTGGCCATCAAGC
SRR940959.226992409.-                                        CTTTTTGAGTGGCCATCAAGC
SRR940959.45621832.1-                                            TTGAGTGGCCATCAAGC
SRR940959.261766996.+                                             TGAGTGGCCATCAAGC
SRR940959.282561611.+                                             TGAGTGGCCATCAAGC
SRR940959.490602461.-                                             CGAGTGGCCTTAAAGC
SRR940959.206664363.+                                                 TGGCCATCAAGC
                      ____________________________________________________________
consensus             ATTCCTATGACTTGAATCTGCAGGAGACTCGAGATTTACCTTCTTGAGTGGCCATCAAGC

                          .    :    .    :    .    :    .    :    .    :    .    :
SRR940959.127184377.+ CAGTG
SRR940959.254721899.+ CAGTG
SRR940959.143983109.+ CAGCGGTAGC
SRR940959.241588087.+ CAGTGGTAGC
SRR940959.447749060.- CAGTGGTAGC
SRR940959.435677203.+ CAGTGGTAGCCTGGGG
SRR940959.13054656.2+ CAGTGGTAGCCTGGGGG
SRR940959.189077901.+ CAGTGGTAGCCTGGGGGGAGGC
SRR940959.154638866.+ CAGTGGTAGCCTGGGGGGAGGCATGGC
SRR940959.262416565.- CAGTGGTAGCCTGGGGGGAGGCATGGC
SRR940959.474281692.- CAGTGGTAGCCTGGGGGGAGGCATGGCTG
SRR940959.501557890.- CAGTGGTAGCCTNGGGGGAGGCATGGCTG
SRR940959.387514720.+ CAGTGGTAGCCTGGGGGGAGGCATGGCTGGCAT
SRR940959.328501364.+ CAGTGGTAGCCTGGGGGGAGGCATGGCTGGCATTG
SRR940959.516537765.- CAGTGGTAGCCTGGGGGGAGGCATGGCTGGCATTGGGGG
SRR940959.188304483.+ CAGTGGTAGCCTGGGGGGAGGCATGGCTGGCATTGGGGGAAGGCAGGG
```

```
SRR940959.304837636.+ CAGTGGTAGCCTGGGGGGAGGCATGGCTGGCATTGGGGGAAGGCAGGG
SRR940959.376600794.+ CAGTGGTAGCCTGGGGGGAGGCATGGCTGGCATTGGGGGAAGGCAGGG
SRR940959.80465343.1+ CAGTGGTAGCCTGGGGGGAGGCATGGCTGGCATTGGGGGAAGGCAGGGT
SRR940959.130853508.+ CAGTGGTAGCCTGGGGGGAGGCATGGCTGGCCTTGGCGGAAGGCAGGGCCAGCA
SRR940959.177098779.- CAGTGGTAGCCTGGGGGGAGGCATGGCTGGCATTGGGGGAAGGCAGGGTCAGCACTCA
SRR940959.78501673.1+ CAGTGGTAGCCTGGGGGGAGGCATGGCTGGCATTGGGGGAAGGCAGGGTCAGCACTCACT
SRR940959.474055874.- CAGTGGTAGCCTGGGGGGAGGCATGGCTGGCATTGGGGGAAGGCAGGGTCAGCACTCACT
SRR940959.263181599.+ CAGTGGTAGCCTGGGGGGAGGCATGGCTGGCATTGGGGGAAGGCAGGGTCAGCACTCACT
SRR940959.330135010.+ CAGTGGCAGCCTGGGGGGAGGCATGGCTGGCATTGGGGGAAGGCAGGGTCAGCACTCACT
SRR940959.70882250.2+ CAGTGGTAGCCTGGGGGGAGGCATGGCTGGCATTGGGGGAAGGCAGGGTCAGCCCTCACT
SRR940959.209589420.+ CAGTGGTAGCCTGGGGGGAGGCATGGCTGGCATTGGGGGAAGGCAGGGTCAGCACTCACT
SRR940959.495402927.+ GAGTGGTAGCCTGGGGGGAGGCATGGCTGGCATTGGGGGAAGGCAGGGTCAGCACTCACT
SRR940959.422487225.+ CAGTGGTAGCCTGGGGGGAGGCATGGCTGGCATTGGGGGAAGGCAGGGTCAGCACTCACT
SRR940959.148495904.+ CAGTGGTAGCCTGGGGGGAGGCATGGCTGGCATTGGGGGAAGGCAGGGTCAGCACTCACT
SRR940959.180366586.+ CAGTGGTAGCCTGGGGGGAGGCATGGCTGGCATTGGGGGAAGGCAGGGTCAGCACTCACT
SRR940959.518528208.+ CAGTGGTAGCCTGGGGGGAGGCATGGCTGGCATTGGGGGAAGGCAGGGTCAGCACTCACT
SRR940959.43947546.1+ CAGTGGTAGCCTGGGGGGAGGCATGGCTGGCATTGGGGGAAGGCAGGGTCAGCACTCACT
SRR940959.226992409.- CAGTGGTAGCCTGTGGGGAGGCATGGCTGGCATTGGGGGAAGGCAGGGTCAGCACTCACT
SRR940959.45621832.1- CAGTGGTAGCCTGGGGGGAGGCATGGCTGGCATTGGGGGAAGGCAGGGTCAGCACTCACT
SRR940959.261766996.+ CAGTGGTAGCCTGGGGGGAGGCATGGCTGGCATTGGGGGAAGGCAGGCTCAGCACTCACT
SRR940959.282561611.+ CAGTGGTAGCCTGGGGGGAGGCATGGCTGGCATTGGGGGAAGGCAGGGTCAGCACTCACT
SRR940959.490602461.- CAGTGGTAGCCTGGGGGGAGGCATGGCTGGCATTGGGGGAAGGCAGGGTCAGCACTCCCT
SRR940959.206664363.+ CAGTGGTAGCCTGGGGGGAGGCATGGCTGGCATTGGGGGAAGGCAGGGTCAGCACTCACT
                      ____________________________________________________________
consensus             CAGTGGTAGCCTGGGGGGAGGCATGGCTGGCATTGGGGGAAGGCAGGGTCAGCACTCACT

                          .    :    .    :    .    :    .    :    .    :    .    :
SRR940959.78501673.1+ A
SRR940959.474055874.- A
SRR940959.263181599.+ AC
SRR940959.330135010.+ AC
SRR940959.70882250.2+ ACTGTT
SRR940959.209589420.+ ACTGTTAGCCA
SRR940959.495402927.+ ACTGTTAGCCA
SRR940959.422487225.+ ACTGTTAGCCAAGA
SRR940959.148495904.+ ACTGTTAGCCAAGAGG
SRR940959.180366586.+ ACTGTTAGCCAAGAGGA
SRR940959.518528208.+ ACTGTTAGCCAAGAGGA
SRR940959.43947546.1+ ACTGTTAGCCAAGAGGAC
SRR940959.226992409.- ACTGTTAGCCAAGAGGACT
SRR940959.45621832.1- ACTGTTAGCCAAGAGGACTGTAC
SRR940959.261766996.+ ACTGTTAGCCAAGAGGACTGTACC
SRR940959.282561611.+ ACTGTTAGCCAAGAGGACTGTACC
SRR940959.490602461.- ACTGTTAGCCAAGAGGACTGTACC
SRR940959.206664363.+ ACTGTTAGCCAAGAGGACTGTACCAGTC
                      ____________________________________________________________
consensus             ACTGTTAGCCAAGAGGACTGTACCAGTC
```

(10) The exon 10 sequence was predicted by assembling WGS data using CAP3. The exon 10 is marked in red.

```
                          .    :    .    :    .    :    .    :    .    :    .    :
SRR940959.392839694.+ AAGGCAGGGTCAGCACTCACTACTGTTAGCCAAGAGGACTGTACCAGTCCCTCTGTGTCT
SRR940959.475728160.-   GGCAGGGTCAGCACTCACTACTGTTAGCCAAGAGGACTGTACCAGTCCCTCTGTGTCT
SRR940959.221082947.-      AGGGCCAGCACTCACTACTGTTAGCCAAGAGGACTGTACCAGTCCCTCTGTGTCT
SRR940959.269432814.+       GGGTCAGCACTCACTACTGTTAGCCAAGAGGACTGTACCAGTCCCTCTGTGTCT
SRR940959.334810478.-         GTCAGCACTCACTACTGTTAGCCAAGAGGACTGTACCAGTCCCTCTGTGTCT
SRR940959.188304483.-            AGCACTCACTACTGTTAGCCAAGAGGACTGTACCAGTCCCTCTGTGTCT
SRR940959.304837636.-            AGCACTCACTACTGTTAGCCAAGAGGACTGTACCAGTCCCTCTGTGTCT
SRR940959.376600794.-            AGCACTCACTACTGTTAGCCAAGAGGACTGTACCAGTCCCTCTGTGTCT
SRR940959.483038640.-             GCACTCACTACTGTTAGCCAAGAGGACTGTACCAGTCCCTCTGTGTCT
SRR940959.97160335.2-               ACTCACTACTGTTAGCCAAGTGGACTGTACCAGTCCCTCTGTGTCT
SRR940959.260875164.-               ACTCACGACTGTTAGCCAAGAGGACTGTACCAGTCCCTCCGTGTCT
SRR940959.345595402.-                  CACTTCTGTTAGCCAAGAGGACTGTACCAGTGGCTCCGTGTCT
SRR940959.905867.1-                        ACTGTTAGCCAAGAGGACTGTACCAGTCCCTCTGTGTCT
SRR940959.236855865.-                      ACTGTTAGCCAAGAGGACTGTACCAGTCCCTCTGTGTCT
SRR940959.483358530.-                        TGTTAGCCAAGAGGACTGTACCAGTCCCTCTGTGTCT
SRR940959.48415732.2+                            AGCCAAGAGGACTGTACCAGTCCCTCTGTGTCT
SRR940959.494269013.+                            AGCCAAGAGGACTGTACCAGTCCCTCTGTGTCT
SRR940959.701105.2-                                CCAAGAGGACTGTACCAGTCCCTCTGTGTCT
SRR940959.475693911.-                                          TACCAGTCCCTCTGTGTCT
SRR940959.172492863.-                                            CCAGTCCCTCTGTGTCT
SRR940959.202247812.+                                              AGTCCCTCTGTGTCT
SRR940959.171022350.-                                                     CTGTGTCT
SRR940959.277806410.-                                                     CTGTGTCT
SRR940959.236694715.+                                                           CT
```

```
SRR940959.269014775.+                                                           CT
SRR940959.98695753.1-                                                            T
                      ____________________________________________________________
consensus             AAGGCAGGGTCAGCACTCACTACTGTTAGCCAAGAGGACTGTACCAGTCCCTCTGTGTCT

                          .    :    .    :    .    :    .    :    .    :    .    :
SRR940959.392839694.+ TACTTTGAGCCTTTGTTTCGCTCTGCACAGGGAGATGAAT
SRR940959.475728160.- TACTTTGAGCCTTTGTTTCGCTCTGCACAGGGAGATGAATTG
SRR940959.221082947.- TACTTTGAGCCTTTGTTTCGCTCTGCACAGGGAGATGAATTGTTG
SRR940959.269432814.+ TACTTTGAGCCTTTGTTTCGCTCTGCACAGGGAGATGAATTGTTGG
SRR940959.334810478.- TACTTTGAGCCTTTGTTTCGCTCTGCACAGGGAGATGAATTGTTGGTA
SRR940959.188304483.- TACTTTGAGCCTTTGTTTCGCTCTGCACAGGGAGATGAATTGTTGGTAGGG
SRR940959.304837636.- TACTTTGAGCCTTTGTTTCGCTCTGCACGGGGAGATGAATTGTTGGTAGGG
SRR940959.376600794.- TACTTTGAGCCTTTGTTTCGCTCTGCACAGGGAGATGAATTGTTGGTAGGG
SRR940959.483038640.- TACTTTGAGCCTTTGTTTCGCTCTGCACAGGGAGATGAATTGTTGGTAGGGT
SRR940959.97160335.2- TACTTTGAGCCTTTGTTGCGCTCTGCACAGGGAGATGAATTGTTGGTAGGGTGT
SRR940959.260875164.- TCCTTTGAGCCTTTGTTTCGCTCCGCACAGGGAGATGAATTGTTGGTNGGGTGT
SRR940959.345595402.- TACTTTGAGCCTTTGTTTCGCTCTGCACAGGGAGATGAATTGTTGGTAGGGTGTCAC
SRR940959.905867.1-   TACTTTGAGCCTTTGTTTCGCTCTGCACAGGGAGATGAATTGTTGGTAGGGTGTCACTAC
SRR940959.236855865.- TACTTTGAGCCTTTGTTTCGCTCTGCACAGGGAGATGAATTGTTGGTAGGGTGTCACTAC
SRR940959.483358530.- TACTTTGAGCCTTTGTTTCGCTCTGCACAGGGAGATGAATTGTTGGTAGGGTGTCACTAC
SRR940959.48415732.2+ TACTTTGAGCCTTTGTTTCGCTCTGCACAGGGAGATGAATTGTTGGTAGGGTGTCACTTC
SRR940959.494269013.+ TACTTTGAGCCTTTGTTTCGCTCTGCACAGGGAGATGAATTGTTGGTAGGGTGTCACTTC
SRR940959.701105.2-   TACTTTGAGCCTTTGTTTCGCTCTGCACAGGGAGATGAATTGTTGGTAGGGTGTCACTAC
SRR940959.475693911.- TACTTTGAGCCTTTGTTTCGCTCTGCACAGGGAGATGAATTGTTGGTAGGGTGTCACTAC
SRR940959.172492863.- TACTTTGAGCCTTTGTTTCGCTCTGCACAGGGAGATGAATTGTTGGTAGGGTGTCACTAC
SRR940959.202247812.+ TACTTTGAGCCTTTGTTTCGCTCTGCACAGGGAGATGAATTGTTGGTAGGGTGTCACTAC
SRR940959.171022350.- TACTTTGAGCCTTTGTTTCGCTCTGCACAGGGAGATGAATTGTTGGTAGGGTGTCACTAC
SRR940959.277806410.- TACTTTGAGCCTTTGTTTCGCTCTGCACAGGGAGATGAATTGTTGGTAGGGTGTCACTAC
SRR940959.236694715.+ TACTTTGAGCCTTTGTTTCGCTCTGCACAGGGAGATGAATTGTTGGTAGGATGTCACTAC
SRR940959.269014775.+ TACTTTGAGCCTTTGTTTCGCTCTGCACAGGGAGATGAATTATTGGGACGGTGTCACTAC
SRR940959.98695753.1- TACTTTGAGCCTTTGTTTCGCTCTGCACAGGGAGATGAATTGTTGGTANGGTGTCACTAC
SRR940959.182030864.-       GAGCCTTTGTTTCGCTCTGCACAGGGAGATGAATTGTTGGTAGGGTGTCACTAC
SRR940959.147851778.-        AGCCTTTGTTTCGCTCTGCACAGGGAGATGAATTGTTGGTAGGGTGTCACTAC
SRR940959.410789150.-          CCTTTGTTTCGCTCTGCACAGGGAGATGAATTGTTGGTAGGGTGTAACTAC
SRR940959.57890170.2+           CTTTGTTTCGCTCTGCACAGGGAGATGAATTGTTGGTAGGGTGTCACTAC
SRR940959.426473175.+           CTTTGTTTCGCTCTGCACAGGGAGATGAGTTGTTGGTAGGGTGTCACTAC
SRR940959.249696441.-            TTTGTTTCGCTCTGCACAGGGAGATGAATTGTTGGTAGGGTGTCACTAC
SRR940959.316756815.+               GTTTCGCTCTGCACAGGGAGATGAATTGTTGGTAGGGTGTCACTAC
SRR940959.455129004.+                         GCACAGGGAGATGAATTGTTGGTAGGGTGTCACTAC
SRR940959.313774269.+                               GGAGATGAATTGTTGGTAGGGTGTCACTAC
SRR940959.370419265.+                               GGAGATGAATTGTTGGTAGGGTGTCACTAC
SRR940959.478870080.+                               GGAGATGAATTGTTGGTAGGGTGTCACTAC
SRR940959.317590714.+                                 AGATGAATTGTTGGTAGGGTGTCACTAC
SRR940959.144010641.+                                   ATGAATTGTTGGTAGGGTGTCACTAC
SRR940959.177348727.+                                   ATGAATTGTTGGTAGGGTGTCACTAC
SRR940959.401629924.+                                     GAATTGTTGGTAGGGTGTCACTAC
SRR940959.436203243.+                                     GAATTGTTGGTAGGGTGTCACTAC
SRR940959.464912229.+                                     GAATTGTTGGTAGGGTGTCACTAC
SRR940959.86983223.2+                                       ATTGTTGGTAGGGTGTCACTAC
SRR940959.83525293.2+                                        TTGTTGGTAGGGTGTCACTAC
SRR940959.522937194.+                                        TTGTTGGTAGGGTGTCACTAC
SRR940959.123481887.+                                             GGTAGGGTGTCACTAC
SRR940959.344163245.+                                                  GGTGTCACTAC
SRR940959.373040488.+                                                  GGTGTCACTAC
SRR940959.11270753.2+                                                        CACTAC
SRR940959.450213670.+                                                        CACTAC
SRR940959.269014775.-                                                           TCC
SRR940959.392839694.-                                                            AC
                      ____________________________________________________________
consensus             TACTTTGAGCCTTTGTTTCGCTCTGCACAGGGAGATGAATTGTTGGTAGGGTGTCACTAC

                          .    :    .    :    .    :    .    :    .    :    .    :
SRR940959.905867.1-   C
SRR940959.236855865.- C
SRR940959.483358530.- CAG
SRR940959.48415732.2+ CAGACGC
SRR940959.494269013.+ CAGACGC
SRR940959.701105.2-   CAGACGCTG
SRR940959.475693911.- CAGACGCTGGACCGTGACTCC
SRR940959.172492863.- CAGACGCTGGACCGTGACTCCTT
SRR940959.202247812.+ CAGACGCTGGACCGTGACTCCTTGA
SRR940959.171022350.- CAGACGCTGGACCGTGACTCCTTGACTTTTGT
SRR940959.277806410.- CAGACGCTGGACCGTGACTCCTTGACTTTTGT
SRR940959.236694715.+ CAGACGCTGGACGGTGACTCCTTGACTTTTGTAAGTAC
SRR940959.269014775.+ CAGACGCTGGACCGTGACTCCTTGACTTTTGTAAGTAC
SRR940959.98695753.1- CAGACGCTGGACCGTGACTCCTTGACTTTTGTAAGTACC
```

```
SRR940959.182030864.- CAGACGCTGGACCGTGACTCCTTGACTTTTGTAAGTACCTCTCTCC
SRR940959.147851778.- CAGACGCTGGACCGTGACTCCTTGACTTTTGTAAGTACCTCTCTCCC
SRR940959.410789150.- CAGACGCTGGACCGTGACTCCTTGACTTTTGTAAGTCCCTCTCTCCCCA
SRR940959.57890170.2+ CAGACGCTGGACCGTGACTCCTTGACTTTTGTAAGTACCTCTCTCCCCAT
SRR940959.426473175.+ CAGACGCTGGACCGTGACTCCTTGACTTATGTAAGTACCTCTCTACCCAT
SRR940959.249696441.- CAGACGCTGGACCGTGACTCCTTGACTTTTGTAAGTACCTCTCTCCCCATC
SRR940959.316756815.+ CAGACGCTGGACCGTGACTCCTTGACTTTTGTAAGTACCTCTCTCCCCATCGTG
SRR940959.455129004.+ CAGACGCTGGACCGTGACTCCTTGACTTTTGTAAGTACCTCTCTCCCCATCGTGGTCAAC
SRR940959.313774269.+ CAGACGCTGGACCGTGACTCCTTGACTTTTGTAAGTACCTCTCTCCCCATCGTGGTCAAC
SRR940959.370419265.+ CAGACGCTGGACCGTGACTCCTTGACTTTTGTAAGTACCTCTCTCCCCATCGTGGTCAAC
SRR940959.478870080.+ CAGACGCTGGACCGTGACTCCTTGACTTTTGTAAGTACCTCTCTCCCCATCGTGGTCAAC
SRR940959.317590714.+ CAGACGCTGGACCGTGACTCCTTGACTTTTGTAAGTACCTCTCTCCCCATCGTGGTCAAC
SRR940959.144010641.+ CAGACGCTGGACCGTGACTCCTTGACTTTTGTAAGTACCTCTCTCCCCATCGTGGTCAAC
SRR940959.177348727.+ CAGACGCTGGACCGTGACTCCTTGACTTTTGTAAGTACCTCTCTCCCCATCGTGGTCAAC
SRR940959.401629924.+ CAGACGCTGGACCGTGACTCCTTGACTTTTGTAAGTACCTCTCTCCCCATCGTGGTCAAC
SRR940959.436203243.+ CAGACGCTGGACCGTGACTCCTTGACTTTTGTAAGTACCTCTCTACCCATCGTGGTCAAC
SRR940959.464912229.+ CAGACGCTGGACCGTGACTCCTTGACTTTTGTAAGTACCTCTCTCCCCATCGTGGTCAAC
SRR940959.86983223.2+ CAGACGCTGGACCGTGACTCCTTGACTTTTGTAAGTACCTCTCTCCCCATCGTGGTCAAC
SRR940959.83525293.2+ CAGACGCTGGACCGTGACTCCTTGACTTTTGTAAGTACCTCTCTCCCCATCGTGGTCAAC
SRR940959.522937194.+ CAGACGCTGGACCGTGACTCCTTGACTTTTGTAAGTACCTCTCTCCCCATCGTGGTCAAC
SRR940959.123481887.+ CAGACGCTGGACCGTGACTCCTTGACTTTTGTAAGTACCTCTCTCCCCATCGTGGTCAAC
SRR940959.344163245.+ CAGACGCTGGACCGTGACTCCTTGACTTTTGTAAGTACCTCTCTCCCCATCGTGGTCAAC
SRR940959.373040488.+ CAGACGCTGGACCGTGACTCCTTGACTTTTGTAAGTACCTCTCTCCCCATCGTGGTCAAC
SRR940959.11270753.2+ CAGACGCTGGACCGTGACTCCTTGACTTTTGTAAGTACCTCTCTCCCCATCGTGGTCAAC
SRR940959.450213670.+ CAGACGCTGGACCGTGACTCCTTGACTTTTGTAAGTACCTCTCTCCCCATCGTGGTCAAC
SRR940959.269014775.- CAGACTCTGGACCATGACTCCTTGACTTTTGTAAGTACCTCTCTCCCCATCGTGGTCAAC
SRR940959.392839694.- CAGACGCTGGACCGTGACTCCTTGACTTTTGTAAGTACCTCTCTCCCCATCGTGGTCAAC
SRR940959.200855360.+  AGACGCTGGACCGTGACTCCTTGACTTTTGTAAGTACCTCTCTCCCCATCGTGGTCAAC
SRR940959.173637992.-           ACCGTGACTCCTTGACTTTTGTAAGTACCTCTCTCCCCATCGTGGTCAAC
SRR940959.242570193.-           ACCGTGACTCCTTGACTTTTGTAAGTGCCTCCCTCCCCATCGTGGTCAAT
SRR940959.275457231.-           ACCGTGACTCCTTGACTTTTGTAAGTACCTCTCTCCCCATCGTGGTCAAC
SRR940959.219331416.+             CGTGACTCCTTGACTTTTGTAAGTACCTCTCTCCCCATCGTGGTCAAC
SRR940959.383627855.+                 ACTCCTTGACTTTTGTAAGTACCTCTCTCCCCATCGTGGTCAAC
SRR940959.76631941.2+                    CCTTGACTTTTGTAAGTACCTCTCTCCCCATCGTGGTCAAC
SRR940959.358665117.+                    CCTTGACTTTTGTAAGTACCTCTCTCCCCATCGTGGTCAAC
SRR940959.295524519.+                    CCTTGACTTTTGTAAGTACCTCTCTCCCCATCGTGGTCAAC
SRR940959.264717641.+                    CCTTGACTTTTGTAAGTACCTCTCTCCCCATCGTGGTCAAC
SRR940959.259102535.+                    CCTTGACTTTTGTAAGTACCTCTCTCCCCATCGTGGTCAAC
SRR940959.462782317.+                    CCTTGACTTTTGTAAGTACCTCTCTCCCCATCGTGGTCAAC
                      ____________________________________________________________
consensus             CAGACGCTGGACCGTGACTCCTTGACTTTTGTAAGTACCTCTCTCCCCATCGTGGTCAAC

                          .    :    .    :    .    :    .    :    .    :    .    :
SRR940959.455129004.+ GACA
SRR940959.313774269.+ GACAGTAGCC
SRR940959.370419265.+ GACAGTAGCC
SRR940959.478870080.+ GACAGTAGCC
SRR940959.317590714.+ GACAGTAGCCTC
SRR940959.144010641.+ GACAGTAGCCTCAG
SRR940959.177348727.+ GACAGTAGCCTCAG
SRR940959.401629924.+ GACAGTAGCCTCCGAA
SRR940959.436203243.+ GACAGTAGCCTCAGAA
SRR940959.464912229.+ GACAGTAGCCTCAGAA
SRR940959.86983223.2+ GACAGTAGCCTCAGAATA
SRR940959.83525293.2+ GACAGTAGCCTCAGAATAG
SRR940959.522937194.+ GACAGTAGCCTCAGAATAG
SRR940959.123481887.+ GACAGTAGCCTCAGAATAGAACCC
SRR940959.344163245.+ GACAGTAGCCTCAGAATAGAACCCCATCC
SRR940959.373040488.+ GACAGTAGCCTCAGAATAGAACCCCATCC
SRR940959.11270753.2+ GACAGTAGCCTCAGAATAGAACCCCATCCCTGGC
SRR940959.450213670.+ GACAGTAGCCTTAGAATAGAACCCCATCCCTGGC
SRR940959.269014775.- GACAGTTTCCTCAGAATAGAACCCCATCCCNGGCCTT
SRR940959.392839694.- GACAGTAGCCTCAGAATAGAACCCCATCCCTGGCCTCT
SRR940959.200855360.+ GACAGTAGCCTCAGAATAGAACCCCATCCCTGGCCTTGGCC
SRR940959.173637992.- GACAGTAGCCTCAGAATAGAACCCCATCCCTGGCCTTGGCCATCACGTGA
SRR940959.242570193.- GACAGTAGCCTCAGAATAGAACCCCATCCCTGGCTTTGGCCATCACGTGA
SRR940959.275457231.- GACAGTAGCCTCAGAATAGAACCCCATCCCTGGCCTTGGCCATCACGTGA
SRR940959.219331416.+ GACAGTAGCCTCAGAATAGAACCCCATCCCTGGCCTTGGCCATCACGTGACT
SRR940959.383627855.+ GACAGTAGCCTCAGAATAGAACCCCATCCCTGGCCTTGGCCATCACGTGACTCAGA
SRR940959.76631941.2+ GACAGTAGCCTCAGAATAGAACCCCATCCCTGGCCTTGGCCATCACGTGACTCAGATTA
SRR940959.358665117.+ GACAGTAGCCTCAGAATAGAACCCCATCCCTGGCCTTGGCCATCACGTGACTCAGATTA
SRR940959.295524519.+ GACAGTAGCCTCAGAATAGAACCCCATCCCTGGCCTTGGCCATCACGTGACTCAGATTA
SRR940959.264717641.+ GACAGTAGCCTCAGAATAGAACCCCATCCCTGGCCTTGGCCATCACGTGACTCAGATTA
SRR940959.259102535.+ GACAGTAGCCTCAGAATAGAACCCCATCCCTGGCCTTGGCCATCACGTGACTCAGATTA
SRR940959.462782317.+ GACAGTAGCCTCAGAATAGAACCCCATCCCTGGCCTTGGCCATCACGTGACTCAGATTA
                      ____________________________________________________________
consensus             GACAGTAGCCTCAGAATAGAACCCCATCCCTGGCCTTGGCCATCACGTGACTCAGATTA
```

(11) The exon 11 sequence was predicted by assembling WGS data using CAP3. The exon 11 is on the reverse strand and marked in red.

```
                          .    :    .    :    .    :    .    :    .    :    .    :
SRR940959.268242407.+ AAAGGCAGACGAATAATTTGTATACAAGTGATCTGACTTCTAGAGGTCACGCTTAAGGGT
SRR940959.437565694.- AAAGGCAGACGAATACTTTGAATACAAGTGATCTGACTTCTAGAGGTCACTCTTAAGGGT
SRR940959.244934029.-    GGCAGACGAATACTTTGTATACAAGTGATCTGACTTCTAGAGGTCACTCTTAAGGGT
SRR940959.268528954.+     GCAGACGAATACTTTATATACAAGTGATCTGACTTCTAGAGGTAACTCGTCAGGGT
SRR940959.444797156.+     GCAGACGAATACTTTGTATACAAGTGATCTGACTTCTAGAGGTCACTCTTAAGGGT
SRR940959.448046329.+      CAGACGAATACTTTGTATACAAGTGATCTGACTTCTAGAGGTCACTCTTAAGGGT
SRR940959.451484845.+      CAGACGAATACTTTGTATACAAGTGATCTGACTTCTAGAGGTCACTCTTAAGGGT
SRR940959.411918903.+         ACGAATACTTTGTATACAAGTAATCTGACTTCTAGAGGTCACTCTTAAGGGT
SRR940959.106926160.+             ATACTTTGTATACAAGTGATCTGACTNCTAGAGGTCACTCTTCNGGGT
SRR940959.201925849.+                CTTTGTATACAAGTGATCTGACTTCTAGAGGTCACTCTTAAGGGT
SRR940959.219537398.+                CTTTGTATACAAGTGATCTGACTTCTAGAGGTCACTCTTAAGGGT
SRR940959.394247670.+                      ATACAAGTGATCTGACTTCTAGAGGTCACTCTTAAGGGT
SRR940959.515025362.+                        ACAAGTGATCTGACTTCTAGAGGTCACTCTTAAGGGT
SRR940959.173030180.+                              GATCTGACTTCTAGAGGTCACTCTTAAGGGT
SRR940959.407859672.+                              GATGTGACTTCAAGAGGTCACTCTTAAGGGT
SRR940959.200226292.-                              GATCTGACTTCTAGAGGTCACTCTTAAGGGT
SRR940959.112764842.+                               ATCTGACTTCTAGAGGTCACTCTTAAGGGT
SRR940959.91143040.1-                                    ACTTCTAGAGGTCACTCTTAAGGGT
SRR940959.209077398.+                                        CTAGAGGTCACTCTTAAGGGT
SRR940959.392347331.+                                        CTAGAGGTCACTCTTAAGGGT
SRR940959.215309298.+                                         TAGAGGTCACTCTTAAGGGT
SRR940959.486100358.+                                         TAGAGGTCACTCTTAAGGGT
SRR940959.134518492.+                                              GTCACTCTTAAGGGT
SRR940959.341920293.+                                                   TCTTAAGGGT
SRR940959.49339647.2+                                                          GGT
SRR940959.510154242.+                                                          GGT
SRR940959.138345957.+                                                          GGT
                      ____________________________________________________________
consensus             AAAGGCAGACGAATACTTTGTATACAAGTGATCTGACTTCTAGAGGTCACTCTTAAGGGT

                          .    :    .    :    .    :    .    :    .    :    .    :
SRR940959.268242407.+ CTTATCTTTCAGGATGACTCGCTCTGATGCCTCCTCCCCC
SRR940959.437565694.- CTTATCTTTCAGGATGACTCACTCTGATGCCTCCTCCCCC
SRR940959.244934029.- CTTATCTTTCAGGATGACTCACTCTGATGCCTCCTCCCCCAGG
SRR940959.268528954.+ CTTATCTTTCAGGATGACTCACTCAGATGCCTCCTCCCCCAGGA
SRR940959.444797156.+ CTTATCTTTCAGGATGACTCACTCTGATGCCTCCTCCCCCAGGA
SRR940959.448046329.+ CTTATCTTTCAGGATGACTCACTCTGATGCCTCCTCCCCCAGGAC
SRR940959.451484845.+ CTTATCTTTCAGGATGACTCACTCTGATGCCTCCTCCCCCAGGAC
SRR940959.411918903.+ CTTATCTTTCAGGATGACTCACTCTGATGCCTCCTCCCCCAGGACATG
SRR940959.106926160.+ CTNATCTTTCAGGATGACTCANTCTGATGCCTCCTCCCCCAGGACATGGGCC
SRR940959.201925849.+ CTTATCTTTCAGGATGACTCACTCTGATGCCTCCTCCCCCAGGACATGGGCCACG
SRR940959.219537398.+ CTTATCTTTCAGGATGACTCACTCTGATGCCTCCTCCCCCAGGACATGGGCCACG
SRR940959.394247670.+ CTTATCTTTCAGGATGACTCACTCTGATGCCTCCTCCCCCAGGACATGGGCCACGTAGAT
SRR940959.515025362.+ CTTATCTTTCAGGATGACTCACTCTGATGCCTCCTCCCCCAGGACATGGGCCACGTAGAT
SRR940959.173030180.+ CTTATCTTTCAGGATGACTCACTCTGATGCCTCCTCCCCCAGGACATGGGCCACGTAGAT
SRR940959.407859672.+ CTTATCTTTCAGGATGACTCACTCTGATGCCTCCTCCCCCAGGACATGGGCCACGTAGAT
SRR940959.200226292.- CTTATCTTTCAGGATGACTCACTCTGATGCCTCCTCCCCCAGGACATGGGCCACGTAGAT
SRR940959.112764842.+ CTTATCTTTCAGGNTGACTCNCTCTGATGCCTCCTCCCCNAGGACATGGGCCACGTAGAT
SRR940959.91143040.1- CTTATCTTTCAGGATGACTCACTCNGATGCCTCCTCCCCCAGGACATGGGCCACGTAGAT
SRR940959.209077398.+ CTTATCTTTCAGGATGACTCACTCTGATGCCTCCTCCCCCAGGACATGGGCCACGTAGAT
SRR940959.392347331.+ CTTATCTTTCAGGATGACTCACTCTGATGCCTCCTCCCCCAGGACATGGGCCACGTAGAT
SRR940959.215309298.+ CTTATCTTTCAGGATGACTCACTCTGATGCCTCCTCCCCCAGGACATGGGCCACGTAGAT
SRR940959.486100358.+ CTTATCTTTCAGGATGACTCACTCTGATGCCTCCTCCCCCAGGACATGGGCCACGTAGAT
SRR940959.134518492.+ CTTATCTTTCAGGATGACTCACTCTGATGCCTCCTCCCCCAGGACATGGGCCACGTAGAT
SRR940959.341920293.+ CTTATCTTTCAGGATGACTCACTCTGATGCCTCCTCCCCCAGGACATGGGCCACGTAGAT
SRR940959.49339647.2+ CTTATCTTTCAGGATGACTCACTCTGATGCCTCCTCCCCCAGGACATGGGCCACGTAGAT
SRR940959.510154242.+ CTTATCTTTCAGGNTGACTAACTCTGATGCNTCCTCCCCCAGGACATGGGCCACGTAGAT
SRR940959.138345957.+ CTTATCTTTCAGGATGACTCACTCTGATGCCTCCTCCCCCAGGACATGGGCCACGTAGAT
SRR940959.84528647.2-       TTTCAGGATGACTCACTCTGATGCTTCCTCCCCCAGGACATGGGCCACGTAGAT
SRR940959.111494979.-        TTCAGGATGACTCACTCTGATGCCTCCTCCCCCTGGACATGGGCCACGTAGAT
SRR940959.140868196.-         TCAGGATGACTCACTCTGATGCCTCCTCCCCCAGGACATGGGCCACGTAGAT
SRR940959.273768471.-         TCAGGATGACTCACTCTGATGCCTCCTCCCCCAGGACATGGGCCACGTAGAT
SRR940959.93689538.1-          CAGGATGACTCACTCTGATGCCTCCTCCCCCAGGGCATGGGCCACGTAGNT
SRR940959.403144584.+          CAGGATGACTCACTCTGATGCCTCCTCCCCCAGGACATGGGCCACGTAGAT
SRR940959.47530694.2+           AGGATGACTCACTCTGATGCCTCCTCCCCCAGGACATGGGCCACGTAGAT
SRR940959.149569432.+           AGGATGACTCACTCTGATGCCTCCTCCCCCAGGACATGGGCCACGTAGAT
SRR940959.231349584.+           AGGATGACTCACTCTGATGCCTCCTCCCCCAGGACATGGGCCACGTAGAT
SRR940959.234661010.+            GGATGACTCACTCTGATGCCTCCTCCCCCAGGACATGGGCCACGTAGAT
SRR940959.22195431.1+             GATGACTCACTCTGATGCCTCCTCCCCCAGGACATGGGCCACGTAGAT
SRR940959.401437588.+             GATGACTCACTCTGATGCCTCCTCCCGCAGGACATGGGCCACGTAGAT
SRR940959.286445579.+              ATGACTCACTCTGATGCCTCCTCCCCCAGGACATGGGCCACGTAGAT
```

```
SRR940959.375075521.+              ATGACTCACTCTGATGCCTCCTTCCCCAGGACATGGGCCACGTAGAT
SRR940959.494641963.+              ATGACTCACTCTGATGCCTCCTCCCCCAGGACATGGGCCACGTAGAT
SRR940959.307057544.+                 ACTCACTTTGATGCCTCCTCCCCCAGGACATGGGCCACGTAGAT
SRR940959.428092955.-                   TCACTCTGATGCCTCCTCCCCCAGGACATGGGCCACGTAGAT
SRR940959.50760203.1-                    CACTCTGATGCCTCCCCCCCCAGGACATGGGCCACGTAGAT
SRR940959.69210509.1-                    CACTCTGATGCCTCCTCCCCCAGGACATGGGCCACGTAGAT
SRR940959.370150726.-                     ACTCTGATGCCTCCTCCCCCAGGACATGGGCCACGTAGAT
SRR940959.364285414.-                      CTCTGATGCCTCCTCCCCCAGGACATGGGCCACGTAGAT
SRR940959.78148102.2+                                             ACATGGGCCACGTAGAT
SRR940959.113633429.-                                                      TCGTAGAT
SRR940959.103942720.+                                                     CACGTAGAT
SRR940959.243236356.+                            ATGCCTCCTCCCCCAGGACATGGGCCACGTAGAT
SRR940959.411918903.-                                                       CGTAGAC
SRR940959.204476140.-                                    TCCCCCAGGACATGGGCCACGTAGAT
SRR940959.205651759.+                                     CCCCCAGGACATGGGCCACGTAGAT
SRR940959.215309298.-                                             ACATGGGCCACGTAGAT
SRR940959.136939171.+                                             ACATGGGCCACGTAGAT
SRR940959.61462478.2+                                             ACATGGGCCACGTAGAT
SRR940959.262575625.+                                             ACATGGGCCACGTAGAT
SRR940959.319272543.-                                         CAGGACATGGGCCACGTAGAT
SRR940959.358612158.-                                 TCCTCCCCCAGGACATGGGCCACGTAGAT
SRR940959.424469311.-                                                       CGTAGAT
SRR940959.10309449.2+                                                        GTAGAT
SRR940959.32971802.1+                                                        GTAGAT
SRR940959.475305655.+                                                        GTAGAT
SRR940959.179827113.-                                                          AGAT
SRR940959.211775318.+                                                          AGAT
                      ____________________________________________________________
consensus             CTTATCTTTCAGGATGACTCACTCTGATGCCTCCTCCCCCAGGACATGGGCCACGTAGAT

                          .    :    .    :    .    :    .    :    .    :    .    :
SRR940959.394247670.+ G
SRR940959.515025362.+ GAT
SRR940959.173030180.+ GATGTCAGC
SRR940959.407859672.+ GATGTCAGC
SRR940959.200226292.- GATGTCAGC
SRR940959.112764842.+ GATGTCAGCG
SRR940959.91143040.1- GATGTCAGCGTACCC
SRR940959.209077398.+ GATGTCAGCGTACCCCATG
SRR940959.392347331.+ GATGTCAGCGTACCCCATG
SRR940959.215309298.+ GATGTCAGCGTACCCCATGC
SRR940959.486100358.+ GATGTCAGCGTACCCCATGC
SRR940959.134518492.+ GATGTCAGCGTACCCCATGCAGCTG
SRR940959.341920293.+ GATGTCAGCGTACCCCATGCAGCTGGAGAT
SRR940959.49339647.2+ GATGTCAGCGTACCCCATGCAGCTGGAGATGTTGTTT
SRR940959.510154242.+ GATGTCAGCGTACCCCATGCAGCTGGAGATGTTGTTT
SRR940959.138345957.+ GATGTCAGCGTACCCCATGCAGCTGGAGATGTTGTTT
SRR940959.84528647.2- GATGTCAGTGTACCCCATGCAGCTGGAGATGTTGTTTCGGATATAG
SRR940959.111494979.- GATGTCAGCGTACCCCATGCAGCTGGAGATGTTGTTTCGGATATAGT
SRR940959.140868196.- GATGTCAGCGTACCCCATGCAGCTGGAGATGTTGTTTCGGATATAGTA
SRR940959.273768471.- GATGTCAGCGTACCCCATGCAGCTGGAGATGTTGTTTCGGATATAGTA
SRR940959.93689538.1- GATGTCAGCGTACCCCATGCAGCTGGAGATGTTGTTTCGGATATAGTAG
SRR940959.403144584.+ GATGTCAGCGTACCCCATGCAGCTGGAGATGTTGTTTCGGATATAGTAG
SRR940959.47530694.2+ GATGTCAGCGTACCCCATGCAGCTGGTGATGTTGTTTCGGATATAGTAGA
SRR940959.149569432.+ GATGTCAGCGTACCCCATGCAGCTGGAGATGTTGTTTCGGATATAGTAGA
SRR940959.231349584.+ GATGTCAGCGTACCCCATGCAGCTGGAGATGTTGTTTCGGATATAGTAGA
SRR940959.234661010.+ GATGTCAGCGTACCCCATGCAGCTGGAGATGTTGTTTCGGATATAGTAGAA
SRR940959.22195431.1+ GATGTCAGCGTACCCCATGCAGCTGGAGATGTTGTTTCGGATATAGTAGAAG
SRR940959.401437588.+ GATGTCAGCGTACCCCATGCAGCTGGAGATGTTGTTTCGGATATAGTAGAAG
SRR940959.286445579.+ GATGTCAGCGTACCCCATGCAGCTGGAGATGTTGTTTCGGATATAGTAGAAGA
SRR940959.375075521.+ GATGTCAGCGTACCCCATGCAGCTGGAGATGTTGTTTCGGATATAGTAGAAGA
SRR940959.494641963.+ GATGTCAGCGTACCCCATGCAGCTGGAGATGTTGTTTCGGATATAGTAGAAGA
SRR940959.307057544.+ GATGTCAGCGTACCCCATGCAGCTGGAGATGTTGTTTCGGATATAGTAGAAGAGAT
SRR940959.428092955.- GATGTCAGCGTACCCCATGCAGCTGGAGATGTTGTTTCGGATATAGTAGAAGAGATAG
SRR940959.50760203.1- GATGTCAGCGTACCCCATGCAGCTGGAGATGTTGTTTCGGATATAGTAGAAGAGATAGA
SRR940959.69210509.1- GATGTCAGCGTACCCCATGCAGCTGGAGATGTTGTTTCGGATATAGTAGAAGAGATAGA
SRR940959.370150726.- GATGTCAGCGTACCCCATGCAGCTGGAGATGTTGTTTCGGATATAGTAGAAGAGATAGAT
SRR940959.364285414.- GATGTCAGCGTACCCCATGCAGCTGGAGATGTTGTTTCGGATATAGTAGAAGAGATAGAT
SRR940959.78148102.2+ GATGGGAGCGTACCCCATGCAGCTGGAGATGTTGGTTCGGATATAGTAGAAGAGATAGAG
SRR940959.113633429.- GATGTCAGCGTACCCCATGCAGCTGNATATGTTGTTTCGGATATAGTAGAAGAGATAGAT
SRR940959.103942720.+ GATGTCACCGTACCCCATGCAGCTGGAGATGTTGTTTCGGANATAGTAGAAGAGATAGAT
SRR940959.243236356.+ GATGTCAGCGTACCCCATGCAGCTGGAGATGTTGTTTCGGAGATAGTAGAATAGATAGAT
SRR940959.411918903.- GATGTCAGCGTACCCCATGCAGCTGGAGATGTTGTTTCGGATATAGTAGAACAGATAGAT
SRR940959.204476140.- GATGTCAGCGTACCCCATGCAGCTGGAGATGTTGTTTCGGATATAGTAGAAGAGATAGAT
SRR940959.205651759.+ GATGTCAGCGTACCCCATGCAGCTGGAGATGTTGTTTCGGATATAGTAGAAGAGATAGAT
SRR940959.215309298.- GATGTCAGCGTACCCCATGCAGCTGGAGATGTTGTTTCGGATATAGTAGAAGAGATAGAT
SRR940959.136939171.+ GATGTCAGCGTACCCCATGCAGCTGGAGATGTTGTTTCGGATATAGTAGAAGAGATAGAT
```

```
SRR940959.61462478.2+ GATGTCAGCGTACCCCATGCAGCTGGAGATGTTGTTTCGGATATAGTAGAAGAGATAGAT
SRR940959.262575625.+ GATGTCAGCGTACCCCATGCAGCTGGAGATGTTGTTTCGGATATAGTAGAAGAGATAGAT
SRR940959.319272543.- GATGTCAGCGTACCCCATGCAGCTGGAGATGTTGTTTCGGATATAGTAGAAGAGATAGAT
SRR940959.358612158.- GATGTCAGCGTACCCCATGCAGCTGGAGATGTTGTTTCGGATATAGTAGAAGAGATAGAT
SRR940959.424469311.- GATGTCAGCGTACCCCATGCAGCTGGAGATGTTGTTTCGGATATAGTAGAAGAGATAGAT
SRR940959.10309449.2+ GATGTCAGCGTACCCCATGCAGCTGGAGATGTTGTTTCGGATATAGTAGAAGAGATAGAT
SRR940959.32971802.1+ GATGTCAGCGTACCCCATGCAGCTGGAGATGTTGTTTCGGATATAGTAGAAGAGATAGAT
SRR940959.475305655.+ GATGTCAGCGTACCCCATGCAGCTGGAGATGTTGTTTCGGATATAGTAGAAGAGATAGAT
SRR940959.179827113.- GATGTCAGCGTACCCCATGCAGCTGGAGATGTTGTTTCGGATATAGTAGAAGAGATAGAT
SRR940959.211775318.+ GATGTCAGCGTACCCCATGCAGCTGGAGATGTTGTTTCGGATATAGTAGAAGAGATAGAT
SRR940959.53071838.1+  ATGTCAGCGTACCCCATGCAGCTGGAGATGTTGTTTCGGATATAGTCGAAGAGATAGAT
SRR940959.119091778.+             CCCCATGCAGCTGGAGATGTTGTTTCGGATATAGTAGAAGAGATAGAT
SRR940959.511624639.+             CCCCATGCAGCTGGAGATGTTGTTTCGGATATAGTAGAAGAGATAGAT
SRR940959.12543660.2-                    CAGTTGGAGATGTTGTTTCGGATATAGTAGAAGAGATAGAT
SRR940959.171901360.-                     AGCTGGAGATTTTGTTTCGGATATAGTAGAAGAGATAGAT
SRR940959.448850382.+                          GAGATGTTGTTTCGGATATAGTAGAAGAGATAGAT
SRR940959.232549148.+                           AGATGTTGTTTCGGATATAGTAGAAGAGATAGAT
SRR940959.99893636.2+                                      CGGATATAGTAGAAGAGATAGAT
SRR940959.476899101.-                                         ATATAGTAGAAGAGATAGAT
SRR940959.53461555.1+                                             AGTAGAAGAGATAGAT
SRR940959.251923358.+                                             AGTAGAGGAGATAGAT
SRR940959.90270943.2+                                             AGTAGAAGAGATAGAT
SRR940959.240600382.-                                             AGTAGAAGAGATAGAT
SRR940959.95022769.1+                                             AGTAGAAGAGATAGAT
SRR940959.208402304.+                                             AGTAGAAGAGATAGAT
SRR940959.464853106.+                                             AGTAGAAGAGATAGAT
SRR940959.157248646.+                                              GTAGAAGAGATAGAT
SRR940959.507797757.+                                              GTAGAAGAGATAGAT
SRR940959.202585888.+                                              GTAGAAGAGATAGAT
SRR940959.107606268.+                                                AGAAGAGATAGAT
SRR940959.377833538.+                                                AGAAGAGATAGAT
SRR940959.29798712.1+                                                 GAAGAGATAGAT
SRR940959.115130534.+                                                 GAAGAGATAGAT
SRR940959.130604048.+                                                  AAGAGATAGAT
SRR940959.295045518.+                                                  AAGAGATAGAT
SRR940959.480312325.+                                                  AAGAGATAGAT
SRR940959.472841393.-                                                       ATAGAT
SRR940959.501523941.+                                                         AGAT

consensus             GATGTCAGCGTACCCCATGCAGCTGGAGATGTTGTTTCGGATATAGTAGAAGAGATAGAT

                          .    :    .    :    .    :    .    :    .    :    .    :
SRR940959.364285414.- G
SRR940959.78148102.2+ AAGGCACATCTCATTAACGGTGC
SRR940959.113633429.- GAGGCACATCTCATTAACGGTGCTGGGACCCC
SRR940959.103942720.+ GAGGCACATCTCATTAACGGTGCTGGGACCC
SRR940959.243236356.+ GAGGCA
SRR940959.411918903.- GAGGCACATCTCATTAACGGTGCTGGGACCCCC
SRR940959.204476140.- GAGGCACATCTCAT
SRR940959.205651759.+ GAGGCACATCTCATT
SRR940959.215309298.- GAGGCACATCTCATTAACGGTGC
SRR940959.136939171.+ GAGGCACATCTCATTAACGGTGC
SRR940959.61462478.2+ GAGGCACATCTCATTAACGGTGC
SRR940959.262575625.+ GAGGCACATCTCATTAACGGTGC
SRR940959.319272543.- GAGGCACATCTCATTAACG
SRR940959.358612158.- GAGGCACATCT
SRR940959.424469311.- GAGGCACATCTCATTAACGGTGCTGGGACCCCC
SRR940959.10309449.2+ GAGGCACATCTCATTAACGGTGCTGGGACCCCCC
SRR940959.32971802.1+ GAGGCACATCTCATTAACGGTGCTGGGACCCCCC
SRR940959.475305655.+ GAGGCACATCTCATTAACGGTGCTGGGACCCCCC
SRR940959.179827113.- GAGGCACATCTCATTAACGGTGCTGGGACCCCCCTG
SRR940959.211775318.+ GAGGTACATCTCATTAACGGTGCTGGGACCCCCCTG
SRR940959.53071838.1+ GAGGCACATCTCATTAACGGTGCTGGGACCCCCCTGGAGAA
SRR940959.119091778.+ GAGGCACATCTCATTAACGGTGCTGGGACCCCCCTGGAGAAGAAAGAGAGAG
SRR940959.511624639.+ GAGGCACATCTCATTAACGGTGCTGGGACCCCCCTGGAGAAGAAAGAGAGAN
SRR940959.12543660.2- GAGGCACATCTCATTAACGGTGCTGGGACCCCCCTGGAGAAGAAAGAGAGAGAGAGAGA
SRR940959.171901360.- GAGGCACATCTCATTAACGGTGCTGGGACCCCCCTGGAGAAGAAAGAGAGAGAGAGAGAG
SRR940959.448850382.+ GAGGCACATCTCATTAACGGTGCTGGGACCCCCCTGGAGAAGAAAGAGAGAGAGAGAGAG
SRR940959.232549148.+ GAGGCACATGTCATTAACGGTGCTGGGACCCCCCTGGAGAAGAAAGAGAGAGAGAGAGAG
SRR940959.99893636.2+ GAGGCACATCTCATTAACGGTGCTGGGACCCCCCTGGAGAAGAAAGAGAGAGAGAGAGAG
SRR940959.476899101.- GAGGCACATCTCATTAACGGTGCTGGGACCCCCCTGGAGAAGAAAGAGAGAGAGAGAGAG
SRR940959.53461555.1+ GAGGCACATCTCATTAACGGTGCTGGGACCCCCCTGGAGAAGAAAGAGAGAGAGAGAGAG
SRR940959.251923358.+ GAGGCACATCTCATGAACGGTGCTGGGACCCCCCTCGAGAAGAACGAGAGAGAGAGAGAG
SRR940959.90270943.2+ GAGGCACATCTCATTAACGGTGCTGGGACCCCCCAGGAGAAGAAAGATAGAGAGAGAGAG
SRR940959.240600382.- GAGGCACATCTCATTAACGGTGCTGGGACCCCCCTGGAGAAGAAAGAGAGAGAGAGAGAT
SRR940959.95022769.1+ GAGGCACATCTCATTAACGGTGCTGGGACCCCCCNGGAGAAGAAAGAGAGAGAGAGAGAG
SRR940959.208402304.+ GAGGCACATCTCATTAACGGTGCTGGGACCCCCCTGGAGAAGAAAGAGAGAGAGAGAGAG
```

```
SRR940959.464853106.+ GAGGCACATCTCATTAACGGTGCTGGGACCCCCCTGGAGAAGAAAGAGAGAGAGAGAGAG
SRR940959.157248646.+ GAGGCACATCTCATTAACGGTGCTGGGACCCCCCTGGAGAAGAAAGAGAGAGAGAGAGAG
SRR940959.507797757.+ GNGGCACATCTCATTAACCGTGCTGGGACCCCCCTGGAGAAGAAAGAGAGAGAGAGAGAG
SRR940959.202585888.+ GAGGCACATCTCATTAACGGTGCTGGGACCCCCCTGGAGAAGAAAGAGAGAGAGAGAGCG
SRR940959.107606268.+ GAGGCACATCTCANTAACGGTGCTGGGACCNCCCTGGNGAAGAAAGAGAGAGAGAGNGAG
SRR940959.377833538.+ GAGGCACATCTCATTAACGGTGCTGGGACACCCCTGGAGAAGAAAGAGAGAGAGAGAGAG
SRR940959.29798712.1+ GAGGCACATCTCATTAACGGTGCTGGGACCCCCCTGGAGAAGAAAGAGAGAGAGAGAGAG
SRR940959.115130534.+ GAGGCACATCTCATNAACGGTGCTGGGACCCNCCTGGANAAGAAAGAGAGAGAGAGANAG
SRR940959.130604048.+ GAGGCACATCTCATTAACGGTGCTGGGACCCCCCTGGAGAAGAAAGAGAGAGAGAGAGAG
SRR940959.295045518.+ GAGGCACATCTCATTAACGGTGCTGGGACCCCCCTGGAGAAGAAAGAGAGAGAGAGAGAG
SRR940959.480312325.+ GAGGCACATCTCATTAACGGTGCTGGGACCCCCCTGGAGAAGAAAGAGAGAGAGAGAGAG
SRR940959.472841393.- GAGGCACATCTCATTAACGGTGCTGGGACCCCCCTGGAGAAGAAAGAGAGAGAGAGAGAG
SRR940959.501523941.+ GAGGCACATCTCATTAACGGTGCTGGGACCCCCCTGGAGAAGAAAGAGAGAGAGAGAGAG
SRR940959.167150541.+ GAGGCACATCTCATTAACGGTGCTGGGACCCCCCTGGAGAAGAAAGAGAGAGAGAGAGAG
SRR940959.74165015.1+      ACATCTCATTAACGGTGCTGGGACCCCCCTGGAGAAGAAAGAGAGAGAGAGAGAG
SRR940959.203108509.+        ATCTCATTAACGGTGCTGGGACCCCCCTGGAGAAGAAAGAGAGAGAGAGAGAG
SRR940959.265058062.+             ATTAACNGTGCTGGGACCCCCCTGGAGAAGAAAGAGAGAGAGAGAGAG
SRR940959.510154242.-               NAACGGTGCTGGGACCCCCCTGGAGAAGAAAGAGAGAGAGAGAGAG
SRR940959.294448553.+                 ACGGTGCTGGGACCCCCCTGGAGAAGAAAGAGAGAGAGAGAGAG
SRR940959.420227343.+                 ACGGTGCTGGGACCCCCCTGGAGAAGAAAGAGAGAGAGAGAGAG
SRR940959.35218028.2+                   GGTGCTGGGACCCCCCTGGAGAAGAAAGAGAGAGAGAGAGAG
SRR940959.486339120.+                   GGTGCTGGGACCCCCCTGGAGAAGAAAGAGAGAGAGAGAGAG
                      ____________________________________________________________
consensus             GAGGCACATCTCATTAACGGTGCTGGGACCCCCCTGGAGAAGAAAGAGAGAGAGAGAGAG

                          .    :    .    :    .    :    .    :    .    :    .    :
SRR940959.448850382.+ AGAGA
SRR940959.232549148.+ AGATGG
SRR940959.99893636.2+ AGAGGGAGAGACAGAGA
SRR940959.476899101.- AGAGATGGAGAGACAGAGAT
SRR940959.53461555.1+ AGAGATGGAGAGACAGAGATACAC
SRR940959.251923358.+ AGAGATGGAGAGACAGAGATACAC
SRR940959.90270943.2+ AGAGATGGAGAGACAGAGATACAC
SRR940959.240600382.- AGAGATGGAGAGACAGAGATACAC
SRR940959.95022769.1+ AGAGATGGAGAGACAGAGATACAC
SRR940959.208402304.+ AGAGATGGAGAGACAGAGATACAC
SRR940959.464853106.+ AGAGATGGAGAGACAGAGATACAC
SRR940959.157248646.+ AGAGATGGAGAGACAGAGATACACA
SRR940959.507797757.+ AGAGATGGAGAGACAGAGATACACA
SRR940959.202585888.+ AGAGATGGAGAGACAGAGATACACA
SRR940959.107606268.+ AGAGATGGAGAGACAGAGATACACAGG
SRR940959.377833538.+ AGAGATGGAGAGACAGAGATACACAGG
SRR940959.29798712.1+ AGAGATGGAGAGACAGAGATACACAGGC
SRR940959.115130534.+ AGAGATGGAGAGACAGAGATACACAGGC
SRR940959.130604048.+ AGAGATGGAGAGACAGAGATACACAGGCA
SRR940959.295045518.+ AGAGATGGAGAGACAGAGATGCACAGGCA
SRR940959.480312325.+ AGAGATGGAGAGACAGAGATACACAGGCA
SRR940959.472841393.- AGAGATGGAGAGACAGAGATCCACAGGCATGCAG
SRR940959.501523941.+ AGAGATGGAGAGACAGAGATACACAGGCATGCAGAN
SRR940959.167150541.+ AGAGATGGAGAGACAGAGATACACAGGCATGCAGAGAGAG
SRR940959.74165015.1+ AGAGATGGAGAGACAGAGATACACAGGCATGCAGAGAGAGGGGAA
SRR940959.203108509.+ AGAGATGGAGAGACAGAGATACACAGGCATGCAGAGAGAGGGGAACA
SRR940959.265058062.+ AGCGAGGGAGAGGCAGAGATACACAGGCATGCAGAGAGAGGGGAACAAGGTG
SRR940959.510154242.- AGAGATGGAGAGACAGAGATACACAGGCATGCAGAGAGAGGGGAACAAGGTAGG
SRR940959.294448553.+ AGAGATGGAGAGACAGAGATACACAGGCATGCAGAGAGAGGCGAACACGGTAGGCA
SRR940959.420227343.+ AGAGATGGAGAGACAGAGATACACAGGCATGCAGAGAGAGGGGAACAAGGTAGGCA
SRR940959.35218028.2+ AGAGATGGAGAGACAGAGATACACAGGCATGGAGAGAGAGGGGAAAAAGGGAGGCAGA
SRR940959.486339120.+ AGAGATGGAGAGACAGAGATACACAGGCATGCAGAGAGAGGGGAACAAGGTAGGCAGA
                      ____________________________________________________________
consensus             AGAGATGGAGAGACAGAGATACACAGGCATGCAGAGAGAGGGGAACAAGGTAGGCAGA
```

(12) The exon 12 sequence was predicted by assembling WGS data using CAP3. The exon 12 is marked in red.

```
                          .    :    .    :    .    :    .    :    .    :    .    :
SRR940959.350034436.+ ACAAAAGTGAGGCTATTTTATACACATTTTGTCAAAGCTTTCAGTCAACCCTAAACTAGT
SRR940959.106641407.+   AAAAGTGAGGCTATTTTATACACATTNTGTCAGAGCTTTCAGTNAACCCTNAACTAGG
SRR940959.299665187.+   AAAAGTGAGGCTATTTTATACACATTTTGTCAAAGCTTTCAGTCAACCCTAAACTAGT
SRR940959.375025440.-     AAGTGAGGCTATTTTATACACATTTTGTCAAAGCTTTCAGTCAACCCTAAACTAGT
SRR940959.217809918.-        TGAGGCTATTTTATACACATTTTGTCAAAGCTTTCAGTCAACCCTAAACTAGT
SRR940959.489340487.-          AGGCTATTTTATACACATTTTGTCAAAGCTTTCAGTCAACCCTAAACTAGT
SRR940959.510755886.+           AGCTATTTTATACACATTTTGTCAAAGCTTTCAGTCAACCCTAAACTAGT
SRR940959.312467701.+                TTTTATACACATTTTGTCAAAGCTTTCAGTCAACCCTAAACTAGT
SRR940959.362062380.+                          ATTTTGTCAAAGCTTTCAGTCAACCCTAAACTAGT
SRR940959.250616835.-                            TTTGTCAAAGCTTTCAGTCAACCCTAAACTAGT
SRR940959.256505207.-                            TTTGTCAAAGCTTTCAGTCAACCCTAAACTAGT
```

```
SRR940959.495498540.-                            TTTGCCAAAGCTTTCAGTCAACCCTAAACTAGT
SRR940959.355049469.-                            TTTGTCAAAGCTTTCAGTCAACCCTAAACTAGT
SRR940959.361356096.-                            TTTGTCAAAGCTTTCAGTCAACCCTAAACTAGT
SRR940959.129765546.-                              TGTCAAAGCTTTCAGTCAACCCTAAACTAGT
SRR940959.385661162.-                              TGTCAAAGCTTTCAGTCAACCCTAAACTAGT
SRR940959.181747166.-                              TGTCAAAGCTTTCAGTCAACCCTAAACTAGT
SRR940959.189573774.-                                GTAAAGCTTTCAGTCAACCCTAAACTAGT
SRR940959.422321056.+                                      CTTTCAGTCAACCCTAAACTAGT
SRR940959.305448832.-                                        TTCAGTCAACCCTAAACTAGT
SRR940959.384040176.+                                         TCAGTCAACCCTAAACTAGT
SRR940959.69767114.2+                                            GTCAACCCTAAACTAGT
SRR940959.361025140.+                                            GTCAACCCTAAACTAGT
SRR940959.255276204.-                                               AACCCTAAACTAGT
SRR940959.469207183.+                                                ACCCTAAACTAGT
SRR940959.49122040.2+                                                      AACTAGT
SRR940959.358912339.+                                                      AACTAGT

consensus             ACAAAAGTGAGGCTATTTTATACACATTTTGTCAAAGCTTTCAGTCAACCCTAAACTAGT

                          .    :    .    :    .    :    .    :    .    :    .    :
SRR940959.350034436.+ GTCTGTTCTTCCTGCCCATGACAGTTCCATGGAGGGCATG
SRR940959.106641407.+ GTCTGTTCTTCNTGCCCATGACAGTTCCATGGAGGGAATGAT
SRR940959.299665187.+ GTCTGTTCTTCCTGCCCATGACAGTTCCATGGAGGGCATGAT
SRR940959.375025440.- GTCTGTTCTTCCTGCCCATGACAGTTCCATGGAGGGCATGATGG
SRR940959.217809918.- GTCTGTTCTTCCTGCCCATGACAGTTCCATGGAGGGCATGATGGCCA
SRR940959.489340487.- GTCTGTTCTTCCTGCCCATGACAGTTCCATGGAGGGCATGATGGCCATG
SRR940959.510755886.+ GTCTGTTCTTCCTGCCCATGACAGTTCCATGGAGGGCATGATGGCCATCN
SRR940959.312467701.+ GTCTGTTCTTCCTGCCCATGACAGTTCCATGGAGGGCATGATGGCCATGAACAAT
SRR940959.362062380.+ GTCTGTTCTTCCTGCCCATGACAGTTCCATGGAGGGCATGATGGCCATGAACAATGTAGA
SRR940959.250616835.- GTCTGTTCTTCCTGCCCATGACAGTTCCATGGAGGGCATGATGGCCATGAACAATGTAGA
SRR940959.256505207.- GTCTGTTCTTCCTGCCCATGACAGTTCCATGGAGGGCATGATGGCCATGAACAATGTAGA
SRR940959.495498540.- GTCTGTTCTTCCTGCCCATGACAGTTCCATGGAGGGCATGATGGCCATGAACAATGTAGA
SRR940959.355049469.- GTCTGTTCTTCCTGCCCATGACAGTTCCATGGAGGGCATGATGGCCATGAACAATGTAGA
SRR940959.361356096.- GTCTGTTCTTCCTGCCCATGACAGTTCCATGGAGGGCATGATGGCCATGAACAATGTAGA
SRR940959.129765546.- GTCTGTTCTTCCTGCCCATGACAGTTCCATGGAGGGCATGATGGCCATGAACAATGTAGA
SRR940959.385661162.- GTCTGTTCTTCCTGCCCATGACAGTTCCATGGAGGGCATGATGGCCATGAACAATGTAGA
SRR940959.181747166.- GTCTGTTCTTCCTGCCCATGACAGTTCCATGGAGGGCATGATGGCCATGAACAATGTAGA
SRR940959.189573774.- GTCTGTTCTTCCTGCCCATGACAGTTCCATGGAGGGCATGATGGCCATGAACAATGTAGA
SRR940959.422321056.+ GTCTGTTCTTCCTGCCCATGACAGTTCCATGGAGGGCATGATGGCCATGAACAATGTAGA
SRR940959.305448832.- GTCTGTTCTTCCTGCCCATGACAGTTCCATGGAGGGCATGATGGCCATGAACAATGTAGA
SRR940959.384040176.+ GTCTGTTCTTCCTGCCCATGACAGTTCCATGGAGGGCATGATGGCCATGAACAATGTAGA
SRR940959.69767114.2+ GTCTGTTCTTCCTGCCCATGACAGTTCCATGGAGGGCATGATGGCCATGAACAATGTAGA
SRR940959.361025140.+ GTCTGTTCTTCCTGCCCATGACAGTTCCATGGAGGGCATGATGGCCATGAACAATGTAGA
SRR940959.255276204.- GTCTGTTCTTCCTGCCCATGACAGTACCATGGAGGGCATGATGGCCATGAACAATTTAGA
SRR940959.469207183.+ GTCTGTTCTTCCTGCCCATGACAGTTCCATGGAGGGCATGATGGCCATGAACAATGTAGA
SRR940959.49122040.2+ GTCTGTTCTTCCTGCCCATGACAGTTCCATGGAGGGCATGATGGCCATGAACAATGTAGA
SRR940959.358912339.+ GTCTGTTCTTCCTGCCCATGACAGTTCCATGGAGGGCATGATGGCCATGAACAATGTAGA
SRR940959.243929948.-      TTCTTCCTGCCCATGACAGTTCCATGGAGGGCATGATGGCCATGAACAATGAAGA
SRR940959.362669368.-             TGCCCATGACAGTTCCATGGAGGGCATGATGGCCATGAACAATGTAGA
SRR940959.299325379.+              GCCCATGACAGTTCCATGGAGGGCATGATGGCCATGAACAATGTAGA
SRR940959.381259921.+                  ATGACAGTTCCATGGAGGGCATGATGGCCATGAACAATGTAGA
SRR940959.199991125.+                     ACAGTTCCATGGAGGGCATGATGGCCATGAACAATGTAGA
SRR940959.508501723.-                     ACAGTTCCATGGAGGGCATGATGGCCATGAACAATGTAGA
SRR940959.255161089.-                        GTTCCATGGAGGGCATGATGGCCATGAACAATGTAGA
SRR940959.204547970.+                                      ATGATAGCCATGAACAATGTAGA
SRR940959.336287676.+                                GAGGGCATGATGGCCATGAACAATGTAGA
SRR940959.164356403.+                                      ATGATGGCCATGAACAATGTAGA
SRR940959.390495608.-                           CCATGGAGGGCATGATGGCCATGAACAATGTAGA
SRR940959.466548155.+                             ATGGAGGGCATGATGGCCATGAACAATGTAGA
SRR940959.513385216.+                                                   ACAATGTAGA
SRR940959.2637312.1+                                                       ATGTAGA
SRR940959.321171471.+                                                        GTAGA

consensus             GTCTGTTCTTCCTGCCCATGACAGTTCCATGGAGGGCATGATGGCCATGAACAATGTAGA

                          .    :    .    :    .    :    .    :    .    :    .    :
SRR940959.362062380.+ GTGGA
SRR940959.250616835.- GTGGACT
SRR940959.256505207.- GTGAACT
SRR940959.495498540.- GTGGACT
SRR940959.355049469.- GTGGACT
SRR940959.361356096.- GTGGACT
SRR940959.129765546.- GTGGACTCT
SRR940959.385661162.- GTGCACTCT
SRR940959.181747166.- GTGGACTCT
SRR940959.189573774.- GTGGACTCTGG
SRR940959.422321056.+ GAGGACTCTGGAGAACA
```

```
SRR940959.305448832.- GTGGACTCTGGAGAACATT
SRR940959.384040176.+ GTGGACTCTGGAGAACATTA
SRR940959.69767114.2+ GTGGACTCTGGAGAACATTAAAA
SRR940959.361025140.+ GTGGACTCTGGAGAACATTAAAA
SRR940959.255276204.- GTGGACTCTGGAGAACATTNAAAAGG
SRR940959.469207183.+ GTGGACTCTGGAGAACATTAAAAAGGC
SRR940959.49122040.2+ GTGGACTCTGGAGAACATTAAAAAGGCTGAGAA
SRR940959.358912339.+ GTGGACTCTGGAGAACATTAAAAAGGCTGAGAA
SRR940959.243929948.- GAGGACTCTGGAGAACATTAAAAAGGCTGAGAAGGCCTGCGAGGA
SRR940959.362669368.- GTGGACTCTGGAGAACATTAAAAAGGCTGAGAAGGCCTGCGAGGAGGCCCAG
SRR940959.299325379.+ GTGGACTCTGGAGAACATTAAAAAGGCTGAGAAGGCCTGCGAGGAGGCCCAGC
SRR940959.381259921.+ GTGGACTCTGGAGAACATTAAATAGGCTGAGAAGGCCTGCGAGGAGGCCCAGCAGAT
SRR940959.199991125.+ GTGGACTCTGGAGAACATTAAAAAGGCTGAGAAGGCCTGCGAGGAGGCCCAGCAGATGGT
SRR940959.508501723.- GTGGACTCTGGAGAACATTAAAAAGGNTGAGAAGGCATGCGAGNAGGCCCAGCAGATGGT
SRR940959.255161089.- GTAGACTCTGGAGAACATTAAAAAGGCTGAGATGGCCTGCGAGGAGGCTCAGCAGANGGT
SRR940959.204547970.+ GTGGACTCTGGAGAACATTAAAAAGGCTGAGAAGGCCTGCGAGGAGGCCCAGCAGATGGT
SRR940959.336287676.+ GTGGACTCTGGAGAACATTAAAAAGGCTGAGAAGGCCTGCGAGGAGGCCCAGCGGATGGT
SRR940959.164356403.+ GTGGACTCTGGAGAACATTAAAAAGGCTGAGAAGGCCTGCGAGGAGGCCCAGCAGATGGT
SRR940959.390495608.- GTGGACTCTGGAGAACATTAAAAAGGCTGAGAAGGCCTGCGAGGAGGCCCAGCAGATGGT
SRR940959.466548155.+ GTGGACTCTGGAGAACATTAAAAAGGCTGAGAAGGCCTGCGAGGAGGCCCAGCAGATGGT
SRR940959.513385216.+ GTGGACTCTGGAGAACATTAAAAAGGCTGAGAAGGCCTGCGAGGAGGCCCAGCAGATGGT
SRR940959.2637312.1+  GTGGACTCTGGAGAACATTAAAAAGGCTGAGAAGGCCTGCGAGGAGGCCCAGCAGATGGT
SRR940959.321171471.+ GTGGACTCTGGAGAACATTAAAAAGGCTGAGAAGGCCTGCGAGGAGGCCCAGCAGATGGT
SRR940959.58163753.1-      CTCTGGAGAACATTAAAAAGGCTGAGAAGGCCTGCGAGGAGGCCCAGCAGATGGT
SRR940959.451412305.-      CTCTGGAGAACATTAAAAAGGCTGAGAAGGCCTGCGAGGAGGCCCAGCAGATGGT
SRR940959.212973662.-      CTCTGGAGAACATTAAAAAGGCTGAGAAGGCCTGCGAGGAGGCCCAGCAGATGGT
SRR940959.127501035.-      CTCTGGAGAACATTAAAAAGGCTGAGAAGGCCTGCGAGGAGGCCCAGCAGATGGT
SRR940959.282964998.-      CTCTGGAGAACATTAAAAAGGCTGAGAAGGCCTGCGAGGAGGCCCAGCAGATGGT
SRR940959.1828870.1-        TCTGGAGAACATTAAAAAGGCTGAGAAGGCCTGCGAGGAGGCCCAGCAGATGGT
SRR940959.475027989.-       TCTGGAGAACATTAAAAAGGCTGAGAAGGCCTGCGAGGAGGCCCAGCAGATGGT
SRR940959.35556388.2-        CTGGAGAACATTAAAAAGGCTGAGAAGGCCTGCGAGGAGGCCCAGCAGATGGT
SRR940959.519620866.+        CTGGAGAACATTAAAAAGGCTGAGAAGGCCTGCGAGGAGGCCAAGCAGATGGT
SRR940959.65486974.1+        CTGGAGAACATTAAAAAGGCTGAGAAGGCCTGCGAGGAGGCCCAGCAGATGGT
SRR940959.236029452.-         TGGAGAACATTAAAAAGGCTGAGAAGGCCTGCGAGGCGGCCCAGCAGACGGT
SRR940959.179648805.+            AGAACATTAAAAAGGCTGAGAAGGCCTGCGAGGAGGCCCAGCAGATGGT
SRR940959.412155320.+            AGAACATTAAAAAGGCTGAGAAGGCCTGCGAGGAGGCCCAGGAGATGGT
SRR940959.226827903.-                     AAAAGGCTGAGAAGGCCTGCGAGGAGGCCCAGCAGATGGT
SRR940959.243908493.-                     AAAAGGCTGAGAAGGCCTGCGAGGAGGCCCAGCAGATGGT
SRR940959.263968702.-                     AAAAGGCTGAGAAGGCCTGCGAGGAGGCCCAGCAGATGGC
SRR940959.503372481.-                        NGGCTGAGAAGGCCTGCGAGGAGGCCCAGCAGATGGT
SRR940959.122190638.+                         GGCTGAGAAGGCCTGCGAGGAGGCCCAGCAGATGGT
SRR940959.453148914.+                             GAGAAGGCCTGCGAGGAGGCCCAGCAGATGGT
SRR940959.326833507.+                               GAAGGCCTGCGAGGAGGCCCAGCAGATGGT
SRR940959.237694524.+                                    CCTGCGAGGAGGCCCAGCAGATGGT
SRR940959.5689026.1+                                                      CAGATGGT
SRR940959.281789585.+                                                        ATGGT
SRR940959.519908744.-                                                           GT

consensus             GTGGACTCTGGAGAACATTAAAAAGGCTGAGAAGGCCTGCGAGGAGGCCCAGCAGATGGT

                          .    :    .    :    .    :    .    :    .    :    .    :
SRR940959.255161089.- GAT
SRR940959.204547970.+ GATAATAAAGACCATTG
SRR940959.336287676.+ GATAATAAAGA
SRR940959.164356403.+ GATAATAAAGACCATTG
SRR940959.390495608.- GATAAT
SRR940959.466548155.+ GATAATAA
SRR940959.513385216.+ GATAATAAAGACCATTGATGTAAGTGTCCC
SRR940959.2637312.1+  GATAATAAAGACCATTGATGTAAGTGTCCCAGG
SRR940959.321171471.+ GATAATAAAGACCATTGATGTAAGTGTCCCAGGAG
SRR940959.58163753.1- GATAATAAAGACCATTGATGTAAGTGTCCCAGGAGACACCACCCT
SRR940959.451412305.- GATAATAAAGCCCATTGATGTAAGTGTCCCAGGAGACACCTCCCT
SRR940959.212973662.- GATAATAAAGACCATTGATGTAAGTGTCCCAGGAGACACCACCCT
SRR940959.127501035.- GATAATAAAGACCATTGATGTAAGTGTCCCAGGAGACACCACCCT
SRR940959.282964998.- GATAATAAAGACCATTGATGTAAGTGTCCCAGGAGACACCACCCT
SRR940959.1828870.1-  GATAATAAAGACCATTGATGTAAGTGTCCCAGGAGACACCACCCTC
SRR940959.475027989.- GATAATAAAGACCATTGATGTAAGTGTCCCAGGAGACACCACCCTC
SRR940959.35556388.2- GATAATAAAGACCATTGATGTAAGTGTCCCAGGAGACACCACCCTCC
SRR940959.519620866.+ GATAATAAAGACCATTGATGTAAGTGTCCCAGGAGACACCACCCTCC
SRR940959.65486974.1+ GATAATAAAGACCATTGATGTAAGTGTCCCAGGAGACACCACCCTCC
SRR940959.236029452.- GATAATAAAGACCATTGATGTAAGTGTCCCAGGAGACACCACCCTCCC
SRR940959.179648805.+ GATAATAAAGACCATTGATGTAAGTGTCCCAGGAGACACCACCCTCCCAGG
SRR940959.412155320.+ GATAATAAAGACCATTGATGTAAGTGTCCCAGGAGACACCACCCGCCCAGG
SRR940959.226827903.- GATAATAAAGACCATTGATGTAAGTGTCCCAGGAGACACCACCCTCCCAGGGTTAGGGAT
SRR940959.243908493.- GATAATAAAAAACATTGATGTAAGTGTCCCAGGAGACACCACCCCCCCAGGGTTAGGGAT
SRR940959.263968702.- GATAATAAAGACCATTGATGTAAGTGTCCCAGGAGACACCACCCTCCCAGGGTNAGGGAT
SRR940959.503372481.- GATAATAAAGACCATTGATGTAAGTGTCCCAGGAGACACCACCCTCCCAGGGTTAGGGAT
```

```
SRR940959.122190638.+ GATAATAAAGACCATTGATGTAAGTGTCCCAGGAGACACCACCCTCCCAGGGTTAGGGAT
SRR940959.453148914.+ GATAATAAAGACCATTGATGTAAGTGTCCCAGGAGACACCACCCTCCCAGGGTTAGGGAT
SRR940959.326833507.+ GATAATAAAGACCATTGATGTAAGTGTCCCAGGAGACACCACCCTCCCAGGGTTAGGGAT
SRR940959.237694524.+ GATAATAAAGACCATTGATGTAAGTGTCCCAGGAGACACCACCCTCCCAGGGTTAGGGAT
SRR940959.5689026.1+  GATAATAAAGACCATTGATGTAAGTGTCCCAGGAGACACCACCCTCCCAGGGTTAGGGAT
SRR940959.281789585.+ GATAATAAAGACCATTGATGTAAGTGTCCCAGGAGACACCACCCTCCCAGGGTTAGGGAT
SRR940959.519908744.- GATAATAAAGACCATTGATGTAAGTGTCCCAGGAGACACCACCCTCCCAGGGTTAGGGAT
SRR940959.178641573.+  ATAATAAAGACCATTGATGTAAGTGTCCCAGGAGACACCACCCTCCCAGGGTTAGGGAT
SRR940959.404070300.+    AATAAAGACCATTGATGTAAGTGTCCCAGGAGACACCACCCTCCCAGGGTTAGGGAT
SRR940959.510086505.+    AATAAAGACCATTGATGTAAGTGTCCCAGGAGACACCACCCTCCCAGGGTTAGGGAT
                      ____________________________________________________________
consensus             GATAATAAAGACCATTGATGTAAGTGTCCCAGGAGACACCACCCTCCCAGGGTTAGGGAT

                          .    :    .    :    .    :    .    :    .    :    .    :
SRR940959.503372481.- GGA
SRR940959.122190638.+ GGAG
SRR940959.453148914.+ GGAGGGGA
SRR940959.326833507.+ GGAGGGGAGT
SRR940959.237694524.+ GGAGGGGAGTGGGAG
SRR940959.5689026.1+  GGAGGGGAGTGGGAGGGTACTGGACGAGGCTG
SRR940959.281789585.+ GGAGGGGAGTGGGAGGGTACTGGACGAGGCTGGAA
SRR940959.519908744.- GGAGGGGAGTGGGAGGGAACTGGACGAGGCTGGAAAAG
SRR940959.178641573.+ GGAGGGGAGTGGGAGGGTACTGGACGAGGCTGGAAAAGAGA
SRR940959.404070300.+ GGAGGGGAGTGGGAGGGTACTGGACGAGGCTGGAAAAGAGAAG
SRR940959.510086505.+ GGAGGGGAGTGGGAGGGTACTGGACGAGGCTGGAAAAGAGAAN
                      ____________________________________________________________
consensus             GGAGGGGAGTGGGAGGGTACTGGACGAGGCTGGAAAAGAGAAG
```

(13) The exon 13 coding sequence was predicted by assembling WGS data using CAP3. The exon 13 is on the reverse strand and marked in red.

```
                          .    :    .    :    .    :    .    :    .    :    .    :
SRR940959.436485935.+ AAAGCAGAGGGAGTTTCTGTGTTTGGTGATAAGGGGAGGCCTGAGCAGGGAGGGGCCTGA
SRR940959.152918893.+      TAGGAGTTATTCTGTGTTTGGTGATAAGGGGAGGCCTGAGCAGGGAGGGGCCTGA
SRR940959.170308128.-            AGTTTCTGTGTTTGGTGATAAGGGGAGGCCTGAGCAGGGAGGGGCCTGA
SRR940959.39693808.2+                     GTTTGGAGATAAGGGGAGGCCTGAGCAGGGAGGGGCCTGA
SRR940959.399545748.+                     GTTTGGTGATAAGGGGAGGCCTGAGCAGGGAGGGGCCTGA
SRR940959.82532350.2+                         GGAGATAAGGGGAGGCCTGAGCAGGGAGGGGCCTGA
SRR940959.134963357.+                         GGTGATAAGGGGAGGCCTGAGCAGGGAGGGGCCTGA
SRR940959.278028986.+                         GGTGATAAGGGGAGGCCTGAGCAGGGAGGGGCCTGA
SRR940959.221189692.+                         GGTGATAAGGGGAGGCCTGAGCAGGGAGGGGCCTGA
SRR940959.452229668.+                         GGTGATAAGGGGAGGCCTGAGCAGGGAGGGGCCTGA
SRR940959.406199307.+                          GTGATAAGGGGAGGCCTGAGCAGGGAGGGGCCTGA
SRR940959.348358772.-                                AGGGGAGGCCTGAGCAGGGAGGGGCCTGA
SRR940959.407662142.-                                AGGGGAGGCCTGAGCAGGGAGGGGCCTGA
SRR940959.499237524.-                                AGGTGAGGCCTGAGCAGGGAGGGGCCTGA
SRR940959.196223017.+                                    GAGGCCTGAGCAGGGAGGGGCCTGA
SRR940959.373829212.+                                                GGGAGGGGCCTGA
SRR940959.506401365.+                                                GGGAGGGGCCTGA
SRR940959.418047859.+                                                     GGGCCTGA
SRR940959.333017899.+                                                            A
SRR940959.502122397.+                                                            A
                      ____________________________________________________________
consensus             AAAGCAAAGAGAGTTTCTGTGTTTGGTGATAAGGGGAGGCCTGAGCAGGGAGGGGCCTGA

                          .    :    .    :    .    :    .    :    .    :    .    :
SRR940959.436485935.+ GTACGTGGCATGAGGAAACGGTATCAGGCTCCAGCCCGCA
SRR940959.152918893.+ GTACGTGGCATGAGGAAACGGTATCAGGCTCCAGCCCACAGGGTG
SRR940959.170308128.- GTACGTGGCATGAGGAAACGGTATCAGGCTCCAGCCCGCAGGGTGGCTAGG
SRR940959.39693808.2+ GTACGTGGCATGAGGAAACGGTATCAGGCTCCAGCCCGCAGGGTGGCTAGGAGCCAAGAC
SRR940959.399545748.+ GTACGTGGCATGAGGAAACGGTATCAGGCTCCAGCCCGCAGGGTGGCTAGGAGCCAAGAC
SRR940959.82532350.2+ GTACGTGGCATGAGGAAACGGGATCAGGCTCCAGCCCGCAGGGAGGCGAGGAGCCAAGAC
SRR940959.134963357.+ GTACGTGGCATGAGGAAACGGTATCAGGCTCCAGCCCGCAGGGTGGCTAGGAGCCAAGAC
SRR940959.278028986.+ GTACGTGGCATGAGGAAACGGTATCAGGCTCCAGCCCGCAGGGTGGCTAGGAGCCAAGAC
SRR940959.221189692.+ GTACGTGGCATGAGGAAACGGTATCAGGCTCCAGCCCGCAGGGTGGCTAGGAGCCAAGAC
SRR940959.452229668.+ GTACGTGGCATGAGGAAACGGTATCAGGCTCCAGCCCGCAGGGTGGCTAGGAGCCAAGAC
SRR940959.406199307.+ GTACGTGGCATGAGGAAACGGTATCAGGCTCCAGCCCGCAGGGTGGCTAGGAGCCAAGAC
SRR940959.348358772.- GTACGTGGCATGAGGAAACGGTATCAGGCTCCAGCCCGCAGGGTGGCTAGGAGCCAAGAC
SRR940959.407662142.- GTACGTGGCATGAGGAAACGGTATCAGGCTCCAGCCCGCAGGGTGCCTAGGAGCCAAGAC
SRR940959.499237524.- GTACGTGGCATGAGGAAACGGTATCAGGCTCCAGCCCGCAGGGTGGCTAGGAGCCAAGAC
SRR940959.196223017.+ GTACGTGGCATGAGGAAACGGTATCAGGCTCCAGCCCGCAGGGTGGCTAGGAGCCAAGAC
SRR940959.373829212.+ GTACGTGGCATGAGGAAACGGTATCAGGCTCCAGCCCGCAGGGTGGCTAGGAGCCAAGAC
SRR940959.506401365.+ GTANGTGGCATGAGGAAACGGTATCAGGCTCCAGCCCGCAGGGTGGCTAGGAGCCAAGAC
SRR940959.418047859.+ GTACGTGGCATGAGGAAACGGTATCAGGCTCCAGCCCGCAGGGTGGCTAGGAGCCAAGAC
SRR940959.333017899.+ GTACGTGGCATGAGGAAACGGTATCAGGCTCCAGCCCGCAGGGTGGCTAGGAGCCAAGAC
SRR940959.502122397.+ GTACGTGGCATGAGGAAACGGTATCAGGCTCCAGCCCGCAGGGTGGCTTGGAGCCAAGAC
```

```
SRR940959.389678581.+ GTACGTGGCATGAGGAAACGGTATCAGGCTCCAGCCCGCAGGGTGGCTAGGAGCCAAGAC
SRR940959.432579153.+ GTACGTGGCATGAGGAAACGGTATCAGGCTCCAGCCCGCAGGGTGGCTAGGAGCCAAGAC
SRR940959.132704467.+           TGAGGAAACGGTATCAGGCTCCAGCCCGCAGGGTGGCTAGGAGCCAAGAC
SRR940959.207459891.+                                                TAGGAGCCAAGAC
SRR940959.78627270.2+                                                        AAGAC
SRR940959.445230531.+                                          GGTGGCTAGGAGCCAAGAC
SRR940959.447554580.+                                 AGCCCGCAGGGTGGCTAGGAGCCAAGAC
                      ____________________________________________________________
consensus             GTACGTGGCATGAGGAAACGGTATCAGGCTCCAGCCCGCAGGGTGGCTAGGAGCCAAGAC

                          .    :    .    :    .    :    .    :    .    :    .    :
SRR940959.82532350.2+ ACGG
SRR940959.134963357.+ ACGG
SRR940959.278028986.+ ACGG
SRR940959.221189692.+ ACGG
SRR940959.452229668.+ ACGG
SRR940959.406199307.+ ACGGC
SRR940959.348358772.- ACGGCCCCCTG
SRR940959.407662142.- ACGGCCCCCTG
SRR940959.499237524.- ACGGCCCCCTG
SRR940959.196223017.+ ACGGCCCCCTGCCCA
SRR940959.373829212.+ ACGGCCCCCTGCCCAAACAAGAGGGCA
SRR940959.506401365.+ ACGGCCCCCTGCCCAAACAAGAGGGCA
SRR940959.418047859.+ ACGGCCCCCTGCCCAAACAAGAGGGGAGTCAG
SRR940959.333017899.+ ACGGCCCCCTGCCCAAACAAGAGGGCAGTCAGGGGGAGG
SRR940959.502122397.+ ACGGCCCCCTGCCCAAACAAGAGGGCAGTCAGGGGGAGN
SRR940959.389678581.+ ACGGCCCCCTGCCCAAACAAGAGGGCAGTCAGGGGGAGGC
SRR940959.432579153.+ ACGGCCCCCTGCCCAAACAAGAGGGCAGTCAGGGGGAGGC
SRR940959.132704467.+ ACGGCCCCCTGCCCAAACAAGAGGGCAGTCAGGAGGAGGCGCCTTGGTGT
SRR940959.259331275.+    GCCCCCNGCCCAAACAAGAGGGCAGTCAGGNGGAGGCGCCTTGGTGTGGCAGTGCTG
SRR940959.97481744.1+                                                       GTGCTG
SRR940959.262117567.-                                                TGTGGCAGTGCTG
SRR940959.88518966.1+               AAACAAGAGGGCAGTCAGGGGGAGGCGCCTTGGGGTGGCAGTGCTG
SRR940959.97072404.2+                                                   GGCAGTGCTG
SRR940959.207459891.+ ACGGCCCCCTGCCCAAACAAGAGGGCAGTCAGGGGGAGGCGCCTTGGTGTGGCAGTGCTG
SRR940959.406199307.-                                              GGTGTGGCAGTGCTG
SRR940959.6020968.2-        CCCTGCCCAAACAAGAGGGCAGTCAGGGGGAGGCGCCTTGGTGTGGCAGTGCTG
SRR940959.22096478.1+                                                       GTGCTG
SRR940959.24665406.1-                                                          CTG
SRR940959.78627270.2+ ACGGCCCCCTGCCCAAACAAGAGGGCAGTCAGGGGGAGGCGCCTTGGTGTGGCAGTGCTG
SRR940959.145184412.+                                    GAGGCGCCTTGGTGTGGCAGTGCTG
SRR940959.156990194.- ACGGCCCCCTGCCCAAACAAGAGGGCAGTCAGGGGGAGGCGCCTTGGTGTGGCAGTGCTG
SRR940959.157407474.+                                                    GCAGTGCTG
SRR940959.308163200.-                                                     CAGTGCTG
SRR940959.378978605.-                                   GGAGGCGCCTTGGTGTGGCAGTGCTG
SRR940959.423902579.-                                          CCTTGGTGTGGCAGTGCTG
SRR940959.445230531.+ ACGGCCCCCTGCCCAAACAAGAGGGCAGTCAGGGGGAGGCGCCTTGGTGTGGCAGTGCTG
SRR940959.447554580.-                                                      AGTGCTG
SRR940959.447554580.+ ACGGCCCCCTGCCCAAACAAGAGGGCAGTCAGGGGGAGGCGCCTTGGTGTGGCAGTGCTG
                      ____________________________________________________________
consensus             ACGGCCCCCTGCCCAAACAAGAGGGCAGTCAGGGGGAGGCGCCTTGGTGTGGCAGTGCTG

                          .    :    .    :    .    :    .    :    .    :    .    :
SRR940959.259331275.+ GAATCCGAGAGGGCCATGGGGGCAGCCCTGAAGCCTACAGGGG
SRR940959.228322367.-             TCCATGGGGGAAGCCGTGAAGCCGACAGGGGTTCTGTCCTGGGGCTCC
SRR940959.475720970.+                  GAAATCAGCCCTGAAGCCTACAGGGGTTCTGTCCTGGGGCTCC
SRR940959.97481744.1+ GAGCCCGATAGGGCCATGGGGGCAGCCCGGAAGCCTACAGGGGTNCTGTCCTGGGGCTCC
SRR940959.262117567.- GAGCCCGAGAGGGCCATGGGGGCAGCACTGAAGCCTACAGGGGTTCTGTCCTGGGGTTCC
SRR940959.254369319.-                    GGGCAGCCCTGAAGCCTACAGGGGTTCTGTCCTGGGGCTGC
SRR940959.88518966.1+ GAGCNCGAGAGGGCCATGGGGGCAGCCCTGAAGCCTACAGGGGTTCTGTCCTGG
SRR940959.97072404.2+ GAGCACGAGAGGGCCATGGGGGCAGCGCTGAAGCCTACAGGGGTTCTGTCCTGGGGCTCC
SRR940959.207459891.+ GAGCCCGAGAGGGCCATGGGGGGAGCG
SRR940959.32803334.2-          GGGGCCATGGGGGCAGCCCTGAAGTCTACAGGGGTTCTGTCCTGGGGCTCC
SRR940959.82399816.1+            GGCCATGGGGGCAGCCCTGAAGCCTACAGGGGTTCTGTCCTGGGGCTCC
SRR940959.406199307.- GAGCCCGAGAGGGCCATGGGGGCAGCCTTGAAGCCTACAGGGGTTCTGTCCTGGGGCTCC
SRR940959.186105903.-          AGGTCCATGGGGGCAGCCCTGAAGCCTACAGGGGTTCTGTCCTGGGGCTCC
SRR940959.6020968.2-  GAGCCCGAGAGGGCCATGGGGGCAGCCCTGAAGCCTACAGGGGTTC
SRR940959.22096478.1+ GAGCCCGAGAGGGCCATGGGGGCAGCCCTGAAGCCTACAGGGGTTCTGTCCTGGGGCTCC
SRR940959.24665406.1- GAGCCCGAGAGGGCCATGGGGGCAGCCCTGAAGCCTACAGGGGTTCTGTCCTGGGGCTCC
SRR940959.78627270.2+ GAGCCCGAGAGGGCCATGGGGGCAGCCCTGAAGCC
SRR940959.99276329.2-                          CCCTGAAGCCTACAGGGGTTCTGTCCTGGGGCTCC
SRR940959.145184412.+ GAGCCCGAGAGGGCCATGGGGGCAGCCCTGAAGCCTACAGGGGTTCTGTCCTGGGGCTCC
SRR940959.156990194.- GAGCCCGAGAGGGCCATGGGGGCAGCCCTGAAGCCTACAG
SRR940959.157407474.+ GAGCCCGAGAGGGCCATGGGGGCAGCCCTGAAGCCTACAGGGGTTCTGTCCTGGGGCTCC
SRR940959.308163200.- GAGCCCGAGAGGGCCATGGGGGCAGCCCTGAAGCCTACAGGGGTTCTGTCCTGGGGCTCC
SRR940959.341899365.-             GCCATGGGGGCAGCCCTGAAGCCTACAGGGGTTCTGTCCTGGGGCTCC
SRR940959.61344586.1+                    GGGCAGCCCTGAAGCCTACAGGGGTTCTGTCCTGGGGCTCC
```

```
SRR940959.370557701.-                    GGGCAGCCCTGAAGCCTACAGGGGTTCTGTCCTGGGGCTCC
SRR940959.378978605.- GAGCCCGAGAGGGCCATGGGGGCAGCCCTGAAGCCTACAGGGGTTCTGTCCTGGGGCTCC
SRR940959.423902579.- GAGCCCGAGAGGGCCATGGGGGCAGCCCTGAAGCCTACAGGGGTTCTGTCCTGGGGCTCC
SRR940959.249155254.+                                      CAGGGGTTCTGTCCTGGGGCTCC
SRR940959.203758249.+                                      CAGGGGTTCTGTCCTGGGGCTCC
SRR940959.443747155.+                                      CAGGGGTTCTGTCCTGGGGCTCC
SRR940959.445230531.+ GAGCCCGAGAGGGCCATGGGG
SRR940959.447554580.- GAGCCCGAGAGGGCCATGGGGGCAGCCCTGAAGCCTACAGGGGTTCTGTCCTGGGGCTCC
SRR940959.447554580.+ GAGCCCGAGAGG
SRR940959.277699872.-          AGGGCCATGGGGGCAGCCCTGAAGCCTACAGGGGTTCTGTCCTGGGGCTCC
SRR940959.3168619.2+           AGGGCCATGGGGGCAGCCCTGAAGCCTACAGGGGTTCTGTCCTGGGGCTCC
SRR940959.481076725.+          AGGGCCATGGGGGCAGCCCTGAAGCCTACAGGGGTTCTGTCCTGGGGCTCC
SRR940959.516572079.+    CCCGAGAGGGCCATGGGGGCAGCCCTGAAGCCTACAGGGGTTCTGTCCTGGGGCTCC
                      ____________________________________________________________
consensus             GAGCCCGAGAGGGCCATGGGGGCAGCCCTGAAGCCTACAGGGGTTCTGTCCTGGGGCTCC

                          .    :    .    :    .    :    .    :    .    :    .    :
SRR940959.228322367.- GCTTTGCCTCTGGAGGACTCTAAGCAAGGACCCCGGGGAGCAGGGATGATTT
SRR940959.475720970.+ GCTTTGCCTCTGGAGGACTCTAAGCAAGGACCCCGGGGAGCAGGGATGATTTCCGGA
SRR940959.97481744.1+ GCTTTGCCTCTGGAGGACTCTAAGCAAGGACCCC
SRR940959.262117567.- GCTTTGCCTCTGGAGGACTCNAAGCAA
SRR940959.254369319.- GCTTTGCCTCTGGAGGACTCTAAGCAAGTACCCCGGGGAGCAGGGATGATTTNCGGAAT
SRR940959.97072404.2+ GCTTTGCCTCTGGAGGACTCTAAGCAAGGA
SRR940959.32803334.2- GCTTTGCCTCTGGAGGACTCTAAGCAAGGACCCCGGGGAGCAGGGATGA
SRR940959.82399816.1+ GNTTTGCCTCTGGAGGACTCTAAGCAAGGACCCCGGGGAGCAGGGATGATT
SRR940959.406199307.- GCTTTGCCTCTGGAGGACTCTAAGC
SRR940959.186105903.- GCTTTGCCTCTGGAGGACTCTAAGCAAGGACCCCGGGGAGCAGGGATGA
SRR940959.22096478.1+ GCTTTGCCTCTGGAGGACTCTAAGCAAGGACCCC
SRR940959.24665406.1- GCTTTGCCTCTGGAGGACTCTAAGCAAGGACCCCGGG
SRR940959.99276329.2- GCTTTGCCTCTGGAGGACTCTAAGCAAGGACCCCGGGGAGCAGGGATGATTTCCGGAATC
SRR940959.145184412.+ GCTTTGCCTCTGGAG
SRR940959.157407474.+ GCTTTGCCTCTGGAGGACTCTAAGCAAGGAC
SRR940959.308163200.- GCTTTGCCTCTGGAGGACTCTAAGCAAGGACC
SRR940959.341899365.- GCTTTGCCTCTGGAGGACTCTAAGCAAGGACCCCGGGGAGCAGGGATGATTT
SRR940959.61344586.1+ GCTTTGCCTCTGGAGGACTCTAAGCAAGGACCCCGGGGAGCAGGGATGATTTCCGGAAT
SRR940959.370557701.- GCTTTGCCTCTGGAGGACTCTAAGCAAGGACCCCGGGGAGCAGGGATGATTTCCGGAAT
SRR940959.378978605.- GCTTTGCCTCTGGA
SRR940959.423902579.- GCTTTGCCTCTGGAGGACTCT
SRR940959.249155254.+ GCTTTGCCTCTGGAGGACTCTAAGCAAGGACCCCGGGGAGCAGGGATGATTTCCGGAATC
SRR940959.203758249.+ GCTTTGCCTCTGGAGGACTCTAAGCAAGGACCCCGGGGAGCAGGGATGATTTCCGGAATC
SRR940959.443747155.+ GCTTTGCCTCTGGAGGACTCTAAGCAAGGACCCCGGGGAGCAGGGATGATTTCCGGAATC
SRR940959.447554580.- GCTTTGCCTCTGGAGGACTCTAAGCAAGGACCC
SRR940959.277699872.- GCTTTGCCTCTGGAGGACTCTAAGCAAGGACCCCGGGGAGCAGGGATGA
SRR940959.3168619.2+  GCTTTGCCTCTGGAGGACTCTAAGCAAGGACCCCGGGGAGCAGGGATGA
SRR940959.481076725.+ GCTTTGCCTCTGGAGGACTCTAAGCAAGGACCCCGGGGAGCAGGGATGA
SRR940959.516572079.+ GCTTTGCCTCTGGAGGACTCTAAGCAAGGACCCCGGGGAGCAG
SRR940959.321171471.+   TTTGCCTCTGGAGGACTCTAAGCAAGGACCCCGGGGAGCAGGGATGATTTCCGGAATC
SRR940959.4839264.1-             GGAGGACTCTAAGCAAGGACCCCGGGGAGCAGGGATGATTTCCGGAATC
SRR940959.237694524.+            GGAGGACTCTAAGCAAGGAGCCCGGGGAGCAGGGATGATTTCCGGAATC
SRR940959.253459548.+            GGAGGACTCTAAGCAAGGACCCCGGGGAGCAGGGATGATTTCCGGAATC
SRR940959.475720970.-                     TAAGCAAGGACCCCGGGGAGCAGGGATGATTTCCGGAATC
SRR940959.195901113.-                        GCAAGGGCCCCGGGGAGCAGGGATGATTTCCGGAATC
SRR940959.3168619.1-                             GGACCCCGGGGAGCAGGGATGATTTCCGGAATC
SRR940959.389465217.-                            GGACCCCGGGGAGCAGGGATGATTTCCGGAATC
SRR940959.481076725.-                            GGACCCCGGGGAGCAGGGATGATTTCCGGAATC
SRR940959.445230531.-                                  CGGGGAGCAGGGATGATTTCCGGAATC
SRR940959.453148914.+                                  CGGGGAGCAGGGATGATTTCCGGAATC
SRR940959.122190638.+                                    GGGAGCAGGGATGATTTCCGGAATC
SRR940959.178641573.+                                         CAGGGATGATTTCCGGAATC
SRR940959.436637228.-                                         CATGGATGATTTCCGGAATC
SRR940959.404070300.+                                           GGGATGATTTCCGGAATC
SRR940959.275810465.+                                             GATGATTTCCGGAATC
SRR940959.179648805.+                                              ATGATTTCCGGAATC
SRR940959.412155320.+                                              ATGATTTCCTGAATC
SRR940959.114808804.-                                               TGATTTCCGGAATC
SRR940959.336287676.+                                                       GGAATC
SRR940959.349332667.-                                                         AATC
                      ____________________________________________________________
consensus             GCTTTGCCTCTGGAGGACTCTAAGCAAGGACCCCGGGGAGCAGGGATGATTTCCGGAATC

                          .    :    .    :    .    :    .    :    .    :    .    :
SRR940959.99276329.2- CAGCC
SRR940959.249155254.+ CAGCCTGTTGTGTTTTC
SRR940959.203758249.+ CAGCCTGTTGTGTTTTC
SRR940959.443747155.+ CAGCCTGTTGTGTTTTC
SRR940959.321171471.+ CAGCCTGTTGTGTTTTCCACTAGCTCCTGCAGGAGAAGGGAG
SRR940959.4839264.1-  CAGCCTGTTGTGTTTTCCACTAGCTCCTGCAGGAGAAGGGAGAAAATGGGT
```

```
SRR940959.237694524.+ CAGCCTGTTGTGTTTTCCACTAGCTCCTGCAGGAGAAGGGAGAAAATGGGT
SRR940959.253459548.+ CAGCCTGTTGTGTTTTCCACTAGCTCCTGCAGGAGAAGGGAGAAAATGGGT
SRR940959.475720970.- CAGCCTGTTGTGTTTTCCACTAGCTCCTGCAGGAGAAGGGAGAAAATGGGTGAAGGTGGA
SRR940959.195901113.- CAGCCTGTTGTGTTTTCCACTAGCTCCTGCAGGAGAAGGGAGAAAATGGGTGAAGGTGGA
SRR940959.3168619.1-  CAGCCTGTTGTGTTTTCCACTAGCTCCTGCAGGAGAAGGGAGAAAATGGGTGAAGGTGGA
SRR940959.389465217.- CAGCCTGTTGTGTTTTCCACTAGCTCCTGCAGGAGAAGGGAGAAAATGGGTGAAGGTGGA
SRR940959.481076725.- CAGCCTGTTGTGTTTTCCACTAGCTCCTGCAGGAGAAGGGAGAAAATGGGTGAAGGTGGA
SRR940959.445230531.- CAGCCTGTTGTGTTGTCCACTAGCTCCTGCAGGAGAAGGGAGAAAATGGGTGAAGGTGGA
SRR940959.453148914.+ CAGCCTGTTGTGTTTTCCACTAGCTCCTGCAGGAGAAGGGAGAAAATGGGTGAAGGTGGA
SRR940959.122190638.+ CAGCCTGTTGTGTTTTCCACTAGCTCCTGCAGGAGAAGGGAGAAAATGGGTGAAGGTGGA
SRR940959.178641573.+ CAGCCTGTTGTGTTTTCCACTAGCTCCTGCAGGAGAAGGGAGAAAATGGGTGAAGGTGGA
SRR940959.436637228.- CGGCCTGTTGTGTTTTCCACTAGCTCCTGCAGGAGAAGGGAGAAAATGGGTGAAGGTGGA
SRR940959.404070300.+ CAGCCTGTTGTGTTTTCCACTAGCTCATGCAGGAGAAGGGAGAAAATGGGTGAAGGTGGA
SRR940959.275810465.+ CAGCCTGTTGTGTTTTCCACTAGCTCCTGCAGGAGAAGGGAGAAAATGGGTGAAGGTGGA
SRR940959.179648805.+ CAGCCTGTTGTGTTTTCCACTAGCTCCTGCAGGAGAAGGGAGAAAATGGGTGAAGGTGGA
SRR940959.412155320.+ CAGCCTGTTGTGTTTTCCACTAGCTCCTGCAGGAGAAGGGAGAAAATGGGTGAAGGTGGA
SRR940959.114808804.- CAGCCTGTTGTGTTTTNCACTAGCTCCTGCAGGAGNAGGGAGNCAATGGGTGAAGGTGGN
SRR940959.336287676.+ CAGCCTGTTGTGTTTTCCACTAGCTCCTGCAGGAGAAGGGAGAAAATGGGTGAAGGTGGA
SRR940959.349332667.- CAGCCTGTTGTGTTTTCCACTAGCTCCTGCAGGAGAAGGGAGAAAATGGGTGAAGGTGGA
SRR940959.412940078.+   GCCTGTTGTGTTTTCCACTAGCTCCTGCAGGAGAAGGGAGAAAATGGGTGAAGGTGGA
SRR940959.22096478.2-       GTTGTGTTTTCCACTAGCTCCTGCAGGAGAAGGGAGAAAATGGGTGAAGGTGGA
SRR940959.200075456.-        TTGTGTTTTCCACTAGCTCCTGCAGGAGAAGGGAGAAAATGGGTGAAGGTGGA
SRR940959.331457456.-        TTGTGTTTTCCACTAGCTCCTGCAGGAGAAGGGAGAAAATGGGTGAAGGTGGA
SRR940959.196223017.-         TGTGTTTTCCACTAGCTCCTGCAGGAGAAGGGAGAAAATGGGTGAAGGTGGA
                      ____________________________________________________________
consensus             CAGCCTGTTGTGTTTTCCACTAGCTCCTGCAGGAGAAGGGAGAAAATGGGTGAAGGTGGA

                          .    :    .    :    .    :    .    :    .    :    .    :
SRR940959.195901113.- AGC
SRR940959.3168619.1-  AGCATCC
SRR940959.389465217.- AGCATCC
SRR940959.481076725.- AGCATCC
SRR940959.445230531.- AGCATCCCCACCT
SRR940959.453148914.+ AGCATCCCCACCT
SRR940959.122190638.+ AGCATCCCCACCTCC
SRR940959.178641573.+ AGCATCCCCACCTCCTTTCC
SRR940959.436637228.- AGCATCCCCACCTCCTTTCC
SRR940959.404070300.+ AGCATCCCCACCTGCTTTCCTC
SRR940959.275810465.+ AGCATCCCCACCTCCTTTCCTCTC
SRR940959.179648805.+ AGCATCCCCACCTCCTTTCCTCTCC
SRR940959.412155320.+ AGCATCCCCACCTCCTTTCCTCTCC
SRR940959.114808804.- AGCATCCCCACCTCCTTTCCTCTCCT
SRR940959.336287676.+ AGCATCCCCACCTCCTTTCCTCTCCTAAGACCTT
SRR940959.349332667.- AGCATCCCCACCTCCTTTCCTCTCCTAAGACCTTAC
SRR940959.412940078.+ AGCATCCCCACCTCCTTTCCTCTCCTAAGACCTTACTTGGTC
SRR940959.22096478.2- AGCATCCCCACCTCCTTTCCTCTCCTAAGACCTTACTTGGTCATCC
SRR940959.200075456.- AGCATCCCCACCTCCTTTCCTCTCCTAAGACCTTACTTGGTCATCCC
SRR940959.331457456.- AGCATCCCCACCTCCTTTCCTCTCCTAAGACCTTACTTGGTCATCCC
SRR940959.196223017.- AGCATCCCCACCTCCTTTCCTCTCCTAAGACCTTACTTGGTCATCCCC
                      ____________________________________________________________
consensus             AGCATCCCCACCTCCTTTCCTCTCCTAAGACCTTACTTGGTCATCCCC
```

52. Baiji (*Lipotes vexillifer*)

```
>Lipotes_vexillifer No=52 length=1854 name="Baiji"
GTGGCCTGTGCCCTTATCTTTCGGCTTCTCCTACTTACGGCCCTGCCGACCCCCTCTCAAGGCAACCACCTGGGCCTCACATCATGTCTGCGTTATTCCA
CATTCCTAGATCCTTCTAATGTCATTTTCCTGCACTGGGACTTTGACCTTGAGGCTGAGATCATCACTTTTGAGCTCCAGGTCCAGACAGCTGGCTGGGT
GGGCTTGGATATCACAAATCGCTACACCAGAGTGGGAAGCAATCTGGTTATTGGAGGAGTCTCGCCGGACGGCAATGTCTATTTCCCGAATCAGCACCTG
GTGGATGAAGACGCTCTGGAGGAGGGAGCCAGGATGCGGAGCTGCAGGCACTGACAGAAGACACCATCTATACCACCATGCGCTTCTCCAGGCCCTTCCA
CTCCTGTGACCCTCAAGACCAAGACATCACGAGTGACACTGTGAGGGTGCTTGCCACTTACGGCCCAGATGACACTCTGAAGCTGGATTGGGAGCGTACT
TTTGTGAGTCCATCTTCCTGCTCCAAACTGTCCACCCTGATGATCTCGATGTCCCCGAGGACACCATCATCCATGACTTGGAGATCACTGATTTCCTCTT
TCCAGAGGATGACACCACGTACGCCTGCACCTTCCTCCCTCTCTCCATCGTTAGCAAGAAGCACCATATCTACAAGTTTGAGCCCAAGTTGGTCCATCAC
AAGGAGACGATGGTGCACCACATCCTGGTGTACCGCCTGTGGCAACGCCAGTGCTCTGCCCAAGGGCATCAGAGACTGCTACGGGGCCAACCCCGCCTCC
TCCCTCTGCTCGCAGGTCATTGTGGGCTGGCCTGTCAGGGGCACAAGTTACCAGTTTCCAGGTGACGTGGGTATCTCTACTGGGATGGCTTTGGACCCCC
AGTGGGTCTGACTGGAGGTTCACTACAGCAATTTGCACAGTCTTCCTGGTGTGTACGGCTCCTCGGGGATTCGAGTGTACTACACGGCGCAGCTGTGCAA
ACATGACATGGGTGTCCTGCAGCTGGGCTTCTGCACTTTCTCCATCCACTTCATACCCCCGGGCGCCGAGTCCTTCGCATCCTATGGGCTGTGTAAGACG
GAGAAGTTTGAAGAGATGTACGGGGCCCTGGTGCCTGGCATCCAGGTCTGCGGCTACCTGCTCCACACCCACTTGGCTGGCCGCACTCTGCAGGCCGTGC
AAAACAGAAATGGAACACAACGCCGAACAATCTGTAAAGATGATTCCTAAGACTTCAATCTGCAGGAGACTCGAGATTTACCTTCTCGAGTGGCCATCAA
GCCGGGAGATGAATTGCTGGTAGGGTGTCACTACCAGACGCTGGACCGCGATTCCTTGACCTTTGGGGGGTCCCAGCACCGTTAATGAGATGTGCCTCAT
CTTTCTCTTCTACTGTCCCCGAAACAACATCTCCAGCTGCATGGGGTAGCCTGACATCATCTACGTGGCCCATGTGCTGGGGGAGGAGGCATCAGATTCC
ATGCAGGGCATGATGGCCATGAACAATGTGGAGTGGACTCCGGAGAACATTAAAAAGGCTGAGAAGGGCTGTGAGGAGGCCCAGCAGACGGTGATAATAA
AGACCATTGACGAGCTAGTGGAAAACACAACAGGCTGGATTCTGGAAATCATCCCTGCTCCCCGGGGTCCTTGCTTAGAGTCCTCCAGAGGCAAAGTGGA
GCCCCAGGACAGAACCCCTGTAGGCTTCAGGGCTGCCCCCATGGCCCTCTCGGGCTCCGGCACTGCCACACCAAGGCGCCGCCCCCTGACTGCCCTCTTG
TTTGGGCAGAGGGCCGTGTCTTGGTTCCTAGCCACGCTGTAGGCTGGAGCCTGA
```

(1) Exon coordinates

Exon	NCBI Accession	Strand	Start	End
1	KE559720	-	7626275	7626562
2	KE559720	-	7625227	7625369
3	KE559720	-	7623845	7624003
4	KE559720	-	7623522	7623605
5	KE559720	-	7622524	7622692
6	KE559720	-	7622257	7622359
7	KE559720	-	7621893	7622059
8	KE559720	-	7621482	7621573
9	KE559720	-	7621103	7621199
10	KE559720	-	7620916	7620975
11	KE559720	-	7619659	7619790
12	KE559720	-	7618997	7619111
13	KE559720	-	7618456	7618698

53. Sperm whale (*Physeter macrocephalus*)

The gene is not present.

54. Minke whale (*Balaenoptera acutorostrata*)

```
>Balaenoptera_acutorostrata No=54 length=1857 name="Minke whale"
ATGGCCTGTGCCCTTCTCTTTTGGCTTCTCCTACTTACGGCCCTGCCGACCTCCTGTCGAGGCAACCACCTGGGCCCCACATCGCGTCTGCGTTATTCCA
CGTTCCTAGATCCTTCTAATGTCATTTTCCTGCACTGGGACTTTGACCTTGAGGCTGAGATCATCACTTTTGAGCTCCAGGTCCGGACAGCTGGCTGGGT
GGGCTTGGGTATCACAAATCGCTACACCAGAGTGGGAAGCGATCTGGTTGTTGGAGGAGTCTCGCCGGACGGCAATGTCTATTTCTCGGATGAGCACCTG
GTAGACGAAGACGCTCTGGAGGAGGACGAGAGCCAGGACGCGGAGCTGCTGGCACTGACAGAAGATGCCATCTATACCACGATGCGCTTCTCCAGGCCCT
TCCGCTCCTGTGACCCTCATGACTAAGACATCACGAGTGACACTGTGAGGGTGCTTGCCGCCTACGGCCCAGATGCCACTCTGAAGTTGGATCAGGAGCG
TACTTTTGTGAAGTCCATCTTCCTGCTCCAAATTGTCCACCCCAATGATCTCGATGTCCCCGAGGACACCATCATCCATGACTTGGAGATCACTGATTTC
CTCATCCCAGAGGATGACACCACGTATGCCTGCACCTTCCTCCCTCTCCCCATCGTTAGCAAGAAGCACCATATCTACAAGTTTGAGCCCAAGTTGGTCC
ATCACAAGGAGACGATGGTGCACCACATCGTGGGGTACGCCTGCGGCAAGGCCAGTGCTCTCCCCAAGGGCATCAGCGACTGCTATGGGGCCCACCCCGC
CTTCTCCCTCTGCTCGCAGGTCATCGTGGGCTGGGCTGTCGGGGGCACAAGTTACCAGTTTCCAGGTGAGGTGGGTATCTCTATTGGGACGCCTTTGGAC
CCCCAGTGGGTCCGACTGGAGATTCACTACAGCAATTTCCACAGCCTTCCTGGTGTGTACGACTCCTCGGGGATTCGAGTGTACCTCACGGCGCAGCTGC
GCAAATATGACATGGGTGTCCTGCAGCTGGGCTTCTTCACTTTCCCCATCCACTTCATACCCCCGGGCGCCGAGTCCTTCGCGTCCTACGGGCTGTGTAA
GACGGAGAAGTTTGAAGAGATGAACGGGGCCCCGATGCCTGACATCCAGGTCTGCGGCTACCTGCTCCACACCCACTTGGCTGGCCGCACTCTGCAGGCC
GTGCAATACAGAAATGGAACACAACTCCGAACAATCTGTAAACATGATTCCTACGACTTCAATCTGCAGGAGACTCGAGATTTACCTTCTCGAGTGGCCA
TCAAGCCGGGAGATGAATTGCTGGTAGAGTGTCACTACCAGACGCTGGACCGCGACTCCTTGACTTTTGGGGGTCCCAGCACCGTTAATGAGATGTGCTT
CATCTTTCTCTTCTACTATCCCCGAAACAACATCTCCAGCTGCATGGGGTACGCTGACATCATCCACGTGGCCCATGAGCTGGGGGAGGAGGCATCAGAT
TCCATGGAAGGCATGATGGCCATGAACAATGTGGAGTGGACCCCGGAGAACATTAAAAAGGCTGAGAAGGCCTGCAAGGAGGCCCAGCAGACGGTGATAA
TAAAGACCATTGATGAGCTAGTGGAAAACACAACAGGCTGGATTCCGGAAATCATCCCTGCTCCCCGGGGTCCTTGCTTGGAGTCCTCCAGAGGCAAAGT
GGAGCCCCAGGACAGAACCCCTGCAGGCTTCAGGGCGGCCCCCATGGCCCTCTCAGGCTCCAGCACTGCCACACCAAGGCGCCTCCCCCTGACTGCCCTC
TTGTTTGGGCAGGGGGCCGTGTCTTGGCTCCTAGCCACCCTGCGGGCTGGAGCCTGA
```

(1) Exon coordinates

Exon	NCBI Accession	Strand	Start	End
1	KI537599	+	141109	141396
2	KI537599	+	142299	142445
3	KI537599	+	143662	143823
4	KI537599	+	144065	144148
5	KI537599	+	144979	145146
6	KI537599	+	145334	145434
7	KI537599	+	145622	145788
8	KI537599	+	146107	146198
9	KI537599	+	146484	146580
10	KI537599	+	146708	146767
11	KI537599	+	147866	147996
12	KI537599	+	148547	148661
13	KI537599	+	148960	149202

55. Fin whale (*Balaenoptera physalus*)

```
>Balaenoptera_physalus No=55 length=1867 name="Fin whale"
ATGGCCTGTGCCCTTCTCTTTCGGCTTCTTCTACTTACGGCCCTGCCGACCTCCTCTCAAGGCAACCACTTGGGCCCCACATCGCGTCTGCGTTATTCCA
CATTCCTAGATCCTTCTAATGTCATTTTCCTGCACTGAGACTTTGACCTTGAGGCTGAGATCATCACTTTTGAGCTCCAGGTCCGGACAGCTGGCTGGGT
GGGCTTGGGTATCACAAATCGCTACACCAGAGTGGGAAGCGATCTGGTTGTTGGAGGAGTCTCGCCGGACGGCAATGTCTATTTCTCGGATGAGCACCTG
GTAGACGAAGATGCTCTGGAGGAGGACGGGAGCCAGGACGCGGAGCTGCTGGCACTGACAGAAAACGCCATCTATACCACCATGCACTTCTCCAGGCCCT
TCCGCTCCTGTGACCCTCATGACTAAGACATCACGAGTGACACTGTGAGGGTGCTTGCTGCCTATGGCCCAGATGCCACTCTGAAGCTGGATTGGGAGTG
TACTTTTGTGAAGTCCATCTTCCTGCTCCAAATTGTCCACCCCGATGATCTCGATGTCCCCGAGGACACCATCATCCATGACTTGGAGATCACTGATTTC
CTCATCCCAGAGGATGACACCACGTATGCCTGCACCTTCCTCCCTCTCCCCATCATTAGCAAGAAGCACCATATCTACAAGTTTGAGCCCAAGTTGGTCC
ATCACAAGGAGACGATGGTGCACCACATCGTGGGGTACGCCTGCAACAAGGCCAGTGCTCTCCCCAAGGGCATCAGCGACTGCTACGGGGCCCACCCCGC
CTTCTCCCTCTGCTCGCAGGTCATCGTGTGCTGGGCTGTCGGGGGCACAAGTTACCAGTTTCCAGGTGATGTGGGTATCTCTATTGGGACGCCTTTGGAC
ACCCAGTGGGTCTGACTGGAGATTCACTACAGCAATCTCCACAGCCTTCCTGATGTGTACGACTCCTCGGGGATTTGAGTGTACCTCACGGCGCAGCTGC
GCAAATATGACATGGGTGTCCTGCAGCTGGGCTTCTTCACTTTCCCCATCCACTTCATACCCCCGGGCGCCGAGTCCTTCGCGTCCTACGGGCTGTGTAA
GAAGGAGCAGTTTGAAGAGATGAACGGGGCCCCGGTGCCTGACATCCAGGTCTGCGGCTACCTGCTCCACACCCACTTGGCTGGCCTCACTCTGCAGGCC
GTGCAATACAGAAATGGAACACAACTCCGAACAATCTGTAAAGATGATTCCTACGACTTCAATCTGCAGGAGACTCGAGATTTACCTTCTCGAGTGGCCA
TCAAGCTGGGAGATGAATTGCTGGTAGAGTGTCACTACCAGACGCTGGACCGCGACTCCTTGACTTTTGGGGGGTCCCAGCACCATTAATGAGATGTGCC
TCATCTTTCTCTTCTACTATCCCCGAAACAACATCTCCAGCTGCATGGGGTACGCTGACATCATCTACATGGCCCATGAGCTGGGGGAGGAGGCATCAGA
CTCCATGGAGGGTATGATGGCCACGAACAAGGTGGAGTGGACCCCGGAGAACATCAAAAAGGCTGAGAAGGCGTGCAAGGAGGCCCAGCAGACGGTGATA
ATAAAGACCATTGACGAGCTAGTGGAAAACACAACAGGCTGGATTCTGGAAATCATCCCTGCTCCCCGGGGTCCTTGCTTGGAGTCCTCCAGAGGCCTCC
TCCTGGTGGACAGAACCAGGACAGAACCCCTGCAGGCTTCAGGGCGGCCCCCATGGCCCTCTCGGGCTCCAGCACTGCCACACCAAGGCGGCTCCCCCTG
ACTGCCCTCTTGTTTGGTCAGGGGGCCCGTGTCTTGGCTCCTAGCCACCCTGCGGGCTGGAGCCTGA
```

(1) The exon 1 coding sequence was predicted by assembling WGS data using CAP3. The exon 1 is marked in red.

```
                          .    :    .    :    .    :    .    :    .    :    .    :
SRR935201.9073785.2-  GGCAGAGATGCATATAAGTCTGTACTTCTTGAGTGTCCATCTAAGGAGAGATGCTTCAGA
SRR935201.424345987.- GGCAGAGATGCATATAAGTCTGTACTTCTTGAGTGTCCATCTAAGGAGAGATGCTTCAGA
SRR935201.364266009.-   CAGAGATGCATATAAGTCTGTACTTCTTGAGTGTCCATCTAAGGAGAGATGCTTCAGA
SRR935201.25861614.2+                       TACTTCTTGAGTGTCCATCTAAGGAGAGATGCTTCAGA
SRR935201.133197178.+                              TGAGTGTCCATCTAAGGAGAGATGCTTCAGA
SRR935201.191454136.+                              TTAGTGTCCATCTAAGGAGAGATGCTTCAGA
SRR935201.426782528.-                              TGAGTGTCCATCTAAGGAGAGATGCTTCAGA
SRR935201.424002009.+                              TGAGTGTCCATCTAAGGAGAGATGCTTCAGA
SRR935201.392249665.+                              TGAGTGTCCATCTAAGGAGAGATGCTTCAGA
SRR935201.361366665.+                              TGAGTGTCCATCTAAGGAGAGATGCTTCAGA
SRR935201.197090405.+                              TGAGTGTCCATCTAAGGAGAGATGCTTCAGA
SRR935201.432976187.+                              TGAGTGTCCATCTAAGGAGAGATGCTTCAGA
SRR935201.344364882.-                               GAGTGTCCATCTAAGGAGAGATGCTTCAGA
SRR935201.76261506.1-                                  CGATCTTCTAAGGAGAGATGCTTCAGA
SRR935201.326812337.-                                  TGTCCATCTAAGGAGAGATGCTTCAGA
SRR935201.575016821.-                                       ATCTAAGGAGAGATGCTTCAGA
SRR935201.76261506.2+                                        TCTAAGGAGAGATGCTTCAGA
SRR935201.59251103.2+                                           AAGGAGAGATGCTTCAGA
SRR935201.132763088.+                                            AGGAGAGATGCTTCAGA
SRR935201.311754011.+                                            AGGAGAGATGCTTCAGA
SRR935201.473271141.+                                            AGGAGAGATGCTTCAGA
SRR935201.243242132.-                                                 AGATGCTTCAGA
SRR935201.69972366.1+                                                     GCTTCAGA
                      ____________________________________________________________
consensus             GGCAGAGATGCATATAAGTCTGTACTTCTTGAGTGTCCATCTAAGGAGAGATGCTTCAGA

                          .    :    .    :    .    :    .    :    .    :    .    :
SRR935201.9073785.2-  CCCCAGGAGCCATGGCCTGTGCCCTTCTCTTTCGGCTTCT
SRR935201.424345987.- CCCCAGGAGCCATGGCCTGTGCCCTTCTCTTTCGGCTTCT
SRR935201.364266009.- CCCCAGGAGCCATGGCCTGTGCCCTTCTCTTTCGGCTTCTTC
SRR935201.25861614.2+ CCCCAGGAGCCATGGCCTGTGCCCTTCTCTTTCGGCTTCTTCTACTTACGGCCCTGCCGA
SRR935201.133197178.+ CCCCAGGAGCCATGGCCTGTGCCCTTCTCTTTCGGCTTCTTCTACTTACGGCCCTGCCGA
SRR935201.191454136.+ CCACAGGAGCCATGGCCTGTGCCCTTCTCTTTCGGCTTCTTCTACTTACGGCCCTGCCCA
SRR935201.426782528.- CCCCAGGAGCCATGGCCTGTGCCCTTCTCTTTCGGCTTCTTCTACTTACGGCCCTGCCGA
SRR935201.424002009.+ CCCCAGGAGCCATGGCCTGTGCCCTTCTCTTTCGGCTTCTTCTACTTACGGCCCTGCCGA
SRR935201.392249665.+ CCCCAGGAGCCATGGCCTGTGCCCTTCTCTTTCGGCTTCTTCTACTTACGGCCCTGCCGA
SRR935201.361366665.+ CCCCAGGAGCCATGGCCTGTGCCCTTCTCTTTCGGCTTCTTCTACTTACGGCCCTGCCGA
SRR935201.197090405.+ CCCCAGGAGCCATGGCCTGTGCCCTTCTCTTTCGGCTTCTTCTACTTACGGCCCTGCCGA
SRR935201.432976187.+ CCCCAGGAGCCATGGCCTGTGCCCTTCTCTTTCGGCTTCTTCTACTTACGGCCCTGCCGA
SRR935201.344364882.- CCCCAGGAGCCATGGCCTGTGCCCTTCTCTTTCGGCTTCTTCTACTTACGGCCCTGCCGA
SRR935201.76261506.1- CCCCAGGAGCCATGGCCTGTGCCCTTCTCTTTCGGCTTCTTCTACTTACGGCCCTGCCGA
SRR935201.326812337.- CCCCAGGAGCCATGGCCTGTGCCCTTCTCTTTCGGCTTCTTCTACTTACGGCCCTGCCGA
SRR935201.575016821.- CCCCAGGAGCCATGGCCTGTGCCCTTCTCTTTCGGCTTCTTCTACTTACGGCCCTGCCGA
SRR935201.76261506.2+ CCCCAGGAGCCATGGCCTGTGCCCTTCTCTTTCGGCTTCTTCTACTTACGGCCCTGCCGA
SRR935201.59251103.2+ CCCCAGGAGCCATGGCCTGTGCCCTTCTCTTTCGGCTTCTTCTACTTACGGCCCTGCCGA
SRR935201.132763088.+ CCCCAGGAGCCATGGCCTGTGCCCTTCTCTTTCGGCTTCTTCTACTTACGGCCCTGCCGA
SRR935201.311754011.+ CCCCAGGAGCCATGGCCTGTGCCCTTCTCTTTCGGCTTCTTCTACTTACGGCCCTGCCGA
SRR935201.473271141.+ CCCCAGGAGCCATGGCCTGTGCCCTTCTCTTTCGGCTTCTTCTACTTACGGCCCTGCCGA
SRR935201.243242132.- CCCCAGGAGCCATGGCCTGTGCCCTTCTCTTTCGGCTTCTTCTACTTACGGCCCTGCCGA
```

```
SRR935201.69972366.1+ CCCCAGGAGCCATGGCCTGTGCCCTTCTCTTTCGGCTTCTTCTACTTACGGCCCTGCCGA
SRR935201.73763321.1+     AGGAGCCATGGCCTGTGCCCTTCTCTTTCGGCTTCTTCTACTTACGGCCCTGCCGA
SRR935201.207457849.+     AGGAGCCATGGCCTGTGCCCTTCTCTTTCGGCTTCTTCTACTTACGGCCCTGCCGA
SRR935201.29158684.1+      GGAGCCATGGCCTGTGCCCTTCTCTTTCGGCTTCTTCTACTTACGGCCCTGCCGA
SRR935201.398277573.+      GGAGCCATGGCCTGTGCCCTTCTCTTTCGGCTTCTTCTACTTACGGCCCTGCCGA
SRR935201.44027846.1-                   GTGCCCTTCTCTTTCGGCTTCTTCTCCTTACGGCCCTGCCGA
SRR935201.1815142.1-                               TCTTTCGGCTTCTTCTACTTACGGCCCTGCCGA
SRR935201.157997388.+                       CCTTCTCTTTCGGCTTCTTCTACTTACGGCCCTGCCGA
SRR935201.218091354.-                                                       TGCCGA
SRR935201.327637811.-                  TGTGCCCTTCTCTTTCGGCTTCTTCTACTTACGGCCCTGCCGA
SRR935201.358546387.+                                   GCTTCTTCTACTTACGGCCCTGCCGA
SRR935201.389518010.-                        CTTCTCTTTCGGCTTCTTCTACTTACGGCCCTGCCGA
SRR935201.150639808.-                                            CTACTTACGGCCCTGCCGA
SRR935201.437690797.-                                            CTACTTACGGCCCTGCCGA
SRR935201.446733940.-                                                                A
SRR935201.132763088.-                 CTGTGCCCTTCTCTTTCGGCTTCTTCTACTTACGGCCCTGCCGA
SRR935201.473271141.-                 CTGTGCCCTTCTCTTTCGGCTTCTTCTACTTACGGCCCTGCCGA
SRR935201.331190069.+                                              ACTTACGGCCCTGCCGA
SRR935201.173248604.-                                              ACTTACGGCCCTGCCGA
SRR935201.564716463.-                                              ACTTACGGCCCTGCCGA
                      ____________________________________________________________
consensus             CCCCAGGAGCCATGGCCTGTGCCCTTCTCTTTCGGCTTCTTCTACTTACGGCCCTGCCGA

                          .    :    .    :    .    :    .    :    .    :    .    :
SRR935201.25861614.2+ CC
SRR935201.133197178.+ CCTCCTCTC
SRR935201.191454136.+ CCTCCTCTC
SRR935201.426782528.- CCTCCTCTC
SRR935201.424002009.+ CCTCCTCTC
SRR935201.392249665.+ CCTCCTCTC
SRR935201.361366665.+ CCTCCTCTC
SRR935201.197090405.+ CCTCCTCTC
SRR935201.432976187.+ CCTCCTCTC
SRR935201.344364882.- CCTCCTCTCA
SRR935201.76261506.1- CCTCCTCTCAAGG
SRR935201.326812337.- CCTCCTCTCAAGG
SRR935201.575016821.- CCTCCTCTCAAGGCAACC
SRR935201.76261506.2+ CCTCCTCTCAAGGAGATCG
SRR935201.59251103.2+ CCTCCTCTCAAGGCAACCACTT
SRR935201.132763088.+ CCTCCTCTCAAGGCAACCACTTG
SRR935201.311754011.+ CCTCCTCTCAAGGCAACCACTTG
SRR935201.473271141.+ CCTCCTCTCAAGGCAACCACTTG
SRR935201.243242132.- CCTCCTCTCAAGGCAACCACTTGGGCCC
SRR935201.69972366.1+ CCTCCTCTCAAGGCAACCACTTGGGCCCCACA
SRR935201.73763321.1+ CCTCCTCTCAAGGCAACCACTTGGGCCCCACATCGCGTCTGCGT
SRR935201.207457849.+ CCTCCTCTCAAGGCAACCACTTGGGCCCCACATCGCGTCTGCGT
SRR935201.29158684.1+ CCTCCTCTCAAGGCAACCACTTGGGCCCCACATCGCGTCTGCGTT
SRR935201.398277573.+ CCTCCTCTCAAGGCAACCACTTGGGCCCCACATCGCGTCTGCGTT
SRR935201.414521675.+                                                            G
SRR935201.575950964.+                ACCACTTGGGCCCCAGATCGCGTCTGCGTGGTTCCACATTCCTAT
SRR935201.318727657.+                                          CGTTATTCCACATTCCTAG
SRR935201.535624784.+                    CTTGGGCCCCACATCGCGTCTGCGTTATTCCACATTCCTAG
SRR935201.44027846.1- CCTCCTCTCAAGGCAACCACTTGGGCCCCACATCGCGTCTGCGTTATTCCACATTCCT
SRR935201.478866481.+                ACCACTTGGGCCCCACATCGCGTCTGCGTTATTCCACATTCCTAG
SRR935201.1815142.1-  CCTCCTCTCAAGGCAACCACTTGGGCCCCACATCGCGTCTGCGTTATTCCACATTCCTAG
SRR935201.53888912.2+               AACCACTTGGGCCCCACATCGCGTCTGCGTTATTCCACATTCCTAG
SRR935201.157997388.+ CCTCCTCTCAAGGCAACCACTTGGGCCCCACATCGCGTCTGCGTTATTCCACATTCCTAG
SRR935201.218091354.- CCTCCTCTCAAGGCAACCACTTGGGCCCCACATCGCGTCTGCGTTATTCCACATTCCTAG
SRR935201.327637811.- CCTCCTCTCAAGGCAACCACTTGGGCCCCACATCGCGTCTGCGTTATTCCACATTCC
SRR935201.329037840.+                                                            G
SRR935201.331190069.-   TCCTCTCAAGGCAACCACTTGGGCCCCACATCGCGTCTGCGTTATTCCACATTCCTAG
SRR935201.358546387.+ CCTCCTCTCAAGGCAACCACTTGGGCCCCACATCGCGTCTGCGTTATTCCACATTCCTAG
SRR935201.379646131.+                                                           AG
SRR935201.389518010.- CCTCCTCTCAAGGCAACCACTTGGGCCCCACATCGCGTCTGCGTTATTCCACATTCCTAG
SRR935201.427979184.+                ACCACTTGGGCCCCACATCGCGTCTGCGTTATTCCACATTCCTAG
SRR935201.150639808.- CCTCCTCTCAAGGCAACCACTTGGGCCCCACATCGCGTCTGCGTTATTCCACATTCCTAG
SRR935201.437690797.- CCTCCTCTCAAGGCAACCACTTGGGCCCCACATCGCGTCTGCGTTATTCCACATTCCTAG
SRR935201.446733940.- CCTCCTCTCAAGGCAACCACTTGGGCCCCACATCGCGTCTGCGTTATTCCACATTCCTAG
SRR935201.132763088.- CCTCCTCTCAAGGCAACCACTTGGGCCCCACATCGCGTCTGCGTTATTCCACATTC
SRR935201.473271141.- CCTCCTCTCAAGGCAACCACTTGGGCCCCACATCGCGTCTGCGTTATTCCACATTC
SRR935201.243994578.+                    CTTGGGCCCCACATCGCGTCTGCGTTATTCCACATTCCTAG
SRR935201.535557448.+                    CTTGGGCCCCACATCGCGTCTGCGTTATTCCACATTCCTAG
SRR935201.513472459.+                                          CGTTATTCCACATTCCTAG
SRR935201.152459712.+                                          CGTTATTCCACATTCCTAG
SRR935201.73635660.2+                                          CGTTATTCCACATTCCTAG
SRR935201.562202398.+                                          CGTTATTCCACATTCCTAG
SRR935201.331190069.+ CCTCCTCTCAAGGCAACCACTTGGGCCCCACATCGCGTCTGCGTTATTCCACATTCCTAG
```

```
SRR935201.173248604.- CCTCCTCTCAAGGCAACCACTTGGGCCCCACATCGCGTCTGCGTTATTCCACATTCCTAG
SRR935201.564716463.- CCTCCTCTCAAGGCAACCACTTGGGCCCCACATCGCGTCTGCGTTATTCCACATTCCTAG
SRR935201.571476978.+                                                        CCTAG
                      ____________________________________________________________
consensus             CCTCCTCTCAAGGCAACCACTTGGGCCCCACATCGCGTCTGCGTTATTCCACATTCCTAG

                          .    :    .    :    .    :    .    :    .    :    .    :
SRR935201.414521675.+ ATCCTTCTTATGTCATTTTCCTGCATTGAGACTTTGACCTTGAGGCTGCGATCATGACTT
SRR935201.575950964.+ ATCCTTCTAATGTCATTTTCCTGCACTGAGACTTTGACCTTGAGGCTGAGATCAT
SRR935201.115065026.-                                              CTGAGATCATCGCTT
SRR935201.318727657.+ ATCCTTCTAATGTCATTTTCCTGCACTGAGACTTTGACCTTGAGGCTGAGATCATCACTT
SRR935201.535624784.+ ATCCTTCTAATGTCATTTTCCTGCACTGAGACTTTGACCTTGAGGCTGCAATCATCACT
SRR935201.289458396.+                                      CCTTGAGGCTGAGATCATCACTT
SRR935201.393122913.-                                       CTTGAGGCTGAGATCATCGCTT
SRR935201.478866481.+ ATCCTTCTAATGTCATTTTCCTGCACTGAGACTTTGACCTTGAGGCTGAGATCCT
SRR935201.515433105.+                       GCACTGAGACTTTGACCTTGAGGCTGAGATCATCACTT
SRR935201.531888339.-                                              CTGAGATCATCACTT
SRR935201.1815142.1-  ATCCTTC
SRR935201.12427912.2-                                         TGAGGCTGAGATCATCACTT
SRR935201.20882218.2+   CCTTCTAATGTCATTTTCCTGCACTGAGACTTTGACCTTGAGGCTGAGATCATCACTT
SRR935201.53888912.2+ ATCCTTCTAATGTCATTTTCCTGCACTGAGACTTTGACCTTGAGGCTGAGATCA
SRR935201.109776520.+                                                      ATCACTT
SRR935201.157997388.+ AT
SRR935201.73763321.2-                                    GACCTTGAGGCTGAGATCATCACTT
SRR935201.207457849.-                                    GACCTTGAGGCTGAGATCATCACTT
SRR935201.218091354.- ATCCTTCTAATGTCATTTTCCTGCACTGAGACTT
SRR935201.321650446.+                          CTGAGACTTTGACCTTGAGGCTGAGATCATCACTT
SRR935201.329037840.+ ATCCTTCTAATGTCATTTTCCTGCACTGAGACTTTGACCTTGAGGCTGAGATCATCACTT
SRR935201.331190069.- ATCCTTCTAATGTCATTTTCCTGCACTGAGACTTTGACCTTG
SRR935201.358546387.+ ATCCTTCTAATGTC
SRR935201.379646131.+ ATCCTTCTAATGTCATTTTCCTGCACTGAGACTTTGACCTTGAGGCTGAGATCATCACTT
SRR935201.389518010.- ATC
SRR935201.379646131.-                                              CTGAGATCATCACTT
SRR935201.424002009.-                                              CTGAGATCATCACTT
SRR935201.427979184.+ ATCCTTCTAATGTCATTTTCCTGCACTGAGACTTTGACCTTGAGGCTGAGATCAT
SRR935201.150639808.- ATCCTTCTAATGTCATTTTCC
SRR935201.437690797.- ATCCTTCTAATGTCATTTTCC
SRR935201.87675960.2+                                      CCTTGAGGCTGAGATCATCACTT
SRR935201.442686300.+                                      CCTTGAGGCTGAGATCATCACTT
SRR935201.446733940.- ATCCTTCTAATGTCATTTTCCTGCACTGAGACTTTGACC
SRR935201.453622329.-                                       CTTGAGGCTGAGATCATCACTT
SRR935201.30853418.2-                         ACTGAGACTTTGACCTTGAGGCTGAGATCATCACTT
SRR935201.504546149.-                         ACTGAGACTTTGACCTTGAGGCTGAGATCATCACTT
SRR935201.243994578.+ ATCCTTCTAATGTCATTTTCCTGCACTGAGACTTTGACCTTGAGGCTGAGATCATCACT
SRR935201.535557448.+ ATCCTTCTAATGTCATTTTCCTGCACTGAGACTTTGACCTTGAGGCTGAGATCATCACT
SRR935201.513472459.+ ATCCTTCTAATGTCATTTTCCTGCACTGAGACTTTGACCTTGAGGCTGAGATCATCACTT
SRR935201.152459712.+ ATCCTTCTAATGTCATTTTCCTGCACTGAGACTTTGACCTTGAGGCTGAGATCATCACTT
SRR935201.73635660.2+ ATCCTTCTAATGTCATTTTCCTGCACTGAGACTTTGACCTTGAGGCTGAGATCATCACTT
SRR935201.562202398.+ ATCCTTCTAATGTCATTTTCCTGCACTGAGACTTTGACCTTGAGGCTGAGATCATCACTT
SRR935201.331190069.+ ATCCTTCTAATGTCATTTTCCTG
SRR935201.173248604.- ATCCTTCTAATGTCATTTTCCTG
SRR935201.564716463.- ATCCTTCTAATGTCATTTTCCTG
SRR935201.571476978.+ ATCCTTCTAATGTCATTTTCCTGCACTGAGACTTTGACCTTGAGGCTGAGATCATCACTT
                      ____________________________________________________________
consensus             ATCCTTCTAATGTCATTTTCCTGCACTGAGACTTTGACCTTGAGGCTGAGATCATCACTT

                          .    :    .    :    .    :    .    :    .    :    .    :
SRR935201.414521675.+ TTGAGCTCCATCTCCGGACAGCTGGCTGGCTGGGCTTGG
SRR935201.115065026.- TTGAGCTCCAGGTCCGGACAGCTGGCTGGGTGGGCTTGGGTATCGCAAATCGCTGCACCA
SRR935201.318727657.+ TTGGGCTCCTTGTCCGGACAG
SRR935201.289458396.+ TTGAGCTCCAGGTCCGGACAGCTGGCTGGGGGGGCTTGGGTATCACAAATCGCTACACCA
SRR935201.393122913.- TTGAGCTCCAGGTCCGGACAGCTGGCTGGGTGGGCTTGGGTATCACAAATCGCTACACCA
SRR935201.515433105.+ TTGAGCTCCAGGTCCGGACAGCTGGCTGGGTGGGCTTGGGTATCACAAATCGCTACACCA
SRR935201.531888339.- TTGAGCTCCAGGTCCGGACAGCTGGCTGGGTGGGCTTGGGTATCACAAATCGCTACACCA
SRR935201.12427912.2- TTGAGCTCCAGGTCCGGACAGCTGGCTGGGTGGGCTTGGGTATCACAAATCGCTACACCA
SRR935201.20882218.2+ TTGAGCTCCAGGTCCGGACAGCTGGCTGGGTGGGCTTGGGTA
SRR935201.109776520.+ TTGAGCTCCAGGTCCGGACAGCTGGCTGGGTGGGCTTGGGTATCACAAATCGCTACACCA
SRR935201.142711767.-            GTCCGGACAGCTGGCTGGGTGGGCTTGGGTATCACAAATCGCTACACCA
SRR935201.73763321.2- TTGAGCTCCAGGTCCGGACAGCTGGCTGGGTGGGCTTGGGTATCACAAATCGCTACACCA
SRR935201.207457849.- TTGAGCTCCAGGTCCGGACAGCTGGCTGGGTGGGCTTGGGTATCACAAATCGCTACACCA
SRR935201.321650446.+ TTGAGCTCCAGGTCCGGACAGCTGGCTGGGTGGGCTTGGGTATCACAAATCGCTACACCA
SRR935201.329037840.+ TTGAGCTCCAGGTCCGGACAGCTGGCTGGGTGGGCTTGG
SRR935201.379646131.+ TTGAGCTCCAGGTCCGGACAGCTGGCTGGGTGGGCTTG
SRR935201.385489389.+        CCAGGTCCGGACAGCTGGCTGGGTGGGCTTGGGTATCACAAATCGCTACACCA
SRR935201.379646131.- TTGAGCTCCAGGTCCGGACAGCTGGCTGGGTGGGCTTGGGTATCACAAATCGCTACACCA
SRR935201.424002009.- TTGAGCTCCAGGTCCGGACAGCTGGCTGGGTGGGCTTGGGTATCACAAATCGCTACACCA
SRR935201.87675960.2+ TTGAGCTCCAGGTCCGGACAGCTGGCTGGGTGGGCTTGGGTATCACAAATCGCTACACCA
```

```
SRR935201.442686300.+ TTGAGCTCCAGGTCCGGACAGCTGGCTGGGTGGGCTTGGGTATCACAAATCGCTACACCA
SRR935201.453622329.- TTGAGCTCCAGGTCCGGACAGCTGGCTGGGTGGGCTTGGGTATCACAAATCGCTACACCA
SRR935201.30853418.2- TTGAGCTCCAGGTCCGGACAGCTGGCTGGGTGGGCTTGGGTATCACAAATCGCTACACCA
SRR935201.504546149.- TTGAGCTCCAGGTCCGGACAGCTGGCTGGGTGGGCTTGGGTATCACAAATCGCTACACCA
SRR935201.513472459.+ TTGAGCTCCAGGTCCGGACAG
SRR935201.152459712.+ TTGAGCTCCAGGTCCGGACAG
SRR935201.73635660.2+ TTGAGCTCCAGGTCCGGACAG
SRR935201.562202398.+ TTGAGCTCCAGGTCCGGACAG
SRR935201.571476978.+ TTGAGCTCCAGGTCCGGACAGCTGGCTGGGTGGGC
SRR935201.59251103.1-                     GCTGGCTGGGTGGGCTTGGGTATCACAAATCGCTACACCA
SRR935201.385489389.-                            GGGTGGGCTTGGGTATCACAAATCGCTATACCA
SRR935201.257558484.-                             GGTGGGCTTGGGTATCGCAAATCGCTACACCA
SRR935201.25861614.1-                                         TATCACAAATCGCTACACCA
SRR935201.362612925.+                                            CACAAATCGCTACACCA
SRR935201.496945102.+                                            CACAAATCGCTACACCA
SRR935201.368174369.+                                            CACAAATCGCTACACCA
SRR935201.29158684.2-                                                      TACACCA
SRR935201.398277573.-                                                      TACACCA
SRR935201.321650446.-                                                       ACACCA
SRR935201.565423078.-                                                       ACACCA
                      ____________________________________________________________
consensus             TTGAGCTCCAGGTCCGGACAGCTGGCTGGGTGGGCTTGGGTATCACAAATCGCTACACCA

                          .    :    .    :    .    :    .    :    .    :    .    :
SRR935201.115065026.- GAGTGGGAAGCGATCTGGTTGTTGG
SRR935201.289458396.+ GAGTGGGAAGCGATCTG
SRR935201.393122913.- GAGTGGGAAGCGATCTGG
SRR935201.515433105.+ AA
SRR935201.531888339.- GAGTGGGAATCGATCTGGTTGTTGG
SRR935201.12427912.2- GAGTGGGAAGCGATCTGGTT
SRR935201.109776520.+ GAGTGGGAAGCGATCTGGTTGTTGGAGGAGTCT
SRR935201.142711767.- GAGTGGGAAGCGATCTGGTTGTTGGAGGAGTCTCGCCGGACGGCAATGTCT
SRR935201.73763321.2- GAGTGGGAAGCGATC
SRR935201.207457849.- GAGTGGGAAGCGATC
SRR935201.321650446.+ GAGTG
SRR935201.385489389.+ GAGTGGGAAGCGATCTGGTTGTTGGAGGAGTCTCGCCGGACGGCAAT
SRR935201.379646131.- GAGTGGGAAGCGATCTGGTTGTTGG
SRR935201.424002009.- GAGTGGGAAGCGATCTGGTTGTTGG
SRR935201.87675960.2+ GAGTGGGAAGCGATCTG
SRR935201.442686300.+ GAGTGGGAAGCGATCTG
SRR935201.453622329.- GAGTGGGAAGCGATCTGG
SRR935201.30853418.2- GAGT
SRR935201.504546149.- GAGT
SRR935201.59251103.1- GAGTGGGAAGCGATCTGGTTGTTGGAGGAGTCTCGCCGGACGGCAATGTCTATTTCTCGG
SRR935201.385489389.- GAGTGGGAAGCGATCTGGTTGTTGGAGGAGTCTCGCCGGACGGCAATGTCTATTTCTCGG
SRR935201.257558484.- GAGTGGGAAGCGATCTGGTTGTTGGAGGAGTCTCGCCGGACGGCAATGTCTATTTCTCGG
SRR935201.25861614.1- GAGTGGGAAGCGATCTGGTTGTTGGAGGAGTCTCGCCGGACGGCAATGTCTATTTCTCGG
SRR935201.362612925.+ GAGTGGGAAGCGATCTGGTTGTTGGAGGAGTCTCGCCGGACGGCAATGTCTATTTCTCGG
SRR935201.496945102.+ GAGCGGGAAGCGATCTGGTTGTTGGAGGAGTCTCGCCGGACGGCAATGTCTATTTCTCGG
SRR935201.368174369.+ GAGTGGGAAGCGATCTGGTTGTTGGAGGAGTCTCGCCGGACGGCAATGTCTATTTCTCGG
SRR935201.29158684.2- GAGTGGGAAGCGATCTGGTTGTTGGAGGAGTCTCGCCGGACGGCAATGTCTATTTCTCGG
SRR935201.398277573.- GAGTGGGAAGCGATCTGGTTGTTGGAGGAGTCTCGCCGGACGGCAATGTCTATTTCTCGG
SRR935201.321650446.- GAGTGGGAAGCGATCTGGTTGTTGGAGGAGTCTCGCCGGACGGCAATGTCTATTTCTCGG
SRR935201.565423078.- GAGTGGGAAGCGATCTGGTTGTTGGAGGAGTCTCGCCGGACGGCAATGTCTATTTCTCGG
SRR935201.133197178.-  AGTGGGAAGCGATCTGGTTGTTGGAGGAGTCTCGCCGGACGGCAATGTCTATTTCTCGG
SRR935201.191454136.-  AGTGGGAAGCGATCTGGTTGTTGGAGGAGTCTCGCCGGACGGCAATGTCTATTTCTCGG
SRR935201.392249665.-  AGTGGGAAGCGATCTGGTTGTTGGAGGAGTCTCGCCGGACGGCAATGTCTATTTCTCGG
SRR935201.574324593.+     GGGAAGCGATCTGGTTGTTGGAGGAGTCTCGCCGGCCGGCAATGTCTATTTCTCGG
SRR935201.432976187.-        AAGCGATCTGGTTGTTGGAGGAGTCTCGCCGGACGGCAATGTCTATTTCTCGG
SRR935201.170738826.-             ATCTGGTTGTTGGAGGAGTCTCGCCGGACGGCAATGTCTATTTCTCGG
SRR935201.363986206.-             ATCTGGTTGTTGGAGGAGTCTCGCCGGACGGCAATGTCTATTTCTCGG
SRR935201.111717506.+                 GGTTGTTGGAGGAGTCTCGCCGGACGGCAATGTCTATTTCTCGG
SRR935201.473499372.+                 GGTTGTTGGAGGAGTCTCGCCGGACGGCAATGTCTATTTCTCGG
SRR935201.183091863.+                  GTTGTTGGAGGAGTCTCGCCGGACGGCAATGTCTATTTCTCGG
SRR935201.109653199.+                        GGAGGAGTCTCGCCGGACGGCAATGTCTATTTCTCGG
SRR935201.146843232.+                        GGAGGAGTCTCGCCGGACGGCAATGTCTATTTCTCGG
SRR935201.292912934.+                        GGAGGAGTCTCGCCGGACGGCAATGTCTATTTCTCGG
SRR935201.334561442.+                          AGGAGTCTCGCCGGACGGCAATGTCTATTTCTCGG
SRR935201.69972366.2-                               TCTCGCCGGACGGCAATGTCTATTTCTCGG
SRR935201.368174369.-                               TCTCGCCGGACGGCAATGTCTATTTCTCGG
SRR935201.362612925.-                               TCTCGCCGGACGGCAATGTCTATTTCTCGG
SRR935201.496945102.-                               TCTCGCCGGACGGCAATGTCTATTTCTCGG
SRR935201.117862619.+                                          GGCAATGTCTATTTCTCGG
SRR935201.452898640.+                                          GGCAATGTCTATTTCTCGG
SRR935201.479188609.+                                           GCAATGTCTATTTCTCGG
                      ____________________________________________________________
consensus             GAGTGGGAAGCGATCTGGTTGTTGGAGGAGTCTCGCCGGACGGCAATGTCTATTTCTCGG
```

```
                          .    :    .    :    .    :    .    :    .    :    .    :
SRR935201.385489389.- TGACTGA
SRR935201.257558484.- TGACTGTG
SRR935201.25861614.1- TGACTGTGAGGGTGACTCCC
SRR935201.362612925.+ TGACTGTGAGGGTGACTCCCTCC
SRR935201.496945102.+ TGACTGTGAGGGTGACTCCCTCC
SRR935201.368174369.+ TGACTGTGAGGGTGACTCCCTCC
SRR935201.29158684.2- TGACTGTGAGGGTGGCTCCCTCCCAAGCGTGAG
SRR935201.398277573.- TGACTGTGAGGGTGACTCCCTCCCAAGCGTGAG
SRR935201.321650446.- TGACTGTGAGGGTGACTCCCTCCCAAGCGTGAGG
SRR935201.565423078.- TGACTGTGAGGGTGACTCCCTCCCAAGCGTGAGG
SRR935201.133197178.- TGACTGTGAGGGTGACTCCCTCCCAAGCGTGAGGGTCTCTT
SRR935201.191454136.- TGACTGTGAGGGTGACTCCCTCCCAAGCGTGAGGGTCTCTT
SRR935201.392249665.- TGACTGTGAGGGTGACTCCCTCCCAAGCGTGAGGGTCTCTT
SRR935201.574324593.+ TGACTGTGAGGGTGACTCCCTCCCAAGCGTGAGGGTCTCTTGCT
SRR935201.432976187.- TGACTGTGAGGGTGACTCCCTCCCAAGCGTGAGGGTCTCTTGCTGGG
SRR935201.170738826.- TGACTGTGAGGGTGACTCCCTCCCAAGCGTGAGGGTCTCTTGCTGGGTGGAG
SRR935201.363986206.- TGACTGTGAGGGTGACTCCCTCCCAAGCGTGAGGGTCTCTTGCTGGGTGGAG
SRR935201.111717506.+ TGACTGTGAGGGTGACTCCCTCCCAAGCGTGAGGGTCTCTTGCTGGGTGGAGGGTG
SRR935201.473499372.+ TGACTGTGAGGGTGACTCCCTCCCAAGCGTGAGGGTCTCTTGCTGGGTGGAGGGTG
SRR935201.183091863.+ TGACTGTGAGGGTGACTCCCTTCCAAGCGTGAGGGTCTCTTGCTGGGTGGAGGGTGG
SRR935201.109653199.+ TGACTGTGAGGGTGACTCCCTCCCAAGCGTGAGGGTCTCTTGCTGGGTGGAGGGTGGCTC
SRR935201.146843232.+ TGACTGTGAGGGTGACTCCCTCCCAAGCGTGAGGGTCTCTTGCTGGGTGGAGGGTGGCTC
SRR935201.292912934.+ TGACTGTGAGGGTGACTCCCTCCCAAGCGTGAGGGTCTCTTGCTGGGTGGAGGGTGGCTC
SRR935201.334561442.+ TGACTGTGAGGGTGACTCCCTCCCAAGCGTGAGGGTCTCTTGCTGGGTGGAGGGTGGCTC
SRR935201.69972366.2- TGACTGTGAGGGTGACTCCCTCCCAAGCGTGAGGGTCTCTTGCTGGGTGGAGGGTGGCTC
SRR935201.368174369.- TGACTGTGAGGGTGACTCCCTCCCAAGCGTGAGGGTCTCTTGCTGGGTGGAGGGTGGCTC
SRR935201.362612925.- TGACTGTGAGGGTGACTCCCTCCCAAGCGTGAGGGTCTCTTGCTGGGTGGAGGGTGGCTC
SRR935201.496945102.- TGACTGTGAGGGTGACTCCCTCCCAAGCGTGAGGGTCTCTTGCTGGGTGGAGGGTGGCTC
SRR935201.117862619.+ TGACTGTGAGGGTGACTCCCTCCCAAGCGTGAGGGTCTCTTGCTGGGTGGAGGGTGGCTC
SRR935201.452898640.+ TGACTGTGAGGGTGACTCCCTCCCAAGCGTGAGGGTCTCTTGCTGGGTGGAGGGTGGCTC
SRR935201.479188609.+ TGACTGTGAGGGTGACTCCCTCCCAAGCGTGAGGGTCTCTTGCTGGGTGGAGGGTGGCTC
                      ____________________________________________________________
consensus             TGACTGTGAGGGTGACTCCCTCCCAAGCGTGAGGGTCTCTTGCTGGGTGGAGGGTGGCTC

                          .    :    .    :    .    :    .    :    .    :    .    :
SRR935201.109653199.+ TGA
SRR935201.146843232.+ TGA
SRR935201.292912934.+ TGA
SRR935201.334561442.+ TGAGT
SRR935201.69972366.2- TGAGTCCAGG
SRR935201.368174369.- TGAGTCCAGG
SRR935201.362612925.- TGAGTCCAGG
SRR935201.496945102.- TGAGTCCAGG
SRR935201.117862619.+ TGAGTCCAGGAAGGATCCGCA
SRR935201.452898640.+ TGAGTCCAGGAAGGATCCGCA
SRR935201.479188609.+ TGAGTCCAGGAAGGATCCGCAT
                      ____________________________________________________________
consensus             TGAGTCCAGGAAGGATCCGCAT
```

(2) The exon 2 sequence was predicted by assembling WGS data using CAP3. The exon 2 is on the reverse strand and marked in red.

```
                          .    :    .    :    .    :    .    :    .    :    .    :
SRR935201.396075686.+ CCAGATTGGGGGTGACTTCACCTCCTAGAAGGAGAGGCTGCTTGTGAGCAAATGCTCCTC
SRR935201.282773776.-        GGGGGTGACTTCACCTCCTAGAAGGAGAGGCTGCTTGTGAGCAAATGCTCCTC
SRR935201.148777678.+          GGGTGACTTCACCTCCTAGAAGGAGAGGCTGCTTGTGAGCAAATGCTCCTC
SRR935201.500985101.-                      CTCCTAGAAGGAGAGGCTGCTTGTGAGCAAATGCTCCTC
SRR935201.149544437.+                        CCTAGAAGGAGAGGCTGCTTGTGAGCAAATGCTCCTC
SRR935201.296733102.+                        ACTAGAAGGAGAGGCTGCTTGTGAGCAAATGCTCCTC
SRR935201.568966094.+                        CCTAGAAGGAGAGGCTGCTTGTGAGCAAATGCTCCTC
SRR935201.158853085.+                           AGAAGGAGAGGCTGCTTGTGAGCAAATGCTCCTC
SRR935201.371555550.+                           AGAAGGAGAGGCTGCTTGTGAGCAAATGCTCCTC
SRR935201.408624667.-                                  GAGGCTGCTTGTGAGCAAATGCTCCTC
SRR935201.81279148.2+                                    GGCTGCTTGTGAGCAAATGCTCCTC
SRR935201.223225072.+                                    GGCTGCTTGTGAGCAAATGCTCCTC
SRR935201.551926939.+                                    GGCTGCTTGTGAGCAAATGCTCCTC
SRR935201.13557940.2-                                                        TCCTC
                      ____________________________________________________________
consensus             CCAGATTGGGGGTGACTTCACCTCCTAGAAGGAGAGGCTGCTTGTGAGCAAATGCTCCTC

                          .    :    .    :    .    :    .    :    .    :    .    :
SRR935201.396075686.+ TCTCCTCCCTCTTTTACCGTGATGTCTTAGTCATGAGGGT
SRR935201.282773776.- TCTCCTCCCTCTTTTACCGTGATGTCTTGGTCATGAGGGTCACAGGA
SRR935201.148777678.+ TCTCCTCCCTCTTTTACCGTGATGTCTTGGTCATGAGGGTCACAGGAGC
```

```
SRR935201.500985101.- TCTCCTCCCTCTTTTACCGTGATGTCTTGGTCATGAGGGTCACAGGAGCGGAAGGGCCTG
SRR935201.149544437.+ TCTCCTCCCTCTTTTACCGTGATGTCTTAGTCATGAGGGTCACAGGAGCGGAAGGGCCTG
SRR935201.296733102.+ TCTCCTCCCTCTTTTACCGTGATGTCTTAGTCATGAGGGTCACAGGAGCGGAAGGGCCTG
SRR935201.568966094.+ TCTCGTCCCTCTTTTACCGTGATGTCTTAGTCATGAGGGTCACAGGAGCGGAAGGGCCTG
SRR935201.158853085.+ TCTCCTCCCTCTTTTACCGTGATGTCTTGGTCATGAGGGTCACAGGAGCGGAAGGGCCTG
SRR935201.371555550.+ TCTCCTCCCTCTTTTACCGTGATGTCTTGGTCCTGAGGGTCACAGGAGCGGAAGCGCCTG
SRR935201.408624667.- TCTCCTCCCTCTTTTACCGTGATGTCTTGGTCATGAGGGTCACAGGAGCGGAAGGGCCTG
SRR935201.81279148.2+ TCTCCTCCCTCTTTTACCGTGATGGCTTAGTCATGAGGGTCACAGGAGCGGAAGGGCCTG
SRR935201.223225072.+ TCTCCTCCCTCTTTTACCGTGATGTCTTAGTCATGAGGGTCACAGGAGCGGAAGGGCCTG
SRR935201.551926939.+ TCTCCTCCCTCTTTTACCGTGATGTCTTAGTCATGAGGGTCACAGGAGCGGAAGGGCCTG
SRR935201.13557940.2- TCTCCTCCCTCTTTTACCGTGATGTCTTGGTCATGAGGGTCACAGGAGCGGAAGGGCCTG
SRR935201.168361519.-   TCCTCCCTCTTTTACCGTGATGTCTTAGTCATGAGGGTCACAGGAGCGGAAGGGCCTG
SRR935201.437657520.-   TCCTCCCTCTTTTACCGTGATGTCTTAGTCATGAGGGTCACAGGAGCGGAAGGGCCTG
SRR935201.545365925.-                                    AGGGTCACAGGAGCGGAAGGGCCTG
SRR935201.48846068.2+                                                            G
SRR935201.136017825.+                             GGTCATGAGGGTCACAGGAGCGGAAGGGCCTG
SRR935201.152456925.+                             GGTCATGAGGGTCACAGGAGCGGAAGGGCCTG
SRR935201.251505053.+                    TGATGTCTTGGTCATGAGGGTCACAGGAGCGGAAGGGCCTG
SRR935201.289956990.-                                                     AGGGCCTG
SRR935201.101790242.-                       TGTCTTAGTCATGAGGGTCACAGGAGCGGAAGGGCCTG
SRR935201.39871593.1-                                                 CGGAAGGGCCTG
SRR935201.232407083.-                                                 CGGAAGGGCCTG
SRR935201.266211034.+                    TGATGTCTTAGTCATGAGGGTCACAGGAGCGGAAGGGCCTG
SRR935201.359556998.+                                                  GGAAGGGCCTG
SRR935201.394741490.-                         TCTTAGTCATGAGGGTCACAGGAGCGGAAGGGCCTG
SRR935201.445511092.-                                         CACAGGAGCGGAAGGGCCTG
SRR935201.352530289.-                                                     AGGGCCTG
SRR935201.484929360.-                                                     AGGGCCTG
SRR935201.58686060.2+                                                         CCTG
SRR935201.567838055.+                                                         CCTG

consensus             TCTCCTCCCTCTTTTACCGTGATGTCTTAGTCATGAGGGTCACAGGAGCGGAAGGGCCTG

                          .    :    .    :    .    :    .    :    .    :    .    :
SRR935201.500985101.- G
SRR935201.149544437.+ GAG
SRR935201.296733102.+ GAG
SRR935201.568966094.+ GAG
SRR935201.158853085.+ GAGAAG
SRR935201.371555550.+ CAGAAG
SRR935201.408624667.- GAGAAGTGCATGG
SRR935201.81279148.2+ GAGAAGTGCATGGGG
SRR935201.223225072.+ GAGAAGTGCATGGTG
SRR935201.551926939.+ GAGAAGTGCATGGTG
SRR935201.13557940.2- GAGAAGTGCATGGTGGTATAGATGGCGTTTTCTGT
SRR935201.168361519.- GAGAAGTGCATGGTGGTATAGATGGCGTTTTCTGTCAGTGCC
SRR935201.437657520.- GAGAAGTGCATGGTGGTATAGATGGCGTTTTCTGTCAGTGCC
SRR935201.545365925.- GAGAAGTGCATGGTGGTATAGATCGCGTTTTGTGTCAGTGACACAAGCTCCGCGTCCTGG
SRR935201.48846068.2+ GAGAAGTGCATGGTGGTATAGATGGCGTTTTCTGTCAGCGCCAGCAGCTCCGCGTCCTGG
SRR935201.136017825.+ GAGAAGTGCATGGTGGTATAGATGGCGTTTTCTGTCAGTGCCAGCAGCTCCGCGTCCTGG
SRR935201.152456925.+ GAGAAGTGCATGGTGGTATAGATGGCGTTTTCTGTCAGTGCCAGCAGCTCCGCGTCCTGG
SRR935201.251505053.+ GAGAAGTGCATGGTGGTATAGATGGCGTTTTCTGTCAGTGCCAGCAGCTCCGCGTCCTG
SRR935201.289956990.- GAGAAGTGCATGGTGGTATAGATCGCGTTTTCTGTCAGTGCCAGCAGCTCCGCGTCCTGG
SRR935201.101790242.- GAGAAGTGCATGGTGGTATAGATGGCGTTTTCTGTCAGTGCCAGCAGCTCCGCGTCCTGG
SRR935201.39871593.1- GAGAAGTGCATGGTGGTATAGATGGCGTTTTCTGTCAGTGCCAGCAGCTCCGCGTCCTGG
SRR935201.232407083.- GAGAAGTGCATGGTGGTATAGATGGCGTTTTCTGTCAGTGCCAGCAGCTCCGCGTCCTGG
SRR935201.266211034.+ GAGAAGTGCATGGTGGTATAGATGGCGTTTTCTGTCAGTGCCAGCAGCTCCGCGTCCTG
SRR935201.326142233.+     AGTGCATGGTGGTATAGATGGCGTTTTCTGTCAGTGCCAGCAGCTCCGCGTCCTGG
SRR935201.359556998.+ GAGAAGTGCATGGTGGTATAGATGGCGTTTTCTGTCAGTGCCAGCAGCTCCGCGTCCTGG
SRR935201.394741490.- GAGAAGTGCATGGTGGTATAGATGGCGTTTTCTGTCAGTGCCAGCAGCTCCGCGTCCTGG
SRR935201.445511092.- GAGAAGTGCATGGTGGTATAGATGGCGTTTTCTGTCAGTGCCAGCAGCTCCGCGTCCTGG
SRR935201.352530289.- GAGAAGTGCATGGTGGTATAGATGGCGTTTTCTGTCAGTGCCAGCAGCTCCGCGTCCTGG
SRR935201.484929360.- GAGAAGTGCATGGTGGTATAGATGGCGTTTTCTGTCAGTGCCAGCAGCTCCGCGTCCTGG
SRR935201.58686060.2+ GAGAAGTGCATGGTGGTATAGATGGCGTTTTCTGTCAGTGCCAGCAGCTCCGCGTCCTGG
SRR935201.567838055.+ GAGAAGTGCATGGTGGTATAGATGGCGTTTTCTGTCAGTGCCAGCAGCTCCGCGTCCTGG
SRR935201.306736291.+      GTGCATGGTGGTATAGATGGCGTTTTCTGTCAGTGCCAGCAGCTCCGCGTCCTGG
SRR935201.1261219.2+         GCATGGTGGTATAGATGGCGTTTTCTGTCAGTGCCAGCAGCTCCGCGTCCTGG
SRR935201.372868966.+        GCATGGTGGTATAGATGGCGTTTTCTGTCAGTGCCAGCAGCTCCGCGTCCTGG
SRR935201.351218217.+        GCATGGTGGTATAGATGGCGTTTTCTGTCAGTGCCAGCAGCTCCGCGTCCTGG
SRR935201.269233410.+        GCATGGTGGTATAGATGGCGTTTTCTGTCAGTGCCAGCAGCTCCGCGTCCTGG
SRR935201.201193914.+        GCATGGTGGTATAGATGGCGTTTTCTGTCAGTGCCAGCAGCTCCGCGTCCTGG
SRR935201.529704741.-        GCATGGTGGTATAGATGGCGTTTTCTGTCAGTGCCAGCAGCTCCGCGTCCTGG
SRR935201.213051544.+         CATGGTGGTATAGATGGCGTTTTCTGTCAGTGCCAGCAGCTCCGCGTCCTGG
SRR935201.544468074.+                 TATAGATGGCGTTTTCTGTCAGTGCCAGCAGCTCCGCGTCCTGG
SRR935201.141583959.-                   TAGATGGCGTTTTCTGTCAGTGCCAGCAGCTCCGCGTCCTGG
SRR935201.433162811.+                     GATGGCGTTTTCTGTCAGTGCCAGCAGCTCCGCGTCCTGG
SRR935201.487247569.+                     GATGGCGTTTTCTGTCAGTGCCAGCAGCTCCGCGTCCTGG
```

```
SRR935201.58686060.1-                      ATGGCGTTTTCTGTCAGTGCCAGCAGCTCCGCGTCCTGG
SRR935201.567838055.-                      ATGGCGTTTTCTGTCAGTGCCAGCAGCTCCGCGTCCTGG
SRR935201.24847203.2-                            TTTTCTGTCAGTGCCAGCAGCTCCGCGTCCTGG
SRR935201.359556998.-                              TTCTGTCAGTGCCAGCAGCTCCGCGTCCTGG
SRR935201.337549776.+                                     AGTGCCAGCAGCTCCGCGTCCTGG
SRR935201.526398210.+                                     AGTGCCAGCAGCTCCGCGTCCTGG
SRR935201.1261219.1-                                          CCAGCAGCTCCGCGTCCTGG
SRR935201.445506628.-                                         CCAGCAGGTCCGCGTCCTGG
SRR935201.269233410.-                                         CGAGCAGCTCCGCGTCCTGG
SRR935201.543036164.+                                          CAGCAGCTCCGCGTCCTGG
SRR935201.411357332.+                                                           GG
                      ____________________________________________________________
consensus             GAGAAGTGCATGGTGGTATAGATGGCGTTTTCTGTCAGTGCCAGCAGCTCCGCGTCCTGG

                          .    :    .    :    .    :    .    :    .    :    .    :
SRR935201.545365925.- CTCCCGTCCTCCTCC
SRR935201.48846068.2+ CTCCCGTCCTCCTCCAGAGCATCTTCGTCTACCAGGTGC
SRR935201.136017825.+ CTCCCGTC
SRR935201.152456925.+ CTCCCGTC
SRR935201.289956990.- CTCCCGTCCTCCTCCAGAGCATCTTCGTCTAC
SRR935201.101790242.- CT
SRR935201.39871593.1- CTCCCGTCCTCCTCCAGAGCATCTTCGT
SRR935201.232407083.- CTCCCGTCCTCCTCCAGAGCATCTTCGT
SRR935201.326142233.+ CTCCCGTCCTCCTCCAGAGCATCTTCGTCTACCAGGTGCTCATC
SRR935201.359556998.+ CTCCCGTCCTCCTCCAGAGCATCTTCGTC
SRR935201.394741490.- CTCC
SRR935201.445511092.- CTCCCGTCCTCCTCCAGAGC
SRR935201.352530289.- CTCCCGTCCTCCTCCAGAGCATCTTCGTCTAC
SRR935201.484929360.- CTCCCGTCCTCCTCCAGAGCATCTTCGTCTAC
SRR935201.58686060.2+ CTCCCGTCCTCCTCCAGAGCATCTTCGTCTACCAGG
SRR935201.567838055.+ CTCCCGTCCTCCTCCAGAGCATCTTCGTCTACCAGG
SRR935201.306736291.+ CTCCCGTCCTCCTCCAGAGCATCTTCGTCTACCAGGTGCTCATCC
SRR935201.1261219.2+  CTCCCGTCCTCCTCCAGAGCATCTTCGTCTACCAGGTGCTCATCCTG
SRR935201.372868966.+ CTCCCCTCCTCCTCCAGAGCATCTTCGTCTACCAGGTGCTCATCCTG
SRR935201.351218217.+ CTCCCGTCCTCCTCCAGAGCATCTTCGTCTACCAGGTGCTCATCCTG
SRR935201.269233410.+ CTCCCGTCCTCCTCCAGAGCATCTTCGTCTACCAGGTGCTCATCCTG
SRR935201.201193914.+ CTCCCGTCCTCCTCCAGAGCATCTTCGTCTACCAGGTGCTCATCCTG
SRR935201.529704741.- CTCCCGTCCTCCTCCAGAGCATCTTCGTCTACCAGGTGCTCATCCTG
SRR935201.213051544.+ CTCCCGTCCTCCTCCAGCGCATCTTCGTCTACCAGGCGCTCATCCTGG
SRR935201.544468074.+ CTCCCGTCCTCCTCCAGAGCATCTTCGTCTACCAGGTGCTCATCCTGGGGTCCAGG
SRR935201.141583959.- CTCCCGTCCTCCTCCAGAGCATCTTCGTCTACCAGGTGCTCATCCTGGGGTCCAGGGA
SRR935201.433162811.+ CTCCCGTCCTCCTCCAGAGCATCTTCGTCTACCAGGTGCTCATCCTGGGGTCCAGGGAAG
SRR935201.487247569.+ CTCCCGTCCTCCTCCAGAGCATCTTCGTCTACCAGGTGCTCATCCTGGGGTCCAGGGCAG
SRR935201.58686060.1- CTCCCGTCCTCCTCCAGAGCATCTTCGTCTACCAGGTGCTCATCCTGGGGTCCAGGGAAG
SRR935201.567838055.- CTCCCGTCCTCCTCCAGAGCATCTTCGTCTACCAGGTGCTCATCCTGGGGTCCAGGGACG
SRR935201.24847203.2- CTCCCGTCCTCCTCCAGAGCATCTTCGTCTACCAGGTGCTCATCCTGGGGTCCAGGGAAG
SRR935201.359556998.- CTCCCGTCCTCCTCCAGAGCATCTTCGTCTACCAGGTGCTCATCCTGGGGTCCAGGGAAG
SRR935201.337549776.+ CTCCCGTCCTCCTCCAGAGCATCTTCGTCTACCAGGTGCTCATCCTGGGGTCCAGGGAAG
SRR935201.526398210.+ CTCCCGTCCTCCTCCAGAGCAGCTTCGTCTACCAGGTGCTCATCCTGGGGTCCAGGGAAG
SRR935201.1261219.1-  CTCCCGTCCTCCTCCAGAGCATCTTCGTCTACCAGGTGCTCATCCTGGGGTCCAGGGAAG
SRR935201.445506628.- CTCCCATCCTCCTCCAGAGCATCTTCGTCTACCGGGTGCTCATCCTGGGGTCCAGGGAAG
SRR935201.269233410.- CTCCCGTCCTCCTCCAGAGCATCTTCGTCTACCAGGTGCTCATCCTGGGGTCCAGGGAAG
SRR935201.543036164.+ CTCCCGTCCTCCTCCAGAGCATCTTCGTCTACCAGGTGCTCATCCTGGGGTCCAGGGAAG
SRR935201.411357332.+ CTCCCGTCCTCCTCCAGAGCATCTTCGTCTACCAGGTGCTCATCCTGGGGTCCAGGGAAG
SRR935201.398692296.+           CCTCCAGAGCATCTTCGTCTACCAGGTGCTCATCCTGGGGTCCAGGGAAG
SRR935201.421019527.+           CCTCCAGAGCATCTTCGTCTACCAGGTGCTCATCCTGGGGTCCAGGGAAG
SRR935201.454474790.+           CCTCCAGAGCATCTTCGTCTACCAGGTGCTCATCCTGGGGTCCAGGGAAG
SRR935201.382122362.+                   GCATCTTCGTCTACCAGGTGCTCATCCTGGGGTCCAGGGAAG
SRR935201.504795914.-                       CTTCGTCTACCAGGTGCTCATCCTGGGGTCCAGGGAAG
SRR935201.209166132.-                        TTCGTCTACCAGGTGCTCATCCTGGGGTCCAGGGAAG
SRR935201.396075686.-                            TCTACCAGGTGCTCATCCTGGGGTCCAGGGAAG
SRR935201.330842177.+                                CCAGGTGCTCATCCTGGGGTCCAGGGAAG
SRR935201.534089827.-                                CCAGGTGCTCATCCTGGGGTCCAGGGAAG
                      ____________________________________________________________
consensus             CTCCCGTCCTCCTCCAGAGCATCTTCGTCTACCAGGTGCTCATCCTGGGGTCCAGGGAAG

                          .    :    .    :    .    :    .    :    .    :    .    :
SRR935201.58686060.1- G
SRR935201.567838055.- G
SRR935201.24847203.2- GTCAAGG
SRR935201.359556998.- GTCAAGGGG
SRR935201.337549776.+ GTCAAGGGGTAAGAAC
SRR935201.526398210.+ GTCAAGGGGTAAGAAC
SRR935201.1261219.1-  GTCAAGGGGTAAGAACCAGG
SRR935201.445506628.- GTCAAGGGGTAAGAACCAGG
SRR935201.269233410.- GTCAAGGGGTAAGAACCAGG
SRR935201.543036164.+ GTCAAGGGGTAAGAACCAGGA
```

```
SRR935201.411357332.+ GTCAAGGGGTAAGAACCAGGAGCCCTAAGTGGTCACGG
SRR935201.398692296.+ GTCAAGGGGTAAGAACCAGGAGCCCTAAGTGGTCACGGATTCCGAGTAGT
SRR935201.421019527.+ GTCAAGGGGTAAGAACCAGGAGCCCTAAGTGGTCACGGATTCCGAGTAGT
SRR935201.454474790.+ GTCAAGGGGTAAGAACCAGGAGCCCTAAGTGGTCACGGATTCCGAGTAGT
SRR935201.382122362.+ GTCAAGGGGCAAGAACCAGGAGCCCTAAGTGGTCACGGATTCCGAGTAGTCTGGGTCC
SRR935201.504795914.- GTCAAGGGGTAAGAACCAGGAGCCCTAAGTGGTCACGGATTCCGAGTAGTCTGGGTCCAT
SRR935201.209166132.- GTCAAGGGGTAAGAACCAGGAGCCCTAAGTGGTCACGGATTCCGAGTAGTCTGGGTCCAT
SRR935201.396075686.- GTCAAGGGGTAAGAACCAGGAGCCCTAAGTGGTCACGGATTCCGAGTAGTCTGGGTCCAT
SRR935201.330842177.+ GTCAAGGGGTAAGAACCAGGAGCCCTAAGTGGTCACGGATTCCGAGTAGTCTGGGTCCAT
SRR935201.534089827.- GTCAAGGGGTAAGAACCAGGAGCCCTAAGTGGTCACGGATTCCGAGTAGTCTGGGTCCAT
                      ____________________________________________________________
consensus             GTCAAGGGGTAAGAACCAGGAGCCCTAAGTGGTCACGGATTCCGAGTAGTCTGGGTCCAT

                          .    :    .    :    .    :    .    :    .    :    .    :
SRR935201.504795914.- TT
SRR935201.209166132.- TTA
SRR935201.396075686.- TTAGCCC
SRR935201.330842177.+ TTAGCCCCCCT
SRR935201.534089827.- TTAGCCCCCCT
                      ____________________________________________________________
consensus             TTAGCCCCCCT
```

(3) The exon 3 sequence was predicted by assembling WGS data using CAP3. The exon 3 is on the reverse strand and marked in red.

```
                          .    :    .    :    .    :    .    :    .    :    .    :
SRR935201.1450886.2+  AATATTGGGTGCAAGAGGAAGGTCTGGATTGAGAGGAAGATGGGCAGTGGCTGGAGGCGG
SRR935201.96075817.1+    ATTGGGTGCAAGAGGAAGGTCTGGATTGAGAGGAAGATGGGCAGTGGCTGGAGGCGG
SRR935201.46290734.1-       GGGTGCAAGAGGAAGGTCTGGATTGAGAGGAAGATGGGCAGTGGCTGGAGGCGG
SRR935201.138486347.-       GGGTGCAAGAGGAGTGTCTGGATTGAGAGGAAGATGGGCAGTGGCTGGAGGCGG
SRR935201.229081599.+        GGTGCAAGAGGAAGGTCTGGATTGAGAGGAAGATGGGCAGTGGCTGGAGGCGG
SRR935201.350514294.+        GGTGCAAGAGGAAGGTCTGGATTGAGAGGAAGATGGGCAGTGGCTGGAGGCGG
SRR935201.20637280.2+                 GGAAGGTCTGGATTGAGAGGAAGATGGGCAGTGGCTGGAGGCGG
SRR935201.168295365.+                  GAAGGTCTGGATTGAGAGGAAGATGGGCAGTGGCTGGAGGCGG
SRR935201.1450886.1-                          TGGATTGAGAGGAAGATGGGCAGTGGCTGGAGGCGG
SRR935201.375948980.-                         TGGATTGAGAGGAAGATGGGCAGTGGCTGGAGGCGG
SRR935201.378081450.-                         TGGATTGAGAGGAAGATGGGCAGTGGCTGGAGGCGG
SRR935201.267709538.+                           GATTGAGAGGAAGATGGGCAGTGGCTGGAGGCGG
SRR935201.379650762.+                           GATTGAGAGGAAGATGGGCAGTGGCTGGAGGCGG
SRR935201.31577150.2-                               GAGAGGAAGATGGGCAGTGGCTGGAGGCGG
SRR935201.406390881.-                               GAGAGGAAGATAGGCAGTGGCTGGAGGCGG
SRR935201.79492010.2-                                           GGCAGTGGCTGGAGGCGG
SRR935201.575179256.+                                           GTCAGTGGCTGGAGGCGG
SRR935201.454390135.+                                           GGCAGTGGCTGGAGGCGG
                      ____________________________________________________________
consensus             AATATTGGGTGCAAGAGGAAGGTCTGGATTGAGAGGAAGATGGGCAGTGGCTGGAGGCGG

                          .    :    .    :    .    :    .    :    .    :    .    :
SRR935201.1450886.2+  CTGGGTGGGAAGGAGGGGTCTTACATCAGTGATCTCCAAG
SRR935201.96075817.1+ CTGGGTGGGAAGGAGGGGTCTTACATCAGTGATCTCCAAGCCA
SRR935201.46290734.1- CTGGGTGGGAAGGAGGGGTCTTACATCAGTGATCTCCAAGTCATGG
SRR935201.138486347.- CTGGGTGGGAAGGAGGGGTCTTACATCAGTGATCTCCAAGTCATGG
SRR935201.229081599.+ CTGGGGGGGAAGGAGGGGGCTTACATCAGTGATCTCCAAGTCATGGA
SRR935201.350514294.+ CTGGGGGGGAAGGAGGGGTCTTACATCAGTGATCTCCAAGTCATGGA
SRR935201.20637280.2+ CTGGGTGGGAAGGAGGGGTCTTACATCAGTGATCTCCAAGTCATGGATGATGGTGT
SRR935201.168295365.+ CTGGGTGGGAAGGAGGGGGCTTACATCAGTGATCTCCAAGTCATGGATGATGGTGGC
SRR935201.1450886.1-  CTGGGTGGGAAGGAGGGGTCTTACATCAGTGATCTCCAAGTCATGGATGATGGTGTCCTC
SRR935201.375948980.- CTGGGTGGGAAGGAGGGGTCTTACATCAGTGATCTCCAAGTCATGGATGATGGTGTCCTC
SRR935201.378081450.- CTGGGTGGGAAGGAGGGGTCTTACATCAGTGATCTCCAAGTCATGGATGATGGTGTCCTC
SRR935201.267709538.+ CTGGGAGGGAAGGAGGGGTCTTACATCAGTGATCTCCAAGTCATGGATGATGGGGTCCTC
SRR935201.379650762.+ CTGGGTGGGAAGGAGGGGTCTTACATCAGTGATCTCCAAGTCATGGATGATGGGGTCCTC
SRR935201.31577150.2- CTGGGTGGGAAGGAGGGGTCTTACATCAGTGATCTCCAAGTCATGGATGATGGTGTCCTC
SRR935201.406390881.- CTGGGTGGGAAGGAGGGGTCTTACATCAGTGATCTCCAAGTCATTGATGATGGTGTCCTC
SRR935201.79492010.2- CTGGGTGGGAAGGAGGGGTCTTACATCAGTGATCTCCAAGTCATGGATGATGGTGTCCTC
SRR935201.575179256.+ CTGGGGGGGAAGGAGGGGTCTTACCTCAGTGATCTCCAAGTCATGGATGATGGTGTCCTC
SRR935201.454390135.+ CTGGGTGGGAAGGAGGGGGCTTACATCAGTGATCTCCAAGTCATGGATGATGGTGTCCTC
SRR935201.322540647.-               GGGGTCTTACATCAGTGATCTCCAAGTCATGGATGATGGTGTCCTC
SRR935201.78357886.1-                     TTACATCAGTGATCTCCAAGTCATGGATGATGGTGTCCTC
SRR935201.455677027.+                       ACATCAGTGATCTCCAAGTCATGGATGATGGTGTCCTC
SRR935201.528816682.-                          TCAGTGAGCTCCAAGTCATGGATGATGGTGTCCTC
SRR935201.133357305.+                                ATCTCCAAGTCATGGATGATGGTGTCCTC
SRR935201.349910216.-                                                        TCCTC
SRR935201.399785030.+                                                            C
SRR935201.421146447.+                                                            C
SRR935201.516094922.+                           CAGTGATCTCCAAGTCATGGATGATGGTGTCCTC
SRR935201.158067269.+                                ATCTCCAAGTCATGGATGATGGTGTCCTC
```

```
SRR935201.178529342.+                                                            C
SRR935201.198622325.-                            TCAGTGATCTCCAAGTCATGGATGATGGTGTCCTC
SRR935201.245425336.+                                                         CCTC
SRR935201.318823823.+                                                         CCTC
SRR935201.320962983.+                                             GGATGATGGTGTCCTC
SRR935201.349910216.+                                    CCAAGTCATGGATGATGGTGTCCTC
SRR935201.359550837.+                                          CATGGATGATGGTGTCCTC
SRR935201.335857468.+                                          CATGGATGATGGTGTCCTC
SRR935201.416875487.+                                          CATGGATGATGGTGTCCTC
SRR935201.417956087.+                             CAGTGATCTCCAAGTCATGGATGATGGTGTCCTC
                      ____________________________________________________________
consensus             CTGGGTGGGAAGGAGGGGTCTTACATCAGTGATCTCCAAGTCATGGATGATGGTGTCCTC

                          .    :    .    :    .    :    .    :    .    :    .    :
SRR935201.1450886.1-  GGGG
SRR935201.375948980.- GGGG
SRR935201.378081450.- GGGG
SRR935201.267709538.+ GGGGAC
SRR935201.379650762.+ GGGGAC
SRR935201.31577150.2- GGGGACATCG
SRR935201.406390881.- GGGGACATCG
SRR935201.79492010.2- GGGGACATCGAGATCATCGGGG
SRR935201.575179256.+ GGGGACATCGAGATCATCGGGG
SRR935201.454390135.+ GGGGACATCGAGATCATCGGGG
SRR935201.322540647.- GGGGACATCGAGATCATCGGGGTGGACAATTTGGAGCAGGAAGATGGACTTCAC
SRR935201.78357886.1- GGGGACATCGAGATCATCGGGGTGGACAATTTGGAGCAGGAAGATGGACTTCACAAAAGT
SRR935201.455677027.+ GGGGACATCGAGATCATCGGGGTGGACAATTTGGAGCAGGAAGATGGACTTCACAAAAGT
SRR935201.528816682.- GGGGACATCGAGATCATTGGGGTGGACAATTTGGAGCAGGAAGATGGACTTCACAAAAGT
SRR935201.133357305.+ GGGGACATCGAGATCATCGGGGTGGACAATTTGGAGCAGGAAGATGGAATTCACAAAAGT
SRR935201.349910216.- GGGGACATCGAGATCATCGGGGTGGACAATTTGGAGCAGGAAGATGGACTTCACAAAAGT
SRR935201.399785030.+ GGGGACATCGAGATCATCGGGGTGGACAATTTGGAGCAGGAAGATGGACTTCACAAAAGT
SRR935201.421146447.+ GGGGACATCGAGATCATCGGGGCGGACAATTTGGAGCAGGAAGATGGACTTCACAAAAGT
SRR935201.516094922.+ GGGGACATCGAGATCATCGGGGTGGACAATTTGGAGCAGGAAGATGGACTTCACCAAGT
SRR935201.26000639.2-                ATCGGGGTGGACAATTTGGAGCAGGAAGATGGACTTCACAAAAGT
SRR935201.158067269.+ GGGGACATCGAGATCATCGGGGTGGACAATTTGGAGCAGGAAGATGGACTTCACAAAAGT
SRR935201.178529342.+ GGGGACATCGAGATCATCGGGGTGGACAATTTGGAGCAGGAAGATGGACTTCACAAAAGT
SRR935201.198622325.- GGGGACATCGAGATCATCGGGGTGGACAATTTGGAGCAGGAAGATGGACTTCACAAAAGT
SRR935201.245846499.+          GAGATCATCGGGGTGGACAATTTGGAGCAGGAAGATGGACTTCACAAAAGT
SRR935201.245425336.+ GGGGACATCGAGATCATCGGGGTGGACAATTTGGAGCAGGAAGATGGACTTCACAAAAGT
SRR935201.318823823.+ GGGGACATCGAGATCATCGGGGTGGACAATTTGGAGCAGGAAGATGGACTTCACAAAAGT
SRR935201.320962983.+ GGGGACATCGAGATCATCGGGGTGGACAATTTGGAGCAGGAAGATGGACTTCACAAAAGT
SRR935201.345522966.-               CATCGGGGTGGACAATTTGGAGCAGGAAGATGGACTTCACAAAAGT
SRR935201.345991670.+      CATCGAGATCATCGGGGTGGACAATTTGGAGCAGGAAGATGGACTTCACAAAAGT
SRR935201.349910216.+ GGGGACATCGAGATCATCGGGGTGGACAATTTGGAGCAGGAAGATGGACTTCACAAAAGT
SRR935201.359550837.+ GGGGACATCGAGATCATCGGGGTGGACAATTTGGAGCAGGAAGATGGACTTCACAAAAGT
SRR935201.335857468.+ GGGGACATCGAGATCATCGGGGTGGACAATTTGGAGCAGGAAGATGGACTTCACAAAAGT
SRR935201.416875487.+ GGGGACATCGAGATCATCGGGGTGGACAATTTGGAGCAGGAAGATGGACTTCACAAAAGT
SRR935201.417956087.+ GGGGACATCGAGATCATCGGGGTGGACAATTTGGAGCAGGAAGATGGACTTCACAAAAGT
SRR935201.427225310.+   GGACATCGAGATCATCGGGGTGGACAATTTGGAGCAGGAAGATGGACTTCACAAAAGT
SRR935201.145591115.+            GATCATCGGGGTGGACAATTTGGAGCAGGAAGATGGACTTCACAAAAGT
SRR935201.499942980.+            GATCATCGGGGTGGACAATTTGGAGCAGGAAGATGGACTTCACAAAAGT
SRR935201.470468193.+                             ATTTGGAGCAGGAAGATGGACTTCACAAAAGT
SRR935201.332321096.+                                 GGAGCAGGAAGATGGACTTCACAAAAGT
SRR935201.296918896.-                                      AGGAAGATGGACTTCACAAAAGT
SRR935201.454390135.-                                                    CACAAAAGT
SRR935201.575179256.-                                                    CACAAAAGT
                      ____________________________________________________________
consensus             GGGGACATCGAGATCATCGGGGTGGACAATTTGGAGCAGGAAGATGGACTTCACAAAAGT

                          .    :    .    :    .    :    .    :    .    :    .    :
SRR935201.455677027.+ AC
SRR935201.528816682.- ACACT
SRR935201.133357305.+ ACACTCCCAAT
SRR935201.349910216.- ACCCTCCCAATCCAGCTTCAGAGTGGCATCTGGGC
SRR935201.399785030.+ ACAATCCCAATCCAGCTTCAGAGTGGCATCTGGGCCATA
SRR935201.421146447.+ ACACTCCCAATCCAGCTTCAGAGTGGCATCTGGGCCATA
SRR935201.516094922.+ ACACTC
SRR935201.26000639.2- ACACTCCCAATCCAGCTTCAGAGTGGCATCTGGGCCATAGGCAGCAAGCACCCTC
SRR935201.158067269.+ ACACTCCCAAT
SRR935201.178529342.+ ACACTCCCAATCCAGCTTCAGAGTGGCATCTGGGCCATA
SRR935201.198622325.- ACACT
SRR935201.245846499.+ ACACTCCCAATCCAGCTTCAGAGTGGCATCTGGGCCATAGGCAGCAAGC
SRR935201.245425336.+ ACACTCCCAATCCAGCTTCAGAGTGGCATCTGGGCC
SRR935201.318823823.+ ACACTCCCAATCCAGCTTCAGAGTGGCATCTGGGCC
SRR935201.320962983.+ ACACTCCCAATCCAGCTTCAGAGT
SRR935201.345522966.- ACACTCCCAATCCAGCTTCAGAGTGGCATCTGGGCCATAGGCAGCAAGCACCCT
SRR935201.345991670.+ ACACTCCCAATCCAGCTTCAGAGTGGCATCTGGGCCATAGGCAGC
```

```
SRR935201.349910216.+ ACACTCCCAATCCAG
SRR935201.359550837.+ ACACTCCCAATCCAGCTTCAG
SRR935201.335857468.+ ACACTCCCAATCCAGCTTCAG
SRR935201.416875487.+ ACACTCCCAATCCAGCTTCAG
SRR935201.417956087.+ ACACTC
SRR935201.427225310.+ ACACTCCCAATCCAGCTTCAGAGTGGCATCTGGGCCATAGGC
SRR935201.145591115.+ ACACTCCCAATCCAGCTTCAGAGTGGCATCTGGGCCATAGGCAGCAAGCAC
SRR935201.499942980.+ ACACTCCCAATCCAGCTTCAGAGTGGCATCTGGGCCATAGGCAGCAAGCAC
SRR935201.470468193.+ ACACTCCCAATCCAGCTTCAGAGTGGCATCTGGGCCATAGGCAGCAAGCACCCTCACAGT
SRR935201.332321096.+ ACACTCCCAATCCAGCTTCAGAGTGGCATCTGGGCCATAGGCAGCAAGCACCCTCACAGT
SRR935201.296918896.- ACACTCCCAATCCAGCTTCAGAGTGGCATCTGGGCCATAGGCAGCAAGCACCCTCACAGT
SRR935201.454390135.- ACACTCCCAATCCAGCTTCAGAGTGGCATCTGGGCCATAGGCAGCAAGCACCCTCACAGT
SRR935201.575179256.- ACACTCCCAATCCAGCTTCAGAGTGGCATCTGGGCCATAGGCAGCAAGCACCCTCACAGT
                      ____________________________________________________________
consensus             ACACTCCCAATCCAGCTTCAGAGTGGCATCTGGGCCATAGGCAGCAAGCACCCTCACAGT

                          .    :    .    :    .    :    .    :    .    :    .    :
SRR935201.470468193.+ GTCACTCT
SRR935201.332321096.+ GTCACTCTGGGG
SRR935201.296918896.- GTCACTCTGGGGGTGTC
SRR935201.454390135.- GTCACTCTGGGGGTGCTTGCTGCCTATGGCC
SRR935201.575179256.- GTCACTCTGGGGGTGCTTGCTGCCTATGGCC
                      ____________________________________________________________
consensus             GTCACTCTGGGGGTGCTTGCTGCCTATGGCC
```

(4) The exon 4 sequence was predicted by assembling WGS data using CAP3. The exon 4 is on the reverse strand and marked in red.

```
                          .    :    .    :    .    :    .    :    .    :    .    :
SRR935201.377350377.- AACACCATCTCTTCAGAGTGATCCATGCCCACTGCCTATCTCAACCACCTTGACAATGAC
SRR935201.56458410.2+       ATCTCTTCAGAGAGAACCATGCCCACTGCCTATCTCAACCACCTTGACAATGTC
SRR935201.405885811.-               AGAGTGAACCATGCCCACTGCCTATCTCAACCACCTTGACAATGTC
SRR935201.421950586.-               AGAGTGAACCATGCCCACTGCCTATCTCAACCACCTTGACAATGTC
SRR935201.37795310.1-                                GCCTATCTCAACCACCTTGACAATGTC
SRR935201.190316807.-                                GCCTATCTCAACCACCTTGACAATGTC
SRR935201.174012081.-                                  CTATCTCAACCACCTTGACAATGTC
SRR935201.206475851.-                                                  GACAATGTC
SRR935201.214238192.+                                                  GACAATGTC
SRR935201.343615011.-                                                  GACAATGTC
SRR935201.446693248.-                                                  GACAATGTC
SRR935201.187795884.+                                                         TC
SRR935201.138486347.+                                                          C
                      ____________________________________________________________
consensus             AACACCATCTCTTCAGAGTGAACCATGCCCACTGCCTATCTCAACCACCTTGACAATGTC

                          .    :    .    :    .    :    .    :    .    :    .    :
SRR935201.377350377.- CTGAAGTCTGTTTCATGGTACCTTGTAGATATGGTGCTTC
SRR935201.56458410.2+ CTGAAGTCTGTTTCATGGTACCTTGTAGATATGGTGCTTCTTGCTA
SRR935201.405885811.- CTGAAGTCTGTTTCATGGTACCTTGTAGATATGGTGCTTCTTGCTAATGATGGG
SRR935201.421950586.- CTGAAGTCTGTTTCATGGTACCTTGTAGATATGGTGCTTCTTGCTAACGATGGG
SRR935201.37795310.1- CTGAAGTCTGTTTCATGGTACCTTGTAGATATGGTGCTTCTTGCTAACGATGGGGAGAGG
SRR935201.190316807.- CTGAAGTCTGTTTCATGGTACCTTGTAGATATGGTGCTTCTTGCTAACGATGGGGAGAGG
SRR935201.174012081.- CTGAAGTCTGTTTCATGGTACCTTGTAGATATGGTGCTTCTTGCTAACGATGGGGAGAGG
SRR935201.206475851.- CTGAAGTCTGTTTCATGGTACCTTGTAGATATGGTGCTTCTTGCTAACGATGGGGAGAGG
SRR935201.214238192.+ CTGAAGTCTGTTTCATGGTACCTTGTAGATATGGTGCTTCTTGCTAATGATGGGGAGAGG
SRR935201.343615011.- CTGAAGTCTGTTTCATGGTACCTTGTAGATATGGTGCTTCTTGCTAACGATGGGGAGAGG
SRR935201.446693248.- CTGAAGTCTGTTTCATGGTACCTTGTAGATATGGTGCTTCTTGCTAACGATGGGGAGAGG
SRR935201.187795884.+ CTGAAGTCTGTTTCATGGTACCTTGTAGATATGGTGCTTCTTGCTAATGATGGGGAGAGG
SRR935201.138486347.+ CTGAAGTCTGTTTCATGGTTCCTTGTAGATATGGTGCTTCTTGCTAACGATGGGGAGCGG
SRR935201.31577150.1+   GAAGTCTGTTTCATGGTACCTTGTAGATATGGTGCTTCTTGCTAATGATGGGGAGAGG
SRR935201.367839838.-    AAGTCTGTTTCATGGTACCTTGTAGATATGGTGCTTCTTGCTAATGATGGGGAGAGG
SRR935201.444113834.-    AAGTCTGTTTCATGGTACCTTGTAGATATGGTGCTTCTTGCTAACGATGGGGAGAGG
SRR935201.133049552.-                     CCTTGTAGATATGGTGCTTCTTGCTAATGAGGGGGAGAGA
SRR935201.254656120.+                     CCTTGTAGATATGGTGCTTCTTGCTAATGATGGGGAGAGG
SRR935201.335292289.+                            GATATGGTGCTTCTTGCTAACGATGGGGAGAGG
SRR935201.198622325.+                                 GGTGCTTCTTGCTAACGATGGGGAGAGG
SRR935201.375948980.+                                    GCTTCTTGCTAACGATGGGGAGAGG
SRR935201.21920591.1-                                     CTTCTTGCTAATGATGGGGAGAGG
SRR935201.79492010.1+                                           GCTAATGATGGGGAGAGG
SRR935201.322540647.+                                             TAATGATGGGGAGAGG
SRR935201.78357886.2+                                               ATGATGGGGAGAGG
SRR935201.263261049.-                                                     GGGGAGAGG
SRR935201.505312267.-                                                     GGGGAGAGG
SRR935201.327390425.+                                                      GGGAGAGG
SRR935201.531849569.-                                                       GGAGAGG
                      ____________________________________________________________
```

```
consensus             CTGAAGTCTGTTTCATGGTACCTTGTAGATATGGTGCTTCTTGCTAACGATGGGGAGAGG

                          .    :    .    :    .    :    .    :    .    :    .    :
SRR935201.37795310.1- GAGGAAGGTGCAG
SRR935201.190316807.- GAGGAAGGTGCAG
SRR935201.174012081.- GAGGAAGGTGCAGGC
SRR935201.206475851.- GAGGAAGGTGCAGGCATACGTGGTGTCATCC
SRR935201.214238192.+ GAGGAAGGTGCAGGCATACGTGGTGTCATCC
SRR935201.343615011.- GAGGAAGGTGCAGGCATACGTGGTGTCATCC
SRR935201.446693248.- GAGGAAGGTGCAGGCATACGTGGTGTCATCC
SRR935201.187795884.+ GAGGAAGGTGCAGGCATACGTGGTGTCATCCTCTGGGA
SRR935201.138486347.+ GAGGAAGGTGCAGGCATACGTGGTGTCATCCTCTGGGAT
SRR935201.31577150.1+ GAGGAAGGTGCAGGCATACGTGGTGTCATCCTCTGGGATGAG
SRR935201.367839838.- GAGGAAGGTGCAGGCATACGTGGTGTCATCCTCTGGGATGAGG
SRR935201.444113834.- GAGGAAGGTGCAGGCATACGTGGTGTCATCCTCTGGGATGAGG
SRR935201.133049552.- GAGGAAGGTGCAGGCATACGTGGTGTCATCCTCTGGGATGAGGAACTGGAGGAGGACACA
SRR935201.254656120.+ GAGGAAGGTGCAGGCATACGTGGTGTCATCCTCTGGGATGAGGAACTGGAGGAGGACACA
SRR935201.335292289.+ GAGGAAGGTGCAGGCATACGTGGTGTCATCCTCTGGGATGAGGAACTGGAGGAGGACACA
SRR935201.198622325.+ GAGGAAGGTGCAGGCATACGTGGTGTCATCCTCTGGGATGAGGAACTGGAGGAGGACACA
SRR935201.375948980.+ GAGGAAGGTGCAGGCATACGTGGTGTCATCCTCTGGGATGAGGAACTGGAGGAGGACACA
SRR935201.21920591.1- GAGGAAGGTGCAGGCATACGTGGTGTCATCCTCTGGGATGAGGAACTGGAGGAGGACACA
SRR935201.79492010.1+ GAGGAAGGTGCAGGCATACGTGGTGTCATCCTCTGGGATGAGGAACTGGAGGAGGACACA
SRR935201.322540647.+ GAGGAAGGTGCAGGCATACGTGGTGTCATCCTCTGGGATGAGGAACTGGAGGAGGACACA
SRR935201.78357886.2+ GAGGAAGGTGCAGGCATACGTGGTGTCATCCTCTGGGATGAGGAACTGGAGGAGGACACA
SRR935201.263261049.- GAGGAAGGTGCAGGCATACGTGGTGTCATCCTCTGGGATGAGGAACTGGAGGAGGACACA
SRR935201.505312267.- GAGGAAGGTGCAGGCATACGTGGTGTCATCCTCTGGGATGAGGAACTGGAGGAGGACACA
SRR935201.327390425.+ GAGGAAGGTGCAGGCATACGTGGTGTCATCCTCTGGGATGAGGAACTGGAGGAGGACACA
SRR935201.531849569.- GAGGAAGGTGCAGGCATACGTGGTGTCATCCTCTGGGATGAGGAACTGGAGGAGGACACA
SRR935201.345522966.+   GGAAGGTGCAGGCATACGTGGTGTCATCCTCTGGGATGAGGAACTGGAGGAGGACACA
SRR935201.46290734.2+           CAGGCATACGTGGTGTCATCCTCTGGGATGAGGAACTGGAGGAGGACACA
SRR935201.95297502.1+             GGCATACGTGGTGTCATCCTCTGGGATGAGGAACTGGAGGAGGACACA
SRR935201.497355211.+              GCATACGTGGTGTCATCCTCTGGGATGAGGAACTGGAGGAGGACACA
SRR935201.168146969.+                ATACGTGGTGTCATCCTCTGGGATGAGGAACTGGAGGAGGACACA
SRR935201.315384175.+                ATACGTGGTGTCATCCTCTGGGATGAGGAACTGGAGGAGGACACA
SRR935201.528816682.+                       GTGTCATCCTCTGGGATGAGAAACTGGAGGAGGACACA
SRR935201.233922287.+                              CCTCTGGGATGAGGAACTGGAGGAGGACACA
                      ____________________________________________________________
consensus             GAGGAAGGTGCAGGCATACGTGGTGTCATCCTCTGGGATGAGGAACTGGAGGAGGACACA

                          .    :    .    :    .    :    .    :    .    :    .    :
SRR935201.335292289.+ CAGAGGC
SRR935201.198622325.+ CAGAGGCTGAGC
SRR935201.375948980.+ CAGAGGCTGAGCAGG
SRR935201.21920591.1- CAGAGGCTGAGCAGGT
SRR935201.79492010.1+ CAGAGGCTGAGCAGGTGGTACG
SRR935201.322540647.+ CAGAGGCTGAGCAGGTGGTACGGA
SRR935201.78357886.2+ CAGAGGCTGAGCAGGTGGGACGGAGG
SRR935201.263261049.- CAGAGGCTGAGCAGGTGGTACGGAGGCTCCC
SRR935201.505312267.- CAGAGGCTGAGCAGGTGGTACGGAGGCTCAC
SRR935201.327390425.+ CAGAGGCTGAGCAGGTGGTACGGAGGCTCCCG
SRR935201.531849569.- CAGAGGCTGAGCAGGTGTTACGGAGGCTGTTCC
SRR935201.345522966.+ CAGAGGCTGAGCAGGTGGTACGGAGGCTCCCGGAGAAACAGG
SRR935201.46290734.2+ CAGAGGCTGAGCAGGTGGTACGGAGGCTCCCGGAGAAACAGGGGAAGGA
SRR935201.95297502.1+ CAGAGGCTGAGCAGGTGGTACGGAGGCTCCCGGAGAAACAGGGGAAGGGACA
SRR935201.497355211.+ CAGAGGCTGAGCAGGTGGTACGGAGGCTCCCGGAGAAACAGGGGAAGGGACAG
SRR935201.168146969.+ CAGAGGCTGAGCAGGTGGTACGGAGGCTCCCGGAGAAACAGGGGAAGGGACAGGT
SRR935201.315384175.+ CAGAGGCTGAGCAGGTGGTACGGTGGCTCCCGGAGAAACAGGGGAAGGGACAGGT
SRR935201.528816682.+ CAGAGGCTGAGCAGGTGGTACGGAGGCTCCCGGAGAAACAGGGGAAGGGACAGGTCGGGG
SRR935201.233922287.+ CAGAGGCTGAGCAGGTGGTACGGAGGCTCCCGGAGAAACAGGGGAAGGGACAGGTCGGGG
                      ____________________________________________________________
consensus             CAGAGGCTGAGCAGGTGGTACGGAGGCTCCCGGAGAAACAGGGGAAGGGACAGGTCGGGG

                          .    :    .    :    .    :    .    :    .    :    .    :
SRR935201.528816682.+ AA
SRR935201.233922287.+ AAGGGCGGC
                      ____________________________________________________________
consensus             AAGGGCGGC
```

(5) The exon 5 sequence was predicted by assembling WGS data using CAP3. The exon 5 is on the reverse strand and marked in red.

```
                          .    :    .    :    .    :    .    :    .    :    .    :
SRR935201.326485891.+ CCCCACCTTCCATCAATCCAACAATCCCAGTTGCCCAACCACACGTGTCCCCTTAGCCGC
SRR935201.168348465.+                   CAACAATCCCAGTTGCCCAACCACACGTGTCCCCTTAGCCGC
SRR935201.217277601.+                   CAACAATCCCAGTTGCCCAACCACACGTGTCCCCTTAGCCGC
SRR935201.474485070.+                   CAACAATCCCAGTTGCCCAAACACACGTGTCCCCTTAGCCGC
```

```
SRR935201.244710570.+                   CAACAATCCCAGTTGCCCAACCACACGTGTCCCCTTAGCCGC
SRR935201.270328450.+                   CAACAATCCCAGTTGCCCAACCACACGTGTCCCCTTAGCCGC
SRR935201.365807312.-                                 GCCCAACCACACGTGTCCCCTTAGCCGC
SRR935201.517333075.-                                                TGTCCCCTTAGCCGC
SRR935201.496144677.+                                                 GTCCCCTTAGCCGC
SRR935201.107336025.+                                                    CCCTTAGCCGC
SRR935201.421359281.-                                                     CCTTAGCCGC
SRR935201.47735361.1+                                                             GC
SRR935201.244281313.+                                                             GC
SRR935201.362541150.+                                                             GC
                      ____________________________________________________________
consensus             CCCCACCTTCCATCAATCCAACAATCCCAGTTGCCCAACCACACGTGTCCCCTTAGCCGC

                          .    :    .    :    .    :    .    :    .    :    .    :
SRR935201.326485891.+ TGATTTCTCTCATTAACACGACTCACTGTGCCCCCGACAG
SRR935201.168348465.+ TGATTTCTCTCATTAACACGACTCACTGTGCCCCCGCCAGCCCAGCACACGATGACCT
SRR935201.217277601.+ TGATTTCTCTCATTAACACGACTCACTGTGCCCCCGACAGCCCAGCACACGATGACCT
SRR935201.474485070.+ TGATTTCTCTCATTAACACGACTCACTGTGCCCCCGACAGCACAGCACACGATGACCT
SRR935201.244710570.+ TGATTTCTCTCATTAACACGACTCACTGTGCCCCCGACAGCCCAGCACACGATGACCT
SRR935201.270328450.+ TGATTTCTCTCATTAACACGACTCACTGTGCCCCCGACAGCCCAGCACACGATGACCT
SRR935201.365807312.- TGATTTCTCTCATTAACACGACTCACTGTGCCCCCGACAGCCCAGCACACGATGACCTGC
SRR935201.517333075.- TGATTTCTCTCATTAACACGACTCACTGTCCCCCCGACAGCCCAGCACACGATGACCTGC
SRR935201.496144677.+ TGATTTCTCTCATTAACACGACTCACTGTGCCCCCGACAGCCCAGCACACGATGACCTGC
SRR935201.107336025.+ TGATTTCTCTCATTAACACGACTCACTGTGCCCCCGACAGCCCAGCACACGATGACCTGC
SRR935201.421359281.- TGATTTCTCTCATTAACACGACTCACTGTGCCCCCGACAGCCCAGCACACGATGACCCGC
SRR935201.47735361.1+ TGATTTCTCTCATTAACACGACTCACTGTGCCCCCGACAGCCCAGCACACGATGACCTGC
SRR935201.244281313.+ TGATTTCTCTCATTAACACGACTCACTGTGCCCCCGACAGCCCAGCACACGATGACCTGC
SRR935201.362541150.+ TGATTTCTCTCATTAACACGACTCACTGTGCCCCCGACAGCCCAGCACACGATGACCTGC
SRR935201.120428108.+         CTCATTAACACGACTCACTGTGCCCCCGACAGCCCAGCACACGATGACCTGC
SRR935201.476854894.-          TCATTAACACGACTCACTGTGCCCCCGACAGCCCAGCACACGATGACCTGC
SRR935201.264292282.+               AACACGACTCACTGTGCCCCCGACAGCCCAGCACACGATGACCTGC
SRR935201.154959351.+                ACACGACTCACTGTGCCCCCGACAGCCCAGCACACGATGACCTGC
SRR935201.532384649.+                ACACGACTCACTGTGCCCCCGACAGCCCAGCACACGATGACCTGC
SRR935201.217520941.+                 CACGACTCACTGTGCCCCCGACAGCCCAGCACACGATGACCTGC
SRR935201.217277601.-                         ACTGTGCCCCCGACAGCCCAGCACACGATGACCTGC
SRR935201.244710570.-                         ACTGTGCCCCCGACAGCCCAGCACACGATGACCTGC
SRR935201.140642164.-                                                            C
SRR935201.369572431.+                                                         CTGC
SRR935201.527597468.+                                                         CTGC
SRR935201.330329116.-                                           CAGCACACGATGACCTGC
SRR935201.117996621.+                                                        CCTGC
SRR935201.298104247.+                                        GCCCAGCACACGATGACCTGC
SRR935201.393070564.+                                             GCACACGATGACCTGC
SRR935201.431238920.+                                                   GATGACCTGC
SRR935201.486494719.-                                           CAGCACACGATGACCTGC
                      ____________________________________________________________
consensus             TGATTTCTCTCATTAACACGACTCACTGTGCCCCCGACAGCCCAGCACACGATGACCTGC

                          .    :    .    :    .    :    .    :    .    :    .    :
SRR935201.365807312.- GAGCAGAGGGAG
SRR935201.517333075.- GAGCAGAGGGAGAAGGCGGGGTGGG
SRR935201.496144677.+ AAGCAGAGGGAGAAGGCGGGGTGGGC
SRR935201.107336025.+ GAGCAGAGGGAGAAGGCGGGGTGGGCCCC
SRR935201.421359281.- GAGCAGAGGGAGAAGGCGGGGTGGGCCCCG
SRR935201.47735361.1+ GAGCAGAGGGTGAATGCGGGGTGGGCCCCGTAGCAGCC
SRR935201.244281313.+ GAGCAGAGGGAGAAGGCGGGGTGGGCCCCGTAGCAGTC
SRR935201.362541150.+ GAGCAGAGGGAGAAGGCGGGGTGGGCCCCGTAGCAGTC
SRR935201.120428108.+ AAGCAGAGGGAGAAGGCGGGGTGGGCCCCGTAGCAGTCGCTGATGCCC
SRR935201.476854894.- AAGCAGAGGGAGAAGGCGGGGTGGGCCCCGTAGCAGTCGCTGATGCCCT
SRR935201.264292282.+ AAGCAGAGGGAGAAGGCGGGGTGGGCCCCGTAGCAGTCGCTGATGCCCTTGGGG
SRR935201.154959351.+ AAGCAGAGGGAGAAGGCGGGGTGGGCCCCGTAGCAGTCGCTGATGCCCTTGGGGA
SRR935201.532384649.+ GAGCAGAGGGAGAAGGCGGGGTGGGCCCCGTAGCAGTCGCTGATGCCCTTGGGGA
SRR935201.217520941.+ GAGCAGAGGGAGAAGGCGGGGTGGGCCCCGTAGCAGTCGCTGATGCCCTTGGGGAG
SRR935201.217277601.- AAGCAGAGGGAGAAGGCGGGGTGGGCCCCGTAGCAGTCGCTGATGCCCTTGGGGAGAGCA
SRR935201.244710570.- AAGCAGAGGGAGAAGGCGGGGTGGGCCCCGTAGCAGTCGCTGATGCCCTTGGGGAGAGCA
SRR935201.356049264.+                CCAGTGCTCTCCCCGTAGCAGTCGCTGATGCCCTTGGGGAGAGCA
SRR935201.480500077.+      GAGGGAGAAGGCGGGGTGGGCCCCGTAGAAGTCGCTGATGCCCTTGGGGAGAGCA
SRR935201.140642164.- AAGCAGAGGGAGAAGGCGGGGTGGGCCCCGTAGCAGTCGCTGATGCCCTTGGGGAGAGCA
SRR935201.369572431.+ AAGCAGAGGGAGAAGGCGGGGTGGGCCCCGTAGCAGTCGCTGATGCCCTTGGGGAGAGCA
SRR935201.527597468.+ AAGCAGAGGGAGAAGGCGGGGTGGGCCCCGTAGCAGTCGCTGATGCCCTTGGGAAGAGCA
SRR935201.330329116.- GAGCAGAGGGAGAAGGCGGGGTGGGCCCCGTAGCAGTCGCTGATGCCCTTTGGGAGAGCA
SRR935201.117996621.+ GAGCAGAGGGAGAAGGCGGGGTGGGCCCCGTAGCAGTCGCTGATGCCCTTGGGGAGAGCA
SRR935201.298104247.+ GAGCAGAGGGAGAAGGCGGGGTGGGCCCCGTAGCAGTCGCTGATGCCCTTGGGGAGAGCA
SRR935201.376790451.+             AAGGCGGGGTGGGCCCCGTAGCAGTCGCTGATGCCCTTGGGGAGAGCA
SRR935201.393070564.+ GAGCAGAGGGAGAAGGCGGGGTGGGCCCCGTAGCAGTCGCTGATGCCCTTGGGGAGAGCA
SRR935201.431238920.+ GAGCAGAGGGAGAAGGCGGGGTGGGCCCCGTAGCAGTCGCTGATGCCCTTGGGGAGAGCA
SRR935201.486494719.- GAGCAGAGGGAGAAGGCGGGGTGGGCCCCGTAGCAGTCGCTGATGCCCTTGGGGAGAGCA
```

```
SRR935201.523341983.+  AGCAGAGGGAGAAGGCGGGGTGGGCCCCGTAGCAGTCGCTGATGCCCTTGGGGAGAGCA
SRR935201.531574061.+         GGAGAAGGCGGGGTGGGCCCCGTAGCAGTCGCTGATGCCCTTGGGGAGAGCA
SRR935201.282298483.+         GGAGAAGGCGGGGTGGGCCCCGTAGCAGTCGCTGATGCCCTTGGGGAGAGCA
SRR935201.543185073.+         GGAGAAGGCGGGGTGGGCCCCGTAGCAGTCGCTGATGCCCTTGGGGAGAGCA
SRR935201.310242798.-                  GGGGTGGGCCCCGTAGCAGTCGCTGATGCCCTTGGGGAGAGCA
SRR935201.569937326.-                  GGGGTGGGCCCCGTAGCAGTCGCTGATGCCCTTGGGGAGAGCA
SRR935201.574500633.+      GAGGGAGAAGGCGGGGTGGGCCCCGTAGCAGTCGCTGATGCCCTTGGGGAGAGCA
SRR935201.570309401.-                                                            CA
                      ____________________________________________________________
consensus             GAGCAGAGGGAGAAGGCGGGGTGGGCCCCGTAGCAGTCGCTGATGCCCTTGGGGAGAGCA

                          .    :    .    :    .    :    .    :    .    :    .    :
SRR935201.217277601.- CTGG
SRR935201.244710570.- CTGG
SRR935201.356049264.+ CTGGCCTTGTTGCAGGCGTACCCCACGATGTGGTGCACCATCGTCTCCTTGTGAT
SRR935201.480500077.+ CTGGCCTTGTTGCAGGCGTACCCCACGATGTTGTGCACCATCGGT
SRR935201.140642164.- CTGGCCTTGTTGCAGGCGTACCCCACGATGTGGTGCACC
SRR935201.369572431.+ CTGGCCTTGTTGCAGGCGTACCCCACGATGTGGTGC
SRR935201.527597468.+ CTGGCCTTGTTGCAGGCGTACCCCACGATGTGGTGC
SRR935201.330329116.- CTGGCCTTGTTGCAGGCGTACC
SRR935201.117996621.+ CTGGCCTTGTTGCAGGCGTACCCCACGATGTGGTG
SRR935201.298104247.+ CTGGCCTTGTTGCAGGCGT
SRR935201.376790451.+ CTGGCCTTGTTGCAGGCGTACCCCACGATGTGGTGCACCATCGTCTCCTTGT
SRR935201.393070564.+ CTGGCCTTGTTGCAGGCGTACCCC
SRR935201.431238920.+ CTGGCCTTGTTGCAGGCGTACCCCACGATG
SRR935201.486494719.- CTGGCCTTGTTGCAGGCGTACC
SRR935201.523341983.+ CTGGCCTTGTTGCAGGCGTACCCCACGATGTGGTGCACCAT
SRR935201.531574061.+ CTGGCCTTGTTGCAGGCGTACCCCACGATGTGGTGCACCATCGTCTCC
SRR935201.282298483.+ CTGGCCTTGTTGCAGGCGTACCCCACGATGTGGTGCACCATCGTCTCC
SRR935201.543185073.+ CTGGCCTTGTTGCAGGCGTACCCCACGATGTGGTGCACCATCGTCTCC
SRR935201.310242798.- CTGGCCTTGTTGCAGGCGTACCCCACGATGTGGTGCACCATCGTCTCCTTGTGATGG
SRR935201.569937326.- CTGGCCTTGTTGCAGGCGTACCCCACGATGTGGTGCACCATCGTCTCCTTGTGATGG
SRR935201.574500633.+ CTGGCCTTGTTGCAGGCGTACCCCACGATGTGGTGCACCATCGTC
SRR935201.570309401.- CTGGCCTTGTTGCAGGCGTACCCCACGATGTGGTGCACCATCGTCTCCTTGTGATGGACC
SRR935201.322433116.-    TCCTTGTTGCAGGCGTACCCCACGATGTGGTGCACCATCGTCTCCTTGTGATGGACC
SRR935201.406873144.-    GCCTTGTTGCAGGCGTACCCCACGATGTGGTGCACCATCGTCTCCTTGTGATGGACC
SRR935201.331894944.-               GGCGTACCCCACGATGTGGTGCACCATCGTCTCCTTGTGATGGACC
SRR935201.97378867.2-                  GTACCCCACGATGTGGTGCACCATCGTCTCCTTGTGATGGACC
SRR935201.39238612.1-                            ATGTGGTGCACCATCGTCTCCTTGTGATGGACC
SRR935201.527597468.-                             TGTGGTGCACCATCGTCTCCTTGTGATGGACC
SRR935201.140911798.-                                  TGCACCATCGTCTCCTTGTGATGGACC
SRR935201.4427099.1-                                    GCACCATCGTCTCCTTGTGATGGACC
SRR935201.288981003.+                                     ACCATCGTCTCCTTGTGATGGACC
SRR935201.447200043.+                                     ACCATCGTCTCCTTGTGATGGACC
SRR935201.179466762.-                                         TCGTCTCCTTGTGATGGACC
SRR935201.285799402.-                                         TCGTCTCCTTGTGATGGACC
SRR935201.213399218.-                                            TCTCCTTGTGATGGACC
SRR935201.288981003.-                                                  TGTGATGGACC
SRR935201.447200043.-                                                  TGTGATGGACC
SRR935201.10230508.2+                                                          ACC
SRR935201.100893176.+                                                          ACC
                      ____________________________________________________________
consensus             CTGGCCTTGTTGCAGGCGTACCCCACGATGTGGTGCACCATCGTCTCCTTGTGATGGACC

                          .    :    .    :    .    :    .    :    .    :    .    :
SRR935201.570309401.- AACTTGGGCTCAAACTGGGCGGGGGACGCAGGACAGAG
SRR935201.322433116.- AACTTGGGCTCAAACTGGGCGGGGGACGCAGGACAGAGTGTGG
SRR935201.406873144.- AACTTGGGCTCAAACTGGGCGGGGGACGCAGGACAGAGTGTGG
SRR935201.331894944.- AACTTGGGCTCAAACTGGGCGGGGGACGCAGGACAGAGTGTGGTCAAGATCTTC
SRR935201.97378867.2- AACTTGGGCTCAAACTGGGCGGGGGACGCAGGACAGAGTGTGGTCAAGATCTTCTGG
SRR935201.39238612.1- AACTTGGGCTCAAACTGGGCGGGGGACGCAGGACAGAGTGTGGTCAAGATCTTCTGGCC
SRR935201.527597468.- AACTTGGGCTCAAACTGGGCGGGGGACGCAGGACAGAGTGTGGTCAAGATCTTCTGGCC
SRR935201.140911798.- AACTTGGGCTCAAACTGGGCGGGGGACGCAGGACAGAGTGTGGTCAAGATCTTCTGGCC
SRR935201.4427099.1-  AACTTGGGCTCAAACTGGGCGGGGGACGCAGGACAGAGTGTGGTCAAGATCTTCTGGCC
SRR935201.288981003.+ AACTTGGGCTCAAACTGGGCGGGGGACGCAGGACAGAGTGTGGTCAAGATCTTCTGGCC
SRR935201.447200043.+ AACTTGGGCTCAAACTGGGCGGGGGACGCAGGACAGAGTGCGGTCAAGATCTTCTGGCC
SRR935201.179466762.- AACTTGGGCTCAAACTGGGCGGGGGACGCAGGACAGAGTGTGGTCAAGATCTTCTGGCC
SRR935201.285799402.- AACTTGGGCTCAAACTGGGCGGGGGACGCAGGACAGAGTGTGGTCAAGATCTTCTGGCC
SRR935201.213399218.- AACTTGGGCTCAAACTGGGCGGGGGACGCAGGACAGAGTGTGGTCAAGATCTTCTGGCC
SRR935201.288981003.- AACTTGGGCTCAAACTGGGCGGGGGACGCAGGACAGAGTGTGGTCAAGATCTTCTGGCC
SRR935201.447200043.- AACTTGGGCTCAAACTGGGCGGGGGACGCAGGACAGAGTGTGGTCAAGATCTTCTGGCC
SRR935201.10230508.2+ AACTTGGGCTCAAACTGGGCGGGGGACGCAGGACAGAGTGTGGTCAAGATCTTCTGGCC
SRR935201.100893176.+ AACTTGGGCTCAAACTGGGCGGGGGACGCAGGACAGAGTGTGGTCAAGATCTTCTGGCC
SRR935201.208884615.+  ACTTGGGCTCAAACTGGGCGGGGGACGCAGGACAGAGTGTGGCCAAGATCTTCTGGCC
                      ____________________________________________________________
consensus             AACTTGGGCTCAAACTGGGCGGGGGACGCAGGACAGAGTGTGGTCAAGATCTTCTGGCC
```

```
                          .    :    .    :    .    :    .    :    .    :    .    :
SRR935201.39238612.1- ATGTCCT
SRR935201.527597468.- ATGTCCTT
SRR935201.140911798.- ATGTCCTTATGCC
SRR935201.4427099.1-  ATGTCCTTATGCCC
SRR935201.288981003.+ ATGTCCTTATGCCCCC
SRR935201.447200043.+ ATGTCCTTATGCCCCC
SRR935201.179466762.- ATGTCCTTATGCCCCCTATT
SRR935201.285799402.- ATGTCCTTATGCCCCCTATT
SRR935201.213399218.- ATGTCCTTATGCCCCCTATTCCT
SRR935201.288981003.- ATGTCCTTATGCCCCCTATTCCTTTTCCC
SRR935201.447200043.- ATGTCCTTATGCCCCCTATTCCTTTTCCC
SRR935201.10230508.2+ ATGTCCTTATGCCCCCTATTCCTTTTCCCCAAATAAA
SRR935201.100893176.+ ATGTCCTTATGCCCCCTATTCCTTTTCCCCAAATAAA
SRR935201.208884615.+ ATGTCCTTATGCCCCCTATTCCTTTTCCCCAAATAATACTA
                      ____________________________________________________________
consensus             ATGTCCTTATGCCCCCTATTCCTTTTCCCCAAATAAAACTA
```

(6) The exon 6 sequence was predicted by assembling WGS data using CAP3. The exon 6 is on the reverse strand and marked in red.

```
                          .    :    .    :    .    :    .    :    .    :    .    :
SRR935201.153991311.- GCGTGCACCCTGCATCCCCTTCCCGACACCTAGAGGCCTCAGCCAGCCTCACCCGCTGCT
SRR935201.383862398.-         CCTGCATCCCCTTCCCGACACCTAGAGGACTCAGCCAGCCTCACCCGCTGCT
SRR935201.570770564.-         CCTGCATCCCCTTCCCGACACCTAGAGGACTCAGCCAGCCTCACCCGCTGCT
SRR935201.50960315.1+                                          GCCAGCCTCACCCGCTGCT
SRR935201.25870791.1+                                                  CACCCGCTGCT
SRR935201.330329116.+                                                  CACCCGCTGCT
SRR935201.486494719.+                                                  CACCCGCTGCT
SRR935201.266221065.-                                                          GCT
                      ____________________________________________________________
consensus             GCGTGCACCCTGCATCCCCTTCCCGACACCTAGAGGACTCAGCCAGCCTCACCCGCTGCT

                          .    :    .    :    .    :    .    :    .    :    .    :
SRR935201.153991311.- CTCCCGTAAATGCTCTAGGTGCTCACCAGGAAGGCTGTGG
SRR935201.383862398.- CTCCCGTAAATGCTCTAGGTGCTCACCAGGAAGGCTGTGGAGATTGCT
SRR935201.570770564.- CTCCCGTAAATGCTCTAGGTGCTCACCAGGAAGGCTGTGGAGATTGCT
SRR935201.50960315.1+ CTCCCGTAAATGCTCTAGGTGCTCACCAGGAAGGCTGTGGAGATTGCTGTAGTGAATCTC
SRR935201.25870791.1+ CTCCCGTAAATGCTCTAGGTGCTCACCAGGAAGGCTGTGGAGATTGCTGTAGTGAATCTC
SRR935201.330329116.+ CTCCCGTAAATGCTCTAGGTGCTCACCAGGAAGGCTGTGGAGATTGCTGTAGTGAATCTC
SRR935201.486494719.+ CTCCCGTAAATGCTCTAGGTGCTCACCAGGAAGGCTGTGGAGATTGCTGTAGTGAATCTC
SRR935201.266221065.- CTCCCGTAAATGCTCTAGGTGCTCACCAGGAAGGCTGTGGAGATAGCTATAGTGAATCTC
SRR935201.98902771.2-      GTAAATGCTCTAGGTGCTCACCAGGAAGGCTGTGGAGATTGCTGTAGTGAATCTC
SRR935201.25870791.2-               CTAGGTGCTCACCAGGAAGGCTGTGGAGATTGCTGTAGTGAATCTC
SRR935201.428162991.+               CTAGGTGCTCACCAGGAAGGCTGTGGAGATTGCTGTAGTGAATCTC
SRR935201.41135630.2+                   GTGCTCACCAGGAAGGCTGTGGAGATTGCTGTAGTGAATCTC
SRR935201.517333075.+                     GCTCACCAGGAAGGCTGTGGAGATTGCTGTAGTGAATCTC
SRR935201.541971169.+                             GGAAGGCTGTGGAGGTTGCTGTAGTGAATCTC
SRR935201.57733419.1+                             GGAAGGCTGTGGAGATTGCTGTAGTGAATCTC
SRR935201.439329178.+                             GGAAGGCTGTGGAGATTGCTGTAGTGAATCTC
SRR935201.365807312.+                                 GGCTGTGGAGATTGCTGTAGTGAATCTC
SRR935201.66261162.2-                                   CTGTGGAGATTGCTGTAGTGAATCTC
SRR935201.356133435.+                                       GGAGATTGCTGTAGTGAATCTC
SRR935201.421359281.+                                               CTGTAGTGAATCTC
SRR935201.41135630.1-                                                          CTC
SRR935201.41663511.1+                                                          CTC
SRR935201.272971580.+                                                          CTC
SRR935201.386328907.+                                                            C
                      ____________________________________________________________
consensus             CTCCCGTAAATGCTCTAGGTGCTCACCAGGAAGGCTGTGGAGATTGCTGTAGTGAATCTC

                          .    :    .    :    .    :    .    :    .    :    .    :
SRR935201.50960315.1+ CAGTCAGACCCACTGGGTGTC
SRR935201.25870791.1+ CAGTCAGACCCACTGGGTGTCCAAAGGCG
SRR935201.330329116.+ CAGTCAGACCCACTGGGGGTCCAAAGGCG
SRR935201.486494719.+ CAGTCAGACCCACTGGGGGTCCCAAGGCG
SRR935201.266221065.- CAGTCAGACCCACTGGGTGTCCAAAGGCGTCCCAATA
SRR935201.98902771.2- CAGTCAGACCCACTGGGTGTCCAAAGGCGTCCCAATAGAGATACC
SRR935201.25870791.2- CAGTCAGACCCACTGGGTGTCCAAAGGCGTCCCAATAGAGATACCCACATCACC
SRR935201.428162991.+ CAGTCAGACCCACTGGGTGTCCAAAGGCGTCCCAATAGAGATACCCACATCACC
SRR935201.41135630.2+ CAGTCAGACCCACTGGGTGTCCAAAGGCGTCCCAATAGAGATACCCACATCACCTGGA
SRR935201.517333075.+ CAGTCAGACCCACTGGGGGTCCAAAGGCGTCCCAATAGAGATACCCACATCACCTGGAAA
SRR935201.541971169.+ CAGTCAGACCCACTGGGTGTCCAAAGGCGTCCCAATAGAGATACCCACATCACCTGGAAA
SRR935201.57733419.1+ CAGTCAGACCCACTGGGTGTCCAAAGGCGTCCCAATAGAGATACCCACATCACCTGGAAA
SRR935201.439329178.+ CAGTCAGACCCACTGGGTGTCCAAAGGCGTCCCAATAGAGATACCCACATCACCTGGAAA
SRR935201.365807312.+ CAGTCAGACCCACTGGGGGTCCAAAGGCGTCCCAATAGAGATACCCACATCACCTGGAAA
```

```
SRR935201.66261162.2- CAGTCAGACCCACTGGGTGTCCAAAGGCGTCCCAATAGAGATACCCACATCACCTGGAAA
SRR935201.356133435.+ CAGTCAGACCCACTGGGGGTCCAAAGGCGTCCCAATAGAGATACCCACATCACCTGGAAA
SRR935201.421359281.+ CAGTCAGACCCACTGGGGGTCCAAAGGCGTCCCAATAGAGATACCCACATCACCTGGAAA
SRR935201.41135630.1- CAGTCAGACCCTCTGGGTGTCCAAAGGCGTCCCAATAGAGATACCCACATCACCTGGAAA
SRR935201.41663511.1+ CAGTCAGACCCACTGGGGGTCCAAAGGCGTCCCAATAGAGATACCCACATCACCTGGAAA
SRR935201.272971580.+ CAGTCAGACCCACTGGGGGTCCAAAGGCGTCCCAATAGAGATACCCACATCACCTGGAAA
SRR935201.386328907.+ CAGTCAGACCCACTTGGTGTCCAAAGGCGTCGCAATAGAGATACCCACATCACCTGGAAA
SRR935201.266554575.-      AGACCCACTGGGTGTCCAAAGGCGTCCCAATAGAGATACCCACATCACCTGGAAA
SRR935201.467008251.-        ACCCACTGGGTGTCCAAAGGCGTCCCAATAGAGATGCCCACATCACCTGGAAA
SRR935201.50960315.2-                 GTGTCCAAAGGCGTCCCAATAGAGATACCCACATCACCTGGAAA
SRR935201.514879736.+                    TCCAAAGGCGTCCCAATAGAGATACCCACATCACCTGGAAA
SRR935201.564660261.+                    TCCAAAGGCGTCCCAATAGAGATACCCACATCACCTGGAAA
SRR935201.331417648.+                      CAAAGGCGTCCCAATAGAGATACCCACATCACCTGGAAA
SRR935201.436946858.+                      CAAAGGCGTCCCAATAGAGATACCCACATCACCTGGAAA
SRR935201.322433116.+                        AAGGCGTCCCAATAGAGATACACACATCACCTGGAAA
SRR935201.406873144.+                        AAGGCGTCCCAATAGAGATACCCACATCACCTGGAAA
SRR935201.289804414.-                          GGCGTCCCAATAGAGATACCCACATCACCTGGAAA
SRR935201.4427099.2+                                CCCAATAGAGATACCCACATCACCTGGAAA
SRR935201.415965982.+                                 CAATAGAGATACCCACATCACCTGGAAA
SRR935201.310242798.+                                            CCCACATCACCTGGAAA
SRR935201.179466762.+                                                     CCTGGAAA
SRR935201.285799402.+                                                     CCTGGAAA
                      ____________________________________________________________
consensus             CAGTCAGACCCACTGGGTGTCCAAAGGCGTCCCAATAGAGATACCCACATCACCTGGAAA

                          .    :    .    :    .    :    .    :    .    :    .    :
SRR935201.541971169.+ CTGGTAAC
SRR935201.57733419.1+ CTGGTAAC
SRR935201.439329178.+ CTGGTAAC
SRR935201.365807312.+ CTGGTAACTCTA
SRR935201.66261162.2- CTGGTAACTCTATT
SRR935201.356133435.+ CTGGTAACTCTATTGGAT
SRR935201.421359281.+ CTGGTAACTCTATTGGATGGAGGGGG
SRR935201.41135630.1- CTGGTAACTCTATTGGATGGAGGGTGATTTGGCGGCA
SRR935201.41663511.1+ CTGGTAACTCTATTGGATGGAGGGTGATTTGGCGGCC
SRR935201.272971580.+ CTGGTAACTCTATTGGATGGAGGGGGATTTGGCGGCA
SRR935201.386328907.+ CTGGGAACTCTATTGGATGGAGGGTGATTTGGCGGCAAG
SRR935201.266554575.- CTGGTAACTCTATTGGATGGAGGGTGATTTGGCGGCAAGAATGGC
SRR935201.467008251.- CTGGTAACTCTATTGGATGGAGGGTGATTTGGCGGCAAGAATGGCTG
SRR935201.50960315.2- CTGGTAACTCTATTGGATGGAGGGTGATTTGGCGGCAAGAATGGCTGGGAAGTAGG
SRR935201.514879736.+ CTGGTAACTCTATTGGATGGAGGGAGATTTGGCGGCAAGAATGGCTGGGAAGTAGGGGC
SRR935201.564660261.+ CTGGTAACTCTATTGGATGGAGGGTGATTTGGCGGCAAGAATGGCTGGGAAGAACGTGC
SRR935201.331417648.+ CTGGTAACTCTATTGGATGGAGGGTGATTTGGCGGCAAGAATGGCTGGGAAGAAGGTGCA
SRR935201.436946858.+ CTGGTAACTCTATTGGATGGAGGGTGATTTGGCGGCAAGAATGGCTGGGAAGGAGGTGCA
SRR935201.322433116.+ CTGGTAACTCTATTGGATGGAGGGTGATTTGGCGGCAAGAATGGCTGGGAAGTACGCGCA
SRR935201.406873144.+ CTGGTAACTCTATTGGATGGAGGGTGATTTGGCGGCAAGAATGGCTGGGAAGTAGGTGCA
SRR935201.289804414.- CTGGTAACTCTATTGGATGGAGGGTGATTTGGCGGCAAGAATGGCTGGGAACTAGGTGCA
SRR935201.4427099.2+  CTGGTAACTCTATTGGATGGAGGGTGATTTGGCGGCAAGAATGGCTGGGAAGTAGGTGCA
SRR935201.415965982.+ CTGGTAACTCTATTGGATGGAGGGTGATTTGGCGGCAAGAATGGCTGGGAAGTAGGTGAA
SRR935201.310242798.+ CTGGTAACTCTATTGGATGGAGGGTGATTTGGCGGCAAGAATGGCTGGGAAGTAGGTGCA
SRR935201.179466762.+ CTGGTAACTCTATTGGATGGAGGGTGATTTGGCGGCAAGAATGGGTGGGGAGGAGGTGGA
SRR935201.285799402.+ CTGGTAACTCTATTGGATGGAGGGTGATTTGGCGGCAAGAATGGCTGGGAAGTAGGTGCA
                      ____________________________________________________________
consensus             CTGGTAACTCTATTGGATGGAGGGTGATTTGGCGGCAAGAATGGCTGGGAAGTAGGTGCA

                          .    :    .    :    .    :    .    :    .    :    .    :
SRR935201.331417648.+ G
SRR935201.436946858.+ G
SRR935201.322433116.+ AGG
SRR935201.406873144.+ GGG
SRR935201.289804414.- GGGAA
SRR935201.4427099.2+  GGGACCCGGG
SRR935201.415965982.+ GGGACCCGGGCC
SRR935201.310242798.+ GGGACCCGGGCCCGCCCTTGTCT
SRR935201.179466762.+ GGGGCCCGGGCCCCCCCTTTTCTTTTGTTCTT
SRR935201.285799402.+ GGGACCCGGGCCCGCCCTTGGCTTTTGGCCCT
                      ____________________________________________________________
consensus             GGGACCCGGGCCCGCCCTTGTCTTTTGGCCCT
```

(7) The exon 7 sequence was predicted by assembling WGS data using CAP3. The exon 7 is marked in red.

```
                          .    :    .    :    .    :    .    :    .    :    .    :
SRR935201.467008251.- CCCCCACCGCTTTCCCAGGGCCTCTTTCTCCCCACCGTGCGACACAGTCTCCTGCATCCT
SRR935201.205800054.+                              CCCCACCGTGCGACACAGTCTCCTGCATCCT
SRR935201.355749658.+                              CCCCACCGTGCGACACAGTCTCCTGCATCCT
SRR935201.98902771.1-                                                TCTCCTGCATCCT
```

```
SRR935201.205800054.-                                                TCTCCTGCATCCT
SRR935201.355749658.-                                                TCTCCTGCATCCT
SRR935201.537692978.-                                                 CTCCTGCATCCT
SRR935201.450482846.+                                                   CCTGCATCCT

consensus             CCCCCACCGCTTTCCCAGGGCCTCTTTCTCCCCACCGTGCGACACAGTCTCCTGCATCCT

                          .    :    .    :    .    :    .    :    .    :    .    :
SRR935201.467008251.- GTGATTGGTCCTTCCTGCACCAGATGTGTACGACTCCTCG
SRR935201.205800054.+ GTGATTGGTCCTTCCTGCACCAGATGTGTACGACTCCTCGGGGATTTGAGTGTACCTCAC
SRR935201.355749658.+ GTGATTGGTCCTTCCTGCACCAGATGTGTACGACTCCTCGGGGATTTGAGTGTACCTCAC
SRR935201.98902771.1- GTGATTGGTCCTTCCTGCACCAGATGTGTACGACTCCTCGGGGATTTGAGTGTACCTCAC
SRR935201.205800054.- GTGATTGGTCCTTCCTGCACCAGATGTGTACGACTCCTCGGGGATTTGAGTGTACCTCAC
SRR935201.355749658.- GTGATTGGTCCTTCCTGCACCAGATGTGTACGACTCCTCGGGGATTTGAGTGTACCTCAC
SRR935201.537692978.- GTGATTGGTCCTTCCTGCACCAGATGTGTACGACTCCTCGGGGATTTGAGTGTACCTCAC
SRR935201.450482846.+ GTGATTGGTCCTTCCTGCACCAGATGTGTACGACTCCTCGGGGATTTGAGTGTACCTCAC
SRR935201.9864856.1+  GTGATTGGTCCTTCCTGCACCAGATGTGTACGACTCCTCGGGGATTTGAGTGTACCTCAC
SRR935201.173550667.+ GTGATTGGTCCTTCCTGCACCAGATGTGTACGACTCCTCGGGGATTTGAGTGTACCTCAC
SRR935201.426369219.+  TGATTGGTCCTTCCTGCACCAGATGTGTACGACTCCTCGGGGATTTGAGTGTACCTCAC
SRR935201.436709078.+          CCTTCCTGCACCAGATGTGTACGACTCCTCGGGGATTTGAGTGTACCTCAC
SRR935201.102550728.+                 GCACCAGATGTGTACGACTCCTCGGGGATTTGAGTGTACCTCAC
SRR935201.283854653.+                 GCACCAGATGTGTACGACTCCTCGGGGATTTGAGTGTACCTCAC
SRR935201.66261162.1-                     CAGATGTGTACGACTCCTCGGGGATTTGAGTGTACCTCAC
SRR935201.240906730.+                      AGATGTGTACGACTCCTCGGGGATTTGAGTGTACCTCAC
SRR935201.151655622.+                       GATGTGTACGACTCCTCGGGGATTTGAGTGTACCTAAC
SRR935201.266221065.-                                            ATTTGAGTGTACCCCAC
SRR935201.326040602.+                                       CGGGGATTTGAGTGTACCTCAC
SRR935201.157177416.+                                       CGGGGATTTGAGTGTACCTCAC
SRR935201.240906730.-                                GACTCCTCGGGGATTTGAGTGTACCTCAC

consensus             GTGATTGGTCCTTCCTGCACCAGATGTGTACGACTCCTCGGGGATTTGAGTGTACCTCAC

                          .    :    .    :    .    :    .    :    .    :    .    :
SRR935201.205800054.+ GGCGCAGCT
SRR935201.355749658.+ GGCGCAGCT
SRR935201.98902771.1- GGCGCAGCTGCGCAAATATGACATGGG
SRR935201.205800054.- GGCGCAGCTGCGCAAATATGACATGGG
SRR935201.355749658.- GGCGCAGCTGCGCAAATATGACATGGG
SRR935201.537692978.- GGCGCAGCTGCGCAAATATGACATGGGT
SRR935201.450482846.+ GGCGCAGCTGCGCAAATATGACATGGGTGT
SRR935201.9864856.1+  GGCGCAGCTGCGCAAATATGACATGGGTGTCCTGCAGCTG
SRR935201.173550667.+ GGCGCAGCTGCGCAAATATGACATGGGTGTCCTGCAGCTG
SRR935201.426369219.+ GGCGCAGCTGCGCAAATATGACATGGGTGTCCTGCAGCTGG
SRR935201.436709078.+ GGCGCAGCTGCGCAAATATGACATGGGTGTCCTGCAGCTGGGCTTCTTC
SRR935201.102550728.+ GGCGCAGCTGCGCAAATATGACATGGGTGTCCTGCAGCTGGGCTTCTTCACTTTCC
SRR935201.283854653.+ GGCGCAGCTGCGCAAATATGACATGGGTGTCCTGCAGCTGGGCTTCTTCACTTTCC
SRR935201.66261162.1- GGCGCAGCTGCGCAAATATGACATGGGTGTCCTGCAGCTGGGCTTCTTCACTTTCCCCAT
SRR935201.240906730.+ GGCGCAGCTGCGCAAATATGACATGGGTGTCCTGCAGCTGGGCTTCTTCACTTTCCCCAT
SRR935201.151655622.+ GGCGCAGCTGCGCAAATATGACATGGGTGTCCTGCAGCTGGGCTTCTTCACTTTCCCCAT
SRR935201.266221065.- GGCGCAGCAGCGCAAATATGACATGGGTGTCCTGCAGCTGGGCTTCTTCACTTTCCCCAT
SRR935201.326040602.+ GGCGCAGCTGCGCAAATATGACATGGGTGTCCTGCAGCTGGGCTTCTTCACTTTCCCCAT
SRR935201.105175657.-                 TATGACATGGGTGTCCTGCAGCTGGGCTTCTTCACTTTCCCCAT
SRR935201.157177416.+ GGCGCAGCTGCGCAAATATGACATGGGTGTCCTGCAGCTGGGCTTCTTCACTTTCCCCAT
SRR935201.240906730.- GGCGCAGCTGCGCAAATATGACATGGGTGTCCTGCAGCTGGGCTTCTTCACTTTCCCCAT
SRR935201.326807734.+              AAATATGACATGGGTGTCCTGCAGCTGGGCTTCTTCACTTTCCCCAT
SRR935201.379146860.+              AAATATGACATGGGTGTCCTGCAGCTGGGCTTCTTCACTTTCCCCAT
SRR935201.436709078.-          GCGCAAATATGACATGGGTGTCCTGCAGCTGGGCTTCTTCACTTTCCCCAT
SRR935201.473842114.+               AATATGACATGGGTGTCCTGCAGCTGGGCTTCTTCACTTTCCCCAT
SRR935201.157177416.-                                  GCAGCTGGGCTTCTTCACTTTCCCCAT
SRR935201.326040602.-                                  GCAGCTGGGCTTCTTCACTTTCCCCAT
SRR935201.102550728.-                                    AGCTGGGCTTCTTCACTTTCCCCAT
SRR935201.283854653.-                                    AGCTGGGCTTCTTCACTTTCCCCAT
SRR935201.312656503.+                                       TGGGCTTCTTCACTTTCCCCAT
SRR935201.180638705.+                                         GGCTTCTTCACTTTCCCCAT
SRR935201.9864856.2-                                                        CCCCAT
SRR935201.173550667.-                                                       CCCCAT
SRR935201.538478090.+                                                        CCCAT

consensus             GGCGCAGCTGCGCAAATATGACATGGGTGTCCTGCAGCTGGGCTTCTTCACTTTCCCCAT

                          .    :    .    :    .    :    .    :    .    :    .    :
SRR935201.240906730.+ C
SRR935201.151655622.+ CC
SRR935201.266221065.- CCACTTCATACCCCCGGGCGCCG
SRR935201.326040602.+ CCCCTTCATACCCCCGGG
SRR935201.105175657.- CCACTTCATACCCCCGGGCGCCGAGTCCTTCGCGTCCTACGGGCTGTGTAAGAAGG
SRR935201.157177416.+ CCACTTCATACCCCCGGG
```

```
SRR935201.240906730.- CCACTTCATAC
SRR935201.326807734.+ CCACTTCATACCCCCGGGCGCCGAGTCCTTCGCGTCCTACGGGCTGTGTAAGA
SRR935201.379146860.+ CCACTTCATACCCCCGGGCGCCGAGTCCTTCGCGTCCTACGGGCTGTGTAAGA
SRR935201.436709078.- CCACTTCATACCCCCGGGCGCCGAGTCCTTCGCGTCCTACGGGCTGTGT
SRR935201.473842114.+ CCACTTCATACCCCCGGGCGCCGAGTCCTTCGCGTCCTACGGGCTGTGTAAGAA
SRR935201.157177416.- CCACTTCATACCCCCGGGCGCCGAGTCCTTCGCGTCCTACGGGCTGTGTAAGAAGGAGCA
SRR935201.326040602.- CCACTTCATCCCCCCGGGCGCCGAGTCCTTCGCGTCCTACGGGCTGTGTAAGAAGGAGCA
SRR935201.102550728.- CCACTTCATACCCCCGGGCGCCGAGTCCTTCGCGTCCTACGGGCTGTGTAAGAAGGAGCA
SRR935201.283854653.- CCACTTCATACCCCCGGGCGCCGAGTCCTTCGCGTCCTACGGGCTGTGTAAGAAGGAGCA
SRR935201.312656503.+ CCACTTCATACCCCCGGGCGCCGAGTCCTTCGCGTCCTACGGGCTGTGTAAGAAGGAGCA
SRR935201.180638705.+ CCACTTCATACCCCCGGGCGCCGAGTCCTTCGCGTCCTACGGGCTGTGTAAGAAGGAGCA
SRR935201.9864856.2-  CCACTTCATACCCCCGGGCGCCGAGTCCTTCGCGTCCTACGGGCTGTGTAAGAAGGAGCA
SRR935201.173550667.- CCACTTCATACCCCCGGGCGCCGAGTCCTTCGCGTCCTACGGGCTGTGTAAGAAGGAGCA
SRR935201.538478090.+ CCACTTCATACCCCCGGGCGCCGAGTCCTTCGCGTCCTACGGGCTGTGTAAGAAGGAGCA
SRR935201.201822459.+       CATACCCCCGGGCGCCGAGTCCTTCGCGTCCTACGGGCTGTGTAAGAAGGAGCA
SRR935201.47097248.1-         TACCCCCGGGCGCCGAGTCCTTCGCGTCCTACGGGCTGTGTAAGAAGGAGCA
SRR935201.96702305.1-         TACCCCCGGGCGCCGAGTCCTTCGCGTCCTACGGGCTGTGTAAGAAGGAGCA
SRR935201.570770564.-         TACCCCCGGGCGCCGAGTCCTTCGCGTCCTACGGGCTGTGTAAGAAGGAGCA
SRR935201.162168186.-                   CGCCGAGTCCTTCGCGTCCTACGGGCTGTGTAAGAAGGAGCA
SRR935201.241673916.-                   CGCCGAGTCCTTCGCGTCCTACGGGCTGTGTAAGAAGGAGCA
SRR935201.321557695.+                                       ACGGGCTGTGTAAGAAGGAGCA
SRR935201.94445969.2+                                               TGTAAGAAGGAGCA
SRR935201.98852448.1+                                               TGTAAGAAGGAGCA
                      ____________________________________________________________
consensus             CCACTTCATACCCCCGGGCGCCGAGTCCTTCGCGTCCTACGGGCTGTGTAAGAAGGAGCA

                          .    :    .    :    .    :    .    :    .    :    .    :
SRR935201.157177416.- GTTTGAAGAGGTG
SRR935201.326040602.- GTTTGAAGAGGTG
SRR935201.102550728.- GTTTGAAGAGGTGAA
SRR935201.283854653.- GTTTGAAGAGGTGAA
SRR935201.312656503.+ GTTTGAAGAGGTGAAGCC
SRR935201.180638705.+ GTTTGAAGAGGTGAAGCCGG
SRR935201.9864856.2-  GTTTGAAGAGGTGAAGCCGGGAGGCACCCCTCCT
SRR935201.173550667.- GTTTGAAGAGGTGAAGCCGGGAGGCACCCCTCCT
SRR935201.538478090.+ GTTTGAAGAGGTGAAGCCGGGAGGCACCCCTCCTC
SRR935201.201822459.+ GTTTGAAGAGGTGAAGCCGGGAGGCACCCCTCCTCCCCTGATCTTC
SRR935201.47097248.1- GTTTGAAGAGGTGAAGCCGGGAGGCACCCCTCCTCCCCTGATCTTCCC
SRR935201.96702305.1- GTTTGAAGAGGTGAAGCCGGGAGGCACCCCTCCTCCCCTGATCTTCCC
SRR935201.570770564.- GTTTGAAGAGGTGAAGCCGGGAGGCACCCCTCCTCCCCTGATCTTCCC
SRR935201.162168186.- GTTTGAAGAGGTGAAGCCGGGAGGCACCCCTCCTCCCCTGATCTTCCCGGTTCCCCAT
SRR935201.241673916.- GTTTGAAGAGGTGAAGCCGGGAGGCACCCCTCCTCCCCTGATCTTCCCGGTTCCCCAT
SRR935201.321557695.+ GTTTGAAGAGGTGAAGCCGGGAGGCACCCCTCCTCCCCTGATCTTCCCGGTTCCCCATCG
SRR935201.94445969.2+ GTTTGAAGAGGTGAAGCCGGGAGGCACCCCTCCTCCCCTGATCTTCCCGGTTCCCCATCG
SRR935201.98852448.1+ GTTTGAAGAGGTGAAGCCGGGAGGCACCCCTCCTCCCCTGATCTTCCCGGTTCCCCATCG
                      ____________________________________________________________
consensus             GTTTGAAGAGGTGAAGCCGGGAGGCACCCCTCCTCCCCTGATCTTCCCGGTTCCCCATCG

                          .    :    .    :    .    :    .    :    .    :    .    :
SRR935201.321557695.+ CAAATCTGGTGTGACCCA
SRR935201.94445969.2+ CAAATCTGGTGTGACCCACCAGGAAC
SRR935201.98852448.1+ CAAATCTGGTGTGACCCACCAGGCAC
                      ____________________________________________________________
consensus             CAAATCTGGTGTGACCCACCAGGAAC
```

(8) The exon 8 sequence was predicted by assembling WGS data using CAP3. The exon 8 is marked in red.

```
                          .    :    .    :    .    :    .    :    .    :    .    :
SRR935201.98852448.2- CAGATATGAAAGGAGGAAACCGAGGAGCGGGGCCAGACGAGAGTTCCTAACAAGCCAGGT
SRR935201.283871238.+ CAGATATGAAAGGAGGAAACCGAGGAGCGGGGCCAGACGAGAGTTCCTAACAAGCCAGGT
SRR935201.254814860.+ CAGATATGAAAGGAGGAAACCGAGGAGCGGGGCCAGACGAGAGTTCCTAACAAGCCAGGT
SRR935201.388764534.+ CAGATATGAAAGGAGGAAACCGAGGAGCGGGGCCAGACGAGAGTTCCTAACAAGCCAGGT
SRR935201.455775437.-        GAAAGGAGAAAACCGAGGAGCGGGGCCAGACGAGAGTTCCTAACAAGCCAGGT
SRR935201.249300765.+         AAAGGAGGAAACCGAGGAGCGGGGCCAGACGAGAGTTCCTAACAAGCCAGGT
SRR935201.2622049.2-            AGGAGGAAACCGAGGAGCGGGGCCAGACGAGAGTTCCTAACAAGCCAGGT
SRR935201.257273696.-           AGGAGGAACCCGAGGAGCGGGGCCAGACGAGAGTTCCTAACAAGCCAGGT
SRR935201.479593882.+           AGGAGGAAACCGAGGAGCGGGGCCAGACGAGAGTTCCTAACAAGCCAGGT
SRR935201.312656503.-                   ACCGAGGGGCGGGGCCAGACGAGAGTTCCTAACAAGCCAGGT
SRR935201.98675356.1-                       GGGAGGGGGGCCAGACGAGAGTCCCTAACAAGCCGGGT
SRR935201.396576525.-                       AGGAGCGGGGCCAGACGAGAGTTCCTAACAAGCCAGGT
SRR935201.149743829.+                           GCGGGGCCAGACGAGAGTTCCTAACAAGCCAGGT
SRR935201.201822459.-                           GCGGGGCCAGACGAGAGTTCCTAACAAGCCAGGT
SRR935201.343931083.-                                      CGAGAGTTCCTAACAAGCCAGGT
SRR935201.506844625.-                                      CGAGAGTCCCTAACAAGCCAGGT
SRR935201.556926357.-                                      CGAGAGTTCCTAACAAGCCAGGT
SRR935201.479593882.-                                          ATTTCATAACAAGCCAGGT
```

```
SRR935201.253621609.-                                                   CAAGCCAGGT
SRR935201.246230290.+                                                         AGGT
                      ____________________________________________________________
consensus             CAGATATGAAAGGAGGAAACCGAGGAGCGGGGCCAGACGAGAGTTCCTAACAAGCCAGGT

                          .    :    .    :    .    :    .    :    .    :    .    :
SRR935201.98852448.2- GGTCTTCTCCCTGCCACAGATGAACGGGGCCCCGGTGCCT
SRR935201.283871238.+ GGTCTTCTCCCTGCCACAGATGAACGGGGCCCCGGTGCCT
SRR935201.254814860.+ GGTCTTCTCCCTGCCACAGATGAACGGGGCCCCGGTGCCT
SRR935201.388764534.+ GGTCTTCTCCCTGCCACAGATGAACGGGGCCCCGGTGCCT
SRR935201.455775437.- GGTCTTCTCCCTGCCACAGATGAACGGGGCCCCGGTGCCTGACATCC
SRR935201.249300765.+ GGTCTTCTCCCTGCCACAGATGAACGGGGCCCCGGTGCCTGACATCCA
SRR935201.2622049.2-  GGTCTTCTCCCTGCCACAGATGAACGGGGCCCCGGTGCCTGACATCCAGG
SRR935201.257273696.- GGTCTTCTCCCTGCCACAGATGAACGGGGCCCCGGTGCCTGACATCCAGG
SRR935201.479593882.+ GGTCTTCTCCCTGCCACAGATGAACGGGGCCCCGGTGCCTGACATCCAGG
SRR935201.312656503.- GGTCTTCTCCCTGCCACAGATGAACGGGGCCCCGGTGCCTGACATCCAGGTCTGCGGC
SRR935201.98675356.1- GGTCTCCTCCCTGCCACAGATGAACGGGGCCCCGGTGCCTGACATCCAGGTCTGCGGCTA
SRR935201.396576525.- GGTCTTCTCCCTGCCGCAGATGAACGGGGCCCCGGTGCCTGACATCCAGGTCTGCGGCTA
SRR935201.149743829.+ GGTCTTCTCCCTGCCACAGATGAACGGGGCCCCGGTGCCTGACATCCAGGTCTGCGGCTA
SRR935201.201822459.- GGTCTTCTCCCTGCCACAGATGAACGGGGCCCCGGTGCCTGACATCCAGGTCTGCGGCTA
SRR935201.343931083.- GGTCTTCTCCCTGCCACAGATGAACGGGGCCCCGGTGCCTGACATCCAGGTCTGCGGCTA
SRR935201.506844625.- GGTCTTCTCCCTGCCACAGATGAACGGGGCCCCGGTGCCTGACATCCAGGTCTGCGGCTA
SRR935201.556926357.- GGTCTTCTCCCTGCCACAGATGAACGGGGCCCCGGTGCCTGACATCCAGGTCTGCGGCTA
SRR935201.479593882.- GGTCTCCCCCCTGCCACAGATGAACGGGGCCCCGGTGCCTGACATCCAGGTCTGCGGCTA
SRR935201.253621609.- GGTCTTCTCCCTGCCACAGATGAACGGGGCTCCGGTGCCTGACATCCAGGTCTGCGGCTA
SRR935201.246230290.+ GGTCTTCTCCCTGCCACAGATGAACGGGGCCCCGGTGCCTGACATCCAGGTCTGCGGCTA
SRR935201.283871238.-     TTCGCCCTGCCACAGATGAACGGGGCCCCGGTGCCTGACATCCAGGTCTGCGGCTA
SRR935201.388764534.-     TTCTCCCTGCCACAGATGAACGGGGCCCCGGTGCCTGACATCCAGGTCTGCGGCTA
SRR935201.94445969.1-                       AACGGGGCCCCGGTGCCTGACATCCAGGTCTGCGGCTA
SRR935201.299358183.-                                 CGGTGCCTGACATCCAGGTCTGCGGCTA
SRR935201.332205628.+                                 CGGTGCCTGACATCCAGGTCTGCGGCTA
SRR935201.483614660.+                                 CGGTGCCTGACATCCAGGTCTGCGGCTA
SRR935201.92990560.1+                                      CCTGACATCCAGGTCTGCGGCTA
SRR935201.365174113.+                                                 GGTCTGCGGCTA
SRR935201.513734907.-                                                   TCTGCGGCTA
SRR935201.397211580.+                                                            A
                      ____________________________________________________________
consensus             GGTCTTCTCCCTGCCACAGATGAACGGGGCCCCGGTGCCTGACATCCAGGTCTGCGGCTA

                          .    :    .    :    .    :    .    :    .    :    .    :
SRR935201.98675356.1- CC
SRR935201.396576525.- CC
SRR935201.149743829.+ CCTGCT
SRR935201.201822459.- CCTGCT
SRR935201.343931083.- CCTGCTCCACACCCACT
SRR935201.506844625.- CCTGCTCCACACCCACT
SRR935201.556926357.- CCTGCTCCACACCCACT
SRR935201.479593882.- CCTGCTCCACACCCACTTGGC
SRR935201.253621609.- CCTGCTCCACACCCACTTGGCTGGCCTCAC
SRR935201.246230290.+ CCTGCTCCACACCCACTTGGCTGGCCTCACTCTGCA
SRR935201.283871238.- CCTGCTCCACACCCACTTGGCTGGCCTCACTCTGCAGGCCGTGC
SRR935201.388764534.- CCTGCTCCACACCCACTTGGCTGGCCTCACTCTGCAGGCCGTGC
SRR935201.94445969.1- CCTGCTCCACACCCGCTTGGCTGGCCTCACTCTGCAGGCCGTGCAATACAGGTAAGGAAG
SRR935201.299358183.- CCTGCTCCACACCCACTTGGCTGGCCTCACTCTGCAGGCCGTGCAATACAGGTAAGGCAG
SRR935201.332205628.+ CCTGCTCCACACCCACTTGGCTGGCCTCACTCTGCAGGCCGTGCAATACAGGTAAGGAAG
SRR935201.483614660.+ CCTGCTCCACACCCACTTGGCTGGCCTCACTCTGCAGGCCGTGCAATACAGGTAAGGAAG
SRR935201.92990560.1+ CCTGCTCCACACCCACTTGGCTGGCCTCACTCTGCAGGCCGTGCAATACAGGTAAGGCAG
SRR935201.365174113.+ CCTGCTCCACACCCACTTGGCTGGCCTCACTCTGCAGGCCGTGCAATACAGGTAAGGCAG
SRR935201.513734907.- CCTGCTCCACACCCACTTGGCTGGCCTCACTCTGCAGGCCGTGCAATACAGGTAAGGCAG
SRR935201.397211580.+ CCTGCTCCACACCCACTTGGCTGGCCTCACTCTGCAGGCCGTGCAATACAGGTAAGGCAG
SRR935201.496714316.+ CCTGCTCCACACCCACTTGGCTGGCCTCACTCTGCAGGCCGTGCAATACAGGTAAGGCAG
SRR935201.479744008.-     CTCCACACCCACTTGGCTGGCCTCACTCTGCAGGCCGTGCAATACAGGTAAGGAAG
SRR935201.26744852.1+       CCACACCCACTTGGCTGGCCTCACTCTGCAGGCCGTGCAATACAGGTAAGGAAG
SRR935201.58333969.1+       CCACACCCACTTGGCTGGCCTCACTCTGCAGGCCGTGCAATACAGGTAAGGAAG
SRR935201.246833021.+       CCACACCCACTTGGCTGGCCTCACTCTGCAGGCCGTGCAATACAGGTAAGGAAG
SRR935201.78571776.1+               ACTTGGCTGGCCTCACTCTGCAGGCCGTGCAATACAGGTAAGGCAG
SRR935201.312267115.+               ACTTGGCTGGCCTCACTCTGCAGGCCGTGCAATACAGGTAAGGCAG
SRR935201.451982999.+               ACTTGGCTGGCCTCACTCTGCAGGCCGTGCAATACAGGTAAGGCAG
SRR935201.534145484.+                            CACTCTGCAGGCCGTGCAATACAGGTAAGGCAG
SRR935201.226222709.-                                        CGTGCAATACAGGTAAGGCAG
                      ____________________________________________________________
consensus             CCTGCTCCACACCCACTTGGCTGGCCTCACTCTGCAGGCCGTGCAATACAGGTAAGGCAG

                          .    :    .    :    .    :    .    :    .    :    .    :
SRR935201.94445969.1- AC
SRR935201.299358183.- ACACGGATTGGA
SRR935201.332205628.+ ACACGGATTGGA
```

```
SRR935201.483614660.+ ACACGGATTGGA
SRR935201.92990560.1+ ACACGGATTGGAAGCTC
SRR935201.365174113.+ ACACGGATTGGAAGCTCTCCCTTCTGTA
SRR935201.513734907.- ACATGGATTGGAAGCTCTCCCTTCTGCATT
SRR935201.397211580.+ ACACGGATTGGAAGCTCTCCCTTCTGTATTTGGAAATAA
SRR935201.496714316.+ ACACGGATTGGAAGCTCTCCCTTCTGTATTTGGAAATAAA
SRR935201.479744008.- ACACGGATTGGAAGCTCTCCCTTCTGTATTTGGAAATAAATGTT
SRR935201.26744852.1+ ACACGGATTGGAAGCTCTCCCTTCTGTATTTGGAAATAAATGTTAG
SRR935201.58333969.1+ ACACGGATTGGAAGCTCTCCCTTCTGTATTTGGAAATAAATGTTAG
SRR935201.246833021.+ ACACGGATTGGAAGCTCTCCCTTCTGTATTTGGAAATAAATGTTAG
SRR935201.78571776.1+ ACACGGATTGGAAGCTCTCCCTTCTGTATTTGGAAATAAATGTTAGCAATAATT
SRR935201.312267115.+ ACACGGATTGGAAGCTCTCCCTTCTGTATTTGGAAATAAATGTTAGCAATAATT
SRR935201.451982999.+ ACACGGATTGGAAGCTCTCCCTTCTGTATTTGGAAATAAATGTTAGCAATAATT
SRR935201.534145484.+ ACACGGATTGGAAGCTCTCCCTTCTGTATTTGGAAATAAATGTTAGCAATAATTATTTAT
SRR935201.226222709.- ACACGGATTGGAAGCTCTCCCTTCTGTATTTGGAAATAAATGTTAGCAATAATTATTTAT
                      ____________________________________________________________
consensus             ACACGGATTGGAAGCTCTCCCTTCTGTATTTGGAAATAAATGTTAGCAATAATTATTTAT

                          .    :    .    :    .    :    .    :    .    :    .    :
SRR935201.534145484.+ TCTGCGG
SRR935201.226222709.- TCTGCGGCATGACGTTATG
                      ____________________________________________________________
consensus             TCTGCGGCATGACGTTATG
```

(9) The exon 9 sequence was predicted by assembling WGS data using CAP3. The exon 9 is marked in red.

```
                          .    :    .    :    .    :    .    :    .    :    .    :
SRR935201.12396866.2+ ACTGCACACGCTGCTTCTTTGTTGATAGACCCCCTCCCTGCTTCATCCTCTCCTTATTCC
SRR935201.104415620.-             GCTTCTTTGTTGATAGACCCCCTCCCTGCTTCATCCTCTCCTTATTCC
SRR935201.414390461.-              CTTCTTTGTTGATAGACCCCCTCCCTGCTTCATCCTCTCCTTATTCC
SRR935201.326730574.-                TCTTTGTTGATAGACCCCCTCCCTGCTTCATCCTCTCCTTATTCC
SRR935201.300137540.-                        GATAGACCCCCTCCCTGCTTCATCCTCTCCTTATTCC
SRR935201.397211580.-                             ACCCCCTCCCTGCTTCATCCTCTCCTTATTCC
SRR935201.474377955.-                             AGCCCCTCCCTGCTTCATCCTCTCCTTATTCC
SRR935201.248455697.-                               GATCTCCCTGCTTCATCCTCTCCTTATTCC
SRR935201.206450931.+                                    CTCTGCTTCATCCTCTCCTTATTCC
SRR935201.248455697.+                                    CCCTGCTTCATCCTCTCCTTATTCC
SRR935201.206450931.-                                             ATCCTCTCCTTATTCC
SRR935201.332205628.-                                              TCCTCTCCTTATTCC
SRR935201.483614660.-                                              TCCTCTCCTTATTCC
SRR935201.534145484.-                                              TCCTCTCCTTATTCC
                      ____________________________________________________________
consensus             ACTGCACACGCTGCTTCTTTGTTGATAGACCCCCTCCCTGCTTCATCCTCTCCTTATTCC

                          .    :    .    :    .    :    .    :    .    :    .    :
SRR935201.12396866.2+ TGTCCCCTCCCCTTGTTCCCGTCCAGAAATGGAACACAAC
SRR935201.104415620.- TGTCCCCTCCCCTTGTTCCCGTCCAGAAATGGAACACAACTCCGAACAATCT
SRR935201.414390461.- TGTCCCCTCCCCTTGTTCCCGTCCAGAAATGGAACACAACTCCGAACAATCTG
SRR935201.326730574.- TGTCCCCTCCCCTTGTTCCCGTCCAGAAATGGAACACAACTCCGAACAATCTGTA
SRR935201.300137540.- TGTCCCCTCCCCTTGTTCCCGTCCAGAAATGGAACACAACTCCGAACAATCTGTAAAGAT
SRR935201.397211580.- TGTCCCCTCCCCTTGTTCCCGTCCAGAAATGGAACACAACTCCGAACAATCTGTAAAGAT
SRR935201.474377955.- TGTCCCCTCCCCTTGTTCCCGTCCAGAAATGGAACACAACTCCGAACAATCTGTAAAGAT
SRR935201.248455697.- TGTCCCCTCCCCTTGTTCCCGTCCAGAAATGGAACACAACTCCGAACAATCTGTAAAGAT
SRR935201.206450931.+ TGTCCCCTCCCCTTGTTCCCGTCCAGAAATGGAACACAACTCCGAACAATCTGTAAAGAT
SRR935201.248455697.+ TGTCCCCTCCCCTTGTTCTCGTCCAGAAATGGAACACAACTCCGAACAATCTGTAAATAT
SRR935201.206450931.- TGTCCCCTCCCCTTGTTCCCGTCCAGAAATGGAACACAACTCCGAACAATCTGTAAAGAT
SRR935201.332205628.- TGTCCCCTCCCCTTGTTCCCGTCCAGAAATGGAACACAACTCCGAACAATCTGTAAAGAT
SRR935201.483614660.- TGTCCCCTCCCCTTGTTCCCGTCCAGAAATGGAACACAACTCCGAACAATCTGTAAAGAT
SRR935201.534145484.- TGTCCCCTCCCCTTGTTCCCGTCCAGAAATGGAACACAACTCCGAACAATCTGTAAAGAT
SRR935201.25135829.1-  GTCCCCTCCCCTTGTTCCCGTCCAGAAATGGAACACAACTCCGAACAATCTGTAAAGAT
SRR935201.92990560.2-     CCCTCCCCTTGTTCCCGTCCAGAAATGGAACACAACTCCGAACAATCTGTAAAGAT
SRR935201.115259408.+                            AATGGAACACAACTCCGAACAATCTGTAAAGAT
SRR935201.291253002.+                            AATGGAACACAACTCCGAACAATCTGTAAAGAT
SRR935201.328346332.+                            AATGGAACACAACTCCGAACAATCTGTAAAGAT
SRR935201.44826791.2-                                GAACACAACTCCGAACAATCTGTAAAGAT
SRR935201.322757516.-                                AAACACAACTCCGAACAATCTGTAAAGAT
SRR935201.38440900.1+                                                   CTGTAAAGAT
SRR935201.108357972.+                                                    TGTAAAGAT
SRR935201.405958695.+                                                    TGTAAAGAT
SRR935201.300646345.+                                                        AAGAT
SRR935201.349958237.+                                                        AAGAT
SRR935201.481767328.+                                                          GAT
                      ____________________________________________________________
consensus             TGTCCCCTCCCCTTGTTCCCGTCCAGAAATGGAACACAACTCCGAACAATCTGTAAAGAT

                          .    :    .    :    .    :    .    :    .    :    .    :
```

```
SRR935201.300137540.- GAT
SRR935201.397211580.- GATTCCTA
SRR935201.474377955.- GATTCCTA
SRR935201.248455697.- GATTCCTACG
SRR935201.206450931.+ GATTCCTACGACTTC
SRR935201.248455697.+ GATTCTTACGAGATC
SRR935201.206450931.- GATTCCTACGACTTCAATCTGCAG
SRR935201.332205628.- GATTCCTACGACTTCAATCTGCAGG
SRR935201.483614660.- GATTCCTACGACTTCAATCTGCAGG
SRR935201.534145484.- GATTCCTACGACTTCAATCTGCAGG
SRR935201.25135829.1- GATTCCTACGACTTCAATCTGCAGGAGACTCGAGATTTACC
SRR935201.92990560.2- GATTCCTACGACTTCAATCTGCAGGAGACTCGAGATTTACCTTC
SRR935201.115259408.+ GATTCCTACGACTTCAATCTGCAGGAGACTCGAGATTTACCTTCTCGCGTGGCCATCAAG
SRR935201.291253002.+ GATTCCTACGACTTCAATCTGCAGGAGACTCGAGATTTACCTTCTCGAGTGGCCATCAAG
SRR935201.328346332.+ GATTCCTACGACTTCAATCTGCAGGAGACTCGAGATTTACCTTCTCGAGTGGCAATCAAG
SRR935201.44826791.2- GATTCCTACGACTTCAATCTGCAGGAGACTCGAGATTTACCTTCTCGAGTGGCCATCAAG
SRR935201.322757516.- GATTCCTACGACTTCAATCTGCAGGAGTCTCGAGATTTACCTTCTCGAGTGGCCATCAAG
SRR935201.38440900.1+ GATTCCTACGACTTCAATCTGCAGGAGACTCGAGATTTACCTTCTCGAGTGGCCATCAAG
SRR935201.108357972.+ GATTCCTACGACTTCAATCTGCAGGAGACTCGAGATTTACCTTCTCGAGTGGCCATCAAG
SRR935201.405958695.+ GATTCCTACGACTTCAATCTGCAGGAGACTCGAGATTTACCTTCTCGAGTGGCCATCAAG
SRR935201.300646345.+ GATTCCTACGACTTCAATCTGCAGGAGACTCGAGATTTACCTTCTCGAGTGGCCATCAAG
SRR935201.349958237.+ GATTCCTACGACTTCAATCTGCAGGAGACTCGAGATTTACCTTCTCGAGTGGCCATCAAG
SRR935201.481767328.+ GATTCCTACGACTTCAATCTGCAGGAGACTCGAGATTTACCTTCTCGAGTGGCCATCAAG
SRR935201.228004051.+ GATTCCTACGACTTCAATCTGCAGGAGACTCGAGATTTACCTTCTCGAGTGGCCATCAAG
SRR935201.182423332.+     CCTACGACTTCAATCTGCAGGAGACTCGAGATTTACCTTCTCGAGTGGCCATCAAG
SRR935201.4884455.1+                      GCAGGAGACTCGAGATTTACCTTCTCGAGTGGCCATCAAG
SRR935201.11008539.1+                     GCAGGAGACTCGAGATTTACCTTCTCGAGTGGCCATCAAG
SRR935201.286256253.+                     GCAGGAGACTCGAGATTTACCTTCTCGAGTGGCCATCAAG
SRR935201.89653627.1+                     GCAGGAGACTCGAGATTTACCTTCTCGAGTGGCCATCAAG
SRR935201.557940360.+                     GCAGGAGACTCGAGATTTACCTTCTCGAGTGGCCATCAAG
SRR935201.187848779.+                      CAGGAGACTCGAGATTTACCTTCTCGAGTGGCCATCAAG
SRR935201.293466426.+                      CAGGAGACTCGAGATTTACCTTCTCGAGTGGCCATCAAG
SRR935201.556099272.+                      CAGGAGACTCGAGATTTACCTTCTCGAGTGGCCATCAAG
SRR935201.207099838.+                        GGAGACTCGAGATTTACCTTCTCGAGTGGCCATCAAG
SRR935201.380622336.+                        GGAGACTCGAGATTTACCTTCTCGAGTAGCCATCAAG
SRR935201.423017175.+                        GGAGACTCGAGATTTACCTTCTCGAGTGGCCATCAAG
SRR935201.349868662.+                        GGAGACTCGAGATTTACCTTCTCGAGTGGCCATCAAG
SRR935201.506148578.+                        GGAGACTCGAGATTTACCTTCTCGAGTGGCCATCAAG
SRR935201.22968908.2+                          AGACTCGAGATTTACCTTCTCGAGTGGCCATCAAG
SRR935201.551865621.+                               CGAGATTTACCTTCTCGAGTGGCCATCAAG
SRR935201.339615116.+                                       ACCTTCTCGAGTGGCCATCAAG
SRR935201.108377682.-                                             TCGAGTGGCCATCAAG
SRR935201.409608547.-                                             TCGAGTGGCCATCAAG
SRR935201.313566938.+                                               GAGTGGCCATCAAG

consensus             GATTCCTACGACTTCAATCTGCAGGAGACTCGAGATTTACCTTCTCGAGTGGCCATCAAG

                          .    :    .    :    .    :    .    :    .    :    .    :
SRR935201.115259408.+ CTGGTGG
SRR935201.291253002.+ CTGGTGG
SRR935201.328346332.+ ATGGTGG
SRR935201.44826791.2- CTGGTGGGAGC
SRR935201.322757516.- CTGGTGGGAGC
SRR935201.38440900.1+ CTGGTGGGAGCCTGGGGGGAGGCACGGCTG
SRR935201.108357972.+ CTGGTGGGAGCCTGGGGGGAGGCACGGGTGG
SRR935201.405958695.+ CTGGTGGGAGCCTGGGGGGAGGCACGGCTGG
SRR935201.300646345.+ CTGGTGGGAGCCTGGGGGGAGGCACGGCTGGCGGC
SRR935201.349958237.+ CTGGTGGGAGCCTGGGGGGAGGCACGGCTGGCGTC
SRR935201.481767328.+ CTGGTGGGAGCCTGGGGGGAGGCACGGCTGGCGTCGG
SRR935201.228004051.+ CTGGTGGGAGCCTGGGGGGAGGCACGGCTGGGGGCGGGGG
SRR935201.182423332.+ CTGGTGGGAGCCTGGGGGGAGGCACGGCTGGCGTCGGGGGAAGG
SRR935201.4884455.1+  CTGGTGGGAGCCTGGGGGGAGGCACGGCTGGCGGCGGGGGAAGGGAGGGGCAGCCCACAC
SRR935201.11008539.1+ CTGGTGGGAGCCTGGGGGGAGGCACGGCTGGCGTCGGGGGAAGGCAGGGGCAGCACTCAC
SRR935201.286256253.+ CTGGTGGGAGCCTGGGGGGAGGCACGGCTGGCGTCGGGGGAAGGCAGGGGCAGGACTCAC
SRR935201.89653627.1+ CTGGTGGGAGCCTGGGGGGAGGCACGGCTGGCGTCGGGGGAAGGCAGGGTCAGCACTCAC
SRR935201.557940360.+ CTGGTGGGAGCCTGGGGGGAGGCACGGCTGGCGTCGGGGGAAGGCAGGGGCAGCACTCAC
SRR935201.187848779.+ CTGGTGGGAGCCTGGGGGGAGGCACGGCTGGCGTCGGGGGAAGGCAGGGTCAGCACTCAC
SRR935201.293466426.+ CTGGTGGGAGCCTGGGGGGAGGCACGGCTGGCGTCGGGGGAAGGCAGGGGCAGCACTCAC
SRR935201.556099272.+ CTGGTGGGAGCCTGGGGGGAGGCACGGCTGGCGTCGGGGGAAGGCAGGGTCAGCACTCAC
SRR935201.207099838.+ CTGGTGGGAGCCTGGGGGGAGGCACGGCTGGCGTCGGGGGAAGGCAGGGTCAGCACTCAC
SRR935201.380622336.+ CTGGTTGGAGCCTGGGGGAAGGCACGGCTGGCGTCGGGGGCAGGCAGGGTCAGGACTCAC
SRR935201.423017175.+ CTGGTGGGAGCCTGGGGGGAGGCACGGCTGGCGTCGGGGGAAGGGAGGGTCAGCACTCAC
SRR935201.349868662.+ CTGGTGGGAGCCTGGGGGGAGGCACGGCTGGCGTCGGGGGAAGGCAGGGTCAGCACTCAC
SRR935201.506148578.+ CTGGTGGGAGCCTGGGGGGAGGCACGGCTGGCGTCGGGGGAAGGCAGGGTCAGCACTCAC
SRR935201.22968908.2+ CTGGTGGGAGCCTGGGGGGAGGCACGGCTGGCGTCGGGGGAAGGCAGGGTCAGCACTCAC
SRR935201.551865621.+ CTGGGGGGAGCCTGGGGGGAGGCACGGCTGGCGTCGGGGGAAGGCAGGGTCAGCACTCAC
SRR935201.339615116.+ CTGGTGGGAGCCTGGGGGGAGGCACGGCTGGCGTCGGGGGAAGGCAGGGCCAGCACTCAC
```

```
SRR935201.108377682.- CTGGTGGGAGCCTGGGGGGAGGCACGGCTGGCGTCGGGGGAAGGCAGGGTCAGCACTCAC
SRR935201.409608547.- CTGGTGGGAGCCTGGGGGGAGGCACGGCTGGCGTCGGGGGAAGGCAGGGTCAGCACTCAC
SRR935201.313566938.+ CTGGTGGGAGCCTGGGGGGAGGCACGGCTGGCGTCGGGGGAAGGCAGGGTCAGCACTCAC
                      ____________________________________________________________
consensus             CTGGTGGGAGCCTGGGGGGAGGCACGGCTGGCGTCGGGGGAAGGCAGGGTCAGCACTCAC

                          .    :    .    :    .    :    .    :    .    :    .    :
SRR935201.187848779.+ T
SRR935201.293466426.+ T
SRR935201.556099272.+ T
SRR935201.207099838.+ TAC
SRR935201.380622336.+ TAC
SRR935201.423017175.+ TAC
SRR935201.349868662.+ TAC
SRR935201.506148578.+ TAC
SRR935201.22968908.2+ TACTG
SRR935201.551865621.+ TACTGTTAGC
SRR935201.339615116.+ TACTGTTAGCCAAGAGGA
SRR935201.108377682.- TACTGTTAGCCAAGAGGACTGTAC
SRR935201.409608547.- TACTGTTAGCCAAGAGGACTGTAC
SRR935201.313566938.+ TACTGTTAGCCAAGAGGACTGTACCA
                      ____________________________________________________________
consensus             TACTGTTAGCCAAGAGGACTGTACCA
```

(10) The exon 10 sequence was predicted by assembling WGS data using CAP3. The exon 10 is marked in red.

```
                          .    :    .    :    .    :    .    :    .    :    .    :
SRR935201.37348018.2- GCAGGGTCAGCACTCACTACTGTTAGCCAAGAGGACTGTACCAGTCCCTCTGTGTCTTAC
SRR935201.168307309.-  CAGGGTCAGCACTCACTACTGTTAGCCAAGAGGACTGTACCAGTCCCTCTGTGTCTTAC
SRR935201.499327607.-  CAGGGTCAGCACTCACTACTGTTAGCCAAGAGGACTGTACCAGTCCCTCTGTGTCTTAC
SRR935201.341471654.-  CAGGGTCAGCACTCACTACTGTTAGCCAAGAGGACTGTACCAGTCCCTCTGTGTCTTAC
SRR935201.568499170.-  CAGGGTCAGCACTCACTACTGTTAGCCAAGAGGACTGTACCAGTCCCTCTGTGTCTTAC
SRR935201.506148578.-     GGTCAGCACTCACTACTGTTAGCCAAGAGGACTGTACCAGTCCCTCTGTGTCTTAC
SRR935201.538515239.-     GGTCAGCACTCACTACTGTTAGCCAAGAGGACTGTACCAGTCCCTCTGTGTCTTAC
SRR935201.269139399.-                  TACTGTTAGCCAAGAGGACTGTACCAGTCCCTCTGTGTCTTAC
SRR935201.506330554.-                  TACTGTTAGCCAAGAGGACTGTACCAGTCCCTCTGTGTCTTAC
SRR935201.411645211.-                  TACTGTTAGCCAAGAGGACTGTACCAGTCCCTCTGTGTCTTAC
SRR935201.573095479.-                  TACTGTTAGCCAAGAGGACTGTACCAGTCCCTCTGTGTCTTAC
SRR935201.4238465.2-                    ACTGTTAGCCAAGAGGACTGTACCAGTCCCTCTGTGTCTTAC
SRR935201.161521485.-                   ACTGTTAGCCAAGAGGACTGTACCAGTCCCTCTGTGTCTTAC
SRR935201.524048284.-                   ACTGTTAGCCAAGAGGACTGTACCAGTCCCTCTGTGTCTTAC
SRR935201.473437099.+                    CTGTTAGCCAAGAGGACTGTACCAGTCCCTCTGTGTCTTAC
SRR935201.554239656.+                           CCAAGAGGACTGTACCAGTCCCTCTGTGTCTTAC
SRR935201.156047135.+                                         CCAGTCCCTCTGTGTCTTAC
SRR935201.411816489.-                                         CCAGTCCCTCTGTGTCTTAC
SRR935201.293466426.-                                          CAGTCCCTCTGTGTCTTAC
SRR935201.556099272.-                                          CAGTCCCTCTGTGTCTTAC
SRR935201.411104089.-                                            GTCCCTCTGTGTCTTAC
SRR935201.22968908.1-                                                      GTCTTGC
SRR935201.40823642.1+                                                           AC
                      ____________________________________________________________
consensus             GCAGGGTCAGCACTCACTACTGTTAGCCAAGAGGACTGTACCAGTCCCTCTGTGTCTTAC

                          .    :    .    :    .    :    .    :    .    :    .    :
SRR935201.37348018.2- TCTGAGCCTTTGTTTCGCTCTGCACAAGGAGATGAATTGC
SRR935201.168307309.- TCTGAGCCTTTGTTTCGCTCTGCACAAGGAGATGAATTGCT
SRR935201.499327607.- TCTGAGCCTTTGTTTCGCTCTGCACAAGGAGATGAATTGCT
SRR935201.341471654.- TCTGAGCCTTTGTTTCGCTCTGCACAAGGAGATGAATTGCT
SRR935201.568499170.- TCTGAGCCTTTGTTTCGCTCTGCACAAGGAGATGAATTGCT
SRR935201.506148578.- TCTGAGCCTTTGTTTCGCTCTGCACAAGGAGATGAATTGCTGGT
SRR935201.538515239.- TCTGAGCCTTTGTTTCGCTCTGCACAAGGAGATGAATTGCTGGT
SRR935201.269139399.- TCTGAGCCTTTGTTTCGCTCTGCACAAGGAGATGAATTGCTGGTAGAGTGTCACTAC
SRR935201.506330554.- TCTGAGCCTTTGTTTCGCTCTGCACAAGGAGATGAATTGCTGGTAGAGTGTCACTAC
SRR935201.411645211.- TCTGAGCCTTTGTTTCGCTCTGCACAAGGAGATGAATTGCTGGTAGAGTGTCACTAC
SRR935201.573095479.- TCTGAGCCTTTGTTTCGCTCTGCACAAGGAGATGAATTGCTGGTAGAGTGTCACTAC
SRR935201.4238465.2-  TCTGAGCCTTTGTTTCGCTCTGCACAAGGAGATGAATTGCTGGTAGAGTGTCACTACC
SRR935201.161521485.- TCTGAGCCTTTGTTTCGCTCTGCACAAGGAGATGAATTGCTGGTAGAGTGTCACTACC
SRR935201.524048284.- TCTGAGCCTTTGTTTCGCTCTGCACAAGGAGATGAATTGCTGGTAGAGTGTCACTACC
SRR935201.473437099.+ TCTGAGCCTTTGTTTCGCTCTGCACAAGGAGATGAATTGCTGGTAGAGTGTCACTACCA
SRR935201.554239656.+ TCTGAGCCTTTGTTTCGCTCTGCACAAGGAGATGAATTGCTGGTAGAGTGTCACTACCAG
SRR935201.156047135.+ TCTGAGCCTTTGTTTCGCTCTGCACAAGGAGATGAATTGCTGGTAGAGTGTCACTACCAG
SRR935201.411816489.- TCTGAGCCTTTGTTTCGCTCTGCACAAGGAGATGAATTGCTGGTAGAGTGTCACTACCAG
SRR935201.293466426.- TCTGAGCCTTTGTTTCGCTCTGCACAAGGAGATGAATTGCTGGTAGAGTGTCACTACCAG
SRR935201.556099272.- TCTGAGCCTTTGTTTCGCTCTGCACAAGGAGATGAATTGCTGGTAGAGTGCCACTACCAG
SRR935201.411104089.- TCTGAGCCTTTGTTTCGCTCTGCACAAGGAGATGAATTGCTGGTAGAGTGTCACTACCAG
```

```
SRR935201.22968908.1- TCTGAGCCTTTGTTTCGCTCTGCACAAGGAGATGAATTGCTGGTAGAGTGTCACTACCAG
SRR935201.40823642.1+ TCTGAGCCTTTGTTTCGCTCTGCACAAGGAGATGAATTGCTGGTAGAGTGTCACTACCAG
SRR935201.461138727.-          TTGTTGCGCTCTGCACAAGGAGATGAATTGCTGGTAGAGTGTCACTTCCAG
SRR935201.12396866.1-                        ACAAGGAGATGAATTGCTGGTAGAGTGTCACTACCAG
SRR935201.224199452.+                             GAGATGAATTGCTGGTAGAGTGTCACTACCAG
SRR935201.415015000.+                             GAGATGAATTGCTGGTAGAGTGTCACTACCAG
SRR935201.374163613.+                                         TGGTAGAGTGTCACTACCAG
SRR935201.73930900.2+                                          GGTAGAGTGTCACTACCAG
SRR935201.513093324.+                                          GGTAGATTGTCACTACCAG
SRR935201.435717164.+                                          GGTAGAGTGTCACTACCAG
SRR935201.423135014.+                                          GGTAGAGTGTCACTACCAG
SRR935201.412300226.+                                          GGTAGAGTGTCACTACCAG
SRR935201.407825235.+                                          GGTAGAGTGTCACTACCAG
SRR935201.329252438.+                                          GGTAGAGTGTCACTACCAG
SRR935201.212508742.+                                          GGTAGAGTGTCACTACCAG
SRR935201.557402389.+                                          GGTAGAGTGTCACTACCAG
SRR935201.368205975.+                                             AGAGTGTCACTACCAG
SRR935201.562719691.+                                             AGAGTGTCACTACCAG
SRR935201.157721503.+                                               AGTGTCACTACCAG
SRR935201.505957160.+                                               AGTGTCACTACCAG
SRR935201.330495544.+                                                  GTCACTACCAG
SRR935201.416678493.+                                                    CACTACCAG
SRR935201.366008826.+                                                     ACTACCAG
SRR935201.427910512.+                                                     ACTACCAG
SRR935201.467596046.+                                                        ACCAG
                      ____________________________________________________________
consensus             TCTGAGCCTTTGTTTCGCTCTGCACAAGGAGATGAATTGCTGGTAGAGTGTCACTACCAG

                          .    :    .    :    .    :    .    :    .    :    .    :
SRR935201.554239656.+ ACGCTG
SRR935201.156047135.+ ACGCTGGACCGCGACTCCTT
SRR935201.411816489.- ACGCTGGACCGCGACTCCTT
SRR935201.293466426.- ACGCTGGACCGCGACTCCTTG
SRR935201.556099272.- ACGCTGGACCGCGACTCCTTG
SRR935201.411104089.- ACGCTGGACCGCGACTCCTTGAC
SRR935201.22968908.1- ACGCTGGACCGCGACTCCTTGACTTTTGTAAGT
SRR935201.40823642.1+ ACGCTGGACCGCGACTCCTTGACTTTTGTAAGTACCTC
SRR935201.461138727.- ACGCTGGACCGCGACTCCTTGACTTTTGTAAGTACCTCTCTCCCCATCG
SRR935201.12396866.1- ACGCTGGACCGCGACTCCTTGACTTTTGTAAGTACCTCTCTCCCCATCGTGGTCAATGAC
SRR935201.224199452.+ ACGCTGGACCGCGACTCCTTGACTTTTGTAAGTACCTCTCTCCCCATCGTGGTCAATGAC
SRR935201.415015000.+ ACGCTGGACCGCGACTCCTTGACTTTTGTAAGTACCTCTCTCCCCATCGTGGTCAATGAC
SRR935201.374163613.+ ACGCTGGACCGCGACTCCTTGACTTTTGTAAGTACCTCTCTCCCCATCGTGGTCAATGAC
SRR935201.73930900.2+ ACGCTGGACCGCGACTCCTTGACTTTTGTAAGTACCTCTCTCCCCATCGTGGTCAATGAC
SRR935201.513093324.+ ACGCTGGACCGCGACTCCTTGACTTTTGTAAGTACCTCTCTCCCCATCGTGGTCAATGAC
SRR935201.435717164.+ ACGCTGGACCGCGACTCCTTGACTTTTGTAAGTACCTCTCTCCCCATCGTGGTCAATGAC
SRR935201.423135014.+ ACGCTGGACCGCGACTCCTTGACTTTTGTAAGTACCTCTCTCCCCATCGTGGTCAATGAC
SRR935201.412300226.+ ACGCTGGACCGCGACTCCTTGACTTTTGTAAGTACCTCTCTCCCCATCGTGGTCAATGAC
SRR935201.407825235.+ ACGCTGGACCGCGACTCCTTGACTTTTGTAAGTACCTCTCTCCCCATCGTGGTCAATGAC
SRR935201.329252438.+ ACGCTGGACCGCGACTCCTTGACTTTTGTAAGTACCTCTCTCCCCATCGTGGTCAATGAC
SRR935201.212508742.+ ACGCTGGACCGCGACTCCTTGACTTTTGTAAGTACCTCTCTCCCCATCGTGGTCAATGAC
SRR935201.557402389.+ ACGCTGGACCGCGACTCCTTGACTTTTGTAAGTACCTCTCTCCCCATCGTGGTCAATGAC
SRR935201.368205975.+ ACGCTGGACCGCGACTCCTTGACTTTTGTAAGTACCTCTCTCCCCATCGTGGTCAATGAC
SRR935201.562719691.+ ACGCTGGACCGCGACTCCTTGACTTTTGTAAGTACCTCTCTCCCCATCGTGGTCAATGAC
SRR935201.157721503.+ ACGCTGGACCGCGACTCCTTGACTTTTGTAAGTACCTCTCTCCCCATCGTGGTCAATGAC
SRR935201.505957160.+ ACGCTGGACCGCGACTCCTTGACTTTTGTAAGTACCTCTCTCCCCATCGTGGTCAATGAC
SRR935201.330495544.+ ACGCTGGACCGCGACTCCTTGACTTTTGTAAGTACCTCTCTCCCCATCGTGGTCAATGAC
SRR935201.416678493.+ ACGCTGGACCGCGACTCCTTGACTTTTGTAAGTACCTCTCTCCCCATCGTGGTCAATGAC
SRR935201.366008826.+ ACGCTGGACCGCGACTCCTTGACTTTTGTAAGTACCTCTCTCCCCATCGTGGTCAATGAC
SRR935201.427910512.+ ACGCTGGACCGCGACTCCTTGACTTTTGTAAGTACCTCTCTCCCCATCGTGGTCAATGAC
SRR935201.467596046.+ ACGCTGGACCGCGACTCCTTGACTTTTGTAAGTACCTCTCTCCCCATCGTGGTCAATGAC
SRR935201.101823809.+   GCTGGACCGCGACTCCTTGACTTTTGTAAGTACCTCTCTCCCCATCGTGGTCAATGAC
SRR935201.3985358.1+                  CCTTGACTTTTGTAAGTACCTCTCTCCCCATCGTGGTCAATGAC
SRR935201.445949331.+                 CCTTGACTTTTGTAAGTACCTCTCTCCCCATCGTGGTCAATGAC
SRR935201.433861364.+                 CCTTGACTTTTGTAAGTACCTCTCTCCCCATCGTGGTCAATGAC
SRR935201.197782883.+                 CCTTGACTTTTGTAAGTACCTCTCTCCCCATCGTGGTCAATGAC
SRR935201.98568428.1+                 CCTTGACTTTTGTAAGTACCTCTCTCCCCATCGTGGTCAATGAC
SRR935201.33074310.1+                 CCTTGACTTTTGTAAGTACCTCTCTCCCCATCGTGGTCAATGAC
SRR935201.515420402.+                 CCTTGACTTTTGTAAGTACCTCTCTCCCCATCGTGGTCAATGAC
                      ____________________________________________________________
consensus             ACGCTGGACCGCGACTCCTTGACTTTTGTAAGTACCTCTCTCCCCATCGTGGTCAATGAC

                          .    :    .    :    .    :    .    :    .    :    .    :
SRR935201.12396866.1- GGT
SRR935201.224199452.+ GGTAGCCT
SRR935201.415015000.+ GGTAGCCT
SRR935201.374163613.+ GGTAGCCTCAGAATAGAGCC
SRR935201.73930900.2+ GGTAGCCTCAGAATAGAGCCC
```

```
SRR935201.513093324.+ GGTAGCCTCAGAATAGAGCCC
SRR935201.435717164.+ GGTAGCCTCAGAATAGAGCCC
SRR935201.423135014.+ GGTAGCCTCAGAATAGAGCCC
SRR935201.412300226.+ GGTAGCCTCAGAATAGAGCCC
SRR935201.407825235.+ GGTAGCCTCAGAATAGAGCCC
SRR935201.329252438.+ GGTAGCCTCAGAATAGAGCCC
SRR935201.212508742.+ GGTAGCCTCAGAATAGAGCCC
SRR935201.557402389.+ GGTAGCCTCAGAATAGAGCCC
SRR935201.368205975.+ GGTAGCCTCAGAATAGAGCCCCAT
SRR935201.562719691.+ GGTAGCCTCAGAATAGAGCCCCAT
SRR935201.157721503.+ GGTAGCCTCAGAATAGAGCCCCATCC
SRR935201.505957160.+ GGTAGCCTCAGAATAGAGCCCCATCC
SRR935201.330495544.+ GGTAGCCTCAGAATAGAGCCCCATCCCTG
SRR935201.416678493.+ GGTAGCCTCAGAATAGAGCCCCATCCCTGGC
SRR935201.366008826.+ GGTAGCCTCAGAATAGAGCCCCATCCCCGGCC
SRR935201.427910512.+ GGTAGCCTCAGAATAGAGCCCCATCCCTGGCC
SRR935201.467596046.+ GGTAGCCTCAGAATAGAGCCCCATCCCTGGCCTTG
SRR935201.101823809.+ GGTAGCCTCAGAATAGAGCCCCATCCCTGGCCTTGGCTATCA
SRR935201.3985358.1+  GGTAGCCTCAGAATAGAGCCCCATCCCTGGCCTTGGCTATCACGTGACTCAGATTA
SRR935201.445949331.+ GGTAGCCTCAGAATAGAGCCCCATCCCTGGCCTTGGCTATCACGTGACTCAGATTA
SRR935201.433861364.+ GGTAGCCTCAGAATAGAGCCCCATCCCTGGCCTTGGCTATCACGTGACTCAGATTA
SRR935201.197782883.+ GGTAGCCTCAGAATAGAGCCCCATCCCTGGCCTTGGCTATCACGTGACTCAGATTA
SRR935201.98568428.1+ GGTAGCCTCAGAATAGAGCCCCATCCCTGGCCTTGGCTATCACGTGACTCAGATTA
SRR935201.33074310.1+ GGTAGCCTCAGAATAGAGCCCCATCCCTGGCCTTGGCTATCACGTGACTCAGATTA
SRR935201.515420402.+ GGTAGCCTCAGAATAGAGCCCCATCCCTGGCCTTGGCTATCACGTGACTCAGATTA
                      ____________________________________________________________
consensus             GGTAGCCTCAGAATAGAGCCCCATCCCTGGCCTTGGCTATCACGTGACTCAGATTA
```

(11) The exon 11 sequence was predicted by assembling WGS data using CAP3. The exon 11 is on the reverse strand and marked in red.

```
                          .    :    .    :    .    :    .    :    .    :    .    :
SRR935201.352828868.+ GAGAAAAGGGCAGACAAATAGTTTGTACACAGGTGATCTAACTTCTAGAGGTCACTCTTG
SRR935201.87765771.2+         GGCAGACAAATACTTTGTACACAAGTGATCTAACTTCTAGAGGTCACTCTTG
SRR935201.206867574.+          GCAGACAAATACTTTGTACACAAGTGATCTAACTTCTAGAGGTCACTCTTG
SRR935201.295367054.-          GCAGACAAATACTTTGTACACAAGTGATCTAACTTCTAGAGGTCACTCTTG
SRR935201.234200256.-          GCAGACAAATACTTTGTACACAAGTGATCTAACTTCTAGAGGTCACTCTTG
SRR935201.332849002.+          GCAGACAAATACTTTGTACACAAGTGATCTAACTTCTAGAGGTCACTCTTG
SRR935201.252100657.+            AGACAAATACTTTGTACACAAGTGATCTAACTTCTAGAGGTCACTCTTG
SRR935201.552696772.+            AGACAAATACTTTGTACACAAGTGATCTAACTTCTAGAGGTCACTCTTG
SRR935201.499737647.+            AGACAAATACTTTGTACACAAGTGATCTAACTTCTAGAGGTCACTCTTG
SRR935201.193245635.+             GACAAATACTTTGTACACAAGTGCTCTCACTTCTAGAGGTCACTCTTT
SRR935201.528680347.+             GACAAATACTTTGTACACAAGTGATCTAACTTCTAGAGGTCACTCTTG
SRR935201.565527638.+             GACAAATACTTTGTACACAAGTGATCTAACTTCTAGAGGTCACTCTTG
SRR935201.56330955.1-                     CTTTGTACACAAGTGATCTAACTTCTAGAGGTCACTCTTG
SRR935201.514969456.-                     CTTTGTACACAAGTGATCTAACTTCTAGAGGTCACTCTTG
SRR935201.541511852.-                     CTTTGTACACAAGTGATCTAACTTCTAGAGGTCACTCTTG
SRR935201.37175875.2+                          TACACAAGTGATCTAACTTCTAGAGGTCACTCTTG
SRR935201.253998948.+                               AAGTGATCTAACTTCTAGAGGTCACTCTTG
SRR935201.262986663.+                                    ATCTAACTTCTAGAGGTCACTCTTG
SRR935201.13908406.1-                                       TAACTTCTAGAGGTCACTCTTG
SRR935201.149543303.-                                         ACTTCTAGAGGTCACTCTTG
SRR935201.351902081.+                                         ACTTCTAGAGGTCACTCTTG
SRR935201.141791723.-                                             CTAGAGGTCACTCTTG
SRR935201.18149375.1-                                              TAGAGGTCACTCTTG
SRR935201.470671059.-                                              TAGAGGTCACTCTTG
SRR935201.32364080.1-                                                    TCACTCTTG
SRR935201.349574253.-                                                    TCACTCTTG
SRR935201.435313688.+                                                            G
                      ____________________________________________________________
consensus             GAGAAAAGGGCAGACAAATACTTTGTACACAAGTGATCTAACTTCTAGAGGTCACTCTTG

                          .    :    .    :    .    :    .    :    .    :    .    :
SRR935201.352828868.+ AGGGTTTTATCTTCCAGGATGACTCACTCTGATGCCTCCT
SRR935201.87765771.2+ AGGGTTTTATCTTCCAGGATGACTCACTCTGATGCCTCCTCCCCCAGC
SRR935201.206867574.+ AGGGTTTTATCTTCCAGGATGACTCACTCTGATGCCTCCTCCCCCAGCT
SRR935201.295367054.- AGGGTTTTATCTTCCAGGATGACTCACTCTGATGCCTCCTCCCCCAGCT
SRR935201.234200256.- AGGGTTTTATCTTCCAGGATGACTCACTCTGATGCCTCCTCCCCCAGCT
SRR935201.332849002.+ AGGGTTTTATCTTCCAGGATGACTCACTCTGATGCCTCCTCCCCCAGCT
SRR935201.252100657.+ AGGGTTTTATCTTCCAGGATGACTCACTCTGATGCCTCCTCCCCCAGCTCA
SRR935201.552696772.+ AGGGATTTATCTTCCAGGATGACTCACTCTGATGCCTCCTCCCCCAGCTCA
SRR935201.499737647.+ AGGGTTTTATCTTCCAGGATGACTCACTCTGATGCCTCCTCCCCCAGCTCA
SRR935201.193245635.+ AGGGTTTTATGTTCCAGGATGACTCACTCTGATGCCTCCTCCCCCAGCTCAT
SRR935201.528680347.+ AGGGTTTTATCTTCCAGGATGACTCACTCTGATGCCTCCTCCCCCAGCTCAT
SRR935201.565527638.+ AGGGTTTTATCTTCCAGGATGACTCACTCTGATGCCTCCTCCCCCAGCTCAT
SRR935201.56330955.1- AGGGTTTTATCTTCCAGGATGACTCACTCTGATGCCTCCTCCCCCAGCTCATGGGCCATG
```

```
SRR935201.514969456.- AGGGTTTTATCTTCCAGGATGACTCACTCTGATGCCTCCTCCCCCAGCCCATGGGCCATG
SRR935201.541511852.- AGGGTTTTATCTTCCAGGATGACTCACTCTGATGCCTCCTCCCCCAGCTCATGGGCCATG
SRR935201.37175875.2+ AGGGTTTTATCTTCCAGGATGACTCACTCTGATGCCTCCTCCCCCAGCTCATGGGCCATG
SRR935201.253998948.+ AGGGTTTTATCTTCCAGGATGACTCACTCTGATTCCTCCTCCCCCAGCTCATGGGCCATG
SRR935201.262986663.+ AGGGTTTTATCTTCCAGGATGACTCACTCTGATGCCTCCTCCCCCAGCTCATGGGCCATG
SRR935201.13908406.1- AGGGTTTTATCTTCCAGGATGACTCACTCTGATGCCTCCTCCCCCAGCTCATGGGCCATG
SRR935201.149543303.- AGGGTTTTATCTTCCAGGATGACTCACTCTGATGCCTCCTCCCCCAGCTCATGGGCCATG
SRR935201.351902081.+ AGGGTTTTATCTTCCAGGATGACTCACTCTGATGCCTCCTCCCCCAGCTCATGGGCCATG
SRR935201.141791723.- AGGGTTTTATCTTCCAGGATGACTCACTCTGATGCCTCCTCCCCCAGCTCATGGGCCATG
SRR935201.18149375.1- AGGGTTTTATCTTCCAGGATGACTCACTCTGATGCCTCCTCCCCCAGCTCATGGGCCATG
SRR935201.470671059.- AGGGTTTTATCTTCCAGGATGACTCACTCTGATGCCTCCTCCCCCAGCTCATGGGCCATG
SRR935201.32364080.1- AGGGTTTTATCTTCCAGGATGACTCACTCTGATGCCTCCTCCCCCAGCTCATGGGCCATG
SRR935201.349574253.- AGGGTTTTATCTTCCAGGATGACTCACTCTGATGCCTCCTCCCCCAGCTCATGGGCCATG
SRR935201.435313688.+ AGGGTTTTATCTTCCAGGATGACTCACTCTGATGCCTCCTCCCCCAGCTCATGGGCCATG
SRR935201.131570264.-             TCCAGGATGACTCACTCTGATGCCTCCTCCCCCAGCTCATGGGCCATG
SRR935201.252100657.-                AGGATGACTCACTCTGATGCCTCCTCCCCCAGCTCATGGGCCATG
SRR935201.499737647.-                AGGATGACTCACTCTGATGCCTCCTCCCCCAGCTCATGGGCCATG
SRR935201.552696772.-                AGGATGACTCACTCTGATGCCTCCTCCCCCAGCTCATGGGCCATG
SRR935201.13348490.1+                 GGATGACTCACTCTGATGCCTCCTCCCCCAGCTCATGGGCCATG
SRR935201.384946346.+                 GGATGACTCACTCTAATGCCTCCTCCCCCACCTCATGGGCCATG
SRR935201.523622302.+                 GGATGACTCACTCTGATGCCTCCTCCCCCAGCTCATGGGCCATG
SRR935201.514160843.+                 GGATGACTCACTCTGATGCCTCCTCCCCCAGCTCATGGGCCATG
SRR935201.456701842.+                 GGATGACTCACTCTGATGCCTCCTCCCCCAGCTCATGGGCCATG
SRR935201.35701431.1+                 GGATGACTCACTCTGATGCCTCCTCCCCCAGCTCATGGGCCATG
SRR935201.568569070.+                 GGATGACTCACTCTGATGCCTCCTCCCCCAGCTCATGGGCCATG
SRR935201.193591880.+                   ATGACTCACTCTGATGCCTCCTCCCCCAGCTCATGGGCCATG
SRR935201.199728885.-                    TGACTCACTCTGATGCCTCCTCCCCCAGCTCATGGGCCATG
SRR935201.419502220.+                     TTCTCACTCTGATGCCTCCTCCCCCAGCTCATGGGCCATG
SRR935201.12780193.2+                           CTCTGATGCCTCCTCCCCCAGCTCATGGGCCATG
SRR935201.352772938.-                                                AGCTCATGGGCCATG
SRR935201.342952883.-                                          TCCCCCAGCTCATGGGCCATG
SRR935201.430474189.+                                              CCAGCTCATGGGCCATG
SRR935201.207941965.+                               GATGCCTCCTCCCCCAGCTCATGGGCCATG
SRR935201.517443781.+                               GATGCCTCCTCCCCCAGCTCATGGGCCATG
                      ____________________________________________________________
consensus             AGGGTTTTATCTTCCAGGATGACTCACTCTGATGCCTCCTCCCCCAGCTCATGGGCCATG

                          .    :    .    :    .    :    .    :    .    :    .    :
SRR935201.37175875.2+ TAGAT
SRR935201.253998948.+ TACATGATGA
SRR935201.262986663.+ TAGATGATGTCAGCG
SRR935201.13908406.1- TAGATGATGTCAGCGTAC
SRR935201.149543303.- TAGATGATGTCAGCGTACCC
SRR935201.351902081.+ TAGATGATGTCAGCGTACCC
SRR935201.141791723.- TAGATGATGTCAGCGTACCCCATG
SRR935201.18149375.1- TAGATGATGTCAGCGTACCCCATGC
SRR935201.470671059.- TAGATGATGTCAGCGTACCCCATGC
SRR935201.32364080.1- TAGATGATGTCAGCGTACCCCATGCAGCTGG
SRR935201.349574253.- TAGATGATGTCAGCGTACCCCATGCAGCTGG
SRR935201.435313688.+ TAGATGATGTCAGCGTACCCCATGCAGCTGGAGATGTTG
SRR935201.131570264.- TAGATGATGTCAGCGTACCCCATGCAGCTGGAGATGTTGTTTCGGGGATAGT
SRR935201.252100657.- TAGATGATGTCAGCGTACCCCATGCAGCTGGAGATGTTGTTTCGGGGATAGTAGA
SRR935201.499737647.- TAGATGATGTCAGCGTACCCCATGCAGCTGGAGATGTTGTTTCGGGGATAGTAGA
SRR935201.552696772.- TAGATGATGTCAGCGTACCCCATGCAGCTGGAGATGTTGTTTCGGGGATAGTAGA
SRR935201.13348490.1+ TAGATGATGTCAGCGTACCCCATGCAGCTGGAGATGTTGTTTCGGGGATAGTAGAA
SRR935201.384946346.+ TAGATGATGTCAGCGTACCCCATGCAGCTGGAGATGTTGTTTCTGCGATAGTAGAA
SRR935201.523622302.+ TAGATGATGTCAGCGTACCCCATGCAGCTGGAGATGTTGTTTCGGGGATAGTAGAA
SRR935201.514160843.+ TAGATGATGTCAGCGTACCCCATGCAGCTGGAGATGTTGTTTCGGGGATAGTAGAA
SRR935201.456701842.+ TAGATGATGTCAGCGTACCCCATGCAGCTGGAGATGTTGTTTCGGGGATAGTAGAA
SRR935201.35701431.1+ TAGATGATGTCAGCGTACCCCATGCAGCTGGAGATGTTGTTTCGGGGATAGTAGAA
SRR935201.568569070.+ TAGATGATGTCAGCGTACCCCATGCAGCTGGAGATGTTGTTTCGGGGATAGTAGAA
SRR935201.193591880.+ TAGATGATGTCAGCGTACCCCATGCAGCTGGAGATGTTGTTTCGGGGATAGTAGAAGA
SRR935201.199728885.- TAGATGATGTCAGCGTACCCCATGCAGCTGGAGATGTTGTTTCGGGGATAGTAGAAGAG
SRR935201.419502220.+ TAGATGATGTCAGCGTACCCCATGCAGCTGGAGATGTTGTTTCGGGGATAGTAGAAGAGA
SRR935201.12780193.2+ TAGATGATGTCAGCGTACCCCATGCAGCTGGAGATGTTGTTTCGGGGATAGTAGAAGAGA
SRR935201.352772938.- TAGATGATGTCAGCGTACCCCATGCAGCTGGAGATGTTGTTTCGGGGATAGTAGAAAAGA
SRR935201.342952883.- TAGATGATGTCAGCGTACCCCATGCAGCTGGAGATGTTGTTTCGGGGATAGTAGAAGAGA
SRR935201.430474189.+ TAGATGATGTCAGCGTACCCCATGCAGCTGGAGATGTTGTTTCGGGGATAGTAGAAGAGA
SRR935201.207941965.+ TAGATGATGTCAGCGTACCCCATGCAGCTGGAGATGTTGTTTCGGGGATAGTAGAAGAGA
SRR935201.517443781.+ TAGATGATGTCAGCGTACCCCATGCAGCTGGAGATGTTGTTTCGGGGATAGTAGAAGAGA
SRR935201.329136588.- TAGATGATGTCAGCGTACCCCATGCAGCTGGAGATGTTGTTTCGGGGATAGTAGAAGAGA
SRR935201.128456882.+   GATGATGTCAGCGTACCCCATGCAGCTGGAGATGTTGTTTCGGGGATAGTAGAAGAGA
SRR935201.76840105.1+         GTCAGCGTACCCCATGCAGCTGGAGATGTTGTTTCGGGGATAGTAGAAGAGA
SRR935201.222717944.+         GTCAGCGTACCCCATGCAGCTGGAGATGTTGTTTCGGGGATAGTAGAAGAGA
SRR935201.425222722.+         GTCAGCGTACCCCATGCAGCTGGAGATGTTGTTTCGGGGATAGTAGAAGAGA
SRR935201.134749511.-                TACCCCATGCAGCTGGAGATGTTGTTTCGGGTATAGTAGAAGAGA
SRR935201.36097474.1+                 ACCCCATGCAGCTGGAGATGTTGTTTCGGGGATAGTAGAAGAGA
```

```
SRR935201.210401001.+                 ACCCCATGCAGCTGGAGATGTTGTTTCGGGGATAGTAGAAGAGA
SRR935201.209752593.-                       TGCAGCTGGAGATGGTGTTTCGGGGATAGTAGAAGAGA
SRR935201.457084483.-                            CTGGAGATGTTGTTTCGGGGATAGTAGAAGAGA
SRR935201.525008230.-                            GTGGCGATGTTGTTTCGGGTATAGTCGAAGAGA
SRR935201.123132856.+                              GGAGATGTTGTTTCGGGGATAGTAGAAGAGA
SRR935201.547109235.+                                  ATGTTGTTTCGGGGATAGTAGAAGAGA
SRR935201.478949874.-                                     TTGTTTCGGGGATAGTAGAAGAGA
SRR935201.351379665.+                                       GTTTCGGGGATAGTAGAAGAGA
SRR935201.12780193.1-                                              GGATAGTAGAAGAGA
SRR935201.13348490.2-                                              GGATAGTAGAAGAGA
SRR935201.191132237.+                                                      GAAGAGA
SRR935201.69624728.2+                                                           GA
                      ____________________________________________________________
consensus             TAGATGATGTCAGCGTACCCCATGCAGCTGGAGATGTTGTTTCGGGGATAGTAGAAGAGA

                          .    :    .    :    .    :    .    :    .    :    .    :
SRR935201.12780193.2+ AAGATG
SRR935201.352772938.- AAGATGAGGCACATCTCATTAATGG
SRR935201.342952883.- AAGATGAGGCACATCTCAT
SRR935201.430474189.+ AAGATGAGGCACATCTCATTAAT
SRR935201.207941965.+ AAGATGAGGC
SRR935201.517443781.+ AAGATGAGGC
SRR935201.329136588.- AAGATGAGGCACATCTCATTAATGGTGCTGGGACCCCCC
SRR935201.128456882.+ AAGATGAGGCACATCTCATTAATGGTGCTGGGACCCCCCCTT
SRR935201.76840105.1+ AAGATGAGGCACATCTCATTAATGGTGCTGGGACCCCCCTTGAGAAGA
SRR935201.222717944.+ AAGATGAGGCACATCTCATTAATGGTGCTGGGACCCCCCTTGAGAAGA
SRR935201.425222722.+ AAGATGAGGCACATCTCATTAATGGTGCTGGGACCCCCCTTGAGAAGA
SRR935201.134749511.- AAGATGAGGCACATCTCATTAATGGTGCTGGGACCCCCCCTTGAGAAGAAGAGAG
SRR935201.36097474.1+ AAGATGAGGCACATCCCATTAATGGTGCTGGGACCCCCCTTGGGAAGAAGAGAGA
SRR935201.210401001.+ AAGATGAGGCACATCTCATTAATGGTGCTGGGACCCCCCCTTGAGAAGAAGAGAGA
SRR935201.209752593.- AAGATGAGGCACATCTCATTAATGGTGCTGGGACCCCCCCTTGAGAAGAAGAGAGAGAGA
SRR935201.457084483.- AAGATGAGGCACATCTCATTAATGGTGCTGGGACCCCCCCTTGAGAAGAAGAGAGAGAGA
SRR935201.525008230.- AAGTTGAGGCACATCTTATTAATGGTGCTGGGACCCCCCCTTGAGAAGAAGAGAGAGAGA
SRR935201.123132856.+ AAGATGAGGCACATCTCATTAATGGTGCTGGGACCCCCCCTTGAGCAGAAGAGAGAGAGA
SRR935201.547109235.+ AAGATAAGGCACATCTCATTAATGGTGCTGGGACCCCCCCTTGAGAAGAAGAGAGAGAGA
SRR935201.478949874.- AAGATGAGGCACATCTCATTAATGGTGCTGGGACCCCCCCTTGAGAAGAAGAGAGAGAGA
SRR935201.351379665.+ AAGATGAGGCACATCTCATTAATGGTGCTGGGACCCCCC-TTGAGAAGAAGAGAGAGAGA
SRR935201.12780193.1- AAGATGAGGCACATCTCATTAATGGTGCTGGGACCCCCCCTTGAGAAGAAGAGAGAGAGA
SRR935201.13348490.2- AAGATGAGGCACATCTCATTAATGGTGCTGGGACCCCCC-TTGAGAAGAAGAGAGAGAGA
SRR935201.191132237.+ AAGATGAGGCACATCTCATTAATGGTGCTGGGACCCCCCCTTGAGAAGAAGAGAGAGAGA
SRR935201.69624728.2+ AAGATGAGGCACATCTCATTAATGGTGCTGGGACCCCCC-TTGAGAAGAAGAGAGAGAGA
SRR935201.76840105.2-  AGATGAGGCACATCTCATTAATGGTGCTGGGACCCCCC-TTGAGAAGAAGAGAGAGAGA
SRR935201.218049226.+   GATGAGGCACATCTCATTAATGGTGCTGGGACCCCCC-TTGAGAAGAAGAGAGAGAGA
SRR935201.566153054.+         GCACATCTCATTAATGGTGCTGGGACCCCCC-TTGAGAAGAAGAGAGAGAGA
SRR935201.128456882.-             ATCTCATTAATGGTGCTGGGACCCCCCCTTGAGAAGAAGAGAGAGAGA
SRR935201.439165094.+                TCATTAATGGGGCTGGGACCCCCCCTTGAGAAGAAGAGAGAGAGA
SRR935201.217555372.+                     AATGGTGCTGGGACCCCCCCTTGAGAAGAAGAGAGAGAGA
                      ____________________________________________________________
consensus             AAGATGAGGCACATCTCATTAATGGTGCTGGGACCCCCCCTTGAGAAGAAGAGAGAGAGA

                          .    :    .    :    .    :    .    :    .    :    .    :
SRR935201.209752593.- GA
SRR935201.457084483.- GAGAGAT
SRR935201.525008230.- GAGAGAT
SRR935201.123132856.+ GAGAGATGG
SRR935201.547109235.+ GAGAGATGGAGAG
SRR935201.478949874.- GAGAGATGGAGAGACA
SRR935201.351379665.+ GAGAGATGGAGAGACAGAG
SRR935201.12780193.1- GAGAGATGGAGAGACAGAGATACAC
SRR935201.13348490.2- GAGAGATGGAGAGACAGAGATACACA
SRR935201.191132237.+ GAGAGATGGAGAGACAGAGATACACAGGCATGC
SRR935201.69624728.2+ GAGAGATGGAGAGACAGAGATACACAGGCATGCAGAGAG
SRR935201.76840105.2- GAGAGATGGAGAGACAGAGATACACAGGCATGCAGAGAGAGG
SRR935201.218049226.+ GAGAGATGGAGAGACAGAGATACACAGGCATGCAGAGGGAGGA
SRR935201.566153054.+ GAGAGATGGAGAGACAGAGATACACAGGCATGCAGAGAGAGGAGAACAA
SRR935201.128456882.- GAGAGATGGAGAGACAGAGATACACAGGCATGCAGAGAGAGGAGAACAAGGT
SRR935201.439165094.+ GAGAGATGGAGAGACAGAGATACACAGGCATGCAGAGAGAGGAGAACAAGGTAAG
SRR935201.217555372.+ GAGAGATGGAGAGACAGAGATACACAGGCATGCAGAGAGAGGAGCACAAGGTAAGGAGAA
                      ____________________________________________________________
consensus             GAGAGATGGAGAGACAGAGATACACAGGCATGCAGAGAGAGGAGAACAAGGTAAGGAGAA
```

(12) The exon 12 sequence was predicted by assembling WGS data using CAP3. The exon 12 is marked in red.

```
                          .    :    .    :    .    :    .    :    .    :    .    :
SRR935201.493717938.+ ACTTTATACACATTTTGTCAAAGCTTTCAGTCAACCCTCAACTAGTGTCTGTTCTTCCTG
```

```
SRR935201.258879956.-                        GCTTTCAGTCAACCCTCAACTAGTGTCTGTTCTTCCTG
SRR935201.209855162.-                                     CCTCAACTAGTGTCTGTTCTTCCTG
SRR935201.487084336.+                                     CCTCAACTAGTGTCTGTTCTTCCTG
SRR935201.16734176.1-                                                    GTTCTTCCTG
SRR935201.481570138.-                                                    GTTCTTCCTG
SRR935201.381291224.-                                                    GTTCTTCCTG
SRR935201.137753478.-                                                    GTTCTTCCTG
SRR935201.71368998.1-                                                    GTTCTTCCTG
SRR935201.487689041.-                                                    GTTCTTCCTG
                      ____________________________________________________________
consensus             ACTTTATACACATTTTGTCAAAGCTTTCAGTCAACCCTCAACTAGTGTCTGTTCTTCCTG

                          .    :    .    :    .    :    .    :    .    :    .    :
SRR935201.493717938.+ CCCATGACAGCTCCATGGAGGGCATGATGGCCACGAACAA
SRR935201.258879956.- CCCATGACAGCTCCATGGAGGGTATGATGGCCACGAACAAGGTGGAGTGGACCCCGGAGA
SRR935201.209855162.- CCCATGACAGCTCCATGGAGGGTATGATGGCCACGAACAAGGTGGAGTGGACCCCGGAGA
SRR935201.487084336.+ CCCATGACAGCTCCATGGAGGGTATGATGGCCACGAACAAGGTGGAGTGGACCCCGGAGA
SRR935201.16734176.1- CCCATGACAGCTCCATGGAGGGTATGATGGCCACGAACAAGGTGGAGTGGACCCCGGAGA
SRR935201.481570138.- CCCATGACAGCTCCATGGAGGGTATGATGGCCACGAACAAGGTGGAGTGGACCCCGGAGA
SRR935201.381291224.- CCCATGACAGCTCCATGGAGGGTATGATGGCCACGAACAAGGTGGAGTGGACCCCGGAGA
SRR935201.137753478.- CCCATGACAGCTCCATGGAGGGTATGATGGCCACGAACAAGGTGGAGTGGACCCCGGAGA
SRR935201.71368998.1- CCCATGACAGCTCCATGGAGGGTATGATGGCCACGAACAAGGTGGAGTGGACCCCGGAGA
SRR935201.487689041.- CCCATGACAGCTCCATGGAGGGTATGATGGCCACGAACAAGGTGGAGTGGACCCCGGAGA
SRR935201.117924569.-                 GGAGGGTATGATGGCCACGAACAAGGTGGAGTGGACCCCGGAGA
SRR935201.265465484.-                       TATGATGGCCACGAACAAGGTGGAGTGGACCCCGGAGA
SRR935201.14572050.1+                             GGCCACGAACAAGGTGGAGTGGACCCCGGAGA
SRR935201.265705458.+                             GGCCACGAACAAGGTGGAGTGGACCCCGGAGA
SRR935201.233315670.+                                         GGTGGAGTGGACCCCGGAGA
SRR935201.55757069.2-                                               GTGGACCCCGGAGA
                      ____________________________________________________________
consensus             CCCATGACAGCTCCATGGAGGGTATGATGGCCACGAACAAGGTGGAGTGGACCCCGGAGA

                          .    :    .    :    .    :    .    :    .    :    .    :
SRR935201.258879956.- AC
SRR935201.209855162.- ACATCAAAAAGGCTG
SRR935201.487084336.+ ACATCAAAAAGGCTG
SRR935201.16734176.1- ACATCAAAAAGGCTGAGAAGGCGTGCAAGG
SRR935201.481570138.- ACATCAAAAAGGCTGAGAAGGCGTGCAAGG
SRR935201.381291224.- ACATCAAAAAGGCTGAGAAGGCGTGCAAGG
SRR935201.137753478.- ACATCAAAAAGGCTGAGAAGGCGTGCAAGG
SRR935201.71368998.1- ACATCAAAAAGGCTGAGAAGGCGTGCAAGG
SRR935201.487689041.- ACATCAAAAAGGCTGAGAAGGCGTGCAAGG
SRR935201.117924569.- ACATCAAAAAGGCTGAGAAGGCGTGCAAGGAGGCCCAGCAGACGGTGATAATAAAG
SRR935201.265465484.- ACATCAAAAAGGCTGAGAAGGCGTGCAAGGAGGCCCAGCAGACGGTGATAATAAAGACCA
SRR935201.14572050.1+ ACATCAAAAAGGCTGAGAAGGCGTGCAAGGAGGCCCAGCAGACGGTGATAATAAAGACCA
SRR935201.265705458.+ ACATCAAAAAGGCTGAGAAGGCGTGCAAGGAGGCCCAGCAGACGGTGATAATAAAGACCA
SRR935201.233315670.+ ACATCAAAAAGGCTGAGAAGGCGTGCAAGGAGGCCCAGCAGACGGTGATAATAAAGACCA
SRR935201.55757069.2- ACATCAAAAAGGCTGAGAAGGCGTGCAAGGAGGCCCAGCAGACGGTGATAATAAAGACCA
SRR935201.472550254.+       AAAAGGCTGAGAAGGCGTGCAAGGAGGCCCAGCAGACGGTGATAATAAAGACCA
SRR935201.19276136.2+           GGCTGAGAAGGCGTGCAAGGAGGCCCAGCAGACGGTGATAATAAAGACCA
SRR935201.530425511.+           GGCTGAGAAGGCGTGCAAGGAGGCCCAGCAGACGGTGATAATAAAGACCA
SRR935201.173174351.+           GGCTGAGAAGGCGTGCAAGGAGGCCCAGCAGACGGTGATAATAAAGACCA
SRR935201.373005701.+                            AGGAGGCCCAGCAGACGGTGATAATAAAGACCA
SRR935201.495010717.+                            AGGAGGCCCAGCAGACGGTGATAATAAAGACCA
SRR935201.2236430.1+                                    CCAGCAGACGGTGATAATAAAGACCA
SRR935201.15553217.1+                                   CCAGCAGACGGTGATAATAAAGACCA
SRR935201.444328212.-                                     AGCAGACGGTGATAATAAAGACCA
SRR935201.293791015.+                                            GGTGATAATAAAGACCA
SRR935201.343780495.-                                             GTGATAATAAAGACCA
SRR935201.441800006.+                                                 TAATAAAGACCA
SRR935201.477791685.+                                                 TAATAAAGACCA
                      ____________________________________________________________
consensus             ACATCAAAAAGGCTGAGAAGGCGTGCAAGGAGGCCCAGCAGACGGTGATAATAAAGACCA

                          .    :    .    :    .    :    .    :    .    :    .    :
SRR935201.265465484.- TT
SRR935201.14572050.1+ TTGACGTA
SRR935201.265705458.+ TTGACGTA
SRR935201.233315670.+ TTGACGTAAGTGTCCCAGGA
SRR935201.55757069.2- TTGACGTAAGTGTCCCAGGAGACACC
SRR935201.472550254.+ TTGACGTAAGTGTCCCAGGAGACACCACGCTCCCGGGGTTAGGGAT
SRR935201.19276136.2+ TTGACGTAAGTGTCCCAGGAGACACCACGCTCCCGGGGTTAGGGATGGAG
SRR935201.530425511.+ TTGACGTAAGTGTCCCAGGAGACACCACGCTCCCGGGGTTAGAGATGGAG
SRR935201.173174351.+ TTGACGTAAGTGTCCCAGGAGACACCACGCTCCCGGGGTTAGGGATGGAG
SRR935201.373005701.+ TTGACGTAAGTGTCCCAGGAGACACCACGCTCCCGGGGTTAGGGATGGAGGGGTGTGGGA
SRR935201.495010717.+ TTGACGTAAGTGTCCCAGGAGACACCACGCTCCCGGGGTTAGGGATGGAGGGGTGTGGGA
SRR935201.2236430.1+  TTGACGTAAGTGTCCCAGGAGACACCACGCTCCCGGGGTTAGGGATGGAGGGGTGTGGGA
```

```
SRR935201.15553217.1+ TTGACGTAAGTGTCCCAGGAGACACCACGCTCCCGGGGTTAGGGATGGAGGGGTGTGGGA
SRR935201.444328212.- TTGACGTAAGTGTCCCAGGAGACACCACGCTCCCGGGGTTAGGGATGGAGGGGTGTGGGA
SRR935201.293791015.+ TTGACGTAAGTGTCCCAGGAGACACCACGCTCCCGGGGTTAGGGATGGAGGGGTGTGGGA
SRR935201.343780495.- TTGACGTAAGTGTCCCAGGAGACACCACGCTCCCGGGGTTAGGGATGGAGGGGTGTGGGA
SRR935201.441800006.+ TTGACGTAAGTGTCCCAGGAGACACCACGCTCCCGGGGTTAGGGATGGAGGGGTGTGGGA
SRR935201.477791685.+ TTGACGTAAGTGTCCCAGGAGACACCACGCTCCCGGGGTTAGGGATGGAGGGGTGTGGGA
                      ____________________________________________________________
consensus             TTGACGTAAGTGTCCCAGGAGACACCACGCTCCCGGGGTTAGGGATGGAGGGGTGTGGGA

                          .    :    .    :    .    :    .    :    .    :    .    :
SRR935201.373005701.+ AGGTACT
SRR935201.495010717.+ AGGTACT
SRR935201.2236430.1+  GGGGACTGGACGTG
SRR935201.15553217.1+ GGGTACTGGACGTG
SRR935201.444328212.- GGGTACTGGACGTGGC
SRR935201.293791015.+ AGGTACTGGACGTGGCCTGAAAA
SRR935201.343780495.- GGGTACTGGACGTGGCCTGAAAAG
SRR935201.441800006.+ AGGTACTGGACGTGGCCTGAAAAGAGAA
SRR935201.477791685.+ GGGTACTGGACGTGGCCTGAAAAGAGAA
                      ____________________________________________________________
consensus             GGGTACTGGACGTGGCCTGAAAAGAGAA
```

(13) The exon 13 coding sequence was predicted by assembling WGS data using CAP3. The exon 13 is on the reverse strand and marked in red.

```
                          .    :    .    :    .    :    .    :    .    :    .    :
SRR935201.306142695.- GATAAGGGGAGGCCTGAGCAGGGAGGGGCCTGAGTATGTGACATGAGGAGACGGTATCAG
SRR935201.375616248.-   TAAGGGGAGGCCTGAGCAGGGAGGGGCCTGAGTATGTGACATGAGGAGACGGTATCAG
SRR935201.562527020.-   TAAGGGGAGGCCTGAGCAGGGAGGGGCCTGAGTATGTGACATGAGGAGACGGTATCAG
SRR935201.545369629.-   TAAGGGGAGGCCTGAGCAGGGAGGGGCCTGAGTATGTGACATGAGGAGACGGTATCAG
SRR935201.425527244.-   TAAGGGGAGGCCTGAGCAGGGAGGGGCCTGAGTATGTGACATGAGGAGACGGTATCAG
SRR935201.421427599.-   TAAGGGGAGGCCTGAGCAGGGAGGGGCCTGAGTATGTGACATGAGGAGACGGTATCAG
SRR935201.564892068.-   TAAGGGGAGGCCTGAGCAGGGAGGGGCCTGAGTATGTGACATGAGGAGACGGTATCAG
SRR935201.129484088.+        GGAGGCCTGAGCAGGGAGGGGCCTGAGTATGTGACATGAGGAGACGGTATCAG
SRR935201.505038823.-             CCTGAGCAGGGAGGGGCCTGAGTATGTGACATGAGGAGACGGTATCAG
SRR935201.329246503.+                        AGGGGCCTGAGTATGTGACATGAGGAGACGGTATCAG
SRR935201.225701595.+                                 AGTATGTGACATGAGGAGACGGTATCAG
SRR935201.306772253.+                                 AGTATGTGACATGAGGAGACGGTATCAG
SRR935201.524545761.+                                 AGTATGTGACATGAGGAGACGGTATCAG
SRR935201.283276181.+                                    ATGTGACATGAGGAGACGGTATCAG
SRR935201.393625307.-                                       TGACATGAGGAGACGGTATCAG
SRR935201.77518185.1+                                               GGAGACGGTATCAG
SRR935201.397142593.+                                               GGAGACGGTATCAG
SRR935201.222363759.+                                                GAGACGGTATCAG
                      ____________________________________________________________
consensus             GATAAGGGGAGGCCTGAGCAGGGAGGGGCCTGAGTATGTGACATGAGGAGACGGTATCAG

                          .    :    .    :    .    :    .    :    .    :    .    :
SRR935201.306142695.- GCTCCAGCCCGCAGGGTGGCTAGGAGCCAAGACACGGGCC
SRR935201.375616248.- GCTCCAGCCCGCAGGGTGGCTAGGAGCCAAGACACGGGCCCC
SRR935201.562527020.- GCTCCAGCCCGCAGGGTGGCTAGGAGCCAAGACACGGGCCCC
SRR935201.545369629.- GCTCCAGCCCGCAGGGTGGCTAGGAGCCAAGACACGGGCCCC
SRR935201.425527244.- GCTCCAGCCCGCAGGGTGGCTAGGAGCCAAGACACGGGCCCC
SRR935201.421427599.- GCTCCAGCCCGCAGGGTGGCTAGGAGCCAAGACACGGGCCCC
SRR935201.564892068.- GCTCCAGCCCGCAGGGTGGCTAGGAGCCAAGACACGGGCCCC
SRR935201.129484088.+ GCTCCAGCCCGCAGGGTGGCTAGGAGCCAAGACACGGGGCCCCTGAC
SRR935201.505038823.- GCTCCCGCCCGCAGGGTGGCTAGGAGCCAAGACACGGGCCCCCTGACCAAAC
SRR935201.329246503.+ GCTCCAGCCCGCAGGGTGGCTAGGAGCCAAGACACGGGCCCCCTGACCAAACAAGAGGGC
SRR935201.225701595.+ GCTCCAGCCCGCAGGGTGGCTAGGAGCCAAGACACGGGCCCCCTGACCAAACAAGAGGGC
SRR935201.306772253.+ GCTCCAGCCCGCAGGGTGGCTAGGAGCCAAGACACGGGCCCCCTGACCAAACAAGAGGGC
SRR935201.524545761.+ GCTCCAGCCCGCAGGGTGGCTAGGAGCCAAGACACGGGCCCCCTGACCAAACAAGAGGGC
SRR935201.283276181.+ GCTCCAGCCCGCAGGGTGGCTAGGAGCCAAGACACGGGCCCCCTGCCCAAACAAGAGGGC
SRR935201.393625307.- GCTCCAGCCCGCAGGGTGGCTAGGAGCCAAGACACGGGCCCCCTGACCAAACAAGAGGGC
SRR935201.77518185.1+ GCTCCAGCCCGCAGGGTGGCTAGGAGCCAAGACACGGGCCCCCTGACCAAACAAGAGGGC
SRR935201.397142593.+ GCTCCAGCCCGCAGGGTGGCTAGGAGCCAAGACACGGGCCCCCTGACCAAACAAGAGGGC
SRR935201.222363759.+ GCTCCAGCCCGCAGGGTGGCTAGGAGCCAAGACACGGGCCCCCTGACCAAACAAGAGGGC
SRR935201.200366302.+    CCAGCCCGCAGGGTGGCTAGGAGCCAAGACACGGGCCCCCTGGCCAAACAAGAGGGC
SRR935201.513020269.+                                                          GGC
SRR935201.418000868.-                                                         GGGC
SRR935201.113694267.-                                                         GGGC
SRR935201.102093471.+                               GACACGGGCCCCCTGACCAAACAAGAGGGC
SRR935201.154768927.+                  GGCTAGGAGCCAAGACACGGGCCCCCTGACCAAACAAGAGGGC
SRR935201.239816510.+                          GCCAAGACACGGGCCCCCTGACCAAACAAGAGGGC
SRR935201.253514129.+                   GCTAGGAGCCAAGACACGGGCCCCCTGACCAAACAAGAGGGC
SRR935201.314478064.-                                               CCAAACAAGAGGGC
SRR935201.424680749.-                                                            C
```

```
SRR935201.374945015.-                                                            C
SRR935201.18675173.1+                          GCCAAGACACGGGCCCCCTGACCAAACAAGAGGGC
SRR935201.234469652.+                  GGCTAGGAGCCAAGACACGGGCCCCCTGACCAAACAAGAGGGC
SRR935201.67281580.2-                                                            C
SRR935201.9276802.2-                                                             C
SRR935201.454523064.-                                                            C
SRR935201.524574091.-                                                         GGGC
SRR935201.168742574.+       GCCCGCAGGGTGGCTAGGAGCCAAGACACGGGCCCCCTGACCAAACAAGAGGGC
SRR935201.552953233.+       GCCCGCAGGGTGGCTAGGAGCCAAGACACGGGCCCCCTGACCAAACAAGAGGGC

consensus             GCTCCAGCCCGCAGGGTGGCTAGGAGCCAAGACACGGGCCCCCTGACCAAACAAGAGGGC

                          .    :    .    :    .    :    .    :    .    :    .    :
SRR935201.329246503.+ AGT
SRR935201.225701595.+ AGTCAGGGGGAG
SRR935201.306772253.+ AGTCAGGGGGAG
SRR935201.524545761.+ AGTCAGGGGGAG
SRR935201.283276181.+ AGTCAGGGGGAGCCG
SRR935201.393625307.- AGTCAGGGGGAGGCGCCT
SRR935201.77518185.1+ AGTCAGGGGGAGGCGCCTTGGTGTGG
SRR935201.397142593.+ AGTCAGGGGGAGGGGCCTTGGGGGGG
SRR935201.222363759.+ AGTCAGGGGGAGGCGCCTTGGTGTGGC
SRR935201.200366302.+ AGTCCGGGGGAGCCGCCGTGGAGTGGCAGTGCTGGAGCCCGAG
SRR935201.513020269.+ AGTCAGTGGGAGCCGCCTTGCTGTGGCAGTGCTGGAGCCCGAGAGGGCCATGGGGGCCGC
SRR935201.418000868.- AGTCAGGGGGAGCCGCCTTGGTGTGGCAGTGCTGGAGCCCGAGAGGGCCATGGGGGCAGC
SRR935201.113694267.- AGGCAGGGGGAGCCTCCTTGGTGTGGCAGTGCTGGAGCCCGAGAGGGCCATGGGGGCCGC
SRR935201.102093471.+ AGTCAGGGGGAGGCGCCTTGGTGTGGCAGTGCTGGAGCCCGAGAGGGCCATGGGGGCCGC
SRR935201.154768927.+ AGTCAGGGGGAGCCGCCCTGGTGTGGCAGTGCTGGAGCCCGAGAGGGCCATGGGGGC
SRR935201.239816510.+ AGTCAGGGGGAGGCGCCTTGGTGTGGCAGTGCTGGAGCCCGAGAGGGCCATGGGGGCCGC
SRR935201.253514129.+ AGTCAGGGGGAGGCGCCTTGGTGTGGCAGTGCTGGAGCCCGAGAGGGCCATGGGGGCC
SRR935201.314478064.- AGTCAGGGGGAGGCGCCTTGGTGTGGCAGTGCTGGAGCCCGAGAGGGCCATGGGGGCCGC
SRR935201.424680749.- AGTCAGGGGGAGCCGCCTTGGTGTGGCAGTGCTGGAACCCGAGAGGGCCATGGGGGCCGC
SRR935201.374945015.- AGTCAGGGGGAGGCGCCTTGGTGTGGCAGTGCTGGAGCCCGAGAGGGCCATGGGGGCCGC
SRR935201.18675173.1+ AGTCAGGGGGAGCCGCCTTGGTGTGGCAGTGCTGGAGCCCGAGAGGGCCATGGGGGCCGC
SRR935201.234469652.+ AGTCAGGGGGAGCCGCCTTGGTGTGGCAGTGCTGGAGCCCGAGAGGGCCATGGGGGC
SRR935201.322935779.-                CCTTGGTGTGGCAGTGCTGGAGCCCGAGAGGGCCATGGGGGCCGC
SRR935201.67281580.2- AGTCAGGGGGAGCCGCCTTGGTGTGGCAGTGCTGGAGCCCGAGAGGGCCATGGGGGCCGC
SRR935201.9276802.2-  AGTCAGGGGGAGCCGCCTTGGTGTGGCAGTGCTGGAGCCCGAGAGGGCCATGGGGGCCGC
SRR935201.454523064.- AGTCAGGGGGAGCCGCCTTGGTGTGGCAGTGCTGGAGCCCGAGAGGGCCATGGGGGCCGC
SRR935201.524574091.- AGTCAGGGGGAGCCGCCTTGGTGTGGCAGTGCTGGAGCCCGAGAGGGCCATGGGGGCCGC
SRR935201.168742574.+ AGTCAGGGGGAGCCGCCTTGGTGTGGCAGTGCTGGAGCCCGAGAGG
SRR935201.552953233.+ AGTCAGGGGGAGCCGCCTTGGTGTGGCAGTGCTGGAGCCCGAGAGG

consensus             AGTCAGGGGGAGCCGCCTTGGTGTGGCAGTGCTGGAGCCCGAGAGGGCCATGGGGGCCGC

                          .    :    .    :    .    :    .    :    .    :    .    :
SRR935201.513020269.+ CCTGAAGCCTGCAGGGGGTCTGCCCTGGTTCTGTCCA
SRR935201.418000868.- CCTGAAGCCTGCAGGGGTTCTGTACTGGTTCTGTAC
SRR935201.113694267.- CCTGAAGCCTGCAGGGGTTCTGTCCTGGTTCTGTCC
SRR935201.102093471.+ CCTGAAGCCT
SRR935201.239816510.+ CCTGA
SRR935201.314478064.- CCTGAAGCCTGCAGGGGTTCTGTCCT
SRR935201.424680749.- CCTGAAGCCTGCAGGGGTTCTGTCCTGGTTCTGTCCACC
SRR935201.374945015.- CCTGAAGCCTGCAGGGGTTCTGTCCTGGTTCTGTCCACC
SRR935201.18675173.1+ CCTGA
SRR935201.322935779.- CCTGAAGCCTGCAGGGGTTCTGTCCTGGTTCTGTCCACCAGGAGGAGGCCTCTGG
SRR935201.67281580.2- CCTGAAGCCTGCAGGGGTTCTGTCCTGGTTCTGTCCACC
SRR935201.9276802.2-  CCTGAAGCCTGCAGGGGTTCTGTCCTGGTTCTGTCCACC
SRR935201.454523064.- CCTGAAGCCTGCAGGGGTTCTGTCCTGGTTCTGTCCACC
SRR935201.524574091.- CCTGAAGCCTGCAGGGGTTCTGTCCTGGTTCTGTCC
SRR935201.41279653.1+                               CTGTCCACCAGGAGGAGGCCTCTGGAGGAC
SRR935201.121862708.+                               CTGTCCACCAGGAGGAGGCCTCTGGAGGAC
SRR935201.231343747.+                                         GGAGGAGGCCTCTGGAGGAC
SRR935201.514074079.+                                          GAGGAGGCCTCTGGAGGAC
SRR935201.405966170.+                                            GGAGGCCTCTGGAGGAC
SRR935201.245647471.-                                                  CTCTGGAGGAC
SRR935201.14572050.2+                                                      GGAGGAC
SRR935201.265705458.+                                                      GGAGGAC
SRR935201.394415951.+                                                       GAGGAC

consensus             CCTGAAGCCTGCAGGGGTTCTGTCCTGGTTCTGTCCACCAGGAGGAGGCCTCTGGAGGAC

                          .    :    .    :    .    :    .    :    .    :    .    :
SRR935201.41279653.1+ TCCAAGCAAGGACCCCGGGGAGCAGGGATGATTTCCAGAATCCAGCCTGTTGTGTTTTCC
SRR935201.121862708.+ TCCAAGCAAGGACCCCGGGGAGCAGGGATGATTTCCAGAATCCAGCCTGTTGTGTTTTCC
SRR935201.231343747.+ TCCAAGCAAGGACCCCGGGGAGCAGGGATGATTTCCGGAATCCAGCCTGTTGTGTTTTCC
SRR935201.514074079.+ TCCAAGCAAGGACCCCGGGGAGCAGGGATGATTTCCAGAATCCAGCCTGTTGTGTTTTCC
```

```
SRR935201.405966170.+ TCCAAGCAAGGACCCCGGGGAGCAGGGATGATTTCCGGAATCCAGCCTGTTGTGTTTTCC
SRR935201.245647471.- TCCAAGCAAGGACCCCGGGGAGCAGGGATGATTTCCAGAATCCAGCCTGTTGTGTTTTCC
SRR935201.14572050.2+ TCCAAGCAAGGACCCCGGGGAGCAGGGATGATTTCCAGAATCCAGCCTGTTGTGTTTTCC
SRR935201.265705458.+ TCCAAGCAAGGACCCCGGGGAGCAGGGATGATTTCCAGAATCCAGCCTGTTGTGTTTTCC
SRR935201.394415951.+ TCCAAGCAAGGACCCCGGGGAGCAGGGATGATTTCCGGAATCCAGCCTGTTGTGTTTTCC
SRR935201.71546215.1-   CAAGCAAGGACCCCGGGGAGCAGGGATGATTTCCAGAATCCAGCCTGTTGTGTTTTCC
SRR935201.2236430.2+      AGCAAGGACCCCGGGGAGCAGGGATGATTTCCGGAATCCAGCCTGTTGTGTTTTCC
SRR935201.15553217.2+     AGCAAGGACCCCGGGGAGCAGGGATGATTTCCGGAATCCAGCCTGTTGTGTTTTCC
SRR935201.314284053.+     AGCAAGGACCCCGGGGAGCAGGGATGATTTCCGGAATCCAGCCTGTTGTGTTTTCC
SRR935201.561047431.+             CCCCGGGGAGCAGGGATGATTTCCGGAATCCAGCCTGTTGTGTTTTTC
SRR935201.19276136.1+                   GGAGCAGGGATGATTTCCGGAATCCAGCCTGTTGTGTTTTCC
SRR935201.477791685.+                         GGGATGATTTCCGGAATCCAGCCTGTTGTGTTTTCC
SRR935201.200366302.-                            ATGATTTCCAGAATCCAGCCTGTTGTGTTTTCC
SRR935201.329246503.-                                    CAGAATCCAGCCTGTTGTGTTTTCC
SRR935201.129484088.-                                       AATCCAGCCTGTTGTGTTTTCC
SRR935201.524545761.-                                                    TTGTCTTCC
                      ____________________________________________________________
consensus             TCCAAGCAAGGACCCCGGGGAGCAGGGATGATTTCCAGAATCCAGCCTGTTGTGTTTTCC

                          .    :    .    :    .    :    .    :    .    :    .    :
SRR935201.41279653.1+ ACTAGCTCCT
SRR935201.121862708.+ ACTAGCTCCT
SRR935201.231343747.+ ACTAGCTCCTGCAGGAGAAG
SRR935201.514074079.+ ACTAGCTCCTGCAGGAGAAGG
SRR935201.405966170.+ ACTAGCTCCTGCAGGAGAAGGGA
SRR935201.245647471.- ACTAGCTCCTGCAGGAGAAGGGAGAACAT
SRR935201.14572050.2+ ACTAGCTCCTGCAGGAGAAGGGAGAACATGGGT
SRR935201.265705458.+ ACTAGCTCCTGCAGGAGAAGGGAGAACATGGGT
SRR935201.394415951.+ ACTAGCTCCTGCAGGAGAAGGGAGAACATGGGTG
SRR935201.71546215.1- ACTAGCTCCTGCAGGAGAAGGGAGAACATGGGTGAAGGTGGA
SRR935201.2236430.2+  ACTAGCTCCTGCAGGAGAAGGGAGAACATGGGTGAAGGTGGAAG
SRR935201.15553217.2+ ACTAGCTCCTGCAGGAGAAGGGAGAACATGGGTGAAGGTGGAAG
SRR935201.314284053.+ ACTAGCTCCTGCAGGAGAAGGGAGAACATGGGTGAAGGTGGAAG
SRR935201.561047431.+ ACTAGCTCCTGCAGGAGAAGGGAGAACATGGGTGAAGGTGGAAGCATCCCCA
SRR935201.19276136.1+ ACTAGCTCCTGCAGGAGAAGGGAGAACATGGGTGAAGGTGGAAGCATCCCCACCTCCT
SRR935201.477791685.+ ACTAGCTCCTGCAGGAGAAGGGAGAACATGGGTGAAGGTGGAAGCATCCCCACCTCCTTT
SRR935201.200366302.- ACTAGCTCCTGCAGGAGAAGGGAGAACATGGGTGAAGGTGGAAGCATCCCCACCTCCTTT
SRR935201.329246503.- ACTAGCTCCTGCAGGAGAAGGGAGAACATGGGTGAAGGTGGAAGCATCCCCACCTCCTTT
SRR935201.129484088.- ACTAGCTCCTGCAGGAGAAGGGAGAACATGGGTGAAGGTGGAAGCATCCCCACCTCCTTT
SRR935201.524545761.- ACTAGCTCCTGCAGGAGAAGGGAGAACGTTGGTGAAGGTGGAAGCATCCCCACCTCCTTT
                      ____________________________________________________________
consensus             ACTAGCTCCTGCAGGAGAAGGGAGAACATGGGTGAAGGTGGAAGCATCCCCACCTCCTTT

                          .    :    .    :    .    :    .    :    .    :    .    :
SRR935201.477791685.+ CCTC
SRR935201.200366302.- CCTCTCC
SRR935201.329246503.- CCTCTCCTAAGACTT
SRR935201.129484088.- CCTCTCCTAAGACTTTAC
SRR935201.524545761.- CCTCTCCTAAGACTTTACTCGGTAATCCCCA
                      ____________________________________________________________
consensus             CCTCTCCTAAGACTTTACTCGGTAATCCCCA
```

56. Western European hedgehog (*Erinaceus europaeus*)

```
>Erinaceus_europaeus No=56 length=1854 name="Western European hedgehog"
ATGGCCTGTGCCCTCTTCATCGGGGTGCTGCTCCTCATGGTGGTCCTGGCAGCTCCCACCCAGGGCAGGCTCCTGGGCCCCTCATCCCCCCTGCGTTACT
CCCGTTTCCTAGACCCTTCCAATGCCATGTTCCTGCGCTGGGACTTTGACCTGGATAGCGAGATCATTACTTTTGAGCTGAAGGTCCGGACAGCTGGCTG
GGTGGGCTTGGGTGTTACCAATCGCTATACCAGGGCAGGCAGTGACCTGGTGGTTGGGGGCGTCCTACCAGATGGCAATGTCTACTTCTCGGATTGGCAC
CTGCTGGATGAAGACACCATGGAGGAGGACTCCAGCCAGGATGCTGAGCTGCAGGGGCTGATGGAAGATGCTATCTACACCACCATGCGATTCTCCAGGC
CCCTCCGTTCCTGTGACCCTCATGACATGGACATTGCGGGAGACACGGTCAGGGTGCTGGCCGCCTACGGACTGGATGACACGCCAAAGCTGGATCGAGA
GCGGACCTTCGTCAAGTCCATCTTCCTGCTGCAGATAGTCCATCCCGACGAACTCGAGGTCCCCGAGGACACCATCATCCACGACCTGGAGCTCACAAAT
TTCCTCATTCCAGAAGATGACACCACCTATGCCTGCACCTTCCTCCCTCTCCCCATCGTTAGCCAGAAGCACCACATCTACAAGTTTGAGCCCAAGTTGG
TAGAGCACAACGAGACGATGGTGCACCACATCCTGGTCTACGCCTGTGGCAACGCCAGTGTCCTCCCCACGGGGGTCGACGACTGCTATGGGGCCAACCC
CTCCTTCTCCCTCTGCTCACAGGTCATCGTGGGCTGGGCTGTCGGGGGCACTAGTTACCAGTTTCCAGACGATGTGGGTATCTCCATCGGGATGCCCCTA
GACCCTCAGTGGATCCGACTAGAAATTCATTACAGCAATTTCCGCAACCTTGCTGGCATATATGACTCCTCGGGCATCCGCGTGTACTACACAGCGCGCC
TGCGTAAATATGACATGGGTGTCCTGCAGCTGGGCTTCTTCACCTTCCCCATCCACTTCCTACCCCCTGGCGCGGAGTCCTACATGTCCTACGGGTTGTG
CAAGACGGAGAAGTTTGAGGAGATAAATGGGGCCCCGGTGCCTGACATACAGGTTTACGGCTACCTGCTCCACACACACTTGACTGGGCGAGCTCTGAAG
GCTGTGCAGTACAGAAATGGAACACAACTCCGAACAATCTGTAAAGATGATTTCTATGACTTCAATCTGCAGGAGACAAGAGACTTGCCTCATCGTGTGG
AGATTAAACCGGGAGATGAGTTGCTGGTTGAGTGTCACTACCAGACGCTGGACCGAGATTCAATGACTTTTGGAGGACCCAGCACTACTAATGAGATGTG
CCTCATCTTCCTCTTCTATTATCCCAGAAACAACATCTCTAGTTGCATGGGGTACCCTGACATCATCTCCGTAGCCCATGAGCTGGGGGAGGATGCAATG
GATCCCATGGATGCTGTGATGGCCCTGAACAACATTGACTGGACCCCAGAGAACATTAAGATGGCCGAGAAAGCCTGCAAGGAGGCCCAGCAGACCGTGA
TCATAAAGACCATTGATGAGTTTGTGGAAAATACAACAGGCTGGATTCAAGACATCACCCCTACACCCAGGGGAGTCTGCTTAGAGTCCATAGGAGGCAA
GGTGGAGCCCCTGGACAAAATTCCCGCTGGCTTCAGGTCGGCCCCTGCAGCCCTCAGGGGCTCAGGAATGATCCCTCCACAGCACTTCTCCCTGGCCTCC
CTCTTGTTTGCCCAGGGTGCCCTATACTGGCTCCTTGCCACACTTCAGGCTGGTATATGA

>Erinaceus_europaeus No=56 length=617 name="Western European hedgehog"
MACALFIGVLLLMVVLAAPTQGRLLGPSSPLRYSRFLDPSNAMFLRWDFDLDSEIITFELKVRTAGWVGLGVTNRYTRAGSDLVVGGVLPDGNVYFSDWH
LLDEDTMEEDSSQDAELQGLMEDAIYTTMRFSRPLRSCDPHDMDIAGDTVRVLAAYGLDDTPKLDRERTFVKSIFLLQIVHPDELEVPEDTIIHDLELTN
FLIPEDDTTYACTFLPLPIVSQKHHIYKFEPKLVEHNETMVHHILVYACGNASVLPTGVDDCYGANPSFSLCSQVIVGWAVGGTSYQFPDDVGISIGMPL
DPQWIRLEIHYSNFRNLAGIYDSSGIRVYYTARLRKYDMGVLQLGFFTFPIHFLPPGAESYMSYGLCKTEKFEEINGAPVPDIQVYGYLLHTHLTGRALK
AVQYRNGTQLRTICKDDFYDFNLQETRDLPHRVEIKPGDELLVECHYQTLDRDSMTFGGPSTTNEMCLIFLFYYPRNNISSCMGYPDIISVAHELGEDAM
DPMDAVMALNNIDWTPENIKMAEKACKEAQQTVIIKTIDEFVENTTGWIQDITPTPRGVCLESIGGKVEPLDKIPAGFRSAPAALRGSGMIPPQHFSLAS
LLFAQGALYWLLATLQAGI
```

(1) Exon coordinates

Exon	NCBI Accession	Strand	Start	End
1	AMDU01081675	-	10668	10958
2	AMDU01081675	-	9191	9337
3	AMDU01081675	-	8139	8300
4	AMDU01081675	-	7827	7910
5	AMDU01081675	-	5056	5223
6	AMDU01081675	-	4775	4877
7	AMDU01081675	-	3758	3924
8	AMDU01081675	-	2780	2871
9	AMDU01081675	-	1610	1706
10	AMDU01081675	-	1413	1472
11	AMDU01081675	-	96	226
12	AMDU01081674	-	5312	5432
13	AMDU01081674	-	4487	4729

57. African bush elephant (*Loxodonta africana*)

>Loxodonta_africana No=57 length=1857 name="African bush elephant"
ATGACGTGTACCCTCTTCTCCTGGCTAATCCTGCTCCTGGCCCTGACCACCCCATCCCAAGGCCACCACTTCGGCCCAAAATCTCGCCTGCGATATTCCA
GGTTCCTAGATCCGTCCAATGTCATGTTCCTGTGCTGGGACTTTGACCTTGAGGCTGAGGTCATCACGTTTGAGCTGCAGGTCCGGACAGCTGGCTGGGT
GGGCCTGGGTGTCACGAATCGCTACACCAACGTGGGAGGCGATCTGGTGGTTGGTGGGGTCTTGCCTGACGGCAACGTCTATTTCTCGGATCAGCATCTG
GTGGACGAAGATACCCTGAAGGAGGACGGGAGTCAGGATGCTGAGCTGCAGGGGCTCATGGAAGATGCCTTCTACACCACGATGCGTTTCTCCCGGCCCT
TCCGCTCCTGCGACCCTCATGACCAGGACATCACGAGTGACACAGTGCGGGTGCTAGCTGCATATGGCCAAGATGATACCCTGAAGCTGGACCGGGAGCA
CATCTTTGTCAAGTCCATCTTCCTGCTGCAGATTGTCCACCCCGATGATCTTGATGTCCCCGAGGACACCATTATCCATGACCTGGAGATCACTGATTTC
CTCATTCCAGAAGATGACACCACCTATGCCTGCACCTTCCTCCCTCTGCCCATCGTGAGCAAGAAGCACCACATCTACAAGTTTGAGCCCAAGCTGGCCC
ACCGCAATGAAACCATGGTGCACCACATCCTGGTGTATGCCTGCGGCAACGCTAGCACCCTCCCCACCGGCATCAGCGACTGCTACGGGGCAGACCCGGC
TTTCTCGCTCTGCTCCCAGGTCGTCGTGGGCTGGGCCGTGGGAGGCACCAGCTACCAGTTCCCGGATGATGTAGGCATCTCTATTGGGACCCCCATGGAC
CCCCAGTGGATCCGGCTGGAGATTCATTACAGCAATTTCCACAACCTCCCTGGCATCTATGACTCCTCCGGGATCCGCATGTACTACAGCCCGCGGCTGC
GCAGATACGACATGGGCGTCCTGCAGCTGGGCTTCTTTACTTTCCCCATCCACTTCATCCCCCCGGGCACCCAGTCCTTCATGTCTTACGGGCTGTGCAA
GACGGAGAAGTTCGAGGAGATGAACGGGACTCCGGTGCCCGACATACAGGTGTATGGCTACCTGCTCCACACCCACCTGGCAGGCCGGGCTCTGCAGGCC
GTGCAGTACAGAAATGGATCCCAGCTCCGAACCATCTGTAGAGATGGCTCCTATGACTTCAGCCTGCAGGAGACTCGAGATTTACCTGCACGAGTGGAGA
TCAAGCCGGGAGACGAACTGCTGGTGGAGTGTCAGTACCAGACGCTGGACCGAGACTCCTTGACTTTTGGGGGACCCAGCACCATCAATGAGATGTGCCT
CATCTTCCTTTTCTACTACCCCCGGAACAACATCTCCAGCTGCATGGGGTATCCTGACATCATCTACGTGGCCCACGAGCTGGGGGAGGAGGCATCTGAC
TCCATGGAGGGCATGATGGCCATGAATAATGTTGAGTGGACCCCAGAGAACATTAAGAAGGCAGAGAAAGCCTGCAAGGAGGCCCAGCAGACGGTGATCA
TAAAGACGATTGACGAGGTGGTGGAGAACACCACAGGTTGGATCCCTGACATCATCCCTACTCCCCGAGGACCCTGCTTGGAGTCTACTGGGGGCAAGGT
AGAGCCCCATGACAAGACCCCTGCAGGCTTCAGGGCCAAACCCCTGTCCCTCTCGGGAGCCAGCACGGCTTCCCCAGGGAGCCTCCCCCTGGCCACCCTC
CTGTTTACACAGGGGACCCTCTCATTGTTCTTAGCTACACTGCAAGCTAGAGACTGA

>Loxodonta_africana No=57 length=618 name="African bush elephant"
MTCTLFSWLILLLALTTPSQGHHFGPKSRLRYSRFLDPSNVMFLCWDFDLEAEVITFELQVRTAGWVGLGVTNRYTNVGGDLVVGGVLPDGNVYFSDQHL
VDEDTLKEDGSQDAELQGLMEDAFYTTMRFSRPFRSCDPHDQDITSDTVRVLAAYGQDDTLKLDREHIFVKSIFLLQIVHPDDLDVPEDTIIHDLEITDF
LIPEDDTTYACTFLPLPIVSKKHHIYKFEPKLAHRNETMVHHILVYACGNASTLPTGISDCYGADPAFSLCSQVVVGWAVGGTSYQFPDDVGISIGTPMD
PQWIRLEIHYSNFHNLPGIYDSSGIRMYYSPRLRRYDMGVLQLGFFTFPIHFIPPGTQSFMSYGLCKTEKFEEMNGTPVPDIQVYGYLLHTHLAGRALQA
VQYRNGSQLRTICRDGSYDFSLQETRDLPARVEIKPGDELLVECQYQTLDRDSLTFGGPSTINEMCLIFLFYYPRNNISSCMGYPDIIYVAHELGEEASD
SMEGMMAMNNVEWTPENIKKAEKACKEAQQTVIIKTIDEVVENTTGWIPDIIPTPRGPCLESTGGKVEPHDKTPAGFRAKPLSLSGASTASPGSLPLATL
LFTQGTLSLFLATLQARD

(1) Exon coordinates

Exon	NCBI Accession	Strand	Start	End
1	AAGU03080315	+	56614	56901
2	AAGU03080315	+	58014	58160
3	AAGU03080315	+	58679	58840
4	AAGU03080315	+	59088	59171
5	AAGU03080315	+	60285	60452
6	AAGU03080315	+	60590	60692
7	AAGU03080315	+	60845	61011
8	AAGU03080315	+	61281	61372
9	AAGU03080315	+	61662	61758
10	AAGU03080315	+	61974	62033
11	AAGU03080315	+	63686	63816
12	AAGU03080315	+	64511	64625
13	AAGU03080315	+	64859	65101

58. Florida manatee (*Trichechus manatus latirostris*)

>Trichechus_manatus_latirostris No=58 length=1857 name="Florida manatee"
ATGACCTGTGCCCTCCTCTCCCGGCTGCTGCTGCTCCTGGCCCTGGCCGCCCCCTCCCAAGGCCACCACTTGGGCCCCAAGTCTCGCCTGCGCTATTCCA
GGTTCCTAGATCCAGCCAATGTCATGTTCCTGCGCTGGGACTTTGACCTGGAGGCCGAGGTCATCATGTTTGAGCTGCAGGTCCAGACGGCTGGCTGGGT
GGGCCTGGGTGTCACGAATCGCTACACCAACGTGGGAGGCGATCTGGTGGTTGGCGGGGTCTTGCCTGATGGCAACGTCTATTTCTCGGATCAGCACCTG
GTGGATGAAGACACCCTGACGGCGGACGGGAGCCAGGATGCCGAGCTGCAGGGGCTCACAGAAGATGCTGTCTACACCACCATGCGTTTCTCCCGGCCCT
TCCGTTCCTGTGACCATCATGACCAGGATATCACGAGTGACACAGTGCGTGTGCTAGCTGCCTATGGCCAGGATGATACCTTGAAGTTGGACCGGGAGCA
CATCTTTGTCAAGTCCATCTTCCTGCTACAGATTATCCACCCCGATGATCTTGATGTCCCCGAGGACACCATTATCCATGACCTGGAGATCACTGACTTC
CTCATTCCAGAAGATGACACCACCTACGCCTGCACCTTCCTCCCTCTGCCCATCGTGAGTGAGAAGCACCACATCTACAAGTTCGAGCCCAAGCTGGCCC
ACCACAATGAGACCATGGTGCACCACATCCTGGTGTATGCCTGCGGCAATGCCAGCGCCCTCCCCACCGGCATCAGCGACTGCTATGGTGCTGACCCGGC
CTTCTCGCTCTGCTCGCAGGTTGTCCTAGGCTGGGCCTTGGGCGGCACTAGCTACCAGTTCCCGGATGATGTAGGCATATCTATCGGGACCTCCATGGAC
CCCCAGTGGATCCGGCTGGAGATTCATTACAGCAATTTCTACAACCTCCCTGGCATCTACGACTCCTCTGGGATCCGCATGTACTACACCCCGCGGCTGC
GCAGACACGACATGGGCGTCCTGCAGCTGGGCATCTTCACCTTCCCCATTCACTTCATACCCCCAGGCGCCCAGTCCTTCATATCTTACGGGCTGTGCAA
GACGGAGAAGTTCGAGGAGATGAACGGGGCCCCGTTGCCCGACATACAAGTGTATGGCTACTTGCTCCACACCCACCTGACAGGCCGGGCTCTGCAGGCC
GTGCAGTACAGAAATGGTTCCCAGCTCCGAACCATCTGTAAAGATGACTCCTATGACTTCAACCTGCAGGAGACTCGGGATTTACCTGCTCGCGTGGAGA
TCAAGCCTGGAGACGAGCTGCTGGTGGAGTGTCACTACCAGACGCTGGACCGAGACTTCTTGACTTTTGCGGGGCCCAGCACGGTCAATGAAATGTGCCT
CATCTTCCTTTTCTACTACCCCCGGAACAACATCTCCAGCTGCATGGGGTATCCCGACATCGTCTCTGTGGCCCGTGAGCTGGAGGAGGAGGTGTCTGAC
TCCATGGAGGCCATGATGGCCATGAACAATGTCGAGTGGACCCCAGAGAACATTAAGAAGGCAGAGAAAGCCTGCAAGGAGGCCGAGCAAACGGTGATCA
TAAAGACGATTGACGAGGTGGTGGGGAACACCACAGGCTGGATCCCCGACATTGTCCCTATTCCCCGGGGTCCCTGCTTGGAGTCCACCGGGGGCAAGGT
GGAGCCCCACGACAAGAACCCTGTGGGCTTCAGGGCCATCCCCCTGGCCCTCTCGGGTGCCAGCATGGCTGCCCCCAGGATCCTCCCCCTAGTCACCCTC
CTGTTTGCACAGGGGACCCTCTTGTGGCTCTTAGCTTCCCTGCAGAGTAGAGACTGA

>Trichechus_manatus_latirostris No=58 length=618 name="Florida manatee"
MTCALLSRLLLLLALAAPSQGHHLGPKSRLRYSRFLDPANVMFLRWDFDLEAEVIMFELQVQTAGWVGLGVTNRYTNVGGDLVVGGVLPDGNVYFSDQHL
VDEDTLTADGSQDAELQGLTEDAVYTTMRFSRPFRSCDHHDQDITSDTVRVLAAYGQDDTLKLDREHIFVKSIFLLQIIHPDDLDVPEDTIIHDLEITDF
LIPEDDTTYACTFLPLPIVSEKHHIYKFEPKLAHHNETMVHHILVYACGNASALPTGISDCYGADPAFSLCSQVVLGWALGGTSYQFPDDVGISIGTSMD
PQWIRLEIHYSNFYNLPGIYDSSGIRMYYTPRLRRHDMGVLQLGIFTFPIHFIPPGAQSFISYGLCKTEKFEEMNGAPLPDIQVYGYLLHTHLTGRALQA
VQYRNGSQLRTICKDDSYDFNLQETRDLPARVEIKPGDELLVECHYQTLDRDFLTFAGPSTVNEMCLIFLFYYPRNNISSCMGYPDIVSVARELEEEVSD
SMEAMMAMNNVEWTPENIKKAEKACKEAEQTVIIKTIDEVVGNTTGWIPDIVPIPRGPCLESTGGKVEPHDKNPVGFRAIPLALSGASMAAPRILPLVTL
LFAQGTLLWLLASLQSRD

(1) Exon coordinates

Exon	NCBI Accession	Strand	Start	End
1	AHIN01087863	+	1232	1519
2	AHIN01087863	+	2608	2754
3	AHIN01087863	+	3307	3468
4	AHIN01087863	+	3712	3795
5	AHIN01087863	+	4915	5082
6	AHIN01087863	+	5226	5328
7	AHIN01087863	+	5481	5647
8	AHIN01087863	+	5897	5988
9	AHIN01087863	+	6228	6324
10	AHIN01087863	+	6725	6784
11	AHIN01087863	+	8518	8648
12	AHIN01087863	+	9374	9488
13	AHIN01087863	+	9728	9970

59. Cape sengi (elephant shrew) (*Elephantulus edwardii*)

>Elephantulus_edwardii No=59 length=1843 name="Cape sengi (elephant shrew)"
ATGACTGGCGTCCTCCTCTTCCAGCTCGTCTTATTTCTACCCCTGGCAGCCCCTTCCCAAGGCCATCACCATGGCCCTAAGTCTCGCCTGCGCTATTCCA
GGTTCCTAGATTCATCCAATGTCCTGTTCCTGCGTTGGGACTTTGACCTTGAGGCTGAAGTCATCACGTTCGAGCTGAAGGTCCGGACAGCCGGCTGGGT
GGGCCTGGGTGTCACAAATCGCTACACCAACGTAGGTGCAGACCTGGTGGTGGGCGGCGTCTTGCCTAACGGCAACGTCTATTTCTCGGACCAACACCTG
GTGGATGAAGACACCTTGCAGGAGGACGGGAGCCAGGATGCTGAGCTCCAGGGGCTCACGGAAGATGCCGTCTACACCACCATGCGTTTCTCCCGGCCTT
TCCGCTCCTGTGACCCGCATGACCAGGATATCACGAGTGACACCGTGAGGGTCCTGGCAGCCTATGGCCAGGACGACACTCCGAAGCTGGACCGGGAACA
CATCTTCGTCAAGTCCATCTTCCTGCTGCAGATCATCCACCCCGATGACCTCCAAGTCCCCGAGGACACCATTATCCATGACCTGGAGCTCACTAACTTC
CTCATTCCAGAAGATGACACCACTTATGCCTGCACCTTCCTCCCTCTGCCCATCGTGAGCAAGAAACACCACATCCACAAGTTTGAACCCAAGCTGATCT
CCCACAACGAGACCATGGTGCACCACATCCTGGTGTACGCCTGTGGCAACGCCAGCATCCTCCCCACGGGCACCAGCGACTGCTACGGAGCTGACCCGGC
CTTCTCACTCTGCTCACAAGTCATTGTGGGCTGGGCCGTGGGCGGCTTGAGCTACCAGTTCCCGGATGACGTGGGCATCTCTCTCGGAACACCCCTGGAC
CCCCAGTGGATCCGCCTGGAGATTCATTATAGCAACTTCCACAACCTTCCTGGGATCTACGACTCGTCCGGGATCCGCCTGTACTACAGTCCTCAGCTGC
GCAGATACGACATGGGTGTCCTACAGCTGGGCTTCTTCACCTTCCCCATCCACTTCATCCCCCCGGGCGCCCAGTCCTTCATGTCCTACGGGCTGTGCAA
GACGGAGAAGTTTGAGGAGATCAACGGTGCCCCGGTGCCCGATATACAGGTGTATGGCTACCTTCTCCACACCCACCTGGCAGGCCGGGCTCTGAAGGCT
GTGCAGTACAGAAACGGGTCCGAGCTCCGAACCATCTGTAAAGATGACTCCTATGACTTCAACTTGCAGGAAACGCGGGATTTACCTGCTCGCGTGGAGA
TCAAGCCTGGCGATGAACTGCTGGTGGAGTGTCACTACCAAACACTGGACCGAGACTCCCTAACTTTTGGGGGGCCCAGCACAATCAATGAGATGTGCCT
CATCTTCCTCTTCTACTATCCCCGGAACAACATCTCCAGCTGCATGGGGTACCCCGACATTGTCTCCGTGGCTCATGAACTGGGAGAGGAGGCCTCTGAC
TCCATGGAGGGCATGATGGCCATGAACAATGTTGAGTGGACTCCAGAGAACATAAAGAAAGCAGAGAAGGCCTGCAAGGAGGCCCAGCAGACAGTGATCA
TCAAGACTATTGACGAGGTGGTGGAGAACACTACAGGCTGGATCCCCGACATTGCTCCTACATCCAGGGGACCCTGCTTGGAGTCCACAGGGGGCAAGGT
GGAGCCCCAGGACAAGACCCCTGCAGGCTTCAGGGCAGCCACCCCTGTGACCCTCTCTGGCACCAGCACCATTGCCCCCAGGACCCTCCCCTTGGCCACC
CTGCTGATCACACAAGGGGCCCTTTCGTGGCTCCTGGCCCTGA

>Elephantulus_edwardii No=59 length=614 name="Cape sengi (elephant shrew)"
MTGVLLFQLVLFLPLAAPSQGHHHGPKSRLRYSRFLDSSNVLFLRWDFDLEAEVITFELKVRTAGWVGLGVTNRYTNVGADLVVGGVLPNGNVYFSDQHL
VDEDTLQEDGSQDAELQGLTEDAVYTTMRFSRPFRSCDPHDQDITSDTVRVLAAYGQDDTPKLDREHIFVKSIFLLQIIHPDDLQVPEDTIIHDLELTNF
LIPEDDTTYACTFLPLPIVSKKHHIHKFEPKLISHNETMVHHILVYACGNASILPTGTSDCYGADPAFSLCSQVIVGWAVGGLSYQFPDDVGISLGTPLD
PQWIRLEIHYSNFHNLPGIYDSSGIRLYYSPQLRRYDMGVLQLGFFTFPIHFIPPGAQSFMSYGLCKTEKFEEINGAPVPDIQVYGYLLHTHLAGRALKA
VQYRNGSELRTICKDDSYDFNLQETRDLPARVEIKPGDELLVECHYQTLDRDSLTFGGPSTINEMCLIFLFYYPRNNISSCMGYPDIVSVAHELGEEASD
SMEGMMAMNNVEWTPENIKKAEKACKEAQQTVIIKTIDEVVENTTGWIPDIAPTSRGPCLESTGGKVEPQDKTPAGFRAATPVTLSGTSTIAPRTLPLAT
LLITQGALSWLLAL

(1) Exon coordinates

Exon	NCBI Accession	Strand	Start	End
1	AMGZ01099395	-	869	1156
2	AMGZ01099394	-	2200	2346
3	AMGZ01099394	-	1550	1711
4	AMGZ01099394	-	1237	1320
5	AMGZ01099393	-	14381	14548
6	AMGZ01099393	-	14159	14261
7	AMGZ01099393	-	13774	13940
8	AMGZ01099393	-	13431	13522
9	AMGZ01099393	-	13100	13196
10	AMGZ01099393	-	12321	12380
11	AMGZ01099393	-	10616	10746
12	AMGZ01099393	-	9810	9924
13	AMGZ01099393	-	9359	9584

60. Cape golden mole (*Chrysochloris asiatica*)

```
>Chrysochloris_asiatica No=60 length=1851 name="Cape golden mole"
ATGACCCGTGTCCTGCTGTTCTGGCTGCTCCTGATCCTGACTCCCCCTTCCCAAGGCCACCGTCTCGGCCCCAAGTCTCGCTTGCGTTATTCCAGGTTCC
TTGATTCATCTAATGACGTGTTCCTGCGCTGGGACTTTGACCTTGAGGCTGAGGTCATCACATTTGAACTGCAGGTCCAGACAGCTGGCTGGGTTGGCCT
CGGTGTCACAAATCGCTACACCAACGTGGGAGGTGATCTGGTAGTTGGCGGGGTCTTGCCTGGTGGCAACGTCTATTTCTCGGATCAGCACCTGAAGGAT
GAAGACACCCTGGAGGAGGACGGGAGTCAGGATGCGGAGCTGCTGGGACTCACAGAAGACGCTGTCTATACCACCATGCGTTTCTCCAGACCTTTCCGCT
CCTGTGATCCCCACGACCACGACATCACGAGTGACACTGTGCGGGTGTTGGCTGCATATGGCCAGGATGACACGCTGAAGCTGGACCGGGATCACATCTT
TGTCAAGTCCATCTTCCTGCTGCAGATCATCCACCCCGATGACCTTGAAGTCCCTGAGGACACTATTATCCACGACCTGGAGATCACTGATTTCCTCATT
CCAGAAGATGACACCACCTACGCCTGTACCTTCCTTCCTCTGCCCATCGTAAGCAAGAAACATCACATCCACAAGTTTGAACCCAAACTGATCGACCACA
ACGAGAGTATGGTTCACCACATCCTAGTGTATGCCTGCGGCAACGCCAGCGCCCTCCCCACCGGCATCAGCGACTGCTATGGAGCGGACCCGGCCTTCTC
GCTCTGCTCCCAAGTCATTGTTGGCTGGGCGGTCGGCGGCACAAGCTACCAGTTCCCGGATGATGTGGGCATCTCTATCGGGACCCCACGGGACCCCCAA
TGGATTCGGCTGGAGATTCATTACAGCAATTTCCACAATCTCCCTGGTATTTACGACTCGTCCGGGATCCGTGTGTACTACAGCCCACGGCTGCGCAGAT
ACGACATGGGTGTCCTCCAGCTGGGTTTCTTCACCTTCCCCATCCACTTCATCCCCCCGGGGGCCCAGTCCTTCCTGTCCTACGGGCTGTGCAAGACCGA
GAAGTTCGAGGAGATGAACGGGGCCCCGGTACCCGACATCCAGGTGTATGGCTACCTGCTTCATACTCACCTGGCAGGCCGGGCTCTGCAGACAGTACAG
TACAGAAACGGATCCCAGCTGCGAACCATCTGTAAAGACGACGCCTATGACTTCAACCTACAGGAGACTCGGGATTTACCTGAGCGCTTGGAGATCAAGA
CTGGAGATGAGCTACTGGTGGAGTGTCAGTATCAGACGCTGGACAGAGACACCATGACTTTCGGGGGGCCCAGCACCATCAATGAGATGTGTCTCGTCTT
CCTCTTCTACTACCCCCGGAACAACATCTCCAGCTGCATGGGCTATCCTGACATCATCTATGTGGCCCACGAGCTGGGGGAAGAGGCATCCGACTCCATG
GAGGGCATGATGGCCATGAACAACGTTGAGTGGACCCCAGAGAGCATTAAGAAGGCAGAGAAAGCCTGCAAGGAGGCTCAGCAGACAGTGATCATAAAGA
CCATTGATGAGGTGGTGGAGAATACAACAGGCTGGATCCGAGACATCCTCCCTACTCCCCGGGGGCCCTGCTTGGAGTCCACTGGCGGCAAGGTGGAGCC
CCAGGACAAGACCCCTGCGGGTTTCAGGGCCAGCCCATTGGTCCTTTCCAGTTCCAGCACCACTGCCCCCAAAGGTCTCCCACCGGTGACCCTCCTGTTC
ACACAGTGGGCTCTCTCATGGCTCCTCATCACCCTACAGGCTAGAGACTGA

>Chrysochloris_asiatica No=60 length=616 name="Cape golden mole"
MTRVLLFWLLLILTPPSQGHRLGPKSRLRYSRFLDSSNDVFLRWDFDLEAEVITFELQVQTAGWVGLGVTNRYTNVGGDLVVGGVLPGGNVYFSDQHLKD
EDTLEEDGSQDAELLGLTEDAVYTTMRFSRPFRSCDPHDHDITSDTVRVLAAYGQDDTLKLDRDHIFVKSIFLLQIIHPDDLEVPEDTIIHDLEITDFLI
PEDDTTYACTFLPLPIVSKKHHIHKFEPKLIDHNESMVHHILVYACGNASALPTGISDCYGADPAFSLCSQVIVGWAVGGTSYQFPDDVGISIGTPRDPQ
WIRLEIHYSNFHNLPGIYDSSGIRVYYSPRLRRYDMGVLQLGFFTFPIHFIPPGAQSFLSYGLCKTEKFEEMNGAPVPDIQVYGYLLHTHLAGRALQTVQ
YRNGSQLRTICKDDAYDFNLQETRDLPERLEIKTGDELLVECQYQTLDRDTMTFGGPSTINEMCLVFLFYYPRNNISSCMGYPDIIYVAHELGEEASDSM
EGMMAMNNVEWTPESIKKAEKACKEAQQTVIIKTIDEVVENTTGWIRDILPTPRGPCLESTGGKVEPQDKTPAGFRASPLVLSSSSTTAPKGLPPVTLLF
TQWALSWLLITLQARD
```

(1) Exon coordinates

Exon	NCBI Accession	Strand	Start	End
1	AMDV01101067	-	7224	7505
2	AMDV01101067	-	5712	5858
3	AMDV01101067	-	5078	5239
4	AMDV01101067	-	4668	4751
5	AMDV01101067	-	3080	3247
6	AMDV01101067	-	2885	2987
7	AMDV01101067	-	2594	2760
8	AMDV01101067	-	2253	2344
9	AMDV01101067	-	1923	2019
10	AMDV01101067	-	1328	1387
11	AMDV01101065	-	10673	10803
12	AMDV01101065	-	9530	9644
13	AMDV01101065	-	9060	9302

61. Lesser hedgehog tenrec (*Echinops telfairi*)

>Echinops_telfairi No=61 length=1863 name="Lesser hedgehog tenrec"
ATGGCCCATGTTTTTCTCATCCGGCTGATCCTGCTCTTGGCCTTGACCTTGGTCACCCACACCAAAGACAGCCGCCTGGGCCCCAGGTCTCGCCTGCGCT
ATTCCAGGTTCCTAGACCCGTCCAATGTCATGTTCCTGCGCTGGGACTTTGACCATGAGGCCGAGGTCATCACGTTTGAGCTGAAGGTCCGCACAGCTGG
TTGGGTGGGCCTGGGCGTGACGAATCGCTACACCAACGTGGGAGGCGATCTGGTGGTCGGCGGGGTCTTGCCCAACGGAAATGTCTACTTCTCGGACCAG
CACCTGGTAGATGAGTACACCCTGGAGGAGGATGGGAGCCAGGATGCGGAGCTGCAGGGGCTCACGGAAGATGCGGTCTACACCACCATGCGCTACTCAA
GGCCCTTCCGCTCCTGCGACCCTCATGACCAGGACATCTCGGTGGACACGGTACGGGTGCTGGCTGCGTACGGCCAGGACGACACGCTGACCCTGTCCCC
GGAGCACATCTTTGTCAAGTCCATCTTCCTGCTGCAGATGGTCCACCCCGACGACCTTGAAGTCCCCGAGGACACCCTTATCCATGACCTGGAGATCACT
GATTTCCTCATTCCGGAAGACGATACCACCTACGCCTGCACCTTCCTCCCTCTGCCCATCGTGAGCAAGAAGCACCACATTCACAAGTTTGAGCCCAAAC
TCATCTACCACAATGAGACCATGGTGCACCACATTCTGGTGTATGCCTGCGGCAATGCCAGCGCCCTCCCCACCGGCATCAGCGACTGCTATGGGGCCGA
CCCAGCCTTCTCGCTCTGCTCCCAGGTCATTGTGGGCTGGGCTGTTGGCGGCATGAGCTACCAGTTCCCGGATGACGTGGGGATCTCCATCGGGACCCCC
CTGGACCCCCAGTGGATCCGGCTAGAGATTCACTACAGCAATTTCCACAACCTCCAGGGCATCTACGACTCCTCTGGGATCCGTGTGTACTACAGCCCGC
GGCTGCGCAAATACGACATGGGTGTGCTGCAGCTGGGCTTCTTCACCTTCCCCATCCACTTCATCCCCCCGGGTGCCGAGTCCTACCTGTCCTATGGGCT
GTGCAAGTCGGAGAAGTTCGAGGAGATCAATGGGGCCCCGGTGCCCGACATCCAAGTGTTTGGCTACCTGCTGCATACCCACCTGGCAGGCCGTGCCCTT
CAGGCGGTGCAGTACAGAAATGGAAAGCAGATCAAGACCATCTGCAAAGATGACTCCTACGACTTCAATCTACAGGAGACTCGGGATTTACCCAAGCGCG
TGGTGATCAAGGCGGGAGACGACCTGCTGGTGGAGTGTCACTACCAGACGCTGGACCGAGACACCTTGACCTTTGGGGGTCCCAGCACCATCAATGAGAT
GTGCCTCATCTTCCTCTTCTACTATCCCCGGAACAACATCTCCAGCTGCATGGGGTACCCCGACATCATCTCCGTGGCCCATGAGATGGGGGAGGAAGCA
TCTGACCCCATGGAGGGCATGATGGCGATGAACAACATTGAGTGGACCCCGGAGAACATTAAGAAGGCCGAGAAAGCCTGCAAGGAGGCCCAGCAGATTG
TGGTCATCAAGACCATCGATGAGGTGGTAGAGAACACCACAGGCCAGATCCCCGACATCGTGCCCACTCCGCGGGGTCCCTGCCTGGAGTCCACTGGAGG
CAAGGTGGAGGCGCAGGATAAGACCCCTGCGGGCTTCAGGGCCGCCCCCCTGACCCTCTCTAGCGCCAGCACTGCCTCCTCCGGGGGCTTCTCCCTGGCC
ACCCTCCTGTTTGCACAGGGAGCTCTCTCGTGGCTCTTTGCCAGCCTGCAGGCTAGAGACTGA

>Echinops_telfairi No=61 length=620 name="Lesser hedgehog tenrec"
MAHVFLIRLILLLALTLVTHTKDSRLGPRSRLRYSRFLDPSNVMFLRWDFDHEAEVITFELKVRTAGWVGLGVTNRYTNVGGDLVVGGVLPNGNVYFSDQ
HLVDEYTLEEDGSQDAELQGLTEDAVYTTMRYSRPFRSCDPHDQDISVDTVRVLAAYGQDDTLTLSPEHIFVKSIFLLQMVHPDDLEVPEDTLIHDLEIT
DFLIPEDDTTYACTFLPLPIVSKKHHIHKFEPKLIYHNETMVHHILVYACGNASALPTGISDCYGADPAFSLCSQVIVGWAVGGMSYQFPDDVGISIGTP
LDPQWIRLEIHYSNFHNLQGIYDSSGIRVYYSPRLRKYDMGVLQLGFFTFPIHFIPPGAESYLSYGLCKSEKFEEINGAPVPDIQVFGYLLHTHLAGRAL
QAVQYRNGKQIKTICKDDSYDFNLQETRDLPKRVVIKAGDDLLVECHYQTLDRDTLTFGGPSTINEMCLIFLFYYPRNNISSCMGYPDIISVAHEMGEEA
SDPMEGMMAMNNIEWTPENIKKAEKACKEAQQIVVIKTIDEVVENTTGQIPDIVPTPRGPCLESTGGKVEAQDKTPAGFRAAPLTLSSASTASSGGFSLA
TLLFAQGALSWLFASLQARD

(1) Exon coordinates

Exon	NCBI Accession	Strand	Start	End
1	AAIY02229631	+	13973	14266
2	AAIY02229631	+	15183	15329
3	AAIY02229631	+	15823	15984
4	AAIY02229631	+	16194	16277
5	AAIY02229631	+	17064	17231
6	AAIY02229631	+	17361	17463
7	AAIY02229631	+	17860	18026
8	AAIY02229631	+	18255	18346
9	AAIY02229631	+	18557	18653
10	AAIY02229631	+	18837	18896
11	AAIY02229631	+	20768	20898
12	AAIY02229631	+	21574	21688
13	AAIY02229631	+	21908	22150

62. Nine-banded armadillo (*Dasypus novemcinctus*)

```
>Dasypus_novemcinctus No=62 length=1857 name="Nine-banded armadillo"
ATGACCTGTGCCCTTCTCTTCAGGCTTCTTCTACTCCTGGTTCTGTCTGCTTCCTCTCAAGGCCATCACCACAGCTCCACATCTCACCTGCGGTATTCCA
GGTTCCTAGATCCATCCAATGTCATTTTCCTGAGCTGGGACTTTGACTTTGATGCTGAGATCATCACTTTTGAGCTGAAGGTCCGGACAGCTGGCTGGGT
GGGTTTGGGTGTCACAAATCGCTATACCAACGTGGGAGGTGATCTGGTTGTCGGAGGAGTCTTGCCTGATGGCAATGTCTATTTCTCGGATCAGCACCTG
GTAGATGAAGACACCCTAGAGGAAGACCGGAGCCAGGATGCTGAACTGCAGGGGCTAATGGAAGATGCTAAATACACCACCATGCGATTTTCCAGGCCTT
TCCGCTCCTGTGACCCTCATGACCAAGACATTACGAGTGACACCGTGAGGGTGTTGGCTGCCTATGGCCTAGATGACACTCTGAAGCTGGATCGGGAGCA
CATTTTTGTGAAGTCCATCTTCCTGCTACAAATAGTCCACCCTGATGATCTTGAAGTCCCTGAGGACACCATCATCCATGACTTGGAGATCACTGATTTC
CTTATTCCAGAAGATGACACCACGTATGCCTGCACCTTTCTTCCTCTCCCCATCGTTAGCAAGAAACATCACATCCACAAGTTTGAACAAAAATTGGTCG
ACCACAATGAGACAATGGTGCATCACATCCTGGTGTACGCCTGTGGCAATGCCAGTGCTCTCCCGACAGGCATCAGCGACTGCTATGGGGCTGACCCTGC
CTTCTCCCTCTGCTCACAGGTCATCGTGGGCTGGGCTGTCGGGGGCACTAGTTACCAGTTCCCAGATGATGTGGGCATCTCTATTGGAACTCCCTTGGAC
CCCCAGTGGATCCGATTAGAGATTCATTACAGCAATTTTCACAACCGCCCTGGTGTGTATGACTCCTCAGGGATTCGCATATACTACAGCCCAAAACTGC
GCAAATATGACATGGGAGTCCTCCAGCTGGGCTTCTTCACTTTCCCCATCCACTTCATACCCCCGGGCGCTGAGTCCTTCATGTCCTATGGGCTGTGTAA
GACGGACAAGTTTGAAGAGATAAACAGGGCTCCAGTGCCTGACATACAAGTATATGGATACCTGCTCCACACCCACTTGGCAGGGCGGTCTCTACAGGCC
GTGCAATACAGAAATGGAACCCAACTCCGAACAATCTGTAAAGATGATGCCTATGACTTTAATCTGCAGGAGACCCGAGATTTACCTTATCGAGTGGAGA
TCAAGCCGGGAGATGAATTGCTGGTAGAGTGTCACTATCAGACACTGGACCGTGACTCCTTGACTTTTGGAGGGCCCAGCACCATTAATGAGATGTGCCT
TATCTTCCTCTTCTACTATCCCCGAAACAACATCTCCAGCTGCATGGGGTACCCTGACATCATCTACGTGGCTCATGAGCTGGGGGAGGAGGTATCAGAT
TCCATGGAGGGCATGATGGCCATGAACAATGTGGAGTGGACCCCAGAGAACATTAAGAAGGCTGAGAAAGCTTGCAAGGAGGCCCAGCAGACAGTGATAA
TAAAGACCATTGATGAGGTGGTAGAAAACACAACAGGCTGGATTCCAGACATCATTCCTACTCCTCGTGGACCCTGTTTAGAATCCTCTGGAGGCAAAGT
GGAGCCTCAGGACAAGAACCCTGCAGGCTTCAGGGCTGCCCCGCTGGCCCTCTTGGACTCCAGCACTGCCACCCTGAGGTGCCTCCCCCTGGCAGCCCTT
TTATTTGGGCAGGGGGCACTCTCATGGCTCCTTGCCAGCCTGCAGGCTGGAGTCTGA

>Dasypus_novemcinctus No=62 length=618 name="Nine-banded armadillo"
MTCALLFRLLLLLVLSASSQGHHHSSTSHLRYSRFLDPSNVIFLSWDFDFDAEIITFELKVRTAGWVGLGVTNRYTNVGGDLVVGGVLPDGNVYFSDQHL
VDEDTLEEDRSQDAELQGLMEDAKYTTMRFSRPFRSCDPHDQDITSDTVRVLAAYGLDDTLKLDREHIFVKSIFLLQIVHPDDLEVPEDTIIHDLEITDF
LIPEDDTTYACTFLPLPIVSKKHHIHKFEQKLVDHNETMVHHILVYACGNASALPTGISDCYGADPAFSLCSQVIVGWAVGGTSYQFPDDVGISIGTPLD
PQWIRLEIHYSNFHNRPGVYDSSGIRIYYSPKLRKYDMGVLQLGFFTFPIHFIPPGAESFMSYGLCKTDKFEEINRAPVPDIQVYGYLLHTHLAGRSLQA
VQYRNGTQLRTICKDDAYDFNLQETRDLPYRVEIKPGDELLVECHYQTLDRDSLTFGGPSTINEMCLIFLFYYPRNNISSCMGYPDIIYVAHELGEEVSD
SMEGMMAMNNVEWTPENIKKAEKACKEAQQTVIIKTIDEVVENTTGWIPDIIPTPRGPCLESSGGKVEPQDKNPAGFRAAPLALLDSSTATLRCLPLAAL
LFGQGALSWLLASLQAGV
```

(1) Exon coordinates

Exon	NCBI Accession	Strand	Start	End
1	AAGV03115163	-	11312	11599
2	AAGV03115163	-	10218	10364
3	AAGV03115163	-	9470	9631
4	AAGV03115163	-	9145	9228
5	AAGV03115163	-	8155	8322
6	AAGV03115163	-	7847	7949
7	AAGV03115163	-	7498	7664
8	AAGV03115163	-	7075	7166
9	AAGV03115163	-	6699	6795
10	AAGV03115163	-	6499	6558
11	AAGV03115163	-	4879	5009
12	AAGV03115163	-	4052	4166
13	AAGV03115163	-	3500	3742

63. Gray short-tailed opossum (*Monodelphis domestica*)

```
>Monodelphis_domestica No=63 length=1845 name="Gray short-tailed opossum"
ATGACTTGTGTCCTCCTTCCCAGGCTACTCCTCCTCTTGCCCTTGGCTGCTCCTGCCACAGGAATCCACCATGGCCCCACATCTCCCCTTCGTTACTCAA
GATTCCTTGACCCATCCAATGTCATGTATCTACGCTGGGACTTTGATCTTGAAACTGAGATTATCACCTTTGATCTGCAGGTCCGGACAACTGGCTGGGT
GGGTCTTGGTATCACTAATCGCTACACCAATGTGGGGGCCGATCTAGTGGTTGGAGGTGTCCTGCCTGATGGGACTGTCTACTTCTCGGACCAGCATTTG
GTAGATGATGAAACTCTGCACGAGGATGGCAGCCAAGATGCAGAGCTTCAATTGCTGAAAGAAGATGCTGTCTATACCACCATGCGCTTCTCCCGGCCCT
TCCGCTCCTGTGATTCCCATGATGGAGACATCACAAGTGACACAATTCGGGTCCTGGGTGCTTATGGGTTGGATGACACAGTGAAGCTGGATAGGAAGCG
AACCTTTGTCAAATCAATCTTCCTGCTGCAGATTGTCCATCCTGATGATCTTGATGCCCCCAAAATCTCTTATATCCATGACTTGGAGATCACTGATTTT
CTGATTCCTGAAGATGATACAACTTATGCCTGTACCTTTCTTCCTCTGCCCATCGTCAAGGAGAAGCATCACATCTATAAGTTTGAGCCGAAGCTGATCT
ACCACAATGAGACAATGGTACATCACATCCTGGTTTATGCCTGTGGAAATGCCAGTTCCCTCCCAACAGGCATTAGTGACTGCTATGGTGCTGACCCTGC
CTTCTCCCTCTGCTCCCAAGTGATCGTGGGCTGGGCTGTCGGGGGCACTAGTTACCAGTTTCCAGATGATGTGGGCATTTCCATTGGAACTCCCTTGGAT
CCCCAGTGGATCCGACTAGAGGTTCACTACAGTAATTTCCACAATCTTCCTGGTGTGTATGACTCTTCTGGGATTCGAGTTTATTTCACCCCTATCCTGC
GCAAATATGACATGGGAGTCCTTCAGCTTGGATTCTTTACCTTCCCCATCCACTTCATACCACCAGGAGCAGAATCTTTTATGTCCTATGGGCTCTGTGA
AACCGAGAAGTTTGAAGAGATGAATGGGACTCCTGTGCCTGACATCCAAGTTTATGGTTATCTTCTGCACACTCACCTAGCAGGGCGGTCTTTGCAAGCC
GTGCAATACAGGAATGGAAAGCAGATCAAGACCATCTGCAAGGATGATGCCTATGACTTCAACCTACAAGAGACTAGAGACTTGGATAAGCGTGTGACAA
TCAAGATGGGAGATGAATTGTTGGTGGAGTGTCACTACCAGACCCTTGATCGTACCACACTCACCTTTGGTGGCCCAAGCACCATTAATGAGATGTGCCT
CATCTTTCTCTTCTACTATCCCCGAAACAACATCTCCAGTTGCATGGGGTACCCAGACATCATTCATGTGGCTCATGAACTTGGAGAAGAAGCAACTGAC
TCCATGGAGGGCATGATGGCCATGAATAATATTGAATGGAACCCAGAAATCATCAAGAAGGCAGAGAAAGCCTGCAAAGAGGCTGACCAGGTGGTGGTAA
TAAAGACCATTGATGAGATGGTGATGAATCAGACAGGCCACATTCCAGATATAATCCCTACTCCCCGTGGTCCTTGCTTGGAGTCCATAGGGGGCAAAGT
AGAGCCACAGGACAAGACCCCTGCAGGATTCCGAGCTGCTCCTCCAATCCAATCTGGCTGTAGTTCTGCATTTTCCTTGACTATTCTCTTACTCCTACAG
GGTACCTTTTCTTGGCTTCTGATTTCCCTCCAAAGTGCAATCTGA
```

```
>Monodelphis_domestica No=63 length=614 name="Gray short-tailed opossum"
MTCVLLPRLLLLLPLAAPATGIHHGPTSPLRYSRFLDPSNVMYLRWDFDLETEIITFDLQVRTTGWVGLGITNRYTNVGADLVVGGVLPDGTVYFSDQHL
VDDETLHEDGSQDAELQLLKEDAVYTTMRFSRPFRSCDSHDGDITSDTIRVLGAYGLDDTVKLDRKRTFVKSIFLLQIVHPDDLDAPKISYIHDLEITDF
LIPEDDTTYACTFLPLPIVKEKHHIYKFEPKLIYHNETMVHHILVYACGNASSLPTGISDCYGADPAFSLCSQVIVGWAVGGTSYQFPDDVGISIGTPLD
PQWIRLEVHYSNFHNLPGVYDSSGIRVYFTPILRKYDMGVLQLGFFTFPIHFIPPGAESFMSYGLCETEKFEEMNGTPVPDIQVYGYLLHTHLAGRSLQA
VQYRNGKQIKTICKDDAYDFNLQETRDLDKRVTIKMGDELLVECHYQTLDRTTLTFGGPSTINEMCLIFLFYYPRNNISSCMGYPDIIHVAHELGEEATD
SMEGMMAMNNIEWNPEIIKKAEKACKEADQVVVIKTIDEMVMNQTGHIPDIIPTPRGPCLESIGGKVEPQDKTPAGFRAAPPIQSGCSSAFSLTILLLLQ
GTFSWLLISLQSAI
```

(1) Exon coordinates

Exon	NCBI Accession	Strand	Start	End
1	AAFR03013986	-	69333	69620
2	AAFR03013986	-	66325	66471
3	AAFR03013986	-	63325	63486
4	AAFR03013986	-	62266	62349
5	AAFR03013986	-	61227	61394
6	AAFR03013986	-	60946	61048
7	AAFR03013986	-	60582	60748
8	AAFR03013986	-	59676	59767
9	AAFR03013986	-	59267	59363
10	AAFR03013986	-	59042	59101
11	AAFR03013986	-	57743	57873
12	AAFR03013986	-	56438	56552
13	AAFR03013986	-	55828	56058

64. Tasmanian devil (*Sarcophilus harrisii*)

>Sarcophilus_harrisii No=64 length=1845 name="Tasmanian devil"
ATGACTTGTGCCCTGCTCCCCAAGCTACTCCTGCTCTTAACCTTGGCTGCTCCTGCCACAGGAATCCCTCATGGTCCCACCTCCCCCCTTCGCTACTCGA
GATTCCTTGGCCCCTCCAACGTCATGTATCTGCGCTGGGACTTTGACATTCAGACCGAGGTCATCACTTTCGATCTGCAGGTCCGGACCACTGGCTGGGT
GGGTCTTGGTGTCACTAATCGCTACACCAATGTGGGAGCTGACCTAGTGGTTGGAGGTGTCCTGCCTGATGGAACTGTCTACTTCTCGGACCAGCACTTG
GTAGATGATGATACTCTGAAGGAGGATGGGAGCCAGGATGCAGAGTTACAATTGCTGAAAGAAGATGCTGTCTACACTACCATGCGCTTCTCCCGACCCT
TCCGCACCTGTGACTCCCATGATGGAGACATCACAAGTGACACAATCCGGGTCCTGGCAGCTTATGGACTGGATGACACAGTAAAGCTGGAGAGGGAGCG
CACCTTTGTCAAGTCAATCTTTCTGCTGCAGATTATCCATCCTGATGATCTTGATGCCCCTGCAATCTCATACATTCATGACTTGGAGATCACTGATTTT
CTCATCCCTGAAGATGACACGACTTATGCCTGCACCTTTCTTCCCCTGCCCATCGTCAAGGAGAAGCATCATATCTATAAGTTTGAGCCAAAGCTGATCT
ACCACAATGAGACGATGGTGCACCACATCCTGGTTTATGCCTGTGGCAATGCCAGTGCCCTCCCGACGGGCATCAGTGACTGCTATGGTGCTGATCCTGC
CTTCTCCCTGTGCTCCCAAGTGATCATGGGCTGGGCTGTCGGAGGCACTAGTTACCAGTTTCCAGATGATGTGGGCATTTCCATTGGGACTCCCCTGGAC
CCCCAGTGGATCCGGCTAGAGATTCACTACAGCAATTTCCACAATCTTCCTGGCATATATGACTCTTCTGGGATTCGAGTTTACTTCACCCCCATCCTGC
GCAAATATGACATGGGAGTCCTCCAGCTTGGATTCTTTACCTTCCCCATCCACTTCATACCACCAGGAGCAGAGTCTTTCACGTCCTATGGGCTCTGTAT
GACTGAGCAGTTTGATGAGATAAATGGGGTTCCTGTGCCTGACATACAAGTTTATGGTTATCTTCTGCATACTCACCTAGCAGGGAGGTCTTTGCAAGCT
GTGCAGTACAGGAAAGGAAAGCAGGTCGGGATCATCTGCAAGGATGACGCCTATGACTTTAATCTACAAGAGACTCGAGACTTAAAAGAGCGAATAACAA
TCAAGATGGGAGATGAATTGTTGGTGGAGTGTCACTACCAGACCCTCGATCGTACCACACTCACCTTTGGTGGCCCAAGTACCATTAATGAGATGTGTCT
CATCTTTCTCTTCTACTATCCCCGGAACAACATCTCCAGCTGCATGGGGTACCCAGACATCATCCACGTGGCCCACGAACTTGGAGAAGAAGCATCGGAC
TCCATGGAAGGCATGATGGCCATGAATAATGTCGACTGGACCCCAGAAAACATCAAAAAAGCAGAGAAAGCCTGCAAGATGGCTGACCAGGTGGTGATAA
TAAAGACCATTGATGAGTTGGTGAAGAATCAGACAGGCCACATTCCAGACATAATTCCTACTCCCCGTGGGCCTTGCTTGGAGTCCACAGGGGGCAAAGT
AGAACCAGGAGACAAGACCCCTGCAGGATACCGAGCTGCCCCTCTAGTCCAATCTGGCTCCAGTCCTACAGTTCCCTTAACTATTCTCCTACTCCTACAG
GGGGTCTTTTCTTGGCTTCTGGATTCCCTCCAAGGTGCAGTCTGA

>Sarcophilus_harrisii No=64 length=614 name="Tasmanian devil"
MTCALLPKLLLLLTLAAPATGIPHGPTSPLRYSRFLGPSNVMYLRWDFDIQTEVITFDLQVRTTGWVGLGVTNRYTNVGADLVVGGVLPDGTVYFSDQHL
VDDDTLKEDGSQDAELQLLKEDAVYTTMRFSRPFRTCDSHDGDITSDTIRVLAAYGLDDTVKLERERTFVKSIFLLQIIHPDDLDAPAISYIHDLEITDF
LIPEDDTTYACTFLPLPIVKEKHHIYKFEPKLIYHNETMVHHILVYACGNASALPTGISDCYGADPAFSLCSQVIMGWAVGGTSYQFPDDVGISIGTPLD
PQWIRLEIHYSNFHNLPGIYDSSGIRVYFTPILRKYDMGVLQLGFFTFPIHFIPPGAESFTSYGLCMTEQFDEINGVPVPDIQVYGYLLHTHLAGRSLQA
VQYRKGKQVGIICKDDAYDFNLQETRDLKERITIKMGDELLVECHYQTLDRTTLTFGGPSTINEMCLIFLFYYPRNNISSCMGYPDIIHVAHELGEEASD
SMEGMMAMNNVDWTPENIKKAEKACKMADQVVIIKTIDELVKNQTGHIPDIIPTPRGPCLESTGGKVEPGDKTPAGYRAAPLVQSGSSPTVPLTILLLLQ
GVFSWLLDSLQGAV

(1) Exon coordinates

Exon	NCBI Accession	Strand	Start	End
1	AFEY01427030	+	8049	8336
2	AFEY01427030	+	11469	11615
3	AFEY01427030	+	13698	13859
4	AFEY01427030	+	14753	14836
5	AFEY01427030	+	15431	15598
6	AFEY01427030	+	15793	15895
7	AFEY01427030	+	16125	16291
8	AFEY01427030	+	17145	17236
9	AFEY01427030	+	17516	17612
10	AFEY01427030	+	17769	17828
11	AFEY01427030	+	19027	19157
12	AFEY01427030	+	20767	20881
13	AFEY01427030	+	21265	21495
