# Supplementary material for: Frequent Loss and Alteration of the MOXD2 Gene in Catarrhines and Whales: A Possible Connection with the Evolution of Olfaction
Source: PLoS One. 2014 Aug 7;9(8):e104085. doi: 10.1371/journal.pone.0104085 (PMC4125168; doi:10.1371/journal.pone.0104085)
Supplement: Data S2 — The sequence data file, tree files, control files, and main result files for the codeml analyses of catarrhine MOXD2 gene. (PDF) [file pone.0104085.s005.pdf]

Data S2. Files used to perform the codeml analyses to generate Tables 2 and 3.

Sequence data file: catarrhines.phy

```
13 1494

Homo_sapiens
ATG GCC CAT GAC CTT CTC TTC AGG CTT TTT CCA CTT TTG GCC CTG GGA GTC CCC TTA CAA AGC AAC
CGC CTT GGC CCC ACA TCT CGC CTG CGC TAT TCC AGG TTC CTA GAT CCT TCT AAT GTC ATT TTC CTG
CGT TGG GAC TTT GAC CTT GAG GCT GAA ATC ATC AGT TTT GAG CTC CAG GTC CGT ACA GCT GGC TGG
GTG GGC TTC GGT GTC ACA AAT CGC TAC ACC AAC GTG GGA AGT GAT CTG GTT GTT GGA GGA GTC TTG
CCT AAT GGC AAT GTC TAT TTC TCG GAT CAG CAC TTG GTG GAC GAA GAC ACT CTG AAG GAG GAT GGG
AGC CAG GAT GCT GAG CTG CTG CGG CTG ACG GAA GAT GCT GTC TAC ACC ACC ATG CAC TTT TCC AGG
CCC TTC CGC TCC TGC GAC CCT CAT GAC CTG GAT ATT ACG AGC AAC ACC GTG AGG GTG CTG GCC ACC
TAT GGC CTG GAT GAC ACT CTG AAG CTG TAT CGG GAG CGT ACT TTT GTC AAG TCC ATC TTT CTG CTA
CAA GTC GTC CAC CCT GAC GAT CTG GAT GTC CCT GAG GAC ACC ATC ATC CAT GAC TTG GAG ATC ACT
AAT TTC CTC ATT CCA GAG GAT GAC ACC ACG TAT GCC TGC ACC TTT CTT CCT CTC CCT ATT GTG AGC
GAG AAG CAT CAC ATT TAC AAG TTT GAG CCT AAG TTG GTC TAC CAC AAT GAG ACA ACG GTG CAT CAC
ATC CTG GTG TAC GCC TGC GGC AAT GCT AGC GTT CTC CCC ACA GGC ATC AGC GAC TGC TAT GGG GCC
GAC CCT GCC TTC TCC CTT TGC TCA CAG GTC ATC GTG GGC TGG GCT GTC GGG GGC ACA AGC TAC CAG
TTT CCA GAT GAC GTA GGC GTC TCT ATT GGG ACC CCC TTG GAC CTT CAG TGG ATC CGA CTG GAG ATT
CAT TAC AGC AAT TTT AAC AAC CTT CCT GGT GTG TAT GAT TCC TCG GGG ATT TGC GTG TAC TAC ACT
TCT CAG CTG CGC AAA TAC GAC ACG GAT GTC CTC CAG CTG GGC TTC TTC ACG TTT CCC ATC CAC TTC
ATC CCC CCG GGC GCT GAG TCC TTC ATG TCC TAT GGG CTG TGT AGG ACG GAG AAG TTT GAG GAG ATG
AAT GGA GCT CCT ATG CCT GAC ATA CAG GTG TAT GGC TAC CTG CTG CAC ACC CAC TTG GCT GGA CGG
GCT CTG CAA GCA GTG TAC AGG AAT GGA ACA CAA CTT CGA AAA ATC TGT AAA GAC GAT TCC TAT GAC
TTC AAC CTG CAG GAG ACT CGA GAT TTG CCC TCT AGA GTG GAG ATC AAG CCG GGA GAT GAA TTG CTG
GTA GAA TGT CAC TAC CAG ACA CTG GAC CGT GAC TCC ATG ACA TTT GGA GGC CCC AGC ACC ATT AAT
GAG ATG TGC CTC ATC TTC CTC TTC TAC TAT CCC CAA AAT AAC ATC TCC AGC TGC ATG GGG TAC CCT
GAC ATT ATC TAC GTG GCC CAC GAG CTG GGG GAG GAG GCA TCA
Pan_troglodytes
... ... ... ... ... ... ... ... ... ... ... ... ... ... ... ... .C. ... ... ... ... ...
... ... ... ... ... ... ... ... ... ... ... ... ... ... ... ... ... ... ... ... ... ...
... ... ... ... ... ... ... ... ... ... ... ... ... ... ... ... ... ... ... ... ... ...
... ... ... ... ... ... ... ... ... ... ... ... ... ... ... ... ... ... ... ... ... ...
... ... ... ... ... ... ... ... ... ... ... ... ... ... ... ... ... ... ... ... ... ...
... ... ... ... ... ... ... G.. ... ... ... ... ... ... ... ... ... ... .G. ... ... ...
... ... ... ... ... ... ... ... ... ... ... ... ... ... G.. ... ... ... ... ... ... G..
... ... ... ... ... ... ... ... ... G.. ... ... ... ... ... ... ... ... ... ... ... ...
... ... ... ... ... ... ... ... ... ... ... ... ... ... ... ... ... ... ... ... ... ...
G.. ... ... ... ... ... ... ... ... ... ... ... ... ... ... ... ... ... ... ... ... ...
... ... ... ... ... ... ... ... ... ... ... ... ... ... ... ... ... ... ... ... ... ...
... ... ... ... ... ..T ... ... ... ... ... ... ... ... ... ... ... ... ... ... ... ...
... ... ... ... ... ... ... ... ... ... ... ... ... ... ... ... ... ... ... ... ... ...
... ... ... ... ... ... ... ... ... ... ... ... ... ... .C. ... ... ... ... ... ... ...
... ... ... ... ... ... ... ... ... ... ..A ... ... ... ... ... ... C.. ... ... ... ...
... ... ... ... ... ... ... .T. .G. ... ... ... ... ... ... ... ... ... ... ... ... ...
... ... ... ... ... ... ... ... ... ... ... ... ... ... ... ... ... ... ... ... ... ...
... ... ... ... ... ... ... ... ... ... ... ... ... ... ... ... ... ... ... ... ... ...
... ... ... ... ... ... ... ... ... ... ... ... ... ... ... ... ... ... ... ... ... ...
... ... ... ... ... ... ... ... ... ... ... C.. ... ... ... ... ... ... ... ... ... ...
... ... ... ... ... ... ... ... ... ... ... ... ... ... ... ... ... ... ... ... ... ...
... ... ... ... ... ... ... ... ... ... ... .G. ... ... ... ... ... ... ... ... ... ...
... ... ... ... ... ... ... ... ... ... ... ... ... ...
Gorilla_gorilla_gorilla
... ... ... ... ... ... ... ... ... ... ... ... ... ... ... ... ... ... ... ... ... ...
... ... ... ... ... ... ... ... ... ... ... ... ... ... ... ... ... ... ... ... ... ...
A.. ... ... ... ... ... ... ... ... ... ... ... ... ... ... ... ... ... ... ... ... ...
... ... ... ... ... ... ... ... ... ... ... ... ... ... ... ... ... ... ... ... ... ...
... ... ... ... ... ... ... ... ... ... ... ... ... ... ... ... ... ... ... ... ... ...
... ... ... ... ... ... ... G.. ... ... ... ... ... ... ... ... ... ... .G. ... ... ...
... ... ... ... ... ... ... ... ... ... ... ... ... ... G.. ... ... ... ... ... ... G..
... ... ... A.. ... ... ... ... ... G.. ... ... ... ... ... ... ... ... ... ... ... ...
... ... ... ... ... ... ... ... ... ... ... ... ... ... ... ... ... ... ... ... ... ...
G.. ... ... ... ... ... ... ... ... ... ... ... ... ... ... ... ... ... ... ... ... ...
... ... ... ... ... ... ... ... ... ... ... ... ... ... ... ... ... ... ... ... ... ...
... ... ... ... ... ..T ... ... ... ... ... ... ... ... ... ... ... ... ... ... ... ...
... ... ... ... ... ... ... ... ... ... ... ... ... ... ... ... ... ... ... ... ..T ...
... ... ... ... ... ... A.. ... ... ... ... ..T ... ... .C. ... ... ... ... ... ... ...
... ... ... ... ... ... ... ... ... ... ... ... ... ... ... ... ... C.. ... ... ... ...
... ... ... ... ... ... ... .T. .G. ... ... ... ... ... ... ... ... ... ... ... ... ...
... ... ... ... ... ... ... ... ... ... ... ... ... ... ... ... ... ... ... ... ... ...
... ... ... ... ... ... ... ..G ... ... ... ... ... ... ... ... ... ... ... ... ... ...
... ... ... ... ... ... ... ... ... ... ... ... ... ... ... ... ... ... ... ... ... ...
... ... ... ... ... ... ... ... ... ... ... C.. ... C.. ... ... ... ... ... ... ... ...
... ... ... ... ... ... ... ... ... ... ... ... G.. ... ... ... ... ... ... ... ... ...
```

```
... ... ... ... ... ... ... ... ... ... ... .G. ... ... ... ... ... ... ... ... ... ...
... ... ... ... ... ... ... ... ... ... ... ... ... ...
Pongo_abelii
... ... ... ... ... ... ... ... ... ... ... ... ... ... ... ... .C. ... ... ... ... ...
... ... ... ... ... ... ... ... T.. ... ... ... ... ... ... ... ... ... ... ... ... ...
... ... ... ... ... ... ... ... ... ... ... ... ... ... ... ... ... ..G .G. ... ... ...
... ... ..G ... ... ... ... ... ... ... ... ... ... ... ..C ... ..C ... ... ... ... ...
... ..A ... ... ... ... ... ... ... ... ... ... ... ... ... ... ... ... ... ... ... ...
... ... ... A.. ... ... ... G.. ... ... ... ... ... ..T ... ... ... ... .GT ... ... ...
... ... ... ... ... ..T ... ... ... ... ... ... .T. ... G.. ..T ... ... ... ... ..T G..
... ... ... ... ... ... .C. ... ... G.. .A. ... ... ... ... ... ... ..T ... ... ... ...
... ... ... ... ... ... A.. ... ... ... ... ... ... ... ... ... ... ... ... ... ... ...
G.. ... ... ... ... ... ... ... ... ... ... ... ... ... ... ... ... ... ... ... ... ...
C.. ... ... ... ... ... ... ... ... ... ... ... ... ... ... ... ... ... .T. ... ... ...
... ... ... ..T ... ..T ... ... ... ... ... ... ... ... ... ... ... ... ... ... ... ...
... ..C ... ... ... ..C ... ... ... ... ... ... ... ... ... ... A.. ... ..T ... ... ...
... ... ... ... ... ... ... ... ... ... ... ... ..A ... .C. ... ... ... ... ... ... ...
... ... ... ... ... ... ... ... ... ... ... ... ... ... ..A ... ... CA. ... ... ... ...
... ... ... ... ... ..T ... .T. .GC ... ... ... ... ... ... C.. ... ... ... ... ... ...
... ... ... ... ... ... ... ... ... ... .G. ... ... ... ... ... ... ... ... ... ... ...
... ... ... ... ... ... ... ... ... ..A ... ... ... ... ... ... ... ... ..T ... ... ...
... ... ... ... ... ... ... ... ... ... ... ... ... .C. ... ... ... ... ... ... ..C ...
... ... ... ... ... ... ... ... ... ... ... C.. ... ... ... ... .T. ... ... .G. ... ...
... ... ... ... ... ... ... ... ... T.. ... ... ... ... ... ... ... ... ... ... ... ...
... ... ... ... ... ... ... ... ... ... ... .G. ... ... ... ..T ... ... ... ... ... G..
... ..C ... ... ... ... ... ... ... ... ... ... ... ...
Macaca_mulatta
... ... ... ... ... ... ... ... ... ... ..G ... ... ... ... .C. .C. ... ... ... ... ...
... .C. ... ... ... ... ... ... ... ... ... ... ... ... ... ... ..C ... ... ... ... ...
... ... ... ... ..T ... ... ... ... ... ... ... ... ... ... ... ... ..G ... ... ... ...
... ... ..A ... ... ... ... ... ... ... ..T ... ... ... ... ... ... ... ... ... ... ...
..C ... ... ... ... ... ... ... ... ... ... ... ..A A.T .G. ... ... ... ... ... ... ...
... ... ... ... ... ... ... G.. ... ..A ... ... ... ... ... ... ... ... .GT ... ... ...
... ... ... ... ..T ... .TG ... ... ... ... ... ... ..T G.. ..T ... ... ... ... ... G..
... ..T ... ... ... ... ... ... ... G.. .A. ... ... ... ... ... ... ... ... ... ... ...
... ..T ... ... ... ..T ... ... ... ... ..C A.. ... ... ... ... ... ... ... ... ... ...
G.. ... ... ... ... ... ... ... ... ... ..C ... ... ... ... ... ... ... ..C ... ..T ...
... ..A ... ... ... ... ... ... ... ... ... ... ... ... ... ... ... ... .T. ... ... ...
... ... ... ..T ... ..T ... ... ... ... A.. ... ... ... ... ... ... ... ... ... ... ...
... ... ... ... ... ..C ... ... ... ... ... ... ... ... ... ... ... ... ..T ... ... ...
... ... ... ..T ..G ..T ... ... ... ... ..T ... ... ... .C. ... ... ... ... ... ... ...
... ... ... ... ... ... ... ..G ... ... ... ... ... ... ... ... ... C.. ... ... ... ...
... ... ... ... ... ..T ... .T. .GC ... ... ... ... ... ... ... ... ... ... G.. ... ...
... ... ... ... ... ... ... ... C.. ... ..C ... ... ... ... ..A ... ... ... ... ... ...
... ... ..C ..C G.. ... ... ... ... ... ... ... ... ... ... ... ... ... ... ... ... .A.
... ... ... ..C ... ... ... ... ... ... ... ... ... .C. ... ... ... ... ... ... ..C ...
... ..T ... ... ... ... ... ... ... ... ... C.. ... ... ... ... ... ... ... ... ... ...
... ... ... ... ... ... ... ... ... ... ... .T. ... ... ... ... ... ... ... ... ... ...
... ... ... ... ... ... ... ... ... ... ..G .G. ... ... ... ... ... ... ... ... ... ...
... ... ... ... ... ... ... ..A ... AA. ... ... ... ...
Papio_anubis
... ... ... ... ... ... ... ... ... ... ..G ... ... ... ... .C. .C. ... ... ... ... ...
... .C. ... ... ... ... ... ... ... ... ... ... ... ... ... ... ..C ... ... ... ... ...
... ... ... ... ... ... ... ... ... ... ... ... ... ... ... ... ... ..G ... ... ... ...
... ... ..A ... ... ... ... ... ... ... ... ... ... ... ... ... ... ... ... ... ... ...
..C ... ... ... ... ... ... ... ... ... ... ... ..A A.. .G. ... ... ... ... ... ... ...
... ... ... ... ... ... ... GA. ... ..A ... ... ... ... ... ... ... ... .G. ... ... ...
... ... ... ... ..T ... ..G ... ... ... ... ... ... ..T G.. ..T ... ... ... ... ... G..
..C ..T ... ... ... ... ... ... ... G.. .A. ... ... ... ... ... ... ... ... ... ... ...
... ..T ... ... ... ..T ... ... ... ... ..C A.. ... ... ... ... ... ... ... ... ... ...
G.. ... ... ... ... ... ... ... ... ... ..C ... ... ... ... ... ... ... ..C ... ..T ...
... ..A ... ... ... ... ... ... ... ... ... ... ... ... ... ... ... ... .T. ... ... ...
... ... ... ..T ... ..T ... ... ... ... ... ... ... ... ... ... ... ... ... ... ... ...
..T ... ... ... ... ..C ... ... ... ... ... ... ... ... ... ... ... ... ..T ... ... ...
... ... ... ..T ..G ..T ... ... ... ... ..T ... ... ... .C. ... ... ... ... ... ... ...
... ... ... ... ... ... ... ..G ... ... ... ... ... ... ... ... ... C.. ... ... ... ...
... ... ... ... ... ..T ... .T. .GC ... ... ... ... ... ... ... ... ... ... ... ... ...
... ... ... ... ... ... ... ... C.. ... ..C ... ... ... ... ..C ... ... ... ... ... ...
... ... ..C ..C G.. ... ... ... ... ... ... ... ... ... ... ... ... ... ... ... ... .A.
... ... ... ..C ... ... ... ... ... ... ... ... ... .C. ... ... ... ... ... ... ..C ...
... ..T ... ... ... ... ... ... ... ... ... C.. ... ... ... ... .T. ... ... ... ... ...
... ... ... ... ... ... ... ... ... ... ... .T. ... ... ... ... ... ... ... ... ... ...
... ... ... ... ... ... ... ... ... ... ... .G. ... ... ... ... ... ... ... ... ... ...
... ... ... ... ... ... ... ... ... ... ... ... ... ...
Chlorocebus_sabaeus
... ... ... ... ... ... ... ... ... ... .TG ... ... ... ... .C. .C. ... ... ... ... ...
... .C. ... ... ..C ... ... ... ... ... ... ... ... ... ... ... ..C ... ... ... ... ...
```

... ...G ... ...
... .A ... ...
..C ... ...A A.. .G. ... ...
... ... G.. ... ..A ... ... .G. ... ...
... ...G ... ...T G.. ..T ... ... G..
..C ..T ... ... G.. .A. ... ...
... ...C A.. ... ...
G.. ... ...C ... ...C ... ..C ...
... ..A ... ... .T. ... ...
... ...T ... ..T ... ...
... ...C ... ...T ... ...
... ...T ..G ..T ... ...T ... ... .C. ... ...
... ...G ... ... C.. ... ...
... ...T ..T .T. .GC ... ...
... ... C.. ... ...A ... ...
... ... .GC ..C G.. ... ...
... ...C ... ... A.. ... ... .A. .C. ... ...C ...
... ..T ... ... C.. ... ...
... ... .T. ... ...G ...
... ... .G. ... ...
... ...
Saimiri_boliviensis
... ... TG. ... ... .TG ... ... .C. .C. ... .G ... ...
... ...C ... .G ... ...
.T. ... ... T.. ... ... .AG ... ...
... .G ... ...
... ...A ... ... C.. ... ...
... ...C ... G.. ... ... .G. ... ...
... ...T G.. ..T ... ...T G..
... ..T ... ... G.. ... ...
... ..T ... ...T ... ...C ... ..T G.. ... ..T ... ...
G.. ... ..T ... ...C ... ..T ..T
... ...C ... ... .T. ... ...
... ...T ... ...T ... ...T ... ...
... ...C ... ...T ... .T ... ...
... ...T ..G ... A.. ... ... .C. ... .T ... ...
... ...C ... ... C.T ... ...
G.. ... ..C ... .T ... .T. .G. ... ...C ... ...
... ...C
... .C ..C G.. ... ...T ... ...T ... ... A..
... T.. ... ..T ... ... .C. ... ...
... ..T ... ...T ... C.. ... ...
... ... G.. ... ...
... ... .G. ... ...
... ...T ... ...
Callithrix_jacchus
... ... TG. ... ... .TG ... ... .C. .CT G.. ... ...
... ...G ... .C .G. ... ..G ... ...
.T. ... ...A ... ...G ... ...
... .G ... ...
... ...A ... ... C.. ... ... .C.
... ... G.. ... ... A.. ... ... .G. ... ...
... ...A ... ...T G.. ..T ... ... G..
... ... G.. ... ... .A. ... .T ... ...
... ..T ..T ... ...C ... ..T G.. ... ..T ... ...
G.. ... ..T ... ...T ... ...C ... ..T ..T
... ...C ... ... .T. ... ...
... ...T ... ..T ... ... T.. ... .T ... ...C ... ..T
... ...C ... ...T ... .T ... ...
... ...T ..G ... A.. ... ... .C. ... ...
... ...C ... ... C.T ... ...
G.. ... ..C ... .T ... .T. .GA ... ...A ..C ... ...
... ...
... .C ..C G.. ... ...T ... ...T ... ... A..
... ...T ... ... .C. ... ...T ... ...
... ..T ... ...T ... C.. ... ...
... ... G.. ... ...
... ... .G. ... ...
... ...T ... .T ... ...
Tarsius_syrichta
... T.. TG. .C. ... T.. ... ...C ATG T.. C.. ... ... AT. .CA ... .CC .T. G.. ...
..T ..A ..T .T. ... ...T .T. ... ...C ... ... G.. ... ...
..C ... ... A.. ... ... A.. ... ..G ..A ... .C. ..C ... ...G ..G ... ...
... .G ... A.. .A. ... ...C ... ... C..
... G.. ... .T ... ..T ... ..C ... ... C.C ... .T ... ..C ... G.A ... ..C A..
... ... T.. .A. G.. ... ... .G. ... ... .G. ... ..A ..A
... ...T ... ...C ... ...T G.. ..T ... ...T G..

.T. ... ... G.. ... ..A ... ...
... ..A ... ...T ... ... TA. ..C ... ...T ... ...
G.A ... ...A ... ...C ... ...C ..T .A.
A.. ... .T ..C ... ...A ..C ... ... A.. ... ...T .T. ... ...
... ...T ... ..T ... .C ..T ... ... .AG ... ...
... ...T ... ..C ... ..G ... ...C ..T ... .T ..T ... ...
... ...T A.. ... ... .C. ... ...
... ..T ... ... C.. ... ...C ... .T ... C.A ... ... .G.
... ... .T. .GC ... .A T.. ... ...A .C ... ...
..A ... ..A ... ...C ..A ... ... .AA ... ..T ... ...
... ..G .TC ..A T.T ... ...A ... ...T ... ...G ...
... .G ..T ... .A ... ... .C. ... ...T ... ...
... ..T ... .A ... ...T ... C.. ... ...
... ...T ... ...T ... .T ... ...
... .T ... G.. ..T ... ... .G. ... ...
... ..C ... ..T ... ..T ..T ..A ... .A ... ...
Microcebus_murinus
... ... TGC AC. ... ... G.. ... ..C .TG ... C.. .G. ... .A. .CT ... .CC .CT G.. ...
T.. ..C .CT ... ...T ... ...C ..C ... ...
..C ... ... A.. ... ... G.. ... .C. ... ...A ... ..G ... ...
... .G ... ... .G. ... ... .GA .C. ... ...
..C G.. ... ...T ... ..C ... ... C.. ..A ... ...C ... ..A ... ..C ...
... ... .AC G.. ... ... C.. ..C ... ... .G. ... ...
... ...C ... ... .A. ..C ..C ... GCT G.. ... A.. ... ... G..
..C ... ... G.. ... ..C ... ..C ... ...C ... T..
... ..A ... ...C ... ...
... ...C ... .C ... ...C ... .G ... ..C ... .C ..C ..T ...
A.. ... ...C ... ...C ... ..A A.G GC. .G. ..C ... ..G .T. ... ...
... ...C ..C ... .CC ... ..G ..C ... ...C ... ...
... ...C ... ..G ... A.. ... ...C ... .T ... ...
... ...G ... A.. ... ... .C. ... ... G..
... ...C ... C.. ... .G. ... ...C ..C ... ..A ..C ... C.. A.. ... ... .GC
... ..C ... ... .T. .GC ... ...T ..C ... ...
..A ... ... T.. ... ...C ... ... .A. ... ...A ... ...
..C ..G .GC ..C G.. ..C ... ... C.. ..G ..G ...
... .G ..C ... .A ... ...C .A. .CC ... ..C ... ...
... ... C.A ..T G.C C.C ... .T. ... ...G ... ...
..G ... ... C.. ..T ..C ... ..T ... ...
... ... .G. C
... ..C ... ... C.. ... .T. ..C
Daubentonia_madagascariensis
... ... TG. .C. ... ... C.C .TG ... C.. ... ... .A. .C. ... .CC .C. G.. ...
T.. ..C A.T .AT ... ...T ... ...C ... ...
..C ... ... G.. ... .C. ... .T ... .G ... ...
... .G ... ... .GA ... ...
..C G.. ... ...T ... ... C.. ... ..T ... .C ... ...
.A. ... .A ... ... .AC G.. ... ... .G. ... ... .G. ... ...
... ... .GC ... ... .A. ..C ..C ... G.T G.. ..T ... ... G..
..C ... ... G.. ... A.C ..C ..C ... ... G.. ... .G
... ..A ... ...C ... ...C ... ...
G.. ... ...C ... .G ... ..C ... .C ..C ..T ...
A.. ... ...C ... ...C .C. ... ..G G.. ... ... .T. ... ...
... ...C ..C ... .C. ... ..G ..T ... ...C ... ...
... ...C ... ..G ... ...C ..T ... ...
... ...G ... A.. ... ... .C. ... ... G..
... ..T ... ..C ... C.. ... ...C ..C ... ..A ..A ... C.A ... ... .GC
... ... .T. .GC ... ..G ... ...T ..C ... ...
..A ... ...C ... ... .A. ... ...A ... ...
... ..G .GC ... G.. ... ... C.. ..A ..G ...
... .G .C ... .A ... ...C .A. .CC ... ...
... ..T ... ... C.A ..T ..C C.. ... .T. ... ...G ... ..T
... ...T ... .T ... ...
... ... .G. C
... ..C ... ...T ... ...T
Otolemur_garnettii
... ... TG. .C. ... ... T.T ... ..C .TG ... C.A ... ... .A. ... ..T ..C .TG G.. ...
T.. T.C ... ... .TG ... ... T.A ..T ... ..T ... ...C ... ... G.G ... ...
... ... A.. ... ... T.. ... .C. ... ...A ... ..C ... ...
... .G ... ... .GA .CA ..C ... ..C ... ...A
T.C G.C ... ...T ... ... C.. ... ... .GA ..A ..C ..C
... .C ..C ... ... .AC G.. ... ... .G. ... ... .G. ..C ... ...
... ... .A. ..C ..C ... G.T G.. ..T ... ... A.. ..T G..
..C ... ...C ... ..A ... G.. ... A.C ..G .T. ... ...
... ..A ... ..T ... ...A ... ...
G.. ... ..T ... ...A ..C ... .A ..C ..C ... ..G ..C ... ..T ...
A.. ... ...C ... ...A ..C ... ... A.. ... ..C ... ..G .T. ... ...
... ...T ... ..C ..C ... .CC ... ..G ..C ... ...C ... ...

```
... ... ... ... ... ..C ... ..C ... ... ... ... ... ... ... ..T ... ... ..T ..T ... ...
... ..C ... ... ..G ... A.. ... ... ... ... ... ... ... .C. ... ... ... ..C ... ... G..
... ... ... ..C ... C.. ... ... ... ... ... ..C ..C ... ..A ..C ..C C.T ... ... ... ..C
AG. ..C ... ... ... ... ... .T. .GC ... ... ... ..C ... ... ... ..A ..C ... ... ... ...
..A ... ..A ... ..C ... ... ... ... ... ..C ... ... ... .A. ... ... ... ... ..A ... ...
..C ..G ..G ..C G.C ... ... ... ... ..A ..C ... ... ... ... ... ... ... C.. ... ..C ...
... ... ..G ..C ... ... ..A ... ... ... ... ..C .A. .CC ... ... ... ..T ... ... ..C ...
... ... ... ... ... ... ... ... C.A ..T G.C C.. ... .CA ... ... ... ... ..C ... ... ...
... ... ..C ... ... ..A ..C ... ... ... ..A G.. T.. ..T ... ... ..T ... ... ... ..C ...
... ... ... ... ..T ... ... ... ... ..C ..T .G. ... ... ... ... ... ... ... ... ..T ...
... ..C ... ... ... ..A ..G ... ..T ... ... ... .T. ...
```

Tree file for model A: catarrhines-A.tree

```
((((((Homo_sapiens, Pan_troglodytes), Gorilla_gorilla_gorilla), Pongo_abelii), ((Macaca_mulatta,
Papio_anubis), Chlorocebus_sabaeus)), (Saimiri_boliviensis, Callithrix_jacchus)), Tarsius_syrichta,
((Microcebus_murinus, Daubentonia_madagascariensis), Otolemur_garnettii));
```

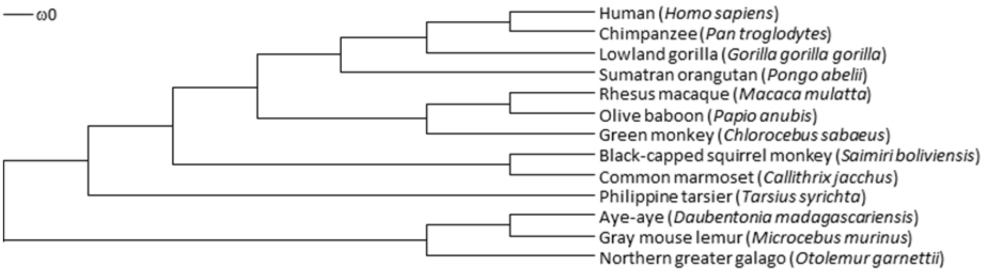

Tree file for model B: catarrhines-B.tree

```
((((((Homo_sapiens, Pan_troglodytes), Gorilla_gorilla_gorilla), Pongo_abelii), ((Macaca_mulatta,
Papio_anubis), Chlorocebus_sabaeus)), (Saimiri_boliviensis, Callithrix_jacchus)), Tarsius_syrichta,
((Microcebus_murinus, Daubentonia_madagascariensis), Otolemur_garnettii));
```

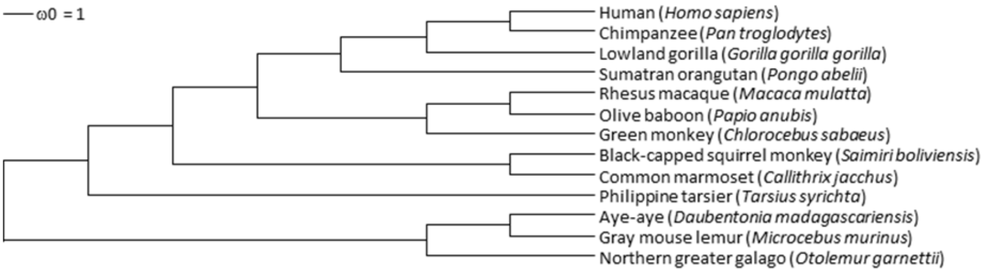

Tree file for model C: catarrhines-C.tree

```
((((((Homo_sapiens: #1, Pan_troglodytes), Gorilla_gorilla_gorilla), Pongo_abelii: #1),
((Macaca_mulatta, Papio_anubis), Chlorocebus_sabaeus)), (Saimiri_boliviensis, Callithrix_jacchus)),
Tarsius_syrichta, ((Microcebus_murinus, Daubentonia_madagascariensis), Otolemur_garnettii));
```

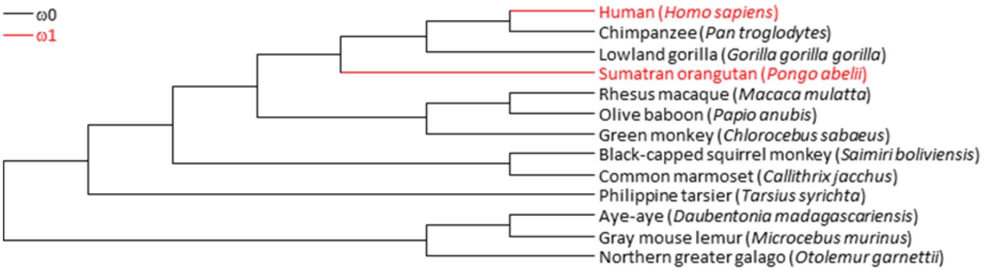

Tree file for model D: catarrhines-D.tree

```
((((((Homo_sapiens: #1, Pan_troglodytes), Gorilla_gorilla_gorilla), Pongo_abelii: #1),
((Macaca_mulatta, Papio_anubis), Chlorocebus_sabaeus)), (Saimiri_boliviensis, Callithrix_jacchus)),
Tarsius_syrichta, ((Microcebus_murinus, Daubentonia_madagascariensis), Otolemur_garnettii));
```

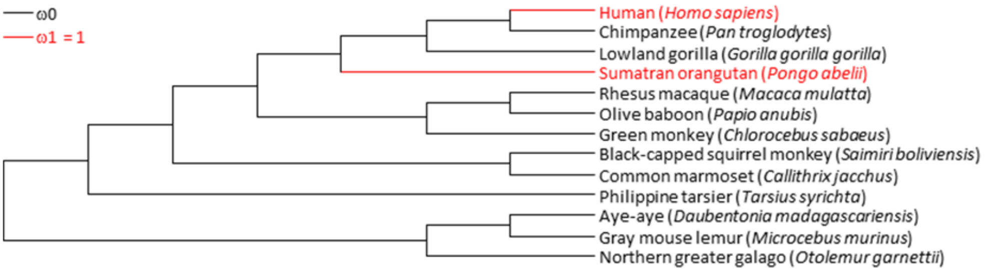

Tree file for model E: catarrhines-E.tree

```
((((((Homo_sapiens: #1, Pan_troglodytes), Gorilla_gorilla_gorilla: #1), Pongo_abelii: #1),
((Macaca_mulatta, Papio_anubis), Chlorocebus_sabaeus)), (Saimiri_boliviensis, Callithrix_jacchus)),
Tarsius_syrichta, ((Microcebus_murinus, Daubentonia_madagascariensis), Otolemur_garnettii));
```

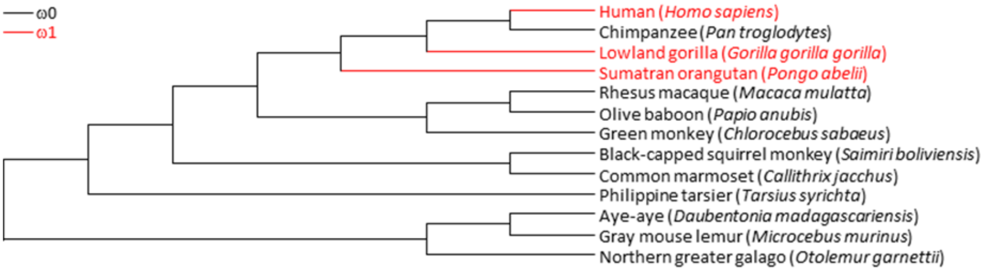

Tree file for model F: catarrhines-F.tree

```
((((((Homo_sapiens: #1, Pan_troglodytes), Gorilla_gorilla_gorilla: #1), Pongo_abelii: #1),
((Macaca_mulatta, Papio_anubis), Chlorocebus_sabaeus)), (Saimiri_boliviensis, Callithrix_jacchus)),
Tarsius_syrichta, ((Microcebus_murinus, Daubentonia_madagascariensis), Otolemur_garnettii));
```

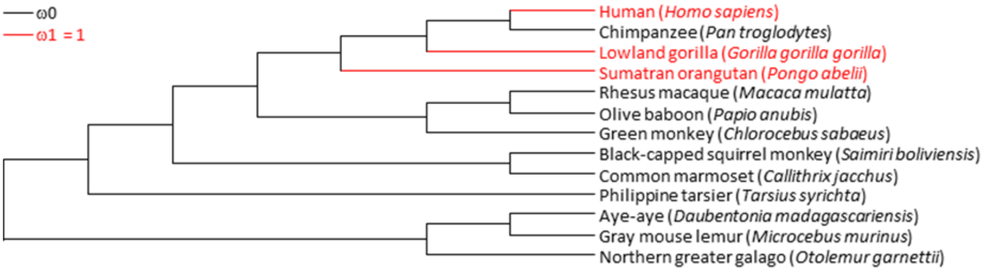

Tree file for model G: catarrhines-G.tree

```
((((((Homo_sapiens: #1, Pan_troglodytes: #1): #1, Gorilla_gorilla_gorilla: #1): #1, Pongo_abelii: #1):
#1, ((Macaca_mulatta, Papio_anubis), Chlorocebus_sabaeus)), (Saimiri_boliviensis, Callithrix_jacchus)),
Tarsius_syrichta, ((Microcebus_murinus, Daubentonia_madagascariensis), Otolemur_garnettii));
```

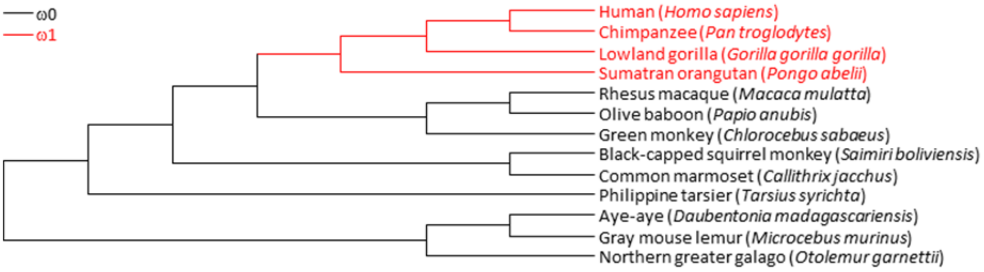

Tree file for model H: catarrhines-H.tree

```
((((((Homo_sapiens: #1, Pan_troglodytes: #1): #1, Gorilla_gorilla_gorilla: #1): #1, Pongo_abelii: #1):
#1, ((Macaca_mulatta, Papio_anubis), Chlorocebus_sabaeus)), (Saimiri_boliviensis, Callithrix_jacchus)),
Tarsius_syrichta, ((Microcebus_murinus, Daubentonia_madagascariensis), Otolemur_garnettii));
```

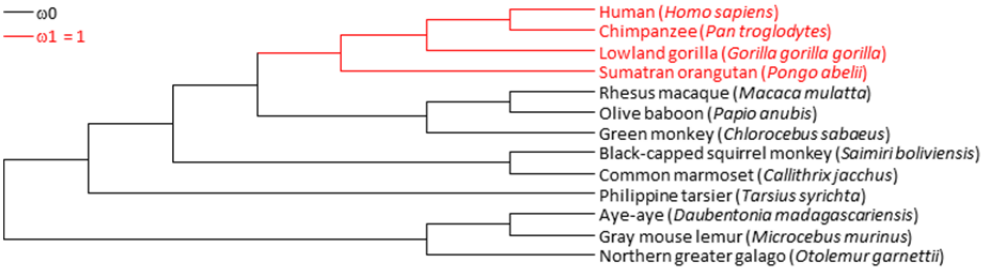

Tree file for model I: catarrhines-I.tree

```
((((((Homo_sapiens: #1, Pan_troglodytes), Gorilla_gorilla_gorilla: #1), Pongo_abelii: #1),
((Macaca_mulatta: #1, Papio_anubis: #1): #1, Chlorocebus_sabaeus: #1): #1), (Saimiri_boliviensis,
Callithrix_jacchus)), Tarsius_syrichta, ((Microcebus_murinus, Daubentonia_madagascariensis),
Otolemur_garnettii));
```

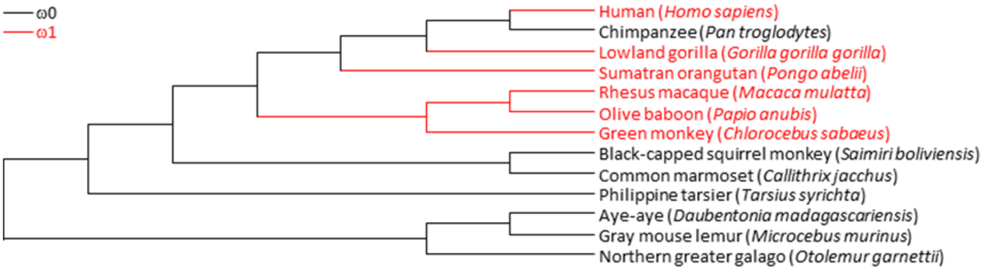

Tree file for model J: catarrhines-J.tree

```
((((((Homo_sapiens: #1, Pan_troglodytes), Gorilla_gorilla_gorilla: #1), Pongo_abelii: #1),
((Macaca_mulatta: #1, Papio_anubis: #1): #1, Chlorocebus_sabaeus: #1): #1), (Saimiri_boliviensis,
Callithrix_jacchus)), Tarsius_syrichta, ((Microcebus_murinus, Daubentonia_madagascariensis),
Otolemur_garnettii));
```

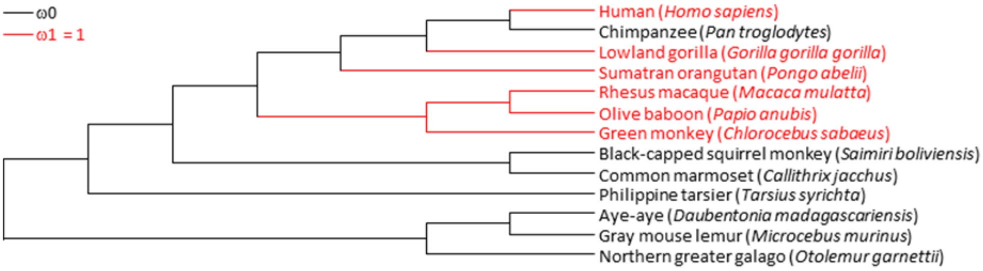

Tree file for model K: catarrhines-K.tree

```
((((((Homo_sapiens: #1, Pan_troglodytes), Gorilla_gorilla_gorilla: #1), Pongo_abelii: #1),
((Macaca_mulatta: #2, Papio_anubis: #2): #2, Chlorocebus_sabaeus: #2): #2), (Saimiri_boliviensis,
Callithrix_jacchus)), Tarsius_syrichta, ((Microcebus_murinus, Daubentonia_madagascariensis),
Otolemur_garnettii));
```

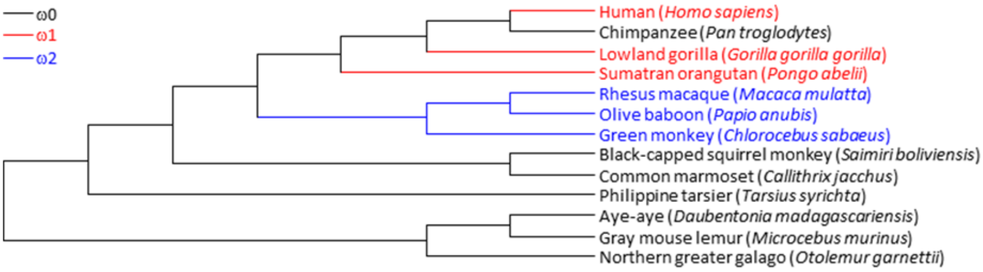

Tree file for model L: catarrhines-L.tree

```
((((((Homo_sapiens, Pan_troglodytes), Gorilla_gorilla_gorilla), Pongo_abelii), ((Macaca_mulatta,
Papio_anubis), Chlorocebus_sabaeus)), (Saimiri_boliviensis, Callithrix_jacchus)), Tarsius_syrichta,
((Microcebus_murinus, Daubentonia_madagascariensis), Otolemur_garnettii));
```

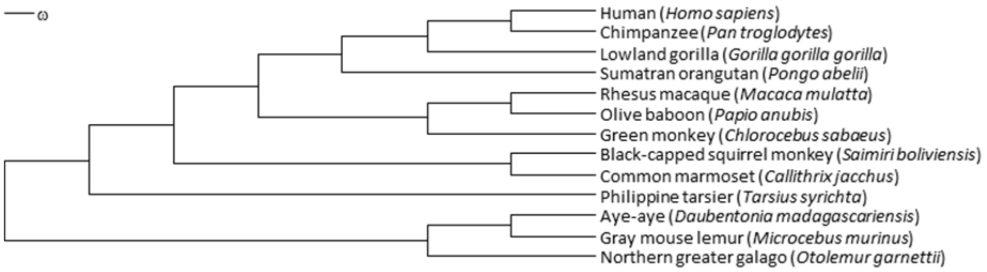

Control file for model A: catarrhines-A.ctl

```
      seqfile = catarrhines.phy
     treefile = catarrhines-A.tree
      outfile = catarrhines-A.mlc

        noisy = 9  * 0,1,2,3,9: how much rubbish on the screen
      verbose = 0  * 0: concise; 1: detailed, 2: too much
      runmode = 0  * 0: user tree;  1: semi-automatic;  2: automatic
                   * 3: StepwiseAddition; (4,5):PerturbationNNI; -2: pairwise

      seqtype = 1  * 1:codons; 2:AAs; 3:codons-->AAs
    CodonFreq = 2  * 0:1/61 each, 1:F1X4, 2:F3X4, 3:codon table
        clock = 0  * 0:no clock, 1:clock; 2:local clock; 3:CombinedAnalysis
        model = 0
                   * models for codons:
                       * 0:one, 1:b, 2:2 or more dN/dS ratios for branches

      NSsites = 0  * 0:one w; 1:neutral; 2:selection; 3:discrete; 4:freqs;
                   * 5:gamma; 6:2gamma; 7:beta; 8:beta&w; 9:beta&gamma;
                   * 10:beta&gamma+1; 11:beta&normal>1; 12:0&2normal>1;
                   * 13:3normal>0
        icode = 0  * 0:universal code; 1:mammalian mt; 2-10:see below

    fix_kappa = 0  * 1: kappa fixed, 0: kappa to be estimated
        kappa = 2  * initial or fixed kappa
    fix_omega = 0  * 1: omega or omega_1 fixed, 0: estimate
        omega = 2  * initial or fixed omega, for codons or codon-based AAs

    fix_alpha = 1  * 0: estimate gamma shape parameter; 1: fix it at alpha
        alpha = .0 * initial or fixed alpha, 0:infinity (constant rate)
       Malpha = 0  * different alphas for genes
        ncatG = 4  * # of categories in dG of NSsites models

        getSE = 0  * 0: don't want them, 1: want S.E.s of estimates
 RateAncestor = 0  * (0,1,2): rates (alpha>0) or ancestral states (1 or 2)
       method = 0  * 0: simultaneous; 1: one branch at a time
```

Control file for model B: catarrhines-B.ctl

```
      seqfile = catarrhines.phy
     treefile = catarrhines-B.tree
      outfile = catarrhines-B.mlc

        noisy = 9  * 0,1,2,3,9: how much rubbish on the screen
      verbose = 0  * 0: concise; 1: detailed, 2: too much
      runmode = 0  * 0: user tree;  1: semi-automatic;  2: automatic
                   * 3: StepwiseAddition; (4,5):PerturbationNNI; -2: pairwise

      seqtype = 1  * 1:codons; 2:AAs; 3:codons-->AAs
    CodonFreq = 2  * 0:1/61 each, 1:F1X4, 2:F3X4, 3:codon table
        clock = 0  * 0:no clock, 1:clock; 2:local clock; 3:CombinedAnalysis
        model = 0
                   * models for codons:
                       * 0:one, 1:b, 2:2 or more dN/dS ratios for branches

      NSsites = 0  * 0:one w; 1:neutral; 2:selection; 3:discrete; 4:freqs;
                   * 5:gamma; 6:2gamma; 7:beta; 8:beta&w; 9:beta&gamma;
                   * 10:beta&gamma+1; 11:beta&normal>1; 12:0&2normal>1;
                   * 13:3normal>0
        icode = 0  * 0:universal code; 1:mammalian mt; 2-10:see below

    fix_kappa = 0  * 1: kappa fixed, 0: kappa to be estimated
        kappa = 2  * initial or fixed kappa
    fix_omega = 1  * 1: omega or omega_1 fixed, 0: estimate
        omega = 1  * initial or fixed omega, for codons or codon-based AAs

    fix_alpha = 1  * 0: estimate gamma shape parameter; 1: fix it at alpha
        alpha = .0 * initial or fixed alpha, 0:infinity (constant rate)
       Malpha = 0  * different alphas for genes
        ncatG = 4  * # of categories in dG of NSsites models

        getSE = 0  * 0: don't want them, 1: want S.E.s of estimates
 RateAncestor = 0  * (0,1,2): rates (alpha>0) or ancestral states (1 or 2)
       method = 0  * 0: simultaneous; 1: one branch at a time
```

Control file for model C: catarrhines-C.ctl

```
      seqfile = catarrhines.phy
     treefile = catarrhines-C.tree
      outfile = catarrhines-C.mlc

        noisy = 9  * 0,1,2,3,9: how much rubbish on the screen
      verbose = 0  * 0: concise; 1: detailed, 2: too much
      runmode = 0  * 0: user tree;  1: semi-automatic;  2: automatic
                   * 3: StepwiseAddition; (4,5):PerturbationNNI; -2: pairwise

      seqtype = 1  * 1:codons; 2:AAs; 3:codons-->AAs
    CodonFreq = 2  * 0:1/61 each, 1:F1X4, 2:F3X4, 3:codon table
        clock = 0  * 0:no clock, 1:clock; 2:local clock; 3:CombinedAnalysis
        model = 2
                   * models for codons:
                       * 0:one, 1:b, 2:2 or more dN/dS ratios for branches

      NSsites = 0  * 0:one w; 1:neutral; 2:selection; 3:discrete; 4:freqs;
                   * 5:gamma; 6:2gamma; 7:beta; 8:beta&w; 9:beta&gamma;
                   * 10:beta&gamma+1; 11:beta&normal>1; 12:0&2normal>1;
                   * 13:3normal>0
        icode = 0  * 0:universal code; 1:mammalian mt; 2-10:see below

    fix_kappa = 0  * 1: kappa fixed, 0: kappa to be estimated
        kappa = 2  * initial or fixed kappa
    fix_omega = 0  * 1: omega or omega_1 fixed, 0: estimate
        omega = 2  * initial or fixed omega, for codons or codon-based AAs

    fix_alpha = 1  * 0: estimate gamma shape parameter; 1: fix it at alpha
        alpha = .0 * initial or fixed alpha, 0:infinity (constant rate)
       Malpha = 0  * different alphas for genes
        ncatG = 4  * # of categories in dG of NSsites models

        getSE = 0  * 0: don't want them, 1: want S.E.s of estimates
 RateAncestor = 0  * (0,1,2): rates (alpha>0) or ancestral states (1 or 2)
       method = 0  * 0: simultaneous; 1: one branch at a time
```

Control file for model D: catarrhines-D.ctl

```
      seqfile = catarrhines.phy
     treefile = catarrhines-D.tree
      outfile = catarrhines-D.mlc

        noisy = 9  * 0,1,2,3,9: how much rubbish on the screen
      verbose = 0  * 0: concise; 1: detailed, 2: too much
      runmode = 0  * 0: user tree;  1: semi-automatic;  2: automatic
                   * 3: StepwiseAddition; (4,5):PerturbationNNI; -2: pairwise

      seqtype = 1  * 1:codons; 2:AAs; 3:codons-->AAs
    CodonFreq = 2  * 0:1/61 each, 1:F1X4, 2:F3X4, 3:codon table
        clock = 0  * 0:no clock, 1:clock; 2:local clock; 3:CombinedAnalysis
        model = 2
                   * models for codons:
                       * 0:one, 1:b, 2:2 or more dN/dS ratios for branches

      NSsites = 0  * 0:one w; 1:neutral; 2:selection; 3:discrete; 4:freqs;
                   * 5:gamma; 6:2gamma; 7:beta; 8:beta&w; 9:beta&gamma;
                   * 10:beta&gamma+1; 11:beta&normal>1; 12:0&2normal>1;
                   * 13:3normal>0
        icode = 0  * 0:universal code; 1:mammalian mt; 2-10:see below

    fix_kappa = 0  * 1: kappa fixed, 0: kappa to be estimated
        kappa = 2  * initial or fixed kappa
    fix_omega = 1  * 1: omega or omega_1 fixed, 0: estimate
        omega = 1  * initial or fixed omega, for codons or codon-based AAs

    fix_alpha = 1  * 0: estimate gamma shape parameter; 1: fix it at alpha
        alpha = .0 * initial or fixed alpha, 0:infinity (constant rate)
       Malpha = 0  * different alphas for genes
        ncatG = 4  * # of categories in dG of NSsites models

        getSE = 0  * 0: don't want them, 1: want S.E.s of estimates
 RateAncestor = 0  * (0,1,2): rates (alpha>0) or ancestral states (1 or 2)
       method = 0  * 0: simultaneous; 1: one branch at a time
```

Control file for model E: catarrhines-E.ctl

```
      seqfile = catarrhines.phy
     treefile = catarrhines-E.tree
      outfile = catarrhines-E.mlc

        noisy = 9  * 0,1,2,3,9: how much rubbish on the screen
      verbose = 0  * 0: concise; 1: detailed, 2: too much
      runmode = 0  * 0: user tree;  1: semi-automatic;  2: automatic
                   * 3: StepwiseAddition; (4,5):PerturbationNNI; -2: pairwise

      seqtype = 1  * 1:codons; 2:AAs; 3:codons-->AAs
    CodonFreq = 2  * 0:1/61 each, 1:F1X4, 2:F3X4, 3:codon table
        clock = 0  * 0:no clock, 1:clock; 2:local clock; 3:CombinedAnalysis
        model = 2
                   * models for codons:
                       * 0:one, 1:b, 2:2 or more dN/dS ratios for branches

      NSsites = 0  * 0:one w; 1:neutral; 2:selection; 3:discrete; 4:freqs;
                   * 5:gamma; 6:2gamma; 7:beta; 8:beta&w; 9:beta&gamma;
                   * 10:beta&gamma+1; 11:beta&normal>1; 12:0&2normal>1;
                   * 13:3normal>0
        icode = 0  * 0:universal code; 1:mammalian mt; 2-10:see below

    fix_kappa = 0  * 1: kappa fixed, 0: kappa to be estimated
        kappa = 2  * initial or fixed kappa
    fix_omega = 0  * 1: omega or omega_1 fixed, 0: estimate
        omega = 2  * initial or fixed omega, for codons or codon-based AAs

    fix_alpha = 1  * 0: estimate gamma shape parameter; 1: fix it at alpha
        alpha = .0 * initial or fixed alpha, 0:infinity (constant rate)
       Malpha = 0  * different alphas for genes
        ncatG = 4  * # of categories in dG of NSsites models

        getSE = 0  * 0: don't want them, 1: want S.E.s of estimates
 RateAncestor = 0  * (0,1,2): rates (alpha>0) or ancestral states (1 or 2)
       method = 0  * 0: simultaneous; 1: one branch at a time
```

Control file for model F: catarrhines-F.ctl

```
      seqfile = catarrhines.phy
     treefile = catarrhines-F.tree
      outfile = catarrhines-F.mlc

        noisy = 9  * 0,1,2,3,9: how much rubbish on the screen
      verbose = 0  * 0: concise; 1: detailed, 2: too much
      runmode = 0  * 0: user tree;  1: semi-automatic;  2: automatic
                   * 3: StepwiseAddition; (4,5):PerturbationNNI; -2: pairwise

      seqtype = 1  * 1:codons; 2:AAs; 3:codons-->AAs
    CodonFreq = 2  * 0:1/61 each, 1:F1X4, 2:F3X4, 3:codon table
        clock = 0  * 0:no clock, 1:clock; 2:local clock; 3:CombinedAnalysis
        model = 2
                   * models for codons:
                       * 0:one, 1:b, 2:2 or more dN/dS ratios for branches

      NSsites = 0  * 0:one w; 1:neutral; 2:selection; 3:discrete; 4:freqs;
                   * 5:gamma; 6:2gamma; 7:beta; 8:beta&w; 9:beta&gamma;
                   * 10:beta&gamma+1; 11:beta&normal>1; 12:0&2normal>1;
                   * 13:3normal>0
        icode = 0  * 0:universal code; 1:mammalian mt; 2-10:see below

    fix_kappa = 0  * 1: kappa fixed, 0: kappa to be estimated
        kappa = 2  * initial or fixed kappa
    fix_omega = 1  * 1: omega or omega_1 fixed, 0: estimate
        omega = 1  * initial or fixed omega, for codons or codon-based AAs

    fix_alpha = 1  * 0: estimate gamma shape parameter; 1: fix it at alpha
        alpha = .0 * initial or fixed alpha, 0:infinity (constant rate)
       Malpha = 0  * different alphas for genes
        ncatG = 4  * # of categories in dG of NSsites models

        getSE = 0  * 0: don't want them, 1: want S.E.s of estimates
 RateAncestor = 0  * (0,1,2): rates (alpha>0) or ancestral states (1 or 2)
       method = 0  * 0: simultaneous; 1: one branch at a time
```

Control file for model G: catarrhines-G.ctl

```
      seqfile = catarrhines.phy
     treefile = catarrhines-G.tree
      outfile = catarrhines-G.mlc

        noisy = 9  * 0,1,2,3,9: how much rubbish on the screen
      verbose = 0  * 0: concise; 1: detailed, 2: too much
      runmode = 0  * 0: user tree;  1: semi-automatic;  2: automatic
                   * 3: StepwiseAddition; (4,5):PerturbationNNI; -2: pairwise

      seqtype = 1  * 1:codons; 2:AAs; 3:codons-->AAs
    CodonFreq = 2  * 0:1/61 each, 1:F1X4, 2:F3X4, 3:codon table
        clock = 0  * 0:no clock, 1:clock; 2:local clock; 3:CombinedAnalysis
        model = 2
                   * models for codons:
                       * 0:one, 1:b, 2:2 or more dN/dS ratios for branches

      NSsites = 0  * 0:one w; 1:neutral; 2:selection; 3:discrete; 4:freqs;
                   * 5:gamma; 6:2gamma; 7:beta; 8:beta&w; 9:beta&gamma;
                   * 10:beta&gamma+1; 11:beta&normal>1; 12:0&2normal>1;
                   * 13:3normal>0
        icode = 0  * 0:universal code; 1:mammalian mt; 2-10:see below

    fix_kappa = 0  * 1: kappa fixed, 0: kappa to be estimated
        kappa = 2  * initial or fixed kappa
    fix_omega = 0  * 1: omega or omega_1 fixed, 0: estimate
        omega = 2  * initial or fixed omega, for codons or codon-based AAs

    fix_alpha = 1  * 0: estimate gamma shape parameter; 1: fix it at alpha
        alpha = .0 * initial or fixed alpha, 0:infinity (constant rate)
       Malpha = 0  * different alphas for genes
        ncatG = 4  * # of categories in dG of NSsites models

        getSE = 0  * 0: don't want them, 1: want S.E.s of estimates
 RateAncestor = 0  * (0,1,2): rates (alpha>0) or ancestral states (1 or 2)
       method = 0  * 0: simultaneous; 1: one branch at a time
```

Control file for model H: catarrhines-H.ctl

```
      seqfile = catarrhines.phy
     treefile = catarrhines-H.tree
      outfile = catarrhines-H.mlc

        noisy = 9  * 0,1,2,3,9: how much rubbish on the screen
      verbose = 0  * 0: concise; 1: detailed, 2: too much
      runmode = 0  * 0: user tree;  1: semi-automatic;  2: automatic
                   * 3: StepwiseAddition; (4,5):PerturbationNNI; -2: pairwise

      seqtype = 1  * 1:codons; 2:AAs; 3:codons-->AAs
    CodonFreq = 2  * 0:1/61 each, 1:F1X4, 2:F3X4, 3:codon table
        clock = 0  * 0:no clock, 1:clock; 2:local clock; 3:CombinedAnalysis
        model = 2
                   * models for codons:
                       * 0:one, 1:b, 2:2 or more dN/dS ratios for branches

      NSsites = 0  * 0:one w; 1:neutral; 2:selection; 3:discrete; 4:freqs;
                   * 5:gamma; 6:2gamma; 7:beta; 8:beta&w; 9:beta&gamma;
                   * 10:beta&gamma+1; 11:beta&normal>1; 12:0&2normal>1;
                   * 13:3normal>0
        icode = 0  * 0:universal code; 1:mammalian mt; 2-10:see below

    fix_kappa = 0  * 1: kappa fixed, 0: kappa to be estimated
        kappa = 2  * initial or fixed kappa
    fix_omega = 1  * 1: omega or omega_1 fixed, 0: estimate
        omega = 1  * initial or fixed omega, for codons or codon-based AAs

    fix_alpha = 1  * 0: estimate gamma shape parameter; 1: fix it at alpha
        alpha = .0 * initial or fixed alpha, 0:infinity (constant rate)
       Malpha = 0  * different alphas for genes
        ncatG = 4  * # of categories in dG of NSsites models

        getSE = 0  * 0: don't want them, 1: want S.E.s of estimates
 RateAncestor = 0  * (0,1,2): rates (alpha>0) or ancestral states (1 or 2)
       method = 0  * 0: simultaneous; 1: one branch at a time
```

Control file for model I: catarrhines-I.ctl

```
      seqfile = catarrhines.phy
     treefile = catarrhines-I.tree
      outfile = catarrhines-I.mlc

        noisy = 9  * 0,1,2,3,9: how much rubbish on the screen
      verbose = 0  * 0: concise; 1: detailed, 2: too much
      runmode = 0  * 0: user tree;  1: semi-automatic;  2: automatic
                   * 3: StepwiseAddition; (4,5):PerturbationNNI; -2: pairwise

      seqtype = 1  * 1:codons; 2:AAs; 3:codons-->AAs
    CodonFreq = 2  * 0:1/61 each, 1:F1X4, 2:F3X4, 3:codon table
        clock = 0  * 0:no clock, 1:clock; 2:local clock; 3:CombinedAnalysis
        model = 2
                   * models for codons:
                       * 0:one, 1:b, 2:2 or more dN/dS ratios for branches

      NSsites = 0  * 0:one w; 1:neutral; 2:selection; 3:discrete; 4:freqs;
                   * 5:gamma; 6:2gamma; 7:beta; 8:beta&w; 9:beta&gamma;
                   * 10:beta&gamma+1; 11:beta&normal>1; 12:0&2normal>1;
                   * 13:3normal>0
        icode = 0  * 0:universal code; 1:mammalian mt; 2-10:see below

    fix_kappa = 0  * 1: kappa fixed, 0: kappa to be estimated
        kappa = 2  * initial or fixed kappa
    fix_omega = 0  * 1: omega or omega_1 fixed, 0: estimate
        omega = 2  * initial or fixed omega, for codons or codon-based AAs

    fix_alpha = 1  * 0: estimate gamma shape parameter; 1: fix it at alpha
        alpha = .0 * initial or fixed alpha, 0:infinity (constant rate)
       Malpha = 0  * different alphas for genes
        ncatG = 4  * # of categories in dG of NSsites models

        getSE = 0  * 0: don't want them, 1: want S.E.s of estimates
 RateAncestor = 0  * (0,1,2): rates (alpha>0) or ancestral states (1 or 2)
       method = 0  * 0: simultaneous; 1: one branch at a time
```

Control file for model J: catarrhines-J.ctl

```
      seqfile = catarrhines.phy
     treefile = catarrhines-J.tree
      outfile = catarrhines-J.mlc

        noisy = 9  * 0,1,2,3,9: how much rubbish on the screen
      verbose = 0  * 0: concise; 1: detailed, 2: too much
      runmode = 0  * 0: user tree;  1: semi-automatic;  2: automatic
                   * 3: StepwiseAddition; (4,5):PerturbationNNI; -2: pairwise

      seqtype = 1  * 1:codons; 2:AAs; 3:codons-->AAs
    CodonFreq = 2  * 0:1/61 each, 1:F1X4, 2:F3X4, 3:codon table
        clock = 0  * 0:no clock, 1:clock; 2:local clock; 3:CombinedAnalysis
        model = 2
                   * models for codons:
                       * 0:one, 1:b, 2:2 or more dN/dS ratios for branches

      NSsites = 0  * 0:one w; 1:neutral; 2:selection; 3:discrete; 4:freqs;
                   * 5:gamma; 6:2gamma; 7:beta; 8:beta&w; 9:beta&gamma;
                   * 10:beta&gamma+1; 11:beta&normal>1; 12:0&2normal>1;
                   * 13:3normal>0
        icode = 0  * 0:universal code; 1:mammalian mt; 2-10:see below

    fix_kappa = 0  * 1: kappa fixed, 0: kappa to be estimated
        kappa = 2  * initial or fixed kappa
    fix_omega = 1  * 1: omega or omega_1 fixed, 0: estimate
        omega = 1  * initial or fixed omega, for codons or codon-based AAs

    fix_alpha = 1  * 0: estimate gamma shape parameter; 1: fix it at alpha
        alpha = .0 * initial or fixed alpha, 0:infinity (constant rate)
       Malpha = 0  * different alphas for genes
        ncatG = 4  * # of categories in dG of NSsites models

        getSE = 0  * 0: don't want them, 1: want S.E.s of estimates
 RateAncestor = 0  * (0,1,2): rates (alpha>0) or ancestral states (1 or 2)
       method = 0  * 0: simultaneous; 1: one branch at a time
```

Control file for model K: catarrhines-K.ctl

```
      seqfile = catarrhines.phy
     treefile = catarrhines-K.tree
      outfile = catarrhines-K.mlc

        noisy = 9  * 0,1,2,3,9: how much rubbish on the screen
      verbose = 0  * 0: concise; 1: detailed, 2: too much
      runmode = 0  * 0: user tree;  1: semi-automatic;  2: automatic
                   * 3: StepwiseAddition; (4,5):PerturbationNNI; -2: pairwise

      seqtype = 1  * 1:codons; 2:AAs; 3:codons-->AAs
    CodonFreq = 2  * 0:1/61 each, 1:F1X4, 2:F3X4, 3:codon table
        clock = 0  * 0:no clock, 1:clock; 2:local clock; 3:CombinedAnalysis
        model = 2
                   * models for codons:
                       * 0:one, 1:b, 2:2 or more dN/dS ratios for branches

      NSsites = 0  * 0:one w; 1:neutral; 2:selection; 3:discrete; 4:freqs;
                   * 5:gamma; 6:2gamma; 7:beta; 8:beta&w; 9:beta&gamma;
                   * 10:beta&gamma+1; 11:beta&normal>1; 12:0&2normal>1;
                   * 13:3normal>0
        icode = 0  * 0:universal code; 1:mammalian mt; 2-10:see below

    fix_kappa = 0  * 1: kappa fixed, 0: kappa to be estimated
        kappa = 2  * initial or fixed kappa
    fix_omega = 0  * 1: omega or omega_1 fixed, 0: estimate
        omega = 2  * initial or fixed omega, for codons or codon-based AAs

    fix_alpha = 1  * 0: estimate gamma shape parameter; 1: fix it at alpha
        alpha = .0 * initial or fixed alpha, 0:infinity (constant rate)
       Malpha = 0  * different alphas for genes
        ncatG = 4  * # of categories in dG of NSsites models

        getSE = 0  * 0: don't want them, 1: want S.E.s of estimates
 RateAncestor = 0  * (0,1,2): rates (alpha>0) or ancestral states (1 or 2)
       method = 0  * 0: simultaneous; 1: one branch at a time
```

Control file for model L: catarrhines-L.ctl

```
      seqfile = catarrhines.phy
     treefile = catarrhines-L.tree
      outfile = catarrhines-L.mlc

        noisy = 9  * 0,1,2,3,9: how much rubbish on the screen
      verbose = 0  * 0: concise; 1: detailed, 2: too much
      runmode = 0  * 0: user tree;  1: semi-automatic;  2: automatic
                   * 3: StepwiseAddition; (4,5):PerturbationNNI; -2: pairwise

      seqtype = 1  * 1:codons; 2:AAs; 3:codons-->AAs
    CodonFreq = 2  * 0:1/61 each, 1:F1X4, 2:F3X4, 3:codon table
        clock = 0  * 0:no clock, 1:clock; 2:local clock; 3:CombinedAnalysis
        model = 1
                   * models for codons:
                       * 0:one, 1:b, 2:2 or more dN/dS ratios for branches

      NSsites = 0  * 0:one w; 1:neutral; 2:selection; 3:discrete; 4:freqs;
                   * 5:gamma; 6:2gamma; 7:beta; 8:beta&w; 9:beta&gamma;
                   * 10:beta&gamma+1; 11:beta&normal>1; 12:0&2normal>1;
                   * 13:3normal>0
        icode = 0  * 0:universal code; 1:mammalian mt; 2-10:see below

    fix_kappa = 0  * 1: kappa fixed, 0: kappa to be estimated
        kappa = 2  * initial or fixed kappa
    fix_omega = 0  * 1: omega or omega_1 fixed, 0: estimate
        omega = 2  * initial or fixed omega, for codons or codon-based AAs

    fix_alpha = 1  * 0: estimate gamma shape parameter; 1: fix it at alpha
        alpha = .0 * initial or fixed alpha, 0:infinity (constant rate)
       Malpha = 0  * different alphas for genes
        ncatG = 4  * # of categories in dG of NSsites models

        getSE = 0  * 0: don't want them, 1: want S.E.s of estimates
 RateAncestor = 0  * (0,1,2): rates (alpha>0) or ancestral states (1 or 2)
       method = 0  * 0: simultaneous; 1: one branch at a time
```

Main result file for model A: catarrhines-A.mlc

```
CODONML (in paml version 4.7b, October 2013)  catarrhines.phy
Model: One dN/dS ratio for branches,
Codon frequency model: F3x4
ns =  13  ls = 498

Codon usage in sequences
--------------------------------------------------------------------------------------------------------------------------------------
Phe TTT  13  13  13  14  13  13 | Ser TCT   5   5   5   7   4   4 | Tyr TAT  11  10  11  10   9   8 | Cys TGT   3   4   4   6   5   5
    TTC  15  15  15  13  15  15 |     TCC  11  11  11   9  11  11 |     TAC  16  16  15  15  17  18 |     TGC   8   6   6   7   5   5
Leu TTA   1   1   1   2   2   2 |     TCA   2   2   2   3   2   2 | *** TAA   0   0   0   0   0   0 | *** TGA   0   0   0   0   0   0
    TTG   9   9   9   8   9   9 |     TCG   2   2   2   1   2   2 |     TAG   0   0   0   0   0   0 | Trp TGG   4   4   4   4   4   4
--------------------------------------------------------------------------------------------------------------------------------------
Leu CTT  10   9   9   8   6   6 | Pro CCT  13  14  15  12  10  10 | His CAT   6   6   6   6   6   6 | Arg CGT   4   4   3   3   4   3
    CTC   8   8   8  10   9   9 |     CCC  10  10   9  11  13  14 |     CAC  11  10  10  11  10  10 |     CGC   6   8   8   5   7   8
    CTA   2   2   2   2   2   2 |     CCA   3   3   3   3   2   2 | Gln CAA   5   4   4   4   4   4 |     CGA   3   5   5   5   5   5
    CTG  27  27  27  27  30  30 |     CCG   2   2   2   2   4   3 |     CAG  11  11  12  13  13  13 |     CGG   3   2   2   2   1   1
--------------------------------------------------------------------------------------------------------------------------------------
Ile ATT  10  10  10   9  11  10 | Thr ACT   6   6   6   9   9   9 | Asn AAT  12  11  12  11  14  12 | Ser AGT   2   2   3   2   3   3
    ATC  17  17  18  18  16  17 |     ACC  11  10  10   9   8   9 |     AAC   7   6   6   6   4   6 |     AGC  10  10  10  10   9   9
    ATA   1   1   0   1   1   1 |     ACA   9   9   9   8  11  10 | Lys AAA   3   3   3   3   3   3 | Arg AGA   1   0   0   1   0   0
Met ATG   8   9   9  11   8   8 |     ACG   7   6   6   4   3   3 |     AAG   8   8   8   8   9   8 |     AGG   6   6   6   7   6   6
--------------------------------------------------------------------------------------------------------------------------------------
Val GTT   3   3   3   3   4   5 | Ala GCT  10  10  10  11   9   9 | Asp GAT  17  18  17  17  21  21 | Gly GGT   2   3   3   2   4   4
    GTC  16  15  15  15  15  14 |     GCC   8  10   9   9  12  12 |     GAC  23  24  24  24  20  20 |     GGC  14  14  14  15  13  13
    GTA   2   3   2   3   2   2 |     GCA   2   2   2   2   2   2 | Glu GAA   5   5   5   4   5   4 |     GGA   9   9   9  10   9   9
    GTG  15  14  16  14  15  15 |     GCG   0   0   0   0   0   0 |     GAG  22  22  21  21  20  22 |     GGG   8   9   9   8   8   8
--------------------------------------------------------------------------------------------------------------------------------------


--------------------------------------------------------------------------------------------------------------------------------------
Phe TTT  13  13  12  16  10  12 | Ser TCT   4   3   4   4   4   4 | Tyr TAT   9  12  12  11   5   6 | Cys TGT   4   4   6   7   2   4
    TTC  15  15  15  15  17  14 |     TCC  11  11  11  13  14  14 |     TAC  17  14  14  14  20  19 |     TGC   6   7   5   4  10   8
Leu TTA   2   1   1   0   2   0 |     TCA   2   2   2   2   1   1 | *** TAA   0   0   0   0   0   0 | *** TGA   0   0   0   0   0   0
    TTG   9  11  10   9   5   6 |     TCG   2   2   2   3   2   2 |     TAG   0   0   0   0   0   0 | Trp TGG   4   4   4   4   4   4
--------------------------------------------------------------------------------------------------------------------------------------
Leu CTT   6  10  11   5   4   7 | Pro CCT  10  11  11  12   9   9 | His CAT   6   6   7   8   5   6 | Arg CGT   3   3   2   4   4   2
    CTC   9   9   8  12  12  11 |     CCC  14  13  12  10  15  12 |     CAC  10   9   9   8  12  12 |     CGC   8   7   7   6   9   8
    CTA   3   2   2   4   2   2 |     CCA   2   2   2   4   2   3 | Gln CAA   5   3   4   3   5   3 |     CGA   4   5   5   6   3   5
    CTG  29  25  27  25  30  30 |     CCG   3   2   2   1   3   3 |     CAG  12  14  12  12  12  13 |     CGG   2   1   2   3   3   3
--------------------------------------------------------------------------------------------------------------------------------------
Ile ATT   9  11  10   8   4   5 | Thr ACT   9   9   9   9   6   8 | Asn AAT  12  12  11  11   5   9 | Ser AGT   3   5   5   5   3   3
    ATC  17  17  18  19  22  19 |     ACC   9   7   7  10  14  12 |     AAC   6   5   5   5   9   7 |     AGC   9   7   8   5   8   7
    ATA   1   1   1   4   2   2 |     ACA   9   9  10   5   6   6 | Lys AAA   3   2   2   4   3   2 | Arg AGA   1   0   0   2   2   2
Met ATG   9   9  10  10  11   9 |     ACG   4   5   4   5   6   6 |     AAG   8   7   7  10  10   9 |     AGG   6   7   7   4   3   4
--------------------------------------------------------------------------------------------------------------------------------------
Val GTT   3   7   8   6   4   4 | Ala GCT   9  12  12  10   9   7 | Asp GAT  20  20  20  19  11  17 | Gly GGT   4   4   3   6   3   5
    GTC  16  12   9  10  12  14 |     GCC  11  12  12   9  13  13 |     GAC  21  22  22  24  31  25 |     GGC  14  13  13  14  18  15
    GTA   2   2   2   4   3   2 |     GCA   2   2   2   2   0   3 | Glu GAA   4   5   6  11   5   6 |     GGA   8   8   9   7   6   7
    GTG  15  14  15  13  14  18 |     GCG   0   0   1   0   2   0 |     GAG  21  22  21  14  17  19 |     GGG   9   9   8   8  10  10
--------------------------------------------------------------------------------------------------------------------------------------


------------------------------------------------------
Phe TTT  10 | Ser TCT   3 | Tyr TAT   3 | Cys TGT   5
    TTC  20 |     TCC  12 |     TAC  22 |     TGC   8
Leu TTA   2 |     TCA   2 | *** TAA   0 | *** TGA   0
    TTG   6 |     TCG   1 |     TAG   0 | Trp TGG   4
------------------------------------------------------
Leu CTT   7 | Pro CCT  13 | His CAT   6 | Arg CGT   4
    CTC  10 |     CCC  10 |     CAC  11 |     CGC   7
    CTA   4 |     CCA   2 | Gln CAA   5 |     CGA   3
    CTG  26 |     CCG   2 |     CAG  11 |     CGG   3
------------------------------------------------------
Ile ATT   5 | Thr ACT   7 | Asn AAT   6 | Ser AGT   3
    ATC  20 |     ACC  13 |     AAC  10 |     AGC   7
    ATA   2 |     ACA   6 | Lys AAA   3 | Arg AGA   3
Met ATG  10 |     ACG   4 |     AAG   9 |     AGG   3
------------------------------------------------------
Val GTT   5 | Ala GCT   7 | Asp GAT  11 | Gly GGT   5
    GTC  14 |     GCC  15 |     GAC  28 |     GGC  19
    GTA   4 |     GCA   3 | Glu GAA  11 |     GGA   5
    GTG  14 |     GCG   1 |     GAG  15 |     GGG   8
------------------------------------------------------

Codon position x base (3x4) table for each sequence.

#1: Homo_sapiens
position  1:    T:0.20080    C:0.24900    A:0.23695    G:0.31325
position  2:    T:0.31526    C:0.20281    A:0.31526    G:0.16667
position  3:    T:0.25502    C:0.38353    A:0.09639    G:0.26506
Average         T:0.25703    C:0.27845    A:0.21620    G:0.24833

#2: Pan_troglodytes
position  1:    T:0.19679    C:0.25100    A:0.22892    G:0.32329
position  2:    T:0.31325    C:0.20482    A:0.30924    G:0.17269
position  3:    T:0.25703    C:0.38153    A:0.09839    G:0.26305
Average         T:0.25569    C:0.27912    A:0.21218    G:0.25301
```

```
#3: Gorilla_gorilla_gorilla
position  1:    T:0.19679    C:0.25100    A:0.23293    G:0.31928
position  2:    T:0.31526    C:0.20281    A:0.30924    G:0.17269
position  3:    T:0.26104    C:0.37751    A:0.09438    G:0.26707
Average         T:0.25770    C:0.27711    A:0.21218    G:0.25301

#4: Pongo_abelii
position  1:    T:0.19880    C:0.24900    A:0.23494    G:0.31727
position  2:    T:0.31727    C:0.20080    A:0.30723    G:0.17470
position  3:    T:0.26104    C:0.37550    A:0.10241    G:0.26104
Average         T:0.25904    C:0.27510    A:0.21486    G:0.25100

#5: Macaca_mulatta
position  1:    T:0.19679    C:0.25301    A:0.23092    G:0.31928
position  2:    T:0.31727    C:0.20482    A:0.31124    G:0.16667
position  3:    T:0.26506    C:0.36948    A:0.10040    G:0.26506
Average         T:0.25971    C:0.27577    A:0.21419    G:0.25033

#6: Papio_anubis
position  1:    T:0.19679    C:0.25301    A:0.22892    G:0.32129
position  2:    T:0.31727    C:0.20482    A:0.31124    G:0.16667
position  3:    T:0.25703    C:0.38153    A:0.09639    G:0.26506
Average         T:0.25703    C:0.27979    A:0.21218    G:0.25100

#7: Chlorocebus_sabaeus
position  1:    T:0.19679    C:0.25301    A:0.23092    G:0.31928
position  2:    T:0.31727    C:0.20281    A:0.30924    G:0.17068
position  3:    T:0.24900    C:0.38755    A:0.09639    G:0.26707
Average         T:0.25435    C:0.28112    A:0.21218    G:0.25234

#8: Saimiri_boliviensis
position  1:    T:0.19880    C:0.24498    A:0.22691    G:0.32932
position  2:    T:0.31928    C:0.20482    A:0.30723    G:0.16867
position  3:    T:0.28514    C:0.36145    A:0.08835    G:0.26506
Average         T:0.26774    C:0.27041    A:0.20750    G:0.25435

#9: Callithrix_jacchus
position  1:    T:0.19679    C:0.24699    A:0.22892    G:0.32731
position  2:    T:0.31928    C:0.20683    A:0.30522    G:0.16867
position  3:    T:0.28715    C:0.35141    A:0.09639    G:0.26506
Average         T:0.26774    C:0.26841    A:0.21017    G:0.25368

#10: Tarsius_syrichta
position  1:    T:0.20482    C:0.24699    A:0.23293    G:0.31526
position  2:    T:0.32129    C:0.19880    A:0.30924    G:0.17068
position  3:    T:0.28313    C:0.35743    A:0.11647    G:0.24297
Average         T:0.26975    C:0.26774    A:0.21954    G:0.24297

#11: Microcebus_murinus
position  1:    T:0.19277    C:0.26104    A:0.22892    G:0.31727
position  2:    T:0.30924    C:0.21285    A:0.30120    G:0.17671
position  3:    T:0.17671    C:0.47390    A:0.08434    G:0.26506
Average         T:0.22624    C:0.31593    A:0.20482    G:0.25301

#12: Daubentonia_madagascariensis
position  1:    T:0.18876    C:0.25904    A:0.22088    G:0.33133
position  2:    T:0.31124    C:0.20683    A:0.30723    G:0.17470
position  3:    T:0.21687    C:0.42169    A:0.08835    G:0.27309
Average         T:0.23896    C:0.29585    A:0.20549    G:0.25971

#13: Otolemur_garnettii
position  1:    T:0.19679    C:0.24900    A:0.22289    G:0.33133
position  2:    T:0.31928    C:0.20281    A:0.30321    G:0.17470
position  3:    T:0.20080    C:0.45382    A:0.11044    G:0.23494
Average         T:0.23896    C:0.30187    A:0.21218    G:0.24699

Sums of codon usage counts
------------------------------------------------------------------------------
Phe F TTT     165 | Ser S TCT      56 | Tyr Y TAT     117 | Cys C TGT      59
      TTC     199 |       TCC     150 |       TAC     217 |       TGC      85
Leu L TTA      17 |       TCA      25 | *** * TAA       0 | *** * TGA       0
      TTG     109 |       TCG      25 |       TAG       0 | Trp W TGG      52
------------------------------------------------------------------------------
Leu L CTT      98 | Pro P CCT     149 | His H CAT      80 | Arg R CGT      43
      CTC     123 |       CCC     153 |       CAC     133 |       CGC      94
      CTA      31 |       CCA      33 | Gln Q CAA      53 |       CGA      59
      CTG     360 |       CCG      31 |       CAG     159 |       CGG      28
```

```
------------------------------------------------------------------------------
Ile I ATT     112 | Thr T ACT     102 | Asn N AAT     138 | Ser S AGT      42
      ATC     235 |       ACC     129 |       AAC      82 |       AGC     109
      ATA      18 |       ACA     107 | Lys K AAA      37 | Arg R AGA      12
Met M ATG     121 |       ACG      63 |       AAG     109 |       AGG      71
------------------------------------------------------------------------------
Val V GTT      58 | Ala A GCT     125 | Asp D GAT     229 | Gly G GGT      48
      GTC     177 |       GCC     145 |       GAC     308 |       GGC     189
      GTA      33 |       GCA      26 | Glu E GAA      76 |       GGA     105
      GTG     192 |       GCG       4 |       GAG     257 |       GGG     112
------------------------------------------------------------------------------


Codon position x base (3x4) table, overall

position  1:    T:0.19710    C:0.25131    A:0.22969    G:0.32190
position  2:    T:0.31634    C:0.20436    A:0.30816    G:0.17115
position  3:    T:0.25039    C:0.39049    A:0.09762    G:0.26151
Average         T:0.25461    C:0.28205    A:0.21182    G:0.25152


Nei & Gojobori 1986. dN/dS (dN, dS)
(Note: This matrix is not used in later ML. analysis.
Use runmode = -2 for ML pairwise comparison.)

Homo_sapiens
Pan_troglodytes      1.2604 (0.0106 0.0084)
Gorilla_gorilla_gorilla 1.3389 (0.0151 0.0113) 0.7351 (0.0062 0.0084)
Pongo_abelii         0.4378 (0.0296 0.0676) 0.2910 (0.0187 0.0643) 0.3714 (0.0250 0.0674)
Macaca_mulatta       0.2054 (0.0269 0.1309) 0.1261 (0.0160 0.1271) 0.1712 (0.0224 0.1306) 0.2017
(0.0278 0.1377)
Papio_anubis         0.2052 (0.0242 0.1177) 0.1169 (0.0133 0.1141) 0.1672 (0.0196 0.1174) 0.1773
(0.0232 0.1311) 0.2434 (0.0062 0.0255)
Chlorocebus_sabaeus  0.2404 (0.0260 0.1080) 0.1448 (0.0151 0.1045) 0.1989 (0.0214 0.1078) 0.2216
(0.0269 0.1212) 0.2126 (0.0098 0.0459) 0.2498 (0.0071 0.0284)
Saimiri_boliviensis  0.1999 (0.0273 0.1366) 0.1180 (0.0165 0.1395) 0.1466 (0.0210 0.1430) 0.1860
(0.0296 0.1590) 0.1788 (0.0237 0.1326) 0.1758 (0.0210 0.1193) 0.1607 (0.0205 0.1277)
Callithrix_jacchus   0.1917 (0.0301 0.1568) 0.1211 (0.0187 0.1545) 0.1469 (0.0232 0.1582) 0.1798
(0.0323 0.1798) 0.1694 (0.0264 0.1560) 0.1665 (0.0237 0.1424) 0.1612 (0.0232 0.1442) 0.1129 (0.0080
0.0705)
Tarsius_syrichta     0.1616 (0.0555 0.3433) 0.1311 (0.0442 0.3376) 0.1428 (0.0489 0.3424) 0.1715
(0.0578 0.3372) 0.1490 (0.0524 0.3518) 0.1446 (0.0496 0.3429) 0.1330 (0.0486 0.3656) 0.1388 (0.0448
0.3229) 0.1430 (0.0467 0.3267)
Microcebus_murinus   0.1916 (0.0639 0.3333) 0.1568 (0.0534 0.3407) 0.1656 (0.0572 0.3455) 0.1719
(0.0667 0.3880) 0.1667 (0.0615 0.3691) 0.1692 (0.0587 0.3468) 0.1561 (0.0549 0.3515) 0.1398 (0.0539
0.3851) 0.1458 (0.0567 0.3891) 0.1536 (0.0581 0.3783)
Daubentonia_madagascariensis 0.1723 (0.0506 0.2938) 0.1328 (0.0394 0.2968) 0.1463 (0.0441 0.3013)
0.1601 (0.0525 0.3279) 0.1535 (0.0464 0.3025) 0.1536 (0.0446 0.2902) 0.1386 (0.0408 0.2945) 0.1258
(0.0398 0.3167) 0.1313 (0.0427 0.3247) 0.1513 (0.0473 0.3126) 0.2022 (0.0288 0.1425)
Otolemur_garnettii   0.1351 (0.0619 0.4579) 0.1163 (0.0524 0.4509) 0.1189 (0.0543 0.4566) 0.1462
(0.0652 0.4459) 0.1262 (0.0601 0.4759) 0.1256 (0.0572 0.4552) 0.1227 (0.0553 0.4505) 0.1057 (0.0481
0.4554) 0.1097 (0.0510 0.4649) 0.1315 (0.0609 0.4633) 0.1247 (0.0380 0.3045) 0.1087 (0.0334 0.3073)


TREE #  1:  ((((((1, 2), 3), 4), ((5, 6), 7)), (8, 9)), 10, ((11, 12), 13));   MP score: 518
lnL(ntime: 23  np: 25):  -4803.200405      +0.000000
  14..15   15..16   16..17   17..18   18..19   19..1    19..2    18..3    17..4    16..20   20..21
21..5    21..6    20..7    15..22   22..8    22..9    14..10   14..23   23..24   24..11   24..12
23..13
 0.099974 0.022612 0.033175 0.020956 0.000004 0.028669 0.002131 0.018309 0.068084 0.041538 0.009845
0.025875 0.008172 0.020139 0.034847 0.027491 0.041536 0.184432 0.093856 0.029604 0.118299 0.058887
0.214027 4.011412 0.189675

Note: Branch length is defined as number of nucleotide substitutions per codon (not per neucleotide
site).

tree length =   1.20246

((((((1: 0.028669, 2: 0.002131): 0.000004, 3: 0.018309): 0.020956, 4: 0.068084): 0.033175, ((5:
0.025875, 6: 0.008172): 0.009845, 7: 0.020139): 0.041538): 0.022612, (8: 0.027491, 9: 0.041536):
0.034847): 0.099974, 10: 0.184432, ((11: 0.118299, 12: 0.058887): 0.029604, 13: 0.214027): 0.093856);

((((((Homo_sapiens: 0.028669, Pan_troglodytes: 0.002131): 0.000004, Gorilla_gorilla_gorilla: 0.018309):
0.020956, Pongo_abelii: 0.068084): 0.033175, ((Macaca_mulatta: 0.025875, Papio_anubis: 0.008172):
0.009845, Chlorocebus_sabaeus: 0.020139): 0.041538): 0.022612, (Saimiri_boliviensis: 0.027491,
Callithrix_jacchus: 0.041536): 0.034847): 0.099974, Tarsius_syrichta: 0.184432, ((Microcebus_murinus:
0.118299, Daubentonia_madagascariensis: 0.058887): 0.029604, Otolemur_garnettii: 0.214027): 0.093856);
```

Detailed output identifying parameters

kappa (ts/tv) = 4.01141

omega (dN/dS) = 0.18967

dN & dS for each branch

branch	t	N	S	dN/dS	dN	dS	N*dN	S*dS
14..15	0.100	1091.2	402.8	0.1897	0.0155	0.0816	16.9	32.9
15..16	0.023	1091.2	402.8	0.1897	0.0035	0.0185	3.8	7.4
16..17	0.033	1091.2	402.8	0.1897	0.0051	0.0271	5.6	10.9
17..18	0.021	1091.2	402.8	0.1897	0.0032	0.0171	3.5	6.9
18..19	0.000	1091.2	402.8	0.1897	0.0000	0.0000	0.0	0.0
19..1	0.029	1091.2	402.8	0.1897	0.0044	0.0234	4.8	9.4
19..2	0.002	1091.2	402.8	0.1897	0.0003	0.0017	0.4	0.7
18..3	0.018	1091.2	402.8	0.1897	0.0028	0.0150	3.1	6.0
17..4	0.068	1091.2	402.8	0.1897	0.0105	0.0556	11.5	22.4
16..20	0.042	1091.2	402.8	0.1897	0.0064	0.0339	7.0	13.7
20..21	0.010	1091.2	402.8	0.1897	0.0015	0.0080	1.7	3.2
21..5	0.026	1091.2	402.8	0.1897	0.0040	0.0211	4.4	8.5
21..6	0.008	1091.2	402.8	0.1897	0.0013	0.0067	1.4	2.7
20..7	0.020	1091.2	402.8	0.1897	0.0031	0.0164	3.4	6.6
15..22	0.035	1091.2	402.8	0.1897	0.0054	0.0285	5.9	11.5
22..8	0.027	1091.2	402.8	0.1897	0.0043	0.0225	4.6	9.0
22..9	0.042	1091.2	402.8	0.1897	0.0064	0.0339	7.0	13.7
14..10	0.184	1091.2	402.8	0.1897	0.0286	0.1506	31.2	60.7
14..23	0.094	1091.2	402.8	0.1897	0.0145	0.0766	15.9	30.9
23..24	0.030	1091.2	402.8	0.1897	0.0046	0.0242	5.0	9.7
24..11	0.118	1091.2	402.8	0.1897	0.0183	0.0966	20.0	38.9
24..12	0.059	1091.2	402.8	0.1897	0.0091	0.0481	10.0	19.4
23..13	0.214	1091.2	402.8	0.1897	0.0332	0.1748	36.2	70.4

tree length for dN: 0.1863
tree length for dS: 0.9820

Main result file for model B: catarrhines-B.mlc

```
CODONML (in paml version 4.7b, October 2013)  catarrhines.phy
Model: One dN/dS ratio for branches,  omega = 1.000 fixed

Codon frequency model: F3x4
ns =  13  ls = 498

Codon usage in sequences
--------------------------------------------------------------------------------------------------------------------------------------
Phe TTT  13  13  13  14  13  13 | Ser TCT   5   5   5   7   4   4 | Tyr TAT  11  10  11  10   9   8 | Cys TGT   3   4   4   6   5   5
    TTC  15  15  15  13  15  15 |     TCC  11  11  11   9  11  11 |     TAC  16  16  15  15  17  18 |     TGC   8   6   6   7   5   5
Leu TTA   1   1   1   2   2   2 |     TCA   2   2   2   3   2   2 | *** TAA   0   0   0   0   0   0 | *** TGA   0   0   0   0   0   0
    TTG   9   9   9   8   9   9 |     TCG   2   2   2   1   2   2 |     TAG   0   0   0   0   0   0 | Trp TGG   4   4   4   4   4   4
--------------------------------------------------------------------------------------------------------------------------------------
Leu CTT  10   9   9   8   6   6 | Pro CCT  13  14  15  12  10  10 | His CAT   6   6   6   6   6   6 | Arg CGT   4   4   3   3   4   3
    CTC   8   8   8  10   9   9 |     CCC  10  10   9  11  13  14 |     CAC  11  10  10  11  10  10 |     CGC   6   8   8   5   7   8
    CTA   2   2   2   2   2   2 |     CCA   3   3   3   3   2   2 | Gln CAA   5   4   4   4   4   4 |     CGA   3   5   5   5   5   5
    CTG  27  27  27  27  30  30 |     CCG   2   2   2   2   4   3 |     CAG  11  11  12  13  13  13 |     CGG   3   2   2   2   1   1
--------------------------------------------------------------------------------------------------------------------------------------
Ile ATT  10  10  10   9  11  10 | Thr ACT   6   6   6   9   9   9 | Asn AAT  12  11  12  11  14  12 | Ser AGT   2   2   3   2   3   3
    ATC  17  17  18  18  16  17 |     ACC  11  10  10   9   8   9 |     AAC   7   6   6   6   4   6 |     AGC  10  10  10  10   9   9
    ATA   1   1   0   1   1   1 |     ACA   9   9   9   8  11  10 | Lys AAA   3   3   3   3   3   3 | Arg AGA   1   0   0   1   0   0
Met ATG   8   9   9  11   8   8 |     ACG   7   6   6   4   3   3 |     AAG   8   8   8   8   9   8 |     AGG   6   6   6   7   6   6
--------------------------------------------------------------------------------------------------------------------------------------
Val GTT   3   3   3   3   4   5 | Ala GCT  10  10  10  11   9   9 | Asp GAT  17  18  17  17  21  21 | Gly GGT   2   3   3   2   4   4
    GTC  16  15  15  15  15  14 |     GCC   8  10   9   9  12  12 |     GAC  23  24  24  24  20  20 |     GGC  14  14  14  15  13  13
    GTA   2   3   2   3   2   2 |     GCA   2   2   2   2   2   2 | Glu GAA   5   5   5   4   5   4 |     GGA   9   9   9  10   9   9
    GTG  15  14  16  14  15  15 |     GCG   0   0   0   0   0   0 |     GAG  22  22  21  21  20  22 |     GGG   8   9   9   8   8   8
--------------------------------------------------------------------------------------------------------------------------------------


--------------------------------------------------------------------------------------------------------------------------------------
Phe TTT  13  13  12  16  10  12 | Ser TCT   4   3   4   4   4   4 | Tyr TAT   9  12  12  11   5   6 | Cys TGT   4   4   6   7   2   4
    TTC  15  15  15  15  17  14 |     TCC  11  11  11  13  14  14 |     TAC  17  14  14  14  20  19 |     TGC   6   7   5   4  10   8
Leu TTA   2   1   1   0   2   0 |     TCA   2   2   2   2   1   1 | *** TAA   0   0   0   0   0   0 | *** TGA   0   0   0   0   0   0
    TTG   9  11  10   9   5   6 |     TCG   2   2   2   3   2   2 |     TAG   0   0   0   0   0   0 | Trp TGG   4   4   4   4   4   4
--------------------------------------------------------------------------------------------------------------------------------------
Leu CTT   6  10  11   5   4   7 | Pro CCT  10  11  11  12   9   9 | His CAT   6   6   7   8   5   6 | Arg CGT   3   3   2   4   4   2
    CTC   9   9   8  12  12  11 |     CCC  14  13  12  10  15  12 |     CAC  10   9   9   8  12  12 |     CGC   8   7   7   6   9   8
    CTA   3   2   2   4   2   2 |     CCA   2   2   2   4   2   3 | Gln CAA   5   3   4   3   5   3 |     CGA   4   5   5   6   3   5
    CTG  29  25  27  25  30  30 |     CCG   3   2   2   1   3   3 |     CAG  12  14  12  12  12  13 |     CGG   2   1   2   3   3   3
--------------------------------------------------------------------------------------------------------------------------------------
Ile ATT   9  11  10   8   4   5 | Thr ACT   9   9   9   9   6   8 | Asn AAT  12  12  11  11   5   9 | Ser AGT   3   5   5   5   3   3
    ATC  17  17  18  19  22  19 |     ACC   9   7   7  10  14  12 |     AAC   6   5   5   5   9   7 |     AGC   9   7   8   5   8   7
    ATA   1   1   1   4   2   2 |     ACA   9   9  10   5   6   6 | Lys AAA   3   2   2   4   3   2 | Arg AGA   1   0   0   2   2   2
Met ATG   9   9  10  10  11   9 |     ACG   4   5   4   5   6   6 |     AAG   8   7   7  10  10   9 |     AGG   6   7   7   4   3   4
--------------------------------------------------------------------------------------------------------------------------------------
Val GTT   3   7   8   6   4   4 | Ala GCT   9  12  12  10   9   7 | Asp GAT  20  20  20  19  11  17 | Gly GGT   4   4   3   6   3   5
    GTC  16  12   9  10  12  14 |     GCC  11  12  12   9  13  13 |     GAC  21  22  22  24  31  25 |     GGC  14  13  13  14  18  15
    GTA   2   2   2   4   3   2 |     GCA   2   2   2   2   0   3 | Glu GAA   4   5   6  11   5   6 |     GGA   8   8   9   7   6   7
    GTG  15  14  15  13  14  18 |     GCG   0   0   1   0   2   0 |     GAG  21  22  21  14  17  19 |     GGG   9   9   8   8  10  10
--------------------------------------------------------------------------------------------------------------------------------------


------------------------------------------------------
Phe TTT  10 | Ser TCT   3 | Tyr TAT   3 | Cys TGT   5
    TTC  20 |     TCC  12 |     TAC  22 |     TGC   8
Leu TTA   2 |     TCA   2 | *** TAA   0 | *** TGA   0
    TTG   6 |     TCG   1 |     TAG   0 | Trp TGG   4
------------------------------------------------------
Leu CTT   7 | Pro CCT  13 | His CAT   6 | Arg CGT   4
    CTC  10 |     CCC  10 |     CAC  11 |     CGC   7
    CTA   4 |     CCA   2 | Gln CAA   5 |     CGA   3
    CTG  26 |     CCG   2 |     CAG  11 |     CGG   3
------------------------------------------------------
Ile ATT   5 | Thr ACT   7 | Asn AAT   6 | Ser AGT   3
    ATC  20 |     ACC  13 |     AAC  10 |     AGC   7
    ATA   2 |     ACA   6 | Lys AAA   3 | Arg AGA   3
Met ATG  10 |     ACG   4 |     AAG   9 |     AGG   3
------------------------------------------------------
Val GTT   5 | Ala GCT   7 | Asp GAT  11 | Gly GGT   5
    GTC  14 |     GCC  15 |     GAC  28 |     GGC  19
    GTA   4 |     GCA   3 | Glu GAA  11 |     GGA   5
    GTG  14 |     GCG   1 |     GAG  15 |     GGG   8
------------------------------------------------------

Codon position x base (3x4) table for each sequence.

#1: Homo_sapiens
position  1:    T:0.20080    C:0.24900    A:0.23695    G:0.31325
position  2:    T:0.31526    C:0.20281    A:0.31526    G:0.16667
position  3:    T:0.25502    C:0.38353    A:0.09639    G:0.26506
Average         T:0.25703    C:0.27845    A:0.21620    G:0.24833

#2: Pan_troglodytes
position  1:    T:0.19679    C:0.25100    A:0.22892    G:0.32329
position  2:    T:0.31325    C:0.20482    A:0.30924    G:0.17269
position  3:    T:0.25703    C:0.38153    A:0.09839    G:0.26305
Average         T:0.25569    C:0.27912    A:0.21218    G:0.25301
```

```
#3: Gorilla_gorilla_gorilla
position  1:    T:0.19679    C:0.25100    A:0.23293    G:0.31928
position  2:    T:0.31526    C:0.20281    A:0.30924    G:0.17269
position  3:    T:0.26104    C:0.37751    A:0.09438    G:0.26707
Average         T:0.25770    C:0.27711    A:0.21218    G:0.25301

#4: Pongo_abelii
position  1:    T:0.19880    C:0.24900    A:0.23494    G:0.31727
position  2:    T:0.31727    C:0.20080    A:0.30723    G:0.17470
position  3:    T:0.26104    C:0.37550    A:0.10241    G:0.26104
Average         T:0.25904    C:0.27510    A:0.21486    G:0.25100

#5: Macaca_mulatta
position  1:    T:0.19679    C:0.25301    A:0.23092    G:0.31928
position  2:    T:0.31727    C:0.20482    A:0.31124    G:0.16667
position  3:    T:0.26506    C:0.36948    A:0.10040    G:0.26506
Average         T:0.25971    C:0.27577    A:0.21419    G:0.25033

#6: Papio_anubis
position  1:    T:0.19679    C:0.25301    A:0.22892    G:0.32129
position  2:    T:0.31727    C:0.20482    A:0.31124    G:0.16667
position  3:    T:0.25703    C:0.38153    A:0.09639    G:0.26506
Average         T:0.25703    C:0.27979    A:0.21218    G:0.25100

#7: Chlorocebus_sabaeus
position  1:    T:0.19679    C:0.25301    A:0.23092    G:0.31928
position  2:    T:0.31727    C:0.20281    A:0.30924    G:0.17068
position  3:    T:0.24900    C:0.38755    A:0.09639    G:0.26707
Average         T:0.25435    C:0.28112    A:0.21218    G:0.25234

#8: Saimiri_boliviensis
position  1:    T:0.19880    C:0.24498    A:0.22691    G:0.32932
position  2:    T:0.31928    C:0.20482    A:0.30723    G:0.16867
position  3:    T:0.28514    C:0.36145    A:0.08835    G:0.26506
Average         T:0.26774    C:0.27041    A:0.20750    G:0.25435

#9: Callithrix_jacchus
position  1:    T:0.19679    C:0.24699    A:0.22892    G:0.32731
position  2:    T:0.31928    C:0.20683    A:0.30522    G:0.16867
position  3:    T:0.28715    C:0.35141    A:0.09639    G:0.26506
Average         T:0.26774    C:0.26841    A:0.21017    G:0.25368

#10: Tarsius_syrichta
position  1:    T:0.20482    C:0.24699    A:0.23293    G:0.31526
position  2:    T:0.32129    C:0.19880    A:0.30924    G:0.17068
position  3:    T:0.28313    C:0.35743    A:0.11647    G:0.24297
Average         T:0.26975    C:0.26774    A:0.21954    G:0.24297

#11: Microcebus_murinus
position  1:    T:0.19277    C:0.26104    A:0.22892    G:0.31727
position  2:    T:0.30924    C:0.21285    A:0.30120    G:0.17671
position  3:    T:0.17671    C:0.47390    A:0.08434    G:0.26506
Average         T:0.22624    C:0.31593    A:0.20482    G:0.25301

#12: Daubentonia_madagascariensis
position  1:    T:0.18876    C:0.25904    A:0.22088    G:0.33133
position  2:    T:0.31124    C:0.20683    A:0.30723    G:0.17470
position  3:    T:0.21687    C:0.42169    A:0.08835    G:0.27309
Average         T:0.23896    C:0.29585    A:0.20549    G:0.25971

#13: Otolemur_garnettii
position  1:    T:0.19679    C:0.24900    A:0.22289    G:0.33133
position  2:    T:0.31928    C:0.20281    A:0.30321    G:0.17470
position  3:    T:0.20080    C:0.45382    A:0.11044    G:0.23494
Average         T:0.23896    C:0.30187    A:0.21218    G:0.24699
```

Sums of codon usage counts

Phe F	TTT	165	Ser S	TCT	56	Tyr Y	TAT	117	Cys C	TGT	59
	TTC	199		TCC	150		TAC	217		TGC	85
Leu L	TTA	17		TCA	25	*** *	TAA	0	*** *	TGA	0
	TTG	109		TCG	25		TAG	0	Trp W	TGG	52
Leu L	CTT	98	Pro P	CCT	149	His H	CAT	80	Arg R	CGT	43
	CTC	123		CCC	153		CAC	133		CGC	94
	CTA	31		CCA	33	Gln Q	CAA	53		CGA	59

```
      CTG     360 |       CCG      31 |       CAG     159 |       CGG      28
------------------------------------------------------------------------------
Ile I ATT     112 | Thr T ACT     102 | Asn N AAT     138 | Ser S AGT      42
      ATC     235 |       ACC     129 |       AAC      82 |       AGC     109
      ATA      18 |       ACA     107 | Lys K AAA      37 | Arg R AGA      12
Met M ATG     121 |       ACG      63 |       AAG     109 |       AGG      71
------------------------------------------------------------------------------
Val V GTT      58 | Ala A GCT     125 | Asp D GAT     229 | Gly G GGT      48
      GTC     177 |       GCC     145 |       GAC     308 |       GGC     189
      GTA      33 |       GCA      26 | Glu E GAA      76 |       GGA     105
      GTG     192 |       GCG       4 |       GAG     257 |       GGG     112
------------------------------------------------------------------------------


Codon position x base (3x4) table, overall

position  1:    T:0.19710    C:0.25131    A:0.22969    G:0.32190
position  2:    T:0.31634    C:0.20436    A:0.30816    G:0.17115
position  3:    T:0.25039    C:0.39049    A:0.09762    G:0.26151
Average         T:0.25461    C:0.28205    A:0.21182    G:0.25152


Nei & Gojobori 1986. dN/dS (dN, dS)
(Note: This matrix is not used in later ML. analysis.
Use runmode = -2 for ML pairwise comparison.)

Homo_sapiens
Pan_troglodytes      1.2604 (0.0106 0.0084)
Gorilla_gorilla_gorilla 1.3389 (0.0151 0.0113) 0.7351 (0.0062 0.0084)
Pongo_abelii         0.4378 (0.0296 0.0676) 0.2910 (0.0187 0.0643) 0.3714 (0.0250 0.0674)
Macaca_mulatta       0.2054 (0.0269 0.1309) 0.1261 (0.0160 0.1271) 0.1712 (0.0224 0.1306) 0.2017
(0.0278 0.1377)
Papio_anubis         0.2052 (0.0242 0.1177) 0.1169 (0.0133 0.1141) 0.1672 (0.0196 0.1174) 0.1773
(0.0232 0.1311) 0.2434 (0.0062 0.0255)
Chlorocebus_sabaeus  0.2404 (0.0260 0.1080) 0.1448 (0.0151 0.1045) 0.1989 (0.0214 0.1078) 0.2216
(0.0269 0.1212) 0.2126 (0.0098 0.0459) 0.2498 (0.0071 0.0284)
Saimiri_boliviensis  0.1999 (0.0273 0.1366) 0.1180 (0.0165 0.1395) 0.1466 (0.0210 0.1430) 0.1860
(0.0296 0.1590) 0.1788 (0.0237 0.1326) 0.1758 (0.0210 0.1193) 0.1607 (0.0205 0.1277)
Callithrix_jacchus   0.1917 (0.0301 0.1568) 0.1211 (0.0187 0.1545) 0.1469 (0.0232 0.1582) 0.1798
(0.0323 0.1798) 0.1694 (0.0264 0.1560) 0.1665 (0.0237 0.1424) 0.1612 (0.0232 0.1442) 0.1129 (0.0080
0.0705)
Tarsius_syrichta     0.1616 (0.0555 0.3433) 0.1311 (0.0442 0.3376) 0.1428 (0.0489 0.3424) 0.1715
(0.0578 0.3372) 0.1490 (0.0524 0.3518) 0.1446 (0.0496 0.3429) 0.1330 (0.0486 0.3656) 0.1388 (0.0448
0.3229) 0.1430 (0.0467 0.3267)
Microcebus_murinus   0.1916 (0.0639 0.3333) 0.1568 (0.0534 0.3407) 0.1656 (0.0572 0.3455) 0.1719
(0.0667 0.3880) 0.1667 (0.0615 0.3691) 0.1692 (0.0587 0.3468) 0.1561 (0.0549 0.3515) 0.1398 (0.0539
0.3851) 0.1458 (0.0567 0.3891) 0.1536 (0.0581 0.3783)
Daubentonia_madagascariensis 0.1723 (0.0506 0.2938) 0.1328 (0.0394 0.2968) 0.1463 (0.0441 0.3013)
0.1601 (0.0525 0.3279) 0.1535 (0.0464 0.3025) 0.1536 (0.0446 0.2902) 0.1386 (0.0408 0.2945) 0.1258
(0.0398 0.3167) 0.1313 (0.0427 0.3247) 0.1513 (0.0473 0.3126) 0.2022 (0.0288 0.1425)
Otolemur_garnettii   0.1351 (0.0619 0.4579) 0.1163 (0.0524 0.4509) 0.1189 (0.0543 0.4566) 0.1462
(0.0652 0.4459) 0.1262 (0.0601 0.4759) 0.1256 (0.0572 0.4552) 0.1227 (0.0553 0.4505) 0.1057 (0.0481
0.4554) 0.1097 (0.0510 0.4649) 0.1315 (0.0609 0.4633) 0.1247 (0.0380 0.3045) 0.1087 (0.0334 0.3073)


TREE #  1:  ((((((1, 2), 3), 4), ((5, 6), 7)), (8, 9)), 10, ((11, 12), 13));   MP score: 518
check convergence..
lnL(ntime: 23  np: 24):  -4969.609175      +0.000000
  14..15   15..16   16..17   17..18   18..19   19..1    19..2    18..3    17..4    16..20   20..21
21..5    21..6    20..7    15..22   22..8    22..9    14..10   14..23   23..24   24..11   24..12
23..13
 0.096815 0.023154 0.032827 0.021136 0.000004 0.028734 0.002157 0.018388 0.066963 0.040876 0.010059
0.026002 0.008354 0.020007 0.033750 0.028000 0.041400 0.176244 0.087136 0.031267 0.111169 0.057642
0.197347 5.768056

Note: Branch length is defined as number of nucleotide substitutions per codon (not per neucleotide
site).

tree length =   1.15943

((((((1: 0.028734, 2: 0.002157): 0.000004, 3: 0.018388): 0.021136, 4: 0.066963): 0.032827, ((5:
0.026002, 6: 0.008354): 0.010059, 7: 0.020007): 0.040876): 0.023154, (8: 0.028000, 9: 0.041400):
0.033750): 0.096815, 10: 0.176244, ((11: 0.111169, 12: 0.057642): 0.031267, 13: 0.197347): 0.087136);

((((((Homo_sapiens: 0.028734, Pan_troglodytes: 0.002157): 0.000004, Gorilla_gorilla_gorilla: 0.018388):
0.021136, Pongo_abelii: 0.066963): 0.032827, ((Macaca_mulatta: 0.026002, Papio_anubis: 0.008354):
0.010059, Chlorocebus_sabaeus: 0.020007): 0.040876): 0.023154, (Saimiri_boliviensis: 0.028000,
```

Callithrix_jacchus: 0.041400): 0.033750): 0.096815, Tarsius_syrichta: 0.176244, ((Microcebus_murinus: 0.111169, Daubentonia_madagascariensis: 0.057642): 0.031267, Otolemur_garnettii: 0.197347): 0.087136);

Detailed output identifying parameters

kappa (ts/tv) = 5.76806

dN & dS for each branch

branch	t	N	S	dN/dS	dN	dS	N*dN	S*dS
14..15	0.097	1071.8	422.2	1.0000	0.0323	0.0323	34.6	13.6
15..16	0.023	1071.8	422.2	1.0000	0.0077	0.0077	8.3	3.3
16..17	0.033	1071.8	422.2	1.0000	0.0109	0.0109	11.7	4.6
17..18	0.021	1071.8	422.2	1.0000	0.0070	0.0070	7.6	3.0
18..19	0.000	1071.8	422.2	1.0000	0.0000	0.0000	0.0	0.0
19..1	0.029	1071.8	422.2	1.0000	0.0096	0.0096	10.3	4.0
19..2	0.002	1071.8	422.2	1.0000	0.0007	0.0007	0.8	0.3
18..3	0.018	1071.8	422.2	1.0000	0.0061	0.0061	6.6	2.6
17..4	0.067	1071.8	422.2	1.0000	0.0223	0.0223	23.9	9.4
16..20	0.041	1071.8	422.2	1.0000	0.0136	0.0136	14.6	5.8
20..21	0.010	1071.8	422.2	1.0000	0.0034	0.0034	3.6	1.4
21..5	0.026	1071.8	422.2	1.0000	0.0087	0.0087	9.3	3.7
21..6	0.008	1071.8	422.2	1.0000	0.0028	0.0028	3.0	1.2
20..7	0.020	1071.8	422.2	1.0000	0.0067	0.0067	7.1	2.8
15..22	0.034	1071.8	422.2	1.0000	0.0112	0.0112	12.1	4.7
22..8	0.028	1071.8	422.2	1.0000	0.0093	0.0093	10.0	3.9
22..9	0.041	1071.8	422.2	1.0000	0.0138	0.0138	14.8	5.8
14..10	0.176	1071.8	422.2	1.0000	0.0587	0.0587	63.0	24.8
14..23	0.087	1071.8	422.2	1.0000	0.0290	0.0290	31.1	12.3
23..24	0.031	1071.8	422.2	1.0000	0.0104	0.0104	11.2	4.4
24..11	0.111	1071.8	422.2	1.0000	0.0371	0.0371	39.7	15.6
24..12	0.058	1071.8	422.2	1.0000	0.0192	0.0192	20.6	8.1
23..13	0.197	1071.8	422.2	1.0000	0.0658	0.0658	70.5	27.8

tree length for dN: 0.3865
tree length for dS: 0.3865

Main result file for model C: catarrhines-C.mlc

```
CODONML (in paml version 4.7b, October 2013)  catarrhines.phy
Model: several dN/dS ratios for branches for branches,
Codon frequency model: F3x4
ns =  13  ls = 498

Codon usage in sequences
--------------------------------------------------------------------------------------------------------------------------------------
Phe TTT  13  13  13  14  13  13 | Ser TCT   5   5   5   7   4   4 | Tyr TAT  11  10  11  10   9   8 | Cys TGT   3   4   4   6   5   5
    TTC  15  15  15  13  15  15 |     TCC  11  11  11   9  11  11 |     TAC  16  16  15  15  17  18 |     TGC   8   6   6   7   5   5
Leu TTA   1   1   1   2   2   2 |     TCA   2   2   2   3   2   2 | *** TAA   0   0   0   0   0   0 | *** TGA   0   0   0   0   0   0
    TTG   9   9   9   8   9   9 |     TCG   2   2   2   1   2   2 |     TAG   0   0   0   0   0   0 | Trp TGG   4   4   4   4   4   4
--------------------------------------------------------------------------------------------------------------------------------------
Leu CTT  10   9   9   8   6   6 | Pro CCT  13  14  15  12  10  10 | His CAT   6   6   6   6   6   6 | Arg CGT   4   4   3   3   4   3
    CTC   8   8   8  10   9   9 |     CCC  10  10   9  11  13  14 |     CAC  11  10  10  11  10  10 |     CGC   6   8   8   5   7   8
    CTA   2   2   2   2   2   2 |     CCA   3   3   3   3   2   2 | Gln CAA   5   4   4   4   4   4 |     CGA   3   5   5   5   5   5
    CTG  27  27  27  27  30  30 |     CCG   2   2   2   2   4   3 |     CAG  11  11  12  13  13  13 |     CGG   3   2   2   2   1   1
--------------------------------------------------------------------------------------------------------------------------------------
Ile ATT  10  10  10   9  11  10 | Thr ACT   6   6   6   9   9   9 | Asn AAT  12  11  12  11  14  12 | Ser AGT   2   2   3   2   3   3
    ATC  17  17  18  18  16  17 |     ACC  11  10  10   9   8   9 |     AAC   7   6   6   6   4   6 |     AGC  10  10  10  10   9   9
    ATA   1   1   0   1   1   1 |     ACA   9   9   9   8  11  10 | Lys AAA   3   3   3   3   3   3 | Arg AGA   1   0   0   1   0   0
Met ATG   8   9   9  11   8   8 |     ACG   7   6   6   4   3   3 |     AAG   8   8   8   8   9   8 |     AGG   6   6   6   7   6   6
--------------------------------------------------------------------------------------------------------------------------------------
Val GTT   3   3   3   3   4   5 | Ala GCT  10  10  10  11   9   9 | Asp GAT  17  18  17  17  21  21 | Gly GGT   2   3   3   2   4   4
    GTC  16  15  15  15  15  14 |     GCC   8  10   9   9  12  12 |     GAC  23  24  24  24  20  20 |     GGC  14  14  14  15  13  13
    GTA   2   3   2   3   2   2 |     GCA   2   2   2   2   2   2 | Glu GAA   5   5   5   4   5   4 |     GGA   9   9   9  10   9   9
    GTG  15  14  16  14  15  15 |     GCG   0   0   0   0   0   0 |     GAG  22  22  21  21  20  22 |     GGG   8   9   9   8   8   8
--------------------------------------------------------------------------------------------------------------------------------------


--------------------------------------------------------------------------------------------------------------------------------------
Phe TTT  13  13  12  16  10  12 | Ser TCT   4   3   4   4   4   4 | Tyr TAT   9  12  12  11   5   6 | Cys TGT   4   4   6   7   2   4
    TTC  15  15  15  15  17  14 |     TCC  11  11  11  13  14  14 |     TAC  17  14  14  14  20  19 |     TGC   6   7   5   4  10   8
Leu TTA   2   1   1   0   2   0 |     TCA   2   2   2   2   1   1 | *** TAA   0   0   0   0   0   0 | *** TGA   0   0   0   0   0   0
    TTG   9  11  10   9   5   6 |     TCG   2   2   2   3   2   2 |     TAG   0   0   0   0   0   0 | Trp TGG   4   4   4   4   4   4
--------------------------------------------------------------------------------------------------------------------------------------
Leu CTT   6  10  11   5   4   7 | Pro CCT  10  11  11  12   9   9 | His CAT   6   6   7   8   5   6 | Arg CGT   3   3   2   4   4   2
    CTC   9   9   8  12  12  11 |     CCC  14  13  12  10  15  12 |     CAC  10   9   9   8  12  12 |     CGC   8   7   7   6   9   8
    CTA   3   2   2   4   2   2 |     CCA   2   2   2   4   2   3 | Gln CAA   5   3   4   3   5   3 |     CGA   4   5   5   6   3   5
    CTG  29  25  27  25  30  30 |     CCG   3   2   2   1   3   3 |     CAG  12  14  12  12  12  13 |     CGG   2   1   2   3   3   3
--------------------------------------------------------------------------------------------------------------------------------------
Ile ATT   9  11  10   8   4   5 | Thr ACT   9   9   9   9   6   8 | Asn AAT  12  12  11  11   5   9 | Ser AGT   3   5   5   5   3   3
    ATC  17  17  18  19  22  19 |     ACC   9   7   7  10  14  12 |     AAC   6   5   5   5   9   7 |     AGC   9   7   8   5   8   7
    ATA   1   1   1   4   2   2 |     ACA   9   9  10   5   6   6 | Lys AAA   3   2   2   4   3   2 | Arg AGA   1   0   0   2   2   2
Met ATG   9   9  10  10  11   9 |     ACG   4   5   4   5   6   6 |     AAG   8   7   7  10  10   9 |     AGG   6   7   7   4   3   4
--------------------------------------------------------------------------------------------------------------------------------------
Val GTT   3   7   8   6   4   4 | Ala GCT   9  12  12  10   9   7 | Asp GAT  20  20  20  19  11  17 | Gly GGT   4   4   3   6   3   5
    GTC  16  12   9  10  12  14 |     GCC  11  12  12   9  13  13 |     GAC  21  22  22  24  31  25 |     GGC  14  13  13  14  18  15
    GTA   2   2   2   4   3   2 |     GCA   2   2   2   2   0   3 | Glu GAA   4   5   6  11   5   6 |     GGA   8   8   9   7   6   7
    GTG  15  14  15  13  14  18 |     GCG   0   0   1   0   2   0 |     GAG  21  22  21  14  17  19 |     GGG   9   9   8   8  10  10
--------------------------------------------------------------------------------------------------------------------------------------


------------------------------------------------------
Phe TTT  10 | Ser TCT   3 | Tyr TAT   3 | Cys TGT   5
    TTC  20 |     TCC  12 |     TAC  22 |     TGC   8
Leu TTA   2 |     TCA   2 | *** TAA   0 | *** TGA   0
    TTG   6 |     TCG   1 |     TAG   0 | Trp TGG   4
------------------------------------------------------
Leu CTT   7 | Pro CCT  13 | His CAT   6 | Arg CGT   4
    CTC  10 |     CCC  10 |     CAC  11 |     CGC   7
    CTA   4 |     CCA   2 | Gln CAA   5 |     CGA   3
    CTG  26 |     CCG   2 |     CAG  11 |     CGG   3
------------------------------------------------------
Ile ATT   5 | Thr ACT   7 | Asn AAT   6 | Ser AGT   3
    ATC  20 |     ACC  13 |     AAC  10 |     AGC   7
    ATA   2 |     ACA   6 | Lys AAA   3 | Arg AGA   3
Met ATG  10 |     ACG   4 |     AAG   9 |     AGG   3
------------------------------------------------------
Val GTT   5 | Ala GCT   7 | Asp GAT  11 | Gly GGT   5
    GTC  14 |     GCC  15 |     GAC  28 |     GGC  19
    GTA   4 |     GCA   3 | Glu GAA  11 |     GGA   5
    GTG  14 |     GCG   1 |     GAG  15 |     GGG   8
------------------------------------------------------

Codon position x base (3x4) table for each sequence.

#1: Homo_sapiens
position  1:    T:0.20080    C:0.24900    A:0.23695    G:0.31325
position  2:    T:0.31526    C:0.20281    A:0.31526    G:0.16667
position  3:    T:0.25502    C:0.38353    A:0.09639    G:0.26506
Average         T:0.25703    C:0.27845    A:0.21620    G:0.24833

#2: Pan_troglodytes
position  1:    T:0.19679    C:0.25100    A:0.22892    G:0.32329
position  2:    T:0.31325    C:0.20482    A:0.30924    G:0.17269
position  3:    T:0.25703    C:0.38153    A:0.09839    G:0.26305
Average         T:0.25569    C:0.27912    A:0.21218    G:0.25301
```

```
#3: Gorilla_gorilla_gorilla
position  1:    T:0.19679    C:0.25100    A:0.23293    G:0.31928
position  2:    T:0.31526    C:0.20281    A:0.30924    G:0.17269
position  3:    T:0.26104    C:0.37751    A:0.09438    G:0.26707
Average         T:0.25770    C:0.27711    A:0.21218    G:0.25301

#4: Pongo_abelii
position  1:    T:0.19880    C:0.24900    A:0.23494    G:0.31727
position  2:    T:0.31727    C:0.20080    A:0.30723    G:0.17470
position  3:    T:0.26104    C:0.37550    A:0.10241    G:0.26104
Average         T:0.25904    C:0.27510    A:0.21486    G:0.25100

#5: Macaca_mulatta
position  1:    T:0.19679    C:0.25301    A:0.23092    G:0.31928
position  2:    T:0.31727    C:0.20482    A:0.31124    G:0.16667
position  3:    T:0.26506    C:0.36948    A:0.10040    G:0.26506
Average         T:0.25971    C:0.27577    A:0.21419    G:0.25033

#6: Papio_anubis
position  1:    T:0.19679    C:0.25301    A:0.22892    G:0.32129
position  2:    T:0.31727    C:0.20482    A:0.31124    G:0.16667
position  3:    T:0.25703    C:0.38153    A:0.09639    G:0.26506
Average         T:0.25703    C:0.27979    A:0.21218    G:0.25100

#7: Chlorocebus_sabaeus
position  1:    T:0.19679    C:0.25301    A:0.23092    G:0.31928
position  2:    T:0.31727    C:0.20281    A:0.30924    G:0.17068
position  3:    T:0.24900    C:0.38755    A:0.09639    G:0.26707
Average         T:0.25435    C:0.28112    A:0.21218    G:0.25234

#8: Saimiri_boliviensis
position  1:    T:0.19880    C:0.24498    A:0.22691    G:0.32932
position  2:    T:0.31928    C:0.20482    A:0.30723    G:0.16867
position  3:    T:0.28514    C:0.36145    A:0.08835    G:0.26506
Average         T:0.26774    C:0.27041    A:0.20750    G:0.25435

#9: Callithrix_jacchus
position  1:    T:0.19679    C:0.24699    A:0.22892    G:0.32731
position  2:    T:0.31928    C:0.20683    A:0.30522    G:0.16867
position  3:    T:0.28715    C:0.35141    A:0.09639    G:0.26506
Average         T:0.26774    C:0.26841    A:0.21017    G:0.25368

#10: Tarsius_syrichta
position  1:    T:0.20482    C:0.24699    A:0.23293    G:0.31526
position  2:    T:0.32129    C:0.19880    A:0.30924    G:0.17068
position  3:    T:0.28313    C:0.35743    A:0.11647    G:0.24297
Average         T:0.26975    C:0.26774    A:0.21954    G:0.24297

#11: Microcebus_murinus
position  1:    T:0.19277    C:0.26104    A:0.22892    G:0.31727
position  2:    T:0.30924    C:0.21285    A:0.30120    G:0.17671
position  3:    T:0.17671    C:0.47390    A:0.08434    G:0.26506
Average         T:0.22624    C:0.31593    A:0.20482    G:0.25301

#12: Daubentonia_madagascariensis
position  1:    T:0.18876    C:0.25904    A:0.22088    G:0.33133
position  2:    T:0.31124    C:0.20683    A:0.30723    G:0.17470
position  3:    T:0.21687    C:0.42169    A:0.08835    G:0.27309
Average         T:0.23896    C:0.29585    A:0.20549    G:0.25971

#13: Otolemur_garnettii
position  1:    T:0.19679    C:0.24900    A:0.22289    G:0.33133
position  2:    T:0.31928    C:0.20281    A:0.30321    G:0.17470
position  3:    T:0.20080    C:0.45382    A:0.11044    G:0.23494
Average         T:0.23896    C:0.30187    A:0.21218    G:0.24699

Sums of codon usage counts
------------------------------------------------------------------------------
Phe F TTT     165 | Ser S TCT      56 | Tyr Y TAT     117 | Cys C TGT      59
      TTC     199 |       TCC     150 |       TAC     217 |       TGC      85
Leu L TTA      17 |       TCA      25 | *** * TAA       0 | *** * TGA       0
      TTG     109 |       TCG      25 |       TAG       0 | Trp W TGG      52
------------------------------------------------------------------------------
Leu L CTT      98 | Pro P CCT     149 | His H CAT      80 | Arg R CGT      43
      CTC     123 |       CCC     153 |       CAC     133 |       CGC      94
      CTA      31 |       CCA      33 | Gln Q CAA      53 |       CGA      59
      CTG     360 |       CCG      31 |       CAG     159 |       CGG      28
```

```
------------------------------------------------------------------------------
Ile I ATT     112 | Thr T ACT     102 | Asn N AAT     138 | Ser S AGT      42
      ATC     235 |       ACC     129 |       AAC      82 |       AGC     109
      ATA      18 |       ACA     107 | Lys K AAA      37 | Arg R AGA      12
Met M ATG     121 |       ACG      63 |       AAG     109 |       AGG      71
------------------------------------------------------------------------------
Val V GTT      58 | Ala A GCT     125 | Asp D GAT     229 | Gly G GGT      48
      GTC     177 |       GCC     145 |       GAC     308 |       GGC     189
      GTA      33 |       GCA      26 | Glu E GAA      76 |       GGA     105
      GTG     192 |       GCG       4 |       GAG     257 |       GGG     112
------------------------------------------------------------------------------


Codon position x base (3x4) table, overall

position  1:    T:0.19710    C:0.25131    A:0.22969    G:0.32190
position  2:    T:0.31634    C:0.20436    A:0.30816    G:0.17115
position  3:    T:0.25039    C:0.39049    A:0.09762    G:0.26151
Average         T:0.25461    C:0.28205    A:0.21182    G:0.25152


Nei & Gojobori 1986. dN/dS (dN, dS)
(Note: This matrix is not used in later ML. analysis.
Use runmode = -2 for ML pairwise comparison.)

Homo_sapiens
Pan_troglodytes      1.2604 (0.0106 0.0084)
Gorilla_gorilla_gorilla 1.3389 (0.0151 0.0113) 0.7351 (0.0062 0.0084)
Pongo_abelii         0.4378 (0.0296 0.0676) 0.2910 (0.0187 0.0643) 0.3714 (0.0250 0.0674)
Macaca_mulatta       0.2054 (0.0269 0.1309) 0.1261 (0.0160 0.1271) 0.1712 (0.0224 0.1306) 0.2017
(0.0278 0.1377)
Papio_anubis         0.2052 (0.0242 0.1177) 0.1169 (0.0133 0.1141) 0.1672 (0.0196 0.1174) 0.1773
(0.0232 0.1311) 0.2434 (0.0062 0.0255)
Chlorocebus_sabaeus  0.2404 (0.0260 0.1080) 0.1448 (0.0151 0.1045) 0.1989 (0.0214 0.1078) 0.2216
(0.0269 0.1212) 0.2126 (0.0098 0.0459) 0.2498 (0.0071 0.0284)
Saimiri_boliviensis  0.1999 (0.0273 0.1366) 0.1180 (0.0165 0.1395) 0.1466 (0.0210 0.1430) 0.1860
(0.0296 0.1590) 0.1788 (0.0237 0.1326) 0.1758 (0.0210 0.1193) 0.1607 (0.0205 0.1277)
Callithrix_jacchus   0.1917 (0.0301 0.1568) 0.1211 (0.0187 0.1545) 0.1469 (0.0232 0.1582) 0.1798
(0.0323 0.1798) 0.1694 (0.0264 0.1560) 0.1665 (0.0237 0.1424) 0.1612 (0.0232 0.1442) 0.1129 (0.0080
0.0705)
Tarsius_syrichta     0.1616 (0.0555 0.3433) 0.1311 (0.0442 0.3376) 0.1428 (0.0489 0.3424) 0.1715
(0.0578 0.3372) 0.1490 (0.0524 0.3518) 0.1446 (0.0496 0.3429) 0.1330 (0.0486 0.3656) 0.1388 (0.0448
0.3229) 0.1430 (0.0467 0.3267)
Microcebus_murinus   0.1916 (0.0639 0.3333) 0.1568 (0.0534 0.3407) 0.1656 (0.0572 0.3455) 0.1719
(0.0667 0.3880) 0.1667 (0.0615 0.3691) 0.1692 (0.0587 0.3468) 0.1561 (0.0549 0.3515) 0.1398 (0.0539
0.3851) 0.1458 (0.0567 0.3891) 0.1536 (0.0581 0.3783)
Daubentonia_madagascariensis 0.1723 (0.0506 0.2938) 0.1328 (0.0394 0.2968) 0.1463 (0.0441 0.3013)
0.1601 (0.0525 0.3279) 0.1535 (0.0464 0.3025) 0.1536 (0.0446 0.2902) 0.1386 (0.0408 0.2945) 0.1258
(0.0398 0.3167) 0.1313 (0.0427 0.3247) 0.1513 (0.0473 0.3126) 0.2022 (0.0288 0.1425)
Otolemur_garnettii   0.1351 (0.0619 0.4579) 0.1163 (0.0524 0.4509) 0.1189 (0.0543 0.4566) 0.1462
(0.0652 0.4459) 0.1262 (0.0601 0.4759) 0.1256 (0.0572 0.4552) 0.1227 (0.0553 0.4505) 0.1057 (0.0481
0.4554) 0.1097 (0.0510 0.4649) 0.1315 (0.0609 0.4633) 0.1247 (0.0380 0.3045) 0.1087 (0.0334 0.3073)


TREE #  1:  ((((((1, 2), 3), 4), ((5, 6), 7)), (8, 9)), 10, ((11, 12), 13));   MP score: 518
lnL(ntime: 23  np: 26):  -4793.179060      +0.000000
  14..15   15..16   16..17   17..18   18..19   19..1    19..2    18..3    17..4    16..20   20..21
21..5    21..6    20..7    15..22   22..8    22..9    14..10   14..23   23..24   24..11   24..12
23..13
 0.100256 0.022659 0.033395 0.021518 0.000004 0.028712 0.002080 0.018360 0.066310 0.041481 0.009844
0.025877 0.008170 0.020141 0.035103 0.027437 0.041681 0.185748 0.094684 0.029630 0.119172 0.059054
0.216303 4.024497 0.166487 0.702059

Note: Branch length is defined as number of nucleotide substitutions per codon (not per neucleotide
site).

tree length =   1.20762

((((((1: 0.028712, 2: 0.002080): 0.000004, 3: 0.018360): 0.021518, 4: 0.066310): 0.033395, ((5:
0.025877, 6: 0.008170): 0.009844, 7: 0.020141): 0.041481): 0.022659, (8: 0.027437, 9: 0.041681):
0.035103): 0.100256, 10: 0.185748, ((11: 0.119172, 12: 0.059054): 0.029630, 13: 0.216303): 0.094684);

((((((Homo_sapiens: 0.028712, Pan_troglodytes: 0.002080): 0.000004, Gorilla_gorilla_gorilla: 0.018360):
0.021518, Pongo_abelii: 0.066310): 0.033395, ((Macaca_mulatta: 0.025877, Papio_anubis: 0.008170):
0.009844, Chlorocebus_sabaeus: 0.020141): 0.041481): 0.022659, (Saimiri_boliviensis: 0.027437,
Callithrix_jacchus: 0.041681): 0.035103): 0.100256, Tarsius_syrichta: 0.185748, ((Microcebus_murinus:
0.119172, Daubentonia_madagascariensis: 0.059054): 0.029630, Otolemur_garnettii: 0.216303): 0.094684);
```

Detailed output identifying parameters

kappa (ts/tv) = 4.02450

w (dN/dS) for branches: 0.16649 0.70206

dN & dS for each branch

branch	t	N	S	dN/dS	dN	dS	N*dN	S*dS
14..15	0.100	1091.0	403.0	0.1665	0.0142	0.0854	15.5	34.4
15..16	0.023	1091.0	403.0	0.1665	0.0032	0.0193	3.5	7.8
16..17	0.033	1091.0	403.0	0.1665	0.0047	0.0284	5.2	11.5
17..18	0.022	1091.0	403.0	0.1665	0.0031	0.0183	3.3	7.4
18..19	0.000	1091.0	403.0	0.1665	0.0000	0.0000	0.0	0.0
19..1	0.029	1091.0	403.0	0.7021	0.0086	0.0122	9.4	4.9
19..2	0.002	1091.0	403.0	0.1665	0.0003	0.0018	0.3	0.7
18..3	0.018	1091.0	403.0	0.1665	0.0026	0.0156	2.8	6.3
17..4	0.066	1091.0	403.0	0.7021	0.0198	0.0282	21.6	11.4
16..20	0.041	1091.0	403.0	0.1665	0.0059	0.0353	6.4	14.2
20..21	0.010	1091.0	403.0	0.1665	0.0014	0.0084	1.5	3.4
21..5	0.026	1091.0	403.0	0.1665	0.0037	0.0220	4.0	8.9
21..6	0.008	1091.0	403.0	0.1665	0.0012	0.0070	1.3	2.8
20..7	0.020	1091.0	403.0	0.1665	0.0029	0.0172	3.1	6.9
15..22	0.035	1091.0	403.0	0.1665	0.0050	0.0299	5.4	12.1
22..8	0.027	1091.0	403.0	0.1665	0.0039	0.0234	4.2	9.4
22..9	0.042	1091.0	403.0	0.1665	0.0059	0.0355	6.4	14.3
14..10	0.186	1091.0	403.0	0.1665	0.0263	0.1582	28.7	63.8
14..23	0.095	1091.0	403.0	0.1665	0.0134	0.0807	14.6	32.5
23..24	0.030	1091.0	403.0	0.1665	0.0042	0.0252	4.6	10.2
24..11	0.119	1091.0	403.0	0.1665	0.0169	0.1015	18.4	40.9
24..12	0.059	1091.0	403.0	0.1665	0.0084	0.0503	9.1	20.3
23..13	0.216	1091.0	403.0	0.1665	0.0307	0.1842	33.5	74.3

tree length for dN: 0.1862
tree length for dS: 0.9882

dS tree:
((((((Homo_sapiens: 0.012232, Pan_troglodytes: 0.001772): 0.000003, Gorilla_gorilla_gorilla: 0.015639): 0.018329, Pongo_abelii: 0.028250): 0.028446, ((Macaca_mulatta: 0.022042, Papio_anubis: 0.006960): 0.008385, Chlorocebus_sabaeus: 0.017156): 0.035334): 0.019301, (Saimiri_boliviensis: 0.023371, Callithrix_jacchus: 0.035504): 0.029901): 0.085398, Tarsius_syrichta: 0.158220, ((Microcebus_murinus: 0.101511, Daubentonia_madagascariensis: 0.050303): 0.025239, Otolemur_garnettii: 0.184247): 0.080652);
dN tree:
((((((Homo_sapiens: 0.008588, Pan_troglodytes: 0.000295): 0.000001, Gorilla_gorilla_gorilla: 0.002604): 0.003052, Pongo_abelii: 0.019833): 0.004736, ((Macaca_mulatta: 0.003670, Papio_anubis: 0.001159): 0.001396, Chlorocebus_sabaeus: 0.002856): 0.005883): 0.003213, (Saimiri_boliviensis: 0.003891, Callithrix_jacchus: 0.005911): 0.004978): 0.014218, Tarsius_syrichta: 0.026342, ((Microcebus_murinus: 0.016900, Daubentonia_madagascariensis: 0.008375): 0.004202, Otolemur_garnettii: 0.030675): 0.013428);

w ratios as labels for TreeView:
((((((Homo_sapiens #0.7021 , Pan_troglodytes #0.1665) #0.1665 , Gorilla_gorilla_gorilla #0.1665) #0.1665 , Pongo_abelii #0.7021) #0.1665 , ((Macaca_mulatta #0.1665 , Papio_anubis #0.1665) #0.1665 , Chlorocebus_sabaeus #0.1665) #0.1665) #0.1665 , (Saimiri_boliviensis #0.1665 , Callithrix_jacchus #0.1665) #0.1665) #0.1665 , Tarsius_syrichta #0.1665 , ((Microcebus_murinus #0.1665 , Daubentonia_madagascariensis #0.1665) #0.1665 , Otolemur_garnettii #0.1665) #0.1665);

Main result file for model D: catarrhines-D.mlc

```
CODONML (in paml version 4.7b, October 2013)  catarrhines.phy
Model: several dN/dS ratios for branches for branches,  omega = 1.000 fixed

Codon frequency model: F3x4
ns =  13  ls = 498

Codon usage in sequences
--------------------------------------------------------------------------------------------------------------------------------------
Phe TTT  13  13  13  14  13  13 | Ser TCT   5   5   5   7   4   4 | Tyr TAT  11  10  11  10   9   8 | Cys TGT   3   4   4   6   5   5
    TTC  15  15  15  13  15  15 |     TCC  11  11  11   9  11  11 |     TAC  16  16  15  15  17  18 |     TGC   8   6   6   7   5   5
Leu TTA   1   1   1   2   2   2 |     TCA   2   2   2   3   2   2 | *** TAA   0   0   0   0   0   0 | *** TGA   0   0   0   0   0   0
    TTG   9   9   9   8   9   9 |     TCG   2   2   2   1   2   2 |     TAG   0   0   0   0   0   0 | Trp TGG   4   4   4   4   4   4
--------------------------------------------------------------------------------------------------------------------------------------
Leu CTT  10   9   9   8   6   6 | Pro CCT  13  14  15  12  10  10 | His CAT   6   6   6   6   6   6 | Arg CGT   4   4   3   3   4   3
    CTC   8   8   8  10   9   9 |     CCC  10  10   9  11  13  14 |     CAC  11  10  10  11  10  10 |     CGC   6   8   8   5   7   8
    CTA   2   2   2   2   2   2 |     CCA   3   3   3   3   2   2 | Gln CAA   5   4   4   4   4   4 |     CGA   3   5   5   5   5   5
    CTG  27  27  27  27  30  30 |     CCG   2   2   2   2   4   3 |     CAG  11  11  12  13  13  13 |     CGG   3   2   2   2   1   1
--------------------------------------------------------------------------------------------------------------------------------------
Ile ATT  10  10  10   9  11  10 | Thr ACT   6   6   6   9   9   9 | Asn AAT  12  11  12  11  14  12 | Ser AGT   2   2   3   2   3   3
    ATC  17  17  18  18  16  17 |     ACC  11  10  10   9   8   9 |     AAC   7   6   6   6   4   6 |     AGC  10  10  10  10   9   9
    ATA   1   1   0   1   1   1 |     ACA   9   9   9   8  11  10 | Lys AAA   3   3   3   3   3   3 | Arg AGA   1   0   0   1   0   0
Met ATG   8   9   9  11   8   8 |     ACG   7   6   6   4   3   3 |     AAG   8   8   8   8   9   8 |     AGG   6   6   6   7   6   6
--------------------------------------------------------------------------------------------------------------------------------------
Val GTT   3   3   3   3   4   5 | Ala GCT  10  10  10  11   9   9 | Asp GAT  17  18  17  17  21  21 | Gly GGT   2   3   3   2   4   4
    GTC  16  15  15  15  15  14 |     GCC   8  10   9   9  12  12 |     GAC  23  24  24  24  20  20 |     GGC  14  14  14  15  13  13
    GTA   2   3   2   3   2   2 |     GCA   2   2   2   2   2   2 | Glu GAA   5   5   5   4   5   4 |     GGA   9   9   9  10   9   9
    GTG  15  14  16  14  15  15 |     GCG   0   0   0   0   0   0 |     GAG  22  22  21  21  20  22 |     GGG   8   9   9   8   8   8
--------------------------------------------------------------------------------------------------------------------------------------


--------------------------------------------------------------------------------------------------------------------------------------
Phe TTT  13  13  12  16  10  12 | Ser TCT   4   3   4   4   4   4 | Tyr TAT   9  12  12  11   5   6 | Cys TGT   4   4   6   7   2   4
    TTC  15  15  15  15  17  14 |     TCC  11  11  11  13  14  14 |     TAC  17  14  14  14  20  19 |     TGC   6   7   5   4  10   8
Leu TTA   2   1   1   0   2   0 |     TCA   2   2   2   2   1   1 | *** TAA   0   0   0   0   0   0 | *** TGA   0   0   0   0   0   0
    TTG   9  11  10   9   5   6 |     TCG   2   2   2   3   2   2 |     TAG   0   0   0   0   0   0 | Trp TGG   4   4   4   4   4   4
--------------------------------------------------------------------------------------------------------------------------------------
Leu CTT   6  10  11   5   4   7 | Pro CCT  10  11  11  12   9   9 | His CAT   6   6   7   8   5   6 | Arg CGT   3   3   2   4   4   2
    CTC   9   9   8  12  12  11 |     CCC  14  13  12  10  15  12 |     CAC  10   9   9   8  12  12 |     CGC   8   7   7   6   9   8
    CTA   3   2   2   4   2   2 |     CCA   2   2   2   4   2   3 | Gln CAA   5   3   4   3   5   3 |     CGA   4   5   5   6   3   5
    CTG  29  25  27  25  30  30 |     CCG   3   2   2   1   3   3 |     CAG  12  14  12  12  12  13 |     CGG   2   1   2   3   3   3
--------------------------------------------------------------------------------------------------------------------------------------
Ile ATT   9  11  10   8   4   5 | Thr ACT   9   9   9   9   6   8 | Asn AAT  12  12  11  11   5   9 | Ser AGT   3   5   5   5   3   3
    ATC  17  17  18  19  22  19 |     ACC   9   7   7  10  14  12 |     AAC   6   5   5   5   9   7 |     AGC   9   7   8   5   8   7
    ATA   1   1   1   4   2   2 |     ACA   9   9  10   5   6   6 | Lys AAA   3   2   2   4   3   2 | Arg AGA   1   0   0   2   2   2
Met ATG   9   9  10  10  11   9 |     ACG   4   5   4   5   6   6 |     AAG   8   7   7  10  10   9 |     AGG   6   7   7   4   3   4
--------------------------------------------------------------------------------------------------------------------------------------
Val GTT   3   7   8   6   4   4 | Ala GCT   9  12  12  10   9   7 | Asp GAT  20  20  20  19  11  17 | Gly GGT   4   4   3   6   3   5
    GTC  16  12   9  10  12  14 |     GCC  11  12  12   9  13  13 |     GAC  21  22  22  24  31  25 |     GGC  14  13  13  14  18  15
    GTA   2   2   2   4   3   2 |     GCA   2   2   2   2   0   3 | Glu GAA   4   5   6  11   5   6 |     GGA   8   8   9   7   6   7
    GTG  15  14  15  13  14  18 |     GCG   0   0   1   0   2   0 |     GAG  21  22  21  14  17  19 |     GGG   9   9   8   8  10  10
--------------------------------------------------------------------------------------------------------------------------------------


------------------------------------------------------
Phe TTT  10 | Ser TCT   3 | Tyr TAT   3 | Cys TGT   5
    TTC  20 |     TCC  12 |     TAC  22 |     TGC   8
Leu TTA   2 |     TCA   2 | *** TAA   0 | *** TGA   0
    TTG   6 |     TCG   1 |     TAG   0 | Trp TGG   4
------------------------------------------------------
Leu CTT   7 | Pro CCT  13 | His CAT   6 | Arg CGT   4
    CTC  10 |     CCC  10 |     CAC  11 |     CGC   7
    CTA   4 |     CCA   2 | Gln CAA   5 |     CGA   3
    CTG  26 |     CCG   2 |     CAG  11 |     CGG   3
------------------------------------------------------
Ile ATT   5 | Thr ACT   7 | Asn AAT   6 | Ser AGT   3
    ATC  20 |     ACC  13 |     AAC  10 |     AGC   7
    ATA   2 |     ACA   6 | Lys AAA   3 | Arg AGA   3
Met ATG  10 |     ACG   4 |     AAG   9 |     AGG   3
------------------------------------------------------
Val GTT   5 | Ala GCT   7 | Asp GAT  11 | Gly GGT   5
    GTC  14 |     GCC  15 |     GAC  28 |     GGC  19
    GTA   4 |     GCA   3 | Glu GAA  11 |     GGA   5
    GTG  14 |     GCG   1 |     GAG  15 |     GGG   8
------------------------------------------------------

Codon position x base (3x4) table for each sequence.

#1: Homo_sapiens
position  1:    T:0.20080    C:0.24900    A:0.23695    G:0.31325
position  2:    T:0.31526    C:0.20281    A:0.31526    G:0.16667
position  3:    T:0.25502    C:0.38353    A:0.09639    G:0.26506
Average         T:0.25703    C:0.27845    A:0.21620    G:0.24833

#2: Pan_troglodytes
position  1:    T:0.19679    C:0.25100    A:0.22892    G:0.32329
position  2:    T:0.31325    C:0.20482    A:0.30924    G:0.17269
position  3:    T:0.25703    C:0.38153    A:0.09839    G:0.26305
Average         T:0.25569    C:0.27912    A:0.21218    G:0.25301
```

```
#3: Gorilla_gorilla_gorilla
position  1:    T:0.19679    C:0.25100    A:0.23293    G:0.31928
position  2:    T:0.31526    C:0.20281    A:0.30924    G:0.17269
position  3:    T:0.26104    C:0.37751    A:0.09438    G:0.26707
Average         T:0.25770    C:0.27711    A:0.21218    G:0.25301

#4: Pongo_abelii
position  1:    T:0.19880    C:0.24900    A:0.23494    G:0.31727
position  2:    T:0.31727    C:0.20080    A:0.30723    G:0.17470
position  3:    T:0.26104    C:0.37550    A:0.10241    G:0.26104
Average         T:0.25904    C:0.27510    A:0.21486    G:0.25100

#5: Macaca_mulatta
position  1:    T:0.19679    C:0.25301    A:0.23092    G:0.31928
position  2:    T:0.31727    C:0.20482    A:0.31124    G:0.16667
position  3:    T:0.26506    C:0.36948    A:0.10040    G:0.26506
Average         T:0.25971    C:0.27577    A:0.21419    G:0.25033

#6: Papio_anubis
position  1:    T:0.19679    C:0.25301    A:0.22892    G:0.32129
position  2:    T:0.31727    C:0.20482    A:0.31124    G:0.16667
position  3:    T:0.25703    C:0.38153    A:0.09639    G:0.26506
Average         T:0.25703    C:0.27979    A:0.21218    G:0.25100

#7: Chlorocebus_sabaeus
position  1:    T:0.19679    C:0.25301    A:0.23092    G:0.31928
position  2:    T:0.31727    C:0.20281    A:0.30924    G:0.17068
position  3:    T:0.24900    C:0.38755    A:0.09639    G:0.26707
Average         T:0.25435    C:0.28112    A:0.21218    G:0.25234

#8: Saimiri_boliviensis
position  1:    T:0.19880    C:0.24498    A:0.22691    G:0.32932
position  2:    T:0.31928    C:0.20482    A:0.30723    G:0.16867
position  3:    T:0.28514    C:0.36145    A:0.08835    G:0.26506
Average         T:0.26774    C:0.27041    A:0.20750    G:0.25435

#9: Callithrix_jacchus
position  1:    T:0.19679    C:0.24699    A:0.22892    G:0.32731
position  2:    T:0.31928    C:0.20683    A:0.30522    G:0.16867
position  3:    T:0.28715    C:0.35141    A:0.09639    G:0.26506
Average         T:0.26774    C:0.26841    A:0.21017    G:0.25368

#10: Tarsius_syrichta
position  1:    T:0.20482    C:0.24699    A:0.23293    G:0.31526
position  2:    T:0.32129    C:0.19880    A:0.30924    G:0.17068
position  3:    T:0.28313    C:0.35743    A:0.11647    G:0.24297
Average         T:0.26975    C:0.26774    A:0.21954    G:0.24297

#11: Microcebus_murinus
position  1:    T:0.19277    C:0.26104    A:0.22892    G:0.31727
position  2:    T:0.30924    C:0.21285    A:0.30120    G:0.17671
position  3:    T:0.17671    C:0.47390    A:0.08434    G:0.26506
Average         T:0.22624    C:0.31593    A:0.20482    G:0.25301

#12: Daubentonia_madagascariensis
position  1:    T:0.18876    C:0.25904    A:0.22088    G:0.33133
position  2:    T:0.31124    C:0.20683    A:0.30723    G:0.17470
position  3:    T:0.21687    C:0.42169    A:0.08835    G:0.27309
Average         T:0.23896    C:0.29585    A:0.20549    G:0.25971

#13: Otolemur_garnettii
position  1:    T:0.19679    C:0.24900    A:0.22289    G:0.33133
position  2:    T:0.31928    C:0.20281    A:0.30321    G:0.17470
position  3:    T:0.20080    C:0.45382    A:0.11044    G:0.23494
Average         T:0.23896    C:0.30187    A:0.21218    G:0.24699

Sums of codon usage counts
------------------------------------------------------------------------------
Phe F TTT     165 | Ser S TCT      56 | Tyr Y TAT     117 | Cys C TGT      59
      TTC     199 |       TCC     150 |       TAC     217 |       TGC      85
Leu L TTA      17 |       TCA      25 | *** * TAA       0 | *** * TGA       0
      TTG     109 |       TCG      25 |       TAG       0 | Trp W TGG      52
------------------------------------------------------------------------------
Leu L CTT      98 | Pro P CCT     149 | His H CAT      80 | Arg R CGT      43
      CTC     123 |       CCC     153 |       CAC     133 |       CGC      94
      CTA      31 |       CCA      33 | Gln Q CAA      53 |       CGA      59
```

```
      CTG     360 |       CCG      31 |       CAG     159 |       CGG      28
------------------------------------------------------------------------------
Ile I ATT     112 | Thr T ACT     102 | Asn N AAT     138 | Ser S AGT      42
      ATC     235 |       ACC     129 |       AAC      82 |       AGC     109
      ATA      18 |       ACA     107 | Lys K AAA      37 | Arg R AGA      12
Met M ATG     121 |       ACG      63 |       AAG     109 |       AGG      71
------------------------------------------------------------------------------
Val V GTT      58 | Ala A GCT     125 | Asp D GAT     229 | Gly G GGT      48
      GTC     177 |       GCC     145 |       GAC     308 |       GGC     189
      GTA      33 |       GCA      26 | Glu E GAA      76 |       GGA     105
      GTG     192 |       GCG       4 |       GAG     257 |       GGG     112
------------------------------------------------------------------------------


Codon position x base (3x4) table, overall

position  1:    T:0.19710    C:0.25131    A:0.22969    G:0.32190
position  2:    T:0.31634    C:0.20436    A:0.30816    G:0.17115
position  3:    T:0.25039    C:0.39049    A:0.09762    G:0.26151
Average         T:0.25461    C:0.28205    A:0.21182    G:0.25152


Nei & Gojobori 1986. dN/dS (dN, dS)
(Note: This matrix is not used in later ML. analysis.
Use runmode = -2 for ML pairwise comparison.)

Homo_sapiens
Pan_troglodytes      1.2604 (0.0106 0.0084)
Gorilla_gorilla_gorilla 1.3389 (0.0151 0.0113) 0.7351 (0.0062 0.0084)
Pongo_abelii         0.4378 (0.0296 0.0676) 0.2910 (0.0187 0.0643) 0.3714 (0.0250 0.0674)
Macaca_mulatta       0.2054 (0.0269 0.1309) 0.1261 (0.0160 0.1271) 0.1712 (0.0224 0.1306) 0.2017
(0.0278 0.1377)
Papio_anubis         0.2052 (0.0242 0.1177) 0.1169 (0.0133 0.1141) 0.1672 (0.0196 0.1174) 0.1773
(0.0232 0.1311) 0.2434 (0.0062 0.0255)
Chlorocebus_sabaeus  0.2404 (0.0260 0.1080) 0.1448 (0.0151 0.1045) 0.1989 (0.0214 0.1078) 0.2216
(0.0269 0.1212) 0.2126 (0.0098 0.0459) 0.2498 (0.0071 0.0284)
Saimiri_boliviensis  0.1999 (0.0273 0.1366) 0.1180 (0.0165 0.1395) 0.1466 (0.0210 0.1430) 0.1860
(0.0296 0.1590) 0.1788 (0.0237 0.1326) 0.1758 (0.0210 0.1193) 0.1607 (0.0205 0.1277)
Callithrix_jacchus   0.1917 (0.0301 0.1568) 0.1211 (0.0187 0.1545) 0.1469 (0.0232 0.1582) 0.1798
(0.0323 0.1798) 0.1694 (0.0264 0.1560) 0.1665 (0.0237 0.1424) 0.1612 (0.0232 0.1442) 0.1129 (0.0080
0.0705)
Tarsius_syrichta     0.1616 (0.0555 0.3433) 0.1311 (0.0442 0.3376) 0.1428 (0.0489 0.3424) 0.1715
(0.0578 0.3372) 0.1490 (0.0524 0.3518) 0.1446 (0.0496 0.3429) 0.1330 (0.0486 0.3656) 0.1388 (0.0448
0.3229) 0.1430 (0.0467 0.3267)
Microcebus_murinus   0.1916 (0.0639 0.3333) 0.1568 (0.0534 0.3407) 0.1656 (0.0572 0.3455) 0.1719
(0.0667 0.3880) 0.1667 (0.0615 0.3691) 0.1692 (0.0587 0.3468) 0.1561 (0.0549 0.3515) 0.1398 (0.0539
0.3851) 0.1458 (0.0567 0.3891) 0.1536 (0.0581 0.3783)
Daubentonia_madagascariensis 0.1723 (0.0506 0.2938) 0.1328 (0.0394 0.2968) 0.1463 (0.0441 0.3013)
0.1601 (0.0525 0.3279) 0.1535 (0.0464 0.3025) 0.1536 (0.0446 0.2902) 0.1386 (0.0408 0.2945) 0.1258
(0.0398 0.3167) 0.1313 (0.0427 0.3247) 0.1513 (0.0473 0.3126) 0.2022 (0.0288 0.1425)
Otolemur_garnettii   0.1351 (0.0619 0.4579) 0.1163 (0.0524 0.4509) 0.1189 (0.0543 0.4566) 0.1462
(0.0652 0.4459) 0.1262 (0.0601 0.4759) 0.1256 (0.0572 0.4552) 0.1227 (0.0553 0.4505) 0.1057 (0.0481
0.4554) 0.1097 (0.0510 0.4649) 0.1315 (0.0609 0.4633) 0.1247 (0.0380 0.3045) 0.1087 (0.0334 0.3073)


TREE #  1:  ((((((1, 2), 3), 4), ((5, 6), 7)), (8, 9)), 10, ((11, 12), 13));   MP score: 518
lnL(ntime: 23  np: 25):  -4793.763533      +0.000000
  14..15   15..16   16..17   17..18   18..19   19..1    19..2    18..3    17..4    16..20   20..21
21..5    21..6    20..7    15..22   22..8    22..9    14..10   14..23   23..24   24..11   24..12
23..13
 0.100213 0.022753 0.033515 0.021843 0.000004 0.028743 0.002076 0.018363 0.065865 0.041420 0.009843
0.025875 0.008173 0.020140 0.035133 0.027394 0.041743 0.185729 0.094717 0.029715 0.119196 0.059065
0.216286 4.053354 0.166602

Note: Branch length is defined as number of nucleotide substitutions per codon (not per neucleotide
site).

tree length =   1.20780

((((((1: 0.028743, 2: 0.002076): 0.000004, 3: 0.018363): 0.021843, 4: 0.065865): 0.033515, ((5:
0.025875, 6: 0.008173): 0.009843, 7: 0.020140): 0.041420): 0.022753, (8: 0.027394, 9: 0.041743):
0.035133): 0.100213, 10: 0.185729, ((11: 0.119196, 12: 0.059065): 0.029715, 13: 0.216286): 0.094717);

((((((Homo_sapiens: 0.028743, Pan_troglodytes: 0.002076): 0.000004, Gorilla_gorilla_gorilla: 0.018363):
0.021843, Pongo_abelii: 0.065865): 0.033515, ((Macaca_mulatta: 0.025875, Papio_anubis: 0.008173):
0.009843, Chlorocebus_sabaeus: 0.020140): 0.041420): 0.022753, (Saimiri_boliviensis: 0.027394,
Callithrix_jacchus: 0.041743): 0.035133): 0.100213, Tarsius_syrichta: 0.185729, ((Microcebus_murinus:
```

0.119196, Daubentonia_madagascariensis: 0.059065): 0.029715, Otolemur_garnettii: 0.216286): 0.094717);

Detailed output identifying parameters

kappa (ts/tv) = 4.05335

w (dN/dS) for branches: 0.16660 1.00000

dN & dS for each branch

branch	t	N	S	dN/dS	dN	dS	N*dN	S*dS
14..15	0.100	1090.6	403.4	0.1666	0.0142	0.0853	15.5	34.4
15..16	0.023	1090.6	403.4	0.1666	0.0032	0.0194	3.5	7.8
16..17	0.034	1090.6	403.4	0.1666	0.0048	0.0285	5.2	11.5
17..18	0.022	1090.6	403.4	0.1666	0.0031	0.0186	3.4	7.5
18..19	0.000	1090.6	403.4	0.1666	0.0000	0.0000	0.0	0.0
19..1	0.029	1090.6	403.4	1.0000	0.0096	0.0096	10.4	3.9
19..2	0.002	1090.6	403.4	0.1666	0.0003	0.0018	0.3	0.7
18..3	0.018	1090.6	403.4	0.1666	0.0026	0.0156	2.8	6.3
17..4	0.066	1090.6	403.4	1.0000	0.0220	0.0220	23.9	8.9
16..20	0.041	1090.6	403.4	0.1666	0.0059	0.0353	6.4	14.2
20..21	0.010	1090.6	403.4	0.1666	0.0014	0.0084	1.5	3.4
21..5	0.026	1090.6	403.4	0.1666	0.0037	0.0220	4.0	8.9
21..6	0.008	1090.6	403.4	0.1666	0.0012	0.0070	1.3	2.8
20..7	0.020	1090.6	403.4	0.1666	0.0029	0.0171	3.1	6.9
15..22	0.035	1090.6	403.4	0.1666	0.0050	0.0299	5.4	12.1
22..8	0.027	1090.6	403.4	0.1666	0.0039	0.0233	4.2	9.4
22..9	0.042	1090.6	403.4	0.1666	0.0059	0.0355	6.5	14.3
14..10	0.186	1090.6	403.4	0.1666	0.0263	0.1581	28.7	63.8
14..23	0.095	1090.6	403.4	0.1666	0.0134	0.0806	14.6	32.5
23..24	0.030	1090.6	403.4	0.1666	0.0042	0.0253	4.6	10.2
24..11	0.119	1090.6	403.4	0.1666	0.0169	0.1015	18.4	40.9
24..12	0.059	1090.6	403.4	0.1666	0.0084	0.0503	9.1	20.3
23..13	0.216	1090.6	403.4	0.1666	0.0307	0.1841	33.4	74.3

tree length for dN: 0.1894
tree length for dS: 0.9790

dS tree:
((((((Homo_sapiens: 0.009581, Pan_troglodytes: 0.001767): 0.000003, Gorilla_gorilla_gorilla: 0.015629): 0.018591, Pongo_abelii: 0.021955): 0.028525, ((Macaca_mulatta: 0.022023, Papio_anubis: 0.006956): 0.008378, Chlorocebus_sabaeus: 0.017142): 0.035254): 0.019365, (Saimiri_boliviensis: 0.023315, Callithrix_jacchus: 0.035528): 0.029902): 0.085293, Tarsius_syrichta: 0.158078, ((Microcebus_murinus: 0.101451, Daubentonia_madagascariensis: 0.050271): 0.025291, Otolemur_garnettii: 0.184086): 0.080615);
dN tree:
((((((Homo_sapiens: 0.009581, Pan_troglodytes: 0.000294): 0.000001, Gorilla_gorilla_gorilla: 0.002604): 0.003097, Pongo_abelii: 0.021955): 0.004752, ((Macaca_mulatta: 0.003669, Papio_anubis: 0.001159): 0.001396, Chlorocebus_sabaeus: 0.002856): 0.005873): 0.003226, (Saimiri_boliviensis: 0.003884, Callithrix_jacchus: 0.005919): 0.004982): 0.014210, Tarsius_syrichta: 0.026336, ((Microcebus_murinus: 0.016902, Daubentonia_madagascariensis: 0.008375): 0.004214, Otolemur_garnettii: 0.030669): 0.013431);

w ratios as labels for TreeView:
((((((Homo_sapiens #1.0000 , Pan_troglodytes #0.1666) #0.1666 , Gorilla_gorilla_gorilla #0.1666) #0.1666 , Pongo_abelii #1.0000) #0.1666 , ((Macaca_mulatta #0.1666 , Papio_anubis #0.1666) #0.1666 , Chlorocebus_sabaeus #0.1666) #0.1666) #0.1666 , (Saimiri_boliviensis #0.1666 , Callithrix_jacchus #0.1666) #0.1666) #0.1666 , Tarsius_syrichta #0.1666 , ((Microcebus_murinus #0.1666 , Daubentonia_madagascariensis #0.1666) #0.1666 , Otolemur_garnettii #0.1666) #0.1666);

Main result file for model E: catarrhines-E.mlc

```
CODONML (in paml version 4.7b, October 2013)  catarrhines.phy
Model: several dN/dS ratios for branches for branches,
Codon frequency model: F3x4
ns =  13  ls = 498

Codon usage in sequences
------------------------------------------------------------------------------------------------------
Phe TTT  13  13  13  14  13  13 | Ser TCT   5   5   5   7   4   4 | Tyr TAT  11  10  11  10   9   8 | Cys TGT   3   4   4   6   5   5
    TTC  15  15  15  13  15  15 |     TCC  11  11  11   9  11  11 |     TAC  16  16  15  15  17  18 |     TGC   8   6   6   7   5   5
Leu TTA   1   1   1   2   2   2 |     TCA   2   2   2   3   2   2 | *** TAA   0   0   0   0   0   0 | *** TGA   0   0   0   0   0   0
    TTG   9   9   9   8   9   9 |     TCG   2   2   2   1   2   2 |     TAG   0   0   0   0   0   0 | Trp TGG   4   4   4   4   4   4
------------------------------------------------------------------------------------------------------
Leu CTT  10   9   9   8   6   6 | Pro CCT  13  14  15  12  10  10 | His CAT   6   6   6   6   6   6 | Arg CGT   4   4   3   3   4   3
    CTC   8   8   8  10   9   9 |     CCC  10  10   9  11  13  14 |     CAC  11  10  10  11  10  10 |     CGC   6   8   8   5   7   8
    CTA   2   2   2   2   2   2 |     CCA   3   3   3   3   2   2 | Gln CAA   5   4   4   4   4   4 |     CGA   3   5   5   5   5   5
    CTG  27  27  27  27  30  30 |     CCG   2   2   2   2   4   3 |     CAG  11  11  12  13  13  13 |     CGG   3   2   2   2   1   1
------------------------------------------------------------------------------------------------------
Ile ATT  10  10  10   9  11  10 | Thr ACT   6   6   6   9   9   9 | Asn AAT  12  11  12  11  14  12 | Ser AGT   2   2   3   2   3   3
    ATC  17  17  18  18  16  17 |     ACC  11  10  10   9   8   9 |     AAC   7   6   6   6   4   6 |     AGC  10  10  10  10   9   9
    ATA   1   1   0   1   1   1 |     ACA   9   9   9   8  11  10 | Lys AAA   3   3   3   3   3   3 | Arg AGA   1   0   0   1   0   0
Met ATG   8   9   9  11   8   8 |     ACG   7   6   6   4   3   3 |     AAG   8   8   8   8   9   8 |     AGG   6   6   6   7   6   6
------------------------------------------------------------------------------------------------------
Val GTT   3   3   3   3   4   5 | Ala GCT  10  10  10  11   9   9 | Asp GAT  17  18  17  17  21  21 | Gly GGT   2   3   3   2   4   4
    GTC  16  15  15  15  15  14 |     GCC   8  10   9   9  12  12 |     GAC  23  24  24  24  20  20 |     GGC  14  14  14  15  13  13
    GTA   2   3   2   3   2   2 |     GCA   2   2   2   2   2   2 | Glu GAA   5   5   5   4   5   4 |     GGA   9   9   9  10   9   9
    GTG  15  14  16  14  15  15 |     GCG   0   0   0   0   0   0 |     GAG  22  22  21  21  20  22 |     GGG   8   9   9   8   8   8
------------------------------------------------------------------------------------------------------


------------------------------------------------------------------------------------------------------
Phe TTT  13  13  12  16  10  12 | Ser TCT   4   3   4   4   4   4 | Tyr TAT   9  12  12  11   5   6 | Cys TGT   4   4   6   7   2   4
    TTC  15  15  15  15  17  14 |     TCC  11  11  11  13  14  14 |     TAC  17  14  14  14  20  19 |     TGC   6   7   5   4  10   8
Leu TTA   2   1   1   0   2   0 |     TCA   2   2   2   2   1   1 | *** TAA   0   0   0   0   0   0 | *** TGA   0   0   0   0   0   0
    TTG   9  11  10   9   5   6 |     TCG   2   2   2   3   2   2 |     TAG   0   0   0   0   0   0 | Trp TGG   4   4   4   4   4   4
------------------------------------------------------------------------------------------------------
Leu CTT   6  10  11   5   4   7 | Pro CCT  10  11  11  12   9   9 | His CAT   6   6   7   8   5   6 | Arg CGT   3   3   2   4   4   2
    CTC   9   9   8  12  12  11 |     CCC  14  13  12  10  15  12 |     CAC  10   9   9   8  12  12 |     CGC   8   7   7   6   9   8
    CTA   3   2   2   4   2   2 |     CCA   2   2   2   4   2   3 | Gln CAA   5   3   4   3   5   3 |     CGA   4   5   5   6   3   5
    CTG  29  25  27  25  30  30 |     CCG   3   2   2   1   3   3 |     CAG  12  14  12  12  12  13 |     CGG   2   1   2   3   3   3
------------------------------------------------------------------------------------------------------
Ile ATT   9  11  10   8   4   5 | Thr ACT   9   9   9   9   6   8 | Asn AAT  12  12  11  11   5   9 | Ser AGT   3   5   5   5   3   3
    ATC  17  17  18  19  22  19 |     ACC   9   7   7  10  14  12 |     AAC   6   5   5   5   9   7 |     AGC   9   7   8   5   8   7
    ATA   1   1   1   4   2   2 |     ACA   9   9  10   5   6   6 | Lys AAA   3   2   2   4   3   2 | Arg AGA   1   0   0   2   2   2
Met ATG   9   9  10  10  11   9 |     ACG   4   5   4   5   6   6 |     AAG   8   7   7  10  10   9 |     AGG   6   7   7   4   3   4
------------------------------------------------------------------------------------------------------
Val GTT   3   7   8   6   4   4 | Ala GCT   9  12  12  10   9   7 | Asp GAT  20  20  20  19  11  17 | Gly GGT   4   4   3   6   3   5
    GTC  16  12   9  10  12  14 |     GCC  11  12  12   9  13  13 |     GAC  21  22  22  24  31  25 |     GGC  14  13  13  14  18  15
    GTA   2   2   2   4   3   2 |     GCA   2   2   2   2   0   3 | Glu GAA   4   5   6  11   5   6 |     GGA   8   8   9   7   6   7
    GTG  15  14  15  13  14  18 |     GCG   0   0   1   0   2   0 |     GAG  21  22  21  14  17  19 |     GGG   9   9   8   8  10  10
------------------------------------------------------------------------------------------------------


------------------------------------------------------
Phe TTT  10 | Ser TCT   3 | Tyr TAT   3 | Cys TGT   5
    TTC  20 |     TCC  12 |     TAC  22 |     TGC   8
Leu TTA   2 |     TCA   2 | *** TAA   0 | *** TGA   0
    TTG   6 |     TCG   1 |     TAG   0 | Trp TGG   4
------------------------------------------------------
Leu CTT   7 | Pro CCT  13 | His CAT   6 | Arg CGT   4
    CTC  10 |     CCC  10 |     CAC  11 |     CGC   7
    CTA   4 |     CCA   2 | Gln CAA   5 |     CGA   3
    CTG  26 |     CCG   2 |     CAG  11 |     CGG   3
------------------------------------------------------
Ile ATT   5 | Thr ACT   7 | Asn AAT   6 | Ser AGT   3
    ATC  20 |     ACC  13 |     AAC  10 |     AGC   7
    ATA   2 |     ACA   6 | Lys AAA   3 | Arg AGA   3
Met ATG  10 |     ACG   4 |     AAG   9 |     AGG   3
------------------------------------------------------
Val GTT   5 | Ala GCT   7 | Asp GAT  11 | Gly GGT   5
    GTC  14 |     GCC  15 |     GAC  28 |     GGC  19
    GTA   4 |     GCA   3 | Glu GAA  11 |     GGA   5
    GTG  14 |     GCG   1 |     GAG  15 |     GGG   8
------------------------------------------------------

Codon position x base (3x4) table for each sequence.

#1: Homo_sapiens
position  1:    T:0.20080    C:0.24900    A:0.23695    G:0.31325
position  2:    T:0.31526    C:0.20281    A:0.31526    G:0.16667
position  3:    T:0.25502    C:0.38353    A:0.09639    G:0.26506
Average         T:0.25703    C:0.27845    A:0.21620    G:0.24833

#2: Pan_troglodytes
position  1:    T:0.19679    C:0.25100    A:0.22892    G:0.32329
position  2:    T:0.31325    C:0.20482    A:0.30924    G:0.17269
position  3:    T:0.25703    C:0.38153    A:0.09839    G:0.26305
Average         T:0.25569    C:0.27912    A:0.21218    G:0.25301
```

```
#3: Gorilla_gorilla_gorilla
position  1:    T:0.19679    C:0.25100    A:0.23293    G:0.31928
position  2:    T:0.31526    C:0.20281    A:0.30924    G:0.17269
position  3:    T:0.26104    C:0.37751    A:0.09438    G:0.26707
Average         T:0.25770    C:0.27711    A:0.21218    G:0.25301

#4: Pongo_abelii
position  1:    T:0.19880    C:0.24900    A:0.23494    G:0.31727
position  2:    T:0.31727    C:0.20080    A:0.30723    G:0.17470
position  3:    T:0.26104    C:0.37550    A:0.10241    G:0.26104
Average         T:0.25904    C:0.27510    A:0.21486    G:0.25100

#5: Macaca_mulatta
position  1:    T:0.19679    C:0.25301    A:0.23092    G:0.31928
position  2:    T:0.31727    C:0.20482    A:0.31124    G:0.16667
position  3:    T:0.26506    C:0.36948    A:0.10040    G:0.26506
Average         T:0.25971    C:0.27577    A:0.21419    G:0.25033

#6: Papio_anubis
position  1:    T:0.19679    C:0.25301    A:0.22892    G:0.32129
position  2:    T:0.31727    C:0.20482    A:0.31124    G:0.16667
position  3:    T:0.25703    C:0.38153    A:0.09639    G:0.26506
Average         T:0.25703    C:0.27979    A:0.21218    G:0.25100

#7: Chlorocebus_sabaeus
position  1:    T:0.19679    C:0.25301    A:0.23092    G:0.31928
position  2:    T:0.31727    C:0.20281    A:0.30924    G:0.17068
position  3:    T:0.24900    C:0.38755    A:0.09639    G:0.26707
Average         T:0.25435    C:0.28112    A:0.21218    G:0.25234

#8: Saimiri_boliviensis
position  1:    T:0.19880    C:0.24498    A:0.22691    G:0.32932
position  2:    T:0.31928    C:0.20482    A:0.30723    G:0.16867
position  3:    T:0.28514    C:0.36145    A:0.08835    G:0.26506
Average         T:0.26774    C:0.27041    A:0.20750    G:0.25435

#9: Callithrix_jacchus
position  1:    T:0.19679    C:0.24699    A:0.22892    G:0.32731
position  2:    T:0.31928    C:0.20683    A:0.30522    G:0.16867
position  3:    T:0.28715    C:0.35141    A:0.09639    G:0.26506
Average         T:0.26774    C:0.26841    A:0.21017    G:0.25368

#10: Tarsius_syrichta
position  1:    T:0.20482    C:0.24699    A:0.23293    G:0.31526
position  2:    T:0.32129    C:0.19880    A:0.30924    G:0.17068
position  3:    T:0.28313    C:0.35743    A:0.11647    G:0.24297
Average         T:0.26975    C:0.26774    A:0.21954    G:0.24297

#11: Microcebus_murinus
position  1:    T:0.19277    C:0.26104    A:0.22892    G:0.31727
position  2:    T:0.30924    C:0.21285    A:0.30120    G:0.17671
position  3:    T:0.17671    C:0.47390    A:0.08434    G:0.26506
Average         T:0.22624    C:0.31593    A:0.20482    G:0.25301

#12: Daubentonia_madagascariensis
position  1:    T:0.18876    C:0.25904    A:0.22088    G:0.33133
position  2:    T:0.31124    C:0.20683    A:0.30723    G:0.17470
position  3:    T:0.21687    C:0.42169    A:0.08835    G:0.27309
Average         T:0.23896    C:0.29585    A:0.20549    G:0.25971

#13: Otolemur_garnettii
position  1:    T:0.19679    C:0.24900    A:0.22289    G:0.33133
position  2:    T:0.31928    C:0.20281    A:0.30321    G:0.17470
position  3:    T:0.20080    C:0.45382    A:0.11044    G:0.23494
Average         T:0.23896    C:0.30187    A:0.21218    G:0.24699

Sums of codon usage counts
------------------------------------------------------------------------------
Phe F TTT     165 | Ser S TCT      56 | Tyr Y TAT     117 | Cys C TGT      59
      TTC     199 |       TCC     150 |       TAC     217 |       TGC      85
Leu L TTA      17 |       TCA      25 | *** * TAA       0 | *** * TGA       0
      TTG     109 |       TCG      25 |       TAG       0 | Trp W TGG      52
------------------------------------------------------------------------------
Leu L CTT      98 | Pro P CCT     149 | His H CAT      80 | Arg R CGT      43
      CTC     123 |       CCC     153 |       CAC     133 |       CGC      94
      CTA      31 |       CCA      33 | Gln Q CAA      53 |       CGA      59
      CTG     360 |       CCG      31 |       CAG     159 |       CGG      28
```

```
------------------------------------------------------------------------------
Ile I ATT     112 | Thr T ACT     102 | Asn N AAT     138 | Ser S AGT      42
      ATC     235 |       ACC     129 |       AAC      82 |       AGC     109
      ATA      18 |       ACA     107 | Lys K AAA      37 | Arg R AGA      12
Met M ATG     121 |       ACG      63 |       AAG     109 |       AGG      71
------------------------------------------------------------------------------
Val V GTT      58 | Ala A GCT     125 | Asp D GAT     229 | Gly G GGT      48
      GTC     177 |       GCC     145 |       GAC     308 |       GGC     189
      GTA      33 |       GCA      26 | Glu E GAA      76 |       GGA     105
      GTG     192 |       GCG       4 |       GAG     257 |       GGG     112
------------------------------------------------------------------------------


Codon position x base (3x4) table, overall

position  1:    T:0.19710    C:0.25131    A:0.22969    G:0.32190
position  2:    T:0.31634    C:0.20436    A:0.30816    G:0.17115
position  3:    T:0.25039    C:0.39049    A:0.09762    G:0.26151
Average         T:0.25461    C:0.28205    A:0.21182    G:0.25152


Nei & Gojobori 1986. dN/dS (dN, dS)
(Note: This matrix is not used in later ML. analysis.
Use runmode = -2 for ML pairwise comparison.)

Homo_sapiens
Pan_troglodytes      1.2604 (0.0106 0.0084)
Gorilla_gorilla_gorilla 1.3389 (0.0151 0.0113) 0.7351 (0.0062 0.0084)
Pongo_abelii         0.4378 (0.0296 0.0676) 0.2910 (0.0187 0.0643) 0.3714 (0.0250 0.0674)
Macaca_mulatta       0.2054 (0.0269 0.1309) 0.1261 (0.0160 0.1271) 0.1712 (0.0224 0.1306) 0.2017
(0.0278 0.1377)
Papio_anubis         0.2052 (0.0242 0.1177) 0.1169 (0.0133 0.1141) 0.1672 (0.0196 0.1174) 0.1773
(0.0232 0.1311) 0.2434 (0.0062 0.0255)
Chlorocebus_sabaeus  0.2404 (0.0260 0.1080) 0.1448 (0.0151 0.1045) 0.1989 (0.0214 0.1078) 0.2216
(0.0269 0.1212) 0.2126 (0.0098 0.0459) 0.2498 (0.0071 0.0284)
Saimiri_boliviensis  0.1999 (0.0273 0.1366) 0.1180 (0.0165 0.1395) 0.1466 (0.0210 0.1430) 0.1860
(0.0296 0.1590) 0.1788 (0.0237 0.1326) 0.1758 (0.0210 0.1193) 0.1607 (0.0205 0.1277)
Callithrix_jacchus   0.1917 (0.0301 0.1568) 0.1211 (0.0187 0.1545) 0.1469 (0.0232 0.1582) 0.1798
(0.0323 0.1798) 0.1694 (0.0264 0.1560) 0.1665 (0.0237 0.1424) 0.1612 (0.0232 0.1442) 0.1129 (0.0080
0.0705)
Tarsius_syrichta     0.1616 (0.0555 0.3433) 0.1311 (0.0442 0.3376) 0.1428 (0.0489 0.3424) 0.1715
(0.0578 0.3372) 0.1490 (0.0524 0.3518) 0.1446 (0.0496 0.3429) 0.1330 (0.0486 0.3656) 0.1388 (0.0448
0.3229) 0.1430 (0.0467 0.3267)
Microcebus_murinus   0.1916 (0.0639 0.3333) 0.1568 (0.0534 0.3407) 0.1656 (0.0572 0.3455) 0.1719
(0.0667 0.3880) 0.1667 (0.0615 0.3691) 0.1692 (0.0587 0.3468) 0.1561 (0.0549 0.3515) 0.1398 (0.0539
0.3851) 0.1458 (0.0567 0.3891) 0.1536 (0.0581 0.3783)
Daubentonia_madagascariensis 0.1723 (0.0506 0.2938) 0.1328 (0.0394 0.2968) 0.1463 (0.0441 0.3013)
0.1601 (0.0525 0.3279) 0.1535 (0.0464 0.3025) 0.1536 (0.0446 0.2902) 0.1386 (0.0408 0.2945) 0.1258
(0.0398 0.3167) 0.1313 (0.0427 0.3247) 0.1513 (0.0473 0.3126) 0.2022 (0.0288 0.1425)
Otolemur_garnettii   0.1351 (0.0619 0.4579) 0.1163 (0.0524 0.4509) 0.1189 (0.0543 0.4566) 0.1462
(0.0652 0.4459) 0.1262 (0.0601 0.4759) 0.1256 (0.0572 0.4552) 0.1227 (0.0553 0.4505) 0.1057 (0.0481
0.4554) 0.1097 (0.0510 0.4649) 0.1315 (0.0609 0.4633) 0.1247 (0.0380 0.3045) 0.1087 (0.0334 0.3073)


TREE #  1:  ((((((1, 2), 3), 4), ((5, 6), 7)), (8, 9)), 10, ((11, 12), 13));   MP score: 518
check convergence..
lnL(ntime: 23  np: 26):  -4789.177593      +0.000000
  14..15   15..16   16..17   17..18   18..19   19..1    19..2    18..3    17..4    16..20   20..21
21..5    21..6    20..7    15..22   22..8    22..9    14..10   14..23   23..24   24..11   24..12
23..13
 0.100394 0.022672 0.033449 0.021585 0.000004 0.028747 0.002051 0.018433 0.066172 0.041487 0.009839
0.025877 0.008169 0.020146 0.035153 0.027419 0.041713 0.186187 0.094946 0.029616 0.119448 0.059107
0.217052 4.029470 0.159836 0.774534

Note: Branch length is defined as number of nucleotide substitutions per codon (not per neucleotide
site).

tree length =   1.20967

((((((1: 0.028747, 2: 0.002051): 0.000004, 3: 0.018433): 0.021585, 4: 0.066172): 0.033449, ((5:
0.025877, 6: 0.008169): 0.009839, 7: 0.020146): 0.041487): 0.022672, (8: 0.027419, 9: 0.041713):
0.035153): 0.100394, 10: 0.186187, ((11: 0.119448, 12: 0.059107): 0.029616, 13: 0.217052): 0.094946);

((((((Homo_sapiens: 0.028747, Pan_troglodytes: 0.002051): 0.000004, Gorilla_gorilla_gorilla: 0.018433):
0.021585, Pongo_abelii: 0.066172): 0.033449, ((Macaca_mulatta: 0.025877, Papio_anubis: 0.008169):
0.009839, Chlorocebus_sabaeus: 0.020146): 0.041487): 0.022672, (Saimiri_boliviensis: 0.027419,
Callithrix_jacchus: 0.041713): 0.035153): 0.100394, Tarsius_syrichta: 0.186187, ((Microcebus_murinus:
```

```
0.119448, Daubentonia_madagascariensis: 0.059107): 0.029616, Otolemur_garnettii: 0.217052): 0.094946);

Detailed output identifying parameters

kappa (ts/tv) =  4.02947

w (dN/dS) for branches:  0.15984 0.77453

dN & dS for each branch

 branch          t       N       S   dN/dS      dN      dS  N*dN  S*dS

  14..15     0.100  1090.9   403.1  0.1598  0.0138  0.0866  15.1  34.9
  15..16     0.023  1090.9   403.1  0.1598  0.0031  0.0196   3.4   7.9
  16..17     0.033  1090.9   403.1  0.1598  0.0046  0.0288   5.0  11.6
  17..18     0.022  1090.9   403.1  0.1598  0.0030  0.0186   3.2   7.5
  18..19     0.000  1090.9   403.1  0.1598  0.0000  0.0000   0.0   0.0
  19..1      0.029  1090.9   403.1  0.7745  0.0089  0.0115   9.7   4.6
  19..2      0.002  1090.9   403.1  0.1598  0.0003  0.0018   0.3   0.7
  18..3      0.018  1090.9   403.1  0.7745  0.0057  0.0074   6.2   3.0
  17..4      0.066  1090.9   403.1  0.7745  0.0205  0.0264  22.3  10.6
  16..20     0.041  1090.9   403.1  0.1598  0.0057  0.0358   6.2  14.4
  20..21     0.010  1090.9   403.1  0.1598  0.0014  0.0085   1.5   3.4
  21..5      0.026  1090.9   403.1  0.1598  0.0036  0.0223   3.9   9.0
  21..6      0.008  1090.9   403.1  0.1598  0.0011  0.0070   1.2   2.8
  20..7      0.020  1090.9   403.1  0.1598  0.0028  0.0174   3.0   7.0
  15..22     0.035  1090.9   403.1  0.1598  0.0048  0.0303   5.3  12.2
  22..8      0.027  1090.9   403.1  0.1598  0.0038  0.0236   4.1   9.5
  22..9      0.042  1090.9   403.1  0.1598  0.0057  0.0360   6.3  14.5
  14..10     0.186  1090.9   403.1  0.1598  0.0257  0.1606  28.0  64.7
  14..23     0.095  1090.9   403.1  0.1598  0.0131  0.0819  14.3  33.0
  23..24     0.030  1090.9   403.1  0.1598  0.0041  0.0255   4.5  10.3
  24..11     0.119  1090.9   403.1  0.1598  0.0165  0.1030  18.0  41.5
  24..12     0.059  1090.9   403.1  0.1598  0.0081  0.0510   8.9  20.5
  23..13     0.217  1090.9   403.1  0.1598  0.0299  0.1872  32.6  75.5

tree length for dN:       0.1862
tree length for dS:       0.9907

dS tree:
((((((Homo_sapiens: 0.011471, Pan_troglodytes: 0.001769): 0.000003, Gorilla_gorilla_gorilla: 0.007355):
0.018615, Pongo_abelii: 0.026404): 0.028847, ((Macaca_mulatta: 0.022317, Papio_anubis: 0.007045):
0.008485, Chlorocebus_sabaeus: 0.017374): 0.035779): 0.019552, (Saimiri_boliviensis: 0.023647,
Callithrix_jacchus: 0.035974): 0.030317): 0.086581, Tarsius_syrichta: 0.160570, ((Microcebus_murinus:
0.103014, Daubentonia_madagascariensis: 0.050975): 0.025541, Otolemur_garnettii: 0.187189): 0.081883);
dN tree:
((((((Homo_sapiens: 0.008885, Pan_troglodytes: 0.000283): 0.000001, Gorilla_gorilla_gorilla: 0.005697):
0.002975, Pongo_abelii: 0.020451): 0.004611, ((Macaca_mulatta: 0.003567, Papio_anubis: 0.001126):
0.001356, Chlorocebus_sabaeus: 0.002777): 0.005719): 0.003125, (Saimiri_boliviensis: 0.003780,
Callithrix_jacchus: 0.005750): 0.004846): 0.013839, Tarsius_syrichta: 0.025665, ((Microcebus_murinus:
0.016465, Daubentonia_madagascariensis: 0.008148): 0.004082, Otolemur_garnettii: 0.029920): 0.013088);

w ratios as labels for TreeView:
((((((Homo_sapiens #0.7745 , Pan_troglodytes #0.1598 ) #0.1598 , Gorilla_gorilla_gorilla #0.7745 )
#0.1598 , Pongo_abelii #0.7745 ) #0.1598 , ((Macaca_mulatta #0.1598 , Papio_anubis #0.1598 ) #0.1598 ,
Chlorocebus_sabaeus #0.1598 ) #0.1598 ) #0.1598 , (Saimiri_boliviensis #0.1598 , Callithrix_jacchus
#0.1598 ) #0.1598 ) #0.1598 , Tarsius_syrichta #0.1598 , ((Microcebus_murinus #0.1598 ,
Daubentonia_madagascariensis #0.1598 ) #0.1598 , Otolemur_garnettii #0.1598 ) #0.1598 );
```

Main result file for model F: catarrhines-F.mlc

```
CODONML (in paml version 4.7b, October 2013)  catarrhines.phy
Model: several dN/dS ratios for branches for branches,  omega = 1.000 fixed

Codon frequency model: F3x4
ns =  13  ls = 498

Codon usage in sequences
--------------------------------------------------------------------------------------------------------------------------------------
Phe TTT  13  13  13  14  13  13 | Ser TCT   5   5   5   7   4   4 | Tyr TAT  11  10  11  10   9   8 | Cys TGT   3   4   4   6   5   5
    TTC  15  15  15  13  15  15 |     TCC  11  11  11   9  11  11 |     TAC  16  16  15  15  17  18 |     TGC   8   6   6   7   5   5
Leu TTA   1   1   1   2   2   2 |     TCA   2   2   2   3   2   2 | *** TAA   0   0   0   0   0   0 | *** TGA   0   0   0   0   0   0
    TTG   9   9   9   8   9   9 |     TCG   2   2   2   1   2   2 |     TAG   0   0   0   0   0   0 | Trp TGG   4   4   4   4   4   4
--------------------------------------------------------------------------------------------------------------------------------------
Leu CTT  10   9   9   8   6   6 | Pro CCT  13  14  15  12  10  10 | His CAT   6   6   6   6   6   6 | Arg CGT   4   4   3   3   4   3
    CTC   8   8   8  10   9   9 |     CCC  10  10   9  11  13  14 |     CAC  11  10  10  11  10  10 |     CGC   6   8   8   5   7   8
    CTA   2   2   2   2   2   2 |     CCA   3   3   3   3   2   2 | Gln CAA   5   4   4   4   4   4 |     CGA   3   5   5   5   5   5
    CTG  27  27  27  27  30  30 |     CCG   2   2   2   2   4   3 |     CAG  11  11  12  13  13  13 |     CGG   3   2   2   2   1   1
--------------------------------------------------------------------------------------------------------------------------------------
Ile ATT  10  10  10   9  11  10 | Thr ACT   6   6   6   9   9   9 | Asn AAT  12  11  12  11  14  12 | Ser AGT   2   2   3   2   3   3
    ATC  17  17  18  18  16  17 |     ACC  11  10  10   9   8   9 |     AAC   7   6   6   6   4   6 |     AGC  10  10  10  10   9   9
    ATA   1   1   0   1   1   1 |     ACA   9   9   9   8  11  10 | Lys AAA   3   3   3   3   3   3 | Arg AGA   1   0   0   1   0   0
Met ATG   8   9   9  11   8   8 |     ACG   7   6   6   4   3   3 |     AAG   8   8   8   8   9   8 |     AGG   6   6   6   7   6   6
--------------------------------------------------------------------------------------------------------------------------------------
Val GTT   3   3   3   3   4   5 | Ala GCT  10  10  10  11   9   9 | Asp GAT  17  18  17  17  21  21 | Gly GGT   2   3   3   2   4   4
    GTC  16  15  15  15  15  14 |     GCC   8  10   9   9  12  12 |     GAC  23  24  24  24  20  20 |     GGC  14  14  14  15  13  13
    GTA   2   3   2   3   2   2 |     GCA   2   2   2   2   2   2 | Glu GAA   5   5   5   4   5   4 |     GGA   9   9   9  10   9   9
    GTG  15  14  16  14  15  15 |     GCG   0   0   0   0   0   0 |     GAG  22  22  21  21  20  22 |     GGG   8   9   9   8   8   8
--------------------------------------------------------------------------------------------------------------------------------------


--------------------------------------------------------------------------------------------------------------------------------------
Phe TTT  13  13  12  16  10  12 | Ser TCT   4   3   4   4   4   4 | Tyr TAT   9  12  12  11   5   6 | Cys TGT   4   4   6   7   2   4
    TTC  15  15  15  15  17  14 |     TCC  11  11  11  13  14  14 |     TAC  17  14  14  14  20  19 |     TGC   6   7   5   4  10   8
Leu TTA   2   1   1   0   2   0 |     TCA   2   2   2   2   1   1 | *** TAA   0   0   0   0   0   0 | *** TGA   0   0   0   0   0   0
    TTG   9  11  10   9   5   6 |     TCG   2   2   2   3   2   2 |     TAG   0   0   0   0   0   0 | Trp TGG   4   4   4   4   4   4
--------------------------------------------------------------------------------------------------------------------------------------
Leu CTT   6  10  11   5   4   7 | Pro CCT  10  11  11  12   9   9 | His CAT   6   6   7   8   5   6 | Arg CGT   3   3   2   4   4   2
    CTC   9   9   8  12  12  11 |     CCC  14  13  12  10  15  12 |     CAC  10   9   9   8  12  12 |     CGC   8   7   7   6   9   8
    CTA   3   2   2   4   2   2 |     CCA   2   2   2   4   2   3 | Gln CAA   5   3   4   3   5   3 |     CGA   4   5   5   6   3   5
    CTG  29  25  27  25  30  30 |     CCG   3   2   2   1   3   3 |     CAG  12  14  12  12  12  13 |     CGG   2   1   2   3   3   3
--------------------------------------------------------------------------------------------------------------------------------------
Ile ATT   9  11  10   8   4   5 | Thr ACT   9   9   9   9   6   8 | Asn AAT  12  12  11  11   5   9 | Ser AGT   3   5   5   5   3   3
    ATC  17  17  18  19  22  19 |     ACC   9   7   7  10  14  12 |     AAC   6   5   5   5   9   7 |     AGC   9   7   8   5   8   7
    ATA   1   1   1   4   2   2 |     ACA   9   9  10   5   6   6 | Lys AAA   3   2   2   4   3   2 | Arg AGA   1   0   0   2   2   2
Met ATG   9   9  10  10  11   9 |     ACG   4   5   4   5   6   6 |     AAG   8   7   7  10  10   9 |     AGG   6   7   7   4   3   4
--------------------------------------------------------------------------------------------------------------------------------------
Val GTT   3   7   8   6   4   4 | Ala GCT   9  12  12  10   9   7 | Asp GAT  20  20  20  19  11  17 | Gly GGT   4   4   3   6   3   5
    GTC  16  12   9  10  12  14 |     GCC  11  12  12   9  13  13 |     GAC  21  22  22  24  31  25 |     GGC  14  13  13  14  18  15
    GTA   2   2   2   4   3   2 |     GCA   2   2   2   2   0   3 | Glu GAA   4   5   6  11   5   6 |     GGA   8   8   9   7   6   7
    GTG  15  14  15  13  14  18 |     GCG   0   0   1   0   2   0 |     GAG  21  22  21  14  17  19 |     GGG   9   9   8   8  10  10
--------------------------------------------------------------------------------------------------------------------------------------


------------------------------------------------------
Phe TTT  10 | Ser TCT   3 | Tyr TAT   3 | Cys TGT   5
    TTC  20 |     TCC  12 |     TAC  22 |     TGC   8
Leu TTA   2 |     TCA   2 | *** TAA   0 | *** TGA   0
    TTG   6 |     TCG   1 |     TAG   0 | Trp TGG   4
------------------------------------------------------
Leu CTT   7 | Pro CCT  13 | His CAT   6 | Arg CGT   4
    CTC  10 |     CCC  10 |     CAC  11 |     CGC   7
    CTA   4 |     CCA   2 | Gln CAA   5 |     CGA   3
    CTG  26 |     CCG   2 |     CAG  11 |     CGG   3
------------------------------------------------------
Ile ATT   5 | Thr ACT   7 | Asn AAT   6 | Ser AGT   3
    ATC  20 |     ACC  13 |     AAC  10 |     AGC   7
    ATA   2 |     ACA   6 | Lys AAA   3 | Arg AGA   3
Met ATG  10 |     ACG   4 |     AAG   9 |     AGG   3
------------------------------------------------------
Val GTT   5 | Ala GCT   7 | Asp GAT  11 | Gly GGT   5
    GTC  14 |     GCC  15 |     GAC  28 |     GGC  19
    GTA   4 |     GCA   3 | Glu GAA  11 |     GGA   5
    GTG  14 |     GCG   1 |     GAG  15 |     GGG   8
------------------------------------------------------

Codon position x base (3x4) table for each sequence.

#1: Homo_sapiens
position  1:    T:0.20080    C:0.24900    A:0.23695    G:0.31325
position  2:    T:0.31526    C:0.20281    A:0.31526    G:0.16667
position  3:    T:0.25502    C:0.38353    A:0.09639    G:0.26506
Average         T:0.25703    C:0.27845    A:0.21620    G:0.24833

#2: Pan_troglodytes
position  1:    T:0.19679    C:0.25100    A:0.22892    G:0.32329
position  2:    T:0.31325    C:0.20482    A:0.30924    G:0.17269
position  3:    T:0.25703    C:0.38153    A:0.09839    G:0.26305
Average         T:0.25569    C:0.27912    A:0.21218    G:0.25301
```

```
#3: Gorilla_gorilla_gorilla
position  1:    T:0.19679    C:0.25100    A:0.23293    G:0.31928
position  2:    T:0.31526    C:0.20281    A:0.30924    G:0.17269
position  3:    T:0.26104    C:0.37751    A:0.09438    G:0.26707
Average         T:0.25770    C:0.27711    A:0.21218    G:0.25301

#4: Pongo_abelii
position  1:    T:0.19880    C:0.24900    A:0.23494    G:0.31727
position  2:    T:0.31727    C:0.20080    A:0.30723    G:0.17470
position  3:    T:0.26104    C:0.37550    A:0.10241    G:0.26104
Average         T:0.25904    C:0.27510    A:0.21486    G:0.25100

#5: Macaca_mulatta
position  1:    T:0.19679    C:0.25301    A:0.23092    G:0.31928
position  2:    T:0.31727    C:0.20482    A:0.31124    G:0.16667
position  3:    T:0.26506    C:0.36948    A:0.10040    G:0.26506
Average         T:0.25971    C:0.27577    A:0.21419    G:0.25033

#6: Papio_anubis
position  1:    T:0.19679    C:0.25301    A:0.22892    G:0.32129
position  2:    T:0.31727    C:0.20482    A:0.31124    G:0.16667
position  3:    T:0.25703    C:0.38153    A:0.09639    G:0.26506
Average         T:0.25703    C:0.27979    A:0.21218    G:0.25100

#7: Chlorocebus_sabaeus
position  1:    T:0.19679    C:0.25301    A:0.23092    G:0.31928
position  2:    T:0.31727    C:0.20281    A:0.30924    G:0.17068
position  3:    T:0.24900    C:0.38755    A:0.09639    G:0.26707
Average         T:0.25435    C:0.28112    A:0.21218    G:0.25234

#8: Saimiri_boliviensis
position  1:    T:0.19880    C:0.24498    A:0.22691    G:0.32932
position  2:    T:0.31928    C:0.20482    A:0.30723    G:0.16867
position  3:    T:0.28514    C:0.36145    A:0.08835    G:0.26506
Average         T:0.26774    C:0.27041    A:0.20750    G:0.25435

#9: Callithrix_jacchus
position  1:    T:0.19679    C:0.24699    A:0.22892    G:0.32731
position  2:    T:0.31928    C:0.20683    A:0.30522    G:0.16867
position  3:    T:0.28715    C:0.35141    A:0.09639    G:0.26506
Average         T:0.26774    C:0.26841    A:0.21017    G:0.25368

#10: Tarsius_syrichta
position  1:    T:0.20482    C:0.24699    A:0.23293    G:0.31526
position  2:    T:0.32129    C:0.19880    A:0.30924    G:0.17068
position  3:    T:0.28313    C:0.35743    A:0.11647    G:0.24297
Average         T:0.26975    C:0.26774    A:0.21954    G:0.24297

#11: Microcebus_murinus
position  1:    T:0.19277    C:0.26104    A:0.22892    G:0.31727
position  2:    T:0.30924    C:0.21285    A:0.30120    G:0.17671
position  3:    T:0.17671    C:0.47390    A:0.08434    G:0.26506
Average         T:0.22624    C:0.31593    A:0.20482    G:0.25301

#12: Daubentonia_madagascariensis
position  1:    T:0.18876    C:0.25904    A:0.22088    G:0.33133
position  2:    T:0.31124    C:0.20683    A:0.30723    G:0.17470
position  3:    T:0.21687    C:0.42169    A:0.08835    G:0.27309
Average         T:0.23896    C:0.29585    A:0.20549    G:0.25971

#13: Otolemur_garnettii
position  1:    T:0.19679    C:0.24900    A:0.22289    G:0.33133
position  2:    T:0.31928    C:0.20281    A:0.30321    G:0.17470
position  3:    T:0.20080    C:0.45382    A:0.11044    G:0.23494
Average         T:0.23896    C:0.30187    A:0.21218    G:0.24699

Sums of codon usage counts
------------------------------------------------------------------------------
Phe F TTT     165 | Ser S TCT      56 | Tyr Y TAT     117 | Cys C TGT      59
      TTC     199 |       TCC     150 |       TAC     217 |       TGC      85
Leu L TTA      17 |       TCA      25 | *** * TAA       0 | *** * TGA       0
      TTG     109 |       TCG      25 |       TAG       0 | Trp W TGG      52
------------------------------------------------------------------------------
Leu L CTT      98 | Pro P CCT     149 | His H CAT      80 | Arg R CGT      43
      CTC     123 |       CCC     153 |       CAC     133 |       CGC      94
      CTA      31 |       CCA      33 | Gln Q CAA      53 |       CGA      59
```

```
      CTG     360 |       CCG      31 |       CAG     159 |       CGG      28
------------------------------------------------------------------------------
Ile I ATT     112 | Thr T ACT     102 | Asn N AAT     138 | Ser S AGT      42
      ATC     235 |       ACC     129 |       AAC      82 |       AGC     109
      ATA      18 |       ACA     107 | Lys K AAA      37 | Arg R AGA      12
Met M ATG     121 |       ACG      63 |       AAG     109 |       AGG      71
------------------------------------------------------------------------------
Val V GTT      58 | Ala A GCT     125 | Asp D GAT     229 | Gly G GGT      48
      GTC     177 |       GCC     145 |       GAC     308 |       GGC     189
      GTA      33 |       GCA      26 | Glu E GAA      76 |       GGA     105
      GTG     192 |       GCG       4 |       GAG     257 |       GGG     112
------------------------------------------------------------------------------


Codon position x base (3x4) table, overall

position  1:    T:0.19710    C:0.25131    A:0.22969    G:0.32190
position  2:    T:0.31634    C:0.20436    A:0.30816    G:0.17115
position  3:    T:0.25039    C:0.39049    A:0.09762    G:0.26151
Average         T:0.25461    C:0.28205    A:0.21182    G:0.25152


Nei & Gojobori 1986. dN/dS (dN, dS)
(Note: This matrix is not used in later ML. analysis.
Use runmode = -2 for ML pairwise comparison.)

Homo_sapiens
Pan_troglodytes      1.2604 (0.0106 0.0084)
Gorilla_gorilla_gorilla 1.3389 (0.0151 0.0113) 0.7351 (0.0062 0.0084)
Pongo_abelii         0.4378 (0.0296 0.0676) 0.2910 (0.0187 0.0643) 0.3714 (0.0250 0.0674)
Macaca_mulatta       0.2054 (0.0269 0.1309) 0.1261 (0.0160 0.1271) 0.1712 (0.0224 0.1306) 0.2017
(0.0278 0.1377)
Papio_anubis         0.2052 (0.0242 0.1177) 0.1169 (0.0133 0.1141) 0.1672 (0.0196 0.1174) 0.1773
(0.0232 0.1311) 0.2434 (0.0062 0.0255)
Chlorocebus_sabaeus  0.2404 (0.0260 0.1080) 0.1448 (0.0151 0.1045) 0.1989 (0.0214 0.1078) 0.2216
(0.0269 0.1212) 0.2126 (0.0098 0.0459) 0.2498 (0.0071 0.0284)
Saimiri_boliviensis  0.1999 (0.0273 0.1366) 0.1180 (0.0165 0.1395) 0.1466 (0.0210 0.1430) 0.1860
(0.0296 0.1590) 0.1788 (0.0237 0.1326) 0.1758 (0.0210 0.1193) 0.1607 (0.0205 0.1277)
Callithrix_jacchus   0.1917 (0.0301 0.1568) 0.1211 (0.0187 0.1545) 0.1469 (0.0232 0.1582) 0.1798
(0.0323 0.1798) 0.1694 (0.0264 0.1560) 0.1665 (0.0237 0.1424) 0.1612 (0.0232 0.1442) 0.1129 (0.0080
0.0705)
Tarsius_syrichta     0.1616 (0.0555 0.3433) 0.1311 (0.0442 0.3376) 0.1428 (0.0489 0.3424) 0.1715
(0.0578 0.3372) 0.1490 (0.0524 0.3518) 0.1446 (0.0496 0.3429) 0.1330 (0.0486 0.3656) 0.1388 (0.0448
0.3229) 0.1430 (0.0467 0.3267)
Microcebus_murinus   0.1916 (0.0639 0.3333) 0.1568 (0.0534 0.3407) 0.1656 (0.0572 0.3455) 0.1719
(0.0667 0.3880) 0.1667 (0.0615 0.3691) 0.1692 (0.0587 0.3468) 0.1561 (0.0549 0.3515) 0.1398 (0.0539
0.3851) 0.1458 (0.0567 0.3891) 0.1536 (0.0581 0.3783)
Daubentonia_madagascariensis 0.1723 (0.0506 0.2938) 0.1328 (0.0394 0.2968) 0.1463 (0.0441 0.3013)
0.1601 (0.0525 0.3279) 0.1535 (0.0464 0.3025) 0.1536 (0.0446 0.2902) 0.1386 (0.0408 0.2945) 0.1258
(0.0398 0.3167) 0.1313 (0.0427 0.3247) 0.1513 (0.0473 0.3126) 0.2022 (0.0288 0.1425)
Otolemur_garnettii   0.1351 (0.0619 0.4579) 0.1163 (0.0524 0.4509) 0.1189 (0.0543 0.4566) 0.1462
(0.0652 0.4459) 0.1262 (0.0601 0.4759) 0.1256 (0.0572 0.4552) 0.1227 (0.0553 0.4505) 0.1057 (0.0481
0.4554) 0.1097 (0.0510 0.4649) 0.1315 (0.0609 0.4633) 0.1247 (0.0380 0.3045) 0.1087 (0.0334 0.3073)


TREE #  1:  ((((((1, 2), 3), 4), ((5, 6), 7)), (8, 9)), 10, ((11, 12), 13));   MP score: 518
lnL(ntime: 23  np: 25):  -4789.535826      +0.000000
  14..15   15..16   16..17   17..18   18..19   19..1    19..2    18..3    17..4    16..20   20..21
21..5    21..6    20..7    15..22   22..8    22..9    14..10   14..23   23..24   24..11   24..12
23..13
 0.100364 0.022743 0.033543 0.021834 0.000004 0.028770 0.002049 0.018450 0.065845 0.041442 0.009838
0.025877 0.008172 0.020146 0.035173 0.027386 0.041760 0.186179 0.094976 0.029682 0.119468 0.059114
0.217052 4.054155 0.159937

Note: Branch length is defined as number of nucleotide substitutions per codon (not per neucleotide
site).

tree length =   1.20987

((((((1: 0.028770, 2: 0.002049): 0.000004, 3: 0.018450): 0.021834, 4: 0.065845): 0.033543, ((5:
0.025877, 6: 0.008172): 0.009838, 7: 0.020146): 0.041442): 0.022743, (8: 0.027386, 9: 0.041760):
0.035173): 0.100364, 10: 0.186179, ((11: 0.119468, 12: 0.059114): 0.029682, 13: 0.217052): 0.094976);

((((((Homo_sapiens: 0.028770, Pan_troglodytes: 0.002049): 0.000004, Gorilla_gorilla_gorilla: 0.018450):
0.021834, Pongo_abelii: 0.065845): 0.033543, ((Macaca_mulatta: 0.025877, Papio_anubis: 0.008172):
0.009838, Chlorocebus_sabaeus: 0.020146): 0.041442): 0.022743, (Saimiri_boliviensis: 0.027386,
Callithrix_jacchus: 0.041760): 0.035173): 0.100364, Tarsius_syrichta: 0.186179, ((Microcebus_murinus:
```

0.119468, Daubentonia_madagascariensis: 0.059114): 0.029682, Otolemur_garnettii: 0.217052): 0.094976);

Detailed output identifying parameters

kappa (ts/tv) = 4.05416

w (dN/dS) for branches: 0.15994 1.00000

dN & dS for each branch

branch	t	N	S	dN/dS	dN	dS	N*dN	S*dS
14..15	0.100	1090.6	403.4	0.1599	0.0138	0.0865	15.1	34.9
15..16	0.023	1090.6	403.4	0.1599	0.0031	0.0196	3.4	7.9
16..17	0.034	1090.6	403.4	0.1599	0.0046	0.0289	5.0	11.7
17..18	0.022	1090.6	403.4	0.1599	0.0030	0.0188	3.3	7.6
18..19	0.000	1090.6	403.4	0.1599	0.0000	0.0000	0.0	0.0
19..1	0.029	1090.6	403.4	1.0000	0.0096	0.0096	10.5	3.9
19..2	0.002	1090.6	403.4	0.1599	0.0003	0.0018	0.3	0.7
18..3	0.018	1090.6	403.4	1.0000	0.0062	0.0062	6.7	2.5
17..4	0.066	1090.6	403.4	1.0000	0.0219	0.0219	23.9	8.9
16..20	0.041	1090.6	403.4	0.1599	0.0057	0.0357	6.2	14.4
20..21	0.010	1090.6	403.4	0.1599	0.0014	0.0085	1.5	3.4
21..5	0.026	1090.6	403.4	0.1599	0.0036	0.0223	3.9	9.0
21..6	0.008	1090.6	403.4	0.1599	0.0011	0.0070	1.2	2.8
20..7	0.020	1090.6	403.4	0.1599	0.0028	0.0174	3.0	7.0
15..22	0.035	1090.6	403.4	0.1599	0.0048	0.0303	5.3	12.2
22..8	0.027	1090.6	403.4	0.1599	0.0038	0.0236	4.1	9.5
22..9	0.042	1090.6	403.4	0.1599	0.0058	0.0360	6.3	14.5
14..10	0.186	1090.6	403.4	0.1599	0.0257	0.1605	28.0	64.7
14..23	0.095	1090.6	403.4	0.1599	0.0131	0.0819	14.3	33.0
23..24	0.030	1090.6	403.4	0.1599	0.0041	0.0256	4.5	10.3
24..11	0.119	1090.6	403.4	0.1599	0.0165	0.1030	18.0	41.5
24..12	0.059	1090.6	403.4	0.1599	0.0081	0.0509	8.9	20.6
23..13	0.217	1090.6	403.4	0.1599	0.0299	0.1871	32.6	75.5

tree length for dN: 0.1889
tree length for dS: 0.9829

dS tree:
((((((Homo_sapiens: 0.009590, Pan_troglodytes: 0.001766): 0.000003, Gorilla_gorilla_gorilla: 0.006150): 0.018816, Pongo_abelii: 0.021948): 0.028908, ((Macaca_mulatta: 0.022301, Papio_anubis: 0.007043): 0.008478, Chlorocebus_sabaeus: 0.017362): 0.035715): 0.019600, (Saimiri_boliviensis: 0.023601, Callithrix_jacchus: 0.035990): 0.030313): 0.086495, Tarsius_syrichta: 0.160452, ((Microcebus_murinus: 0.102959, Daubentonia_madagascariensis: 0.050946): 0.025580, Otolemur_garnettii: 0.187058): 0.081851);
dN tree:
((((((Homo_sapiens: 0.009590, Pan_troglodytes: 0.000282): 0.000001, Gorilla_gorilla_gorilla: 0.006150): 0.003009, Pongo_abelii: 0.021948): 0.004623, ((Macaca_mulatta: 0.003567, Papio_anubis: 0.001126): 0.001356, Chlorocebus_sabaeus: 0.002777): 0.005712): 0.003135, (Saimiri_boliviensis: 0.003775, Callithrix_jacchus: 0.005756): 0.004848): 0.013834, Tarsius_syrichta: 0.025662, ((Microcebus_murinus: 0.016467, Daubentonia_madagascariensis: 0.008148): 0.004091, Otolemur_garnettii: 0.029918): 0.013091);

w ratios as labels for TreeView:
((((((Homo_sapiens #1.0000 , Pan_troglodytes #0.1599) #0.1599 , Gorilla_gorilla_gorilla #1.0000) #0.1599 , Pongo_abelii #1.0000) #0.1599 , ((Macaca_mulatta #0.1599 , Papio_anubis #0.1599) #0.1599 , Chlorocebus_sabaeus #0.1599) #0.1599) #0.1599 , (Saimiri_boliviensis #0.1599 , Callithrix_jacchus #0.1599) #0.1599) #0.1599 , Tarsius_syrichta #0.1599 , ((Microcebus_murinus #0.1599 , Daubentonia_madagascariensis #0.1599) #0.1599 , Otolemur_garnettii #0.1599) #0.1599);

Main result file for model G: catarrhines-G.mlc

```
CODONML (in paml version 4.7b, October 2013)  catarrhines.phy
Model: several dN/dS ratios for branches for branches,
Codon frequency model: F3x4
ns =  13  ls = 498

Codon usage in sequences
------------------------------------------------------------------------------------------------------
Phe TTT  13  13  13  14  13  13 | Ser TCT   5   5   5   7   4   4 | Tyr TAT  11  10  11  10   9   8 | Cys TGT   3   4   4   6   5   5
    TTC  15  15  15  13  15  15 |     TCC  11  11  11   9  11  11 |     TAC  16  16  15  15  17  18 |     TGC   8   6   6   7   5   5
Leu TTA   1   1   1   2   2   2 |     TCA   2   2   2   3   2   2 | *** TAA   0   0   0   0   0   0 | *** TGA   0   0   0   0   0   0
    TTG   9   9   9   8   9   9 |     TCG   2   2   2   1   2   2 |     TAG   0   0   0   0   0   0 | Trp TGG   4   4   4   4   4   4
------------------------------------------------------------------------------------------------------
Leu CTT  10   9   9   8   6   6 | Pro CCT  13  14  15  12  10  10 | His CAT   6   6   6   6   6   6 | Arg CGT   4   4   3   3   4   3
    CTC   8   8   8  10   9   9 |     CCC  10  10   9  11  13  14 |     CAC  11  10  10  11  10  10 |     CGC   6   8   8   5   7   8
    CTA   2   2   2   2   2   2 |     CCA   3   3   3   3   2   2 | Gln CAA   5   4   4   4   4   4 |     CGA   3   5   5   5   5   5
    CTG  27  27  27  27  30  30 |     CCG   2   2   2   2   4   3 |     CAG  11  11  12  13  13  13 |     CGG   3   2   2   2   1   1
------------------------------------------------------------------------------------------------------
Ile ATT  10  10  10   9  11  10 | Thr ACT   6   6   6   9   9   9 | Asn AAT  12  11  12  11  14  12 | Ser AGT   2   2   3   2   3   3
    ATC  17  17  18  18  16  17 |     ACC  11  10  10   9   8   9 |     AAC   7   6   6   6   4   6 |     AGC  10  10  10  10   9   9
    ATA   1   1   0   1   1   1 |     ACA   9   9   9   8  11  10 | Lys AAA   3   3   3   3   3   3 | Arg AGA   1   0   0   1   0   0
Met ATG   8   9   9  11   8   8 |     ACG   7   6   6   4   3   3 |     AAG   8   8   8   8   9   8 |     AGG   6   6   6   7   6   6
------------------------------------------------------------------------------------------------------
Val GTT   3   3   3   3   4   5 | Ala GCT  10  10  10  11   9   9 | Asp GAT  17  18  17  17  21  21 | Gly GGT   2   3   3   2   4   4
    GTC  16  15  15  15  15  14 |     GCC   8  10   9   9  12  12 |     GAC  23  24  24  24  20  20 |     GGC  14  14  14  15  13  13
    GTA   2   3   2   3   2   2 |     GCA   2   2   2   2   2   2 | Glu GAA   5   5   5   4   5   4 |     GGA   9   9   9  10   9   9
    GTG  15  14  16  14  15  15 |     GCG   0   0   0   0   0   0 |     GAG  22  22  21  21  20  22 |     GGG   8   9   9   8   8   8
------------------------------------------------------------------------------------------------------


------------------------------------------------------------------------------------------------------
Phe TTT  13  13  12  16  10  12 | Ser TCT   4   3   4   4   4   4 | Tyr TAT   9  12  12  11   5   6 | Cys TGT   4   4   6   7   2   4
    TTC  15  15  15  15  17  14 |     TCC  11  11  11  13  14  14 |     TAC  17  14  14  14  20  19 |     TGC   6   7   5   4  10   8
Leu TTA   2   1   1   0   2   0 |     TCA   2   2   2   2   1   1 | *** TAA   0   0   0   0   0   0 | *** TGA   0   0   0   0   0   0
    TTG   9  11  10   9   5   6 |     TCG   2   2   2   3   2   2 |     TAG   0   0   0   0   0   0 | Trp TGG   4   4   4   4   4   4
------------------------------------------------------------------------------------------------------
Leu CTT   6  10  11   5   4   7 | Pro CCT  10  11  11  12   9   9 | His CAT   6   6   7   8   5   6 | Arg CGT   3   3   2   4   4   2
    CTC   9   9   8  12  12  11 |     CCC  14  13  12  10  15  12 |     CAC  10   9   9   8  12  12 |     CGC   8   7   7   6   9   8
    CTA   3   2   2   4   2   2 |     CCA   2   2   2   4   2   3 | Gln CAA   5   3   4   3   5   3 |     CGA   4   5   5   6   3   5
    CTG  29  25  27  25  30  30 |     CCG   3   2   2   1   3   3 |     CAG  12  14  12  12  12  13 |     CGG   2   1   2   3   3   3
------------------------------------------------------------------------------------------------------
Ile ATT   9  11  10   8   4   5 | Thr ACT   9   9   9   9   6   8 | Asn AAT  12  12  11  11   5   9 | Ser AGT   3   5   5   5   3   3
    ATC  17  17  18  19  22  19 |     ACC   9   7   7  10  14  12 |     AAC   6   5   5   5   9   7 |     AGC   9   7   8   5   8   7
    ATA   1   1   1   4   2   2 |     ACA   9   9  10   5   6   6 | Lys AAA   3   2   2   4   3   2 | Arg AGA   1   0   0   2   2   2
Met ATG   9   9  10  10  11   9 |     ACG   4   5   4   5   6   6 |     AAG   8   7   7  10  10   9 |     AGG   6   7   7   4   3   4
------------------------------------------------------------------------------------------------------
Val GTT   3   7   8   6   4   4 | Ala GCT   9  12  12  10   9   7 | Asp GAT  20  20  20  19  11  17 | Gly GGT   4   4   3   6   3   5
    GTC  16  12   9  10  12  14 |     GCC  11  12  12   9  13  13 |     GAC  21  22  22  24  31  25 |     GGC  14  13  13  14  18  15
    GTA   2   2   2   4   3   2 |     GCA   2   2   2   2   0   3 | Glu GAA   4   5   6  11   5   6 |     GGA   8   8   9   7   6   7
    GTG  15  14  15  13  14  18 |     GCG   0   0   1   0   2   0 |     GAG  21  22  21  14  17  19 |     GGG   9   9   8   8  10  10
------------------------------------------------------------------------------------------------------


------------------------------------------------------
Phe TTT  10 | Ser TCT   3 | Tyr TAT   3 | Cys TGT   5
    TTC  20 |     TCC  12 |     TAC  22 |     TGC   8
Leu TTA   2 |     TCA   2 | *** TAA   0 | *** TGA   0
    TTG   6 |     TCG   1 |     TAG   0 | Trp TGG   4
------------------------------------------------------
Leu CTT   7 | Pro CCT  13 | His CAT   6 | Arg CGT   4
    CTC  10 |     CCC  10 |     CAC  11 |     CGC   7
    CTA   4 |     CCA   2 | Gln CAA   5 |     CGA   3
    CTG  26 |     CCG   2 |     CAG  11 |     CGG   3
------------------------------------------------------
Ile ATT   5 | Thr ACT   7 | Asn AAT   6 | Ser AGT   3
    ATC  20 |     ACC  13 |     AAC  10 |     AGC   7
    ATA   2 |     ACA   6 | Lys AAA   3 | Arg AGA   3
Met ATG  10 |     ACG   4 |     AAG   9 |     AGG   3
------------------------------------------------------
Val GTT   5 | Ala GCT   7 | Asp GAT  11 | Gly GGT   5
    GTC  14 |     GCC  15 |     GAC  28 |     GGC  19
    GTA   4 |     GCA   3 | Glu GAA  11 |     GGA   5
    GTG  14 |     GCG   1 |     GAG  15 |     GGG   8
------------------------------------------------------

Codon position x base (3x4) table for each sequence.

#1: Homo_sapiens
position  1:    T:0.20080    C:0.24900    A:0.23695    G:0.31325
position  2:    T:0.31526    C:0.20281    A:0.31526    G:0.16667
position  3:    T:0.25502    C:0.38353    A:0.09639    G:0.26506
Average         T:0.25703    C:0.27845    A:0.21620    G:0.24833

#2: Pan_troglodytes
position  1:    T:0.19679    C:0.25100    A:0.22892    G:0.32329
position  2:    T:0.31325    C:0.20482    A:0.30924    G:0.17269
position  3:    T:0.25703    C:0.38153    A:0.09839    G:0.26305
Average         T:0.25569    C:0.27912    A:0.21218    G:0.25301
```

```
#3: Gorilla_gorilla_gorilla
position  1:    T:0.19679    C:0.25100    A:0.23293    G:0.31928
position  2:    T:0.31526    C:0.20281    A:0.30924    G:0.17269
position  3:    T:0.26104    C:0.37751    A:0.09438    G:0.26707
Average         T:0.25770    C:0.27711    A:0.21218    G:0.25301

#4: Pongo_abelii
position  1:    T:0.19880    C:0.24900    A:0.23494    G:0.31727
position  2:    T:0.31727    C:0.20080    A:0.30723    G:0.17470
position  3:    T:0.26104    C:0.37550    A:0.10241    G:0.26104
Average         T:0.25904    C:0.27510    A:0.21486    G:0.25100

#5: Macaca_mulatta
position  1:    T:0.19679    C:0.25301    A:0.23092    G:0.31928
position  2:    T:0.31727    C:0.20482    A:0.31124    G:0.16667
position  3:    T:0.26506    C:0.36948    A:0.10040    G:0.26506
Average         T:0.25971    C:0.27577    A:0.21419    G:0.25033

#6: Papio_anubis
position  1:    T:0.19679    C:0.25301    A:0.22892    G:0.32129
position  2:    T:0.31727    C:0.20482    A:0.31124    G:0.16667
position  3:    T:0.25703    C:0.38153    A:0.09639    G:0.26506
Average         T:0.25703    C:0.27979    A:0.21218    G:0.25100

#7: Chlorocebus_sabaeus
position  1:    T:0.19679    C:0.25301    A:0.23092    G:0.31928
position  2:    T:0.31727    C:0.20281    A:0.30924    G:0.17068
position  3:    T:0.24900    C:0.38755    A:0.09639    G:0.26707
Average         T:0.25435    C:0.28112    A:0.21218    G:0.25234

#8: Saimiri_boliviensis
position  1:    T:0.19880    C:0.24498    A:0.22691    G:0.32932
position  2:    T:0.31928    C:0.20482    A:0.30723    G:0.16867
position  3:    T:0.28514    C:0.36145    A:0.08835    G:0.26506
Average         T:0.26774    C:0.27041    A:0.20750    G:0.25435

#9: Callithrix_jacchus
position  1:    T:0.19679    C:0.24699    A:0.22892    G:0.32731
position  2:    T:0.31928    C:0.20683    A:0.30522    G:0.16867
position  3:    T:0.28715    C:0.35141    A:0.09639    G:0.26506
Average         T:0.26774    C:0.26841    A:0.21017    G:0.25368

#10: Tarsius_syrichta
position  1:    T:0.20482    C:0.24699    A:0.23293    G:0.31526
position  2:    T:0.32129    C:0.19880    A:0.30924    G:0.17068
position  3:    T:0.28313    C:0.35743    A:0.11647    G:0.24297
Average         T:0.26975    C:0.26774    A:0.21954    G:0.24297

#11: Microcebus_murinus
position  1:    T:0.19277    C:0.26104    A:0.22892    G:0.31727
position  2:    T:0.30924    C:0.21285    A:0.30120    G:0.17671
position  3:    T:0.17671    C:0.47390    A:0.08434    G:0.26506
Average         T:0.22624    C:0.31593    A:0.20482    G:0.25301

#12: Daubentonia_madagascariensis
position  1:    T:0.18876    C:0.25904    A:0.22088    G:0.33133
position  2:    T:0.31124    C:0.20683    A:0.30723    G:0.17470
position  3:    T:0.21687    C:0.42169    A:0.08835    G:0.27309
Average         T:0.23896    C:0.29585    A:0.20549    G:0.25971

#13: Otolemur_garnettii
position  1:    T:0.19679    C:0.24900    A:0.22289    G:0.33133
position  2:    T:0.31928    C:0.20281    A:0.30321    G:0.17470
position  3:    T:0.20080    C:0.45382    A:0.11044    G:0.23494
Average         T:0.23896    C:0.30187    A:0.21218    G:0.24699

Sums of codon usage counts
------------------------------------------------------------------------------
Phe F TTT     165 | Ser S TCT      56 | Tyr Y TAT     117 | Cys C TGT      59
      TTC     199 |       TCC     150 |       TAC     217 |       TGC      85
Leu L TTA      17 |       TCA      25 | *** * TAA       0 | *** * TGA       0
      TTG     109 |       TCG      25 |       TAG       0 | Trp W TGG      52
------------------------------------------------------------------------------
Leu L CTT      98 | Pro P CCT     149 | His H CAT      80 | Arg R CGT      43
      CTC     123 |       CCC     153 |       CAC     133 |       CGC      94
      CTA      31 |       CCA      33 | Gln Q CAA      53 |       CGA      59
      CTG     360 |       CCG      31 |       CAG     159 |       CGG      28
```

```
------------------------------------------------------------------------------
Ile I ATT     112 | Thr T ACT     102 | Asn N AAT     138 | Ser S AGT      42
      ATC     235 |       ACC     129 |       AAC      82 |       AGC     109
      ATA      18 |       ACA     107 | Lys K AAA      37 | Arg R AGA      12
Met M ATG     121 |       ACG      63 |       AAG     109 |       AGG      71
------------------------------------------------------------------------------
Val V GTT      58 | Ala A GCT     125 | Asp D GAT     229 | Gly G GGT      48
      GTC     177 |       GCC     145 |       GAC     308 |       GGC     189
      GTA      33 |       GCA      26 | Glu E GAA      76 |       GGA     105
      GTG     192 |       GCG       4 |       GAG     257 |       GGG     112
------------------------------------------------------------------------------
```

```
Codon position x base (3x4) table, overall

position  1:    T:0.19710    C:0.25131    A:0.22969    G:0.32190
position  2:    T:0.31634    C:0.20436    A:0.30816    G:0.17115
position  3:    T:0.25039    C:0.39049    A:0.09762    G:0.26151
Average         T:0.25461    C:0.28205    A:0.21182    G:0.25152
```

```
Nei & Gojobori 1986. dN/dS (dN, dS)
(Note: This matrix is not used in later ML. analysis.
Use runmode = -2 for ML pairwise comparison.)

Homo_sapiens
Pan_troglodytes      1.2604 (0.0106 0.0084)
Gorilla_gorilla_gorilla 1.3389 (0.0151 0.0113) 0.7351 (0.0062 0.0084)
Pongo_abelii         0.4378 (0.0296 0.0676) 0.2910 (0.0187 0.0643) 0.3714 (0.0250 0.0674)
Macaca_mulatta       0.2054 (0.0269 0.1309) 0.1261 (0.0160 0.1271) 0.1712 (0.0224 0.1306) 0.2017
(0.0278 0.1377)
Papio_anubis         0.2052 (0.0242 0.1177) 0.1169 (0.0133 0.1141) 0.1672 (0.0196 0.1174) 0.1773
(0.0232 0.1311) 0.2434 (0.0062 0.0255)
Chlorocebus_sabaeus  0.2404 (0.0260 0.1080) 0.1448 (0.0151 0.1045) 0.1989 (0.0214 0.1078) 0.2216
(0.0269 0.1212) 0.2126 (0.0098 0.0459) 0.2498 (0.0071 0.0284)
Saimiri_boliviensis  0.1999 (0.0273 0.1366) 0.1180 (0.0165 0.1395) 0.1466 (0.0210 0.1430) 0.1860
(0.0296 0.1590) 0.1788 (0.0237 0.1326) 0.1758 (0.0210 0.1193) 0.1607 (0.0205 0.1277)
Callithrix_jacchus   0.1917 (0.0301 0.1568) 0.1211 (0.0187 0.1545) 0.1469 (0.0232 0.1582) 0.1798
(0.0323 0.1798) 0.1694 (0.0264 0.1560) 0.1665 (0.0237 0.1424) 0.1612 (0.0232 0.1442) 0.1129 (0.0080
0.0705)
Tarsius_syrichta     0.1616 (0.0555 0.3433) 0.1311 (0.0442 0.3376) 0.1428 (0.0489 0.3424) 0.1715
(0.0578 0.3372) 0.1490 (0.0524 0.3518) 0.1446 (0.0496 0.3429) 0.1330 (0.0486 0.3656) 0.1388 (0.0448
0.3229) 0.1430 (0.0467 0.3267)
Microcebus_murinus   0.1916 (0.0639 0.3333) 0.1568 (0.0534 0.3407) 0.1656 (0.0572 0.3455) 0.1719
(0.0667 0.3880) 0.1667 (0.0615 0.3691) 0.1692 (0.0587 0.3468) 0.1561 (0.0549 0.3515) 0.1398 (0.0539
0.3851) 0.1458 (0.0567 0.3891) 0.1536 (0.0581 0.3783)
Daubentonia_madagascariensis 0.1723 (0.0506 0.2938) 0.1328 (0.0394 0.2968) 0.1463 (0.0441 0.3013)
0.1601 (0.0525 0.3279) 0.1535 (0.0464 0.3025) 0.1536 (0.0446 0.2902) 0.1386 (0.0408 0.2945) 0.1258
(0.0398 0.3167) 0.1313 (0.0427 0.3247) 0.1513 (0.0473 0.3126) 0.2022 (0.0288 0.1425)
Otolemur_garnettii   0.1351 (0.0619 0.4579) 0.1163 (0.0524 0.4509) 0.1189 (0.0543 0.4566) 0.1462
(0.0652 0.4459) 0.1262 (0.0601 0.4759) 0.1256 (0.0572 0.4552) 0.1227 (0.0553 0.4505) 0.1057 (0.0481
0.4554) 0.1097 (0.0510 0.4649) 0.1315 (0.0609 0.4633) 0.1247 (0.0380 0.3045) 0.1087 (0.0334 0.3073)
```

```
TREE #  1:  ((((((1, 2), 3), 4), ((5, 6), 7)), (8, 9)), 10, ((11, 12), 13));   MP score: 518
check convergence..
lnL(ntime: 23  np: 26):  -4796.874289      +0.000000
  14..15   15..16   16..17   17..18   18..19   19..1    19..2    18..3    17..4    16..20   20..21
21..5    21..6    20..7    15..22   22..8    22..9    14..10   14..23   23..24   24..11   24..12
23..13
 0.100172 0.022633 0.032035 0.020856 0.000004 0.028636 0.002141 0.018316 0.067340 0.042311 0.010495
0.025878 0.008137 0.019514 0.035570 0.027641 0.041419 0.186011 0.094750 0.029462 0.119263 0.059044
0.216677 4.023875 0.164307 0.398342

Note: Branch length is defined as number of nucleotide substitutions per codon (not per neucleotide
site).

tree length =   1.20830

((((((1: 0.028636, 2: 0.002141): 0.000004, 3: 0.018316): 0.020856, 4: 0.067340): 0.032035, ((5:
0.025878, 6: 0.008137): 0.010495, 7: 0.019514): 0.042311): 0.022633, (8: 0.027641, 9: 0.041419):
0.035570): 0.100172, 10: 0.186011, ((11: 0.119263, 12: 0.059044): 0.029462, 13: 0.216677): 0.094750);

((((((Homo_sapiens: 0.028636, Pan_troglodytes: 0.002141): 0.000004, Gorilla_gorilla_gorilla: 0.018316):
0.020856, Pongo_abelii: 0.067340): 0.032035, ((Macaca_mulatta: 0.025878, Papio_anubis: 0.008137):
0.010495, Chlorocebus_sabaeus: 0.019514): 0.042311): 0.022633, (Saimiri_boliviensis: 0.027641,
Callithrix_jacchus: 0.041419): 0.035570): 0.100172, Tarsius_syrichta: 0.186011, ((Microcebus_murinus:
```

0.119263, Daubentonia_madagascariensis: 0.059044): 0.029462, Otolemur_garnettii: 0.216677): 0.094750);

Detailed output identifying parameters

kappa (ts/tv) = 4.02388

w (dN/dS) for branches: 0.16431 0.39834

dN & dS for each branch

branch	t	N	S	dN/dS	dN	dS	N*dN	S*dS
14..15	0.100	1091.0	403.0	0.1643	0.0141	0.0857	15.4	34.5
15..16	0.023	1091.0	403.0	0.1643	0.0032	0.0194	3.5	7.8
16..17	0.032	1091.0	403.0	0.3983	0.0076	0.0190	8.3	7.7
17..18	0.021	1091.0	403.0	0.3983	0.0049	0.0124	5.4	5.0
18..19	0.000	1091.0	403.0	0.3983	0.0000	0.0000	0.0	0.0
19..1	0.029	1091.0	403.0	0.3983	0.0068	0.0170	7.4	6.9
19..2	0.002	1091.0	403.0	0.3983	0.0005	0.0013	0.6	0.5
18..3	0.018	1091.0	403.0	0.3983	0.0043	0.0109	4.7	4.4
17..4	0.067	1091.0	403.0	0.3983	0.0159	0.0400	17.4	16.1
16..20	0.042	1091.0	403.0	0.1643	0.0059	0.0362	6.5	14.6
20..21	0.010	1091.0	403.0	0.1643	0.0015	0.0090	1.6	3.6
21..5	0.026	1091.0	403.0	0.1643	0.0036	0.0221	4.0	8.9
21..6	0.008	1091.0	403.0	0.1643	0.0011	0.0070	1.2	2.8
20..7	0.020	1091.0	403.0	0.1643	0.0027	0.0167	3.0	6.7
15..22	0.036	1091.0	403.0	0.1643	0.0050	0.0304	5.5	12.3
22..8	0.028	1091.0	403.0	0.1643	0.0039	0.0236	4.2	9.5
22..9	0.041	1091.0	403.0	0.1643	0.0058	0.0354	6.4	14.3
14..10	0.186	1091.0	403.0	0.1643	0.0261	0.1591	28.5	64.1
14..23	0.095	1091.0	403.0	0.1643	0.0133	0.0810	14.5	32.7
23..24	0.029	1091.0	403.0	0.1643	0.0041	0.0252	4.5	10.2
24..11	0.119	1091.0	403.0	0.1643	0.0168	0.1020	18.3	41.1
24..12	0.059	1091.0	403.0	0.1643	0.0083	0.0505	9.1	20.4
23..13	0.217	1091.0	403.0	0.1643	0.0304	0.1853	33.2	74.7

tree length for dN: 0.1861
tree length for dS: 0.9893

dS tree:
((((((Homo_sapiens: 0.017026, Pan_troglodytes: 0.001273): 0.000002, Gorilla_gorilla_gorilla: 0.010890): 0.012400, Pongo_abelii: 0.040038): 0.019047, ((Macaca_mulatta: 0.022133, Papio_anubis: 0.006959): 0.008976, Chlorocebus_sabaeus: 0.016690): 0.036188): 0.019358, (Saimiri_boliviensis: 0.023641, Callithrix_jacchus: 0.035425): 0.030423): 0.085676, Tarsius_syrichta: 0.159093, ((Microcebus_murinus: 0.102004, Daubentonia_madagascariensis: 0.050500): 0.025199, Otolemur_garnettii: 0.185321): 0.081039);
dN tree:
((((((Homo_sapiens: 0.006782, Pan_troglodytes: 0.000507): 0.000001, Gorilla_gorilla_gorilla: 0.004338): 0.004939, Pongo_abelii: 0.015949): 0.007587, ((Macaca_mulatta: 0.003637, Papio_anubis: 0.001143): 0.001475, Chlorocebus_sabaeus: 0.002742): 0.005946): 0.003181, (Saimiri_boliviensis: 0.003884, Callithrix_jacchus: 0.005821): 0.004999): 0.014077, Tarsius_syrichta: 0.026140, ((Microcebus_murinus: 0.016760, Daubentonia_madagascariensis: 0.008297): 0.004140, Otolemur_garnettii: 0.030450): 0.013315);

w ratios as labels for TreeView:
((((((Homo_sapiens #0.3983 , Pan_troglodytes #0.3983) #0.3983 , Gorilla_gorilla_gorilla #0.3983) #0.3983 , Pongo_abelii #0.3983) #0.3983 , ((Macaca_mulatta #0.1643 , Papio_anubis #0.1643) #0.1643 , Chlorocebus_sabaeus #0.1643) #0.1643) #0.1643 , (Saimiri_boliviensis #0.1643 , Callithrix_jacchus #0.1643) #0.1643) #0.1643 , Tarsius_syrichta #0.1643 , ((Microcebus_murinus #0.1643 , Daubentonia_madagascariensis #0.1643) #0.1643 , Otolemur_garnettii #0.1643) #0.1643);

Main result file for model H: catarrhines-H.mlc

```
CODONML (in paml version 4.7b, October 2013)  catarrhines.phy
Model: several dN/dS ratios for branches for branches,  omega = 1.000 fixed

Codon frequency model: F3x4
ns =  13  ls = 498

Codon usage in sequences
--------------------------------------------------------------------------------------------------------------------------------------
Phe TTT  13  13  13  14  13  13 | Ser TCT   5   5   5   7   4   4 | Tyr TAT  11  10  11  10   9   8 | Cys TGT   3   4   4   6   5   5
    TTC  15  15  15  13  15  15 |     TCC  11  11  11   9  11  11 |     TAC  16  16  15  15  17  18 |     TGC   8   6   6   7   5   5
Leu TTA   1   1   1   2   2   2 |     TCA   2   2   2   3   2   2 | *** TAA   0   0   0   0   0   0 | *** TGA   0   0   0   0   0   0
    TTG   9   9   9   8   9   9 |     TCG   2   2   2   1   2   2 |     TAG   0   0   0   0   0   0 | Trp TGG   4   4   4   4   4   4
--------------------------------------------------------------------------------------------------------------------------------------
Leu CTT  10   9   9   8   6   6 | Pro CCT  13  14  15  12  10  10 | His CAT   6   6   6   6   6   6 | Arg CGT   4   4   3   3   4   3
    CTC   8   8   8  10   9   9 |     CCC  10  10   9  11  13  14 |     CAC  11  10  10  11  10  10 |     CGC   6   8   8   5   7   8
    CTA   2   2   2   2   2   2 |     CCA   3   3   3   3   2   2 | Gln CAA   5   4   4   4   4   4 |     CGA   3   5   5   5   5   5
    CTG  27  27  27  27  30  30 |     CCG   2   2   2   2   4   3 |     CAG  11  11  12  13  13  13 |     CGG   3   2   2   2   1   1
--------------------------------------------------------------------------------------------------------------------------------------
Ile ATT  10  10  10   9  11  10 | Thr ACT   6   6   6   9   9   9 | Asn AAT  12  11  12  11  14  12 | Ser AGT   2   2   3   2   3   3
    ATC  17  17  18  18  16  17 |     ACC  11  10  10   9   8   9 |     AAC   7   6   6   6   4   6 |     AGC  10  10  10  10   9   9
    ATA   1   1   0   1   1   1 |     ACA   9   9   9   8  11  10 | Lys AAA   3   3   3   3   3   3 | Arg AGA   1   0   0   1   0   0
Met ATG   8   9   9  11   8   8 |     ACG   7   6   6   4   3   3 |     AAG   8   8   8   8   9   8 |     AGG   6   6   6   7   6   6
--------------------------------------------------------------------------------------------------------------------------------------
Val GTT   3   3   3   3   4   5 | Ala GCT  10  10  10  11   9   9 | Asp GAT  17  18  17  17  21  21 | Gly GGT   2   3   3   2   4   4
    GTC  16  15  15  15  15  14 |     GCC   8  10   9   9  12  12 |     GAC  23  24  24  24  20  20 |     GGC  14  14  14  15  13  13
    GTA   2   3   2   3   2   2 |     GCA   2   2   2   2   2   2 | Glu GAA   5   5   5   4   5   4 |     GGA   9   9   9  10   9   9
    GTG  15  14  16  14  15  15 |     GCG   0   0   0   0   0   0 |     GAG  22  22  21  21  20  22 |     GGG   8   9   9   8   8   8
--------------------------------------------------------------------------------------------------------------------------------------

--------------------------------------------------------------------------------------------------------------------------------------
Phe TTT  13  13  12  16  10  12 | Ser TCT   4   3   4   4   4   4 | Tyr TAT   9  12  12  11   5   6 | Cys TGT   4   4   6   7   2   4
    TTC  15  15  15  15  17  14 |     TCC  11  11  11  13  14  14 |     TAC  17  14  14  14  20  19 |     TGC   6   7   5   4  10   8
Leu TTA   2   1   1   0   2   0 |     TCA   2   2   2   2   1   1 | *** TAA   0   0   0   0   0   0 | *** TGA   0   0   0   0   0   0
    TTG   9  11  10   9   5   6 |     TCG   2   2   2   3   2   2 |     TAG   0   0   0   0   0   0 | Trp TGG   4   4   4   4   4   4
--------------------------------------------------------------------------------------------------------------------------------------
Leu CTT   6  10  11   5   4   7 | Pro CCT  10  11  11  12   9   9 | His CAT   6   6   7   8   5   6 | Arg CGT   3   3   2   4   4   2
    CTC   9   9   8  12  12  11 |     CCC  14  13  12  10  15  12 |     CAC  10   9   9   8  12  12 |     CGC   8   7   7   6   9   8
    CTA   3   2   2   4   2   2 |     CCA   2   2   2   4   2   3 | Gln CAA   5   3   4   3   5   3 |     CGA   4   5   5   6   3   5
    CTG  29  25  27  25  30  30 |     CCG   3   2   2   1   3   3 |     CAG  12  14  12  12  12  13 |     CGG   2   1   2   3   3   3
--------------------------------------------------------------------------------------------------------------------------------------
Ile ATT   9  11  10   8   4   5 | Thr ACT   9   9   9   9   6   8 | Asn AAT  12  12  11  11   5   9 | Ser AGT   3   5   5   5   3   3
    ATC  17  17  18  19  22  19 |     ACC   9   7   7  10  14  12 |     AAC   6   5   5   5   9   7 |     AGC   9   7   8   5   8   7
    ATA   1   1   1   4   2   2 |     ACA   9   9  10   5   6   6 | Lys AAA   3   2   2   4   3   2 | Arg AGA   1   0   0   2   2   2
Met ATG   9   9  10  10  11   9 |     ACG   4   5   4   5   6   6 |     AAG   8   7   7  10  10   9 |     AGG   6   7   7   4   3   4
--------------------------------------------------------------------------------------------------------------------------------------
Val GTT   3   7   8   6   4   4 | Ala GCT   9  12  12  10   9   7 | Asp GAT  20  20  20  19  11  17 | Gly GGT   4   4   3   6   3   5
    GTC  16  12   9  10  12  14 |     GCC  11  12  12   9  13  13 |     GAC  21  22  22  24  31  25 |     GGC  14  13  13  14  18  15
    GTA   2   2   2   4   3   2 |     GCA   2   2   2   2   0   3 | Glu GAA   4   5   6  11   5   6 |     GGA   8   8   9   7   6   7
    GTG  15  14  15  13  14  18 |     GCG   0   0   1   0   2   0 |     GAG  21  22  21  14  17  19 |     GGG   9   9   8   8  10  10
--------------------------------------------------------------------------------------------------------------------------------------


------------------------------------------------------
Phe TTT  10 | Ser TCT   3 | Tyr TAT   3 | Cys TGT   5
    TTC  20 |     TCC  12 |     TAC  22 |     TGC   8
Leu TTA   2 |     TCA   2 | *** TAA   0 | *** TGA   0
    TTG   6 |     TCG   1 |     TAG   0 | Trp TGG   4
------------------------------------------------------
Leu CTT   7 | Pro CCT  13 | His CAT   6 | Arg CGT   4
    CTC  10 |     CCC  10 |     CAC  11 |     CGC   7
    CTA   4 |     CCA   2 | Gln CAA   5 |     CGA   3
    CTG  26 |     CCG   2 |     CAG  11 |     CGG   3
------------------------------------------------------
Ile ATT   5 | Thr ACT   7 | Asn AAT   6 | Ser AGT   3
    ATC  20 |     ACC  13 |     AAC  10 |     AGC   7
    ATA   2 |     ACA   6 | Lys AAA   3 | Arg AGA   3
Met ATG  10 |     ACG   4 |     AAG   9 |     AGG   3
------------------------------------------------------
Val GTT   5 | Ala GCT   7 | Asp GAT  11 | Gly GGT   5
    GTC  14 |     GCC  15 |     GAC  28 |     GGC  19
    GTA   4 |     GCA   3 | Glu GAA  11 |     GGA   5
    GTG  14 |     GCG   1 |     GAG  15 |     GGG   8
------------------------------------------------------

Codon position x base (3x4) table for each sequence.

#1: Homo_sapiens
position  1:    T:0.20080    C:0.24900    A:0.23695    G:0.31325
position  2:    T:0.31526    C:0.20281    A:0.31526    G:0.16667
position  3:    T:0.25502    C:0.38353    A:0.09639    G:0.26506
Average         T:0.25703    C:0.27845    A:0.21620    G:0.24833

#2: Pan_troglodytes
position  1:    T:0.19679    C:0.25100    A:0.22892    G:0.32329
position  2:    T:0.31325    C:0.20482    A:0.30924    G:0.17269
position  3:    T:0.25703    C:0.38153    A:0.09839    G:0.26305
Average         T:0.25569    C:0.27912    A:0.21218    G:0.25301
```

```
#3: Gorilla_gorilla_gorilla
position  1:    T:0.19679    C:0.25100    A:0.23293    G:0.31928
position  2:    T:0.31526    C:0.20281    A:0.30924    G:0.17269
position  3:    T:0.26104    C:0.37751    A:0.09438    G:0.26707
Average         T:0.25770    C:0.27711    A:0.21218    G:0.25301

#4: Pongo_abelii
position  1:    T:0.19880    C:0.24900    A:0.23494    G:0.31727
position  2:    T:0.31727    C:0.20080    A:0.30723    G:0.17470
position  3:    T:0.26104    C:0.37550    A:0.10241    G:0.26104
Average         T:0.25904    C:0.27510    A:0.21486    G:0.25100

#5: Macaca_mulatta
position  1:    T:0.19679    C:0.25301    A:0.23092    G:0.31928
position  2:    T:0.31727    C:0.20482    A:0.31124    G:0.16667
position  3:    T:0.26506    C:0.36948    A:0.10040    G:0.26506
Average         T:0.25971    C:0.27577    A:0.21419    G:0.25033

#6: Papio_anubis
position  1:    T:0.19679    C:0.25301    A:0.22892    G:0.32129
position  2:    T:0.31727    C:0.20482    A:0.31124    G:0.16667
position  3:    T:0.25703    C:0.38153    A:0.09639    G:0.26506
Average         T:0.25703    C:0.27979    A:0.21218    G:0.25100

#7: Chlorocebus_sabaeus
position  1:    T:0.19679    C:0.25301    A:0.23092    G:0.31928
position  2:    T:0.31727    C:0.20281    A:0.30924    G:0.17068
position  3:    T:0.24900    C:0.38755    A:0.09639    G:0.26707
Average         T:0.25435    C:0.28112    A:0.21218    G:0.25234

#8: Saimiri_boliviensis
position  1:    T:0.19880    C:0.24498    A:0.22691    G:0.32932
position  2:    T:0.31928    C:0.20482    A:0.30723    G:0.16867
position  3:    T:0.28514    C:0.36145    A:0.08835    G:0.26506
Average         T:0.26774    C:0.27041    A:0.20750    G:0.25435

#9: Callithrix_jacchus
position  1:    T:0.19679    C:0.24699    A:0.22892    G:0.32731
position  2:    T:0.31928    C:0.20683    A:0.30522    G:0.16867
position  3:    T:0.28715    C:0.35141    A:0.09639    G:0.26506
Average         T:0.26774    C:0.26841    A:0.21017    G:0.25368

#10: Tarsius_syrichta
position  1:    T:0.20482    C:0.24699    A:0.23293    G:0.31526
position  2:    T:0.32129    C:0.19880    A:0.30924    G:0.17068
position  3:    T:0.28313    C:0.35743    A:0.11647    G:0.24297
Average         T:0.26975    C:0.26774    A:0.21954    G:0.24297

#11: Microcebus_murinus
position  1:    T:0.19277    C:0.26104    A:0.22892    G:0.31727
position  2:    T:0.30924    C:0.21285    A:0.30120    G:0.17671
position  3:    T:0.17671    C:0.47390    A:0.08434    G:0.26506
Average         T:0.22624    C:0.31593    A:0.20482    G:0.25301

#12: Daubentonia_madagascariensis
position  1:    T:0.18876    C:0.25904    A:0.22088    G:0.33133
position  2:    T:0.31124    C:0.20683    A:0.30723    G:0.17470
position  3:    T:0.21687    C:0.42169    A:0.08835    G:0.27309
Average         T:0.23896    C:0.29585    A:0.20549    G:0.25971

#13: Otolemur_garnettii
position  1:    T:0.19679    C:0.24900    A:0.22289    G:0.33133
position  2:    T:0.31928    C:0.20281    A:0.30321    G:0.17470
position  3:    T:0.20080    C:0.45382    A:0.11044    G:0.23494
Average         T:0.23896    C:0.30187    A:0.21218    G:0.24699

Sums of codon usage counts
------------------------------------------------------------------------------
Phe F TTT     165 | Ser S TCT      56 | Tyr Y TAT     117 | Cys C TGT      59
      TTC     199 |       TCC     150 |       TAC     217 |       TGC      85
Leu L TTA      17 |       TCA      25 | *** * TAA       0 | *** * TGA       0
      TTG     109 |       TCG      25 |       TAG       0 | Trp W TGG      52
------------------------------------------------------------------------------
Leu L CTT      98 | Pro P CCT     149 | His H CAT      80 | Arg R CGT      43
      CTC     123 |       CCC     153 |       CAC     133 |       CGC      94
      CTA      31 |       CCA      33 | Gln Q CAA      53 |       CGA      59
```

```
      CTG     360 |       CCG      31 |       CAG     159 |       CGG      28
------------------------------------------------------------------------------
Ile I ATT     112 | Thr T ACT     102 | Asn N AAT     138 | Ser S AGT      42
      ATC     235 |       ACC     129 |       AAC      82 |       AGC     109
      ATA      18 |       ACA     107 | Lys K AAA      37 | Arg R AGA      12
Met M ATG     121 |       ACG      63 |       AAG     109 |       AGG      71
------------------------------------------------------------------------------
Val V GTT      58 | Ala A GCT     125 | Asp D GAT     229 | Gly G GGT      48
      GTC     177 |       GCC     145 |       GAC     308 |       GGC     189
      GTA      33 |       GCA      26 | Glu E GAA      76 |       GGA     105
      GTG     192 |       GCG       4 |       GAG     257 |       GGG     112
------------------------------------------------------------------------------
```

```
Codon position x base (3x4) table, overall

position  1:    T:0.19710    C:0.25131    A:0.22969    G:0.32190
position  2:    T:0.31634    C:0.20436    A:0.30816    G:0.17115
position  3:    T:0.25039    C:0.39049    A:0.09762    G:0.26151
Average         T:0.25461    C:0.28205    A:0.21182    G:0.25152
```

```
Nei & Gojobori 1986. dN/dS (dN, dS)
(Note: This matrix is not used in later ML. analysis.
Use runmode = -2 for ML pairwise comparison.)

Homo_sapiens
Pan_troglodytes      1.2604 (0.0106 0.0084)
Gorilla_gorilla_gorilla 1.3389 (0.0151 0.0113) 0.7351 (0.0062 0.0084)
Pongo_abelii         0.4378 (0.0296 0.0676) 0.2910 (0.0187 0.0643) 0.3714 (0.0250 0.0674)
Macaca_mulatta       0.2054 (0.0269 0.1309) 0.1261 (0.0160 0.1271) 0.1712 (0.0224 0.1306) 0.2017
(0.0278 0.1377)
Papio_anubis         0.2052 (0.0242 0.1177) 0.1169 (0.0133 0.1141) 0.1672 (0.0196 0.1174) 0.1773
(0.0232 0.1311) 0.2434 (0.0062 0.0255)
Chlorocebus_sabaeus  0.2404 (0.0260 0.1080) 0.1448 (0.0151 0.1045) 0.1989 (0.0214 0.1078) 0.2216
(0.0269 0.1212) 0.2126 (0.0098 0.0459) 0.2498 (0.0071 0.0284)
Saimiri_boliviensis  0.1999 (0.0273 0.1366) 0.1180 (0.0165 0.1395) 0.1466 (0.0210 0.1430) 0.1860
(0.0296 0.1590) 0.1788 (0.0237 0.1326) 0.1758 (0.0210 0.1193) 0.1607 (0.0205 0.1277)
Callithrix_jacchus   0.1917 (0.0301 0.1568) 0.1211 (0.0187 0.1545) 0.1469 (0.0232 0.1582) 0.1798
(0.0323 0.1798) 0.1694 (0.0264 0.1560) 0.1665 (0.0237 0.1424) 0.1612 (0.0232 0.1442) 0.1129 (0.0080
0.0705)
Tarsius_syrichta     0.1616 (0.0555 0.3433) 0.1311 (0.0442 0.3376) 0.1428 (0.0489 0.3424) 0.1715
(0.0578 0.3372) 0.1490 (0.0524 0.3518) 0.1446 (0.0496 0.3429) 0.1330 (0.0486 0.3656) 0.1388 (0.0448
0.3229) 0.1430 (0.0467 0.3267)
Microcebus_murinus   0.1916 (0.0639 0.3333) 0.1568 (0.0534 0.3407) 0.1656 (0.0572 0.3455) 0.1719
(0.0667 0.3880) 0.1667 (0.0615 0.3691) 0.1692 (0.0587 0.3468) 0.1561 (0.0549 0.3515) 0.1398 (0.0539
0.3851) 0.1458 (0.0567 0.3891) 0.1536 (0.0581 0.3783)
Daubentonia_madagascariensis 0.1723 (0.0506 0.2938) 0.1328 (0.0394 0.2968) 0.1463 (0.0441 0.3013)
0.1601 (0.0525 0.3279) 0.1535 (0.0464 0.3025) 0.1536 (0.0446 0.2902) 0.1386 (0.0408 0.2945) 0.1258
(0.0398 0.3167) 0.1313 (0.0427 0.3247) 0.1513 (0.0473 0.3126) 0.2022 (0.0288 0.1425)
Otolemur_garnettii   0.1351 (0.0619 0.4579) 0.1163 (0.0524 0.4509) 0.1189 (0.0543 0.4566) 0.1462
(0.0652 0.4459) 0.1262 (0.0601 0.4759) 0.1256 (0.0572 0.4552) 0.1227 (0.0553 0.4505) 0.1057 (0.0481
0.4554) 0.1097 (0.0510 0.4649) 0.1315 (0.0609 0.4633) 0.1247 (0.0380 0.3045) 0.1087 (0.0334 0.3073)
```

```
TREE #  1:  ((((((1, 2), 3), 4), ((5, 6), 7)), (8, 9)), 10, ((11, 12), 13));   MP score: 518
check convergence..
lnL(ntime: 23  np: 25):  -4804.596104      +0.000000
  14..15   15..16   16..17   17..18   18..19   19..1    19..2    18..3    17..4    16..20   20..21
21..5    21..6    20..7    15..22   22..8    22..9    14..10   14..23   23..24   24..11   24..12
23..13
 0.099582 0.023327 0.029792 0.020824 0.000004 0.028685 0.002153 0.018362 0.066945 0.043974 0.011178
0.025884 0.008111 0.018844 0.036729 0.027810 0.041262 0.186047 0.095057 0.029518 0.119416 0.059032
0.216920 4.163706 0.164505

Note: Branch length is defined as number of nucleotide substitutions per codon (not per neucleotide
site).

tree length =   1.20945

((((((1: 0.028685, 2: 0.002153): 0.000004, 3: 0.018362): 0.020824, 4: 0.066945): 0.029792, ((5:
0.025884, 6: 0.008111): 0.011178, 7: 0.018844): 0.043974): 0.023327, (8: 0.027810, 9: 0.041262):
0.036729): 0.099582, 10: 0.186047, ((11: 0.119416, 12: 0.059032): 0.029518, 13: 0.216920): 0.095057);

((((((Homo_sapiens: 0.028685, Pan_troglodytes: 0.002153): 0.000004, Gorilla_gorilla_gorilla: 0.018362):
0.020824, Pongo_abelii: 0.066945): 0.029792, ((Macaca_mulatta: 0.025884, Papio_anubis: 0.008111):
0.011178, Chlorocebus_sabaeus: 0.018844): 0.043974): 0.023327, (Saimiri_boliviensis: 0.027810,
```

Callithrix_jacchus: 0.041262): 0.036729): 0.099582, Tarsius_syrichta: 0.186047, ((Microcebus_murinus: 0.119416, Daubentonia_madagascariensis: 0.059032): 0.029518, Otolemur_garnettii: 0.216920): 0.095057);

Detailed output identifying parameters

kappa (ts/tv) = 4.16371

w (dN/dS) for branches: 0.16451 1.00000

dN & dS for each branch

branch	t	N	S	dN/dS	dN	dS	N*dN	S*dS
14..15	0.100	1089.1	404.9	0.1645	0.0140	0.0849	15.2	34.4
15..16	0.023	1089.1	404.9	0.1645	0.0033	0.0199	3.6	8.1
16..17	0.030	1089.1	404.9	1.0000	0.0099	0.0099	10.8	4.0
17..18	0.021	1089.1	404.9	1.0000	0.0069	0.0069	7.6	2.8
18..19	0.000	1089.1	404.9	1.0000	0.0000	0.0000	0.0	0.0
19..1	0.029	1089.1	404.9	1.0000	0.0096	0.0096	10.4	3.9
19..2	0.002	1089.1	404.9	1.0000	0.0007	0.0007	0.8	0.3
18..3	0.018	1089.1	404.9	1.0000	0.0061	0.0061	6.7	2.5
17..4	0.067	1089.1	404.9	1.0000	0.0223	0.0223	24.3	9.0
16..20	0.044	1089.1	404.9	0.1645	0.0062	0.0375	6.7	15.2
20..21	0.011	1089.1	404.9	0.1645	0.0016	0.0095	1.7	3.9
21..5	0.026	1089.1	404.9	0.1645	0.0036	0.0221	4.0	8.9
21..6	0.008	1089.1	404.9	0.1645	0.0011	0.0069	1.2	2.8
20..7	0.019	1089.1	404.9	0.1645	0.0026	0.0161	2.9	6.5
15..22	0.037	1089.1	404.9	0.1645	0.0052	0.0313	5.6	12.7
22..8	0.028	1089.1	404.9	0.1645	0.0039	0.0237	4.2	9.6
22..9	0.041	1089.1	404.9	0.1645	0.0058	0.0352	6.3	14.2
14..10	0.186	1089.1	404.9	0.1645	0.0261	0.1586	28.4	64.2
14..23	0.095	1089.1	404.9	0.1645	0.0133	0.0810	14.5	32.8
23..24	0.030	1089.1	404.9	0.1645	0.0041	0.0252	4.5	10.2
24..11	0.119	1089.1	404.9	0.1645	0.0167	0.1018	18.2	41.2
24..12	0.059	1089.1	404.9	0.1645	0.0083	0.0503	9.0	20.4
23..13	0.217	1089.1	404.9	0.1645	0.0304	0.1849	33.1	74.9

tree length for dN: 0.2018
tree length for dS: 0.9446

dS tree:
((((((Homo_sapiens: 0.009562, Pan_troglodytes: 0.000718): 0.000001, Gorilla_gorilla_gorilla: 0.006121): 0.006941, Pongo_abelii: 0.022315): 0.009931, ((Macaca_mulatta: 0.022068, Papio_anubis: 0.006915): 0.009530, Chlorocebus_sabaeus: 0.016066): 0.037492): 0.019888, (Saimiri_boliviensis: 0.023711, Callithrix_jacchus: 0.035180): 0.031315): 0.084903, Tarsius_syrichta: 0.158623, ((Microcebus_murinus: 0.101814, Daubentonia_madagascariensis: 0.050330): 0.025167, Otolemur_garnettii: 0.184945): 0.081045);
dN tree:
((((((Homo_sapiens: 0.009562, Pan_troglodytes: 0.000718): 0.000001, Gorilla_gorilla_gorilla: 0.006121): 0.006941, Pongo_abelii: 0.022315): 0.009931, ((Macaca_mulatta: 0.003630, Papio_anubis: 0.001138): 0.001568, Chlorocebus_sabaeus: 0.002643): 0.006168): 0.003272, (Saimiri_boliviensis: 0.003901, Callithrix_jacchus: 0.005787): 0.005151): 0.013967, Tarsius_syrichta: 0.026094, ((Microcebus_murinus: 0.016749, Daubentonia_madagascariensis: 0.008280): 0.004140, Otolemur_garnettii: 0.030424): 0.013332);

w ratios as labels for TreeView:
((((((Homo_sapiens #1.0000 , Pan_troglodytes #1.0000) #1.0000 , Gorilla_gorilla_gorilla #1.0000) #1.0000 , Pongo_abelii #1.0000) #1.0000 , ((Macaca_mulatta #0.1645 , Papio_anubis #0.1645) #0.1645 , Chlorocebus_sabaeus #0.1645) #0.1645) #0.1645 , (Saimiri_boliviensis #0.1645 , Callithrix_jacchus #0.1645) #0.1645) #0.1645 , Tarsius_syrichta #0.1645 , ((Microcebus_murinus #0.1645 , Daubentonia_madagascariensis #0.1645) #0.1645 , Otolemur_garnettii #0.1645) #0.1645);

Main result file for model I: catarrhines-I.mlc

```
CODONML (in paml version 4.7b, October 2013)  catarrhines.phy
Model: several dN/dS ratios for branches for branches,
Codon frequency model: F3x4
ns =  13  ls = 498

Codon usage in sequences
--------------------------------------------------------------------------------------------------------------------------------------
Phe TTT  13  13  13  14  13  13 | Ser TCT   5   5   5   7   4   4 | Tyr TAT  11  10  11  10   9   8 | Cys TGT   3   4   4   6   5   5
    TTC  15  15  15  13  15  15 |     TCC  11  11  11   9  11  11 |     TAC  16  16  15  15  17  18 |     TGC   8   6   6   7   5   5
Leu TTA   1   1   1   2   2   2 |     TCA   2   2   2   3   2   2 | *** TAA   0   0   0   0   0   0 | *** TGA   0   0   0   0   0   0
    TTG   9   9   9   8   9   9 |     TCG   2   2   2   1   2   2 |     TAG   0   0   0   0   0   0 | Trp TGG   4   4   4   4   4   4
--------------------------------------------------------------------------------------------------------------------------------------
Leu CTT  10   9   9   8   6   6 | Pro CCT  13  14  15  12  10  10 | His CAT   6   6   6   6   6   6 | Arg CGT   4   4   3   3   4   3
    CTC   8   8   8  10   9   9 |     CCC  10  10   9  11  13  14 |     CAC  11  10  10  11  10  10 |     CGC   6   8   8   5   7   8
    CTA   2   2   2   2   2   2 |     CCA   3   3   3   3   2   2 | Gln CAA   5   4   4   4   4   4 |     CGA   3   5   5   5   5   5
    CTG  27  27  27  27  30  30 |     CCG   2   2   2   2   4   3 |     CAG  11  11  12  13  13  13 |     CGG   3   2   2   2   1   1
--------------------------------------------------------------------------------------------------------------------------------------
Ile ATT  10  10  10   9  11  10 | Thr ACT   6   6   6   9   9   9 | Asn AAT  12  11  12  11  14  12 | Ser AGT   2   2   3   2   3   3
    ATC  17  17  18  18  16  17 |     ACC  11  10  10   9   8   9 |     AAC   7   6   6   6   4   6 |     AGC  10  10  10  10   9   9
    ATA   1   1   0   1   1   1 |     ACA   9   9   9   8  11  10 | Lys AAA   3   3   3   3   3   3 | Arg AGA   1   0   0   1   0   0
Met ATG   8   9   9  11   8   8 |     ACG   7   6   6   4   3   3 |     AAG   8   8   8   8   9   8 |     AGG   6   6   6   7   6   6
--------------------------------------------------------------------------------------------------------------------------------------
Val GTT   3   3   3   3   4   5 | Ala GCT  10  10  10  11   9   9 | Asp GAT  17  18  17  17  21  21 | Gly GGT   2   3   3   2   4   4
    GTC  16  15  15  15  15  14 |     GCC   8  10   9   9  12  12 |     GAC  23  24  24  24  20  20 |     GGC  14  14  14  15  13  13
    GTA   2   3   2   3   2   2 |     GCA   2   2   2   2   2   2 | Glu GAA   5   5   5   4   5   4 |     GGA   9   9   9  10   9   9
    GTG  15  14  16  14  15  15 |     GCG   0   0   0   0   0   0 |     GAG  22  22  21  21  20  22 |     GGG   8   9   9   8   8   8
--------------------------------------------------------------------------------------------------------------------------------------


--------------------------------------------------------------------------------------------------------------------------------------
Phe TTT  13  13  12  16  10  12 | Ser TCT   4   3   4   4   4   4 | Tyr TAT   9  12  12  11   5   6 | Cys TGT   4   4   6   7   2   4
    TTC  15  15  15  15  17  14 |     TCC  11  11  11  13  14  14 |     TAC  17  14  14  14  20  19 |     TGC   6   7   5   4  10   8
Leu TTA   2   1   1   0   2   0 |     TCA   2   2   2   2   1   1 | *** TAA   0   0   0   0   0   0 | *** TGA   0   0   0   0   0   0
    TTG   9  11  10   9   5   6 |     TCG   2   2   2   3   2   2 |     TAG   0   0   0   0   0   0 | Trp TGG   4   4   4   4   4   4
--------------------------------------------------------------------------------------------------------------------------------------
Leu CTT   6  10  11   5   4   7 | Pro CCT  10  11  11  12   9   9 | His CAT   6   6   7   8   5   6 | Arg CGT   3   3   2   4   4   2
    CTC   9   9   8  12  12  11 |     CCC  14  13  12  10  15  12 |     CAC  10   9   9   8  12  12 |     CGC   8   7   7   6   9   8
    CTA   3   2   2   4   2   2 |     CCA   2   2   2   4   2   3 | Gln CAA   5   3   4   3   5   3 |     CGA   4   5   5   6   3   5
    CTG  29  25  27  25  30  30 |     CCG   3   2   2   1   3   3 |     CAG  12  14  12  12  12  13 |     CGG   2   1   2   3   3   3
--------------------------------------------------------------------------------------------------------------------------------------
Ile ATT   9  11  10   8   4   5 | Thr ACT   9   9   9   9   6   8 | Asn AAT  12  12  11  11   5   9 | Ser AGT   3   5   5   5   3   3
    ATC  17  17  18  19  22  19 |     ACC   9   7   7  10  14  12 |     AAC   6   5   5   5   9   7 |     AGC   9   7   8   5   8   7
    ATA   1   1   1   4   2   2 |     ACA   9   9  10   5   6   6 | Lys AAA   3   2   2   4   3   2 | Arg AGA   1   0   0   2   2   2
Met ATG   9   9  10  10  11   9 |     ACG   4   5   4   5   6   6 |     AAG   8   7   7  10  10   9 |     AGG   6   7   7   4   3   4
--------------------------------------------------------------------------------------------------------------------------------------
Val GTT   3   7   8   6   4   4 | Ala GCT   9  12  12  10   9   7 | Asp GAT  20  20  20  19  11  17 | Gly GGT   4   4   3   6   3   5
    GTC  16  12   9  10  12  14 |     GCC  11  12  12   9  13  13 |     GAC  21  22  22  24  31  25 |     GGC  14  13  13  14  18  15
    GTA   2   2   2   4   3   2 |     GCA   2   2   2   2   0   3 | Glu GAA   4   5   6  11   5   6 |     GGA   8   8   9   7   6   7
    GTG  15  14  15  13  14  18 |     GCG   0   0   1   0   2   0 |     GAG  21  22  21  14  17  19 |     GGG   9   9   8   8  10  10
--------------------------------------------------------------------------------------------------------------------------------------


------------------------------------------------------
Phe TTT  10 | Ser TCT   3 | Tyr TAT   3 | Cys TGT   5
    TTC  20 |     TCC  12 |     TAC  22 |     TGC   8
Leu TTA   2 |     TCA   2 | *** TAA   0 | *** TGA   0
    TTG   6 |     TCG   1 |     TAG   0 | Trp TGG   4
------------------------------------------------------
Leu CTT   7 | Pro CCT  13 | His CAT   6 | Arg CGT   4
    CTC  10 |     CCC  10 |     CAC  11 |     CGC   7
    CTA   4 |     CCA   2 | Gln CAA   5 |     CGA   3
    CTG  26 |     CCG   2 |     CAG  11 |     CGG   3
------------------------------------------------------
Ile ATT   5 | Thr ACT   7 | Asn AAT   6 | Ser AGT   3
    ATC  20 |     ACC  13 |     AAC  10 |     AGC   7
    ATA   2 |     ACA   6 | Lys AAA   3 | Arg AGA   3
Met ATG  10 |     ACG   4 |     AAG   9 |     AGG   3
------------------------------------------------------
Val GTT   5 | Ala GCT   7 | Asp GAT  11 | Gly GGT   5
    GTC  14 |     GCC  15 |     GAC  28 |     GGC  19
    GTA   4 |     GCA   3 | Glu GAA  11 |     GGA   5
    GTG  14 |     GCG   1 |     GAG  15 |     GGG   8
------------------------------------------------------

Codon position x base (3x4) table for each sequence.

#1: Homo_sapiens
position  1:    T:0.20080    C:0.24900    A:0.23695    G:0.31325
position  2:    T:0.31526    C:0.20281    A:0.31526    G:0.16667
position  3:    T:0.25502    C:0.38353    A:0.09639    G:0.26506
Average         T:0.25703    C:0.27845    A:0.21620    G:0.24833

#2: Pan_troglodytes
position  1:    T:0.19679    C:0.25100    A:0.22892    G:0.32329
position  2:    T:0.31325    C:0.20482    A:0.30924    G:0.17269
position  3:    T:0.25703    C:0.38153    A:0.09839    G:0.26305
Average         T:0.25569    C:0.27912    A:0.21218    G:0.25301
```

```
#3: Gorilla_gorilla_gorilla
position  1:    T:0.19679    C:0.25100    A:0.23293    G:0.31928
position  2:    T:0.31526    C:0.20281    A:0.30924    G:0.17269
position  3:    T:0.26104    C:0.37751    A:0.09438    G:0.26707
Average         T:0.25770    C:0.27711    A:0.21218    G:0.25301

#4: Pongo_abelii
position  1:    T:0.19880    C:0.24900    A:0.23494    G:0.31727
position  2:    T:0.31727    C:0.20080    A:0.30723    G:0.17470
position  3:    T:0.26104    C:0.37550    A:0.10241    G:0.26104
Average         T:0.25904    C:0.27510    A:0.21486    G:0.25100

#5: Macaca_mulatta
position  1:    T:0.19679    C:0.25301    A:0.23092    G:0.31928
position  2:    T:0.31727    C:0.20482    A:0.31124    G:0.16667
position  3:    T:0.26506    C:0.36948    A:0.10040    G:0.26506
Average         T:0.25971    C:0.27577    A:0.21419    G:0.25033

#6: Papio_anubis
position  1:    T:0.19679    C:0.25301    A:0.22892    G:0.32129
position  2:    T:0.31727    C:0.20482    A:0.31124    G:0.16667
position  3:    T:0.25703    C:0.38153    A:0.09639    G:0.26506
Average         T:0.25703    C:0.27979    A:0.21218    G:0.25100

#7: Chlorocebus_sabaeus
position  1:    T:0.19679    C:0.25301    A:0.23092    G:0.31928
position  2:    T:0.31727    C:0.20281    A:0.30924    G:0.17068
position  3:    T:0.24900    C:0.38755    A:0.09639    G:0.26707
Average         T:0.25435    C:0.28112    A:0.21218    G:0.25234

#8: Saimiri_boliviensis
position  1:    T:0.19880    C:0.24498    A:0.22691    G:0.32932
position  2:    T:0.31928    C:0.20482    A:0.30723    G:0.16867
position  3:    T:0.28514    C:0.36145    A:0.08835    G:0.26506
Average         T:0.26774    C:0.27041    A:0.20750    G:0.25435

#9: Callithrix_jacchus
position  1:    T:0.19679    C:0.24699    A:0.22892    G:0.32731
position  2:    T:0.31928    C:0.20683    A:0.30522    G:0.16867
position  3:    T:0.28715    C:0.35141    A:0.09639    G:0.26506
Average         T:0.26774    C:0.26841    A:0.21017    G:0.25368

#10: Tarsius_syrichta
position  1:    T:0.20482    C:0.24699    A:0.23293    G:0.31526
position  2:    T:0.32129    C:0.19880    A:0.30924    G:0.17068
position  3:    T:0.28313    C:0.35743    A:0.11647    G:0.24297
Average         T:0.26975    C:0.26774    A:0.21954    G:0.24297

#11: Microcebus_murinus
position  1:    T:0.19277    C:0.26104    A:0.22892    G:0.31727
position  2:    T:0.30924    C:0.21285    A:0.30120    G:0.17671
position  3:    T:0.17671    C:0.47390    A:0.08434    G:0.26506
Average         T:0.22624    C:0.31593    A:0.20482    G:0.25301

#12: Daubentonia_madagascariensis
position  1:    T:0.18876    C:0.25904    A:0.22088    G:0.33133
position  2:    T:0.31124    C:0.20683    A:0.30723    G:0.17470
position  3:    T:0.21687    C:0.42169    A:0.08835    G:0.27309
Average         T:0.23896    C:0.29585    A:0.20549    G:0.25971

#13: Otolemur_garnettii
position  1:    T:0.19679    C:0.24900    A:0.22289    G:0.33133
position  2:    T:0.31928    C:0.20281    A:0.30321    G:0.17470
position  3:    T:0.20080    C:0.45382    A:0.11044    G:0.23494
Average         T:0.23896    C:0.30187    A:0.21218    G:0.24699

Sums of codon usage counts
------------------------------------------------------------------------------
Phe F TTT     165 | Ser S TCT      56 | Tyr Y TAT     117 | Cys C TGT      59
      TTC     199 |       TCC     150 |       TAC     217 |       TGC      85
Leu L TTA      17 |       TCA      25 | *** * TAA       0 | *** * TGA       0
      TTG     109 |       TCG      25 |       TAG       0 | Trp W TGG      52
------------------------------------------------------------------------------
Leu L CTT      98 | Pro P CCT     149 | His H CAT      80 | Arg R CGT      43
      CTC     123 |       CCC     153 |       CAC     133 |       CGC      94
      CTA      31 |       CCA      33 | Gln Q CAA      53 |       CGA      59
      CTG     360 |       CCG      31 |       CAG     159 |       CGG      28
```

```
------------------------------------------------------------------------------
Ile I ATT     112 | Thr T ACT     102 | Asn N AAT     138 | Ser S AGT      42
      ATC     235 |       ACC     129 |       AAC      82 |       AGC     109
      ATA      18 |       ACA     107 | Lys K AAA      37 | Arg R AGA      12
Met M ATG     121 |       ACG      63 |       AAG     109 |       AGG      71
------------------------------------------------------------------------------
Val V GTT      58 | Ala A GCT     125 | Asp D GAT     229 | Gly G GGT      48
      GTC     177 |       GCC     145 |       GAC     308 |       GGC     189
      GTA      33 |       GCA      26 | Glu E GAA      76 |       GGA     105
      GTG     192 |       GCG       4 |       GAG     257 |       GGG     112
------------------------------------------------------------------------------
```

Codon position x base (3x4) table, overall

```
position  1:    T:0.19710    C:0.25131    A:0.22969    G:0.32190
position  2:    T:0.31634    C:0.20436    A:0.30816    G:0.17115
position  3:    T:0.25039    C:0.39049    A:0.09762    G:0.26151
Average         T:0.25461    C:0.28205    A:0.21182    G:0.25152
```

Nei & Gojobori 1986. dN/dS (dN, dS)
(Note: This matrix is not used in later ML. analysis.
Use runmode = -2 for ML pairwise comparison.)

```
Homo_sapiens
Pan_troglodytes      1.2604 (0.0106 0.0084)
Gorilla_gorilla_gorilla 1.3389 (0.0151 0.0113) 0.7351 (0.0062 0.0084)
Pongo_abelii         0.4378 (0.0296 0.0676) 0.2910 (0.0187 0.0643) 0.3714 (0.0250 0.0674)
Macaca_mulatta       0.2054 (0.0269 0.1309) 0.1261 (0.0160 0.1271) 0.1712 (0.0224 0.1306) 0.2017
(0.0278 0.1377)
Papio_anubis         0.2052 (0.0242 0.1177) 0.1169 (0.0133 0.1141) 0.1672 (0.0196 0.1174) 0.1773
(0.0232 0.1311) 0.2434 (0.0062 0.0255)
Chlorocebus_sabaeus  0.2404 (0.0260 0.1080) 0.1448 (0.0151 0.1045) 0.1989 (0.0214 0.1078) 0.2216
(0.0269 0.1212) 0.2126 (0.0098 0.0459) 0.2498 (0.0071 0.0284)
Saimiri_boliviensis  0.1999 (0.0273 0.1366) 0.1180 (0.0165 0.1395) 0.1466 (0.0210 0.1430) 0.1860
(0.0296 0.1590) 0.1788 (0.0237 0.1326) 0.1758 (0.0210 0.1193) 0.1607 (0.0205 0.1277)
Callithrix_jacchus   0.1917 (0.0301 0.1568) 0.1211 (0.0187 0.1545) 0.1469 (0.0232 0.1582) 0.1798
(0.0323 0.1798) 0.1694 (0.0264 0.1560) 0.1665 (0.0237 0.1424) 0.1612 (0.0232 0.1442) 0.1129 (0.0080
0.0705)
Tarsius_syrichta     0.1616 (0.0555 0.3433) 0.1311 (0.0442 0.3376) 0.1428 (0.0489 0.3424) 0.1715
(0.0578 0.3372) 0.1490 (0.0524 0.3518) 0.1446 (0.0496 0.3429) 0.1330 (0.0486 0.3656) 0.1388 (0.0448
0.3229) 0.1430 (0.0467 0.3267)
Microcebus_murinus   0.1916 (0.0639 0.3333) 0.1568 (0.0534 0.3407) 0.1656 (0.0572 0.3455) 0.1719
(0.0667 0.3880) 0.1667 (0.0615 0.3691) 0.1692 (0.0587 0.3468) 0.1561 (0.0549 0.3515) 0.1398 (0.0539
0.3851) 0.1458 (0.0567 0.3891) 0.1536 (0.0581 0.3783)
Daubentonia_madagascariensis 0.1723 (0.0506 0.2938) 0.1328 (0.0394 0.2968) 0.1463 (0.0441 0.3013)
0.1601 (0.0525 0.3279) 0.1535 (0.0464 0.3025) 0.1536 (0.0446 0.2902) 0.1386 (0.0408 0.2945) 0.1258
(0.0398 0.3167) 0.1313 (0.0427 0.3247) 0.1513 (0.0473 0.3126) 0.2022 (0.0288 0.1425)
Otolemur_garnettii   0.1351 (0.0619 0.4579) 0.1163 (0.0524 0.4509) 0.1189 (0.0543 0.4566) 0.1462
(0.0652 0.4459) 0.1262 (0.0601 0.4759) 0.1256 (0.0572 0.4552) 0.1227 (0.0553 0.4505) 0.1057 (0.0481
0.4554) 0.1097 (0.0510 0.4649) 0.1315 (0.0609 0.4633) 0.1247 (0.0380 0.3045) 0.1087 (0.0334 0.3073)
```

```
TREE #  1:  ((((((1, 2), 3), 4), ((5, 6), 7)), (8, 9)), 10, ((11, 12), 13));   MP score: 518
check convergence..
lnL(ntime: 23  np: 26):  -4792.275301      +0.000000
  14..15   15..16   16..17   17..18   18..19   19..1    19..2    18..3    17..4    16..20   20..21
21..5    21..6    20..7    15..22   22..8    22..9    14..10   14..23   23..24   24..11   24..12
23..13
 0.100989 0.022824 0.034086 0.021350 0.000004 0.028714 0.002058 0.018396 0.066777 0.039716 0.009913
0.025893 0.008206 0.020087 0.035149 0.027389 0.041756 0.187253 0.094997 0.029406 0.119768 0.059176
0.218250 4.034729 0.151816 0.437096
```

Note: Branch length is defined as number of nucleotide substitutions per codon (not per neucleotide site).

tree length =   1.21216

```
((((((1: 0.028714, 2: 0.002058): 0.000004, 3: 0.018396): 0.021350, 4: 0.066777): 0.034086, ((5:
0.025893, 6: 0.008206): 0.009913, 7: 0.020087): 0.039716): 0.022824, (8: 0.027389, 9: 0.041756):
0.035149): 0.100989, 10: 0.187253, ((11: 0.119768, 12: 0.059176): 0.029406, 13: 0.218250): 0.094997);

((((((Homo_sapiens: 0.028714, Pan_troglodytes: 0.002058): 0.000004, Gorilla_gorilla_gorilla: 0.018396):
0.021350, Pongo_abelii: 0.066777): 0.034086, ((Macaca_mulatta: 0.025893, Papio_anubis: 0.008206):
0.009913, Chlorocebus_sabaeus: 0.020087): 0.039716): 0.022824, (Saimiri_boliviensis: 0.027389,
Callithrix_jacchus: 0.041756): 0.035149): 0.100989, Tarsius_syrichta: 0.187253, ((Microcebus_murinus:
```

0.119768, Daubentonia_madagascariensis: 0.059176): 0.029406, Otolemur_garnettii: 0.218250): 0.094997);

Detailed output identifying parameters

kappa (ts/tv) = 4.03473

w (dN/dS) for branches: 0.15182 0.43710

dN & dS for each branch

branch	t	N	S	dN/dS	dN	dS	N*dN	S*dS
14..15	0.101	1090.8	403.2	0.1518	0.0134	0.0884	14.6	35.6
15..16	0.023	1090.8	403.2	0.1518	0.0030	0.0200	3.3	8.1
16..17	0.034	1090.8	403.2	0.1518	0.0045	0.0298	4.9	12.0
17..18	0.021	1090.8	403.2	0.1518	0.0028	0.0187	3.1	7.5
18..19	0.000	1090.8	403.2	0.1518	0.0000	0.0000	0.0	0.0
19..1	0.029	1090.8	403.2	0.4371	0.0071	0.0163	7.7	6.6
19..2	0.002	1090.8	403.2	0.1518	0.0003	0.0018	0.3	0.7
18..3	0.018	1090.8	403.2	0.4371	0.0046	0.0104	5.0	4.2
17..4	0.067	1090.8	403.2	0.4371	0.0165	0.0378	18.0	15.2
16..20	0.040	1090.8	403.2	0.4371	0.0098	0.0225	10.7	9.1
20..21	0.010	1090.8	403.2	0.4371	0.0025	0.0056	2.7	2.3
21..5	0.026	1090.8	403.2	0.4371	0.0064	0.0147	7.0	5.9
21..6	0.008	1090.8	403.2	0.4371	0.0020	0.0046	2.2	1.9
20..7	0.020	1090.8	403.2	0.4371	0.0050	0.0114	5.4	4.6
15..22	0.035	1090.8	403.2	0.1518	0.0047	0.0308	5.1	12.4
22..8	0.027	1090.8	403.2	0.1518	0.0036	0.0240	4.0	9.7
22..9	0.042	1090.8	403.2	0.1518	0.0056	0.0366	6.1	14.7
14..10	0.187	1090.8	403.2	0.1518	0.0249	0.1640	27.2	66.1
14..23	0.095	1090.8	403.2	0.1518	0.0126	0.0832	13.8	33.5
23..24	0.029	1090.8	403.2	0.1518	0.0039	0.0257	4.3	10.4
24..11	0.120	1090.8	403.2	0.1518	0.0159	0.1049	17.4	42.3
24..12	0.059	1090.8	403.2	0.1518	0.0079	0.0518	8.6	20.9
23..13	0.218	1090.8	403.2	0.1518	0.0290	0.1911	31.6	77.0

tree length for dN: 0.1860
tree length for dS: 0.9939

dS tree:
((((((Homo_sapiens: 0.016250, Pan_troglodytes: 0.001802): 0.000004, Gorilla_gorilla_gorilla: 0.010411): 0.018694, Pongo_abelii: 0.037791): 0.029845, ((Macaca_mulatta: 0.014654, Papio_anubis: 0.004644): 0.005610, Chlorocebus_sabaeus: 0.011368): 0.022477): 0.019984, (Saimiri_boliviensis: 0.023981, Callithrix_jacchus: 0.036561): 0.030776): 0.088424, Tarsius_syrichta: 0.163956, ((Microcebus_murinus: 0.104868, Daubentonia_madagascariensis: 0.051813): 0.025748, Otolemur_garnettii: 0.191097): 0.083178);
dN tree:
((((((Homo_sapiens: 0.007103, Pan_troglodytes: 0.000274): 0.000001, Gorilla_gorilla_gorilla: 0.004551): 0.002838, Pongo_abelii: 0.016518): 0.004531, ((Macaca_mulatta: 0.006405, Papio_anubis: 0.002030): 0.002452, Chlorocebus_sabaeus: 0.004969): 0.009824): 0.003034, (Saimiri_boliviensis: 0.003641, Callithrix_jacchus: 0.005550): 0.004672): 0.013424, Tarsius_syrichta: 0.024891, ((Microcebus_murinus: 0.015921, Daubentonia_madagascariensis: 0.007866): 0.003909, Otolemur_garnettii: 0.029012): 0.012628);

w ratios as labels for TreeView:
((((((Homo_sapiens #0.4371 , Pan_troglodytes #0.1518) #0.1518 , Gorilla_gorilla_gorilla #0.4371) #0.1518 , Pongo_abelii #0.4371) #0.1518 , ((Macaca_mulatta #0.4371 , Papio_anubis #0.4371) #0.4371 , Chlorocebus_sabaeus #0.4371) #0.4371) #0.1518 , (Saimiri_boliviensis #0.1518 , Callithrix_jacchus #0.1518) #0.1518) #0.1518 , Tarsius_syrichta #0.1518 , ((Microcebus_murinus #0.1518 , Daubentonia_madagascariensis #0.1518) #0.1518 , Otolemur_garnettii #0.1518) #0.1518);

Main result file for model J: catarrhines-J.mlc

```
CODONML (in paml version 4.7b, October 2013)  catarrhines.phy
Model: several dN/dS ratios for branches for branches,  omega = 1.000 fixed

Codon frequency model: F3x4
ns =  13  ls = 498

Codon usage in sequences
--------------------------------------------------------------------------------------------------------------------------------------
Phe TTT  13  13  13  14  13  13 | Ser TCT   5   5   5   7   4   4 | Tyr TAT  11  10  11  10   9   8 | Cys TGT   3   4   4   6   5   5
    TTC  15  15  15  13  15  15 |     TCC  11  11  11   9  11  11 |     TAC  16  16  15  15  17  18 |     TGC   8   6   6   7   5   5
Leu TTA   1   1   1   2   2   2 |     TCA   2   2   2   3   2   2 | *** TAA   0   0   0   0   0   0 | *** TGA   0   0   0   0   0   0
    TTG   9   9   9   8   9   9 |     TCG   2   2   2   1   2   2 |     TAG   0   0   0   0   0   0 | Trp TGG   4   4   4   4   4   4
--------------------------------------------------------------------------------------------------------------------------------------
Leu CTT  10   9   9   8   6   6 | Pro CCT  13  14  15  12  10  10 | His CAT   6   6   6   6   6   6 | Arg CGT   4   4   3   3   4   3
    CTC   8   8   8  10   9   9 |     CCC  10  10   9  11  13  14 |     CAC  11  10  10  11  10  10 |     CGC   6   8   8   5   7   8
    CTA   2   2   2   2   2   2 |     CCA   3   3   3   3   2   2 | Gln CAA   5   4   4   4   4   4 |     CGA   3   5   5   5   5   5
    CTG  27  27  27  27  30  30 |     CCG   2   2   2   2   4   3 |     CAG  11  11  12  13  13  13 |     CGG   3   2   2   2   1   1
--------------------------------------------------------------------------------------------------------------------------------------
Ile ATT  10  10  10   9  11  10 | Thr ACT   6   6   6   9   9   9 | Asn AAT  12  11  12  11  14  12 | Ser AGT   2   2   3   2   3   3
    ATC  17  17  18  18  16  17 |     ACC  11  10  10   9   8   9 |     AAC   7   6   6   6   4   6 |     AGC  10  10  10  10   9   9
    ATA   1   1   0   1   1   1 |     ACA   9   9   9   8  11  10 | Lys AAA   3   3   3   3   3   3 | Arg AGA   1   0   0   1   0   0
Met ATG   8   9   9  11   8   8 |     ACG   7   6   6   4   3   3 |     AAG   8   8   8   8   9   8 |     AGG   6   6   6   7   6   6
--------------------------------------------------------------------------------------------------------------------------------------
Val GTT   3   3   3   3   4   5 | Ala GCT  10  10  10  11   9   9 | Asp GAT  17  18  17  17  21  21 | Gly GGT   2   3   3   2   4   4
    GTC  16  15  15  15  15  14 |     GCC   8  10   9   9  12  12 |     GAC  23  24  24  24  20  20 |     GGC  14  14  14  15  13  13
    GTA   2   3   2   3   2   2 |     GCA   2   2   2   2   2   2 | Glu GAA   5   5   5   4   5   4 |     GGA   9   9   9  10   9   9
    GTG  15  14  16  14  15  15 |     GCG   0   0   0   0   0   0 |     GAG  22  22  21  21  20  22 |     GGG   8   9   9   8   8   8
--------------------------------------------------------------------------------------------------------------------------------------

--------------------------------------------------------------------------------------------------------------------------------------
Phe TTT  13  13  12  16  10  12 | Ser TCT   4   3   4   4   4   4 | Tyr TAT   9  12  12  11   5   6 | Cys TGT   4   4   6   7   2   4
    TTC  15  15  15  15  17  14 |     TCC  11  11  11  13  14  14 |     TAC  17  14  14  14  20  19 |     TGC   6   7   5   4  10   8
Leu TTA   2   1   1   0   2   0 |     TCA   2   2   2   2   1   1 | *** TAA   0   0   0   0   0   0 | *** TGA   0   0   0   0   0   0
    TTG   9  11  10   9   5   6 |     TCG   2   2   2   3   2   2 |     TAG   0   0   0   0   0   0 | Trp TGG   4   4   4   4   4   4
--------------------------------------------------------------------------------------------------------------------------------------
Leu CTT   6  10  11   5   4   7 | Pro CCT  10  11  11  12   9   9 | His CAT   6   6   7   8   5   6 | Arg CGT   3   3   2   4   4   2
    CTC   9   9   8  12  12  11 |     CCC  14  13  12  10  15  12 |     CAC  10   9   9   8  12  12 |     CGC   8   7   7   6   9   8
    CTA   3   2   2   4   2   2 |     CCA   2   2   2   4   2   3 | Gln CAA   5   3   4   3   5   3 |     CGA   4   5   5   6   3   5
    CTG  29  25  27  25  30  30 |     CCG   3   2   2   1   3   3 |     CAG  12  14  12  12  12  13 |     CGG   2   1   2   3   3   3
--------------------------------------------------------------------------------------------------------------------------------------
Ile ATT   9  11  10   8   4   5 | Thr ACT   9   9   9   9   6   8 | Asn AAT  12  12  11  11   5   9 | Ser AGT   3   5   5   5   3   3
    ATC  17  17  18  19  22  19 |     ACC   9   7   7  10  14  12 |     AAC   6   5   5   5   9   7 |     AGC   9   7   8   5   8   7
    ATA   1   1   1   4   2   2 |     ACA   9   9  10   5   6   6 | Lys AAA   3   2   2   4   3   2 | Arg AGA   1   0   0   2   2   2
Met ATG   9   9  10  10  11   9 |     ACG   4   5   4   5   6   6 |     AAG   8   7   7  10  10   9 |     AGG   6   7   7   4   3   4
--------------------------------------------------------------------------------------------------------------------------------------
Val GTT   3   7   8   6   4   4 | Ala GCT   9  12  12  10   9   7 | Asp GAT  20  20  20  19  11  17 | Gly GGT   4   4   3   6   3   5
    GTC  16  12   9  10  12  14 |     GCC  11  12  12   9  13  13 |     GAC  21  22  22  24  31  25 |     GGC  14  13  13  14  18  15
    GTA   2   2   2   4   3   2 |     GCA   2   2   2   2   0   3 | Glu GAA   4   5   6  11   5   6 |     GGA   8   8   9   7   6   7
    GTG  15  14  15  13  14  18 |     GCG   0   0   1   0   2   0 |     GAG  21  22  21  14  17  19 |     GGG   9   9   8   8  10  10
--------------------------------------------------------------------------------------------------------------------------------------

------------------------------------------------------
Phe TTT  10 | Ser TCT   3 | Tyr TAT   3 | Cys TGT   5
    TTC  20 |     TCC  12 |     TAC  22 |     TGC   8
Leu TTA   2 |     TCA   2 | *** TAA   0 | *** TGA   0
    TTG   6 |     TCG   1 |     TAG   0 | Trp TGG   4
------------------------------------------------------
Leu CTT   7 | Pro CCT  13 | His CAT   6 | Arg CGT   4
    CTC  10 |     CCC  10 |     CAC  11 |     CGC   7
    CTA   4 |     CCA   2 | Gln CAA   5 |     CGA   3
    CTG  26 |     CCG   2 |     CAG  11 |     CGG   3
------------------------------------------------------
Ile ATT   5 | Thr ACT   7 | Asn AAT   6 | Ser AGT   3
    ATC  20 |     ACC  13 |     AAC  10 |     AGC   7
    ATA   2 |     ACA   6 | Lys AAA   3 | Arg AGA   3
Met ATG  10 |     ACG   4 |     AAG   9 |     AGG   3
------------------------------------------------------
Val GTT   5 | Ala GCT   7 | Asp GAT  11 | Gly GGT   5
    GTC  14 |     GCC  15 |     GAC  28 |     GGC  19
    GTA   4 |     GCA   3 | Glu GAA  11 |     GGA   5
    GTG  14 |     GCG   1 |     GAG  15 |     GGG   8
------------------------------------------------------

Codon position x base (3x4) table for each sequence.

#1: Homo_sapiens
position  1:    T:0.20080    C:0.24900    A:0.23695    G:0.31325
position  2:    T:0.31526    C:0.20281    A:0.31526    G:0.16667
position  3:    T:0.25502    C:0.38353    A:0.09639    G:0.26506
Average         T:0.25703    C:0.27845    A:0.21620    G:0.24833

#2: Pan_troglodytes
position  1:    T:0.19679    C:0.25100    A:0.22892    G:0.32329
position  2:    T:0.31325    C:0.20482    A:0.30924    G:0.17269
position  3:    T:0.25703    C:0.38153    A:0.09839    G:0.26305
Average         T:0.25569    C:0.27912    A:0.21218    G:0.25301
```

```
#3: Gorilla_gorilla_gorilla
position  1:    T:0.19679    C:0.25100    A:0.23293    G:0.31928
position  2:    T:0.31526    C:0.20281    A:0.30924    G:0.17269
position  3:    T:0.26104    C:0.37751    A:0.09438    G:0.26707
Average         T:0.25770    C:0.27711    A:0.21218    G:0.25301

#4: Pongo_abelii
position  1:    T:0.19880    C:0.24900    A:0.23494    G:0.31727
position  2:    T:0.31727    C:0.20080    A:0.30723    G:0.17470
position  3:    T:0.26104    C:0.37550    A:0.10241    G:0.26104
Average         T:0.25904    C:0.27510    A:0.21486    G:0.25100

#5: Macaca_mulatta
position  1:    T:0.19679    C:0.25301    A:0.23092    G:0.31928
position  2:    T:0.31727    C:0.20482    A:0.31124    G:0.16667
position  3:    T:0.26506    C:0.36948    A:0.10040    G:0.26506
Average         T:0.25971    C:0.27577    A:0.21419    G:0.25033

#6: Papio_anubis
position  1:    T:0.19679    C:0.25301    A:0.22892    G:0.32129
position  2:    T:0.31727    C:0.20482    A:0.31124    G:0.16667
position  3:    T:0.25703    C:0.38153    A:0.09639    G:0.26506
Average         T:0.25703    C:0.27979    A:0.21218    G:0.25100

#7: Chlorocebus_sabaeus
position  1:    T:0.19679    C:0.25301    A:0.23092    G:0.31928
position  2:    T:0.31727    C:0.20281    A:0.30924    G:0.17068
position  3:    T:0.24900    C:0.38755    A:0.09639    G:0.26707
Average         T:0.25435    C:0.28112    A:0.21218    G:0.25234

#8: Saimiri_boliviensis
position  1:    T:0.19880    C:0.24498    A:0.22691    G:0.32932
position  2:    T:0.31928    C:0.20482    A:0.30723    G:0.16867
position  3:    T:0.28514    C:0.36145    A:0.08835    G:0.26506
Average         T:0.26774    C:0.27041    A:0.20750    G:0.25435

#9: Callithrix_jacchus
position  1:    T:0.19679    C:0.24699    A:0.22892    G:0.32731
position  2:    T:0.31928    C:0.20683    A:0.30522    G:0.16867
position  3:    T:0.28715    C:0.35141    A:0.09639    G:0.26506
Average         T:0.26774    C:0.26841    A:0.21017    G:0.25368

#10: Tarsius_syrichta
position  1:    T:0.20482    C:0.24699    A:0.23293    G:0.31526
position  2:    T:0.32129    C:0.19880    A:0.30924    G:0.17068
position  3:    T:0.28313    C:0.35743    A:0.11647    G:0.24297
Average         T:0.26975    C:0.26774    A:0.21954    G:0.24297

#11: Microcebus_murinus
position  1:    T:0.19277    C:0.26104    A:0.22892    G:0.31727
position  2:    T:0.30924    C:0.21285    A:0.30120    G:0.17671
position  3:    T:0.17671    C:0.47390    A:0.08434    G:0.26506
Average         T:0.22624    C:0.31593    A:0.20482    G:0.25301

#12: Daubentonia_madagascariensis
position  1:    T:0.18876    C:0.25904    A:0.22088    G:0.33133
position  2:    T:0.31124    C:0.20683    A:0.30723    G:0.17470
position  3:    T:0.21687    C:0.42169    A:0.08835    G:0.27309
Average         T:0.23896    C:0.29585    A:0.20549    G:0.25971

#13: Otolemur_garnettii
position  1:    T:0.19679    C:0.24900    A:0.22289    G:0.33133
position  2:    T:0.31928    C:0.20281    A:0.30321    G:0.17470
position  3:    T:0.20080    C:0.45382    A:0.11044    G:0.23494
Average         T:0.23896    C:0.30187    A:0.21218    G:0.24699

Sums of codon usage counts
------------------------------------------------------------------------------
Phe F TTT     165 | Ser S TCT      56 | Tyr Y TAT     117 | Cys C TGT      59
      TTC     199 |       TCC     150 |       TAC     217 |       TGC      85
Leu L TTA      17 |       TCA      25 | *** * TAA       0 | *** * TGA       0
      TTG     109 |       TCG      25 |       TAG       0 | Trp W TGG      52
------------------------------------------------------------------------------
Leu L CTT      98 | Pro P CCT     149 | His H CAT      80 | Arg R CGT      43
      CTC     123 |       CCC     153 |       CAC     133 |       CGC      94
      CTA      31 |       CCA      33 | Gln Q CAA      53 |       CGA      59
```

```
      CTG     360 |       CCG      31 |       CAG     159 |       CGG      28
------------------------------------------------------------------------------
Ile I ATT     112 | Thr T ACT     102 | Asn N AAT     138 | Ser S AGT      42
      ATC     235 |       ACC     129 |       AAC      82 |       AGC     109
      ATA      18 |       ACA     107 | Lys K AAA      37 | Arg R AGA      12
Met M ATG     121 |       ACG      63 |       AAG     109 |       AGG      71
------------------------------------------------------------------------------
Val V GTT      58 | Ala A GCT     125 | Asp D GAT     229 | Gly G GGT      48
      GTC     177 |       GCC     145 |       GAC     308 |       GGC     189
      GTA      33 |       GCA      26 | Glu E GAA      76 |       GGA     105
      GTG     192 |       GCG       4 |       GAG     257 |       GGG     112
------------------------------------------------------------------------------


Codon position x base (3x4) table, overall

position  1:    T:0.19710    C:0.25131    A:0.22969    G:0.32190
position  2:    T:0.31634    C:0.20436    A:0.30816    G:0.17115
position  3:    T:0.25039    C:0.39049    A:0.09762    G:0.26151
Average         T:0.25461    C:0.28205    A:0.21182    G:0.25152


Nei & Gojobori 1986. dN/dS (dN, dS)
(Note: This matrix is not used in later ML. analysis.
Use runmode = -2 for ML pairwise comparison.)

Homo_sapiens
Pan_troglodytes      1.2604 (0.0106 0.0084)
Gorilla_gorilla_gorilla 1.3389 (0.0151 0.0113) 0.7351 (0.0062 0.0084)
Pongo_abelii         0.4378 (0.0296 0.0676) 0.2910 (0.0187 0.0643) 0.3714 (0.0250 0.0674)
Macaca_mulatta       0.2054 (0.0269 0.1309) 0.1261 (0.0160 0.1271) 0.1712 (0.0224 0.1306) 0.2017
(0.0278 0.1377)
Papio_anubis         0.2052 (0.0242 0.1177) 0.1169 (0.0133 0.1141) 0.1672 (0.0196 0.1174) 0.1773
(0.0232 0.1311) 0.2434 (0.0062 0.0255)
Chlorocebus_sabaeus  0.2404 (0.0260 0.1080) 0.1448 (0.0151 0.1045) 0.1989 (0.0214 0.1078) 0.2216
(0.0269 0.1212) 0.2126 (0.0098 0.0459) 0.2498 (0.0071 0.0284)
Saimiri_boliviensis  0.1999 (0.0273 0.1366) 0.1180 (0.0165 0.1395) 0.1466 (0.0210 0.1430) 0.1860
(0.0296 0.1590) 0.1788 (0.0237 0.1326) 0.1758 (0.0210 0.1193) 0.1607 (0.0205 0.1277)
Callithrix_jacchus   0.1917 (0.0301 0.1568) 0.1211 (0.0187 0.1545) 0.1469 (0.0232 0.1582) 0.1798
(0.0323 0.1798) 0.1694 (0.0264 0.1560) 0.1665 (0.0237 0.1424) 0.1612 (0.0232 0.1442) 0.1129 (0.0080
0.0705)
Tarsius_syrichta     0.1616 (0.0555 0.3433) 0.1311 (0.0442 0.3376) 0.1428 (0.0489 0.3424) 0.1715
(0.0578 0.3372) 0.1490 (0.0524 0.3518) 0.1446 (0.0496 0.3429) 0.1330 (0.0486 0.3656) 0.1388 (0.0448
0.3229) 0.1430 (0.0467 0.3267)
Microcebus_murinus   0.1916 (0.0639 0.3333) 0.1568 (0.0534 0.3407) 0.1656 (0.0572 0.3455) 0.1719
(0.0667 0.3880) 0.1667 (0.0615 0.3691) 0.1692 (0.0587 0.3468) 0.1561 (0.0549 0.3515) 0.1398 (0.0539
0.3851) 0.1458 (0.0567 0.3891) 0.1536 (0.0581 0.3783)
Daubentonia_madagascariensis 0.1723 (0.0506 0.2938) 0.1328 (0.0394 0.2968) 0.1463 (0.0441 0.3013)
0.1601 (0.0525 0.3279) 0.1535 (0.0464 0.3025) 0.1536 (0.0446 0.2902) 0.1386 (0.0408 0.2945) 0.1258
(0.0398 0.3167) 0.1313 (0.0427 0.3247) 0.1513 (0.0473 0.3126) 0.2022 (0.0288 0.1425)
Otolemur_garnettii   0.1351 (0.0619 0.4579) 0.1163 (0.0524 0.4509) 0.1189 (0.0543 0.4566) 0.1462
(0.0652 0.4459) 0.1262 (0.0601 0.4759) 0.1256 (0.0572 0.4552) 0.1227 (0.0553 0.4505) 0.1057 (0.0481
0.4554) 0.1097 (0.0510 0.4649) 0.1315 (0.0609 0.4633) 0.1247 (0.0380 0.3045) 0.1087 (0.0334 0.3073)


TREE #  1:  ((((((1, 2), 3), 4), ((5, 6), 7)), (8, 9)), 10, ((11, 12), 13));   MP score: 518
lnL(ntime: 23  np: 25):  -4800.197653      +0.000000
  14..15   15..16   16..17   17..18   18..19   19..1    19..2    18..3    17..4    16..20   20..21
21..5    21..6    20..7    15..22   22..8    22..9    14..10   14..23   23..24   24..11   24..12
23..13
 0.101416 0.023768 0.035336 0.022254 0.000004 0.028777 0.002048 0.018454 0.065489 0.037564 0.009970
0.025974 0.008254 0.020092 0.035288 0.027167 0.042015 0.187901 0.094903 0.029498 0.119901 0.059224
0.218657 4.200943 0.151989

Note: Branch length is defined as number of nucleotide substitutions per codon (not per neucleotide
site).

tree length =   1.21395

((((((1: 0.028777, 2: 0.002048): 0.000004, 3: 0.018454): 0.022254, 4: 0.065489): 0.035336, ((5:
0.025974, 6: 0.008254): 0.009970, 7: 0.020092): 0.037564): 0.023768, (8: 0.027167, 9: 0.042015):
0.035288): 0.101416, 10: 0.187901, ((11: 0.119901, 12: 0.059224): 0.029498, 13: 0.218657): 0.094903);

((((((Homo_sapiens: 0.028777, Pan_troglodytes: 0.002048): 0.000004, Gorilla_gorilla_gorilla: 0.018454):
0.022254, Pongo_abelii: 0.065489): 0.035336, ((Macaca_mulatta: 0.025974, Papio_anubis: 0.008254):
0.009970, Chlorocebus_sabaeus: 0.020092): 0.037564): 0.023768, (Saimiri_boliviensis: 0.027167,
Callithrix_jacchus: 0.042015): 0.035288): 0.101416, Tarsius_syrichta: 0.187901, ((Microcebus_murinus:
```

0.119901, Daubentonia_madagascariensis: 0.059224): 0.029498, Otolemur_garnettii: 0.218657): 0.094903);

Detailed output identifying parameters

kappa (ts/tv) = 4.20094

w (dN/dS) for branches: 0.15199 1.00000

dN & dS for each branch

branch	t	N	S	dN/dS	dN	dS	N*dN	S*dS
14..15	0.101	1088.6	405.4	0.1520	0.0134	0.0885	14.6	35.9
15..16	0.024	1088.6	405.4	0.1520	0.0032	0.0207	3.4	8.4
16..17	0.035	1088.6	405.4	0.1520	0.0047	0.0308	5.1	12.5
17..18	0.022	1088.6	405.4	0.1520	0.0030	0.0194	3.2	7.9
18..19	0.000	1088.6	405.4	0.1520	0.0000	0.0000	0.0	0.0
19..1	0.029	1088.6	405.4	1.0000	0.0096	0.0096	10.4	3.9
19..2	0.002	1088.6	405.4	0.1520	0.0003	0.0018	0.3	0.7
18..3	0.018	1088.6	405.4	1.0000	0.0062	0.0062	6.7	2.5
17..4	0.065	1088.6	405.4	1.0000	0.0218	0.0218	23.8	8.9
16..20	0.038	1088.6	405.4	1.0000	0.0125	0.0125	13.6	5.1
20..21	0.010	1088.6	405.4	1.0000	0.0033	0.0033	3.6	1.3
21..5	0.026	1088.6	405.4	1.0000	0.0087	0.0087	9.4	3.5
21..6	0.008	1088.6	405.4	1.0000	0.0028	0.0028	3.0	1.1
20..7	0.020	1088.6	405.4	1.0000	0.0067	0.0067	7.3	2.7
15..22	0.035	1088.6	405.4	0.1520	0.0047	0.0308	5.1	12.5
22..8	0.027	1088.6	405.4	0.1520	0.0036	0.0237	3.9	9.6
22..9	0.042	1088.6	405.4	0.1520	0.0056	0.0367	6.1	14.9
14..10	0.188	1088.6	405.4	0.1520	0.0249	0.1639	27.1	66.5
14..23	0.095	1088.6	405.4	0.1520	0.0126	0.0828	13.7	33.6
23..24	0.029	1088.6	405.4	0.1520	0.0039	0.0257	4.3	10.4
24..11	0.120	1088.6	405.4	0.1520	0.0159	0.1046	17.3	42.4
24..12	0.059	1088.6	405.4	0.1520	0.0079	0.0517	8.5	20.9
23..13	0.219	1088.6	405.4	0.1520	0.0290	0.1907	31.6	77.3

tree length for dN: 0.2040
tree length for dS: 0.9433

dS tree:
((((((Homo_sapiens: 0.009592, Pan_troglodytes: 0.001786): 0.000003, Gorilla_gorilla_gorilla: 0.006151): 0.019412, Pongo_abelii: 0.021830): 0.030824, ((Macaca_mulatta: 0.008658, Papio_anubis: 0.002751): 0.003323, Chlorocebus_sabaeus: 0.006697): 0.012521): 0.020733, (Saimiri_boliviensis: 0.023698, Callithrix_jacchus: 0.036650): 0.030782): 0.088466, Tarsius_syrichta: 0.163909, ((Microcebus_murinus: 0.104591, Daubentonia_madagascariensis: 0.051662): 0.025731, Otolemur_garnettii: 0.190738): 0.082785);
dN tree:
((((((Homo_sapiens: 0.009592, Pan_troglodytes: 0.000271): 0.000001, Gorilla_gorilla_gorilla: 0.006151): 0.002950, Pongo_abelii: 0.021830): 0.004685, ((Macaca_mulatta: 0.008658, Papio_anubis: 0.002751): 0.003323, Chlorocebus_sabaeus: 0.006697): 0.012521): 0.003151, (Saimiri_boliviensis: 0.003602, Callithrix_jacchus: 0.005570): 0.004679): 0.013446, Tarsius_syrichta: 0.024912, ((Microcebus_murinus: 0.015897, Daubentonia_madagascariensis: 0.007852): 0.003911, Otolemur_garnettii: 0.028990): 0.012582);

w ratios as labels for TreeView:
((((((Homo_sapiens #1.0000 , Pan_troglodytes #0.1520) #0.1520 , Gorilla_gorilla_gorilla #1.0000) #0.1520 , Pongo_abelii #1.0000) #0.1520 , ((Macaca_mulatta #1.0000 , Papio_anubis #1.0000) #1.0000 , Chlorocebus_sabaeus #1.0000) #1.0000) #0.1520 , (Saimiri_boliviensis #0.1520 , Callithrix_jacchus #0.1520) #0.1520) #0.1520 , Tarsius_syrichta #0.1520 , ((Microcebus_murinus #0.1520 , Daubentonia_madagascariensis #0.1520) #0.1520 , Otolemur_garnettii #0.1520) #0.1520);

Main result file for model K: catarrhines-K.mlc

```
CODONML (in paml version 4.7b, October 2013)  catarrhines.phy
Model: several dN/dS ratios for branches for branches,
Codon frequency model: F3x4
ns =  13  ls = 498

Codon usage in sequences
--------------------------------------------------------------------------------------------------------------------------------------
Phe TTT  13  13  13  14  13  13 | Ser TCT   5   5   5   7   4   4 | Tyr TAT  11  10  11  10   9   8 | Cys TGT   3   4   4   6   5   5
    TTC  15  15  15  13  15  15 |     TCC  11  11  11   9  11  11 |     TAC  16  16  15  15  17  18 |     TGC   8   6   6   7   5   5
Leu TTA   1   1   1   2   2   2 |     TCA   2   2   2   3   2   2 | *** TAA   0   0   0   0   0   0 | *** TGA   0   0   0   0   0   0
    TTG   9   9   9   8   9   9 |     TCG   2   2   2   1   2   2 |     TAG   0   0   0   0   0   0 | Trp TGG   4   4   4   4   4   4
--------------------------------------------------------------------------------------------------------------------------------------
Leu CTT  10   9   9   8   6   6 | Pro CCT  13  14  15  12  10  10 | His CAT   6   6   6   6   6   6 | Arg CGT   4   4   3   3   4   3
    CTC   8   8   8  10   9   9 |     CCC  10  10   9  11  13  14 |     CAC  11  10  10  11  10  10 |     CGC   6   8   8   5   7   8
    CTA   2   2   2   2   2   2 |     CCA   3   3   3   3   2   2 | Gln CAA   5   4   4   4   4   4 |     CGA   3   5   5   5   5   5
    CTG  27  27  27  27  30  30 |     CCG   2   2   2   2   4   3 |     CAG  11  11  12  13  13  13 |     CGG   3   2   2   2   1   1
--------------------------------------------------------------------------------------------------------------------------------------
Ile ATT  10  10  10   9  11  10 | Thr ACT   6   6   6   9   9   9 | Asn AAT  12  11  12  11  14  12 | Ser AGT   2   2   3   2   3   3
    ATC  17  17  18  18  16  17 |     ACC  11  10  10   9   8   9 |     AAC   7   6   6   6   4   6 |     AGC  10  10  10  10   9   9
    ATA   1   1   0   1   1   1 |     ACA   9   9   9   8  11  10 | Lys AAA   3   3   3   3   3   3 | Arg AGA   1   0   0   1   0   0
Met ATG   8   9   9  11   8   8 |     ACG   7   6   6   4   3   3 |     AAG   8   8   8   8   9   8 |     AGG   6   6   6   7   6   6
--------------------------------------------------------------------------------------------------------------------------------------
Val GTT   3   3   3   3   4   5 | Ala GCT  10  10  10  11   9   9 | Asp GAT  17  18  17  17  21  21 | Gly GGT   2   3   3   2   4   4
    GTC  16  15  15  15  15  14 |     GCC   8  10   9   9  12  12 |     GAC  23  24  24  24  20  20 |     GGC  14  14  14  15  13  13
    GTA   2   3   2   3   2   2 |     GCA   2   2   2   2   2   2 | Glu GAA   5   5   5   4   5   4 |     GGA   9   9   9  10   9   9
    GTG  15  14  16  14  15  15 |     GCG   0   0   0   0   0   0 |     GAG  22  22  21  21  20  22 |     GGG   8   9   9   8   8   8
--------------------------------------------------------------------------------------------------------------------------------------


--------------------------------------------------------------------------------------------------------------------------------------
Phe TTT  13  13  12  16  10  12 | Ser TCT   4   3   4   4   4   4 | Tyr TAT   9  12  12  11   5   6 | Cys TGT   4   4   6   7   2   4
    TTC  15  15  15  15  17  14 |     TCC  11  11  11  13  14  14 |     TAC  17  14  14  14  20  19 |     TGC   6   7   5   4  10   8
Leu TTA   2   1   1   0   2   0 |     TCA   2   2   2   2   1   1 | *** TAA   0   0   0   0   0   0 | *** TGA   0   0   0   0   0   0
    TTG   9  11  10   9   5   6 |     TCG   2   2   2   3   2   2 |     TAG   0   0   0   0   0   0 | Trp TGG   4   4   4   4   4   4
--------------------------------------------------------------------------------------------------------------------------------------
Leu CTT   6  10  11   5   4   7 | Pro CCT  10  11  11  12   9   9 | His CAT   6   6   7   8   5   6 | Arg CGT   3   3   2   4   4   2
    CTC   9   9   8  12  12  11 |     CCC  14  13  12  10  15  12 |     CAC  10   9   9   8  12  12 |     CGC   8   7   7   6   9   8
    CTA   3   2   2   4   2   2 |     CCA   2   2   2   4   2   3 | Gln CAA   5   3   4   3   5   3 |     CGA   4   5   5   6   3   5
    CTG  29  25  27  25  30  30 |     CCG   3   2   2   1   3   3 |     CAG  12  14  12  12  12  13 |     CGG   2   1   2   3   3   3
--------------------------------------------------------------------------------------------------------------------------------------
Ile ATT   9  11  10   8   4   5 | Thr ACT   9   9   9   9   6   8 | Asn AAT  12  12  11  11   5   9 | Ser AGT   3   5   5   5   3   3
    ATC  17  17  18  19  22  19 |     ACC   9   7   7  10  14  12 |     AAC   6   5   5   5   9   7 |     AGC   9   7   8   5   8   7
    ATA   1   1   1   4   2   2 |     ACA   9   9  10   5   6   6 | Lys AAA   3   2   2   4   3   2 | Arg AGA   1   0   0   2   2   2
Met ATG   9   9  10  10  11   9 |     ACG   4   5   4   5   6   6 |     AAG   8   7   7  10  10   9 |     AGG   6   7   7   4   3   4
--------------------------------------------------------------------------------------------------------------------------------------
Val GTT   3   7   8   6   4   4 | Ala GCT   9  12  12  10   9   7 | Asp GAT  20  20  20  19  11  17 | Gly GGT   4   4   3   6   3   5
    GTC  16  12   9  10  12  14 |     GCC  11  12  12   9  13  13 |     GAC  21  22  22  24  31  25 |     GGC  14  13  13  14  18  15
    GTA   2   2   2   4   3   2 |     GCA   2   2   2   2   0   3 | Glu GAA   4   5   6  11   5   6 |     GGA   8   8   9   7   6   7
    GTG  15  14  15  13  14  18 |     GCG   0   0   1   0   2   0 |     GAG  21  22  21  14  17  19 |     GGG   9   9   8   8  10  10
--------------------------------------------------------------------------------------------------------------------------------------


------------------------------------------------------
Phe TTT  10 | Ser TCT   3 | Tyr TAT   3 | Cys TGT   5
    TTC  20 |     TCC  12 |     TAC  22 |     TGC   8
Leu TTA   2 |     TCA   2 | *** TAA   0 | *** TGA   0
    TTG   6 |     TCG   1 |     TAG   0 | Trp TGG   4
------------------------------------------------------
Leu CTT   7 | Pro CCT  13 | His CAT   6 | Arg CGT   4
    CTC  10 |     CCC  10 |     CAC  11 |     CGC   7
    CTA   4 |     CCA   2 | Gln CAA   5 |     CGA   3
    CTG  26 |     CCG   2 |     CAG  11 |     CGG   3
------------------------------------------------------
Ile ATT   5 | Thr ACT   7 | Asn AAT   6 | Ser AGT   3
    ATC  20 |     ACC  13 |     AAC  10 |     AGC   7
    ATA   2 |     ACA   6 | Lys AAA   3 | Arg AGA   3
Met ATG  10 |     ACG   4 |     AAG   9 |     AGG   3
------------------------------------------------------
Val GTT   5 | Ala GCT   7 | Asp GAT  11 | Gly GGT   5
    GTC  14 |     GCC  15 |     GAC  28 |     GGC  19
    GTA   4 |     GCA   3 | Glu GAA  11 |     GGA   5
    GTG  14 |     GCG   1 |     GAG  15 |     GGG   8
------------------------------------------------------

Codon position x base (3x4) table for each sequence.

#1: Homo_sapiens
position  1:    T:0.20080    C:0.24900    A:0.23695    G:0.31325
position  2:    T:0.31526    C:0.20281    A:0.31526    G:0.16667
position  3:    T:0.25502    C:0.38353    A:0.09639    G:0.26506
Average         T:0.25703    C:0.27845    A:0.21620    G:0.24833

#2: Pan_troglodytes
position  1:    T:0.19679    C:0.25100    A:0.22892    G:0.32329
position  2:    T:0.31325    C:0.20482    A:0.30924    G:0.17269
position  3:    T:0.25703    C:0.38153    A:0.09839    G:0.26305
Average         T:0.25569    C:0.27912    A:0.21218    G:0.25301
```

```
#3: Gorilla_gorilla_gorilla
position  1:    T:0.19679    C:0.25100    A:0.23293    G:0.31928
position  2:    T:0.31526    C:0.20281    A:0.30924    G:0.17269
position  3:    T:0.26104    C:0.37751    A:0.09438    G:0.26707
Average         T:0.25770    C:0.27711    A:0.21218    G:0.25301

#4: Pongo_abelii
position  1:    T:0.19880    C:0.24900    A:0.23494    G:0.31727
position  2:    T:0.31727    C:0.20080    A:0.30723    G:0.17470
position  3:    T:0.26104    C:0.37550    A:0.10241    G:0.26104
Average         T:0.25904    C:0.27510    A:0.21486    G:0.25100

#5: Macaca_mulatta
position  1:    T:0.19679    C:0.25301    A:0.23092    G:0.31928
position  2:    T:0.31727    C:0.20482    A:0.31124    G:0.16667
position  3:    T:0.26506    C:0.36948    A:0.10040    G:0.26506
Average         T:0.25971    C:0.27577    A:0.21419    G:0.25033

#6: Papio_anubis
position  1:    T:0.19679    C:0.25301    A:0.22892    G:0.32129
position  2:    T:0.31727    C:0.20482    A:0.31124    G:0.16667
position  3:    T:0.25703    C:0.38153    A:0.09639    G:0.26506
Average         T:0.25703    C:0.27979    A:0.21218    G:0.25100

#7: Chlorocebus_sabaeus
position  1:    T:0.19679    C:0.25301    A:0.23092    G:0.31928
position  2:    T:0.31727    C:0.20281    A:0.30924    G:0.17068
position  3:    T:0.24900    C:0.38755    A:0.09639    G:0.26707
Average         T:0.25435    C:0.28112    A:0.21218    G:0.25234

#8: Saimiri_boliviensis
position  1:    T:0.19880    C:0.24498    A:0.22691    G:0.32932
position  2:    T:0.31928    C:0.20482    A:0.30723    G:0.16867
position  3:    T:0.28514    C:0.36145    A:0.08835    G:0.26506
Average         T:0.26774    C:0.27041    A:0.20750    G:0.25435

#9: Callithrix_jacchus
position  1:    T:0.19679    C:0.24699    A:0.22892    G:0.32731
position  2:    T:0.31928    C:0.20683    A:0.30522    G:0.16867
position  3:    T:0.28715    C:0.35141    A:0.09639    G:0.26506
Average         T:0.26774    C:0.26841    A:0.21017    G:0.25368

#10: Tarsius_syrichta
position  1:    T:0.20482    C:0.24699    A:0.23293    G:0.31526
position  2:    T:0.32129    C:0.19880    A:0.30924    G:0.17068
position  3:    T:0.28313    C:0.35743    A:0.11647    G:0.24297
Average         T:0.26975    C:0.26774    A:0.21954    G:0.24297

#11: Microcebus_murinus
position  1:    T:0.19277    C:0.26104    A:0.22892    G:0.31727
position  2:    T:0.30924    C:0.21285    A:0.30120    G:0.17671
position  3:    T:0.17671    C:0.47390    A:0.08434    G:0.26506
Average         T:0.22624    C:0.31593    A:0.20482    G:0.25301

#12: Daubentonia_madagascariensis
position  1:    T:0.18876    C:0.25904    A:0.22088    G:0.33133
position  2:    T:0.31124    C:0.20683    A:0.30723    G:0.17470
position  3:    T:0.21687    C:0.42169    A:0.08835    G:0.27309
Average         T:0.23896    C:0.29585    A:0.20549    G:0.25971

#13: Otolemur_garnettii
position  1:    T:0.19679    C:0.24900    A:0.22289    G:0.33133
position  2:    T:0.31928    C:0.20281    A:0.30321    G:0.17470
position  3:    T:0.20080    C:0.45382    A:0.11044    G:0.23494
Average         T:0.23896    C:0.30187    A:0.21218    G:0.24699

Sums of codon usage counts
------------------------------------------------------------------------------
Phe F TTT     165 | Ser S TCT      56 | Tyr Y TAT     117 | Cys C TGT      59
      TTC     199 |       TCC     150 |       TAC     217 |       TGC      85
Leu L TTA      17 |       TCA      25 | *** * TAA       0 | *** * TGA       0
      TTG     109 |       TCG      25 |       TAG       0 | Trp W TGG      52
------------------------------------------------------------------------------
Leu L CTT      98 | Pro P CCT     149 | His H CAT      80 | Arg R CGT      43
      CTC     123 |       CCC     153 |       CAC     133 |       CGC      94
      CTA      31 |       CCA      33 | Gln Q CAA      53 |       CGA      59
      CTG     360 |       CCG      31 |       CAG     159 |       CGG      28
```

```
--------------------------------------------------------------------------------
Ile I ATT     112 | Thr T ACT     102 | Asn N AAT     138 | Ser S AGT      42
      ATC     235 |       ACC     129 |       AAC      82 |       AGC     109
      ATA      18 |       ACA     107 | Lys K AAA      37 | Arg R AGA      12
Met M ATG     121 |       ACG      63 |       AAG     109 |       AGG      71
--------------------------------------------------------------------------------
Val V GTT      58 | Ala A GCT     125 | Asp D GAT     229 | Gly G GGT      48
      GTC     177 |       GCC     145 |       GAC     308 |       GGC     189
      GTA      33 |       GCA      26 | Glu E GAA      76 |       GGA     105
      GTG     192 |       GCG       4 |       GAG     257 |       GGG     112
--------------------------------------------------------------------------------


Codon position x base (3x4) table, overall

position  1:    T:0.19710    C:0.25131    A:0.22969    G:0.32190
position  2:    T:0.31634    C:0.20436    A:0.30816    G:0.17115
position  3:    T:0.25039    C:0.39049    A:0.09762    G:0.26151
Average         T:0.25461    C:0.28205    A:0.21182    G:0.25152


Nei & Gojobori 1986. dN/dS (dN, dS)
(Note: This matrix is not used in later ML. analysis.
Use runmode = -2 for ML pairwise comparison.)

Homo_sapiens
Pan_troglodytes      1.2604 (0.0106 0.0084)
Gorilla_gorilla_gorilla 1.3389 (0.0151 0.0113) 0.7351 (0.0062 0.0084)
Pongo_abelii         0.4378 (0.0296 0.0676) 0.2910 (0.0187 0.0643) 0.3714 (0.0250 0.0674)
Macaca_mulatta       0.2054 (0.0269 0.1309) 0.1261 (0.0160 0.1271) 0.1712 (0.0224 0.1306) 0.2017
(0.0278 0.1377)
Papio_anubis         0.2052 (0.0242 0.1177) 0.1169 (0.0133 0.1141) 0.1672 (0.0196 0.1174) 0.1773
(0.0232 0.1311) 0.2434 (0.0062 0.0255)
Chlorocebus_sabaeus  0.2404 (0.0260 0.1080) 0.1448 (0.0151 0.1045) 0.1989 (0.0214 0.1078) 0.2216
(0.0269 0.1212) 0.2126 (0.0098 0.0459) 0.2498 (0.0071 0.0284)
Saimiri_boliviensis  0.1999 (0.0273 0.1366) 0.1180 (0.0165 0.1395) 0.1466 (0.0210 0.1430) 0.1860
(0.0296 0.1590) 0.1788 (0.0237 0.1326) 0.1758 (0.0210 0.1193) 0.1607 (0.0205 0.1277)
Callithrix_jacchus   0.1917 (0.0301 0.1568) 0.1211 (0.0187 0.1545) 0.1469 (0.0232 0.1582) 0.1798
(0.0323 0.1798) 0.1694 (0.0264 0.1560) 0.1665 (0.0237 0.1424) 0.1612 (0.0232 0.1442) 0.1129 (0.0080
0.0705)
Tarsius_syrichta     0.1616 (0.0555 0.3433) 0.1311 (0.0442 0.3376) 0.1428 (0.0489 0.3424) 0.1715
(0.0578 0.3372) 0.1490 (0.0524 0.3518) 0.1446 (0.0496 0.3429) 0.1330 (0.0486 0.3656) 0.1388 (0.0448
0.3229) 0.1430 (0.0467 0.3267)
Microcebus_murinus   0.1916 (0.0639 0.3333) 0.1568 (0.0534 0.3407) 0.1656 (0.0572 0.3455) 0.1719
(0.0667 0.3880) 0.1667 (0.0615 0.3691) 0.1692 (0.0587 0.3468) 0.1561 (0.0549 0.3515) 0.1398 (0.0539
0.3851) 0.1458 (0.0567 0.3891) 0.1536 (0.0581 0.3783)
Daubentonia_madagascariensis 0.1723 (0.0506 0.2938) 0.1328 (0.0394 0.2968) 0.1463 (0.0441 0.3013)
0.1601 (0.0525 0.3279) 0.1535 (0.0464 0.3025) 0.1536 (0.0446 0.2902) 0.1386 (0.0408 0.2945) 0.1258
(0.0398 0.3167) 0.1313 (0.0427 0.3247) 0.1513 (0.0473 0.3126) 0.2022 (0.0288 0.1425)
Otolemur_garnettii   0.1351 (0.0619 0.4579) 0.1163 (0.0524 0.4509) 0.1189 (0.0543 0.4566) 0.1462
(0.0652 0.4459) 0.1262 (0.0601 0.4759) 0.1256 (0.0572 0.4552) 0.1227 (0.0553 0.4505) 0.1057 (0.0481
0.4554) 0.1097 (0.0510 0.4649) 0.1315 (0.0609 0.4633) 0.1247 (0.0380 0.3045) 0.1087 (0.0334 0.3073)


TREE #  1:  ((((((1, 2), 3), 4), ((5, 6), 7)), (8, 9)), 10, ((11, 12), 13));   MP score: 518
lnL(ntime: 23  np: 27):  -4788.118330      +0.000000
  14..15   15..16   16..17   17..18   18..19   19..1    19..2    18..3    17..4    16..20   20..21
21..5    21..6    20..7    15..22   22..8    22..9    14..10   14..23   23..24   24..11   24..12
23..13
 0.100709 0.022744 0.033747 0.021643 0.000004 0.028749 0.002050 0.018434 0.066120 0.040750 0.009868
0.025872 0.008184 0.020115 0.035181 0.027404 0.041749 0.186928 0.095169 0.029532 0.119778 0.059173
0.218087 4.034876 0.151936 0.776653 0.240515

Note: Branch length is defined as number of nucleotide substitutions per codon (not per neucleotide
site).

tree length =   1.21199

((((((1: 0.028749, 2: 0.002050): 0.000004, 3: 0.018434): 0.021643, 4: 0.066120): 0.033747, ((5:
0.025872, 6: 0.008184): 0.009868, 7: 0.020115): 0.040750): 0.022744, (8: 0.027404, 9: 0.041749):
0.035181): 0.100709, 10: 0.186928, ((11: 0.119778, 12: 0.059173): 0.029532, 13: 0.218087): 0.095169);

((((((Homo_sapiens: 0.028749, Pan_troglodytes: 0.002050): 0.000004, Gorilla_gorilla_gorilla: 0.018434):
0.021643, Pongo_abelii: 0.066120): 0.033747, ((Macaca_mulatta: 0.025872, Papio_anubis: 0.008184):
0.009868, Chlorocebus_sabaeus: 0.020115): 0.040750): 0.022744, (Saimiri_boliviensis: 0.027404,
Callithrix_jacchus: 0.041749): 0.035181): 0.100709, Tarsius_syrichta: 0.186928, ((Microcebus_murinus:
0.119778, Daubentonia_madagascariensis: 0.059173): 0.029532, Otolemur_garnettii: 0.218087): 0.095169);
```

Detailed output identifying parameters

kappa (ts/tv) = 4.03488

w (dN/dS) for branches: 0.15194 0.77665 0.24051

dN & dS for each branch

branch	t	N	S	dN/dS	dN	dS	N*dN	S*dS
14..15	0.101	1090.8	403.2	0.1519	0.0134	0.0882	14.6	35.5
15..16	0.023	1090.8	403.2	0.1519	0.0030	0.0199	3.3	8.0
16..17	0.034	1090.8	403.2	0.1519	0.0045	0.0295	4.9	11.9
17..18	0.022	1090.8	403.2	0.1519	0.0029	0.0189	3.1	7.6
18..19	0.000	1090.8	403.2	0.1519	0.0000	0.0000	0.0	0.0
19..1	0.029	1090.8	403.2	0.7767	0.0089	0.0115	9.7	4.6
19..2	0.002	1090.8	403.2	0.1519	0.0003	0.0018	0.3	0.7
18..3	0.018	1090.8	403.2	0.7767	0.0057	0.0073	6.2	3.0
17..4	0.066	1090.8	403.2	0.7767	0.0205	0.0263	22.3	10.6
16..20	0.041	1090.8	403.2	0.2405	0.0073	0.0305	8.0	12.3
20..21	0.010	1090.8	403.2	0.2405	0.0018	0.0074	1.9	3.0
21..5	0.026	1090.8	403.2	0.2405	0.0047	0.0194	5.1	7.8
21..6	0.008	1090.8	403.2	0.2405	0.0015	0.0061	1.6	2.5
20..7	0.020	1090.8	403.2	0.2405	0.0036	0.0151	3.9	6.1
15..22	0.035	1090.8	403.2	0.1519	0.0047	0.0308	5.1	12.4
22..8	0.027	1090.8	403.2	0.1519	0.0036	0.0240	4.0	9.7
22..9	0.042	1090.8	403.2	0.1519	0.0056	0.0365	6.1	14.7
14..10	0.187	1090.8	403.2	0.1519	0.0249	0.1636	27.1	66.0
14..23	0.095	1090.8	403.2	0.1519	0.0127	0.0833	13.8	33.6
23..24	0.030	1090.8	403.2	0.1519	0.0039	0.0259	4.3	10.4
24..11	0.120	1090.8	403.2	0.1519	0.0159	0.1049	17.4	42.3
24..12	0.059	1090.8	403.2	0.1519	0.0079	0.0518	8.6	20.9
23..13	0.218	1090.8	403.2	0.1519	0.0290	0.1909	31.6	77.0

tree length for dN: 0.1861
tree length for dS: 0.9936

dS tree:
((((((Homo_sapiens: 0.011450, Pan_troglodytes: 0.001794): 0.000004, Gorilla_gorilla_gorilla: 0.007342): 0.018945, Pongo_abelii: 0.026335): 0.029542, ((Macaca_mulatta: 0.019360, Papio_anubis: 0.006124): 0.007384, Chlorocebus_sabaeus: 0.015052): 0.030493): 0.019909, (Saimiri_boliviensis: 0.023989, Callithrix_jacchus: 0.036546): 0.030797): 0.088159, Tarsius_syrichta: 0.163634, ((Microcebus_murinus: 0.104851, Daubentonia_madagascariensis: 0.051799): 0.025852, Otolemur_garnettii: 0.190910): 0.083309);
dN tree:
((((((Homo_sapiens: 0.008893, Pan_troglodytes: 0.000273): 0.000001, Gorilla_gorilla_gorilla: 0.005702): 0.002878, Pongo_abelii: 0.020453): 0.004488, ((Macaca_mulatta: 0.004656, Papio_anubis: 0.001473): 0.001776, Chlorocebus_sabaeus: 0.003620): 0.007334): 0.003025, (Saimiri_boliviensis: 0.003645, Callithrix_jacchus: 0.005553): 0.004679): 0.013394, Tarsius_syrichta: 0.024862, ((Microcebus_murinus: 0.015931, Daubentonia_madagascariensis: 0.007870): 0.003928, Otolemur_garnettii: 0.029006): 0.012658);

w ratios as labels for TreeView:
((((((Homo_sapiens #0.7767 , Pan_troglodytes #0.1519) #0.1519 , Gorilla_gorilla_gorilla #0.7767) #0.1519 , Pongo_abelii #0.7767) #0.1519 , ((Macaca_mulatta #0.2405 , Papio_anubis #0.2405) #0.2405 , Chlorocebus_sabaeus #0.2405) #0.2405) #0.1519 , (Saimiri_boliviensis #0.1519 , Callithrix_jacchus #0.1519) #0.1519) #0.1519 , Tarsius_syrichta #0.1519 , ((Microcebus_murinus #0.1519 , Daubentonia_madagascariensis #0.1519) #0.1519 , Otolemur_garnettii #0.1519) #0.1519);

Main result file for model L: catarrhines-L.mlc

```
CODONML (in paml version 4.7b, October 2013)  catarrhines.phy
Model: free dN/dS Ratios for branches for branches,
Codon frequency model: F3x4
ns =  13  ls = 498

Codon usage in sequences
--------------------------------------------------------------------------------------------------------------------------------------
Phe TTT  13  13  13  14  13  13 | Ser TCT   5   5   5   7   4   4 | Tyr TAT  11  10  11  10   9   8 | Cys TGT   3   4   4   6   5   5
    TTC  15  15  15  13  15  15 |     TCC  11  11  11   9  11  11 |     TAC  16  16  15  15  17  18 |     TGC   8   6   6   7   5   5
Leu TTA   1   1   1   2   2   2 |     TCA   2   2   2   3   2   2 | *** TAA   0   0   0   0   0   0 | *** TGA   0   0   0   0   0   0
    TTG   9   9   9   8   9   9 |     TCG   2   2   2   1   2   2 |     TAG   0   0   0   0   0   0 | Trp TGG   4   4   4   4   4   4
--------------------------------------------------------------------------------------------------------------------------------------
Leu CTT  10   9   9   8   6   6 | Pro CCT  13  14  15  12  10  10 | His CAT   6   6   6   6   6   6 | Arg CGT   4   4   3   3   4   3
    CTC   8   8   8  10   9   9 |     CCC  10  10   9  11  13  14 |     CAC  11  10  10  11  10  10 |     CGC   6   8   8   5   7   8
    CTA   2   2   2   2   2   2 |     CCA   3   3   3   3   2   2 | Gln CAA   5   4   4   4   4   4 |     CGA   3   5   5   5   5   5
    CTG  27  27  27  27  30  30 |     CCG   2   2   2   2   4   3 |     CAG  11  11  12  13  13  13 |     CGG   3   2   2   2   1   1
--------------------------------------------------------------------------------------------------------------------------------------
Ile ATT  10  10  10   9  11  10 | Thr ACT   6   6   6   9   9   9 | Asn AAT  12  11  12  11  14  12 | Ser AGT   2   2   3   2   3   3
    ATC  17  17  18  18  16  17 |     ACC  11  10  10   9   8   9 |     AAC   7   6   6   6   4   6 |     AGC  10  10  10  10   9   9
    ATA   1   1   0   1   1   1 |     ACA   9   9   9   8  11  10 | Lys AAA   3   3   3   3   3   3 | Arg AGA   1   0   0   1   0   0
Met ATG   8   9   9  11   8   8 |     ACG   7   6   6   4   3   3 |     AAG   8   8   8   8   9   8 |     AGG   6   6   6   7   6   6
--------------------------------------------------------------------------------------------------------------------------------------
Val GTT   3   3   3   3   4   5 | Ala GCT  10  10  10  11   9   9 | Asp GAT  17  18  17  17  21  21 | Gly GGT   2   3   3   2   4   4
    GTC  16  15  15  15  15  14 |     GCC   8  10   9   9  12  12 |     GAC  23  24  24  24  20  20 |     GGC  14  14  14  15  13  13
    GTA   2   3   2   3   2   2 |     GCA   2   2   2   2   2   2 | Glu GAA   5   5   5   4   5   4 |     GGA   9   9   9  10   9   9
    GTG  15  14  16  14  15  15 |     GCG   0   0   0   0   0   0 |     GAG  22  22  21  21  20  22 |     GGG   8   9   9   8   8   8
--------------------------------------------------------------------------------------------------------------------------------------


--------------------------------------------------------------------------------------------------------------------------------------
Phe TTT  13  13  12  16  10  12 | Ser TCT   4   3   4   4   4   4 | Tyr TAT   9  12  12  11   5   6 | Cys TGT   4   4   6   7   2   4
    TTC  15  15  15  15  17  14 |     TCC  11  11  11  13  14  14 |     TAC  17  14  14  14  20  19 |     TGC   6   7   5   4  10   8
Leu TTA   2   1   1   0   2   0 |     TCA   2   2   2   2   1   1 | *** TAA   0   0   0   0   0   0 | *** TGA   0   0   0   0   0   0
    TTG   9  11  10   9   5   6 |     TCG   2   2   2   3   2   2 |     TAG   0   0   0   0   0   0 | Trp TGG   4   4   4   4   4   4
--------------------------------------------------------------------------------------------------------------------------------------
Leu CTT   6  10  11   5   4   7 | Pro CCT  10  11  11  12   9   9 | His CAT   6   6   7   8   5   6 | Arg CGT   3   3   2   4   4   2
    CTC   9   9   8  12  12  11 |     CCC  14  13  12  10  15  12 |     CAC  10   9   9   8  12  12 |     CGC   8   7   7   6   9   8
    CTA   3   2   2   4   2   2 |     CCA   2   2   2   4   2   3 | Gln CAA   5   3   4   3   5   3 |     CGA   4   5   5   6   3   5
    CTG  29  25  27  25  30  30 |     CCG   3   2   2   1   3   3 |     CAG  12  14  12  12  12  13 |     CGG   2   1   2   3   3   3
--------------------------------------------------------------------------------------------------------------------------------------
Ile ATT   9  11  10   8   4   5 | Thr ACT   9   9   9   9   6   8 | Asn AAT  12  12  11  11   5   9 | Ser AGT   3   5   5   5   3   3
    ATC  17  17  18  19  22  19 |     ACC   9   7   7  10  14  12 |     AAC   6   5   5   5   9   7 |     AGC   9   7   8   5   8   7
    ATA   1   1   1   4   2   2 |     ACA   9   9  10   5   6   6 | Lys AAA   3   2   2   4   3   2 | Arg AGA   1   0   0   2   2   2
Met ATG   9   9  10  10  11   9 |     ACG   4   5   4   5   6   6 |     AAG   8   7   7  10  10   9 |     AGG   6   7   7   4   3   4
--------------------------------------------------------------------------------------------------------------------------------------
Val GTT   3   7   8   6   4   4 | Ala GCT   9  12  12  10   9   7 | Asp GAT  20  20  20  19  11  17 | Gly GGT   4   4   3   6   3   5
    GTC  16  12   9  10  12  14 |     GCC  11  12  12   9  13  13 |     GAC  21  22  22  24  31  25 |     GGC  14  13  13  14  18  15
    GTA   2   2   2   4   3   2 |     GCA   2   2   2   2   0   3 | Glu GAA   4   5   6  11   5   6 |     GGA   8   8   9   7   6   7
    GTG  15  14  15  13  14  18 |     GCG   0   0   1   0   2   0 |     GAG  21  22  21  14  17  19 |     GGG   9   9   8   8  10  10
--------------------------------------------------------------------------------------------------------------------------------------


------------------------------------------------------
Phe TTT  10 | Ser TCT   3 | Tyr TAT   3 | Cys TGT   5
    TTC  20 |     TCC  12 |     TAC  22 |     TGC   8
Leu TTA   2 |     TCA   2 | *** TAA   0 | *** TGA   0
    TTG   6 |     TCG   1 |     TAG   0 | Trp TGG   4
------------------------------------------------------
Leu CTT   7 | Pro CCT  13 | His CAT   6 | Arg CGT   4
    CTC  10 |     CCC  10 |     CAC  11 |     CGC   7
    CTA   4 |     CCA   2 | Gln CAA   5 |     CGA   3
    CTG  26 |     CCG   2 |     CAG  11 |     CGG   3
------------------------------------------------------
Ile ATT   5 | Thr ACT   7 | Asn AAT   6 | Ser AGT   3
    ATC  20 |     ACC  13 |     AAC  10 |     AGC   7
    ATA   2 |     ACA   6 | Lys AAA   3 | Arg AGA   3
Met ATG  10 |     ACG   4 |     AAG   9 |     AGG   3
------------------------------------------------------
Val GTT   5 | Ala GCT   7 | Asp GAT  11 | Gly GGT   5
    GTC  14 |     GCC  15 |     GAC  28 |     GGC  19
    GTA   4 |     GCA   3 | Glu GAA  11 |     GGA   5
    GTG  14 |     GCG   1 |     GAG  15 |     GGG   8
------------------------------------------------------

Codon position x base (3x4) table for each sequence.

#1: Homo_sapiens
position  1:    T:0.20080    C:0.24900    A:0.23695    G:0.31325
position  2:    T:0.31526    C:0.20281    A:0.31526    G:0.16667
position  3:    T:0.25502    C:0.38353    A:0.09639    G:0.26506
Average         T:0.25703    C:0.27845    A:0.21620    G:0.24833

#2: Pan_troglodytes
position  1:    T:0.19679    C:0.25100    A:0.22892    G:0.32329
position  2:    T:0.31325    C:0.20482    A:0.30924    G:0.17269
position  3:    T:0.25703    C:0.38153    A:0.09839    G:0.26305
Average         T:0.25569    C:0.27912    A:0.21218    G:0.25301
```

```
#3: Gorilla_gorilla_gorilla
position  1:    T:0.19679    C:0.25100    A:0.23293    G:0.31928
position  2:    T:0.31526    C:0.20281    A:0.30924    G:0.17269
position  3:    T:0.26104    C:0.37751    A:0.09438    G:0.26707
Average         T:0.25770    C:0.27711    A:0.21218    G:0.25301

#4: Pongo_abelii
position  1:    T:0.19880    C:0.24900    A:0.23494    G:0.31727
position  2:    T:0.31727    C:0.20080    A:0.30723    G:0.17470
position  3:    T:0.26104    C:0.37550    A:0.10241    G:0.26104
Average         T:0.25904    C:0.27510    A:0.21486    G:0.25100

#5: Macaca_mulatta
position  1:    T:0.19679    C:0.25301    A:0.23092    G:0.31928
position  2:    T:0.31727    C:0.20482    A:0.31124    G:0.16667
position  3:    T:0.26506    C:0.36948    A:0.10040    G:0.26506
Average         T:0.25971    C:0.27577    A:0.21419    G:0.25033

#6: Papio_anubis
position  1:    T:0.19679    C:0.25301    A:0.22892    G:0.32129
position  2:    T:0.31727    C:0.20482    A:0.31124    G:0.16667
position  3:    T:0.25703    C:0.38153    A:0.09639    G:0.26506
Average         T:0.25703    C:0.27979    A:0.21218    G:0.25100

#7: Chlorocebus_sabaeus
position  1:    T:0.19679    C:0.25301    A:0.23092    G:0.31928
position  2:    T:0.31727    C:0.20281    A:0.30924    G:0.17068
position  3:    T:0.24900    C:0.38755    A:0.09639    G:0.26707
Average         T:0.25435    C:0.28112    A:0.21218    G:0.25234

#8: Saimiri_boliviensis
position  1:    T:0.19880    C:0.24498    A:0.22691    G:0.32932
position  2:    T:0.31928    C:0.20482    A:0.30723    G:0.16867
position  3:    T:0.28514    C:0.36145    A:0.08835    G:0.26506
Average         T:0.26774    C:0.27041    A:0.20750    G:0.25435

#9: Callithrix_jacchus
position  1:    T:0.19679    C:0.24699    A:0.22892    G:0.32731
position  2:    T:0.31928    C:0.20683    A:0.30522    G:0.16867
position  3:    T:0.28715    C:0.35141    A:0.09639    G:0.26506
Average         T:0.26774    C:0.26841    A:0.21017    G:0.25368

#10: Tarsius_syrichta
position  1:    T:0.20482    C:0.24699    A:0.23293    G:0.31526
position  2:    T:0.32129    C:0.19880    A:0.30924    G:0.17068
position  3:    T:0.28313    C:0.35743    A:0.11647    G:0.24297
Average         T:0.26975    C:0.26774    A:0.21954    G:0.24297

#11: Microcebus_murinus
position  1:    T:0.19277    C:0.26104    A:0.22892    G:0.31727
position  2:    T:0.30924    C:0.21285    A:0.30120    G:0.17671
position  3:    T:0.17671    C:0.47390    A:0.08434    G:0.26506
Average         T:0.22624    C:0.31593    A:0.20482    G:0.25301

#12: Daubentonia_madagascariensis
position  1:    T:0.18876    C:0.25904    A:0.22088    G:0.33133
position  2:    T:0.31124    C:0.20683    A:0.30723    G:0.17470
position  3:    T:0.21687    C:0.42169    A:0.08835    G:0.27309
Average         T:0.23896    C:0.29585    A:0.20549    G:0.25971

#13: Otolemur_garnettii
position  1:    T:0.19679    C:0.24900    A:0.22289    G:0.33133
position  2:    T:0.31928    C:0.20281    A:0.30321    G:0.17470
position  3:    T:0.20080    C:0.45382    A:0.11044    G:0.23494
Average         T:0.23896    C:0.30187    A:0.21218    G:0.24699

Sums of codon usage counts
------------------------------------------------------------------------------
Phe F TTT     165 | Ser S TCT      56 | Tyr Y TAT     117 | Cys C TGT      59
      TTC     199 |       TCC     150 |       TAC     217 |       TGC      85
Leu L TTA      17 |       TCA      25 | *** * TAA       0 | *** * TGA       0
      TTG     109 |       TCG      25 |       TAG       0 | Trp W TGG      52
------------------------------------------------------------------------------
Leu L CTT      98 | Pro P CCT     149 | His H CAT      80 | Arg R CGT      43
      CTC     123 |       CCC     153 |       CAC     133 |       CGC      94
      CTA      31 |       CCA      33 | Gln Q CAA      53 |       CGA      59
      CTG     360 |       CCG      31 |       CAG     159 |       CGG      28
```

```
------------------------------------------------------------------------------
Ile I ATT     112 | Thr T ACT     102 | Asn N AAT     138 | Ser S AGT      42
      ATC     235 |       ACC     129 |       AAC      82 |       AGC     109
      ATA      18 |       ACA     107 | Lys K AAA      37 | Arg R AGA      12
Met M ATG     121 |       ACG      63 |       AAG     109 |       AGG      71
------------------------------------------------------------------------------
Val V GTT      58 | Ala A GCT     125 | Asp D GAT     229 | Gly G GGT      48
      GTC     177 |       GCC     145 |       GAC     308 |       GGC     189
      GTA      33 |       GCA      26 | Glu E GAA      76 |       GGA     105
      GTG     192 |       GCG       4 |       GAG     257 |       GGG     112
------------------------------------------------------------------------------


Codon position x base (3x4) table, overall

position  1:    T:0.19710    C:0.25131    A:0.22969    G:0.32190
position  2:    T:0.31634    C:0.20436    A:0.30816    G:0.17115
position  3:    T:0.25039    C:0.39049    A:0.09762    G:0.26151
Average         T:0.25461    C:0.28205    A:0.21182    G:0.25152


Nei & Gojobori 1986. dN/dS (dN, dS)
(Note: This matrix is not used in later ML. analysis.
Use runmode = -2 for ML pairwise comparison.)

Homo_sapiens
Pan_troglodytes      1.2604 (0.0106 0.0084)
Gorilla_gorilla_gorilla 1.3389 (0.0151 0.0113) 0.7351 (0.0062 0.0084)
Pongo_abelii         0.4378 (0.0296 0.0676) 0.2910 (0.0187 0.0643) 0.3714 (0.0250 0.0674)
Macaca_mulatta       0.2054 (0.0269 0.1309) 0.1261 (0.0160 0.1271) 0.1712 (0.0224 0.1306) 0.2017
(0.0278 0.1377)
Papio_anubis         0.2052 (0.0242 0.1177) 0.1169 (0.0133 0.1141) 0.1672 (0.0196 0.1174) 0.1773
(0.0232 0.1311) 0.2434 (0.0062 0.0255)
Chlorocebus_sabaeus  0.2404 (0.0260 0.1080) 0.1448 (0.0151 0.1045) 0.1989 (0.0214 0.1078) 0.2216
(0.0269 0.1212) 0.2126 (0.0098 0.0459) 0.2498 (0.0071 0.0284)
Saimiri_boliviensis  0.1999 (0.0273 0.1366) 0.1180 (0.0165 0.1395) 0.1466 (0.0210 0.1430) 0.1860
(0.0296 0.1590) 0.1788 (0.0237 0.1326) 0.1758 (0.0210 0.1193) 0.1607 (0.0205 0.1277)
Callithrix_jacchus   0.1917 (0.0301 0.1568) 0.1211 (0.0187 0.1545) 0.1469 (0.0232 0.1582) 0.1798
(0.0323 0.1798) 0.1694 (0.0264 0.1560) 0.1665 (0.0237 0.1424) 0.1612 (0.0232 0.1442) 0.1129 (0.0080
0.0705)
Tarsius_syrichta     0.1616 (0.0555 0.3433) 0.1311 (0.0442 0.3376) 0.1428 (0.0489 0.3424) 0.1715
(0.0578 0.3372) 0.1490 (0.0524 0.3518) 0.1446 (0.0496 0.3429) 0.1330 (0.0486 0.3656) 0.1388 (0.0448
0.3229) 0.1430 (0.0467 0.3267)
Microcebus_murinus   0.1916 (0.0639 0.3333) 0.1568 (0.0534 0.3407) 0.1656 (0.0572 0.3455) 0.1719
(0.0667 0.3880) 0.1667 (0.0615 0.3691) 0.1692 (0.0587 0.3468) 0.1561 (0.0549 0.3515) 0.1398 (0.0539
0.3851) 0.1458 (0.0567 0.3891) 0.1536 (0.0581 0.3783)
Daubentonia_madagascariensis 0.1723 (0.0506 0.2938) 0.1328 (0.0394 0.2968) 0.1463 (0.0441 0.3013)
0.1601 (0.0525 0.3279) 0.1535 (0.0464 0.3025) 0.1536 (0.0446 0.2902) 0.1386 (0.0408 0.2945) 0.1258
(0.0398 0.3167) 0.1313 (0.0427 0.3247) 0.1513 (0.0473 0.3126) 0.2022 (0.0288 0.1425)
Otolemur_garnettii   0.1351 (0.0619 0.4579) 0.1163 (0.0524 0.4509) 0.1189 (0.0543 0.4566) 0.1462
(0.0652 0.4459) 0.1262 (0.0601 0.4759) 0.1256 (0.0572 0.4552) 0.1227 (0.0553 0.4505) 0.1057 (0.0481
0.4554) 0.1097 (0.0510 0.4649) 0.1315 (0.0609 0.4633) 0.1247 (0.0380 0.3045) 0.1087 (0.0334 0.3073)


TREE #  1:  ((((((1, 2), 3), 4), ((5, 6), 7)), (8, 9)), 10, ((11, 12), 13));   MP score: 518
lnL(ntime: 23  np: 47):  -4780.198190      +0.000000
  14..15   15..16   16..17   17..18   18..19   19..1    19..2    18..3    17..4    16..20   20..21
21..5    21..6    20..7    15..22   22..8    22..9    14..10   14..23   23..24   24..11   24..12
23..13
 0.103636 0.022857 0.033886 0.021146 0.000004 0.028861 0.002022 0.018485 0.066757 0.041095 0.010001
0.026010 0.007819 0.020087 0.034484 0.027770 0.041576 0.185251 0.091891 0.032861 0.117152 0.057541
0.222249 4.041988 0.126834 0.206700 0.052288 0.156677 2.433962 2.265640 0.000100 1.317366 0.471091
0.199092 0.100282 0.245181 0.414118 0.383765 0.211128 0.106043 0.159115 0.168745 0.204496 0.067548
0.236967 0.184192 0.113921

Note: Branch length is defined as number of nucleotide substitutions per codon (not per neucleotide
site).

tree length =   1.21344

((((((1: 0.028861, 2: 0.002022): 0.000004, 3: 0.018485): 0.021146, 4: 0.066757): 0.033886, ((5:
0.026010, 6: 0.007819): 0.010001, 7: 0.020087): 0.041095): 0.022857, (8: 0.027770, 9: 0.041576):
0.034484): 0.103636, 10: 0.185251, ((11: 0.117152, 12: 0.057541): 0.032861, 13: 0.222249): 0.091891);

((((((Homo_sapiens: 0.028861, Pan_troglodytes: 0.002022): 0.000004, Gorilla_gorilla_gorilla: 0.018485):
0.021146, Pongo_abelii: 0.066757): 0.033886, ((Macaca_mulatta: 0.026010, Papio_anubis: 0.007819):
0.010001, Chlorocebus_sabaeus: 0.020087): 0.041095): 0.022857, (Saimiri_boliviensis: 0.027770,
```

Callithrix_jacchus: 0.041576): 0.034484): 0.103636, Tarsius_syrichta: 0.185251, ((Microcebus_murinus: 0.117152, Daubentonia_madagascariensis: 0.057541): 0.032861, Otolemur_garnettii: 0.222249): 0.091891);

Detailed output identifying parameters

kappa (ts/tv) = 4.04199

w (dN/dS) for branches: 0.12683 0.20670 0.05229 0.15668 2.43396 2.26564 0.00010 1.31737 0.47109 0.19909 0.10028 0.24518 0.41412 0.38377 0.21113 0.10604 0.15911 0.16875 0.20450 0.06755 0.23697 0.18419 0.11392

dN & dS for each branch

branch	t	N	S	dN/dS	dN	dS	N*dN	S*dS
14..15	0.104	1090.7	403.3	0.1268	0.0121	0.0953	13.2	38.4
15..16	0.023	1090.7	403.3	0.2067	0.0037	0.0181	4.1	7.3
16..17	0.034	1090.7	403.3	0.0523	0.0019	0.0367	2.1	14.8
17..18	0.021	1090.7	403.3	0.1567	0.0029	0.0183	3.1	7.4
18..19	0.000	1090.7	403.3	2.4340	0.0000	0.0000	0.0	0.0
19..1	0.029	1090.7	403.3	2.2656	0.0113	0.0050	12.4	2.0
19..2	0.002	1090.7	403.3	0.0001	0.0000	0.0025	0.0	1.0
18..3	0.018	1090.7	403.3	1.3174	0.0066	0.0050	7.2	2.0
17..4	0.067	1090.7	403.3	0.4711	0.0171	0.0363	18.6	14.6
16..20	0.041	1090.7	403.3	0.1991	0.0066	0.0330	7.2	13.3
20..21	0.010	1090.7	403.3	0.1003	0.0010	0.0097	1.1	3.9
21..5	0.026	1090.7	403.3	0.2452	0.0047	0.0193	5.2	7.8
21..6	0.008	1090.7	403.3	0.4141	0.0019	0.0046	2.1	1.8
20..7	0.020	1090.7	403.3	0.3838	0.0047	0.0122	5.1	4.9
15..22	0.034	1090.7	403.3	0.2111	0.0057	0.0271	6.2	10.9
22..8	0.028	1090.7	403.3	0.1060	0.0028	0.0266	3.1	10.7
22..9	0.042	1090.7	403.3	0.1591	0.0057	0.0359	6.2	14.5
14..10	0.185	1090.7	403.3	0.1687	0.0265	0.1571	28.9	63.3
14..23	0.092	1090.7	403.3	0.2045	0.0149	0.0731	16.3	29.5
23..24	0.033	1090.7	403.3	0.0675	0.0023	0.0343	2.5	13.8
24..11	0.117	1090.7	403.3	0.2370	0.0209	0.0882	22.8	35.6
24..12	0.058	1090.7	403.3	0.1842	0.0087	0.0474	9.5	19.1
23..13	0.222	1090.7	403.3	0.1139	0.0239	0.2098	26.1	84.6

tree length for dN: 0.1860
tree length for dS: 0.9954

dS tree:
((((((Homo_sapiens: 0.005000, Pan_troglodytes: 0.002497): 0.000001, Gorilla_gorilla_gorilla: 0.005002): 0.018342, Pongo_abelii: 0.036251): 0.036662, ((Macaca_mulatta: 0.019313, Papio_anubis: 0.004554): 0.009715, Chlorocebus_sabaeus: 0.012172): 0.032986): 0.018105, (Saimiri_boliviensis: 0.026650, Callithrix_jacchus: 0.035896): 0.027107): 0.095293, Tarsius_syrichta: 0.157080, ((Microcebus_murinus: 0.088166, Daubentonia_madagascariensis: 0.047430): 0.034312, Otolemur_garnettii: 0.209814): 0.073065);
dN tree:
((((((Homo_sapiens: 0.011328, Pan_troglodytes: 0.000000): 0.000002, Gorilla_gorilla_gorilla: 0.006590): 0.002874, Pongo_abelii: 0.017077): 0.001917, ((Macaca_mulatta: 0.004735, Papio_anubis: 0.001886): 0.000974, Chlorocebus_sabaeus: 0.004671): 0.006567): 0.003742, (Saimiri_boliviensis: 0.002826, Callithrix_jacchus: 0.005712): 0.005723): 0.012086, Tarsius_syrichta: 0.026507, ((Microcebus_murinus: 0.020892, Daubentonia_madagascariensis: 0.008736): 0.002318, Otolemur_garnettii: 0.023902): 0.014942);

w ratios as labels for TreeView:
((((((Homo_sapiens #2.2656 , Pan_troglodytes #0.0001) #2.4340 , Gorilla_gorilla_gorilla #1.3174) #0.1567 , Pongo_abelii #0.4711) #0.0523 , ((Macaca_mulatta #0.2452 , Papio_anubis #0.4141) #0.1003 , Chlorocebus_sabaeus #0.3838) #0.1991) #0.2067 , (Saimiri_boliviensis #0.1060 , Callithrix_jacchus #0.1591) #0.2111) #0.1268 , Tarsius_syrichta #0.1687 , ((Microcebus_murinus #0.2370 , Daubentonia_madagascariensis #0.1842) #0.0675 , Otolemur_garnettii #0.1139) #0.2045);
