# Supplementary material for: Frequent Loss and Alteration of the MOXD2 Gene in Catarrhines and Whales: A Possible Connection with the Evolution of Olfaction
Source: PLoS One. 2014 Aug 7;9(8):e104085. doi: 10.1371/journal.pone.0104085 (PMC4125168; doi:10.1371/journal.pone.0104085)
Supplement: Data S3 — The sequence data file, tree files, control files, and main result files for the codeml analyses of whale MOXD2 gene. (PDF) [file pone.0104085.s006.pdf]

Data S3. Files used to perform the codeml analyses to generate Tables 4 and 5.

Sequence data file: whales.phy

```
8 1791

Orcinus_orca
ATG GCC TGT GCC CTT CTC TTT CGG CTT CTC CTA CTT ATG GCC CTG CTG ACC TCC TCT CAA GGC AAC
CAC CTG GGC CTC ACA TTA TGT CTG CAT TAT TCC ATG TTC CTA GAT CCT TCT AAT GTT ACT TTC CTG
CAC GAC TTT GAC CTT GAG GCT GAG ATC GTC ACT TTT GAT CTC CAG GTC CAG ACA GCT GGC TGG GTG
GGC TTG GGT ATC ACA AAT CGC TAC ACC ATG GTG GGA AGC AAT CTG GTT GTT GGA GGA GTC TCG CCG
GAT GGC AAT GTC TAT TTC TCG AAT CAG CAC CTG GTG GAT GAA GAC GCT CTG GAG GAG AGC CAG AAC
GCA GAG CTG CAG GCA CTG ACA GAA GAC ACC ATC TAT ACC ACC ATG CGC TTC TCC AGG CCC TTC TGC
TCC TGT GAC CCT CAA GAC GAC ATC ACG AGT GAC ACT GTG AGG GTG CTT GCC ACC TAC GGC CCA GAT
GAC ACT CTG AAG CTG GAT CGG GAG CGT ACT TTT GTG TCC ATC CAA ATT GTC CAC CCC GAT GAT CTC
GAT GTC CCC GAG GAC ACC ATC ATC CAT GAC TTG GAG ATC ACT GAT TTC TTC ATC CCA GAG GAT GAC
ACC ACG TAC GCC TGC ACC TTC CTC CCT CTC TCC ATC GTT AGC AAG AAG CAC CGT ATC TAC AAG TTT
GAG CCC AAG TTG GTC CAT CAC AAG GAG ACG ATG GTG CAC CAC ATC CTG GTG TAC GCC TGC AGC AAA
ACC AGT GCT CTG CCC AAG GGC ATC AGC GAC TGC TAC GGG GCT GAC CCC GCC TCC TCC CTC TGC TCA
CAG GTC ATC GTG GGC TGG GCT ATC GGG GAC ACA AGT TAC CAG TTT CCA GGT GAC GTG GGT ATC TCT
ACT GGG ATG GCT TTG GAC CCC CAG TGG GTC CTG GAG ATT CAC TAC AGC AAT TTG CAC AGT CTT CCT
GGT GTG TAC GGC TCC TCG GGG ATT GTG TAC TAC ACG GCG CAG CTA CGC AAA TAT GAC ATG GGT GTC
CTG CAG CTG GGC TTC TGC ACT TTG CCC ATC CAC TTC GTA CCC CCG GGT GCC GAG TCC TTC GCG TCC
TAT GGG CTG TGT AAG GCC GAG AAG TTT GAT GAG ATG AAC GGG GCC CCG GTG CCT GAC ATC CAG GTC
TGC GGC TAC CTG CTC CAC ACC CAC TTG GCT GGC CGC ACT CTG CAG GCC GTG CAA TAC AGA AAT GGA
ACA CAA CGC CAA ACA ATC TGG AAA GAT GAT ACC GAC TTG AAT CTG CAG GAG ACT GAT TTA CCT TCT
GTG GCC ATC AAG CCA GGA GAT GAA TTG TTG GTA GGG TGT CAC CAT CAG ACG CTG GAC CAC GAC TCC
TTG ACT TTT GGG GGT CCC AGC ACC GTT AAT GAG ATG TGC CTC ATC TTT CTC TTC TAC TAT ATC AAC
AAC ATC TCC AGC TGC ATG GGG GCT GAC ATC ATC TAC GTG GCC CAT GTG CTG GGG GAG GAG GCA TCA
GAT TCT ATG GAG GGC ATG ATG GCC ATG AAC AAT GTG GAG TGG ACT CCG GAG AAC ATT AAA AAG GCT
GAG AAG GCC TGC GAG GAG GCC CAG CAG ATG GTG CTA ATA AAG ACC ATT GAC GAG CTA GTG GAA AAC
AGA ACA GGC TGG ATT CTG GAA ATC ATC CCT GCT CCC CGG GGT CCT TGC TTA GAG TCC TCC AGA CCC
CAG GAC AGA ACC CCT GTA GGC TTC AGG GCT GCC CCC ATG GCC CTC TCG GGC TCC AGC ACT GCC ACA
CCA AGG CGC CCC CTG ACT GCC CTC TTG TTT GGG CAG GGG GCC GTG TCT TGG CTC CTA GCC ACC CTG
GCT GGA GCC
Tursiops_truncatus
... ... ... ... ... ... ... ... ... ... ... ... .C. ... ... .C. ... ... ... ... ... ...
... ... ... ... ... .C. C.. ... .G. ... ... ... ... ... ... ... ... ... ... ... ... ...
... ... ... ... ... ... ... ... G.. ... ... ... ..G ... ... ... ... ... ... ... ... ...
... ... ... ... ... ... A.. ... ... ... ... ... ... ... ... ... ... ... ... ... ... ...
..C ... ... ... ... ... ... ... ... ... ... ... ... ... ... ... ... ... ... ... ... G..
... ... ... ... ... ... ... ... ... ... ... ... ... ... ... T.. ... ... ... ... ... ...
... ... ... ... ... ... ... ... ... ... ... ... ... ... ... ... ... ... ... ... ... ...
... ... ... ... ... ... ... ... ... ... ... ... ... ... ... ... ... ... ... ... ... ..T
... ... ... ... ... ... ... ... ... ... ... ... ... ... ... ... ... ... ... ... ... ...
... ... ... ... ... ... ... ... ... ... ... ... ... ... ... ... ... ... ... ... ... ...
... ... ... ... ... ... ... ... ... ... .C. ... ... ... ... ... ... ... ... ... G.. ..C
... ... ... ... ... ... ... ... ... ... ... ... ... ..C ... ... ... ... ... ... ... ...
... ... ... ... ... ... ... G.. ... ... ... ... ... ... ... ... ... ... ... ... ... ...
... ... ... ... ... ... ... ... ... ... ... ... ... ... ... ... .G. ... ... ... ... ...
... ..A ... ... ... ... ... ... ... ... ... ... ... ... ... ... ... ... ... ... ... ...
... ... ... ... ... ... ... ... ... ... ... ... A.. ... ... A.. ... ... ... ... ... ...
... ... ... ... ... ..G ... ... ... ..C ... ... ... ... ... ... ... ... ... ... ... ...
... ... ... ... T.. ... ... ... ... ... ... ... ... ... ... ... ... ... ... ... ... ...
... ... ... ... ... ... ... ... ... ... ... ... ... ... ... ... ... ... ... ... ... ...
... ... ... ... ... ... ... ... ... ... ... ... ... ... T.. ... ... ... ... ... ... ...
... ... ... ... ... ... ... ... ... ... ... ... ... ... ... ... ... ... ... ... ... ...
... ... ... ... ... ... ... ... ... ... ... ... ... ... ... ... ... ... ... ... ... ...
... ..C ... ... ... ... ... ... ... ... ... ... ..A ... ... ... ... ... ... ... ... ...
... ... ... ... ... ... ... ... ... ... ... A.. ... ... ... ... ... ..A ... ... ... ...
... ... ... ... ... .C. .G. ... ... ... ... ... ... ... ... ... ... ... ... ... ... ...
... ... ... ... ... ... ... ... ... ... ... ... ... ... ... ... ... ... ... G.. ... ...
... ... ... ... .C. ... ... ... ... ... ... ... ... ... ... ... ... ... ... ... ... ...
... ... ...
Neophocaena_phocaenoides
... ... ... ... ... ... ... ... ... ... ... ... ... ... ... ... ... ... ... ... ... ...
... ... ... ... ... .C. C.. ... .G. ... ... ... ... ... ... ... ... ... ... .T. ... ...
... ... ... ... ... ... ... ... ... A.. ... ... ..G ... ... ... ... ... ... ... ... ...
... ... ... ... ... ... ... ... ... ... ... ... ... ... ... ... ... ... ... ... ... ..A
..C ... ... ... ... ... ... ... ... ... ... ... ... ... ... ... ... .G. ... ... ... G..
... ... ... .T. ... ... ... ... ... ... ... ... ... ... ... ... .C. ... ... ... ... C..
... ..C ... ... ... ... ... ... ... ... ... ... ... ... ... ... ... ... ... ... ... ...
... ... ... ... ... ... ... ... ... ... ... ... ... ... ..C ... ... ... ... ... ... ...
... ... ... ... ... ... ... G.. ... ... ... ... ... ... ... ... ... ... ... ... ... ...
... ... ... ... ... C.. ... ... ... ... ... ... ... G.. ... ... ... ... ... .C. ... ...
... ... ... ... ... ... ... ... .G. ... .C. ... ... ... ... ... ... ... ... ..G G.. ..C
```

... ...
... ... G.. ... ...
... ...
... ...
... ... A.. ... ... A.. ...
... ...A ... ...C ... ...
... A.. ... ... G.. ... ...
... ... T.. .G. ... ... T.. ... ...
... ... T.C ... ... .GT ... ...
... ... .A. ... ...
... ...C ... ...
... ..C ... ...A ... ... .T. ... ...
... ... A.. ... ...T ... ...
.C. ... ... .C. ... ...
... ...
... ...
... ...
Lipotes_vexillifer
G.. ... ... A.. ... ... .C. ... ... .C. ... C.. ... ...
... ... .C. ... ... .G. ... ... .CA ... ...C .T. ... ...
... ... A.. ... .G ... ...
... ... .A. ... ... .GA ... ... A.. ... ...
..C ... ... C.. ... ... G.T
..G ... ... CA.
... ...T ... ...
... ... T.. ... ... .C. ... .T ... ...
... ... C.. T.T ... ...
... ... .A. ... ...
... ...T G.. ..C
G.. ... ...A ... ...C A.. ... ...G
... .T ... ... C.. G.. A.. .G. ... ...
... ... G.. ... ...
... ...G T.. ... C.. ... ...
... ...C T.. ... ... A.. ... .C ... ...A ...
... ... A.G ... ...A ... ... T.. ... ... .T. ... ... .G. ... ...
... ... A.. ... ...
... ... .G. ... .T ... ... T.. ... ..C ... ...
... ...G ... ... C.. ... ... T.C ... ... .G. ..T ...
... ..C ... ... .G. CC. ...
... ... C.. ... ...
... ..C ... C.. ... ...
... ... .G. ..T ... ... .C. ... A.. ... ...
.C. ... ...
... ... G.. ... ...
... ... A.. ... ... T.. ... .G ...
... ...
Balaenoptera_acutorostrata
... ... T.. ... ... .C. ... ... .C. ... ... .G. .G. ... ...
... ... .C. ... .CG C.. ... .G. ... ... .C. ... ...C .T. ... ...
... ... A.. ... .G ... ... .G. ... ...
... ... .GA ... ... G.. ... ...
..C ... ... G.. G.. ... .A ..C ... ... G..
..G ... ... .T. ... ...T G.. ... ...G ... ... C..
... ...T ... ... G.. ... ...
.C. ... ... T.. ... .A. ... ... A.. ... ...
... ... C.. ... ...
... .T ... ... C.. ... ... .A. ... ...
... ... G.. .G. ... ... G.. ..G
G.. ... .C ... ...T ... ..C C.. ... ... .T. ... ...G
... ... G.. ... .G. ... ...G ... ...
.T. ... .C. C.. ... ...C ... ..C ... ...
... ... .A. ... ... CT. ... ...G ... ...
... ... .T. ... ..C ... ... A.. ... .C ... ...
..C ... ... A.G ... ...A ... ... A.. ... ...
... ...
... ... .T. .G. ... .T ... C.. ... T.. ... ..C ... ...
... ...G ... ... C.. ... .A. ... ... T.C ... ... .G. ... ...
... ... T.. ... ... CC. ...
... ... C.. ... ... .A. ... ...
... ..C ... ..A ... ...C ... ...
... ... A.. ... ... .C. ... A.. ... ...T ... ...
.C. ... ... .C. ... ...G ... ...
... ... .C. ... ...G ... ...A ... ...
... ...
... ...
Balaenoptera_physalus
... ...T ... ... .C. ... ... .C. ... ...

... T.. ... .C. ... .CG C.. ... .G. ... ... .CA ... ...C .T. ... ...
... ... A.. ... .G ... ... .G. ... ...
... ... .GA ... ... G.. ... ...
..C ... ... G.. G.. ... .A ..C ... ..T ... ... G..
..G ... ... .T. ... ... A.. G.. ... ... .A. ... ... C..
... ...T ... ...T G.. ..T ... ...
.C. ... ... T.. ... T.. ... ...
... ... C.. ... ...
... .T ... ... C.. ... A.. ... ... .A. ... ...
... ... G.. .G. ... ... .A. ..G
G.. ... .C ... ...C C.. ... ... .T. ... ...G
... ... T.. ... ... G.. ... .G. ... ...T ... ...
.T. ... .C. C.. ... ... A.. ... ... C.C ... ..C ... ...
.A. ... ... .A. ... ... CT. ... ...G ... ...
... ... .T. ... ..C ... ... A.. ... .C ... ...
..C ... ... AAG ... C.. ... ..A ... ...
... ... .T. ... ...
... ... .T. .G. ... .T ... ... T.. ... ..C ... ...
... ... .TG ... ... C.. ... .A. ... ... T.C ... ... .G. ... ...
... ... A.. ... ... CC. ...
... ... A.. ... ... .A. ... ...
..C ..C ... .T ... ... .C. ... ..G ... ...C ... ...C ... ...
... .G ... A.. ... ... .C. ... A.. ... ...
.C. ... ...G ... ... AA.
... ... .C. ... ...G ... ...
... .G ... ...T ... ...
... ...
Bos_taurus
... ... .CC T.. ... ... T.. .C. ... ..T GCA ..T C.. ..C ... T.. ..T
.G. ..T ... .C. .TG CCG C.. ... .G. ... ... .G. ..T ... ..C ... .C .C. GT. ... ...
.G. ... ... T.. ... ... A.. ... ..C ..G ... ... .G. ... ...
..T ... ... G.. ... G.. ... ... .G. .GA .C. ... ... G.C ... .C ... ..C ... .T
..C ... ... G.C ... ... A.. A.G ... ... G.T
... ..A ... ... .GG ..A ... .T G.. G.. ..C ... ... C..
... ..C ... .C ... ...C ... ...A ..G ..T G.. ... ... .TG ...
... ..C .CA ... A.. ... .A ... ...C ... .T ... ...
... .C. ..T ... ... C.. ... ...
... ... C.. ... ... .AC C
... ...A ... ...T ... ...T G.. ..T
G.. ..C ... ..C ... .C. ... ...T ... ..C ... ... .T. ... ... A..
... ... G.. ... .G. ... ... .A. ... .C ... ...
.T. ... .C. C.C ... ... A.. ... ...T ... ...T ... .A. ..G ...
... C.. ... .A. ... ..A ... ..C ..A ... .C ..A ..C ..G ... ..G .T. ... .C ...
... ... G.. .T. ... ..C ... ... A.C ... ..C ..C ..T ... ... AT. ...
..C ..A ... ... A.G ... ...A ... ...T ..A ... ..C ..T ... ...
.TT ... ...T G.. A.A ... ..A ... ..G ... .C ...
..T ... .T. .GC GT. ... ..T ... ... G.. ... ..C ..C ..A ... ... .A.
... .TG ... .G ..G ... ... C.. ... .A. ... .G. T.C ... ... .G. ... ...
... ..A ... ... A.C ... ... T.. ... ... CC. ...
..T G.. ... ... CA. ... C.G ... ...C .A. ... ... .T. ...
..C ..C ... ... .G. ... ...C ... ... .G. ... ..G ... .T.
... ... A.. ... ... .CA ... A.. ... ...T ... ...
.C. ... ... .A. ..C ... .A. ... A.. ... ...T ..G ... .T G.. ...
... ... .A. ... ... .C. ... ...G ... .T .T. ... ..A A.G G.. ... .G. C
.TG ... T.. ... ... G.C ... ... C.. ... ...T ... ...
T.. ... AT.
Sus_scrofa
... ... CAC ... ... C.C .A. ... ..T ..G ... TCA ... ... GCA G.. C.. ..C ... ...
.G. ..C ... .C. ..C .CG A.. ... .G. ... ..T .G. ... ...C .T. ... ...
.G. ... ...G ..C ... ... A.. G.. ... ..G ... .G. ... .G. ... ...
... .C ... ..C ... ... .GA .CA ..C ... G.. ... .C ... ..G ... .T
..C ..T C.. ... ... G.. ... ..T G.. ... ... A.C ... ..A ... ... G..
..G ... ... .GG ... ..G ... ... G.. G.. ..C ... ... C..
... ..C ... ..C ..G ... ...A ... ...G ..T G.. ..T ... ..T ...
... ..C ... ...G .TG ..C ... ...G ..C A.. ... .C ... ...
..C .C. ... ... G.. ... .C ... C.. ... .G ... ... C.. ..T ... ...
... ..C ..T ... ... C.. ... ...T .AC ... ...
... .A C.. .AA .C. ... ..C ... ..C ... ...A ... ... G.. ..C
G.. ..C ..C ..C ... .C. ... ..A ... ...C ... ..T ... .T. C
... ... G.. ... .G. ... ...G .A. ... .C ... ...
.T. ... .C. C.C C.. ... ... A.. ... ...T ..T ... .T ... .A. C
..G ... ... .A. ... .A ... ..C ... ...A A.. ..G ... .C ... ...
... ... .T. ..G ..C ... ... A.C ... .C ... ... AT. ...
..C ... ... A.G ..C ... .G ... .T ... ... .T. ... ...G
.AT ... ...G G.. ... ..A ... ...
... ... .T. ... ...T ... .C T.. ..T ..C ... ... .A.
A.. .T. ... .G ... ... C.. C.. ... .A. ... ... T.C ... ... .GA ... .T.

```
... ..C ... ... ..G ... ... ... A.. ... ... ... ... ... ... ..C ... ... ... ... CC. ...
... ... ... ... ... ... ..C C.C ... ... ... ..T ... ... ... .A. ... ... ... ... ..T ...
... ..C ... ... ... ... ... ... ... ... ... ... ... ... ..C ... ..A .G. ... ..G ... ..A
... ..A ... ... A.. ... ... ... ... .C. ... A.. ... ... ... ... ... ... ... ... ... ...
.CC ... ... ... ... .C. ... ..T G.. ... A.. ... ..A ..C ..C ... ..G ... ... ... G.. ...
G.. ... CA. ... ..C .CT ... ... ... ... ..A ..A ... ... ... ..C .T. ... ... G.. ..T ..T
... ..T ... ... ... G.. ... T.. ..A C.. ... ... ... A.. C.C ... ... ... ..T ... ... ...
... ... .T.
```

Tree file for model A: whales-A.tree

```
(((((Orcinus_orca, Tursiops_truncatus), Neophocaena_phocaenoides), Lipotes_vexillifer),
(Balaenoptera_acutorostrata, Balaenoptera_physalus)), Bos_taurus, Sus_scrofa);
```

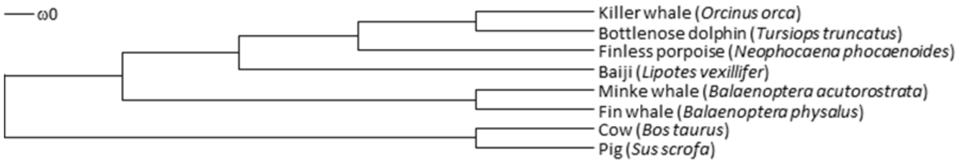

Tree file for model B: whales-B.tree

```
(((((Orcinus_orca, Tursiops_truncatus), Neophocaena_phocaenoides), Lipotes_vexillifer),
(Balaenoptera_acutorostrata, Balaenoptera_physalus)), Bos_taurus, Sus_scrofa);
```

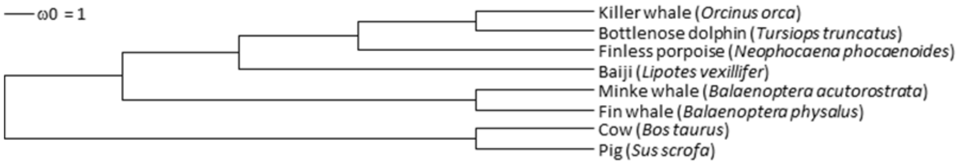

Tree file for model C: whales-C.tree

```
(((((Orcinus_orca: #1, Tursiops_truncatus: #1): #1, Neophocaena_phocaenoides: #1): #1,
Lipotes_vexillifer: #1): #1, (Balaenoptera_acutorostrata: #1, Balaenoptera_physalus: #1): #1),
Bos_taurus, Sus_scrofa);
```

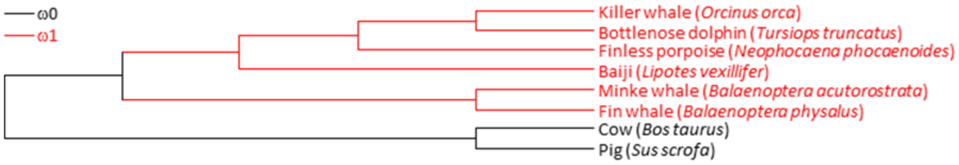

Tree file for model D: whales-D.tree

```
(((((Orcinus_orca: #1, Tursiops_truncatus: #1): #1, Neophocaena_phocaenoides: #1): #1,
Lipotes_vexillifer: #1): #1, (Balaenoptera_acutorostrata: #1, Balaenoptera_physalus: #1): #1),
Bos_taurus, Sus_scrofa);
```

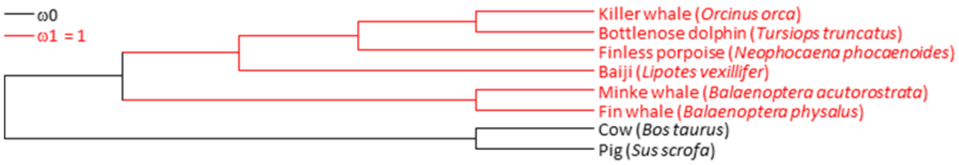

Tree file for model E: whales-E.tree

```
(((((Orcinus_orca: #1, Tursiops_truncatus: #1): #1, Neophocaena_phocaenoides: #1): #1,
Lipotes_vexillifer: #1): #1, (Balaenoptera_acutorostrata: #1, Balaenoptera_physalus: #1): #1): #1,
Bos_taurus, Sus_scrofa);
```

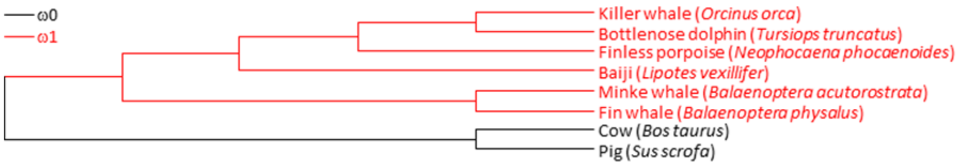

Tree file for model F: whales-F.tree

```
(((((Orcinus_orca: #1, Tursiops_truncatus: #1): #1, Neophocaena_phocaenoides: #1): #1,
Lipotes_vexillifer: #1): #1, (Balaenoptera_acutorostrata: #1, Balaenoptera_physalus: #1): #1): #1,
Bos_taurus, Sus_scrofa);
```

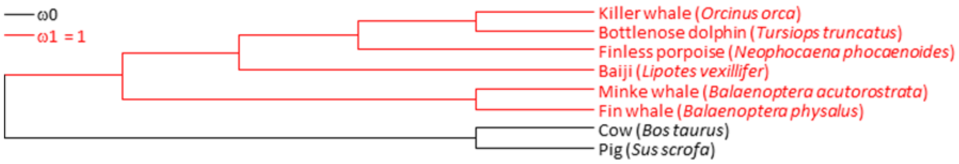

Tree file for model G: whales-G.tree

```
(((((Orcinus_orca, Tursiops_truncatus), Neophocaena_phocaenoides), Lipotes_vexillifer),
(Balaenoptera_acutorostrata, Balaenoptera_physalus)), Bos_taurus, Sus_scrofa);
```

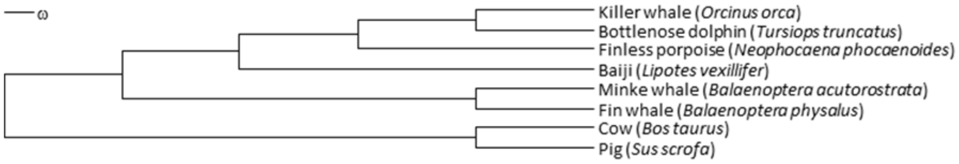

Control file for model A: whales-A.ctl

```
      seqfile = whales.phy
     treefile = whales-A.tree
      outfile = whales-A.mlc

        noisy = 9  * 0,1,2,3,9: how much rubbish on the screen
      verbose = 0  * 0: concise; 1: detailed, 2: too much
      runmode = 0  * 0: user tree;  1: semi-automatic;  2: automatic
                   * 3: StepwiseAddition; (4,5):PerturbationNNI; -2: pairwise

      seqtype = 1  * 1:codons; 2:AAs; 3:codons-->AAs
    CodonFreq = 2  * 0:1/61 each, 1:F1X4, 2:F3X4, 3:codon table
        clock = 0  * 0:no clock, 1:clock; 2:local clock; 3:CombinedAnalysis
        model = 0
                   * models for codons:
                       * 0:one, 1:b, 2:2 or more dN/dS ratios for branches

      NSsites = 0  * 0:one w; 1:neutral; 2:selection; 3:discrete; 4:freqs;
                   * 5:gamma; 6:2gamma; 7:beta; 8:beta&w; 9:beta&gamma;
                   * 10:beta&gamma+1; 11:beta&normal>1; 12:0&2normal>1;
                   * 13:3normal>0
        icode = 0  * 0:universal code; 1:mammalian mt; 2-10:see below

    fix_kappa = 0  * 1: kappa fixed, 0: kappa to be estimated
        kappa = 2  * initial or fixed kappa
    fix_omega = 0  * 1: omega or omega_1 fixed, 0: estimate
        omega = 2  * initial or fixed omega, for codons or codon-based AAs

    fix_alpha = 1  * 0: estimate gamma shape parameter; 1: fix it at alpha
        alpha = .0 * initial or fixed alpha, 0:infinity (constant rate)
       Malpha = 0  * different alphas for genes
        ncatG = 4  * # of categories in dG of NSsites models

        getSE = 0  * 0: don't want them, 1: want S.E.s of estimates
 RateAncestor = 0  * (0,1,2): rates (alpha>0) or ancestral states (1 or 2)
       method = 0  * 0: simultaneous; 1: one branch at a time
```

Control file for model B: whales-B.ctl

```
      seqfile = whales.phy
     treefile = whales-B.tree
      outfile = whales-B.mlc

        noisy = 9  * 0,1,2,3,9: how much rubbish on the screen
      verbose = 0  * 0: concise; 1: detailed, 2: too much
      runmode = 0  * 0: user tree;  1: semi-automatic;  2: automatic
                   * 3: StepwiseAddition; (4,5):PerturbationNNI; -2: pairwise

      seqtype = 1  * 1:codons; 2:AAs; 3:codons-->AAs
    CodonFreq = 2  * 0:1/61 each, 1:F1X4, 2:F3X4, 3:codon table
        clock = 0  * 0:no clock, 1:clock; 2:local clock; 3:CombinedAnalysis
        model = 0
                   * models for codons:
                       * 0:one, 1:b, 2:2 or more dN/dS ratios for branches

      NSsites = 0  * 0:one w; 1:neutral; 2:selection; 3:discrete; 4:freqs;
                   * 5:gamma; 6:2gamma; 7:beta; 8:beta&w; 9:beta&gamma;
                   * 10:beta&gamma+1; 11:beta&normal>1; 12:0&2normal>1;
                   * 13:3normal>0
        icode = 0  * 0:universal code; 1:mammalian mt; 2-10:see below

    fix_kappa = 0  * 1: kappa fixed, 0: kappa to be estimated
        kappa = 2  * initial or fixed kappa
    fix_omega = 1  * 1: omega or omega_1 fixed, 0: estimate
        omega = 1  * initial or fixed omega, for codons or codon-based AAs

    fix_alpha = 1  * 0: estimate gamma shape parameter; 1: fix it at alpha
        alpha = .0 * initial or fixed alpha, 0:infinity (constant rate)
       Malpha = 0  * different alphas for genes
        ncatG = 4  * # of categories in dG of NSsites models

        getSE = 0  * 0: don't want them, 1: want S.E.s of estimates
 RateAncestor = 0  * (0,1,2): rates (alpha>0) or ancestral states (1 or 2)
       method = 0  * 0: simultaneous; 1: one branch at a time
```

Control file for model C: whales-C.ctl

```
      seqfile = whales.phy
     treefile = whales-C.tree
      outfile = whales-C.mlc

        noisy = 9  * 0,1,2,3,9: how much rubbish on the screen
      verbose = 0  * 0: concise; 1: detailed, 2: too much
      runmode = 0  * 0: user tree;  1: semi-automatic;  2: automatic
                   * 3: StepwiseAddition; (4,5):PerturbationNNI; -2: pairwise

      seqtype = 1  * 1:codons; 2:AAs; 3:codons-->AAs
    CodonFreq = 2  * 0:1/61 each, 1:F1X4, 2:F3X4, 3:codon table
        clock = 0  * 0:no clock, 1:clock; 2:local clock; 3:CombinedAnalysis
        model = 2
                   * models for codons:
                       * 0:one, 1:b, 2:2 or more dN/dS ratios for branches

      NSsites = 0  * 0:one w; 1:neutral; 2:selection; 3:discrete; 4:freqs;
                   * 5:gamma; 6:2gamma; 7:beta; 8:beta&w; 9:beta&gamma;
                   * 10:beta&gamma+1; 11:beta&normal>1; 12:0&2normal>1;
                   * 13:3normal>0
        icode = 0  * 0:universal code; 1:mammalian mt; 2-10:see below

    fix_kappa = 0  * 1: kappa fixed, 0: kappa to be estimated
        kappa = 2  * initial or fixed kappa
    fix_omega = 0  * 1: omega or omega_1 fixed, 0: estimate
        omega = 2  * initial or fixed omega, for codons or codon-based AAs

    fix_alpha = 1  * 0: estimate gamma shape parameter; 1: fix it at alpha
        alpha = .0 * initial or fixed alpha, 0:infinity (constant rate)
       Malpha = 0  * different alphas for genes
        ncatG = 4  * # of categories in dG of NSsites models

        getSE = 0  * 0: don't want them, 1: want S.E.s of estimates
 RateAncestor = 0  * (0,1,2): rates (alpha>0) or ancestral states (1 or 2)
       method = 0  * 0: simultaneous; 1: one branch at a time
```

Control file for model D: whales-D.ctl

```
      seqfile = whales.phy
     treefile = whales-D.tree
      outfile = whales-D.mlc

        noisy = 9  * 0,1,2,3,9: how much rubbish on the screen
      verbose = 0  * 0: concise; 1: detailed, 2: too much
      runmode = 0  * 0: user tree;  1: semi-automatic;  2: automatic
                   * 3: StepwiseAddition; (4,5):PerturbationNNI; -2: pairwise

      seqtype = 1  * 1:codons; 2:AAs; 3:codons-->AAs
    CodonFreq = 2  * 0:1/61 each, 1:F1X4, 2:F3X4, 3:codon table
        clock = 0  * 0:no clock, 1:clock; 2:local clock; 3:CombinedAnalysis
        model = 2
                   * models for codons:
                       * 0:one, 1:b, 2:2 or more dN/dS ratios for branches

      NSsites = 0  * 0:one w; 1:neutral; 2:selection; 3:discrete; 4:freqs;
                   * 5:gamma; 6:2gamma; 7:beta; 8:beta&w; 9:beta&gamma;
                   * 10:beta&gamma+1; 11:beta&normal>1; 12:0&2normal>1;
                   * 13:3normal>0
        icode = 0  * 0:universal code; 1:mammalian mt; 2-10:see below

    fix_kappa = 0  * 1: kappa fixed, 0: kappa to be estimated
        kappa = 2  * initial or fixed kappa
    fix_omega = 1  * 1: omega or omega_1 fixed, 0: estimate
        omega = 1  * initial or fixed omega, for codons or codon-based AAs

    fix_alpha = 1  * 0: estimate gamma shape parameter; 1: fix it at alpha
        alpha = .0 * initial or fixed alpha, 0:infinity (constant rate)
       Malpha = 0  * different alphas for genes
        ncatG = 4  * # of categories in dG of NSsites models

        getSE = 0  * 0: don't want them, 1: want S.E.s of estimates
 RateAncestor = 0  * (0,1,2): rates (alpha>0) or ancestral states (1 or 2)
       method = 0  * 0: simultaneous; 1: one branch at a time
```

Control file for model E: whales-E.ctl

```
      seqfile = whales.phy
     treefile = whales-E.tree
      outfile = whales-E.mlc

        noisy = 9  * 0,1,2,3,9: how much rubbish on the screen
      verbose = 0  * 0: concise; 1: detailed, 2: too much
      runmode = 0  * 0: user tree;  1: semi-automatic;  2: automatic
                   * 3: StepwiseAddition; (4,5):PerturbationNNI; -2: pairwise

      seqtype = 1  * 1:codons; 2:AAs; 3:codons-->AAs
    CodonFreq = 2  * 0:1/61 each, 1:F1X4, 2:F3X4, 3:codon table
        clock = 0  * 0:no clock, 1:clock; 2:local clock; 3:CombinedAnalysis
        model = 2
                   * models for codons:
                       * 0:one, 1:b, 2:2 or more dN/dS ratios for branches

      NSsites = 0  * 0:one w; 1:neutral; 2:selection; 3:discrete; 4:freqs;
                   * 5:gamma; 6:2gamma; 7:beta; 8:beta&w; 9:beta&gamma;
                   * 10:beta&gamma+1; 11:beta&normal>1; 12:0&2normal>1;
                   * 13:3normal>0
        icode = 0  * 0:universal code; 1:mammalian mt; 2-10:see below

    fix_kappa = 0  * 1: kappa fixed, 0: kappa to be estimated
        kappa = 2  * initial or fixed kappa
    fix_omega = 0  * 1: omega or omega_1 fixed, 0: estimate
        omega = 2  * initial or fixed omega, for codons or codon-based AAs

    fix_alpha = 1  * 0: estimate gamma shape parameter; 1: fix it at alpha
        alpha = .0 * initial or fixed alpha, 0:infinity (constant rate)
       Malpha = 0  * different alphas for genes
        ncatG = 4  * # of categories in dG of NSsites models

        getSE = 0  * 0: don't want them, 1: want S.E.s of estimates
 RateAncestor = 0  * (0,1,2): rates (alpha>0) or ancestral states (1 or 2)
       method = 0  * 0: simultaneous; 1: one branch at a time
```

Control file for model F: whales-F.ctl

```
      seqfile = whales.phy
     treefile = whales-F.tree
      outfile = whales-F.mlc

        noisy = 9  * 0,1,2,3,9: how much rubbish on the screen
      verbose = 0  * 0: concise; 1: detailed, 2: too much
      runmode = 0  * 0: user tree;  1: semi-automatic;  2: automatic
                   * 3: StepwiseAddition; (4,5):PerturbationNNI; -2: pairwise

      seqtype = 1  * 1:codons; 2:AAs; 3:codons-->AAs
    CodonFreq = 2  * 0:1/61 each, 1:F1X4, 2:F3X4, 3:codon table
        clock = 0  * 0:no clock, 1:clock; 2:local clock; 3:CombinedAnalysis
        model = 2
                   * models for codons:
                       * 0:one, 1:b, 2:2 or more dN/dS ratios for branches

      NSsites = 0  * 0:one w; 1:neutral; 2:selection; 3:discrete; 4:freqs;
                   * 5:gamma; 6:2gamma; 7:beta; 8:beta&w; 9:beta&gamma;
                   * 10:beta&gamma+1; 11:beta&normal>1; 12:0&2normal>1;
                   * 13:3normal>0
        icode = 0  * 0:universal code; 1:mammalian mt; 2-10:see below

    fix_kappa = 0  * 1: kappa fixed, 0: kappa to be estimated
        kappa = 2  * initial or fixed kappa
    fix_omega = 1  * 1: omega or omega_1 fixed, 0: estimate
        omega = 1  * initial or fixed omega, for codons or codon-based AAs

    fix_alpha = 1  * 0: estimate gamma shape parameter; 1: fix it at alpha
        alpha = .0 * initial or fixed alpha, 0:infinity (constant rate)
       Malpha = 0  * different alphas for genes
        ncatG = 4  * # of categories in dG of NSsites models

        getSE = 0  * 0: don't want them, 1: want S.E.s of estimates
 RateAncestor = 0  * (0,1,2): rates (alpha>0) or ancestral states (1 or 2)
       method = 0  * 0: simultaneous; 1: one branch at a time
```

Control file for model G: whales-G.ctl

```
      seqfile = whales.phy
     treefile = whales-G.tree
      outfile = whales-G.mlc

        noisy = 9  * 0,1,2,3,9: how much rubbish on the screen
      verbose = 0  * 0: concise; 1: detailed, 2: too much
      runmode = 0  * 0: user tree;  1: semi-automatic;  2: automatic
                   * 3: StepwiseAddition; (4,5):PerturbationNNI; -2: pairwise

      seqtype = 1  * 1:codons; 2:AAs; 3:codons-->AAs
    CodonFreq = 2  * 0:1/61 each, 1:F1X4, 2:F3X4, 3:codon table
        clock = 0  * 0:no clock, 1:clock; 2:local clock; 3:CombinedAnalysis
        model = 1
                   * models for codons:
                       * 0:one, 1:b, 2:2 or more dN/dS ratios for branches

      NSsites = 0  * 0:one w; 1:neutral; 2:selection; 3:discrete; 4:freqs;
                   * 5:gamma; 6:2gamma; 7:beta; 8:beta&w; 9:beta&gamma;
                   * 10:beta&gamma+1; 11:beta&normal>1; 12:0&2normal>1;
                   * 13:3normal>0
        icode = 0  * 0:universal code; 1:mammalian mt; 2-10:see below

    fix_kappa = 0  * 1: kappa fixed, 0: kappa to be estimated
        kappa = 2  * initial or fixed kappa
    fix_omega = 0  * 1: omega or omega_1 fixed, 0: estimate
        omega = 2  * initial or fixed omega, for codons or codon-based AAs

    fix_alpha = 1  * 0: estimate gamma shape parameter; 1: fix it at alpha
        alpha = .0 * initial or fixed alpha, 0:infinity (constant rate)
       Malpha = 0  * different alphas for genes
        ncatG = 4  * # of categories in dG of NSsites models

        getSE = 0  * 0: don't want them, 1: want S.E.s of estimates
 RateAncestor = 0  * (0,1,2): rates (alpha>0) or ancestral states (1 or 2)
       method = 0  * 0: simultaneous; 1: one branch at a time
```

Main result file for model A: whales-A.mlc

```
CODONML (in paml version 4.7b, October 2013)  whales.phy
Model: One dN/dS ratio for branches,
Codon frequency model: F3x4
ns =   8  ls = 597

Codon usage in sequences
--------------------------------------------------------------------------------------------------------------------------------------
Phe TTT  10  10   9  11  10  10 | Ser TCT   6   5   5   5   4   5 | Tyr TAT   6   7   7   4   7   7 | Cys TGT   5   4   3   9   6   6
    TTC  13  14  12  15  18  16 |     TCC  16  17  20  18  16  16 |     TAC  15  15  15  16  13  14 |     TGC  11  12  11   9   9  10
Leu TTA   3   2   2   2   1   1 |     TCA   2   3   3   2   2   1 | *** TAA   0   0   0   0   0   0 | *** TGA   0   0   0   0   0   0
    TTG  12  12  12   9  10  10 |     TCG   4   4   4   4   5   6 |     TAG   0   0   0   0   0   0 | Trp TGG   7   7   8   7   7   7
--------------------------------------------------------------------------------------------------------------------------------------
Leu CTT   6   7   6   6   6   7 | Pro CCT   9   9   9  12  10  10 | His CAT   5   3   3   5   6   5 | Arg CGT   2   4   5   2   3   2
    CTC  14  12  14  13  16  18 |     CCC  14  14  15  14  17  15 |     CAC  15  15  15  15  16  16 |     CGC   6   4   6   6   7   4
    CTA   6   5   5   4   4   4 |     CCA   5   5   6   4   4   4 | Gln CAA   6   6   4   5   3   4 |     CGA   0   0   1   1   2   1
    CTG  26  23  27  28  24  26 |     CCG   4   7   3   6   7   5 |     CAG  18  18  16  19  16  16 |     CGG   3   3   3   2   2   4
--------------------------------------------------------------------------------------------------------------------------------------
Ile ATT   6   6   7   7   8   9 | Thr ACT  14  13  13  14  11  11 | Asn AAT  10   9  10  10   9   7 | Ser AGT   4   6   4   4   3   3
    ATC  27  25  26  25  26  27 |     ACC  16  16  14  13  12  14 |     AAC   8   8   8   9   7  10 |     AGC   9   9   8   6   9   9
    ATA   1   3   3   3   3   3 |     ACA   9   9  10  11  10  11 | Lys AAA   4   3   3   3   3   3 | Arg AGA   4   4   3   5   4   4
Met ATG  17  15  16  12  13  12 |     ACG   5   7   7   9  11   9 |     AAG  13  13  13  13  15  16 |     AGG   4   4   4   6   4   4
--------------------------------------------------------------------------------------------------------------------------------------
Val GTT   5   5   5   4   4   2 | Ala GCT  13  13  13  10  10  11 | Asp GAT  17  14  15  17  15  17 | Gly GGT   8   7   8   6   7   8
    GTC  11  13  13  12  12  12 |     GCC  23  23  22  23  27  25 |     GAC  25  28  27  22  23  24 |     GGC  16  17  17  22  18  15
    GTA   3   3   3   2   2   2 |     GCA   3   3   4   3   3   3 | Glu GAA   5   6   5   6   7   6 |     GGA   6   7   6   6   6   6
    GTG  21  20  19  22  18  18 |     GCG   2   3   1   2   4   5 |     GAG  27  26  27  27  30  30 |     GGG  12  12  14  10  12  11
--------------------------------------------------------------------------------------------------------------------------------------


----------------------------------------------------------------------
Phe TTT  13   7 | Ser TCT   5   4 | Tyr TAT   5   9 | Cys TGT   6   3
    TTC  17  20 |     TCC  14  16 |     TAC  17  15 |     TGC   9   9
Leu TTA   1   2 |     TCA   3   2 | *** TAA   0   0 | *** TGA   0   0
    TTG   9   4 |     TCG   2   4 |     TAG   0   0 | Trp TGG   7   6
----------------------------------------------------------------------
Leu CTT   5   8 | Pro CCT  11   8 | His CAT   2   5 | Arg CGT   4   1
    CTC  15  17 |     CCC  19  22 |     CAC  14  12 |     CGC   9   7
    CTA   5   2 |     CCA   4   3 | Gln CAA   4   6 |     CGA   0   2
    CTG  24  30 |     CCG   4   6 |     CAG  19  16 |     CGG   3   5
----------------------------------------------------------------------
Ile ATT   7  10 | Thr ACT   9   3 | Asn AAT   8   9 | Ser AGT   2   4
    ATC  28  25 |     ACC  14  20 |     AAC   8   7 |     AGC  13  10
    ATA   3   3 |     ACA   9   6 | Lys AAA   2   4 | Arg AGA   2   2
Met ATG  12  15 |     ACG   9  10 |     AAG  15  12 |     AGG   6   4
----------------------------------------------------------------------
Val GTT   4   2 | Ala GCT  10  14 | Asp GAT  18  15 | Gly GGT   5   2
    GTC  15  14 |     GCC  23  24 |     GAC  27  31 |     GGC  18  21
    GTA   5   2 |     GCA   5   5 | Glu GAA   7   8 |     GGA   6   6
    GTG  16  17 |     GCG   2   1 |     GAG  27  27 |     GGG  12  13
----------------------------------------------------------------------

Codon position x base (3x4) table for each sequence.

#1: Orcinus_orca
position  1:    T:0.18425    C:0.23283    A:0.25293    G:0.32998
position  2:    T:0.30318    C:0.24288    A:0.29146    G:0.16248
position  3:    T:0.21106    C:0.40034    A:0.09548    G:0.29313
Average         T:0.23283    C:0.29202    A:0.21329    G:0.26186

#2: Tursiops_truncatus
position  1:    T:0.18760    C:0.22613    A:0.25126    G:0.33501
position  2:    T:0.29313    C:0.25293    A:0.28643    G:0.16750
position  3:    T:0.20436    C:0.40536    A:0.09883    G:0.29146
Average         T:0.22836    C:0.29481    A:0.21217    G:0.26466

#3: Neophocaena_phocaenoides
position  1:    T:0.18593    C:0.23116    A:0.24958    G:0.33333
position  2:    T:0.29983    C:0.24958    A:0.28141    G:0.16918
position  3:    T:0.20436    C:0.40704    A:0.09715    G:0.29146
Average         T:0.23004    C:0.29592    A:0.20938    G:0.26466

#4: Lipotes_vexillifer
position  1:    T:0.18593    C:0.23786    A:0.25126    G:0.32496
position  2:    T:0.29313    C:0.25126    A:0.28643    G:0.16918
position  3:    T:0.21106    C:0.39866    A:0.09548    G:0.29481
Average         T:0.23004    C:0.29592    A:0.21106    G:0.26298

#5: Balaenoptera_acutorostrata
position  1:    T:0.18090    C:0.23953    A:0.24791    G:0.33166
position  2:    T:0.29313    C:0.25628    A:0.28476    G:0.16583
position  3:    T:0.19933    C:0.41206    A:0.09045    G:0.29816
```

```
Average         T:0.22446    C:0.30262    A:0.20771    G:0.26521

#6: Balaenoptera_physalus
position  1:    T:0.18258    C:0.23618    A:0.25461    G:0.32663
position  2:    T:0.29648    C:0.25293    A:0.29313    G:0.15745
position  3:    T:0.20101    C:0.41039    A:0.08878    G:0.29983
Average         T:0.22669    C:0.29983    A:0.21217    G:0.26131

#7: Bos_taurus
position  1:    T:0.18090    C:0.23786    A:0.24623    G:0.33501
position  2:    T:0.29983    C:0.23953    A:0.28978    G:0.17085
position  3:    T:0.19095    C:0.43551    A:0.09380    G:0.27973
Average         T:0.22390    C:0.30430    A:0.20994    G:0.26186

#8: Sus_scrofa
position  1:    T:0.16918    C:0.25126    A:0.24121    G:0.33836
position  2:    T:0.29816    C:0.24791    A:0.29481    G:0.15913
position  3:    T:0.17420    C:0.45226    A:0.08878    G:0.28476
Average         T:0.21385    C:0.31714    A:0.20826    G:0.26075

Sums of codon usage counts
------------------------------------------------------------------------------
Phe F TTT      80 | Ser S TCT      39 | Tyr Y TAT      52 | Cys C TGT      42
      TTC     125 |       TCC     133 |       TAC     120 |       TGC      80
Leu L TTA      14 |       TCA      18 | *** * TAA       0 | *** * TGA       0
      TTG      78 |       TCG      33 |       TAG       0 | Trp W TGG      56
------------------------------------------------------------------------------
Leu L CTT      51 | Pro P CCT      78 | His H CAT      34 | Arg R CGT      23
      CTC     119 |       CCC     130 |       CAC     118 |       CGC      49
      CTA      35 |       CCA      35 | Gln Q CAA      38 |       CGA       7
      CTG     208 |       CCG      42 |       CAG     138 |       CGG      25
------------------------------------------------------------------------------
Ile I ATT      60 | Thr T ACT      88 | Asn N AAT      72 | Ser S AGT      30
      ATC     209 |       ACC     119 |       AAC      65 |       AGC      73
      ATA      22 |       ACA      75 | Lys K AAA      25 | Arg R AGA      28
Met M ATG     112 |       ACG      67 |       AAG     110 |       AGG      36
------------------------------------------------------------------------------
Val V GTT      31 | Ala A GCT      94 | Asp D GAT     128 | Gly G GGT      51
      GTC     102 |       GCC     190 |       GAC     207 |       GGC     144
      GTA      22 |       GCA      29 | Glu E GAA      50 |       GGA      49
      GTG     151 |       GCG      20 |       GAG     221 |       GGG      96
------------------------------------------------------------------------------


Codon position x base (3x4) table, overall

position  1:    T:0.18216    C:0.23660    A:0.24937    G:0.33187
position  2:    T:0.29711    C:0.24916    A:0.28853    G:0.16520
position  3:    T:0.19954    C:0.41520    A:0.09359    G:0.29167
Average         T:0.22627    C:0.30032    A:0.21050    G:0.26291


Nei & Gojobori 1986. dN/dS (dN, dS)
(Note: This matrix is not used in later ML. analysis.
Use runmode = -2 for ML pairwise comparison.)

Orcinus_orca
Tursiops_truncatus   0.8408 (0.0178 0.0212)
Neophocaena_phocaenoides 1.0453 (0.0270 0.0258) 0.8334 (0.0255 0.0306)
Lipotes_vexillifer   0.8298 (0.0479 0.0577) 0.8313 (0.0479 0.0576) 0.7981 (0.0496 0.0621)
Balaenoptera_acutorostrata 0.7447 (0.0553 0.0743) 0.7507 (0.0538 0.0716) 0.7507 (0.0543 0.0723) 0.7527
(0.0527 0.0700)
Balaenoptera_physalus 0.6855 (0.0616 0.0899) 0.7105 (0.0629 0.0885) 0.6728 (0.0634 0.0943) 0.7024
(0.0582 0.0828) 0.5288 (0.0239 0.0453)
Bos_taurus           0.3522 (0.1025 0.2911) 0.3368 (0.0996 0.2958) 0.3597 (0.1011 0.2812) 0.3377
(0.0971 0.2877) 0.2775 (0.0729 0.2627) 0.2814 (0.0831 0.2952)
Sus_scrofa           0.2496 (0.0892 0.3574) 0.2370 (0.0848 0.3580) 0.2536 (0.0882 0.3479) 0.2408
(0.0840 0.3489) 0.1883 (0.0634 0.3365) 0.2189 (0.0730 0.3335) 0.1460 (0.0649 0.4443)


TREE #  1:  (((((1, 2), 3), 4), (5, 6)), 7, 8);   MP score: 489
lnL(ntime: 13  np: 15):  -5047.274014      +0.000000
   9..10   10..11   11..12   12..13   13..1    13..2    12..3    11..4    10..14   14..5    14..6
9..7     9..8
 0.101487 0.049742 0.042195 0.014024 0.031379 0.025788 0.043279 0.076650 0.024286 0.028541 0.061740
0.229862 0.233444 4.150731 0.315098
```

Note: Branch length is defined as number of nucleotide substitutions per codon (not per neucleotide site).

tree length = 0.96242

(((((1: 0.031379, 2: 0.025788): 0.014024, 3: 0.043279): 0.042195, 4: 0.076650): 0.049742, (5: 0.028541, 6: 0.061740): 0.024286): 0.101487, 7: 0.229862, 8: 0.233444);

(((((Orcinus_orca: 0.031379, Tursiops_truncatus: 0.025788): 0.014024, Neophocaena_phocaenoides: 0.043279): 0.042195, Lipotes_vexillifer: 0.076650): 0.049742, (Balaenoptera_acutorostrata: 0.028541, Balaenoptera_physalus: 0.061740): 0.024286): 0.101487, Bos_taurus: 0.229862, Sus_scrofa: 0.233444);

Detailed output identifying parameters

kappa (ts/tv) = 4.15073

omega (dN/dS) = 0.31510

dN & dS for each branch

branch	t	N	S	dN/dS	dN	dS	N*dN	S*dS
9..10	0.101	1349.3	441.7	0.3151	0.0220	0.0699	29.7	30.9
10..11	0.050	1349.3	441.7	0.3151	0.0108	0.0343	14.6	15.1
11..12	0.042	1349.3	441.7	0.3151	0.0092	0.0291	12.4	12.8
12..13	0.014	1349.3	441.7	0.3151	0.0030	0.0097	4.1	4.3
13..1	0.031	1349.3	441.7	0.3151	0.0068	0.0216	9.2	9.5
13..2	0.026	1349.3	441.7	0.3151	0.0056	0.0178	7.6	7.8
12..3	0.043	1349.3	441.7	0.3151	0.0094	0.0298	12.7	13.2
11..4	0.077	1349.3	441.7	0.3151	0.0166	0.0528	22.4	23.3
10..14	0.024	1349.3	441.7	0.3151	0.0053	0.0167	7.1	7.4
14..5	0.029	1349.3	441.7	0.3151	0.0062	0.0197	8.4	8.7
14..6	0.062	1349.3	441.7	0.3151	0.0134	0.0425	18.1	18.8
9..7	0.230	1349.3	441.7	0.3151	0.0499	0.1583	67.3	69.9
9..8	0.233	1349.3	441.7	0.3151	0.0507	0.1608	68.4	71.0

tree length for dN: 0.2089
tree length for dS: 0.6628

Main result file for model B: whales-B.mlc

```
CODONML (in paml version 4.7b, October 2013)  whales.phy
Model: One dN/dS ratio for branches,  omega = 1.000 fixed

Codon frequency model: F3x4
ns =   8  ls = 597

Codon usage in sequences
--------------------------------------------------------------------------------------------------------------------------------------
Phe TTT  10  10   9  11  10  10 | Ser TCT   6   5   5   5   4   5 | Tyr TAT   6   7   7   4   7   7 | Cys TGT   5   4   3   9   6   6
    TTC  13  14  12  15  18  16 |     TCC  16  17  20  18  16  16 |     TAC  15  15  15  16  13  14 |     TGC  11  12  11   9   9  10
Leu TTA   3   2   2   2   1   1 |     TCA   2   3   3   2   2   1 | *** TAA   0   0   0   0   0   0 | *** TGA   0   0   0   0   0   0
    TTG  12  12  12   9  10  10 |     TCG   4   4   4   4   5   6 |     TAG   0   0   0   0   0   0 | Trp TGG   7   7   8   7   7   7
--------------------------------------------------------------------------------------------------------------------------------------
Leu CTT   6   7   6   6   6   7 | Pro CCT   9   9   9  12  10  10 | His CAT   5   3   3   5   6   5 | Arg CGT   2   4   5   2   3   2
    CTC  14  12  14  13  16  18 |     CCC  14  14  15  14  17  15 |     CAC  15  15  15  15  16  16 |     CGC   6   4   6   6   7   4
    CTA   6   5   5   4   4   4 |     CCA   5   5   6   4   4   4 | Gln CAA   6   6   4   5   3   4 |     CGA   0   0   1   1   2   1
    CTG  26  23  27  28  24  26 |     CCG   4   7   3   6   7   5 |     CAG  18  18  16  19  16  16 |     CGG   3   3   3   2   2   4
--------------------------------------------------------------------------------------------------------------------------------------
Ile ATT   6   6   7   7   8   9 | Thr ACT  14  13  13  14  11  11 | Asn AAT  10   9  10  10   9   7 | Ser AGT   4   6   4   4   3   3
    ATC  27  25  26  25  26  27 |     ACC  16  16  14  13  12  14 |     AAC   8   8   8   9   7  10 |     AGC   9   9   8   6   9   9
    ATA   1   3   3   3   3   3 |     ACA   9   9  10  11  10  11 | Lys AAA   4   3   3   3   3   3 | Arg AGA   4   4   3   5   4   4
Met ATG  17  15  16  12  13  12 |     ACG   5   7   7   9  11   9 |     AAG  13  13  13  13  15  16 |     AGG   4   4   4   6   4   4
--------------------------------------------------------------------------------------------------------------------------------------
Val GTT   5   5   5   4   4   2 | Ala GCT  13  13  13  10  10  11 | Asp GAT  17  14  15  17  15  17 | Gly GGT   8   7   8   6   7   8
    GTC  11  13  13  12  12  12 |     GCC  23  23  22  23  27  25 |     GAC  25  28  27  22  23  24 |     GGC  16  17  17  22  18  15
    GTA   3   3   3   2   2   2 |     GCA   3   3   4   3   3   3 | Glu GAA   5   6   5   6   7   6 |     GGA   6   7   6   6   6   6
    GTG  21  20  19  22  18  18 |     GCG   2   3   1   2   4   5 |     GAG  27  26  27  27  30  30 |     GGG  12  12  14  10  12  11
--------------------------------------------------------------------------------------------------------------------------------------

----------------------------------------------------------------------
Phe TTT  13   7 | Ser TCT   5   4 | Tyr TAT   5   9 | Cys TGT   6   3
    TTC  17  20 |     TCC  14  16 |     TAC  17  15 |     TGC   9   9
Leu TTA   1   2 |     TCA   3   2 | *** TAA   0   0 | *** TGA   0   0
    TTG   9   4 |     TCG   2   4 |     TAG   0   0 | Trp TGG   7   6
----------------------------------------------------------------------
Leu CTT   5   8 | Pro CCT  11   8 | His CAT   2   5 | Arg CGT   4   1
    CTC  15  17 |     CCC  19  22 |     CAC  14  12 |     CGC   9   7
    CTA   5   2 |     CCA   4   3 | Gln CAA   4   6 |     CGA   0   2
    CTG  24  30 |     CCG   4   6 |     CAG  19  16 |     CGG   3   5
----------------------------------------------------------------------
Ile ATT   7  10 | Thr ACT   9   3 | Asn AAT   8   9 | Ser AGT   2   4
    ATC  28  25 |     ACC  14  20 |     AAC   8   7 |     AGC  13  10
    ATA   3   3 |     ACA   9   6 | Lys AAA   2   4 | Arg AGA   2   2
Met ATG  12  15 |     ACG   9  10 |     AAG  15  12 |     AGG   6   4
----------------------------------------------------------------------
Val GTT   4   2 | Ala GCT  10  14 | Asp GAT  18  15 | Gly GGT   5   2
    GTC  15  14 |     GCC  23  24 |     GAC  27  31 |     GGC  18  21
    GTA   5   2 |     GCA   5   5 | Glu GAA   7   8 |     GGA   6   6
    GTG  16  17 |     GCG   2   1 |     GAG  27  27 |     GGG  12  13
----------------------------------------------------------------------

Codon position x base (3x4) table for each sequence.

#1: Orcinus_orca
position  1:    T:0.18425    C:0.23283    A:0.25293    G:0.32998
position  2:    T:0.30318    C:0.24288    A:0.29146    G:0.16248
position  3:    T:0.21106    C:0.40034    A:0.09548    G:0.29313
Average         T:0.23283    C:0.29202    A:0.21329    G:0.26186

#2: Tursiops_truncatus
position  1:    T:0.18760    C:0.22613    A:0.25126    G:0.33501
position  2:    T:0.29313    C:0.25293    A:0.28643    G:0.16750
position  3:    T:0.20436    C:0.40536    A:0.09883    G:0.29146
Average         T:0.22836    C:0.29481    A:0.21217    G:0.26466

#3: Neophocaena_phocaenoides
position  1:    T:0.18593    C:0.23116    A:0.24958    G:0.33333
position  2:    T:0.29983    C:0.24958    A:0.28141    G:0.16918
position  3:    T:0.20436    C:0.40704    A:0.09715    G:0.29146
Average         T:0.23004    C:0.29592    A:0.20938    G:0.26466

#4: Lipotes_vexillifer
position  1:    T:0.18593    C:0.23786    A:0.25126    G:0.32496
position  2:    T:0.29313    C:0.25126    A:0.28643    G:0.16918
position  3:    T:0.21106    C:0.39866    A:0.09548    G:0.29481
Average         T:0.23004    C:0.29592    A:0.21106    G:0.26298

#5: Balaenoptera_acutorostrata
position  1:    T:0.18090    C:0.23953    A:0.24791    G:0.33166
position  2:    T:0.29313    C:0.25628    A:0.28476    G:0.16583
```

```
position  3:    T:0.19933    C:0.41206    A:0.09045    G:0.29816
Average         T:0.22446    C:0.30262    A:0.20771    G:0.26521

#6: Balaenoptera_physalus
position  1:    T:0.18258    C:0.23618    A:0.25461    G:0.32663
position  2:    T:0.29648    C:0.25293    A:0.29313    G:0.15745
position  3:    T:0.20101    C:0.41039    A:0.08878    G:0.29983
Average         T:0.22669    C:0.29983    A:0.21217    G:0.26131

#7: Bos_taurus
position  1:    T:0.18090    C:0.23786    A:0.24623    G:0.33501
position  2:    T:0.29983    C:0.23953    A:0.28978    G:0.17085
position  3:    T:0.19095    C:0.43551    A:0.09380    G:0.27973
Average         T:0.22390    C:0.30430    A:0.20994    G:0.26186

#8: Sus_scrofa
position  1:    T:0.16918    C:0.25126    A:0.24121    G:0.33836
position  2:    T:0.29816    C:0.24791    A:0.29481    G:0.15913
position  3:    T:0.17420    C:0.45226    A:0.08878    G:0.28476
Average         T:0.21385    C:0.31714    A:0.20826    G:0.26075

Sums of codon usage counts
------------------------------------------------------------------------------
Phe F TTT      80 | Ser S TCT      39 | Tyr Y TAT      52 | Cys C TGT      42
      TTC     125 |       TCC     133 |       TAC     120 |       TGC      80
Leu L TTA      14 |       TCA      18 | *** * TAA       0 | *** * TGA       0
      TTG      78 |       TCG      33 |       TAG       0 | Trp W TGG      56
------------------------------------------------------------------------------
Leu L CTT      51 | Pro P CCT      78 | His H CAT      34 | Arg R CGT      23
      CTC     119 |       CCC     130 |       CAC     118 |       CGC      49
      CTA      35 |       CCA      35 | Gln Q CAA      38 |       CGA       7
      CTG     208 |       CCG      42 |       CAG     138 |       CGG      25
------------------------------------------------------------------------------
Ile I ATT      60 | Thr T ACT      88 | Asn N AAT      72 | Ser S AGT      30
      ATC     209 |       ACC     119 |       AAC      65 |       AGC      73
      ATA      22 |       ACA      75 | Lys K AAA      25 | Arg R AGA      28
Met M ATG     112 |       ACG      67 |       AAG     110 |       AGG      36
------------------------------------------------------------------------------
Val V GTT      31 | Ala A GCT      94 | Asp D GAT     128 | Gly G GGT      51
      GTC     102 |       GCC     190 |       GAC     207 |       GGC     144
      GTA      22 |       GCA      29 | Glu E GAA      50 |       GGA      49
      GTG     151 |       GCG      20 |       GAG     221 |       GGG      96
------------------------------------------------------------------------------


Codon position x base (3x4) table, overall

position  1:    T:0.18216    C:0.23660    A:0.24937    G:0.33187
position  2:    T:0.29711    C:0.24916    A:0.28853    G:0.16520
position  3:    T:0.19954    C:0.41520    A:0.09359    G:0.29167
Average         T:0.22627    C:0.30032    A:0.21050    G:0.26291


Nei & Gojobori 1986. dN/dS (dN, dS)
(Note: This matrix is not used in later ML. analysis.
Use runmode = -2 for ML pairwise comparison.)

Orcinus_orca
Tursiops_truncatus   0.8408 (0.0178 0.0212)
Neophocaena_phocaenoides 1.0453 (0.0270 0.0258) 0.8334 (0.0255 0.0306)
Lipotes_vexillifer   0.8298 (0.0479 0.0577) 0.8313 (0.0479 0.0576) 0.7981 (0.0496 0.0621)
Balaenoptera_acutorostrata 0.7447 (0.0553 0.0743) 0.7507 (0.0538 0.0716) 0.7507 (0.0543 0.0723) 0.7527
(0.0527 0.0700)
Balaenoptera_physalus 0.6855 (0.0616 0.0899) 0.7105 (0.0629 0.0885) 0.6728 (0.0634 0.0943) 0.7024
(0.0582 0.0828) 0.5288 (0.0239 0.0453)
Bos_taurus           0.3522 (0.1025 0.2911) 0.3368 (0.0996 0.2958) 0.3597 (0.1011 0.2812) 0.3377
(0.0971 0.2877) 0.2775 (0.0729 0.2627) 0.2814 (0.0831 0.2952)
Sus_scrofa           0.2496 (0.0892 0.3574) 0.2370 (0.0848 0.3580) 0.2536 (0.0882 0.3479) 0.2408
(0.0840 0.3489) 0.1883 (0.0634 0.3365) 0.2189 (0.0730 0.3335) 0.1460 (0.0649 0.4443)


TREE #  1:  (((((1, 2), 3), 4), (5, 6)), 7, 8);   MP score: 489
lnL(ntime: 13  np: 14):  -5122.182036      +0.000000
   9..10   10..11   11..12   12..13   13..1    13..2    12..3    11..4    10..14   14..5    14..6
9..7     9..8
 0.096965 0.048306 0.042261 0.013858 0.031423 0.025798 0.043387 0.076292 0.024611 0.028559 0.061004
0.222692 0.223105 4.977184
```

Note: Branch length is defined as number of nucleotide substitutions per codon (not per neucleotide site).

tree length =   0.93826

(((((1: 0.031423, 2: 0.025798): 0.013858, 3: 0.043387): 0.042261, 4: 0.076292): 0.048306, (5: 0.028559, 6: 0.061004): 0.024611): 0.096965, 7: 0.222692, 8: 0.223105);

(((((Orcinus_orca: 0.031423, Tursiops_truncatus: 0.025798): 0.013858, Neophocaena_phocaenoides: 0.043387): 0.042261, Lipotes_vexillifer: 0.076292): 0.048306, (Balaenoptera_acutorostrata: 0.028559, Balaenoptera_physalus: 0.061004): 0.024611): 0.096965, Bos_taurus: 0.222692, Sus_scrofa: 0.223105);

Detailed output identifying parameters

kappa (ts/tv) = 4.97718

dN & dS for each branch

branch	t	N	S	dN/dS	dN	dS	N*dN	S*dS
9..10	0.097	1341.1	449.9	1.0000	0.0323	0.0323	43.3	14.5
10..11	0.048	1341.1	449.9	1.0000	0.0161	0.0161	21.6	7.2
11..12	0.042	1341.1	449.9	1.0000	0.0141	0.0141	18.9	6.3
12..13	0.014	1341.1	449.9	1.0000	0.0046	0.0046	6.2	2.1
13..1	0.031	1341.1	449.9	1.0000	0.0105	0.0105	14.0	4.7
13..2	0.026	1341.1	449.9	1.0000	0.0086	0.0086	11.5	3.9
12..3	0.043	1341.1	449.9	1.0000	0.0145	0.0145	19.4	6.5
11..4	0.076	1341.1	449.9	1.0000	0.0254	0.0254	34.1	11.4
10..14	0.025	1341.1	449.9	1.0000	0.0082	0.0082	11.0	3.7
14..5	0.029	1341.1	449.9	1.0000	0.0095	0.0095	12.8	4.3
14..6	0.061	1341.1	449.9	1.0000	0.0203	0.0203	27.3	9.1
9..7	0.223	1341.1	449.9	1.0000	0.0742	0.0742	99.6	33.4
9..8	0.223	1341.1	449.9	1.0000	0.0744	0.0744	99.7	33.5

tree length for dN: 0.3128
tree length for dS: 0.3128

Main result file for model C: whales-C.mlc

```
CODONML (in paml version 4.7b, October 2013)  whales.phy
Model: several dN/dS ratios for branches for branches,
Codon frequency model: F3x4
ns =   8  ls = 597

Codon usage in sequences
--------------------------------------------------------------------------------------------------------------------------------------
Phe TTT  10  10   9  11  10  10 | Ser TCT   6   5   5   5   4   5 | Tyr TAT   6   7   7   4   7   7 | Cys TGT   5   4   3   9   6   6
    TTC  13  14  12  15  18  16 |     TCC  16  17  20  18  16  16 |     TAC  15  15  15  16  13  14 |     TGC  11  12  11   9   9  10
Leu TTA   3   2   2   2   1   1 |     TCA   2   3   3   2   2   1 | *** TAA   0   0   0   0   0   0 | *** TGA   0   0   0   0   0   0
    TTG  12  12  12   9  10  10 |     TCG   4   4   4   4   5   6 |     TAG   0   0   0   0   0   0 | Trp TGG   7   7   8   7   7   7
--------------------------------------------------------------------------------------------------------------------------------------
Leu CTT   6   7   6   6   6   7 | Pro CCT   9   9   9  12  10  10 | His CAT   5   3   3   5   6   5 | Arg CGT   2   4   5   2   3   2
    CTC  14  12  14  13  16  18 |     CCC  14  14  15  14  17  15 |     CAC  15  15  15  15  16  16 |     CGC   6   4   6   6   7   4
    CTA   6   5   5   4   4   4 |     CCA   5   5   6   4   4   4 | Gln CAA   6   6   4   5   3   4 |     CGA   0   0   1   1   2   1
    CTG  26  23  27  28  24  26 |     CCG   4   7   3   6   7   5 |     CAG  18  18  16  19  16  16 |     CGG   3   3   3   2   2   4
--------------------------------------------------------------------------------------------------------------------------------------
Ile ATT   6   6   7   7   8   9 | Thr ACT  14  13  13  14  11  11 | Asn AAT  10   9  10  10   9   7 | Ser AGT   4   6   4   4   3   3
    ATC  27  25  26  25  26  27 |     ACC  16  16  14  13  12  14 |     AAC   8   8   8   9   7  10 |     AGC   9   9   8   6   9   9
    ATA   1   3   3   3   3   3 |     ACA   9   9  10  11  10  11 | Lys AAA   4   3   3   3   3   3 | Arg AGA   4   4   3   5   4   4
Met ATG  17  15  16  12  13  12 |     ACG   5   7   7   9  11   9 |     AAG  13  13  13  13  15  16 |     AGG   4   4   4   6   4   4
--------------------------------------------------------------------------------------------------------------------------------------
Val GTT   5   5   5   4   4   2 | Ala GCT  13  13  13  10  10  11 | Asp GAT  17  14  15  17  15  17 | Gly GGT   8   7   8   6   7   8
    GTC  11  13  13  12  12  12 |     GCC  23  23  22  23  27  25 |     GAC  25  28  27  22  23  24 |     GGC  16  17  17  22  18  15
    GTA   3   3   3   2   2   2 |     GCA   3   3   4   3   3   3 | Glu GAA   5   6   5   6   7   6 |     GGA   6   7   6   6   6   6
    GTG  21  20  19  22  18  18 |     GCG   2   3   1   2   4   5 |     GAG  27  26  27  27  30  30 |     GGG  12  12  14  10  12  11
--------------------------------------------------------------------------------------------------------------------------------------


------------------------------------------------------------------
Phe TTT  13   7 | Ser TCT   5   4 | Tyr TAT   5   9 | Cys TGT   6   3
    TTC  17  20 |     TCC  14  16 |     TAC  17  15 |     TGC   9   9
Leu TTA   1   2 |     TCA   3   2 | *** TAA   0   0 | *** TGA   0   0
    TTG   9   4 |     TCG   2   4 |     TAG   0   0 | Trp TGG   7   6
------------------------------------------------------------------
Leu CTT   5   8 | Pro CCT  11   8 | His CAT   2   5 | Arg CGT   4   1
    CTC  15  17 |     CCC  19  22 |     CAC  14  12 |     CGC   9   7
    CTA   5   2 |     CCA   4   3 | Gln CAA   4   6 |     CGA   0   2
    CTG  24  30 |     CCG   4   6 |     CAG  19  16 |     CGG   3   5
------------------------------------------------------------------
Ile ATT   7  10 | Thr ACT   9   3 | Asn AAT   8   9 | Ser AGT   2   4
    ATC  28  25 |     ACC  14  20 |     AAC   8   7 |     AGC  13  10
    ATA   3   3 |     ACA   9   6 | Lys AAA   2   4 | Arg AGA   2   2
Met ATG  12  15 |     ACG   9  10 |     AAG  15  12 |     AGG   6   4
------------------------------------------------------------------
Val GTT   4   2 | Ala GCT  10  14 | Asp GAT  18  15 | Gly GGT   5   2
    GTC  15  14 |     GCC  23  24 |     GAC  27  31 |     GGC  18  21
    GTA   5   2 |     GCA   5   5 | Glu GAA   7   8 |     GGA   6   6
    GTG  16  17 |     GCG   2   1 |     GAG  27  27 |     GGG  12  13
------------------------------------------------------------------

Codon position x base (3x4) table for each sequence.

#1: Orcinus_orca
position  1:    T:0.18425    C:0.23283    A:0.25293    G:0.32998
position  2:    T:0.30318    C:0.24288    A:0.29146    G:0.16248
position  3:    T:0.21106    C:0.40034    A:0.09548    G:0.29313
Average         T:0.23283    C:0.29202    A:0.21329    G:0.26186

#2: Tursiops_truncatus
position  1:    T:0.18760    C:0.22613    A:0.25126    G:0.33501
position  2:    T:0.29313    C:0.25293    A:0.28643    G:0.16750
position  3:    T:0.20436    C:0.40536    A:0.09883    G:0.29146
Average         T:0.22836    C:0.29481    A:0.21217    G:0.26466

#3: Neophocaena_phocaenoides
position  1:    T:0.18593    C:0.23116    A:0.24958    G:0.33333
position  2:    T:0.29983    C:0.24958    A:0.28141    G:0.16918
position  3:    T:0.20436    C:0.40704    A:0.09715    G:0.29146
Average         T:0.23004    C:0.29592    A:0.20938    G:0.26466

#4: Lipotes_vexillifer
position  1:    T:0.18593    C:0.23786    A:0.25126    G:0.32496
position  2:    T:0.29313    C:0.25126    A:0.28643    G:0.16918
position  3:    T:0.21106    C:0.39866    A:0.09548    G:0.29481
Average         T:0.23004    C:0.29592    A:0.21106    G:0.26298

#5: Balaenoptera_acutorostrata
position  1:    T:0.18090    C:0.23953    A:0.24791    G:0.33166
position  2:    T:0.29313    C:0.25628    A:0.28476    G:0.16583
position  3:    T:0.19933    C:0.41206    A:0.09045    G:0.29816
```

```
Average         T:0.22446    C:0.30262    A:0.20771    G:0.26521

#6: Balaenoptera_physalus
position  1:    T:0.18258    C:0.23618    A:0.25461    G:0.32663
position  2:    T:0.29648    C:0.25293    A:0.29313    G:0.15745
position  3:    T:0.20101    C:0.41039    A:0.08878    G:0.29983
Average         T:0.22669    C:0.29983    A:0.21217    G:0.26131

#7: Bos_taurus
position  1:    T:0.18090    C:0.23786    A:0.24623    G:0.33501
position  2:    T:0.29983    C:0.23953    A:0.28978    G:0.17085
position  3:    T:0.19095    C:0.43551    A:0.09380    G:0.27973
Average         T:0.22390    C:0.30430    A:0.20994    G:0.26186

#8: Sus_scrofa
position  1:    T:0.16918    C:0.25126    A:0.24121    G:0.33836
position  2:    T:0.29816    C:0.24791    A:0.29481    G:0.15913
position  3:    T:0.17420    C:0.45226    A:0.08878    G:0.28476
Average         T:0.21385    C:0.31714    A:0.20826    G:0.26075

Sums of codon usage counts
------------------------------------------------------------------------------
Phe F TTT      80 | Ser S TCT      39 | Tyr Y TAT      52 | Cys C TGT      42
      TTC     125 |       TCC     133 |       TAC     120 |       TGC      80
Leu L TTA      14 |       TCA      18 | *** * TAA       0 | *** * TGA       0
      TTG      78 |       TCG      33 |       TAG       0 | Trp W TGG      56
------------------------------------------------------------------------------
Leu L CTT      51 | Pro P CCT      78 | His H CAT      34 | Arg R CGT      23
      CTC     119 |       CCC     130 |       CAC     118 |       CGC      49
      CTA      35 |       CCA      35 | Gln Q CAA      38 |       CGA       7
      CTG     208 |       CCG      42 |       CAG     138 |       CGG      25
------------------------------------------------------------------------------
Ile I ATT      60 | Thr T ACT      88 | Asn N AAT      72 | Ser S AGT      30
      ATC     209 |       ACC     119 |       AAC      65 |       AGC      73
      ATA      22 |       ACA      75 | Lys K AAA      25 | Arg R AGA      28
Met M ATG     112 |       ACG      67 |       AAG     110 |       AGG      36
------------------------------------------------------------------------------
Val V GTT      31 | Ala A GCT      94 | Asp D GAT     128 | Gly G GGT      51
      GTC     102 |       GCC     190 |       GAC     207 |       GGC     144
      GTA      22 |       GCA      29 | Glu E GAA      50 |       GGA      49
      GTG     151 |       GCG      20 |       GAG     221 |       GGG      96
------------------------------------------------------------------------------


Codon position x base (3x4) table, overall

position  1:    T:0.18216    C:0.23660    A:0.24937    G:0.33187
position  2:    T:0.29711    C:0.24916    A:0.28853    G:0.16520
position  3:    T:0.19954    C:0.41520    A:0.09359    G:0.29167
Average         T:0.22627    C:0.30032    A:0.21050    G:0.26291


Nei & Gojobori 1986. dN/dS (dN, dS)
(Note: This matrix is not used in later ML. analysis.
Use runmode = -2 for ML pairwise comparison.)

Orcinus_orca
Tursiops_truncatus   0.8408 (0.0178 0.0212)
Neophocaena_phocaenoides 1.0453 (0.0270 0.0258) 0.8334 (0.0255 0.0306)
Lipotes_vexillifer   0.8298 (0.0479 0.0577) 0.8313 (0.0479 0.0576) 0.7981 (0.0496 0.0621)
Balaenoptera_acutorostrata 0.7447 (0.0553 0.0743) 0.7507 (0.0538 0.0716) 0.7507 (0.0543 0.0723) 0.7527
(0.0527 0.0700)
Balaenoptera_physalus 0.6855 (0.0616 0.0899) 0.7105 (0.0629 0.0885) 0.6728 (0.0634 0.0943) 0.7024
(0.0582 0.0828) 0.5288 (0.0239 0.0453)
Bos_taurus           0.3522 (0.1025 0.2911) 0.3368 (0.0996 0.2958) 0.3597 (0.1011 0.2812) 0.3377
(0.0971 0.2877) 0.2775 (0.0729 0.2627) 0.2814 (0.0831 0.2952)
Sus_scrofa           0.2496 (0.0892 0.3574) 0.2370 (0.0848 0.3580) 0.2536 (0.0882 0.3479) 0.2408
(0.0840 0.3489) 0.1883 (0.0634 0.3365) 0.2189 (0.0730 0.3335) 0.1460 (0.0649 0.4443)


TREE #  1:  (((((1, 2), 3), 4), (5, 6)), 7, 8);   MP score: 489
lnL(ntime: 13  np: 16):  -5013.940863      +0.000000
   9..10   10..11   11..12   12..13   13..1    13..2    12..3    11..4    10..14   14..5    14..6
9..7     9..8
 0.106307 0.048737 0.042640 0.013846 0.031366 0.025762 0.043292 0.075642 0.023719 0.028999 0.060460
0.243417 0.247255 4.211270 0.159259 0.742337
```

Note: Branch length is defined as number of nucleotide substitutions per codon (not per neucleotide site).

tree length = 0.99144

(((((1: 0.031366, 2: 0.025762): 0.013846, 3: 0.043292): 0.042640, 4: 0.075642): 0.048737, (5: 0.028999, 6: 0.060460): 0.023719): 0.106307, 7: 0.243417, 8: 0.247255);

(((((Orcinus_orca: 0.031366, Tursiops_truncatus: 0.025762): 0.013846, Neophocaena_phocaenoides: 0.043292): 0.042640, Lipotes_vexillifer: 0.075642): 0.048737, (Balaenoptera_acutorostrata: 0.028999, Balaenoptera_physalus: 0.060460): 0.023719): 0.106307, Bos_taurus: 0.243417, Sus_scrofa: 0.247255);

Detailed output identifying parameters

kappa (ts/tv) = 4.21127

w (dN/dS) for branches: 0.15926 0.74234

dN & dS for each branch

branch	t	N	S	dN/dS	dN	dS	N*dN	S*dS
9..10	0.106	1348.7	442.3	0.1593	0.0154	0.0966	20.7	42.7
10..11	0.049	1348.7	442.3	0.7423	0.0150	0.0202	20.2	8.9
11..12	0.043	1348.7	442.3	0.7423	0.0131	0.0176	17.7	7.8
12..13	0.014	1348.7	442.3	0.7423	0.0043	0.0057	5.7	2.5
13..1	0.031	1348.7	442.3	0.7423	0.0096	0.0130	13.0	5.7
13..2	0.026	1348.7	442.3	0.7423	0.0079	0.0107	10.7	4.7
12..3	0.043	1348.7	442.3	0.7423	0.0133	0.0179	17.9	7.9
11..4	0.076	1348.7	442.3	0.7423	0.0232	0.0313	31.3	13.8
10..14	0.024	1348.7	442.3	0.7423	0.0073	0.0098	9.8	4.3
14..5	0.029	1348.7	442.3	0.7423	0.0089	0.0120	12.0	5.3
14..6	0.060	1348.7	442.3	0.7423	0.0186	0.0250	25.0	11.1
9..7	0.243	1348.7	442.3	0.1593	0.0352	0.2211	47.5	97.8
9..8	0.247	1348.7	442.3	0.1593	0.0358	0.2246	48.2	99.4

tree length for dN: 0.2075
tree length for dS: 0.7055

dS tree:
(((((Orcinus_orca: 0.012972, Tursiops_truncatus: 0.010654): 0.005726, Neophocaena_phocaenoides: 0.017905): 0.017635, Lipotes_vexillifer: 0.031284): 0.020157, (Balaenoptera_acutorostrata: 0.011993, Balaenoptera_physalus: 0.025005): 0.009810): 0.096580, Bos_taurus: 0.221145, Sus_scrofa: 0.224632);
dN tree:
(((((Orcinus_orca: 0.009630, Tursiops_truncatus: 0.007909): 0.004251, Neophocaena_phocaenoides: 0.013291): 0.013091, Lipotes_vexillifer: 0.023223): 0.014963, (Balaenoptera_acutorostrata: 0.008903, Balaenoptera_physalus: 0.018562): 0.007282): 0.015381, Bos_taurus: 0.035219, Sus_scrofa: 0.035775);

w ratios as labels for TreeView:
(((((Orcinus_orca #0.7423 , Tursiops_truncatus #0.7423) #0.7423 , Neophocaena_phocaenoides #0.7423) #0.7423 , Lipotes_vexillifer #0.7423) #0.7423 , (Balaenoptera_acutorostrata #0.7423 , Balaenoptera_physalus #0.7423) #0.7423) #0.1593 , Bos_taurus #0.1593 , Sus_scrofa #0.1593);

Main result file for model D: whales-D.mlc

```
CODONML (in paml version 4.7b, October 2013)  whales.phy
Model: several dN/dS ratios for branches for branches,  omega = 1.000 fixed

Codon frequency model: F3x4
ns =   8  ls = 597

Codon usage in sequences
--------------------------------------------------------------------------------------------------------------------------------------
Phe TTT  10  10   9  11  10  10 | Ser TCT   6   5   5   5   4   5 | Tyr TAT   6   7   7   4   7   7 | Cys TGT   5   4   3   9   6   6
    TTC  13  14  12  15  18  16 |     TCC  16  17  20  18  16  16 |     TAC  15  15  15  16  13  14 |     TGC  11  12  11   9   9  10
Leu TTA   3   2   2   2   1   1 |     TCA   2   3   3   2   2   1 | *** TAA   0   0   0   0   0   0 | *** TGA   0   0   0   0   0   0
    TTG  12  12  12   9  10  10 |     TCG   4   4   4   4   5   6 |     TAG   0   0   0   0   0   0 | Trp TGG   7   7   8   7   7   7
--------------------------------------------------------------------------------------------------------------------------------------
Leu CTT   6   7   6   6   6   7 | Pro CCT   9   9   9  12  10  10 | His CAT   5   3   3   5   6   5 | Arg CGT   2   4   5   2   3   2
    CTC  14  12  14  13  16  18 |     CCC  14  14  15  14  17  15 |     CAC  15  15  15  15  16  16 |     CGC   6   4   6   6   7   4
    CTA   6   5   5   4   4   4 |     CCA   5   5   6   4   4   4 | Gln CAA   6   6   4   5   3   4 |     CGA   0   0   1   1   2   1
    CTG  26  23  27  28  24  26 |     CCG   4   7   3   6   7   5 |     CAG  18  18  16  19  16  16 |     CGG   3   3   3   2   2   4
--------------------------------------------------------------------------------------------------------------------------------------
Ile ATT   6   6   7   7   8   9 | Thr ACT  14  13  13  14  11  11 | Asn AAT  10   9  10  10   9   7 | Ser AGT   4   6   4   4   3   3
    ATC  27  25  26  25  26  27 |     ACC  16  16  14  13  12  14 |     AAC   8   8   8   9   7  10 |     AGC   9   9   8   6   9   9
    ATA   1   3   3   3   3   3 |     ACA   9   9  10  11  10  11 | Lys AAA   4   3   3   3   3   3 | Arg AGA   4   4   3   5   4   4
Met ATG  17  15  16  12  13  12 |     ACG   5   7   7   9  11   9 |     AAG  13  13  13  13  15  16 |     AGG   4   4   4   6   4   4
--------------------------------------------------------------------------------------------------------------------------------------
Val GTT   5   5   5   4   4   2 | Ala GCT  13  13  13  10  10  11 | Asp GAT  17  14  15  17  15  17 | Gly GGT   8   7   8   6   7   8
    GTC  11  13  13  12  12  12 |     GCC  23  23  22  23  27  25 |     GAC  25  28  27  22  23  24 |     GGC  16  17  17  22  18  15
    GTA   3   3   3   2   2   2 |     GCA   3   3   4   3   3   3 | Glu GAA   5   6   5   6   7   6 |     GGA   6   7   6   6   6   6
    GTG  21  20  19  22  18  18 |     GCG   2   3   1   2   4   5 |     GAG  27  26  27  27  30  30 |     GGG  12  12  14  10  12  11
--------------------------------------------------------------------------------------------------------------------------------------


--------------------------------------------------------------------
Phe TTT  13   7 | Ser TCT   5   4 | Tyr TAT   5   9 | Cys TGT   6   3
    TTC  17  20 |     TCC  14  16 |     TAC  17  15 |     TGC   9   9
Leu TTA   1   2 |     TCA   3   2 | *** TAA   0   0 | *** TGA   0   0
    TTG   9   4 |     TCG   2   4 |     TAG   0   0 | Trp TGG   7   6
--------------------------------------------------------------------
Leu CTT   5   8 | Pro CCT  11   8 | His CAT   2   5 | Arg CGT   4   1
    CTC  15  17 |     CCC  19  22 |     CAC  14  12 |     CGC   9   7
    CTA   5   2 |     CCA   4   3 | Gln CAA   4   6 |     CGA   0   2
    CTG  24  30 |     CCG   4   6 |     CAG  19  16 |     CGG   3   5
--------------------------------------------------------------------
Ile ATT   7  10 | Thr ACT   9   3 | Asn AAT   8   9 | Ser AGT   2   4
    ATC  28  25 |     ACC  14  20 |     AAC   8   7 |     AGC  13  10
    ATA   3   3 |     ACA   9   6 | Lys AAA   2   4 | Arg AGA   2   2
Met ATG  12  15 |     ACG   9  10 |     AAG  15  12 |     AGG   6   4
--------------------------------------------------------------------
Val GTT   4   2 | Ala GCT  10  14 | Asp GAT  18  15 | Gly GGT   5   2
    GTC  15  14 |     GCC  23  24 |     GAC  27  31 |     GGC  18  21
    GTA   5   2 |     GCA   5   5 | Glu GAA   7   8 |     GGA   6   6
    GTG  16  17 |     GCG   2   1 |     GAG  27  27 |     GGG  12  13
--------------------------------------------------------------------

Codon position x base (3x4) table for each sequence.

#1: Orcinus_orca
position  1:    T:0.18425    C:0.23283    A:0.25293    G:0.32998
position  2:    T:0.30318    C:0.24288    A:0.29146    G:0.16248
position  3:    T:0.21106    C:0.40034    A:0.09548    G:0.29313
Average         T:0.23283    C:0.29202    A:0.21329    G:0.26186

#2: Tursiops_truncatus
position  1:    T:0.18760    C:0.22613    A:0.25126    G:0.33501
position  2:    T:0.29313    C:0.25293    A:0.28643    G:0.16750
position  3:    T:0.20436    C:0.40536    A:0.09883    G:0.29146
Average         T:0.22836    C:0.29481    A:0.21217    G:0.26466

#3: Neophocaena_phocaenoides
position  1:    T:0.18593    C:0.23116    A:0.24958    G:0.33333
position  2:    T:0.29983    C:0.24958    A:0.28141    G:0.16918
position  3:    T:0.20436    C:0.40704    A:0.09715    G:0.29146
Average         T:0.23004    C:0.29592    A:0.20938    G:0.26466

#4: Lipotes_vexillifer
position  1:    T:0.18593    C:0.23786    A:0.25126    G:0.32496
position  2:    T:0.29313    C:0.25126    A:0.28643    G:0.16918
position  3:    T:0.21106    C:0.39866    A:0.09548    G:0.29481
Average         T:0.23004    C:0.29592    A:0.21106    G:0.26298

#5: Balaenoptera_acutorostrata
position  1:    T:0.18090    C:0.23953    A:0.24791    G:0.33166
position  2:    T:0.29313    C:0.25628    A:0.28476    G:0.16583
```

```
position  3:    T:0.19933    C:0.41206    A:0.09045    G:0.29816
Average         T:0.22446    C:0.30262    A:0.20771    G:0.26521

#6: Balaenoptera_physalus
position  1:    T:0.18258    C:0.23618    A:0.25461    G:0.32663
position  2:    T:0.29648    C:0.25293    A:0.29313    G:0.15745
position  3:    T:0.20101    C:0.41039    A:0.08878    G:0.29983
Average         T:0.22669    C:0.29983    A:0.21217    G:0.26131

#7: Bos_taurus
position  1:    T:0.18090    C:0.23786    A:0.24623    G:0.33501
position  2:    T:0.29983    C:0.23953    A:0.28978    G:0.17085
position  3:    T:0.19095    C:0.43551    A:0.09380    G:0.27973
Average         T:0.22390    C:0.30430    A:0.20994    G:0.26186

#8: Sus_scrofa
position  1:    T:0.16918    C:0.25126    A:0.24121    G:0.33836
position  2:    T:0.29816    C:0.24791    A:0.29481    G:0.15913
position  3:    T:0.17420    C:0.45226    A:0.08878    G:0.28476
Average         T:0.21385    C:0.31714    A:0.20826    G:0.26075

Sums of codon usage counts
------------------------------------------------------------------------------
Phe F TTT      80 | Ser S TCT      39 | Tyr Y TAT      52 | Cys C TGT      42
      TTC     125 |       TCC     133 |       TAC     120 |       TGC      80
Leu L TTA      14 |       TCA      18 | *** * TAA       0 | *** * TGA       0
      TTG      78 |       TCG      33 |       TAG       0 | Trp W TGG      56
------------------------------------------------------------------------------
Leu L CTT      51 | Pro P CCT      78 | His H CAT      34 | Arg R CGT      23
      CTC     119 |       CCC     130 |       CAC     118 |       CGC      49
      CTA      35 |       CCA      35 | Gln Q CAA      38 |       CGA       7
      CTG     208 |       CCG      42 |       CAG     138 |       CGG      25
------------------------------------------------------------------------------
Ile I ATT      60 | Thr T ACT      88 | Asn N AAT      72 | Ser S AGT      30
      ATC     209 |       ACC     119 |       AAC      65 |       AGC      73
      ATA      22 |       ACA      75 | Lys K AAA      25 | Arg R AGA      28
Met M ATG     112 |       ACG      67 |       AAG     110 |       AGG      36
------------------------------------------------------------------------------
Val V GTT      31 | Ala A GCT      94 | Asp D GAT     128 | Gly G GGT      51
      GTC     102 |       GCC     190 |       GAC     207 |       GGC     144
      GTA      22 |       GCA      29 | Glu E GAA      50 |       GGA      49
      GTG     151 |       GCG      20 |       GAG     221 |       GGG      96
------------------------------------------------------------------------------


Codon position x base (3x4) table, overall

position  1:    T:0.18216    C:0.23660    A:0.24937    G:0.33187
position  2:    T:0.29711    C:0.24916    A:0.28853    G:0.16520
position  3:    T:0.19954    C:0.41520    A:0.09359    G:0.29167
Average         T:0.22627    C:0.30032    A:0.21050    G:0.26291


Nei & Gojobori 1986. dN/dS (dN, dS)
(Note: This matrix is not used in later ML. analysis.
Use runmode = -2 for ML pairwise comparison.)

Orcinus_orca
Tursiops_truncatus   0.8408 (0.0178 0.0212)
Neophocaena_phocaenoides 1.0453 (0.0270 0.0258) 0.8334 (0.0255 0.0306)
Lipotes_vexillifer   0.8298 (0.0479 0.0577) 0.8313 (0.0479 0.0576) 0.7981 (0.0496 0.0621)
Balaenoptera_acutorostrata 0.7447 (0.0553 0.0743) 0.7507 (0.0538 0.0716) 0.7507 (0.0543 0.0723) 0.7527
(0.0527 0.0700)
Balaenoptera_physalus 0.6855 (0.0616 0.0899) 0.7105 (0.0629 0.0885) 0.6728 (0.0634 0.0943) 0.7024
(0.0582 0.0828) 0.5288 (0.0239 0.0453)
Bos_taurus           0.3522 (0.1025 0.2911) 0.3368 (0.0996 0.2958) 0.3597 (0.1011 0.2812) 0.3377
(0.0971 0.2877) 0.2775 (0.0729 0.2627) 0.2814 (0.0831 0.2952)
Sus_scrofa           0.2496 (0.0892 0.3574) 0.2370 (0.0848 0.3580) 0.2536 (0.0882 0.3479) 0.2408
(0.0840 0.3489) 0.1883 (0.0634 0.3365) 0.2189 (0.0730 0.3335) 0.1460 (0.0649 0.4443)


TREE #  1:  (((((1, 2), 3), 4), (5, 6)), 7, 8);   MP score: 489
lnL(ntime: 13  np: 15):  -5015.893325      +0.000000
   9..10    10..11   11..12   12..13   13..1    13..2    12..3    11..4    10..14   14..5    14..6
9..7     9..8
 0.106310 0.048454 0.042752 0.013802 0.031387 0.025770 0.043341 0.075546 0.023700 0.029083 0.060244
0.243947 0.247447 4.298470 0.159382
```

Note: Branch length is defined as number of nucleotide substitutions per codon (not per neucleotide site).

tree length = 0.99178

(((((1: 0.031387, 2: 0.025770): 0.013802, 3: 0.043341): 0.042752, 4: 0.075546): 0.048454, (5: 0.029083, 6: 0.060244): 0.023700): 0.106310, 7: 0.243947, 8: 0.247447);

(((((Orcinus_orca: 0.031387, Tursiops_truncatus: 0.025770): 0.013802, Neophocaena_phocaenoides: 0.043341): 0.042752, Lipotes_vexillifer: 0.075546): 0.048454, (Balaenoptera_acutorostrata: 0.029083, Balaenoptera_physalus: 0.060244): 0.023700): 0.106310, Bos_taurus: 0.243947, Sus_scrofa: 0.247447);

Detailed output identifying parameters

kappa (ts/tv) = 4.29847

w (dN/dS) for branches: 0.15938 1.00000

dN & dS for each branch

branch	t	N	S	dN/dS	dN	dS	N*dN	S*dS
9..10	0.106	1347.7	443.3	0.1594	0.0154	0.0964	20.7	42.8
10..11	0.048	1347.7	443.3	1.0000	0.0162	0.0162	21.8	7.2
11..12	0.043	1347.7	443.3	1.0000	0.0143	0.0143	19.2	6.3
12..13	0.014	1347.7	443.3	1.0000	0.0046	0.0046	6.2	2.0
13..1	0.031	1347.7	443.3	1.0000	0.0105	0.0105	14.1	4.6
13..2	0.026	1347.7	443.3	1.0000	0.0086	0.0086	11.6	3.8
12..3	0.043	1347.7	443.3	1.0000	0.0144	0.0144	19.5	6.4
11..4	0.076	1347.7	443.3	1.0000	0.0252	0.0252	33.9	11.2
10..14	0.024	1347.7	443.3	1.0000	0.0079	0.0079	10.6	3.5
14..5	0.029	1347.7	443.3	1.0000	0.0097	0.0097	13.1	4.3
14..6	0.060	1347.7	443.3	1.0000	0.0201	0.0201	27.1	8.9
9..7	0.244	1347.7	443.3	0.1594	0.0353	0.2213	47.5	98.1
9..8	0.247	1347.7	443.3	0.1594	0.0358	0.2245	48.2	99.5

tree length for dN: 0.2178
tree length for dS: 0.6736

dS tree:
(((((Orcinus_orca: 0.010462, Tursiops_truncatus: 0.008590): 0.004601, Neophocaena_phocaenoides: 0.014447): 0.014251, Lipotes_vexillifer: 0.025182): 0.016151, (Balaenoptera_acutorostrata: 0.009694, Balaenoptera_physalus: 0.020081): 0.007900): 0.096441, Bos_taurus: 0.221301, Sus_scrofa: 0.224476);
dN tree:
(((((Orcinus_orca: 0.010462, Tursiops_truncatus: 0.008590): 0.004601, Neophocaena_phocaenoides: 0.014447): 0.014251, Lipotes_vexillifer: 0.025182): 0.016151, (Balaenoptera_acutorostrata: 0.009694, Balaenoptera_physalus: 0.020081): 0.007900): 0.015371, Bos_taurus: 0.035271, Sus_scrofa: 0.035777);

w ratios as labels for TreeView:
(((((Orcinus_orca #1.0000 , Tursiops_truncatus #1.0000) #1.0000 , Neophocaena_phocaenoides #1.0000) #1.0000 , Lipotes_vexillifer #1.0000) #1.0000 , (Balaenoptera_acutorostrata #1.0000 , Balaenoptera_physalus #1.0000) #1.0000) #0.1594 , Bos_taurus #0.1594 , Sus_scrofa #0.1594);

Main result file for model E: whales-E.mlc

```
CODONML (in paml version 4.7b, October 2013)  whales.phy
Model: several dN/dS ratios for branches for branches,
Codon frequency model: F3x4
ns =   8  ls = 597

Codon usage in sequences
--------------------------------------------------------------------------------------------------------------------------------------
Phe TTT  10  10   9  11  10  10 | Ser TCT   6   5   5   5   4   5 | Tyr TAT   6   7   7   4   7   7 | Cys TGT   5   4   3   9   6   6
    TTC  13  14  12  15  18  16 |     TCC  16  17  20  18  16  16 |     TAC  15  15  15  16  13  14 |     TGC  11  12  11   9   9  10
Leu TTA   3   2   2   2   1   1 |     TCA   2   3   3   2   2   1 | *** TAA   0   0   0   0   0   0 | *** TGA   0   0   0   0   0   0
    TTG  12  12  12   9  10  10 |     TCG   4   4   4   4   5   6 |     TAG   0   0   0   0   0   0 | Trp TGG   7   7   8   7   7   7
--------------------------------------------------------------------------------------------------------------------------------------
Leu CTT   6   7   6   6   6   7 | Pro CCT   9   9   9  12  10  10 | His CAT   5   3   3   5   6   5 | Arg CGT   2   4   5   2   3   2
    CTC  14  12  14  13  16  18 |     CCC  14  14  15  14  17  15 |     CAC  15  15  15  15  16  16 |     CGC   6   4   6   6   7   4
    CTA   6   5   5   4   4   4 |     CCA   5   5   6   4   4   4 | Gln CAA   6   6   4   5   3   4 |     CGA   0   0   1   1   2   1
    CTG  26  23  27  28  24  26 |     CCG   4   7   3   6   7   5 |     CAG  18  18  16  19  16  16 |     CGG   3   3   3   2   2   4
--------------------------------------------------------------------------------------------------------------------------------------
Ile ATT   6   6   7   7   8   9 | Thr ACT  14  13  13  14  11  11 | Asn AAT  10   9  10  10   9   7 | Ser AGT   4   6   4   4   3   3
    ATC  27  25  26  25  26  27 |     ACC  16  16  14  13  12  14 |     AAC   8   8   8   9   7  10 |     AGC   9   9   8   6   9   9
    ATA   1   3   3   3   3   3 |     ACA   9   9  10  11  10  11 | Lys AAA   4   3   3   3   3   3 | Arg AGA   4   4   3   5   4   4
Met ATG  17  15  16  12  13  12 |     ACG   5   7   7   9  11   9 |     AAG  13  13  13  13  15  16 |     AGG   4   4   4   6   4   4
--------------------------------------------------------------------------------------------------------------------------------------
Val GTT   5   5   5   4   4   2 | Ala GCT  13  13  13  10  10  11 | Asp GAT  17  14  15  17  15  17 | Gly GGT   8   7   8   6   7   8
    GTC  11  13  13  12  12  12 |     GCC  23  23  22  23  27  25 |     GAC  25  28  27  22  23  24 |     GGC  16  17  17  22  18  15
    GTA   3   3   3   2   2   2 |     GCA   3   3   4   3   3   3 | Glu GAA   5   6   5   6   7   6 |     GGA   6   7   6   6   6   6
    GTG  21  20  19  22  18  18 |     GCG   2   3   1   2   4   5 |     GAG  27  26  27  27  30  30 |     GGG  12  12  14  10  12  11
--------------------------------------------------------------------------------------------------------------------------------------


----------------------------------------------------------------------
Phe TTT  13   7 | Ser TCT   5   4 | Tyr TAT   5   9 | Cys TGT   6   3
    TTC  17  20 |     TCC  14  16 |     TAC  17  15 |     TGC   9   9
Leu TTA   1   2 |     TCA   3   2 | *** TAA   0   0 | *** TGA   0   0
    TTG   9   4 |     TCG   2   4 |     TAG   0   0 | Trp TGG   7   6
----------------------------------------------------------------------
Leu CTT   5   8 | Pro CCT  11   8 | His CAT   2   5 | Arg CGT   4   1
    CTC  15  17 |     CCC  19  22 |     CAC  14  12 |     CGC   9   7
    CTA   5   2 |     CCA   4   3 | Gln CAA   4   6 |     CGA   0   2
    CTG  24  30 |     CCG   4   6 |     CAG  19  16 |     CGG   3   5
----------------------------------------------------------------------
Ile ATT   7  10 | Thr ACT   9   3 | Asn AAT   8   9 | Ser AGT   2   4
    ATC  28  25 |     ACC  14  20 |     AAC   8   7 |     AGC  13  10
    ATA   3   3 |     ACA   9   6 | Lys AAA   2   4 | Arg AGA   2   2
Met ATG  12  15 |     ACG   9  10 |     AAG  15  12 |     AGG   6   4
----------------------------------------------------------------------
Val GTT   4   2 | Ala GCT  10  14 | Asp GAT  18  15 | Gly GGT   5   2
    GTC  15  14 |     GCC  23  24 |     GAC  27  31 |     GGC  18  21
    GTA   5   2 |     GCA   5   5 | Glu GAA   7   8 |     GGA   6   6
    GTG  16  17 |     GCG   2   1 |     GAG  27  27 |     GGG  12  13
----------------------------------------------------------------------

Codon position x base (3x4) table for each sequence.

#1: Orcinus_orca
position  1:    T:0.18425    C:0.23283    A:0.25293    G:0.32998
position  2:    T:0.30318    C:0.24288    A:0.29146    G:0.16248
position  3:    T:0.21106    C:0.40034    A:0.09548    G:0.29313
Average         T:0.23283    C:0.29202    A:0.21329    G:0.26186

#2: Tursiops_truncatus
position  1:    T:0.18760    C:0.22613    A:0.25126    G:0.33501
position  2:    T:0.29313    C:0.25293    A:0.28643    G:0.16750
position  3:    T:0.20436    C:0.40536    A:0.09883    G:0.29146
Average         T:0.22836    C:0.29481    A:0.21217    G:0.26466

#3: Neophocaena_phocaenoides
position  1:    T:0.18593    C:0.23116    A:0.24958    G:0.33333
position  2:    T:0.29983    C:0.24958    A:0.28141    G:0.16918
position  3:    T:0.20436    C:0.40704    A:0.09715    G:0.29146
Average         T:0.23004    C:0.29592    A:0.20938    G:0.26466

#4: Lipotes_vexillifer
position  1:    T:0.18593    C:0.23786    A:0.25126    G:0.32496
position  2:    T:0.29313    C:0.25126    A:0.28643    G:0.16918
position  3:    T:0.21106    C:0.39866    A:0.09548    G:0.29481
Average         T:0.23004    C:0.29592    A:0.21106    G:0.26298

#5: Balaenoptera_acutorostrata
position  1:    T:0.18090    C:0.23953    A:0.24791    G:0.33166
position  2:    T:0.29313    C:0.25628    A:0.28476    G:0.16583
position  3:    T:0.19933    C:0.41206    A:0.09045    G:0.29816
```

```
Average         T:0.22446    C:0.30262    A:0.20771    G:0.26521

#6: Balaenoptera_physalus
position  1:    T:0.18258    C:0.23618    A:0.25461    G:0.32663
position  2:    T:0.29648    C:0.25293    A:0.29313    G:0.15745
position  3:    T:0.20101    C:0.41039    A:0.08878    G:0.29983
Average         T:0.22669    C:0.29983    A:0.21217    G:0.26131

#7: Bos_taurus
position  1:    T:0.18090    C:0.23786    A:0.24623    G:0.33501
position  2:    T:0.29983    C:0.23953    A:0.28978    G:0.17085
position  3:    T:0.19095    C:0.43551    A:0.09380    G:0.27973
Average         T:0.22390    C:0.30430    A:0.20994    G:0.26186

#8: Sus_scrofa
position  1:    T:0.16918    C:0.25126    A:0.24121    G:0.33836
position  2:    T:0.29816    C:0.24791    A:0.29481    G:0.15913
position  3:    T:0.17420    C:0.45226    A:0.08878    G:0.28476
Average         T:0.21385    C:0.31714    A:0.20826    G:0.26075

Sums of codon usage counts
------------------------------------------------------------------------------
Phe F TTT      80 | Ser S TCT      39 | Tyr Y TAT      52 | Cys C TGT      42
      TTC     125 |       TCC     133 |       TAC     120 |       TGC      80
Leu L TTA      14 |       TCA      18 | *** * TAA       0 | *** * TGA       0
      TTG      78 |       TCG      33 |       TAG       0 | Trp W TGG      56
------------------------------------------------------------------------------
Leu L CTT      51 | Pro P CCT      78 | His H CAT      34 | Arg R CGT      23
      CTC     119 |       CCC     130 |       CAC     118 |       CGC      49
      CTA      35 |       CCA      35 | Gln Q CAA      38 |       CGA       7
      CTG     208 |       CCG      42 |       CAG     138 |       CGG      25
------------------------------------------------------------------------------
Ile I ATT      60 | Thr T ACT      88 | Asn N AAT      72 | Ser S AGT      30
      ATC     209 |       ACC     119 |       AAC      65 |       AGC      73
      ATA      22 |       ACA      75 | Lys K AAA      25 | Arg R AGA      28
Met M ATG     112 |       ACG      67 |       AAG     110 |       AGG      36
------------------------------------------------------------------------------
Val V GTT      31 | Ala A GCT      94 | Asp D GAT     128 | Gly G GGT      51
      GTC     102 |       GCC     190 |       GAC     207 |       GGC     144
      GTA      22 |       GCA      29 | Glu E GAA      50 |       GGA      49
      GTG     151 |       GCG      20 |       GAG     221 |       GGG      96
------------------------------------------------------------------------------


Codon position x base (3x4) table, overall

position  1:    T:0.18216    C:0.23660    A:0.24937    G:0.33187
position  2:    T:0.29711    C:0.24916    A:0.28853    G:0.16520
position  3:    T:0.19954    C:0.41520    A:0.09359    G:0.29167
Average         T:0.22627    C:0.30032    A:0.21050    G:0.26291


Nei & Gojobori 1986. dN/dS (dN, dS)
(Note: This matrix is not used in later ML. analysis.
Use runmode = -2 for ML pairwise comparison.)

Orcinus_orca
Tursiops_truncatus   0.8408 (0.0178 0.0212)
Neophocaena_phocaenoides 1.0453 (0.0270 0.0258) 0.8334 (0.0255 0.0306)
Lipotes_vexillifer   0.8298 (0.0479 0.0577) 0.8313 (0.0479 0.0576) 0.7981 (0.0496 0.0621)
Balaenoptera_acutorostrata 0.7447 (0.0553 0.0743) 0.7507 (0.0538 0.0716) 0.7507 (0.0543 0.0723) 0.7527
(0.0527 0.0700)
Balaenoptera_physalus 0.6855 (0.0616 0.0899) 0.7105 (0.0629 0.0885) 0.6728 (0.0634 0.0943) 0.7024
(0.0582 0.0828) 0.5288 (0.0239 0.0453)
Bos_taurus           0.3522 (0.1025 0.2911) 0.3368 (0.0996 0.2958) 0.3597 (0.1011 0.2812) 0.3377
(0.0971 0.2877) 0.2775 (0.0729 0.2627) 0.2814 (0.0831 0.2952)
Sus_scrofa           0.2496 (0.0892 0.3574) 0.2370 (0.0848 0.3580) 0.2536 (0.0882 0.3479) 0.2408
(0.0840 0.3489) 0.1883 (0.0634 0.3365) 0.2189 (0.0730 0.3335) 0.1460 (0.0649 0.4443)


TREE #  1:  (((((1, 2), 3), 4), (5, 6)), 7, 8);   MP score: 489
lnL(ntime: 13  np: 16):  -5011.673318      +0.000000
   9..10   10..11   11..12   12..13   13..1    13..2    12..3    11..4    10..14   14..5    14..6
9..7     9..8
 0.079138 0.048547 0.042436 0.013868 0.031367 0.025759 0.043283 0.075861 0.023862 0.028856 0.060679
0.263204 0.267419 4.217912 0.124715 0.697209
```

Note: Branch length is defined as number of nucleotide substitutions per codon (not per neucleotide site).

tree length =   1.00428

(((((1: 0.031367, 2: 0.025759): 0.013868, 3: 0.043283): 0.042436, 4: 0.075861): 0.048547, (5: 0.028856, 6: 0.060679): 0.023862): 0.079138, 7: 0.263204, 8: 0.267419);

(((((Orcinus_orca: 0.031367, Tursiops_truncatus: 0.025759): 0.013868, Neophocaena_phocaenoides: 0.043283): 0.042436, Lipotes_vexillifer: 0.075861): 0.048547, (Balaenoptera_acutorostrata: 0.028856, Balaenoptera_physalus: 0.060679): 0.023862): 0.079138, Bos_taurus: 0.263204, Sus_scrofa: 0.267419);

Detailed output identifying parameters

kappa (ts/tv) = 4.21791

w (dN/dS) for branches: 0.12471 0.69721

dN & dS for each branch

branch	t	N	S	dN/dS	dN	dS	N*dN	S*dS
9..10	0.079	1348.6	442.4	0.6972	0.0238	0.0342	32.1	15.1
10..11	0.049	1348.6	442.4	0.6972	0.0146	0.0210	19.7	9.3
11..12	0.042	1348.6	442.4	0.6972	0.0128	0.0183	17.2	8.1
12..13	0.014	1348.6	442.4	0.6972	0.0042	0.0060	5.6	2.6
13..1	0.031	1348.6	442.4	0.6972	0.0094	0.0135	12.7	6.0
13..2	0.026	1348.6	442.4	0.6972	0.0078	0.0111	10.5	4.9
12..3	0.043	1348.6	442.4	0.6972	0.0130	0.0187	17.6	8.3
11..4	0.076	1348.6	442.4	0.6972	0.0228	0.0328	30.8	14.5
10..14	0.024	1348.6	442.4	0.6972	0.0072	0.0103	9.7	4.6
14..5	0.029	1348.6	442.4	0.6972	0.0087	0.0125	11.7	5.5
14..6	0.061	1348.6	442.4	0.6972	0.0183	0.0262	24.6	11.6
9..7	0.263	1348.6	442.4	0.1247	0.0321	0.2573	43.3	113.9
9..8	0.267	1348.6	442.4	0.1247	0.0326	0.2615	44.0	115.7

tree length for dN: 0.2073
tree length for dS: 0.7233

dS tree:
(((((Orcinus_orca: 0.013543, Tursiops_truncatus: 0.011122): 0.005988, Neophocaena_phocaenoides: 0.018689): 0.018323, Lipotes_vexillifer: 0.032755): 0.020961, (Balaenoptera_acutorostrata: 0.012459, Balaenoptera_physalus: 0.026200): 0.010303): 0.034170, Bos_taurus: 0.257341, Sus_scrofa: 0.261462);
dN tree:
(((((Orcinus_orca: 0.009443, Tursiops_truncatus: 0.007754): 0.004175, Neophocaena_phocaenoides: 0.013030): 0.012775, Lipotes_vexillifer: 0.022837): 0.014614, (Balaenoptera_acutorostrata: 0.008687, Balaenoptera_physalus: 0.018267): 0.007183): 0.023824, Bos_taurus: 0.032094, Sus_scrofa: 0.032608);

w ratios as labels for TreeView:
(((((Orcinus_orca #0.6972 , Tursiops_truncatus #0.6972) #0.6972 , Neophocaena_phocaenoides #0.6972) #0.6972 , Lipotes_vexillifer #0.6972) #0.6972 , (Balaenoptera_acutorostrata #0.6972 , Balaenoptera_physalus #0.6972) #0.6972) #0.6972 , Bos_taurus #0.1247 , Sus_scrofa #0.1247);

Main result file for model F: whales-F.mlc

```
CODONML (in paml version 4.7b, October 2013)  whales.phy
Model: several dN/dS ratios for branches for branches,  omega = 1.000 fixed

Codon frequency model: F3x4
ns =   8  ls = 597

Codon usage in sequences
--------------------------------------------------------------------------------------------------------------------------------------
Phe TTT  10  10   9  11  10  10 | Ser TCT   6   5   5   5   4   5 | Tyr TAT   6   7   7   4   7   7 | Cys TGT   5   4   3   9   6   6
    TTC  13  14  12  15  18  16 |     TCC  16  17  20  18  16  16 |     TAC  15  15  15  16  13  14 |     TGC  11  12  11   9   9  10
Leu TTA   3   2   2   2   1   1 |     TCA   2   3   3   2   2   1 | *** TAA   0   0   0   0   0   0 | *** TGA   0   0   0   0   0   0
    TTG  12  12  12   9  10  10 |     TCG   4   4   4   4   5   6 |     TAG   0   0   0   0   0   0 | Trp TGG   7   7   8   7   7   7
--------------------------------------------------------------------------------------------------------------------------------------
Leu CTT   6   7   6   6   6   7 | Pro CCT   9   9   9  12  10  10 | His CAT   5   3   3   5   6   5 | Arg CGT   2   4   5   2   3   2
    CTC  14  12  14  13  16  18 |     CCC  14  14  15  14  17  15 |     CAC  15  15  15  15  16  16 |     CGC   6   4   6   6   7   4
    CTA   6   5   5   4   4   4 |     CCA   5   5   6   4   4   4 | Gln CAA   6   6   4   5   3   4 |     CGA   0   0   1   1   2   1
    CTG  26  23  27  28  24  26 |     CCG   4   7   3   6   7   5 |     CAG  18  18  16  19  16  16 |     CGG   3   3   3   2   2   4
--------------------------------------------------------------------------------------------------------------------------------------
Ile ATT   6   6   7   7   8   9 | Thr ACT  14  13  13  14  11  11 | Asn AAT  10   9  10  10   9   7 | Ser AGT   4   6   4   4   3   3
    ATC  27  25  26  25  26  27 |     ACC  16  16  14  13  12  14 |     AAC   8   8   8   9   7  10 |     AGC   9   9   8   6   9   9
    ATA   1   3   3   3   3   3 |     ACA   9   9  10  11  10  11 | Lys AAA   4   3   3   3   3   3 | Arg AGA   4   4   3   5   4   4
Met ATG  17  15  16  12  13  12 |     ACG   5   7   7   9  11   9 |     AAG  13  13  13  13  15  16 |     AGG   4   4   4   6   4   4
--------------------------------------------------------------------------------------------------------------------------------------
Val GTT   5   5   5   4   4   2 | Ala GCT  13  13  13  10  10  11 | Asp GAT  17  14  15  17  15  17 | Gly GGT   8   7   8   6   7   8
    GTC  11  13  13  12  12  12 |     GCC  23  23  22  23  27  25 |     GAC  25  28  27  22  23  24 |     GGC  16  17  17  22  18  15
    GTA   3   3   3   2   2   2 |     GCA   3   3   4   3   3   3 | Glu GAA   5   6   5   6   7   6 |     GGA   6   7   6   6   6   6
    GTG  21  20  19  22  18  18 |     GCG   2   3   1   2   4   5 |     GAG  27  26  27  27  30  30 |     GGG  12  12  14  10  12  11
--------------------------------------------------------------------------------------------------------------------------------------


------------------------------------------------------------------
Phe TTT  13   7 | Ser TCT   5   4 | Tyr TAT   5   9 | Cys TGT   6   3
    TTC  17  20 |     TCC  14  16 |     TAC  17  15 |     TGC   9   9
Leu TTA   1   2 |     TCA   3   2 | *** TAA   0   0 | *** TGA   0   0
    TTG   9   4 |     TCG   2   4 |     TAG   0   0 | Trp TGG   7   6
------------------------------------------------------------------
Leu CTT   5   8 | Pro CCT  11   8 | His CAT   2   5 | Arg CGT   4   1
    CTC  15  17 |     CCC  19  22 |     CAC  14  12 |     CGC   9   7
    CTA   5   2 |     CCA   4   3 | Gln CAA   4   6 |     CGA   0   2
    CTG  24  30 |     CCG   4   6 |     CAG  19  16 |     CGG   3   5
------------------------------------------------------------------
Ile ATT   7  10 | Thr ACT   9   3 | Asn AAT   8   9 | Ser AGT   2   4
    ATC  28  25 |     ACC  14  20 |     AAC   8   7 |     AGC  13  10
    ATA   3   3 |     ACA   9   6 | Lys AAA   2   4 | Arg AGA   2   2
Met ATG  12  15 |     ACG   9  10 |     AAG  15  12 |     AGG   6   4
------------------------------------------------------------------
Val GTT   4   2 | Ala GCT  10  14 | Asp GAT  18  15 | Gly GGT   5   2
    GTC  15  14 |     GCC  23  24 |     GAC  27  31 |     GGC  18  21
    GTA   5   2 |     GCA   5   5 | Glu GAA   7   8 |     GGA   6   6
    GTG  16  17 |     GCG   2   1 |     GAG  27  27 |     GGG  12  13
------------------------------------------------------------------

Codon position x base (3x4) table for each sequence.

#1: Orcinus_orca
position  1:    T:0.18425    C:0.23283    A:0.25293    G:0.32998
position  2:    T:0.30318    C:0.24288    A:0.29146    G:0.16248
position  3:    T:0.21106    C:0.40034    A:0.09548    G:0.29313
Average         T:0.23283    C:0.29202    A:0.21329    G:0.26186

#2: Tursiops_truncatus
position  1:    T:0.18760    C:0.22613    A:0.25126    G:0.33501
position  2:    T:0.29313    C:0.25293    A:0.28643    G:0.16750
position  3:    T:0.20436    C:0.40536    A:0.09883    G:0.29146
Average         T:0.22836    C:0.29481    A:0.21217    G:0.26466

#3: Neophocaena_phocaenoides
position  1:    T:0.18593    C:0.23116    A:0.24958    G:0.33333
position  2:    T:0.29983    C:0.24958    A:0.28141    G:0.16918
position  3:    T:0.20436    C:0.40704    A:0.09715    G:0.29146
Average         T:0.23004    C:0.29592    A:0.20938    G:0.26466

#4: Lipotes_vexillifer
position  1:    T:0.18593    C:0.23786    A:0.25126    G:0.32496
position  2:    T:0.29313    C:0.25126    A:0.28643    G:0.16918
position  3:    T:0.21106    C:0.39866    A:0.09548    G:0.29481
Average         T:0.23004    C:0.29592    A:0.21106    G:0.26298

#5: Balaenoptera_acutorostrata
position  1:    T:0.18090    C:0.23953    A:0.24791    G:0.33166
position  2:    T:0.29313    C:0.25628    A:0.28476    G:0.16583
```

```
position  3:    T:0.19933    C:0.41206    A:0.09045    G:0.29816
Average         T:0.22446    C:0.30262    A:0.20771    G:0.26521

#6: Balaenoptera_physalus
position  1:    T:0.18258    C:0.23618    A:0.25461    G:0.32663
position  2:    T:0.29648    C:0.25293    A:0.29313    G:0.15745
position  3:    T:0.20101    C:0.41039    A:0.08878    G:0.29983
Average         T:0.22669    C:0.29983    A:0.21217    G:0.26131

#7: Bos_taurus
position  1:    T:0.18090    C:0.23786    A:0.24623    G:0.33501
position  2:    T:0.29983    C:0.23953    A:0.28978    G:0.17085
position  3:    T:0.19095    C:0.43551    A:0.09380    G:0.27973
Average         T:0.22390    C:0.30430    A:0.20994    G:0.26186

#8: Sus_scrofa
position  1:    T:0.16918    C:0.25126    A:0.24121    G:0.33836
position  2:    T:0.29816    C:0.24791    A:0.29481    G:0.15913
position  3:    T:0.17420    C:0.45226    A:0.08878    G:0.28476
Average         T:0.21385    C:0.31714    A:0.20826    G:0.26075

Sums of codon usage counts
------------------------------------------------------------------------------
Phe F TTT      80 | Ser S TCT      39 | Tyr Y TAT      52 | Cys C TGT      42
      TTC     125 |       TCC     133 |       TAC     120 |       TGC      80
Leu L TTA      14 |       TCA      18 | *** * TAA       0 | *** * TGA       0
      TTG      78 |       TCG      33 |       TAG       0 | Trp W TGG      56
------------------------------------------------------------------------------
Leu L CTT      51 | Pro P CCT      78 | His H CAT      34 | Arg R CGT      23
      CTC     119 |       CCC     130 |       CAC     118 |       CGC      49
      CTA      35 |       CCA      35 | Gln Q CAA      38 |       CGA       7
      CTG     208 |       CCG      42 |       CAG     138 |       CGG      25
------------------------------------------------------------------------------
Ile I ATT      60 | Thr T ACT      88 | Asn N AAT      72 | Ser S AGT      30
      ATC     209 |       ACC     119 |       AAC      65 |       AGC      73
      ATA      22 |       ACA      75 | Lys K AAA      25 | Arg R AGA      28
Met M ATG     112 |       ACG      67 |       AAG     110 |       AGG      36
------------------------------------------------------------------------------
Val V GTT      31 | Ala A GCT      94 | Asp D GAT     128 | Gly G GGT      51
      GTC     102 |       GCC     190 |       GAC     207 |       GGC     144
      GTA      22 |       GCA      29 | Glu E GAA      50 |       GGA      49
      GTG     151 |       GCG      20 |       GAG     221 |       GGG      96
------------------------------------------------------------------------------


Codon position x base (3x4) table, overall

position  1:    T:0.18216    C:0.23660    A:0.24937    G:0.33187
position  2:    T:0.29711    C:0.24916    A:0.28853    G:0.16520
position  3:    T:0.19954    C:0.41520    A:0.09359    G:0.29167
Average         T:0.22627    C:0.30032    A:0.21050    G:0.26291


Nei & Gojobori 1986. dN/dS (dN, dS)
(Note: This matrix is not used in later ML. analysis.
Use runmode = -2 for ML pairwise comparison.)

Orcinus_orca
Tursiops_truncatus   0.8408 (0.0178 0.0212)
Neophocaena_phocaenoides 1.0453 (0.0270 0.0258) 0.8334 (0.0255 0.0306)
Lipotes_vexillifer   0.8298 (0.0479 0.0577) 0.8313 (0.0479 0.0576) 0.7981 (0.0496 0.0621)
Balaenoptera_acutorostrata 0.7447 (0.0553 0.0743) 0.7507 (0.0538 0.0716) 0.7507 (0.0543 0.0723) 0.7527
(0.0527 0.0700)
Balaenoptera_physalus 0.6855 (0.0616 0.0899) 0.7105 (0.0629 0.0885) 0.6728 (0.0634 0.0943) 0.7024
(0.0582 0.0828) 0.5288 (0.0239 0.0453)
Bos_taurus           0.3522 (0.1025 0.2911) 0.3368 (0.0996 0.2958) 0.3597 (0.1011 0.2812) 0.3377
(0.0971 0.2877) 0.2775 (0.0729 0.2627) 0.2814 (0.0831 0.2952)
Sus_scrofa           0.2496 (0.0892 0.3574) 0.2370 (0.0848 0.3580) 0.2536 (0.0882 0.3479) 0.2408
(0.0840 0.3489) 0.1883 (0.0634 0.3365) 0.2189 (0.0730 0.3335) 0.1460 (0.0649 0.4443)


TREE #  1:  (((((1, 2), 3), 4), (5, 6)), 7, 8);   MP score: 489
lnL(ntime: 13  np: 15):  -5014.681347      +0.000000
   9..10    10..11   11..12   12..13   13..1    13..2    12..3    11..4    10..14   14..5    14..6
9..7     9..8
 0.072474 0.048129 0.042557 0.013814 0.031392 0.025768 0.043342 0.075750 0.023845 0.028945 0.060422
0.270103 0.273835 4.329792 0.121100
```

Note: Branch length is defined as number of nucleotide substitutions per codon (not per neucleotide site).

tree length = 1.01038

(((((1: 0.031392, 2: 0.025768): 0.013814, 3: 0.043342): 0.042557, 4: 0.075750): 0.048129, (5: 0.028945, 6: 0.060422): 0.023845): 0.072474, 7: 0.270103, 8: 0.273835);

(((((Orcinus_orca: 0.031392, Tursiops_truncatus: 0.025768): 0.013814, Neophocaena_phocaenoides: 0.043342): 0.042557, Lipotes_vexillifer: 0.075750): 0.048129, (Balaenoptera_acutorostrata: 0.028945, Balaenoptera_physalus: 0.060422): 0.023845): 0.072474, Bos_taurus: 0.270103, Sus_scrofa: 0.273835);

Detailed output identifying parameters

kappa (ts/tv) = 4.32979

w (dN/dS) for branches: 0.12110 1.00000

dN & dS for each branch

branch	t	N	S	dN/dS	dN	dS	N*dN	S*dS
9..10	0.072	1347.4	443.6	1.0000	0.0242	0.0242	32.5	10.7
10..11	0.048	1347.4	443.6	1.0000	0.0160	0.0160	21.6	7.1
11..12	0.043	1347.4	443.6	1.0000	0.0142	0.0142	19.1	6.3
12..13	0.014	1347.4	443.6	1.0000	0.0046	0.0046	6.2	2.0
13..1	0.031	1347.4	443.6	1.0000	0.0105	0.0105	14.1	4.6
13..2	0.026	1347.4	443.6	1.0000	0.0086	0.0086	11.6	3.8
12..3	0.043	1347.4	443.6	1.0000	0.0144	0.0144	19.5	6.4
11..4	0.076	1347.4	443.6	1.0000	0.0253	0.0253	34.0	11.2
10..14	0.024	1347.4	443.6	1.0000	0.0079	0.0079	10.7	3.5
14..5	0.029	1347.4	443.6	1.0000	0.0096	0.0096	13.0	4.3
14..6	0.060	1347.4	443.6	1.0000	0.0201	0.0201	27.1	8.9
9..7	0.270	1347.4	443.6	0.1211	0.0322	0.2657	43.4	117.9
9..8	0.274	1347.4	443.6	0.1211	0.0326	0.2694	44.0	119.5

tree length for dN: 0.2203
tree length for dS: 0.6906

dS tree:
(((((Orcinus_orca: 0.010464, Tursiops_truncatus: 0.008589): 0.004605, Neophocaena_phocaenoides: 0.014447): 0.014186, Lipotes_vexillifer: 0.025250): 0.016043, (Balaenoptera_acutorostrata: 0.009648, Balaenoptera_physalus: 0.020141): 0.007948): 0.024158, Bos_taurus: 0.265742, Sus_scrofa: 0.269415);
dN tree:
(((((Orcinus_orca: 0.010464, Tursiops_truncatus: 0.008589): 0.004605, Neophocaena_phocaenoides: 0.014447): 0.014186, Lipotes_vexillifer: 0.025250): 0.016043, (Balaenoptera_acutorostrata: 0.009648, Balaenoptera_physalus: 0.020141): 0.007948): 0.024158, Bos_taurus: 0.032181, Sus_scrofa: 0.032626);

w ratios as labels for TreeView:
(((((Orcinus_orca #1.0000 , Tursiops_truncatus #1.0000) #1.0000 , Neophocaena_phocaenoides #1.0000) #1.0000 , Lipotes_vexillifer #1.0000) #1.0000 , (Balaenoptera_acutorostrata #1.0000 , Balaenoptera_physalus #1.0000) #1.0000) #1.0000 , Bos_taurus #0.1211 , Sus_scrofa #0.1211);

Main result file for model G: whales-G.mlc

```
CODONML (in paml version 4.7b, October 2013)  whales.phy
Model: free dN/dS Ratios for branches for branches,
Codon frequency model: F3x4
ns =   8  ls = 597

Codon usage in sequences
--------------------------------------------------------------------------------------------------------------------------------------
Phe TTT  10  10   9  11  10  10 | Ser TCT   6   5   5   5   4   5 | Tyr TAT   6   7   7   4   7   7 | Cys TGT   5   4   3   9   6   6
    TTC  13  14  12  15  18  16 |     TCC  16  17  20  18  16  16 |     TAC  15  15  15  16  13  14 |     TGC  11  12  11   9   9  10
Leu TTA   3   2   2   2   1   1 |     TCA   2   3   3   2   2   1 | *** TAA   0   0   0   0   0   0 | *** TGA   0   0   0   0   0   0
    TTG  12  12  12   9  10  10 |     TCG   4   4   4   4   5   6 |     TAG   0   0   0   0   0   0 | Trp TGG   7   7   8   7   7   7
--------------------------------------------------------------------------------------------------------------------------------------
Leu CTT   6   7   6   6   6   7 | Pro CCT   9   9   9  12  10  10 | His CAT   5   3   3   5   6   5 | Arg CGT   2   4   5   2   3   2
    CTC  14  12  14  13  16  18 |     CCC  14  14  15  14  17  15 |     CAC  15  15  15  15  16  16 |     CGC   6   4   6   6   7   4
    CTA   6   5   5   4   4   4 |     CCA   5   5   6   4   4   4 | Gln CAA   6   6   4   5   3   4 |     CGA   0   0   1   1   2   1
    CTG  26  23  27  28  24  26 |     CCG   4   7   3   6   7   5 |     CAG  18  18  16  19  16  16 |     CGG   3   3   3   2   2   4
--------------------------------------------------------------------------------------------------------------------------------------
Ile ATT   6   6   7   7   8   9 | Thr ACT  14  13  13  14  11  11 | Asn AAT  10   9  10  10   9   7 | Ser AGT   4   6   4   4   3   3
    ATC  27  25  26  25  26  27 |     ACC  16  16  14  13  12  14 |     AAC   8   8   8   9   7  10 |     AGC   9   9   8   6   9   9
    ATA   1   3   3   3   3   3 |     ACA   9   9  10  11  10  11 | Lys AAA   4   3   3   3   3   3 | Arg AGA   4   4   3   5   4   4
Met ATG  17  15  16  12  13  12 |     ACG   5   7   7   9  11   9 |     AAG  13  13  13  13  15  16 |     AGG   4   4   4   6   4   4
--------------------------------------------------------------------------------------------------------------------------------------
Val GTT   5   5   5   4   4   2 | Ala GCT  13  13  13  10  10  11 | Asp GAT  17  14  15  17  15  17 | Gly GGT   8   7   8   6   7   8
    GTC  11  13  13  12  12  12 |     GCC  23  23  22  23  27  25 |     GAC  25  28  27  22  23  24 |     GGC  16  17  17  22  18  15
    GTA   3   3   3   2   2   2 |     GCA   3   3   4   3   3   3 | Glu GAA   5   6   5   6   7   6 |     GGA   6   7   6   6   6   6
    GTG  21  20  19  22  18  18 |     GCG   2   3   1   2   4   5 |     GAG  27  26  27  27  30  30 |     GGG  12  12  14  10  12  11
--------------------------------------------------------------------------------------------------------------------------------------


----------------------------------------------------------------------
Phe TTT  13   7 | Ser TCT   5   4 | Tyr TAT   5   9 | Cys TGT   6   3
    TTC  17  20 |     TCC  14  16 |     TAC  17  15 |     TGC   9   9
Leu TTA   1   2 |     TCA   3   2 | *** TAA   0   0 | *** TGA   0   0
    TTG   9   4 |     TCG   2   4 |     TAG   0   0 | Trp TGG   7   6
----------------------------------------------------------------------
Leu CTT   5   8 | Pro CCT  11   8 | His CAT   2   5 | Arg CGT   4   1
    CTC  15  17 |     CCC  19  22 |     CAC  14  12 |     CGC   9   7
    CTA   5   2 |     CCA   4   3 | Gln CAA   4   6 |     CGA   0   2
    CTG  24  30 |     CCG   4   6 |     CAG  19  16 |     CGG   3   5
----------------------------------------------------------------------
Ile ATT   7  10 | Thr ACT   9   3 | Asn AAT   8   9 | Ser AGT   2   4
    ATC  28  25 |     ACC  14  20 |     AAC   8   7 |     AGC  13  10
    ATA   3   3 |     ACA   9   6 | Lys AAA   2   4 | Arg AGA   2   2
Met ATG  12  15 |     ACG   9  10 |     AAG  15  12 |     AGG   6   4
----------------------------------------------------------------------
Val GTT   4   2 | Ala GCT  10  14 | Asp GAT  18  15 | Gly GGT   5   2
    GTC  15  14 |     GCC  23  24 |     GAC  27  31 |     GGC  18  21
    GTA   5   2 |     GCA   5   5 | Glu GAA   7   8 |     GGA   6   6
    GTG  16  17 |     GCG   2   1 |     GAG  27  27 |     GGG  12  13
----------------------------------------------------------------------

Codon position x base (3x4) table for each sequence.

#1: Orcinus_orca
position  1:    T:0.18425    C:0.23283    A:0.25293    G:0.32998
position  2:    T:0.30318    C:0.24288    A:0.29146    G:0.16248
position  3:    T:0.21106    C:0.40034    A:0.09548    G:0.29313
Average         T:0.23283    C:0.29202    A:0.21329    G:0.26186

#2: Tursiops_truncatus
position  1:    T:0.18760    C:0.22613    A:0.25126    G:0.33501
position  2:    T:0.29313    C:0.25293    A:0.28643    G:0.16750
position  3:    T:0.20436    C:0.40536    A:0.09883    G:0.29146
Average         T:0.22836    C:0.29481    A:0.21217    G:0.26466

#3: Neophocaena_phocaenoides
position  1:    T:0.18593    C:0.23116    A:0.24958    G:0.33333
position  2:    T:0.29983    C:0.24958    A:0.28141    G:0.16918
position  3:    T:0.20436    C:0.40704    A:0.09715    G:0.29146
Average         T:0.23004    C:0.29592    A:0.20938    G:0.26466

#4: Lipotes_vexillifer
position  1:    T:0.18593    C:0.23786    A:0.25126    G:0.32496
position  2:    T:0.29313    C:0.25126    A:0.28643    G:0.16918
position  3:    T:0.21106    C:0.39866    A:0.09548    G:0.29481
Average         T:0.23004    C:0.29592    A:0.21106    G:0.26298

#5: Balaenoptera_acutorostrata
position  1:    T:0.18090    C:0.23953    A:0.24791    G:0.33166
position  2:    T:0.29313    C:0.25628    A:0.28476    G:0.16583
position  3:    T:0.19933    C:0.41206    A:0.09045    G:0.29816
```

```
Average         T:0.22446    C:0.30262    A:0.20771    G:0.26521

#6: Balaenoptera_physalus
position  1:    T:0.18258    C:0.23618    A:0.25461    G:0.32663
position  2:    T:0.29648    C:0.25293    A:0.29313    G:0.15745
position  3:    T:0.20101    C:0.41039    A:0.08878    G:0.29983
Average         T:0.22669    C:0.29983    A:0.21217    G:0.26131

#7: Bos_taurus
position  1:    T:0.18090    C:0.23786    A:0.24623    G:0.33501
position  2:    T:0.29983    C:0.23953    A:0.28978    G:0.17085
position  3:    T:0.19095    C:0.43551    A:0.09380    G:0.27973
Average         T:0.22390    C:0.30430    A:0.20994    G:0.26186

#8: Sus_scrofa
position  1:    T:0.16918    C:0.25126    A:0.24121    G:0.33836
position  2:    T:0.29816    C:0.24791    A:0.29481    G:0.15913
position  3:    T:0.17420    C:0.45226    A:0.08878    G:0.28476
Average         T:0.21385    C:0.31714    A:0.20826    G:0.26075

Sums of codon usage counts
------------------------------------------------------------------------------
Phe F TTT      80 | Ser S TCT      39 | Tyr Y TAT      52 | Cys C TGT      42
      TTC     125 |       TCC     133 |       TAC     120 |       TGC      80
Leu L TTA      14 |       TCA      18 | *** * TAA       0 | *** * TGA       0
      TTG      78 |       TCG      33 |       TAG       0 | Trp W TGG      56
------------------------------------------------------------------------------
Leu L CTT      51 | Pro P CCT      78 | His H CAT      34 | Arg R CGT      23
      CTC     119 |       CCC     130 |       CAC     118 |       CGC      49
      CTA      35 |       CCA      35 | Gln Q CAA      38 |       CGA       7
      CTG     208 |       CCG      42 |       CAG     138 |       CGG      25
------------------------------------------------------------------------------
Ile I ATT      60 | Thr T ACT      88 | Asn N AAT      72 | Ser S AGT      30
      ATC     209 |       ACC     119 |       AAC      65 |       AGC      73
      ATA      22 |       ACA      75 | Lys K AAA      25 | Arg R AGA      28
Met M ATG     112 |       ACG      67 |       AAG     110 |       AGG      36
------------------------------------------------------------------------------
Val V GTT      31 | Ala A GCT      94 | Asp D GAT     128 | Gly G GGT      51
      GTC     102 |       GCC     190 |       GAC     207 |       GGC     144
      GTA      22 |       GCA      29 | Glu E GAA      50 |       GGA      49
      GTG     151 |       GCG      20 |       GAG     221 |       GGG      96
------------------------------------------------------------------------------


Codon position x base (3x4) table, overall

position  1:    T:0.18216    C:0.23660    A:0.24937    G:0.33187
position  2:    T:0.29711    C:0.24916    A:0.28853    G:0.16520
position  3:    T:0.19954    C:0.41520    A:0.09359    G:0.29167
Average         T:0.22627    C:0.30032    A:0.21050    G:0.26291


Nei & Gojobori 1986. dN/dS (dN, dS)
(Note: This matrix is not used in later ML. analysis.
Use runmode = -2 for ML pairwise comparison.)

Orcinus_orca
Tursiops_truncatus   0.8408 (0.0178 0.0212)
Neophocaena_phocaenoides 1.0453 (0.0270 0.0258) 0.8334 (0.0255 0.0306)
Lipotes_vexillifer   0.8298 (0.0479 0.0577) 0.8313 (0.0479 0.0576) 0.7981 (0.0496 0.0621)
Balaenoptera_acutorostrata 0.7447 (0.0553 0.0743) 0.7507 (0.0538 0.0716) 0.7507 (0.0543 0.0723) 0.7527
(0.0527 0.0700)
Balaenoptera_physalus 0.6855 (0.0616 0.0899) 0.7105 (0.0629 0.0885) 0.6728 (0.0634 0.0943) 0.7024
(0.0582 0.0828) 0.5288 (0.0239 0.0453)
Bos_taurus           0.3522 (0.1025 0.2911) 0.3368 (0.0996 0.2958) 0.3597 (0.1011 0.2812) 0.3377
(0.0971 0.2877) 0.2775 (0.0729 0.2627) 0.2814 (0.0831 0.2952)
Sus_scrofa           0.2496 (0.0892 0.3574) 0.2370 (0.0848 0.3580) 0.2536 (0.0882 0.3479) 0.2408
(0.0840 0.3489) 0.1883 (0.0634 0.3365) 0.2189 (0.0730 0.3335) 0.1460 (0.0649 0.4443)


TREE #  1:  (((((1, 2), 3), 4), (5, 6)), 7, 8);   MP score: 489
check convergence..
lnL(ntime: 13  np: 27):  -5006.309880      +0.000000
   9..10   10..11   11..12   12..13   13..1    13..2    12..3    11..4    10..14   14..5    14..6
9..7     9..8
 0.092421 0.048363 0.042699 0.013653 0.031377 0.025791 0.043457 0.075615 0.023553 0.029074 0.060868
0.245923 0.267419 4.229337 0.363766 0.812344 0.607465 2.477583 1.085954 0.694064 0.801790 0.836343
```

0.884852 0.489851 0.531208 0.179503 0.094138

Note: Branch length is defined as number of nucleotide substitutions per codon (not per neucleotide site).

tree length =   1.00021

(((((1: 0.031377, 2: 0.025791): 0.013653, 3: 0.043457): 0.042699, 4: 0.075615): 0.048363, (5: 0.029074, 6: 0.060868): 0.023553): 0.092421, 7: 0.245923, 8: 0.267419);

(((((Orcinus_orca: 0.031377, Tursiops_truncatus: 0.025791): 0.013653, Neophocaena_phocaenoides: 0.043457): 0.042699, Lipotes_vexillifer: 0.075615): 0.048363, (Balaenoptera_acutorostrata: 0.029074, Balaenoptera_physalus: 0.060868): 0.023553): 0.092421, Bos_taurus: 0.245923, Sus_scrofa: 0.267419);

Detailed output identifying parameters

kappa (ts/tv) = 4.22934

w (dN/dS) for branches: 0.36377 0.81234 0.60747 2.47758 1.08595 0.69406 0.80179 0.83634 0.88485 0.48985 0.53121 0.17950 0.09414

dN & dS for each branch

branch	t	N	S	dN/dS	dN	dS	N*dN	S*dS
9..10	0.092	1348.5	442.5	0.3638	0.0215	0.0591	29.0	26.2
10..11	0.048	1348.5	442.5	0.8123	0.0153	0.0188	20.6	8.3
11..12	0.043	1348.5	442.5	0.6075	0.0123	0.0202	16.6	8.9
12..13	0.014	1348.5	442.5	2.4776	0.0053	0.0022	7.2	1.0
13..1	0.031	1348.5	442.5	1.0860	0.0107	0.0098	14.4	4.3
13..2	0.026	1348.5	442.5	0.6941	0.0078	0.0112	10.5	4.9
12..3	0.043	1348.5	442.5	0.8018	0.0137	0.0170	18.4	7.5
11..4	0.076	1348.5	442.5	0.8363	0.0240	0.0287	32.4	12.7
10..14	0.024	1348.5	442.5	0.8849	0.0076	0.0086	10.3	3.8
14..5	0.029	1348.5	442.5	0.4899	0.0077	0.0157	10.4	7.0
14..6	0.061	1348.5	442.5	0.5312	0.0167	0.0314	22.5	13.9
9..7	0.246	1348.5	442.5	0.1795	0.0385	0.2145	51.9	94.9
9..8	0.267	1348.5	442.5	0.0941	0.0264	0.2803	35.6	124.1

tree length for dN: 0.2073
tree length for dS: 0.7175

dS tree:
(((((Orcinus_orca: 0.009823, Tursiops_truncatus: 0.011170): 0.002154, Neophocaena_phocaenoides: 0.017027): 0.020204, Lipotes_vexillifer: 0.028747): 0.018774, (Balaenoptera_acutorostrata: 0.015735, Balaenoptera_physalus: 0.031357): 0.008596): 0.059134, Bos_taurus: 0.214458, Sus_scrofa: 0.280342);
dN tree:
(((((Orcinus_orca: 0.010668, Tursiops_truncatus: 0.007753): 0.005338, Neophocaena_phocaenoides: 0.013652): 0.012273, Lipotes_vexillifer: 0.024043): 0.015251, (Balaenoptera_acutorostrata: 0.007708, Balaenoptera_physalus: 0.016657): 0.007606): 0.021511, Bos_taurus: 0.038496, Sus_scrofa: 0.026391);

w ratios as labels for TreeView:
(((((Orcinus_orca #1.0860 , Tursiops_truncatus #0.6941) #2.4776 , Neophocaena_phocaenoides #0.8018) #0.6075 , Lipotes_vexillifer #0.8363) #0.8123 , (Balaenoptera_acutorostrata #0.4899 , Balaenoptera_physalus #0.5312) #0.8849) #0.3638 , Bos_taurus #0.1795 , Sus_scrofa #0.0941);
